# 9th Trends in Medical Mycology Held on 11–14 October 2019, Nice, France, Organized under the Auspices of EORTC-IDG and ECMM

**DOI:** 10.3390/jof5040095

**Published:** 2019-10-08

**Authors:** Jean-Pierre Gangneux, Olivier Lortholary, Oliver A. Cornely, Livio Pagano

**Affiliations:** 1 UMR_S 1085—Inserm, Institut de Recherche en Santé, Environnement et Travail, CHU de Rennes, Université de Rennes, 35000 Rennes, France; jean-pierre.gangneux@univ-rennes1.fr; 2 Necker - Pasteur Center for Infectious Diseases and Tropical Medicine, Necker-Enfants malades Hospital, AP-HP, Paris Descartes University, 75015 Paris, France; 3 Institut Pasteur, Molecular Mycology Unit, National Reference Center for Invasive Mycoses and Antifungals, CNRS UMR 2000, 75015 Paris, France; olivier.lortholary@aphp.fr; 4 Department I of Internal Medicine, Clinical Trials Centre Cologne, ZKS Köln, Cologne Excellence Cluster on Cellular Stress Responses in Aging-Associated Diseases (CECAD), University of Cologne, 50931 Cologne, Germany; oliver.cornely@uk-koeln.de; 5 Department of Hematology, Università Cattolica del Sacro Cuore, 00168 Rome, Italy; livio.pagano@unicatt.it

## Introduction

Dear Friends and Colleagues,

It is a great honor and pleasure for us to invite you cordially to participate in the 9th Congress on Trends in Medical Mycology (TIMM-9), which will be held in 11–14 October 2019 at Nice-Acropolis Convention Center, Nice, France. TIMM-9 is the 9th in the series of TIMM mycological international meetings organized jointly by the European Confederation of Medical Mycology (ECMM) and the Infectious Diseases Group of the European Organisation for Research and Treatment of Cancer (EORTC-IDG).

TIMM has become an important and essential meeting in the field of fungal infections, a forum in which researchers and clinicians from all over the world present the most important advances and research findings in clinical mycology. TIMM-9 will cover all aspects of mycology, with special focus on evidence-based and personalized approach to medical mycology, as well as diagnostic–therapeutic integrative efforts in the quest to improve the present knowledge of epidemiology, diagnosis, clinical course, and pathophysiological mechanisms of fungal diseases. It would be the place to present recent innovations in medical mycology.

The meeting is designed for medical microbiologists, medical mycologists, hematologists, oncologists, transplant physicians, intensivists, immunologists, and all those with interest in medical mycology. We expect TIMM-9 to be at least as successful as previous TIMM Congresses, which brought together around 1000 international delegates from all over the world. Therefore, we would like to invite you to TIMM-9 in Nice to enjoy with us excellent science in a stimulating environment. 

We look forward to greeting you in Nice!

*Jean-Pierre Gangneux*
*Olivier Lortholary*
*Oliver A. Cornely*
*Livio Pagano*

TIMM-9 Executive Committee

 

**COMMITTEES**

**Executive Committee**

Jean-Pierre Gangneux, ECMM, France
Olivier Lortholary, EORTC, France
Livio Pagano, EORTC, Italy
Oliver A. Cornely, ECMM, Germany

**Local Scientific Committee**

Hamdi Akan, EORTC, Turkey
Valentina Arsić Arsenijević, ECMM, Serbia
Sharon Chen, ECMM, Australia
Raoul Herbrecht, EORTC, France
Martin Hoenigl, ECMM, Austria
Nikolai Klimko, ECMM, Rusland
Katrien Lagrou, ECMM, Belgium
Johan Maertens, EORTC, Belgium
Jacques F. Meis, ECMM, the Netherlands
Tony Pagliuca, EORTC, UK
Zděnek Racil, EORTC, Czech Republic
Esther Segal, ECMM, Israel
Don Sheppard, ECMM, Canada
Paul Verweij, EORTC, the Netherlands
Thomas Walsh, ECMM, USA

**International Scientific Committee**

Jean-Pierre Gangneux, Rennes
Olivier Lortholary, Paris
Alexandre Alanio, Paris
Jean-Philippe Bouchara, Angers
Marie-Elizabeth Bougnoux, Paris
Stephane Bretagne, Paris
Christophe D’Enfert, Paris
Jean-Paul Latgé, Paris
Laurence Millon, Besançon

**CONTENT**
Welcome Address   1Introduction to the Scientific Programme   5Scientific Programme   6Honorary Lectures   16Plenary Sessions   17Meet the Expert Sessions   29Symposia   31Poster Presentations   97

**Introduction to the Scientific Programme**

**Plenary Sessions:**

No session will be held in parallel to these sessions.

*Plenary sessions are indicated by the prefix: PS*

**Symposia:**

Each day, symposia will convene renowned speakers from several continents, who will cover a wide range of recent developments in their fields.

Symposia are indicated by the prefix: S

A part of the symposia includes a selected submitted abstract. These abstracts are marked with an asterisk (*).

**Meet the Expert Sessions:**

The audience will actively participate in these small sessions.

*Meet the expert sessions are indicated by the prefix: M*

**Poster Sessions:**

All poster boards are situated on the exhibition floor of the congress centre. The poster exhibition is open to all participants during the entire congress. The numbers on the poster boards correspond with the abstract numbers in this abstract supplement. 

All authors of odd poster numbers must be present at their poster on Saturday 12 October, from 11:30 to 12:30. All authors of even poster numbers must be present at their poster on Sunday 13 October, from 11:30 to 12:30.

*All posters are indicated by the prefix: P*

**SCIENTIFIC PROGRAMME                        **

**FRIDAY 11 OCTOBER 2019**

**09:00**


**ECMM Council Meeting**

**12:25**


**Welcome Address**

**12:45–13:45**


**Plenary Session 1—Tropical**



*Chairs: Jean-Pierre Gangneux, FECMM, France & Olivier Lortholary, France*
12:45PS1.1Histoplasmosis


*Matthieu Nacher, France*
13:15PS 1.2Emergomycosis


*Nelesh Govender, FECMM, South Africa*

**13:45–15:15**


**Parallel Symposia 1–4**

**Symposium 1**


**Water Quality (Mis-)Management—An Opportunity for Fungal Contamination**



*Chairs: João Brandão, Portugal & Esther Segal, FECMM, Israel*
13:45S01.1 Fungal Contaminants in Drinking Water Regulation? A Tale of Ecology, Exposure, Purification, and Clinical Relevance


*Monica Novak Babič, Slovenia*
14:05S01.2 Hospital Environment: Water Supply and Containment of Aerosolised Fungal Particles

How Far Must We Go in Times of Antimicrobial Resistance?


*Raquel Sabino, FECMM, Portugal*
14:25S01.3 Potential Transmission Pathways of Clinically Relevant Fungi in Indoor Swimming Pool Facilities


*Ciska Schets, the Netherlands*
14:45S01.4* Study of Fungal Environmental Contamination in Nests of the Penguin Enclosure of a Large French Animal Zoo Park


*Guillaume Desoubeaux, France*
14:55 S01.5* Spectrum of Indoor Fungi Isolated from Indoor Environments in Busia—Kenya


*Olga Mashedi, Kenya*

**Symposium 2**


***Pneumocystis jirovecii*, an Airborne Transmissible and Human-Derived Ascomycete Showing Strong Pulmonary Tropism**



*Chairs: Philippe Hauser, Switzerland & Gilles Nevez, France*
13:45S02.1 *Pneumocystis jirovecii*: An Obligate Parasite of Human Lungs with Unique Camouflage and Sex Strategies


*Philippe Hauser, Switzerland*
14:10S02.2 High-Throughput Methodologies in Molecular Epidemiology of *Pneumocystis*



*Jirovecii Olga Matos, Portugal*
14:35S02.3 Airborne Acquisition and Transmission of *Pneumocystis jirovecii*: An Update


*Gilles Nevez, France*
15:00S02.5* Does *Pneumocystis jirovecii* Infection Aggravate the Prognosis of Invasive Pulmonary Aspergillosis? Data from the RESSIF Network in France (2012–2016)


*Florence Robert-Gangneux, France*

**Symposium 3**


**Mucormycosis**



*Chairs: Fanny Lanternier, FECMM, France & Arunaloke Chakrabarti, FECMM, India*
13:45 S03.1 Hospital-Related Mucormycosis


*Anna Skiada, Greece*
14:00 S03.3 The New Treatment of Mucormycosis 


*Livio Pagano, FECMM, Italy*
14:15 S03.4 Clinical Features Associated to Fungal Species


*Dea Garcia Hermoso, France*
14:30 S03.5 Molecular diagnosis


*Laurence Millon, France*
14:45 S03.6 Management of Mucormycosis in Low- and Middle-Income Countries


*Arunaloke Chakrabarti, FECMM, India*
15:00 
Discussion
**Symposium 4**


**MSG Symposium (Trial Design in Clinical Mycology: Innovative Approaches)**



*Chairs: Peter Pappas, USA & Sharon Chen, FECMM, Australia*
13:45 S04.1 Candidiasis


*Bart-Jan Kullberg, The Netherlands*
14:05 S04.2 Endemic Mycoses


*George Thompson, USA*
14:25 S04.3 Cryptococcosis


*David Boulware, USA*
14:45 S04.4 Aspergillosis and other molds


*Tom Patterson, USA*
15:05 S04.5* The Lung Transplant Community Is Interested in a Clinical Trial to Determine the Optimal Strategy to Prevent Invasive Mold Disease


*Ricardo La Hoz, USA*
18:30EDL
**E. Drouhet Lecture (ECMM)**



*Chair: Martin Hoenigl, FECMM, Austria*


How to Convince European institutions that Medical Mycology is a Major Science


*Jean-Paul Latgé, France*


**SATURDAY 12 OCTOBER 2019**

**08:00–08:45**


**Meet the Expert Sessions**
08:00M01 Hematology


*Malgorzata Mikulska, FECMM, Italy & Alessandro Busca, Italy*
08:00M02 Peadiatrics


*Simone Cesaro, Italy & Adillia Warris, FECMM, UK*
08:00M03 Candida in the ICU


*Matteo Bassetti, Italy & Jean-François Timsit, France*
08:00M04 Diagnostics/Laboratory


*Alida Talento, Ireland & Michaela Lackner, FECMM, Austria*
08:00M05 Tropical


*Arunaloke Chakrabarti, FECMM, India & Rita Oladele, FECMM, Nigeria*

**09:00–10:00**


**Plenary Session 2—Highlights on Fungal Biology**



*Chairs: Vishukumar Aimanianda, France & Don Sheppard, FECMM, Canada*
08:50 PS 2.1 Candida


*Christophe D’Enfert, France*
09:20PS 2.2 Aspergillus


*Agostinho Carvalho, FECMM, Portugal*
09:50PS 2.3* Lineage-Specific Behavioural Differences in Isolates of *Candida auris*


*Andrew Borman, UK*

**10:30–11:30**


**Plenary Session 3—One World One Guideline ECMM MSG-ERC (EFISG) ISHAM Guidelines Initiative**



*Chairs: Martin Hoenigl, FECMM, Austria & John Perfect, USA*
10:30 PS3.1 Mucormycosis


*Oliver Cornely, FECMM, Germany*
10:45 PS3.2 Endemic


*George Thompson, USA*
11:00 PS3.3 Rare Moulds


*Martin Hoenigl, FECMM, Austria*
11:15 PS3.4 Rare Yeasts


*Sharon Chen, FECMM, Australia*

**11:30-12:30**


**Poster Session**

**14:15-15:45**


**Parallel Symposia 5–9**

**Symposium 5**


**Lung Transplantation**



*Chairs: Shahid Husain, FECMM, Canada & Paolo Grossi, Italy*
14:15S05.1 Pretransplant Assessment


*Blandine Rammaert, France*
14:35 S05.2 Pathophysiology and Epidemiology of Invasive Aspergillosis in Lung Transplant Recipients


*Claire Aguilar, France*
14:55 S05.3 Current Guidelines


*Shahid Husain, FECMM, Canada*
15:15 S05.4 Prophylaxis


*John Perfect, USA*
15:35 S05.5* From the Lung to the Heart: Fatal Dissemination of Azole-Resistant Aspergillus Fumigatus in a Lung Transplant Patient


*Rose-Anne Lavergne, France*

**Symposium 6**


**Prophylaxis during Hematology Malignancies**



*Chairs: Livio Pagano, FEMCC, Italy & Oliver Cornely, FECMM, Germany*
14:15 S06.1 AML—in the Era of FLT3 Inhibitors


*Russel Lewis, Italy*
14:35 S06.2 ALL—There Is a Role for Prophylaxis


*Daniel Teschner, Germany*
14:55 S06.3 Personalized Medicine/Approach by Genetic Risk Factors


*Pierre-Yves Bochud, Switzerland*
15:15 S06.4 Baseline CT upon Diagnosis of Acute Leukemia


*Stefan Schwartz, Germany*
15:35 S06.5* Investigating the Impact of Posaconazole Prophylaxis on Systematic Fungal Screening Using Galactomannan Antigen, Aspergillus qPCR and Mucorales qPCR


*Anne-Pauline Bellanger, France*

**Symposium 7**


**Paediatric Mycology (EPMyN)**



*Chairs: Emmanuel Roilides, FECMM, Greece & Roger Brüggemann, FECMM, The Netherlands*
14:15S07.1 Fluconazole and Micafungin Dosing in Neonates


*Roger Brüggemann, The Netherlands*
14:35S07.2 Antifungal Susceptibility of Pediatric Candidemia


*Zoi-Dorothea Pana, Greece*
14:55S07.3 Primary Immunodeficiencies Characterized by Fungal Infections


*Fanny Lanternier, FECMM, France*
15:25S07.5* Isavuconazole use in pediatric hematoncologic patients: the Italian Association of Pediatric Hematology Oncology (AIEOP) experience


*Nunzia Decembrino, Italy*

**Symposium 8**


**Immunologic Markers for Diagnosis and Treatment Stratification in Invasive Mold Infection**



*Chairs: Martin Hoenigl, FECMM, Austria & Agostinho Carvalho, FECMM, Portugal*
14:15S08.1 Antimold Immune Response: the Impact of the Host, the Pathogen, and Translational Implications


*Agostinho Carvalho, FECMM, Portugal*
14:35S08.2 Immunologic Markers for Diagnosis of Invasive Aspergillosis and Other Invasive Mold Infections


*Carol Garcia-Vidal, Spain*
14:55S08.3 Immunologic Markers For Treatment Stratification in Invasive Mold Infection


*Philipp Köhler, FECMM, Germany*
15:15S08.4 Fungal Translocation: From Persistent Inflammation to Non-AIDS Events


*Martin Hoenigl, FECMM, Austria*
15:35S08.5* Immunological Characteristics of Broncho Alveolar Lavage in Neutropenic Patients with Invasive Aspergillosis


*Claire Aguilar, France*

**Symposium 9**


**Chronic Pulmonary Aspergillosis**



*Chairs: David Denning, FECMM, United Kingdom & Aleksandra Barac, FECMM, Serbia*
14:15S09.1 Effect of Patient Immunodeficiencies on the Diagnostic Performance of Serological Assays to Detect Aspergillus-Specific Antibodies in Chronic Pulmonary Aspergillosis


*Elizabeth Hunter, UK*
14:35S09.2 Diagnosis of CPA. Where Do We Stand?


*Aleksandra Barac, FECMM, Serbia*
14:55S09.3Current Treatment Options for CPA


*David Denning, FECMM, UK*
15:15S09.4 Future Directions


*David Denning, FECMM, UK*
15:25S09.5* Raised Amphotericin B MIC in Aspergillus fumigatus Isolates from Patients with Chronic Pulmonary Aspergillosis


*Fiona Lynch, UK*

**16:15–17:45**


**Parallel Symposia 10–14**

**Symposium 10**


**Sensing the Host**



*Chairs: Muriel Cornet, France & Mihai Mares, FECMM, Romania*
16:15S10.1 S10.1 Cell wall of Aspergillus fumigatus in murine lung tissue


*Thierry Fontaine, France*
16:45S10.2 Hybrid Histidine Kinases: Major Sensing Proteins in Pathogenic Fungi


*Nicolas Papon, France*
17:15S10.3 Adapting to the Host: How Candida Causes Bloodstream Infection


*Oliver Kurzai, Germany*

**Symposium 11**


**The Antifungal Pipeline**



*Chairs: David Denning, FECMM, United Kingdom & Oliver Cornely, FECMM, Germany*
16:15S11.1Olorofim—A Novel Mould-Active Antifungal (F2G)


*John Rex, FECMM, UK*
16:40S11.2 Fosmanogepix: A Novel, Broad Spectrum Antifungal Therapy in Clinical Development (Amplyx)


*Michael Hodges, USA*
17:05S11.3 Ibrexafungerp (formerly SCY-078) (Scynexis)


*David Angulo, USA*

**Symposium 12**


**Environment and Fungal Outbreaks**



*Chairs: Jean-Pierre Gangneux, FECMM, France & Tom Chiller, USA*
16:15S12.1 Frogs


*Matthew Fisher, UK*
16:35S12.2 Cryptococcus gattii


*Ferry Hagen, FECMM, The Netherlands*
16:55S12.3 Natural Disasters


*Tom Chiller, US*
17:15S12.4 Genomic Sequencing


*Marie Desnos, France*
17:35S12.5* Outbreak of Fluconazole-Resistant Candida Parapsilosis in a Hospital Ward: Arguments for Clonal Transmission and Environmental Persistence


*Arnaud Fekkar, France*

**Symposium 13**


**Fungal Respiratory Infections in Cystic Fibrosis (the ECMM/ISHAM Working Group Fri-CF)**



*Chairs: Jean-Philippe Bouchara, France & Petr Hamal, Czech Republic*
16:15S13.1 Rasamsonia Species and Other Emerging Fungi in CF


*Solène Le Gal, France*
16:35S13.2 Immunodiagnosis of Scedosporium/Lomentospora Infection—Lessons from Immunoproteomic Studies


*Andoni Ramirez-Garcia, Spain*
16:55S13.3 Bacterial/Fungal Interactions in the CF Mucus


*Françoise Botterel, France*
17:15S13.4* Morphology, Growth, and Biofilm Formation of the Black Yeast-Like Fungus Exophiala dermatitidis is Influenced by Pseudomonas aeruginosa under in Vitro Cystic Fibrosis Conditions


*Lisa Kirchhoff, Germany*
17:25S13.6* Study of Antigenic Markers for Serological Detection of Scedosporium spp. in Cystic Fibrosis Patients


*Leire Martin-Souto, Spain*
17:35
Discussion
**Symposium 14**


**Teaching Medical Mycology: What, How, to Whom and When**



*Chairs: Patricia Muñoz, Spain & Esther Segal, FECMM, Israel*
16:15S14.1 Mycology Teaching in Medical School—How Much, What to Choose, at What Level


*Peter Rath, FECMM, Germany*
16:35S14.2 Mycology Teaching in Medical School—Is Student Demography Important to What Should Be Taught?


*Ester Segal, FECMM, Israel*
16:55S14.3 Specific Courses Outside the European Continent—The Indian Example


*Ruth Ashbee, United Kingdom*
17:15S14.4 Teaching Clinical Laboratory Mycology—How and Who Is the Audience?


*Sevtap Arikan-Akdagli, FECMM, Turkey*
17:35S14.5 The African Example


*Aude Sturny, France*


**SUNDAY 13 OCTOBER 2019**

**08:00–08:45**


**Meet the Expert Sessions 6–10**
08:00M06 Hematology—hematopoietic stem cell transplantation


*Nikolai Klimko, FECMM, Russia & Tony Pagliuca, UK*
08:00M07 Molecular Diagnostics


*Maurizio Sanguinetti, Italy & Dieter Buchheidt, FECMM, Germany*
08:00M08 Surgery/Transplantation


*Patricia Munoz, Spain & Paolo Grossi, Italy*
08:00M09 Neonates


*Thomas Walsh, FECMM, USA & Emmanuel Roilides, FECMM, Greece*
08:00M10 Meet Mr. and Mrs. Fungus


*Cornelia Lass-Flörl, FECMM, Austria & Neil Gow, FECMM, UK*

**08:50–09:50**


**Plenary Session 4—Top 10 papers in Mycology**



*Chairs: Maiken Arendrup, Denmark & Malcolm Richardson, FECMM, UK*
08:50PS4.1 The Clinical and Translational Perspective


*Don Sheppard, FECMM, Canada*
09:20PS4.2The Microbiology Perspective


*Katrien Lagrou, FECMM, Belgium*



**ECMM Academy and Excellence Centers: A Story of Success**



*Chair: Martin Hoenigl, FECMM, Austria*
09:50
ECMM Academy


*Katrien Lagrou, FECMM, Belgium*
09:55
ECMM Excellence Centers


*Cornelia Lass-Flörl, FECMM, Austria*

**10:30–11:30**


**Plenary Session 5—ICU—Candida Infections/Breaking News from Hematology and ICU**



*Chairs: Johan Maertens, FECMM, Belgium / Maricela Valerio, Spain*
10:30 PS5.1 Abdominal Candidiasis in ICU Patients


*Philipp Köhler, FECMM, Germany*
10:45 PS5.2 Are the New Management Strategies Useful?


*Arnaldo Colombo, FECMM, Brazil*
11:00 PS5.3 CNS Infections


*Anna Candoni, Italy*
11:15 PS5.4 CMV and Aspergillosis in HSCT Patients


*Johan Maertens, FECMM, Belgium*

**11:30–12:30**


**Poster Session 2**

**11:30**


**Video Session**
11:30V01 Subcutaneous Nodule Caused by Phaeoacremonium fuscum in a Non-Immunocompromised Patient


*Sofie Colman, Belgium*
11:40V02 Trichosporon Diagnosis—The Right Path


*Thayanidhi Premamalini, India*
11:50V03 Disseminated Rhinosporidiosis with Different Morphological Lesions Involving Various Anatomical Sites


*Jagdish Chander, India*

**14:15–15:45**


**Parallel Symposia 15–19**

**Symposium 15**


**Aspergillus in the ICU**



*Chairs: Katrien Lagrou, FECMM, Belgium & Alessandro Pasqualotto, FECMM, Brazil*
14:15 S15.1 Influenza


*Joost Wauters, Belgium*
14:35S15.2 Invasive Aspergillosis in Patients with Underlying Liver Cirrhosis


*Juergen Prattes, Austria*
14:55 S15.3 Renal Failure


*Riina Richardson Rautema, FECMM, UK*
15:15 S15.4* Fungal Pneumonia in Critically Ill Cirrhotics: Spectrum, Outcomes, Comparison of Diagnostic Methods and Biomarkers


*Pratibha Kale, India*
15:25 S15.5* IPAFLU Survey: Invasive Aspergillosis among Patients with Severe Influenza in Intensive Care Units


*Joost Wauters, Belgium*
15:35 S15.6* Trend of Candidemia with Bloodstream Infection in Intensive Care Units from 2006 to 2017: Results from the Korean National Healthcare-Associated Infections Surveillance System


*Young Hwa Choi, Korea*

**Symposium 16**


**Dermatology**



*Chairs: Valentina Arsic Arsenijevic, ECMM, Serbia & Sevtap Arıkan Akdagli, FECMM, Turkey*
14:15S16.1 Cutaneous Aspergillosis, Is It So Rare?


*Sevtap Arıkan Akdagli, FECMM, Turkey*
14:35S16.2 Is Resistance a Problem for Dermatophytosis?


*Pietro Nenoff, Germany*
14:55S16.3 Phaeohyphomycosis


*Teresa Martin, Spain*
15:15S16.4 Blastomycosis


*Ilan Schwarz, Canada*
15:35S16.5* Galleria mellonella as a Novelty Model to Study Host—Pathogen Interaction in Malassezia furfur CBS 1878


*Adriana Marcela Celis Ramírez, Colombia*

**Symposium 17**


**HIV-Associated Cryptococcal Meningitis**



*Chairs: John Perfect, USA & Olivier Lortholary, France*
14:15S17.1Screening in Low-Resource Areas


*Elvis Temfack, Cameroon*
14:35S17.2 Current Therapeutic Strategies


*Olivier Lortholary, France*
14:55S17.3 Towards Having Antifungal Drugs in Low-Resource Areas


*Ida Kolte, UK*
15:15S17.4 Early versus Delayed Antiretroviral Treatment in HIV-Positive People with Cryptococcal Meningitis


*Tihana Bicanic, FECMM, UK*
15:35S17.5 Current and Future Clinical Trials on HIV-Associated Cryptococcal Meningitis


*Tom Harrison, UK*

**Symposium 18**


**Antifungal Stewardship in the Era of Resistance**



*Chairs: Paul Verweij, FECMM, The Netherlands & Souha Kanj, FECMM Lebanon*
14:15S18.1 Antifungal Stewardship: A Practical Experience in a Tertiary Care Institution


*Maricela Valerio, Spain*
14:35S18.2Stewardship and Azole-Resistant Aspergillosis: A Challenge for Farmer or Physician?


*Paul Verweij, FECMM, The Netherlands*
14:55S18.3 Rapid Diagnosis of Fungal Infections: Impact on Stewardship


*Souha Kanj, FECMM, Lebanon*
15:15S18.4 Mycology Laboratory Diagnostic Capabilities in Different Areas of the World


*Alessandro Pasqualotto, FECMM, Brazil*
15:35S18.5* Optimising Antifungal Stewardship: An Evaluation of Candidaemia Guideline Compliance and Clinical Outcome


*Laura Cottom, UK*

**Symposium 19**


**Pneumocystis**



*Chairs: Enrique Calderon, Spain & Stephane Bretagne, FECMM, France*
14:15S19.1 Diagnosis


*Alexandre Alanio, FECMM, France*
14:40S19.2 Pneumocystosis in HIV and Non-HIV Patients


*Joseph Kovacs, USA*
15:05S19.4 Pneumocystosis in Neonates


*Enrique Calderon, Spain*
15:30S19.5* Evaluation of a Novel Commercial Loop-Mediated Isothermal Amplification (LAMP) Assay for Rapid Detection of Pneumocystis jirovecii


*Dirk Schmidt, Germany*

**16:15–17:45**


**Parallel Symposia 20–24**

**Symposium 20**


**Candida auris**



*Chairs: Nelesh Govender, FECMM, South Africa & Anuradha Chowdhary, FECMM, India*
16:15S20.1Schizophrenic Gram-Negative Yeast Conquering the World


*Jacques Meis, FECMM, The Netherlands*
16:35S20.2 In the US


*Tom Chiller, USA*
16:55S20.3 Outbreak Control


*Alba Ruiz, Spain*
17:15S20.4 In Resource-Limited Countries


*Anuradha Chowdhary, FECMM, India*
17:35S20.5* Understanding Echinocandin Activity towards *Candida auris*


*Milena Kordalewska, USA*

**Symposium 21**


**Immunotherapy for Opportunistic Fungal Infections**



*Chairs: Carol Garcia-Vidal, Spain & Dimitrios Kontoyiannis, FECMM, USA*
16:15S21.1 CAR-T Cells, NK Cells


*Dimitrios Kontoyiannis, FECMM, USA*
16:35S21.2 WBC Transfusions


*Livio Pagano, FECMM, Italy*
16:55S21.3 Cytokines


*George Chamilos, Greece*
17:15S21.4* Novel Chimeric Antigen Receptor T Cells for Invasive Aspergillosis Immunotherapy


*Michelle Seif, Germany*
17:25S21.5* Comparison of Circulating Lymphocyte Populations CD4+ and CD8+ T Cells, B and NK lymphocytes According to the Favorable or Worsening Evolution of Patients with Pneumocystosis


*Eléna Charpentier, France*
17:35S21.6* Glucosylceramides from Lomentospora prolificans Induce Cytokines Production and Increase the Microbicidal Activity of Macrophages


*Mariana Ingrid Dutra Silva Xisto, Brazil*

**Symposium 22**


**NGS and Mycobiota**



*Chairs: Laurence Delhaes, France & Jean-Pierre Gangneux, FECMM, France*
16:15S22.1 Bacterial–Fungal Interactions at the Airway Mucosa and Implications for Chronic Rhinosinusitis


*Emily Cope, USA*
16:35S22.2 Respiratory Mycobiome—A Clinical Perspective


*Robert Krause, FECMM, Austria*
16:55S22.3 Characterization of Indoor Dust Microbiota in Homes of Asthma and Non-Asthma Patients


*Jean-Pierre Gangneux, FECMM, France*
17:15S22.4 Understanding the Role of the Gut Mycobiome in Health and Disease


*Maria Vehreschild, FECMM, Germany*
17:35S22.5* An ECMM-ESCMID Survey on Goals and Practices for Mycobiota Characterization Using Next-Generation Sequencing in European Laboratories


*Jean-Pierre Gangneux, FECMM, France*

**Symposium 23**


**The Funny Life of C. albicans in the Oral Tract**



*Chairs: Marie-Elisabeth Bougnoux, France & Anna Maria Tortorano, Italy*
16:15S23.1 Within-Host Genomic Diversity of Candida albicans in Healthy Carriers


*Marie-Elisabeth Bougnoux, France*
16:35S23.2 Activation of Oral Immune Responses by C. *albicans*


*Julian Naglik, UK*
16:55S23.3 Modulation of the Fungal–Host Interaction by the Intraspecies Diversity of *C. albicans*


*Dominique Sanglard, Switzerland*
17:15S23.4 Intestinal Th17 Drives Airway Inflammation in ABPA


*Petra Bacher, Germany*
17:35S23.5* Persistence of Clonal Azole-Resistant Isolates of *Candida albicans* from a Patient with Chronic Mucocutaneous Candidiasis in Colombia


*Andres Ceballos-Garzon, Colombia*

**Symposium 24**


**Fungal Neglected Tropical Diseases**



*Chairs: Flavio Queiroz-Telles, Brazil & Leila Lopes Bezerra, Brazil*
16:15S24.1 Mycetoma


*Wendy van de Sande, FECMM, The Netherlands*
16:35S24.2 Chromoblastomycosis


*Flavio de Queiroz-Telles, FECMM, Brazil*
16:55S24.3 Sporotrichosis


*Leila Lopes Bezerra, Brazil*
17:15S24.4 Novel Diagnostic and Treatment Strategies for the Southeast Asian Fungus *Talaromyces marneffei*


*Thuy Le, USA*
17:25
Discussion

**MONDAY 14 OCTOBER 2019**

**08:00–8:45**


**Meet the Expert Sessions 11–15**
08:00M11 HIV


*Blandine Denis, France & Nelesh Govender (FECMM), South Africa*
08:00M12 Environment


*Ester Segal, FECMM, Israel & Raquel Sabino, FECMM, Portugal*
08:00M13 Diagnostics/Imaging


*Frédéric Lamoth, FECMM, France & Christopher Thornton, UK*
08:00M14 Resistance


*Maiken Arendrup, Denmark & Lewis White, FECMM, UK*
08:00M15 Registries


*Danila Seidel, Germany & Oscar Marchetti, Switzerland*

**08:50–10:00**


**Plenary Session 6—Management of IFD in Pediatrics (EPMyN)**



*Chairs: Adilia Warris, FECMM, United Kingdom & Andreas Groll, FECMM, Germany*
08:50PS6.1 Biomarker-Based Diagnostic Work-Up of IFD in Immunocompromised Pediatric Patients


*Thomas Lehrnbecher, Germany*
09:20PS6.2 Pre-Emptive Versus Empiric Antifungal Therapy in Children with Cancer


*Maria Elena Santolaya, Chile*
09:50PS6.3* Invasive Mucormycosis in Children with Hematological Malignancies and Solid Tumors: Report from the Infection Working Group of the Hellenic Society of Pediatric Hematology Oncology (2008–2017).


*Emmanuel Roilides, Greece*

**10:30–11:15**


**Plenary Session 7—Superficial and Dermatology**



*Chairs: Arnaldo Colombo, FECMM, Brazil & Yee-Chun Chen, Taiwan, Province of China*
10:30PS7.1 When the Skin Is the Portal of Invasive Infections


*Yee-Chun Chen, Taiwan, Province of China*
11:00PS7.2 When the Skin Talks for Systemic Infections


*Fanny Lanternier, FECMM, France*

**11:15–12:30**


**Plenary Session 8—New (Antineoplastic) Drugs, New Risks**



*Chairs: Livio Pagano, FECMM, Italy & Johan Maertens, FECMM Belgium*
11:30PS8.1 In Hematology


*Alessandro Busca, Italy*
12:00PS8.2 In Oncology


*Lubos Drgona, FECMM, Slovakia*

**12:30**


**Closing TIMM-9**


## Honorary Lectures

### E. Drouhet lecture EDL. How to convince European institutions that Medical Mycology is a major science

LatgéJ.-P.University of crete, Heraklion, Greece

How to convince Europe that Medical Mycology is a major science

Biochemistry; Molecular biology; Immunology

There are many reasons for flying the flag of Medical Mycology in medicine but also in science in Europe. First, morbidity and mortality due to fungi is much higher than the one due to tuberculosis and malaria in Europe. Second, essential advances in immunology such as TLR or C-type lectins, had their root in the analysis of the host response against fungal infections. Fungi continue to be models of choice for deciphering new immunological pathways. Cell wall which is a unique feature of these eukaryotic microorganisms, has been instrumental on this medical theme but also on the finding of new essential enzymes in glycobiology. Third, Mycobiota is a majority underrecognized and understudied component of the microbiota which is more and more recognized as an important constituent of human health. Fourth, analysis of fungal biochemical and molecular pathways can help understanding human diseases or metabolic dysregulations or stem cell dormancy. Finally, more than other microbes, fungi have to be loved for their natural beauty. All these cases will be illustrated with examples taken from the study of Aspergillus fumigatus which has been my “baby” for the last 30 years.

## Plenary session 1—Tropical

### PS1.2. Emergomycosis

GovenderN.National Institute for Communicable Diseases, Johannesburg, South Africa

Five species of thermally-dimorphic fungi within a new genus *Emergomyces* cause a disseminated mycosis among immunocompromised persons. Distinct from the closely-related *Emmonsia* and *Blastomyces* genera, *Emergomyces* strains have only been isolated from human infections and all species produce yeast cells (usually <5 µm diameter and with narrow-based budding) in the thermotolerant phase. The type species, *Emergomyces pasteurianus* was first described from a case in 1992 and has an apparently cosmopolitan distribution with cases diagnosed in Europe, Africa and Asia. The other four species were described to have emerged over the last decade, coinciding with increasing use of molecular diagnostic techniques in clinical and research laboratories, and may be geographically restricted. Overall, *Emergomyces africanus* has been implicated in the largest number of reported cases of emergomycosis. Restricted to southern Africa and first described by Kenyon et al in 2013, *E. africanus* causes a multi-system disease among persons living with advanced HIV disease. Systemic infection is presumed to occur following inhalation of air-borne conidia from a soil reservoir, with a subsequent temperature-mediated phase transition to a yeast form and dissemination through the reticuloendothelial system among immunocompromised individuals. Most cases are diagnosed by conventional culture of blood, tissues and fluids and/or histopathological tissue examination, both of which require technical expertise. Limited pulmonary disease is probably under-diagnosed in resource-limited settings; this has only been described to occur in the single case of *Emergomyces europaeus* infection. The full spectrum of clinical infection and prevalence in different populations could potentially be determined by better use of non-culture-based methods, including antigen and PCR assays, in clinical settings and for epidemiological surveillance. For instance, *E. africanus* is known to cross-react with a commercially-available *Histoplasma* galactomannan antigen assay and *Emergomyces canadensis* with a commercial DNA probe for *Blastomyces dermatitidis.* Although the attributable mortality has not been defined, the crude mortality in a South African case series was approximately 50%. Screening for emergomycosis among high-risk patients in endemic areas could detect active disease earlier and thus reduce mortality associated with late presentation. Treatment recommendations for emergomycosis are the same as for patients with disseminated histoplasmosis and are based only on observational data.

## Plenary session 2—Highlights on fungal biology

### PS2.1. Candida albicans genome diversity: mechanisms and consequences

D’EnfertC.Fungal Biology and Pathogenicity, Institut Pasteur, INRA, Paris, France

The fungal pathogen *Candida albicans* shows significant diversity at the genetic and phenotypic levels. Here, I will review our current knowledge of the *C. albicans* diploid genome and its variability, the genetic structure of the *C. albicans* population and the mechanisms that are involved in *C. albicans* genome dynamics, with a focus on the parasexual cycle and loss-of-heterozygosity events. I will further explore the impact of genetic diversity and genome dynamics on *C. albicans* phenotypic diversity. Finally, I will discuss how our current knowledge of *C. albicans* genetic diversity could be leveraged in the future in order to get insights in the mechanisms underlying important biological attributes that are subject to variations across *C. albicans* isolates.

### PS2.2. Metabolic regulation of innate immunity to Aspergillus fumigatus

GonçalvesS.CunhaC.CarvalhoA.Life and Health Sciences Research Institute (icvs), University of Minho, Braga, Portugal

The reprogramming of cellular metabolism was recently recognized as a fundamental mechanism through which innate immune cells meet the energetic and anabolic needs during host defense against invading pathogens. Sensing of microbial ligands by macrophages drives the upregulation of glycolysis, which delivers a rapid source of energy to support antimicrobial functions and the production of cytokines and other inflammatory mediators. The stimulation of immune cells with structural components of the fungal cell wall, such as β-1,3-glucan, has been demonstrated to promote the metabolic and functional reprogramming of immune cells during infection with the yeast *Candida albicans*. However, how the immune response to other fungi, such as *Aspergillus fumigatus*, is also regulated at the metabolic level and whether other cell wall constituents also participate in these signaling events remains unknow. Fungal melanin is a major virulence factor, endowing *A. fumigatus* with the ability to survive killing by phagocytes. By resorting to in vitro and in vivo models of infection and patients suffering from invasive pulmonary aspergillosis (IPA), and using different pharmacological and genetic tools to manipulate both the host and the pathogen, we reveal a novel mechanism whereby fungal melanin is perceived by the host to regulate adequate immunometabolic responses and susceptibility to infection. Specifically, we demonstrate that the host counters the immune inhibitory mechanisms deployed by fungal melanin, by “sensing” its removal during intracellular swelling inside the phagosome, and using these signals to rewire cellular metabolism towards enhanced glycolysis and promote antifungal immune responses. The contribution of glucose homeostasis to human antifungal immunity is further supported by genetic variation in glycolytic regulators that act as cytokine quantitative trait loci and predispose patients at-risk to IPA. Given the current limitations in diagnosis and therapy of fungal diseases as well as concerns over the emergence of antifungal resistance, these results may contribute towards the design of innovative therapeutic approaches or metabolic adjuncts to reorient host cells towards immune protection against IPA.

### PS2.3. Lineage-specific behavioural differences in isolates of Candida auris

BormanA.^1^SzekelyA.^1^MajorosL.^2^LockhartS.^3^HoogG.S. De^4^Ben-AmiR.^5^JohnsonE.^1^1Public Health England UK National Mycology Reference Laboratory, Bristol, United Kingdom2Department of Medical Microbiology, University of Debrecen, Debrecen, Hungary3Centres For Disease Control and Prevention, Atlanta, United States of America4Westerdijk Institute, Utrecht, Netherlands5Tel Aviv Sourasky Medical Centre, Tel Aviv, Israel

**Objectives:***Candida auris* is a serious nosocomial health risk, with widespread outbreaks in hospitals worldwide. Sequence analyses of outbreak isolates revealed that *C. auris* has simultaneously emerged as multiple distinct, geographically restricted clonal lineages. We previously reported multiple independent introductions of *C. auris* isolates from at least three of these lineages (S. Asia, S. Africa and Japan/Korea) into hospitals across the UK, and that isolates circulating in the UK displayed two different cell phenotypes which correlated with differences in virulence in *Galleria mellonella*. The present study aimed to more fully characterise clade-specific differences in the behaviour of *Candida auris* isolates. **Methods:** Multiple independent *Candida auris* isolates corresponding to 4 of the known geographically-restricted clonal lineages (S. Asia, S. Africa, S. America/Israeli and Japanese/Korean) were subjected to extensive phenotypic characterisation (cellular morphology, actidione tolerance, virulence in insect [*Galleria mellonella*] and mammalian [mouse] infection models, antifungal susceptibility profiles, antifungal drug-mediated morphological changes) using well-established methodologies. **Results:** Significant clade-dependent differences in *C. auris* isolate behaviour were noted with respect to all of the biological features examined, including cellular morphology, resistance to actidione, virulence, antifungal susceptibility and morphological responses to antifungal drug exposure. Several of these differences may be correlated with previously described differences in cellular aggregation capacity, *ERG11* resistance mutations and gene copy numbers, biofilm formation and efflux pump activity. **Conclusion:** The present data demonstrate that “one size does not fit all for *Candida auris*” and that the behaviour of individual isolates is at least in part dependent on the clonal lineages from which they originate. Further studies will aim to elucidate whether such differences have clinical significance, and to attempt to establish why isolates of three of the principal clonal lineages (S. Asian, S. African, S. American/Israeli) have been reported from large scale nosocomial outbreaks but none to date have been attributed to isolates from the Japanese/Korean clade.

## Plenary session 3—One World One Guideline ECMM MSG-ERC (EFISG) ISHAM Guidelines initiative

### PS3.1. Global guideline for the diagnosis and management of mucormycosis: An initiative of the European Confederation of Medical Mycology in cooperation with the Mycoses Study Group Education and Research Consortium

CornelyO.A.^1^,^2^,^3^,^4^Alastruey-IzquierdoA.^5^ArenzD.^1^,^3^ChenS.C.-A.^6^DannaouiE.^7^HochheggerB.^8^,^9^HoeniglM.^10^,^11^JensenH.E.^12^LagrouK.^13^LewisR.^14^MellinghoffS.^1^,^3^MerM.^15^PanaZ.^16^SeidelD.^1^,^3^SheppardD.C.^17^WhabaR.^18^ChakrabartiA.^19^AkovaM.^20^AlanioA.^21^Al-HatmiA.^22^Arikan-AkdagliS.^23^BadaliH.^24^Ben-AmiR.^25^BonifazA.^26^BretagneS.^21^CastagnolaE.^27^ChayakulkeereeM.^28^ColomboA.^29^Corzo-LeónD.E.^30^DrgonaL.^31^GrollA.H.^32^GuineaJ.^33^HeusselC.-P.^34^IbrahimA.S.^35^KanjS.^36^KlimkoN.^37^LacknerM.^38^LamothF.^39^LanternierF.^40^Lass-FlörlC.^38^LeeD.-G.^41^LehrnbecherT.^42^LmimouniB.E.^43^MaresM.^44^MaschmeyerG.^45^MeisJ.^46^MeletiadisJ.^47^MorrisseyO.^48^NucciM.^49^OladeleR.^50^PaganoL.^51^PasqualottoA.^52^PatelA.^53^RacilZ.^54^RichardsonM.D.^55^RoilidesE.^16^RuhnkeM.^56^SeyedmousaviS.^24^,^57^,^58^SidharthanN.^59^SinghN.^60^SinkoJ.^61^SkiadaA.^62^SlavinM.^63^,^64^SomanR.^65^SpellbergB.^66^SteinbachW.^67^TanB.H.^68^UllmannA.J.^69^VehreschildJ.-J.^1^,^2^VehreschildM.J.G.T.^1^,^2^,^70^WalshT.^71^WhiteP.L.^72^WiederholdN.^73^ZaoutisT.^74^1Department I of Internal Medicine, University Hospital of Cologne, Cologne, Germany2German Centre for Infection Research (DZIF) partner site Bonn-Cologne, Cologne, Germany3Cecad Cluster of Excellence, University of Cologne, Cologne, Germany4Clinical Trials Center Cologne, University Hospital of Cologne, Cologne, Germany5Mycology Reference Laboratory, National Centre For Microbiology, Instituto de Salud Carlos III, Madrid, Spain6Centre For Infectious Diseases and Microbiology Laboratory Services, New South Wales Health Pathology, and The Department of Infectious Diseases, Westmead Hospital, School of Medicine, University of Sydney, Sydney, Australia7Unité De Parasitologie-mycologie, Service De Microbiologie, Université Paris-Descartes, Faculté de Médecine, APHP, Hôpital Européen Georges Pompidou, Paris, France8Radiology, Hospital São Lucas da Pontificia Universidade Catolica do Rio Grande do Sul (PUCRS), Porto Alegre, Brazil9Radiology, Universidade Federal de Ciências da Saúde de Porto Alegre (UFCSPA), Porto Alegre, Brazil10Section of Infectious Diseases and Tropical Medicine and Division of Pulmonology, Medical University of Graz, Graz, Austria11Division of Infectious Diseases, Department of Medicine, University of California San Diego, San Diego, United States of America12Faculty of Health and Medical Sciences, University of Copenhagen, Copenhagen, Denmark13Department of Microbiology, Immunology and Transplantation, Clinical Department of Laboratory Medicine and National Reference Center For Mycosis, KU Leuven, University Hospitals Leuven, Leuven, Belgium14Infectious Diseases Clinic, Sant’Orsola-Malpighi Hospital, Department of Medical and Surgical Sciences, University of Bologna, Bologna, Italy15Divisions of Critical Care and Pulmonology, Department of Medicine, Charlotte Maxeke Johannesburg Academic Hospital, Johannesburg, South Africa16Infectious Diseases Unit, 3rd Department of Paediatrics, Faculty of Medicine, Aristotle University School of Health Sciences, Hippokration General Hospital, Thessaloniki, Greece17Division of Infectious Diseases, Department of Medicine, Microbiology and Immunology, McGill University, Montreal, Canada18Department of General, Visceral and Cancer Surgery, University Hospital of Cologne, Cologne, Germany19Department of Medical Microbiology, Postgraduate Institute of Medical Education & Research, Chandigarh, India20Department of Infectious Diseases, Hacettepe University School of Medicine, Ankara, Turkey21Institut Pasteur, National Reference Center For Invasive Mycoses and Antifungals, Department of Mycology, Cnrs Umr2000, Parasitology-mycology Laboratory, Fernand Widal Hospitals, Assistance Publique-Hôpitaux de Paris (AP-HP), Université de Paris, Paris, France22Directorate General of Health Services, Ministry of Health, Ibri, Oman23Department of Medical Microbiology, Hacettepe University School of Medicine, Ankara, Turkey24Department of Medical Mycology/invasive Fungi Research Center (ifrc), School of Medicine, Mazandaran University of Medical Sciences, Sari, Iran25Infectious Diseases Unit, Tel Aviv Medical Center, Sackler Faculty of Medicine, Tel Aviv University, Tel Aviv, Israel26Dermatology Service & Mycology Department, Hospital General de México “Dr. Eduardo Liceaga”, Mexico City, Mexico27Infectious Diseases Unit, Istituto Giannina Gaslini Children’s Hospital, Genova, Italy28Department of Medicine, Faculty of Medicine, Siriraj Hospital, Mahidol University, Bangkok, Thailand29Special Mycology Laboratory, Division of Infectious Diseases, Department of Medicine, Universidade Federal de São Paulo (UNIFESP), Sao Paulo, Brazil30Department of Epidemiology and Infectious Diseases, Hospital General Dr. Manuel Gea González, Mexico City, Mexico31Oncohematology Clinic, Faculty of Medicine, Comenius University and National Cancer Institute, Bratislava, Slovak Republic32Infectious Disease Research Program, Department of Paediatric Hematology/oncology and Center For Bone Marrow Transplantation, University Children’s Hospital Münster, Münster, Germany33Clinical Microbiology and Infectious Diseases, Hospital General Universitario Gregorio Marañón, Madrid, Spain34Diagnostic and Interventional Radiology, Thoracic Clinic, University Hospital Heidelberg, Heidelberg, Germany35Division of Infectious Diseases, Los Angeles Biomedical Research Institute, Harbor-University of California, Torrance, United States of America36Department of Internal Medicine, Division of Infectious Diseases, American University of Beirut Medical Center, Beirut, Lebanon37Department of Clinical Mycology, Allergology and Immunology, North Western State Medical University, St. Petersburg, Russian Federation38Division of Hygiene and Medical Microbiology, Department of Hygiene, Microbiology and Public Health, Medical University Innsbruck, Innsbruck, Austria39Infectious Diseases Service, Department of Medicine and Institute of Microbiology, Lausanne University Hospital, Lausanne, Switzerland40Institut Pasteur, National Reference Center for Invasive Mycoses and Antifungals, Department of Mycology, CNRS UMR2000, Paris Descartes University, Necker-Enfants Malades University Hospital, Department of Infectious Diseases and Tropical Medicine, Centre, Paris, France41Division of Infectious Diseases, Department of Internal Medicine, Catholic Hematology Hospital, College of Medicine, The Catholic University of Korea, Seoul, Korea, Democratic People’s Republic of42Division of Paediatric Haematology and Oncology, Hospital for Children and Adolescents, Johann Wolfgang Goethe-University, Frankfurt, Germany43School of Medicine and Pharmacy, University Mohammed the fifth, Rabat, Morocco44Laboratory of Antimicrobial Chemotherapy, Ion Ionescu de la Brad University, Iasi, Romania45Department of Hematology, Oncology and Palliative Care, Klinikum Ernst von Bergmann, Potsdam, Germany46Department of Medical Microbiology and Infectious Diseases, Centre of Expertise In Mycology, Radboudumc/Canisius Wilhelmina Hospital, Nijmegen, Netherlands47Clinical Microbiology Laboratory, Attikon University Hospital, National and Kapodistrian University of Athens, Department of Medical Microbiology and Infectious Diseases, Erasmus Medical Center, Athens, Greece48Department of Infectious Diseases, Alfred Health & Monash University, Melbourne, Australia49Department of Internal Medicine, Universidade Federal do Rio de Janeiro, Rio de Janeiro, Brazil50Department of Medical Microbiology & Parasitology, College of Medicine, University of Lagos, Faculty of Biology, Medicine and Health, The University of Manchester, Manchester, United Kingdom51Department of Hematology, Fondazione Policlinico Universitario A. Gemelli –irccs–, Universita Cattolica del Sacro Cuore, Rome, Italy52Federal University of Health Sciences of Porto Alegre, Hospital Dom Vicente Scherer, Porto Alegre, Brazil53Infectious Diseases Clinic, Vedanta Institute of Medical Sciences, Ahmeddabad, India54Department of Internal Medicine, Hematology and Oncology, Masaryk University and University Hospital Brno, Brno, Czech Republic55UK NHS Mycology Reference Centre, Manchester University NHS Foundation Trust, Manchester, United Kingdom56Department Haematology / Oncology, Paracelsus Klinik, Osnabrück, Germany57Center of Expertise in Microbiology, Infection Biology and Antimicrobial Pharmacology, Tehran, Iran58Molecular Microbiology Section, Laboratory of Clinical Immunology and Microbiology, National Institute of Allergy and Infectious Diseases, National Institutes of Health, Bethesda, United States of America59Department of Hemato Oncology, Amrita Institute of Medical Sciences, Amrita Viswa Vidyapeetham University, Kochi, India60Division of Infecatious Diseases, University of Pittsburgh Medical Center and VA Pittsburgh Helthcare System, Infectious Diseases Section, University of Pittsburgh, Pittsburgh, United States of America61Infectious Diseases Unit, Szent Istvan and Szent Laszlo Hospital, Budapest, Hungary62Department of Infectious Diseases, Laiko General Hospital, National and Kapodistrian University of Athens, Athens, Greece63University of Melbourne, Melbourne, Australia64The National Centre for Infections in Cancer, Peter MacCallum Cancer Centre, Melbourne, Australia65Dept. of Medicine, P. D. Hinduja Hospital & Medical Research Centre, Mumbai, India66Los Angeles County + University of Southern California (LAC+USC) Medical Center, Los Angeles, United States of America67Division of Pediatric Infectious Diseases, Department of Pediatrics, Duke University Medical Center, Durham, United States of America68Department of Infectious Diseases, Singapore General Hospital, Singapore, Singapore69Department For Internal Medicine Ii, University Hospital Würzburg, Würzburg, Germany70Department of Internal Medicine, Infectious Diseases, Goethe University Frankfurt, Frankfurt, Germany71Departments of Medicine, Pediatrics, Microbiology & Immunology, Weill Cornell Medicine, and New York Presbyterian Hospital, New York, United States of America72Public Health Wales Microbiology Cardiff, UHW, Cardiff, United Kingdom73Fungus Testing Laboratory, The University of Texas Health Science Center at San Antonio, San Antonio, United States of America74Division of Infectious Diseases, The Children’s Hospital of Philadelphia, Philadelphia, United States of AmericaBackground Mucormycosis is a difficult to diagnose rare disease with high morbidity and mortality. Diagnosis is often delayed, and disease tends to progress rapidly. Urgent surgical and medical intervention is lifesaving. Guidance on the complex multidisciplinary management has potential to improve prognosis, but approaches differ between health care settings. Methods from January 2018, authors from 33 countries in all United Nations regions analysed the published evidence on mucormycosis management and provided consensus recommendations addressing differences between the regions of the world as part of the “One World One Guideline” initiative of the European Confederation of Medical Mycology (ECMM). The author group based in 17 time zones, relied on electronic media including video tutorial on methodology, and central document repository with several daily updates. Results Signs and symptoms of mucormycosis depend on organ patterns and underlying conditions. Diagnostic management does not differ greatly between world regions. Upon suspicion of mucormycosis appropriate imaging is strongly recommended to document extent of disease and is followed by strongly recommended surgical intervention. First-line treatment with high-dose liposomal amphotericin B is strongly recommended, while intravenous isavuconazole and intravenous or delayed release tablet posaconazole are recommended with moderate strength. Both triazoles are strongly recommended salvage treatments. Amphotericin B deoxycholate is recommended against, because of substantial toxicity, but may be the only option in resource limited settings. Conclusion Management of mucormycosis depends on recognising disease patterns and on early diagnosis. Limited availability of contemporary treatments burdens patients in low and middle income settings. Areas of uncertainty were identified and future research directions specified.

### PS3.3. Rare moulds: Clinical Practice Guideline of the ECMM and the MSG-ERC

HoeniglM.^1^,^2^1Division of Infectious Diseases, University of California, San Diego, United States of America2Section of Infectious Diseases and Tropical Medicine and Division of Pulmonology, Medical University of Graz, Graz, AustriaIn the context of a growing population of immunocompromised patients at risk for opportunistic infections, prevalence of invasive mould infections, including moulds other than *Aspergillus* and Mucorales, are on the rise. While new diagnostic and therapeutic options are now available to tackle rare invasive mould infections, up to date guidance for the correct utilization in the clinical setting is urgently needed. On that background, ECMM together with MSG-ERC set out an unprecedented orphan diseases guidance initiative involving all disciplines involved in diagnosis and treatment of rare mould infections. Utilizing the global network of the ECMM Academy and the ECMM Excellence Centers, clinicians, microbiologists and other medical professionals from around the world will be invited by ECMM to contribute their expertise to the project "One World-One Guideline". The rare mould guideline covers diagnosis and treatment of infections caused by Fusarium, Lomentospora, Phaeohyphomycetes/dematiaceous fungi/black fungi, Rasamsonia, Scedosporium, Schizophyllum and other basidiomycetes, Scopulariopsis, Penicillium, and Talaromyces other than marneffei. The guideline will include taxonomy and epidemiology and give detailed recommendations regarding diagnosis and clinical management of these rare mould infections for adults and the pediatric population. The guideline follows the structure and definitions of the ESCMID/ECMM guidelines on invasive fungal infection, and the ECMM MSG-ERC guideline on mucormycosis, which are in accord with the GRADE and AGREE systems.

## Plenary session 5—ICU—candida infections/Breaking news from Hematology and ICU

### PS5.1. Abdominal Candidiasis in ICU patients

KöhlerP.Department I for Internal Medicine, European Excellence Center for Medical Mycology (ecmm), Germany and Cecad Cluster of Excellence, University of Cologne, Cologne, GermanyWith increased admission rates to intensive care unit and high morbidity and mortality in the presence of severe sepsis or septic shock abdominal candidiasis is a frequent complication in surgical patients. In the majority of cases *Candida* species are part of polymicrobial infections after upper gastrointestinal tract perforation, anastomotic leaks after bowel surgery, acute necrotizing pancreatitis or peritoneal dialysis. Further risk factors comprise premorbid conditions, total parenteral nutrition, and previous antibiotic therapy among others. Blood cultures are often negative and the serum beta-d-glucan assay can support diagnosis in patients with suspected intraabdominal candidiasis. The diagnosis is achieved by aspiration of fluid under ultrasound or CT guidance or at the time of surgery. Current treatment strategies comprise both surgical intervention and antifungal therapy, preferably with an echinocandin. Therapy should continue for at least two weeks and often longer, until the abscess and all signs and symptoms of peritonitis are resolved.

### PS5.2. Are the new management strategies useful?

ColomboA.Medicine, Federal University of São Paulo, São Paulo, BrazilAlong the last decades, we faced a substantial progress in the clinical validation of new diagnostic and therapeutic tools for treating patients with invasive Candidiasis (IC). However, we still have several gaps in knowledge that may mitigate our results in the clinical management of complex patients with invasive candidiasis. We selected 3 main topics to be addressed along this presentation: (1) How Candida Biomarkers might be utilized in the clinics, (2) Should we continuous to order universal screening of eye infection and endocarditis for candidemic patients? (3) Concerns in the clinical management of neutropenic patients with candidemia.

### PS5.3. Breaking news from Hematology and ICU: CNS infections

CandoniA.University Hospital, Asuiud, Division of Hematology and Stem Cells Transplantation, Udine, ItalyFungal Infections of the Central Nervous System (IFI-CNS) are rare life-threatening infections in hematologic patients and their management remains a challenge despite availability of new diagnostic techniques and novel antifungal agents (**1**–**4**). In addition, analyses of large cohort of patients focusing on these rare IFIs, are still lacking. The most important causative agent remains *Aspergillus* species and a concomitant extra-CNS involvement (mainly lung) is common. Recent reports highlight that the fungal biomarkers in CSF could be highly informative reinforcing that diagnostic lumbar puncture should be performed when IFI-CNS is suspected, enabling diagnosis confirmation and prompt initiation of targeted therapy (**2**). Surgical approach of this complications is feasible only in a minority of cases (those with focal, cortical-subcortical lesions and no severe thrombocytopenia), and, even with a broad spectrum of antifungal drugs, overall response rate remains poor (less than 40%) with a 12-months survival probability less than 30% (**1**–**4**). Availability of new drugs, with better CNS permeability and less interaction with immunosuppressive agents and chemotherapy (such as Isavuconazole), and prompt diagnostic work-up (high diagnostic value of CSF biomarkers) can guide a rapid, specific and aggressive therapy (including combination antifungal therapy ± surgery) to further improve outcomes of IFI-CNS in hematologic patients (**1**–**4**). (1) Pagano L, Caira M, Falcucci P, Fianchi L. Fungal CNS infections in patients with hematologic malignancy*. Expert Rev Anti Infect Ther*. 2005;3:775–85. (2) Candoni A, Klimko N, Busca A, et al. Fungal infections of the central nervous system and paranasal sinuses in onco-haematologic patients. Epidemiological study reporting the diagnostic-therapeutic approach and outcome in 89 cases. *Mycoses.* 2019;62(3):252–260. (3) Schwartz S, Kontoyiannis DP, Harrison T, Runke M. Advances in the diagnosis and treatment of fungal infections of the CNS. *Lancet Neurology.* 2018;17:362–372. (4) McCarthy M, Rosengart A, Schuetz AN, et al. Mold Infections of the Central Nervous System. *N Engl J Med*. 2014; 10(371-2): 150–160.

## Plenary session 6—Management of IFD in paediatrics (EPMyN)

### PS6.1. Biomarker based diagnostic work-up of IFD in immunocompromised paediatric patients

LehrnbecherT.Pediatric Hematology and Oncology, University of Frankfurt, Frankfurt, GermanyImmunocompromised pediatric patients such as children undergoing therapy for hematological malignancies or receiving allogeneic hematopoietic stem cell transplantation are at high risk for invasive fungal diseases, which are a major cause of morbidity and mortality in these patient populations. Unfortunately, clinical signs and symptoms of invasive fungal disease are mostly unspecific, which makes early diagnosis difficult. However, early diagnosis and prompt treatment of invasive fungal disease is associated with better outcome. Non-invasive antigen-based assays such as galactomannan (GM) or β-d-glucan and molecular biomarkers in peripheral blood have the potential to indicate invasive fungal disease prior to the development of clinical symptoms, and may therefore be helpful in the decision to institute and choose antifungal compounds and also to guide duration of therapy. When GM in blood is used as a diagnostic test in neutropenic children with prolonged febrile neutropenia unresponsive to broad-spectrum antibiotics or in children with a pulmonary infiltrate in the CT scan, sensitivity and specificity of the assay do not significantly differ between children and adults. However, the utility of the assay is clearly limited by the poor positive predictive value and the fact that despite the high negative predictive value of the test, a negative GM assay does not rule out non-*Aspergillus* molds. Data on β-d-glucan in immunocompromised children are too limited to give a final recommendation on its use, and similarly, the results of PCR testing in blood are variable and do not allow a firm conclusion on its utility. However, despite these limitations, biomarkers in blood may help to plan invasive diagnostic procedures such as broncho-alveolar lavage (BAL) or bioptic procedures. As compared to blood, both galactomannan and PCR testing in BAL lavage seem to be superior, but again, further studies in the pediatric population are warranted. Regarding the value of biomarker in CNS samples such as cerebrospinal fluid, little is known in both pediatric and adult patients. Further pediatric studies are warranted to evaluate whether the combination of diagnostic methods can improve sensitivity and specificity, but in addition, new diagnostic tools are needed that will improve the early and reliable diagnosis of invasive fungal disease in children.

### PS6.2. Pre-emptive versus empiric antifungal therapy in children with cancer

SantolayaM.E.Hospital de niños Dr. Luis Calvo Mackenna, Santiago, ChileInvasive fungal disease (IFD) causes significant morbidity and mortality in paediatric cancer patients with high-risk febrile neutropenia (HRFN), along with high utilization of resources for prevention, diagnosis and treatment. Early diagnosis of IFD and prompt implementation of aggressive antifungal treatment have proven to be critical for patient survival. Nevertheless, early identification of the causal pathogen of an IFD continues to be difficult. The classic approach is currently based on clinical, imaging, microbiological (cultures from sterile sites) and histological studies. Major advances for early diagnosis of IFD have been made by the development of non-culture assays such as detection of galactomannan (GM) antigen, (1-3)-b-d-glucan antigen detection and nucleic acid detection, by PCR techniques. Despite these advances, IFD diagnosis continues to be a challenge and current recommendations propose to initiate empirical antifungal therapy in IFD high-risk paediatric patients with persistent (96 h) fever and neutropenia that are unresponsive to broad-spectrum antibacterial agents. The downside of this approach is the overtreatment of patients meeting the above criteria but who do not have an IFD, leading to an increase in adverse events, prolonged hospitalizations and elevated costs associated with the use of antifungal drugs. A more reasonable approach in cancer subjects would be to consider early identification of patients at high risk of IFD, application of a complete screening diagnosis strategy followed by a rational approach to antifungal therapy based on results of this early and extensive diagnostic workup, adopting a more selective preemptive treatment strategy in patients with persistent fever and neutropenia. We performed a prospective, multicenter study in children with cancer and persistent HRFN, aimed to determine the efficacy of pre-emptive treatment compared with current standard empirical antifungal treatment. In our study, pre-emptive antifungal therapy was as effective as empirical antifungal therapy, with a significant reduction in antifungal use. A reduction of antifungal use, based on stringent diagnostic criteria, could favor the adoption of evidence-based management strategies in this population. This approach requires optimal laboratory support with rapid turnaround response. Introduction of non-culture-based diagnostic techniques into clinical practice could contribute to better management of these patients, favoring the possibility of patient-based individualized therapy. Active monitoring and early diagnostic workup is a necessary step prior to proposing an evidence-based management strategy. In our experience, 58% of patients of the pre-emptive group did not receive antifungal therapy, with similar clinical outcome to the empirical group, with the aim of reserving antifungal therapy for the subset of patients who have early evidence of IFD by careful clinical, laboratory, imaging and microbiological assessment. The main findings of our study lead us to propose a step forward in the rational approach to treating children with cancer focusing on one yet-unresolved issue in the management of the patients: adoption of a more selective pre-emptive antifungal treatment strategy in children with prolonged fever and neutropenia.

### PS6.3. Invasive mucormycosis in children with hematological malignancies and solid tumors: Report from the Infection Working Group of the Hellenic Society of Pediatric Hematology Oncology (2008-2017)

AntoniadiC.^1^LialiasI.^2^TsipouH.^3^KazakouN.^4^IosifidisE.^2^PapakonstantinouE.^5^PolychronopoulouS.^1^KattamisA.^3^RoilidesE.^2^TragiannidisA.^4^1Department of Pediatric Hematology-oncology, "Agia Sofia" Children’s Hospital, Athens, ATHENS, Greece23rd Pediatric Department, Aristotle University of Thessaloniki, Hippokration Hospital, THESSALONIKI, Greece3Pediatric Hematology-oncology Unit, 1st Pediatric Department, National and Kapodistrian University of Athens, "Agia Sofia" Children’s Hospital, ATHENS, Greece4Haematology-oncology Unit, 2nd Pediatric Department, Aristotle University of Thessaloniki, AHEPA Hospital, Thessaloniki, Greece5Department of Pediatric Hematology-oncology, Hippokration Hospital, THESSALONIKI, Greece

**Objectives:** Mucormycosis is an invasive, life-threatening fungal infection that mainly affects immunocompromised hosts. Zygomycetes can invade virtually any tissue or organ, resulting in a variety of clinical presentations. The Infection Working Group (IWG) of the Hellenic Society of Pediatric Hematology-Oncology (HELSPHO) collected and analyzed data of pediatric mucormycosis cases from 7 Hematology-Oncology Departments within 2008–2017.

**Methods:** Six cases of proven invasive mucormycosis were reported retrospectively (female/male: 2/4, median age: 9.6 years, range: 2–15 years) during chemotherapy for hematologic malignancies and solid tumors within 2008–2017.

**Results:** Among the 6 children with invasive mucormycosis, three presented with acute lymphoblastic leukaemia (ALL), one with acute myeloid leukemia (AML), one with neuroblastoma and one child with brain tumor, as the primary underlying malignancy. In 4/6 children (66%), mucormycosis occurred within the first 20 days from the diagnosis of the underlying disease. One child with ALL developed mucormycosis 4 months since the initial diagnosis, with prior invasive aspergillosis two months earlier treated with voriconazole. Four out of 6 patients had received prolonged corticosteroid treatment and 3/6 had severe neutropenia with a neutrophil count <500 μL at the time of diagnosis. Four out of 6 patients (66%) had received intensive chemotherapy within the last 4 weeks and all patients had been hospitalized for at least 20 days. All patients presented with fever (100%), 4/6 with pain (66%), 3/6 with palpable mass (50%) (one at the left forearm and two at the orbit) and 3 patients presented with central nervous system symptoms (seizures, headache, and blur of vision) (50%). Primary infection sites were combined paranasal sinus with orbital involvement (2), central nervous system (1), occipital area (1), orbital involvement (1) and forearm (1). The diagnosis of mucormycosis was documented by histology and mycology (PCR or culture) in all patients (2 *Rhizopus arrhizus*,1 *Mucor* spp., 1 *Absidia* spp., 2 unidentified Mucorales). Dissemination was defined by clinical signs, imaging studies and histology in 4/6 (66%) patients. Four (4) out of 6 patients had received prophylactic treatment with antifungal agents (2/4 patients received micafungin and 2/4 l-Amphotericin B). Therapeutically, all patients received l-Amphotericin B (l-AmB) (median dose: 7.5mg/kg, range: 3-10 mg/kg). Two (2) patients received combined antifungal treatment with l-AmB and caspofungin, one (1) l-AmB and voriconazole, one (1) l-AmB and posaconazole and one (1) l-AmB, voriconazole and caspofungin. Three (3) patients received maintenance treatment with posaconazole. Surgical excision was performed in 4/6 cases (66%). Two out of 6 patients (33%) died, after 6 and 12 months, respectively.

**Conclusion:** Pediatric invasive mucormycosis mainly presents as paranasal sinus and orbital disease and is still related to high mortality rates. Our data demonstrated that the disease occurs early after initiation of intensive chemotherapy and febrile neutropenia and prolonged corticosteroids are the main predisposing factors. Early recognition and prompt intervention with combined prolonged antifungal and surgical treatment may rescue the patients and improve overall survival.

## Plenary session 8—New (antineoplastic) drugs, new risks

### PS8.1. New antineoplastic drugs, new risks in hematology

BuscaA.Division of Hematology, AOU Citta della Salute e della Scienza, Torino, Turin, ItalyOver the last 10 to 15 years, therapeutic options for patients with hematologic malignancies have largely increased with the growth of new immunotherapeutic agents. At least two issues regarding invasive fungal infections (IFI) and the new immunotherapeutic agents should be addressed. First, there are relevant pharmacokinetic interactions of the new targeted therapies with coadministered mold active azoles, namely posaconazole and voriconazole that are strong inhibitors of CYP3A4. Awareness of these interactions is of paramount importance for optimization of the treatment of patients, since we are asked to monitor for potential toxicity, or to modulate the dose of the drugs or rather to avoid the combination and consider alternative therapy. Second, is the risk of IFI increased with the use of the new agents? For many of these, for instance monoclonal antibodies (MoAb), bcl-2 inhibitors (i.e. venetoclax) and tyrosine kinase inhibitors (TKI) (i.e. sorafenib and midostaurin) when used for the treatment of patients with acute leukemia, it is hard to realize whether they have an additive risk to the underlying disease: indeed, there are very few information respect the incidence of IFI based on the clinical trials published so far. By contrast, several studies raised concern that the Bruton’s tyrosine kinase inhibitor Ibrutinib may increase the risk for IFI in patients with lymphomas. In this respect, different mechanisms by which Ibrunitib may favor the occurrence of IFI have been described, including alveolar macrophage, neutrophil, T-cell, and platelet function as well as alterations of cytokine environment. A recent study of Ibrutinib treatment for primary CNS lymphoma reported a 39% rate of invasive aspergillosis in patients concurrently receiving steroids even in absence of neutropenia, while in other reports the incidence appears to be much lower in the 3–4% range. Conversely, the risk of IFI with the use of Idelalisib, another B-cell receptor inhibitor, seems to be restricted to *Pneumocystis jirovecii* pneumonia only. Until more detailed epidemiological data will be available, anti-mold prophylaxis is not recommended for patients receiving Ibrutinib or Idelalisib. Chimeric antigen receptor (CAR) T cell therapy is an innovative strategy to harness the immune system to treat patients with diffuse large B cell lymphoma (DLBCL). CAR-T cells are T lymphocytes genetically engineered to express a tumor-targeting receptor, directing T cells to bind to a tumor-associated antigen, namely the CD19 in the case of DLBCL. A recent study evaluated infectious complications in 133 patients with ALL and lymphomas treated with CD19 CAR-T cells: IFI occurred in 5% of the patients during the first 28 days after CAR-T cell infusion, all of whom had severe cytokine release syndrome (CRS) or neurotoxicity requiring tocilizumab and/or corticosteroids. CRS severity was the only factor after CAR-T cell infusion associated with infection in a multivariable analysis. In summary, the risk of IFI in patients receiving targeted therapies needs to be defined by further experience in clinical practice and ongoing research trials. Preventive strategies of IFI in patients managed long term with immunotherapy remain at the present a challenge.

### PS8.2. In oncology

DrgonaL.Department of Oncohematology, Comenius University and National Cancer Institute, Bratislava, Slovak RepublicThe field of new antineoplastic therapeutical agents is increasing rapidly over the last decade. New drugs in oncology are basically considered as targeted ones due to their mechanism of action. These drugs directly target the cells or pathways involved in pathophysiology of neoplastic disease. Cell surface receptors, cytokines, immunoglobulines, intracellular enzymes are the most common targets involved in the therapy. Other drugs are focusing on the patient’s own immune system with the aim to boost the responsible cells to fight against the disease. The new drugs have different mechanisms of action resulted in less “classical“ toxicity known from chemotherapy agents. The adverse events after the targeted therapy are mainly caused by interaction with the immune system—targeted sites are often key elements of physiological processes like cell cycle control. This may have an influence on responses to acute infection or on control of latent/chronic infection. Theoretically, the potential of the new drugs to predispose to infection depends on their site of action. But this is simplification of the whole problem, because cancer patients are treated for months or years before the installation of the innovative therapy. Thus, an old, traditional risk factors for infectious complications should be added to the overall risk for the given patient. Invasive fungal diseases (IFDs) are associated with the treatment of cancer, patients with solid tumors have lower risk than majority of patients with hematological malignancies. The obvious risk factors for IFDs in patients with solid tumors are neutropenia, steroids, central venous catheter, progressive or refractory malignancy, gastrointestinal surgery with leak of anastomosis among others. Invasive candidiasis followed by invasive aspergillosis are the most common IFDs in this setting, other pathogens are less involved. With the advent of the new therapeutic approaches in oncology patients and physicians are challenged with the new spectrum of adverse events including infections. Generally, monoclonal antibodies, tyrosine kinase inhibitors, check point inhibitors and other targeted and biological agents are not associated with the increasing risk of IFDs in solid tumor patients. In special circumstancies patient undergoing such antineoplastic therapy may have an additional risk for IFDs. An example are the patients with melanoma or lung cancer treated with check point inhibitor developing an immune related adverse event. High dose steroids with or without anti -TNF blocker are recommended in the management of the serious toxicity; these patients are potentially at risk for IFD development due the supportive treatment of AE. Risk of IFD will be discussed in the patients with solid tumors reflecting the advances of antineoplastic treatment. Despite the relatively low risk for the development of IFDs and slowly growing experience some recommendations could be made. Anticipation, prediction and alertness are the pillars of action in this setting of cancer patients.

## Meet the expert sessions

### M04. Novelties in the molecular diagnostics of fungal infections

LacknerM.Institute of Hygiene and Medical Microbiology, Medical University of Innsbruck, Innsbruck, Austria

The aim of this meet the expert session is to provide an overview on the most recent developed diagnostic kits in the field of medical mycology. We aim to present and compare novel diagnostic tools with established assay based on our personal experience and available literature. The presentation will be based on a literature review of the past 18 months and a web search for commercially available products. A mix of currently developed assays of research groups will be presented, as well as CE-IVD labelled commercial tests. The most promising innovative diagnostic assays will be presented and discussed. The audience is invited to share their experience with novel assays to enable a lively discussion and exchange of opinions.

### M12. Environment

SabinoR.^1^SegalE.^2^1Reference Unit for Parasitic and Fungal Infections, Infectious Diseases, National Health Institute Dr. Ricardo Jorge, Lisbon, Portugal2Sackler School of Medicine Tel-Aviv University, Tel-Aviv, IsraelExposure to environmental fungi, whether this occurs indoors or outdoors, in leisure activities, in the workplace or in the home, is an everyday occurrence. Different types of environment may contain variable levels of fungal particles and include viable and non-viable yeasts, conidia, hyphal fragments, as well as fragments derived from the fungal cell wall. Mycotoxins and other volatile organic compounds should also be considered as environmental potential hazards. Recognition of the importance of the environment as a source of human infection has come about, at least in part, as result of the emergence of an unprecedented number of ubiquitous environmental fungi as major causes of disease. Exposure to environmental fungi is associated with high number of hospital admissions, and asthma related ailments. Allergic bronchopulmonary mycosis, rhinosinositis, asthma with fungal sensitization and hypersensivity pneumonitis are among the diseases more frequently associated with fungal exposure. In addition, immunocompromised patients are at higher risk for the development of invasive infections. Changes in environmental factors, including changing land-use patterns, use of antifungals in agriculture, and climate changes have led to epidemiological shifts. Therefore, special attention should be paid in regard to the isolated fungal species. Environmental fungal species such as *Aspergillus* spp., *Mucorales, Candida* spp., dermatophytes and dimorphic fungi will be discussed during this session; emphasizing their importance as etiological agents of fungal infections.

### M13. Diagnostics/Imaging

ThorntonC.Biosciences, University of Exeter and ISCA Diagnostics Ltd., Exeter, United KingdomBench-to-Bedside Diagnosis of Invasive Pulmonary Aspergillosis. Invasive pulmonary aspergillosis (IPA) is a life-threatening lung disease of haematological malignancy and bone marrow transplant patients caused by the air-borne mould *Aspergillus*. Current diagnostic tests for the disease lack sensitivity or specificity, and culture of the fungus from invasive lung biopsy, considered the gold standard for IPA detection, is slow and often not possible in critically ill patients. While Computed Tomography (CT) is a non-invasive diagnostic procedure, it has limited clinical utility for IPA diagnosis, but is nevertheless used as a trigger for commencing antifungal treatment in a number of centres. Innovative approaches to the diagnosis of IPA are needed to enable diagnostic-driven treatment with mould active drugs. In this talk, I will discuss the development of diagnostic technologies for IPA detection based on the *Aspergillus*-specific mouse monoclonal antibody JF5, including the CE-marked *Aspergillus* lateral-flow device (OLM Diagnostics), and CE-marked *Aspergillus*-ELISA (Euroimmun AG). In addition, I will describe the humanisation of mAb JF5 for PET/MR imaging of *Aspergillus* lung infections *in vivo*, and translation of the imaging technology to the clinical setting. I will finish by describing the recent development of a mAb, PD7, specific to *A. fumigatus*, and its use to detect a novel protein biomarker of IPA in urine.

### M14. Resistance—Utilizing molecular methods

WhiteP.Microbiology, Public Health Wales Microbiology, Cardiff, United KingdomUntil recently the identification of antifungal resistance has been based on the availability of a cultured organism to perform susceptibility testing to determine a MIC against a particular antifungal agent. The development of molecular techniques has provided an alternative option, by identifying genetic markers potentially associated with resistance or identifying species that may by inherently resistant, or have an increased likelihood of resistance to particular drugs. Indeed, molecular assays offer the only alternative to culture dependent procedures, that themselves can suffer from limited sensitivity and delayed time to result. However, molecular approaches are not without limitations. PCR based methods targeting specific mutations (for example: single nucleotide polymorphisms and/or tandem repeats), by design have a limited detection range, missing existing, likely less prevalent mutations, but also the presentation of novel genetic mechanisms. PCR methods that identify species that are usually resistant to certain antifungal drugs will be reliant on the knowledge of local epidemiology and the subsequent susceptibility testing of relevant cultures to guide antifungal prescribing. The use of PCR-sequencing, but more so next generation sequencing, will help identify a wider range of polymorphisms in genes encoding the proteins targeted by antifungal therapy and also in promoter regions that may enhance up-regulation, but the detection of novel mutations may lack the necessary phenotypic evidence confirming associated resistance. In addition, PCR-sequencing is associated with a significant processing time and has only had limited evaluation of direct specimen testing, where sensitivity may be lacking when targeting single copy genes. As of yet none of these methods can be confidently associated with a specific MIC range, and the presence of well defined polymorphisms have been associated with typically susceptible MIC values. This presentation will describe the benefits and limitations of using molecular methods for determining antifungal resistance, while providing data on the clinical performance of assays, including those that are commercially manufactured.

## Symposium 1 Water quality (mis-)management—an opportunity for fungal contamination

### S01.1. Fungal Contaminants in Drinking Water Regulation? A Tale of Ecology, Exposure, Purification and Clinical Relevance

BabičM. Novak^1^Gunde-CimermanN.^1^BrandãoJ.^2^1Department of Biology, University of Ljubljana, Biotechnical Faculty, Ljubljana, Slovenia2Department of Environmental Health, National Institute of Health Doutor Ricardo Jorge, Lisbon, Portugal

Safe drinking water is one of the fundamental human rights according to the Resolution 64/292 issued by the United Nations Human Rights Council [1]. Although the parameters for safe drinking water depend on the living conditions and vary between continents and countries, they strive for a common goal: drinking water is suitable for users when it does not contain dangerous chemical substances and microorganisms [2]. To achieve this, raw water from an underground or surface source is subject to various purification techniques before entering the distribution system. The processes usually include aeration, coagulation, flocculation, sedimentation, and ultrafiltration, depending on the location and quality of raw water [3]. Finally, water quality in Europe is controlled based on the parameters listed in Drinking Water Directive (98/83/CE). Water not reaching the required microbiological minimum is additionally subject to chemical treatment, mostly chlorination. Fungi are not listed in the current directive and are therefore not specifically monitored. Yet, their presence in fresh water is well documented and has been studied in at least 19 countries of Europe over the past 30 years [3]. More than 400 different species of fungi have been reported from water sources intended for human consumption, with prevailing presence of ascomycetous fungi of the genera Alternaria, Acremonium, Aspergillus, Aureobasidium, Candida, Cladosporium, Exophiala, Fusarium, Penicillium, Phoma, Sarocladium, Scopulariopsis, Sporothrix, Stachybotrys and Trichoderma, followed by Absidia, Mortierella, Mucor and Rhizopus from subphylum Mucoromycotina. Most commonly encountered yeasts were basidiomycetous genera Cystobasidium, Naganishia (formerly Cryptococcus) and Rhodotorula [3]. While water purification procedures remove 8–90% of fungal propagules, the remaining will ultimately form mixed biofilms inside the tap water distribution systems, later affecting the taste and odour of drinking water. Also, several water-related fungal species were recognised as opportunistic or emerging pathogens, although their transmission and effect on the health of drinking water consumers remains poorly investigated. Besides drinking, consumers use potable water also for daily activities such as cooking, washing and personal hygiene. During these activities they are in constant contact with fungi and their metabolites directly with the skin while washing, the digestive system while drinking, and through the respiratory system by inhaling aqueous aerosols [3,4]. With increasing transitory and serious immune alterations among patients as well as an increase of azole-resistant fungal strains in recent years also a need for monitoring of fungi increased, not only in drinking water but also in relation to materials in contact with drinking water.

References:

[1] UN. Resolution adopted by the General Assembly on 28 July 2010 64/292. The human right to water and sanitation. United Nations: New York, USA, 2010; p. 3.
[2] WHO. Guidelines for Drinking Water Quality, 4th ed.; World Health Organization: Geneva, Switzerland, 2011; p. 564.
[3] Novak Babič, M., et al. Fungal Contaminants in DrinkingWater Regulation? A Tale of Ecology, Exposure, Purification and Clinical Relevance. Int. J. Environ. Res. Public Health 2017, 14, 636.
[4] DEFRA. A Review of Fungi in Drinking Water and the Implications for Human Health, 1st ed.; BIO Intelligence Service: Paris, France, 2011; p. 107.

### S01.2. Hospital environment: water supply and containment of aerosolised fungal particles. How far must we go in times of antimicrobial resistance?

SabinoR.Reference Unit for Parasitic and Fungal Infections, Infectious Diseases, National Health Institute Dr. Ricardo Jorge, Lisbon, Portugal

Invasive fungal infections depend on the interplay between host susceptibility and environmental exposure. Therefore, hospital environment is one of the major concerns in the management of nosocomial fungal infections, especially in wards bearing immunocompromised patients. Particular attention should be paid to the environmental risks associated with water since fungi can be aerosolized at water taps and showers. This may lead to fungal exposure by inhaled and ingested droplets, or even by direct contact with mucosae. Studies report that filamentous fungi and yeasts are commonly found on water-pipe inner surfaces, even in the presence of free chlorine. Air levels of *Fusarium* and *Aspergillus* conidia were found to increase in hospital environments after running showers multiple times. Species of these two genera are described as the most frequently found in this setting due to their conidial dispersion mode, as well as their ability to form biofilms. Despite the intrinsic resistance found in some species of these two genera, fungal exposure to antifungal agents via medical or agricultural use of these compounds, appears to have a major impact on acquisition of resistance to azoles; namely in *Aspergillus fumigatus*. The isolation of this species in hospital water and water reservoirs is therefore an even bigger matter of concern. More recently, several reports on nosocomial outbreaks caused by *Candida auris* have been described. This species is resistant to several classes of antifungals and is associated with high mortality. Contamination of hospital environment or transient colonization of medical devices and equipment may display an important role in the transmission of this species. *C. auris* was already found in water samples and therefore this reservoir should not be excluded as possible source of infection. In conclusion, fungal counts and detection of potential pathogenic species in water were, until a few years ago, the major concern of clinical and scientific community towards the reduction of nosocomial fungal infections originating in water devices. The emergence of infections caused by fungal isolates with intrinsic or acquired antifungal resistance triggered new levels of alert in this field.

### S01.5. Spectrum of indoor fungi isolated from indoor environments in Busia-Kenya

MashediO.^1^AmukoyeE.^2^BiiC.^3^KimaniB.^3^ChepchirchirA.^4^OkothS.^5^1Centre For Respiratory Dise Ases Research, Kenya Medical Research Institute, Nairobi, Kenya2Centre For Respiratory Diseases Research, Kenya Medical Research Institute, Nairobi, Kenya3Centre For Microbiology Research, Kenya Medical Research Institute, Nairobi, Kenya4School of Health Sciences, University of Nairobi, Nairobi, Kenya5School of Biological Sciences, University of Nairobi, Nairobi, Kenya

**Objectives:** The unplanned growth, rapid evolution of industrialization has deteriorated the ambient air quality. Anthropogenic indoor air pollution continues to be seen as an environmental and public health problem. Its seriousness lies in exposure to fungi that can trigger allergic reactions, hypersensitivity pneumonitis, allergic rhinitis, and some types of asthma. Very few studies in Kenya have been conducted to determine the possible types of indoor fungi allergens in indoor environment and to evaluate the relationship between allergen exposure in residential environments and respiratory morbidity. The aim of this study is to determine the prevalence of fungal indoor pollution in residential environments and to establish allergens which are associated with fungal pathogens.

**Methods:** This was a cross-sectional study carried out in 60 households in Busia County. Sedimentary and Volumetric sample collection method was used on water, air, swabs of dust and surfaces from homes. Mycological procedures in identification and analysis were used in fungal characterization. Comprehensive questionnaires were administered to determine the risk factors associated with indoor fungi infestation.

**Results:** 60 study participants were interviewed from Busia urban setting with mean age of 35.2 ± 7.7 (SD) years where most 60.3% of them were aged between 40–49 years; while 19.0% were aged 20–29 years. 68.4% of the respondents were females. As regard to education background, 61.4% of the respondents had primary education as the highest education level whereas 3.5% had tertiary level. 43.1% of the households were occupied by more than 5 people. Filamentous and yeast fungi were both isolated. *Candida albicans* and *Rhodotorula* accounted for the 39% of the isolated yeast species. Among the filamentous fungi *Fusarium* had the highest prevalence of 79.7%, followed by *Cladosporium spp*, *Alternaria spp, Acremonium Apergillus* and *Penicillium* respectively. There was a significant association between *Fusarium* and *Acremonium* moulds and respiratory conditions. *Acremonium* species was significantly associated with itchy eyes allergies, *p* = 0.023.

**Conclusion:** There is a need for the identification and monitoring of fungi in our home environment. Residential environment could potentially be considered an effective target for interventions aimed at reducing inciting fungi exposures to prevent upper respiratory allergies and infection in urban populations.

## Symposium 2 Pneumocystis jirovecii, an airborne transmissible and human derived ascomycete showing strong pulmonary tropism

### S02.1. Pneumocystis jirovecii: an obligate parasite of human lungs with unique camouflage and sex strategies

HauserP.Institute of Microbiology, CHUV Hospital, Lausanne, Switzerland*Pneumocystis jirovecii* is a fungus colonizing the human lungs. Should the immune system weaken, it can turn into an opportunistic pathogen causing severe pneumonia that can be fatal if not treated. A method of in vitro long-term culture for this pathogen is not available yet. Nevertheless, the advent of the high throughput DNA sequencing methods allowed sequencing the *P. jirovecii* genome out of the lung microbiome. The analysis of this genome revealed the loss of several synthesis pathways, demonstrating that this fungus is an obligate parasite. The presence of a fusion of the minus and plus mating-type loci suggested that its mode of sexual reproduction is primary homothallism (self-fertility). The sexuality of *P. jirovecii* proved to be obligatory with human lungs, which is compatible with asci and/or ascospores being the particles responsible for the transmission between humans by the air route. Long reads sequencing permitted characterizing six hypervariable gene families encoding surface antigens within the subtelomeres of all chromosomes. These gene families are responsible for surface antigenic variation allowing presumably escape from the human immune system, as well as for adhesion to host cells. The most abundant family is subject to mutually exclusive expression of a single gene, whereas each gene of the other families possesses its own promoter, suggesting independent expression. Recombinations between members of each family is thought to generate gene mosaicism that contribute to antigenic variation. The mechanisms involved in *P. jirovecii* antigenic variation suggest that the infecting populations are antigenically heterogeneous, *i.e*., made of subpopulations displaying each a unique antigen of the most abundant family, as well as a minority of antigens of the other families.

### S02.2. High-throughput methodologies in molecular epidemiology of Pneumocystis jirovecii

MatosO.Medical Parasitology Unit, Group of Opportunistic Protozoa/hiv and Other Protozoa, Global Health and Tropical Medicine, Instituto de Higiene e Medicina Tropical, Universidade Nova de Lisboa, Lisboa, PortugalThe microscopic fungus *Pneumocystis jirovecii* is a pathogen that can cause fatal pneumonia (PcP) in immunocompromised patients (AIDS, transplant recipients, patients with haematologic and solid malignancies, patients with rheumatoid conditions and connective tissue disorders, patients receiving immuno-modulatory therapies or with pre-existing chronic lung conditions) worldwide. In the absence of an in vitro culture system to isolate and maintain live organisms, previous efforts to understand transmission patterns to develop methods of intervention and management for PcP relied on PCR-based approaches. Several genotyping methods have been used so far to address the genetic heterogeneity of *P. jirovecii*, PCR of multiple *loci* followed by direct DNA sequencing (Sanger), restriction fragment length polymorphism (RFLP), single-strand conformation polymorphism (SSCP), short tandem repeat (STR) or single-base extension (SBE). These methodologies were followed by the introduction of next generation sequencing (NGS), which is considered a revolution in the field of DNA analysis. NGS has been applied in the assembly of *P. jirovecii* whole genome and in amplicon analysis, especially for outbreaks investigation. Here we will explore the advantages and disadvantages of these approaches applied in the expansion of knowledge of the epidemiology of *P. jirovecii*.

### S02.3. Airborne acquisition and transmission of Pneumocystis jirovecii: an update

NevezG.^1^,^2^LaurenceP.^1^,^2^GalS. Le^1^,^2^1University of Brest, Brest, France2Host-Pathogen Interaction Study Group, SFR ICAT 4208, Univ Angers, Univ Brest, Institute of Biology in Health, IRIS, University Hospital Center, Angers, France, Angers Cedex, FranceAirborne transmission of Pneumocystis sp. from host to host has been demonstrated in rodent models and several observations suggest that interindividual transmission occurs in humans. Moreover, it is accepted that the Pneumocystis organisms infecting each mammalian species are host specific. Thus, the hypothesis of an animal reservoir for *Pneumocystis jirovecii* (*P. jirovecii*), the human-specific Pneumocystis species, can be excluded. An exosaprophytic form of the fungus cannot be strictly ruled out. Nonetheless, these data point out that: (i) the specific host may serve as its own reservoir (ii) Pneumocystis infection in humans is an anthroponosis with humans as a reservoir for *P. jirovecii*. This review highlights the main data on host-to-host transmission of Pneumocystis sp. in rodent models and in humans by the airborne route. Specifically, recent data *on P. jirovecii* exhalation by patients developing PCP or with pulmonary colonization in their surrounding environment are described. Taken together, these recent findings render it possible to provide a rationale for prevention of *P. jirovecii* acquisition and transmission within the hospitals.

### S02.5. Does Pneumocystis jiroveci infection aggravate the prognosis of invasive pulmonary aspergillosis? Data from the RESSIF network in France (2012–2016)

Robert-GangneuxF.^1^BouzilleG.^2^DromerF.^3^GangneuxJ.P.^4^T.H.E. French Mycoses Study Group^5^1Univ Rennes, CHU Rennes, Inserm, EHESP, IRSET UMR S_U1085, RENNES, France2Univ Rennes, CHU Rennes, Inserm, LTSI – UMR 1099, RENNES, France3National Reference Center For Invasive Mycoses & Antifungals, Molecular Mycology Unit, Cnrs Umr2000, Institut Pasteur, Paris, France4Laboratoire De Parasitologie-mycologie, CHU de Rennes, Rennes, France5National Reference Center For Invasive Mycoses & Antifungals, Molecular Mycology Unit, Cnrs Umr2000, Institut Pasteur, RENNES, France

**Objectives:***Pneumocystis jiroveci* pneumonia (PjP) is a frequent opportunistic infection in immunocompromised patients with various immune deficiency backgrounds, whereas invasive aspergillosis (IA) mostly occurs in patients with neutropenia or neutrophil impairment. Occasionally both fungi can be detected simultaneously. In that instance, Pj detection is sometimes considered as simple colonization, particularly when microscopic examination is negative and PCR is positive. In this study, we addressed the question of the presence of Pj as an aggravating factor during IA.

**Methods:** We took advantage of the surveillance of invasive fungal infections (IFIs) implemented by the National Reference Center for Invasive Mycoses and Antifungals through the RESSIF network. All cases of IA and PjP recorded during a 5-year period (2012–2016) were analyzed. Multivariate analysis compared cases of IA and cases of IA+PjP co-infection, using Cox regression after multiple imputation of missing data. All cases with a follow-up of 90 days were taken into account, extra-pulmonary aspergillosis was excluded. The following variable were included for analysis: age, sex, clinical background (hematological malignancy, allogeneic stem cell transplantation (allo-HSCT), auto-HSCT, graft versus host (GVH) disease, solid organ transplantation (SOT), cancer), corticotherapy or biotherapy, delay of AI onset, AI classification (proven, probable), and *Aspergillus* species.

**Results:** 730 patients were included, of whom 701 had IA and 29 had IA+PjP. The mean age of patients with IA and IA+PjP was 58.5 and 61.3, respectively (*p* = 0.08). There were no statistical differences in the sex ratio, clinical background, type of SOT, corticotherapy or biotherapy exposure, time from transplantation to diagnosis, time from hospitalization to IA diagnosis, time to death, nor classification of IA, between the two groups. The mortality rate was higher in the group IA+PjP than in IA alone (65% versus 39%, respectively, *p* = 0.009). Species from the Fumigati section accounted for 58.6% and 43.1% of cases in IA+PjP and IA, respectively (ns). In the multivariate analysis, the factos independently associated with death at 3 months were: age > 60 years (HR = 1.74 ; CI95% [1.4–2.2]), solid cancer (HR = 2.2 ; CI95% [1.1–4.6]), co-infection AI+PjP (HR = 1.9 ; CI95% [1.2–3.1]) and section Fumigati IA (HR = 1.65 ; CI95% [1.3–2.1]).

**Conclusion:** Pj infection is an aggravating factor of IA, particularly in patients with solid cancer. No other associated risk factor was uncovered probably due to the limited number of co-infections recorded. We plan to extend the study period to increase the statistical power of the analysis.

## Symposium 3 Mucormycosis

### S03.1. Hospital related mucormycosis

SkiadaA.1st Dpt. of Medicine, Laiko Hospital, Athens University, Athens, GreeceMucormycosis is a rare invasive fungal infection due to fungi of the order Mucorales. The usual underlying diseases are hematological malignancies and diabetes mellitus, but a significant proportion of patients are immunocompetent. The main clinical presentations are rhinocerebral, pulmonary, cutaneous and disseminated. The agents of mucormycosis are ubiquitous in nature, and they are transmitted by inhalation, direct inoculation or ingestion. There have been multiple reports of healthcare associated mucormycosis, either as isolated cases or as outbreaks. In a publication from India, during an eighteen month period, 75 cases of mucormycosis were reported, of which 9% were nosocomial. Healthcare associated mucormycosis has been attributed to various exposures in the hospital environment, including: (a) Use of non-sterile products. This is the most commonly suspected cause of infection. Bandages, adhesives, nitroglycerin patches, contaminated linen, wooden tongue depressors, ostomy bags, probiotics, have all been implicated. There has even been a report of an outbreak due to allopurinol tablets and pre-packaged food. (b) Various procedures and medical devices, such as catheters, insulin pumps and finger sticks, as well as insertion of tubes, tooth extractions and surgery. (c) Environmental factors may also be a source of infection. Molds may be found in the air, dust, water or any surfaces in the hospital. Construction works increase the risk of invasive fungal infections. Outbreaks have been linked to defective ventilation systems and water leakage. The clinical presentation varies, depending on the source of infection. Infections due to bandages, adhesives or contaminated wound dressings are mostly cutaneous. Percutaneous exposure in immunocompromised patients has led to disseminated disease. Inhalation leads to pulmonary and rhino-cerebral infection, while ingestion of tablets or food, as well as the use of tongue depressors, are responsible for gastrointestinal mucormycosis. Dialysis catheters have been linked to peritonitis. It is not easy to investigate a possible mucormycosis outbreak. The Centers of Disease Control of USA have recently published a guide to investigating suspected outbreaks due to mucormycosis. Due to the rarity of the disease, investigation is warranted even when it is unclear if there is a true outbreak. The first step is to verify the diagnosis and define a case. Histopathology and culture are the mainstay of diagnosis. In recent years, newer tools have been added to our diagnostic armamentarium. These include PCR, whole-genome sequencing and matrix-assisted laser desorption/ionization time-of-flight mass spectrometry (MALDI-TOF MS). The next step in the investigation of an outbreak is to describe the epidemiologic features of the cases, scrutinize patient care activities and determine environmental involvement. The management of outbreaks is multi-disciplinary and should include treating physicians, hospital administration, workers in the microbiology laboratory and health departments. Rapid identification of cases and initiation of treatment is important, in order to decrease mortality of existing cases and prevent the emergence of new ones.

### S03.3. The new treatment of mucormycosis

PaganoL.Fondazione Policlinico Universitario A. Gemelli—IRCCS—Universita Cattolica del Sacro Cuore, Rome, Italy

The antifungal drug that showed the best in vivo activity in the past has been deoxycolate Amphotericin B (d-AmB). Although this molecule, although extremely effective, has been characterized by an important series of side effects (i.e. renal insufficiency, hypokalemia) which have made its use disadvantageous. In more recent years, the introduction of new formulations of AmB in lipid formulations (l-AmB, ABCD, ABLC) have mitigated the unpleasant side effects without eliminating them completely but have made their use more manageable. Nowadays lipid formulations, and especially l-AmB, are considered by all the international guidelines as the first-choice drugs for the treatment of mucormycosis. In recent years new azole formulations, first and second-generation azoles (i.e. posaconazole, isavuconazole) have shown considerable efficacy in the treatment of these molds even in highly immunocompromised patients such as those suffering from acute leukemia or undergoing bone marrow transplantation, whereas voriconazole and itraconazole show no effect on mucorales. Posaconazole was registered as a drug to be used as an alternative to liposomal amphotericin and often the availability of the oral solution alone has considerably limited its use. The oral-only route of posaconazole is anyway a great limitation, especially in hematologic patients, often inable to take drug per os or undergoing chemotherapy with subsequent high incidence of vomiting. However the availability of posaconazole in intravenous formulation and in coated tablets has favored a greater absorbability and, consequently, greater efficacy. Isavuconazole in registrative trial has shown a good efficacy in the treatment of this fungal complication and is now recognized as an excellent alternative. We must also take into account the possibility, supported in recent years by multiple studies in real life, to approach the most aggressive and disseminated forms of mucormycosis with the combination of multiple drugs. l-Amb combined with caspofungin would appear to be preferable due to the lack of negative interactions between the drugs used, however there are multiple reports showing that l-Amb plus posaconazole may have a positive role.

### S03.4. Clinical features associated to fungal species

Garcia-HermosoD.^1^SitbonK.^1^GautierC.^1^DromerF.^2^T.H.E. French Mycoses Study Group^2^1National Reference Center for Invasive Mycoses & Antifungals, Molecular Mycology Unit, Cnrs Umr2000, Institut Pasteur, PARIS, France2National Reference Center for Invasive Mycoses & Antifungals, Molecular Mycology Unit, Cnrs Umr2000, Institut Pasteur, Paris, France

**Background** Fungi of the order of *Mucorales* are ubiquitous saprobes that can be responsible for life-threatening infections especially in immunocompromised patients but also in immunocompetent individuals. An increased incidence of mucormycosis has been reported mostly due to the rise of populations at risk and the use of better diagnostic tools. There are more than 20 pathogenic species involved in this type of infections, with some of them associated with specific body sites. The main localizations of mucormycosis are rhino-orbito-cerebral and pulmonary infections, depending on the underlying conditions of the patient. We present here a preliminary analysis aiming to dissect the association between clinical manifestations and *Mucorales* species using the active surveillance system (RESSIF) implemented in France at the NRCMA since 2012. 

**Methods** Three-hundred cases of probable and proven mucormycosis were so far collected through RESSIF. The isolates were characterized using phenotypic analysis by morphological methods, and antifungal susceptibility testing of 8 antifungal drugs done using EUCAST microdilution method. In addition, a multi-locus sequencing based on LSU, ITS, TEF-1 was performed. 

**Results** Among those 300 cases of invasive mucormycosis, lung involvement was the main presentation (52%) followed by rhinocerebral (18%) and cutaneous (15%) involvement. Deep infections occurred most often in patients with hematological malignancies. Six major species of *Mucorales* were identified with *Rhizopus arrhizus* being the most frequent fungus. Our current data expand previous findings showing that distribution of these 6 major species depended on the body site localizations and underlying condition of the patient.

### S03.5. Molecular diagnosis of mucormycosis

MillonL.Parasitology—Mycology—Umr6249 Cnrs Chrono-environnement, University Hospital Besançon, Besançon, France

Molecular techniques have provided a new understanding of the epidemiology of mucormycosis. Several factors may explain the growing incidence of mucormycosis, especially the increase in the number of susceptible people and the change in antifungal practices (which may have altered the relative frequency of mucormycosis and aspergillosis among patients at risk for both infection). In addition, the routine use of molecular techniques has helped to accurately identify Mucorales fungi in tissue samples when cultures are negative. As a result, the number of mucormycosis cases diagnosed in the last 10 years has increased. The use of molecular techniques has also improved the therapeutic management of mucormycosis. Indeed, mucormycosis and aspergillosis have similar underlying conditions, and similar radiological and clinical signs, but their antifungal treatments are different. For each of these infections, early diagnosis and early initiation of directed treatment are essential to improving patient outcome Molecular techniques may help to provide fast, effective treatment by accurately identifying the causative fungi. PCR amplification and sequencing were first applied to better identify isolates grown from cultures of biopsies or bronchalveolar lavage samples collected in patients with Mucorales infection. Molecular techniques have also been used to identify the fungus directly from the tissue samples when cultures were negative. The first technical approach for detection fungi in tissue samples consists of using panfungal primers, targeting ITS regions, followed by sequencing. The second possible approach consists of using Mucorales-specific primers. Diverse PCR techniques were tested and were successful, and several studies confirmed that PCR results were better in fresh/ frozen samples than in formalin-fixed paraffin-embedded samples. Molecular detection of fungi on hyphal positive biopsy samples is now strongly recommended (AII) by the European ESMID and ECMM joint clinical guidelines. Recent studies confirmed that Mucorales quantitative PCR (qPCR) applied on bronchoalveolar lavage fluid could provide additional arguments favoring earlier initiation of specific antifungal therapy, thus improving the outcome of pulmonary mucormycosis patients. However, these tools require invasive sampling (biopsy, bronchoalveolar lavage), which is not feasible in patients in poor condition in Hematology or Intensive Care units. QPCR-based procedures to detect Mucorales DNA in non-invasive samples such as plasma or serum have proved successful in diagnosing mucormycosis early (as early as 8 days before mycological diagnosis and 3 days before imaging in patients with hematological malignancies). The test can be performed in all patients, even those who cannot endure a biopsy or bronchoalveolar lavage. The Mucorales qPCR performed on serum or plasma is becoming essential to improve the management of at-risk patients. The main problem at the moment is a lack of standardization, because of the use of multiple in-house assays. Efforts from the Fungal PCR Initiative (FPCRI)/Mucorales Lab working group of the International Society for Human and animal Mycology (ISHAM) are currently ongoing to improve standardization and provide recommendations, as was previously done for *Aspergillus* PCR assays by the European *Aspergillus* PCR Initiative (EAPCRI) working group.

### S03.6. Management of mucormycosis in low and middle income countries

ChakrabartiA.Department of Medical Microbiology, Postgraduate Institute of Medical Education & Research, Chandigarh, India

The epidemiology of mucormycosis is different in low and middle income countries (LMIC) from developed nations. In LMIC the number of mucormycosis cases is alarmingly high in diabetic patients, the spectrum of causative agents is wide with lack of knowledge on antifungal susceptibility of the new agents. Occasional unusual clinical presentation baffles the clinicians about diagnosis, and delay in seeking healthcare by patients leads to poor outcome of the disease. Additionally, the lack of awareness of the disease among clinicians, the lack of availability of appropriate diagnostic facilities, poor availability and affordability of antifungal agents pose serious challenge in the management of the disease. In a recent descriptive study covering 465 mucormycosis cases from 13 centres in India, it was noted that 24% patients could not complete or start antifungal therapy due to affordability issue and nearly 20% patients were treated with amphotericin B deoxycholate. In 4.2% patients the therapy was shifted from liposomal to deoxycholate preparation of amphotericin B after certain period due to financial constraints, and improper antifungal therapy was prescribed in 7% patients. Due to those challenges, 90-day mortality was 52.0%, though majority (78.2%) of the patients had rhino-cerebral or cutaneous mucormycosis, which were not difficult for early diagnosis. Deferasirox of label use was also noted in certain number of patients.

## Symposium 4 MSG symposium (Trial Design in Clinical Mycology: Innovative Approaches)

### S04.5. The Lung Transplant Community is Interested in a Clinical Trial to Determine the Optimal Strategy to Prevent Invasive Mold Disease

HozR. La^1^JonesC.^2^McgwinG.^3^PattersonT.^4^PappasP.^3^BaddleyJ.^3^MorrisseyO.^5^1Internal Medicine, UT Southwestern Medical Center, Dallas, United States of America2The Ohio State University, Columbus, United States of America3University of Alabama at Birmingham, Birmingham, United States of America4University of Texas Health Science Center at San Antonio, San Antonio, United States of America5Monash University, Melbourne, Australia

**Objectives:** The objectives of this survey were to: (1) describe the strategies used to prevent invasive mold disease in lung transplant recipients, (2) quantify the interest in participating in a clinical trial to determine the optimal prophylactic strategy for invasive mold disease in lung transplant recipients and (3) to describe the design types and factors that influence willingness to participate in the clinical trial.

**Methods:** We invited transplant providers to participate in an anonymous cross-sectional web-based survey via email between December 2018 and February 2019. The 139-item survey was hosted on the University of Alabama at Birmingham Research Electronic Data Capture (REDCap) server. The contact list was generated from a variety of sources. The study was approved the University of Alabama at Birmingham Institutional Review Board.

**Results:** of the 238 practitioners contacted, 50 (21.0%) responded to the survey. Responses were mainly from the United States 85.7% (36/42) and were transplant infectious diseases physicians 65.2% (30/43). The number of transplants performed at the responder’s centers during 2016 was less than 20 in 44.4% (16/36), 21 to 50 in 19.4% (7/36) and more than 50 in 36.1% (13/16). Most responders, 81.0% (30/37), had a mold prevention protocol at their program. Universal prophylaxis was the most common prevention strategy 75.7% (28/37), followed by pre-emptive prophylaxis 10.8% (4/37), and targeted prophylaxis 5.4% (2/37). A quarter of the responders (7/34) used prophylaxis for <3 months, 58.8% (20/34) for 3 to 6 months, 11.8% (4/34) for 6 to 12 months and 8.8% (3/27) for more than 12 months. The majority of the responders, 84.6% (22/26) were interested in participating in a clinical trial to assess the optimal prophylactic strategy for mold infections in lung transplant recipients. Almost a quarter of the participants were willing to participate of the clinical trial with a universal prophylaxis study design of new azole formulation to mold active azole (Figure 1). The study design arms and the anti-fungals selected for the study arms were the factors that had the most influence in study participation (Figure 2). 

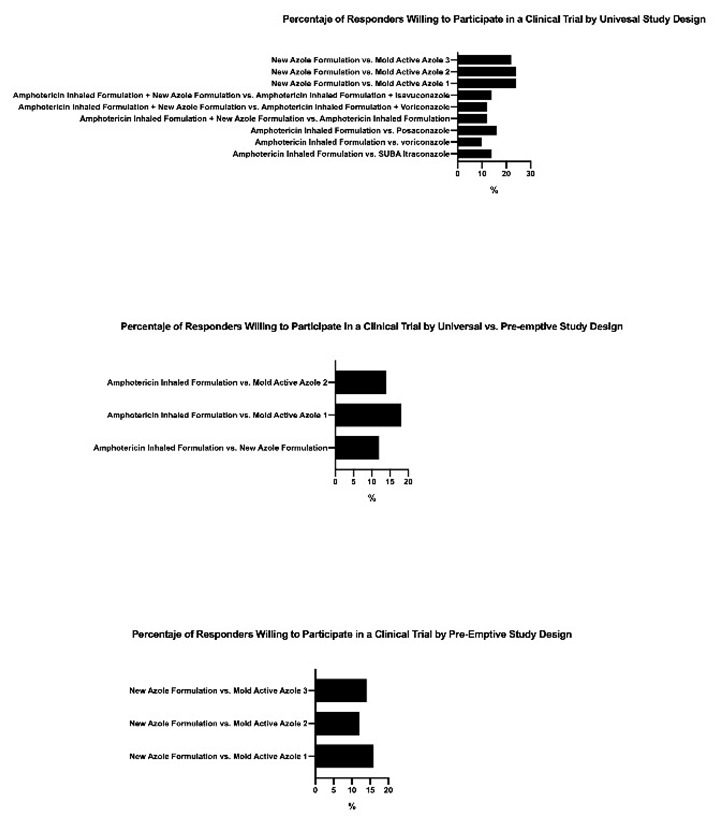

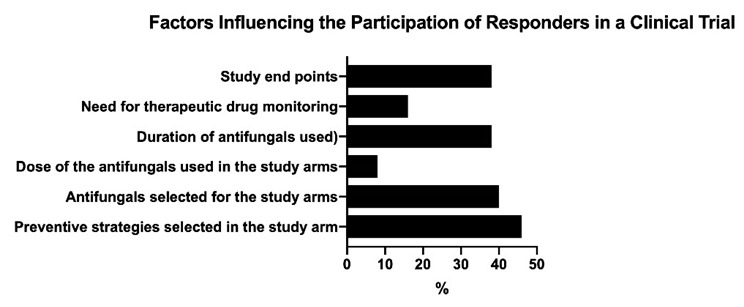





**Conclusion:** Universal prophylaxis was the most common strategy to prevent invasive mold disease in lung transplant recipients. The majority of the responders were interested in a clinical trial to determine the optimal strategy to prevent mold disease in lung transplant recipients. The study design arms and the anti-fungals selected for the study arms were the factors that had the most influence in study participation.

## Symposium 5 Lung transplantation

### S05.1. Pre-transplant assessment

RammaertB.^1^,^2^,^3^1Infectious Diseases, CHU Poitiers, Poitiers, France2U1070, INSERM, Poitiers, France3Faculté De Médecine Et Pharmacie, Université de Poitiers, Poitiers, France

All the three main chronic pulmonary diseases leading to lung transplantation are at risk for fungal respiratory tract colonization: cystic fibrosis (CF), chronic obstructive pulmonary disease (COPD), and interstitial lung diseases (ILD). *Aspergillus* and *Scedosporium* species are the two main molds found in the lower respiratory tract of lung transplant candidates. Colonization in CF patients could be explained by impairment of muco-ciliary clearance, and in COPD and ILD patients by use of inhaled or systemic corticosteroids that alter pulmonary immunity. Mortality due to invasive fungal diseases in post-transplantation can reach 60% depending on the causal species. Therefore, fungal colonization of the lower respiratory tract is on the list of relative contraindications for lung transplantation. Although passage from colonization to infection is not well understood, fungal spores could remain quiescent in the lungs, and secondarily reactivate once patients have received immunosuppressive drugs. Donor-derived fungal infection is one of the best examples of this reactivation. Pre-transplant colonization is not considered predictive for fungal invasive disease in post-transplantation. However, the timing and bronchoalveolar lavage protocols for fungal screening in respiratory tract before lung transplantation are heterogeneous between centers. In addition, culture of lower respiratory tract specimens has to be done on specific media such as Sabouraud glucose agar or yeast extract-peptone-dextrose-agar (YPDA), which could miss out on some fungi. Utilization of culture media supplemented with antibiotics has increased the number of CF patients culture-positive for fungi in their respiratory specimens from 18% to 78%. *Scedosporium* specific media such as Scesel+ increase the detection of *Scedosporium* spp. compared to Sabouraud glucose agar. Active screening for fungal colonization during the pre-transplant period is consequentley crucial not only in patients on a waiting list, but also in donors. There is an urgent need for bronchoalveolar lavage and fungal culture standardization in the different mycological laboratories dealing with lower respiratory tract specimens before focusing on pre-transplant fungal colonization.

### S05.5. From the lung to the heart: fatal dissemination of azole-resistant Aspergillus fumigatus in a lung transplant patient

LavergneR.-A.^1^,^2^LepoivreT.^3^GrootT. De^4^JeddiF.^2^BoutoilleD.^5^Danner-BoucherI.^6^MorioF.^1^,^2^MeisJ.^7^PapeP. Le^1^,^2^1Ea1155 Parasitology and Mycology Department, Nantes University, Nantes, France2Parasitology and Mycology Laboratory, Nantes University Hospital, Nantes, France3Thoracic Transplantation Intensive Care Unit, Nantes University Hospital, Nantes, France4Medical Microbiology and Infectious Diseases, Canisius-Wilhelmina Hospital, Nijmegen, Netherlands5Tropical Infectious Diseases, University Hospital of Nantes, Nantes, France6Pneumology Unit, Nantes University Hospital, Nantes, France7Department of Medical Microbiology and Infectious Diseases, Excellence Center For Medical Mycology (ECMM), Canisius Wilhelmina Hospital, Nijmegen, Netherlands

Azole resistance in *Aspergillus fumigatus* has emerged as a worrisome problem since the last ten years. Patients suffering from cystic fibrosis (CF) are at risk of being colonized with azole-resistant isolates. At our CF reference center hospital, prevalence of azole resistance in *A. fumigatus* reached 6.8% in 2015. Though resistance is increasingly described, its impact in CF patients is yet poorly known. Here, we describe the case of a CF patient having long-term colonization of the respiratory tract with a non-*cyp51A* mediated azole-resistant *Aspergillus fumigatus* isolate, prior to lung transplantation. Then, proven azole-resistant invasive aspergillosis and mucormycosis were diagnosed in the first days after transplantation and treated with liposomal amphotericin B. Two years later, azole-resistant *Aspergillus fumigatus* mitral-aortic endocarditis was diagnosed. Despite surgery and multiple antifungal regimens, the patient died with intracerebral hematoma. **Objectives**: The aim of the study was to investigate the genetic relationship between isolates collected at the time of transplantation and those from the native valve. **Methods**: Five isolates were retrospectively investigated for azole susceptibility to itraconazole (ITR), voriconazole (VOR), posaconazole (POS) and isavuconazole (ISA) (EUCAST method). Identification of *A. fumigatus sensu stricto* was confirmed using partial beta-tubulin sequencing. Molecular mechanisms leading to resistance (nucleotide sequencing of *cyp51A* gene and its promotor) and STR*Af* genotypes were determined. **Results:** Two isolates were collected at time of transplantation (bronchial secretion (*n* = 1) and postoperative wound (*n* = 1)) and three isolates were collected during endocarditis episode (respiratory tract (*n* = 2) and mitral valve (*n* = 1)). All isolates were identified as *Aspergillus fumigatus* and were pan-azole resistant (MIC ITR: > 8 mg/L, VOR: 4 to 16 mg/L, POS: 0.5 to 1 mg/L, ISA: 8 to >16 mg/L). Sequencing of the *cyp51A* gene and its promotor (4 isolates) showed wildtype *cyp51A* and promotor. All isolates shared the same STR*Af* genotype (M2: 26-20-8 M3: 35-9-7 M4: 9-10-20) confirming dissemination of this isolate from the lung tissue to the heart. **Conclusion:** This case report highlights the need for regular screening for azole resistance in CF patients especially for those awaiting lung transplantation. Physicians should keep in mind that azole-resistant colonizing isolates could be a possible source of life threatening invasive infection after lung transplantation. Yet, antifungal treatment of *Aspergillus* endocarditis is based on voriconazole or lipid formulation of amphotericin B but no data are available in case of azole-resistant isolate.

## Symposium 6 Prophylaxis during hematology malignancies

### S06.1. AML—in the era of FLT3 inhibitors

LewisR.^1^,^2^1Infectious Diseases Clinic, Sant’Orsola-Malpighi Hospital, Department of Medical and Surgical Sciences, University of Bologna, Bologna, Italy2Department of Medical and Surgical Sciences, University of Bologna, Bologna, Italy

FLT3 mutations are among the most common genetic mutations in acute myeloid leukemia (AML) associated with increased risk of disease relapse and poorer survival. A number of small molecule tyrosine kinase inhibitors (TKI) of FLT3 signalling have shown promise for reducing relapse of FLT3+ AML. One TKI, midostaurin, received fast-track approval in 2017 for the treatment of FLT3 positive AML on the basis of data demonstrating, for the first time in over 45 years, improved survival compared to standard induction 7+3 (cytarabine/anthracycline) chemotherapy. The use of FLT3 inhibitors, however, is problematic in patients who require antifungal prophylaxis or treatment with triazole antifungals. In general FLT3 inhibitors are extensively metabolized by CYP3A4 to active metabolites with high potency and potential off-target effects. When coadministered with triazoles, FLT3 exposures may increase between 1.3 to 10 fold and alter production of active metabolites essential for FLT3 inhibition or increase off-target effects. Indeed, higher rates of pulmonary toxicity including fatal events, were observed among patients receiving midostarin with azole antifungals. Currently, the midostaurin SmPC does not recommend the co-administration of strong CYP3A4 inhibitors such as voriconazole or posaconazole; although this recommendation is not practical for many patients especially with active fungal disease. Unfortunately, second-generation FLT3 inhibitors currently in clinical studies (e.g., quizartinib) also appear to have similar risks as midostaurin as well as greater risk for dose-limiting myelotoxicity and QTC prolongation. It is clear that striking the balance between optimal antifungal prophylaxis vs. optimal FLT3 inhibition will become a major challenge in the management of AML in the coming decade. This presentation will discuss the pros and cons of possible strategies for managing drug-drug interactions with FLT3 inhibitors and antifungals in the absence of clearly-defined guidance.

### S06.4. Baseline CT upon diagnosis of acute leukemia

SchwartzS.Charité Berlin, Berlin, Germany

Computed tomography (CT) is a routine diagnostic procedure in immunocompromised patients (i.e. patients with long-lasting neutropenia) with suspected fungal pneumonia. Particular radiological appearances of lung infiltrates (e.g. halo-sign) have been associated with lung infections caused by molds and findings have been incorporated into the well acknowledged definitions of invasive fungal infections developed by the EORTC/MSG (1). A conventional chest X-ray is usually performed in immunocompromised patients with fever, but the value of a conventional chest X-ray has been questioned due to its poor diagnostic yield and most recommendations advocate CT (2). A baseline chest CT in asymptomatic patients scheduled for therapies resulting in a profound immunosuppression (e.g. induction chemotherapy for acute leukemia) appears meaningful, but published data about this are limited. In a prospective study, 198 adult patients scheduled for hematopoietic stem cell transplantation or intensive chemotherapy were evaluated with a baseline CT (3). Overall, 72 (36%) patients showed lung abnormalities. of these, 19 exhibited disease-defining lesions according to the EORTC/MSG definitions (nodules, halo sign, cavitation) and 53 patients showed other abnormalities (most frequently consolidations or ground-glass opacifications). Nine patients in each group were subsequently categorized as having proven or probable invasive mold infections. The risk of developing an invasive pulmonary mold infection was significantly higher among patients with disease-defining lesions according to the EORTC/MSG definitions or, less pronounced, with other abnormalities compared to patients with a normal baseline CT. In another study, 145 baseline CT scans from patients with acute leukemia scheduled for their first induction therapy were assessed retrospectively (4). Remarkably, the majority of baseline CT scans disclosed abnormalities (127; 88%) with nodules in 71 (49%) patients. Cavitations or lesions with a halo- or an air-crescent-sign were not present in this patient series. However, an independent radiological review and results from a comprehensive diagnostic evaluation were not available from this study. The high frequency of lung lesions at baseline, including disease-defining lesions associated with invasive mold infections, in patients scheduled for intensive chemotherapy or hematopoietic stem cell transplantation is striking. A baseline CT scan in these patients appears reasonable, but the overall benefit of this diagnostic approach in asymptomatic patients should be explored further in future studies. It would be desirable to evaluate in more detail other underlying, infectious/non-fungal and non-infectious, conditions (e.g. opacities associated with hyperleukocytosis) associated with lung lesions (5). **Literature** 1. de Pauw B, et al. Clin Infect Dis 2008;46(12): 1813–21. 2. Maschmeyer G. Curr Opin Oncol 2001;13: 229-35. 3. Ceesay MM, et al. Br J Haematol 2018;182: 723–27. 4. Vallipuram J, et al. Leuk Lymphoma 2017;58: 834–41. 5. Stefanski M, et al. Medicine 2016;95: e5285.

### S06.5. Investigating the impact of posaconazole prophylaxis on systematic fungal screening using galactomannan antigen, Aspergillus qPCR and Mucorales qPCR

BellangerA.-P.^1^TatoyanN.^1^BerceanuA.^2^Gbaguidi-HaoreH.^3^MonnotT.^4^BichardD.^4^MillonL.^1^1Mycology, University Hospital Jean Minjoz, Besancon, France2Hematology, University Hospital Jean Minjoz, BESANCON, France3Infectious Risk Control, University Hospital Jean Minjoz, BESANCON, France4Pharmacy, University Hospital Jean Minjoz, BESANCON, France

**Objectives:** At the University Hospital of Besancon, a systematic panel of fungal biomarkers is applied systematically to screen for invasive fungal infection (IFI) highly immunocompromised patients of the Hematology Department. This panel of biomarkers does include the galactomannan antigen (GM), Aspergillus qPCR and Mucorales qPCR. Most of the stem cell transplant (SCT) recipients receive now posaconazole prophylaxis. The pertinence of systematic fungal screening has been discussed in the literature for patients under active antifungal prophylaxis, especially the realization of the GM. In this context, our main objective was to investigate retrospectively if the posaconazole prophylaxis influenced the numeric value of the GM. Our secondary aims were to assess the contribution of Aspergillus qPCR and Mucorales qPCR as well as the prevalence of IFI under active antifungal prophylaxis.

**Methods:** The results of systematic fungal screening (GM, Aspergillus and Mucorales qPCR) were collected for all the SCT recipients, treated with posaconazole as prophylaxis treatment for more than 8 days, from January 2015 to December 2016. Similarly, results of systematic fungal screening were collected for patients without posaconazole as prophylaxis treatment (induction chemotherapy-January 2010 to December 2015). The statistical analysis of data was performed using the Stata 2 software.

**Results:** For the group of patients receiving posaconazole prophylaxis: 60 patients were included, with a mean period of prophylaxis of 107 days and a total 916 fungal screenings. The mean value of the GM was 0.17 (±0.32), the frequency of positive GM was 4.37%, the frequency of positive Aspergillus qPCR was 0.68% and the frequency of positive Mucorales qPCR was 3.66%. The frequency of IFI in this group was estimated at 5%. For the control group: 38 patients were included with a total of 392 fungal screenings. The mean value of the GM was 0.22 (±0.64), the frequency of positive GM was 5.61%, the frequency of positive Aspergillus qPCR was 12.35% and the frequency of positive Mucorales qPCR was 5.33%. The frequency of IFI in this group was estimated at 13%. The statistical analysis showed that posaconazole prophylaxis did not affect significantly neither the numeric value nor the frequency of positivity of the GM. A significant decrease of the frequency of positive Aspergillus qPCR was observed (0.68 *vs* 12.35, *p* = 0.015). A tendency of decreased frequency of IFI was also observed (5% *vs* 13%, *p* = 0.076).

**Conclusion:** Our study did not demonstrate any negative effect of the posaconazole prophylaxis on the GM. This study highlights the high efficacy of the posaconazole prophylaxis, especially when using the tablet form, which was supported by a significant decreased frequency of positive Aspergillus qPCR and relatively lower frequency of IFI. The current screening strategy combining GM and fungal qPCRs is of high interest, even if the posaconazole prophylaxis is efficient, in a context of emerging infection with azole resistant strains of *Aspergillus fumigatus* and risk of *A. fumigatus*-Mucorales mixed infections.

## Symposium 7 Paediatric Mycology (EPMyN)

### S07.5. Isavuconazole use in pediatric hematoncologic patients: the Italian Association of Pediatric Hematology Oncology (AIEOP) experience

DecembrinoN.^1^PerruccioK.^2^ColombiniA.^3^CaloreE.^4^MuggeoP.^5^SonciniE.^6^ComelliA.^7^MolinaroM.^8^GoffredoB.M.^9^GregoriS. De^8^GiardiniI.^8^CairoliS.^9^ScudellerL.^10^ZeccaM.^1^CesaroS.^11^1Pediatric Hematology/oncology, Fondazione IRCCS Policlinico San Matteo, Pavia, Pavia, Italy2Division of Pediatric Hematology, University Hospital of Perugia, Perugia, Italy3Pediatric Hematology/oncology, Fondazione Monza e Brianza per il Bambino e la Mamma, Monza, Monza, Italy4Clinic of Pediatric Hemato- Oncology, Department of Women’s and Children’s Health, University Hospital of Padova, Padova, Italy, Padova, Japan5Department of Pediatric Oncology and Hematology, University Hospital of Policlinico, Bari, Italy, Bari, Italy6Pediatric Hematology/oncology, Spedali Civili, Brescia, Italy, Brescia, Italy7Department of Infectious and Tropical Diseases, Spedali Civili, Brescia, Italy, Brescia, Italy8Clinical and Experimental Pharmacokinetics Lab, Fondazione IRCCS Policlinico San Matteo, Pavia, Pavia, Italy9Metabolic Pathology Lab, Ospedale Pediatrico Bambino Gesù, Roma, Roma, Italy10Clinical Epidemiology and Biometric Unit, Scientific Direction, Fondazione IRCCS Policlinico San Matteo, Pavia, Pavia, Italy11Pediatric Hematology/oncology, Azienda Ospedaliera Universitaria Integrata, Verona, Italy, Verona, Italy

**Objectives:** Isavuconazole (ISA) is a new triazole approved for invasive aspergillosis and mucormycoses treatment in adults only. Advantages are activity against both moulds and yeasts spp, excellent bioavailability after oral administration without relevant food or gastric pH effect, a water-soluble prodrug without nephrotoxic excipients, poor drug-drug interactions. We analyzed ISA use, safety and efficacy in immunocompromised children.

**Methods:** Italian Association Paediatric Hematology Oncology (AIEOP) multicentric retrospective analysis of pediatric hematoncologic patients who received ISA asinvasive fungal disease (IFD) treatment or prophylaxis. Due to the lack of recommended dosing in children and of PK/PD target value, therapeutic monitoring (TDM) was applied by a validated liquid chromatography-tandem mass spectrometry (HLPC-MS/MS) assay technique. ISA trough plasma concentrations (C0) and 3 hours after drug intake (C3h) were measured.

**Results:** Twenty-nine patients were included, (M/F 20/9); median age: 14.5 years (range 3–18), median bodyweight 47 kg (range 15–80). Ten patients only received chemotherapy, 19 patients received allogeneic hematopoietic stem cell transplantation (HSCT). Donors were haploidentical in 9 patients, matched-sibling in 4, allogenic-unrelated in 4 cases. ISA was used as prophylaxis in 5 patients, as IFD treatment in 24 cases: 20 for previous therapeutic failure (7 l-AMB, 4 Voriconazole, 5 combination therapy, 1 Posaconazole, 3 Caspofungin), 4 as first line therapy. According to EORTC criteria, IFD was proven in 5 patients (1 *Aspergillus spp*, 1 *Aspergillus flavus*, 1 *Aspergillus fumigatus*, 1 *Penicillium spp*. and 1 *Mucor spp*), probable in 9 and possible in 10 patients. Lungs were the main localization (18 cases), liver was involved in 2 cases, 3 patients had sinus involvement (2 with central nervous system localizations), 1 had a disseminated infection. Patients under 30 kg bodyweight received half ISA dose, the others received adult schedule (200 mg TID loading dose on days 1 and 2, 200 mg/die maintenance); only 13 patients received loading dose. Ten patients only received oral therapy, in the others IV route was switched to oral during treatment. ISA was administered for a median of 75.5 days (range 6–523 day), in combination with other antifungals in 7 patients (Caspofungin in 3 cases, l-AMB in 4). Overall response rate was 70.8%. IFD complete remission was achieved in 12 cases, partial remission in 5. Treatment failure was experienced by 5 patients, in 3 cases fungal lesions remained stable. No breakthrough infections were registered in prophylaxis group. TDM was applied to 17 patients including 1with severe veno-occlusive disease and 1 with renal failure secondary to thrombotic-microngiopathy. Overall median ISA Ctrough_ss_ concentration observed was 4.91 mg/L (2.15–8.54); Conc/Dose (kg) ratio was 1.13 (0.47–3.42). In 6 cases a 12h-PK profile was carried out and median AUC0–12h of 153,16 mgxh/L (range 86.31–169.45) was obtained. CTAE grade II-III toxicity was observed in 6 patients, with increased transaminase and/or creatinine levels which resolved after temporary ISA withdrawal. We did not observe drug-drug interactions in the patients receiving immunosuppressant as graft versus host disase prophylaxis (5 cyclosporine, 2 sirolimus, 1 tacrolimus).

**Conclusion:** Isavuconazole may be useful and safe in the pediatric population with hematoncologic diseases, even in the HSCT setting. Prospective studies are warranted.

## Symposium 8 Immunologic markers for diagnosis and treatment stratification in invasive mold infection

### S08.3. Immunologic Markers for Treatment Stratification in Invasive Mold Infection

KöhlerP.Department I of Internal Medicine, University Hospital of Cologne, Cologne, Germany

The two major entities, invasive aspergillosis and mucormycosis account for the majority of fungal mould infections in haematology and oncology. With increased hospital admission rates and admission to intensive care unit and high morbidity and mortality the need for prompt identification and outcome prediction is needed. Current in vitro diagnostic approaches comprise direct observation of fungi in biopsy or bronchoalveolar lavage samples, blood cultures and detection of diagnostic antigens such as galactomannan. New immunologic markers for diagnosis and treatment stratification have been recently published. Fungus-reactive CD4 T cells, interleukin profiling and inflammation parameters will be discussed with regard to treatment stratification depending of response of invasive mould infections. Possible developments could lead to algorithms incorporating measurement of these biomarkers at admission and throughout initial treatment for the purpose of identifying patients who warrant more aggressive treatment.

### S08.4. Fungal Translocation: from persistent inflammation to non-AIDS events

HoeniglM.^1^,^2^1Division of Infectious Diseases, Department of Medicine, UCSD, San Diego, United States of America2Section of Infectious Diseases and Tropical Medicine and Division of Pulmonology, Medical University of Graz, Graz, Austria1,3-β-d-glucan is a fungal cell wall polysaccharide produced by ascomycete fungi including Candida albicans and other yeast that colonize the GI tract. Recently we and others have shown that in the absence of an invasive fungal infection blood 1,3-β-d-glucan levels may serve as a marker of microbial translocation in patient populations with hypoperfusion of the gut and those with persistent inflammation, including patients with advanced liver cirrhosis, sepsis or HIV infection, where blood 1,3 β-d-glucan have been shown to be elevated compared to HIV negative controls and to correlate with other biomarkers of microbial translocation, with markers of immune activation and with lower Lactobacillales proportion (i.e. less protective) in the gut microbiome. In animal models 1,3-β-d-glucan has been shown to decrease Dectin-1 (innate pattern recognition receptor essential for the control of fungal infections), and also NKp30 (NK receptor mediates direct fungal recognition and killing) on monocytes and NK cells respectively, and induced pro-inflammatory cytokines secretion, indicating a potential role in pathogenesis of clinical events. Going one step further, studies have shown clear correlations between blood 1,3-β-d-glucan levels and non-AIDS events, including cardiopulmonary morbidity, and neurocognitive impairment. Only few studies to date have evaluated a potential benefit of antifungal treatment for tackling fungal translocation and reducing blood 1,3-β-d-glucan levels. In vitro model of mixed rat neuronal cultures that screened more than 2000 compounds, half of which were US Food and Drug Administration approved drugs for putative neuroprotective effects against oxidative stress-mediated neuronal injury not only paroxetine, a selective serotonin reuptake inhibitor, but also fluconazole, protected hippocampal neurons. In another study, the combination of paroxetine and fluconazole protected macaques from simian immunodeficiency virus–associated neurodegeneration. However, a phase 1/2, randomized, double-blind, placebo-controlled study for the treatment of HIV-associated neurocognitive impairment did not show an improvement of neurocognitive outcomes among the 9 participants randomized to fluconazole treatment alone, indicating that more research and larger studies are needed on how to prevent/treat fungal translocation.

### S08.5. Immunological characteristics of Broncho alveolar lavage in neutropenic patients with invasive aspergillosis

AguilarC.^1^MoshkelgoshaS.^2^GohirW.^1^YeeK.^3^SchimmerA.^3^MindenM.^3^SchuhA.^3^RyanC.^4^JuvetS.^2^HusainS.^1^1Transplant Infectious Diseases, University Health Network, Toronto, Canada2Lung Transplant Program, University Health Network, Toronto, Canada3Hematology Department, University Health Network, Toronto, Canada4Respirology, University Health Network, Toronto, Canada

**Objectives:** Invasive aspergillosis (IA) is a major complication in patients with acute leukemia receiving high dose chemotherapy. Diagnosis of IA classically relies on radiological criteria associated with direct and/or indirect microbiological tests, which have some limitations. some limitations. Antifungal immunity involves phagocytic cells including neutrophils and macrophages, as well a dendritic cells presenting antigen to T-CD4^+^ T cells. Antigen presentation leads to differentiation of CD4^+^ T cells into Th1 and Th17 cells, under the influence of cytokinic environment. Those cells secrete cytokines and amplify effector functions of phagocytic cells in order to kill the fungus. The triggering of those mechanisms relies on the recognition of fungal patterns by receptors of innate immunity, both soluble (such as PTX-3) and membrane bound (such as Dectin-1 and Dectin-2). In this study we aimed to assess the immunological parameters of broncho alveolar lavage (BAL) fluid of neutropenic patients with or without IA.

**Methods:** We included acute myeloid leukemia patients who were neutropenic after receiving high dose chemotherapy and who underwent a bronchoscopy for diagnosis of pneumonia. Ten milliliters of BAL fluid were collected for the research study. Supernatant and cell pellet were separated and frozen until use. The level of 35 cytokines (EGF, Eotaxin, FGF basic, G-CSF, GM-CSF, HGF, IFN-α, IFN-γ, IL1-ra, IL-1α, IL1-β, IL-2, IL-2r, IL-3, IL-4, IL-5, IL-6, IL-7, IL-7, IL-8, IL-9, IL-10, IL-12 (p40/p70), IL-13, IL-15, IL-17A, IL-17F, IL-22, IP-10, MCP-1/CCL2- MIG, MIP-α, RANTES, TNF-α, and VEGF) was measured in BAL supernatant using Luminex technology. PTX-3 level in BAL supernatant was measured by Enzyme-linked immunosorbent assy. The cell pellet was analyzed by flow cytometry for expression of CD45, CD206, CD163, Dectin-1 and Dectin-2.

**Results:** Forty three patients were included in the study. Seven patients were diagnosed with probable IA and 20 with possible IA. One patient had mucormycosis diagnosed on lung biopsy and 1 patient had fusariosis. While PTX-3 level was not significantly different in patients with probable IA compared to patients without IA, IL-6, IL-8 land GM-CSF levels were higher in patients with probable IA compared to patients without invasive fungal infection. Flow cytometry could be performed on 31 BAL cell pellets. The proportion of alveolar macrophages (CD163^+^ CD206^+^) among CD45 positive cells was similar in all groups. Dectin-1 and Dectin-2 expression on alveolar macrophages (CD163^+^ CD206^+^ cells) was similar in patients with and without invasive fungal infection.

**Conclusion:** Neutropenic patients with probable IA exhibit higher IL-8, IL-6 and GM-CSF levels in BAL supernatant compared to neutropenic patients without any fungal infection. The cellular expression of membranar receptors Dectin-1 and Dectin-2, as well as the level of soluble PTX-3 were not significantly modified in patients with IA. Further studies are needed to assess the role of immunological parameters, especially cytokines profile, in the panel of tests used for the diagnosis of IA in immunocompromised patients.

## Symposium 9 Chronic Pulmonary Aspergillosis

### S09.2. Diagnosis of CPA. Where do we stand?

BaracA.Clinic for Infectious and Tropical Diseases, Clinical Center of Serbia, Belgrade, SerbiaChronic pulmonary aspergillosis (CPA) is an uncommon, slowly destructive pulmonary disease characterized by progressive cavitation, fibrosis, and pleural thickening. CPA is usually seen in immunocompetent individuals with underlying respiratory disorders. Estimates suggest that up to 3 million people are affected worldwide causing high rates of morbidity and mortality. Diagnosis of CPA is often challenging and delayed and should be based upon a combination of characteristics. Patients are present with indolent, non-specific symptoms such as fatigue, weight loss, sweats, cough, dyspnea, or hemoptysis. The disease often remains undiagnosed for years, resulting in progression. A high index of suspicion is required in patients with predisposing factors such as previous tuberculosis (TB) with residual cavities, severe chronic obstructive pulmonary disease, previous treatment for lung cancer, or nontuberculous mycobacteria. The most important diagnostic finding is changes on chest imaging, usually CT scan. The most common presentation is one or more cavities, characteristically with an irregular or thick wall, but occasionally thin-walled, with or without intracavitary material or well-formed aspergilloma. Cavities tend to affect the upper lobes and often cause para-cavitary or adjacent pleural fibrosis. In early stages, nodules may be present, which may develop into cavitary disease over time. Differential diagnosis includes mycobacterial infection, bacterial abscess, cancer, or vasculitis. Expertise of the reporting radiologist is essential in highlighting the possibility of CPA. In addition to compatible radiological findings, microbiological and/or serological evidence of Aspergillus infection is required, in addition to ruling out alternative diagnoses. Direct microscopy for hyphae, fungal culture of respiratory secretions and histology are all recommended for diagnosis of CPA. Sensitivity of sputum or bronchoalveolar lavage (BAL) culture remains moderate at best, even when specific fungal media are used. Culturing a higher volume of sputum may lead to increased sensitivity. Sensitivity is significantly higher with the high-volume culture, which in addition detected azole-resistant isolates that could be missed with conventional culture. When microscopy and cultures are negative, molecular markers like Aspergillus polymerase chain reaction (PCR), as well as beta D-glucan, galactomannan (GM) testing in BAL samples can be used to increase sensitivity. In addition, using these tests in combination may result in better PPV. The usefulness of testing GM in sputum is not clear in a cohort of patients with CPA and ABPA. Therefore, sputum GM cannot be recommended for the diagnosis of CPA at present. Serological methods can contribute to the diagnosis of CPA. Aspergillus precipitins were used previously but have been supplanted by more sensitive serologic assays. Several assays detecting Aspergillus-specific immunoglobulin G (IgG) are commercially available. In summary, clinical and radiological findings, along with microbiological and serological tests are used in combination to diagnose CPA. There is no individual reference-standard test as all tests are characterized by suboptimal sensitivity and specificity. A high index of suspicion is needed, along with the need to rule out alternative diagnoses like TB or lung cancer.

### S09.5. Raised amphotericin B MIC in Aspergillus fumigatus isolates from patients with Chronic Pulmonary Aspergillosis

LynchF.^1^,^2^MooreC.^2^,^3^Rautemaa-RichardsonR.^1^,^2^,^4^1Infectious Diseases, Manchester University NHS Foundation Trust, Manchester, United Kingdom2National Aspergillosis Centre, Manchester, United Kingdom3Mycology Reference Centre Manchester, Manchester University NHS Foundation Trust, Manchester, United Kingdom4Division of Infection, Immunity and Respiratory Medicine, University of Manchester, Manchester, United Kingdom

**Objectives:** Chronic pulmonary aspergillosis (CPA) is a fungal infection of the lung caused by *Aspergillus* species, which usually affects patients with an underlying respiratory condition. The mainstay of treatment is prolonged antifungal therapy to improve symptoms and prevent disease progression. *Aspergillus fumigatus* with elevated minimum inhibitory concentrations (MIC) for amphotericin B (AMB) have been isolated. The aim of this study is to investigate the emergence and clinical relevance of elevated AMB MICs in *A. fumigatus*.

**Methods:** Patients with CPA reported with AMB resistant (MIC > 4 mg/L) *A. fumigatus* were identified from the Mycology Reference Centre Manchester databases. All *A. fumigatus* isolates reported from these patients were included in the analyses. The date of sampling and resistance patterns of the isolates were analysed against the antifungal history of each patient. EUCAST antifungal breakpoints were used to determine sensitivity. Clinic letters and dispensing records were used to determine medical history, antifungal therapy and clinical outcomes.

**Results:** Nine patients reported with one or more AMB resistant *A. fumigatus* isolates and a total of 148 isolates from these patients were identified. of the isolates, 34 (22%) were resistant to AMB; 13 (38%) had an MIC of 4 mg/L, five (15%) had an MIC of 8 mg/L and 16 (47%) had an MIC of >8 mg/L. In addition, 18 (12%) isolates were reported as intermediate to AMB (MIC = 2 mg/L). Five of the nine patients had prior exposure to AMB (IV AmBisome). Patients received 3-5 mg/kg once daily and different treatment regimens were used; one patient had one 3-week course, two patients had two 3-week courses and two patients received long term therapy three times weekly (for up to 3 years). Four patients had no exposure to AMB. of the 5 patients exposed to AMB, 4 developed resistance during therapy, and 1 patient first showed resistance 5 years after their 3-week course. All patients had prior azole exposure, and the duration of azole exposure prior to AMB resistance was from 8 months to 5 years. All AMB resistant isolates showed some level of azole resistance and 27 (82%) were pan-azole resistant. Example patient timeline.

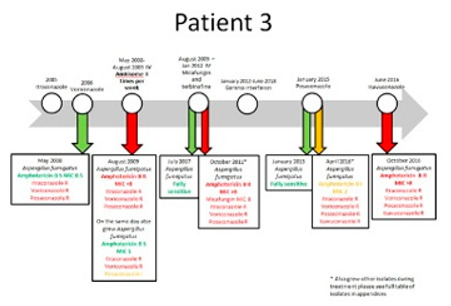



**Conclusion:** All patients reported with *A. fumigatus* with elevated MIC for AMB had previously been treated with azole antifungals and developed resistance against one or more azoles. Many AMB resistant isolates were pan-azole resistant suggesting a link between AMB and azole resistance. Antifungal drug resistance can have significant implications on patient care due to limited treatment options. A better understanding of the development, genetic mechanism, and clinical relevance of AMB resistance is needed. Therefore, all isolates identified in this study will next be sequenced and typed.

## Symposium 10 Symposium 10 Sensing the host

### S10.2. Hybrid histidine kinases: major sensing proteins in pathogenic fungi

PaponN.^1^KabbaraS.^1^HérivauxA.^1^BidonB.^1^KilaniJ.^1^GovicY. Le^1^GasteboisA.^1^BernonvilleT. Dugé De^2^ClastreM.^2^CourdavaultV.^2^GalS. Le^1^NevezG.^1^BoucharaJ.-P.^1^1Host-Pathogen Interaction Study Group, SFR ICAT 4208, Univ Angers, Univ Brest, University Hospital Center, Angers, France2Ea2106 “biomolécules Et Biotechnologies Végétales”, Université de Tours, Tours, France

Protein phosphorylation is one of the most extensively used modifications in signal transduction pathways in both prokaryotic and eukaryotic cells. Prominent families of enzymes that perform these protein phosphorylations encompass serine/threonine kinases, tyrosine kinases, and histidine kinases (HKs). While HKs dominate prokaryotic signaling pathways, they are less prevalent in eukaryotes, and most importantly, absent in animals. Historically, a small number of eukaryotic HKs have been studied in plants, yeasts, filamentous fungi, and slime molds. Recent studies have expanded characterization of HKs in other eukaryotic lineages and archaea, allowing a broader assessment of the types of signaling systems mediated by HKs, their phylogenetic distribution and evolution. HKs are central to regulatory systems that impact agriculture, the environment, and both beneficial and pathogenic interactions of microbes with humans and other animals. Their great diversity, versatility, and broad distribution make them attractive targets for therapeutics, biotechnological interventions, and also as building blocks for engineered biosensor systems. From a structural perspective, fungal HKs are commonly composed of three main regions. The first region referred to as the “sensing domain” corresponds to a highly variable N-terminal sequence that determines the stimulus perceived. The central “transmitter region” is made of two conserved domains: a dimerization histidine phosphotransfer (DHp) domain and a catalytic ATP-binding (CA) domain. The DHp domain includes an H-box, usually containing the phosphorylatable histidine residue, and an X-box. The CA subdomain includes four distinct sequence motifs: the N-, G1-, F-, and G2-boxes. In contrast to prokaryotic HKs, most eukaryotic HKs harbor an additional C-terminal receiver domain (REC) that includes a phosphorylatable aspartate residue. Thus, eukaryotic HKs are generally called “hybrid HKs” (HHKs). From a mechanistic point of view, HHKs constitute in fungal cells the initial sensing proteins of a four-step phosphorelay signaling pathway involving phosphorylation events of two downstream effectors, *i.e.* histidine phosphotransfer shuttle proteins (Hpts) and response regulators (RRs) (Figure 1). The perception of a stimulus induces the trans-autophosphorylation of a conserved His residue in the DHp domain by the CA catalytic part of the HHK. The phosphate of this histidine is then transferred to the conserved Asp residue in the REC domain prior to transfer to the Hpt domain, and finally to the conserved aspartate residue of the protein belonging to the RR family. The phosphorylation state of this RR orchestrates subsequent molecular events underlying the response to the input signal.

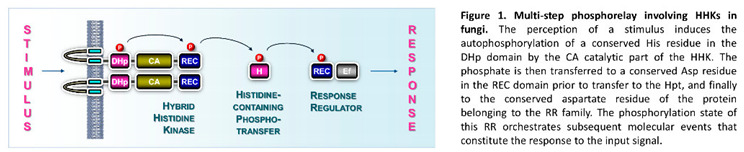



Here, we review the distinct genetic approaches carried out over the last fifteen years and that allowed the elucidation of the function of several HKs in prominent fungal pathogens. However, because their roles in regulating fundamental cellular processes are still largely obscure in fungi, we will discuss the major challenges that remain to improve our knowledge about the function of HKs in fungi and to design specific inhibitors for future therapeutic development.

### S10.5. The analysis on the genes related to the biofilm formation of Candida glabrata

ChenX.^1^WidiyantoT.W.^1^ChibanaH.^2^KajiwaraS.^1^1School of Life Science and Technology, Tokyo Institute of Technology, Yokohama, Japan2Medical Mycology Research Center, Chiba University, Chiba, Japan

Objectives: Candida glabrata is capable of developing biofilm on the surface of host cells and medical materials. The biofilm formation is considered as one of pathogenic factors on infection to the host, which shows increase in the resistance to antifungals and host immune defense compared to the planktonic cells. Until now, the mechanism of biofilm formation of C. glabrata has not been studied well compared with C. albicans. Therefore, the aim of our study was to isolate and characterize some genes related with the biofilm formation of C. glabrata. Methods: The in vitro model of biofilm formation was constructed by using multi-well plates and the metabolic activity were detected by XTT assay. The susceptibility of biofilm formation was also detected towards different antifungals. Gene expression was analyzed by qPCR. Vacuolar morphology was observed by staining with FM4-64 fluorescent dye. The growth fitness was detected in buffered media with different pH and intracellular pH was measured with flow cytometry. Results: Upon the result of a genetic comprehensive screening by using C. glabrata mutant library, two gene mutants decreased metabolic activity of biofilms drastically and the transcriptional expressions of these two genes in biofilm formation showed more than two times higher than those of planktonic cell growth. One gene showed high homology with Saccharomyces cerevisiae SYN8 gene, then was named as CgSYN8. S. cerevisiae Syn8p was identified as a SNARE protein, acting in membrane fusion of vesicles. C. glabrata syn8Δ mutant was defective in both the metabolic activity and the biomass of the biofilm. Deletion of SYN8 also led to decrease in the adhesion ability during the biofilm formation, which may link to the repression of two adhesin genes, EPA10 and EPA22. Additionally, C. glabrata syn8Δ mutant revealed the abnormal vacuolar morphology. Therefore, it was suggested Syn8p is not only required for the normal vacuolar function but also influence the biofilm formation of C. glabrata. Another gene related to the biofilm formation of C. glabrata was named as CgQDR2. QDR (quinidine drug resistance) family genes in S. cerevisiae belong to MFS (Major Facilitator Superfamily) transporters, which mediate the ability of membrane transport to actively efflux the drug out of the cell. C. glabrata qdr2Δ mutant exhibited significant reduction in the biofilm formation and fluconazole drug susceptibility during biofilm formation. In addition, our results suggested QDR2 deletion caused an impaired ability of C. glabrata to maintain pH homeostasis, then might lead to the reduction of cell growth at neutral-basic pH conditions. These results may explain how the QDR2 gene affect the biofilm susceptibility and drug efflux towards antifungal drugs in C. glabrata. Conclusion: CgSYN8 and CgQDR2 were suggested to be involved in biofilm formation of C. glabrata. These findings provide more understanding on the biofilm formation of this fungus and more information for the development of clinical treatment in future.

## Symposium 12 Environment & fungal outbreaks

### S12.1. ‘Once is a misfortune, twice is carelessness…’: Lessons to be learnt from pandemic fungal infections of amphibians

FisherM.Department of Infectious Disease Epidemiology, Imperial College London, London, United KingdomDiscovering that chytrid fungi cause the disease chytridiomycosis in amphibians represented a paradigm shift in our understanding of how emerging infectious diseases contribute to global patterns of biodiversity loss. In this lecture I describe how the use of multidisciplinary biological approaches has been essential to pinpoint the origins of pathogenic chytrids, to time their emergence and pathways of spread and to identify the core mechanisms that underpin their pathogenicity. I will discuss the potential for using bioinformatics toolkits and experimental methods to allow a fuller understanding of these chytrids biology in order to leverage emerging technologies aimed at their control. The impacts of infectious diseases in wild populations are not restricted to wild animals and plants; they also threaten public health through the attrition of ecosystem services as well acting as reservoirs and amplifiers of disease inocula. I will argue that the lessons learnt from tracking amphibian chytrid fungi are also of relevance to understanding and tackling emerging patterns of fungal disease in humans. Paraphrasing Oscar Wilde, ‘to allow one fungal pandemic is misfortune, to allow two is carelessness, to allow three…’.

### S12.2. The Cryptococcus gattii species complex

HagenF.^1^,^2^,^3^1Medical Mycology, Westerdijk Fungal Biodiversity Institute, Utrecht, Netherlands2Medical Microbiology, University Medical Center Utrecht, Utrecht, Netherlands3Department of Dermatology, Jining No. 1 People’s Hospital, Jining, ChinaThe *Cryptococcus gattii* species complex placed itself in the spotlights of the medical mycology community with the unprecedented and ongoing outbreak that emerged in the temperate climate of Vancouver Island (British Columbia, Canada) during the late 1990s. A few years thereafter the outbreak was found to have spread to the Canadian mainland and the Pacific Northwest of the U.S.A. This pathogenic yeast, at that time named *Cryptococcus neoformans* variety *gattii*, was mostly known as being a tropical or subtropical pathogen that caused disease among apparently immunocompetent subjects. It was observed that the outbreak in Canada and the U.S.A. was due to an until then rare genotype named AFLP6/VGII, mostly known from Australia and the South American continents. Subsequent molecular typing showed that the outbreak was caused by a major (AFLP6A/VGIIa) and a minor (AFLP6B/VGIIb) genotype that had different virulence properties. In 2002 *C. neoformans* variety *gattii* was raised to species level as *Cryptococcus gattii*. Using several molecular techniques five lineages were recognized, nowadays known as molecular types (genotypes) VGI (AFLP4), VGII (AFLP6), VGIII (AFLP5) and VGIV (AFLP7), the fifth lineage is extremely rare and known as genotype AFLP10/VGIIIc. Although *C. gattii* was just proposed as being a separate species, the debate was already ongoing whether or not to further differentiate it into more species related to the molecular defined lineages. A further refinement for a taxonomic revision was supported by the availability of more molecular, phenotypic, antifungal sensitivity, geographic distribution and predilection for certain hosts. In 2015 a proposal was made to raise the five lineages to species level: *C. gattii sensu stricto* (AFLP4/VGI), *C. bacillisporus* (AFLP5/VGIII), *C. deuterogattii* (AFLP6/VGII), *C. tetragattii* (AFLP7/VGIV) and *C. decagattii* (AFLP10/VGIIIc). Due to the outbreak with *C. deuterogattii* in North America most of the focus was on this species, but what do we know about the other members of the *C. gattii* species complex in terms of environmental distribution and clinical relevance?

### S12.4. Whole genome sequencing: a valuable tool to study outbreaks due to uncommon yeast species

Desnos-OllivierM.National Reference Center For Invasive Mycoses & Antifungals, Molecular Mycology Unit, Cnrs Umr2000, Institut Pasteur, Paris, FranceRapid genotyping tools such as Multi Locus Sequence Typing (MLST) and Variable Number Tandem Repeat (VNTR) are frequently used for common yeast species involved in invasive human infection (Candida albicans, Candida glabrata, Candida tropicalis, Candida parapsilosis, Pichia kudriavzevii, Cryptococcus neoformans/gattii complex). These methods are very useful to determine potential link between clinical and/or environmental isolates but implementation/validation requires a large number of unrelated isolates. Grouped-cases of infection or colonization due to uncommon or emerging pathogenic yeast species are usually considered to be due to a single strain. Since there is no genotyping method for these uncommon species and considering that origin of the infection can be endogenous and/or exogenous, this hypothesis should be verified. Whole genome sequencing (WGS) has become relatively simple, fast and inexpensive but analysis remains time-consuming and requires bioinformatic expertise. Since 2012 and the first French outbreak due to Saprochaete clavata, we started using WGS to study relationship between isolates involved in grouped cases and belonging to uncommon yeast species. Different approaches involving different parts of the genome can be considered. For S. clavata, alignment of whole genome allowed to identify different clades, one of which was responsible for the national epidemic with specific Single Nucleotide Polymorphism positions (SNP). Then, some SNPs were selected to design a real time PCR with allele-specific oligonucleotide (ASO) which allowed us to rapidly detect isolate belonging to the epidemic clade. Another approach was the use of WGS data of Yarrowia lipoytica to identify intergenic regions highly polymorphic and usable as molecular tool. Finally, the alignment of SNPs positions localized all over the genome was used to study genomic diversity of Trichosporon asahii and Magnusiomyces capitatus population. Although the presence of a rare species in several patients in the same hospital over a short period of time suggest a common source of infection, this is not always the case especially for species that are supposed to be part of the normal biota of skin and/or gastrointestinal tract. Our data confirm the importance of data and isolate centralization in a Reference Laboratory for molecular epidemiology. The routine WGS as a typing method for uncommon yeasts requires prior validation with varied sampling together with bioinformatics experts and mycologists to avoid misinterpretation.

### S12.5. Outbreak of fluconazole-resistant Candida parapsilosis in a hospital ward: arguments for clonal transmission and environmental persistence

FekkarA.^1^,^2^BougleA.^3^NormandA.C.^1^PalousM.^1^PiarrouxR.^1^ImbertS.^1^1Laboratoire De Parasitologie-mycologie, APHP La Pitié Salpétrière, PARIS, France2Sorbonne Université, Inserm, CNRS, Centre d’Immunologie et des Maladies Infectieuses, Cimi-Paris, F-75013, PARIS, France3Réanimation Chirurgie Cardiaque, La Pitie Salpetriere Hospital, Paris, France

**Objectives:** The emergence of multidrug-resistant fungal agent is a threat for human health. In recent years, breakthroughs of infections due to fluconazole-resistant *Candida parapsilosis* isolates have been increasingly reported in different countries. We have recently faced with invasive infections due to resistant isolates and have decided to further investigate this phenomenon.

**Methods:** We conducted a monocentric study at La Pitié-Salpêtrière Hospital, a tertiary care center in Paris, France. All *Candida parapsilosis* isolates collected between January 2012 and April 2019 for which sensitivity to antifungal agents had been determined were analyzed. Antifungal susceptibility testing was performed using Etest (Biomerieux) method and confirmed according the EUCAST broth microdilution reference method. Minimal Inhibitory Concentrations were determined for azoles drugs (i.e. fluconazole, itraconazole, voriconazole, posaconazole and isavuconazole). For fluconazole resistant isolates and a random selection of susceptible isolates, the *erg11* gene was sequenced and isolates were genotyped by a microsatellite method based on 6 markers.

**Results:** 193 isolates (193 patients) sampled between 2012 and 2019 were analyzed. We observed an increase of fluconazole resistance rate among *Candida parapsilosis* isolates (MIC > = 8 µg/mL) between the periods 2012-2016 (3/97, 3.1%) and January 2017-April 2019 (10/96, 10.4%) (p<0.05 by Fisher’s Exact test): three fluconazole-resistant isolates were detected in 2012-2013, none in 2014-2016 and 10 between January 2017 and April 2019. All the resistant isolates presented the previously described A395T mutation in the *erg11* gene that confers the amino acid change Y132F. This mutation was absent in azole-sensitive isolates. Genotyping showed 4 different microsatellite profiles for resistant isolates (R1, R2, R3 and R4) while susceptible isolates were not related each other’s and showed a greater diversity (Figure 1).

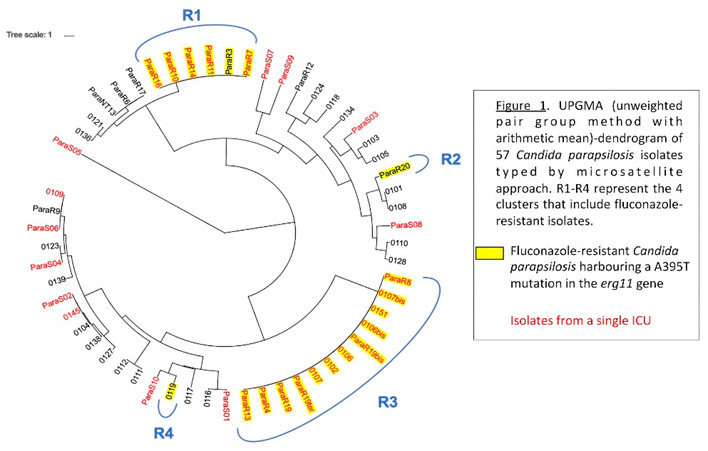



Two resistant clusters (R1 and R3) were related to grouped cases in a single intensive care unit (ICU). Isolates belonging to the R3 profile (*n* = 7) were detected since November 2017, were responsible of 7 deep infections and presented the highest MICs to fluconazole (> 64 mg/L) and a cross resistance with voriconazole (MIC > 0.5 mg/L). Epidemiological and chronological analyses furnish clues for a patient-to-patient transmission but also suggest an ability of resistant isolates to persist in the ward environment. Indeed, two isolates of the same genotype R1 were detected 4 years apart in the same ICU.

**Conclusion:** Our work shows an increase of the incidence of fluconazole-resistant *Candida parapsilosis* isolates in intensive care units in relation to the clonal spread of few strains. Our results provide evidence of i. patient-to-patient transmission and ii. persistence of resistant clone over time in the same unit. Our study urges for a better investigation of fungal resistance and improved eradication measures to limit the spread of this resistant emerging pathogen.

## Symposium 13 Fungal respiratory infections in cystic fibrosis (the ECMM/ISHAM working Group Fri-CF)

### S13.1. Rasamsonia species and other emerging fungi in CF

GalS. Le^1^,^2^NevezG.^1^,^2^FavennecL.^3^,^4^BoucharaJ.-P.^5^,^6^1Laboratory of Parasitology-Mycology, University Hospital Center, Brest, France, Brest, France2Host-pathogen Interaction Study Group Ea3142, University of Brest - University of Angers, BREST, France3Laboratoire De Parasitologie-mycologie, CHU de Rouen, Rouen, France4Protozooses Transmises Par L’alimentation, University of Rouen - University of Reims, Rouen, France5Host-Pathogen Interaction Study Group, SFR ICAT 4208, Univ Angers, Univ Brest, Institute of Biology in Health, IRIS, University Hospital Center, Angers, France, Angers Cedex, France6Laboratory of Parasitology-Mycology, University Hospital Center, Angers, France, Angers, FranceCystic fibrosis (CF) is the most common genetic inherited disease in the European Caucasian population. The disease is caused by mutations in the gene CFTR (Cystic Fibrosis Transmembrane conductance Regulator) which encodes a membrane transporter involved in electrolytic exchanges across the apical membrane of epithelial cells. Several organs are therefore affected by these mutations, but the clinical picture is dominated by respiratory infections. Indeed, because of the resulting thickening of the bronchial mucus, the respiratory tract is often colonized by microorganisms, leading to respiratory infections which are the major cause of morbidity and mortality in patients with CF. Bacteria, mainly Staphylococcus aureus and Pseudomonas aeruginosa, are the major cause of these infections. However, several yeasts and filamentous fungi may also colonize the airways of patients with CF, sometimes leading to pulmonary infections and to severe disseminated infections after lung transplantation. Moreover, even in the absence of clinical signs, this fungal colonization could contribute to the inflammatory reaction and thus to the progressive deterioration of the lung function. If the epidemiology and pathophysiology of fungal infections still remain largely unknown, the frequency of these infections increases together with life expectancy of CF patients. Aspergillus fumigatus ranks first among the filamentous fungi colonizing the airways of patients with CF, followed by Scedosporium species, Exophiala dermatitidis, Lomentospora prolificans (formerly Scedosporium prolificans), and Aspergillus terreus. The prevalence of these fungi varies from one study to another because of: i) differences in patient recruitment (age, CFTR genotype); ii) lack of standardization of the procedures used for mycological examination of respiratory secretions; iii) geographical and climatic variations. In addition, other fungal species have been increasingly reported during the past decade, such as the yeasts Candida blankii, Trichomonascus ciferrii and Blastobotrys adeninivorans / Blastobotrys raffinosifermentans. These yeasts share the capability to use diamines, such as putrescine, which may explain that they multiply in the bronchial mucus during pulmonary exacerbations since the concentration of putrescine increases in this context. Likewise, some filamentous fungi including Arthrographis kalrae and species of the Rasamsonia argillacea complex (formerly Geosmithia argillacea, but first reported in CF as Penicillium emersonii) seem to be emerging in CF. Although it is usually well tolerated, the chronic colonization of the airways by these fungi should not be disregarded since it constitutes a risk factor for disseminated infections in the case of lung transplantation. The emergence of these fungi could be explained at least in part by improvements in diagnostic methods, or by selection pressure caused by repeated antifungal cures. Our knowledge of the ecology of these filamentous fungi still remains in infancy; nevertheless, regarding species of the Rasamsonia argillacea complex, some observations suggest that they could be xylophagous fungi that have spread in Europe since the end of the last century as a consequence of global warming and of the many storms that have crossed Europe since the end of the nineties.

### S13.2. Immunodiagnosis of Scedosporium/Lomentospora infection - lessons from immunoproteomic studies

Ramirez-GarciaA.^1^Martin-SoutoL.^1^Aparicio-FernandezL.^1^BuldainI.^1^AntoranA.^1^BoucharaJ.-P.^2^Martin-GomezM.T.^3^RementeriaA.^1^HernandoF.L.^1^1Immunology, Microbiology and Parasitology, University of the Basque Country (UPV/EHU), Leioa, Spain2Laboratory of Parasitology-Mycology, University Hospital Center, Angers, France, Angers, France3Microbiology, Vall d’Hebron Universitary Hospital, Barcelona, SpainThe detection and diagnosis of *Scedosporium/Lomentospora* still relays on traditional methods and, in spite of being the second most common etiological agent among filamentous fungal infections, very few advances have been achieved in this field during the last decades. This fact is especially worrying in Cystic Fibrosis (CF) patients, whose characteristics favor the microbial presence. In these patients, the fungal detection does not mean that an infection is occurring, but it may increase the risk of suffering from it or other health problems. Therefore, it would be interesting to dispose an easy, rapid and non-invasive detection method for monitoring these patients and study the evolution of the fungus. With this purpose, some groups are focusing their efforts on finding new approaches to design a serologic system. In fact, techniques such as counterimmunoelectrophoresis or ELISA using recombinant proteins of some well-described virulence factors are being used by some laboratories, which design their own assays. Our research group addressed this problem using different immunoproteomics-based approaches. First, four different *Scedosporium boydii* protein extracts were studied by ELISA to select the most useful one for IgG detection in CF patients. The extracts used were a whole-cell extract, fungal cell-surface extract, conidia cell surface extract and secreted extract, all of them being able to discriminate acceptably the *Scedosporium*/*Lomentospora*-infected patients from *Aspergillus-*infected and non-infected patients. However, the best results were obtained with the whole-cell extract, which showed specificity, sensitivity, predicted positive and negative values comparable with commercial kits for other fungal diagnosis purposes. In spite of the excellent results obtained, to increase the specificity and avoid the problems derived from the use of a complex extract, the immunoreactivity of a collection of sera from CF patients infected with *Scedosporium/Lomentopora* against *S. boydii* was also studied in comparing with sera from CF patients infected with *Aspergillus* or without fungus. The analysis, made by 2-DE and WB, allowed the identification of antigens specifically detected by CF patients with *Scedosporium/Lomentopora,* two of them being identified because of their promising results. In addition, to shed light on the problematic of whether the fungal presence in CF patients is a colonization or an infection, we used sera from mice infected intravenously with different species of *Scedosporium*/*Lomentospora* to detect infection-related antigens of *S. boydii.* Interestingly, all the sera showed a very similar immunorecognition patterns, but different from the recognized by sera from *Aspergillus*-infected mice. Therefore, we purified six antigens and tested their interest for diagnosis analyzing their immunoreactivity by WB against sera from CF patients, two of them showing interesting results. Altogether, these results obtained by immunoproteomics-based techniques open up door in the looking for a serologic test for the detection of *Scedosporium*/*Lomentospora* in CF patients, as well as the monitoring of the fungal presence. Specifically, although the results have to be further studied and standardized, the ELISA test using whole-cell extract appears to be a very useful, and the antigens identified seems to be interesting candidates to be include in the diagnostic procedure.

### S13.4. Morphology, growth and biofilm formation of the black yeast-like fungus Exophiala dermatitidis is influenced by Pseudomonas aeruginosa under in vitro cystic fibrosis conditions.

KirchhoffL.^1^RathP.-M.^1^SteinmannJ.^1^,^2^1Institute of Medical Microbiology, University Hospital Essen, Essen, Germany2Institute of Clinical Hygiene, Medical Microbiology and Clinical Infectiology, Klinikum Nuernberg, Nuremberg, Germany

**Objectives:** *Exophiala dermatitidis*, belonging to the melanized fungi, is frequently colonizing the respiratory tract of cystic fibrosis (CF) patients in rates up to 19 %. It was recently reported that *E. dermatitidis* is capable to form biofilms in a strain-dependent manner. However, little is known about *E. dermatitidis* behavior in co-culture with other CF-relevant pathogens, e.g. one of the most prevalent bacterial species, *Pseudomonas aeruginosa*. Growth, biofilm formation capabilities and morphology of *E. dermatitidis* in an artificial sputum medium (ASM), mimicking the CF sputum conditions, were assessed in mono- and in co-culture with *P. aeruginosa*.

**Methods:** *P. aeruginosa* (PA07 & PA14, including PA14 ΔlasR and ΔrhlR) and *E. dermatitidis* (clinical isolate) were analyzed in growth experiments over a period of 48 hours at 36°. Growth was determined by colony forming unit (CFU) counts. Biofilm, formed on polystyrene surfaces under standard protocols, was determined after 24 and 48 hours of incubation at 36°C without agitation by stain with crystal violet, by CFU counts after biofilm detachment using 0.1% dithiothreitol, for species-specific cell counts and by confocal laser scan microscopy determining the thickness of extracellular matrix (ECM). Morphology of the dimorphic *E. dermatitidis* was monitored in light microscopy. Lengths of fungal hyphae were estimated and compared.

**Results:** *P. aeruginosa* showed growth inhibiting effects on *E. dermatitidis*. Cell count of the fungus was decreasing in co-culture with *P. aeruginosa*. In contrast, *E. dermatitidis* biofilm formation was induced after 24 h and reduced after 48 h in co-culture with *P. aeruginosa*. A lack of *P. aeruginosa* quorum sensing (QS) systems lasR and rhlR resulted in induced fungal biofilm formation. At the same time, presence of lasR and rhlR lacking mutants caused an increased amount of *E. dermatitidis* hyphal structures whereas co-cultivation with the *P. aeruginosa* wildtype resulted in decreased hyphae amounts, compared to mono-culture.

**Conclusion:** Interactions between *P. aeruginosa* and *E. dermatitidis* result in altered growth, biofilm formation capabilities and morphology of the fungus in an *in vitro* CF model. A role of *P. aeruginosa* QS systems in these interactions is suggested.

### S13.6. Study of antigenic markers for serological detection of Scedosporium spp. in Cystic Fibrosis patients

Martin-SoutoL.^1^AreitioM.^1^Aparicio-FernandezL.^1^BuldainI.^1^AntoranA.^1^BoucharaJ.-P.^2^Martin-GomezM.T.^3^RementeriaA.^4^HernandoF.L.^4^Ramirez-GarciaA.^4^1Immunology, Microbiology and Parasitology, University of the Basque Country (UPV/EHU), Leioa, Spain2CHU d’Angers, Angers, France3Microbiology, Hospital Universitari Vall d’Hebron, Barcelona, Spain4Immunology, Microbiology and Parasitology, University of the Basque Country (UPV/EHU), Leioa, Spain

**Objectives:** Cystic fibrosis (CF) is the major genetic disorder among the Caucasian population. CF patients produce a thick and sticky bronchial mucus in their respiratory tract, which facilitates the growth of airborne pathogens. In addition to bacteria, several fungi colonize the respiratory tracts of these patients. Species of *Scedosporium* and *Lomentospora* genera are emergent fungal pathogens ranking the second, only behind *Aspergillus* spp., among filamentous fungi causing a chronic colonization of the airways of CF patients. This may lead to chronic inflammation or even to life-threatening invasive disease in cases of severe immunosuppression. Detection of *Scedosporium/Lomentospora* relies upon low sensitivity and specificity non-standardized procedures. To contribute to the finding of new diagnostic methods, this work aims to characterize some molecular targets and to study their usefulness for serological detection of infections caused by *Scedosporium* spp. in CF patients.

**Methods:** A total of 191 CF patients’ sera were used in this study. First, we classified these sera into 3 groups: patients with positive sputum cultures for *Scedosporium* (S+), for *Aspergillus* (A+), and patients with negative fungal cultures (Ctrl). A serological study of the sera was carried out to detect specific IgG response against *Scedosporium boydii* total protein extract by ELISA. In the search of diagnostic markers that allow the discrimination between colonization and infection, we performed an *in vivo* infection in a murine model to obtain sera that allow the detection of antigens associated with a disseminated infection by two dimensional western blot. These antigens were electroeluted from protein gels and blotted individually against CF patients’ sera to observe the immunological response.

**Results:** The ELISA assay provided good specificity, and positive and negative predictive values to discriminate among S+, A+ and Ctrl patients. However, the sensitivity was influenced by differences in the humoral response of the S+ group. This suggests different levels of fungal presence, from colonization to infection, among S+ patients. To solve this problem, we looked for *S. boydii* antigens associated with a disseminated infection in a murine model selecting the six proteins with the highest immunoreactivity. Two of them were specifically recognized by most of S+ sera, especially by those that were more reactive in the ELISA assay.

**Conclusion:** Two proteins of *S. boydii* resulted to be potential diagnostic markers for the detection of the infection caused by *Scedosporium* spp. in CF patients. The use of them in combination with an ELISA using *S. boydii* total protein extract might help in the detection of *Scedosporium* spp. in CF patients and the monitoring of the fungal presence.

## Symposium 14 Teaching Medical Mycology: what, how, to whom and when

### S14.2. Mycology teaching in Medical School—Is student demography important to what should be taught

SegalE.Sackler School of Medicine, Clinical Microbiology and Immunology, Tel Aviv University, Tel Aviv, IsraelMycology which is the discipline devoted to the study of fungi, is one of the four parts comprising Microbiology, the basis for understanding infectious diseases. Medical Mycology is the part of Mycology which focuses on those fungi known to cause or potentially can cause infections in human hosts. As such, study of Medical Mycology is an integral part of the education of physicians and is included in the curriculum of Medical Schools. One of the questions associated with the teaching of Medical Mycology is at which level should Medical Mycology be taught: at the basic sciences level, with other parts of Microbiology, or at a later stage in the frame of Infectious Diseases, as part of Internal Medicine. The answer to this dilemma may vary among different Medical Schools. Another question associated with teaching Medical Mycology in Medical Schools is as to the content of the subject: should it reflect the specific geographic–climatic conditions of the given Medical School or should a more general, broader approach be used. An additional dilemma regarding teaching of Medical Mycology in Medical Schools is whether the specific student demography should affect what is taught and to what extent. The latter is the subject of the following presentation. In the presentation different teaching possibilities will be demonstrated and discussed, as to pro- or contra- of the impact of student demography on what is taught. Examples of different approaches to the dilemma will be shown and discussed. Participation of the audience in the discussion will be encouraged.

### S14.3. Specific courses outside European Continent – The Indian Example

AshbeeR.University of Leeds, Leeds, United Kingdom

HR Ashbee, RD Sahni & JS Michael Teaching medical mycology in the Indian setting presents some unique challenges and opportunities. The diversity of mycoses seen is extensive and presentations can be unusual with disease sometimes occurring in patients not traditionally ‘at risk’. Whilst dermatophytosis and superficial candidosis is often neither diagnosed nor treated, fungal keratitis and sinusitis, subcutaneous and systemic mycoses represent a significant burden on the healthcare system. In combination with this, diagnostic facilities vary from virtually nil to fully equipped laboratories with well-trained staff. The Christian Medical College Hospital in Tamil Nadu, south India has had a mycology laboratory since the 1950’s, has highly skilled staff and a massive number of clinical samples – hence it is ideally suited to deliver comprehensive mycology training. The first course in 2007 was aimed at technical staff working in laboratories and junior faculty, primarily focusing on basic medical mycology and identification of fungal pathogens. The course was attended by 30 participants from all over India, some traveling for 30 hours by train to take part in the course. The need for training was apparent and further courses were planned The current course has been re-designed and is run in two parts. The first 3-day part is aimed at registrars/residents in microbiology and provides an integrated approach to teaching with clinicians presenting cases and the laboratory aspects taught in practical, hands-on sessions after the cases. The full range of diseases are covered, including the superficial mycoses, subcutaneous mycoses, candidosis, cryptococcosis, mucormycosis, aspergillosis and endemic mycoses. Hands-on practical sessions also cover setting up of antifungal susceptibility tests and their interpretation. The second part of the course held over 2 ½ days is for consultant microbiologists and this includes a full day of practical molecular mycology, including DNA extraction, setting up PCR and trouble-shooting molecular assays, use of commercially available assays, as well as molecular detection of antifungal resistance. Antifungal susceptibility testing is taught in another full day practical session with participants taken through the whole CLSI method, from making up the medium, preparing antifungal dilutions, making up plates and preparing the inoculum, to reading and interpreting results. Other methods such as E-test and disc diffusion are also taught. The practicals take place in small groups so every participant can get hands-on experience of each technique. Discussion sessions make up the remainder of the time with ample opportunity for participants to ask questions and clarify any problems. Participants are also encouraged to maintain contact after the workshop and help is provided with testing or fungal identification by CMC staff if required. The courses represent a significant investment of time and resources by the staff of CMC and overseas faculty but feedback has been extremely good and demonstrates that provision of high quality, comprehensive mycology training is both very much needed but also very well received.

### S14.4. Teaching Clinical Laboratory Mycology: How and who is the audience?

Arikan-AkdagliS.Medical Microbiology, Mycology Laboratory, Hacettepe University Medical School, ANKARA, Turkey

Expertise in Clinical Laboratory Mycology requires hands-on experience and in-depth basic knowledge. Challenges regarding teaching Clinical Laboratory Mycology are numerous. First, the number of invasive fungal infections is increasing with more infections due to rare and less-recognized species and the education for management of this growing problem is yet inadequate. Second, Mycology education was rather less emphasized until recent years. Teaching for Clinical Laboratory Mycology practices is usually limited during training of a Clinical Microbiologist and the numbers of well-equipped Clinical Mycology Laboratories and experts are also relatively limited worldwide. Third, the characteristics of Y (and Z) generation(s) are unique. The members of generation Y are primarily among the current audience and they are largely influenced by technology. Therefore, broader training and diverse teaching methods are required to meet all these challenges. Clinicial Laboratory Mycology training is a part of speciality/subspeciality, PhD or Master of Science training programs in Medical Microbiology/Mycology. Also, Infectious Diseases/Infectious Diseases and Clinical Microbiology Joint Training, and Pathology Training Programs include training in Mycology at required levels. For Medical Faculty students, on the other hand, basic Clinical Laboratory Mycology teaching is incorporated in the program at some institutions in order to integrate the previously taught theoretical knowledge with that on mycological diagnosis in the last year(s) of education. Laboratory technicians are also among the audience for teaching Clinical Laboratory Mycology at required and specifically technical level. Combining conventional teaching methods with digital technologies is needed for contemporary high quality Clinical Laboratory Mycology education. The common approach for teaching requires expert(s), a well-equipped Mycology Laboratory, and reliable basic information sources, including physical books or e-books. In lack of these facilities or for advancement for higher levels, in-person courses and workshops are very beneficial. However, they are costly and require qualified instructors and resources. E-learning courses, webinars, and educational websites are also very helpful in expanding the trained audience. Teaching Clinical Laboratory Mycology is a difficult task and requires dedicated Mycologists.

### S14.5. Capacity building for invasive fungal infections in Africa: concrete examples through mycology courses and the DREAMM implementation project

LeclèreA. Sturny^1^Garcia-HermosoD.^2^Boyer-ChammardT.^1^KolteI.^3^DromerF.^4^AlanioA.^5^LortholaryO.^6^LoyseA.^3^1Molecular Mycology, IP Paris, Paris, France2National Reference Center For Invasive Mycoses & Antifungals, Molecular Mycology Unit, Cnrs Umr2000, Institut Pasteur, PARIS, France3Institute of Infection and Immunity, St. George’s University of London, London, United Kingdom4National Reference Center For Invasive Mycoses & Antifungals, Molecular Mycology Unit, Cnrs Umr2000, Institut Pasteur, Paris, France5Paris-diderot, Sorbonne Paris Cité University, Institut Pasteur, Molecular Mycology Unit, Cnrs Cmr2000, Parasitology-Mycology Laboratory, Lariboisière Saint-Louis Fernand Widal Hospitals, Assistance Publique-Hôpitaux de Paris, Paris, France6Infectious Diseases, APHP, Hopital Necker-Enfants malades, Paris, France

Across the world, fungal pathogens are omnipresent in the environment, affect over a billion and kill more than 1.5 million people. Despite these figures, invasive fungal infections (IFIs) do not gather as much press coverage as tuberculosis or malaria. AIDS represents one of the major risk factors for IFIs together with other acquired immunodeficiencies. Invasive fungal infections should be a concern everywhere by their incidence, severity and high burden. Worldwide IFIs are not recognized due to lack of surveillance systems together with a lack of mandatory reporting, limited teaching in medical schools and therefore limited awareness and expertise of clinicians and biologists, lack of reliable diagnostic procedures for many IFIs, and limited number of commercialized antifungal drugs. In resource limited countries, the lack of basic laboratory equipment and poor access to essential medicines are additional major barriers. Outcomes of cryptococcal meningitis, a leading cause of HIV-related death, are still poor despite updated WHO guidelines. The DREAMM project aims to reduce the mortality of cryptoccocal meningitis in resource limited settingsby providing rapid diagnostic tests, essential medicines and reinforcing basic and mycology trainings for all staff members involved (technicians, nurses, physicians, biologists). Through this program, a better awareness of all IFIs is expected and results are already visible.

## Symposium 15 Aspergillus in the ICU

### S15.1. Influenza-associated aspergillosis in ICU: when the flu get mouldy

WautersJ.Medical Intensive Care Unit, University Hospitals Leuven, Leuven, Belgium

Worldwide, 3-5 million people develop severe influenza every year, leading to 50.000-100.000 deaths annually in the European Union and the USA. of the patients hospitalized, 5-10% needs ICU admission due to severe illness. Patients with severe illness initially present with typical influenza symptoms, but are rapidly evolving into respiratory deterioration due to bacterial superinfection but also influenza in itself can cause severe acute respiratory distress syndrome (ARDS), the latter being associated with a mortality of 14% to 41%. Although bacterial co-infection is already known for decades, influenza-associated aspergillosis (IAA) was recently found to be a frequent and severe complication of influenza pneumonia in critically ill patients. Classically, invasive pulmonary aspergillosis (IPA) typically occurs in a severely immunocompromised host. Influenza-associated aspergillosis (IAA) was occasionally described decades ago and several small case series were reported recently. 65% of the reported cases did not have classic host factors for IPA as defined by the EORTC/MSG. Recently, we published a large multicentre case-control study within the Dutch-Belgian Mycosis Study Group (7 Belgian and Dutch ICUs, 2009-2016) including 432 severe influenza patients (positive influenza polymerase chain reaction (PCR)), 315 of them being EORTC negative. As a control group, 315 influenza negative and EORTC negative patients, admitted to the ICU with severe community-acquired pneumonia (CAP), were selected. In the 432 severe influenza patients, IPA was diagnosed in 19% (83/432), a median of 3 (IQR 0-7) days after ICU admission. The IPA incidence in the 117 immunocompromised (EORTC positive) influenza patients was as high as 32% (38/117), while 14% (45/315) of the non-immunocompromised (EORTC negative) influenza patients developed IPA. In contrast, only 5% (16/315) of the non-immunocompromised influenza-negative controls developed IPA (p<0·0001). The 90-day mortality in influenza patients with and without IPA was 51% and 28%, respectively (p<0·0001). Moreover, in this retrospective cohort study, influenza was found to be independently associated with IPA (aOR 5·2, 95% CI 2·6-10·3, p<0·0001), besides a higher APACHE II score, male sex and use of corticosteroids. Though awareness of IAA is rising, it remains unclear why patients with severe influenza infection develop IPA. Influenza-induced necrosis of the bronchial tree might provide a gateway for *Aspergillus* infection. Further, severe influenza might alter innate and adaptive host facilitating fungal infection and genetic variation might also influence the susceptibility to IPA. Finally, corticosteroid (CS) therapy is a known independent risk factor for the development of IPA at the ICU. In summary, IAA is an early and frequent complication of influenza pneumonia and increases the probability of death with a factor 2 to 3 in critically ill patients with severe influenza. An aggressive diagnostic approach should be pursued, but early diagnosis is difficult due to unspecific clinical and radiological presentation. Future studies should evaluate whether a faster diagnosis and/or antifungal prophylaxis could improve outcome of influenza-associated aspergillosis. Reference: Schauwvlieghe AFAD, Rijnders BJA, Philips N et al. Dutch-Belgian Mycosis study group. Invasive aspergillosis in patients admitted to the intensive care unit with severe influenza: a retrospective cohort study. Lancet Respir Med. 2018;6(10):782-792.

### S15.2. Invasive aspergillosis in patients with underlying liver cirrhosis

PrattesJ.Section of Infectious Diseases and Tropical Medicine, Medical University of Graz, Graz, AustriaInvasive aspergillosis (IA) has increasingly reported in critical ill patients without classical risk factors like hematological malignancy, neutropenia or solid organ transplantation. Liver cirrhosis represents the main underlying disease in some of these patients, highlighting the relevance of cirrhosis as a risk factor for IA development. Whereas the overall incidence of IA in patients with liver cirrhosis is very low (<1%), the risk for IA increases when liver function deteriorates and patients are in need for intensive care. Reasons for susceptibility of cirrhotic patients for developing IA are diverse. Cirrhosis-associated immune dysfunction summarizes both, systemic inflammation due to cirrhosis and cirrhosis associated immunodeficiency. The latter one may be due to systemic glucocorticoid treatment but also due to alterations of the innate as well as adaptive immune functions. Impaired expression of Fc*γ* –receptor on neutrophils, impaired neutrophil mobilization, phagocytic activity, chemotaxis, bacterial phagocytosis and killing are present in cirrhotic patients. However, the degree of immune dysfunction correlates with the stage of the liver disease putting patients with end stage liver disease at highest risk for IA development. Awareness for IA in cirrhotic patients has to be warranted as diagnosis may be challenging. Blood biomarkers, like galactomannan and beta-d-glucan, lack sensitivity and clinical presentation may be atypical. In case of IA suspicion early chest CT scan and bronchoscopy may be needed.

### S15.3. Aspergillus in the ICU – Management of IA in Renal Failure

Rautemaa-RichardsonR.^1^,^2^,^3^,^4^1Division of Infection, Immunity and Respiratory Medicine, University of Manchester, Manchester, United Kingdom2Mycology Reference Centre, Excellence Center For Medical Mycology (ecmm), Wythenshawe Hospital, Manchester University NHS Foundation Trust, Manchester, United Kingdom3National Aspergillosis Centre, Manchester, United Kingdom4Department of Infectious Diseases, Manchester University NHS Foundation Trust, Manchester, United KingdomRenal failure is a common complication in critically ill patients. In addition, angioinvasive aspergillosis is associated with renal lesions and renal failure. The challenge is that the main intravenous drugs used to manage invasive aspergillosis (IA), namely amphotericin B and voriconazole (with cyclodextrin) are nephrotoxic. Also, in critically ill patients with multi organ failure, the pharmacodynamics and pharmacokinetics are highly variable, which results in a significant risk for toxicity as well as suboptimal dosing and treatment failure. The impact of renal failure on the management of IA depends on whether the patient is on renal replacement therapy (RRT) or not. Once on RRT, the main consideration is the risk for suboptimal drug levels until the system is saturated with the drug. However, it is important to keep in mind that the initiation of RRT does not remove the risk for further or permanent drug-induced kidney injury. Once the patient starts to respond to the treatment, their haemodynamics stabilise and their kidney function improves, there is a risk for both toxicity and subtherapeutic levels. Therefore, in the case of azoles, critically ill patients on intensive care unit (ICU) require regular therapeutic drug monitoring (TDM) and repeated dose adjustments. This also applies to azoles not associated with kidney toxicity (isavuconazole). Whilst TDM is an important part of monitoring the safety and efficacy of antifungal treatment, microbiological diagnostics are another important tool in patient management. Sputum high-volume culture, PCR and galactomannan measurements can be used to monitor treatment response whereas blood galactomannan is less useful in non-neutropenic setting. However, it is not rare that these tests remain positive for prolonged periods despite good clinical response.

### S15.4. Fungal pneumonia in critically ill cirrhotics: Spectrum, Outcomes, Comparison of diagnostic methods and Biomarkers

KaleP.^1^KhillanV.^1^SarinS.K.^2^1Clinical Microbiology, Institute of Liver and Biliary Sciences, New Delhi, India2Hepatology, Institute of Liver and Biliary Sciences, New Delhi, India

**Objectives:** Liver cirrhosis causes immune dysregulation and increased susceptibility to fungal infections. Futher there are associated co-morbidities like diabetes mellitus, renall impairment and malignancy which predispose to fungal infections. Hence early diagnosis of fungal infections and initiation of appropriate empiric or pre-emptive antifungal is essential to reduce morbidity and mortality associated with it. We studied the epidemiology, spectrum, risk factors of fungal infections and antifungal susceptibility pattern for guiding empiric therapy. We also compared the rapid diagnostic methods and biomarkers for early diagnosis fungal pneumonia in critically ill cirrhotics.

**Methods:** Single-center, prospective cohort study of 100 critically ill cirrhotics with fungal pneumonia between January to September 2018. All patients met the diagnostic criteria for liver cirrhosis by clinical, biochemical, and ultrasonography findings. Respiratory samples were processed for fungal culture and real time PCR. Antifungal susceptibility testing was done by Broth microdilution method in accordance with CLSI guidelines. Biomarkers; bronchoalveolar lavage (BAL) and serum Galactomannan, and serum procalcitonin measured on day 1, 3 7. Mortality within one month of diagnosis or discharge was analyzed.

**Results:** *Aspergillus flavus* was the most common species (70/100,70%). Risk factors for fungal pneumonia included neutropenia (p 0.03), steroids prior to ICU admission (p 0.02), prolonged (>21 days) hospitalization (p<0.05). Culture positivity was 80%. Culture was not inferior to real time PCR for diagnosis of fungal pneumonia. Antifungal susceptibility testing revealed, all *A. flavus* and *A. fumigatus* isolates sensitive to azoles, amphotericin B and echinocandins. BAL galactomannan was early prognostic marker with median rise >3.5 over the index value. Median PCT level was higher from day 1in the fungal pneumonia non-survivor than survivor group (3.29 vs. 0.8ng/ml), with higher 30-day mortality (72%). Baseline PCT at admission to ICU was higher in non- survivors; levels on D3 and D7 were persistently higher. Higher PCT level was associated with bacterial co-infection (48%), antibiotic (74%) and antifungal therapy and renal failure and mortality.

**Conclusion:** Cirrhotics who are neutropenic, have prolonged hospitalization and exposed to steroids have a high risk of fungal pneumonia. *Aspergillus flavus* predominate as in consensus with Asian epidemiology. Culture methods are reliable and combination of molecular test with BAL galactomannan is useful for rapid diagnosis. However the cutoff should be defined with epidemiological correlation. High serum procalcitonin level is an independent prognostic biomarker of mortality risk in fungal pneumonia which reaches nearly 70%. In our study the baseline PCT at admission to ICU was higher in non- survivor group, levels on D3 and D7 were persistantly higher. High index of suspicion and early detection is required in advanced cirrhosis

### S15.5. IPAFLU survey: Invasive Aspergillosis among Patients with Severe Influenza in Intensive Care Units

HoltappelsM.^1^JacobsC.^1^ChillerT.^2^FortenberryJ.^3^JacksonB.^4^LagrouK.^5^TodaM.^4^VeerdonkF. Van De^6^VerweijP.E.^7^WautersJ.^8^1Laboratory For Clinical Infectious and Inflammatory Disorders, KU Leuven, Leuven, Belgium2Mycotic Diseases Branch, Centre for Disease Control, Altanta, United States of America3Pediatric Critical Care, Children’s Healthcare of Atlanta, Atlanta, United States of America4Mycotic Diseases Branch, Centre for Diseases Control, Altanta, United States of America5Department of Microbiology, Immunology and Transplantation, KU Leuven, Leuven, Belgium6Infectieziekten, Afdeling Interne Geneeskunde, Nijmegen, Netherlands7Medical Microbiology, RadboudUMC, Nijmegen, Netherlands8Medical Icu, UZLeuven, Leuven, Belgium

**Objectives:** Invasive aspergillosis usually occurs in people with weakened immune systems. However, several reports describe fatal *Aspergillus* pulmonary infections in previously healthy patients who were hospitalized with a severe influenza virus infection. Recently, a retrospective cohort study, conducted at seven intensive care units (ICUs) in Belgium and the Netherlands during 2009–2016, found that invasive pulmonary aspergillosis occurred in 19% of patients with severe influenza requiring admission to the ICU. We conducted an international survey to determine broader knowledge and awareness of influenza-associated aspergillosis (IAA), as well as the diagnostic use of galactomannan (GM) in bronchoalveolar lavage (BAL) and serum in critically ill patients with severe influenza.

**Methods:** Members of the Extracorporeal Life Support Organization (ELSO), the Society of Critical Care Medicine (SCCM), and the European society of intensive medicine (ESICM) network (*n* = 20,093) were invited to participate in an online 11-question survey. Participants were asked about their roles in the ICU, ICU size, number of patients with influenza admitted to the ICU per influenza season, use of neuraminidase inhibitors (NAIs), frequency of obtaining lower respiratory specimens and GM testing on serum and BAL samples, and their knowledge on the occurrence of IAA.

**Results:** A total of 468 (2.3%) members responded, with 225 (48%) residing in the United States, 151 (32%) in Europe, and 92 (20%) in other countries, including Australia and countries in South America, Asia and Africa. Most (89%) participants were critical care physicians. Large ICU departments were more common in the United States compared with Europe. The majority of participants reported <30 influenza cases per season, and NAIs were the preferred antiviral treatment (92%). European participants reported obtaining lower respiratory specimens and determining GM on BAL and serum more commonly than U.S. participants (p < 0.001) (Figure 1). Globally, 66% (*n* = 303) of respondents denied hearing of or seeing IAA cases in the past five years (Figure 2). However, 57% of European participants reported seeing ≥1 patient with IAA compared with only 17% of U.S. participants (p < 0.001) and 36% of participants in other countries (p = 0.007).

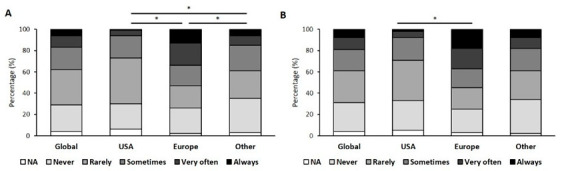

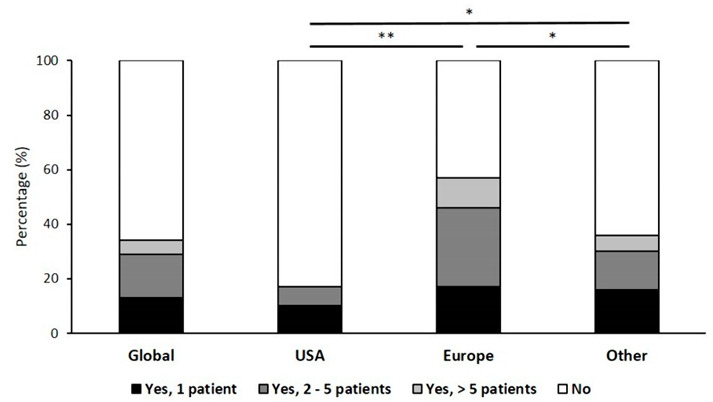





**Conclusion:** More participants in Europe reported having seen IAA cases in compared with the United States and other countries. Although the observed differences in IAA cases could be explained by true variation in IAA prevalence, the condition might be underdiagnosed outside Europe. The results suggest that such underdiagnosis might partly explain the differences in IAA awareness in Europe compared with the United States and other countries.

### S15.6. Trend of Candidemia with Bloodstream infection in Intensive care units from 2006 to 2017: Results from the Korean National Healthcare-associated Infections Surveillance System

ChoiY.H.^1^KimE.J.^1^KwakY.G.^2^YooH.M.^3^ChoiJ.Y.^4^ShinM.J.^5^KimS.R.^6^YooS.-Y.^7^ChoN.-H.^8^1Department of Infectious Diseases, Ajou University School of Medicine, Suwon-si, Gyeonggi-do, Republic of Korea2Department of Internal Medicine, Inje University Ilsan Paik Hospital, Goyang, Republic of Korea3Infection Control Office, Inje University Sanggye Paik Hospital, Seoul, Republic of Korea4Infection Control Unit, Chung-Ang University Healthcare System, Seoul, Republic of Korea5Infection Control Office, Seoul National University Bundang Hospital, Seongnam, Republic of Korea6Infection Control Office, Korea University Guro Hospital, Seoul, Republic of Korea7Adjunct Assistant Professor, College of Nursing, The Catholic University of Korea, Seoul, Republic of Korea8Department of Infection Control, Gangnam severance hospital, Yonsei university, Seoul, Republic of Korea

**Objectives:** Candidemia is an important healthcare-associated infections (HAIs) in intensive care units (ICUs). The incidence trend and distribution of candidemia over a 12-year period through the Korean National Healthcare-associated Infections Surveillance System (KONIS) data were analyzed.

**Methods:** The KONIS system was established in 2006, and has performed prospective surveillance for HAIs including bloodstream infection (BSI) and causative pathogen in ICU. We evaluated yearly trends of the frequencies of the causative pathogens and candidemia. All statistical analyses were 2-sided and performed the Cochran-Armitage test for trend and the Cochran–Mantel–Haenszel mean score test, by use of SAS software.

**Results:** From 2006 until 2017, 2,248 candidemia cases occurred in 9,184,264 patients-days. For BSI, the proportion of candidemia gradually increased (for BSI from 15.2% in 2006 to 16.6% in 2017, *P* < 0.05) (Figure 1). The pooled mean incidence rate of candidemia was 0.24 per 1,000 patient days. Most frequent pathogen for BSI was *Staphylococcus aureus* from 2006 to 2012, however, *candida* species emerged as the most frequent pathogen since 2013. *Candida albicans* (39.9%) was the most frequent pathogen for candidemia, followed by *C. tropicalis* (20.2%) and *C. parapsilosis* (18.2%) (Figure 2). There was no significant change in the distribution of *candida* species by year (*P* = 0.29). Central-line associated BSI were the majority (92.5%). In subgroup analysis, there was a significant association with the increase in the proportion of candidemia by year in the presence of organ transplant wards (from 18.9% in 2006 to 21.1% in 2017), hospitals with less than 500 beds (from 2.7% in 2006 to 13.6% in 2017), or surgical ICUs (from 16.2% in 2006 to 21.7% in 2017) (*P* < 0.05).

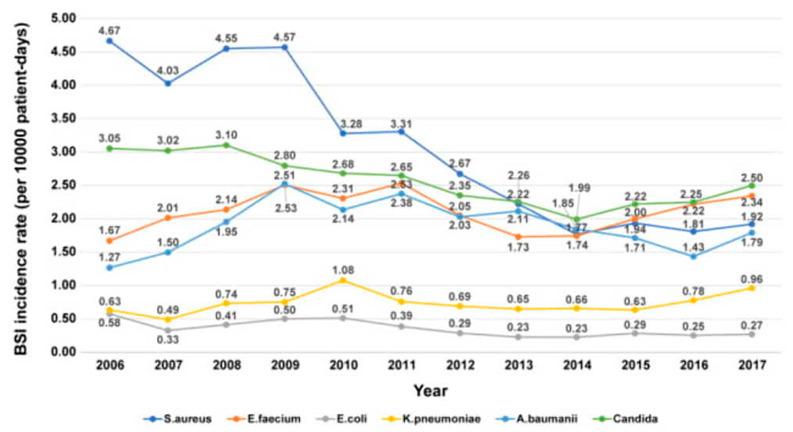

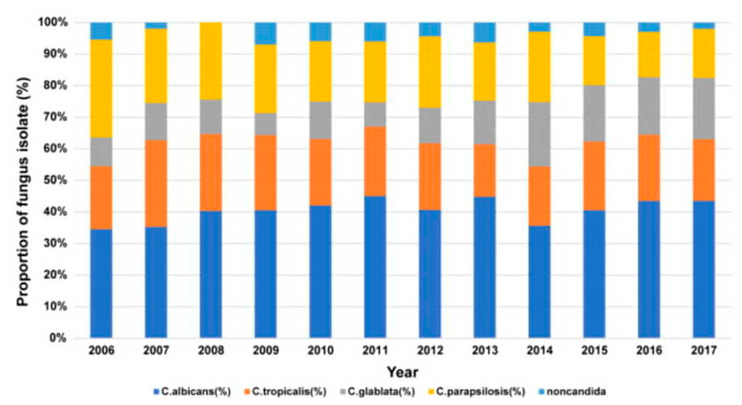





**Conclusion:** The proportion of candida as nosocomial pathogen for BSI has increased in Korea. Especially, the proportion in hospitals with less than 500 beds and surgical ICUs is increasing, so appropriate infection control program is needed. This work was supported by the Research Program funded (M2018A060000011) by the Korea Centers for Disease Control and Prevention.

## Symposium 16 Dermatology

### S16.1. Cutaneous Aspergillosis: Is it so rare?

Arikan-AkdagliS.Medical Microbiology, Mycology Laboratory, Hacettepe University Medical School, ANKARA, TurkeyInvasion of skin by *Aspergillus* is an uncommonly encountered clinical picture. Primary cutaneous aspergillosis (PCA) indicates lesions due to direct inoculation of the fungus at the otherwise injured site. The lesions usually develop following trauma, surgery or burn wounds. One significant feature of PCA is that it may be observed in immunosuppressed as well as immunocompetent individuals. Premature neonates also constitute a unique group of predisposed hosts due to skin fragility. PCA may disseminate and be lethal particularly in immunocompromised patients. Secondary cutaneous aspergillosis (SCA), on the other hand, develops either due to hematogenous spread or direct invasion of the skin from adjacent infected structures, such as paranasal sinuses. Skin and visceral involvement or multiple skin lesions in two or more noncontiguous areas suggest disseminated *Aspergillus* infection. It is estimated that skin involvement in invasive aspergillosis occurs at rates of 1-5%. The skin lesions in CA appear to have variable presentations, including macule, papule, plaque, subcutaneous nodule, ulceration with necrosis, and hemorrhagic bulla. The exact prevalence of CA is unknown and there are limited number of dedicated series and case reports. Cases of CA are probably underdiagnosed and underreported due also to difficulties in diagnosing fungal infections in general. In addition, the affected patient population is heterogeneous and complex with multiple comorbidities and this further complicates the issue. For optimal management of patients with CA, skin biopsy should immediately be taken from suspected lesions particularly in predisposed individuals for histopathological and direct microscopic examinations and mycological culture.

### S16.2. Is resistance a problem for dermatophytosis?

NenoffP.^1^MonodM.^2^VermaS.B.^3^BurmesterA.^4^EbertA.^1^SingalA.^5^GuptaS.^6^WiegandC.^4^HiplerU.-C.^7^WittigF.^1^KrügerC.^1^KochD.^1^VasaniR.^8^SaraswatA.^9^MadhuR.^10^PandaS.^11^DasA.^11^KuraM.^12^JainA.^13^GraeserY.^14^UhrlaβS.^1^1Partnership Dr. C. Krueger & Prof. P. Nenoff, Laboratory of medical microbiology, Roetha OT Moelbis, Germany2Dermatology Service, Centre Hospitalier Universitaire Vaudois, Lausanne, Switzerland3“Nirvan” and “In Skin” Clinics, Vadodara, India4Klinik Für Hautkrankheiten, Universitätsklinikum Jena, Jena, Germany5Department of Dermatology & Std, University College of Medical Sciences & GTB Hospital (University of Delhi), Delhi, India6Department of Dermatology and Venereolgy, Maharishi Markandeshwar Institute of Medical Sciences and Research, Mullana, India7Klinik Für Hautkrankheiten, Universitätsklinikum, Jena, Germany8Bhojani Clinic, Mumbai, India9Dermatology, Indushree Skin Clinic, Lucknow, India10Department of Dermatology (mycology), Madras Medical College, Chennai, India11Department of Dermatology, KPC medical college, Kolkata, India12Department of Dermatology, Grant Medical College, Mumbai & Sir JJ Group of Hospitals, Mumbai, India1312415, Doctor’s Nest, New Rajeev Gandhi Nagar, Kota, India14Institut Für Mikrobiologie Und Hygiene, Universitätsmedizin – Charité, Berlin, Germany

**Objectives:** Resistance of dermatophytes against antifungal agents was not perceived as a problem for decades. Therapy failure in onychomycosis or tinea capitis was never related to a reduced *in vitro* susceptibility of the causative species of dermatophytes. The situation has changed dramatically. The starting point, was, most likely, India. An incredible increase in chronic recalcitrant dermatophytoses over the past few years has been noted in India. The main causative pathogen is the zoophilic dermatophyte *Trichophyton* (*T*.) *mentagrophytes* ITS genotype VIII. 

**Patients and methods:** Three epidemiologic exercises, for the purpose of speciation and also to look into the possibility of antifungal resistance were undertaken with cooperation from nine Indian centers with German and Swiss mycologists. A total of 291 isolates (278 *T*. *mentagrophytes* VIII, and 13 *T*. *rubrum*) were included in the terbinafine antifungal susceptibility testing and genetic point mutation analysis of the squalene epoxidase (SQLE) gene. Itraconazole and voriconazole minimal inhibitory concentrations (MICs) were determined. 

**Results:** High resistance rates of 66.7% (*T*. *mentagrophytes* VIII), and 27.3% (*T. rubrum*) to terbinafine were found in the first multicentric study from India. 76.0% *T*. *mentagrophytes* VIII and 57.1% *T. rubrum* strains of two further studies (New Delhi and Mullana) showed *in vitro* resistance to terbinafine. The *T. mentagrophytes* VIII strains collected from multiple geographic regions of India showed high frequency of single point mutations in the SQLE gene leading to terbinafine resistance. All resistant *T. mentagrophytes* strains (200) harboured missense mutations with subsequent amino acid substitutions, most frequently Phe^397^Leu (90.0%), either as a single substitution or in combination with Ala^448^Thr. On the other hand, terbinafine sensitive strains (78) showed almost exclusively a SQLE wild type (23.1%) or a single Ala^448^Thr substitution (73.1%). Substitutions less commonly encountered included Leu^393^Phe, Leu^393^Ser, Ser^395^Pro, Gln^408^Leu, His^440^Tyr and Ser^443^Pro. The number of terbinafine resistant dermatophytes in Northern India has increased significantly from 2017 to 2019 (69.0% in 2017; 72.0% in 2018; 78.0% in 2019). Additionally, we noted a rise in SQLE double mutants, which shows a selection advantage for this genotype combination. 

**Conclusion:** The dramatic increase in terbinafine resistant *T*. *mentagrophytes* ITS VIII from all over India within such a short period of time underscores the issue of development of resistance in patients with chronic dermatophytoses. A strong association was found between *in vitro* terbinafine resistance of *T. mentagrophytes* and the occurrence of single point mutations of the SQLE gene and distinct amino acid substitutions, respectively. Alterations at position Phe^397^Leu, Leu^393^Ser, Leu^393^Phe, Gln^408^Leu, His^440^Tyr, of the SQLE were associated with terbinafine resistance. Increasing number of patients in Europe (Germany, Finland, Estonia) and from United States have been seen to be presenting with chronic recalcitrant dermatophytosis due to terbinafine-resistant *T*. *mentagrophytes* VIII from India. Transmission of the Indian *T*. *mentagrophytes* VIII to other countries due to travel, migration and in general, globalization, appears to be a serious issue even from a public health perspective. Further studies are the need of the day to understand this phenomenon better and find answers to combat it.

### S16.4. Blastomycosis

SchwartzI.S.University of Alberta, Edmonton, CanadaAmong the main endemic mycoses of North America, blastomycosis is least understood. Until recently, blastomycosis was thought to be a single clinical disease caused by a single pathogen, *Blastomyces dermatitidis*. However, recent re-analyses of isolates from North American and global collections have revealed substantial genetic and phenotypic diversity in *Blastomyces*. Classical blastomycosis, now known to be caused by *B. dermatitidis* and the cryptic species *B. gilchristii*, is limited primarily to eastern and midwestern parts of North America. In addition, *B. helicus*, a species morphologically and physiologically distinct from *B. dermatitidis* species complex, has been described as the cause of atypical, disseminated blastomycosis in immunocompromised hosts from western and mountainous parts of the United States and Canada. Moreover, blastomycosis from Africa and the Middle East - long recognized to have clinical differences from the disease in North America - has been determined to be primarily caused by *B. percursus* and an additional novel species. Clinicians and microbiologists should be aware of these additional species, how they differ from *B. dermatitidis*, and the clinical and epidemiological spectrum of blastomycosis.

### S16.5. Galleria mellonella as a novelty model to study host – pathogen interaction in Malassezia furfur CBS 1878

RamírezA.M. Celis^1^GiraldoC.M. Parra^2^PinedaE.N. Pinzón^1^1Biological Sciences, Universidad de los Andes, Bogotá, Colombia2Microbiology, Pontificia Universidad Javeriana, Bogotá, Colombia

**Objectives:** *Malassezia furfur* is a lipid-dependent yeast that is part of the animal and human skin mycobiota. This species can cause dermatological infections in the immunocompetent host until fungemia in immunosuppressed patients and neonates with parental lipid nutrition. However, aspects related to virulence traits and antifungal resistance is not entirely understood. Thus, the establishment of a model to study host-pathogen interaction has been a matter of attention in the last decade. The silkworm *Galleria mellonella* is a useful animal model characterized by its tolerance to a wide range of temperatures, including human temperature 37 °C; also, the immune response against pathogens is similar to the innate immunity in humans. Hence, this exciting model has been used for the large-scale screening of fungal pathogens such as *Candida* spp., *Cryptococcus neoformans*, and the activity of antifungal drugs in dermatophytes. This study aim to evaluate the feasibility of using *G. mellonella* larvae as an invertebrate model for infection with *Malassezia furfur* CBS 1878

**Methods:** *M. furfur* CBS 1878 (Westerdijk institute, Utrecht, The Netherlands) was used for all experiments. An inoculum was adjusted to four different concentrations (1.5 × 10^9^ UFC/mL to 1.5 × 10^6^ UFC/mL) and 10 μl were injected in the last pro leg of *G. mellonella*. Survival analysis was performed for statistical processing using Prism software (GraphPad Software, Inc.). Infected larvae and controls were maintained at 33°C and checked daily. Hemolymph analysis was conducted to evaluate the capacity of phagocytosis of hemocytes. Experiments to corroborate the fungal load were also conducted. Each treatment was conducted in triplicate (technical replicates), and the whole experiment was repeated three times (biological replicates). Statistical analysis were performed using R package.

**Results:** Survival test reveals that at least all silkworms injected with *M. furfur* CBS 1878 at 1.5 × 10^9^ UFC/mL died within five days, whereas all silkworms injected with saline were alive. Fungal load data were in agreement with the inoculums tested. Besides, activation of the immune system of *G. mellonella* and the interaction between the yeast with different cells of the hemolymph was observed.

**Conclusion:** This study is the first approach to the implementation of an invertebrate model *Galleria mallonella* to the future study of the host-pathogen interaction and the antifungal activity in *M. furfur* CBS1878. We demonstrated that injection of this yeast into silkworm hemolymph killed silkworms. We found the most effective inoculum was 1.5 × 10^9^ UFC/mL with a short time of survival on average of five days. This model promise to be an efficient infection model to conduct easy and reliable approaches that perform to underpin our knowledge in *Malassezia* genus.

## Symposium 17 HIV associated cryptococcal meningitis

### S17.3. Towards having antifungal drugs in low-resource areas

KolteI.^1^LeclèreA. Sturny^2^Boyer-ChammardT.^2^KivuyoS. Lesikari^3^MfinangaS.^3^KanyamaC.^4^KouanfackC.^5^LortholaryO.^6^DromerF.^7^BicanicT.^1^MolloyS.^1^HarrisonT.^1^LoyseA.^1^1Infection & Immunity, St Georges University of London, London, United Kingdom2Molecular Mycology, IP Paris, Paris, France3National Institute for Medical Research, Dar es Salaam, Tanzania4UNC Project-Malawi, Lilongwe, Malawi5Hôpital Central de Yaoundé, Yaoundé, Cameroon6Infectious Diseases, APHP, Hopital Necker-Enfants malades, Paris, France7National Reference Center For Invasive Mycoses & Antifungals, Molecular Mycology Unit, Cnrs Umr2000, Institut Pasteur, Paris, FranceThe results of the ACTA trial underpinned the development of new 2018 WHO guidance for the treatment of cryptococcal meningitis with short course amphotericin B deoxycholate (AmBd) + flucytosine (5FC) becoming the new gold standard for resource limited settings. The alternative recommended regimen is a two-week oral course of 5FC and fluconazole. However, access to key diagnostic tests such as the cryptococcal antigen lateral flow assay (CrAg LFA) and essential antifungal medicines including 5FC for the treatment of HIV-related cryptococcal meningitis is severely lacking in African low-and middle-income countries (LMICs) where disease burden and mortality is highest. This talk will highlight longstanding advocacy and implementation efforts to ensure access to these lifesaving tools and train frontline healthcare workers (HCWs) working on their optimal use so as to effectively reduce mortality in resource limited settings. We will focus in particular on illustrating the respective work of the cryptococcal meningitis action group (cryptoMAG), the DREAMM project, and outline the recently announced Unitaid/CHAI project on advanced HIV disease. The cryptoMAG group is an international group of stakeholders including St George’s University of London (SGUL), Centers for Disease Prevention & Control (CDC), WHO, Médecins sans Frontières (MSF) and Institut Pasteur amongst notable others that has been advocating for access to diagnostic tests and essential medicines for cryptococcal meningitis since 2013. DREAMM (Driving REduced AIDS-associated Meningo-encephalitis Mortality) is an implementation project currently ongoing in Tanzania, Cameroon and Malawi. It uses mixed methodology including co-designed training programs and local health system strengthening centred around African leadership to effectively and sustainably reduce mortality linked to HIV-related meningitis, a leading cause of advanced HIV disease. Lastly, in January 2019 Unitaid announced its program on AHD in partnership with the Clinton Health Access Initiative (CHAI). This program will enable access to the CrAg LFA and essential antifungal medicines including 5FC in 7 African LMICs, cryptococcal meningitis being a leading cause of death from advanced HIV disease.

### S17.4. Fluconazole resistance in cryptococcal meningitis

BicanicT.Infection & Immunity, St Georges University of London, London, United Kingdom

In much of sub-saharan Africa, where Cryptococcus is the most common cause of community acquired meningitis, the gold standard treatment of Amphotericin B with flucytosine is not available, and fluconazole monotherapy is the norm for all phases of CM treatment: induction, consolidation and maintenance. Even at higher doses of 800-1200 mg/d, fluconazole is slow to clear Cryptococcus from CSF. Although primary resistance is rare in global surveys using MIC testing, the intrinsic mechanism of fluconazole resistance, reversible disomy of chromosome 1, is unstable: in the absence of drug pressure, it occurs in only a very small subpopulation of colonies and will be missed by conventional MIC testing. In a study in Tanzania in patients with HIV-associated cryptococcal meningitis, we performed serial lumbar punctures in 20 patients receiving fluconazole monotherapy versus fluconazole combined with flucytosine, plotting the rate of clearance of the total population and subpopulation able to grow on fluconazole, and saving colonies for subsequent whole genome sequencing. Fluconazole heteroresistance was detectable in all clinical Cryptococcal isolates.from CSF of all patients prior to initiation of therapy. The proportion of resistant colonies in CSF increased during the first two weeks of treatment in all patients receiving Fluconazole at 800-1200mg/d, but was suppressed in those receiving combination with flucytosine. Genomic analyses revealed high rates of aneuploidy in heteroresistant colonies, as well as in clinical isolates from patiets experiencing relapse. The predmonant mechanism in human infectionwas disomy of chromosome 1 (containing the drug target gene ERG11 and the efflux pump AFR1) and heteroresistance positively correlated with in vitro drug efflux pump activity. We also undertook fluconazole PK analyses in CSF for a subpopulation of patients: Monte Carlo simulations from the clinical PK/PD model predicted that only a minority of patients (13%) receiving doses of fluconazole at 1200 mg/d sterilise their CSF after 2 weeks, with 83% having a persistant subpopulation resistant to fluconazole. Our findings demonstrate that fluconazole when used alone is a sub-optimal agent for treating cryptococcal meningitis due to rapid selection of a resistant sub-population which comes becomes predominant within 2 weeks. Efforts are underway to make the combination oral therapy of cluconazole with flucytosine more widely available and implementable in Africa.

### S17.5. Current and future clinical trials on HIV-associated cryptococcal meningitis.

HarrisonT.Centre For Global Health, Institute of Infection and Immunity, St George’s University of London, London, United Kingdom

The ACTA trial demonstrated that in centres in Sub-Saharan Africa, an induction regimen of 1 week amphotericin B (AmB) and flucytosine (5FC), followed by fluconazole (FLU) 1200 mg/d in the second week, was associated with reduced 10-week mortality compared with the then internationally recommended regimen of 2 weeks of AmB+5FC. 2 weeks of the oral combination of FLU plus 5FC was non-inferior to this standard; and 5FC was superior to FLU, as the partner drug given with AmB. The results have been further supported through 12-month outcome and economic analyses, and have driven both a change in WHO guidelines for the treatment of HIV-associated cryptococcal meningitis, and increasing access to 5FC through a Unitaid programme. The ongoing AMBITION-CM trial is testing single high dose (10mg/kg) liposomal amphotericin B (L-AmB), given with a 2-week oral FLU+5FC backbone, against the new standard of 1 week AmB+5FC. A randomized phase II study had shown non-inferior early fungicidal activity (EFA) with single high dose L-AmB vs daily L-AmB at 3 mg/kg/d for 14 days. The hope is that the L-AmB arm will be at least as effective and better tolerated and easier to deliver than one week of AmB deoxycholate. The trial includes a pre-planned health economic evaluation. Over 300 participants have been enrolled. Any further improvements in treatment may depend on introduction of new drugs into induction regimens, or novel host-directed or mechanical strategies. Viamet-1598, a fungal Cyp51 inhibitor, and a compound series from Amplyx Pharmaceuticals that target Gwt1, an enzyme required for fungal cell wall localization of GPI-anchored mannoproteins, are 2 promising candidates, and it is hoped both can advance to phase II EFA studies. Immunotherapeutic approaches should be further investigated but will need to be carefully targeted based on rapid assays of patient specific immunity. Investigators at Duke University are exploring the possibility of using neurapheresis to filter fungal cells from the CSF and rapidly reduce the organism load. Meanwhile efforts continue to prevent the development of clinical cryptococcal infection in patients with advanced HIV disease, and improve the results of the cryptococcal antigen (CrAg) screen and pre-emptive treatment strategy for patients with low CD4 cell counts. Despite pre-emptive fluconazole, mortality for those screening CrAg positive is significantly higher than for those who are CrAg negative; many of those with high CrAg titre have evidence of sub-clinical meningitis; and fluconazole failures have been increasingly well documented. A study looking at single high dose L-AmB to treat CrAg positive patients in screening will take place in Uganda, and a trial to test the oral FLU+5FC combination against FLU alone for CrAg positive patients identified at screening is planned in South Africa and Tanzania.

## Symposium 18 Antifungal stewardship in the era of resistance

### S18.1. Stewardship and azole resistant aspergillosis: a challenge for farmer or physician?

VerweijP.E.Center of Expertise in Mycology Radboudumc/CWZ, Nijmegen, the Netherlands

Aims of antibiotic stewardship include treating infectious diseases with the appropriate drug, preventing overuse and minimizing resistance selection. These goals are frustrated by the emergence of azole resistance in Aspergillus fumigatus. Recent studies showed that voriconazole-treated patients with invasive aspergillosis have a 20% lower survival when the infection was caused by a voriconazole-resistant isolate compared with voriconazole-susceptible infection. Appropriate initial therapy is thus critical but diagnosis of resistance is slow when based on fungal culture and MIC-testing. To ensure appropriate initial antifungal therapy combination antifungal therapy is recommended if resistance frequencies exceed 10%. The Dutch guideline for management of invasive aspergillosis indeed recommends voriconazole/isavuconazole in combination with an echinocandin of liposomal amphotericin B is the azole-susceptibility is unknown as resistance surveillance indicated a resistance rate of 15%. Therefore, many patients are exposed to increased toxicity and drug interactions, while the majority still have an infection due to an azole-susceptible isolate. To improve antifungal stewardship programs accurate resistance surveillance is required as well as rapid and sensitive diagnostic resistance tests. As the main driver of resistance to medical azoles in A. fumigatus is azole fungicide use in the environment, interventions are needed to reduce the burden. Recent studies have identified specific conditions that facilitate resistance selection and the next step is to design and evaluate interventions that preclude resistance selection in the environment.

The azoles represent an important class for both medical treatments and food production. Maintaining this class for both application is therefore critical and requires investment in rapid diagnostic tests and stewardship programs.

### S18.2. Rapid diagnosis of fungal infections: Impact on stewardship

KanjS.Department of Internal Medicine, Division of Infectious Diseases, American University of Beirut Medical Center, Beirut, Lebanon

Invasive fungal infections (IFI) remain a challenge to the treating physician because of difficulty in diagnosis and high rates of morbidity and mortality associated with these infections, which occur predominantly in the immunosuppressed and critically ill patients. The incidence of invasive fungal infections, especially candidiasis, aspergillosis, and mucormycosis, continues to rise. With the excessive use of azole antifungal agents, we have witnessed a global shift of fungal population to more resistant species and genera. In addition, the increased consumption of antifungal agents in agriculture have selected for resistant *Aspergillus* species. There is a wide variation in the epidemiology of fungal infections worldwide. It is no longer acceptable to manage IFI without identifying the species of the infecting organism and its susceptibility profile to the various antifungal agents. Neighboring countries might have a different epidemiology depending on antifungal practices in humans and the environment. Whereas guidelines can be of great help in the management of patients, making the correct diagnosis is of paramount importance. Studies have shown that delay in the initiation of appropriate antifungal therapy is associated with a significant increase in mortality. Therefore, there is an urgent need to make a prompt diagnosis of IFI. In the past, histopathology, fungal cultures, and Candida scores in the right clinical setting guided the diagnosis and therapy of IFI. More recently, the availability of newer diagnostic tools such as Aspergillus galactomannan, β-D glucan, and polymerase chain reaction, has been of great help in speeding the diagnosis of IFI. These tools have also been used for screening and guiding pre-emptive therapy in high-risk patients. Studies have shown that combining these new tools increases the sensitivity and specificity with an impact on fungal infection-free survival in some patients and allows for early discontinuation. More recently, the development of new molecular tools such as Matrix-Assisted Laser Desorption/Ionization – Time of Flight, FilmArray, Light Cycler, T2Candida assay, and others, allows the rapid detection of fungal pathogens and determines resistance markers with a turnaround time of only a few hours. Investigational tools such as Proximity Ligation Assay, Breath Fungal Secondary Metabolite Signature, and Siderophore-based Molecular Infection Imaging are also promising in facilitating the diagnosis of IFI in the future. In addition, the development of point-of-care testing is helpful, especially in diagnosing cryptococcal meningitis in some developing countries where access to microbiology laboratory is limited, or in diagnosing invasive Aspergillosis using Lateral Flow Devices. All these new diagnostic tools have a tremendous impact on antifungal stewardship as they allow physicians to discontinue antifungal agents earlier when not needed or to de-escalate them to narrower-spectrum agents. These efforts will ultimately translate into decreasing antifungal resistance and reducing costs to healthcare, which are essential elements of stewardship.

### S18.5. Optimising antifungal stewardship: An evaluation of candidaemia guideline compliance and clinical outcome

CottomL.^1^JonesB.^2^1Department of Medical Microbiology, Glasgow Royal Infirmary, NHS Greater Glasgow & Clyde, Glasgow, United Kingdom2Department of Medical Microbiology, Glasgow Royal Infirmary, Glasgow, United Kingdom

**Objectives:** Candidaemia remains an important cause of nosocomial infection, associated with significant morbidity and mortality. Over the last decade there has been a growing awareness that improvements to stewardship and robust surveillance is needed to ensure the judicious use of antifungal therapy. A guideline should never be construed or serve as a standard of care, rather recommendations should always be interpreted alongside individual patient findings and be guided by local epidemiology and antifungal susceptibility knowledge. The aim of this study was to evaluate compliance with the current 2016 Infectious Diseases Society of America (IDSA) guidelines and assess the impact on clinical outcome. Adherence to management recommendations was assessed using the EQUAL Candida Score of the European Confederation of Medical Mycology (ECMM). A secondary aim was to assess clinical outcome (30 day, 90 day and all-cause mortality) with an echinocandin compared to fluconazole as the initial treatment (primary therapy) for candidaemia.

**Methods:** A retrospective analysis was performed over 18-months (January 2017 to June 2018). Data was analysed from five large university teaching hospitals within Greater Glasgow and Clyde; the largest NHS organisation in Scotland serving a population of 1.2 million. Patients were identified using our laboratory information management system and electronic clinical record systems. Only cases where a diagnosis of candidaemia had been confirmed and the patient had been actively treated were included for analysis. The source of infection and antifungal management was reviewed for each patient. The EQUAL candida score was calculated for each case. The 30-day, 90-day and all-cause mortality rate was determined for the study population.

**Results:** A total of 131 patients were identified as having a candidaemia. The most common *Candida species* isolated was *C.albicans* (40%; 53/131) followed by *C.glabrata* (27%; 35/131). An echinocandin was initiated as primary therapy in 52% (68/131) of cases. In 62% of cases the patient had an indwelling line that was attributed as the direct source/nidus of infection. 21% of cases were related to a urinary tract source and/or urological instrumentation. The mean EQUAL Candida Score was found to be 13 (range 9-17), with the maximum score for guideline adherence being 16 for non-CVC patients and 19 for CVC carrier (score adjusted as initial blood culture volume was not known for study population). No statistically significant difference in clinical outcome (30 day mortality) was found when comparing an EQUAL Candida Score of ≤13 with > 13 (chi-square calculation; *p*-value 0.76). For the study population, a statistically significant difference in clinical outcome (30 day mortality) was found when comparing fluconazole to an echinocandin as initial treatment/primary therapy with a survival benefit being found in favour of fluconazole (chi-square calculation; *p*-value 0.039).

**Conclusion:** Our study provides a valuable evaluation of compliance. Interesting, increased compliance was not found to improve clinical outcome. Furthermore, the results from our study suggest that fluconazole as initial therapy was non-inferior to an echinocandin for the treatment of candidaemia, with a survival benefit being observed. This study supports the importance of an effective program of surveillance to promote antifungal stewardship and the judicious use of antifungal agents.

## Symposium 19 Pneumocystis

### S19.1. Recent diagnostic strategies in Pneumocystis pneumonia

AlanioA.Paris-diderot, Sorbonne Paris Cité University, Institut Pasteur, Molecular Mycology Unit, Cnrs Cmr2000, Parasitology-Mycology Laboratory, Lariboisière Saint-Louis Fernand Widal Hospitals, Assistance Publique-Hôpitaux de Paris, Paris, France

Pneumocystis jirovecii pneumonia (PCP) is one of the most commonly diagnosed opportunistic infection in HIV-positive patients. In addition, the rising number of immunocompromised HIV-negative patients at risk of P. jirovecii infection (immunosuppressive therapies, allogeneic bone marrow or solid organ transplantation) is an emerging concern. PcP is difficult to diagnose in HIV-negative patients owing to the non-specific pulmonary symptoms and signs associated. Microscopic visualization of cysts or trophic forms in respiratory specimens based on immunofluorescence stainings is the most sensitive microscopic method and still considered as the gold-standard test to diagnose this infection. Bronchoalveolar lavage fluids are the most sensitive specimen type, since P. jirovecii is an organism associated to lung alveoli. PCR-based methods play an increasing role in the lab, initially developed to circumvent decreased fungal load in HIV-negative patients and detection in upper respiratory specimens. Real-time quantitative PCR (qPCR) is definitely the only format adapted to detect P. jirovecii since the risk of contamination is minimal and quantification is possible. A negative qPCR in BAL, but not in non-invasive respiratory specimens, allows to rule out PCP diagnosis. Quantitative results have been used for years to try to discriminate PCP (high fungal load) from carriage/colonization (low fungal load). However, these methodologies have limited use since intermediate fungal load are inconclusive. Recent advances in the comparison of the performances of the different qPCR assays showed that standardization is certainly one of the clues to move forward with this question. In parallel, the combination with (1-3)-b-D-Glucan (BG) detection in serum helps but do not completely resolve the problem. BG detection has a high sensitivity and a high negative predictive value for diagnosis of PcP in immunocompromised HIV-positive and -negative patients, but a positive result may also indicate the presence of other invasive fungal infections. The clinical utility of these diagnostic tests and the diagnostic strategies in HIV or non-HIV patients should be further assessed in prospective, randomized interventional studies.In the meantime, the status of the host should be better defined with regard to the evolution of P. jirovecii carriage. New screening and prophylaxis strategies to prevent transmission in hospital settings should be proposed and discussed

### S19.2. Management of Pneumocystis Pneumonia in HIV-infected Patients

KovacsJ.Associate Clinical Professor of Medicine, The George Washington University School of Medicine and Health Sciences, Washington, DC, United States of America

*Pneumocystis* pneumonia (PCP) remains one of the most common life-threatening opportunistic infections in HIV-infected patients, despite the tremendous benefits seen with combination antiretroviral therapies. PCP continues to be seen in the current era primarily because at-risk patients are unaware of their HIV infection or are not in continuous medical care. Infection appears to represent recent acquisition in many cases rather than reactivation of latent infection. Primary clinical manifestations include fever, shortness of breath, and a non-productive cough. CXR will typically show diffuse infiltrates though it may be normal in 10-20% of cases; chest CT scans will almost invariably be abnormal, however. Diagnosis is based on detection of the organism in respiratory samples such as induced sputum or BAL. Because the organism cannot be cultured, organism detection relies on colorimetric or immunofluorescent staining, or PCR assays. The latter are highly sensitive but less specific than the former, in part because PCR can identify colonization or subclinical infection not requiring therapy. Assays to measure (1-3)-β-D-glucan levels have poor specificity and should not be relied on as the exclusive diagnostic modality. The preferred first-line drug for treatment is trimethoprim-sulfamethoxazole, which targets 2 steps in *Pneumocystis* folate metabolism. Alternatives for patients with mild to moderate disease (A-a O_2_ gradient <45 mm Hg) include clindamycin-primaquine, trimethoprim plus dapsone, and atovaquone. Alternatives for patients with more severe disease requiring parenteral therapy include clindamycin-primaquine (primaquine is available only for oral administration) and pentamidine. Caspofungin should not be used as a single agent since it affects only the cyst form and not the more numerous trophic form of the organism; further, there are no controlled studies evaluating its efficacy. Corticosteroids should be administered in patients with PaO_2_ <70 mm Hg or (A-a) O_2_ gradient >35 mm Hg, ideally within 3 days of starting therapy, and be continued using a tapering regimen while anti-*Pneumocystis* therapy is administered. Although there are no randomized trials, meta-analyses suggest that clindamycin-primaquine is more efficacious than pentamidine. For patients failing therapy, there are no randomized trials to address optimal management, and whether it is better to add drugs or to change regimens. PCP can be effectively prevented in at-risk patients (e.g. CD4 <200 cells/mm^3^) by using trimethoprim-sulfamethoxazole, or alternatively dapsone, dapsone-pyrimethamine, atovaquone, or aerosol pentamidine. In patients recovering from an episode of PCP, secondary prophylaxis should be initiated after completion of therapy. Prophylaxis can be discontinued after the immune system has reconstituted to CD4 >200 cells/mm^3^ for at least 3 months. In patients with poor immune reconstitution (CD4 >100 but <200 cells/mm^3^) but with HIV suppression to below detection limits of commercial assays (typically <20 copies/ml) for 3-6 months, prophylaxis can also be discontinued. Prophylaxis should be continued in patients with CD4 <100 cells/mm^3^ even with HIV suppression. Mutations have been identified in the dihydropteroate (DHPS) gene of *Pneumocystis*, the target of sulfamethoxazole and dapsone, primarily in patients receiving those drugs for prophylaxis. Although these mutations appear to confer at least low level resistance, their clinical significance is uncertain.

### S19.4. Pneumocystosis in neonates

CalderonE.Instituto de Biomedicina de Sevilla and CIBER de Epidemiología y Salud Pública, Seville, Spain

Pneumocystosis has been well recognized as a severe pulmonary disease and an important cause of morbidity and mortality in premature infants or debilitated neonates since the mid-twentieth century when *Pneumocystis* was acknowledged as the causing agent of interstitial plasma cell pneumonia. This form of pneumocystosis was observed before, during and after World War II in Europe, where it reached epidemic proportion with more than 500 cases confirmed by autopsy. Unexpectedly, this epidemic pneumocystosis disappeared in the same manner it had been emerged in Europe. Although data on pediatric *Pneumocystis* pneumonia (PcP) from developing countries are scarce mostly based on small series of cases, PcP in infants seem to remain as an important medical problem in those areas. Nowadays, the interest in *Pneumocystis* infection goes beyond PcP because a new spectrum of *Pneumocystis*-related disease seems to emerge in immunocompetent infants. Serological studies and use of molecular techniques to identified *Pneumocystis jirovecii* have shown that this microorganism probably is one of the more frequent infectious agents faced by humans in everyday life and that the first exposition to this pathogen happen in most children early in life, probably in the neonatal period. However, primary *Pneumocystis* infection currently goes unrecognized because it has been presumed to be an asymptomatic or mild nonspecific disease. However, recent reports indicate that it can present clinically as a self-limiting upper or lower acute respiratory tract infection. *Pneumocystis* infection was identified by specific PCR in 24% of infants with bronchiolitis from France and in 29.4% of Cuban infants and toddlers with whooping cough. In another study of 422 children hospitalized with acute respiratory tract infection, *Pneumocystis* was detected in 16% of infants. However, a marked difference occurred in the age distribution, as the prevalence was 48% in infants ages 50 to 112 days, 13% in infants ages 113 to 265 days, and 2% in in infants younger than 49 days. In a study carried out in Santiago de Chile, *Pneumocystis* DNA was detected in specimens from 51.7% of infants who died unexpectedly in the community, but only 15% had pneumonia. This study also showed that *Pneumocystis* infection is more frequent than viruses before the age of 6 months, and that it has a consistent peak between 2 and 5 months of age. The significance of these findings is unclear but shows the high prevalence of *Pneumocystis* infection in infants. Recently, in Spain, a high prevalence of *Pneumocystis* infection has been described in preterm neonates and this infection was associated to higher risk to develop neonatal respiratory distress syndrome. Increasing evidence suggests that the most common respiratory infection-affecting infants is the mild and sneaky, primary infection by *Pneumocystis*. This infection goes currently unrecognized and has been neglected as a subclinical irrelevant infection by contrast with the severe PcP affecting the immunocompromised patients. However, compelling new evidence suggests that this infection may be pathogenic to certain infant age groups and that microbiome host interactions in early life may condition the development of altered immune responses in older infants or adults.

### S19.5. Evaluation of a Novel Commercial Loop-mediated Isothermal Amplification (LAMP) Assay for Rapid Detection of Pneumocystis jirovecii

SchmidtD.ScharmannU.BuerJ.RathP.-M.Institute of Medical Microbiology, University Hospital Essen, Essen, Germany

**Objectives:** *Pneumocystis jirovecii* pneumonia (PCP) is an increasing problem in immunocompromised patients. Detection of *P. jirovecii* in respiratory samples is usually based on microscopy or PCR. Both methods require experienced technical personnel and are either insensitive or time-consuming. In this study, a prototype of the eazyplex *Pneumocystis jirovecii* assay (Amplex Diagnostics, Germany) was established. The assay is based on LAMP technique (loop-mediated isothermal amplification). It requires minimal hands on time for sample manipulation (below 5 minutes) and performance of the assay itself (25 minutes) and could be a reliable technique to detect *P. jirovecii*.

**Methods:** In total, 145 respiratory samples from 134 critically ill patients (65% male, median age = 64 years) were investigated, using the LAMP assay in comparison with the RealStar Pneumocystis jirovecii PCR 1.0 (altona Diagnostics, Germany). For the LAMP assay 25µl of sample were mixed with 125µl RALF buffer (resuspension and lysis fluid, Amplex) and incubated for 3 minutes at 99°C. 25µl of this suspension were added to the wells of the test strip afterwards. The LAMP assay was performed in a Genie II Mk2 device (Amplex). For qPCR, 300µl of sample were extracted using the Maxwell16 nucleic acid extraction platform (Promega, Germany). 10µl of DNA were used for qPCR according to the manufacturer´s recommendations. QPCR was performed on a RotorGeneQ cycler (Qiagen, Germany) in a final volume of 30µl. To determine the detection limit of the LAMP assay, compared to the qPCR, tenfold serial dilution of extracted P. jirovecii DNA and of a high positive P. jirovecii respiratory sample were investigated in both assays. Additionally, for each serial dilution time-to-positivity of the LAMP assay was plotted against cycle threshold values (ct values) of the qPCR. For quantification of the fungal load the quantification standards of the RealStar qPCR were used (1x10^4^ -1x10^1^ copies/µl).

**Results:** The LAMP assay detected 17 of 19 qPCR positive samples and 117 of 117 qPCR negative samples. The assay was invalid in nine samples that were negative by qPCR. Time-to-positivity of the LAMP assay ranged from 11-22 minutes in patient samples. Detection limit of the assay was approximately 15-20 copies of genomic DNA/µl in patient samples and in extracted DNA which corresponded with ct values of 28-29 in the qPCR.

**Conclusion:** The LAMP assay is an accurate and time saving tool for the detection of P. jirovecii in respiratory samples. Two samples that were positive by qPCR were not detected by the assay which is probably due to the significantly lower sample volume and the crude extraction method the assay uses. Nevertheless, the convenience of the performance of the assay and interpretation of the results allows laboratories with less experience and less technical equipment to perform molecular assays on a high standard.

## Symposium 20 *Candida auris*

### S20.1. Schizophrenic gram negative yeast conquering the world

MeisJ.Department of Medical Microbiology and Infectious Diseases, Excellence Center For Medical Mycology (ECMM), Canisius Wilhelmina Hospital, Nijmegen, Netherlands

*Candida auris* has been named as the new “fungal superbug” which poses a significant threat to public health during outbreaks. *C. auris* was first named and described only 10 years ago. Follow-up genomic analysis has revealed that *C. auris* has emerged almost simultaneously in this short period on 3 different continents. At present 4 major clades have been identified and last month a fifth clade was discovered. In the beginning *C. auris* was often mistaken for other Candida or yeast species when using phenotypic assimilation/fermentation identification tests. Wide introduction of MALDI-TOF technology has significantly improved accurate identification in the past few years and is now the gold standard in addition to molecular sequencing. *C. auris* has also shown resistance to several different antifungal drugs and classes of drugs. While its resistance profile varies geographically, this new yeast pathogen has acquired resistant to fluconazole, a key drug in the azole class of antifungal drugs. *C. auris* spreads in healthcare facilities in a similar way as bacteria while the occurrence and niche in the environment is elusive. Infections have been reported in a range of countries across continents, but current estimates are probably inaccurate. Impervious to both antiseptics and the three major classes of anti-fungal medications, *C. auris* has been quietly transmitted within hospitals and nursing homes in at least 37 countries such as the UK, Spain, India, Pakistan, Middle East, South Africa, Latin America and parts of the USA. Patients can be colonised with *C. auris* without becoming sick, but the pathogen seems to prefer to colonise patients who are already sick or immunocompromised (eg, cancer or transplant patients), and the very young or very old among hospitalised inpatients. Among at-risk patients who have *C. auris* candidemia or invasive candidiasis, depending on the world region, high mortality rates (~ 30–60%) have been observed. But because infection typically occurs when a person is already very sick, it may be difficult to disentangle the attributable mortality. It is important to realize that *C. auris* is predominantly a healthcare-associated infection. Prior broad-spectrum antibiotics and antifungal use can influence the risk of infection. Finally an important question is still unanswered *C. auris*: *Unde venis et quo tendis*? Reference Meis JF, Chowdhary A. *Candida auris*: a global fungal public health threat. Lancet Infect Dis. 2018;18(12):1298-1299

### S20.3. Outbreak control: "Controlling a multidrug-resistant Candida auris outbreak"

Ruiz-GaitanA.^1^,^2^1Severe Infection Research Group, Medical Research Institute La Fe, Valencia, Spain2Department of Clinical Microbiology, La Fe University and Polytechnic Hospital, Valencia, Spain

**Background:** *Candida auris* is an emerging, multidrug-resistant yeast causing hospital outbreaks. The outbreaks by *C. auris* described in Spain as well as in other countries with large outbreaks, are characterized by an exponential increase in the number of cases in a short period, suggesting a high transmission rate. **Objectives** We report the first 24 months of the ongoing *C. auris* outbreak in a tertiary hospital in Spain. The epidemiological, clinical and microbiological characteristics of candidemia episodes and environmental samples by *C. auris* were also analyzed. **Results:** 228 patients were involved in the case–control study (114 colonized/candidemia and 114 controls). All candidemia episodes were observed in adult patients (21–81 years old) and 87.8% of them were admitted to SICU. The most common underlying condition observed in both colonized and candidemia patients was polytrauma (*n* = 13, 32%) followed by cardiovascular disease (*n* = 10, 25%) and cancer (*n* = 7, 17%). Indwelling CVC (odds ratio {OR}, 13.48), parenteral nutrition (OR, 3.49), and mechanical ventilation (OR, 2.43) were the more frequently invasive procedures observed in these two groups and remained significant predictors of *C. auris* colonization/candidemia. All *C. auris* isolates were resistant to fluconazole (MICs >64 mg/L) and had significantly reduced susceptibility to voriconazole (GM, 1.8 mg/ L). All isolates were susceptible to itraconazole, posaconazole, isavuconazole, and echinocandins. Environmental sampling showed presence of the *C. auris* on sphygmomanometer cuffs (25%) patient tables (10.2%), keyboards (10.2%), and infusion pumps (8.2%). **Conclusions:** - Predictor conditions to *C. auris* colonization/candidemia are similar to other Candida species. *C. auris* colonizes multiple patient’s environment surfaces. All isolates are resistant to fluconazole and had significant reduced susceptibility to voriconazole. - Due to its high transmissibility and survival in the hospital environment, *C. auris* can cause long duration outbreaks that are difficult to detect in early stages, and it makes it difficult to control and eradicate. - The implementation of early and strict surveillance and control measures is essential to preventing the spread of the outbreak representing a significant risk to critical patients. - Immediate notification of *C. auris* to clinical and infection control teams, as well as to health authorities and institutions, is essential to implementing infection control precautions at all levels in a timely way, to prevent transmission inside and outside the hospital and to prevent the development of infections in patients who are already colonized. - This report is intended to demonstrate not only the complexity of handling and containing a *C. auris* outbreak, but also how, with the use of a series of effective infection prevention measures, the spread of this pathogen can be successfully controlled.

### S20.4. Candida auris: in developing world 

ChowdharyAnuradhaDepartment of Medical Mycology, Vallabhbhai Patel Chest Institute, University of Delhi, Delhi, India

Candida auris a multidrug-resistant(MDR) yeast that exhibits resistance to fluconazole and markedly variable susceptibility to other azoles, amphotericin B, and echinocandins has globally emerged as a nosocomial pathogen that can cause invasive infections. Candida auris was first described in 2009 by Satoh et al. as a novel Candida species, in the Candida haemulonii complex(Metchnikowiaceae), from a patient in Japan after its isolation from the external ear canal. Subsequent to the first nosocomial outbreak in South Korea in 2011, several hospitals associated outbreaks have been reported in developing world especially in India, Pakistan and Africa. Alarmingly, in less than a decade this yeast, which is difficult to treat, has become widespread across several countries causing a broad range of healthcare associated invasive infections that display clonal inter- and intra-hospital transmission. C. auris in routine microbiology laboratories remains an unnoticed pathogen as 90% of the isolates characterized by commercial biochemical identification systems such as API 20C, Vitek 2 (bioMérieux), Phoenix (BD), and MicroScan (Beckman Coulter), misidentify as a range of other Candida species. Most commonly, these isolates have been misidentified as C. haemulonii, but also C. famata, C. sake, Rhodotorula glutinis, Rhodotorula mucilaginosa, and Saccharomyces species. Rarely, C. auris has been identified as C. catenulate, C. lusitaniae, C. guilliermondii, or C. parapsilosis. However, MALDI-TOF MS is considered a more rapid and robust diagnostic technique for C. auris identification. Mass spectra can be easily added to the MALDI-TOF MS database, leading to accurate identification of C. auris to the species level. Sequencing of genetic loci, including D1/D2, RPB1, RPB2, and internal transcribed spacer (ITS) domains of the rRNA, has proven useful in the identification of C. auris, but it is not routinely used.

However, in the routine microbiology laboratories this yeast remains unidentified especially in developing world due to lack of fully equipped mycological diagnostic facilities. Further, the true burden of C. auris remains unexplored as effective surveillance of Candida spp is not available in several countries in the developing world. Antifungal susceptibility data for C. auris is of primary concern as C. auris exhibit consistently high fluconazole MICs and variable susceptibility to the other azoles, echinocandins and amphotericin B. No antifungal clinical breakpoints reported for C. auris. Studies examining the susceptibility of this organism to antifungals have used a variety of methods, including Clinical and Laboratory Standards Institute (CLSI) broth microdilution, Etest, and the Vitek 2 yeast susceptibility system. Regarding azole resistance mutations in Erg11 associated with the development of fluconazole resistance in C. albicans have also been detected in C. auris isolates. The fact that this yeast exhibit MDR clonal strains which are nosocomially transmitted is unusual in other Candida species. Therefore, the possible threat of its rapid spread in affected countries and its emergence in unaffected countries will not only challenge clinicians for its effective therapeutic management but will also bring high economic burden to countries especially in resource limited settings where modern identification facilities and access to antifungals other than FLU are limited.

### S20.5. Understanding Echinocandin Activity Towards Candida auris

KordalewskaM.^1^LeeA.^1^Garcia-RubioR.^1^ParkS.^1^BerrioI.^2^ChowdharyA.^3^ZhaoY.^1^PerlinD.S.^1^1Center For Discovery and Innovation, Hackensack Meridian Health, Nutley, United States of America2Hospital General de Medellín “Luz Castro de Gutiérrez” ESE, Medellin, Colombia3Department of Medical Mycology, Vallabhbhai Patel Chest Institute, University of Delhi, Delhi, India

**Objectives:** *Candida auris* is a recognized cause of invasive infections and health care associated outbreaks around the world. Development of echinocandin resistance has been documented in *C. auris* isolates recovered from patients treated with these drugs. Thus, a thorough understanding of *C. auris* echinocandin susceptibility profiles and resistance mechanisms is crucial for improved management. In this study, we performed a comprehensive analysis of *C. auris* echinocandin susceptibility and molecular resistance determinants.

**Methods:** A total of 106 *C. auris* isolates (Colombia, India, AR Bank) were investigated in this study. Antifungal susceptibility testing (AFST) with echinocandins (anidulafungin, caspofungin, micafungin) was performed in accordance with CLSI document M27-A3. *FKS1* gene, encoding the target enzyme for echinocandin class antifungal drugs, was amplified and sequenced. Mutation prevention concentration (MPC) determination assay was performed for echinocandin-susceptible (micafungin MIC range 0.03-0.5 mg/l), *FKS1* wild-type *C. auris*, *C. albicans* and *C. glabrata* control strains. Isolates were cultured in RPMI-1640 containing 2-fold increasing concentrations (0.03 to 128 mg/l) of micafungin, then culture aliquots were plated onto YPD plates and number of surviving cells were calculated. Recovered colonies were screened for the presence of *FKS1* mutations. Moreover, *in vivo* drug response of *C. auris* isolates to caspofungin was assessed in a murine model of invasive candidiasis.

**Results:** Four Indian isolates from a total of 106 isolates (3.8%) exhibited highly elevated MICs ≥4 mg/l at 24 h and were considered presumptively resistant to all tested echinocandins. These isolates were found to harbor an S639F amino acid substitution in Fks1. The remaining 102 echinocandin-sensitive isolates presented wild-type genotype. Micafungin was the most potent echinocandin (MIC_50_ = 0.125 mg/l). We encountered a significant challenge obtaining an accurate MIC readout for caspofungin since all tested isolates exhibited an Eagle effect (paradoxical growth effect), with the intensities of the Eagle effect varying among the isolates. In MPC determination assay with micafungin, *C. auris* isolates did not present a classical bi-modal killing pattern characteristic for fungicidal drugs activity against other *Candida* species. Response of *C. auris* to micafungin differed between isolates, but micafungin did not reach the fungicidal endpoint for any of the isolates, even when high drug concentrations (128 mg/L) were tested. Since no *FKS1* mutation was revealed by sequencing, we hypothesize the presence of extreme tolerance mechanism in *C. auris*. Echinocandins were effective *in vivo* in the treatment of invasive murine candidiasis caused by *FKS1* wild-type *C. auris* isolates (significant (P < 0.05) ~1 log10 CFU/g kidney burden reduction upon caspofungin treatment relative to vehicle controls), despite the presence of the Eagle effect *in vitro*. Mice challenged with *fks1* S639F mutants failed to respond to the drug and their kidney burdens were not much different between caspofungin treated and untreated groups.

**Conclusion:** Without established breakpoints for *C. auris*, AFST results interpretation and conclusions regarding resistance, especially when testing caspofungin, should be made with caution. The high echinocandin tolerance of *C. auris* observed *in vitro* should be further explored. Presently, the only validated determinant impacting *C. auris* pharmacodynamic response to echinocandins is the *FKS1* genotype.

## Symposium 21 Immunotherapy for opportunistic fungal infections

### S21.1. CAR T cell and NK cell therapy of invasive mycoses

KontoyiannisD.Ut Md Anderson Cancer Center, 1515 Holcombe, Houston, United States of America

Invasive fungal infections remain a major threat to the survival of immunocompromised patients and no curative therapies are currently available to treat drug resistant opportunistic fungi. In this brief talk, I will provide a conceptual overview of recent developments to use specific chimeric antigen receptor (CAR) expressing T cells and NK cells as adoptive immunotherapy. CAR T cells have been successfully used to treat B-cell malignancies. To render CAR T cell therapy specific for fungi, we designed a CAR which combines the carbohydrate-binding domain of Dectin-1 with T cell signaling domains, designated as Dectin-1-CAR (D-CAR). The pattern recognition receptor Dectin-1 was selected based on its property to bind selectively to β-1,3-glucan present on the cell wall of life threatening fungal species. I will be discussing our efforts to manufacture clinical grade D-CAR^+^ T cells and to increase T cell persistence. However, allogeneic CAR T cells carry a significant risk of graft-versus-host disease, and logistical problems of rapid and robust production remain. As the role of natural killer (NK) cells in fungal immunity has been increasingly deciphered, I will be discussing the potential benefits of cord-blood derived NK cells as an attractive, allogeneic, off-the-self source for future, logistically feasible immunotherapeutic approaches to treat invasive mycoses.

### S21.2. WBC transfusions Granulocyte Transfusions As Adjunctive Treatment of Invasive Fungal Diseases In Neutropenic Patients

PaganoL.Fondazione Policlinico Universitario A. Gemelli - IRCCS - Universita Cattolica del Sacro Cuore, Rome, Italy

The degree and duration of neutropenia are crucial prognostic factors in hematological patients (pts) with invasive fungal infections (IFIs). Since the introduction of granulocyte colony stimulating factor (G-CSF), there has been a renewal of interest in granulocyte transfusions (GTX). Granulocyte transfusions (GTX) are seldom used as a life-saving therapy for neutropenic patients with severe infections. Despite several compelling evidences of GTX efficacy in retrospective and prospective case series, no study has been successful in demonstrating a definite advantage for recipients in controlled clinical trials. Some specific issues relevant to the efficacy of this therapeutic approach, such as the primary infection, the delivered doses and schedules, and the immunological effects of GTX, are still subject to discussion today. Importantly, the awareness of biological effects accompanying the transfusion of neutrophils might support their use at standardized doses and may definitely convey significant advantages to the recipient patients. At present, despite statistical evidences are lacking, GTX are still perceived as a lifesaving tool to support neutropenic patients with life threatening IFIs until their bone marrow recovery. Sharing procedures for donor identification and cell mobilization, pursuing common criteria to identify which patients will benefit of GTX during febrile neutropenia and define indications and therapeutic cell doses are absolutely urgent to pinpoint the true advantage of using GTX. On the other hand, adopting equal end points and outcomes to evaluate both clinical response to treatment and biological functions of neutrophils and chemokines during IFI, need to be clarified.

### S21.4. Novel chimeric antigen receptor T cells for invasive aspergillosis immunotherapy

SeifM.^1^NerreterT.^1^MachwirthM.^1^EbelF.^2^EinseleH.^1^HudecekM.^1^LöfflerJ.^1^1Medizinische Klinik Und Poliklinik Ii, Universitätsklinikum Würzburg, Würzburg, Germany2Institut Für Infektionsmedizin Und Zoonosen, Ludwig-Maximilians-Universität München, München, Germany

**Objectives:** Immunocompromised patients are susceptible to invasive fungal infections mainly caused by *Aspergillus fumigatus* (Af). Adoptive transfer of *Aspergillus*-specific T cells has been shown to reduce the burden of invasive aspergillosis. Such T cells are hard to isolate and expand. An alternative option is the use of T cells modified to express a chimeric antigen receptor (CAR). CARs are recombinant receptor constructs composed of an extracellular targeting element linked to an intracellular signaling module. A recent study using Dectin 1 as targeting element proved the applicability for antifungal CAR T cells *in vitro* and *in vivo*. One main limitation of Dectin 1 is the differential expression of its target β-glucan on Af morphotypes cell wall. β-glucan is mainly exposed on the surface of swollen conidia and early germ tubes but less on hyphae. Thus, better specificity would be highly valuable. Here we propose a highly specific and effective way of redirecting T cells towards Af.

**Methods:** For the construction of AF-specific CARs, we fused an scFv, derived from an antibody directed against the Af hyphal cell wall, to extracellular IgG4-Fc spacer domains of different lengths followed by CD28 and CD3-ζ signaling domains. We non-virally expressed the CARs on the surface of primary human CD4^+^ and CD8^+^ T cells using the Sleeping Beauty retrotransposon system and co-cultured CAR T cells with Af germ tubes to evaluate specific T cell activation and direct hyphal damage.

**Results:** We could show that our CAR T cells are specific for *Aspergillus fumigatus*, as no cross-reactivity with other Aspergilli was observed. Activated CD8^+^ CAR T cells secreted mainly Th1-related cytokines (IFN-γ, TNFα, IL-8 and GM-CSF) and chemokines (mainly CCL3 and CCL4), and only one Th2 cytokine (IL-13). Similarly, CD4^+^ CAR T cells secreted mostly Th1 cytokines and chemokines, but also some Th2 cytokines (mainly IL-13 and IL-4). CARs equipped with a long extracellular spacer containing the full hinge-CH2-CH3-motif conferred superior T cell activation as compared to CARs having a short "hinge-only" spacer. Noteworthy, mainly CAR T cells containing a long spacer underwent proliferation upon activation. Upon binding to the target, the cytolytic machinery of CD8^+^ CAR T cells was activated, leading to the release of perforin and granzyme B and up to 45% hyphal damage. Furthermore, when cocultured with macrophages, both CD4^+^ or CD8^+^CAR T cells enhanced antifungal activity of macrophages.

**Conclusion:** Taken together, our results show that we successfully redirected CD4^+^ and CD8^+^ CAR T cells against Af hyphae and that fine-tuning of CAR constructs might allow the control of fungal infections in high-risk patients.

### S21.5. Comparison of circulating lymphocyte populations CD4+ and CD8+ T cells, B and NK lymphocytes according to the favorable or worsening evolution of patients with pneumocystosis

CharpentierE.^1^,^2^MarquesC.^1^MenardS.^1^BlanchardN.^1^ChauvinP.^2^GuemasE.^1^CassaingS.^2^FillauxJ.^2^ValentinA.^2^BerryA.^1^,^2^IriartX.^1^,^2^1Center for Pathophysiology of Toulouse Purpan - INSERM UMR 1043, Toulouse, France2Parasitology and Mycology, CHU de Toulouse, Toulouse, France

**Objectives:** Pneumocystosis is a severe opportunistic disease in which the host inflammatory response to the infection is decisive for the disease evolution. Although a reduced count of CD4+ T lymphocytes has been associated to a major risk of pneumocystosis, especially in HIV-positive subjects, little is known about other lymphocyte populations (CD8+ T cells, B and NK lymphocytes) or about CD4+ and CD8+ T cells subpopulations. This study aimed to compare circulating lymphocyte populations in patients suffering from pneumocystosis according to the patient evolution.

**Methods:** Levels of circulating lymphocyte populations were evaluated in the blood of 24 patients diagnosed with a pneumocystosis at Toulouse Teaching Hospital between 2016 and 2018. Among them, five were solid organ transplant (SOT) recipients, seven had a haematological malignancy (HM), five had a solid cancer (SC), three had an inflammatory disease (ID) and four were HIV-positive. CD4+ and CD8+ T cells, B lymphocytes and NK cells were evaluated with flow cytometry as well as Th1, Th2, Th17, Treg and naive, effector, memory subpopulations. The lymphocyte populations were compared between patients with favorable evolution (*n* = 16) and deceased patients (*n* = 8; 2 SOT, 2 HM, 3 SC, 1 HIV).

**Results:** Interestingly, the most significant results concerned CD8+ T cell subpopulations. Although there was no difference in global CD8+ T lymphocyte count, the proportions of memory CD8+ T lymphocytes (both central memory and effector memory) were lower in deceased subjects. At the opposite, the proportions of naive and effector CD8 lymphocytes were increased in this group. Effector memory CD8+ lymphocytes of deceased patients harboured less CD127 (IL7 receptor that induces lymphocyte survival and proliferation) whereas their CD8+ effector cells contained more CD127+ CD25+ (IL2 receptor and activation marker) cells in comparison to the CD8+ effector cells of surviving patients. These differences in CD8+ T cells were associated with reduced Th1 CD4 subpopulation and Th1/Th2 ratio in deceased patients. NK cells were also reduced in this group. There was no significant difference, either in CD4+ T cells count or in other CD4 subpopulations (Th2, Th17, Treg, naive, effector, memory) or in B cells count. Patients age, fungal load and CMV presence or absence were not significantly different in the two groups.

**Conclusion:** The implication of CD8+ T cells in pneumocystosis is currently a controversial topic with a discussed role of these cells in the fungus clearance and a suspected association with alveolar lesions. According to this study, high CD8+ memory subpopulations would be associated with a better evolution of the disease, along with Th1 CD4+ T cells. However, high naive and effector CD8 populations would be associated to a worsening evolution of the patient. The disparity of CD8+ lymphocytes subpopulations might be related to a difference of immunity stimulation by *Pneumocystis jirovecii* antigens or it could be connected with a difference of CD8+ subpopulations homeostasis possibly affected by some therapeutics or living conditions. Despite the dogma that establishes CD4+ T lymphocytes as the center of pneumocystosis pathogenesis, CD8+ T cells may also have an important role in the disease pathogenesis.

### S21.6. Glucosylceramides from Lomentospora prolificans Induce Cytokines Production and Increase the Microbicidal Activity of Macrophages

XistoM.I.D.S.^1^MuñozJ.E.^2^DiasL.S.^3^SantosG.M.P.^2^CalixtoR.O.R.^1^BernardinoM.C.^1^TabordaC.P.^4^Barreto-BergterE.^1^1Instituto De Microbiologia Paulo De Góes, Universidade Federal do Rio de Janeiro, Rio de Janeiro, Brazil2Instituto Biomédico, Departamento De Microbiologia E Parasitologia, Universidade Federal Fluminense, Niterói, Brazil3Department of Pediatric, School of Medicine and Public Health, University of Wisconsin-Madison, Madison, United States of America4Institute of Biomedical Sciences, department of Microbiology, University of São Paulo, São Paulo, Brazil

**Objectives:** *Lomentospora prolificans* is an emerging opportunistic fungus with a high resistance to antifungal agents and it can cause localized infections in immunocompetent patients and disseminated infections with a high mortality rate in immunosuppressed patients. Glucosylceramides (GlcCer) are synthetized in the majority of known fungal pathogens. They are bioactive molecules presenting different functions such as involvement in fungal growth and morphological transitions in several fungi. In this study, we report the characterization of GlcCer species in *L. prolificans* and the role of these molecules in the activation of the innate immune response.

**Methods:** GlcCer species were isolated from mycelium and conidia forms of *L. prolificans* and their chemical structures were elucidated by mass spectrometry (ESI-MS). The reactivity of *L. prolificans* GlcCer species to anti-GlcCer Mab was analyzed by ELISA. The GlcCer distribution on the surface of *L. prolificans* was analyzed by immunofluorescence using anti-GlcCer Mab. The cytokine analysis was performed by the GlcCer injection in BALB/c mice and the cytokines were quantified by ELISA according to the manufacturer’s instructions. Recruitment of Cells to the Peritoneum Cavity was analyzed FACS.

**Results:** GlcCer purified from both forms presented a major species at m/z 750 that corresponds to *N*-2-hydroxyhexadecanoyl-1-β-D-glucopyranosyl-9-methyl-4,8-sphingadienine. Monoclonal antibodies against GlcCer could recognize *L. prolificans* GlcCer species from mycelium and conidia, suggesting a conserved epitope in fungal GlcCer. In addition, *in vivo* assays showed that purified GlcCer species from both forms was able to induce a high secretion of pro-inflammatory cytokines by splenocytes. GlcCer species also promote the recruitment of polymorphonuclear, eosinophils, small peritoneal macrophage (SPM) and mononuclear cells to the peritoneal cavity. GlcCer species were also able to induce the oxidative burst by peritoneal macrophages with NO and superoxide radicals production, and to increase the killing of *L. prolificans* conidia by peritoneal macrophages.

**Conclusion:** Our results indicate that GlcCer species from *L. prolificans* are potent immune response activators. These molecules induce a strong production of NO in peritoneal macrophages with a high killing activity. *In vivo*, GlcCer species induce an immune response composed by a Th1 and Th17 cytokine profile, with recruitment of inflammatory cells to the peritoneum cavity. In this way, we believed that these molecules are very important to the immune response against *L. prolificans*, and could be used to produce antibodies, vaccines or as an adjuvant.

## Symposium 22 NGS and mycobiota

### S22.1. Bacterial-Fungal Interactions at the Airway Mucosa and Implications for Chronic Rhinosinusitis

CopeE.KaskO.KymanS.ZhangI.LeeK.The Pathogen and Microbiome Institute, Northern Arizona University, Flagstaff, United States of America

Emerging studies of the human microbiome (the trillions of bacteria, fungi, viruses, and archaea that inhabit the human body) have demonstrated the myriad of ways that host-associated microbial communities contribute to host health. In the airways, the composition and diversity of the mucosal microbial communities are related to health status. However, the relationship between airway microbiota and inflammation is not well understood. Chronic rhinosinusitis (CRS) is a significant healthcare issue; current estimates suggest that CRS is responsible for 5% of the total healthcare costs in the US and affects up to 16% of the population. In a prior study of the bacterial microbiota of CRS patients, we’ve shown that CRS patients have decreased sinonsasal bacterial diversity compared to healthy individuals. CRS patients were characterized by enrichment of one of four bacterial pathobionts, including *Staphylococcaceae* or *Pseudomonadaceae*. The objectives of this study were to determine whether bacterial and fungal microbiota interact in the airways to influence host immune response. We used ITS2 sequencing in a cohort of 100 CRS patients and healthy controls and found that, although bacterial community composition is variable across CRS patients, *Malassezia* is the predominant fungal genus in the upper airways of healthy individuals and CRS patients. We then hypothesized that species-specific bacterial-fungal interactions differentially influence host mucosal immune response. Thus, we investigated *in vitro* and *in vivo* interactions between *Malassezia sympodialis, P. aeruginosa,* and *S. aureus*. *In vitro* interactions were evaluated using the modified Kirby-Bauer Assay and Crystal Violet assay for biofilm quantification. A murine model of acute sinusitis was used to investigate relationships with the host immune response. *S. aureus* and *P. aeruginosa* were intranasally instilled in the presence or absence of *M. sympodialis*. 16S rRNA gene sequencing and FungiQuant were used to evaluate changes in microbiota composition and burden, and host immune response was measured using quantitative real-time PCR (qRT-PCR). *In vitro,* late stage planktonic and biofilm *P. aeruginosa* inhibited *M. sympodialis* growth. Co-infection of mice with *M. sympodialis* and *P. aeruginosa or S. aureus* differently influenced the immune response. In co-infected mice, expression of fungal sensing receptors (*Dectin-1*)*,* allergic responses (*IL-5* and *IL-13*) and inflammation (*IL-10,* and *IL-17*) in the murine sinonasal cavity depended on the bacterial species that was co-instilled with *M. sympodialis* (p<0.05, Mann Whitney). Mice infected with *M. sympodialis* alone did not have increased Th1, Th2, or Th17 gene expression, suggesting that this taxon can act as a commensal. However, mice that were co-infected with *M. sympodialis* and *S. aureus* had increased *IL-5* gene expression (p = 0.01, Mann Whitney) compared to *M. sympodialis* or *S. aureus* alone. These results show that species-specific interactions in airway associated microbiota relate to distinct host immune response. These interactions may be implicated in some subsets of CRS disease. Our understanding of the role of bacterial-fungal interactions in CRS will contribute to development of novel therapies toward manipulation of the airway microbiota.

### S22.3. Characterization of indoor dust microbiota in homes of asthma and non asthma patients

GangneuxJ.P.^1^SassiM.^1^CannP. Le^2^LemireP.^2^1Laboratoire De Parasitologie-mycologie, CHU de Rennes, Rennes, France2Inserm Irset- Umr 1085, EHESP, Rennes, France

**Objective:** The exposure of occupants of housing to indoor air pollutants has increased in recent decades. Among microbiological contaminants, bacterial and fungal aerosols remain poorly studied and the debate on the impact of these aerosols on respiratory health is still opened. This study aimed to assess the diversity of indoor microbial communities in relationship with the health of occupants.

**Methods:** Measurements were taken from dwellings of 2 cohorts in Brittany (France), one with children without any pathology and the other with children and adults with asthma. Thirty dust samples were analyzed by next generation sequencing with a 16S and 18S targeted metagenomics approach. Analysis of sequencing data was performed using qiime 2, and univariate and multivariate statistical analysis using R software and phyloseq package. Dust samples were collected by vacuuming the floor in the child’s bedroom using a Dustream Collector (Indoor biotechnologies, United Kingdom) sampler-fitted vacuum cleaner (40 μm mesh nylon filter, domestic vacuum cleaner).

**Results:** A total of 2,637 prokaryotic (589 at genus level) and 2,153 eucaryotic taxa were identified (856 fungal taxa (39%) and 573 metazoa (26%)). Among Fungi, only 136 taxa were identified at genus level. The four main bacterial phyla were identified: *Proteobacteria (53%), Firmicutes (27%), Actinobacteria (11%)*, *Bacteroidetes (8%)*. The three main fungal phyla identified were: Ascomycota (84%), Basidiomycota (12%) and Mucoromycota (3%). No bacterial nor fungal taxa were significantly associated with asthma versus control group.

A trend of over representation in Asthma group versus control was observed for *Corynebacterium* (p-value = 0.013, adj. p-value = 0.09) and *Streptococcus* (p-value = 0.025, adj. pvalue = 0.112) genus. No correlations between the populations of fungi and the asthma conditions as well as the habits of the occupants/the characteristics of the dwellings or other environmental characteristic were observed.

**Conclusion:** Our findings provide evidence that dust samples harbor a high diversity of human-associated bacteria and fungi. Molecular methods such as NGS are reliable tools for identifying and tracking the bacterial and fungal diversity in dust samples, a less easy strategy for the detection of eucaryots at least using18S metagenomics approach. This study showed that the detection of some bacteria may be associated to indoor air of asthmatic patients. Regarding fungi, a higher number of samples and sequencing with more depth could allow to reach significant signatures.

### S22.5. An ECMM-ESCMID survey on goals and practices for mycobiota characterization using Next Generation Sequencing in European laboratories

GangneuxJ.P.^1^GueganH.^1^VandenborghtL.-E.^2^Buffet-BataillonS.^1^EnaudR.^3^DelhaesL.^4^The Esghami-Efisg (Escmid) and ecmm NGS Study Group^1^1Laboratoire De Parasitologie-mycologie, CHU de Rennes, Rennes, France2Genoscreen, Lille, France3University of Bordeaux, Centre de Recherche Cardio-Thoracique de Bordeaux, U1045, CHU de Bordeaux, CRCM Pédiatrique, CIC 1401, Bordeaux, France4Parasitology Mycology, Univeristy of Bordeaux, Centre de Recherche Cardio-Thoracique de Bordeaux, U1045, F-33000, Bordeaux, France, Bordeaux, France

**Objectives:** Although substantial efforts have been made to investigate about the composition of the microbiota, fungi that constitute the mycobiota play a pivotal role in maintaining microbial communities and physiological processes in the body. Next generation sequencing (NGS) techniques have clearly renewed methodology to characterize host-associated microbiota in a more comprehensively way, and have consequently revealed a much more diverse microbiota than was previously thought to exist.

**Methods:** We constituted a panel of experts from the European Society of Clinical Microbiology and Infectious Diseases (ESCMID-ESGHAMI and -EFISG study groups) and the European Confederation of Medical Mycology (ECMM) to address an international-survey focusing on laboratory’s current procedures regarding their goals and practices of mycobiota characterization using NGS.

**Results:** The laboratories reported their main reason of using NGS to study the mycobiota primary to understand the pathophysiology of a dysbiosis (*n* = 20), at a second level, to contribute to a diagnosis (*n* = 16) to implement a therapeutic strategy (*n* = 12), or to evaluate the exposome (environmental studies)(*n* = 10). Respiratory tract represented the most studied site, followed by the digestive tract, Sample originated in half of the laboratories from sputum (*n* = 14), or from stool (*n* = 12), broncho-alveolar lavage (*n* = 11), environmental (*n* = 6), oral wash (*n* = 4) and skin (*n* = 1). Target used for mycobiota analysis was equal among 18S rRNA (*n* = 11), ITS1 (*n* = 11) and ITS2 (*n* = 11), while some centers used the shotgun approach (*n* = 5). When asked about data analysis, database used for alignment were mostly UnitE (*n* = 8 labs), and both Genebank and CBS databases (*n* = 7 labs). Other details will be presented.

**Conclusion:** We can therefore report a photography of mycobiota analysis in 28 laboratories from 19 countries, affiliated to working groups from ESCMID and ECMM and draw some conclusions of the diversity of approaches. NGS to study mycobiota is a great challenge for understanding pathophysiological questions in experimental studies as well as for driving diagnostic strategies. Techniques will rapidely develop, moving from targeted NGS to shotgun approaches and whole genome sequencing will also be very useful in the next years in the field of mycology, but also for an holistic reasoning.

## Symposium 23 The funny life of *C. albicans* in the oral tract

### S23.3. Modulation of the Fungal-Host Interaction by the Intra-Species Diversity of C. albicans.

KannanA.^1^LavalG.^2^BougnouxM.-E.^3^DEnfertC.^4^SanglardD.^1^1Microbiology Institute, University Hospital Lausanne, University of Lausanne, Lausanne, Switzerland2Center For Bioinformatics, Institut Pasteur, Paris, France3Laboratoire De Parasitologie Mycologie, APHP Hôpital Necker-Enfants-Malades, Paris, France4Fungal Biology and Pathogenicity, Institut Pasteur, INRA, Paris, France

*Candida albicans* clinical isolates intrinsically display high diversity in their population genetic structure. Regulation of several host factors and development of *C. albicans* diseases can be influenced by diverse genetic backgrounds of the fungus. Using a collection of 96 genome-sequenced isolates, we aim here to understand by so-called Expression Quantitative Trait Loci (eQTLs), the influence of *C. albicans* genome diversity on fungal transcriptomic profiles in contact with human keratinocytes (TR146 cells). The impact of the fungal genomes on the host transcriptome can thus be probed. Pilot experiments using 5 distinct commensal clinical isolates (including the reference isolate SC5314) in contact with TR146 cells at different time points (0.3-, 1-,2- and 6h) revealed distinct transcriptional differences between the isolates. After 6h co-culture, only 194 *C. albicans* genes were found to be commonly differentially regulated (2-fold threshold) as compared to SC5314 in all four isolates. These data suggest a heterogeneous response between these different isolates, even with a small number of isolates. This heterogeneous response underpins the large genome diversity existing between the different *C. albicans* clades. Based on the overall summary of top 200 variable *C. albicans* genes among all the five isolates, we observed that the isolate CEC3665 most varied from SC5314 upon infection. This isolate differed from the others principally by genes involved in hyphal development and adherence. Consistently, CEC3665 exhibited low adherence to TR146 cells as compared to the others. The use of these 5 initial isolates allowed the optimization of several other experimental parameters (optimal exposure time to TR146 cells, variations within biological triplicates) that was used for probing the transcriptional profiles of the entire 96 genome-sequenced collection. The current state of the 96 genome-sequenced collection analysis will be presented.

### S23.4. Intestial Th17 drives airway inflammation in ABPA

BacherP.Institute of Clinical Molecular Biology & Institute of Immunology, Kiel, Germany

Th17 cells provide protection at barrier tissues but may also contribute to immune pathology. The relevance and induction mechanisms of pathologic Th17 responses are poorly understood. In humans, Th17 responses are particularly important against Candida albicans, a mucocutaneous fungal pathobiont. In contrast, the role of Th17 cells for other pathogenic fungal species is unclear. We used antigen-reactive T cell enrichment (ARTE) for the ex vivo analysis of human T helper cell responses against 30 common members of the human mycobiome. We identified the mucocutaneous pathobiont Candida albicans as the major direct inducer of human anti-fungal Th17 cells. Th17 cells directed against other fungal species are induced by T cell cross-reactivity to C. albicans. Intestinal inflammation expands total C. albicans- and cross-reactive Th17 cells. Strikingly, Th17 cells cross-reactive to the airborne fungus Aspergillus fumigatus are selectively activated and expanded in patients with airway inflammation, such as asthma, COPD and cystic fibrosis and especially during acute allergic bronchopulmonary aspergillosis (ABPA), suggesting their specific contribution to lung pathology. This indicates a direct link between protective intestinal Th17 responses against C. albicans and lung inflammation caused by airborne fungi. We identified heterologous immunity to a single, ubiquitous member of the mycobiota as a central mechanism for systemic induction of human anti-fungal Th17 responses and as a risk factor for pulmonary inflammatory diseases.

### S23.5. Persistence of clonal azole-resistant isolates of Candida albicans from a patient with Chronic Mucocutaneous Candidiasis in Colombia

Ceballos-GarzonA.^1^,^2^WintacoL.M.^3^PadillaC. Hernández^4^HozA. De La^4^BeltránS.L. Valderrama^4^MorenoC. Alvarez^4^PapeP. Le^1^RamirezJ.^3^Parra-GiraldoC.M.^2^VelezN.^2^1Department of Parasitology and Medical Mycology of The University of Nantes., Universite de Nantes, Nantes, France2Unidad de Proteómica y Micosis Humanas, Grupo de Enfermedades Infecciosas Departamento de Microbiología, Facultad de Ciencias, Pontificia Universidad Javeriana, Bogotá D.C, Colombia, Bogota, Colombia3Grupo De Investigaciones Microbiológicas-ur (gimur), Universidad del Rosario, bogota, Colombia4Grupo De Investigación En Enfermedades Infecciosas, Unidad De Infectología, Hospital Universitario San Ignacio (husi) Pontificia Universidad Javeriana, Hospital Universitario San Ignacio, bogota, Colombia

**Objectives:** To evaluate and to characterize phenotypically and molecularly clinical *Candida albicans* isolates, obtained from a patient with Chronic Mucocutaneous Candidiasis.

**Methods:** sixteen *C. albicans* were isolated from a 25-year-old male with several recurrent fungal infections and admitted to the San Ignacio Hospital in Bogota, Colombia. The isolates were recovered during four-year from a different anatomical sites. The isolates were typified by MultiLocus Sequence Typing, and evaluate for susceptibility to triazoles, Caspofungin and Amphotericin B by microdilution method (CLSI). Their pathogenic capacity was evaluated in the *Galleria mellonella* model.

**Results:** Genotyping of all clinical isolates showed the persistence of the same Diploid Sequence Type (DST). Isolates changed their susceptibility profile over time acquiring resistance to azole drugs. In Contrast there were no significant statistical differences in pathogenicity during the period. 

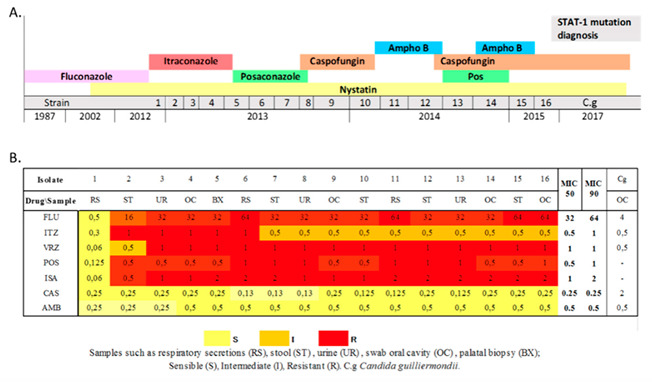



**Figure 1**. Characteristics of *Candida spp* isolates **A.** Antifungal treatment over time **B.** Antifungal susceptibility results.

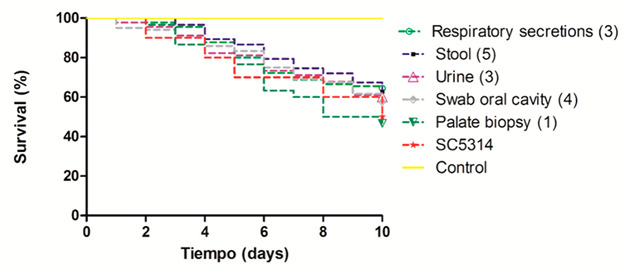



**Figure 2**. Survival curve of average according to anatomical origin. Kaplan-Meier plots of *G. mellonella* survival after infection with *C. albicans* 3x10^5^ UFC/ml. Inside the brackets the number of samples.

## Symposium 24 Fungal neglected tropical diseases

### S24.1. Updates on mycetoma

SandeW. Van DeDepartment of Microbiology and Infectious Diseases, Erasmus Medical Center, Rotterdam, NetherlandsMycetoma is a subcutaneous inflammatory and infectious disease of mainly the foot. It is endemic in tropical and subtropical regions where it causes a high morbidity. It was the first fungal infection recognized as a Neglected Tropical Disease by the World Health Organization by resolution WHA69.21 in 2016. Within this resolution, endemic regions were encouraged to assess the burden of mycetoma and the scientific community was encouraged to develop tools for early diagnosis and to improve treatment of mycetoma. It is now more than 3 years since this resolution and within these three years new information became available on all these subjects. In terms of assessing the burden of infection, case reports and single center studies were published from different regions in the world. Studies from Brazil, China, India, Mexico, the Philippines, Senegal, Sudan and Togo. Also reports from non-endemic regions such as Italy, Spain, Switzerland and the United States of America demonstrated that immigrants and refugees can also take mycetoma to their new homelands where they often cause problems in diagnosis due to lack of awareness. Within these reports it became evident that there are still new mycetoma causative agents discovered every year. To identify the causative agents, morphological identification and/or histology often leaded to misidentification demonstrating the need for more reliable diagnostic tools. To speed up diagnosis and make it more reliable, techniques were developed to isolate DNA directly from mycetoma grains and novel PCR primers were developed to be able to identify more causative agents. Isothermal alternatives were also explored and for histology sections an in situ hybridization probe was developed. For identification on cultures, a Maldi-Tof database was generated. To optimize treatment, an open source drug discovery platform was founded (MycetOS) which identified the fenarimols as a new class of potent anti-mycetoma drugs and other classes such as the orotomides were tested in vitro for their activity against mycetoma causative agents. Based on the in vitro potency of ravuconazole, the first ever clinical trial on a new drug was started at the mycetoma research centre in Khartoum. It is obvious that since the recognition as a Neglected Tropical Disease the mycetoma field has moved forward and will continue as such in the upcoming future.

### S24.2. Chromoblastomycosis: A true neglected tropical disease

Queiroz-TellesF.Public Health, Federal University of Parana, Curitiba, Brazil

Several infectious diseases, including helminthic, protozoal, bacterial, and viral infections but not fungal diseases other than mycetoma and chromoblastomycosis, are currently defined as “neglected diseases” by the WHO. After sporotrichosis, chromoblastomycosis is the second most prevalent implantation mycosis in the world. Chromoblastomycosis (CBM) is defined as a chronic and indolent granulomatous fungal disease caused by the transcutaneous inoculation of propagules from several species of melanized fungi from the Herpotrichiellaceae family. The most prevalent species causing chromoblastomycosis are *Fonsecaea pedrosoi*, *F. monophora*, and *Cladophialophora carrionii*. Advances in molecular taxonomy have shown that the biodiversity of the CBM agents has increased without substantial correlation with specific type of lesions or severity of the disease and response to therapy. Because several CBM sibling etiologic agents are found in soil and plant microbiota, this disease is strongly associated with cutaneous macro or micro traumas during diverse outdoor activities, such as agro pastoralism, hunting, eco-tourism, etc. Chromoblastomycosis mainly occurs in tropical and subtropical zones in Latin America, Africa, Asia and Oceania. According to recent data, the the highest incidence density of CBM (number of reported cases /10 million), occurs in the Dominican Republic (50.1), Madagascar (26.4), Gabon (23.5), Congo and Costa Rica (11.8), respectively (Santos DW, PhD Thesis) Melanized cell wall, muriform cell architecture, hydrophobicity and cell adhesion are considered virulence factors leading to increased pathogenicity, a key point for disease development. After an uncertain period of incubation, the initial lesion starts at the site of inoculation. With time, these can evolve to polymorphic cutaneous lesions. The clinical forms of the disease might be associated with cytokine cellular-mediated response and in severe cases, the production of TNF α and interleukin 10 has been detected. To confirm diagnosis, the visualization of pathognomonic muriform (sclerotic) cells is the most simple, inexpensive, rapid and 100% sensitive method. In culture, the organisms are fastidious. Molecular methods are mandatory for species identification, but they remain expensive and are not important for therapeutic guidelines. Although serologic tests (immunodiffusion and ELISA) have been validated, they are not commercially available. The therapeutic options, for CBM, include physical methods and chemotherapy. Without a doubt, excisional surgery is the best method for the treatment of CBM but only for initial small and well-delimitated cutaneous lesions. According to a few open non-comparative clinical studies, itraconazole (ITZ) is currently the best therapeutic option for CBM, at the daily dose of 200 to 400 mg from 8 to 36 months. The success rate ranges from 15 to 80%. The duration and cure rates are related to the severity of the disease, ITZ gut absorption and the etiologic agent. Terbinafine (TBF) at the daily dose of 250 to 500 mg is the second most used drug. For refractory cases, posaconazole may be used as well as the combination of ITZ with TBF or 5-flucytosine. The only way to prevent CBM is to avoid trauma Individual under occupational risk should use protective equipment such as adequate clothes, shoes and gloves. Similarly, to other implantation mycoses, there are no available immunoprophilaxis for CBM.

### S24.3. Sporotrichosis: past and future

BezerraL. LopesCIETEC/USP, São Paulo, Brazil

The description of new cryptic species with different pathogenic potentials has changed the perspectives on sporotrichosis. The Pathogenic Clade of the *Sporothrix* genus has four pathogenic species: *S. schenckii*, *S. brasiliensis*, *S. globosa* and *S. lurei.* The new emerging pathogen, *S. brasiliensis,* shows higher virulence and is mainly related to zoonotic transmission while *S. schenckii* and *S. globosa* are sapronotic agents. After 20 years of the zoonotic outbreak of sporotrichosis in Brazil the challenges to manage the disease caused by *S. brasiliensis* are enormous, including clinical-epidemiological aspects, treatment and diagnosis. The cat-transmitted disease has already spread from the South to the Northeast of Brazil with impressive number of cases in either humans or felines being reported every year. The increment of cases reported by Centers of Zoonotic Control account for over 500% between 2016 and 2018. For this reason, a national effort among scientists, clinicians and veterinaries was established to propose a guideline for the management of *S. brasiliensis* infection. This group of experts from several endemic areas is working since 2018. The data and conclusions achieved by the Feline and Human Brazilian Working Groups will be presented.

### S24.4. Novel diagnostic and treatment strategies for the Southeast Asian fungus Talaromyces marneffei

LeT.Duke University School of Medicine, DURHAM, United States of America

Novel diagnostic and treatment strategies for the Southeast Asian fungus talaromyces marneffei – Thuy Le, MD DPhil *Talaromyces marneffei* (Tm) is one of seven endemic human fungal pathogens that causes substantial global morbidity and mortality. Over just two decades, Tm has emerged as a leading cause of opportunistic infection and death in adults and children with advanced HIV disease in southern China and Southeast Asia. The mortality on antifungal therapy in both HIV and non-HIV infected patients is as high as 30%. The current diagnosis still relies on microscopy of skin lesions (which is a late manifestation and is absent in 40% of patients) and on cultures of the pathogen which takes up to 14 days. Late diagnosis is the most challenging clinical problem and drive mortality from 24% to 50%. The current treatment options are suboptimal. Intravenous amphotericin B deoxycholate is recommended for induction therapy but has serious side effects and is still not widely available. Itraconazole tablets are cheap and are widely available, but has low oral bioavailability, variable pharmacokinetics and is inferior to amphotericin B in efficacy. This talk presents new data on the comparative performance of real-time PCR assays versus antigen detection assays for rapid diagnosis of talaromycosis. Both assays have excellent specificity of >95%; however, the antigen tests are significantly more sensitive than the real-time PCR tests (>90% compared to 70-80%). Tm antigen can be detected up to 16 weeks prior culture positivity in symptomatic hospitalized AIDS patients, and Tm antigen independently predicts 12-month mortality in patients with a CD4 cell count <100 starting antiretroviral therapy. This talk will also present results of the IVAP trial, a multi-center clinical trial comparing amphotericin B and itraconazole for induction therapy in Vietnam, which demonstrated a 50% mortality reduction in the amphotericin B group. The trial showed that 98% of patients in the amphotericin B group cleared fungemia within 8 days of therapy, paving the road for a planned phase II clinical trial testing short courses of liposomal amphotericin B therapy to reduce toxicity. Efforts to test novel antifungal compounds are ongoing and should offer new and less toxic treatment options in the coming years.

### S24.5. Urban sporotrichosis: neglected micro-epidemia in the State of Minas Gerais, Brazil

StoianoffM.A. ResendeNavarroD.B. SilvaSilvaD.L.SantosD. AssisDept. of Microbiology, Federal University of Minas Gerais, Belo Horizonte MG, Brazil

**Objectives:** Sporotrichosis is a subcutaneous mycosis caused by dimorphic fungus of the *Sporothrix* complex that can affect both humans and other animals, being more common and severe in cats, and occurring more frequently in regions of tropical climate, as is the case in Brazil. Because it is not a compulsory notification disease in most Brazilian states, the true incidence of the disease is unknown. The aim of this study was to report the occurrence of cases of human sporotrichosis in the state of Minas Gerais, Brazil. **Methods:** The secretions of the ulcerated lesions were collected with the aid of sterile swab and the material was subjected to Gram staining, as well as to the culture at 28°C and 37°C. It was confirmed by the micromorphological examination that the samples belong to the genus *Sporothrix*. The molecular identification of the species is underway and the patients were referred for treatment. **Results:** It was reported the occurrence of 11 cases of human sporotrichosis between August 2018 and May 2019, nine of them from the city of Ribeirão das Neves and two from Belo Horizonte, state of Minas Gerais, Brazil. Among the patients, nine belonged to the female gender and two to the male. The patients’ ages ranged from two to 61 years and all of them reported contact with sick cats. The mean time between contact with the animals and the appearance of the lesions was 15 days. Nine patients presented lymphocutaneous form and two presented fixed cutaneous form. The patients presented lesions in the arms, forearms, fingers or in the heel. The Gram staining revealed presence of yeasts of elongated and/or rounded form, sometimes with single bud. The thermal dimorphism and the micromorphology confirmed the identification of *Sporothrix* spp. **Conclusion:** The results suggest that there may be a sporotrichosis microepidemia in the municipality of Ribeirão das Neves, with marked importance of the zoonotic transmission of the disease. According to official information, the conditions of infrastructure and sanitation are precarious in this region. The notification of this disease and studies that relate the socio-environmental and behavioral conditions that can determine the variation in sporotrichosis transmission are of great importance, in order to adopt the appropriate prevention and control measures. The contact of individuals with cats at home environment may be increasing in the last years, a fact that contributes to the increase of the possibility of infection of the population. The control of the source of infection is fundamental in this situation, with priority being given to the investigation of factors involved in the transmission dynamics of the disease, to assist in the surveillance and control measures necessary to contain its growth. Key words: Sporotrichosis, *Sporothrix* spp., Urban sporotrichosis Financial Support: CNPq, CAPES.

## Posters

### P001. induction of antifungal resistance exerted by the exposure of paclobutrazol to Aspergillus fumigatus

AraujoE. Marques De^1^,^2^PapeP. Le^2^Lima-NetoR. Gonçalves De^1^GonçalvesT.^1^GusmaoN. Buarque De^1^1Biologia De Fungos, universidade federal de pernambuco, Recife, Brazil2Ea1155 Iicimed, Nantes University Hospital, NANTES, France

**Objectives:** The resistance of Aspergillus fumigatus to azolic drugs occurs mainly through two routes: in vivo as a consequence of antifungal therapy, and in the enviroment as a consequence of the use of fungicides in agriculture. Placobutrazol is a plant growth retardant used in agriculture, which inhibits cellular growth and hormones composition. Since it is excessively applied and presents chemical stability, paclobutrazol accumulates in the soil and can affect the existing microbiota.The present study aimed to induce to the resistance and consequently the induction of CYP51 gene mutation on azoles-susceptible isolates of Aspergillus fumigatus after exposition against paclobrutazol.

**Methods:** he A. fumigatus (2 from environmental origin and 1 ATCC) were submitted to antifungal susceptibility testing by the microdilution method (MIC-EUCAST) to prove that all strains were sensitive at concentrations of 32 μg/mL of paclobrutazol and 0.5 μg/mL of voriconazole and itraconazole. For the resistance induction test, these fungi were exposed to paclobrutazol for five weeks in RPMI culture medium at 37°C in initial concentration of 4 μg/mL, and increasing to a concentration of 64 μg/mL, it was also checked whether concentration progression influenced induction using the same method, without elevation in concentration of the molecule in the medium. Over the five weeks of the test, the MIC was verified through the micro dilution method established by EUCAST and the yeastone sensitrite commercial method in order to evidence the elevation of MIC after exposure.

**Results:** The MICs obtained over the five weeks of the test showed a decreasing of sensitivity in all strains (100%) against antifungals used in the hospital routine after exposure to the paclobrutazol.

**Conclusion:** Thus, is possible to affirm the exposure of the Aspergillus fumigatus to drugs used on agriculture may induce resistance against azoles usually prescribed. These finds demonstrating the need of a better and more rigid regulation in the use of this molecule in agriculture. The next steps in this research are to confirm that placobutazol exposure may induce a permanent mutation on resistance gene.

### P002. Oral Candidiasis in HIV Infected Patients and its Antifungal Susceptiblity Pattern by Disc Diffusion Method.

ChaudharyP.Microbiology, Sagarmatha chaudhary eye hospital, lahan, siraha, Nepal

**Objectives:** Oropharyngeal candidiasis (OPC) is an opportunistic fungal infection that is commonly found in (Human Immunodeficiency Virus) HIV infected patients. Acquired Immunodeficiency syndrome (AIDS), a disease of human immune system caused by HIV, has emerged as a global crisis since its discovery in summer of 1981 in United States. The low absolute CD4+ T-lymphocyte count has traditionally been cited as the greatest risk factor for the development of OPC and current guidelines suggest increased risk once CD4+T-lymphocyte counts fall below 200 cells/mm³.Hence, a cross-sectional study was conducted in Sukraraj Tropical and Infectious Disease Hospital (STIDH), Teku, Kathmandu over 3 months period with a primary objective, to isolate and identify *Candida* species among HIV infected patients and susceptibility pattern of isolates by disc diffusion method and secondary objective, to estimate the frequency of oral candidiasis according to age, sex, and other factors (CD4+ count, tobacco consumption, URTI, recent antibiotic consumption) in HIV infected patients.

**Methods:** A total of 408 throat swab samples were collected from patients with and without active lesion visiting ART center of STIDH. Flow chart for sample processing and identification of isolates is shown below.

**Results:** 114 isolates were obtained from total sample processed. Among them, 65 isolates were *Candida* species and remaining 49 was bacterial flora and other yeasts. Based on various phenotypic methods, *Candida albicans*, 53 (81.5%), was the most commonly isolated organism followed by *Candida tropicalis* 3 (4.6%), *Candida krusei* 2 (3.1%), *Candida glabrata* 1 (1.5%) and 6 (9.2%) were other *Candida* species. of the 65 yeast isolates from HIV seropositive individuals, 26 patients had CD4+ ≤ 200 cells/mm³ and 39 patients had CD4+ ˃200 cells/mm³. 14 patients exhibited severe immune suppression with CD4+ count less than 100 cells/mm³. There was a significant association between oral *Candida* carriage and CD4+ cell count ≤ 200 cells/mm³ (p<0.001). Antifungal susceptibility test of *Candida* species showed highest sensitivity with Amphotericin B and resistance with Fluconazole respectively. Table below depicts about the susceptibility pattern of the *Candida* spp.

**Conclusion:** *C. albicans* was the predominant species for oral yeast colonization in our study. Different isolates presented different susceptibility to various antifungal agents. Among the antifungal discs tested, Fluconazole was found to show resistance in most of the isolates and Amphotericin B was the most sensitive drug which is an excellent antifungal to control *Candida* spp. since it showed the best result among all antifungal tested. Oral yeast colonization was associated with low CD4+ count (<200 cells/mm³). Thus, oral lesion serves as early marker for HIV infection. The increase incidence of oral candidiasis in HIV patients may be due to low CD4+ count, URTI and recent antibiotic consumption.

### P003. The validation of VIPcheck™ plates to screen Aspergillus fumigatus isolates for phenotypic resistance to triazole antifungal agents in St. James’s Hospital, Dublin.

GriffinA.Microbiology, St. James’s Hospital, Dublin, Dublin, Ireland

**Objectives**: Triazole resistance is an emerging problem in Aspergillus fumigatus (AF) resulting in failure of azole therapy. Triazole resistant AF is acquired through one of two routes - previous exposure to triazole therapy or an environmental source. In vitro antifungal susceptibility testing (AFST) on all AF strains isolated in a microbiology laboratory would be both labour intensive and impractical. A method to screen for triazole resistance would be more favourable. The objective was to validate VIPcheck™ plates (Mediaproducts BV, Groningen, Netherlands) in SJH to investigate whether they would be a suitable screening method for triazole resistance in AF clinically isolated from patients in a large tertiary hospital with a significant cohort of immunocompromised patients.

**Methods**: A total of 18 retrospective isolates (clinical and environmental) of AF were tested using the VIPcheck™ plates (n=2 wild type, n=16 resistant to ≥ 1 triazole drug as previously determined by AFST and/or molecular methods). VIPcheck™ plates are a simple agar based screening method, each 4 well plate containing a growth control (GC) well and 3 wells containing itraconazole (4mg/L), voriconazole (2mg/L) and posaconazole (0.5 mg/L). Briefly, 25ul of a 0.5-2McF suspension AF is inoculated into each well and plates are read after 48hrs incubation at 37 °C. Any growth in a triazole containing well is suggestive of resistance prompting full AFST be carried out.

**Results**: A total of 18 isolates (clinical and environmental) of AF were tested using the VIPcheck™ plates as described above. The wild type isolates showed growth only in the GC well while the resistant strains all showed growth in one or more of the triazole containing wells.

**Conclusion**: VIPcheck™ plates are not intended to give an exact MIC; the preparation of the inoculum has a broad McFarland range of 0.5-2 and only one concentration of each azole drug is included in the plates. Rather, they are useful as a screening method to determine the need for further AFST and/or molecular testing and more importantly for earlier detection of triazole resistance in patients who will require treatment. Currently in SJH, AFST is carried out using gradient strips (Liofilchem™) and results are interpreted using EUCAST breakpoints We validated the VIPcheck™ plates with the intention to include this screening method as part of our AFST for AF isolated from clinical samples and our results suggest that they provide a reliable screening method for triazole resistance.

### P004. In vitro susceptibility to isavuconazole of Fusarium clinical isolates; comparison between eucast and etest methods

BroutinA.^1^BigotJ.^1^NormandA.C.^2^SenghorY.^3^MorenoA.^3^HendrickxM.^4^GuitardJ.^1^HennequinC.^3^1Parasitology-mycology, APHP, hôpital St Antoine, Paris, France2Parasitology-mycology, APHP, Hôpital Pitie-Salpêtrière, Paris, France3Parasitology-mycology, APHP, Hôpital St Antoine, Paris, France4Mycology, Scientific Institute of Public Health, Brussels, Belgium

Objectives: To evaluate the in vitro susceptibility to isavuconazole of a panel of precisely identified clinical *Fusarium* isolates and to assess the value of Etest as an alternative method in this indication.

Methods: We randomly selected 75 clinical isolates previously identified as *Fusarium sp.* The strains were identified using MALDI-TOF technology (MSI System). They were then tested comparatively against isavuconazole (ISA) either using the broth microdilution method proposed by the Eucast consortium according to the recent document E.DEF 9.1 and the Etest technique as recommended by the manufacturer (Liofilchem).

Results: All strains could be identified at the cryptic species-level revealing the existence of 14 different species in the panel (Table 1). *F. solani* (FSSC), *F. oxypsorum* (FOSC) and *F. fujikuroi* (FFSC) were the species complexes the most frequent, encompassing 22, 17 and 31 strains, respectively. Overall, ISA exhibited a limited activity against the *Fusarium* strains with MICs90 >16 µg/ml whatever was the species complex. Strains of the FOSC appeared a little bit more susceptible with a geometric mean at 9.41 µg/ml versus 14.02 µg/ml and 13.68 µg/ml for FSSC and FFSC, respectively. Considering the diversity of species, it was not possible to test a possible difference in susceptibility between the cryptic species within a given complex. Finally, we observed MIC between 2 to >16 µg/ml for strains of the single species of the FOSC, suggesting that precise identification may not be enough to predict the susceptibility to ISA. The concordance between Etest and Eucast results was calculated at 100% within an interval +/- 2 dilutions. The Intraclass Correlation Coefficient was calculated at 0.91, indicative of a strong concordance between the 2 methods.

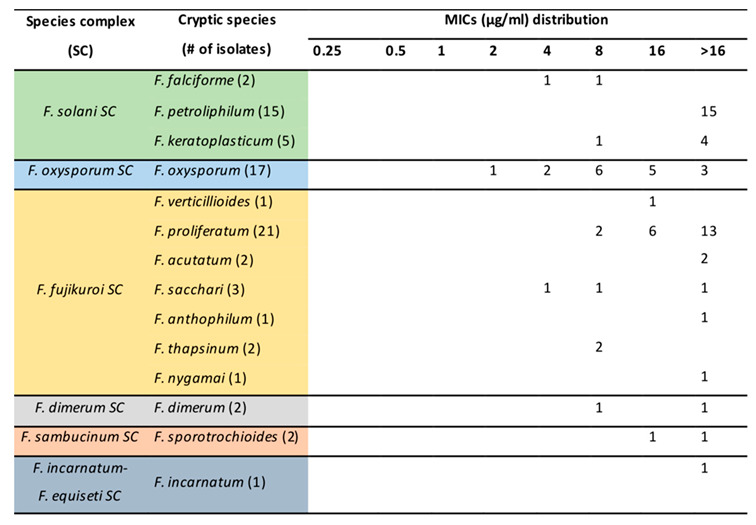



Table 1: Isavuconazole MICs distribution against 75 clinical isolates of *Fusarium*.

**Conclusion:** ISA exhibits a limited *in vitro* activity against clinical isolates of *Fusarium*, with few variations between the species complexes. In the case the drug should be used, Etest appears a valuable alternative to Eucast for evaluating the *in vitro* susceptibility of the infecting strain.

### P005. Species Distribution and Antifungal Susceptibility of Candida Ear Isolates from a Hospital in Korea

ShinJ.H.KwonY.J.ByunS.A.ChoiM.J.WonE.J.KimS.H.Department of Laboratory Medicine, Chonnam National University Medical School, Gwangju, Republic of Korea

**Objectives:** *Candida auris* was first isolated from the ears of Japanese and Korean patients. However, the prevalence of *Candida* species from ear cultures and their antifungal susceptibility profiles in these nations are unclear.

**Methods:** We assessed *Candida* isolates recovered from cultures of ear specimens from a university hospital in Korea over the 4-year period from January 2014 to December 2017. Species identification was performed by matrix-assisted laser desorption/ionization time of flight mass spectrometry and/or sequence analysis. Antifungal minimal inhibitory concentrations (MICs) were determined by the broth microdilution method (M27A) of the Clinical and Laboratory Standards Institute.

**Results:** Candida species were isolated from the ears of 80 patients (75 adult and 5 pediatric patients). Most of these patients suffered from chronic otitis media (83.8%), and had a history of antibiotic use (73.8%) and ear surgery (60.0%). Among 80 non-duplicate isolates from ear cultures, *Candida parapsilosis* was the most frequently detected *Candida* species (35.0%), followed by *C. auris* (28.8%), *Candida metapsilosis* (10.0%), *Candida orthopsilosis* (8.8%), *Candida albicans* (7.5%), and other species (11.1%). The MICs of the isolates were 0.125 to > 64 µg/mL, ≤ 0.03 to 4 µg/mL, 0.25 to 1 µg/mL, 0.125 to 1 µg/mL, and ≤ 0.03 to 2 µg/mL for fluconazole, voriconazole, amphotericin B, caspofungin, and micafungin, respectively. of the 80 isolates, 43.8% (35/80) showed decreased susceptibility to fluconazole (MIC ≥ 4 µg/mL). of 23 *C. auris* isolates, 19 (82.6%) had a fluconazole MIC of ≥ 32 μg/mL. None of the isolates showed resistance to amphotericin B or echinocandins.

**Conclusion:** *C. parapsilosis* complex and *C. auris* were the *Candida* species identified most frequently in ear cultures and exhibited a high rate of fluconazole non-susceptibility, particularly *C. auris.*

### P006. Epidemiological pattern of Malassezia, its phenotypic identification and anti-fungal susceptibility profile to azoles by broth micro-dilution method.

RomaldN.^1^KindoA.J.^2^VeeraragavanM.^3^1Department of Microbiology, Sri Ramachandra Institute of Higher Education and Research, Chennai, India2Microbiology, Sri Ramachandra Medical College and Research Institute, SRIHER, Chennai, India3Dermatology, Sri Ramachandra Medical College and Research Institute, Chennai, India

**Objectives:** Analyse the epidemiological pattern of disease with regards to age, sex and site of predilection To Speciate *Malassezia* phenotypically. To study the anti-fungal susceptibility (AFST) pattern of Azoles to pathogenic *Malassezia* species by recording their MIC values (MIC_50_ & MIC_90_) using Broth Micro dilution method in accordance with CLSI M27-A3. To identify resistant drug/drugs to *Malassezia* species.

**Methods: Prospective study** was done from **August 2016-October2018** with sample size of **150** *Sample collection-*Hyper/hypo pigmented skin lesions by Skin scrapings and swabs *Microscopic examination with 10%KOH* Those showing meat ball and spaghetti appearances were included in the study. *Culture* Samples inoculated into Sabourauds Dextrose Agar (SDA), SDA with olive oil overlay (SDA-O) and Modified Dixon’s agar (MDA) containing chloramphenicol and incubated at 32^o^ C upto 3 weeks. *Phenotypic Identification* Done by colony growth characteristics, gram staining, urease test, catalase test, bile esculin with tween 60 hydrolysis and tween assimilation *AFST* AFST was performed by the broth micro-dilution method, according to the Clinical and Laboratory Standard Institute (CLSI) guidelines M27-A3 (2008) ***Systemic anti-fungals used-Fluconazole, Itraconazole, VoriconazoleTopical Anti-fungals used-Clotrimazole, Miconazole, Luliconazole*** Since *Malassezia* are lipid dependant yeast RPMI 1640 was supplemented with glucose, peptone, ox bile, malt extract, glycerol, tween 80, tween 40 and Chloramphenicol *Culture-Malassezia* isolates grown on MDA for 72 h at 32°C± 2°C were used for inoculum preparation. Anti-fungal stock solutions of drugs were prepared and stored at - 70^0^C *Quality Control-*Each microtitre plate included growth control well with inoculum and supplemented RPMI -1640 medium without the antifungal agent. *Candida krusei* ATCC 6258 was used as a quality control strain for antifungal susceptibility testing. *Incubation and Reading Results-*The microtitre plates were incubated at 32˚C.Results read after 24hrs for *candida* isolate and after 72-96 hours for *Malassezia* isolates. The minimum inhibitory concentration (MIC_90_) (reduction of 90% growth compared to growth control well) and MIC_50_ (50% reduction of growth compared to growth control well) were recorded.

**Results:**

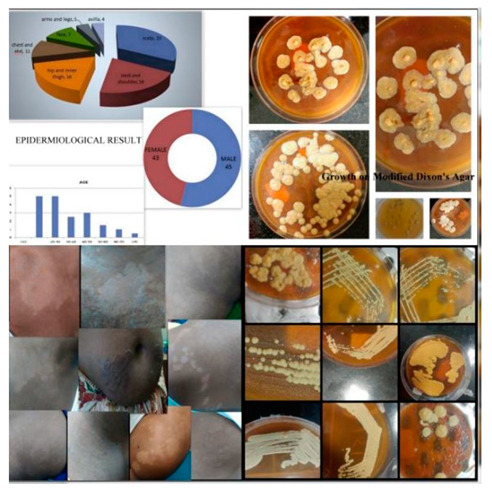

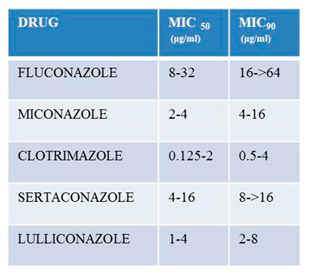





*Age distribution*- The most common age affected was 20 to 30yrs (36%), followed by 30-40yrs (21%) and 10-20yrs (19%) extremes of age showed lower incidence. *Sex distribution-*The incidence among male and female was almost equal, showing little female predominance *Site of infection* - Among the total 150, the most common site was back (47 samples), followed by neck and shoulder (31 samples), and scalp (27 samples). *Skin lesions*-Both hypo and hyperpigmented skin scrapings were collected. But more of hypopigmented lesions were noted in our study **ANTI-FUNGAL SUSCEPTIBILITY PATTERN-**fig

**Conclusion:** Among 150 samples only 95(62%) yielded growth on both SDA with olive oil and MDA. Growth on MDA was soon than SDA with olive oil Overlay. All organisms were urease and catalase positive. Among the 95 samples grown the predominant species was *Malassezia sympodialis*-29(33%) followed by *M.globosa*-26(29.5%), *M.furfur*-21(23%), *M.obtusa*-12(13%) by conventional method. The systemic anti-fungal with the least MIC values were Itraconazole and Voriconazole. Fluconazole shows resistant Pattern with high MIC values The most effective topical anti-fungal was Clotrimazole with low MIC values *Malassezia sympodialis* responded to anti-fungal better than other species isolated

### P007. Prevalence and genotype distribution of fluconazole-resistant Candida parapsilosis bloodstream isolates from a neonatal intensive care unit during a 14-year period

ShinJ.H.^1^HwangI.J.^2^WonE.J.^1^ChoiM.J.^1^JungS.-I.^3^SongE.S.^2^ChoiY.Y.^2^1Department of Laboratory Medicine, Chonnam National University Medical School, Gwangju, Republic of Korea2Paediatrics, Chonnam National University Medical School, Gwangju, Republic of Korea3Chonnam National University Medical School, Gwangju, Republic of Korea

**Objectives:** *Candida parapsilosis* bloodstream infection (BSI) is a significant problem in neonatal intensive care units (NICUs). *C. parapsilosis* is usually susceptible to azole antifungals; however, some recent reports have indicated the emergence of BSIs caused by fluconazole-resistant (FR) *C. parapsilosis* isolates. We investigated the prevalence and genotype distribution of FR *C*. *parapsilosis* BSI isolates recovered from a NICU during a 14-year period.

**Methods:** We assessed a total of 64 *C*. *parapsilosis* BSI isolates comprising 22 non-duplicate isolates from 22 neonates and 42 isolates from 21 neonates (two isolates from each neonate obtained on the first and the last days of *C*. *parapsilosis*-positive culture). All isolates were obtained at the same NICU between 2002 and 2015. *In vitro* antifungal susceptibility testing for fluconazole, amphotericin B, caspofungin, and micafungin was performed using the Clinical and Laboratory Standards Institute (CLSI) M27-A3 microdilution method. All isolates were genotyped via microsatellite typing using four specific microsatellite markers (CP1, CP4, CP6, and B). Clonal strains were defined as the isolation of two or more strains with identical genotype as determined by microsatellite typing.

**Results:** of the 64 *C*. *parapsilosis* BSI isolates, 11 (17%) obtained from eight patients were FR (fluconazole minimal inhibitory concentration [MIC] ≥ 8 mg/L). All 64 isolates were susceptible to amphotericin B, caspofungin, and micafungin. Based on microsatellite typing, the 64 isolates yielded 19 different genotypes, including 12 genotypes unique to a single isolate and seven genotypes shared by 49 isolates (77%). All 11 FR isolates shared two genotypes (1 and II) that displayed closer genetic relatedness than other isolates examined in the microsatellite analysis, according to as an unweighted pair group method with arithmetic mean (UPGMA) tree. of these two genotypes, type I strains (three patient isolates) were isolated within a 6-month period, whereas type II strains (five patient isolates) were isolated over a 4-year period. Among 21 patients showing more than one *C*. *parapsilosis* isolate on 2 or more separate days, two sequential strains from each patient had identical types in 76% (16/21) of patients and different types in 24% (5/21). No difference in antifungal susceptibility was detected between two sequential isolates from the same patient. Among eight patients with FR *C*. *parapsilosis* isolates, 88% had no known prior antifungal exposure. We detected no significant difference in the overall 30-day mortality of *C*. *parapsilosis* BSI due to FR strains, or in that of BSI due to fluconazole-susceptible (FS) strains (25 vs. 20%).

**Conclusion:** The results of the present study suggest that clonal transmission may occur frequently among both FS and FR blood isolates of *C. parapsilosis* in a NICU setting. Increased incidence of BSI due to the FR *C. parapsilosis* strain in this NICU may be associated with clonal transmission rather than prior antifungal exposure.

### P008. Bloodstream Isolates of Azole-Resistant Candida glabrata in Korean hospitals: Resistance Mechanisms and Clinical Features

ShinJ.H.^1^WonE.J.^1^JungS.-I.^2^HwangI.J.^3^ChoiM.J.^1^ByunS.A.^2^KimS.H.^1^ParkY.-J.^4^KimM.-N.^5^1Department of Laboratory Medicine, Chonnam National University Medical School, Gwangju, Republic of Korea2Chonnam National University Medical School, Gwangju, Republic of Korea3Paediatrics, Chonnam National University Medical School, Gwangju, Republic of Korea4The Catholic University of Korea College of Medicine, Seoul, Republic of Korea5University of Ulsan College of Medicine and Asan Medical Center, Seoul, Republic of Korea

**Objectives:** *Candida glabrata* has recently become the most common non-*albicans Candida* species causing bloodstream infection (BSI) in South Korea, and the occurrence of azole-resistant *C. glabrata* isolates has been observed. We investigated the azole resistance mechanisms and clinical features of patients with azole-resistant *C. glabrata* BSI isolates recovered from multicenter surveillance in Korea.

**Methods:** In total, 248 *C*. *glabrata* BSI isolates comprising 43 azole-resistant (fluconazole minimum inhibitory concentration [MIC], ≥ 64 μg/mL and voriconazole MIC, ≥ 1 μg/mL) and 205 non-azole-resistant (non-AR) isolates (fluconazole MIC, ≤32 μg/mL and voriconazole MIC, ≤ 0.5 μg/mL) were analyzed. The isolates were collected from 10 university hospitals over 8 years (2009–2017). The *PDR1* genes of the isolates were sequenced to identify mutations. Expression levels of *CDR1, CDR2,* and *ERG11* genes after fluconazole exposure were quantified by real-time PCR. The clinical features related to risk factors for BSI and the mortality rates were compared. Risk factors for BSI caused by AR isolates and survival rates in patients were assessed using multivariate logistic regression.

**Results:** Pdr1 mutations were identified in 42 of 43 azole-resistant isolates, while no Pdr1 mutation was found in 201 of 205 non-AR isolates. Azole-resistant isolates of *C. glabrata* had higher mean expression levels of *CDR1* (8.3-fold) gene than non-AR isolates (*P* <0.05). However, there were no significant differences in the mean expression levels of *CDR2* and *ERG11* between two groups. Previous azole exposure (odds ratio [OR], 6.369; 95% confidence interval [CI], 2.434-16.664; *P* < 0.001), central venous catheter use (OR, 11.406; 95% CI, 2.462-52.837; *P* = 0.00211), and total parenteral nutrition (OR, 4.042 95% CI, 1.381-11.828; *P* = 0.011) were the independent risk factors for BSI caused by AR *C. glabrata*. *C. glabrata* BSIs caused by AR isolates resulted in a higher 30-day mortality rate (55.8%, 24/43) than BSIs caused by non-AR strains (36.6%, 75/205) (*P* = 0.019). In multivariate analyses, severe sepsis (OR, 5.592; 95% CI, 2.689-11.630; *P* < 0.001), a high age-adjusted Charlson comorbidity index (ACCI) score (OR, 1.131; 95% CI, 1.012-1.263; *P* = 0.03), lack of antifungal therapy (OR, 5.213; 95% CI, 2.487-10.930; *P* < 0.001), and azole-resistance in *C*. *glabrata* BSI isolates (OR, 2.856; 95% CI, 1.161-7.025; *P* = 0.022) were independent predictors of the 30-day mortality among the 248 patients.

**Conclusion:** This study provides that Pdr1 mutation with overexpression of *CDR1* may be the main resistance mechanism of *C. glabrata* in Korea, and that azole-resistance in *C*. *glabrata* BSI isolates is independent predictor of 30-day mortality, and is associated with antifungal exposure, central venous catheter, and total parenteral nutrition use.

### P009. A case of urinary tract candidosis: a risky business

BartonR.SethiK.Microbiology, Leeds Teaching Hospitals Trust, Leeds, United Kingdom

**Case Report:** A 62 year old man with a history of psoriasis treated with methotrexate, poorly controlled type 2 diabetes, and chronic obstructive pulmonary disease had a laparoscopic cholecystectomy for cholecystitis and repair of a cholecystoduodenal fistula. He then presented with severe dysuria, polyuria and complained of passing cloudy urine ever since the surgery. Following a series of urine samples which were positive for yeasts which were not identified and a course of fluconazole (details not available), he was seen at a haematuria clinic with continued dysuria, frequency and urgency, and a flexible cystoscopy showed a "snowstorm" appearance within bladder, and an ultrasound revealed mobile echogenic debris. He was then put on a 14/7 course of 200mg oral fluconazole, pending a cystoscopy and bladder biopsy which revealed erosive cystitis. A subsequent urine sample was identified as *Candida glabrata* which was resistant to fluconazole (MIC >256mg/L) but sensitive to flucytosine (MIC 0.06mg/L). Despite a further 14 day course of 800mg fluconazole, signs and symptoms of *Candida* cystitis were not resolved. The patient was then referred to a urologist at our hospital and various treatment choices were discussed with oral flucytosine and IV Amphotericin deoxycholate being the main two options, associated with the risk of resistance and toxicity respectively. It was decided to treat with a 7 day course of 25mg/kg of oral flucytosine (levels were not monitored due to the short course) but follow up urine samples 9 days after completing the flucytosine course were still positive for *C. glabrata* resistant to both fluconazole (MIC >256mg/L) and flucytosine (MIC >64mg/L). Approximately 2 weeks after completing the course of flucytosine, the patient was admitted via Accident and Emergency with suspected sepsis relating to a biliary source, and a new ultrasound showed that the left kidney was moderately hydronephrotic, the bladder was filled with echogenic debris. A nephrostomy was performed and blood cultures taken which grew *C. glabrata* with identical sensitivities to that from the most recent urine sample. The patient was prescribed a 2 week course of Anidulafungin (200mg loading dose then 100mg 24-hourly IV) to combat the systemic candidosis, and a 7 day course of intravenous Amphotericin deoxycholate at 0.6mg/kg/day. During the Amphotericin B deoxycholarte treatment, renal function was not markedly affected, though increases in alkaline phosphatase levels led to the treatment being stopped at 6 days. Subsequent blood and urine cultures remained negative, CRP declined from 150 to 5.8 mg/L and symptoms resolved. This case is one of the examples of how the significance of the presence of yeasts in urine of patients is not clearly understood by the clinicians. Our patient presented here highlights the increasing prevalence of *C.glabrata* in funguria and non-*albicans* candidaemia in at risk settings, the challenges in its management, the risks and drug related factors which need to be assessed when treating fluconazole resistant urinary tract candidosis.

### P010. Effects of efflux pump modulators on the azole antifungal susceptibility of Microsporum canis

AnekeC.RhimiW.OtrantoD.CafarchiaC.Veterinary Medicine, University of Bari, Aldo Moro, Bari, Italy

**Objectives:** This study investigated the effect of efflux pump modulators, EPMs (i.e. haloperidol-HAL and promethazine-PTZ) and their inhibiting activity on the minimum inhibitory concentrations of itraconazole (ITZ) and fluconazole (FLZ), against selected *M. canis* strains.

**Methods:** *M. canis* strains with low (≤ 1mg/ml itraconazole and <64mg/ml fluconazole) and high (>1mg/ml itraconazole and ≥64 μg/ml fluconazole) azole MIC values were tested, and data analyzed using the model-fractional inhibitory concentration index (FICI) method.

**Results:** The MIC values of ITZ and FLZ of *M. canis* decreased in the presence of sub-inhibitory concentrations of HAL and PTZ, the latter being more effective with a greater increased susceptibility. Synergism was observed in all strains with high azole MICs (FICI<0.5) and no synergism in the strains with low azole MICs.

**Conclusion:** These results suggest that the drug efflux pumps are involved in the defense mechanisms to azole drugs in *M. canis* strains. The synergism might be related to an increased expression of efflux pump genes, eventually resulting in azole resistance phenomena. Additionally, the most effective double combinations were those of PTZ with either FLZ or ITZ as this had a greater increase in azole susceptibility. Complementary studies on *M. canis* resistance are advocated in order to investigate the molecular mechanisms of this phenomenon.

### P011. Antifungal resistance pattern of Candida species isolated from women of child-bearing age in Nsukka Nigeria suffering from different clinical conditions.

MgbeahuruikeA.Department of Veterinary Pathology and Microbiology, Faculty of Veterinary Medicine, University of Nigeria, Nsukka, Nsukka, Enugu, Nigeria

**Objectives:** To isolate and identify Candida species in child bearing women suffering from malaria, typhoid and diabetes To investigate the resistance partarn of the identified Candida species using conventional azoles and plant extracts To trace the evolutionary relatedness of the isolated fungi using phylogenetic analysis

**Methods:** High vaginal swabs were analyzed for *Candida* presence in women of child bearing age suffering from malaria, typhoid and diabetes using Chromogenic agar. Evolutionary relatedness of the *Candida* species was inferred using phylogenetic reconstruction. Growth rate assay was done using Sabourand Dextrose medium supplemented with NaCl and glucose. The resistance pattern of the *Candida* species was tested using fluconazole and extracts (methanol and ethyl) from *Byrsocarpus coccineus.*

**Results:** Out of the 79 fungal samples analyzed, 21 (26.6%) were *C. tropicalis*, 25 (31.6%) were *C. krusei*, 10 (12.7%) were *C. parapsilosis* and 8 (10.1%) were *C. albicans. C*. *tropicalis* was highest in the malaria patients, *C. albicans* dominated in the typhoid patients while *C. krusei* was more in the diabetic patients. The healthy women presented a very low percentage (8.9%) of *Candida* species. Phylogenetically, the sequenced *Candida* species was more closely related to *C. tropicalis*. High concentration of glucose (1g/ml) and NaCl (0.5M) decreased the growth of the fungus. From the study, fluconazole showed a higher activity than the two extracts (methanol and ethyl acetate).

**Conclusion:** In conclusion, although fluconazole showed a higher activity than the two extracts (methanol and ethyl acetate), the plant extracts demonstrated very high activity and can be used as potential antifungal agents. *C. tropicalis* and *C. krusei* were the most abundant *Candida* species found in this study.

### P012. Colistin and isavuconazole interact synergistically in vitro against Aspergillus nidulans and Aspergillus niger

SchwarzP.^1^,^2^DannaouiE.^3^,^4^1Department of Internal Medicine - Respiratory and Critical Care Medicine, University Hospital Marburg, Marburg, Germany2Center For Invasive Mycoses and Antifungals, Philipps University Marburg, Marburg, Germany3Hôpital Européen Georges Pompidou, Laboratoire de Parasitologie-Mycologie, Paris, France4Working Group Dynamyc, Faculté de Médecine, Hôpital Henri Mondor, Créteil, France

**Objectives:** Invasive aspergillosis is a devastating disease in immunocompromised patients. It mostly affects patients with hematological malignancies, especially those with severe and prolonged neutropenia. Colistin is a polypeptide antibiotic from the class of polymyxins. Polymyxins bind to lipopolysaccharides and phospholipids of the outer cell membrane of gram-negative bacteria. Binding leads to competitive displacement of divalent cations from the phosphate groups of the membrane lipids, leading to bactericidal action by disruption of the outer cell membrane and leakage of intracellular contents. Colistin has also shown *in vitro* activity against some fungi. The aim of the present study was to assess the *in vitro* interaction of the broad-spectrum azole isavuconazole with colistin against the most important *Aspergillus* species responsible for human disease.

**Methods:** A panel of 31 Aspergillus isolates belonging to 5 species responsible for human invasive aspergillosis was used for the experiments (10 *Aspergillus fumigatus*, 5 *Aspergillus flavus*, 5 *Aspergillus terreus*, 6 *Aspergillus nidulans*, and 5 *Aspergillus niger*). Combinations of isavuconazole with colistin were tested by a broth microdilution checkerboard technique based on the EUCAST reference methodology. Plates were read spectrophotometrically, and ninety percent of inhibition was used as an endpoint for both, the drugs alone and in combination. Results were interpreted with the fractional inhibitory concentration index (FICI). Drug interactions were defined as synergistic (FICI≤0.5), indifferent (FICI>0.5≤4) or antagonistic (FICI>4).

**Results:** Isavuconazole MICs ranges from 0.5 to 16 µg/ml. When tested alone, colistin exhibited no antifungal activity with MICs of 128 µg/ml for all isolates. Combination of isavuconazole with colistin was synergistic for 100 and 60% of the *A. nidulans*, and *A. niger* isolates, respectively (Table). All other interactions were indifferent. Antagonism was never seen.

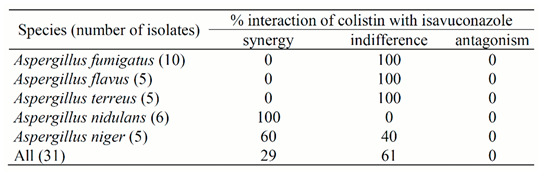



**Conclusion:** Combination of colistin with isavuconazole showed strong synergy against all tested *A. nidulans* isolates and synergy for the majority of the tested *A.niger* isolates. *In vitro* interaction of colistin and isavuconazole will be further evaluated by checkerboard and gradient concentration strips against a larger panel of *A. nidulans* and *A.niger* isolates.

### P013. Update 2016-2018 of the nationwide Danish fungaemia surveillance study: epidemiologic changes in a 15-year perspective

RisumM.^1^DatcuR.^1^JohansenH.K.^2^SchønheyderH.C.^3^RosenvingeF.S.^4^KnudsenI.J.D.^5^RøderB.L.^6^AntsupovaV.S.^7^GertsenJ.B.^8^KristensenL.^8^MøllerJ.K.^9^HareR.^1^AstvadK.M.T.^1^JørgensenK.M.^1^DzajicE.^10^ArendrupM.C.^1^,^2^,^11^1Mycology Unit, Statens Serum Institute, Copenhagen, Denmark2Department of Clinical Microbiology, Rigshospitalet, Copenhagen, Denmark3Department of Clinical Microbiology, Aalborg University Hospital, Aalborg, Denmark4Department of Clinical Microbiology, Odense University Hospital, Odense, Denmark5Department of Clinical Microbiology, Hvidovre Hospital, Hvidovre, Denmark6Department of Clinical Microbiology, Slagelse Sygehus, Slagelse, Denmark7Department of Clinical Microbiology, Herlev Hospital, Herlev, Denmark8Department of Clinical Microbiology, Aarhus University Hospital, Aarhus, Denmark9Department of Clinical Microbiology, Vejle Sygehus, Vejle, Denmark10Department of Clinical Microbiology, Esbjerg Sygehus, Esbjerg, Denmark11University of Copenhagen, Dept. of Clinical Medicine, Copenhagen, Denmark

**Objectives:** The objectives of this study was to provide nationwide contemporary data on the epidemiology of candidaemia, often a serious complication of hospital stay with a crude 30-day mortality rate of about 40% despite recent advantages in diagnosis and management. The motivation was that outcome depend on early appropriate therapy necessitating knowledge on susceptibility pattern of involved species. Moreover, epidemiological changes reflect current practises in prophylactic, empirical and pre-emptive use of antifungals and therefore are also informative regarding the need for adjustment of antifungal stewardship programmes or treatment guidelines.

**Methods:** As part of an active national surveillance programme, fungal isolates from blood culture were referred to Statens Serum Institut (Copenhagen) for confirmatory species identification and EUCAST E.Def 7.3.1 susceptibility testing. A total of 1452 unique isolates (same patient and species isolates were regarded unique if more than 21 days apart or with different susceptibility) were included. 13 isolates (0.9%) were not referred. When MICs for echinocandins were above the clinical breakpoints, *FKS* sequencing of the isolates was performed. An isolate was deemed echinocandin resistant if the MIC was above anidulafungin breakpoint and/or if the isolate had a known *FKS* hotspot*-*mutation. Results were compared to previous data in a 15-year perspective.

**Results:** The mean incidence rate during 2016-2018 was 8.13/100.000 inhabitants. Candidaemia was more frequent in males than females (60% vs 40%) as in accordance with previous years. In comparison to previous data, the fungaemia rate was stable (8.64, 9.03 and 8.38 per 100.000 inhabitants in 2004-7, 2008-11 and 2012-15 respectively). In the years 2016-2018 *C. albicans* accounted for 42.1% of all unique isolates with a decline in 2017 to below 40%. *C. glabrata* accounted for 32.1%. The proportion of candidaemia in 2018 involving *C. albicans* decreased by a third from 64% in 2004 (P<0.0001), and *C. glabrata* almost doubled from 17% in 2004 (P<0.0001) (Figure). We found a fluconazole susceptibility rate across all *Candida* species of 59% of isolates which has decreased since 2004-7 from 69% (P = 0.02). Echinocandin resistance was detected in 18/1294 isolates (1.4%) including 11/459 (2.4%) in *C. glabrata* specifically. No echinocandin resistance was detected in 2004-7. *FKS*-mutations were identified in the following species *C. glabrata:* two L630Q/S663F, four F659S, one L662W, three S663F and one S663P; *C. krusei:* four L701M, two S659F and one S659S/F; *C. albicans* one P1354P/S; *C. dubliniensis* one S645P; and for *Candida lusitaniae* one H657Y/L1243F/I283V. H657Y/L1243F/I283V, P1354P/S and L701M are outside the hot spot, and L701M is not associated as a molecular cause of echinocandin resistance. 

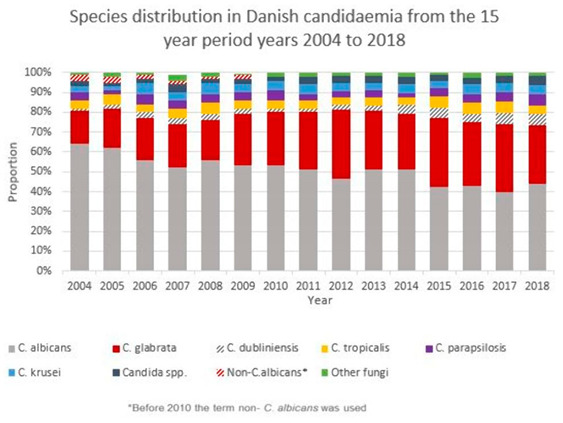



**Conclusion:** We report a stable incidence rate of candidaemia, but the proportion of *C. glabrata* continues to rise at the expense of *C. albicans* which accounted for less than 40% in 2017. The consequent decrease in fluconazole susceptibility along with the presence of *FKS*-mutations particularly in *C. glabrata* raises concern for future perspectives in the treatment of candidaemia.

### P014. In vitro potpourri: Aspergillus terreus and the activity of amphotericin B

ShahandashtiR. VahediDivision of Hygiene and Medical Microbiology, Medical University of Innsbruck, Innsbruck, Austria

**Objectives:** Invasive aspergillosis caused by intrinsically resistant non-*fumigatus Aspergillus* species displays a poor outcome in immunocompromised patients. *Aspergillus terreus* holds an exceptional position within the *aspergilli* due to its intrinsic resistance against the polyene amphotericin B (AmB). Till now, the exact mode of action in polyene resistance is not well understood. Micro-environmental factors at the infection site influence the growth of fungal pathogens and most likely also the efficacy of antifungal drugs. The purpose of this study was to evaluate the impact of hypoxia versus normoxia on the *in vitro* activity (fungistatic and fungicidal) of AmB against *A. terreus*.

**Methods:** In total, 100 clinical strains of *Aspergillus* section *terrei*, collected from 21 various countries were analyzed in detail. Antifungal susceptibility testing was carried out according to EUCAST guideline, with normoxic (20% oxygen/5% carbon dioxide/75% nitrogen) and hypoxic (1% oxygen/5% carbon dioxide/94% nitrogen) conditions. Final drug concentrations used for AMB were 0.03–16 μg/ml. Minimum inhibitory concentration (MIC) was defined as the lowest drug concentration showing no fungal growth and minimum fungicidal concentrations (MFCs) was taken as the first well with ≥90% killing of fungi. MFC was determined by agar and broth-based (flash microbicide method) methods. MICs (μg/ml) and colony forming units were determined after 48h at 37°C. Testing was performed in duplicates.

**Results:** AmB MICs of *A. terreus section terrei* ranged from 0.125-2 µg/ml for hypoxia, and from 0.5-8 µg/ml for normoxia. These data indicate that some strains were more susceptible to AmB in hypoxic conditions compared to normoxia (Figure 1). The majority of strains showed that AmB had no fungicidal effect against tested *A. terreus* under any methods and atmospheric conditions (Figure 2). *A. terreus* representing lower MICs (*n* = 41, ≤0.5 µg/ml) did not result in modest MFCs (≥90% killing in 1-2 µg/ml).

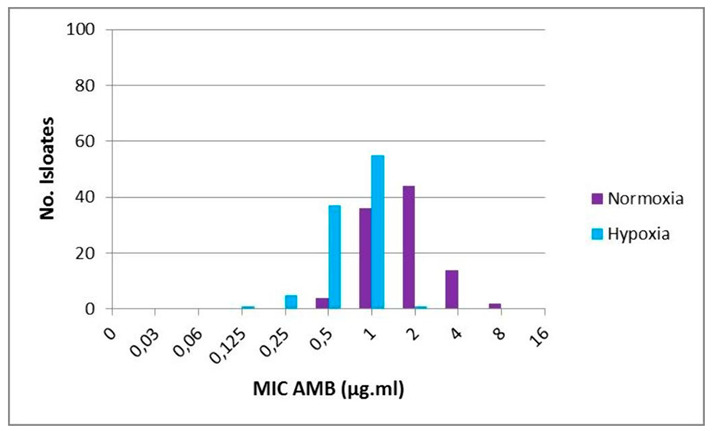



Figure 1. *In vitro* susceptibility to AMB of 100 *A. terreus* isolates in normoxia versus hypoxia, measured by EUCAST.

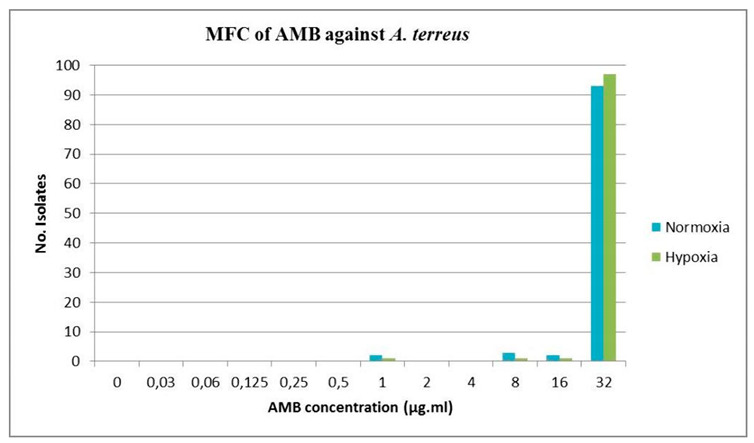



Figure 2. The effect of oxygen on MFC distributions of AMB, agar-based method measured by colony forming units.

**Conclusion:** Simulating the host environment in *in vitro* susceptibility testing will contribute to a better understanding of how these conditions influence antifungal activity. Hypoxic conditions had a marginal influence on the *in vitro* antifungal susceptibility pattern of *A. terreus* species to AmB. A slight reduction of MICs was demonstrated, however, *A. terreus* did not turn to be classified as AmB susceptible (AmB MICs < 0.125 µg/ml) under hypoxic conditions. Remarkably, AmB lacked any fungicidal activity in the majority of examined strains; comparing with MICs, MFC seems to be related more with *in vivo* outcome and might partially explain the high failure rate of AmB therapy *in vivo.*

### P015. In vitro activity of active ingredients of disinfectants against drug-resistant fungi

StaufR.^1^TodtD.^2^SteinmannE.^2^RathP.-M.^3^GabrielH.^4^SteinmannJ.^5^BrillF.H.H.^4^1Institute For Clinical Hygiene, Medical Microbiology and Infectiology, Paracelsus Medical University Nuremberg, Nuremberg, Germany2Department For Molecular and Medical Virology, Ruhr-University Bochum, Bochum, Germany3Institute of Medical Microbiology, University Hospital Essen, Essen, Germany4Institute For Hygiene and Microbiology, Dr. Brill + Partner GmbH, Hamburg, Germany5Institute of Clinical Hygiene, Medical Microbiology and Clinical Infectiology, Klinikum Nuernberg, Nuremberg, Germany

**Objectives:** Prevention of immunosuppressed patients against fungal pathogens is essential in times of emergence of antifungal-resistant yeasts and moulds. Therefore, the use of fungicidal disinfectants is an important measure preventing infections. In this study, the fungicidal activity of common microbial agents of chemical disinfectants against antifungal-resistant and antifungal-susceptible yeasts and moulds was tested in comparison to the reference strains *Candida albicans*, *Candida tropicalis, Aspergillus brasiliensis* and *Aspergillus niger*.

**Methods:** In total, nine clinical isolates from the fungal collection of the University Hospital Essen and four reference strains were included. Species identification was performed by morphological methods and sequencing of the internal transcribed spacer region (ITS). The antifungal susceptibility testing was performed via microdilution assay according to EUCAST standard 9.3 for moulds and 7.3.1 for yeasts. The sensitivity of clinical yeasts and moulds isolates in comparison to the reference isolates was tested in a quantitative suspension test against peracetic acid and ethanol as the common active ingredients of chemical disinfectants.

**Results:** Ethanol was sufficiently active with a log_10_ reduction factor of ≥ 4.0 against all yeasts in a concentration of 50 % v/v within 1 min and against all moulds as 80 % solution within 1 min. Peracetic acid was active as 0.25 % solution in 5 min against all yeasts and as 0.5 % solution in 5 min against all moulds. Compared to the reference strains the clinical isolates showed similar or higher sensitivity to the active ingredients. In addition, antifungal-resistant strains exhibited equivalent sensitivity *in vitro* against ethanol and peracetic acid compared to the susceptible ones.

**Conclusion:** Clinical fungal isolates including multidrug resistant ones did show a similar or higher sensitivity to active ingredients of disinfectants than the reference strains.

### P016. In vitro susceptibility of olorofim against 1,682 clinical Aspergillus isolates

BuilJ.^1^OliverJ.^2^BirchM.^2^LawD.^2^RexJ.^2^Tehupeiory-KooremanM.^1^LeeH. Van Der^1^MelchersW.^1^VerweijP.E.^1^1Medical Microbiology, Radboudumc, Nijmegen, Netherlands2F2G Ltd, Manchester, United Kingdom

**Objectives:** Olorofim (OLO) is a novel antifungal agent with potent activity against filamentous fungi such as *Aspergillus spp.*, *Scedosporium* spp. and *Lomentospora prolificans.* We included OLO in our routine MIC-panel for filamentous fungi and analysed the in vitro susceptibility of OLO against clinical *Aspergillus* isolates received in our laboratory between May 2017 and March 2019.

**Methods:** The *in vitro* activity of OLO was tested against 1,682 clinical *Aspergillus* isolates (987 azole susceptible *A. fumigatus* isolates, 414 azole-resistant *A. fumigatus*, 72 *A. flavus* species complex (SC), 32 *A. nidulans* SC, 110 *A niger* SC, 21 *A. terreus* SC, 7 *A. versicolor* SC, 39 other species). OLO susceptibility was determined using broth microdilution based on EUCAST guidelines. Isolates were identified to the SC level using microscopy and growth characteristics.

**Results:** The olorofim MICs of all clinical *A. fumigatus*, *A. flavus species complex*, *A. terreus species complex, A. niger species complex, A. versicolor species complex* were found to be uniformly <1 mg/L. The MIC50 and MIC90 of species with >10 different isolates are displayed in the table. No significant differences were found in the OLO MICs of azole-susceptible A*. fumigatus* isolates compared to azole-resistant *A. fumigatus* isolates. Isolates of some cryptic species within the *Aspergillus aspergillus* species complex (*A. hollandicus, n = 1 A. glaucus, n = 4 and A. chevalieri n = 1)* were found to have elevated MIC’s: 1 isolate (*A. glaucus*) with a MIC of 1 mg/L and the remainder with a MIC of >2 g/L). 

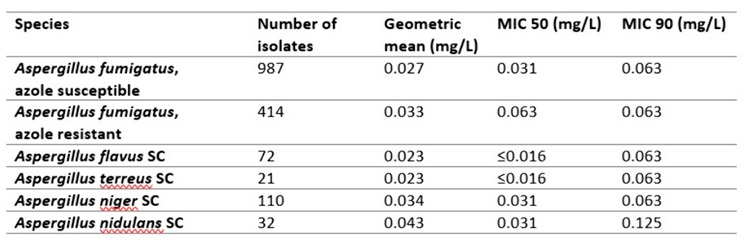



**Conclusion:** OLO showed potent *in vitro* activity against a large collection of clinical *Aspergillus species*, including 1401 *A. fumigatus* isolates, with many of these resistant to one or more azoles. Interestingly, OLO was less potent against a small number of isolates belonging to the *Aspergillus aspergillus* SC (formerly *Eurotium*).

### P017. Detection and Typing of Fluconazole Resistance on Candida albicans with Matrix-Assisted Laser Desorption/Ionization Time-of-Flight Mass

DelavyM.CorthesyM.GreubG.SanglardD.CosteA.T.Microbiology Institute, University Hospital Lausanne, University of Lausanne, Lausanne, Switzerland

**Objectives:** *Candida albicans* causes life-threatening systemic infections in immunosuppressed patients. These infections are commonly treated with fluconazole, an antifungal agent targeting the ergosterol biosynthesis pathway. Current Antifungal Susceptibility Testing (AFST) methods take time and are often subjective. An alternative to the classical AFST methods could use Matrix-Assisted Laser Desorption/Ionization Time-of-Flight (MALDI-TOF) Mass spectrometry (MS). This tool, already used in clinical microbiology for microbial species identification, has already offered promising results to detect antifungal resistance and could be used to detect specific peaks associated with fluconazole resistance.

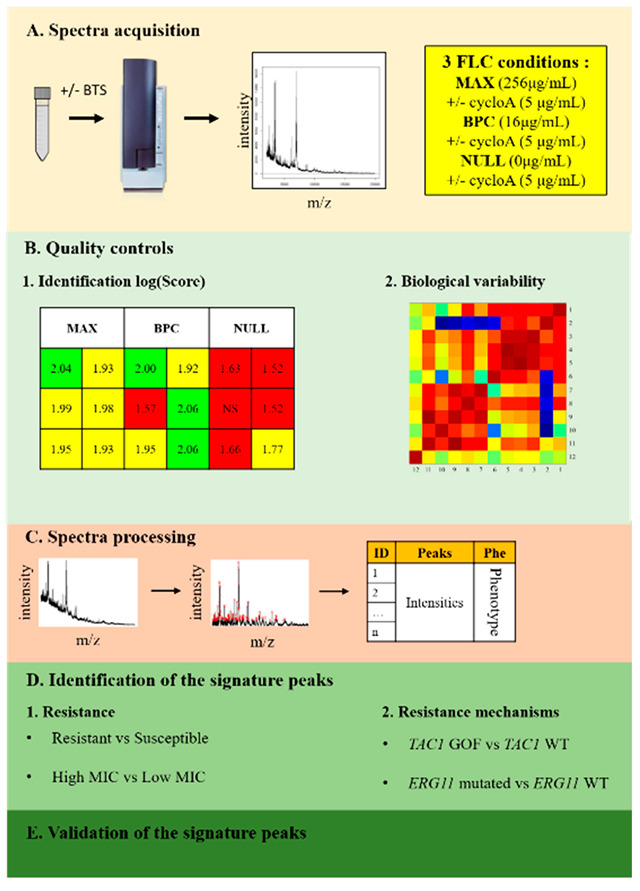



**Methods:** We analyzed MALDI-TOF MS spectra acquired, with the Bruker microflex device, from 21 *C. albicans* clinical strains isolated from 9 patients. Those strains were exposed for 3h to 3 fluconazole concentrations (256, 16, 0μg/mL). We optimized a protein extraction protocol that allowed the acquisition of high-quality spectra. Two steps of quality controls were implemented. First, the quality of the spectrum was assessed through its identification score. Second, the variability of the biological and technical replicates was evaluated, and outliers were removed. We then developed an R pipeline to identify MALDI-TOF MS signature peaks associated with fluconazole resistance or with *TAC1* or *ERG11* mutations known to affect *C. albicans* susceptibility level, and we designed score-based diagnostic tests with these signature peaks. Finally, we evaluated the effects of adding a protein standard (Bacterial Testing Standard, BTS, Bruker) to the proteins extracts to reduce the variability of the peaks position. and then we tested the effect of the tolerance inhibitor cyclosporin A on the susceptible strains’ spectra quality.

**Results:** Up to now, in absence of cyclosporin A or BTS, we identified a 4480 m/z peak, whose intensity after exposition to 16 μg/mL fluconazole, was inversely proportional correlated with fluconazole resistance. The intensity of the peak seems to be more inversely proportional correlated with *TAC1* gain-of-function mutation than with *ERG11* mutations. Since only a single peak was identified, it is likely that the trailing effect of fluconazole might hide other interesting peaks. We thus decide to inhibit fluconazole tolerance using cyclosporin A in further experiments. In addition, the main challenge of this first analysis was to reproducibly align the spectra. To circumvent this issue, we decided to introduce a protein standard (BTS) to the fungal proteins extracts to be analyzed. Effects of BTS or cyclosporin A are currently under investigation.

**Conclusion:** This study acts as a proof-of-concept for the use of MALDI-TOF MS for the typing of fluconazole resistance in *C. albicans* and currently offer promising results.

### P018. Azole resistance in Aspergillus. The first six months data from the Danish nationwide surveillance study

RisumM.^1^HareR.^1^RosenvingeF.S.^2^GertsenJ.B.^3^KristensenL.^3^BangsborgJ.^4^RøderB.L.^5^MarmolinE.S.^6^SulimS.^7^AstvadK.M.T.^8^PedersenM.^8^DatcuR.^1^ArendrupM.C.^1^,^9^,^10^1Mycology Unit, Statens Serum Institute, Copenhagen, Denmark2Department of Clinical Microbiology, Odense University Hospital, Odense, Denmark3Department of Clinical Microbiology, Århus University Hospital, Århus, Denmark4Department of Clinical Microbiology, Herlev Hospital, Herlev, Denmark5Department of Clinical Microbiology, Slagelse Sygehus, Slagelse, Denmark6Department of Clinical Microbiology, Vejle Sygehus, Vejle, Denmark7Department of Clinical Microbiology, Ålborg University Hospital, Ålborg, Denmark8Department of Clinical Microbiology, Hvidovre Hospital, Hvidovre, Denmark9University of Copenhagen, Dept. of Clinical Medicine, Copenhagen, Denmark10Department Og Clinical Microbiology, Rigshospitalet, Copenhagen, Denmark

**Objectives:** Azole resistance (azole-R) in *Aspergillus* has been increasingly reported worldwide. The use of azole fungicides has been proposed to contribute to the emergence of azole-R *A. fumigatus,* and isolates harbouring the resistance mechanisms TR_34_/L98H or TR_46_/Y121F/T289A have been dominant among azole-R *A. fumigatus* in azole naïve patients and environmental samples. These two resistance genotypes have been sporadically found in clinical and environmental samples in Denmark, but the size of the problem remains unknown. The purpose of this study was to establish a nationwide surveillance programme of azole-R *Aspergillus* in Denmark and to report the data from the first six month’s study period.

**Methods:** Unique *Aspergillus* isolates were included from all Danish clinical microbiological departments in the period October-2018 to March-2019. Isolates from same patients were defined as unique if 1) found >30 days apart, 2) confirmation of another species or 3) different susceptibility pattern. Inclusion criteria were: a) *Aspergillus* isolates regarded clinically significant, or b) any *Aspergillus* isolate detected on a Monday regardless of clinical significance. Referral practices varied depending on local mycology skills. Most laboratories referred all moulds or all *Aspergillus* isolates which then underwent species ID and susceptibility testing at the reference lab (SSI). One laboratory (Aarhus University Hospital) reported the species identification for *Aspergillus* (*n* = 214) and only referred part of the isolates. Thus susceptibility was performed for 46/138 *A. fumigatus* isolates from AUH at the time of writing. Referred *Aspergillus* isolates were identified to the complex level except *A. fumigatus* which was identified sensu stricto using thermotolerance testing. *A. fumigatus* isolates underwent screening for azole-R following the EUCAST E.Def 10.1 method and using VIPcheck azole agar plates (Mediaproducts BV, Grönningen, NL). Screening positive *A. fumigatus* isolates and all non-*fumigatus Aspergillus* isolates underwent EUCAST E.Def 9.3.1 susceptibility testing. Isolates were deemed intermediate when classified as such for >1 azole. Isolates with azole MIC(s) above the EUCAST BP(s) underwent *CYP51A* sequencing. Duplicates within 30 days were excluded.

**Results:** A total of 695 *Aspergillus* isolates were included of which 503 were *A. fumigatus.* Susceptibility testing was performed for 411 *A. fumigatus* isolates from 319 patients at time of writing (Table 1). For *A. fumigatus,* 92.7% isolates were azole susceptible, 1.7% intermediate and 5.6% azole-R. Nineteen out of 319 patients harboured an *A. fumigatus* which was azole-R (6%). Among these 18/19 (95%) harboured an isolate with a *CYP*51A mutation (Table 2), and 14/19 (73.7%) a *CYP51A* mutation derived from the environment. Furthermore, 2/9 patients harboured three azole resistant *A. terreus* isolates, each with a *CYP51A* mutation (Table 2).

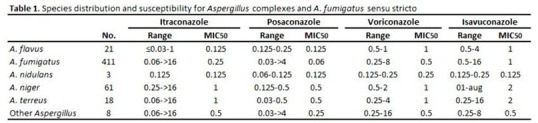

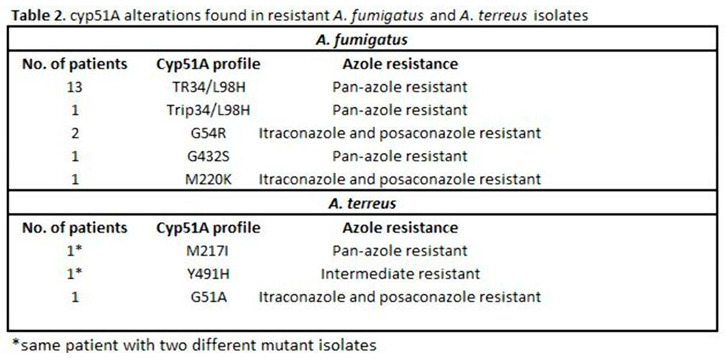





**Conclusion:** We report a nationwide azole-R rate of 6% in *A. fumigatus* at the patient level. The underlying resistance mechanisms was target gene mutations in all but one case and notably, the vast majority were of environmental origin linked to the use of azole fungicides. The fact that such isolates have increasingly been found in Denmark since 2009 is concerning and suggests that a one-health approach involving human and environmental azole management is necessary to limit further rise in azole-R *A. fumigatus*

### P019. Multicentre study to determine the Etest® epidemiological cut-off values of antifungal drugs in Candida spp. and Aspergillus fumigatus SC

SalseM.^1^GangneuxJ.P.^2^CassaingS.^3^DelhaesL.^4^FekkarA.^5^DupontD.^6^BotterelF.^7^CostaD.^8^BourgeoisN.^9^BouteilleB.^10^HouzéS.^11^DannaouiE.^12^GueganH.^2^CharpentierE.^3^PersatF.^13^FavennecL.^8^LachaudL.^9^SassoM.^1^1Laboratoire De Microbiologie, CHU Nîmes, Nîmes, France2Laboratoire De Parasitologie-mycologie, CHU de Rennes, Rennes, France3Jean-pierre.gangneux@chu-rennes.fr, CHU de Toulouse, Toulouse, France4Laboratoire De Parasitologie-mycologie, CHU de Bordeaux, Bordeaux, France5Laboratoire De Parasitologie-mycologie, APHP La Pitié Salpétrière, PARIS, France6Laboratoire De Parasitologie-mycologie, Hospices civils de Lyon, Lyon, France7Laboratoire De Parasitologie-mycologie, APHP Hôpital Henri Mondor, Créteil, France8Laboratoire De Parasitologie-mycologie, CHU de Rouen, Rouen, France9Laboratoire De Parasitologie-mycologie, CHU de Montpellier, Montpellier, France10Laboratoire De Parasitologie-mycologie, CHU de Limoges, Limoges, France11Laboratoire De Parasitologie-mycologie, APHP Hôpital Bichat, Paris, France12Laboratoire De Parasitologie-mycologie, APHP Hôpital Pompidou, Paris, France13Parasitologie Mycologie Médicale, Hospices Civils de Lyon, Lyon, France

**Objectives:** To determine the specific Etest®-based epidemiological cut-off values (ECVs) for the main antifungal agents used in clinical practice, against the most frequent *Candida* spp. and *Aspergillus fumigatus* species complex (SC), in order to harmonise the interpretation of the Etest® minimum inhibitory concentrations (MICs) results in daily routine.

**Methods:** For each antifungal agent, the Etest® MICs in *Candida* spp. and *A. fumigatus* SC isolated from routine specimen cultures were retrospectively collected from 2004 to 2018 from the laboratories of 12 French university hospitals. To ensure that robust and comparable data were included for the ECV estimation, basic requirements and criteria have been fulfilled during the selection of MIC data: aberrant individual distributions were excluded, MIC data were pooled only if there are generated by at least three independent laboratories, and a minimum of 100 MIC values were required after data pooling for each species-antifungal agent combinations. The ECVs were then calculated using the iterative statistical method with a 97.5% cut-off.

**Results:** Forty-eight Etest® ECVs were determined for amphotericin B, caspofungin, micafungin, anidulafungin, fluconazole, voriconazole, posaconazole and itraconazole, after pooling and analysing the MICs of 9,654 *Candida albicans*, 2,939 *Candida glabrata* SC, 1,458 *Candida parapsilosis* SC, 1,148 *Candida tropicalis*, 575 *Candida krusei*, 518 *Candida kefyr*, 241 *Candida lusitaniae*, 131 *Candida guilliermondii*, and 1,526 *A. fumigatus* SC isolates. The ECV results were identical (17/32 ECVs) or within one two-fold dilution (15/32 ECVs) to the previously reported Etest®-based ECVs. They were comparable to the Clinical and Laboratory Standards Institute (CLSI) ECVs in 76.1% (similar or within one two-fold dilution: 35/46 ECVs) and strictly identical in 39.1% of cases (18/46 ECVs). They were comparable to the European Committee on Antimicrobial Susceptibility Testing (EUCAST) ECVs in 81.2% of cases (similar or within one two-fold dilution: 26/32 ECVs) and strictly identical in 28% (9/32 ECVs).

**Conclusion:** On the basis of these and other previous results, we recommend the determination of method-dependent ECVs. As Etest® is the most widely used commercial method in French laboratories, the goal of this study was to calculate specific ECVs for this method and harmonise the interpretation of the Etest® MICs results in daily routine. Etest® ECVs should not be used in place of breakpoints but may identify non-WT isolates with a potential resistance to antifungal agents and suggest that an isolate may not respond as expected to the standard treatment.

### P020. Human cell cultures exposed to antibiotic amphotericin B: toxicity and defence

GrelaE.^1^PietM.^2^LuchowskiR.^1^GrudzinskiW.^1^PaduchR.^2^GruszeckiW.^1^1Biophysics, Maria Curie-Sklodowska University, Lublin, Poland2Virology and Immunology, Maria Curie-Sklodowska University, Lublin, Poland

**Objectives:** Amphotericin B (AmB) is a polyene antifungal antibiotic, which is synthetized by the bacteria *Streptomyces nodosus*. AmB is a “gold standard” in treatment of systemic fungal infections, due to its broad spectrum of action, high effectiveness and rare pathogens resistant to this drug. Unfortunately due to the low selectivity of the action of this antibiotic, it is very toxic for patients. Although, the exact mechanism of action of AmB is still unknown, it is used as a lifesaving medicine. Understanding the mechanism of action of AmB would enable to minimize its toxic side effects, and possibly increase its therapeutic effects. The aim of this study was to get to know the toxicity mechanism of action of AmB.

**Methods:** To understand molecular mechanism underlying the toxicity of AmB, human normal colon epithelial cells CCD 841 CoTr (ATCC No. CRL-1807) and human colon adenocarcinoma cells HT-29 (ATCC No HTB-38) were exposed to the drug and were imaged using Fluorescence Lifetime Imaging Microscopy (FLIM) and Raman scattering spectroscopy. Cultures were incubated for 24 h with AmB. Fluorescence lifetime imaging was carried out on MicroTime 200 (Picoquant GmbH, Germany) linked with Olympus IX71 inverted microscope. The cell samples were excited by 405 nm pulsed laser. Fluorescence lifetimes and fluorescence anisotropy values were analyzed. Raman spectral analysis and imaging on a microscale was carried out with inVia Reflex confocal Raman microscope (Renishaw, UK) with Cobolt o8-NLD 405 nm laser (power at a sample 0.2 mW). Obtained results were analyzed by DCLS spectral deconvolution.

**Results:** The results confirmed high toxicity of antibiotic AmB for both human cell lines but lower for HT-29 cells. The results show that AmB incorporates to the lipid membrane containing cholesterol. The cell defense mechanism against the action of antibiotic was also observed, which is based on removing AmB from the membrane in the form of sterol-deficient exosomes.

**Conclusion:** The lower toxicity of AmB observed for HT-29 cells, may be associated with high proliferation rate or different membrane composition of adenocarcinoma cells compared to CCD 841 CoTr cells. AmB binds to the bilayer in the form of small aggregates, which can cause uncontrolled ion-leakage and lead to the cell death. 

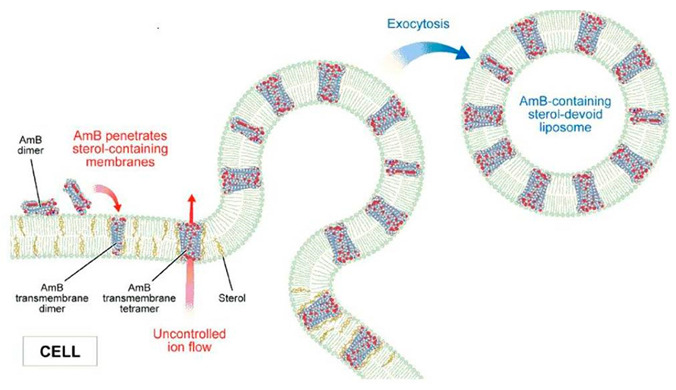



### P021. African ST173 Cryptococcus deuterogattii strains are commonly less susceptible to fluconazole: a tricky mechanism of resistance

BelletV.^1^RogerF.^1^GouveiaT.^1^DrakulovskiP.^1^KassiF.^2^MenanH.^2^KrastevaD.^1^BertoutS.^1^1Laboratoire De Parasitologie Et Mycologie Médicale Umi 233, University of Montpellier, Montpellier, France2CEDRES CHU de Treichville, Abidjan, Côte d’Ivoire

**Objectives:** Fluconazole, alone or in association, is often administered during cryptococcosis treatment especially in Sub-Saharan Africa. Its intensive use leads to the emergence of resistant strains. The mechanisms underlying resistance are poorly documented for yeasts belonging to the *C. gattii* species complex. Literature suggests that resistance could be due to mutations and/or surexpression of ERG11 gene and overexpression of efflux pumps, like MDR or AFR.

**Methods:** In Ivory Coast, we highlighted the presence of strains with a rare subtype in the molecular type VGII associated with high minimum inhibitory concentrations to FCZ compared to VGII strains originating from the Pacific Northwest. We investigated the mechanisms of fluconazole resistance of 18 ivorian clinical strains of *C. deuterogattii* with high MIC to FCZ compared to 10 strains with low MIC to FCZ.

**Results:** We demonstrated that (i) these strains exhibited no mutation in the *ERG11* gene, (ii) some strains had an increased in *ERG11* and *MDR1* mRNA expression while *AFR1* and *AFR2* were not overexpressed in high MIC to FCZ strains versus the low MIC to FCZ strains and (iii) exposure to FCZ in strains with high MIC to FCZ induced AFR1 mRNA overexpression.

**Conclusion:** We demonstrated that the FCZ-resistant mechanism commonly described in *Cryptococcus neoformans* or in *Cryptococcus gattii* from the Pacific Northwest were not responsible for the resistance to FCZ in this rare sub-type strains.

### P022. Novel Technique to Measure Susceptibility to Lipid-Associated Antifungals

FrenkelM.^1^YunikY.^1^SegalE.^2^1Tel Aviv University, Tel Aviv, Israel2Sackler School of Medicine, Clinical Microbiology and Immunology, Tel Aviv University, Tel Aviv, Israel

**Objectives:** Lipid associated antifungals, such as polyenes, are extensively used for therapy in patients with invasive fungal infections. Thus, susceptibility of fungi to the lipid- associated antifungals is of importance. The most reliable method used in susceptibility testing is the broth micro-dilution technique. However, visual- or spectral- assessment of the antifungal activity is difficult, and possibly unreliable, in lipid-associated drugs, due to turbidity caused by the lipid. Hence, the objective of this study was to adapt a method for spectral measurement of susceptibility of fungi to lipid-associated antifungal drugs.

**Methods:** The technique was adapted for Amphotericin B- Intralipid (AMB-IL) and Nystatin- Intralipid (NYT-IL) in comparison to AMB and NYT. Drug preparation: AMB-IL was prepared by adding to 5 mg/ml of AMB 20% Intralipid in DDW to a final concentration of 0.2 mg/ml AMB and shaken at 280 rpm for 18 h at 24^0^ C. Intralipid in DDW was used as control. NYT-IL was prepared similarly, by adding to 20 mg/ml nystatin 20% Intralipid in DMSO to a final concentration of 0.8 mg/mL NYT and shaken at 280 rpm for 18 h at 24^0^ C. Intralipid in DMSO was used as control. Susceptibility test: Susceptibility of the *Candida albicans* strains to AMB, NYT, AMB-IL, and NYT-IL was assessed by the micro-broth dilution assay using micro-titer plates. Susceptibility was evaluated using spectral readings in an EMax+ Microplate Reader at 560nm and 650nm, for the standard (AMB, NYT) and experimental Intralipid-associated polyene formulations (AMB-IL, NYT-IL), respectively. For AMB-IL or for NYT-IL the drug solutions and the relevant controls were diluted in RPMI. Each well contained the same concentration of RPMI, IL, DDW or DMSO, and serial dilutions of the antifungal drugs (AMB, NYT).

**Results:** The validity of the test was evaluated by testing susceptibility of 40 *C. albicans* isolates: 20 from human mucosal infections (M strains) and 20 from candidemia patients (S strains) vs. a control strain *C. albicans* CBS-562, for which susceptibility data were available. We determined 50, 90 & 100% MIC values by spectral readings (see an example of tests with the control stain – CBS 562–Figure 1). The spectral readings were compared to visual readings of the micro-titer plates using an inverted microscope. In addition, minimal fungicidal concentration (MFC) for each strain was determined (Figure 2). 

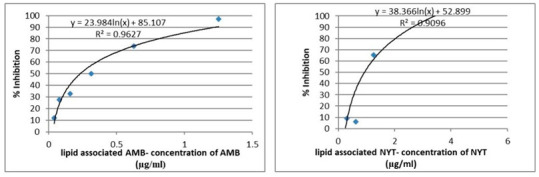



Figure 1: Spectral readings of Control strain –CBS 562 treated with AMB-IL & NYT-IL.

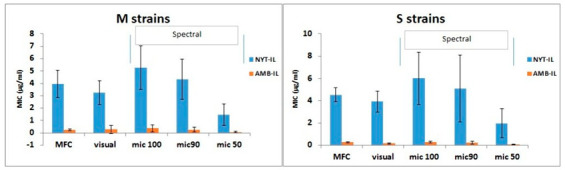



Figure 2: Spectral readings vs. Visual assessment of M and S *Candida albicans* strains.

**Conclusion:** This study presents a spectral method for measuring susceptibility of yeasts to lipid associated polyenes which deals with the difficulties of visual assessment of susceptibility to lipid associated antifungals.

### P023. Antifungal activity of antimicrobial peptides against clinical and environmental Aspergillus fumigatus isolates.

BallardE.^1^YucelR.^2^MelchersW.^3^BrownA.^1^VerweijP.E.^3^WarrisA.^1^1Mrc Centre For Medical Mycology, University of Aberdeen, Aberdeen, United Kingdom2Ifcc, University of Aberdeen, Aberdeen, United Kingdom3Medical Microbiology, RadboudUMC, Nijmegen, Netherlands

**Objectives:** Antimicrobial peptides (AMPs) are a key defence against invading microorganisms. The activity of AMPs against human fungal pathogen *Aspergillus fumigatus* remains poorly understood. We characterised the anti-*Aspergillus* activity of specific human AMPs and determined whether in-host adaption could lead to resistance to specific AMPs.

**Methods:** The antifungal activity of lysozyme, histones, lactoferrin, β-defensin-1 and LL-37, was tested against 15 clinical isolates (from patients with cystic fibrosis, chronic and acute invasive aspergillosis), 5 environmental isolates and 13 isogenic *A. fumigatus* isolates obtained from a single patient over 2 years showing in-host acquired azole resistance. Hyphae grown from 5x10^4^ conidia and incubated with individual AMPs at various concentrations for 2 h at 37oC, after which metabolic activity was determined by XTT. *Aspergillus* conidia (1x10^5^ per well) were incubated for 10 h at 37oC with specific AMPs and fixed in 4% paraformaldehyde fixing solution. Analysis of hyphal length was performed using the Amnis ImageStreamX MK II Imaging Flow Cytometer (Luminex, USA). Data analysis was performed using IDEAS software.

**Results:** Dose-dependent decreases in hyphal metabolic activity in all isolates were shown for lysozyme (20-80 µM; 31-65% and 5-25% decrease respectively; p<00001) and histones (50-100 ug/ml; 35-66% and 6-45% decrease respectively; p<0.0001). No effect was observed for lactoferrin (10-40 µM), β-defensin-1 (1-10 µM) and LL-37 (5-50 µM). Imaging flow cytometry revealed that incubation with histones (100 µg/ml), β-defensin-1 (10 µM) or lactoferrin (40 µM) resulted significantly fewer cells in the hyphae gate (p<0.0001, p = 0.04 and p = 0.003, respectively). No significant differences were observed with lysozyme (80 µM) or LL-37 (12.5 µM).

**Conclusion:** Lysozyme and histones show dose dependent antifungal activity against *A. fumigatus* hyphae, irrespective of the isolate’s azole resistance profile. In addition, histones, lactoferrin and β-defensin-1 display inhibitory activity against germinating conidia. No differences were observed in the susceptibility to AMPs between the groups of *A. fumigatus* isolates used. We did not identify any trends indicating increased resistance to AMPs as a result of in-host adaptation.

### P024. Characterization of I1380T a new FKS1 polymorphism in Candida parapsilosis that confers high-level echinocandin resistance when associated to P660A

AccoceberryI.^1^El-Kirat-ChatelS.^2^QuilèsF.^2^BiteauN.^1^NoëlT.^1^1Cnrs Umr5234, University of Bordeaux, Bordeaux, France2Cnrs Umr 7564, Université de Lorraine, Villers-lès-Nancy, France

**Objectives:** The aim of this study was to determine the impact on echinocandin resistance of a new mutation identified in a *FKS1* allele of a clinical isolate of *C. parapsilosis*.

**Methods:** To assess the role of mutations potentially implicated in echinocandin resistance, a *C. lusitaniae* strain specially engineered for the expression of a single chromosomal copy of *FKS1* was used for the expression of I1380T and P660A *FKS1* alleles. The echinocandin susceptibility of the mutants, as well as their growth rates and phenotypes of interaction with murine macrophages, were evaluated in the absence or in the presence of caspofungin. Atomic force microscopy (AFM) was used to observe the morphological changes induced by caspofungin exposition. Finally, infrared spectroscopy in attenuated total reflection mode (IR-ATR) spectroscopy was used to detect and quantify the biochemical signatures of the cell wall before and after caspofungin treatment.

**Results:** A caspofungin-resistant *C. parapsilosis* bloodstream isolate was recovered from a patient who underwent allogeneic bone marrow hematopoietic stem-cell transplantation (HSCT) for an idiopathic medullary aplasia with positive PNH clone. Full-length nucleotide sequencing of the *FKS1* gene showed that the strain harboured the heterozygous mutation I1380T (isoleucine at position 1380 replaced by threonine) located 4 amino acids beyond the HS2 region (amino acids 1369 to 1376), compared to Fks1p of the *C. parapsilosis* reference strain CBS 604, in addition to the naturally occurring P660A polymorphism responsible for increased echinocandin MICS in this species. To characterize the impact of each mutation on caspofungin resistance, *FKS1* alleles bearing I1380T associated or not to P660A were genetically engineered and expressed in *Candida lusitaniae*. When compared to a wild type allele bearing no mutation, I1380T conferred a 12-fold MIC increase, P660A a 6-fold MIC increase and the combination of both mutations a ≥ 256-fold MIC increase. Measuring the cellular interactions between *C. lusitaniae FKS1* mutants and murine macrophages showed that, in the presence of caspofungin, I1380T enhanced yeast phagocytosis but had no effect on intramacrophagic yeast survival rates. AFM analysis of *FKS1* mutants showed that caspofungin treatment impacts the cell surface topography in both susceptible and resistant strains, and that the single I1380T mutation leads to morphological changes even in the absence of caspofungin treatment. Infrared spectroscopy in attenuated total reflection mode (IR-ATR) showed that under high drug concentration exposure, the *FKS1* I1380T mutants exhibited a massive β-glucan synthesis inhibition that was not proportionally compensated by chitin synthesis.

**Conclusion:** We have identified I1380T, a new mutation located close to the HS2 region of the *FKS1* gene of a clinical isolate of *C. parapsilosis*. We demonstrated that the high caspofungin MIC for this isolate was depending on the combination of I1380T with the naturally occurring P660A polymorphism. Further characterization at the cell wall level suggested a potential fitness cost caused by I1380T.

### P025. Azole resistant Aspergillus fumigatus: surveillance from flowered patios to departments with at-risk patients

GodeauC.SchererE.LaboissièreA.Léchenault-BergerotC.RebouxG.MillonL.RocchiS.Mycology - Umr6249 Cnrs Chrono-environnement, University Hospital Besançon, Besançon, France

**Objectives:** The University Hospital of Besançon is geographically located in a highly agricultural area (wood, vegetables, cereals, fruit and vines), and is very concerned by the emergence of azole resistance in *Aspergillus fumigatus* (ARAf), having an increasing number of cases especially amongst cystic fibrosis patients. For more than 20 years, the University Hospital of Besançon has been taking weekly samples (*n* = 25) to assess fungal air contamination in the 3 departments of hematology and corridors. Since 2015, the number of ARAf found in corridors has been increasing. This study aimed to determine whether plant, tree and flowerbeds within or near the hospital are sources of ARAf and to assess their spread into the hospital.

**Methods:** Four soil sampling areas within the hospital and in the surrounding environment have been identified. Zone A corresponds to an outdoor relaxation area within the hospital, zone B consists of flowerbeds in front of the hospital entrance, zone C (inside) corresponds to pots containing trees below the hospital reception area and finally zone D corresponds to an outdoor area at level -1 below the zone A. From January 2019, external samples were taken using a homemade dust vane sensor equipped with 2 wipes, placed on a terrace at second floor, to capture the prevailing wind. Dust aspiration was also achieved using a filter placed at the end of a vacuum cleaner, towards the emergency door of the department of hematology, in the corridor along zone A, then in hematology consultation department. Soils and dust (wipes and aspiration) samples were seeded on medium containing itraconazole and voriconazole (2mg/L). The usual weekly air samples taken in the hematology departments and corridors from January 2019 were also analyzed. Resistance of *A. fumigatus* growing on azole media was tested using EUCAST method and *cyp51A* gene of ARAf were sequenced.

**Results:** From the 85 soils samples, 137 colonies were isolated on azole medium from zones A, B and D, grew on azole media. Resistance of 90 *A. fumigatus* isolates was evaluated with EUCAST method. Other strains (zone B) were *Neosartorya fisheri.* Moreover, 71% (64/90) of *A. fumigatus* isolates were resistant to at least one azole. So far, 20 strains have been sequenced and all harbored the TR_34_/L98H mutation. 59 ARAf were found in pots containing tulips coming from Netherlands and 5 in soil pots containing trees. Three isolates were detected on azole media with dust vacuum and 2 (hematology department and corridor along zone A) were resistant with TR_34_/L98H mutation. No resistant strains were collected with the external sensor. Four ARAf with the TR_34_/L98H mutation were also isolated from air of corridors.

**Conclusion:** Patios containing Dutch tulips in particular, had a significant number of ARAf. On the other hand, samples taken upstream of the esplanades, in relation to the prevailing wind, did not present any ARAf. Addionally, ARAf were also found in hospital corridors and to emergency exits, but not in hematology departments. Genotyping analyses will be carried out to confirm that tulips can be a source of ARAf found inside the hospital.

### P026. Triazole-resistant Aspergillus fumigatus: Evaluation of the Fungiplex® Aspergillus and Azole-R kits

RocchiS.SchererE.LaboissièreA.Léchenault-BergerotC.GodeauC.BellangerA.-P.RebouxG.MillonL.Mycology - Umr6249 Cnrs Chrono-environnement, University Hospital Besançon, Besançon, France

**Objectives:** Cases of Aspergillosis with azole resistant *Aspergillus fumigatus* are increasingly described. Some resistance mechanisms, with two major mutations on the *cyp51A* gene (TR_34_/L98H and TR_46_/Y121F/T289A), are linked to the unintended impact of triazole fungicides in agriculture. At the University Hospital of Besançon, 216 resistant strains have been isolated since 2012 and 142 sequenced for beta-tubulin: 52% from professional environments, 28% from clinical samples, 10% from hospital corridors, 7% from the inside garden of the Hospital and 3% from patients’ homes. Given the seriousness of resistant infections, it is important to detect cases of resistance as soon as possible. It is in this context that we proposed to test to Fungiplex ® (*Aspergillus* et Azole R) kits (Bruker).

**Methods:** The Fungiplex® *Aspergillus* Azole-R kit was retrospectively evaluated on DNA extracts from a collection for which *cyp51A* gene sequencing and genotyping was previously performed. According to the instructions, the Azole-R kit is made for the identification of azole resistance markers in *Aspergillus* positive species directly from serum, plasma and bronchoalveolar lavage. We are evaluating the kits for the routine testing of cystic fibrosis patients working from isolated strains and directly on sputum. We also are testing the performance of the kits to detect 1) *Aspergillus fumigatus* strains 2) resistant strains directly in soil samples. To do this, we worked on inoculated soils with increasing concentrations of resistant strains (from 5 to 50 000 spores/mL with 200µL sprayed on 250 mg of soil) and directly on soils containing resistant strains.

**Results:** 97 resistant strains previously characterized by sequencing techniques (beta-tubulin and cyp51A) were analyzed: 82 TR_34_/L98H, 2 TR_34_/L98H/S297T/F495I, 9 TR_46_/Y121F/T289A and 4 other resistant strains. Results showed that 99% of strains carrying one of the two mutations are detected. The first prospective results (22 resistant strains in Etest, analysed directly with the kits) made it possible to detect 22 *A. fumigatus* with the TR_34_/L98H mutation. Verification sequencing is in progress. Sputum analysis and tests with cryptic species are also in progress. Tests on inoculated soils show that resistant strains can be detected even for the smallest inoculated quantities (200 µL of solution concentrated at 5 spores/mL). Real soil tests provide a signal for *Aspergillus* but not for the TR_34_ and TR_46_ mutations. Internal reaction controls are also not detected, suggesting a problem with DNA extraction and inhibitors rather than poor kit performance. Different methods for extracting DNA directly from the soil are under evaluation.

**Conclusion:** The kits show ease of use and very good reliability on retrospective analyses with 99% of the strains tested validated. The performance of the *Aspergillus* kit will also be evaluated on cryptic species of *A. fumigatus* (*Neosartorya fisherii, A. oerlinghausenensis, Neosartorya hiratsukae, A. lentulus*). If performance is equally good in ongoing prospective analyses, these kits will be promising to improve early diagnosis.

### P027. Relation between hanseniasis and superficial mycoses: prevalence, fungal identification and antifungal susceptibility.

ScrofernekerM.L.^1^HeidrichD.^2^VettoratoR.^2^,^3^EidtL.^4^RibeiroA. Carvalho^1^PaganiD.^5^HellwigA.H. Da Silva^2^AmaroT.^3^1Department of Microbiology, Immunology and Parasitology, Universidade Federal do Rio Grande do Sul, Porto Alegre, Brazil2Postgraduate Program In Medicine: Medical Sciences, Universidade Federal do Rio Grande do Sul, Porto Alegre, Brazil3Serviço de Dermatologia do complexo Hospitalar Santa Casa de Misericórdia de Porto Alegre, Porto Alegre, Brazil4Ambulatório de Dermatologia Sanitária de Porto Alegre, Porto Alegre, Brazil5Postgraduate Program In Agricultural and Environmental Microbiology, Universidade Federal do Rio Grande do Sul, Porto Alegre, Brazil

**Objectives:** The aim of this study was to evaluate superficial mycoses in patients with hanseniasis in relation to the prevalence of fungal species causing mycoses and susceptibility to antifungals.

**Methods:** A cross-sectional study was carried out with patients who were seen between May and October 2017 at the Hanseniasis Service of the Sanitary Dermatology Ambulatory in the city of Porto Alegre, Brazil. The samples collected were submitted to direct (DME) and cultural (CME) mycological examinations at the G post of the Santa Clara Hospital in Porto Alegre, were identified by region sequencing specified for each genus of fungus and traced sensitivity profile to clinical antifungals using protocols M38-A2 and M27-A3 from the Clinical and Laboratory Standards Institute.

**Results:** A total of 91 hanseniasis patients were evaluated and 37 presented suspicion mycosis. of these, 23 had DME positive and 14 CME cultures were identified, with eight dermatophytes (seven *Trichophyton interdigitale* and one *Epidermophyton floccosum*), two isolates of *Fusarium keratoplasticum*, two *Acremonium* sp., one *Candida albicans* and one *Arthrinium arundinis*. Three patients had claws, caused by hanseniasis, at the mycosis site, indicating a relation of the two diseases (3/5 patients with claw). Terbinafine presented the lowest minimum inhibitory concentrations (MICs) for dermatophyte isolates (0.0078-0.06 μg/ml), while fluconazole MICs were the highest (4-> 64μg/ml). *Fusarium keratoplasticum* and *Acremonium* sp. have higher MICs than all antifungal agents than dermatophytes.

**Conclusion:** This is the second onychomycosis caused by *Arthrinium arundinis* from the literature and showed low sensitivity to antifungals. Itraconazole had higher MICs for dermatophytes isolated from patients with hanseniasis (0.25-1μg/ml) than without the disease cited in the literature, indicating a relation of susceptibility to antifungals between mycosis and hanseniasis.

### P028. Prevalence of azole-resistant Aspergillus fumigatus isolates from respiratory specimens of patients from Lyon and exploration of resistance mechanisms

SimonL.^1^KramerR.^2^DupontD.^3^DéméautisT.^4^GarnierH.^5^LinaB.^6^RabodonirinaM.^7^WallonM.^3^PersatF.^8^MenottiJ.^4^1Parasitology and Medical Mycology, Hospices Civils de Lyon / CHU de Nice, Nice, France2Parasitology and Medical Mycology, and Virology, Hospices Civils de Lyon / EUPHEM, Lyon, France3Parasitology and Medical Mycology / Umr 5292, Université Lyon 1 / Hospices Civils de Lyon, Lyon, France4Parasitology and Medical Mycology / Ea7426 Inflammation and Immunity of The Respiratory Epithelium, Université Lyon 1 / Hospices Civils de Lyon, Lyon, France5Parasitology and Medical Mycology, Hospices Civils de Lyon, Lyon, France6Virology / U1111, Université Lyon 1 / Hospices Civils de Lyon, Lyon, France7Parasitology and Medical Mycology / U1111, Université Lyon 1 / Hospices Civils de Lyon, Lyon, France8Parasitology and Medical Mycology, Université Lyon 1 / Hospices Civils de Lyon, Lyon, France

**Objectives:** Resistance of *Aspergillus fumigatus* strains to triazole antifungals is increasingly reported in Europe. The main mechanisms of resistance described are mutations in the cyp51A gene encoding a lanosterol 14-α-demethylase or its promoter. As few data are available in Southern France, our objective was to assess the burden of *Aspergillus* isolates with azole resistance from clinical specimens in Lyon University Hospitals

**Methods:** In this retrospective cross-sectional study, 203 consecutive *A. fumigatus* isolates were identified during a seven-month period from February to September 2017 from respiratory specimens from 182 patients attending the inpatient and outpatient wards of the Pulmonary Medicine Departments of Lyon University Hospitals. Morphological identification was confirmed by sequence analysis of the β-tubulin gene. Minimum inhibitory concentrations were determined using E-test reagent strips for itraconazole, voriconazole, posaconazole, and isavuconazole. Resistance was defined according to the 2018 EUCAST clinical breakpoints. The molecular resistance mechanisms were searched for by sequence analysis of the cyp51A gene and its promoter region, as well as by gene expression analysis of the cyp51 genes and genes encoding several efflux transporters.

**Results:** PCR and sequence analysis of the β-tubulin gene confirmed the identification of *Aspergillus fumigatus* for the 203 isolates. Four isolates presented with azole resistance: two isolates against itraconazole/posaconazole/isavuconazole and another two against all four triazoles. Out of these four strains, three presented silent polymorphisms in an intronic part of cyp51A and one presented simultaneously the F46Y, M172V and E427K mutations. No mutation was identified in the cyp51A promoter, but significant inductions of cyp51A and cyp51B gene expression were observed for all four isolates and three isolates, respectively. Significant inductions of atrF and crd1B gene expression were observed for two and three isolates, respectively. No significant induction of MDR1-4, MFS56 and M85 gene expression was observed.

**Conclusion:** The prevalence of azole resistance in our study population was 2.2% (95% CI 0.9-5.6). Only polymorphisms were found in the cyp51A gene and no mutation was found in its promoter. Nevertheless, significant inductions of the expression of the cyp51 genes and two genes encoding efflux transporters were evidenced, underlying the diversity of resistance mechanisms to be explored.

### P029. Antifungal activity of a mastoparan analog peptide: mode of action and in vivo evaluation in a Galleria mellonella model

SingulaniJ.^1^GaleaneM.C.^1^RamosM. Dorisse^1^GomesP.C.^1^SantosC.^1^SouzaB. Monson De^2^PalmaM.S.^2^AlmeidaA.M. Fusco^1^GianniniM.J. Soares Mendes^1^1Clinical Analysis, UNESP, Araraquara, Brazil2Biology, UNESP (Sao Paulo State University), Rio Claro, Brazil

**Objectives:** The purpose of this work was to evaluate the antifungal activity and toxicity of a peptide analog of mastoparan class from wasps (MK58911) under both *in vitro* and *in vivo* conditions.

**Methods:** Firstly, MK58911 was tested against *Cryptococcus neoformans* ATCC 90112 using the microdilution susceptibility test according to the CLSI M27-A3 (2008). In addition, the mechanism of action on fungal membrane, death cell events (apoptosis and necrosis) and reactive oxygen species (ROS) was evaluated by flow cytometry. Secondly, the peptide toxicity tests on lung fibroblasts (MRC5) and glioblastoma cells (U87) were performed. Finally, the efficacy and toxicity of the peptide were evaluated *in vivo* using a *Galleria mellonella* model.

**Results:** The results showed a promising activity of the peptide with minimal concentration inhibitory (MIC) of 31.2 μg/mL and low toxicity in MRC and U87 cells (IC50 > 500 μg/mL). Taken together, these results demonstrated that the peptide has a high selectivity by fungal cells compared to mammal cells (selectivity index - SI > 16). The peptide presented a mechanism of action on plasma membrane, leading to death of the fungus mainly by the necrosis process and without production of reactive oxygen species. Finally, the peptide showed no toxic effects on *G. mellonella* and significantly enhanced the survival rates and decreased the fungal burden of larvae infected with *C. neoformans*. These effects were independent of an immunomodulatory activity.

**Conclusion:** The results showed the peptide as a potential new antifungal drug against cryptococcosis. "This study was supported by the Fundação de Amparo à Pesquisa do Estado de São Paulo (FAPESP grants no. 2017/06658-9 and 2016/16212-5)."

### P031. Screening for Triazole resistance in clinically significant Aspergillus species from Pakistan

ZafarA.MoinS.JabeenK.FarooqiJ.LaiqS.Pathology and Laboratory Medicine, Aga Khan University, Karachi, Pakistan

**Objectives:** To determine the frequency of triazole (itraconazole, voriconazole, posaconazole) resistance in clinically significant *Aspergillus* species isolated at a tertiary care centre, Karachi, Pakistan.

**Methods:** A descriptive cross-sectional study was conducted in the Department of Pathology and Laboratory Medicine, Microbiology Section of the Aga Khan University Clinical Laboratories, Karachi, Pakistan from September 2016 to May 2019. One hundred and fourteen, clinically significant *Aspergillus* isolates [*A.fumigatus* (38; 33.3%), *A.flavus* (64; 56.1%), *A.niger* (9; 7.9%) and *A.terreus* (3; 2.6%)] were included in the study. They were assessed for their clinical significance. The clinical spectrum ranged from Invasive Aspergillosis (IA) (*n* = 25; 21.9%), further divided into proven invasive extrapulmonary aspergillosis (*n* = 8; 7%), proven invasive pulmonary aspergillosis (IPA) (*n* = 6; 5.3%), putative/probable IPA (*n* = 11; 9.6%) according to the AspICU and 2008 EORTC criteria, to Chronic Pulmonary Aspergillosis (CPA) (*n* = 58; 50.9%), Allergic Bronchopulmonary Aspergillosis (ABPA) (*n* = 4; 3.5%), Severe Asthma with Fungal Sensitization (SAFS) (*n* = 4; 3.5%) and saprophytic tracheobronchial aspergillosis (*n* = 23; 20.2%). Screening for triazole resistance was performed by antifungal agar screening method as described by Mortenssen et al. The minimum inhibitory concentration (MIC) of 41 representative isolates [*A.flavus* (*n* = 15; 13.2%), *A.fumigatus* (*n* = 15; 13.2%), *A.niger* (*n* = 8; 7%), *A.terreus* (*n* = 3; 2.6%)] representing a clinical spectrum of extrapulmonary IA (*n* = 7; 6.1%), IPA (*n* = 4;3.5%) putative/probable IPA (5; 4.4%), CPA (*n* = 18;15.8%), ABPA (*n* = 3; 2.6%), SAFS (*n* = 1; 0.9%), and saprophytic tracheobronchial aspergillosis (*n* = 3; 2.6%) were tested according to the CLSI broth microdilution method.

**Results:** All the isolates were categorized as triazole-susceptible based on the triazole antifungal agar screening. The MICs of the three azole antifungals for 41 representative isolates tested were found to be ≤1 ml/L and hence according to CLSI breakpoints, all the isolates tested were found to be triazole-susceptible. The MIC 90 of itraconazole, voriconazole and posaconazole of the representative aspergillus isolates was 1mg/L, 1mg/L and 0.5mg/L respectively.

**Conclusion:** This study may set precedence for future periodic antifungal resistance surveillance studies in our region on *Aspergillus* isolates causing invasive disease, as well as other syndromes requiring long term antifungal therapy.

### P032. Antifungal susceptibility profile of invasive Candida glabrata isolates (2009-2019) from a tertiary care hospital laboratory in Pakistan

FarooqiJ.^1^MemonS.^2^LaiqS.^1^NaqviF.^1^ZafarA.^1^JabeenK.^3^1Pathology and Laboratory Medicine, Aga Khan University, Karachi-KHI, Pakistan2Microbiology, Karachi University, Karachi, Pakistan3Pathology and Laboratory Medicine, Aga Khan University, Karachi, Pakistan, Pakistan

**Objectives:** *Candida glabrata* invasive infections are increasingly associated with antifungal resistance and poor clinical outcomes. The objective of this study was to evaluate antifungal resistance and distribution of minimum inhibitory concentrations (MICs) against invasive *C. glabrata* isolates (200-2019) at Aga Khan University (AKU) at Karachi, Pakistan. This laboratory, through its network of satellite collection centers receives specimens from more than 100 major cities and towns of the country.

**Methods:** 162 *Candida glabrata* strains were isolated from blood (101), body fluids (26), pus and wounds (21) tissue (4) and others (10). Isolates were identified by conventional method using API 20C AUX, gross morphology on chromogenic and microscopic morphology on corn meal agar. MICs were determined using colorimetric broth microdilution (YeastOne Sensititre, Trek diagnostics). Susceptibilities were interpreted according to Clinical Laboratory standard Institute breakpoints mentioned in “Performance Standard for Antifungal Susceptibility Testing of Yeasts M60-ED1:2017”.

**Results:** Out of 1917 archived invasive candida, 162 (8.4%) were *C.glabrata* 76% of these isolates were from patients admitted at AKU. Male to female ratio was 1.4 and 62% of the isolates were from ages 18-64 year. MIC_90_ of these strains against fluconazole was 64 µg/ml, voriconazole 2µg/ml, itraconazole 1µg/ml and posaconazole 2µg/ml. Among echinocandins, MIC_90_ was 0.12µg/ml for caspofungin, 0.06µg/ml for anidulafungin and 0.03µg/ml for micafungin. MIC_90_ for amphotericin B was 0.5µg/ml.

**Conclusion:** Antifungal sensitivity testing for invasive candidiasis is essential in the face of emerging resistance as empiric therapy may not have reliable outcomes. Surveillance data of antifungal resistance among common *Candida* species with potential for developing antifungal resistance, like *C. glabrata*, should be monitored closely for identifying resistant strains in circulation.

### P033. Clinical implications of azole-resistant vs. azole-susceptible invasive aspergillosis in hematological malignancy (CLARITY) – a multicenter study

SeidelD.^1^CornelyO.A.^2^KöhlerP.^1^,^3^MeisJ.^4^ZarroukM.^3^Salmanton-GarciaJ.^3^ArenzD.^5^BlennowO.^6^BuchheidtD.^7^VehreschildJ.-J.^3^AlakelN.^8^BergeronA.^9^DesoubeauxG.^10^Falces-RomeroI.^11^KlimkoN.^12^LagrouK.^13^Lass-FlörlC.^14^GovicY. Le^15^MaertensJ.^16^OstojicA.^17^PrattesJ.^18^RacilZ.^19^SharpeA. Reséndiz^16^SchalkE.^20^StanzaniM.^21^SteinmannJ.^22^MelchersW.^23^VehreschildM.J.G.T.^24^VerweijP.E.^23^1Department I of Internal Medicine, Ecmm Excellence Centre of Medical Mycology, Cologne Excellence Cluster On Cellular Stress Responses In Aging-associated Diseases (cecad), University Hospital Cologne, Cologne, Germany2Department I of Internal Medicine, Ecmm Excellence Centre of Medical Mycology, Cologne Excellence Cluster On Cellular Stress Responses In Aging-associated Diseases (cecad), German Centre For Infection Research, Partner Site Bonn-cologne, Clinical Trials C, University Hospital Cologne, Cologne, Germany3Department I of Internal Medicine, University Hospital of Cologne, Cologne, Germany4Department of Medical Microbiology and Infectious Diseases, Excellence Center For Medical Mycology (ecmm), Canisius Wilhelmina Hospital, Nijmegen, Netherlands5Department I For Internal Medicine, Excellence Center For Medical Mycology (ecmm), University of Cologne, Cologne, Germany6Karolinska University Hospital, Stockholm, Sweden7Mannheim University Hospital, Heidelberg University, Mannheim, Germany8University Hospital Dresden, Dresden, Germany9Université Paris Diderot, APHP Saint-Louis Hospital, Paris, France10Cepr Inserm U1100, Université de Tours, Tours, France11Hospital Universitario La Paz, Madrid, Spain12Department of Clinical Mycology, Allergology and Immunology, North-Western State Medical University n.a. I.I. Mechnikov, Saint-Petersburg, Russian Federation13Clinical Bacteriology and Mycology, Katholieke Universiteit Leuven, Leuven, Belgium14Div Hygiene & Med. Microbiology, Med. Univ. Innsbruck, Innsbruck, Austria15Groupe d’Etude des Interactions Hôte Pathogène (GEIHP), Université d’Angers, Angers Cedex, France16KU Leuven, Leuven, Belgium17University Hospital Centre Zagreb, Zagreb, Croatia18Section of Infectious Diseases and Tropical Medicine, Medical University of Graz, Graz, Austria19Department of Internal Medicine - Hematology and Oncology, University Hospital Brno, Brno, Czech Republic20Otto-von-Guericke University, Magdeburg, Germany21“Lorenzo e Ariosto Seràgnoli” S’Orsola-Malpighi Hospital, University of Bologna, Bologna, Italy22Institute of Clinical Hygiene, Medical Microbiology and Clinical Infectiology, Klinikum Nuernberg, Nuremberg, Germany23Medical Microbiology, RadboudUMC, Nijmegen, Netherlands24Uniklinikum Frankfurt, Med. Klinik II, Infektiologie, Frankfurt, Germany

**Objectives:** In recent years, survival of patients with invasive aspergillosis (IA) has improved mainly due to availability of extended spectrum triazoles. These advances are jeopardized by the emergence of azole resistance in *Aspergillus fumigatus*, the most common causative pathogen of IA. Despite several studies suggesting high probability of azole treatment failure in patients with azole-resistant isolates, the clinical implications of azole-resistant IA compared to azole-susceptible IA remain unclear. Thus, we seek to describe the epidemiology and determine the efficacy of antifungal therapy in patients with documented azole-resistant IA compared to azole-sensitive IA in patients with hematological malignancy.

**Methods:** In patients with hematological malignancies, cases of proven or probable IA (EORTC/MSG 2008) caused by *A. fumigatus* are registered. Retrospective data are documented, comprising demographics, diagnosis, treatment, response and outcome. Participating sites provided susceptibility results or isolates. Provided isolates were analyzed in a central laboratory.

**Results:** Since January 2018, 51 sites in 15 countries worldwide enrolled 154 cases diagnosed with IA between 2010 and 2019, of which 23 (14.9%) had azole-resistant IA. of 44 cases, the respective clinical fungal isolate was analyzed in the central laboratory. A mixed fungal infection was reported for 34 patients (22.1%), 1 (2.9%) in the azole-resistant group; most were related to non-*fumigatus Aspergillus* species (*n* = 12, 35.3%) and non-*Aspergillus* molds (*n* = 10, 29.4). Most patients were male (*n* = 98, 63.6%); 19 (82.6%) in the azole-resistant group, 79 (60.3%) in the azole-susceptible group. Age was documented in categories instead of the exact age. Median age group was 50-69 years in both groups (ranging from 7-11 to 70-89 years for azole-resistant cases, 1-12 months to 70-89 years for azole-susceptible cases). Underlying disease and survival are shown in the table. 

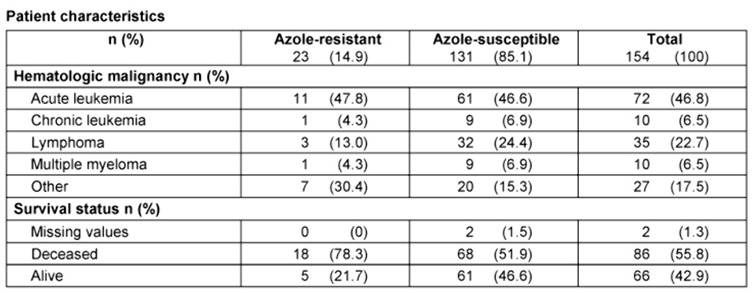



**Conclusion:** A worldwide network of investigators contributes to the CLARITY registry study. Completion of recruitment and subsequent data analysis are planned for 2019. Further sites may be added if azole resistant cases are encountered.

### P034. Sensitivity of isolated dermatophyte strains to antifungal drugs in the Russian Federation

ManoyanM.SokolovV.GurshevaA.GabuzyanN.PaninA.Veterinary Mycology, The All-Russian State Center for Quality of Animal Medicines and Feeds, Moscow, Russian Federation

**Objectives:** The spread of dermatophytosis is a veterinary problem; the spread of dermatophytosis pathogens in the human population is a social problem. In the Russian Federation immunobiological preparations, as well as external agents in the form of suspensions or shampoos are used for a treatment of animals dermatophytosis. As a rule, such agents contain azoles (clotrimazole, miconazole, enilconazole) or terbinafine as an active substance. They are often used unsystematically and can trigger the emergence of resistant strains. Recently, there have been increasing reports about dermatophytoses strains, resistant to antifungal drugs: *M. canis* strains resistant to terbinafine and azoles, and *T. rubrum* strains resistant to terbinafine.

**Methods:** For comparison, the strains of dermatophytes - *M. canis* and *T. mentagrophytes*, earlier collected and freshly isolated from cats and dogs, were chosen. Collection strains, stored in a lyophilized form, were isolated from animals between 1970 and 2000. For the study there were selected ten strains of *M. canis* and *T. mentagrophytes*. Freshly isolated strains were collected from animals between 2015 and 2018, they also were stored in the laboratory’s collection in a lyophilized form; we also selected ten strains of *M. canis* and *T. mentagrophytes*. The choice of antifungal drugs spectrum, for which MIC was determined, was dictated by the data on the use of antifungal drugs in the Russian Federation: for terbinafine there was evidence of resistant strains; ketoconazole and enilconazole were used for animal fungal infections treatment and prevention. Cultures were isolated on Saburo medium with chloramphenicol and cycloheximide, pure cultures for determining MIC were cultivated on Saburo medium without additives for 7 days at +28°C. A method based on EUCAST E.def 9.3.1 was used to determine the MIC (inoculum density was 1.0E+5 CFU/ml, the incubation time was 4days.

**Results:** MIC studies of terbinafine, ketoconazole and enilconazole for strains isolated between 1970 and 2000 showed following. One of ten *M. canis* strains was resistant to the action of terbinafine (MIC > 32 μg/ml), there was no detected any strain resistant to ketoconazole and enilconazole. None of the ten *T. mentagrophytes* strains was resistant to the action of terbinafine, ketoconazole or enilconazole. MIC studies of terbinafine, ketoconazole and enilconazole for strains isolated between 2015 and 2018 showed following. Four of the ten *M. canis* strains were resistant to the action of terbinafine (MIC > 32 μg/ml); one strain was resistant to the action of enilconazole (MIC > 16 μg/ml); and we did not find a ketoconazole resistant strain. Four of the ten *T. mentagrophytes* strains were resistant to the action of terbinafine (MIC > 32 μg/ml); one strain was resistant to ketoconazole (MIC > 16 µg/ml); 2 strains were resistant to the action of enilconazole (MIC > 16 µg/ml). We did not find strains of *M. canis* or *T. mentagrophytes*, resistant to two or three drugs.

**Conclusion:** The number of antifungal resistant dermatophyte strains was higher among those isolated for a later period. This may indicate the emergence of resistant dermatophytosis pathogens strains in the Russian Federation.

### P035. Evaluation of the in vitro susceptibility testing of dermatophytic isolates: preliminary comparison of four methods

MarkantonatouA.-M.SamarasK.ZachrouE.VyzantiadisT.-A.First Department of Microbiology, Medical School, Aristotle University of Thessaloniki, Thessaloniki, Greece

**Objectives:** Superficial infections caused by dermatophytes affect a high percentage of the population. Antifungal susceptibility testing (AST) of these pathogens may offer information about their susceptibility profiles, documentation of the appropriate treatment and reduction of the cost. However, there are two factors that could delay and affect the performance of the AST: the slow growth rate of these fungi and their poor sporulation. The proposed methods by the CLSI or the EUCAST are both laborious for the everyday routine. Though, there are alternative applications that propose the use of an inoculum consisting of a conidia-mycelium mixture or even from plain mycelia, as well as the use of resazurin in order to facilitate the reading. The aim of this study was to compare these approaches to the EUCAST method in order to evaluate their performance.

**Methods:** Three alternative methods of dermatophytic AST were compared to the EUCAST proposed methodology for conidia forming moulds. The methods under evaluation were a) a fragmented mycelia method, b) the EUCAST method with the addition of resazurin sodium salt solution and c) the fragmented mycelia method with the addition of resazurin sodium salt solution. The susceptibility of twenty dermatophytic isolates (8 *Trichophyton interdigitale*, 6 *T. rubrum* and 6 *M. canis*) was tested against the antifungal agents of griseofulvin, terbinafine, fluconazole and itraconazole.

**Results:** After the measurement of the MICs by all four different methods, the essential agreement between them was calculated in percentages. Data analysis revealed sufficient overall essential agreement of the methods with the addition of resazurin to the initial “uncoloured” ones (98.5% and 97.2% for the EUCAST and the fragmented mycelia method respectively). The fragmented mycelia method exhibited a relatively sufficient overall essential agreement to the EUCAST method (88.9%) but not a satisfactory correlation between the MIC values. The mean MICs (by the EUCAST method, in μg/mL) for the twenty isolates were 1.78 for griseofulvin, 0.034 for terbinafine, 25.2 for fluconazole and 0.57 for itraconazole.

**Conclusion:** The addition of resazurin sodium salt solution facilitates the reading and provides a more objective evaluation. The fragmented mycelia method could serve as an alternative in cases of poor or no sporulating dermatophytes.

### P037. Distribution of azole minimal inhibitory concentration (MIC) values and Erg11 amino acid substitutions among Candida auris isolates from different geographic clades

KordalewskaM.^1^HealeyK.R.^2^Jimenez-OrtigosaC.^1^SinghA.^3^WilliamsonB.^2^WilkA.^2^BerrioI.^4^ChowdharyA.^3^PerlinD.S.^1^1Center For Discovery and Innovation, Hackensack Meridian Health, Nutley, United States of America2Department of Biology, William Paterson University, Wayne, United States of America3Department of Medical Mycology, Vallabhbhai Patel Chest Institute, University of Delhi, Delhi, India4Hospital General de Medellín “Luz Castro de Gutiérrez” ESE, Medellin, Colombia

**Objectives:** *Candida auris* isolates are often characterized with reduced susceptibility to fluconazole (FLC) and other azole class drugs: voriconazole (VRC), posaconazole (POS), itraconazole (ITC), and isavuconazole (IVC). Such a situation is extremely worrying, since azoles are a mainstay in the treatment of *Candida* infections and antifungals other than fluconazole might be unavailable in resource-limited countries. Moreover, no antifungal clinical breakpoints are available for *C. auris*. CDC provides guidance for *C. auris* MIC interpretation, based on information gathered for related *Candida* species and expert opinion. Tentative epidemiological cut off values (ECVs) were proposed only for *C. auris* isolates from India. The aim of this study was to analyze distribution of azole minimal inhibitory concentration (MIC) values for isolates belonging to different geographic clades together with the data regarding molecular resistance determinants.

**Methods:** A total of 108 *C. auris* isolates representing four geographic clades (I – South Asian; II – East Asian; III – South African; IV – South American) were investigated in this study. Antifungal susceptibility testing (AFST) with azoles (FLC, ITC, IVC, POS, VRC) was performed in accordance with CLSI document M27-A3. The *ERG11* gene, encoding the target for azole antifungal drugs, was amplified and sequenced. All identified *ERG11* alleles were cloned onto a low-copy vector (pRS416) and expressed within a haploid strain of *Saccharomyces cerevisiae* (BY4741). Azole susceptibilities were then determined for the transformed strains of *S. cerevisiae*.

**Results:** The results of *C. auris* AFST and *ERG11* sequencing are presented in Table 1. Expression of *ERG11* alleles in *S. cerevisiae* revealed that triazole resistance was induced only upon expression of pCauErg11-V125A/F126L, pCauErg11-Y132F, and pCauErg11-K143R, but not pCauErg11-WT, pCauErg11-K177R/N335S/E343D, pCauErg11-I466M, or pCauErg11-Y501H.

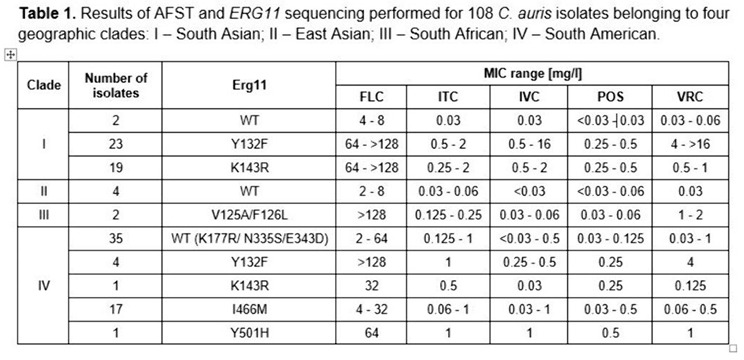



**Conclusion:** Differences in the distribution of MIC values and resistance-conferring *ERG11* mutations between isolates from different geographic clades underscore the need for an extreme caution when categorizing isolates as sensitive/resistant on a basis on AFST results. Only V125A/F126L, Y132F and K143R Erg11 amino acid substitutions identified in *C. auris* isolates directly mediate resistance and may be used as diagnostic markers for *C. auris* azole resistance. Mechanisms other than *ERG11* mutations may also contribute to reduced azole susceptibility in *C. auris*, although this remains to be determined.

### P038. To study the profile of conventional and newer antifungal agents against dermatophytes in a tertiary care hospital in South India

KindoA.J.^1^VeenaH.^1^SubramanianA.^2^KrishnanA.^2^1Microbiology, Sri Ramachandra Medical College and Research Institute, SRIHER, Chennai, India2Dermatology, Sri Ramachandra Medical College and Research Institute, SRIHER, Chennai, India

**Objectives:** 1.Speciation of dermatophytes from the clinical isolates 2.To study the antimicrobial pattern of frequently used antifungal agents 3.To compare the antifungal profile with the newer antifungal agents

**Methods:** Clinically diagnosed dermatophytic patients were sampled for microscopy and culture, grown dermatophytes were subjected to phenotypic identification by conventional method. Antifungal susceptibility testing was done by broth microdilution method according to CLSI M38-A2 document guidelines. *Trichophyton mentagrophytes* ATCC 4439 was included in each run of susceptibility testing as a quality control. Final drug concentration in the microdilution plates ranged from 64 to 0.25 µg/ml for fluconazole and Amphotericin B and from 16 to 0.06 µg/ml for Itraconazole, Voriconazole, Griesofulvin, Terbinafine, Sertaconazole, Luliconazole, Fenticonazole tested.

**Results:**

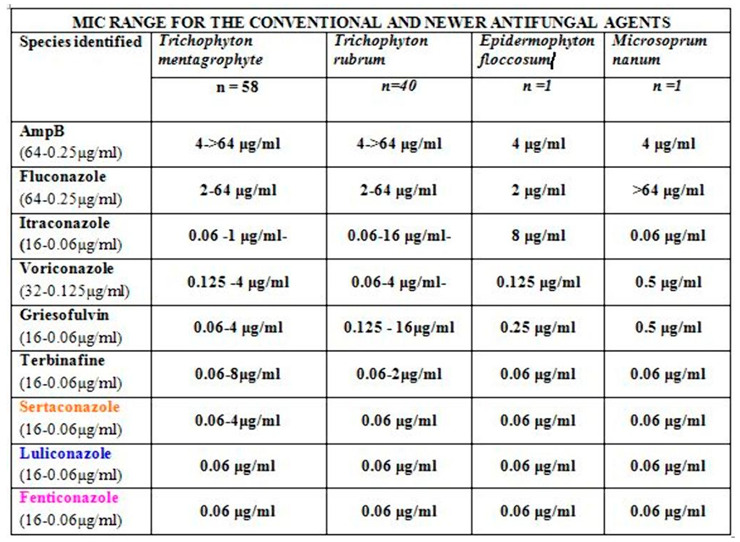



Out of the 100 clinical isolates tested, 58 of them were identified as *Trichophyton mentagrophytes*, 40 were *Trichophyton rubrum.* and the remaining two isolates were identified as *Epidermophyton Floccosum* and *Microsoprum nanum* Antifungal susceptibility testing was carried out for 100 isolates of *Trichophyton* species against 9 antifungal agents. All isolates showed detectable growth after 4-5days of incubation. MICS for *Trichophyton mentagrophytes* ATCC 4439 were within the established range.

**Conclusion:** In this study *Trichophyton mentagrophytes* was the most common cause of dermatophytosis. The most challenging task is not the diagnosis but the treatment, compliance and recurrence. Since it requires a long term treatment, the management with appropriate and responsive drug is the need of the hour. Currently this study suggests that the overall activity of Luliconazole, Fenticonazole, Sertaconazole, as topical agent and Itraconazole, Voriconazole (systemic) are significantly effective against dermatophytes. Current solution to this could be to maintain skin hygiene, prudent use of antifungals appropriate dosing and duration, performing antifungal susceptibility testing, usage of conventional drugs or topical keratolytic agents in combination with newer drugs in appropriate dosages and combination therapy.

### P039. Terbinafine resistant Trichophyton mentagrophytes genotype VIII, Indian type, isolated in Finland

JärvH.^1^UhrlassS.^2^SimkinT.^1^NenoffP.^2^RamirezE. Alvarado^3^ChryssanthouE.^3^MonodM.^4^1SYNLAB Estonia, Tallinn, Estonia2Laboratory For Medical Microbiology, Mölbis, Mölbis, Germany3Clinical Microbiology, Karolinska University Hospital, Stockholm, Sweden4Department of Dermatology, Lausanne University Hospital, Chexbres, Switzerland

**Objectives:** Objective We are reporting first four isolates of *Trichophyton mentagrophytes* representing novel Indian genotype VIII in Finland potentially resistant to terbinafine. Our study points out the need for antifungal susceptibility testing of dermatophytes as a new issue in microbiology laboratories. The roles of classical mycological culture vs DNA-based diagnostics for laboratory diagnostics of dermatophytosis will be discussed.

**Methods:** Primary fungal cultures were made on Sabouraud dextrose and 2% malt extract agar. Identification of fungal isolates was made by micromorphological appearance in SYNLAB laboratory in Estonia and by sequencing the ITS region of rDNA and TEF1-alpha gene in clinical microbiology laboratory in Mölbis, Germany. The genotyping of squalene oxidase gene was made in dermatology department of Vaudoise, Lausanne, Switzerland. The minimal inhibitory concentration (MIC) to terbinafine was determined by broth microdilution method in microbiology laboratory of Karolinska Hospital, Sweden. MIC was detected spectrophotometrically after 5 days incubation at 28 °C.

**Results:** We describe four cases of extensive *tinea cutis glabrae* caused by *T. mentagropytes*. All cases were diagnosed from January to April of 2019 and occurred in geographically distinct regions of Finland. Three of four of *T. mentagrophytes* sub-type VIII (Indian sybtype) strains have high MIC values of terbinafine from 4 mg/L to >8 mg/L.

**Conclusion:** Dermatophytosis has taken epidemic dimensions in India during the last 5 to 6 years. Unusual clinical presentation of dermatophytosis and corticosteroid modified lesions are making diagnosis challenging. Indian experts panel ECTODERM India has published new treatment guidelines in 2018. Nenoff *et al* have demonstrated in a recently published study from different geographical locations from India that 93.25% of the dermatophytes isolated from patients suffering from dermatophytosis correspond to a special genotype (VIII) of *T. mentagrophytes*. Singh with co-workers have published data about alarming terbinafine resistance of *T. interdigitale* strains in India. *Trichophyton* spp isolates with Indian origin have demonstrated high MICs to terbinafine, up to 32 mg/L, and also to azoles. Terbinafine is considered to be the most effective drug for treatment of *Trichophyton* spp infections. In general, we know that pathogens and resistant strains follow the human migration routes. The lack of data about presence of this novel genotype in European countries is because most clinical laboratories do not use multilocus sequencing for routine identification of dermatophytes. SYNLAB Estonia uses classical culture-based diagnostics and DNA-based diagnostics (in house PCR) for dermatophytes, handling approx. 10000 sample per year. We are not able to identify 1/5 of dermatophytes on species level. Most of commercial diagnostic DNA panels for dermatophytes do not discriminate *T. mentagrophytes* and *T. interdigitale*. In the light of new data diagnostic laboratories need to put more effort to identify all dermatophytes on species level by molecular methods. It will help to map the changing epidemiological situation of dermatophytosis in Europe.

### P040. An emerging clone of Candida tropicalis bloodstream isolates with ERG11 gene mutations and overexpression conferring reduced activity of isavuconazole

ChenP.-Y.ChuangY.-C.WuU.-I.SunH.-Y.WangJ.-T.ShengW.-H.ChenY.-C.ChangS.-C.National Taiwan University Hospital, Taipei, Taiwan

**Objectives:** In Asia, in vitro activity of isavuconazole (ISA) against clinical *Candida* isolates are seldomly reported, especially a rising concern that azole-resistant *Candida tropicalis* is common (≥10%) in this region. Also, few studies characterized molecular mechanisms of reduced isavuconazole activity against *C. tropicalis*.

**Methods:** Sixty-four non-duplicated *C. tropicalis* bloodstream isolates were prospectively collected in National Taiwan University Hospital in 2017. In vitro susceptibility to antifungal agents was performed by using the microdilution colorimetric Sensititre YeastOne panel and the results were interpreted by the Clinical and Laboratory Standards Institute. ISA MICs were determined using the EUCAST E.def 7.3.1 method. Wild-type upper limit (wtUL) was defined as two two-fold dilutions above the MIC_50_. Isolates with reduced ISA activity (RIA) were defined as their ISA MICs above wtUL. The *ERG11* gene was sequenced for all isolates, and the results were compared to the *ERG11* wild-type sequence from *C. tropicalis* reference strain ATCC 750 (Genbank accession M23673). Quantitative realtime reverse transcription-polymerase chain reaction (*ERG11*, *CDR1*, and *MDR1*) were performed. Multilocus sequencing types were analyzed by the eBURST to determine putative relationships between isolates.

**Results:** Among sixty-four *C. tropicalis* bloodstream isolates, the resistant/ non-wild type rates of fluconazole, voriconazole, posaconazole or itraconazole were 21.5%, 30.7%, 64.6%, and 13.9%, respectively. The proportions of RIA was 18.8% (*n* = 12). ISA MICs correlated with other azoles MICs by calculating with nonlinear regression fits. The *R*^2^ values were in the following orders: posaconazole (0.74) < itraconazole (0.82) < voriconazole (0.86) < fluconazole (0.88). The mean percentage of ISA trailing was 27.4%. A total of 8 nucleotide mutations were detected in *ERG11* gene of all isolates. Only 2 (A395T/W and C461T/Y) belonged to missense mutations resulting in amino acid substitutions (Y132F and S154F). There were fourteen isolates with one or two these mutations all resistant to fluconazole. Twelve of them had RIA, all of which belonging to the same clone. Gene relative expression levels among three groups (isolates with low and high ISA trailing, and those with RIA) are shown Figure. Only *ERG11* expression levels demonstrated significant difference (*P* < 0.001), and neither did *CDR1* nor *MDR1* (*P* > 0.05). Those with RIA showed the highest mean *ERG11* relative expression level (3.51 ± 0.59 [S.D.]) with statistically significance, while the mean *ERG11* levels between high and low ISA trailing group were not statistically different (1.08 ± 0.69 and 0.93 ± 0.66, respectively).

**Figure**

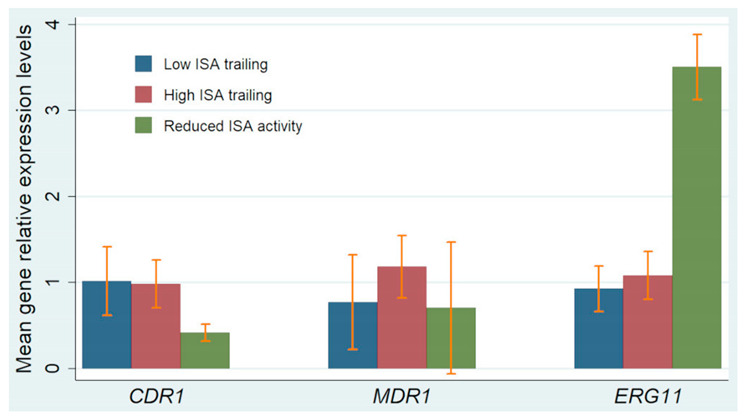



**Conclusion:** *C. tropicalis* bloodstream isolates in our report showed an emerging clone with pan-azole resistance, including RIA. Fluconazole MICs best correlated to ISA MICs. Combined *ERG11* gene missense mutations and *ERG11* overexpression conferred RIA.

### P041. Comparative genomics of Aspergillus fumigatus and the influence of agriculture on ecology and azole resistance

BarberA.E.^1^,^2^,^3^BornJ.^4^Sae-OngT.^2^SchliebnerI.^4^KangK.^2^WaltherG.^2^,^3^PanagiotouG.^2^DeisingH.B.^4^KurzaiO.^1^1University of Wuerzburg, Wuerzburg, Germany2Leibniz HKI Jena, Jena, Germany3National Reference Center for Invasive Fungal Infections, Jena, Germany4Martin Luther University Haale-Wittenberg, Halle (Saale), Germany

**Objectives:** *Aspergillus fumigatus* is an environmentally ubiquitous saprophyte capable of causing life-threatening invasive infections and allergic bronchopulmonary disease and current management strategies rely on the azoles. Unfortunately, over the last decade there has been a global emergence in resistance to the azoles in *A. fumigatus* and the dominant resistance mechanism is of environmental origin, suggesting that resistance is emerging through a selective pressure applied by the widespread usage of azoles in agriculture.

**Methods:** To examine the link between the use of azoles in agriculture and the emergence of clinical resistance, systematic soil sampling was performed over a three year period on seven conventional agricultural sites in Germany utilizing azole fungicides and on six organic sites withholding these compounds. Conventional sites were evaluated both before and after azole application. We also performed WGS on 250 environmental and 50 clinical isolates.

**Results:** In total, 2875 soil samples were analyzed between 2016 and 2018. We observed a high degree of variation in the abundance of *A. fumigatus* across sites, however, fields with high *A. fumigatus* density tended to be consistently so from year to year. Strikingly, we observed a significant reduction in the abundance of *A. fumigatus* on conventional fields following azole treatment – a finding that was not repeated on an organic agriculture control field – indicating that the application of azoles is imposing a bottleneck on *A. fumigatus*. We detected a low overall resistance frequency amongst agricultural isolates, with only 1-5% of isolates from 2016-2018 showing medical azole resistance – a rate lower than the 15-20% resistance frequency observed by the German National Reference Center for Invasive Fungal Infections during the same time period. Susceptibility to commonly-applied agricultural azoles was also assessed and isolates resistant to medical azoles almost always had elevated MICs to these agricultural azoles, suggesting cross resistance. Importantly, we observed an increased tolerance to both agricultural and medical azoles in the samples taken after the growing season and application of azoles. At the genome level, isolates from different regions and types of agriculture did not cluster separately, indicating a lack of population structure in the fungus. Comparison of environmental isolates with clinical isolates revealed several subgroups present in the environment that were not represented among clinical samples. We also observed differences in the distribution of resistance mutations between environmental and clinical isolates. Resistant environmental isolates were exclusively either wild type at the *cyp51a* loci or carried the environmentally-derived TR34/L98H mutation. Clinical isolates, in contrast, showed a much wider range mutations, including substitutions at positions F219, M220, S297, F495, and G448.Ongoing work is focused on defining fungal determinants critical for human infection as well as genetic mechanisms associated with *cyp51a*-independent azole resistance.

**Conclusion:** We observed a marked reduction in *A. fumigatus* fields following azole application and increased azole tolerance after the growing season and azole exposure. No population structure was observed among environmental isolates, but whole genome phylogeny identified genetic backgrounds enriched for among clinical isolates.

### P042. MIC distributions for amphotericin B, fluconazole, itraconazole, voriconazole, flucytosine and anidulafungin and thirty-five uncommon pathogenic yeast species from the UK determined using the CLSI broth microdilution method

BormanA.MullerJ.Walsh-QuantickJ.SzekelyA.PattersonZ.PalmerM.FraserM.JohnsonE.Public Health England UK National Mycology Reference Laboratory, Bristol, United Kingdom

**Objectives:** Epidemiological cut-off values and clinical interpretive breakpoints have been developed for a number of antifungal agents with the most common *Candida* species which account for the majority of infections due to pathogenic yeasts species. However, less common species for which susceptibility data are limited are increasingly reported in high risk patients and breakthrough infections.

**Methods:** The UK National Mycology Reference Laboratory performs routine antifungal susceptibility testing of clinical isolates of pathogenic yeast submitted from across the United Kingdom. Between 2002 and 2016, in excess of 32 000 isolates representing 94 different yeast species were referred to the laboratory. Here we present antifungal susceptibility profiles generated over this 15 year period for amphotericin B, fluconazole, voriconazole, itraconazole, anidulafungin and flucytosine against 35 species of uncommon yeast. Minimum inhibitory concentrations (MICs) were obtained using CLSI broth microdilution methodologies, and MIC data was interpreted against epidemiological cut-off values and clinical breakpoints developed with *Candida albicans*, in order to identify species with unusually skewed MIC distributions that potentially indicate resistance.

**Results:** Potential resistance to at least one antifungal agent (>10% of isolates with MICs greater than the epidemiological cut-off or clinical breakpoint) was evidenced for 29/35 species examined here. Four species exhibited elevated MICs with all of the triazole antifungal drugs against which they were tested, and 21 species exhibited antifungal resistance to agents from at least 2 different classes of antifungal agent.

**Conclusion:** The current study highlights the importance of rapid and accurate yeast identification, and provides data to aid clinicians in deciding which antifungal regimens are appropriate when confronted with infections with rarer yeasts.

### P043. Case-series of Candida albicans and glabrata echinocandin-resistant candidemia in Switzerland

CosteA.T.^1^LiJ.^1^KhannaN.^2^GoldenbergerD.^3^GarzoniC.^4^ZhenderZ.^5^BoggianK.^6^NeofytosD.^7^RiatA.^8^BachmannD.^1^SanglardD.^1^LamothF.^9^,^10^1Microbiology Institute, University Hospital Lausanne, University of Lausanne, Lausanne, Switzerland2Division of Infectious Diseases and Hospital Epidemiology, University Hospital of Basel, Basel, Switzerland3University Hospital Basel, Clinical Microbiology Laboratory Medicine, Basel, Switzerland4Riva Caccia 1b, Lugano, Switzerland5SYNLAB Suisse SA, Bioggio, Switzerland6Department of Infectious Diseases, Cantonal Hospital of Saint Gallen, Saint Gallen, Switzerland7Infectious Diseases, University Hospital of Geneva, Geneva, Switzerland8Geneva University Hospitals, Service of Laboratory Medicine, Geneva, Switzerland9Microbiology Institute, University Hospital Lausanne, Lausanne, Switzerland10Infectious Diseases Service, CHUV, Lausanne, Switzerland

**Objectives:** Echinocandin drugs (anidulafungin, caspofungin, micafungin) represent first-line therapy of candidemia. Echinocandin resistance, due to hotspot mutations in the target *FKS* gene, has emerged among *C. glabrata* and *C. albicans*, which is associated with poor outcome. Therefore, national surveillance programs of echinocandin resistance are mandatory. The aims of this study were: i) to assess the epidemiology of echinocandin resistance among *C. albicans*/*glabrata* in Switzerland, ii) to describe the characteristics of candidemic episodes due to echinocandin-resistant isolates, and iii) to assess the efficacy of high-dose echinocandin against echinocandin-resistant *C. albicans* in an invertebrate model of infection.

**Methods:** Cases of candidemia attributed to *C. albicans*/*glabrata* resistant to echinocandins (CLSI criteria) were identified by: i) screening of a collection of *Candida* bloodstream isolates from the Fungal Infection Network of Switzerland (FUNGINOS) including all candidemic episodes from 26 hospitals in Switzerland from 2004 to 2013, and ii) screening of the microbiology databases of the most important university hospitals from 2014 to 2018. Echinocandin-resistant *Candida* isolates were sent to the reference laboratory for antifungal susceptibility testing (CLSI protocol) and sequencing of *FKS* hotspots. Clinical data of candidemia were collected. In order to assess a possible efficacy of high-dose echinocandins against these isolates, we tested the efficacy of escalating anidulafungin doses in a *Galleria mellonella* model of invasive candidiasis with a *C. albicans FKS* mutant (S645P) compared to the wild-type SC5314 strain.

**Results:** Seven cases of candidemia due to echinocandin-resistant *C. albicans*/*glabrata* were identified: 2 from the 2004-2013 cohort (incidence 0.1%) and 5 from the microbiological database screening from 2014 to 2018. Four infections were due to *C. albicans* and 3 to *C. glabrata*. Candidemia originated from urine (2 cases), intravascular catheter (2), gastro-intestinal tract (1) and primary bloodstream infection (1). All patients received previous echinocandin therapy with a median duration of 18 days (range 11-60). Overall mortality was 43%. One patient (*C. glabrata* infection) was cured by caspofungin therapy despite *in vitro* resistance. Minimal inhibitory concentrations (MIC) ranges for anidulafungin, caspofungin and micafungin were: 2-4, 1->16 and 1-4 µg/mL, respectively. The following hotspot mutations were identified in *C. albicans*: S645P (*n* = 3) and R1361G (*n* = 1). Among *C. glabrata*, 2 isolates displayed the S663P mutation in *FKS2*, while no hotspot *FKS* mutation was recovered from one pan-echinocandin resistant isolate. Because anidulafungin displayed some *in vitro* activity against *C. albicans* S645P mutant (MIC 2 µg/mL), we assessed its *in vivo* activity in a *Galleria mellonella* model of infection (Figure 1). While a dose-dependent response was observed in *C. albicans* wild-type strain-infected larvae, no effect was observed against the *C. albicans FKS* mutant despite high anidulafungin doses (12 mg/kg). 

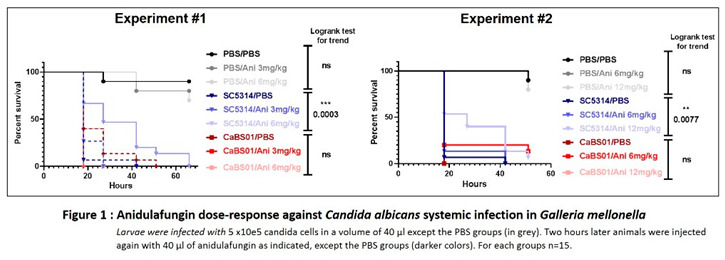



**Conclusion:** Our analysis suggests an increase in echinocandin resistance among *C. albicans/glabrata* in Switzerland with 5 cases identified within the last 5 years, while only two cases were reported in a cohort including all candidemias from 2004 to 2013. Previous echinocandin exposure was observed in all cases. Our invertebrate model suggests that high-dose echinocandins are ineffective against *C. albicans FKS* mutants despite some level of *in vitro* activity.

### P044. Aspergillus isolates from patients with chronic pulmonary aspergillosis mycologically and clinically resistant to azole antifungals are sensitive to Ibrexafungerp (SCY-078)

Rautemaa-RichardsonR.^1^,^2^MooreC.^1^,^2^RawsonK.^2^Novak-FrazerL.^1^,^2^AnguloD.^3^BaratS.^3^RichardsonM.D.^1^,^2^1Division of Infection, Immunity and Respiratory Medicine, University of Manchester, Manchester, United Kingdom2Mycology Reference Centre, Excellence Center For Medical Mycology (ECMM), Wythenshawe Hospital, Manchester University NHS Foundation Trust, Manchester, United Kingdom3Scynexis Inc, New Jersey, United States of America

**Objectives:** Ibrexafungerp (formerly MK-3118, SCY-078) is a novel and structurally distinct triterpenoid glucan synthase inhibitor whose oral availability and efficacy has already been demonstrated against a wide spectrum of *Candida* species. Currently the drug is being evaluated for efficacy in invasive and chronic pulmonary aspergillosis. Ibrexafungerp has been shown to be effective *in vitro* and *in vivo* against a broad range of *Aspergillus* species, including drug-resistant strains. *In vitro* activity is an important efficacy indicator of therapeutic success or failure. The objective of this study was to determine the activity of Ibrexafungerp against a panel of *Aspergillus* strains isolated from the respiratory tract of patients unresponsive to or failing azole therapy, and which were previously shown to be resistant to one or more azole antifungals (itraconazole, voriconazole, posaconazole and isavuconazole) using the EUCAST E.Def 10.1 standard.

**Methods:** Patients failing azole therapy for chronic pulmonary aspergillosis were identified in the weekly departmental multi-disciplinary team meetings and their isolates selected for Ibrexafungerp sensitivity testing. Twenty-two *Aspergillus fumigatus* complex, three *A. flavus* complex and one *A. niger* complex strains with varying degrees of resistance to one or more azole antifungals were tested by measuring the minimum effective concentration (MEC) for Ibrexafungerp following the EUCAST E.Def 10.1 standard. Where required, isolates were sequenced (using primers to the internal transcribed spacers (ITS), beta-tubulin (bt) and calmodulin (cal) genes) to identify to the species level.

**Results:** The Ibrexafungerp MEC range for the 26 *Aspergillus* spp. isolates was 0.008 to 0.25 mg/l. Isolates that were deemed to be resistant to all azoles had an Ibrexafungerp MEC of 0.015 to 0.25 mg/l. One isolate that was resistant to all azoles and amphotericin B was sensitive to Ibrexafungerp (MEC 0.125 mg/l).

**Conclusion:** The exquisite *in vitro* activity of Ibrexafungerp against *Aspergillus fumigatus*, *A. flavus* and *A. niger* isolated from patients with chronic pulmonary aspergillosis suggests that this new compound has potential for treating patients with this condition and similar manifestations of pulmonary aspergillosis.

### P045. Analysis of the susceptibility pattern among Aspergillus isolates against the commonly used Antifungal agents in a tertiary care centre in South India.

FathimaU. Almas^1^KindoA.J.^1^KumarS. Prasanna^2^ThirunarayanM.^3^SreeV. Lakshmi^4^1Microbiology, Sri Ramachandra Institute of Higher Education and Research, Chennai, India2Ent, Sri Ramachandra Institute of Higher Education and Research, Chennai, India3Microbiology, Apollo Hospitals, Chennai, India4Microbiology, Apollo Speciality Hospital, Chennai, India

**Objectives:** To do *in vitro* susceptibility testing and analyse the difference in the Antifungal susceptibility profile of *Aspergillus* isolates by Broth Microdilution method.

**Methods:** *Aspergillus* grown from various clinical samples (Ear swab, Bronchial wash, Endotracheal Aspiration, Paranasal sinus, BAL, Sputum) was subcultured on Sabouraud’s Dextrose Agar/Oat meal Agar. Tease mount/Slide culture was done to study the morphological features of the hyphae, size, shape and arrangement of the conidia. Antifungal susceptibility testing(AFST) was done according to CLSI guidelines M38-A2 2008 documented reference method for Broth Microdilution Antifungal susceptibility of Filamentous fungi. Antifungal stock solutions of AmphotericinB, Voriconazole, Posaconazole, Itraconazole and Caspofungin was prepared in di-methyl sulfoxide and stored at -70^0^ C. RPMI medium was prepared, adjusted to pH 7 and filter sterilized. Sterility was checked and stored at 4^0^ C. Dilution of the stock solution was done as per the guidelines. Inoculum was prepared by adjusting conidial suspension in sterile distilled water to 0.5 McFarland Turbidity standard; to 100 µl of drug solution, 100 µl of inoculum in RPMI 1640 medium was added. Test micro-titre plate was incubated for 48hrs at 35^0^ C. Growth and sterility control wells were included in each test. AFST-READING RESULT: For AmphotericinB, Voriconazole, Posaconazole, Itraconazole - MIC is the first well without visible growth. For Echinocandins, eg. Caspofungin- MEC is the point (lowest value) where a visible change in growth as compared to the positive growth is noted.

**Results:** A total of 30 *Aspergillus* isolates were collected-*Aspergillus niger*(14), *Aspergillus flavus*(9), *Aspergillus fumigatus*(6) & *Aspergillus terreus*(1). The result was compiled based on mean MIC values of 21 isolates are as follows. 

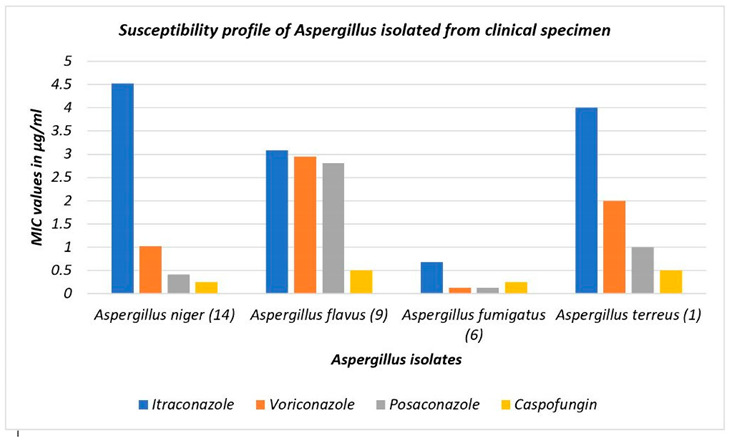



The MIC values of other isolates and Amphotericin B is yet to be completed.

**Conclusion:** The common *Aspergillus* species was *Aspergillus niger* which was obtained from Ear swab. The susceptibility profile showed high MIC for Itraconazole in case of *Aspergillus niger* and high MIC for Itraconazole, Voriconazole and Posaconazole in case of *Aspergillus flavus*. For *Aspergillus fumigatus* no such high MIC was recorded. In case of *Aspergillus terreus*, being resistant in nature the low MIC is recorded only with Caspofungin. Due to less susceptibility of *Aspergillus* to the common Antifungal agents, it is important to perform antifungal susceptibility testing to reduce the morbidity and mortality in patients especially with Invasive Aspergillosis.

### P046. CRISPR-Cas9 as a tool box to investigate antifungal resistance

MorioF.^1^LombardiL.^2^BinderU.^3^LogeC.^1^RobertE.^1^Lass-FlörlC.^3^ButlerG.^2^PapeP. Le^1^1Ea1155 Iicimed, Nantes University Hospital, NANTES, France2School of Biomolecular and Biomedical Science, Conway Institute, University College Dublin, Dublin, Ireland3Department of Hygiene, Microbiology and Public Health, Division of Hygiene and Medical Microbiology, Medical University Innsbruck, Innsbruck, Austria

**Objectives:** Antifungal resistance in human pathogenic fungi is increasingly reported and a public health issue. Deciphering the mechanisms underlying drug resistance strongly relies on genetic manipulation techniques such as generating mutant strains carrying specific mutations or gene deletions. However, these processes have often been time-consuming and cumbersome in fungi. Over the last decade, the groundbreaking discovery of Clustered Regularly Interspaced Short Palindromic Repeats and the associate protein Cas9 (CRISPR-Cas9) revolutionized genome editing. We evaluated the potential of a CRISPR-Cas9 method, as a tool to perform precise genome editing of the *ERG11* gene in the context of azole resistance.

**Methods:** A new plasmid-based and marker-less CRISPR-Cas9 genome-editing method, recently developed for *Candida parapsilosis* (1), was used to investigate the potential contribution to azole resistance of two different single nucleotide polymorphisms (G1126A and G1372A), leading to amino acid substitutions (L376I and G458S, respectively) in the *ERG11* gene, identified in an azole-resistant clinical isolate of *Candida orthopsilosis*. These mutations were introduced by homology directed recombination (HDR) using a donor template into a fluconazole-susceptible *C. orthopsilosis* background isolate. *In vitro* susceptibility of the transformants displaying the expected mutations (two clones for each engineered mutation) to azoles was then performed.

**Results:** This approach allowed us to engineer both mutations in the fluconazole-susceptible background isolate. Though engineering L376I was straightforward, editing G458S required the addition of silent mutations within the core of the Cas9 recognition site to prevent Cas9 from recutting again. Eventually, *in vitro* susceptibility testing of the corresponding transformants demonstrated that G458S, but not L376I, confers resistance to fluconazole (MIC increased by 8-fold by introducing the homozygous mutation) and also affected voriconazole susceptibility. Importantly, after 3 passages on YPD at 30°C, transformants were not resistant to nourseothricin (selection marker) anymore, showing the rapid loss of the plasmid containing all the CRISPR-Cas9 components, thus proving that it did not integrate in the genome.

**Conclusion:** Our work supports the contribution of G458S but not L376I in fluconazole and voriconazole resistance in *C. orthopsilosis* (2). Besides these findings, this study highlights CRISPR-Cas9 genome editing as a powerful tool to streamline the study of antifungal resistance in human pathogenic fungi. Though critical points should be considered (e.g. HDR drops with cut-to-mutation distance), this approach paves the way for an easier screening approach of any SNP, occurring in any gene suspected to be involved in antifungal resistance. 1-Lombardi *et al*., Plasmid-Based CRISPR-Cas9 Gene Editing in Multiple *Candida* Species. **mSphere. 2019** Mar 13;4(2). pii: e00125-19. doi: 10.1128/mSphere.00125-19. 2-Morio *et al*., Precise genome editing using a CRISPR-Cas9 method highlights the role of CoERG11 amino acid substitutions in azole resistance in *Candida orthopsilosis*. **J Antimicrob Chemother. 2019** May 18. pii: dkz204. doi: 10.1093/jac/dkz204.

### P047. Changes in the lipid microenvironment of β-(1,3)-D-glucan synthase of Aspergillus fumigatus is a new clinically important echinocandin resistance mechanism

SatishS.^1^Jimenez-OrtigosaC.^1^ZhaoY.^1^LeeM.H.^1^DolgovE.^1^KrugerT.^2^ParkS.^1^DenningD.^3^KniemeyerO.^2^BrakhageA.^2^PerlinD.S.^1^1Center For Discovery and Innovation, Hackensack Meridian Health, Nutley, United States of America2Leibniz Institute for Natural Product Research and Infection Biology (HKI), Jena, Germany3University of Manchester, Manchester, United Kingdom

**Objectives:** *Aspergillus fumigatus* is a leading cause of opportunistic invasive fungal infections. Resistance to first-line triazole antifungals has led to therapy with echinocandin drugs. Recently, we identified several high Minimum Effective Concentration (MEC) *A. fumigatus* clinical isolates from patients failing echinocandin therapy. Echinocandin resistance in *Candida* is known to arise from amino acid substitutions in β-(1,3)-D-glucan synthase encoded by the *fks1* gene. Yet, these clinical isolates did not contain mutations in *fks1*, indicating an undefined resistance mechanism. The objective of this study was to explore a novel mechanism of echinocandin resistance independent of *fks1* mutations.

**Methods:** To explore this new mechanism, we used a lab-derived strain, RG101, which does not contain *fks1* mutations but is resistant to caspofungin (CAS). Inhibition of glucan synthase enzyme isolated from RG101 grown in the absence and presence of CAS was determined *in vitro* using radioactive substrate and IC_50_ (drug concentration required for 50% reduction of activity) values were measured. Post-translational modifications (PTMs) potentially leading to resistance was explored by evaluating enzyme derived from RG101 grown in the absence and presence of CAS (1 µg/mL) using nano LC-MS/MS. A comprehensive lipidomics analysis was also performed to compare lipid profiles of glucan synthase containing fractions derived from RG101 grown in the absence and presence of CAS (1 µg/mL) using LC-MS/MS. To measure Reactive Oxygen Species (ROS) levels induced by different echinocandins, a 2’,7’-dichlorofluorescin diacetate (DCFDA)-based fluorescence assay was used, and ROS levels measured after one hour of drug exposure.

**Results:** Glucan synthase isolated from RG101 was fully sensitive to echinocandins. Yet, exposure of RG101 to CAS during growth yielded a modified enzyme that was drug insensitive (4 log-orders) in kinetic inhibition assays, and this insensitivity was also observed for enzymes isolated from clinical isolates. To understand this alteration, we analyzed whole enzyme post-translational modifications, but found none linked to resistance. However, analysis of the lipid microenvironment of CAS-induced resistant enzyme revealed a prominent increase in abundance of dihydrosphingosine (DhSph) and phytosphingosine (PhSph). Exogenous addition of DhSph and PhSph to sensitive enzyme recapitulated the drug insensitivity of the CAS-derived enzyme. Further analysis demonstrated that CAS induces mitochondrial-derived ROS, and dampening ROS formation by antimycin A or thiourea eliminated drug-induced resistance.

**Conclusion:** We conclude that CAS-induces cellular stress, promoting formation of ROS, triggering an alteration in the composition of plasma membrane lipids within the local environment of glucan synthase, rendering it insensitive to echinocandins. We have discovered a new mechanism of resistance in *A. fumigatus* independent of the well-characterized *FKS* mutation mechanism observed in *Candida*. This study also identifies an off-target effect of CAS, ROS production in *A. fumigatus*, and integrates oxidative stress and sphingolipid alterations into a novel mechanism of resistance. This stress-induced response has implications for drug resistance and/or tolerance mechanisms in other fungal pathogens.

### P048. In vitro activity of fluconazole in combination with magnolol against Candida auris

De-La-FuenteI.^1^Matilla-GutiérrezA.^1^GuridiA.^1^SevillanoE.^1^PemánJ.^2^ErasoE.^1^QuindósG.^1^1Immunology, Microbiology and Parasitology, University of the Basque Country (UPV/EHU), Bilbao, Spain2Servicio De Microbiología, Hospital Universitario y Politécnico La Fe, Valencia, Spain

**Objectives:** *Candida auris* is an emerging pathogen that causes outbreaks worldwide. This fungus is responsible for severe infections such as invasive candidiasis that present a high overall mortality. Treatment of these infections suppose a clinical challenge due to the high levels of multidrug-resistance in *C. auris*, which makes the finding of new therapeutic alternatives an important issue. Combination of fluconazole with different natural compounds has shown to be effective against fluconazole-resistant *Candida* isolates. For this reason, the aim of this study was to assess the *in vitro* interaction between fluconazole and magnolol, a polyphenol extracted from *Magnolia officinalis*, or epigallocatechin gallate (EGCG), a polyphenol obtained from *Camellia sinensis*, against clinical isolates of *C. auris*.

**Methods:** We studied 21 *C. auris* isolates from different clinical specimens (seven from blood, seven from oropharynx and six from urine). *Candida parapsilosis* ATCC 22019 and *Candida krusei* ATCC 6258 were used as quality controls. Different fluconazole combinations with magnolol or EGCG were studied. Fluconazole concentrations were selected according to the minimum inhibitory concentration (MIC) determined by the European Committee on Antibiotic Susceptibility Testing (EUCAST) EDef 7.3.1 document. MIC was defined as the lowest drug concentration causing 50% growth reduction after 24 h of incubation. Magnolol and EGCG concentrations ranged from 0.125 to 64 μg/mL. The interaction between fluconazole and magnolol or EGCG were assessed by a checkerboard microdilution method. *In vitro* interactions of drug combinations were interpreted in terms of fractional inhibitory concentration index (FICI) as follows: FICI ≤ 0.5, synergistic; 0.5 < FICI ≤ 1, additive; 1 < FICI ≤ 4, indifferent and FICI > 4 antagonistic.

**Results:** Fluconazole MIC range against *C. auris* was 64 to > 64 μg/mL and magnolol MIC range 16 to > 64 μg/mL; however, combination of both drugs reduced the concentrations necessary for inducing 50% reduction in the growth of the isolates. The effect of the combination of fluconazole with magnolol against seven isolates (33.3%) was interpreted as additive. The effect against the other 14 isolates was interpreted as indifferent, although the fluconazole MICs decreased to 1-16 μg/mL. The combination of fluconazole with EGCG was indifferent against all isolates and did not have any influence in the MICs of fluconazole.

**Conclusion:** The combination of fluconazole with magnolol significantly reduced the fluconazole MICs against all of the *C. auris* isolates, showing additive effect in seven of them. The combination of fluconazole with EGCG showed indifferent effect. Funding: This work was co-supported by the Consejería de Educación, Universidades e Investigación (GIC15/78 IT-990-16) of Gobierno Vasco-Eusko Jaurlaritza.

### P049. Resistance to medical importance antifungals in environmental yeasts

ScrofernekerM.L.^1^PaganiD.^2^SouzaC. C. Tavares De^2^VieiraA. M.^3^HellwigA.H. Da Silva^4^RiosI. Da S.^3^HeidrichD.^4^FuentefriaA. M.^2^SilvaP. Valente Da^2^1Department of Microbiology, Immunology and Parasitology, Universidade Federal do Rio Grande do Sul, Porto Alegre, Brazil2Postgraduate Program In Agricultural and Environmental Microbiology, Universidade Federal do Rio Grande do Sul, Porto Alegre, Brazil3Federal University of Rio Grande do Sul, Porto Alegre, Brazil4Postgraduate Program In Medicine: Medical Sciences, Universidade Federal do Rio Grande do Sul, Porto Alegre, Brazil

**Objectives:** The aim of the study was to investigate the presence of potentially pathogenic yeasts from a lagoon in South America.

**Methods:** The lagoon was sampled in the summer of 2019. The water samples were inoculated in acidified YM agar medium (1% glucose, 0.5 % peptone, 0.3 % malt extract, 0.3 % yeast extract, 2 % agar) and incubated at 25 ºC for 30 days. Representative yeast colonies were selected and stored in YM medium with 30 % of glycerol at -20 ºC and in GYMP agar medium (2 % glucose, 2 % malt extract, 0.5 % yeast extract, 0.2 % monobasic sodium phosphate, 2 % agar) agar slants coated with mineral oil, maintained at 4 °C. We tested 8 environmental yeast strains. The antifungal susceptibility test was performed according to CLSI protocol, to select yeasts with possible resistance to clinical importance antifungals. The yeasts were tested in the antifungal concentrations established for *Candida* species against fluconazole, caspofungin and amphotericin B.

**Results:** Four isolates had MIC ≥ 64 μg/mL to fluconazole, none of them were susceptible to caspofungin, with MIC ≥ 2 μg/mL. To amphotericin B, six strains had MIC ≥ 1μg/mL. Three isolates were considered resistant to all tested antifungals, following the guidelines for *Candida* species.

**Conclusion:** The preliminary findings feature this lagoon environment as a possible resistance genes reservoir and fungal infection source. Further studies are needed to have a better understanding of this environment and the fungal resistance.

### P051. A natural substitution in FksB Hot Spot 2 of Saprochaete capitata probably related to reduced susceptibility to echinocandins

Menéndez-ManjónP.^1^Arrieta-AguirreI.^1^CuétaraM.S.^2^López-SoriaL.^3^García-RuizJ.C.^4^MoraguesM.D.^1^1Enfermería I, Universidad del País Vasco UPV/EHU, Leioa, Spain2Microbiologia, Hospital Severo Ochoa, Leganés, Madrid, Spain3Microbiologia, Hospital Universitario Cruces, Barakaldo, Spain4Biocruces Health Research Institute, Hospital Universitario Cruces, Barakaldo, Spain

**Objectives:** *Saprochaete capitata* is an emerging opportunistic pathogen reported in patients with haematological malignancies. Antifungal clinical breakpoints are not established and therapy remains empirical. *S. capitata* shows reduced susceptibility to echinocandins, which inhibit the enzyme β-1,3-D-glucan synthase (Fks) involved in the cell wall synthesis. Resistance to echinocandins is linked to mutations in two highly conserved regions of Fks, Hot Spot 1 (HS1) and Hot Spot 2 (HS2). We already reported a natural substitution F-to-L in Fks-HS1 that might be linked to this fungus low susceptibility to echinocandins (Arrieta-Aguirre et al., 2018). Mutations in HS2 have also been related to reduced susceptibility to echinocandins. Therefore, we resolved to sequence HS2 of *S. capitata* and search for mutations that could be related to echinocandin resistance.

**Methods:** DNA of 11 strains of *S. capitata* was extracted by mechanical cell disruption and purified with the DNeasy Plant Mini Kit (Qiagen). Since HS1 and HS2 are around 2500 bp apart, we applied a primer walking strategy of smaller fragments sequencing to locate them. With a specific primer from the HS1 sequence and a degenerate primer designed for the HS2 region, we obtained an amplicon for one of the strains that allowed us to design downstream specific primers that led us to HS2. Finally, we designed specific primers to sequence HS2 in the rest of the strains and the sequences were compared with other fungal species.

**Results:** The alignment of Fks-HS2 of *S. capitata* and other species revealed a natural substitution R-to-Q in the fourth position of HS2. The recently published genome of *S. capitata* reveals two Fks, FksA and B. Our sequence corresponds to FksB, which is more similar to Fks2 than Fks1 of other species. In *C. glabrata*, when R in the fourth position of Fks2-HS2 is modified it results in a deep decrease in the susceptibility to echinocandins (Jensen, 2016). Therefore, the natural substitution R-to-Q in FksB-HS2 of *S. capitata* could be related to resistance.

**Conclusion:** *S. capitata* has at least two natural substitutions (F-to-L in Fks-HS1 and R-to-Q in Fks-HS2) that may be involved in its reduced susceptibility to echinocandins. However, the effect of both substitutions still needs to be verified empirically. This work contributes to understanding the molecular basis of antifungal resistance of this fungus and may help to improve its treatment. Financial support: Grupos de Investigación Consolidados of the Basque Government (GIC15/103; IT913-16) and Grant FPI of the Basque Government to PM-M.

### P052. Fluconazole resistance in Candida tropicalis blood stream isolates collected from 16 Brazilian Medical Centers

MeloA.^1^MattaD. Da^1^FavarelloL.^1^OliveiraC.^2^SukiennikT.^3^TellesF.^4^NucciM.^5^ColomboA.^1^1Medicine, Federal University of São Paulo, São Paulo, Brazil2Medicine, Hospital Universitário do Oeste do Paraná, Cascavel, PR, Cascavel, Brazil3Medicine, Santa Casa de Porto Alegre, Porto Alegre, RS, Porto Alegre, Brazil4Medicine, Hospital de Clínicas da Universidade Federal do Paraná UFPR, Curitiba PR, Curitiba, Brazil5Medicine, Hospital Universitário da Universidade Federal do Rio de Janeiro, Rio de Janeiro RJ, Rio de Janeiro, Brazil

**Objectives:** *C. tropicalis* is highly prevalent among episodes of candidemia in Latin America. The progressive use of fluconazole and other triazoles in regimens of prophylaxis and empirical therapy in high-risk patients has led to selective pressure and emergence of secondary resistance among *Candida* spp. The aim of this study was to check for temporal trends in the rates of azole resistance among *C. tropicalis* blood stream isolates collected from patients assisted at 16 Brazilian medical centers at any time within 2007 and 2018.

**Methods:** We selected isolates of *C. tropicalis* stored at the culture collection of Laboratório Especial de Micologia (LEMI) – Universidade Federal de São Paulo, Brazil, that were obtained from candidemic patients admitted in 16 medical centers along the period of study. The isolates were identified at species level by *Matrix-Assisted Laser Desorption Ionization-Time of Fligh Mass Spectrometry* (MALDI TOF MS). *In vitro* antifungal susceptibility tests to fluconazole and voriconazole were evaluated by the Clinical & Laboratory Standards Institute (CLSI ) microbroth method (documents M27-A3 and M27-S4). For quality control of in vitro assays we used the reference strains *C. parapsilosis* ATCC 22019, *C. krusei* ATCC 6258 and *C. tropicalis* ATCC 750.

**Results:** In order to check for historical trends of azole resistance among *C. tropicalis* isolates, we compared the MIC values generated with fluconazole and voriconazole against 200 blood stream isolates collect at any time within two different periods: P1 (2007-2012) versus P2 (2013-2018). Statistical differences were checked by student T Test. After testing all 200 isolates, 3.5% of *C. tropicalis* strains were considered to be non-susceptible to azoles. As illustrated bellow, we found resistance rates of *C. tropicalis* against fluconazole of 1.9% and 3.2% in P1 and P2, respectively (p>0.05). In regard to voriconazole, resistance rates of 0 and 1% were documented in P1 and P2, respectively (p>0.05).

**Conclusion:** After testing 200 blood stream isolates of *C. tropicalis* we identified a rate of 3.5% of isolates exhibiting limited susceptibility (SDD/R) to fluconazole and/or voriconazole. Otherwise, we were not able to demonstrate any historical trend in terms of increasing rates of azole resistance among *C. tropicalis* isolates collected along the 2 periods of study.

### P053. Evaluation of spectrophotometric reading of EUCAST antifungal susceptibility testing of Aspergillus fumigatus

JanG.^1^KristensenL.^2^BentsenI. Bach^2^NielsenM.^2^1Department of Clinical Microbiology, Aarhus University Hospital, Aarhus N, Denmark2Department of Clinical Microbiology, Aarhus University Hospital, Aarhus, Denmark

**Objectives:** The rise in azole resistance in *Aspergillus fumigatus*^1^ highlights the importance of antifungal susceptibility testing. While azole containing agar plates have been shown to have excellent sensitivity and specificity in detecting azole resistance^2^, access to MIC testing is still important. As commercially available methods have serious limitations reference broth microdilution is the only feasible option at present. The EUCAST method for antifungal susceptibility testing of moulds relies on visual reading of MICs which is laborious and time-consuming. A recent study^3^ suggests that spectrophotometric determination of MICs of azoles may improve objectivity and allow automatisation of the EUCAST E.Def 9.3 method. The scope of this study is to evaluate the spectrophotometric metod in our center using seven antifungal drugs.

**Methods:** 22 clinical isolates from *A. fumigatus* species complex were selected for the study. 9 isolates were resistant to at least one azole while 13 were wild type. All isolates were tested against itraconazole, voriconazole, isavuconazole, posaconazole, amphotericin B, natamycin and terbinafine using EUCAST E.Def 9.3. Visually determined MICs were read at complete inhibition of growth.

Spectrophotometric reading was performed using a Thermo Multiskan FC microplate reader with a 492 nm filter. The OD values of the negative control wells were subtracted from all wells and MICs were determined as the lowest concentration corresponding to either 5% growth compared to the drug free control or as 0.02 OD values above background.

Spectrophotometrically determined MICs were compared to visually determined MICs. For all drugs the quantitative agreement within 0, 1 and 2 two-fold dilutions was calculated. For itraconazole, voriconazole, isavuconazole, posaconazole and amphotericin B categorical agreement was calculated using EUCAST Clinical breakpoints for fungi v. 9.0.

**Results:**

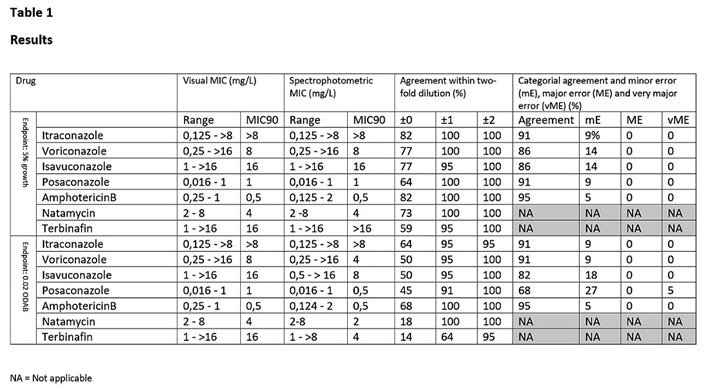



Results are shown in Table 1. When using the 5% growth endpoint we found a high level of essential agreement (95-100%) for all drugs and a high level of categorical agreement (86-95%) for itraconazole, voriconazole, isavuconazole, posaconazole and amphotericin B. of importance no major or very major errors was observed. When using the 0.02 OD values above background endpoint however we found only 64% essential agreement for terbinafine and a categorical agreement of 68% for posaconazole including 5% very major errors.

**Conclusion:** Spectrophotometric determination of MICs using the EUCAST E-Def 9.3 for A. fumigatus species complex appears to be a promising method for all tested drugs. Highest agreement between visual and spectrophotometric MICs is obtained using the 5% growth endpoint. References 1. van der Linden J et al. Prospective Multicenter International Surveillance of Azole Resistance in Aspergillus fumigatus. Emerg Infect Dis. 2015;21(6):1041-1044. 2. Arendrup MC et al; Multicentre validation of 4-well azole agar plates as a screening method for detection of clinically relevant azole-resistant Aspergillus fumigatus, Journal of Antimicrobial Chemotherapy, Volume 72, Issue 12,Pages 3325–3333 3. Meletiadis, J. et al., Spectrophotometric reading of EUCAST antifungal susceptibility testing of Aspergillus fumigatus, Clinical Microbiology and Infection, Volume 23, Issue 2, 98 - 103

### P054. Molecular mechanism and frequency of olorofim resistance in Aspergillus fumigatus

BuilJ.^1^OliverJ.^2^LawD.^2^Tehupeiory-KooremanM.^1^RexJ.^2^HokkenM.^1^MelchersW.^1^BirchM.^2^VerweijP.E.^1^1Medical Microbiology, Radboudumc, Nijmegen, Netherlands2F2G Ltd, Manchester, United Kingdom

**Objectives:** Olorofim (OLO) is a new antifungal agent with a novel mechanism of action, targeting dihydroorotate dehydrogenase (DHODH) in the *de novo* pyrimidine biosynthesis pathway. It is active against both azole-susceptible and azole-resistant strains of *Aspergillus fumigatus*. Thus, OLO may be an important treatment option in patients with *Aspergillus* disease, including azole-resistant cases. Azole resistance selection occurs in >10% of patients with chronic pulmonary aspergillosis during itraconazole (ITZ) therapy. In this study, we analysed the frequency of resistance mutation induction of OLO in *A. fumigatus* in three different strains using two different methods. The underlying OLO resistance mechanism was also investigated.

**Methods:** Method 1: From two *A. fumigatus* isolates, AZN 8196 and V139-36, 10 single colonies were separately inoculated onto Sabouraud agar and incubated at 37°C for 96h. From each culture 1 x 10^8^ conidia were applied to 6 RPMI agar plates containing either 0.5 mg/L OLO or 8 mg/L ITZ.. Plates were incubated at 37°C for up to 7 days. Colonies growing on drug-containing plates were subcultured on RPMI agar containing 0.5 mg/L OLO or 8 mg/L ITZ to confirm resistance. Method 2: Spore stocks of *A. fumigatus* strain Af293 were prepared and inoculated onto yeast nitrogen base with glucose agar (YNBG) containing 0.25 mg/L OLO. A total of 8 x 10^9^ spores were inoculated into 12 x 100 ml YNBG-OLO agar plates that were subsequently incubated for 5 days at 35°C. Colonies growing on drug-containing plates were subcultured on YNBG-OLO to confirm resistance. The mean rate of resistance was calculated for each isolate. The *pyrE* gene which codes for DHODH was sequenced for initial OLO-susceptible strains and -resistant progeny isolates and analysed for mutations.

**Results:** Method 1: The resistance rate of OLO was 1 in 5 x 10^7^ (frequency of 2 x 10^-8^) for AZN 8196 compared to 1 in 5 x 10^6^ (2 x 10^-7^) for ITZ. For V139-36 the resistance rate of OLO was 1 in 9 x 10^7^ (1.1 x 10^-8^) while the rate for ITZ was 1 in 8 x 10^6^ (1.3 x 10^-7^) Method 2: Resistance to OLO in Af293 occurred at a rate of 1 in 6 x 10^8^ (frequency of 1.7 x 10^-9^). Sequencing of pyrE revealed a hotspot for resistance mutations in the pyrE gene for OLO resistance in *A. fumigatus* at locus Gly119. Four amino acid substitutions were found; G119V, G119C, G119S and G119A. A single OLO-resistant mutant was obtained that carried the wild type DHODH sequence, but this mutant was slow growing compared with parent.

**Conclusion:** We demonstrate that OLO resistance can be selected in *A. fumigatus* and can be mediated by mutations in the pyrE gene at locus G119. The frequency of resistance varied from approximately 2 x 10^-8^ to 1.7 x 10^-9^ for spontaneous mutations. The resistance rate of OLO was 5 to 10-fold lower compared to ITZ. The low resistance rate found in these experiments indicates that resistance selection may be less frequent in patients with *Aspergillus* diseases treated with OLO.

### P055. Comparison of killing activity of rezafungin, anidulafungin, caspofungin and micafungin against Candida auris in the presence and absence of serum

TóthZ.^1^ForgácsL.^1^LockeJ.B.^2^KardosG.^1^NagyF.^1^KovácsR.^1^BormanA.^3^MajorosL.^1^1Department of Medical Microbiology, University of Debrecen, Debrecen, Hungary2Cidara Therapeutics, San Diego, United States of America3Public Health England UK National Mycology Reference Laboratory, Bristol, United Kingdom

**Objectives:** *Candida auris* is an emerging, difficult-to-treat multiresistant pathogen against which echinocandins are the recommended standard-of-care treatment. Rezafungin is a next-generation echinocandin with similar *in vitro* activity to existing echinocandins yet it attains much higher *in vivo* concentrations and exposures due to its extended half-life and front-loaded dosing paradigm. Because *in vitro* killing data with existing echinocandins are limited against *C. auris* and are lacking for rezafungin, we compared rezafungin to anidulafungin, caspofungin, and micafungin in time-kill assays against *C. auris* isolates in standard RPMI-1640 medium and also investigated the impact of serum on *in vitro* killing trends.

**Methods:** Two *C. auris* clinical isolates from each clade (Japanese/Korean, South Asian/Indian and South African, obtained from the National Mycology Reference Laboratory, UK) were tested. Both South African isolates were autoaggregative. MICs in RPMI-1640 +/-50% human serum were determined using the standard broth macrodilution method (CLSI M27 Ed4). Time-kill studies with the four echinocandins were performed from 0.25 to 32 mg/L in both media, and killing rates were compared. Positive *k* values indicate killing; negative values indicate growth.

**Results:** MIC values were higher in 50% serum than in RPMI-1640 alone (Table 1). In RPMI-1640, at 1xMIC or higher concentrations all four echinocandins showed only fungistatic effect. The highest *k* value with rezafungin (1.34 1/h) was at 32 mg/L against isolate 209. However, none of the echinocandins produced any CFU decrease against aggregating isolates (*k* values in cases of isolates 2 and 204 were always negative). Similar results were found in cases of caspofungin and micafungin against isolates 12, 15 and 27, and isolate 15, respectively. Re-growth was frequently observed for all four echinocandins. In 50% serum, growth rates were significantly lower. At 1xMIC or higher concentrations, echinocandins showed concentration-dependent killing activity, however, this CFU reduction never reached the fungicidal threshold. The highest *k* values were 0.34, 0.33, 0.34 and 0.29 1/h for rezafungin, anidulafungin, caspofungin and micafungin, respectively. However, *k* values were always negative at 1-32 mg/L, in the case of isolate 2 (an autoaggregative isolate) for all echinocandins and in cases of isolates 27 and 204 for caspofungin. Table 1. MIC values for rezafungin and echinocandin comparators against *C. auris* strains in the presence and absence of 50% serum.

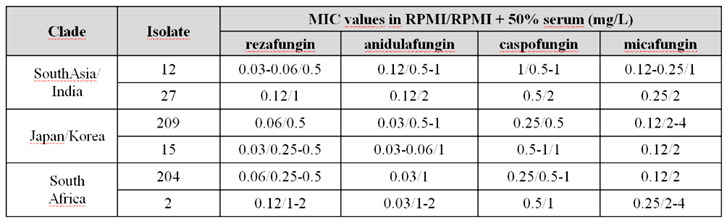



**Conclusion:** Killing activity in RPMI-1640 alone was less consistently positive than in 50% serum and only fungistatic activity was detected. Aggregating isolates were less susceptible to echinocandins than non-aggregating isolates. Rezafungin showed the same or better activity than anidulafungin and micafungin at clinically attainable concentrations. The trend towards stronger killing activity in the presence of serum helps to account for the disconnect between the modest time-kill activity of echinocandins *in vitro* versus their strong efficacy against *C. auris in vivo*, as rezafungin has previously demonstrated in animal models.

### P056. Frequency of paradoxical and trailing effects with rezafungin, anidulafungin, caspofungin and micafungin against Candida species

ForgácsL.^1^TóthZ.^1^LockeJ.B.^2^NagyF.^1^BalázsB.^1^PrépostE.^1^KardosG.^1^KovácsR.^1^SzekelyA.^3^BormanA.^3^MajorosL.^1^1Department of Medical Microbiology, University of Debrecen, Debrecen, Hungary2Cidara Therapeutics, San Diego, United States of America3Public Health England UK National Mycology Reference Laboratory, Bristol, United Kingdom

**Objectives:** Currently, echinocandins are the first-line agents for treatment of invasive *Candida* infections. Echinocandins demonstrate excellent efficacy and fungicidal activity against *Candida* species *in vivo*. However, echinocandins can exhibit some disconnected growth phenomena *in vitro*, such as paradoxical effect (PE) where activity decreases at higher drug concentrations and the trailing effect (TE) where complete growth inhibition is never achieved or occurs at dilutions well above the 50% endpoint used to determine MIC values. PE and TE can significantly alter the determination/interpretation of MIC values and impact other *in vitro* assays. Rezafungin is a next-generation echinocandin capable of attaining high concentrations and exposures *in vivo,* exceeding those measured for the three approved echinocandins, due to its long half-life and front-loaded dosing regimen. Given the relevance of evaluating higher drug concentrations for rezafungin and that rezafungin PE/TE trends have not yet been characterized, herein we compared the occurrence of PE and TE for rezafungin with data generated in parallel for caspofungin, micafungin and anidulafungin against clinically important *Candida* species.

**Methods:** We tested 365 non-duplicate Hungarian clinical isolates belonging to 13 species (see Table). Isolates were collected between 2005 and 2018 and were identified with MALDI Biotyper (Bruker, Bremen, Germany). *C. auris* were obtained from National Mycology Reference Laboratory, UK. MICs were determined by BMD method according to CLSI (M27 Ed4) in RPMI-1640. Concentration ranges of antifungals were 0.06-32 mg/L. PE was defined as visible growth occurring at higher but not at lower supra-MIC concentrations. TE was defined when yeasts show reduced but observable growth in supra-MIC concentrations.

**Results:** The percentages of isolates showing PE or TE after 48 hours are shown in the Table. Cumulative percentage of PE plus TE was the highest for caspofungin (47.7%), followed by anidulafungin (35.9%), micafungin (25.8%) and rezafungin (17.7%). PE was observed most frequently for caspofungin (31.4%) but was uncommon for rezafungin (3.1%). PE with caspofungin, micafungin or anidulafungin occurred most frequently in cases of *C.tropicalis*, *C. albicans*, *C. orthopsilosis* and *C. inconspicua* but was never found incase of *C. krusei*. The majority of *C. auris* and *C. dubliniensis* isolates showed TE for all four echinocandins. Table. Percentages of *Candida* spp. isolates demonstrating paradoxical effect (PE) or trailing effect (TE).

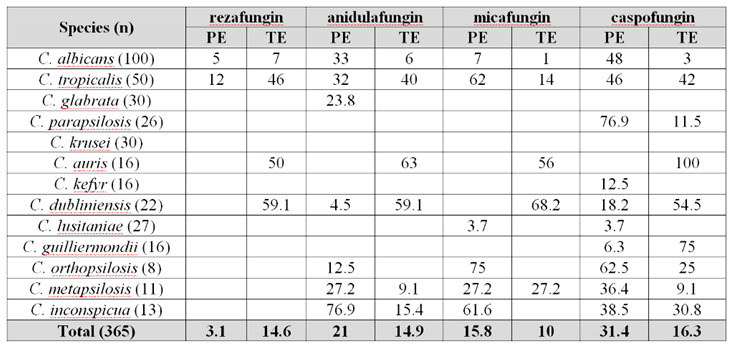



**Conclusion:** PE or TE was widely observed among *Candida* species and were echinocandin- and species-dependent. PE was frequently found with caspofungin, micafungin and anidulafungin but not with rezafungin. This study is the first to characterize PE and TE trends for rezafungin and helps to inform future *in vitro* work with this novel echinocandin.

### P057. In vitro antifungal susceptibility testing of cryptic species of Aspergillus: a multi-centre study

ImbertS.^1^NormandA.C.^2^GabrielF.^3^BonnalC.^4^LachaudL.^5^CostaD.^6^CassaingS.^7^HasseineL.^8^KristensenL.^9^SchuttlerC.^10^RaberinH.^11^BrunS.^12^PiarrouxR.^1^FekkarA.^1^1Laboratoire De Parasitologie-mycologie, APHP La Pitié Salpétrière, PARIS, France2Ap-hp, Groupe Hospitalier Pitié-salpêtrière, Service de Parasitologie Mycologie, Paris, France3Parasitology Mycology, CHU de Bordeaux, Bordeaux, France4Laboratoire De Parasitologie Mycologie, Hôpital Bichat-Claude Bernard, 46 rue Henri Huchard, PARIS, France5Laboratoire De Parasitologie-mycologie, CHU de Montpellier, Montpellier, France6Laboratoire De Parasitologie-mycologie, CHU de Rouen, Rouen, France7CHU de Toulouse, Toulouse, France8Laboratoire De Parasitologie Mycologie, CHU DE NICE, NICE, France9Department of Clinical Microbiology, Aarhus University Hospital, Aarhus, Denmark10Laboratoire BioParisOuest, Levallois Peret, France11CHU Saint Etienne, Saint Etienne, France12Parasitology-mycology, Avicenne Hospital, Bobigny, France

**Objectives:** In recent years, *Aspergillus* species taxonomy has been revolutionized with the introduction of the concepts of cryptic species and species complex. However, these “new” species remain usually rare and their characteristics, including antifungal susceptibility, are little known. Thus, the aim of this study was to collect a large number of isolates per cryptic species and to assess their antifungal susceptibility.

**Methods:** From September 2017, users of the MSI application, a free online MALDI-TOF mass-spectrometry database, were asked to send to our University Hospital, isolates they identified as cryptic species of *Aspergillus*. Identification at the species level was confirmed by sequencing a part of beta-tubulin and calmodulin genes. Antifungal susceptibility for azole drugs (itraconazole, voriconazole, posaconazole and isavuconazole) and amphotericin B was assessed by EUCAST broth microdilution reference method.

**Results:** During the study period, 192 isolates were collected from 11 centers in France and Denmark and were assessed for antifungal susceptibility. According to sequence-based identification, isolates corresponded to 43 species, belonging to 6 species complexes. *Nidulantes* species complex was the most represented (53 isolates, 12 species), followed by the species complexes *Circumdati* (43 isolates, 10 species) and *Fumigati* (40 isolates, 8 species). The last species complexes represented were *Usti* (23 isolates, 4 species), *Flavi* (19 isolates, 5 species) and *Terrei* (14 isolates, 4 species). Regarding the 3 main species complexes, diversity in antifungal susceptibility between cryptic species was observed. Indeed, inside the *Fumigati* complex *A. thermomutatus*, *A. lentulus*, *A. udagawae*, *A. fischeri* and (15, 7, 3 and 3 isolates respectively) showed pan-azole high minimal inhibitory concentrations (MICs), whereas *A. hiratsukae* (8 isolates) showed low MICs. All these species had low MICs for amphotericin B, except *A. lentulus* with MICs ³4 mg/L. Regarding the *Circumdati* species complex, *A. westerdijkiae* (12 isolates) showed the highest MICs for amphotericin B (> 16 mg/L), whereas *A. sclerotiorum* (17 isolates) showed lower MICs (MIC_50_ = 4 mg/L). In contrast *A. sclerotiorum* showed pan-azole high MICs, whereas azole MICs where much lower for *A. westerdijkiae*. Finally, inside the *Nidulantes* complex, all species showed low MICs for all drugs, except *A. creber* and *A. unguis* (6 and 2 isolates respectively) which showed high MICs (> 8 mg/L) for itraconazole only. MICs regarding the 3 other species complexes studied are shown in Table 1.

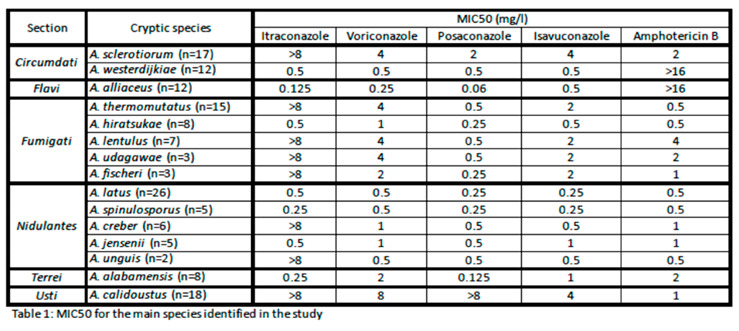



**Conclusion:** This study brings evidence on the diversity of antifungal susceptibility pattern inside the same species complex. However, including more isolates is needed to confirm these preliminary results. Nevertheless, this underline the importance of an accurate and easy-to-perform identification of *Aspergillus* at the species level for the management of *Aspergillus* diseases and the conduct of epidemiological studies.

### P058. Species identification, in vitro antifungal susceptibility testing and human pathogenicity of Fusarium species: a European multicenter study

ImbertS.^1^NormandA.C.^1^BrunS.^2^RanqueS.^3^HasseineL.^4^CostaD.^5^CassaingS.^6^ChryssanthouE.^7^BonnalC.^8^DelhaesL.^9^RubioE.^10^BourgeoisN.^11^SchuttlerC.^12^GuitardJ.^13^SautourM.^14^JostG.^15^BrandenbergerM.^16^KristensenL.^17^SendidB.^18^SeethC. Gronfors^19^LaereE. De^20^HendrickxM.^21^RoseV. Sainte^1^PiarrouxR.^1^FekkarA.^1^1Laboratoire De Parasitologie-mycologie, APHP La Pitié Salpétrière, PARIS, France2Parasitology-mycology, Avicenne Hospital, Bobigny, France3IHU-Méditerranée Infection, Marseille, France4Laboratoire De Parasitologie Mycologie, CHU DE NICE, NICE, France5Laboratoire De Parasitologie-mycologie, CHU de Rouen, Rouen, France6CHU de Toulouse, Toulouse, France7Clinical Microbiology, Karolinska University Hospital, Stockholm, Sweden8Laboratoire De Parasitologie Mycologie, Hôpital Bichat-Claude Bernard, 46 rue Henri Huchard, PARIS, France9Laboratoire De Parasitologie-mycologie, CHU de Bordeaux, Bordeaux, France10Barcelona hospital, Barcelona, Spain11Laboratoire De Parasitologie-mycologie, CHU de Montpellier, Montpellier, France12Laboratoire BioParisOuest, Levallois Peret, France13Parasitology-mycology, APHP, hôpital St Antoine, Paris, France14Laboratoire Parasitology Mycology, CHU Dijon, Dijon, France15Dianalabs, Geneve, Switzerland16synlab, Lucerne, Switzerland17Department of Clinical Microbiology, Aarhus University Hospital, Aarhus, Denmark18Laboratory of Mycology and Parasitology, University Hospital of Lille, Lille Cedex, France19Clinical Microbiology, Karolinska University Hospital, karolinska Institutet, Stockholm, Sweden20AZ Roeselare, Roeselare, Belgium21Mycology, Scientific Institute of Public Health, Brussels, Belgium

**Objectives:** Fungi belonging to the genus *Fusarium* are responsible of a large number of infections that may be difficult to treat because of a low susceptibility to antifungal drugs available. However, in recent years, fungal taxonomy has been revolutionized with the introduction of the concepts of cryptic species and species complex. This is particularly true for the genus *Fusarium*, which taxonomy remains partially unclear. In consequence, pathogenicity and antifungal susceptibility specific to each *Fusarium* species is largely unknown. Thus, the aim of this study was to collect a large number of isolates per species, to collect clinical data on their pathogenicity and to assess their antifungal susceptibility.

**Methods:** During 1 year (January-December 2018), users of the MSI application, a free online MALDI-TOF mass-spectrometry database, were asked when they identified a *Fusarium sp.* to send prospectively to our University Hospital laboratory the isolates and to collect clinical data. Identification at the species level was confirmed by sequencing a part of *TEF1α* gene. Antifungal susceptibility for azole drugs (voriconazole, posaconazole and isavuconazole), terbinafin and amphotericin B was assessed by measuring minimal inhibitory concentrations (MICs) by EUCAST broth microdilution reference method.

**Results:** During the study period, 145 isolates from 20 centers in 6 European countries were collected. According to sequence-based identification, 23 species were identified and were distributed in 6 species complexes (SC) whose main ones are *Fusarium oxysporum* SC (FOSC, 54 isolates), *Fusarium solani* SC (FSSC, 48 isolates) and *Fusarium fujikoroi* SC (FFSC, 36 isolates). Regarding these 3 main species complexes, minimal inhibitory concentrations which inhibited 50% of our isolates (MIC50) were high for all azole drugs, FSSC showing the highest MICs for voriconazole. For amphotericin B, MIC50 were not high (1 mg/l) and no differences were observed between complexes. Finally, terbinafine susceptibility seemed to be related to the species complex. Indeed, FOSC and FSSC showed the highest MICs (MIC50 = 16 mg/l) whereas FFSC showed lower MICs (MIC50 = 2 mg/l). MICs results are shown in Table 1. 

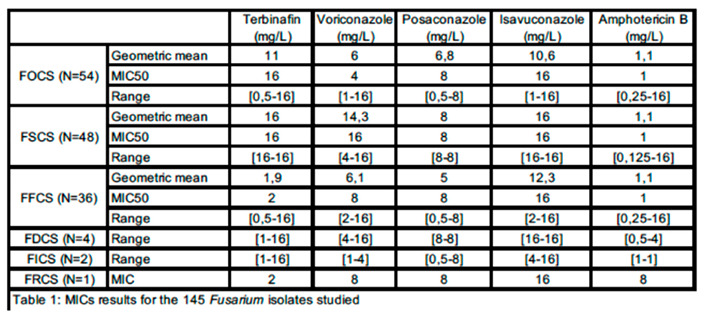



Among isolates collected, clinical data were available for 117 isolates. They were responsible for 11 invasive/disseminated infections, 19 keratitis and 40 superficial infections (34 onychomycosis and 6 intertrigo). Sixteen isolates were considered as pulmonary airways colonization, whereas 31 were considered as culture contaminants. The three main species complexes identified in the study were represented in each category.

**Conclusion:** This study brings evidence on the diversity of *Fusarium* species implicated in human diseases and their differences in terms of *in vitro* antifungal susceptibility, mainly for terbinafin. This underlines the importance of an accurate and easy identification of *Fusarium* species, at least at the complex level, to improve therapeutic management of *Fusarium*-related diseases.

### P059. Tacrolimus and not cyclosporin A or sirolimus interacts synergistically in vitro with isavuconazole against Aspergillus species

SchwarzP.^1^,^2^DannaouiE.^3^,^4^1Department of Internal Medicine - Respiratory and Critical Care Medicine, University Hospital Marburg, Marburg, Germany2Center For Invasive Mycoses and Antifungals, Philipps University Marburg, Marburg, Germany3Hôpital Européen Georges Pompidou, Laboratoire de Parasitologie-Mycologie, Paris, France4Working Group Dynamyc, Faculté de Médecine, Hôpital Henri Mondor, Créteil, France

**Objectives:** Invasive aspergillosis is a devastating disease in immunocompromised patients. It mostly affects patients with hematological malignancies, especially those with severe and prolonged neutropenia, but is also encountered in solid organ transplant recipients. Immunosuppressive agents are used as anti-rejection drugs in organ transplant patients, but besides their immunosuppressive activity, these drugs also possess intrinsic antifungal activity. The aim of the present study was to assess the *in vitro* interaction of the broad-spectrum azole isavuconazole with the calcineurin inhibitors (e.g. tacrolimus or cyclosporin A) or the mTOR pathway inhibitor (e.g. sirolimus) against the most important *Aspergillus* species responsible for human disease.

**Methods:** A panel of 30 Aspergillus isolates belonging to 5 species responsible for human invasive aspergillosis was used for the experiments (10 *Aspergillus fumigatus*, 5 *Aspergillus flavus*, 5 *Aspergillus terreus*, 5 *Aspergillus nidulans*, and 5 *Aspergillus niger*). Combinations of isavuconazole with cyclosporine A, tacrolimus or sirolimus were tested by a broth microdilution checkerboard technique based on the EUCAST reference methodology. Plates were read spectrophotometrically, and 90% of inhibition was used as an endpoint for both, the drugs alone and in combination. Results were interpreted with the fractional inhibitory concentration index (FICI). Drug interactions were defined as synergistic (FICI≤0.5), indifferent (FICI>0.5≤4) or antagonistic (FICI>4).

**Results:** Isavuconazole MICs ranged from 0.25 to 16 µg/ml. When tested alone, immunosuppressive drugs showed poor activity with MICs < 16 µg/ml for only 2, and 3 isolates for tacrolimus and cyclosporin A, respectively. Combinations of isavuconazole with tacrolimus, cyclosporine A or sirolimus were synergistic for 53, 20, and 10% of the tested isolates, respectively. There were some differences between species (Table). Antagonism was never seen.

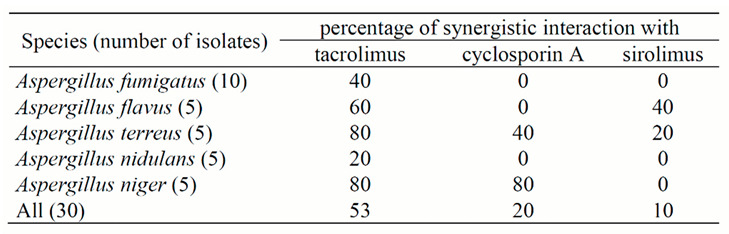



**Conclusion:** Calcineurin is a key enzyme in the calcium-dependent signal transduction pathways of eukaryotes. Antifungal drug resistance and virulence of several pathogenic fungi, including *Aspergillus* species, is regulated by this pathway. Results of the present study showed synergistic interactions between isavuconazole and tacrolimus and underline the role of the calcineurin pathway as a target for the development of new antifungal drugs.

### P060. Synergistic antifungal effect of fluconazole combined with pure culture extract of Candida albicans against Trichophyton mentagrophytes

LemesT.^1^AlmeidaB. De^2^CaetanoM.^3^PolaquiniC.R.^4^RegasiniL.O.^5^Maschio-LimaT.^3^BrizzottiN.^1^,^6^CastilhoE.^6^SiqueiraJ.P.^7^MarquesM.^6^AlmeidaM.^8^1Microbiologia, Unesp, São José do Rio Preto, Brazil2Unesp, São José do Rio Preto, Brazil3UNESP, São José do Rio Preto, Brazil4São Paulo State University - Institute of Biosciences, Humanities and Exact Sciences (UNESP - IBILCE), São José do Rio Preto, Brazil5Chemistry and Environmental Sciences, UNESP (Sao Paulo State University), São José do Rio Preto, Brazil6FAMERP, São José do Rio Preto, Brazil7Infeciosas Disease, FAMERP, São José do Rio Preto, Brazil8Infectious Disease, Medical School, sao jose do rio preto, Brazil

**Objectives:** The present study evaluated the synergistic interaction between a pure culture extract of *Candida albicans* and fluconazole against *Trichophyton mentagrophytes*.

**Methods:** This study used clinical strains of *C. albicans* from the Laboratory of Microbiology of the Medical School in São José do Rio Preto (FAMERP), Brazil. A 500-mL Inoculum prepared in Sabouraud Dextrose Broth was filtered through a 0.2 μm millipore membrane and separated using ethyl acetate as a counter-phase. The ethyl acetate phase was completely dried using a rotary evaporator and subsequently solubilized in sterile distilled water with 10% dimethyl sulfoxide (DMSO). Minimal Inhibitory Concentration (MIC) tests were performed for *T. mentagrophytes* strains following the Clinical and Laboratory Standards Institute (CLSI) M38-A2 guidelines. After obtaining the extract’s MIC, a checkerboard trial with fluconazole was performed to evaluate the synergistic interaction with the extract based on the calculation of the fractional inhibitory concentration index (ICIF) = (MIC fluconazole in the mix / MIC fluconazole alone) + MIC extract in the mix / MIC extract isolated). The synergistic interaction was classified using the method described by Kumar et al., where values ≤ 0.5 indicate significant interactions.

**Results:** The results obtained for the *C. albicans* extract’s MIC against the *T. mentagrophytes* strain and fluconazole in isolation were 1000 μg/mL and 16 μg/mL, respectively. However, when the extract was used in combination with fluconazole, the MIC value was 0,001 μg/mL and of fluconazole it was 2 μg/mL with an ICIF value of 0,01.

**Conclusion:** The extract of *C. albicans* in isolation shows antifungal activity against *T. mentagrophytes*, which may influence the laboratory diagnosis of mixed infections. Furthermore, the action of this extract is greater in association with an azole derivative, thus proving synergy. In the future, the isolation and identification of extract compounds may allow new therapeutic approaches in the control of fungal infections.

### P061. Screening of antifungal susceptibility of Malassezia pachydermatis isolates from dogs using disk diffusion test

HadinaS.^1^BenvinI.^2^ŠtritofZ.^1^HabušJ.^1^StevanovićV.^1^PerharićM.^1^MartinkovićK.^1^MojčecV.^1^BarbićL.^1^MilasZ.^1^StarešinaV.^1^TurkN.^1^PinterL.^1^1Department of Microbiology and Infectious Diseases With Clinic, Faculty of Veterinary Medicine, The University of Zagreb, Zagreb, Croatia2Faculty of Veterinary Medicine, The University of Zagreb, Zagreb, Croatia

**Objectives:** *Malassezia pachydermatis* is lipophilic yeast commonly seen in skin and ear diseases of dogs. Lately, different studies are describing the appearance of reduced susceptibility of *M. pachydermatis* strains. In addition, reports about its zoonotic potential and emerged number of *Malassezia* yeast resistance in human medicine, highlight the need for study of its antifungal susceptibility. The problem of surveillance of potential resistance is lying in no established reference method for antifungal susceptibility testing and no interpretative criteria for this yeast. Various studies used different methodologies and interpreted obtained results based on different recommendations. Modified microdilution method is considered the most accurate, but at the same time more expensive and technically demanding to perform in clinical laboratories. Recent study demonstrated good correlation of results obtained by modified CLSI M44-A2 disk diffusion and broth microdilution method, indicating its suitability for *in vitro* susceptibility testing of *M. pachydermatis* in epidemiological studies (Pasquetti et al., 2015.). The aim of this study was to screen antifungal susceptibility of *M. pachydermatis* for miconazole (MCZ) and clotrimazole (CTZ) using disk diffusion method.

**Methods:** A total of 96 isolates of *M. pachydermatis* were recovered from animals with clinical signs of otitis. Identification was based on their ability to grow on Sabouraud dextrose agar and typical macroscopic and microscopic appearance. Two antifungal tablets containing most commonly azole derivatives used for topical therapy were tested: MCZ and CTZ (10 µg, Neo-Sensitabs, Rosco, Denmark). Disk diffusion test was performed using Mueller-Hinton agar supplemented with 2% glucose and 0.5 μg/l methylene blue. Inoculum of *M. pachydermatis* was prepared in sterile saline from 3 days old cultures, with addition of the lipid source, containing approximately 1-5x10^7^ CFU/mL. Readings were performed 72 hours after incubation at 37°C, and interpreted according to guidelines provided by manufacturer.

**Results:** Out of 96 tested, all isolates demonstrated good susceptibility to CTZ with variations of inhibition zones from 34 to 73 mm (mean ± SD: 56 ± 8,06 mm). Susceptibility testing against MCZ resulted with the range of inhibition zones from 14 to 65 mm (mean ± SD: 41 ± 6,77 mm) and good sensitivity of 95 strains, while one isolate demonstrated intermediate sensitivity. Comparison of the size of the zones showed that CTZ had larger zones of inhibition than MCZ indicating its higher antifungal activity.

**Conclusion:** Screening of antifungal susceptibility of *M. pachydermatis* using disk diffusion method resulted with identification of one strain with reduced susceptibility to MCZ. However, taking into consideration that there are no standardized interpretative criteria for *Malassezia* yeast, follow up study using broth microdilution assay is needed, in order to check if this isolate could be categorized as potentially resistant. Reference: Pasquetti, M., E. Chiavassa, P. Tizzani, P. Danesi, A. Peano (2015): Agar diffusion procedures for susceptibility testing of *Malassezia pachydermatis*: Evaluation of Mueller-Hinton agar plus 2% glucose and 0.5 µg/mL methylene blue as the test medium. Mycopathologia 180, 153-158.

### P062. Susceptibility to voriconazole of Aspergillus spp. strains, isolated from patients in Russia

VasilyevaN.^1^VybornovaI.^2^BogomolovaT.^1^1Kaschkin Research Institute of Medical Mycology. Department of Medical Microbiology, North-Western State Medical University named after I.I. Mechnikov, Saint-Petersburg, Russian Federation2Kaschkin Research Institute of Medical Mycology, North-Western State Medical University named after I.I. Mechnikov, Saint-Petersburg, Russian Federation

**Objectives:** Azole resistance of aspergillosis etiologic agents is an emerging problem in Europe and other parts of the world. The aim of the study: to determine susceptibility to voriconazole of *Aspergillus* spp. strains isolated from patients with aspergillosis in Saint Petersburg, Russia.

**Methods:** Fungal species identification was done using morphological and molecular methods. A total of 230 strains of *Aspergillus* spp. isolated from biological samples of patients with different clinical forms of aspergillosis (invasive, chronic and allergic) in Saint Petersburg during 2014 – 2019 yrs. have been tested by standard disk diffusion method according to CLSI M51A Document. For 34 strains MICs of voriconazole have been determined according to EUCAST Document E Def 9.3.1.

**Results:** Species distribution among *Aspergillus* spp. isolates was as follows: *A. fumigatus –* 68%, *A. niger –* 16%, *A. flavus –* 13%, rare species – 3% (*A. terreus –* 4 strains, *A. calidoustus –* 2, *A. ustus –* 1, *A. sydowii –* 1). By disk diffusion method no voriconazole resistant isolates were found among common species: *A. fumigatus, A. niger, A. flavus.* Only 4 resistant strains were revealed among rare species: *A. terreus –* 1, *A. calidoustus –* 2, *A. ustus –* 1 (inhibition zone diameter < 17 mm). Voriconazole MICs for *A. fumigatus, A. flavus, A. niger* strains ranged from 0,125 to 0,5 mg/L. Only *A. calidoustus* showed high MIC – 4 mg/L.

**Conclusion:** Resistance to voriconazole among etiologic agents of aspergillosis in Saint Petersburg, Russia was found only among rare fungal species (*A. calidoustus, A. ustus, A. terreus*). Future surveillance is necessary to monitor antifungal resistance.

### P064. Antifungal susceptibility testing of Malassezia furfur

BogomolovaT.^1^BogdanovaT.^2^VasilyevaN.^1^AlexeyevA.^1^SpiridonovaV.^3^1Kaschkin Research Institute of Medical Mycology. Department of Medical Microbiology, North-Western State Medical University named after I.I. Mechnikov, Saint-Petersburg, Russian Federation2Department of Medical Microbiology, North-Western State Medical University named after I.I. Mechnikov, Saint-Petersburg, Russian Federation3Kaschkin Research Institute of Medical Mycology, North-Western State Medical University named after I.I. Mechnikov, Saint-Petersburg, Russian Federation

**Objectives:** *Malassezia* spp. may be etiologic agents of superficial and invasive mycoses. There are no standard protocols for evaluation of antifungal susceptibility of these yeasts. The aim of the study was to determine *Malassezia furfur* isolates susceptibility to antifungal agents by two different methods.

**Methods:** Disk diffusion method CLSI M44A2 was modified by addition of lipids to the Muller-Hinton agar. Commercial test panels Sensititre Yeast One were also supplemented by fatty acid RPMI 1640.

**Results:** We tested 11 strains of *M. furfur* isolated from blood of pediatric patients. Both methods showed resistance to fluconazole and voriconazole. By Sensititre Yeast One panels the following MICs were obtained: amphoterecin B – 1 mg/L, fluconazole > 64 mg/L, caspofungin >8 mg/L, voriconazole – 1-8 mg/L, anidulafungin >8 mg/L, micafungin >8 mg/L, 5-fluorocytozne - > 256 mg/L, itraconazole - < 0,015, mg/L, pozaconazole – 0,03-0,06 mg/L.

**Conclusion:** Disk diffusion method may be used for screening antifungal resistance in *Malassezia* spp. isolates. Itraconazole and pozaconazole demonstrated highest activity against *M. furfur.*

### P065. Evaluation of the efficacy of antifungals against invasive aspergillosis in an invertebrate animal model

SanaJ.^1^MelloulÉ.^1^GuillotJ.^1^BotterelF.^1^,^2^DannaouiE.^1^,^3^18 Rue Du General Sarrail, DYNAMYC, Creteil, France2Laboratoire De Parasitologie-mycologie, APHP Hôpital Henri Mondor, Créteil, France3Hôpital Européen Georges Pompidou, Laboratoire de Parasitologie-Mycologie, Paris, France

**Objectives:** Evaluation of new therapeutic strategies for the treatment of invasive aspergillosis is needed due to emergence of azole-resistance in *Aspergillus fumigatus*. Animal models in rodents are generally used but are costly and their use is limited by ethical considerations. For these reasons, evaluation of antifungal efficacy in mini-host models such as *Galleria mellonella* is a promising alternative. The aim of our study was to develop an invasive *Aspergillus* infection in larvae of *Galleria mellonella* to evaluate the efficacy of antifungals.

**Methods:** Three clinical strains of *Aspergillus fumigatus* (HEGP064, HEGP4017 and HEGP2666) have been used. Minimum inhibitory concentration for voriconazole (VRZ) was 0.5, 0.5 and 8µg/mL for HEGP064, HEGP4017 and HEGP2666 respectively. Inoculum finding experiments (with spore suspensions at 10^5^ to 10^8^ conidia/mL) have been performed to determine the concentration that gave 90% mortality at seven days post-infection (LD_90_). The presence of an invasive infection has been demonstrated by direct examination of tissues stained with Gomori-Grocott (GG) and by histopathology (H&S staining). In a first set of experiments, groups of 10 larvae were infected with the LD_90_ and treated with monotherapy by voriconazole (VRZ, [Vfend®] at 0.5, 1, 2, 4, and 8 μg/larva), amphotericin B (AMB, [Fungizone®], or caspofungin (CSP, [Cancidas®] at 0.2, 0.4 and 0.8 μg/larva). Subsequently, HEGP4017 or HEGP2666 infected larvae were treated after two hours by VRZ at 4µg/ml alone and in combination with CSP at 4, 2 or 1µg/larvae. The mortality was estimated daily for 7 days.

**Results:** After infection, an invasive aspergillosis was obtained as shown by the presence of hyphae in tissues. The LD_90_ values for the HEGP064, HEGP4017 and HEGP2666 strains were 4.38×10^7^,9.59×10^7^ and 1×10^8^ CFU/ml respectively. In infected and untreated groups, mortality was at least 90% on day 7 post-infection. The mortality decreased with increasing doses of amphotericin B. At 4 µg/larvae, the mortality was on average 20% for the 3 strains. In animals treated with VRZ, the mortality rates on day 7 post-infection were 35%, 40% and 60% for HEGP064 and were 55%, 65% and 95% for HEGP4017 and 85%, 95% and 95% for 8, 4 and 2 μg/larva, respectively (p = 0.0001 compared to control). VRZ at 8, 4 and 2 μg/larva was statistically more effective for the treatment of larvae infected with HEGP064 or HEGP4017 compared to those infected by HEGP2666. Administration of VRZ 4 did not decreased, significantly, the mortality of HEGP2666 infected larvae (p = 0.304) in contrast to those infected by HEGP4017 (p = 0.0082). The bitherapy VRZ 4µg/larvae and CSP 4µg/larvae lead to a more significant decrease in mortality compared to monotherapy (VRZ 4µg/larvae) and control group infected by HEGP4017(p = 0.026) and HEGP2666 (p = 0.005) strain.

**Conclusion:** *G. mellonella* is a reliable model for inducing invasive aspergillosis. There was a clear dose-response when animals were treated with VRZ and AMB. This model could be used for screening and evaluation of different therapeutic strategies against *Aspergillus spp*.

### P066. Analysis of the efflux-pump activity against resistant Sporothrix schenckii complex isolates

ScrofernekerM.L.^1^PinheiroT.R.^2^SeibertG.^2^PolettoA.L.R.^3^PradeJ.V.^3^StopigliaC.D.O.^3^1Department of Microbiology, Immunology and Parasitology, Universidade Federal do Rio Grande do Sul, Porto Alegre, Brazil2Universidade Federal do Pampa, Uruguaiana, Brazil3Universidade Federal do Pampa, Uruguaiana, RS, Brazil

**Objectives:** The present study aims to assess the efflux-pump activity in the resistant *Sporothrix schenkii* complex strains.

**Methods:** The minimum inhibitory concentration (MIC) of itraconazole (0.03125-16 µg/mL) was determined through broth microdilution method, as described by protocol M38-A2 of the *Clinical and Laboratory Standards Institute* (CLSI, 2008). Ten clinical isolates of *Sporothrix schenckii* complex were prepared from 7-day-old cultures grown on potato dextrose agar at 28 °C. The inocula were adjusted to a final concentration of 0.5–2.5×10^3^ cells/mL. The microdilution plates were incubated at 35 °C for 72h. The MIC of itraconazole was defined as the lowest drug concentration capable of inhibiting total of growth. For the analysis of the efflux-pump activity, the resistant *Sporothrix schenckii* complex strains (04/10) were tested against two efflux-pump inhibitors, promethazine and verapamil by broth microdilution method. Posteriorly, susceptibility test was performed with itraconazole and sub-inhibitory concentrations of promethazine (MIC/8 = 24 µg/mL) and verapamil (MIC/8 = 100µMol).

**Results:** There were no reduction of MIC for itraconazole in the presence of the verapamil and prometazine inhibitors.

**Conclusion:** Thus, the present study suggests that the mechanism of resistance of the isolated evaluated isn’t the presence of efflux pumps.

### P067. Terbinafine resistant Trichophyton mentagrophytes ITS genotype VIII from India in Germany

NenoffP.^1^SüβA.^2^LudesA.^3^SchullerA.^4^FischerE.^5^DessoiS.^6^HofmannW.^6^SchmidtS.^7^VermaS.B.^8^MonodM.^9^SalaminK.^9^KruegerC.^1^EbertA.^1^UhrlassS.^1^1Partnership Dr. C. Krueger & Prof. P. Nenoff, Laboratory of medical microbiology, Roetha OT Moelbis, Germany2Hautärztin, Gemeinschaftspraxis Allgemeinmedizin und Dermatologie, Wittlich, Germany3Allgemeinmedizinische Praxis Dr. med. Alfons Ludes und Thorsten Koech, Leiwen, Germany4Hautarztpraxis, Halle/Saale, Germany5Hautarztpraxis Dr. med. Gunhild Kratzsch, Leipzig, Germany6Hautzentrum Nordwest, Frankfurt am Main, Germany7Hautarztpraxis im MVZ Friedrichstadt, Dresden, Germany8“Nirvan” and “In Skin” Clinics, Vadodara, India9Dermatology Service, Centre Hospitalier Universitaire Vaudois, Lausanne, Switzerland

**Case Report: Objectives**: In India, a dramatic increase of chronic recalcitrant dermatophytoses due to *Trichophyton* (*T*.) *mentagrophytes* genotype VIII is on the rise. Due to travelling and migration, globalization of the mostly terbinafine resistant strains of the causative agent from India to other parts of the world is assumed. First strains of *T*. *mentagrophytes* genotype VIII have reached Germany, now. **Patients**: 1) A 6 months-old-female infant from Bahrain visiting Germany with her family for a holiday was seen for extensive dermatophytosis of the back, buttocks, chest and groins. Topical treatment by terbinafine for over 2 months was not successful. 2) A 28 years old male from Libya, living for 3 years in Germany, suffered from tinea cruris and tinea faciei involving the left upper and lower eyelids. Treatment by oral fluconazole and by terbinafine failed. His German girlfriend was affected by the dermatomycosis, too. Her child was free of signs of a dermatophytosis. The patient had no contact to India and Indians or Arabs, he did not visit Libya in the last years. The man, however, regularly goes to the gym. 3) After a trip to Saudi Arabia, a pregnant German woman suffered from tinea cruris and tinea corporis (trunk). Her husband was affected, too. 4) An Indian male, living in Germany, was treated because of a dermatomycosis of the buttocks and groins by oral terbinafine, without improvement. 5) A couple from Iraq, living for long time in Germany, suffered from chronic recalcitrant dermatophytosis of the groins for at least 1.5 years. Repeated topical treatments by „Combo Creams“ failed. Antifungal topical preparations (ciclopirox olamine, sertaconazole) for 5-6 weeks acted very slowly, progression of the dermatomycoses lead to therapy break-off. Oral itraconazole 200 mg daily was started. **Results**: Mycological examination of skin scrapings from all 5 patients revealed the detection of *T*. *mentagrophytes*. Four out of the five dermatophyte isolates were subject to sequencing of the “internal transcribed spacer” (ITS) region of the fungal rDNA. All these 4 strains belonged to the newly described genotype VIII of *T*. *mentagrophytes*. The until now investigated point mutations revealed for patient 1) TTC/TTA mutation of the squalene epoxidase (SQLE) gene which is closely associated with Phe^397^Leu amino acid substitution of the enzyme. T. *mentagrophytes* isolates of patient 2) and 3) were *in vitro* sensitive to terbinafine. The *T*. *mentagrophytes* strain of patient 3) showed GCT/ACT mutation (Ala^448^Thr). The above mentioned Phe^397^Leu and Ala^448^Thr substitutions are indicative of *in vitro* resistance of the dermatophyte against terbinafine. **Conclusion**: Transmission of the here found Indian ITS genotype VIII of *T*. *mentagrophytes* to other countries due to globalization is a serious issue to be considered. Moreover, a significant percentage of these Indian *T*. *mentagrophytes* strains are resistant to terbinafine both *in vitro* and due to point mutations in the SQLE gene. Some strains are also found to be partially resistant against itraconazole and voriconazole. The here described patients represent the first reports on an infection due to terbinafine-resistant *T*. *mentagrophytes* of the ITS genotype VIII from India in Germany.

### P068. Comparison of two antifungal susceptibility tests for Malassezia yeasts

AbdillahA.^1^PackeuA.^2^KhelaifiaS.^3^HendrickxM.^4^RanqueS.^1^1Vitrome, Aix Marseille Univ, IRD, AP-HM, SSA, Marseille, France2Mycology & Aerobiology, Sciensano, Brussels, Belgium3Culturomics, IHU Méditerranée Infection, Marseille, France4Service of Mycology and Aerobiology, Bccm/ihem Fungal Collection, Sciensano, Brussels, Belgium

**Objectives:** The lipid-dependent *Malassezia* spp. yeast are members of the normal human skin microbiota that are occasionally involved in various skin diseases or in blood stream infections in immunocompromised and neonates patients. A better knowledge of the antifungal susceptibility testing (AFST) profiles of these yeast is pivotal to enhance antifungal treatment strategies. This study aimed to compare two broth microdilution assays for the in *vitro* AFST of *Malassezia* spp.

**Methods:** A total of 19 strains of the BCCM /IHEM collection, including 4 *Malassezia* species: (*n* = 6) *M. furfur*, (*n* = 4) *M. pachydermatis*, (*n* = 5) *M. sympodialis* and (*n* = 4) *M. slooffiae* were tested against ketoconazole, voriconazole, terbinafine and fluconazole using the broth microdilution method (CLSI - M27-A2). Two different media were used as diluent, modified Dixon broth (mDIXB) and FastFung broth, a new medium derived from the Schaedler agar. MIC values were visually read after 48 h of incubation at 30°C. The essential agreement (EA) (±1 or 2 dilution) of the MIC values between both media was estimated. The inter-laboratory reproducibility between Marseille and Brussels was tested.

**Results:** Ketoconazole, voriconazole and terbinafine MICs (mg/l) were low, with MIC_90_ values of 0.06 to 0.5, 0.03 to 0.125, and 0.125, in mDIXB, and of 0.03 to 0.25, 0.03 to 0.125, and 0.06 to 1, in FastFung broth, respectively. Fluconazole displayed the highest MIC values. The EAs varied from 84.2 to 100% for ±1 dilution while EAs of 100% were observed for ±2 dilution. The inter-laboratory EAs (±2 dilutions) ranged from 73.8 to 100% and 94.7 to 100% in mDIXB or FastFung broth, respectively.

**Conclusion:** Our findings showed that both media performed similarly, with relatively higher EAs (>60%), except for terbinafine. The FastFung broth can be used as alternative medium for *Malassezia in vitro* AFTS.

### P075. The effect of farnesol on Candida auris biofilms, virulence and azole susceptibility

NagyF.^1^TóthZ.^1^ForgácsL.^1^BormanA.^2^MajorosL.^1^BalázsB.^1^KovácsR.^1^1Department of Medical Microbiology, University of Debrecen, Debrecen, Hungary2Public Health England UK National Mycology Reference Laboratory, Bristol, United Kingdom

**Objectives:** Farnesol is a fungal quorum-sensing molecule, which has a pivotal role in regulation of fungal morphogenesis. The aim of our study was to examine the effect of farnesol on biofilms, azole susceptibility (fluconazole, voriconazole, itraconazole, posaconazole, isavuconazole) against *Candida auris* clinical isolates as well as *in vivo* tissue fungal burden in a murine bloodstream infection model.

**Methods:** The effect of farnesol on *C. auris* cells was tested in three experiments: i) biofilm forming ability of cells pre-treated with farnesol for 24-hours prior to biofilm formation, ii) biofilm forming ability of cells continuously exposed to farnesol for 24-hours during biofilm formation, iii) effect of farnesol on one-day-old biofilms. Farnesol concentrations tested were 10, 50, 100, 300 μM in all experiments. Metabolic activity of sessile cells was determined at 0, 2, 4, 6, 8, 10, 12 and 24 hours using XTT-assay. One-way ANOVA with Dunnett’s post-testing was used to analyze the metabolic activity change exerted by different farnesol concentrations compared to untreated control. Interactions between farnesol and azoles were examined against one-day-old biofilms by broth microdilution checkerboard method. The tested concentrations were 1.17-300 μM and 0.125-512 mg/L for farnesol and azoles, respectively. Fractional inhibitory concentration index (FICI) was used to assess the nature of interactions. Synergy was specified if the FICI was ≤0.5, between >0.5 and 4 was indifferent and as antagonistic if the FICI was >4. MICs were determined as at least 50% reduction of metabolic activity compared to control in case of individual drugs and isoeffective combinations. Farnesol pre-exposed (75 μM) cells were inoculated intravenously into immuncompromised female BALB/c mice to examine the day six fungal burden in kidneys, heart and liver.

**Results:** The one-day-long farnesol pre-exposure (50-300μM) led to formation of biofilms with significant metabolic activity inhibition after 24 hours (p<0.01). Biofilms formed under farnesol exposure showed significantly decreased metabolic activity in first the 12, 12, 10 and 8 hours for 300, 100, 50 and 10 μM farnesol, respectively (p<0.05-0.001). In case of one-day old biofilms treated with farnesol after formation, only the highest farnesol concentration caused significant inhibition at 24 hours (p<0.001).The median MIC values of azoles and farnesol against sessile *C. auris* clinical isolates are showed in Table 1. Based on FICI values, farnesol was synergistic with all tested azoles (FICIs ranged from 0.061 to 0.375) (Table 1). Fungal tissue burdens caused by farnesol pre-exposed *C. auris* were significantly higher in all examined organs compared to organs infected by farnesol-unexposed fungal cells at sixth day postinoculation.

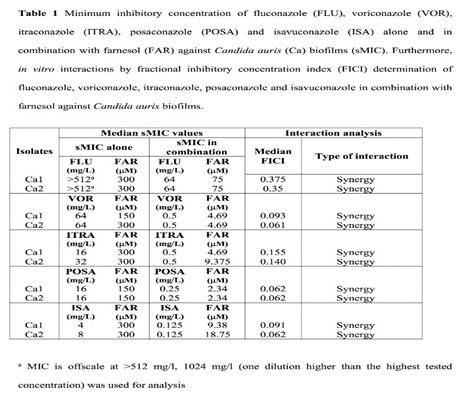



**Conclusion:** Farnesol inhibited the biofilm forming ability of *C. auris* and significantly reduced the metabolic activity of *C. auris* biofilms in a concentration-dependent manner; in addition, it showed a remarkable synergistic effect with azoles. On the other hand, farnesol pre-exposure led to increased tissue fungal burdens. In summary, though its systemic usage may be hindered by a potential enhancement of fungal virulence, farnesol may be a useful addition to therapy where biofilm formation is important, e.g. in catheter-lock therapy against *C. auris*.

### P076. Transcriptional and physiological effect exerted by tyrosol on Candida parapsilosis cells

KovácsR.^1^JakabÁ.^2^TóthZ.^1^NagyF.^1^EmriT.^2^PócsiI.^2^MajorosL.^1^1Department of Medical Microbiology, University of Debrecen, Debrecen, Hungary2Department of Molecular Biotechnology and Microbiology, University of Debrecen, Debrecen, Hungary

**Objectives:** Tyrosol has a potent antifungal activity against *Candida* species; however, the background of its antifungal mechanism is still poorly understood. We examined the effect of tyrosol at supraphysiological concentration on growth, biofilm formation, redox homeostasis, virulence as well as on fluconazole susceptibility to reveal the background of its antifungal effect. To gain further insights into the physiological consequences of tyrosol treatment we examined the caused genome wide gene expression changes using RNA-Seq.

**Methods:** CLIB 214 *Candida parapsilosis* (*sensu stricto*) strain was used and 15 mM tyrosol concentration was applied to examine physiological and transcriptional changes. Following 4 hours incubation of *C. parapsilosis* precultures, we added 15 mM tyrosol to the cultures then growth was examined at 1-hour intervals by determination of cell density both by means of measuring of the absorbance (at 640 nm) and by counting the living cells. Reactive species were measured in the presence or absence of tyrosol by a technique that converts 2’,7’dichlorofluorescin-diacetate to 2’,7’–dichlorofluorescein. One-day-old biofilm formation ability in the presence of tyrosol was determined by quantitative CFU determination of adhered cells. Interaction between fluconazole and tyrosol was evaluated by two-dimensional broth microdilution chequerboard assay. Afterwards, the nature of interaction was analyzed using Fractional Inhibitory Concentration Index determination. Tyrosol induced effect on virulence was examined in immuncompromised systemic BALB/c mouse model.To obtain global transcriptome data regarding tyrosol treatment, high-throughput mRNA sequencing was performed on Illumina NextSeq sequencing platform.

**Results:** Fifteen mM tyrosol caused significant growth inhibition within two hours of the addition of tyrosol (p<0.01). Tyrosol increased the production of reactive oxygen species (p<0.001) and it was associated with elevated superoxide dismutase, glutathione peroxidase and catalase activities (p<0.05). Biofilm formation ability was comparable in the presence or absence of 15 mM tyrosol in terms of living fungal cells.The interaction between fluconazole and tyrosol was antagonistic (FICI 4.5). Fifteen mM daily treatment decreased the fungal tissue burden in the kidneys compare with untreated control (p<0.01).Tyrosol exposure resulted in 1,462 differentially expressed genes. Out of them, 261 and 181 genes with at least 1.5-fold increase or decrease in expression, respectively, were selected for further investigations (Figure 1). Genes involved in ribosome biogenesis showed down-regulation, while genes related to later biofilm events, oxidative stress response and ethanol fermentation were up-regulated. In addition, tyrosol treatment up-regulated the expression of certain fluconazole efflux pump genes including *MDR1* and *CDR1* and down-regulated the *FAD2* and *FAD3* virulence genes belonging to desaturated fatty acid formation. 

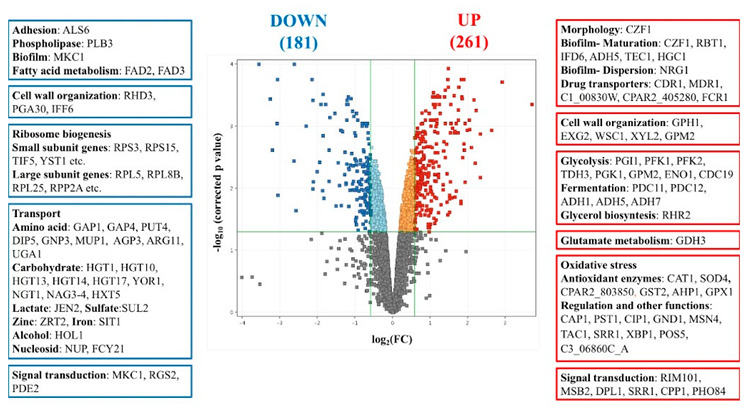



Figure 1 Up-regulated (red) and down-regulated (dark blue) genes were defined as differentially expressed genes (p<0.05) where log_2_(FC)>0.585 or log_2_(FC)<–0.585, respectively, and FC stands for fold change FPKM (fragments per kilobase per million mapped fragments) value.

**Conclusion:** Tyrosol exposure enhanced oxidative stress response and efflux pumps, while inhibited growth and ribosome biogenesis as well as virulence. Metabolism was modulated towards glycolysis and ethanol fermentation. Primer adherence was inhibited, and rather later biofilm events was enhanced. Our findings suggest that tyrosol may be a potential locally active and/or adjuvant agent in the development of alternative treatments targeting quorum-sensing in the future.

### P077. Nonyl 3,4-dihydroxybenzoate as a potent anti-biofilm compound for the treatment of dermatophytosis

OrlandiC.^1^PiresR.H.^2^BilaN.M.^1^Serafim-PintoA.^1^BonattiJ.L.^1^ScorzoniL.^1^SingulaniJ.^1^SantosC.^1^AndradeC.R.^3^SilvaP.^4^ChorilliM.^4^RegasiniL.O.^5^AlmeidaA.M. Fusco^1^GianniniM.J. Soares Mendes^1^1Clinical Analysis, UNESP (Sao Paulo State University), Araraquara, Brazil2Health Promotion, UNIFRAN (University of Franca)), Araraquara, Brazil3Physiology and Pathology, UNESP (Sao Paulo State University), ARARAQUARA, Brazil4Drugs and Pharmaceuticals, UNESP (Sao Paulo State University), Araraquara, Brazil5Chemistry and Environmental Sciences, UNESP (Sao Paulo State University), São José do Rio Preto, Brazil

**Objectives:** This study aimed to verify the *in vitro* susceptibility of nonyl 3,4-dihydroxybenzoate against planktonic cells and biofilms of dermatophytes. In addition, efficacy and toxicity were tested in a murine model of dermatophytosis. The compound was incorporated into nanostructured lipid systems (NLS) in order to prove whether the incorporation was able to maintain its antifungal activity as well as, reduce toxicity in human cell lines and alternative models. In addition, the probable mechanism of action was evaluated.

**Methods:** The preformed biofilms and planktonic cells of *Trichophyton rubrum* and *T. mentagrophytes* were treated with different concentrations of synthetic antifungal drugs and nonyl 3,4-dihydroxybenzoate. The metabolic activities were determined using the XTT reduction assay. The effects of the treatments on biofilms structures were visualized by scanning electron microscopy (SEM). The efficacy of the compound was evaluated in wild type (WT) C57BL/6 mice by determining the fungal burden and histopathological analysis. Hepatic and renal toxicity were assessed by blood analysis in the equipment Vitros 250 chemistry analyzer. After incorporation into NLS, susceptibility tests were conducted according the document M38-A2, proposed by the CLSI (2008). The toxicity of incorporation was evaluated in HaCaT cell lines by the sulforhodamine B method and in *Caenorhabditis elegans* model. Finally, the damage of cell ultrastructure was evaluated by transmission electron microscopy (TEM).

**Results:** Both biofilms and planktonic cells were susceptible to nonyl compound in concentrations ranging from 125 – 250 mg/L. *T. rubrum* and *T. mentagrophytes* biofilms were significantly more resistant to fluconazole, griseofulvin and terbinafine than planktonic cells (p<0.05). Fluconazole, griseofulvin, terbinafine and nonyl were able to prevent biofilm formation in all reference strains and clinical isolates tested (p<0.05). SEM results revealed that the antifungal drugs caused minor or no damage to the structure of the biofilms, and in some cases, stimulated the development of resistance structures. In contrast, the biofilms treated with the nonyl compound were seriously damaged. The fungal burden was significantly reduced after treatment and histopathological analysis showed complete tissue regeneration. In addition, there was no systemic toxicity after treatment in mice. Nonyl incorporated to NSL formed by the surfactant Brij®98 Epikuron® (98-I) presented the best results of minimum inhibitory concentration (MIC), with values between 1.98 and 3.9 mg/L. Cytotoxicity tests indicated that the incorporation resulted in cell viability greater than 80% at all tested concentrations. These results corroborated with *in vivo* testing in *C. elegans* model, which indicated that the incorporation of nonyl significantly increased the survival of larvae. Finally, TEM results indicated that the compound might have more than one mechanism of action in the dermatophytes, causing enlargement of the vacuoles, besides derangement and formation of pores in the plasma membrane, similar to amphotericin B.

**Conclusion:** These findings show that nonyl 3,4-dihydroxybenzoate may be a promising antifungal. Funding: CNPq, Capes and FAPESP Process # 2018/02785-9; 2017/18388-6.

### P078. Anti-dermatophyte biofilm activity of photodynamic therapy using 2-chalcone as photosensitizer

BilaN.M.^1^,^2^OrlandiC.^1^GonçalvesL.C.^1^VasoC.O.^1^SantosC.^1^SilvaP.^3^ChorilliM.^3^RegasiniL.O.^4^PrimoF.L.^5^FontanaC.R.^1^AlmeidaA.M. Fusco^1^GianniniM.J. Soares Mendes^1^1Clinical Analysis, UNESP (Sao Paulo State University), Araraquara, Brazil2Para-clinics, UEM (Eduardo Mondlane University), Maputo, Mozambique3Drugs and Pharmaceuticals, UNESP (Sao Paulo State University), Araraquara, Brazil4Chemistry and Environmental Sciences, UNESP (Sao Paulo State University), São José do Rio Preto, Brazil5Bioprocess and Biotechnology, UNESP (Sao Paulo State University), Araraquara, Brazil

**Objectives:** This study aimed to evaluate the anti-dermatophytic and anti-biofilm activity of 2-chalcone alone and combined with photodynamic therapy (PDT).

**Methods:** Strains of *Trichophyton rubrum*, *T. mentagrophytes* and one clinical isolate of *Microsporum canis* were used. Susceptibility tests were conducted according to the M38-A2 document proposed by CSLI. Planktonic cells, biofilms with 24 h (initial stage) and 72- 96 h of growth (mature) were formed in RPMI-1640 medium and placed in contact with different concentrations of 2-chalcone (1 - 500 mg/L), fluconazole (1,25 - 512 mg/L) and terbinafine (0.002 – 32 mg/L). The metabolic activities after the treatments were quantified using the tetrazolium salt (XTT) reduction method. The topographies of initial and mature biofilms with or without treatment were visualized by scanning electron microscopy (SEM). For the PDT assay, 2-chalcone was used a photosensitizer and a blue led as a source of light with a dose of 150 J.cm^-2^.

**Results:** All the strains, in the planktonic form, were inhibited by treatment with 2-chalcone (MIC = 3.9–7.8 mg/L), terbinafine (MIC = 0.008–0.03 mg/L) and fluconazole (1–64 mg/L). The substance 2- chalcona eradicated the 24 hour biofilm of all strains tested at the concentration of 15.6 mg/L, terbinafine ranging from 0.06 to 16 mg/L, and fluconazole inhibited only the formation of *T. mentagrophytes* biofilm in the concentration of 32 mg/L. Regarding mature biofilms, only 2-chalcone was able to reduce the metabolic activities in the concentration of 31.2 mg/L. Mature biofilms were resistant to both antifungal drugs tested. When planktonic and biofilms (initial and mature) were treated with PDT using 2-chalcone as photosynthesizer, the inhibitory concentrations were reduced by 4 times (2–8 mg/L). The SEM of biofilms treated with 2-chalcone showed a total collapse of the cell walls, resulting from a probable extravasation of cytoplasmic content.

**Conclusion:** The substance 2-chalcone is a promising antifungal drug. Funding: CNPq, IBE process 150/2017, Capes and FAPESP Process # 2018/02785-9; 2017/18388-6.

### P079. Antifungal activity of voriconazole and micafungin against the biofilms and planktonic cells of Candida albicans and Aspergillus fumigatus in vitro

ShiD.^1^LiuW.^2^1Jining No. 1 People’s Hospital, Jining, China2Institute of Dermatology, Chinese Academy of Medical Sciences & Peking Union Medical College, Nanjing, China

**Objectives:** *Candida albicans* and *Aspergillus fumigatus* are among the most common invasive opportunistic pathogens known to have high morbidity and mortality. Micafungin (MCF) and voriconazole (VOR) are the most commonly used first-line therapies for these fungi. However, to our knowledge, there are few data comparing the efficacy of MCF and VOR on biofilm and planktonic cells on both fungi.

**Methods:** We have carried out an *in vitro* study to evaluate the efficacy of VOR and MCF against biofilm development and inhibition of fungal viability within biofilm formed by these two pathogenic fungi.

**Results:** We find that, when compared with planktonic cells, the corresponding fungal biofilm magnificently decreases drug sensitivities. To inhibit more than 50% of cell viability within a mature biofilm, the concentration of each drug must increase by at least 5 to 10 MIC – depending on the specific drug and fungus combination. With specific regard to biofilm formation itself, we find that MCF is more effective than VOR against developing biofilm in *C. albicans*, whereas VOR is more effective at high concentration against developing biofilm in *A. fumigatus*. We also find that the earlier the therapeutic intervention, the greater the biofilm inhibition that will be achieved. The critical time window for biofilm inhibition is 12h for *A. fumigatus* and 8h for *C. albicans*; beyond those points in time safe and effective drug intervention is almost impossible.

**Conclusion:** Our results provide a few clinical hints that may help in choosing antifungal agents when treating common fungal infections.

### P080. Contribution of Galleria mellonella model to understand interactions between Aspergillus fumigatus and Stenotrophomonas maltophilia: study of human, animal and environmental strains.

DurieuxM.-F.^1^,^2^MelloulÉ.^2^RoisinL.^2^WoertherP.-L.^2^,^3^RiscoV.^2^,^4^GuillotJ.^2^,^4^DardéM.-L.^1^DannaouiE.^5^DecousserJ.-W.^2^,^3^BotterelF.^2^,^6^1Hopital Dupuytren, Laboratoire de Parasitologie-Mycologie, Limoges, France2Dynamyc, Créteil, France3Microbiology, Hôpital Henri-Mondor, Créteil, France4École Nationale Vétérinaire D’alfort, Unité de Parasitologie-Mycologie, Maisons-Alfort, France5Hôpital Européen Georges Pompidou, Laboratoire de Parasitologie-Mycologie, Paris, France6Laboratoire De Parasitologie-mycologie, APHP Hôpital Henri Mondor, Créteil, France

**Objectives:** Clinical and environmental strains of *Aspergillus fumigatus* (Af) and *Stenotrophomonas maltophilia* (Sm) can colonize the respiratory tract of patients with chronic lung diseases such as cystic fibrosis, specially in biofilm form. The interactions between these two pathogens are not well known (1, 2). For a decade, insect model *Galleria mellonella* (Gm) can be used to screen microorganism pathogenicity. The aim of this study was to compare several associations of Af – Sm strains of different origin in a Gm model to determine the virulence of these associations.

**Methods:** An *in vivo* infection model with human, animal and environmental strains of Af and Sm, alone and associated, was developed using larvae of Gm. Several couples were tested using different strains of Sm and Af, especially human reference clinical strains (Af13073 and Sm13637). Virulence was analysed by survival curves at 7 days and histology method.

**Results:** Mortality rate with Af alone showed no significant difference between human, animal and environmental strains. For Sm, mortality rates showed variations depending on the strains. The association between reference clinical strains Sm13637 and Af13073 showed a synergistic effect in Gm (60% of mortality at 7 days) versus Sm 13637 alone (10% mortality) and Af13073 alone (30% mortality). This effect was not observed with the other co-infections. Gomori-Grocott coloration of histological sections showed fungal nodules disseminated in the larvae and a Gram coloration demonstrated the presence of the bacteria, validating our infection model (3).

**Conclusion:** For the first time, we described a coinfection of Af and Sm in a Gm model with an interesting synergistic effect between human clinical strains. This effect did not exist for environmental and animal strains showing a particular fitness between the human strains. These preliminary results provide complementary data to the in *vitro* data already obtained. The Gm model has demonstrated its relevance for the study of mixed infections, which remains to be improved (2). Indeed, the extracellular matrix could not be identified in Gm, which could validate the presence of mixed biofilm. (1, 2) Melloul et al. 2016 and 2018 (3) Société Française de Mycologie Médicale price of the best oral communication, 2019.

### P081. Antifungal susceptibility of Lictheimia corymbifera biofilms to Amphotericin B formulations and Posaconazole

VikeloudaA.^1^SimitsopoulouM.^1^AntachopoulosC.^1^SkouraL.^2^ChatzimoschouA.^1^StamouliI.^1^RoilidesE.^1^13rd Department of Pediatrics, Aristotle University of Thessaloniki, Thessaloniki, Greece2Department of Microbiology, Aristotle University, Thessaloniki, Greece

**Objectives:** Mucormycosis is an emerging life-threatening opportunistic fungal infection that affects patients with underlying immunosuppressive conditions, such as hematological malignancies, trauma, hematopoietic stem cell transplantation and diabetes mellitus. The ability of *Mucorales* to form biofilms, structures with reduced susceptibility to antifungal agents, has recently been demonstrated., *Lictheimia Corymbifera* is the second most common clinically significant *Mucorales* species and is related mostly to pulmonary and trauma infections. We investigated the antifungal activity of deoxycholate amphotericin B (D-AMB), liposomal amphotericin B (L-AMB), posaconazole (POS) against planktonic cells and mature biofilms of *Lichteimia corymbifera*. Additionally, we investigated the combinational activity of L-AMB with POS against mature biofilms.

**Methods:** Three *L. corymbifera* clinical strains, were isolated from bronchial secretions and incubated at 10^5 cfu/ml in 96-well microtiter plates at 37^ο^C for 48h. Biofilm formation was assessed by 1% safranin staining and measured spectrophotometrically at 490 nm. In order to determine the MIC, two-fold dilutions of D-AMB, L-AMB and POS (0.007-256 mg/l) were incubated with biofilms or planktonic cells (2x10^5 cfu/ml) for 24h (*n* = 9). XTT metabolic reduction assay was used to assess biofilm damage compared to controls. MIC50 was determined as ≥50% biofilm damage. The activity of the combination of L-AMB (0.5-32 mg/l) with POS (0.125-64 mg/l) against biofilms was determined using a checkerboard microdilution method (*n* = 10). Drug interactions were analyzed using Bliss independence model. The combination effect was defined as synergistic, antagonistic or indifferent when the observed biofilm damage was significantly higher, lower or equal to the expected damage, respectively.

**Results:** All *L. corymbifera* strains exhibited strong biofilm formation. Biofilm MIC50 of D-AMB, L-AMB and POS were 2, 4 and >256 mg/l, respectively, as compared to 0.007, 0.03 and 0.03 mg/l for planktonic cells. Synergistic effects were observed at 1-2 mg/l of L-AMB combined with 8-16 mg/l of POS (mean ΔE value of significant interactions:19% [range: 17% to 21%]; mean SE:1% [range: 1% to 3%]). None of the combinations exhibited antagonism.

**Conclusion:** *L. corymbifera* can produce strong biofilms that possess comparable susceptibility profiles to D-AMB and L-AMB, but are resistant to POS. The combination of L-AMB with POS at super-therapeutic concentration ranges exhibits synergistic activity against mature *L. corymbifera* biofilms.

### P082. Biofilm formation by Histoplasma capsulatum in different culture mediums and oxygen atmospheres

GonçalvesL.C.^1^OrlandiC.^1^BilaN.M.^1^,^2^VasoC.O.^1^SilvaR.A. Moraes Da^1^TaylorM.L.^3^GianniniM.J. Soares Mendes^1^AlmeidaA.M. Fusco^1^1Clinical Analysis, UNESP (Sao Paulo State University), Araraquara, Brazil2Para-clinics, UEM (Eduardo Mondlane University), Maputo, Mozambique3Microbiology and Parasitology, UNAM (Autonomous University of Mexico), México, D.F., Mexico

**Objectives:** This work aimed to study *Histoplasma capsulatum* biofilms, by checking the influence of different culture media and oxygen atmospheres in the development of these communities.

**Methods:** The biofilm formation by two strains (ATCC 26029 – G186A and EH315) was characterized in different nutrient conditions [Brain Heart Infusion (BHI), Roswell Park Memorial Institute (RPMI) with 2% of glucose, Dulbecco’s Modified Eagle Medium (DMEM) supplemented with 10% fetal bovine serum and nutrient medium HAM-F12 (HAM-F12) supplemented with glucose (18.2 g/L), glutamic acid (1 g/L), HEPES (6 g/L), and L-cysteine (8.4 mg/L)] and atmospheres of oxygen (aerobic and microaerophilic). Optical microscopy techniques, XTT reduction assay, as well as crystal violet and safranin staining, and scanning electron microscopy (SEM) were performed.

**Results:** The results indicated that, although all the culture media stimulated the maturation of the communities, the nutrient-rich culture media, such as HAM-F12, provided a better biomass and extracellular matrix development when compared to the others. In addition, microaerophilic conditions were the most favorable than aerobic. The topographies observed in SEM showed yeasts surrounded at several points by an exuberant amount of extracellular matrix. However, the biofilms formed in BHI, RPMI and DMEM presented a reversal yeast for significant filamentation, which needs better investigation.

**Conclusion:** The results obtained so far represent significant advances for the field of biofilms and open new possibilities for the study of virulence and *Histoplasma*-host interaction. Funding: FAPESP (Fundação de Amparo à Pesquisa do Estado de São Paulo Process # 2016/11836-0); CNPq (Conselho Nacional de Desenvolvimento Científico e Tecnológico Process # 130018/2018-0) and Capes (Coordenação de Aperfeiçoamento de Pessoal de Nível Superior).

### P085. Combined treatment of experimental paracoccidiodomycosis with Itraconazole and low-level LASER therapy

BurgerE.^1^GrisoliaJ.C.^1^SantosL.A.^1^DiasN.A.^1^MalaquiasL.C.C.^1^RodriguesA.M.^2^CamargoZ.P.D.^2^1Department of Microbiology and Immunology, Federal University of Alfenas, Alfenas, Brazil2Department of Microbiology, Immunology and Parasitology, Federal University of Sao Paulo, Alfenas, Brazil

**Objectives:** Paracoccidioidomycosis (PCM) is a systemic mycosis caused by the fungus *Paracoccidioides brasiliensis* (Pb), requiring long treatment, justifying studies that amplify the therapeutic options. Itraconazole (Itra) is effective for PCM and requires shorter therapy than other drugs. We propose to study a shorter treatment model with fewer side effects through the activation of neutrophils by the simultaneous administration of the antifungal drug Itraconazole and adjuvant therapy with Low Level LASER Therapy (LLLT) to provide better control in the early stages of the infection, leading to a more efficient and protective immune response, resulting in the control or cure of this disease.

**Methods:** We used a highly purified PMNs preparation by inoculating Pb in subcutaneous air pouches of mice. We evaluated the effect of Itraconazole (Itra) therapy and/or LLLT therapy on their size, cellular composition, mitochondrial activity, protein metabolism, production of reactive oxygen (ROS) and nitrogen (NO) species, production of IL-10, INF-y and TNF-α cytokines, and on tissue architecture and number of viable fungi.

**Results:** The viability of air pouch cells was always greater than 70%, therefore, none of the treatments had adverse effects, and about 80% of the cells were PMN. Mitochondrial activity and cell counts differed significantly between LLLT-treated and controls and between mice treated with different Itra concentrations, with or without LLLT. Cells from 10mg/mL Itra-treated mice produced the highest levels of ROS followed by those of 3mg/mL. When Itra was associated with LLLT treatment, we found an increase in NO production in the 3mg/mL group. Total protein levels were higher when Itra was used, compared to the untreated group or to the group treated with LLLT alone, in which LLLT increased protein production in the 3mg/mL group and decreased in the 10mg/mL group. LLLT increased the production of cytokines IL-10, INF-y and TNF-α, and the use of antifungal decreased their production at all doses, both using only Itra treatments and in combination with LLLT. The highest dose of Itra (50mg/mL) resulted in a significant reduction of air pouch size, volume and weight. The intensity of the inflammatory process and the number of vessels and neovas decreased when the concentration of Itra in combination with LLLT increased. The numbers of viable Pb were significantly reduced when Itra was administered in a dose-response way and more markedly when used in combination with LLLT.

**Conclusion:** Pb numbers decreased significantly when Itra was given at all concentrations, and this effect increased when used in combination with LLLT. Itra at 50 mg/mL caused a significant reduction in air pouch, indicating a dose-response control. Our results strongly suggest that the efficiency of Itra is enhanced by LLLT. Therefore, we could detect a possible additive or synergistic effect of PMNs, the antifungal drug Itra and LASER therapy, suggesting a new, more effective treatment for PCM. Grants: CNPq 305216/2016-3 and FAPEMIG APQ 012941-16. J.C.Grisolia and L.A. Santos are recipients of CAPES scholarships.

### P086. Clinical outcome of chronic pulmonary aspergillosis patients managed surgically

SetianingrumF.^1^,^2^Rautemaa-RichardsonR.^1^,^3^,^4^ShahR.^4^,^5^DenningD.^1^,^3^,^4^1Division of Infection, Immunity and Respiratory Medicine, University of Manchester, Manchester, United Kingdom2Department of Parasitology, Universitas Indonesia, Jakarta, Indonesia3National Aspergillosis Centre, Manchester University NHS Foundation Trust, Manchester, United Kingdom4Manchester Academic Health Science Centre, Manchester, United Kingdom5Department of Cardiology and Cardiothoracic Surgery, Manchester University NHS Foundation Trust, Wythenshawe Hospital, Manchester, United Kingdom

**Objectives:** Surgery is one of the treatment options for chronic pulmonary aspergillosis (CPA), especially for single aspergilloma, for those with recurrent haemoptysis despite bronchial artery embolisation or with the unilateral azole-resistant disease. This study aims to evaluate the outcomes of patients managed surgically and the correlation between *Aspergillus* Immunoglobulin G (IgG) levels and response to treatment.

**Methods:** This was a retrospective cohort study evaluating CPA patients who were treated at National Aspergillosis Centre, Manchester, UK over an 11-year period (2007-2018). Clinical variables including antifungal therapy and *Aspergillus*-IgG (Thermo Fisher/Phadia) were recorded and correlated with surgical success or relapse. *Aspergillus*-IgG were analysed based on 37 patients with complete serology database.

**Results:**

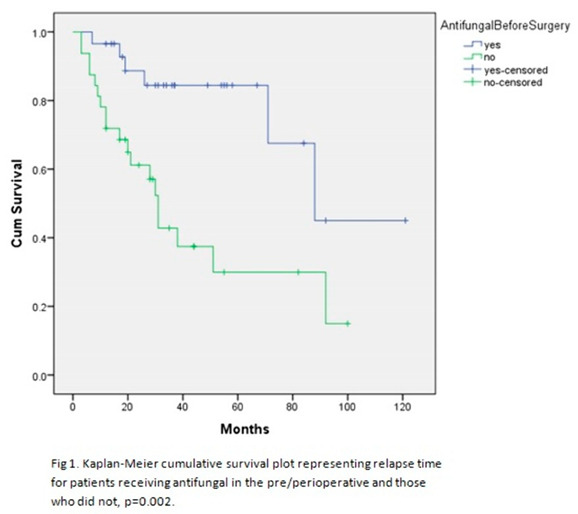



61 patients with CPA underwent surgery with a mean duration of follow-up of 36 months. The mean age was 52.8 (± 14.3) years. The most common presenting symptoms pre-operatively were cough (*n* = 41, 67%), haemoptysis (*n* = 24, 39%), dyspnea (*n* = 23, 38%) and 5 patients (8%) were asymptomatic. Failed medical therapy (*n* = 31, 51%) and presumed malignancy (*n* = 23, 38%) were the most common indications for surgery. The most common sign of failed medical therapy was recurrent haemoptysis (*n* = 19, 31%). Twenty-six patients (43%) had single aspergilloma, 23 patients (38%) showed chronic cavitary pulmonary aspergillosis (CCPA) while 19 patients (31%) had one or more *Aspergillus* nodule. Main underlying diseases were chronic obstructive pulmonary disease (*n* = 19, 31%), asthma (*n* = 18, 30%), bronchiectasis (*n* = 14, 23%) and previous pneumothorax (*n* = 13, 21%). The procedures included lobectomy (*n* = 39, 64%), wedge resection (*n* = 17, 28%), segmentectomy (*n* = 3, 5%), pneumonectomy (*n* = 3, 5%) and decortication (*n* = 2, 3%). Main surgical complications were pleural infection (*n* = 7, 12%), prolonged air leak (*n* = 3, 5%) and persistent pneumothorax (*n* = 3, 5%). Assessed 6-8 months after surgery, 21 patients were asymptomatic (34%), 23 experienced cough (38%), 6 patients (10%) had haemoptysis, and the frequency of these symptoms reduced significantly after surgery (p< 0.05). Relapse occurred in 25 patients (41%) while the rest of the patients (*n* = 36, 59%) showed no sign of relapse. Haemoptysis, cough and dyspnea decreased significantly in the non-relapse group, whereas only haemoptysis was initially less frequent in relapse group (p<0.05). In this relapse group, 6 (24%) patients received antifungal before surgery compared to 23 (64%) in the non-relapse group (p = 0.002). Perioperative antifungal therapy was administered to 5 (20%) patients who relapsed and 19 (53%) who did not (p = 0.01). The median *Aspergillus*-IgG in the non-relapse group (*n* = 12, 32.4%) before surgery was 75 mg/L decreasing to 52 mg/L in the end of follow-up (p = 0.003). The median survival time (free-relapse) was 88 months in patients with antifungal therapy before surgery and 31 months in patients without antifungal therapy before surgery (p = 0.002).

**Conclusion:** Surgery in these selected CPA patients resulted in favourable outcomes with a decrease in clinical symptoms and the *Aspergillus*-IgG level of the non-relapse group of CPA. Pre and perioperative antifungal therapy reduced the likelihood of CPA relapse, but confounding factors may contribute to this. The diagnosis of CPA before surgery is important to optimise pre and perioperative antifungal therapy in patients to improve prognosis.

### P087. The all new, updated antifungal drug interaction database at www.aspergillus.org.uk The all new, updated antifungal drug interaction database at www.aspergillus.org.uk The all new, updated antifungal drug interaction database at www.aspergillus.org.uk

Niazi-AliS.^1^DenningD.^2^,^3^AthertonG.^2^,^3^WalczakM.^3^,^4^1Fungal Interaction Trust, Macclesfield, United Kingdom2University of Manchester, Manchester, United Kingdom3National Aspergillosis Centre, Manchester University NHS Foundation Trust, Manchester, United Kingdom4Manchester Academic Health Science Centre, Manchester University NHS Foundation Trust, Wythenshawe, United Kingdom

**Objectives:** A drug-drug interaction describes the influence of one drug upon another or the change in a drug’s effect on the body when the drug is taken together with a second drug. A drug-drug interaction can delay, decrease, or enhance absorption or metabolism of either drug. The antifungal drug interaction database was first launched in 2012. It is available as web (www.aspergillus.org.uk/content/antifungal-drug-interactions) and app versions to allow information on potential drug interactions with antifungals with a version for patients and another for health professionals. New drugs are constantly being launched and approved. In this era, with the requirement of speed and accurate information due to pressures on all healthcare professionals, a new and updated database with apps (on Apple and Android platforms) has been created. This allows all of the above with the added benefit of real time updates in a more time efficient manner.

**Methods:** Horizon scanning documents were reviewed from 2017, 2018 and 2019 to date. Drugs that were not in the current database were identified and interactions determined using the Summary of Product Characteristics together with further exploration, as needed, with review of papers. CYP P450 and p-glycoprotein are the major interaction pathways where antifungal drugs are involved. However other pathways like OATP1B1 and BCRP were also explored, as there is new and emerging information on the effect of these pathways with drug-drug interactions.

**Results:**

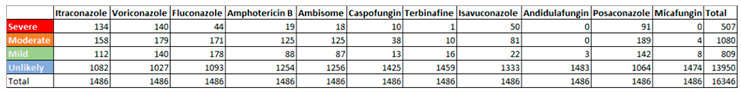



134 new preparations were identified to April 2019 leading to updated information. 16 classed as red (severe interactions with some antifungals), 29 as amber (moderate interactions where co-administration is possible and guidance on any management required) and 89 as green (interaction unlikely). These were added to the database together with references to ensure the user is able to, refer and, find references with ease to validate their decisions. of 16,324 possible interactions for drugs licensed by the end of 2018, 507 are potentially severe (3.1%), 1080 (6.6%) moderate and 809 mild interactions (4.9%). The antifungals with the least interactions are anidulafungin (*n* = 3 mild) and micafungin (*n* = 4 moderate & 8 mild) which contrasts with voriconazole with 459 interactions (140 severe) and itraconazole with 404 interactions (134 severe).

**Conclusion:** The new and revised version of the web and smartphone app-based database of these drug interactions with antifungal agents is now available. This will allow clinicians to effectively evaluate the use of antifungals with all the new drugs on the market and allow the best outcome for their patients. It will also enable them to make the most effective and evidence-based decisions for those drugs that are co-administered and require a little more clinical management to provide the most successful treatment for the wellbeing of their patients.

### P088. Posaconazole Serum Drug Levels Associated with Hypertension and Hypokalemia: The Syndrome of Pseudohyperaldosteronism

NguyenM.-V.^1^DavisM.^1^WittenbergR.^1^MchardyI.^1^BaddleyJ.^1^YoungB.^1^OdermattA.^2^ThompsonG.^1^1Infectious Disease, University of California at Davis, Davis, United States of America2Toxicology, University of Basel, Basel, Switzerland

**Objectives:** Posaconazole tablets are well tolerated and efficacious in the prophylaxis and treatment of aspergillosis, mucormycosis, and other invasive fungal infections There have been case reports of posaconazole-induced pseudohyperaldosteronism, however, its occurrence and association with serum posaconazole drug levels has not previously been investigated.

**Methods:** In this single-center retrospective observational study, we examined the occurrence of posaconazole-induced pseudohyperaldosteronism (PIPH) in outpatients newly starting posaconazole and evaluated differences in serum posaconazole concentrations and clinical characteristics between those with and without this syndrome.

**Results:** Sixty-nine patients receiving posaconazole were included, of whom 16 (23.2%) met the definition of PIPH. Patients with PIPH were significantly older (61.1 vs 44.7 years, *P* = 0.007) and more frequently had hypertension prior to starting posaconazole (68.8% vs 32.1%, *P* = 0.009). Patients with PIPH had a significantly higher median serum posaconazole level than those without PIPH (3.0 vs 1.2 µg/mL, *P <* = 0.0001). There was a positive correlation between serum posaconazole levels and changes in systolic blood pressure (*r* = 0.37, *P* = 0.01), a negative correlation between serum posaconazole levels and changes in serum potassium (*r* = -0.39, *P* = 0.006), and a positive correlation between serum posaconazole levels and serum 11-deoxycortisol (*r* = 0.69, *P* < 0.0001).

**Conclusion:** Posaconazole is associated with secondary hypertension and hypokalemia, consistent with pseudohyperaldosteronism, and development is associated with higher serum posaconazole concentrations, older age, and baseline hypertension.

### P089. A case of primary joint infection caused by Candida pelliculosa

KimS.-H.^1^SongK.Y.^2^ChoiJ.-H.^1^1Department of Internal Medicine, College of Medicine, The Catholic University of Korea, Seoul, Republic of Korea2Department of Orthopaedic Surgery, College of Medicine, The Catholic University of Korea, Seoul, Republic of Korea, Seoul, Republic of Korea

**Case Report:** *Candida* joint infection is most often due to hematogenous seeding during an episode of candidemia in immunocompromised patients. Exogenous inoculation after trauma or surgical procedures also can lead to infection, but primary joint infection caused by *Candida* species is extremely rare. Until now, some *Candida* joint infections by *Candida albicans, Candida krusei, Candida lusitaniae, Candida parapsilosis,* and *Candida tropicalis* have been reported. Herein, we report the first case of primary knee joint infection caused by *Candida pelliculosa* without predisposing factors. A 75-year-old woman came to our hospital with a 3-year history of pain and swelling of the left knee, with the pain particularly aggravated for the past 1 month. The patient had undergone a left total knee arthroplasty (TKA) for incapacitating knee pain at a local hospital 32 months ago. At that time, *C. pelliculosa* was isolated from the tissue taken by intraoperative biopsy, but no antifungal treatment was performed. One year after TKA, *C. pelliculosa* was repeatedly isolated from left knee synovial fluid and the patient was treated with intravenous amphotericin B deoxycholate (0.5 mg/kg/day) for 4 weeks, then intravenous fluconazole (6 mg/kg/day) for 3 weeks, followed by oral fluconazole (4 mg/kg/day) for 2 weeks. However, progressive bone loss around prosthetic components was seen on follow-up radiographs and the patient was referred to tertiary hospital. The patient had no other significant medical history. The patient was admitted to our hospital and arthrocentesis of the left knee was performed. Results of synovial fluid analysis were as follows: leukocyte, 608 cells/μL (neutrophil, 54%); protein, 5.9 g/dL; glucose, 7 mg/dL. Magnetic resonance imaging showed a large abscess around the antero-lateral aspect of the left knee. Fungal culture of synovial fluid reported as *C. peilliculosa* susceptible to amphotericin B (minimum inhibitory concentration [MIC], ≤0.25 mg/L), caspofungin (MIC, ≤0.25 mg/L), fluconazole (MIC, 2 mg/L), micafungin (MIC, ≤0.06 mg/L), and voriconazole (MIC, ≤0.12 mg/L) by Vitek 2 yeast identification system. The isolate was confirmed as *C. pelliculosa* using Internal transcribed spacer 1 and 4 region PCR and sequencing. At 2 weeks of micafungin (100 mg/day) therapy, the patient underwent prosthesis resection with debridement of soft tissue and bone, total synovectomy, and placement of an antibiotic-impregnated spacer. Operative findings showed infected granulation tissue in whole joint space and bony fistula extended to the anterolateral aspect of the lateral tibial component. Postoperatively, the patient was given micafungin for 4 weeks, followed by oral fluconazole (6 mg/kg/day). There were no signs of infection around the left knee and the patient was discharged from the hospital. Oral fluconazole will be given for at least 6 months and implantation of new prosthesis is planned at 3 months after the stage 1 procedure. Rare *Candida* species could be a causative organism of primary joint infection. Even a single colony of *Candida* on synovial fluid or bone biopsy should be considered as pathogenic, and the patient should be treated with antifungal agents. A high index of suspicion is required to establish the diagnosis.

### P090. Transdiaphragmatic Mucormycosis

KöhlerP.^1^ReimerR.^2^WhabaR.^3^Schömig-MarkiefkaB.^4^CornelyO.A.^1^1Department I For Internal Medicine, European Excellence Center For Medical Mycology (ecmm), Germany and Cecad Cluster of Excellence, University of Cologne, Cologne, Germany2Department of Diagnostic and Interventional Radiology, University Hospital Cologne, Cologne, Germany3Department of General, Visceral and Cancer Surgery, University Hospital of Cologne, University of Cologne, Cologne, Germany4Institute of Pathology, University Hospital Cologne, Cologne, Germany

**Objectives: Introduction** Mucormycosis is a life-threatening infection in immunocompromised patients and in haematological malignancy patients in particular. **Objectives** To report the phenomenon of transdiaphragmatic mucormycosis and to raise concerns with regard to the extent of imaging studies in patients with mucormycosis.

**Methods:** Retrospective chart review was performed on patients with Mucormycosis treated at the University Hospital of Cologne between January 2011 and January 2019.

**Results:** In a series of three patients we observed a previously unreported contiguous disease pattern, where the pulmonary type of disease contiguously spread through the diaphragm into abdominal organs, mostly liver or spleen. Patient 1, a 53-year-old women with history of B-NHL, ASCT and chronic renal disease developed mucormycosis of the lungs with continuous growth through the diaphragm into the liver. Histology showed invasive growth of fungal hyphae of the Mucorales order in liver and diaphragm. Patient 2, a 35-year-old man with history of AML and no further underlying conditions developed mucormycosis of the lungs with continuous growth through the diaphragm into the spleen. Splenectomy was performed and histology showed invasive growth of fungal hyphae. Fungal cultures of the surgical sample remained negative, but panfungal PCR resulted in *Rhizomucor* spp. Patient 3, a 42-year-old man with history of poorly controlled IDDM and injection drug use developed mucormycosis of the lungs with continuous growth through the diaphragm into the liver. Fungal culture of BAL fluid showed growth and PCR positivity of *Rhizopus microsporus*.

**Conclusion:** This is the first report of the clinical observation of transdiaphragmatic mucormycosis in 3 patients. We observed this phenomenon in 11% (3/27) of patients, raising concerns with regard to the extent of imaging studies in patients with mucormycosis. Clinicians should become aware of this new-described entity.

### P091. Baseline predictors influencing the prognosis of invasive aspergillosis in adults

KöhlerP.^1^Salmanton-GarciaJ.^2^GräfeS.^3^KöhlerF.^3^MellinghoffS.^4^SeidelD.^1^SteinbachA.^5^CornelyO.A.^6^1Department I of Internal Medicine, Ecmm Excellence Centre of Medical Mycology, Cologne Excellence Cluster On Cellular Stress Responses In Aging-associated Diseases (cecad), University Hospital Cologne, Cologne, Germany2Department I For Internal Medicine, European Excellence Center For Medical Mycology (ecmm), Germany and Cecad Cluster of Excellence, University of Cologne, Cologne, Germany3Cologne Excellence Cluster on Cellular Stress Responses in Aging-Associated Diseases (CECAD), Cologne, Germany4Department I of Internal Medicine, Ecmm Excellence Centre of Medical Mycology, Cologne Excellence Cluster On Cellular Stress Responses In Aging-associated Diseases (cecad), German Centre For Infection Research, Partner Site Bonn-cologne, University Hospital Cologne, Cologne, Germany5Cologne Excellence Cluster on Cellular Stress Responses in Aging-Associated Diseases (CECAD), German Centre for Infection Research, Partner Site Bonn-Cologne, Cologne, Germany6Department I of Internal Medicine, Ecmm Excellence Centre of Medical Mycology, Cologne Excellence Cluster On Cellular Stress Responses In Aging-associated Diseases (cecad), German Centre For Infection Research, Partner Site Bonn-cologne, Clinical Trials C, University Hospital Cologne, Cologne, Germany

**Introduction:** Invasive aspergillosis (IA) is a serious hazard to hematological and critical care patients. 

**Objectives:** To understand prognostic variables we analyzed original articles identifying baseline factors that predict treatment outcome in patients with IA.

**Methods:** PubMed database was searched for publications since database inception until May 2018. Inclusion criteria were published baseline prognostic factors present at the diagnosis of IA.

**Results:** In total, 58 studies from 267 centers reported 7,320 patients with IA, and 40 different predictors. Unfavorable predictors in medical history were kidney (7.4%, 10/136) and liver failure (3.7%, 5/136), ICU admission (3.7%, 5/136), and uncontrolled underlying disease (3.7%, 5/136). Regarding state of immunosuppression, negative outcome predictors were prolonged neutropenia (12.5%, 17/136), corticosteroid treatment (8.1%, 11/136), and graft-versus-host disease (3.7%, 5/136). On the pathogen side, relevant predictors were galactomannan positivity (8.1%, 11/136), *Aspergillus terreus* infection (2.2%, 3/136), and lack of amphotericin B susceptibility (1.5%, 2/136). IA-specific predictors were disseminated disease (5.1%, 7/136) and CNS involvement (2.9%, 4/136). Imaging results associated with negative outcome were multiple consolidations (2.9%, 4/136), bipulmonary lesions (2.2%, 3/136), and pleural effusion (2.2%, 3/136).

**Conclusion:** At diagnosis of IA, most frequently identified predictors of outcome were neutropenia, corticosteroid use, elevated galactomannan, renal failure, and disseminated disease.

### P092. EQUAL Aspergillosis Score 2018: An ECMM score derived from current guidelines to measure QUALity of the clinical management of invasive pulmonary aspergillosis

CornelyO.A.^1^KöhlerP.^2^ArenzD.^3^MellinghoffS.^2^1Department I For Internal Medicine, Excellence Center For Medical Mycology (ecmm), Cecad Cluster of Excellence, Clinical Trials Centre Cologne (zks Köln), University of Cologne, Cologne, Germany2Department I For Internal Medicine, Excellence Center For Medical Mycology (ecmm), Cecad Cluster of Excellence, University of Cologne, Cologne, Germany3Department I For Internal Medicine, Excellence Center For Medical Mycology (ecmm), University of Cologne, Cologne, Germany

**Introduction:** Invasive pulmonary aspergillosis is a serious threat to immunocompromised and critical care patients. Recent detailed guidelines and treatment algorithms lead microbiologists and clinicians in diagnosis and treatment of invasive aspergillosis. 

**Objectives:** Currently, there is no tool available that allows to measure guideline adherence and quality of managing invasive pulmonary aspergillosis.

**Methods:** To develop such a tool, we reviewed current guidelines provided by 5 scientific societies: European Society for Clinical Microbiology and Infectious Diseases, European Confederation of Medical Mycology, the European Respiratory Society, Infectious Diseases Society of America (IDSA), and Infectious Diseases Working Party of the German Society for Hematology and Medical Oncology. We selected the strongest recommendations for management as key components for our scoring tool, broke down guidelines, and grouped recommendations into 3 groups: diagnosis, treatment, follow-up. First, we selected “AI” and “AII” recommendations. Then, we added aspects that are part of a complete work-up, but are either recommended with a lower grade of evidence or not specifically recommended in current guidelines. In a last step, we allocated score points according to levels of evidence and clinical importance.

**Results:** We developed the EQUAL Aspergillosis Score 2018 to measure the quality of clinical management of invasive pulmonary aspergillosis. We integrated diagnostic measures (chest computed tomography, bronchoalveolar lavage with galactomannan, fungal culture, fungal polymerase chain reaction analysis, species identification, and susceptibility testing; histology with silver stain, Periodic acid–Schiff staining, and molecular diagnostics), treatment (antifungal choice and therapeutic drug monitoring), and follow-up computed tomography. For ease of use, we have summarized the EQUAL scores on laminate pocket cards, which will be translated in 15 different languages. The EQUAL Aspergillosis Score 2018 can be used to evaluate guideline adherence in daily clinical practice and thus aims to support antifungal stewardship.

**Conclusion:** The EQUAL Aspergillosis Score 2018 aggregates and weighs the components recommended in the ideal management of invasive pulmonary aspergillosis and provides a tool supporting antifungal stewardship and quantifying guideline adherence.

### P093. EQUAL Mucormycosis Score 2018: An ECMM Score Derived From Current Guidelines to Measure QUALity of Mucormycosis Management in Hematology

KöhlerP.^1^MellinghoffS.^1^AlanioA.^2^ArenzD.^3^HoeniglM.^4^LagrouK.^5^Lass-FlörlC.^6^MeisJ.^7^RichardsonM.D.^8^CornelyO.A.^9^1Department I For Internal Medicine, Excellence Center For Medical Mycology (ecmm), Cecad Cluster of Excellence, University of Cologne, Cologne, Germany2Paris-diderot, Sorbonne Paris Cité University, Institut Pasteur, Molecular Mycology Unit, Cnrs Cmr2000, Parasitology-Mycology Laboratory, Lariboisière Saint-Louis Fernand Widal Hospitals, Assistance Publique-Hôpitaux de Paris, Paris, France3Department I For Internal Medicine, Excellence Center For Medical Mycology (ecmm), University of Cologne, Cologne, Germany4Division of Infectious Diseases, Department of Medicine, UCSD, San Diego, United States of America5Laboratory of Clinical Bacteriology and Mycology, Department of Microbiology and Immunology, Excellence Center For Medical Mycology (ecmm), KU Leuven, Leuven, Belgium6Division of Hygiene and Medical Microbiology, Excellence Center For Medical Mycology (ecmm), Med. Univ. Innsbruck, Innsbruck, Austria7Department of Medical Microbiology and Infectious Diseases, Excellence Center For Medical Mycology (ecmm), Canisius Wilhelmina Hospital, Nijmegen, Netherlands8Mycology Reference Centre, Excellence Center For Medical Mycology (ecmm), Wythenshawe Hospital, Manchester University NHS Foundation Trust, Manchester, United Kingdom9Department I For Internal Medicine, Excellence Center For Medical Mycology (ecmm), Cecad Cluster of Excellence, Clinical Trials Centre Cologne (zks Köln), University of Cologne, Cologne, Germany

**Introduction:** Mucormycosis is a serious infectious threat to immunocompromised patients in general and in hematological malignancy in particular. Detailed standards and treatment algorithms guide microbiologists and clinicians in the diagnosis and treatment of mucormycosis. 

**Objectives:** Currently, there is no tool available that allows the measurement of guideline adherence and the quality of managing mucormycosis.

**Methods:** To develop such a tool, we reviewed current guidelines provided by four scientific societies: the European Society for Clinical Microbiology and Infectious Diseases (ESCMID), the European Confederation of Medical Mycology (ECMM), the European Conference on Infections in Leukemia (ECIL), and the Infectious Diseases Working Party of the German Society for Hematology and Medical Oncology. We selected the strongest recommendations for management as key components for our scoring tool, broke down guidelines, and grouped recommendations into three groups: diagnosis, treatment, follow-up. First, we selected “AI” and “AII” recommendations. Then we added aspects that are part of a complete work-up, but are either recommended with a lower grade of evidence or not specifically recommended in current guidelines. In a last step, we allocated score points to the various levels of evidence and clinical relevance of diagnostic and therapeutic procedures.

**Results:** We developed the EQUAL Mucormycosis Score 2018 to measure the quality of mucormycosis management. We integrated diagnostic measures (chest computed tomography, bronchoalveolar lavage with fungal culture, fungal polymerase chain reaction analysis, species identification, susceptibility testing, histology with silver stain, Periodic acid–Schiff staining, and molecular diagnostics), treatment (antifungal choice, surgical debridement and therapeutic drug monitoring), and follow-up computed tomography. For ease of use, we have summarized the EQUAL scores on laminate pocket cards, which will be translated in 15 different languages. The EQUAL Mucormycosis Score 2018 can be used to evaluate guideline adherence in daily clinical practice and thus aims to support antifungal stewardship.

**Conclusion:** The EQUAL Mucormycosis Score 2018 aggregates and weighs recommendations for management of mucormycosis and provides a tool supporting antifungal stewardship and quantifying guideline adherence.

### P094. Compliance with recurrent vulvovaginal candidiasis guidelines – An audit of RVVC referrals

BrownL.^1^Rautemaa-RichardsonR.^1^1Faculty of Biology, Medicine and Health, University of Manchester, pl, United Kingdom2Division of Infection, Immunity and Respiratory Medicine, University of Manchester, Manchester, United Kingdom

**Objectives:** Recurrent vulvovaginal candidiasis (RVVC) is a debilitating, chronic condition which affects over 138 million (6%) women of reproductive age annually. It is defined as four or more episodes in a 12-month period. Symptoms include vulval itch, soreness and discharge. Diagnosis is often difficult, as it is based on a combination of these non-specific clinical symptoms and detection of *Candida* by high vaginal swab (HVS) microscopy, which is part of the normal vaginal flora in many women. A number of other infectious and non-infectious conditions can present similarly. Treatment of RVVC comprises of vulval skin care with the use of emollients for personal hygiene and antifungal treatment combined with long-term antifungal suppression for 6 months or longer. The British Association for Sexual Health and HIV (BASHH) has published guidelines on the management of RVVC. The aim of our study is to evaluate the compliance of referring doctors to the current guidelines.

**Methods:** This retrospective audit was undertaken in the Infectious Diseases (ID) department of Wythenshawe Hospital, Manchester from October 2017 to May 2019. All patients referred to the tertiary clinic with suspected RVVC were included in the analyses. Electronic patient records were analysed to determine whether appropriate investigations and treatments had been offered prior to referral. The type and pattern of recorded symptoms were assessed against the typical clinical picture of RVVC and whether alternative differentials should have taken precedence.

**Results:** Patient records from a total of 47 RVVC referrals were analysed. Patient age at the time of referral ranged from 17-66 years with a mean age of 37.7 years. Symptom duration prior to referral ranged from 4 months to 24 years, with an average duration of 6.7 years. “Dry”, “sensitive” skin or “eczema” were mentioned in 36% of the cases. RVVC was the final diagnosis in 80% of cases. 19% of referrals were deemed inappropriate due to repeatedly negative HVS for *Candida* and lack of response to antifungal therapy, or reported symptoms not fitting with RVVC. 57% of patients had a secondary diagnosis, the most common being vulvodynia and vulval dermatitis. 94% of the patients were appropriately investigated by high vaginal swab (HVS) microscopy or culture prior to referral. Appropriate antifungal regimens, including long term suppression, were trialled in 53% of patients prior to referral. Only 11% patients were practicing appropriate vulval skin care before attending clinic. Excessive or otherwise inappropriate personal hygiene likely to irritate vulval skin, including the use of soap and shower gel, was mentioned in 21% of cases. MBL deficiency was diagnosed in 28% of the patients.

**Conclusion:** RVVC is often complicated by coexisting vulval pathologies and vulval symptoms labelled as RVVC are often due to alternative diagnoses. Education on vulval skin care is the mainstay of RVVC management and prevention but healthcare professionals are failing to deliver this. Also, there is lack of awareness of the efficacy and safety of long term antifungal suppression. The morbidity related to this debilitating condition could be easily reduced by following the principles summarised in the BASHH guidelines.

### P095. Validation of a clinical decision rule for selecting empiric treatment for invasive aspergillosis in a setting with high triazole resistance.

PeppelR. Van DeGrootveldR. VanHendriksB.PaassenJ. VanKoopmansJ.BorneP. Von DemBeekM. Van DerBoerM. DeInfectious Diseases, Leiden University Medical Center, Leiden, Netherlands

**Objectives:** World-wide, increasing triazole resistance complicates treatment of invasive aspergillosis (IA). In case of detected triazole resistance, the first choice treatment is replaced by lipid formulations of Amphotericin B (LAmB). In settings with substantial (>10%) prevalence of triazole resistance, empiric combination therapy with both a triazole and LAmB has been suggested. However, the expected benefits of this strategy need to be weighed against a higher rate of serious adverse events associated with combination therapy, as well as higher costs. To evade unnecessary toxicity while optimizing outcome, a clinical decision rule guiding to monotherapy with either voriconazole or LAmB was designed and validated.

**Methods:** In 2015, all medical specialties involved in the diagnosis and treatment of patients with IA participated in constructing a treatment algorithm that provided rationale for empiric monotherapy with either voriconazole or LAmB (Figure 1). The designed algorithm was approved by the institution’s antimicrobial steering committee. Protocolized CT-scanning and bronchoalveolar lavage (BAL) were performed upon suspicion of IA. BAL samples were examined by direct microscopy, culture, galactomannan assay (cut off at 0.5 OD) and from 2017 onwards also by PCR. Triazole resistance was routinely tested by four well agar plate. All adult patients diagnosed with IA and treated for this condition in the Leiden University Medical Center from 2015 to 2019 were included. Data about underlying diseases, age, gender, results of diagnostic tests and outcomes, were extracted from the hospital’s electronic patient files and anonymized. Primary outcomes were 30- and 100-day crude- and attributable mortality rates.

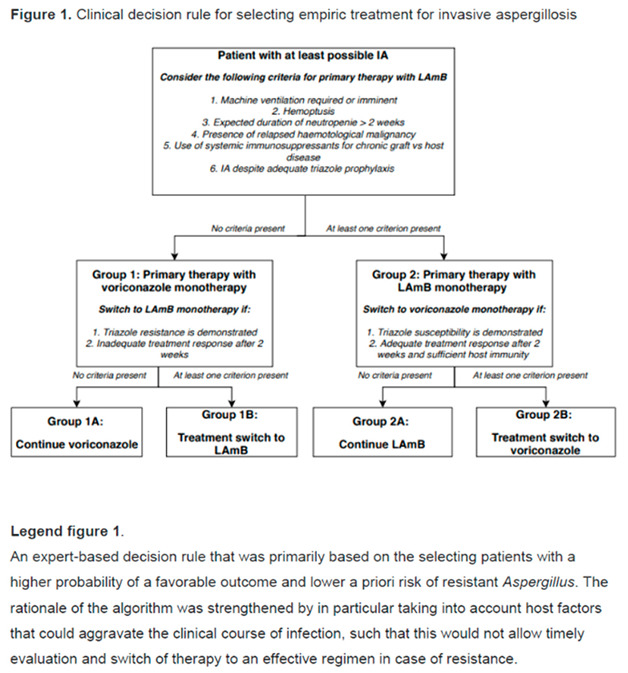



**Results:** At first evaluation, 102 patients had been treated for IA with monotherapy according to the designed algorithm and 3 patients received off-protocol treatment regimens (Figure 2). In the period of study, prevalence of voriconazole resistance in all *Aspergillus* isolates varied per year between 16.3% and 24%. of included patients, 65 (64%) were male; the median age was 64 (range 18-82), and 80 (78%) patients were treated for an underlying hematological malignancy. Fifty-nine patients (58%) were started on voriconazole and 43 (42%) were started on LAmB. Cultures were positive in 16 (15.7%) patients and phenotypical voriconazole resistance was detected in 7/16 (44%). Upon clinical evaluation of empiric therapy and/or resistance data if available, a switch was made to LAmB in 23/59 (39%) of patients started on voriconazole. Crude mortality rates per treatment stratum are listed in Figure 2. Later adaptation of antifungal therapy to LAmB was not associated with a higher crude mortality rate (p = 0.73). Definite therapy consisted of voriconazole in 41 (40%) and of LAmB in 61 (60%) of cases. 

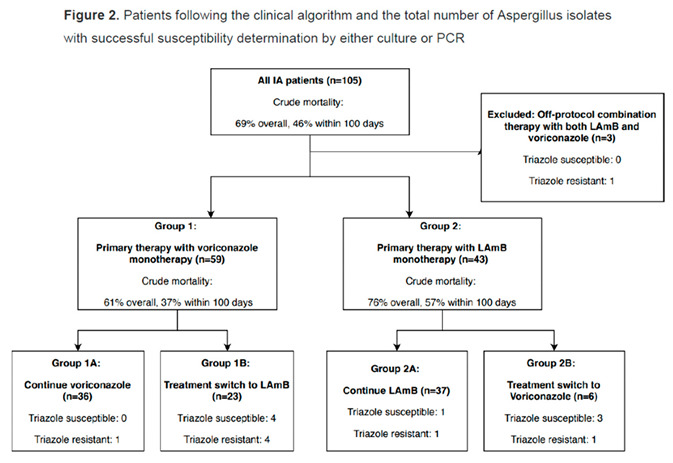



**Conclusion:** By applying a comprehensive clinical decision algorithm in our area with high triazole-resistance rates, 58% of patients were empirically treated with monotherapy voriconazole, without excess crude mortality even if a later switch to LAmB was needed. In 40% of patients therapy with LAmB could be safely avoided during the entire course of treatment. As treatment allocation was not randomized, and confounding by indication plays a role, it remains unclear whether patients directed to initial LAmB monotherapy would have benefited from the addition of a triazole.

### P096. Multicenter study on antifungal prophylaxis in high risk hematology patients - The SAPHIR Study

GangneuxJ.P.^1^PadoinC.^2^MichalletM.^3^SaillioE.^4^KumichelA.^5^TourR. Peffault De La^6^CeballosP.^7^GastinneT.^8^PigneuxA.^9^1Laboratoire De Parasitologie-mycologie, CHU de Rennes, Rennes, France2Pharmacy Department, CHU de Martinique, Fort-de-France, France3Clinical Haematology Department, CH Lyon-Sud, Pierre-Bénite, lyon, France4MSD France, courbevoie, France5ClinSearch, Malakoff, France6Saint-Louis Hospital, APHP, Paris, France7CHRU Lapeyronie de Montpellier, Montpellier, France8CHU de Nantes, Nantes, France9Hospital group Haut Leveque de Pessac, Pessac, France

**Objectives:** The main objective of the SAPHIR study was to describe the diagnostic and therapeutic management of hematology patients at high risk of Invasive Fungal Disease (IFD) under antifungal prophylaxis (AFP) in France. The secondary objectives were the description of (1) parameters and diagnostic procedures concomitant to AF therapy management and (2) preventive procedures adopted in French hematology services.

**Methods:** SAPHIR was an observational, prospective, multicenter, non-comparative French study. The included patients were ≥18 years, in a context of myelosuppressive chemotherapy for acute myeloid leukemia (AML) and initiated an AF prophylactic treatment.

**Results:** The 23 participating hematology services included 404 patients, whose mean age was 56.4±14.0 years and of whom 51.2% were male. Duration between AML diagnosis and inclusion was 65.4±178 days with 65.6% being newly diagnosed. A previous chemotherapy had been undergone by 26.7%. The index chemotherapy, was started 1.06±4.49 days before inclusion and its nature was either induction 79.0%, consolidation 12.6% or relapse 8.4%. Among the 404 patients, 91.6% had experienced a profound neutropenia period with 1.08±0.51 periods per patient and a duration of 23.3±16.6 days. For 373 patients (92.4%), the initially prescribed AFP was posaconazole tablets (90.6%) or suspension (1.73%). Considering all patients, 8.17% changed AFP between 1 and 3 times, with the first change 17.3±23.9 days after inclusion mostly due to absorption issues (65.0%). The mean AFP period was 24.2±32.1 days after which 267 patients (66.8%) stopped because they were at the end of the high-risk period and 126 (31.2%) switched to a non-prophylactic AF treatment (2/3 empirical, 1/3 preemptive/curative), mainly because of fever (67.1%). A total of 18/404 patients (4.46%) have received a curative treatment based on positive mycological exams, imaging, antifungal sensitivity tests and/or blood cultures. Among them, 9/404 (2.23%) were true probable IFDs: 5 probable invasive aspergillosis, 2 pulmonary pneumocystosis, 1 mucormycose and 1 fungemia due to *Saccharomyces* sp. At 7-15 days after AFP discontinuation, 365/387 patients (94.3%) showed no signs and symptoms of infection. In the course of the study, 20 patients (5.0%) had deceased within 57.6±50.3 days after inclusion. of those, 7 patients (35.0%) showed signs and symptoms of infection and the death of 3 patients was directly linked to IFD infections (1 candidiasis and 2 aspergillosis). In order to prevent IFD, additionally to AFP, French haematology services provide the patient with sterile/protected food (94.3%), digestive decontamination (35.8%), and accommodation in a sterile/isolated area (93.5%). To gain a better understanding of the patient characteristics possibly indicating the necessity for a consecutive empirical/preemptive/curative AF treatment, data of patients with and without such a consecutive treatment were compared. Several parameters proved to be significantly different, for instance: cytogenetics and molecular biology prognosis, AML onset, previous chemotherapy treatment but also duration between chemotherapy initiation and inclusion and the type of current chemotherapy cycle used.

**Conclusion:** AF prophylactic treatment is frequently prescribed in French hematology services. Only few prophylactic treatment changes are necessary which reflects treatment tolerance and effectiveness regarding decreased numbers of intercurrent infections and mortality.

### P097. EQUAL Cryptococcus Score 2018: A European Confederation of Medical Mycology Score Derived From Current Guidelines to Measure QUALity of Clinical Cryptococcosis Management

SpecA.^1^Mejia-ChewC.^1^PowderlyW.^1^KöhlerP.^2^,^3^CornelyO.A.^2^,^4^1Internal Medicine, Washington University School of Medicine in St. Louis, St. Louis, MO, United States of America2Department I For Internal Medicine, European Excellence Center For Medical Mycology (ecmm), Germany and Cecad Cluster of Excellence, University of Cologne, Cologne, Germany3Department I of Internal Medicine, Ecmm Excellence Centre of Medical Mycology, Cologne Excellence Cluster On Cellular Stress Responses In Aging-associated Diseases (cecad), University Hospital Cologne, Cologne, Germany4German Centre For Infection Research, Partner Site Bonn-cologne, Cologne, Germany and Clinical Trials Centre Cologne (zks Köln), University of Cologne, Cologne, Germany

**Objectives:** Cryptococcocis is an opportunistic fungal infection with high morbidity and mortality. Guidelines to aid clinicians regarding diagnosis, management, and treatment can be extensive and challenging for clinicians. Currently, there is no tool available that allows the measurement of guideline adherence and the quality of cryptococcosis management.

**Methods:** We reviewed current guidelines from the Infectious Diseases Society of America, the World Health Organization, the American Society of Transplantation and recent significant publications to select the strongest recommendations as vital components of our scoring tool.

**Results:** Items included diagnostic tests (blood, tissue, and cerebrospinal fluid cultures, Cryptococcus antigen, India ink, histopathology with special fungal stains, central nervous system imaging), pharmacological (amphotericin B, flucytosine, azoles) and nonpharmacological treatments (intracranial pressure management, immunomodulation, infectious disease consultation), and follow-up of central nervous system complications. For ease of use, we have summarized the EQUAL scores on laminate pocket cards, which will be translated into 15 different languages.

**Conclusion:** The EQUAL Cryptococcus Score 2018 weighs and aggregates the recommendations for the optimal management of cryptococcosis, providing a tool that could measure guideline adherence or facilitate clinical decision-making.

### P099. An ongoing malady, mucormycosis from a tertiary care centre at Chandigarh, India.

ChanderJ.^1^BhagatA.^1^GulatiN.^1^SinglaN.^1^PuniaR.P.S.^2^AgarwalD.^3^AttriA.K.^4^DassA.^5^1Microbiology, Government Medical College Hospital, Chandigarh, India2Pathology, Government Medical College Hospital, Chandigarh, India3Pulmonary Medicine, Government Medical College Hospitaln, Chandigarh, India4General Surgery, Government Medical College Hospital, Chandigarh, India5Ent, Government Medical College Hospital, Chandigarh, India

**Objectives:** Mucormycosis is an emerging necrotizing angioinvasive infection caused by commonly encountered filamentous fungi of the class Mucormycetes. In our institution, as the awareness is gradually increasing, newer and newer species causing mucormycosis are being recognized. In this very reference a study was undertaken to further enhance our knowledge on risk factors, species distribution and their antifungal susceptibility testing.

**Methods:** The study was conducted on forty-four cases of mucormycosis over a period of eighteen months from January 1, 2017 to June 30, 2018. The samples were subjected to microbiological examination like KOH/CFW wet mount and fungal culture and histopathology. The isolates obtained were identified phenotypically and confirmed by DNA sequencing of internal transcribed spacer region. The antifungal susceptibility testingwas done for amphotericin B, posaconazole, itraconazole and terbinafine by microbroth dilution as per CLSI Reference Method.Study performed by Almyroudis, et al, was referred to define susceptibility for some of the breakpoints for Mucorales.

**Results:** The mean age of patients of mucormycosis was 48.89 ± 17.1 years. The proportion of female patients (54.5%) was slightly more than male patients (45.5%). Rhino-orbito-cerebral mucormycosis (ROCM) was the commonest presentation (61.4%, 27) followed by pulmonary (20.4%, 9) and cutaneous (15.9%, 7). One case of mucormycosis of the middle ear was also seen. The most common risk factor associated with mucormycosis was diabetes mellitus (77.3%, 34/44), which was found to be statistically significant (*P* = 0.003). Tooth extraction was seen as a risk factor in 18.5% (5/27) of ROCM cases. The commonest isolate was *Rhizopus arrhizus* (32.3%, 10), followed by *Rhizopus microsporus* (29%, 9) and *Apophysomyces variabilis* (12.9%, 4). *Rhizopus homothallicus* was isolated from two cases, one of middle ear and other from pulmonary mucormycosis. A case of cutaneous mucormycosis caused by *Thamnostylum piriforme*, which is the second case of this genus and first of this species, is being reported. Amphotericin B was the most effective drug with an MIC range of 0.0313-4 µg/ml giving 94.7 % isolates as sensitive. The only isolate resistant was *Rhizopus homothallicus* with an MIC of 4 µg/ml. Posaconazole gave an MIC range of 0.0625-4 µg/ml with 68.4% isolates as sensitive. Dual management with surgery and antifungals proved to be significantly better (*P* < 0.001) than amphotericin B alone. Treatment with liposomal amphotericin B (LAmB) was associated with a higher survival rate (81%), compared to 28.6% survival when patients were treated with conventional amphotericin B deoxycholate. Twelve (27.3%) patients either left against medical advice or were referred. A total of 12 patients expired giving a mortality rate of 27.3% and 37.5% among the treated patients.

**Conclusion:** India has second largest diabetic population globally with nearly 70% being found with uncontrolled diabetes leading to high incidence of mucormycosis. Due to an ever-growing population at risk and the fatal outcome, need for more prospective studies in this field are emphasized. Increased awareness among clinicians, timely diagnosis and prompt treatment are the key to the successful management.

### P100. The Protective Role of Cinnamaldehyde against Tenuazonic acid-induced Mycotoxicity in Murine model

KumariA.^1^SinghK.^2^1Animal Mycology Laboratory, Zoology, Mmv, Banaras Hindu University, Varanasi, India2Animal Mycology Laboratory, Zoology, Mmv, Banaras Hindu University, VARANASI, India

**Objectives:** Cinnamaldehyde is a natural product obtained from cinnamon and has been reported to have insecticidal, antimicrobial, anti-inflammatory, immunomodulatory, anticancer, anti-angiogenic and potential anti-fungal effects. The present study investigated the possible protective effect of cinnamaldehyde against tenuazonic acid-induced mycotoxicity in murine model. Tenuazonic acid (TeA), a toxin produced by *Alternaria alternata* is a common contaminant in tomato and tomato based products.

**Methods:** The *in-vivo* experimental plan was as follows.

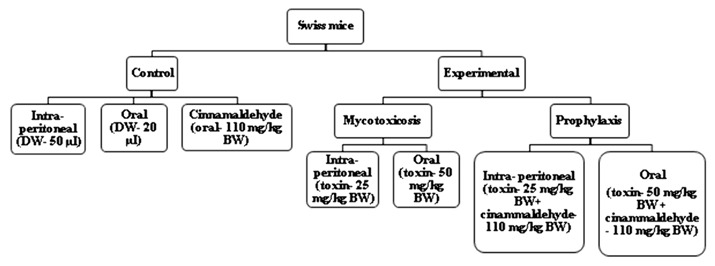



All the experimental groups were administered with TeA for 8 weeks. After 2 weeks, the prophylactic group was administered with cinnamaldehyde along with TeA for the next 6 weeks. Haematological (differential leukocyte count), histopathological (haematoxylin and eosin), biochemical (superoxide dismutase, catalase, malondialdehyde, alanine aminotransferase, aspartate aminotransferase) and cell apoptotic factor (caspase-3) analyses of the experimental and control mice were performed.

**Results:** Behavioral observations- Unlike mycotoxicosis induced groups, no behavioural abnormalities were observed in the animals who received cinnamaldehyde as prophylactic agent. Weights of the mice of the prophylactic group showed reduced body weight gain as compared to that of the mycotoxicosis induced groups. Morphological observations- Hair loss indicating mycotoxicosis was not observed in the prophylactic groups. Anatomical observations- Spleenomegaly, hepatomegaly and gross lesions on liver and kidney were noted on autopsy in the mycotoxicosis induced groups. On the other hand, the prophylactic groups showed normal weights of liver and spleen. The body:organ weight indices of all the organs remained at par to control group in the prophylactic groups except for the lungs and brain where the weights of the organs were significantly high. Haematological analysis- Neutrophilia was observed in IP (mycotoxicosis induced) while neutropenia was observed in the oral (mycotoxicosis induced) group while the differential leukocyte count of the prophylactic groups were found to lie in normal range. Biochemical analyses- Acute intoxication of mice with TeA showed elevated malondialdehyde (MDA), reduced catalase (CAT) and superoxide dismutase (SOD) production; abnormal levels of aspartate transaminase (AST) and alanine transaminase (ALT). Treatment with cinnamaldehyde reversed TeA-induced alterations of antioxidant defense enzyme activities. Histological observations- Histopathological changes characterised by non alcoholic fatty liver, nuclear pyknosis and infiltration of immune cells were observed in the mycotoxicosis induced groups while cinnamaldehyde significantly prevented the TeA-induced organ damage. Cell apoptosis factor- Reduced activity of caspase-3 enzyme was noted in the mycotoxicosis induced groups while significantly high activity of the enzyme was observed in the prophylactic groups.

**Conclusion:** Thus, from the present study we concluded that, cinnamaldehyde showed therapeutic effects and toxicity reduction in TeA induced mycotoxicosis. 

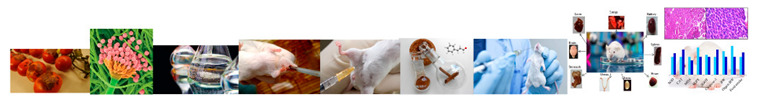



### P101. Candi-SoL: A multi-site retrospective audit and analysis of Candidaemia across three South London Teaching Hospitals

LoganC.^1^FifeA.^2^GoodmanA.^3^WardC.^2^BicanicT.^1^1Infection & Immunity, St Georges university, London, London, United Kingdom2Department of Microbiology, Kings College Hospital, London, United Kingdom3Department of Infection, Guy’s & St Thomas’ Hospital, London, United Kingdom

**Objectives:** Candidaemia is associated with high mortality, particularly in critical care and immunocompromised patients. Emergence of multi-resistant candida species, notably C. *glabrata* and C. *auris*, pose further challenges in treatment. This retrospective surveillance and audit study reviews both adult and paediatric candidaemia cases between January 2012- December 2018 across three Tertiary South London Trusts; St George’s Hospital in London, Kings College hospital London and Guys & St Thomas’ Hospital. Collectively these trusts have over 3500 beds, including over 220 ICU beds (adult surgical, neurological, cardiac and general ICUs and paediatric and neonatal ICU) and onsite specialist surgical, cardiothoracic and medical services, including liver, bone marrow and renal transplant. The aims of this study were to assess trends in candidaemia epidemiology across the South London region, categorise at risk groups, antifungal therapy used and review clinical outcomes.

**Methods:** Blood cultures for adults and children isolating *Candida* species between January 2012-December 2018 were retrospectively identified from the Microbiology database. Species and antifungal susceptibility were reviewed. Persistent candidaemia (identical Candida *sp*. isolated ≤ 30days after the initial blood culture) was analysed as a single episode. Demographics (age, sex); location; risk factors; focus of candidaemia; antifungal treatment received and duration; ECHO performed and outcome; ophthalmology performed and outcome; line removal and culture result; length of stay; 30-day and in-hospital mortality was collected from electronic patient records. Analyses were conducted in Microsoft Excel 2013 and GraphPad Prism V7.

**Results:** Between January 2012 – December 2018, 509 candidaemia episodes were identified across the three Trusts. In total 42% were *C. albicans*, 30% *C. glabrata*, 12% *C. parapsilosis* and 1% of cases were due to *C. auris* following an outbreak in 2016. There was no significant trend towards an increasing proportion of non-albicans species over the 7-year period (Chi-squared test for trend, p = 0.22). Regarding age at the time of candidaemia, 4% were neonates (< 1 month), 9% were children (aged 1 month - <18 years), and 86% were adults (> 18 years). Patients on an intensive care unit (adult, paediatric or neonatal) accounted for 43% of cases. Clinical data regarding the candidaemia foci, the ECHO and ophthalmology findings, antifungal therapy and duration, B-D-glucan result if available, length of stay and mortality will also be presented. Susceptibility profile of isolates will be interrogated and presented to assess if there has been a change over time across the three trusts.

**Conclusion:** This retrospective audit of candidaemia across three large South London Trusts illustrates the changing regional candida epidemiology, with non-albicans species accounting for over half of all isolates and highlights the emergence of multi-drug resistant species such as *C. auris* in the London. The remaining analyses of this large dataset will highlight which are the dominant causes of candidaemia across the South London Trusts and examine clinical outcomes with candidaemia over a diverse group of patients.

### P102. Antifungal Efficacy of Isavuconazole and Liposomal Amphotericin B for Treatment of Experimental Exserohilum rostratum Meningitis and Therapeutic Monitoring with CSF (1→3)-β-D-glucan: A Preclinical Paradigm for Treatment of CNS Phaeohyphomycosis

PetraitisV.PetraitieneR.NaingE.MaungB.B.W.GarciaA.KavaliauskasP.WalshT.J.Infectious Diseases/medicine, Weill Cornell Medicine of Cornell University, New York, United States of America

**Objectives:** Phaeohyphomycosis of the central nervous system (CNS) is a life-threatening infection with serious morbidity and mortality. Treatment options for CNS phaeohyphomycosis are limited. This severity of CNS phaeohyphomycosis was tragically illustrated in the contamination of a corticosteroid injection preparation that resulted in epidemic *Exserohilum rostratum* meningitis (ERM), resulting in meningoradiculitis, brain abscess, neurologic deficits, and death despite antifungal therapy with voriconazole with or without liposomal amphotericin B (LAMB). We hypothesized that isavuconazole may be an alternative approach for patients with ERM and other causes of CNS phaeohyphomycosis, particularly those with severe infection or intolerance to initially high dosages of voriconazole therapy. We therefore investigated the *in vitro* antifungal activity of isavuconazole with or without LAMB followed by treatment in a new model of experimental ERM.

**Methods:** Clinical isolates of *E. rostratum* recovered from patients suffering from ERM were used in all experiments. Broth microdilution methodology was used to prepare an inoculum for each of five clinical isolates of *E. rostratum*. Combination checkerboard plates were used to test the activity of isavuconazole and LAMB, either alone or in combination. Minimum inhibitory concentrations (MICs), minimal lethal concentrations (MLCs) and fractional inhibitory concentration (FIC) indices were determined. For *in vivo* therapeutic studies, *E. rostratum* were inoculated intracisternally with 1.0x106 microconidia to anesthetized rabbits. Immunosuppression was produced with cytarabine-induced profound persistent neutropenia and methylprednisolone. Study groups consisted of isavuconazole, 60 mg/kg/d, LAMB at 5.0, 7.5, and 10 mg/kg/day and untreated controls (UC).

**Results:** As there were no *in vitro* additive, synergistic, or antagonistic interactions for combinations of isavuconazole plus LAMB against the *E. rostratum* isolates, *in vivo* studies in the rabbit model of ERM were conducted with isavuconazole and LAMB as monotherapies. Isavuconazole 60 mg/kg/d and LAMB at 5.0, 7.5, and 10 mg/kg/day demonstrated significant reductions of the residual fungal burden of *E. rostratum* in cerebrum, cerebellum, spinal cord, and paravertebral muscle (*p*<0.01) in comparison to those of UC. Reduction of the residual fungal burden correlated with significant reduction of CSF (1→3)-β-D-glucan levels in comparison to those of UC (*P*<0.05).

**Conclusion:** Isavuconazole alone or LAMB alone significantly reduced residual fungal burden in parallel with reduction of CSF (1→3)-β-D-glucan levels in treatment of experimental *E. rostratum* meningitis and may serve as alternative regimens for treatment of CNS phaeohyphomycosis.

### P103. Pulmonary mucormycosis caused by Lichtheimia ornata (experimental model)

VasilyevaN.^1^BosakI.^2^BogomolovaT.^1^VybornovaI.^2^StepanovaA.^2^AvdeenkoY.^2^ChilinaG.^2^AakO.^2^SolovyevaG.^1^1Kaschkin Research Institute of Medical Mycology. Department of Medical Microbiology, North-Western State Medical University named after I.I. Mechnikov, Saint-Petersburg, Russian Federation2Kaschkin Research Institute of Medical Mycology, North-Western State Medical University named after I.I. Mechnikov, Saint-Petersburg, Russian Federation

**Objectives:** *Lichtheimia ornata* (A.K. Sarbhoy) Alastr. – Izq. & Walter due to the prevalence and virulence is one of three most common species causing mucormycosis. This species was revealed among the agents of invasive mucormycosis in Russia. The purpose of the present research was to develop a murine experimental model of pulmonary mucormycosis caused by *L. ornata.*

**Methods:** For the infection of mice we used the strain RCPF-1507 of *L. ornata*, isolated from the tissue of accessory sinuses of nose from a patient with lymphatic leukemia. For modeling the pulmonary mucormycosis we used outbred mice, males, with body weight 18-20 g. For inducing the condition of neutropenia we intraperitoneally injected cyclophosphamide at the dose 150 mg/kg four times (-3, 0, 4 and 8 days). From fungal culture grown on Sabouraud glucose agar for 3 days at 37° C we made the spore suspension in sterile 0.85% NaCl solution at the concentration 1·10^7^ CFU/ml. Infection of animals was carried out by intranasal injection of 50 μl of fungal spores suspension to a narcotized mouse.

**Results:** Data on mice lethality and results of cultural and histological investiga-tions of murine lungs with invasive mucormycosis were obtained. Death of animals was observed from 8 to 18 days after infection. No mice survived. Culturing of lung tissue from all dead mice revealed growth of *L. ornata*. During the histological investigation of murine lungs taken after eight days from the beginning of infection the different area of localization of hyphal cells were found. Hyphae were wide (10-12 µm), with rare branching at right angle. Hyphal cells in the lung parenchyma generally were located densely and in parallel relatively each other that is characteristic of tissue forms of mucormycetes. In lungs vessels massive aggregation of densely and chaotically oriented fungal hyphae were revealed. Also we observed the hyphal aggregation in gleams of bronchial tubes and an interstitium. The inflammatory process due to a mycotic infection was poorly expressed, provided by poor neutrophilic and lymphocytic infiltration, alteration processes prevailed.

**Conclusion:** The scheme of immunosuppression used in this work (intra-peritoneal introduction of cyclophosphamide) showed efficiency for producing infection in mice by *L. ornata*. Data on survival of the infected animals and also results of cultural and histological investigations confirm existence of mycotic damage of lungs at the infected animals. The developed model of invasive pulmonary mucormycosis can be used for future scientific research, testing of new antifungal drugs and diagnostic methods.

### P107. Case of successful treatment of chronic pulmonary aspergillosis in a patient with polycystic disease

ShadrivovaO.^1^KuznetsovV.^2^DesyatikE.^2^BorzovaY.^2^IgnatyevaS.^2^BogomolovaT.^2^VasilyevaN.^2^KlimkoN.^1^1Department of Clinical Mycology, Allergy and Immunology, North-Western State Medical University named after I.I.Mechnikov, St. Petersburg, Russian Federation2Kashkin Research Institute of Medical Mycology, North-Western State Medical University named after I.I.Mechnikov, St. Petersburg, Russian Federation

**Case Report: Background**. Publications onchronic pulmonary aspergillosis (CPA) in patients with polycystic diseaseare limited. 

**Objection:** We present a case of successful treatment of CPA in a patient with polycystic disease. 

**Methods:** Diagnosis of CPA was made in accordance with the criteria ESCMID/ECMM/ERS, 2016. 

**Result:** Patient V., 70 years old, was admitted to the mycological clinic with complaints of productive cough and dyspnea in September 2018. It is known that in 2015 lesions in left lung lower lobe were detected on chest CT scan. The CPA diagnosis was made on the basis of chest CT scan (multiple aspergillomas in left lung S6), elevated serum *Aspergillus* IgG level, and *A. fumigatus* and *A. niger* in BAL culture. The patient was treated with itraconazole 400 mg/day for 2 weeks. In October 2018, the patient underwent surgery removal of the left lung lower lobe, and CPA diagnosis was confirmed with histology. Therapy with itraconazole 400 mg/day was continued for 1 month. At the end of treatment, there was no data for active CPA: no specific lesions on chest CT scan, with negative BAL microscopy and culture, and serum *Aspergillus* IgG level. On the Pubmed website, we did not find a case reports of CPA in patients with polycystic disease. 

**Conclusions:** Polycystic disease can be underlying disease of chronic pulmonary aspergillosis. Combinations of antimycotic therapy and surgical treatment are necessary for the successful treatment of chronic pulmonary aspergillosis in patients with polycystic disease.

### P108. Chronic pulmonary aspergillosis in patients with idiopathic pulmonary fibrosis

ShadrivovaO.^1^TarasovaM.^1^DesyatikE.^2^NikolaevaN.^3^BorzovaY.^2^IgnatyevaS.^2^BogomolovaT.^2^VasilyevaN.^2^KlimkoN.^1^1Department of Clinical Mycology, Allergy and Immunology, North-Western State Medical University named after I.I.Mechnikov, St. Petersburg, Russian Federation2Kashkin Research Institute of Medical Mycology, North-Western State Medical University named after I.I.Mechnikov, St. Petersburg, Russian Federation3North-Western State Medical University named after I.I.Mechnikov, St. Petersburg, Russian Federation

**Case Report: Introduction**: Publications onchronic pulmonary aspergillosis (CPA) in patients with idiopathic pulmonary fibrosis are limited. 

**Objective**: We present a case of successful treatment of CPA in a patient with idiopathic pulmonary fibrosis. 

**Materials and methods**: Diagnosis CPA performed in accordance with the criteria EORTC/MSG, 2008. 

**Results**: Patient V., 78 years old, was admitted to the mycological clinic with complaints of productive cough, exertional dyspnea in February 2018. It is known that the patient was diagnosed with idiopathic pulmonary fibrosis in 2017. He did not receive basic therapy. On chest CT scan for the first time was detected a cavity of 43x68x29 mm with the soft-tissue formation 12x14 mm in the apex region of the right lung. A positive titer the IgG to *Aspergillus fumigatus* 1: 3200 was determined. *Aspergillus fumigatus* grow in the sputum culture was revealed. The patient received itraconazole with 400 mg per day with a positive clinical effect. Decrease of cough and exertional dyspnea was noted. Duration of antifungal treatment was 3 months. In July 2018, the chest CT scan showed a decrease in the size of the cavity and reducing of the soft-tissue formation in the right lung. The IgG titer to *Aspergillus fumigatus* was decreased to 1: 800. No fungi were found in sputum culture. The patient received antimycotic therapy for another 3 months. In October 2018, the CT scan of the chest showed stabilization of changes. IgG titer to *Aspergillus fumigatus* decreased to 1: 400. The culture study of sputum was negative. Antimycotic therapy was stopped. When analyzing the literature on the Pubmed site, we found 5 cases of aspergillosis with idiopathic pulmonary fibrosis. 1 case where the disease is associated with the intake of glucocorticosteroids, 1 case associated with primary immunodeficiency, 1 case with the influenza B virus and 2 cases in non-immunocompromised patients. **Conclusion**: Idiopathic pulmonary fibrosis can be an additional “background” disease of chronic pulmonary aspergillosis. Prolonged antimycotic therapy is necessary for successfully treatment of chronic pulmonary aspergillosis in patients with idiopathic pulmonary fibrosis.

### P109. The performance of the BALF later-flow device test in the rapid diagnosis of nonneutropenic invasive pulmonary aspergillosis

LiuL.WangY.GuY.LiS.ChenB.ShenK.SuX.Jinling Hospital, Medical School of Nanjing University, Nanjing, China, nanjing, China

**Objectives:** The *Aspergillus* lateral-flow device (LFD) is a new method to test for *Aspergillus* antigen. We performed this study to evaluate the value of the LFD test in diagnosing nonneutropenic invasive pulmonary aspergillosis.

**Methods:** A total of 136 bronchoalveolar lavage fluid (BALF) samples from 136 suspected invasive pulmonary aspergillosis (IPA) patients were finally divided into 2 groups: the aspergillosis group and the non-aspergillosis group. We performed both the LFD and glactomannan (GM) tests on 80 BALF samples and only the LFD test on the remaining 58 BALF samples.

**Results:** After systematic clinical diagnosis, all 136 patients were divided into an aspergillosis group (*n* = 41, proven (*n* = 8) and probable (*n* = 33) diagnosis) and a non-aspergillosis group (*n* = 95, pneumonia, tuberculosis and others). The sensitivity, specificity, positive predictive value, and negative predictive value of LFD for diagnosing pulmonary aspergillosis in all 136 BALF samples were 69.7%, 72.6%, 87.3% and 46.9%,respectively. When comparing the GM and LFD test results for the 80 samples, the sensitivity (75.8% vs 74.1%, P = 0.769) was similar. The specificity of the GM test was higher than that of the LFD test (83.0% vs 66.0%, P = 0.058), but there was no significant difference between the tests. The sensitivity and specificity of the combination of the positive results of both theLFD and GM tests were 63.6%and 89.4%, respectively, and the sensitivity and specificity of either LFD or GM positive results or both LFD and GM positive results were 90.9% and 59.6%, respectively.

**Conclusion:** LFD is a promising alternative diagnostic method to the GM test fortheearly detection of patients with nonneutropenic pulmonary aspergillosis.

### P110. High rates of misidentification of uncommon Candida species fungemia with conventional phenotypic methods

LiuW.-L.^1^,^2^HuangY.-S.^3^WangF.-D.^4^HsiehM.-H.^5^HiiI.-M.^6^LeeY.-L.^6^HoM.-W.^7^1School of Medicine, College of Medicine, Fu Jen Catholic University, New Taipei City, Taiwan2Emergency & Critical Care Medicine, Fu Jen Catholic University Hospital, New Taipei City, Taiwan3Internal Medicine, National Taiwan University Hospital, Taipei, Taiwan4Medicine, Taipei Veterans General Hospital, Taipei, Taiwan5Internal Medicine, Kaohsiung Medical University Hospital, Kaohsiung Medical University, Kaohsiung, Taiwan6Internal Medicine, Changhua Christian Hospital, Changhua, Taiwan7Internal Medicine, China Medical University Hospital, Taichung, Taiwan

**Objectives:** Candidemia caused by uncommon *Candida* species is increasing. Correct identification of *Candida* isolates to species level is important for optimizing antifungal choice. In this study, we aimed to evaluate the accuracy of species-level identification of conventional methods and MALDI-TOF MS system in patients with candidemia due to the uncommon *Candida* species. Sequencing of the internal transcribed spacer (ITS) was performed for comparison and determination of accuracy.

**Methods:** From July 2011 to December 2014, uncommon *Candida* species identified by conventional phenotypic methods from candidemic patients were prospectively collected at 6 hospitals in Taiwan. Identification of theses uncommon *Candida* isolates were performed using MALDI-TOF MS and DNA sequencing of the fungal internal transcribed spacer (ITS). Susceptibility of the non-duplicate isolates were determined using Sensititre^TM^ YeastOne^TM^ and were interpreted with cut-off values recommended by the CLSI.

**Results:** A total of 85 uncommon *Candida* isolates reported by conventional methods were included. By conventional methods, the most frequently reported uncommon *Candida* species were *C. guilliermondii* (*n* = 39), *C. sake* (*n* = 7), and *C. famata* (*n* = 4). Using ITS sequencing analysis as standard, none of the *C. sake* and *C. famata* identified by conventional methods was correct, while MALDI-TOF MS correctly identified 10 of the 11 isolates. With the exclusion of the one unspecified *Candida* isolate reported by ITS sequencing, the accuracy of conventional methods and MALDI-TOF MS in the identification of 84 *Candida* isolates were 64.3% and 86.9%, respectively (p = 0.001). Eight isolates were confirmed to be non-*Candida* yeast species. Compared with other *Candida* species, *C. guilliermondii* showed elevated MIC values of echinocandins. Based on our results, an identification algorithm for uncommon *Candida* species was proposed (Figure 1). 

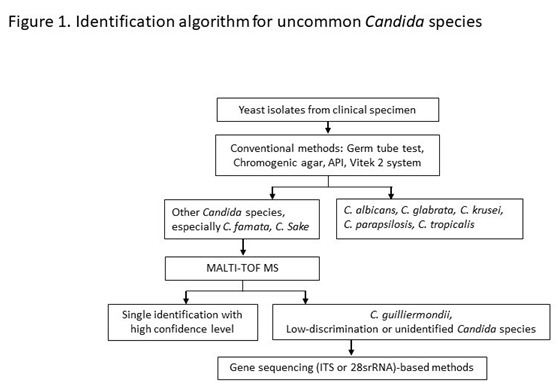



**Conclusion:** Conventional methods could lead to high rate of misidentification of uncommon *Candida* species, especially *C. sake* and *C. famata*. MALDI-TOF MS assisted with DNA-sequencing based methods should be considered for identification of uncommon *Candida* species.

### P112. Cross-sectional study of respiratory Aspergillus spp. colonization or infection in patients with various stages of Chronic Obstructive Pulmonary Disease (COPD) using culture vs non-culture based technique.

WaqasS.^1^,^2^DunneK.^2^TalentoA. Fe^3^,^4^,^5^WilsonG.^1^Martin-LoechesI.^1^KeaneJ.^1^,^2^RogersT. R^1^,^2^1St. James’s Hospital, Dublin, Ireland2Trinity College Dublin, Dublin, Ireland3Our Lady of Lourdes Hospital, Drogheda, Ireland4Royal College of Surgeons Ireland, Dublin, Ireland5Beaumont Hospital, Dublin, Ireland

**Objectives:** (*1st and 2nd Authors are equal contributors) COPD patients are now recognized to be at increased risk of colonization by *Aspergillus* spp. which may progress to invasive pulmonary aspergillosis (IA). Published data on the frequency of *Aspergillus* detection in COPD are limited. The objective of this study was to determine frequency of *Aspergillus* colonization or infection in COPD patients.

**Methods:** A cross-sectional study was undertaken to determine *Aspergillus* colonization or infection in COPD patients undergoing bronchoscopy for any indication.Culture as well as galactomannan antigen (GM) and *Aspergillus* nucleic acid detection (PCR) were performed on bronchoalveolar lavage fluid (BAL).

**Results:** One hundred and fifty patients were included (44.7 % female, mean age 68.2 years). 21.3% were inpatients, 74.7% outpatients and 4% were ICU patients. Investigation of lung masses was the most common indication (43.3%) for bronchoscopy. Most patients (81.3 %) were either GOLD stage 1 or 2 COPD. Cancer was the most frequent co-morbidity (60.48%). 12 % and 48.7 % were on systemic and inhaled steroids respectively. Lung mass was the most common (28.43%) CT imaging finding. Seventeen patients (11.3%) had a positive result for *Aspergillus* (Culture ± Galactomannan ± PCR). 76.4% out of these seventeen were in the early stages (GOLD stage 1 or 2) of COPD.

**Conclusion:** *Aspergillus* sp. was detected in 3.3% of patients by culture, which increased to 11.3% if culture was combined with either a positive GM or PCR result. Overall the frequency of *Aspergillus* detection in this population of COPD patients was low which may reflect the predominance of Gold stages 1 and 2 among the study population.

### P113. Evaluation of new multiplex PCR-based blood assays for detection of candidemia

JobstmannM.^1^,^2^PrattesJ.^2^StelzlE.^1^ZurlC.^1^HoeniglM.^3^RabensteinerJ.^1^KrauseR.^2^KesslerH.^1^1Medical University of Graz, Graz, Austria2Section of Infectious Diseases and Tropical Medicine, Medical University of Graz, Graz, Austria3Division of Infectious Diseases, University of California, San Diego, United States of America

**Objectives:** Blood cultures have been considered as diagnostic gold standard for candidemia. Compared to cultures, assays based on polymerase chain reaction (PCR) may be useful to shorten the time to diagnosis of invasive candidiasis and to initiation of antifungal therapy. Recently, the new CandID® and CandID PLUS® kits (OLM Diagnostics, Newcastle Upon Tyne, England) for molecular detection of different *Candida spp.* have been developed. In this study, the analytical and clinical performance of the new kits was investigated. Reference material and clinical specimens were used.

**Methods:** Nucleic acid extraction was performed on the EMAG® platform (bioMérieux, Marcy-l´Etoile, France) using the specific B protocol. Real-time PCR (qPCR) and detection with the CandID® and CandID PLUS® kits were performed on the Light Cycler® 480 II CE/IVD instrument (Roche Diagnostics, Penzberg, Germany). Both of the kits are based on multiplex qPCR providing detection of three different *Candida spp*. each and an internal control: *Candida albicans*, *Candida glabrata*, *Candida parapsilosis* with the CandID® and *Candida tropicalis*, *Candida krusei*, *Candida dubliniensis* with the CandID PLUS®. The accuracy of the new kits was determined utilizing the Quality Control for Molecular Diagnostics (QCMD) 2018 *Candida spp*. EQA Programme. The panel consisted of 10 members including *Candida albicans*, *Candida auris*, *Candida glabrata*, *Candida krusei*, and vials without *Candida spp*. The clinical performance of the new kits was determined with specimens obtained from three groups of patients: patients with culture-proven candidemia (*n* = 23; EDTA whole blood samples), patients with bacteremia (*n* = 31; plasma samples), and healthy controls (*n* = 25; EDTA whole blood samples). Whole blood samples from candidemic and bacteremic patients were drawn at the same day as the corresponding blood cultures.

**Results:** With the quality control program, all panel members were correctly identified by the new PCR assays. With the clinical study, 2 EDTA whole blood samples obtained from patients with candidemia and 7 EDTA whole blood samples obtained from healthy controls were found to be inhibited and thus excluded from further analysis. In patients with candidemia (mean age, 67 years, range 20 to 89 years), 14 of 21 samples (67%) gave a positive result when employing the new PCR assays. In patients with bacteremia (mean age, 62 years, range 23 to 91 years), 1 of 31 samples gave a false-positive result with the CandID assay. All healthy controls (mean age, 57 years, range 45 to 66 years) remained negative with the new PCR assays. All *Candida spp*. from candidemic patients were correctly identified with the PCR assays.

**Conclusion:** When compared to blood culture, two thirds of samples obtained from patients with candidemia were found positive with the new CandID® or CandID PLUS® kits. While in samples obtained from patients with bacteremia one false-positive result was observed with the new PCR assays, results of samples obtained from healthy controls were all found to be correct.

### P114. Development of a New Quantitative Real-Time PCR Assay for the Detection and Identification of Aspergillosis and Mucormycosis Infections

GodichaudS.^1^VerrierJ.^1^BoissonR.^1^LagardereG.^1^FloriP.^2^1ADEMTECH, PESSAC, France2CHU ST ETIENNE, ST ETIENNE, France

**Objectives:** Mucormycosis is associated with high mortality rates, especially in haematological patients, and remains difficult to diagnose. The early distinction with invasive aspergillosis is of importance because the antifungal treatment for each is different. Unfortunately, the underlying conditions of both infections are similar. Moreover, detection of circulating antigens such as galactomannan and β-D-glucan provides no help for diagnosing mucormycosis, and cultures are often delayed or negative, preventing early management. Delayed directed antifungal treatment impacts the outcome of mucormycosis. The goal of this study is to evaluate a new quantitative PCR assay allowing the simultaneous detection of *Aspergillus* spp. and Mucorales spp.

**Methods:** The MycoGENIE Aspergillus-Mucorales spp. real-time PCR kit (Ademtech, France) is a triplex PCR assay targeting specifically *Aspergillus* spp., Mucorales spp. (including *Lichtheimia, Mucor, Rhizomucor, Rhizopus* and *Cunninghamella* species), and an exogenous Internal Control. This study was performed on a reference panel of 30 biopsy specimen, including 10 positive for *Aspergillus* spp. and 12 positive for Mucorales spp. previously identified by direct examination and culture. Twenty negative serum samples were also included in this study to evaluate the specificity of the assay. DNA extraction was performed using the MycoGENIE DNA Extraction kit (Ademtech, France) and 10 µL of pure DNA was used as a template for the PCR assay. Mucorales-infected samples were also evaluated with other external laboratory-developed PCR tests targeting only *Mucor*, *Rhizomucor*, *Rhizopus* and *Lichtheimia* species.

**Results:** On the 30 samples, the MycoGENIE Aspergillus-Mucorales spp. PCR kit successfully detected 19 *Aspergillus*-positive samples including *A. fumigatus*, *A. niger* and *A. nidulans* identified by culture. Mucorales spp. were also detected in 18 samples. Notably, the PCR assay efficiently detected Aspergillosis-Mucormycosis co-infections in 9 samples. All serum samples returned negative.

**Conclusion:** This new CE-IVD compliant real-time PCR assay, combined with an automated DNA extraction process is a sensitive and specific molecular diagnostic tool for the diagnosis of aspergillosis and mucomycosis infections in biopsy samples. The possibility to efficiently detect aspergillosis/mucormycosis co-infections is highlighted in this study and is of importance for a better therapeutic management.

### P115. Comparison of multiplex nested PCR and Maldi tof to identify clinically isolated candida from Sénégal, a preliminary study

BadianeA.SeneA.GayeA.DiongueK.SeckM.C.NdiayeM.GangneuxJ.P.NdiayeD.Parasitology and Mycology, Universite Cheikh Anta Diop de Dakar, Dakar, Senegal

**Objectives:** The objective of this study is to compare the identification of clinically isolated candida species using Maldi tof and a multiplex nested PCR.

**Methods:** A total of 38 clinically isolated strains of Candida from superficial mycosis and vaginal infection were conserved in brain heart broth additioned with 15 % glycerol and stored at – 20 °C. The strains were then sub cultured in *CHROMagar*™ candida (Becton Dickinson), incubate at 37 °C for 24 to 48 hours and identified using Maldi tof (Bruker) at University of Rennes / France. A multiplex nested PCR were performed directly from the conservative media at Cheikh anta Diop University of Dakar/ Senegal.

**Results:** Thirty five (35) samples gave interpretable results for both methods. 51.43 % of the samples showed the same result with Maldi tof and multiplex PCR, among which *Candida albicans* (45.71 %) and *C. parapsilosis* (2.85%). The species identified by both methods were *C. albicans*, *C. tropicalis* and *C. parapsilosis*. *C. albicans* alone or mix with other species was identified 71.73 % by Maldi tof and 61.70% by PCR nested multiplex. *C. tropicalis* alone or mix with other species was identified 13.04 % with Maldi tof and 8.51% with multiplex nested PCR. *C. parapsilosis* alone or mix with other species was identified 2.17 % with Maldi tof and 23.40% with multiplex nested PCR. *C. glabrata*, *C. pintolepesii*, *C. orthopsilosis* and *C. pararugosa* were identified only by Maldi tof. *C. lusitania* and *C. kefyr* were only identified by Mutiplex nested PCR. Seven (7) mix species were identified with multiplex nested PCR and five (5) with Maldi tof. Among the samples, identified as *C. albicans* using multiplex PCR three were identified as *C. tropicalis* by Maldi Tof. *C. parapsilosis* genotype 2 identified by multiplex PCR has been identified as *C. orthopsilosis* with Maldi tof. Threes strains identified as *C. albicans* by multiplex PCR were identified as *C. tropicalis* by Maldi tof.

**Conclusion:** The multiplex nested PCR and the Maldi tof identified the most prevalent Candida species, but there is discordance for the identification of the non albicans species. The multiplex PCR identified more multiple strains infections than the Maldi tof. The differences observed between these two methods could be due to the absence of these strains in the Maldi tof database and the subculture of predominant strains. Also genetically close species might not be differentiated using PCR.

### P117. Utilization of MALDI-TOF MS for identification of filamentous fungi: a comparative study

VavrovaA.^1^SvobodovaL.^1^MrazekJ.^2^HamalP.^1^1Department of Microbiology, Palacký University, Faculty of Medicine and Dentistry and University Hospital Olomouc, Olomouc, Czech Republic2Department of Bacteriology and Mycology, Institute of Public Health in Ostrava, Ostrava, Czech Republic

**Objectives:** Identification of filamentous fungi based on morphological characteristics is the most simple and available approach used in diagnostic clinical mycology laboratories. In many cases, however, it requires considerable experience of the rater and the ability of strains to produce spores. For its rapidity and accuracy, MALDI-TOF mass spectrometry is invaluable for identification of microorganisms including fungi. It is more challenging to use this method for identification of filamentous fungi than bacteria or yeasts given differences in cell wall structures and a limited spectrum of species in the database. The study aimed to find the optimal way of identifying filamentous fungi in mycology laboratories using MALDI-TOF MS.

**Methods:** The tested group of moulds comprised 193 isolates obtained from clinical specimens. The identification started with morphological assessment (appearance of colonies, micromorphology from slide culture). Then isolates were identified using MALDI-TOF, both directly from culture and following culture in liquid media with extraction; in the latter case, both supernatant and sediment were tested. Finally, all the methods were compared as to the rate of agreement. Subsequently, a small sample of selected isolates was identified by sequencing.

**Results:** Based on morphological criteria, 17 genera were identified; due to a lack of sporulation, another 5 isolates could not be identified. Further, 13 species were identified, mostly within the genus *Aspergillus*. With MALDI-TOF MS performed directly from culture, nine isolates were identified to the genus level and 184 were identified to the species level, with a total of 75 species being noted. With MALDI-TOF MS after culture in liquid media with extraction, 190 isolates were identified to the species level, with 43 species being noted; in only three cases, identification of filamentous fungi was limited to genera. Comparison of identification from supernatant and sediment showed that identical results were always obtained at both the genus and species level and there were only minimal differences in identification scores. The rates of agreement between isolate identification using morphology and MALDI-TOF MS from culture were 58.55% at the genus level and 22.24% at the species level. The rates of agreement between isolate identification using morphology and MALDI-TOF MS after culture in liquid media with extraction were 84.97% at the genus level and 46.11% at the species level. Based on disagreeing results of the above identification methods, 20 isolates from the sample were selected to be identified by sequencing. The highest agreement rate (70%) was found for identification with MALDI-TOF MS after culture in liquid media with extraction and only 35% using MALDI-TOF MS directly from culture.

**Conclusion:** The results suggest that the optimal approach to identification of filamentous fungi in diagnostic medical mycology laboratories is a combination of morphological characteristics and MALDI-TOF MS. For species identification of morphologically typical isolates, assessing the appearance of colonies and slide cultures is sufficient; in the other cases, MALDI-TOF MS after culture in liquid media with extraction is recommended. If identification with the above combination is ambiguous or impossible to perform, molecular genetic methods need to be used, in particular sequencing.

### P118. Risk Factors for Candidemia: A Prospective Matched Case-Control Study

PoissyJ.^1^DamontiL.^2^BignonA.^3^KhannaN.^4^KietzellM. Von^5^BoggianK.^5^NeofytosD.^6^VuottoF.^7^CoiteuxV.^8^ArtruF.^9^ZimmerliS.^2^PaganiJ.-L.^10^CalandraT.^11^SendidB.^12^PoulainD.^12^DeldenC. Van^6^LamothF.^13^MarchettiO.^11^BochudP.-Y.^11^1Intensive Care Department, University Hoospital of Lille, Lille Cedex, France2Department of Infectious Diseases, Inselspital, Bern University Hospital, University of Bern, University Hospital of Bern, Bern, Switzerland3Surgical Intensive Care Department, University hospital of Lille, Lille Cedex, France4Division of Infectious Diseases and Hospital Epidemiology, University Hospital of Basel, Basel, Switzerland5Department of Infectious Diseases, Cantonal Hospital of Saint Gallen, Saint Gallen, Switzerland6Infectious Diseases, University Hospital of Geneva, Geneva, Switzerland7Infectious Diseases Department, University Hospital of Lille, Lille Cedex, France8Hematological Disorders Department, University Hospital of Lille, Lille Cedex, France9Digestive Intensive Care Department, University Hospital of Lille, Lille Cedex, France10Intensive Care Department, University hospital of Lausanne, Lausanne, Switzerland11Infectious Diseases Department, University Hospital of Lausanne, Lausanne, Switzerland12Laboratory of Mycology and Parasitology, University Hospital of Lille, Lille Cedex, France13Microbiology Institute, University Hospital of Lausanne, Lausanne, Switzerland

**Objectives:** Candidemia is an opportunistic infection associated with high morbidity and mortality in hospitalized patients, both inside and outside intensive care units (ICUs). Identification of patients at risk for preemptive approach and early detection is crucial. Prospective control-matched studies and comparison between ICU and non-ICU patients are lacking in this field. We aim to identify and compare specfic risk factors in ICU and non ICU patients.

**Methods:** This was a prospective multicenter matched case-control study assessing risk factors for candidemia and death in candidemic patients, both outside and inside ICUs from 6 teaching hospitals in Switzerland and France. Controls were matched to cases based on age, hospitalization ward, hospitalization duration and, when applicable, type of surgery. Risk factors were analyzed by univariate and multivariate conditional regression models, as a basis for a new scoring system for prediction of candidemia.

**Results:** The study included 183 cases and 378 matched controls. 44% were hospitalized inside ICU and 56% outside. Independent risk factors for candidemia within the ICU population included total parenteral nutrition (TPN) (OR = 9.24, 95%CI 3.39-25.2, p<0.001), acute kidney injury (OR = 6.48, 95%CI 2.39-17.6, p<0.001), solid cancer (OR = 4.59, 95%CI 1.23-17.2, p = 0.02), heart disease (OR = 4.26, 95%CI 1.37-13.3, p = 0.01), invasive mechanical ventilation (OR = 3.37, 95%CI 1.17-9.67, p = 0.02), exposure to fluoroquinolones (OR = 3.05, 95%CI 1.14-8.16, p = 0.03) and exposure to aminoglycosides (OR = 2.47, 95%CI 1.07-5.69, p = 0.03). Independent risk factors for candidemia within the non-ICU population included central venous catheter (CVC) (OR = 10.20, 95%CI 3.87-27.2, p<0.001), TPN (OR = 3.37, 95%CI 1.53-7.41, p = 0.003), exposure to glycopeptides (OR = 4.10, 95%CI 1.57-10.7, p = 0.044), and to nitroimidazoles (OR = 5.14, 95%CI 1.59-16.6, p = 0.006). The weighted ICU-score was as follows: TPN, +2; AKI, +2; heart disease, +1.5; solid cancer, +1.5; invasive mechanical ventilation, +1.0; fluroquinolone: +1.0. aminoglycoside: +1. AUC of the ROC curve was 0.773. The optimal cut-off was ≥5 (sensitivity = 76%, specificity = 64%). The bestl cut-off to optimize specificity was ≥7 (sensitivity = 23%, specificity = 95%). The weighted non-ICU score was as follows: CVC: +3.5; nitroimidazole: +2.5; TPN, +2; Glycopeptide: +2.0. AUC of the ROC curve was 0.717. The optimal cut-off was ≥4 (sensitivity = 83%, specificity = 49%). The best cut-off to optimize specificity was ≥6 (sensitivity = 49%, specificity = 82%). Independent factors for death in candidemic patients in the whole population were septic shock (OR = 7.17, 3.06-16.8, p<0.001), acute kidney injury (OR = 5.04, 2.16-11.8, p<0.001), number of antibiotics (OR = 1.42, 1.15-1.75, per unit, p<0.001). After stratification between ICU and non-ICU patients, only septic shock (OR = 4.49, 1.53-13.1, p = 0.006), acute kidney injury (OR = 3.13, 1.02-9.17, p = 0.04), the number of antibiotics to which patients were exposed before candidemia (OR = 1.35, 1.05-1.75 per unit, p = 0.02) remain significantly and independently associated with death in ICU patients. For non-ICU patients, acute kidney injury (OR = 10.7, 2.16-52.5, p = 0.004) and septic shock (OR = 10.50, 2.80-39.7, p<0.001) were the only variables significantly associated with death

**Conclusion:** Risk factors for candidemia are variable in the ICU versus non-ICU setting, including different patterns of antibiotic exposure. Weighted scores predictive of candidemia can be built based on these risks, with better performances for ICU-patients. An improved prediction of the risk of candidemia may contribute to guide targeted preventive and therapeutic antifungal strategies.

### P119. Inter-laboratory robustness of screening tests for the detection of Aspergillus antibodies for chronic aspergillosis: finding from a French External Quality Assessment

PersatF.^1^,^2^DupontD.^3^EynardJ.-C.^2^PoggiB.^2^WallonM.^3^1Parasitology and Medical Mycology, Hospices Civils de Lyon / Université Lyon 1, Lyon, France2Association Pro.Bio.Qual, Lyon, France3Parasitology and Medical Mycology / Umr 5292, Université Lyon 1 / Hospices Civils de Lyon, Lyon, France

**Objectives:** Six million people worldwide are estimated to have chronic aspergillosis or/and allergic broncho pulmonary aspergillosis. Serum *Aspergillus* assay is an important parameter to detect and follow the immune host response against this fungus. Reliable tests are crucial for patients’ care but also to perform much needed multicentre-studies. However, the current tests are not standardized and may lack in robustness. We have analysed the results of a French External Quality Assessment (EQA) programme to assess the inter-laboratory robustness of four *Aspergillus* antibody techniques among those recommended in France as first-line screening tests.

**Methods:** The study was based on the answers provided by 40 laboratories that took part at least once to the EQA organised by ProBioQual between 2013 to 2018 for *Aspergillus* serology. A total of 24 sera were tested over this time period, 18 positive from patients with chronic or allergic bronchopulmonary aspergillosis and six negative sera from blood donors. The results of the following techniques were analysed: 1) Platelia^TM^*Aspergillus* IgG EIA (Bio-Rad, France); 2) ELISA Classic^TM^*A. fumigatus* IgG (Virion/Serion, Germany); 3) ELISA *Aspergillus fumigatus* IgG (Bordier Affinity Products, Switzerland) since 2014; 4) Aspergillose Fumouze indirect hemagglutination (Biosynex Fumouze, France).

**Results:** reported by the participants for the Bio-Rad test were all negative for five of the six negative sera and all positive for 14 of 18 positive sera. Those reported for the Virion/Serion ELISA were all negative for four of the six negative sera and all positive for 10 of the 18 positive sera. The results reported for the Bordier test were all concordant but only based on five positive sera and one negative serum. Results provided with the hemagglutination test were all negative on all negative sera, but none of 18 positive sera were found by all laboratories to be positive, and 10 of the positive sera were even found by all laboratories to be negative.

**Conclusion:** Hemagglutination technique appears lacking too much in sensitivity to be used as single screening test, compared to the ELISA techniques, probably explaining its decreasing use. ELISA Bio-Rad appeared as more robust than Virion/Serion ELISA. The potentially better performances of ELISA Bordier have to be confirmed.

### P120. rUsefulness of conventional PCR through induced sputum to diagnoses Pneumocystis pneumonia in HIV infected patients

ChagasO.J.^1^BuccheriR.^2^NavesG.^1^PintoP.L. Silva^3^ChioccolaV.L. Pereira^4^BenardG.^1^NegroG.M.B. Del^1^1Laboratório De Investigações Médicas 53, Insituto de Medicina Tropical, São Paulo, Brazil2Hospital Ward, Instituto de Infectologia Emílio Ribas, São Paulo, Brazil3Intitution: Intituto Adolfo Lutz - Núcleo de Enteroparasitas, São Paulo, Brazil4Intituto Adolfo Lutz - Laboratório de Biologia Molecular de Parasitas e Fungos do Centro de Parasitologia e Micologia, São Paulo, Brazil

**Objectives:** The present study aims to determine the usefulness of conventional PCR in induced sputum to diagnoses *Pneumocystis* pneumonia in HIV infected patients.

**Methods:** Study design was of a prospective-cohort with systematic collection of data. Ninety-five HIV-infected patients presenting cough and dyspnoea for more than 7 days were included. They should not have received antiretroviral therapy (ART) or a history of inadequate treatment. Induced sputum, obtained through concentrated saline solution (NaCl 3%-5%) inhalation, was obtained at admission and submitted to O-toluidine staining and conventional PCR for *Pneumocystis*. Clinical, laboratorial, imaging records and clinical outcome from PCR positive and PCR negative patients were compared. We also compared these data in PCR positive patients and confirmed pulmonary tuberculosis (TB) (presence of bacillus-acid-alcohol-resistant or positive rapid-molecular-test).

**Results:** PCR was positive in 34/95 (35.7%) of the patients. This group presented higher rates of dry cough (55.8% X 22.9%, P = 0.003), interstitial infiltrate on chest X-ray (94.1% X 61%, P = 0.001), ground grass on chest tomography (CT) (89.2% X 42.8%, P = 0.0002) and higher lactate dehydrogenase (LDH) levels (488U/L x 329U/L, P<0.0001), when comparing with the PCR negative patients. PCR positive patients had also more non-invasive ventilation (NIV) (47% X 15%, P = 0.002) and more admissions at the intensive care unit (ICU) (35.2% X 13.5%, P = 0.02). Both groups did not differ regarding n. of days with symptoms, PaO2, HIV viral load (VL) and *Candida* infection during hospitalization. There were 32 PCR positive patients without TB and 11 patients with confirmed pulmonary TB. TB was associated with CT budding tree (57,1% X 0, P = 0.0005), CT micronodules (85,7% X 23%, P = 0.008); trend toward higher CD4 cell count (129 X 47 cell/mm³, P = 0.01) and lower use of NIV (6,2% X 50%,P = 0.0417), and less CT ground grass (14,2% X 92,3%, P = 0.0001). There was no difference between LDH, PaO2, HIV VR, and ICU hospitalization between the two groups. of note, 5/34 (14.7%) of the PCR positive patients did not receive any antimicrobial treatment for PCP during hospitalization but improved their initial symptoms, hence the difficult to differentiate between colonization and infection solely based on the conventional PCR. In addition, only two PCR positive patients did not display ground grass at CT: both had pulmonary consolidation.

**Conclusion:** Our PCR assay was useful to define a subgroup of patients with clinical, laboratorial and imaging aspects related to PCP. Besides it, pulmonary CT is a fundamental tool, since suggestive alterations in this exam strongly contribute to the diagnosis, helping to differentiate the pulmonary involvement of PCP from that of TB, the main differential in our country, where TB is highly prevalen. of note, five PCR positive patients did not receive specific treatment during hospitalization, pointing to either the existence of false-positive cases or to *P. jiroveci* colonization. The possibility that differentiation between these patients could be achieved by quantitative real-time-PCR remains an open question, since the literature is controversial, due mainly to the different primers and techniques employed. Another discriminatory assay would be the β-D-Glucan test, which, combined with PCR assay, would be able to discriminate colonized from infected patients.

### P121. Performance characteristics of the MucorGenius® real-time PCR assay for the detection of clinically relevant Mucorales species

GaajetaanG.KampermannT.TegelenD. VanDingemansG.PathoNostics, Maastricht, Netherlands

**Objectives:** Determine the performance characteristics of the commercially available MucorGenius® real-time PCR assay for the detection of clinically relevant Mucorales species. For this purpose, several verification studies were performed including sensitivity, specificity and EQA evaluation.

**Methods:** The analytical sensitivity or limit of detection (LOD) was determined by testing serial dilutions of all reference Mucorales culture extracts including *Rhizopus oryzae*, *Rhizopus microsporus*, *Cunninghamella bertholletiae*, *Lichtheimia corymbifera*, *Rhizomucor pusillus*, *Mucor hiemalis* and *Rhizomucor miehei* on various real-time PCR instruments. The LOD dilution of a Mucorales species was accepted if at least 19 out of 20 reactions were detected. This dilution was finally quantified for each species by using droplet digital PCR (ddPCR). Analytical specificity was determined by testing multiple high concentrations of bacterial and fungal DNA strains that can be present in the respiratory tract. The seven serum samples of the FPCRI Muc Panel (2018-1) were tested with the MucorGenius® PCR after DNA-extraction with the NucliSENS® easyMag system (bioMérieux).

**Results:** The LOD was successfully determined for all seven Mucorales targets on 5 different real-time cyclers. All results of the LOD study are listed in Table 1. *Table 1. Overview of MucorGenius® LOD results.*

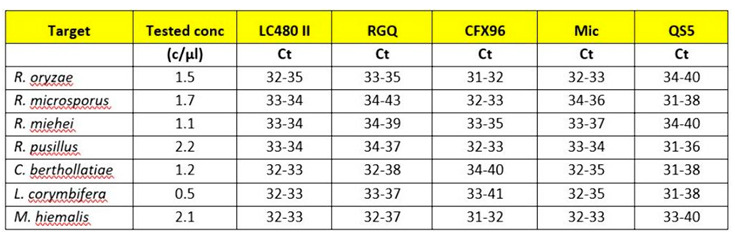



No cross-reactivity was observed with any of the clinically relevant fungal or bacterial strains. Mucorales DNA was successfully detected in 6 out of 7 serum DNA- extracts from the FPCRI panel. All samples showed an internal control signal indicating reliable DNA-extraction performance. These results were in agreement with the data of the FPCRI.

**Conclusion:** The MucorGenius® real-time PCR assay can detect all Mucorales species at low DNA-concentrations of 1-2 copies/µl which corresponds with high Ct-values (Ct 31-43). This indicates reliable analytical sensitivity. No cross-reactivity was observed for any of the tested fungal or bacterial strains, reducing the chance of a false positive result. Although 100% agreement was obtained with the FPCRI Muc Panel, this is a limited data set and only provides an indication of the performance. Clinical validation studies are now ongoing on different sample types to determine the clinical performance and suitability for routine diagnostics.

### P122. Time to and differential time to blood culture positivity for assessing catheter-related candidemia: A longitudinal, 7-year study in a single university hospital

Gits-MuselliM.^1^,^2^VilliersS.^3^HamaneS.^2^BerçotB.^4^DonayJ.-L.^4^DenisB.^5^GuigueN.^2^AlanioA.^6^BretagneS.^6^1Molecular Mycology, Institut Pasteur, PARIS, France2Parasitology-mycology Laboratory, Lariboisière Saint-Louis Fernand Widal Hospital, Assistance Publique-Hôpitaux de Paris (AP-HP), PARIS, France3Anesthesiology Department, Lariboisière Saint-Louis Fernand Widal Hospital, Assistance Publique-Hôpitaux de Paris (AP-HP), PARIS, France4Microbiology Department, Lariboisière Saint-Louis Fernand Widal Hospital, Assistance Publique-Hôpitaux de Paris (AP-HP), PARIS, France5Tropical and Infectious Diseases Department, Lariboisière Saint-Louis Fernand Widal Hospital, Assistance Publique-Hôpitaux de Paris (AP-HP), PARIS, France6Paris-diderot, Sorbonne Paris Cité University, Institut Pasteur, Molecular Mycology Unit, Cnrs Cmr2000, Parasitology-Mycology Laboratory, Lariboisière Saint-Louis Fernand Widal Hospitals, Assistance Publique-Hôpitaux de Paris, Paris, France

**Objectives:** Central venous catheters (CVCs) significantly increase the risk of candidemia, and inversely, candidemia is often associated with intravascular catheters. Most guidelines for candidiasis management recommend removal of existing intravascular catheters, if feasible. Although the recommendation is strong, various authors have recommended a re-consideration of CVC removal on an individual basis. Practically, one must consider the possible deleterious consequence of CVC removal, especially in patients who require long-term CVC for treatment or nutrition. This raises the question of systematic early removal of CVC in the management of candidemia since some authors did not report differences, knowing that patients who cannot tolerate CVC removal have often a worse prognosis. Time to positivity (TTP) and differential time to positivity (DTTP) between central and peripheral blood cultures are commonly used for bacteremia to evaluate the likelihood of CVC related bloodstream infection. Few studies have addressed the application of these approaches to candidemia. This study aimed to evaluate TTP and DTTP to assess CVC-related candidemia (CVC-RC).

**Methods:** We retrospectively analyzed the results from 105 adult patients with incident candidemia, with CVC removed and cultured, collected from 2010 to 2017. The bottles were incubated in a BioMérieux bact/ALERT 3D and kept for at least 5 days. Patients with CVC removed and cultured at the time of fungemia were divided into two groups as previously proposed: (i) CVC-related candidemia (CVC-RC) when the same yeast species was recovered from at least one BC and from the CVC culture; (ii) non-CVC-related candidemia (NCVC-RC) when a yeast species was recovered from at least one BC, and the CVC culture was negative or yielded a different species.

**Results:** of the 105 patients included, most were oncology patients (85.7%) and had of long-term CVC (79.6%); 32 (30.5%) had a positive CVC tip with the same species as in BC, defined as CVC-RC. The main species involved were *C. albicans* (46%), *C. parapsilosis* (19%), *C. glabrata* (15%), and *C. tropicalis* (9%), with no statistical difference between NCVC-RC and CVC-RC. Considering all species, the median TTP of the first bottle collected from CVC was statistically shorter in the CVC-RC group (16.8 h [9.7-28.6]) compared to the NCVC-RC group: median (29.4 h [20.7–41.3], p = 0.001, Figure 1). When analyzing TTP regarding the *Candida* species, a significant shorter TPP was only observed in *C. albicans* with a median of 12.9 h [10.0–22.7] *vs*. 32.3 h [24.4–41]) for CVC-RC and NCVC-RC, respectively (p = 0.001, Figure 1). A TTP < 10 h had the best positive likelihood ratio (21.5), although the sensitivity was only 28%. DTTP was available for 52 patients. A DTTP > 5 h had a sensitivity of 100% and a specificity of 71%.

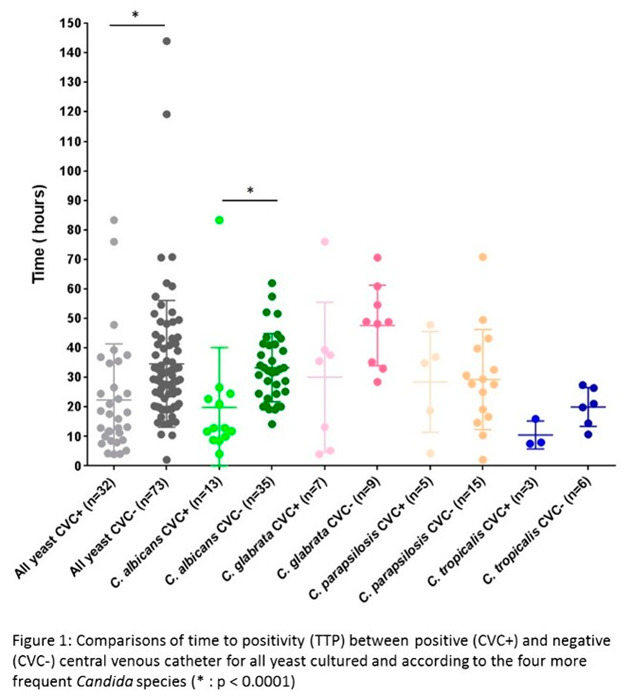



**Conclusion:** Even if some cut-offs supported the diagnosis of CVC-RC, they did not allow the prompt decision on CVC removal, since the median TTP was 17 h. More rapid methods for detecting infected catheters should be tested to avoid withdrawal of non-infected CVC (69.5% of CVCs in our study).

### P123. Diagnostic value of immunological markers of allergic bronchopulmonary aspergilosis in patients with asthma

KozlovaY.^1^FrolovaE.^2^UchevatkinaA.^3^FilippovaL.^1^SolovjevaG.^1^SobolevA.^1^VasilyevaN.^1^KlimkoN.^1^1Department of Clinical Mycology, Allergology and Immunology, North-Western State Medical University named after I.I. Mechnikov, Saint-Petersburg, Russian Federation2Department of Clinical Mycology, Allergology and Immunology, North-Western State Medical University n.a. I.I. Mechnikov, Saint-Petersburg, Russian Federation3North-Western State Medical University n.a. I.I. Mechnikov, Saint-Petersburg, Russian Federation

**Objectives:** Allergic bronchopulmonary aspergillosis (ABPA) is a severe lung disease caused by hypersensitivity to *Aspergillus* spp. The study of the role of different immunological mediators in the formation of chronic allergic inflammation in patients with ABPA is necessary to identify potential targets for therapeutic intervention and timely diagnosis of the disease. To study of the role of immunological mediators in the development of allergic bronchopulmonary aspergillosis in patients with asthma.

**Methods:** A prospective study conducted 58 patients with severe asthma (median of age - 45 years, men - 13, women - 45). The control group consisted of 16 apparently healthy people without allergic disease in history (median age - 24 years old, male - 4 women - 12). All patients underwent allergological (skin tests with fungal allergens, determination of serum levels of total IgE (“Polygnostus”, Russia) and specific IgE (sIgE) for fungal allergens (“AlcorBio”, Russia). Computed tomography of the chest was performed according to the indications. Diagnostic criteria of ABPA 2013 [R. Agarwal et al] were used. The concentration of TARC (“R&D Systems”, USA), TSLP (“R&D Systems”, USA), IL-8 (“Vector-Best”, Russia) in the serum were determined by ELISA. All patients were performed respiratory function and used the AST questionnaire (Asthma Control Test). The obtained data was processed using the STATISTICA 10 software system.

**Results:** ABPA patients demonstrated significantly lower scores on the questionnaire AST and worse lung function FVC and FEV1 compared to patients with asthma. The analysis of the content of TSLP in serum did not establish statistically significant differences between patients with ABPA 13.0 (9.70 ÷ 24.70) and asthma 19.0 (15.0 ÷ 29.0), and data from the control group 10.5 (9.0 ÷ 25.0) pg/ml. It was revealed that the level of IL-8 in patients with ABPA 35.0 (23.0 ÷ 49.0) was significantly higher than in patients with asthma 22.0 (14.4 ÷ 28.0; p = 0.002) and in healthy individuals (12.0 (4.5 ÷ 15.5); p = 0.000) pg/mg. The content of TARC was higher in patients with ABPA 733.5 (599.0 ÷ 909.0) compared with asthma patients 336.1 (208.0 ÷ 571.3; p = 0.000) and control 224.5 (184.0 ÷ 265.0; p = 0.000) pg/mg. The importance of TARC in the development of allergic inflammation in patients with fungal sensitization was confirmed by a positive correlation of the content of the proinflammatory chemokine with the levels of total IgE (r = 0.35, p <0.05), sIgE to *A.fumigatus* (r = 0.39, p <0.05), the number of eosinophils (r = 0.34, p <0.05) and a negative correlation with the indicators of the respiratory function of FEV1 (r = -0.44; p <0.05). The high content of TARC and IL-8 suggests a mixed eosinophilic-neutrophilic type of inflammatory response in patients with ABPA.

**Conclusion:** An increase in serum TARC can serve as a diagnostic biomarker for the development of ABPA in patients with asthma.

### P124. Comparison of serum beta-D-glucan levels obtained by Fungitell Assay and Wako beta-Glucan Test

MarekovićI.PleškoS.VranješV. RezoJandrlićM.Department of Clinical and Molecular Microbiology, University Hospital Centre Zagreb, Zagreb, Croatia

**Objectives:** Fungitell Assay (FA) is the most widely used test for determining serum beta-D-glucan (BDG) levels in Europe. However, the disadvantage of this test is the need for samples’ cummulation to collect appropriate number of samples assuring optimal test usability. It poses a problem in laboratories with limited number of samples because of prolonged turnaorund-time and questionable impact on the diagnostic process and antifungal treatment of invasive fungal infections (IFI). Wako β-Glucan Test (WBG), recently launched in Europe, offers the possibility of individual sample testing. The objective of this study was to compare serum beta-D-glucan levels obtained by these two assays in patients with suspected IFI.

**Methods:** The study was performed at the University Hospital Centre Zagreb, Department of Clinical and Molecular Microbiology. The Fungitell Assay (Associates of Cape Cod Inc.) and Wako β-Glucan Test (FUJIFILM Wako Chemicals Europe Gmbh) were performed on patients’ sera and the results were interpreted according to manufacturers’ instructions. Two to three serum samples were tested in each patient on two or three consecutive days. Discrepant results between two tests were explained and commented in the context of patients’ clinical data.

**Results:** Forty-four serum samples in 24 patients were tested. FA was positive in 13 and WBG in 9 patients. The results obtained with two different tests regarding positivity and negativity were correspondent in 83,3% (20/24) of patients. In 16,7% (4/24) of patients, the results were discordant, with positive FA and negative WBG result. Results for four patients with discordant FA and WBG results are shown in Table 1. When cut-off value for WBG was lowered from manufacturer’s cut-off value of 11 pg/mL to 3,8 pg/mL, as suggested in a recent publication, all the results, except one result in Patient 1, were concordant. Moreover, in Patient 1 diagnosis of IFI was excluded after diagnostic work-up as well as in Patient 2. In Patient 3, with a recent heart transplant, chest HR-CT was done eight days after last serum sample tested with both tests, demonstrated pulmonary infiltrates suspected by radiologist to be of fungal etiology and subsequently determined levels of BDG with FA only showed further increase. *Aspergillus fumigatus* grew in a culture of sputum and patient was treated with voriconazole. Patient 4 had a cystic fibrosis with *A. fumigatus* isolated in sputum and treated with voriconazole. In Patients 1, 2, and 4 the highest level of BDG determined with WBG was 5,1 pg/mL, while in a Patient 3 the highest level was 9.62.

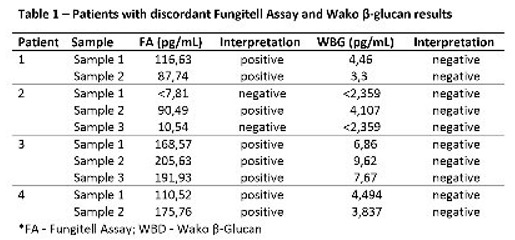



**Conclusion:** In our study FA was more frequently positive in comparison to WBG test. Among patients with positive FA and negative WBG was a also a patient with IFI. This is in concordance with so far conducted studies showing superior sensitivity of FA. Therefore, cut-off value for WBG should be reconsidered. As the results of any of these tests should be evaluated in the context of clinical picture and microbiological and radiological findings, WBG test remains a useful alternative for laboratories with low number of samples.

### P125. An immunonanodiagnostic approach for Pneumocystis pneumonia at point-of-care

TomásA.L.^1^,^2^CardosoF.^1^AlmeidaM.P. De^3^PereiraE.^3^FrancoR.^2^MatosO.^1^1Medical Parasitology Unit, Group of Opportunistic Protozoa/hiv and Other Protozoa, Global Health and Tropical Medicine, Instituto de Higiene e Medicina Tropical, Universidade Nova de Lisboa, Lisboa, Portugal2Departamento De Química, UCIBIO/REQUIMTE, Universidade NOVA de Lisboa, Caparica, Portugal3Departamento De Química E Bioquímica, REQUIMTE/LAQV, Faculdade de Ciências da Universidade do Porto, Porto, Portugal

**Objectives:** *Pneumocystis jirovecii* is an ubiquitous opportunistic fungus capable of causing fatal pneumonia (PcP) in immunocompromised patients worldwide. The diagnosis relies on microscopic visualization of the *P. jirovecii* organisms or on DNA detection, in respiratory specimens obtained by invasive and costly techniques. Thus, the search for a specific serological biomarker and for a faster, cost-effective and less-invasive diagnostic approach is an old ambition of the scientific community. This work aims to develop a point-of-care strip-based platform for anti-*P. jirovecii* antibodies detection in human serum specimens, applying the innovative association of recombinant synthetic antigens (RSA) technology with functionalized gold nanoparticles (AuNPs).

**Methods:** *P. jirovecii’*s surface proteins such as the major surface glycoprotein (Msg) and kexin-like serine protease (Kex1) present highly reactive antigenic properties. Thus, newly recombinant synthetic (multi-epitope) antigens from these proteins were designed based on immunogenicity of their sequences. After synthesis and purification by immobilized metal affinity chromatography, these RSA were applied in ELISA tests as biomarkers of the disease to identify circulating anti-*P. jirovecii* antibodies in serum specimens of patients previously classified in distinct clinical groups. After confirming their applicability as biomarkers of infection, these RSA were conjugated with spherical AuNPs previously functionalized with mercaptoundecanoic acid. The interaction of such RSA-AuNP bionanoconjugates with specific anti-*P. jirovecii* antibodies in patient’s sera, was characterized by agarose gel electrophoresis (AGE). The same RSA-AuNP bionanoconjugates were then utilized in two strip-based lateral flow immunoassays (LFIA) prototypes, projected to detect the presence of human IgM reactive to each of the *P. jirovecii’*s RSA. These prototypes were optimized and tested with positive and negative clinical samples.

**Results:** A synthetic antigen with three reactive Msg epitopes and another with three Kex1 reactive epitopes were produced. ELISA results with both RSA showed that IgM anti-*P. jirovecii* levels were significantly increased in patients with PcP compared with patients colonized by *P. jirovecii* (*p* = 0.002 with Kex1 RSA; *p*<0.001 with Msg RSA) or patients without *P. jirovecii* infection (*p*≤0.001 with both RSA). AGE assays with the RSA-AuNPs bionanoconjugates confirmed that these bionanoconjugates reacted with anti-*P. jirovecii* antibodies present in patient’s serum. The LFIA prototypes developed were tested with sera pools from patients with active PcP (positive sample) and from patients without *P. jirovecii* infection (negative sample). Both samples showed the expected performance, namely, test and control colored lines with the positive sample and only a control colored line with the negative sample.

**Conclusion:** Our results support the possibility to diagnose PcP and distinguish active disease from colonization using RSA as biomarkers of the disease and IgM anti-*P. jirovecii* antibodies as targets for immunonanodiagnosis. Further improvement and validation of the LFIA prototypes developed will help in the global effort to reduce high costs of medical diagnosis and consequently treatment of PcP. This applies not only to more economically advanced regions, but can also be a huge contribution, in terms of public health and economy, in communities with low-income and scarce access to technology.

### P126. Interest of Aspergillus fumigatus Western Blot assay for differential diagnostic between IgE sensitization and Allergic Broncho Pulmonary Aspergillosis.

PiarrouxR.^1^DubusJ.-C.^2^,^3^Reynaud-GaubertM.^3^,^4^GouitaaM.^5^RanqueS.^6^,^7^VitteJ.^8^1Research & Development, LDBIO Diagnostics, Lyon, France2Pneumo-pédiatrie, Crcm Enfants, Aix-Marseille Université AP-HM Hôpital Timone Enfants, Marseille, France3Ihu Méditerranée Infections Mephi, Aix-Marseille Université IRD AP-HM, Marseille, France4Pneumologie Crcm Adultes, Aix-Marseille Université AP-HM Hôpital Nord, Marseille, France5Clinique Des Bronches Et Allergies Du Sommeil, Aix-Marseille Université AP-HM, Marseille, France6IHU-Méditerranée Infection, Marseille, France7Vitrome, Aix Marseille Univ, IRD, AP-HM, SSA, Marseille, France8Ihu Méditerranée Infections Mephi, Aix-Marseille Université IRD AP-HM SSA, Marseille, France

**Objectives:** Diagnosis of Allergic Broncho Pulmonary Aspergillosis (ABPA) is complex and based on a multi-criteria definition. One of the major ABPA criteria, the detection and quantification of immunoglobin E (IgE) responses to *Aspergillus fumigatus* (*Af*), is also associated with *Af*-sensitization. In the past few years, a novel approach assaying IgE responses to molecular antigens enhanced the discrimination between *Af* sensitization and ABPA, but without allowing for a clear-cut distinction between these two diseases. We recently reported that a Western Blot detecting *Af*-specific IgE antibodies could be helpful in separating those two entities. This work aimed to strengthen the evaluation of the *Af* IgE WB assay as a differentiating tool between *Af*-sensitization and ABPA.

**Methods:** 221 sera with known sIgE reactivity (21 ABPA, 200 *Af*-sensitized) were assayed with the LDBIO Asp II WB. Sera were collected 2014-2018 and previously assayed with ImmunoCap® to *Af* extract and molecular antigens. We evaluated the ability of WB LDBIO to detect *Af* sensitization and to discriminate between ABPA and *Af*-sensitized patients, relying on ImmunoCap® and clinical chart conclusions as a reference.

**Results:** Samples displayed 0 to 10 bands in the 10-37 kDa range. Among those bands, four (16, 18-20, 22 and 30 kDa) were most frequent and therefore considered as major bands, while the others were considered as minor bands. *Af* IgE WB positivity was defined by the presence of at least 2 major bands. The *Af* IgE WB was positive in 21/21 ABPA and 115/200 *Af*-sensitization sera (61% sensitivity). *Af* IgE WB positivity was strongly correlated to IgE level and was positive in 95% (75/79) of the sera with IgE to *Af* >2kUa/l. Band patterns formed various profiles for discrimination between ABPA and *Af*-sensitization. of those profiles, one (5 detectable bands, regardless of minor/major classification) had 100% sensitivity (21/21) and 91% specificity (201/221) for ABPA diagnostic. However, by using a more specific profile with 2 major bands and 2 minor bands outside of the 16-30 kDa range, the WB yielded 90% (19/21) sensitivity and 96% (211/221) specificity.

**Conclusion:** This study shows the potential of WB in the work-up of IgE responses to *Af* in asthmatics and cystic fibrosis patients: *Af* IgE WB was able to discriminate between ABPA and *Af*-sensitized patients, with a sensitivity of 90% and a specificity of 96%. In comparison, the ISHAM criteria (tIgE>1000 kUI/l and *Af* IgE>0.35 kUa/l) had 48% sensitivity (10/21) and 98.2% specificity (217/221) in the same population Noteworthy, among the *Af* IgE WB negative patients, 4 were sensitized to another mould (*Alternaria alternata*), thus *Af* IgE WB could avoid unnecessary diagnostic difficulties due to such cross-reactivity. A multicenter evaluation is now needed to confirm those results, but *Af* IgE WB shows a great potential for second-line diagnostic tool when ABPA is suspected.

### P127. Rapid Identification of Candida spp. from positive blood cultures

ÖzenciV.IninbergsK.SeethC. GronforsJanssonK.UllbergM.Clinical Microbiology, Karolinska University Hospital, karolinska Institutet, Stockholm, Sweden

**Objectives:** Candidemia is related to high mortality, morbidity. Rapid and reliable identification of *Candida* spp. and antifungal susceptibility testing is essential for early appropriate antifungal treatment. The aim of the present study was to evaluate two rapid methods for identification of Candida spp. from blood cultures (BC) using matrix assisted laser desorption ionization (MALDI-TOF MS). The methods tested were (i) the Sepsityper kit and (ii) short-term culture followed by MALDI-TOF MS. The effect of using Bact/ALERT FA Plus and BACTEC Mycosis-IC/F blood culture bottles were compared for the performance of both of the rapid methods. The performance of short-term culture on faster antifungal susceptibility testing was also analyzed. Conventional identification and antifungal susceptibility testing methods were used as reference.

**Methods:** In total, 52 clinical Candida spp. were inoculated in two types of BC bottles and incubated in respective BC systems. Each bottle was inoculated with 8 mL human blood from a healthy donor which had been spiked with ~ 125 colony forming units (CFU) per mL. When the BC bottles signaled positive, the samples were analyzed directly from BC bottles by the Sepsityper kit as well as after 6h short-term incubation on sabouraud agar and chrome plates followed by MALDI-TOF MS if growth in 6h was observed.

**Results:** Overall, the Sepsityper kit and short-term culture method performed similarly and could identify Candida spp. in 66/101 (65.3%) and 63/85 (74.1%), respectively (NS). Mycosis-IC/F had higher identification rates than FA Plus BC bottles using the Sepsityper kit 39/51 (77%) and 27/50 (54%), respectively (p<0.05). Similarly Mycosis-IC/F performed better than FA Plus BC bottles using the short-term culture method 38/43 (88%) and 25/42 (60%), respectively (p<0.01). Six hours short term culture followed by Sensititre YeastOne method was tested on ten positive BC bottles. On average the MIC levels for antifungal agents were within one titre difference compared to conventional Sensititre method which was performed after overnight culture plates for both BC bottles (NS).

**Conclusion:** The present data shows that Sepsityper kit and short-term culture method can be used in rapid identification of Candida spp. from blood cultures. Mycosis-IC/F has higher performance in identification of yeast with both methods compared to FA Plus BC bottles. Preliminary data suggest that six hours short term culture followed by Sensititre Yeast One method can be used as a reliable rapid antifungal susceptibility testing method for positive blood cultures.

### P128. Comparison of Bactec Mycosis IC/F and BacT/Alert FA plus bottles in laboratory diagnosis of candidemia

ÖzenciV.^1^LopezN.I.M.^1^SeethC. Gronfors^1^UllbergM.^1^DinnetzP.^2^1Clinical Microbiology, Karolinska University Hospital, karolinska Institutet, Stockholm, Sweden2School of Natural Sciences, Technology and Environmental Studies, Södertörn University, Stockholm, Sweden

**Objectives:** The aim of the present study was to compare the clinical performance of Bactec Mycosis IC/F and BacT/Alert FA blood culture bottles in detection and time to detection of Candida spp. over eight years period in a tertiary care hospital setting.

**Methods:** During a period of eight years, all blood samples that were concurrently taken from patients with suspected candidemia and cultured paralleled in Bactec Mycosis IC/F, BacT/Alert FA, and BacT/Alert FN blood culture bottles were included in the study. Data collection was retrieved from the laboratory information system and from the respective blood culture system. Bottles that did not signal positive in 10 days in the blood culture system were regarded as negative. Time to growth was counted from the time that the incubation occurred until the time the system gave a positive signal. Results analyzed with use of R, conducting a mixed linear model and Chi-square test.

**Results:** In total, 2,748 positive blood cultures with Candida spp. i.e Bactec Mycosis IC/F, BacT/Alert FA Plus, and BacT/Alert FN Plus bottles from 810 patients were analyzed. Triplicates of blood culture bottles that were taken in 24 h were included. There were 274 blood cultures from 261 patients with all three blood culture bottle types. The BacT/Alert FN bottles had too few positives and excluded from further analysis. The Bactec Mycosis IC/F Bottles could detect Candida spp. in 216 (78.83%) of 274 samples, whereas BacT/Alert FA aerobic bottles were able to detect in 233 (85%) of 274 samples. The Mycosis IC/F and BacT/ Alert FA aerobic Bottles did not differ in detection of Candida spp. (NS). There was no species-specificity difference between the two bottles in growth of different Candida spp (NS). There was no difference in to time to detection between the two bottles (NS).

**Conclusion:** The present study shows that the performance of Bactec Mycosis IC/F and BacT/ Alert FA blood culture bottles in detection of Candida spp. are similar. Further analysis of the discrepant results and the patient characteristics will be performed.

### P129. Direct identification of 81% of candidemia from blood culture bottles by MALDI –TOF MS

VanniniM.ChandemerleE.LandreauA.SimonL.PomaresC.HasseineL.Laboratoire De Parasitologie Mycologie, CHU DE NICE, NICE, France

**Objectives:** Candidemia are associated with high mortality rate (40%). Rapid yeast species identification is crucial to introduce as soon as possible appropriate treatment which is associated with an increase of patients survival rate. Because of the emergence of echinocandin resistance, strategies that allow targeted use of antifungals are essential, notably to reduce the duration of echinocandins exposition. With the conventional method, 24 to 48 hours are required to obtain enough fungal colonies for species identification. In this study, we developed a method to directly identify yeast from blood cultures bottles within one hour. In the context of Laboratory Accreditation we performed method validation according to the ISO 15189 norm.

**Methods:** This work was done in the laboratory of parasitology and mycology at the University Hospital Center of Nice, France. We used 37 positive blood cultures obtained from 25 patients from 4^th^ January 2019 to 28 Mars 2019. 1.3 ml of positive blood cultures was transferred into 1.5 ml microtube and each sample was extracted according to the SDS lysis-based protocol (Jeddi *et al* 2017). Supernatants were spotted four times on the target plates that were read by the MADLI-TOF mass spectrometer (Bruker®). We validated species identification when a log (score) from Bruker either ≥ 1.6 or ≥ 1.1 three-times repeatable was found, since we did not observe any species misidentification above these thresholds. In case of failure, spectra were analyzed online with the MSI-Users® database (Mass Spectrometry Identification, Normand *et al 2017)* and identifications were validated when the score was ≥ 17%. We compared our results with the conventional culture and identification methods.

**Results:** 30 blood cultures (81%) were correctly identified with both databases. Only 68 % with Bruker database and 73 % with MSI User database. This method is reproducible and reliable. We tested two mixed infections but they were not totally identified.

**Conclusion:** Our study shows that direct identification of candidemia from blood culture with the SDS protocol allows rapid identification of the causative yeast in 81% of cases. It allows early identification of yeast species and thus appropriate antifungal treatment. This will improve patient’s healthcare in the context of candidemia. Mycological culture remains necessary when our protocol doesn’t work and in case of mixed infections.

### P130. rThe Use of Coffee Agar As An Alternative Medium For The Presumptive Identification of Cryptococcus neoformans In Resource – Limited Settings.

ChidebeluP.NwezeE.Microbiology, University of Nigeria, Nsukka., Enugu, Nigeria

**Objectives:** Cryptococcus neoformans is presumptively identified by its characteristic brown - black colonies on media containing caffeic acid (Katiyar et al., 2011). In low income regions, neither the conventional identification media nor their basic component(s) is readily affordable and available. We proposed an alternative coffee agar media made of coffee which is rich in caffeic acid, for differential recovery of *C.neoformans* colonies from mixed cultures of various sample sources.

**Methods:** The coffee agar media which is a modification of caffeic acid agar was composed with coffee as follows: ammonium sulphate (5.0g), glucose (5.0g), yeast extract (2.0g), dipotasium phosphate (0.8g), magnesium sulphate (0.7g), coffee (18.5g), chloramphenicol (0.05g), ferric citrate solution (4.0ml), agar (15.0g). The final pH of the solution in 1 litre of de-ionized water was adjusted to 6.5 ± 0.3. After sterilization at 121 ºC and 15psi pressure for 15 minutes, the solution was cooled at 45ºC, dispensed in Petri dishes and stored at refrigeration temperature until use. Test isolates (*n* = 12) of Cryptococcus neoformans VNI (ST32) identified by MALDITOF MS and MLST, were streaked on the Coffee agar plates and monitored for pigmentation. Reference strains of Cryptococcus neoformans var. grubii VNI H99 and Candida albicans SC5314 were cultured parallel as the control. A set of the plates was incubated at 30ºC, and the other at 37 ºC for up to 1 week.

**Results:** The colonies of Cryptococcus neoformans (both the test and reference strains) turned brown - black on the coffee agar plates, while Candida albicans SC5314 remained as cream – white colonies. Melanization was more conspicuous at 30ºC after 5 days of incubation.

**Conclusion:** The brown - black pigmentation observed for C. neoformans indicated that coffee is rich in caffeic acid and thus, provided the substrate for the phenoloxidase enzyme laccase, which is absent in C. albicans. Expression of melanin especially at 30ºC remains integral for Cryptococcus’ environmental survival. After the necessary validation, we suggest that coffee agar can provide an alternative detection media especially in resource – limited settings where absence and cost of conventional identification media pose a barrier to research and diagnostic studies of Cryptococcus neoformans and Candida albicans.

### P131. Prospective comparison of colometric and turbidimetric assays for diagnosing invasive fungal infection in a routine laboratory

GuigueN.^1^Gits-MuselliM.^1^,^2^HamaneS.^1^AlanioA.^3^BretagneS.^3^1Parasitology-mycology Laboratory, Lariboisière Saint-Louis Fernand Widal Hospital, Assistance Publique-Hôpitaux de Paris (AP-HP), PARIS, France2Molecular Mycology, Institut Pasteur, PARIS, France3Paris-diderot, Sorbonne Paris Cité University, Institut Pasteur, Molecular Mycology Unit, Cnrs Cmr2000, Parasitology-Mycology Laboratory, Lariboisière Saint-Louis Fernand Widal Hospitals, Assistance Publique-Hôpitaux de Paris, Paris, France

**Objectives:** The detection of serum (1→3)-β-D-glucan (BDG), an antigen common to several fungi, is part of the definition of invasive fungal infections (IFI). The turbidimetric or the colorimetric detection is the basis of two different commercial kits, the Fungitell assay (FA) and the Wako assay (WA), respectively. The FA has been used in Europe since the 2000’s, whereas the WA has been only recently CE approved. We wondered how the WA compares to the FA on a routine use.

**Methods:** We prospectively tested 321 serum samples (median: 1[1-11]) from 171 patients (sex ratio M/F = 60%, mean age 48+/-16 yrs) mainly with hematological conditions (64%; 110/171) using both assays from December 21^st^ 2018 to April 23^rd^ 2019. The tests were performed according to the manufacturers’ recommendations.

**Results:** Twenty-three patients had IFI (pneumocystosis *n* = 12; invasive aspergillosis *n* = 4; candidemia *n* = 3; invasive fusariosis *n* = 2; Hepato-splenic candidiasis *n* = 1; and cryptococcosis *n* = 1). Both assays provided similar AUC around 0.9 (Figure 1). 

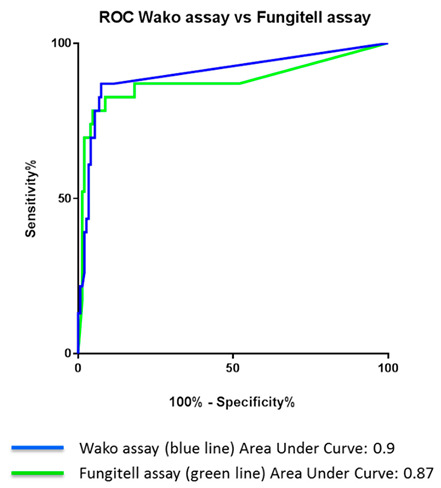



Sensitivity, specificity, positive and negative predictive values were as follow: FA (positivity threshold ≥80 pg/ml): 91%, 89%, 55%, 98%; WA (positivity threshold at 11 pg/ml): 48%, 97%, 67%, 92%; WA (positivity threshold at 4 pg/ml): 83%, 94%, 68%, 97%. When removing the FA negative (<80 pg/ml) and FA overflow values (> 523 pg/ml), the analytical comparison showed a good correlation (R^2^ = 0.81), the FA values being 28 higher in mean than the WA values (Figure 2). Among 73 samples from patients with bacterial sepsis, 21% (15/73) were FA positive, 11% (8/73) were WA positive (positivity threshold at 4 pg/ml), whose 7 positive with both methods. These possible false positive results should be taken with caution knowing that extensive work-up for diagnosing fungal infection was not homogenous between the patients due to the study design and that bacterial and fungal diseases are often concomitant. 

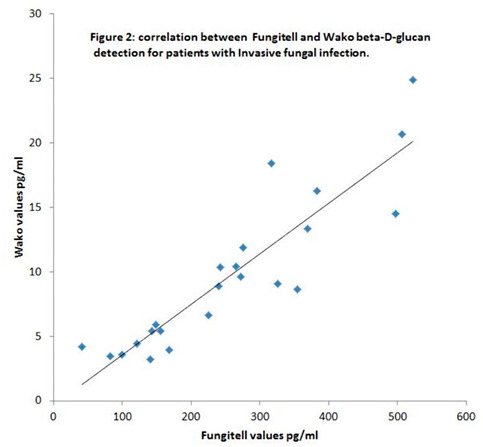



**Conclusion:** The WA performed similarly compared to the Fungitell assay when a lower cutoff (4 instead of 11 pg/ml) is used for the diagnosis of IFI, with a better specificity. The WA is a single sample testing which is clinically relevant when prompt therapeutic decision is on stake.

### P132. The development of a real-time PCR assay for the detection of Mucormycosis infections: Fungiplex Mucorales

GreenJ.DempseyK.Bruker Microbiology, Glasgow, United Kingdom

**Objectives:** Mucorales have been increasingly reported as causes of invasive fungal infections in immunocompromised subjects, particularly in patients with haematological malignancies, uncontrolled diabetes mellitus or those undergoing dialysis. Mucorales are now also being reported in *Aspergillus*-positive patients who are not responding to first line treatments. Histology and culture are still the most important diagnostic approaches for mucormycosis because of the lack of molecular diagnostic methods available, and β-d-Glucan detection is not useful due to the extremely low content of the biomarker in the Mucorales order. Timely diagnosis of invasive mucormycosis is essential due to the rapid progression of the disease, and because signs and symptoms of the infection are consistent with other invasive fungal infections. PCR would improve detection of Mucorales and complement the current Bruker offering within the area of invasive fungal disease.

**Methods:** The Bruker real-time PCR assays are designed in an easy to use format with minimum hands on time and results generated in around 1 hour after extraction. Universal primer and probe sequences have been designed to target the internal transcribed spacer (ITS) region of the rRNA gene for the genera detailed in Table 1. The probes associated with each pathogen have been labelled with different fluorescent dyes enabling some differentiation within the multiplex reaction. A specific control material has been developed to give the users an option of running it as an extraction control or a PCR inhibition control. 

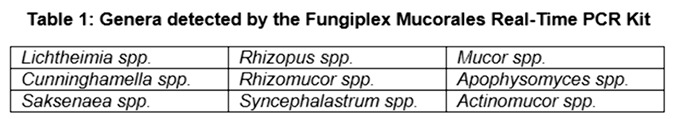



**Results:** The coverage specified in Table 1 has been confirmed using a range of simulated samples prepared from plasmid and genomic DNA. The limit of detection for species representing the highest prevalence of IFD for each genus has been assessed over six thermocycler platforms to ensure accuracy across a variety of systems.

**Conclusion:** Bruker have developed a Mucorales assay which identifies a wide range of clinically relevant genera. The assay is being further developed to produce a kit to provide rapid detection of the main causative agents in invasive mucormycosis.

### P133. The development of a real-time PCR assay for the diagnosis of Pneumocystis pneumonia: Fungiplex Pneumocystis

GreenJ.DonnellyT.Bruker Microbiology, Glasgow, United Kingdom

**Objectives:** *Pneumocystis jirovecii is* the causative organism in *Pneumocystis* pneumonia (PCP); a life-threatening pneumonia in immunocompromised patients. Due to a lack of methods to propagate *P. jirovecii in vitro,* the most common current practice in diagnosis of PCP is the microscopic identification of *P. jirovecii* through staining of bronchoalveolar lavage (BAL) samples. This method lacks sensitivity to detect low fungal burdens, therefore PCR methods of detection would improve diagnosis of PCP and complement the current Bruker offering within the area of invasive fungal disease. Two different Pneumocystis assays have been designed for initial evaluation against various sample types, with the intention of selecting the best performing assay for further development.

**Methods:** Two *Pneumocystis* real-time PCR assays were initially developed, with specific primer and probe sequences designed to target different genes of *Pneumocystis jirovecii*. Assay 1 targeted the internal transcribed spacer 2 (ITS) sequence between the 5.8S and 28S rRNA genes, with Cy5 as the fluorophore. Assay 2 targeted the mitochondrial large subunit (mtLSU) rRNA gene and used Yakima Yellow as fluorophore. During initial feasibility studies a range of plasmid concentrations were amplified in the presence of an amplification and extraction control using the standardised Fungiplex assay real-time PCR conditions. The real-time PCR assays were also tested against QCMD’s *P. jirovecii* pneumonia EQA panels.

**Results:** When assessed against a range of known plasmid concentrations, both assays were found to perform equivalently, with a limit of detection of 20 ipc and a dynamic range of 20 – 2x10^6^ ipc as displayed in Figure 1. When tested against the QCMD 2017 PCP panel, the correct result was obtained in duplicate testing of all extracts using Assay 2, whereas several replicates were undetected using Assay 1. The internal control was detected in all samples, showing that there was no inhibition. Assay 2 was then assessed against the QCMD 2018 PCP panel; again, the correct result was obtained in duplicate testing of all extracts using this assay. 

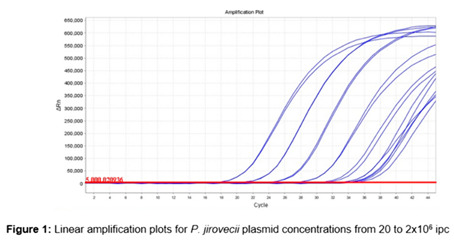



**Conclusion:** The assay designed to target the mtLSU gene, is therefore most suitable and will be developed further. The better performance of this assay is likely due to the presence of multiple copies of the mtLSU gene per organism, compared with just a single copy of ITS2.

### P134. Upgrading the level of diagnosis towards a more targeted treatment. A six year period of laboratory monitoring in a Paediatric Haematoloy-Oncology Unit.

ZarkadaE.^1^TragiannidisA.^2^PapageorgiouT.^2^HatzipantelisE.^2^SamarasK.^1^MarkantonatouA.-M.^1^ZachrouE.^1^VyzantiadisT.-A.^1^1First Department of Microbiology, Medical School, Aristotle University of Thessaloniki, Thessaloniki, Greece2Haematology-oncology Unit, Second Department of Paediatrics, Medical School, Aristotle University of Thessaloniki, Thessaloniki, Greece

**Objectives:** Invasive fungal infections have been proved an important cause of negative prognosis in critically ill patients and notably the immunocompromised, while the paediatric haematolgy-oncology patients form a high risk group among them. Although several laboratory methodologies for the diagnosis of fungal infections are not completely validated in these patients, the criteria provided by the EORTC/MSG, alongside to the recommendations by ESCMID and ECIL, can be followed in order to upgrade the level of diagnosis. The main aim of this study was to show that the implementation of laboratory testing in this group of patients, served as a useful tool towards a more targeted antifungal approach by managing to upgrade the diagnosis from possible to probable or maintain the clinical suspicion in just a state of readiness.

**Methods:** Data was gathered retrospectively, over a period of six years, from the records of the forty-three patients with haematological malignancies that were hospitalised in the Paediatric-Oncology Unit and had at least one mycology result during their follow-up. All mycology studies were performed in the First Department of Microbiology and included specific cultures, testing for galactomannan, mannan and antibodies against *Candida*, as well as PCR for *Aspergillus* or *Candida*. Also, sensitivity testing or TMD for antifungal drugs, when necessary.

**Results:** Among the above 43 haematology patients the 22 were initially considered to have a possible diagnosis according to the EORTC/MSG criteria due to the presence of at least one host factor and a clinical criterion (mainly positive CT imaging). From the 22 patients, nine proved to have a probable infection by *Aspergillus* with positive galactomannan and (in one case) / or (in one case) a positive PCR for *Aspergillus*. One patient had a proven diagnosis, based on the isolation of *Rhizopus arrhizus* from several cultures of face biopsies and one was considered with a “probable” diagnosis due to the presence of positive high titers of mannan, positive *Candida* PCR in blood and kidney ultrasound imaging of a possible mycotic mass. There were also three “probable” patients with sequential positive mannan testing (with decreasing to negative titers after treatment) that although not included in the criteria, they could not be considered as just possible. Consequently, the diagnosis was considered upgraded, after the mycology findings, in almost 64% (14/22) of patients with possible diagnosis. Among the 22 patients with possible diagnosis, the rest eight, as well as all other 21 patients (from the total of 43), remained with a possible or completely negative diagnosis (respectively) for fungal infection proving the utility of negative and often excluding results.

**Conclusion:** The results of this study could argue in favor of a meticulous and well organized protocol of laboratory monitoring in this group of patients. Taking in consideration that the here-mentioned monitoring, due to several reasons, is not performed with the proposed frequency and combination, the laboratory results are already a useful adjunct towards the documentation or even the exclusion of a fungal infection, helping the clinical decision and guiding the targeted treatment.

### P135. Revision of special microscopic techniques for visualization of aspergilli structural elements

VasilyevaN.^1^RiabininI.^1^MihaylovaY.^2^BorzovaY.^1^AlievaL.^1^1Kaschkin Research Institute of Medical Mycology. Department of Medical Microbiology, North-Western State Medical University named after I.I. Mechnikov, Saint-Petersburg, Russian Federation2Laboratory of Biosystematics and Cytology, Komarov Research Institute of Botany, Saint-Petersburg, Russian Federation

**Objectives:** Objective of the study is to select the optimal type of special light optics for obtaining microphotographs of *Aspergillus* spp.

**Methods:** Aspergillus clinical strains belonging to 14 species from the Russian Collection of Pathogenic Fungi (Saint-Petersburg, Russia) were used: *A. fumigatus, A. flavus, A. tamarii, A. terreus, A. niger, A. nidulans, A. ustus, A. calidoustus, A. sydowii, A. versicolor, A ochraceus, A. candidus, A. amstelodami, Neosartorya hiratsukae*. Original strains were previously identified by the target DNA-sequencing of ITS and β-tubulin loci according to the CLSI MM18 protocol (2nd edition). Subcultures were obtained on Czapek yeast extract agar (28°C, 10 days). Micropreparations were studied by three microscopic methods: (1) phase contrast (**PCM**), (2) «dark field» (**DFM**), (3) water-immersion light microscopy with oblique illumination (**WILMOI**) using an «aplanatic» condenser (Leningrad Optical-Mechanical Association, Russia). Presto T55 (Rekam, Canada) and PowerShot A2000IS (Canon, USA) cameras were used for taking photo. Digital image processing was performed in XnView 2.35 and GIMP 2.8.6 software.

**Results:** Series of microphoto for three described techniques were received. As it turned out, the phase-contrast microscopy sharply emphasizes the contours of the aspergilli thallic elements and their conidia, but the most successful microscopic pictures are obtained only in areas of location of the structural elements in single layer. With dark-field microscopy in some species (*A. fumigatus, A. nidulans, Aspergillus spp*. of Versicolores and Usti sections) structural details of the conidial heads are not clearly visible at high magnification. In dark-field microscopy, in comparison with elements of mycelium, vegetative spores and ascospores are better visualized. Dark-field and phase-contrast microscopy to varying degrees distort the natural color of the microscopic elements of the *Aspergillus* spp. cultures. In contrast to the techniques mentioned above, the use of a condenser with a shiftable iris diaphragm and a water-immersion diaphragmatic lens made it possible to take microphoto while preserving the natural pigmentation of aspergilli, sufficient contrast and creating a «relief» of a microscopic picture. In addition, by changing the setting of the aplanatic condenser, it is easy to transform the microscope in dark-field microscopy mode. Examples of taken images are shown in the figure. 

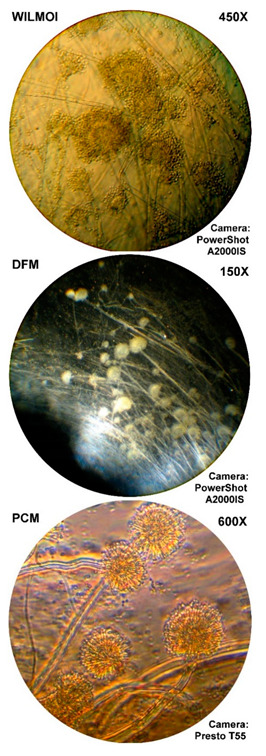



**Figure**. *Aspergillus flavus* micrographs made by using three special microscopic techniques (abbreviations are placed in text).

**Conclusion:** Water-immersion microscopy with oblique illumination allows to obtain the most natural microphoto of aspergillosis causative agents with the effect of «soft» contrasting. Moreover, the WILMOI mode is much faster adjusted by a microscopist than the DFM and PCM modes. We believe high-quality reproduction of the WILMOI technique is fundamentally available on microscopes of various manufacturers. With the help of phase-contrast and dark-field microscopy, it is possible to make quite spectacular images, but for complex representation of the strains they must be supplemented with the results of traditional light microscopy or WILMOI technique.

### P136. Evaluation of 99mTc-Amphotericin B for nuclear imaging of mould infections in a transwell in vitro system

PageL.^1^WursterS.^2^SchadtF.^3^EinseleH.^4^UllmannA.J.^1^SamnickS.^3^1Internal Medicine Ii, Division of Infectious Diseases, University Hospital of Wuerzburg, Wuerzburg, Germany2Ut Md Anderson Cancer Center, 1515 Holcombe, Houston, United States of America3Nuclear Medicine, University Hospital of Wuerzburg, Wuerzburg, Germany4Internal Medicine Ii, University Hospital of Wuerzburg, Wuerzburg, Germany

**Objectives:** Several studies have evaluated molecular imaging using positron emission tomography (PET) and single photon emission computed tomography (SPECT) to diagnose invasive mycoses. The most promising tracers described in the literature include ^68^Ga-labelled *Aspergillus*-derived siderophores [1] or *A. fumigatus*-specific antibodies labelled with ^64^Cu [2]. However, these PET-tracers are cost-intensive and onsite generation may not be feasible at regional medical centers. Therefore, we investigated the potential of Amphotericin B (AMB) labelled by the gamma emitter technetium-99m (^99m^Tc) to visualize pathogenic moulds.

**Methods:** Sodium pertechnetate (derived from a ^99^Mo/^99m^Tc generator, 1000 MBq in 0.35 mL saline) was added to a suspension of 50 µg AMB in 100 µL HBSS, followed by 20 µL of a stannous chloride solution (10 mg SnCl_2_ in 1 mL 0.1 N HCl). After 20 min incubation at room temperature, the ^99m^Tc-AMB solution was neutralized by addition of 3 ml PBS and sterile filtered. To prepare the transwell assay, 1x10^5^ human pulmonary artery endothelial cells (HPAEC) were seeded on the lower side of a transwell insert (24-well format, 3 µm pore size) according to previously published protocols [3-4]. The HPAEC layer was infected with 2.5x10^1^-2.5x10^6^*A. fumigatus* conidia or a bacterial control (*Staphylococcus aureus*). Additional inserts were infected with 2.5x10^5^ spores of different Mucorales species. After an incubation period of 15 h to obtain early mycelial biofilms, ^99m^Tc-AMB was added to the medium at 50 ng/mL, a concentration that is significantly below commonly achieved therapeutic serum levels [5]. Transwell inserts were incubated for 120 min in 600 µL of the tracer solution (initial activity: 30 kBq per well) and were subsequently washed five times with HBSS. Thereafter, the activity was measured using a gamma counter and normalized to the activity of 600 µL tracer-supplemented medium to calculate percent uptakes. Independently prepared inserts were imaged by autoradiography.

**Results:** Radiochemical yield and purity of ^99m^Tc-AMB, as determined by thin layer chromatography, reached >98 % and >99 %, respectively. Minimal unspecific tracer uptake was found in uninfected (0.03-0.14 %) or *S. aureus* infected inserts (0.05-0.16 %). An inoculum-dependent increase in tracer uptake was seen in *A. fumigatus* infected inserts, with a limit of detection at 2.5x10^4^ fungal cells (Panel A). Low inter-assay variability was found (mean coefficient of variation = 0.32, *n* = 3). Quantitative results were validated by autoradiography imaging, confirming inoculum-dependent tracer enrichment in *A. fumigatus*-infected HPAEC monolayers (Panel B). Finally, we tested the applicability of ^99m^Tc-AMB to detect Mucorales. Mean 4.4 and 6.2-fold ^99m^Tc-AMB enrichment compared with an uninfected control was found in inserts infected with 2.5x10^5^ spores of *Rhizopus arrhizus* or *Cunninghamella bertholletiae*, respectively.

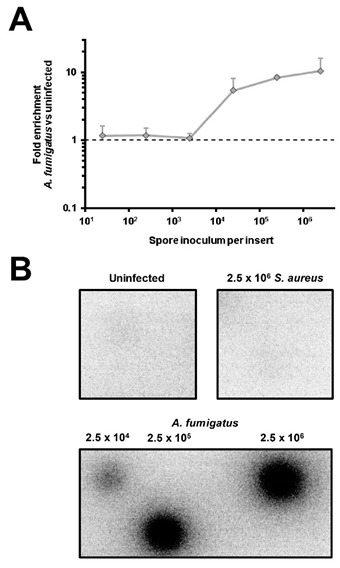



**Conclusion:** Our *in vitro* results suggest promising potential of ^99m^Tc-AMB as an easily producible, cost-efficient alternative for nuclear imaging of pathogenic moulds by SPECT. These data could encourage further *in vivo* evaluation of modified antifungal drugs as nuclear medical tracers. [1] Petrik et al., 2014, Mol Imaging Biol; [2] Rolle et al., 2016, PNAS; [3] Hope et al., 2007, J Infect Dis; [4] Belic et al., 2019, Front Microbiol; [5] Hamill, 2013, Drugs.

### P137. Detection of circulating DNA in serum by PCR for the diagnosis of invasive mucormycosis

SavioJ.MathewJ.Microbiology, St. John’s Medical College, Bangalore, India

**Objectives:** Mucormycosis is an invasive fungal infection with increasing incidence in developing countries like India. Because of its rapid and fatal progression, early diagnosis is essential. Conventional methods like microbiological and histopathological investigations have limitations. In the absence of specific biomarkers for diagnosis, detection of circulating *Mucorales* DNA in serum by PCR proves a better alternative to conventional methods. The objectives of this study were to compare detection of circulating *Mucorales* DNA in serum by PCR with the conventional microbiological and histopathological investigations.

**Methods:** The Institutional Ethics Committee approved this study. 36 patients with a clinical diagnosis of mucormycosis whose samples were received in the microbiology and or histopathology laboratories were included. Three patients with laboratory finding not suggestive of mucromycosis were excluded. Blood samples from all 33 patients were collected. Serum was separated and stored until further analysis. Microbiological investigations included microscopy and culture. Isolates were identified by the conventional techniques. Histopathological examination by microscopy was carried out using H and E, PAS and GMS stains. DNA was extracted from serum by the salting out and TRIZOL methods. Conventional Pan-*Mucorales* PCR was performed on the extracted DNA.

**Results:** of the 33 patients, 20 were males and 13 were females. Mean age was 52 years. 29 of the 33 patients had diabetes mellitus and 3 were diagnosed following admission. Clinical presentation included rhino orbito cerebral in 25, cutaneous in 4, pulmonary in 3 and renal in one. All 32 samples received for microbiological investigations were positive by microscopy and 22 (68.8%) were culture positive. Isolates included *Rhizopus* species 17, *Apophysomyces* 2, *Lichtheimia* 1 and one is yet to be identified. 24 of the 25 samples on histopathological examination was suggestive of mucormycosis. 19 of the 33 serum samples were positive for circulating DNA. PCR was positive in 80% of the culture negative patients. PCR was positive in 42.1% of samples with no angioinvasion. PCR was positive in 50% of ROC and cutaneous manifestation and all 3 patients with pulmonary mucormycosis. 61.9% of PCR positive patients had an unfavorable outcome. 

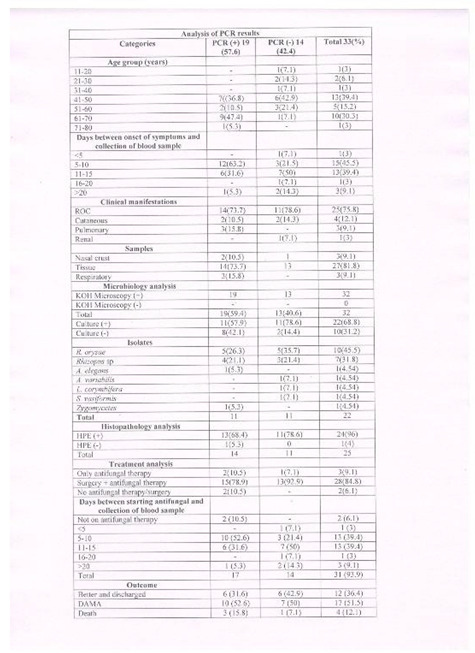

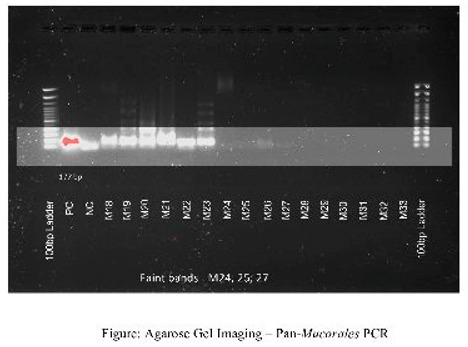





**Conclusion:** Most patients were above 40 years of age and from urban and rural areas DM was the most common underlying risk factor. ROC was the most common presentation. *Rhizopus* species was the most common isolate, *Apophysomyces* and *Saksenaea* were isolated from cutaneous lesions. 57.6% of the samples were positive for circulating *Mucorales* DNA PCR was positive in 80% of culture negative samples PCR was positive in 42.1% of samples with no angioinvasion Detection of circulating *Mucorales* DNA could serve as an indicator of angioinvasion and help in early diagnosis 36.4% of the patients recovered Other factors that could influence the PCR results include: use of different volumes of serum for DNA extraction and commercial extraction kits A larger sample size and serial sampling during the course of illness is required to critically evaluate the utility of this test

### P138. Rapid Diagnosis of Invasive Aspergillosis in Bronchoalveolar Lavage: Performance of the Aspergillus Galactomannan Lateral Flow Assay and the Aspergillus-specific Lateral Flow Test in a Mixed Patient cohort

JenksJ.ReedS.MehtaS.LawN.AslamS.TaplitzR.HoeniglM.Medicine, University of California San Diego, San Diego, United States of America

**Objectives:** Early diagnosis and treatment of invasive pulmonary aspergillosis (IPA) remains the most important factor to reduce mortality and improve outcome. Diagnosis remains a challenge, however, due to unspecific clinical presentation and radiological findings. Only very recently rapid tests for IPA have been developed. The objective of this study was to evaluate the performance of two new CE-marked tests, the Aspergillus Galactomannan Lateral Flow Assay (LFA; IMMY, Norman, Oklahoma, USA) and the Aspergillus-specific Lateral Flow Device Test (LFD; OLM, Newcastle, UK) for IPA in a mixed cohort of patients with and without hematological malignancies.

**Methods:** The LFA and LFD were retrospectively performed according to the manufacturer’s instructions in 106 previously frozen bronchoalveolar lavage fluid samples from 106 patients at risk for IPA (23% with underlying hematological malignancies). Samples were collected between September 2016 and September 2018 at the University of California San Diego, United States. Performance of the LFA and the LFD were compared to Galactomannan, and BAL culture. IPA was classified according to revised EORTC/MSG criteria.

**Results:** Overall, 22 patients met criteria of probable or proven IPA, 9 criteria of possible IPA, while 75 patients did not fulfill EORTC/MSG criteria of IPA. Sensitivity of the Apergilllus Galactomanann LFA for probable/proven IPA was 77% (17/22), and thereby slightly higher than sensitivity for the Aspergillus-specific LFD (59%; 13/22). Sensitivity was similar to BAL GM (77% with a cutoff of 1.0 ODI), but higher compared to BAL culture (23%). The LFA resulted negative in 7/9 cases with possible IPA and 47/73 cases without IPA (overall specificity 66%, 54/82). The less than perfect specificity was driven particularly by non-neutropenic patients (specificity 62%, 43/69), while specificity was 85% among patients with underlying hematological malignancies. Specificity was slightly higher for the LFD (70% specificity; 67% among non-neutropenic patients). Lower specificity among non-neutropenic patients was also observed for the BAL GM (overall specificity 77%, 72% among non-neutropenic patients), and BAL culture (overall 90%, 88% among non-neutropenic patients).

**Conclusion:** Our study indicates that both the LFA and LFD may be useful for rapid diagnosis of IPA in BALF when IPA is clinically suspected. The lower specificity in non-neutropenic patients without underlying hematological malignancies may be explained by limited applicability of the EORTC/MSG criteria in non-neutropenic patients. Better consensus criteria for classifying IPA in those patients are needed.

### P139. Use of the basophil surface marker CD203c in the diagnosis of fungal sensitization in patients with cystic fibrosis

KozlovaY.^1^FrolovaE.^2^UchevatkinaA.^2^FilippovaL.^2^BorzovaY.^2^MakhmutovaV.^3^StepanenkoT.^3^AakO.^2^BogomolovaT.^2^SpiridonovaV.^2^VasilyevaN.^4^KlimkoN.^5^1Department of Clinical Mycology, Allergology and Immunology, North-Western State Medical University named after I.I. Mechnikov, Saint-Petersburg, Russian Federation2Kashkin Research Institute of Medical Mycology, Saint-Petersburg, Russian Federation3Multidisciplinary City Hospital №2, St. Petersburg, Russia, Saint-Petersburg, Russian Federation4Kaschkin Research Institute of Medical Mycology. Department of Medical Microbiology, North-Western State Medical University named after I.I. Mechnikov, Saint-Petersburg, Russian Federation5Department of Clinical Mycology, Allergology and Immunology, North-Western State Medical University n.a. I.I. Mechnikov, Saint-Petersburg, Russian Federation

**Objectives:** Allergic bronchopulmonary aspergillosis (ABPA) is the most common form of aspergillosis in patients with cystic fibrosis (CF). ABPA affects from 2% to 15% of patients with CF and significantly worsens the course of the underlying disease. Diagnosis of ABPA with CF is complicated because many diagnostic criteria intersect with typical manifestations of the underlying disease. Recent studies show that the sensitization to *A.fumigatus* associated with a significant reduction in lung function and an increased incidence of pulmonary exacerbations in CF patients. Thus, early detection of fungal sensitization very important for the diagnosis of ABPA, and appointment of adequate therapy which prevents the progression of lung disease. Now there is a search for new diagnostic tests that will identify fungal sensitization in these patients.To study possibility of basophil activation test use for identification of fungal sensitization in patients with cystic fibrosis.

**Methods:** The study included 190 patients with CF aged 1 to 40 years. All patients underwent allergological (skin tests with fungal allergens, determination of serum levels of total IgE (“Polygnostus”, Russia) and specific IgE (sIgE) for fungal allergens(“AlcorBio”, Russia)) and mycological (microscopy and culture of respiratory substrates) examination. Computed tomography of the chest was performed according to the indications. Diagnostic criteria of ABPA 2003 [Stevens et al] were used. Study of the activation of basophils by flow cytometry was performed using Allergenicity kit (“Beckman Coulter”, USA). Basophils were identified by CD3^-^CRTH2^+^ marker, basophil activation – by elevated expression of CD203c. For basophil stimulation were used allergens *A.fumigatus* (“AlcorBio”, Russia). The basophil activation test with the *A. fumigatus* allergen was conducted in 12 CF patients with ABPA and 25 CF patients without ABPA in addition to the standard allergological examination. The obtained data was processed using the STATISTICA 10 software system.

**Results:** In patients with cystic fibrosis the frequency of sensitization to *A. fumigatus* was 27%, the incidence of allergic bronchopulmonary aspergillosis - 5.7%. The number of eosinophils, total IgE and specific IgE levels in CF patients with ABPA were significantly higher than in CF patients without ABPA. In blood of were identified 77.1 (46.3-87.8) % basophils activated by *A.fumigatus* allergen, the stimulation index was 19.2 (11.3-30.6). In the comparison group stimulation index did not exceed the value of 2.5 (p = 0.000). Direct positive correlation between the level of sIgE to *A. fumigatus* and the number of basophils activated by *A. fumigatus* allergens (r = 0,67; p < 0,05) was established. In CF patients with ABPA the FVC and body mass index were significantly lower comparing with patients without fungal sensitization.

**Conclusion:** The basophil activation test applying, along with traditional methods, will allow a more differentiated approach to assessing the development of ABPA in CF patients. Timely identification of the associated with *A. fumigatus* clinical status of CF patients will facilitate early and effective assignment of specific therapy.

### P140. rComparison of In-house Real-time Assay and MucorGenius Assay performance for testing of Clinical Samples from Immunocompromised Patients

LengerovaM.^1^VolfovaP.^1^LangerJ.^1^PantuckovaD.^1^PospisilovaJ.^1^BezdicekM.^1^KocmanovaI.^2^MayerJ.^1^RacilZ.^1^1Department of Internal Medicine - Hematology and Oncology, University Hospital Brno, Brno, Czech Republic2Department of Clinical Microbiology, University Hospital Brno, Brno, Czech Republic

**Objectives:** Diagnostics of infections caused by mucormycetes is still quite complicated because no serological tests (unlike in aspergillosis) are available. Therefore, PCR methods are currently the methods of choice. The aim of this study was to compare MucorGenius assay results (Pathonostics) on samples that previously tested positively using our in-house PCRs.

**Methods:** In our laboratory, we use nested real-time PCR assay followed by high-resolution melting analysis (RT-HRM) for PCR screening of clinical samples from patients at risk of invasive mucormycosis. This assay enables not only detection but also identification of detected species. If the result is positive, we confirm the result and determine the fungal load with species-specific real-time quantitative PCR (RQ-PCR). In this study, we tested 29 selected clinical samples (10 tissues, 15 bronchoalveolar lavage (BAL)samples, 3 cerebrospinal fluid samples and 1 endosecret), 25 that had been tested as positive using our screening assay and 4 mucormycetes negative samples. The results were compared to tests with the MucorGenius assay.

**Results:** Out of 25 RT-HRM positive samples, 21 were positive using species-specific RQ-PCR. A significant fungal load (Ct £ 35) was detected in 10 samples. Using MucorGenius assay, 17 out of 25 samples (68%) were positive (Ct 18.1 – 37.3). RT-HRM detected *Rhizopus* sp. (17 samples), *Mucor* sp. (3 samples), *Lichtheimia corymbifera* (2 samples), *Lichtheimia ramosa* (2 samples) and *Rhizomucor* sp. (1 sample). 12 out of 17 samples with *Rhizopus* sp. detected revealed low fungal load or were negative. To analyze this discrepancy, sequencing was performed and in 10 samples and *R. stolonifer* was identified (which is not targeted by in-house RQ-PCR). The MucorGenius assay was positive in 7/10 *R. stolonifer* positive samples (Ct 31.0-37.3).

**Conclusion:** Using commercial assays for PCR diagnostics is currently preferred because of standardization; therefore we compared our in-house assay results with a commercial kit for mucormycetes detection. In this study, we also analyzed samples with discrepant results and identified *R. stolonifer* as a frequent finding when testing BAL samples. As this species is rarely described as a cause of infection, its detection most probably represents colonization or contamination. Based on these results, we added *R. stolonifer* specific RQ-PCR to our diagnostic algorithm (if RT-HRM detects *Rhizopus* sp. than *R. microsporus*, *R. oryzae* and *R. stolonifer* RQ-PCRs are used to verify the results). Due to our experience, the MucorGenius kit is easy to use, as it is a one tube real-time PCR assay and has very good sensitivity. The main disadvantage is the inability to identify mucormycetes species. Although this information is not critical to initiating treatment, it is essential for epidemiological reasons and might help to distinguish colonization/contamination from a truly ongoing infection. This study was supported by the Ministry of Health, Czech Republic - conceptual development of research organization (FNBr, 65269705) and TE02000058.

### P141. Pythium insidiosum - sporulation with leaves of grass: Two case reports

MathewJ.SavioJ.MathewJ.ReddyM.S.Microbiology, St. John’s Medical College, Bangalore, India

**Case Report:** *Pythium insidiosum* is a filamentous, aquatic parasite which was till recently considered to be a fungus. It is known to cause sight-threatening keratitis. The filaments of *Pythium* resemble pauci-septate hyphae of *Mucorales*. Case history: Both patients presented with redness, irritation and reduced vision of right eye since one month. There was no history of trauma or any co-morbid conditions. Patient 1 (27 years old gentleman): Ocular examination revealed corneal ulcer with feathery margins, stromal infiltrates and endothelial plaque. Hypopyon and corneal congestion were noted. Fungal keratitis was the clinical diagnosis. KOH microscopy of corneal scraping revealed broad pauci-septate fungal hyphae. The patient was started on oral fluconazole and topical voriconazole and amphotericin-B along with antibiotics. With deterioration in the clinical course, therapeutic penetrating keratoplasty was performed and the patient was discharged on topical Natamycin. Progressive loss of vision in the eye was managed with iris repositioning and tightening of corneal suturing. The patient developed a white corneal opacity and was later lost to follow-up. Patient 2 (26 years old lady): Ocular examination revealed a corneal epithelial defect, satellite lesions, corneal scarring with stromal infiltration and no hypopyon. The impression was healing fungal ulcer/viral keratitis. Initially, she was started on topical acyclovir. On her follow-up OPD visit she still complained of pain in the right eye. KOH microscopy of corneal scraping revealed broad, pauci-septate fungal hyphae. The patient was started on oral ketoconazole and topical voriconazole. Topical linezolid, doxycycline and azithromycin were also added. On follow-up visit, the patient was asymptomatic and ocular examination revealed healed lesions. No surgical management was undertaken. Both samples of corneal scraping on fungal culture yielded a non-sporulating mould. Colonies were flat, feathery, glabrous and light-brown coloured. Attempts were made to induce sporulation using the conventional techniques such as water agar, water culture and using non-nutrient media such as corn meal agar. With colony morphology resembling *Pythium*, literature search was made for specific sporulation techniques. The induction medium required chemicals such as K_2_H_2_PO_4_, MgCl_2_. Due to the non-availability of these in our laboratory, we set out to use the grass on our campus (the common Bermuda grass, *Cynodon dactylon*) with yeast extract and sterile distilled water with the hope to induce sporulation. To our surprise, the isolates produced sporangia and motile zoospores, which were visualized under light microscope. The isolates were sent to PGIMER, Chandigarh for confirmation by gene sequencing and the identification was confirmed as *Pythium insidiosum.* Video link-zoospore: https://drive.google.com/file/d/1cxHGWR-EwHgx0ucuTQZGy81nwfVagFEj/view Conclusion: *Pythium insidiosum* keratitis may present as chronic corneal ulcers. The simple Grass leaf culture as a technique for induction of sporulation of *Pythium insidiosum* can be performed in resource-limited settings. Experimentation can be attempted using leaves from different species of grass. Increased awareness and high index of suspicion on direct microscopy (pauci-septate, ribbon-like hyphae) and colony morphology (flat, feathery colonies) is integral to prevent misdiagnosis as fungal infection. Studies prove that prognosis of such infections is better when treated by a combination of antibiotics and antifungals than antifungals alone, which may occur when misdiagnosed as a fungal infection.

### P142. An Evaluation to assess the performance of the Fungiplex Aspergillus and the Fungiplex Aspergillus Azole-R IVD Real-Time PCR Kits following the implementation of an extraction control

GreenJ.Bruker Microbiology, Glasgow, United Kingdom

**Objectives:** This Performance Evaluation was designed to test contrived samples spiked into serum, plasma and BAL material to assess the impact of a new extraction control on the limit of detection of the Fungiplex Aspergillus and Fungiplex Aspergillus Azole-R IVD Real-Time PCR Kits and to ensure sensitivity was unaffected by this change. The results from this study have been evaluated to determine whether the inclusion of an extraction control has an impact on the limit of detection defined in previous studies.

**Methods:** Simulated samples were prepared and analysed by the clinical evaluators. Samples prepared in serum, plasma and BAL were spiked with specific concentrations of *Aspergillus fumigatus* or *Aspergillus terreus* genomic DNA prior to extraction. *Aspergillus fumigatus* wild type strains were used as well as resistant strains containing DNA mutations TR34 and TR46 in the *Cyp51A* gene. Samples were prepared in replicates of five and negative controls were included for each sample type. Prior to extraction 10 µL of the new extraction control formulation was added to each sample. Extraction was performed using two commercial extraction platforms; the eMAG^®^ by bioMérieux and the BioRobot^®^ EZ1™ by Qiagen. The PCR was prepared in duplicate for each sample and run on the Qiagen Rotor-Gene Q 5plex HRM platform.

**Results:** When tested with the Fungiplex Aspergillus IVD Real-Time PCR Kit, the limit of reproducibility for *Aspergillus fumigatus* and *Aspergillus terreus* samples was 5 ge (genome equivalents). No difference in sensitivity was observed when the results from two different extraction platforms were compared, however, on average the resultant Ct (cycle threshold) values were 1.2 cycles higher when the EZ1™ platform was used. When tested with the Fungiplex Aspergillus Azole-R IVD Real-Time PCR Kit, the limit of reproducibility for *Aspergillus fumigatus* samples containing mutations TR34 and TR46 was 50 ge. This higher limit of reproducibility was expected as the *Cyp51A* gene is single copy. The limit of reproducibility was consistent between all sample types, where in a previous study, without an extraction control, the limit of reproducibility had been reported between 50 and 75 ge depending on which sample type was used. The extraction control was detected in every sample analysed with no significant differences observed in Ct value reported.

**Conclusion:** 100% sensitivity was reported for the Fungiplex Aspergillus Kit when analysing *Aspergillus* samples at 5 ge and for the Fungiplex Aspergillus Azole-R Kit when analysing resistant strains at 50 ge. The sensitivity is not dependent on sample type and the new extraction control implemented in these products did not affect the performance of either Kit. Data has shown that results from this study are equivalent, and in some cases, superior to previous studies performed.

### P143. Evaluation of the clinical usefulness of dermatophyte EQA panels using molecular tests

GaajetaanG.KampermannT.TegelenD. VanDingemansG.PathoNostics, Maastricht, Netherlands

**Objectives:** Determine the relevance of external quality assessment (EQA) panels for detection of dermatophytes by real-time PCR. For this purpose, the commercially available DermaGenius® 2.0 Complete real-time multiplex PCR was used and simultaneously it’s performance was assessed.

**Methods:** Samples from the Dermatophytosis EQA pilot study (years 2016-2018) from QCMD (Quality Control for Molecular Diagnostics, Glasgow, UK) and the dermatophytes EQAS program (years 2017-2018) from INSTAND e.V. (Düsseldorf, Germany) were all tested with the DermaGenius® 2.0 Complete real-time multiplex PCR after suitable DNA-extraction. The samples from QCMD were processed with a NucliSENS® easyMag® DNA extraction system (bioMérieux) and the samples from the EQA from INSTAND e.V. were processed with the PathoNostics Extraction kit.

**Results:** The Dermatophytosis EQA pilot study panel from QCMD is provided once a year and contains 8 samples. All 8 samples in each year were correctly identified with the DermaGenius® 2.0 Complete real-time PCR. The composition of the EQA panel was comparable every year and contains 1 negative, three samples with different *T. rubrum* concentrations (Ct 26-35) and three samples with different *T. interdigitale* concentrations (Ct 27-38). Remarkably, each year the panel contained one sample which was positive for *C. albicans* (Ct 34-36), which is not a dermatophyte (and not in the scope of the panel) but can cause a nail infection. The dermatophytes EQAS panel from INSTAND e.V. is provided twice a year and contains 4 samples. Most samples were correctly identified with the DermaGenius® 2.0 PCR, only *Nannizzia gypsea* was not detected because this dermatopyte target is not included in the PCR assay. Other dermatophytes included in the four panels were *T. rubrum* (4x), *T. tonsurans* (2x), *T. interdigitale* (1x), *M. canis* (1x), *T. violaceum* (1x), *T. benhamiae* (1x) and *M. audouinii* (1x). Almost all dermatophytes were detected at very low Ct-values (Ct 19-26) representing a high DNA load. A negative control was included in each panel and was also identified as negative by the PCR.

**Conclusion:** Both dermatophyte EQA panels are intended for performance evaluation of molecular detection methods. The DermaGenius® 2.0 Complete real-time multiplex PCR showed good performance on both EQA panels, but the panels from these organisations have a different scope. The EQA panel from QCMD contains variable DNA concentrations of common dermatophytes and thereby focuses on sensitivity and clinical prevalence of dermatophyte species. The EQA panel from INSTAND e.V. panel contains a very high load of common but also more rare dermatophytes and thereby focussing more on specificity rather than sensitivity and prevalence/clinical significance. More importantly, the sample type is very difficult to mimic and therefore quality control samples do not really reflect the actual situation. Nevertheless, the EQA panel from INSTAND e.V. supplies a solid sample (lyophilized) and can therefore be processed in a more realistic way in comparison with the liquid samples from the EQA samples from QCMD. Overall, it is important to select a quality assessment program which serves your purpose.

### P144. Diagnosis of fungal peritonitis: optimization by evaluating two culture procedures

MartínezS.^1^VallinaE.^1^CostalesI.^1^MilianL.^2^LlamasE.^1^SandovalM.^1^FernándezJ.^1^ZapicoM.S.^1^PeláezT.^1^1Servicio De Microbiología Y Parasitología, HUCA, Oviedo, Spain2Servicio De Microbiología Y Parasitología, CAUSA, Salamanca, Spain

**Objectives:** Peritonitis is one of the most serious intra-abdominal infections. Among them, the most frequent is secondary peritonitis, usually caused by intestinal perforation and with polymicrobial etiology. Fungal involvement in peritonitis is especially relevant, as fungal peritonitis is associated to a high mortality rate. Regular culture-based diagnosis of fungal peritonitis is difficult, due to bacterial growth may mask the presence of yeasts with slower growth. The use of selective culture media for these microorganisms means an improvement of the diagnosis. The aim of this study is to evaluate the improvement of the microbiological diagnosis of fungal peritonitis, comparing two culture procedures.

**Methods:** Between 2016 and 2019, a total of 1671 peritoneal fluids were retrospectively studied. Two time periods were established to evaluate two culture procedures: Period 1 (January 2016 to May 2018) and Period 2 (June 2018 to April 2019). During Period 1, if sample volume was enough, peritoneal fluids were inoculated in blood-culture bottles (BD BACTEC Plus Aerobic/F culture vials) and incubated in an automatic incubator (BACTEC FX Instruments, Becton-Dickinson). Otherwise, they were inoculated in growth media agar plates (blood agar, chocolate agar, McConkey agar and Brucella agar). During Period 2, all peritoneal fluids were also inoculated at the same time in a fungus selective culture medium (Gentamycin + Chloramphenicol Sabouraud Agar, Biomèrieux).

**Results:** A total of 1671 peritoneal fluids were evaluated, 1254 during the first period and 417 during the second period. of them 147 (8.8%) were positive for fungal growth. The positivity rates of fungal growth during the first and the second period were, respectively: 6.7% vs 15.1%. The new culture protocol (Period 2) increased significantly the number of yeast isolates in peritoneal fluids (p = 0.0001), rising from 7% in the first period to 19.5% in the second (Table 1). Comparing data by yeast species, a significant rise in incidence of *C. albicans* (4.5% vs 10.5%) and *C. parapsilosis* (0.48% vs 3.4%) was observed (Table 2).

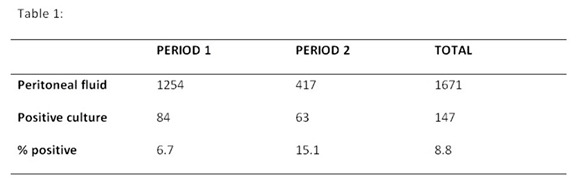

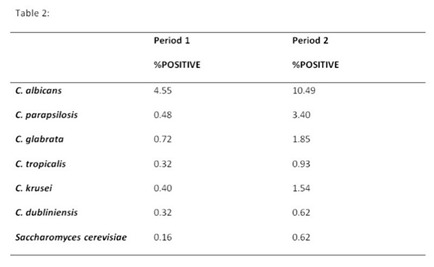





**Conclusion:** Our data show that the incorporation of selective culture medium for fungi improves significantly the microbiological diagnosis of fungal peritonitis, increasing the positivity rates and the recovery of fungal isolates. *C. albicans* was the most frequent species isolated, followed by *C. glabrata* and *C. parapsilosis.* The incidence of *C. albicans* and *C. parapsilosis* increased significantly during the second period of the study.

### P145. Direct detection of Candida auris and its ERG11-associated azole resistance and FKS1-associated echinocandin resistance in clinical skin swabs

KordalewskaM.LeeA.ZhaoY.PerlinD.S.Center For Discovery and Innovation, Hackensack Meridian Health, Nutley, United States of America

**Objectives:** *Candida auris* is a multidrug resistant yeast, recognized as a cause of invasive infections and health care associated outbreaks around the world. Major obstacles impacting control of *C. auris* spread include common misidentification by diagnostic platforms available in clinical and public health laboratories; a poor understanding of resistance to antifungal drugs and disinfectants; and a high propensity to contaminate health care environments which results in transmission of clonal *C. auris* isolates. Ultimately, correct detection and identification of the pathogen and its antifungal susceptibility, followed by strict adherence to appropriate treatment and infection prevention and control strategies is crucial for limiting the spread of *C. auris*. Here, we have developed and validated platforms for direct detection of *C. auris* and its *ERG11*-associated azole resistance and *FKS1*-associated echinocandin resistance in clinical skin swabs.

**Methods:** DNA isolated from 112 de-identified clinical axilla/groin skin swab samples was tested in the recently developed *C. auris*-specific SYBR Green qPCR assay (Kordalewska et al. JCM 2017) with the addition of inhibition control. Enrichment culture (Welsh et al. JCM 2017) followed by sequencing of rDNA was used as a reference method. 24 samples, determined as *C. auris*-positive by both qPCR and reference method, were further screened in the *ERG11*-associated azole resistance and *FKS*-associated echinocandin resistance assays (Hou et al. AAC 2018). Antifungal susceptibility testing with echinocandins (anidulafungin, micafungin) and azoles (fluconazole, voriconazole) according to CLSI guidelines and sequencing of *ERG11*, and *FKS1* were used as reference methods.

**Results:** In the *C. auris*-specific SYBR Green qPCR assay 45% of swabs were *C. auris*-negative (50/112), and 55% were *C. auris*-positive (62/112). However, only 21% (24/112) of swabs were culture-positive. The finding that *C. auris* cells lost their viability in 34% of swabs, but the DNA was still present (giving a positive real-time PCR result), was possibly due to the long swabs storage (4-5 months) before processing. Detection of *C. auris* was not affected by the presence of other microorganisms present in the sample. Thus, the assay was found to produce results comparable to the enrichment culture approach currently implemented at CDC and Antibiotic Resistance Laboratory Network (ARLN) laboratories. The resistance detection assays identified all *C. auris*-positive swabs as *FKS1* wild-type, and *ERG11* wild-type. The molecular diagnostic results from the assays were 100% concordant with DNA sequencing results. Moreover, all isolates exhibited low minimal inhibitory concentration (MIC) values (MIC ranges - anidulafungin: 0.06-0.125 mg/l; micafungin: 0.06-0.125 mg/l; fluconazole: 1-2 mg/l; voriconazole: ≤0.03 mg/l) and were categorized as echinocandin- and azole-susceptible.

**Conclusion:** Overall, we found the *C. auris*-specific SYBR Green qPCR assay can be successfully applied for rapid and accurate detection of *C. auris* in patient skin swabs, thereby increasing high-throughput diagnostic options for this emerging pathogen. Moreover, the *ERG11*-associated azole resistance and *FKS1*-associated echinocandin resistance detection platform has the potential to overcome the deficiencies of existing *in vitro* susceptibility-based assays to identify azole and/or echinocandin resistant *C. auris*, and thus, it holds promise as a surrogate diagnostic method to direct antifungal therapy more effectively. 

### P146. Role of PCR, mannan antigen and 1,3-beta-d-glucan measurements in cerebrospinal fluid for the diagnosis and follow-up of Candida meningitis

KampouriE.^1^ScherzV.^2^MarquisP.^3^PaganiJ.-L.^3^GreubG.^1^,^2^LamothF.^1^,^2^1Infectious Diseases Service, University Hospital of Lausanne, Lausanne, Switzerland2Microbiology Institute, University Hospital of Lausanne, Lausanne, Switzerland3Intensive Care Department, University Hospital of Lausanne, Lausanne, Switzerland

**Objectives:** Infections of the central nervous system due to *Candida spp.* are rare and usually associated with intracranial devices. Diagnosis is challenging because of low sensitivity of cultures and lack of specificity of clinical and biological signs of meningitis. Optimal treatment is not well established and response to therapy is difficult to assess. We present two cases of *Candida albicans* meningitis occurring in the setting of craniofacial surgery without intracranial device for which different fungal markers (1,3-beta-d-glucan, mannan, PCR) in the cerebrospinal fluid (CSF) were used for diagnosis and follow-up.

**Methods:** Platelia^TM^ Candida Ag plus (Bio-Rad), Fungitell^TM^ (Associates of Cape Cod) and a home-made quantitative *C. albicans*-specific PCR (targeting 26S rDNA) were used for detection of mannan antigen (Mn-Ag), 1,3-beta-d-glucan (BDG) and fungal DNA, respectively, in serum and cerebrospinal fluid (CSF).

**Results:** *Candida* meningitis presented as a complication of neurosurgery, following transsphenoidal surgery and skull base reconstruction with CSF leakage respectively. Biological analyses of CSF were consistent with bacterial meningitis, but with negative culture and broad-spectrum 16S rDNA PCR for bacteria. *C. albicans* was detected by CSF culture in one case and PCR in CSF in both cases. Antifungal therapy consisted in fluconazole and liposomal amphotericin B. PCR fungal loads subsequently declined along with clinical and biological improvement. BDG levels in CSF were very high at diagnosis while negative in serum and remained elevated during follow-up in both cases. Mn-Ag was measured in one case with elevated value in CSF (>500 pg/ml vs 20 pg/ml in serum) at diagnosis and significant decline in follow-up.

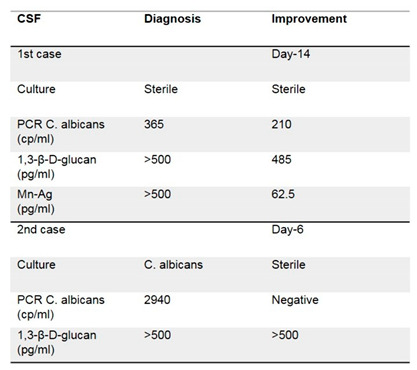



**Conclusion:** These two cases illustrate the potential value of PCR, Mn-Ag and BDG measurements in CSF for the diagnosis of *Candida* meningitis. Further analyses are required to define a threshold of positivity for BDG and Mn-Ag in CSF or the diagnostic value of CSF/serum indexes. Moreover, PCR and Mn-Ag may be useful for monitoring of response to therapy.

### P147. Usefulness of Galactomannan antigen test in Serum or Bronchoalveolar lavage sample in Kidney transplant patients

XessA.^1^XessI.^2^SinghG.^2^1Microbiology, all india institute of medical sciences, new delhi, India2Microbiology, All India Institute of Medical Sciences, new delhi, India

**Objectives:** Evaluation of Galactomannan in BAL and Serum samples in Kidney Transplant Patients

**Methods:** A total of 127 kidney transplant patients (KTP) suspected of invasive pulmonary aspergillosis (IPA) were enrolled for the study. Patients were further investigated radiologically and microbiologically. Galactomannan was done in serum and BAL samples Receiver Operating Curve (ROC) was generated to determine the optimal cut off value for Galactomannan in BAL and serum samples.

**Results:** Out of 127 patients 32(25.2%) were females and 95(74.8%) were males. 76/127(59.8%) patients presented with fever not responding to antibiotics and 62/127(48.8%) presented with cough/respiratory distress. 54/127 (42.5%) presented with both fever and cough. In CT scan, 24/127(18.9%) patients showed consolidation,10/127(7.9%) showed ground glass appearance, 5/127(3.9%) showed nodules and 3/127(2.4%) patients had only opacity. Galactomannan antigen was tested in 138 serum and 47 BAL samples of 127 patients. Forty-four serum samples and 34 BAL samples were positive for galactomannan antigen. Three patients were positive for fungal elements on KOH-calcofluor stain and two were only positive for culture. During the course of hospitalization 12/127(9.4%) received Amphotericin-B, 4/127(3.1%) voriconazole, 3/127(2.4%) caspofungin, 2/127(1.6%) posaconazole and 1/127(0.8%) itraconazole. Mortality was around 22% (28/128). Receiver operating characteristic (ROC) curve was plotted for BAL and Serum galactomannan levels. The value for BAL was found to be 1.0 and for serum it was 0.7.

**Conclusion:** Corrected BAL and serum galactomannan values for diagnosis of invasive fungal aspergillosis in kidney transplant patients were found to be 1.0 and 0.7 respectably, can be useful for diagnosis where the sensitivity of direct demonstration and culture is very low.

### P148. Potential impact of anti-mould prophylaxis on Aspergillus PCR blood testing for the diagnosis of invasive aspergillosis: a Fungal PCR initiative (FPCRI) systematic review and meta-analysis.

CrucianiM.^1^WhiteP.^2^LöfflerJ.^3^MortonC.O.^4^BarnesR.^5^DonnellyJ.P.^6^KlingsporL.^7^BuchheidtD.^8^MaertensJ.^9^HeinzW.J.^10^RogersT. R^11^WarrisA.^12^MengoliC.^13^LockhartD.^12^CordonnierC.^14^1Infectious Diseases, San Bonifacio Hospital, Verona, VERONA, Italy2Microbiology, Public Health Wales Microbiology, Cardiff, United Kingdom3Medizinische Klinik Und Poliklinik Ii, Universitätsklinikum Würzburg, Würzburg, Germany4western Sidney University, Sidney, Australia5Medical Microbiology and Infectious Diseases, Cardiff University School of Medicine, Cardiff, United Kingdom6EAPCRI, Nijmegen, Netherlands7Laboratory Medicine, Karolinska Insitutet, Stockholm, Sweden8Mannheim University Hospital, Heidelberg University, Mannheim, Germany9KU Leuven, Leuven, Belgium10University hospital, Wuerzburg, Germany11Department of Clinical Microbiology, St. James’s Hospital, Dublin, Ireland12Mrc Centre For Medical Mycology, University of Aberdeen, Aberdeen, United Kingdom13University of Padua, Padua, Italy14University of Paris, Paris, France

**Objectives:** To analyse the impact of anti-mould prophylaxis on the diagnostic accuracy of *Aspergillus* PCR for the diagnosis of Invasive Aspergillosis (IA) in immunocompromised patients.

**Methods:** Systematic review and meta-analysis of prospective cohort studies evaluating *Aspergillus* PCR testing of blood from high-risk neutropenic cancer patients, haematopoietic stem cell transplant (HSCT) and solid organ transplant recipients. QUADAS-2 was used to assess methodological quality of included studies. The sensitivity and specificity of *Aspergillus* PCR was evaluated by meta-analytical pooling of logit sensitivity and logit specificity. We also calculated the diagnostic Odd Ratio (DOR). A subgroup analysis of studies using anti-mould prophylaxis compared to studies not using anti-mould prophylaxis (or using placebo or fluconazole) was performed

**Results:** Nine studies used anti-mould prophylaxis (itraconazole, voriconazole, amphotericin B or caspofungin) in the entire population or in a subset of patients, while 13 studies did not use any anti-mould prophylaxis. The incidence of IA was significantly lower in the prophylaxis group compared to the non prophylaxis group (11.43 vs 16.13; p = 0.0005). The use of anti-mould prophylaxis increased sensitivity (from 0.80 to 0.86; p = n.s) and decreased specificity (from 0.78 to 0.64; p = 0.059) and DOR (from 17.10 to 11.14; p = n.s.). In sensitivity analysis, removing 3 studies using prophylaxis with itraconazole has no significant impact on sensitivity, Sensitivity and specificity of PCR test in studies using or not anti-mould prophylaxis. TP, TN = true positive and true negative results of PCR test, compared to total cases of IA according to the reference standard (EORTC/MSG criteria) or no-IA. specificity and DOR.

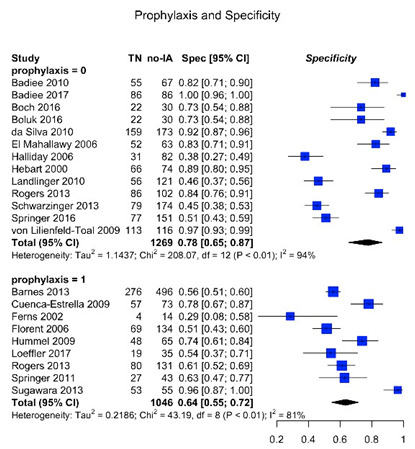

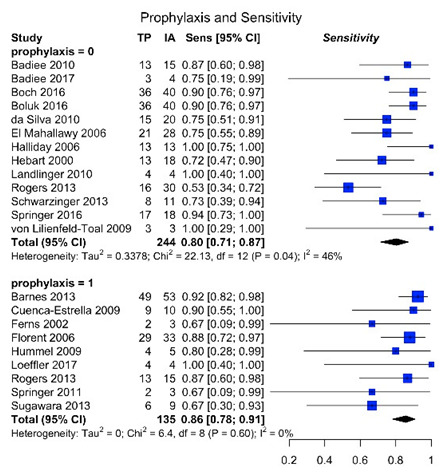





**Conclusion:** The cumulative incidence of IA was significantly lower in patients receiving anti-mould prophylaxis. The use of prophylaxis decreases the specificity of *Aspergillus* PCR testing of blood, which is of borderline statistical significance. Although an increase in sensitivity and decrease in DOR was observed, this did not reach statistical significance between the 2 groups. Anti-mould prophylaxis reduces the proportion of proven/probable cases of IA (according to EORTC/MSG criteria) which is associated with a lower specificity of the *Aspergillus* PCR testing of blood.

### P149. Comparison of fungal cultures and Aspergillus PCR kits results in SOT patients: an evaluation of five new commercial kits on respiratory samples.

ArgyN.^1^BonnalC.^1^GodetC.^2^TimsitJ.F.^3^Lortat-JacobB.^4^HouzéS.^1^1Laboratoire De Parasitologie Mycologie, Hôpital Bichat-Claude Bernard, 46 rue Henri Huchard, PARIS, France2Service De Pneumologie B, Hôpital Bichat-Claude Bernard, 46 rue Henri Huchard, PARIS, France3Service De Réanimation Médicale Et Infectieuse, Hôpital Bichat-Claude Bernard, 46 rue Henri Huchard, PARIS, France4Service De Réanimation Chirurgicale, Hôpital Bichat-Claude Bernard, 46 rue Henri Huchard, PARIS, France

**Objectives:** The diagnosis of invasive aspergillosis (IA) is difficult in patients without hematological malignancies. For diagnosis, direct microscopy, histopathology and culture are strongly recommended and, in solid organ transplant patients (SOT), broncho-alveolar lavage (BAL) galactomannan measures are recommended as well. Recent recommendations suggest that BAL-PCR should be considered in conjunction with other diagnostic tests in lung transplanted patients particularly^1^. Commercially *Aspergillus fumigatus* and *Aspergillus spp* PCR kits are now available but their evaluation in clinical practice remains to be performed. The aim of our study was to compare the results obtained with some of those Aspergillus PCR kits and cultures performed on respiratory samples in non-hematological patients.

**Methods:** Five kits (Ademtech MycoGENIE® *Aspergillus fumigatus* and MycoGENIE® *Aspergillus* species, AsperGenius® Species PathoNostics (*A fumigatus, A terreus, Aspergillus spp*), Fungiplex® IVD PCR (*Aspergillus spp, A terreus*) Bruker and *Aspergillus* ELITe MGB® ElitechGroup (*Aspergillus spp*.)) were evaluated on respiratory samples which were routinely analyzed for patients (including SOT) in our hospital. The PCR results were considered as not acceptable if the matching between fungal cultures and PCR results represents less than 70% of the samples tested (in bold in results).

**Results:** Forty respiratory samples (BAL, bronchial aspirates) from 36 patients were included. For 30 samples, 8 *A fumigatus*, 14 *Aspergillus spp* (4 *A flavus*, 4 *A niger*, 2 *A hiratsukae*, 1 *A terreus*, 1 *A nidulans*, 1 *A calidoustus* and 1 *Aspergillus spp*.) and 8 *Penicillium spp*. were isolated. Twelve were negative. For MycoGENIE^®^ *A fumigatus,* MycoGENIE^®^ *Aspergillus species,* AsperGenius^®^ *A fumigatus,* AsperGenius^®^ *Aspergillus spp,* Fungiplex^®^ and ELITe MGB^®^ respectively, the results were: - PCR positive results/number of samples tested and *A fumigatus* positive cultures: 7/8, 7/8, **5/8**, 6/8, 5/7, **3/8,** - PCR positive results/number of samples tested and *Aspergillus spp* positive cultures: 0/12, **6/12**, 1/10, **6/10**, **6/12**, 2/12, - PCR negative results/number of samples tested and *Penicillium spp*. positive cultures: 6/7, 6/8, 8/8, **3/8, 4/7**, 7/7 - PCR negative results/number of samples tested and negative cultures: 7/8, 10/12, 7/9, **5/9, 8/12**, 11/12. MycoGENIE® *Aspergillus fumigatus* shows a high concordance for *A fumigatus* positive cultures without false positive results for *Aspergillus spp* or *Penicillium spp*. AsperGenius® *A fumigatus* does not give satisfactory results for positive cultures. Neither of the *Aspergillus spp.* PCR kits have a good correlation with the culture results.

**Conclusion:** MycoGENIE® *Aspergillus fumigatus* seems to be suitable for the diagnosis of AI due to *A fumigatus* and must be prospectively studied in non-hematological patients. For *Aspergillus spp.* PCR kits, all except ELITe MGB® gave unsatisfactory results for negative results, mainly because of the cross reactivity with other hyalohyphomycetes. This is a real problem for lung transplanted patients who are often colonized with filamentous fungi and may have positive PCR results without AI. On the other hand, it is difficult to conclude about their correlation with positive cultures because it may reflect colonization without infection. The cohort was too small for a clinical evaluation therefore prospective evaluation of these PCRs in patients without hematological malignancies are needed. Ullmann AJ et al. Clin Microbiol Infect. 2018

### P150. rHigh-Volume Culture and Quantitative Real-Time PCR for the Detection of Aspergillus in Sputum

VergidisP.^1^MooreC.B.^2^Novak-FrazerL.^2^,^3^Rautemaa-RichardsonR.^3^,^4^WalkerA.^3^DenningD.^3^,^4^RichardsonM.D.^2^,^3^1Infectious Diseases, Mayo Clinic, Rochester, United States of America2Mycology Reference Centre, Excellence Center For Medical Mycology (ecmm), Wythenshawe Hospital, Manchester University NHS Foundation Trust, Manchester, United Kingdom3Division of Infection, Immunity and Respiratory Medicine, University of Manchester, Manchester, United Kingdom4National Aspergillosis Centre, Manchester University NHS Foundation Trust, Manchester, United Kingdom

**Objectives:** Sputum culture is an insensitive method for the diagnosis of pulmonary aspergillosis. Growth of the organism allows identification of the causative species and susceptibility testing, both of which can inform treatment choices. The current practice is to culture an aliquot of diluted sputum. We assessed the value of culturing large volumes of unprocessed sputum, a method that we have termed high-volume culture (HVC).

**Methods:** Specimens were processed by conventional culture (using an aliquot of homogenized, diluted sputum on Sabouraud agar at 37°C and 45°C for up to 5 days) and HVC (using undiluted sputum on Sabouraud agar at 30°C for up to 14 days). A separate specimen was tested by quantitative real-time PCR (qPCR). Aspergillus MycAssay (Myconostica) was used from January 2015 until February 2016. Aspergillus FUMI (Progenie) was used from February until October 2016. Aspergillus Elite MGB (ELITechGroup) was used from October 2016 through February 2017. Antifungal susceptibility testing was performed by the EUCAST standard.

**Results:** We obtained 290 sputum specimens from 231 patients with the following conditions: Chronic pulmonary aspergillosis (67.1%), allergic bronchopulmonary aspergillosis/severe asthma with fungal sensitization (25.1%) and *Aspergillus* bronchitis (7.8%). The sensitivity of conventional culture was 16.2% (95% CI: 12.4-20.9%) and that of HVC was 52.4% (95% CI: 46.7-58.1%) (*p*<0.001). qPCR had an overall sensitivity of 49.0% (95% CI: 41.2%-56.9%), comparable to that of HVC (*p* = 0.46). Rates of growth by HVC in correlation with transformed Ct values (TCt) for MycAssay and FUMI are shown in Fig. 1. There is a correlation between higher TCt values and the percentage of growth by HVC. By combining HVC and qPCR, the sensitivity increased to 66.0% (Fig. 2. Solid bars patients not receiving antifungal treatment. Dotted bars represent specimens of patients receiving treatment). *A. fumigatus* was the most commonly isolated species (82.9%) followed by *A. niger* (9.2%), *A. montevidensis* (3.3%) and *A. flavus* (2.6%). Among *A. fumigatus* isolates, pan-azole resistance was detected in 17.2%. At the time of sputum collection, 52.1% of patients were receiving antifungal treatment. Among patients with CPA, the sensitivity of HVC was higher compared to conventional culture for those treated with itraconazole (48.0% vs 12.0%, p<0.001) as well as those not receiving antifungal drug treatment (60.5% vs 26.7%, p<0.001). This trend is replicated in patients treated with other antifungals for all three conditions. HVC allowed for detection of azole-resistant isolates that would have been missed by conventional culture. 

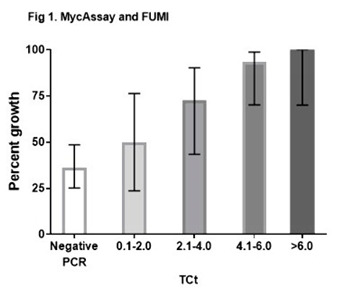

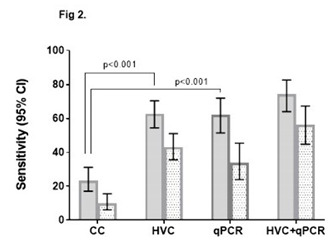





**Conclusion:** The recovery rate of *Aspergillus* spp. is significantly higher for HVC compared to conventional culture. HVC can be performed in any microbiology laboratory without the need for additional equipment or technical skills. The sensitivity of HVC is comparable to that of qPCR, although susceptibility testing is not possible by the latter technique. Combination of the two methods can lead to further increase in the recovery of *Aspergillus* from sputum specimens. Use of HVC of expectorated sputum for the detection of fungi can have a significant impact in the diagnosis and management of patients with pulmonary mycoses.

### P151. Aspergillus, Mucorales, Fusarium: Detectable in BAL by real-time PCR?

SpringerJ.^1^TeschnerD.^2^KesselJ.^3^CornelyO.A.^4^LiebregtsT.^5^KrauseS.W.^6^SchwartzS.^7^KiehlM.G.^8^HeinzW.J.^1^WillingerB.^9^EinseleH.^1^RickertsV.^10^LöfflerJ.^1^1University hospital, Wuerzburg, Germany2University Medical Center of the Johannes Gutenberg University, Mainz, Germany3University Hospital Frankfurt, Frankfurt, Germany4German Centre For Infection Research, Partner Site Bonn-cologne, Cologne, Germany and Clinical Trials Centre Cologne (zks Köln), University of Cologne, Cologne, Germany5University Hospital Essen, Essen, Germany6University Hospital Erlangen, Erlangen, Germany7Charité Berlin, Berlin, Germany8Klinikum Frankfurt/Oder, Frankfurt/Oder, Germany9Medical University of Vienna, Vienna, Austria10Robert Koch Institut Berlin, Berlin, Germany

**Objectives:** Invasive fungal infections (IFI) remain a major complication in patients with haematological malignancies (HM) and post allogeneic haematopoietic stem cell transplantation (HSCT). In these patients, invasive aspergillosis (IA) is the most common cause of mortality due to infection. Early detection of fungal infections has the potential to facilitate a more effective management of invasive disease. Bronchoalveolar lavage samples (BAL) directly obtained from the focus of infection are often used as diagnostic tool. In this study, BAL samples were tested using different real-time PCR assays and correlated to EORTC/MSG classification for IFI (DePauw 2008).

**Methods:** In a multi-centre approach, nine hospitals collected BAL samples from HM/HSCT patients comprising a CT scan suspicious for IFI. DNA was isolated from supernatant and BAL pellet and analysed for the presence of fungal DNA. Analysis was performed in Wuerzburg only by using 3 specific real-time PCR assays and one panfungal assay (Khot 2009, 28S 10f-12r). All samples were analysed for the presence of *Aspergillus* and Mucorales, additionally some for *Fusarium* and fungal DNA.

**Results:** In total, 294 samples were collected. 69/294 showed *Aspergillus* DNA, 20/294 Mucorales, 2/141 *Fusarium* and 62/69 panfungal DNA (Table 1). Sequencing of panfungal amplicons revealed mostly DNA of yeasts (mainly *Malassezia sp*. and *Candida sp*.) which were classified as non-invasive/contaminations. Only in 7 cases (7.9%; 6 *Aspergilli*, 1 Mucorales) the result of the specific assay could be confirmed. Therefore no further PCR analysis using the panfungal assay was performed. In correlation to EORTC/MSG criteria (6 proven, 85 probable, 130 possible IFD and 26 undetermined cases; 47 without data) *Aspergillus* DNA was detected in 42 probable/proven cases, Mucorales in 11, *Fusarium* in 1 and panfungal DNA in 24 (only 7 if excluding contaminations) (Table 2). DNA of *Aspergillus* and Mucorales was detected in just one or none control samples (undetermined cases), 10/69 samples (14.5%) showing DNA of *Aspergillus* were also positive for Mucorales (*n* = 9) or *Fusarium* (*n* = 1). Changing the perspective revealed that almost half of the Mucorales positive samples were double-infected with *Aspergillus* (9/20; 45%).

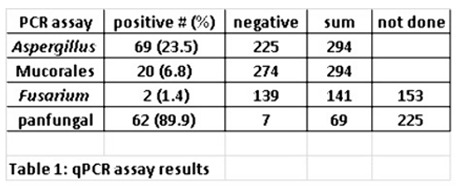

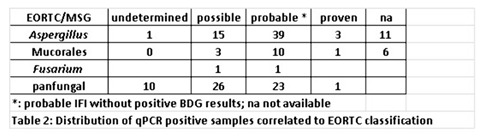





**Conclusion:** qPCR was able to detect DNA of specific fungi in BAL samples. Specific assays for *Aspergillus*, Mucorales and *Fusarium* proved to be helpful to detect fungal pathogen DNA, whereas the panfungal approach mainly detected non-invasive contaminations. The number of double-infections seems to be higher than expected (14.5% to 45%).

### P152. Analysis of the efficacy of bis(methyl)gliotoxin for early invasive aspergillosis detection and to guide antifungal treatment in neutropenic pediatric oncology patients.

DomingoM.P.^1^NabalS.^2^RedradoS.^1^LopezC.^2^Rodriguez-VigilC.^3^MuñozA.^3^CalvoC.^3^RezustaA.^2^PardoJ.^4^GalvezE.^1^1Instituto de Carboquimica (ICB-CSIC), Zaragoza, Spain2Microbiology, Hospital Miguel Servet, Zaragoza, Spain3Oncology Pediatric, Hospital Miguel Servet, Zaragoza, Spain4Centro De Investigaciones Biomedicas, Universidad De Zaragoza, ARAID, Zaragoza, Spain

**Objectives:** Invasive pulmonary aspergillosis (IPA) is a leading cause of morbidity and mortality in immunocompromised patients. This is due in part to the absence of optimal diagnostic modalities, which hamper early specific disease detection. Systemic detection of serum biomarkers is a standard noninvasive practice used to early diagnose and treat IPA in high risk population. During the last years, doubts have been raised regarding the use of galactomannan (GM) in onco-hematological population mainly due to the large number of patients with false positive test as well as the low efficacy in patients under antifungal profylaxis. In a previous study, we demonstrated the high diagnostic accuracy of bis(methyl)gliotoxin (bmGT) compared to GM for IPA diagnosis in adult oncohematological patients. However, the performance of bmGT as a diagnosis biomarker in pediatric patients is unknown. The objective of this study is to compare the ability of bmGT and GMN detection for IPA diagnosis in oncopediatric patients and to analyse the efficacy of bmGT to serve as guide for voriconazol treatment.

**Methods:** The presence of bmGT and voriconazole levels in sequential series of serum from 7 oncopediatric patients at risk of IA (prospective samples) is simultaneously quantified by High Performance Thin Layer Chromatography (HPTLC) as well as LC/MS/MS.

**Results:** We have established a fast and sensitive technique for simultaneous separation, detection and quantification of bmGT and voriconazole in serum and BAL of pediatric patients. bmGT and GM were negative in all possible IA cases (5 patients). Two patients, presenting prolonged neutropenia after chemotherapy treatment, fever, hypoxemia and pulmonary infiltration compatible with Aspergillosis in high resolution CT, were categorised as probable IA. GM and bmGT was monitored in serum and bronchoalveolar lavage. In one patient, GM and bmGT were detected in serum and with the voriconazole treatment GM was negativized and the bmGT levels decreased. bmGT was positive earlier than GM allowing early initiation of treatment. In the second patient, GM was negative and bmGT positive in both serum and BAL, so treatment with voriconazole was started. Treatment decreased bmGT values coinciding with the patient clinical improvement.

**Conclusion:** BmGT has allowed us the early diagnosis and successful treatment of pediatric IA, being a promising tool for early detection of IA in neutropenic pediatric patients.

### P153. Identification for major Aspergillus species in paraffin-embedded tissue: newly developed multiplex real-time PCR method using hydrolysis probes

ChoiJ.-K.^1^,^2^ChoS.-Y.^1^,^2^,^3^ParkC.^2^LeeD.-G.^1^,^2^,^3^1Division of Infectious Diseases, Department of Internal Medicine, College of Medicine, The Catholic University of Korea, Seoul, Republic of Korea2Vaccine Bio Research Institute, Seoul, Republic of Korea3Catholic Hematology Hospital, Seoul, Republic of Korea

**Objectives:** Species identification is crucial for determining appropriate antifungal agents in treatment of fungal infections. However, current culture-based methods are insensitive due to low positive rate and slow process. Microscopic examination of tissue specimens has limitations in distinguishing fungal organisms. Detection of fungal DNA by polymerase chain reaction (PCR) method in tissue samples is expected to be useful for the diagnosis of fungal infections. Herein, we describe the performance and clinical impact of a multiplex fungal PCR assay of formalin-fixed paraffin-embedded (FFPE) sample for the diagnosis of *Aspergillus* infections.

**Methods:** FFPE samples previously collected from patients diagnosed as fungal infections by histopathologic finding between June 2009 and June 2012 were used for PCR analysis, and retrospective chart review was also performed for correlation with clinical diagnosis. Real-time PCR primers and multiplex hydrolysis probes were newly designed for four major *Aspergillus* species (*A. fumigatus*, *A. niger*, *A. terreus*, and *A. flavus*) based on molecular analysis data of β-tubulin gene (*benA*) in *Aspergillus* isolates. The method of real-time PCR was qualified and quantified using the reference genomic DNA (gDNA) of *Aspergillus* species. DNA was extracted from FFPE sample using TaKaRa DEXPAT kit (TaKaRa Biomedicals, Shiga, Japan). The amplified *benA* amplicon was analyzed by comparing with the standard curve of the reference gDNA. Multiplex PCR was performed on 72 FFPE samples. The sample quality was validated by human globulin detection, and 61 qualified samples were analyzed. 

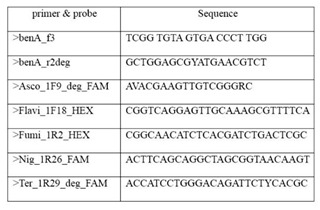



**Results:** Twenty-seven of 35 cases clinically suspected as aspergillosis presented positive results in this real-time PCR method (sensitivity: 77.1%). The positive rate was higher than that of the fungal culture (12/35, 34.3%) or galactomannan test (20/33, 60.6%). Among the 23 cases with culture-negative proven/probable invasive aspergillosis, 17 cases were confirmed as positive by real-time PCR. *Aspergillus* gene was identified in 5 of 9 IFI cases that causative fungal organisms were not confirmed in clinical and pathological examination. In cases with non-*Aspergillus* infection, *Aspergillus* gene was detected in 2 of 13 cases. By real-time PCR for FFPE specimen, additional informations, not only detection of *benA* gene, but also species-level identification, were obtained in 51.7% (32 of 61) cases included in this study.

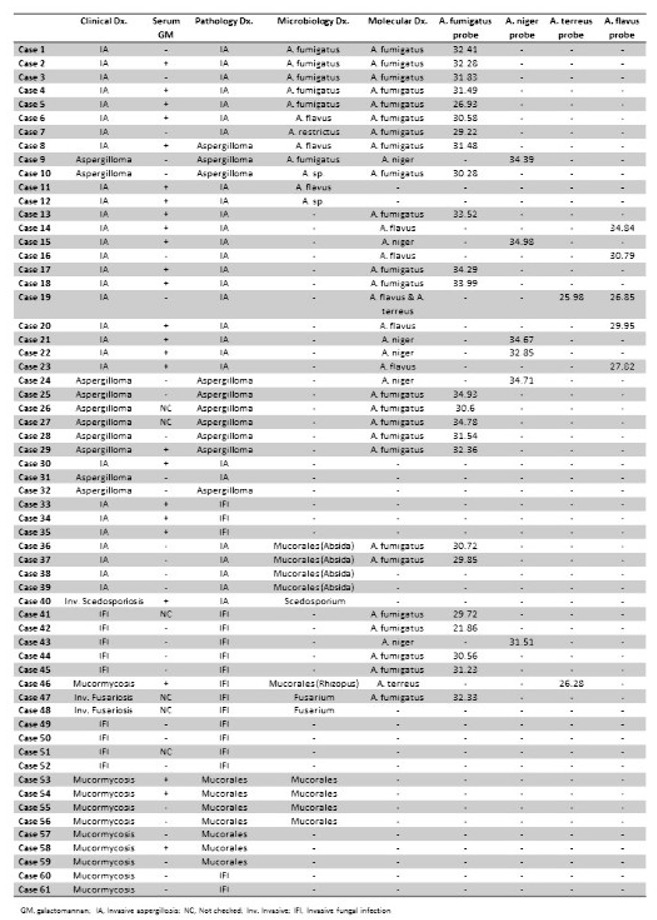



**Conclusion:** This study revealed that this newly developed real-time PCR method for FFPE tissue could improve diagnostic accuracy of aspergillosis. Further study is needed to understand mixed mold infections or false positive cases with additional assays including PCR for non-*aspergillus* mold.

### P154. New approaches to the verification of bacterial and candidal bloodstream infection.

KutsevalovaO.^1^AntonetsA.^2^KitO.^3^PanovaN.^1^LysenkoI.^4^DmitrievaV.^3^PakE.^3^KozyukO.^3^MarykovE.^3^KlyasovaG.^5^1Laboratory of Clinical Microbiology, Rostov Research Institute of Oncology, Rostov-on-Don, Russian Federation2Laboratory of Molecular Oncology, Rostov Research Institute of Oncology, Rostov-on-Don, Russian Federation3Rostov Research Institute of Oncology, Rostov-on-Don, Russian Federation4Oncohematology Department, Rostov Research Institute of Oncology, Rostov-on-Don, Russian Federation5National Research Center for Hematology, Moscow, Russian Federation

**Objectives:** To evaluate the prognostic significance of procalcitonin and mannan antigen of *Candida spp* (MA) for early and accessible verification of the causative agent of bloodstream infection (BSI).

**Methods:** We examined 349 patients with suspected BSI from 16 intensive care, oncology and oncohematology units of 9 hospitals in the Southern Federal District of Russia. The diagnostic algorithm included sterility blood testing and a study of biochemical markers with immunoenzymatic assays. Sterility blood testing was performed using ‘BacT/ALERT3D analyzer. Identification of strains and detection of their antimicrobial drugs sensitivity was made with Vitek-2 automatic analyzer (BioMerieux, France). Simultaneously we measured the levels of procalcitonin and MA with the use of Procalcitonin kit ELISA-BEST (Russia) and Platelia™ Candida Ag Plus kit (France) correspondingly. Only high values of procalcitonin (10 and above ng/ml) were considered as expedient in the diagnosis of bacterial sepsis. The measurement of MA made it possible to judge about the involvement of *Candida spp*. in the infectious process. The result of MA testing was regarded as positive with the concentration ≥125 pg/ml.

**Results:** Positive blood cultures were obtained in 84 (24.1%) patients. Bacteria accounted for 77.4% (65 strains), *Candida spp.* - 22.6% (19 isolates). In 3 cases (3.6%) bacterial-candidal associations were detected which worsened the condition of the patients. In a parallel study of biochemical markers, an increased level of one of them was in 205 (58.7%) patients. High values of procalcitonin accounted for 68 (33.2%) that was in favor of a severe bacterial infection. Positive results for MA were in 118 (57.6%) patients. Positive results of MA testing in the presence of clinical symptoms and risk factors for the development of invasive candidiasis suggested a candidal BSI. In 19 (9.2%) patients two biochemical markers were elevated indicating a possible mixed infection (picture 1,2). For 144 (41.3%) patients with initial negative blood culture testing and normal biomarker levels additional studies were needed to exclude or confirm BSI. As a result, in 26 patients a negative results were regarded as a cancer relapse. 118 patients needed dynamic blood testing. 70 patients received a positive results. 14 of them had high procalcitonin and 56 had a positive MA. 18 patients also had a positive blood culture (4 patients had bacteria isolated, 14 - candida). 52 patients were observed with only positive biomarkers (10 with procalcitonin, 42 with MA). The rest 48 patients were with fever of unknown origin.

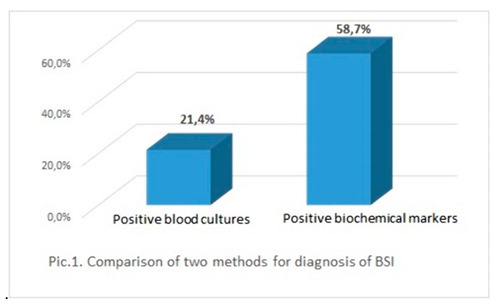

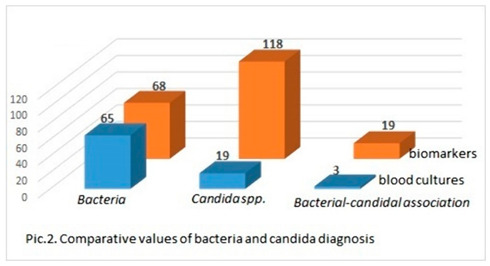





**Conclusion:** A comprehensive approach to the diagnosis of BSI has increased the percentage of pathogen verification to 58.7%. It should be taken into consideration that both blood culture and biomarkers testing of bacterial infection revealed almost the same diagnostic significance. However, MA testing improved dramatically the early diagnosis of a candidal BSI despite negative blood culture testing which is probably due to the prolonged cultivation of *Candida spp*. in the blood. The inclusion of biochemical markers testing in the diagnostic algorithm for suspected BSI allowed to identify the causative infectious agent as early as possible and to start adequate antibacterial or antifungal therapy.

### P155. Validation and ongoing evaluation of a real time PCR assay for Pneumocystis jirovecii detection in bronchoalveolar fluid specimens; data from Pakistan

JabeenK.^1^GhanchiN.^2^FarooqiJ.^2^MasoodK. Iqbal^2^SiddiquiO. Ali^2^HumayunA.^2^HasanZ.^2^1Pathology and Laboratory Medicine, Aga Khan University, Karachi, Pakistan, Pakistan2Pathology and Laboratory Medicine, Aga Khan University, Karachi, Pakistan

**Objectives:** Pneumocystis pneumonia (PCP) is a frequent opportunistic infection in HIV-positive and immunocompromised individuals. Healthy individuals have been shown to harbor the organism without clinical signs of infection and may play a role in the transmission of PCP. PCR based assays for detection of *P. jirovecii* are reported to be more sensitive compared to conventional immunofluorescence assays (IFA). We report experience of a tertiary care hospital laboratory in Pakistan which recently shifted from IFA based to PCR based diagnosis for PCP pneumonia.

**Methods:** The study was conducted at the clinical laboratory of Aga Khan University Hospital, Karachi Pakistan. In the first phase (January-May 2018), a real time PCR assay was validated against twenty archived PCP positive or negative bronchoalveolar lavage (BAL) specimens. Once validation was completed satisfactorily, PCR based assay was introduced and offered to clinicians in June 2018. All BAL specimens submitted for detection for PCP from June 2018-May 2019 after the introduction of test were included and evaluated. Clinical data collected as routine part of reporting was assessed. Positivity rate for *Pneumocystis* detection was evaluated for PCR during this period. Additionally PCP data using IFA was retrieved for the year 2016-2017 to compare the positivity rate of IFA with PCR over these two time periods.

**Results:** Initially twenty samples from patients clinically suspected of PCP were compared using both IAF and PCR. PCR confirmed five (25%) positive cases compared to IAF which confirmed 3(12%). After introduction of PCR in the laboratory, a total of 145 suspected patient BAL samples were tested for *P. jirovecii* of which 25 (17%) were PCR positive. Majority of the PCR confirmed patients were male (72%). Clinical data available on 18/25 (72%) positive patients revealed 4 (16%) patients positive for human immunodeficiency virus; 3 (12%) with underlying autoimmune disease; 6 (20%) with malignancy, 2 (4%) on corticosteroid therapy and 1 patient on immunosuppressive therapy post renal transplant. PCP detection increased from 4.5% during 2016-2017 using IFA based assay to 17% using PCR based assay during June 2018-May 2019.

**Conclusion:** A high positivity rate of 17% was seen in clinically suspected PCP patients using PCR based diagnosis in this study. PCP cases were reported in both HIV positive and negative patients. There was an improvement in case detection using PCR compared to IFA. However, high cost of PCR compared to IFA limits wider availability of PCR based PCP diagnosis in resource limited settings.

### P156. A comparative genome approach to identify specific DNA sequence for PCR diagnosis of Madurella mycetomatis

LimW.^1^SmitS.^2^FahalA.H.^3^SandeW. Van De^4^1Microbiology and Infectious Diseases, Erasmus Medical Center, Rotterdam, Netherlands2Department of Plant Science - Bioinformatics, Wageningen University & Research, Wageningen, Netherlands3Mycetoma Research Center, University of Khartoum, Khartoum, Sudan4Department of Microbiology and Infectious Diseases, Erasmus Medical Center, Rotterdam, Netherlands

**Objectives:** The current gold standard of identifying eumycetoma causative agents is to culture the isolate and assess the morphological characteristics. This method of identification is time consuming and can often lead to misidentifications. Precise and early detection of the causative agent is crucial for clinical management. Currently precise species identification can only be achieved by sequencing the internally transcribed spacer (ITS) regions or by using species-specific PCRs. Only for *M. mycetomatis* a species specific PCR was developed, but recently it was demonstrated that this PCR did cross-react with other *Madurella* species. We therefore looked into the sequenced *M. mycetomatis* genome in search for unique and specific protein coding sequences which could be transformed into a species specific diagnostic PCR.

**Methods:** We used a comparative genome approach to identify unique sequences for *M. mycetomatis* based on its predicted protein coding sequences. The homology of these sequences were identified using blastclust, localization of the proteins was identified using WolfPSORT and SignalP was used to predict the secretion signals in these predicted proteins sequences. Based on these sequences, PCR primers were developed and validated in a collection of 61 *M. mycetomatis*, 4 *Madurella tropicana*, 3 *Madurella pseudomycetomatis,* 3 *Madurella fahalii,* 2 *Falciformispora senegalensis*, 3 *Trematosphaeria subthermophilia*, 3 *Thielavia terrestris,* 3 *Trematosphaeria grisea*, 2 *Chaetomium globosum,* 1 *Aspergillus fumigatus* and 1 *Aspergillus terrus* isolates using conventional PCR.

**Results:** By using a comparative genomic approach, we were able to identify at least 32 *M. mycetomatis* unique protein sequences. For the top 16 most promising candidate sequences, PCR primers were developed. of these primer pairs, only 12 were able to detect all *M. mycetomatis* isolates tested. of these 12, only 1 did not react with any other fungal species tested.

**Conclusion:** Based on this comparative genomics approach, we identified a novel *M. mycetomatis* specific PCR primer pair to detect *M. mycetomatis.*

### P157. Performance of T2Candida Panel for the diagnosis of intra-abdominal candidiasis

LamothF.^1^NguyenM.H.^2^TissotF.^1^SquiresK.^2^EggimannP.^1^FlückigerU.^3^SiegemundM.^3^ZimmerliS.^4^CalandraT.^1^MarchettiO.^1^ClancyC.J.^2^BochudP.-Y.^1^1Infectious Diseases Service, Lausanne University Hospital, Lausanne, Switzerland2University of Pittsburgh Medical Center, Pittsburgh, United States of America3University Hospital of Basel, Basel, Switzerland4Department of Infectious Diseases, University Hospital of Bern, Bern, Switzerland

**Objectives:** Intra-abdominal candidiasis (IAC) is a frequent and life-threatening infection in critically ill patients following complicated abdominal surgery. Diagnosis of IAC is difficult because of the lack of sensitivity of blood cultures and the limited specificity of indirect markers, such as the beta-D-glucan (BDG) test. The T2Candida Panel (T2C, T2Biosystems) is a magnetic resonance-based molecular diagnostic test that identifies the five most common pathogenic *Candida* spp, (*C. albicans* and *C. tropicalis* [CA/CT], *C. glabrata* and *C. krusei* [CG/CK], and *C. parapsilosis*) directly in whole blood specimens within 7 hours. T2C demonstrated excellent performance for the diagnosis of candidemia, but data for non-candidemic invasive candidiasis are lacking. We tested T2C’s efficiency in detecting IAC.

**Methods:** We used the T2C panel to test frozen whole blood samples from critically-ill patients previously enrolled in a prospective cohort study of the Fungal Infection Network of Switzerland (FUNGINOS), who were at high risk for IAC due to at least one of the following conditions: i) recurrent gastro-intestinal tract perforation, ii) abdominal surgery and *Candida* colonization, iii) necrotizing pancreatitis or iv) burns (>25% body surface area). IAC was defined as growth of *Candida* spp. in a per-operative culture sample from an intra-abdominal abscess or peritoneal fluid. The samples were collected within 72h of IAC diagnosis or between days 7-10 from study inclusion for patients without IAC. Results of concomitant serum BDG (Fungitell) determined previously were included in the analysis.

**Results:** Whole blood samples from 46 patients were available for testing. Most patients had *Candida* colonization of ≥1 non-sterile site (98%) and *Candida* score ≥3 (89%). Eighteen (33%) of them had IAC: 13 were due to CA, 2 to CT, 1 to mixed CA/CG and 2 to *Candida* spp. not included in the T2C Panel (*C. kefyr* and *C. lusitaniae*). Antifungal therapy was ongoing at time of IAC diagnosis and blood sampling in 2 (11%) cases. Table 1 shows the comparative diagnostic performance of blood cultures, T2C and BDG. Blood cultures were positive in 2/18 (11%) IAC cases. T2C was positive in 6/18 (33%) IAC cases (including the 2 candidemic patients) with *Candida* spp. matching those isolated in cultures. Analysis limited to IAC cases due to *Candida* spp. of the T2C panel yielded a sensitivity of 37.5%. A positive T2C result was found in 2 of the 28 patients who did not fulfill IAC criteria (specificity 93%). While discordant results for T2C and BDG were observed in 21 (46%) cases, concordant positive results were associated with IAC in 100% of cases and concordant negative results with absence of IAC in 90% of cases. 

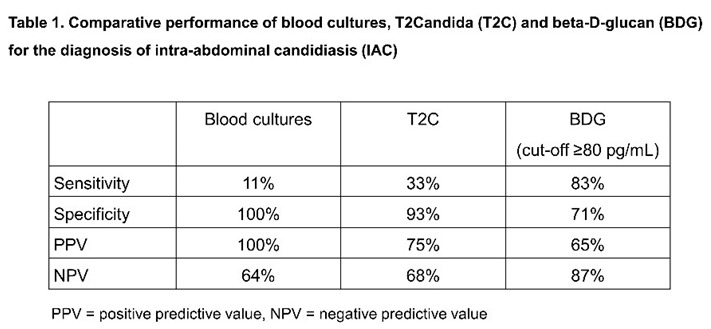



**Conclusion:** T2C has higher sensitivity than blood cultures and higher specificity than BDG for IAC diagnosis. T2C and BDG are anticipated to be most useful in guiding management of patients at risk for IAC, in particular when they provide concordant results to confirm or exclude IAC in cases for which intra-abdominal cultures are not feasible or delayed.

### P158. Performances of the GenMark’s ePlex® blood culture identification fungal pathogen (BCID-FP) panel: a prospective French evaluation

NawelA.-A.^1^FouletF.^1^AngebaultC.^1^SuyyaghS.^1^MaubonD.^2^BotterelF.^1^1Parasitologie-mycologie, CHU Henri MONDOR, Créteil, France2Univ. Grenoble Alpes, CNRS, CHU Grenoble Alpes, Grenoble INP*, TIMC-IMAG, 38000 Grenoble, France * Institute of Engineering Univ. Grenoble Alpes, GRENOBLE Cedex, France

**Objectives:** Fungemia is associated with high rate of morbidity increasing length of hospital stay and mortality. Delay of effective antifungal treatment is associated with unfavorable outcome and rapid species identification contributes to adapt antifungal therapy. ePlex^®^ Blood Culture Identification fungal pathogen panel (ePlex^®^ BCID-FP) developed by GenMark^®^ Diagnostics, Inc. (Carlsbad, CA, USA) is a fully automated easy-to-use cartridge designed to detect 15 fungal pathogens from positive blood culture (BC) including the emerging pathogen *Candida auris*. The objective of this study was to evaluate prospectively the performance of ePlex^®^ BCID-FP test using clinical BC samples.

**Methods:** The evaluation was performed prospectively on consecutive fungal BC over 2 years-period (from April 2017 to Mars 2019) in Henri Mondor University Hospital in France. BCs taken from patients were incubated in BacT/ALERT^®^ 3D automated system (Biomérieux^®^). Once detected positive for fungi, the BC underwent classical subcultures. Then, species identification was performed on MALDI-TOF MS Andromas^®^ system (identification within 24h). In parallel to this conventional procedure, ePlex^®^ BCID-FP was tested (hands-on-time < 5 min and time-to-result: 1.5h).

**Results:** A total of 58 BC, corresponding to 52 patients, were tested on ePlex^®^ using the BCID-FP. Invalid results due to technical issues were obtained in 10/58 tests (17.2%). Among the 48 valid tests, results were fully concordant with classical identification process in 45/48 BC isolated species (94%) including one fungemia caused by two species with *C. tropicalis* and *C. lusitaniae*. Identifications were available the day of BC positivity in less than two hours. This technique saved at least 24 hours compared to conventional methods. The non-concordant results consisted on one *C. parapsilosis* missed by ePlex and two species absent from the BCID-FP panel (1 *C. metapsilosis* and 1 *Saccharomyces cerevisiae*). Species distribution on valid tests was: 19 *C. albicans* (41%), 12 *C. parapsilosis* (26%), 8 *C. glabrata* (18%) and 7 others (*C. krusei* (*n* = 3), *C. tropicalis* (*n* = 2), *C. dublininiensis* (*n* = 1) and *C. lusitaniae* (*n* = 1) (15%). Thus, fluconazole could be introduced in first intention in 76% of cases, saving the use of caspofungin.

**Conclusion:** To our knowledge, this is the first large prospective evaluation of the ePlex^®^ BCID-FP on more than 50 fungal BC. ePlex^®^ BCID-FP accurately identified 78% of the fungal species from positive BC. Focusing on targets which are currently present in the panel, 100% of correct identification was achieved. A prospective clinical study evaluating the time-to-result benefit on antifungal stewardship and on length of hospital stay is needed.

### P159. Integrating miRNA and mRNA expression profiles for improving diagnosis of invasive aspergillosis

ZoranT.^1^,^2^SeelbinderB.^1^SieberP.^1^SchaeubleS.^1^LoefflerJ.^2^1Pidomics, Leibniz Institute for Natural Product Research and Infection Biology - Hans Knoell Institute, Jena, Germany2Department of Internal Medicine Ii, WÜ4i, University Hospital Wuerzburg, Wuerzburg, Germany

**Objectives:** Invasive aspergillosis (IA) is a life-threatening disease affecting immunocompromised patients. Due to unspecific symptoms of this disease and limitation of current methods based on fungal biomarkers, diagnosis of IA remains challenging. MicroRNAs (miRNAs) are small non-coding RNAs involved in the post-transcriptional regulation of several biological processes including various disease pathways and immune response to numerous pathogens. Due to their involvement in gene regulation, their stability and ubiquitous presence in biofluids they are considered as good biomarkers for different diseases. The aim of our *in vitro* study was to investigate miRNA profiles in immune cells following fungal and bacterial exposure and identify potential miRNAs and their target genes that may be useful as diagnostic and prognostic human biomarkers for improving IA diagnosis.

**Methods:** We used next generation sequencing (NGS) to investigate miRNA profiles in monocyte-derived dendritic cells (moDCs) generated from healthy volunteers. Immune cells were stimulated for 12 hours with inactivated *Aspergillus fumigatus* germ tubes and a clinical isolate of *Escherichia coli*. Next, we evaluated transcriptome by RNA-sequencing of moDCs infected with *E. coli* and *A. fumigatus* and investigated *in silico* interaction between differentially expressed miRNAs (DEMs) and their targets. After correlation analysis and prediction of interactions with mRNA, selected miRNA and their immunological relevant target genes were further experimentally investigated.

**Results:** Our data showed 32 significantly DEMs in moDCs stimulated with fungal and bacterial pathogens in comparison to normal expression in unstimulated moDCs. 28 miRNAs were differentially regulated after infection with *A. fumigatus* and 8 miRNAs after infection with *E. coli*. We identified 24 miRNAs significantly differentially regulated in moDCs after fungal but not bacterial infection. Among all significantly DEMs in stimulated moDCs, the following four miRNAs were observed to be differentially expressed after infection with *A. fumigatus* and *E. coli*: hsa-miR-9-5p, hsa-miR-155-5p, hsa-miR-155-3p and hsa-miR-1303. Based on two DEMs specific to *A. fumigatus* exposure and one DEM identified in moDCs infected with *A. fumigatus* as well as *E. coli*, we further investigated potential targets that had been predicted by investigating transcriptome data. Integration of hsa-miR-9-5p and gene expression showed 45 common differentially expressed (DE) target genes in both types of infection. Furthermore, our data showed 26 unique DE target genes in moDCs after exposure to *A. fumigatus* and 13 in moDCs infected with *E. coli* (*n* = 13). We validated the expression of three selected DEMs and their selected immunological relevant target genes in additional donors with Real-Time PCR, where we confirmed our observations from NGS data.

**Conclusion:** Our data showed distinctive miRNA profiles between uninfected moDCs as well as moDCs after exposure to *A. fumigatus* and *E. coli*, suggesting miRNAs profiles can be used to detect and distinguish bacterial from fungal infection. Furthermore, characteristic miRNA and mRNA expression profiles may be used as a diagnostic and prognostic biomarker of IA. Further experimental validation is necessary to confirm predicted miRNA-mRNA interactions and investigate the potential use of miRNA and mRNA expression profiles for IA diagnosis.

### P160. rThe Fungal PCR Initiative’s evaluation of in-house and commercial Pneumocystis jirovecii qPCR assays: towards a standard for a diagnostics assay

Gits-MuselliM.^1^,^2^WhiteP.^3^MengoliC.^4^ChenS.^5^CrowleyB.^6^DingemansG.^7^FrealleE.^8^GortonR.^9^GuiverM.^10^HagenF.^11^HallidayC.^12^JohnsonG.^13^LagrouK.^14^LengerovaM.^15^MelchersW.^16^Novak-FrazerL.^17^Rautemaa-RichardsonR.^18^,^19^SchererE.^20^SteinmannJ.^21^,^22^CrucianiM.^23^BarnesR.^24^DonnellyJ.P.^25^LoefflerJ.^26^BretagneS.^27^AlanioA.^27^1Molecular Mycology, Institut Pasteur, PARIS, France2Parasitology-mycology Laboratory, Lariboisière Saint-Louis Fernand Widal Hospital, Assistance Publique-Hôpitaux de Paris (AP-HP), PARIS, France3Microbiology, Public Health Wales Microbiology, Cardiff, United Kingdom4Molecular Medicine, University of Padova, Padova, Italy5Medicine, The University of Sydney, Camperdown, New South Wales, Australia6Department of Virology, St James’s Hospital, Dublin, Ireland, Dublin, Ireland7PathoNostics, Maastricht, Netherlands8Parasitology-mycology, CHU Lille, Lille, France9Infection Sciences, Health Services Laboratories, London, United Kingdom10Public Health Laboratory, National Infection Service Public Health England, Manchester University NHS Foundation Trust, Manchester, UK, Manchester, United Kingdom11Medical Mycology, Westerdijk Fungal Biodiversity Institute, Utrecht, Netherlands12Clinical Mycology Reference Laboratory, Centre for Infectious Diseases and Microbiology Laboratory Services, Institute of Clinical Pathology and Medical Research, New South Wales Health Pathology, Westmead Hospital, and the University of Sydney, Australia, Sydney, Australia13Scientific Development, OLM diagnostics, Newcastle, United Kingdom14Laboratory of Clinical Bacteriology and Mycology, Department of Microbiology and Immunology, Excellence Center For Medical Mycology (ecmm), KU Leuven, Leuven, Belgium15Department of Internal Medicine - Hematology and Oncology, University Hospital Brno, Brno, Czech Republic16Medical Microbiology, RadboudUMC, Nijmegen, Netherlands17Mycology Reference Centre, Excellence Center For Medical Mycology (ecmm), Wythenshawe Hospital, Manchester University NHS Foundation Trust, Manchester, United Kingdom18Division of Infection, Immunity and Respiratory Medicine, University of Manchester, Manchester, United Kingdom19Manchester Academic Health Science Centre, Manchester, United Kingdom20Parasitology - Mycology - Umr6249 Cnrs Chrono-environnement, University Hospital Besançon, Besançon, France21Institute of Clinical Hygiene, Medical Microbiology and Clinical Infectiology, Klinikum Nuernberg, Nuremberg, Germany22Institute of Medical Microbiology, University Hospital Essen, Essen, Germany23Infectious Diseases, San Bonifacio Hospital, Verona, VERONA, Italy24Medical Microbiology and Infectious Diseases, Cardiff University School of Medicine, Cardiff, United Kingdom25EAPCRI, Nijmegen, Netherlands26Department of Internal Medicine Ii, WÜ4i, University Hospital Wuerzburg, Wuerzburg, Germany27Paris-diderot, Sorbonne Paris Cité University, Institut Pasteur, Molecular Mycology Unit, Cnrs Cmr2000, Parasitology-Mycology Laboratory, Lariboisière Saint-Louis Fernand Widal Hospitals, Assistance Publique-Hôpitaux de Paris, Paris, France

**Objectives:** Quantitative real-time PCR (qPCR) is increasingly used to detect *Pneumocystis jirovecii* for the diagnosis of pneumocystis pneumonia (PCP). PCP is more severe and mortality rates are significantly higher in non-HIV patients than in HIV-infected/AIDS people, despite a lower fungal load during infection. Currently, definite thresholds for Pneumocystis pneumonia (PCP) in non-HIV patients have not been established to be consensual and universally used in different diagnostic laboratories. Therefore, diagnosis of PCP remains a challenge because of the importance of quantification of the low fungal loads. An evaluation of the performance of different qPCR assays is needed to assist laboratory standardization of quantification.

**Methods:** Through the Fungal PCR Initiative, a working group of the International Society for Human and Animal Mycology, one inter- and one intra-laboratory comparison of qPCR assays was performed. For the interlaboratory study, sixteen reference laboratories in eight countries running 19 assays analysed a panel consisting of two negative and three positive specimens from a pool of three different *P. jirovecii* positive bronchoalveolar lavage (BAL) fluids at three different dilutions (pure 1:1, 1:100 and 1:1000). Participants were asked to test the panel specimens as part of their routine molecular biology workflow. The results were collected using a dedicated Google form that included requests for the qualitative result (positive/negative), the quantification cycle (Cq) values for each replicate related to each specimen and specific information regarding the methods and workflows applied. The intra-laboratory study compared 10 different assays with the same person and qPCR thermocycler, by testing 10 previously confirmed *P. jirovecii* qPCR-negative and 9 previously confirmed *P. jirovecii* qPCR-positive BALs. Those 10 assays consisted of 5 in-house assays and 5 commercial kits and included 3 RT-qPCR (1 commercial and 2 in-house) and 7 qPCR assays (4 commercial and 3 in-house)

**Results:** from 20 qPCR assays (16 qPCR amplifying DNA only and 4 RT-qPCR assays amplifying Whole nucleic acids (WNA, i.e DNA+RNA) were obtained from 16 laboratories across eight countries (including seven European countries and one Australian). The different targets evaluated were mtSSU (*n* = 2), mtLSU (*n* = 16), MSG (*n* = 1) and beta-tubulin (Tub, *n* = 1). The inter-laboratory analytical sensitivity was 100% at 1:1 genomic load, 95% at 1:100, and 82.5% at 1:1000. Analytical specificity was 100% for all assays but yielded a false-positive test (95% specificity). For both evaluations and for all genomic loads, testing whole nucleic acid (RNA plus DNA) using reverse-transcriptase RT-qPCR was superior to qPCR (p≤0.001) testing DNA only. (Figure 1 and Figure 2). The target gene mitochondrial small sub-unit (mtSSU) was significantly more sensitive than the mitochondrial large subunit, the major surface glycoprotein, or the beta-tubulin (Figure 1 and Figure 2).

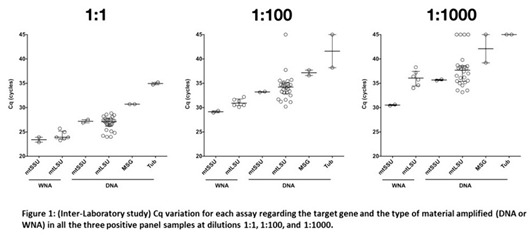

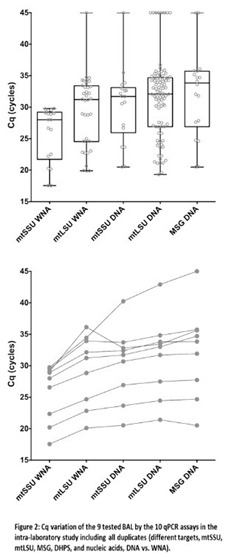





**Conclusion:** Thus, RT-qPCR targeting the mtSSU gene had the best sensitivity and could serve as a basis for standardizing the *P. jirovecii* load, which is essential if qPCR is to be incorporated into clinical care pathways.

### P161. Identification of Cyp51A-mediated triazole resistance in Aspergillus fumigatus using pyrosequencing: an audit of UK National Aspergillosis Centre cases

BrownL.^1^Rautemaa-RichardsonR.^1^,^2^,^3^Novak-FrazerL.^1^,^3^HillS.^1^,^3^HassanD.^3^RichardsonM.D.^1^,^3^1Division of Infection, Immunity and Respiratory Medicine, University of Manchester, Manchester, United Kingdom2National Aspergillosis Centre, Manchester University NHS Foundation Trust, Manchester, United Kingdom3Mycology Reference Centre, Excellence Center For Medical Mycology (ecmm), Wythenshawe Hospital, Manchester University NHS Foundation Trust, Manchester, United Kingdom

**Objectives:** Globally, triazole resistance rates are rising. Azole-naïve patients acquiring an azole-resistant isolate from the environment, or because of long courses of azole treatment is a growing concern. TR34/L98H within the *cyp51A* gene of *A. fumigatus* leads to pan-triazole resistance and is the most common mutation found in environmental isolates. Other mutations in this gene have also been associated with various patterns of azole resistance. Failure of microbiological testing to detect resistance delays the initiation of appropriate antifungal therapy and negatively impacts the clinical outcomes of patients with aspergillosis. Therefore, empiric combination therapies are increasingly used which has significant stewardship implications. We compared the performance of high-volume culture (HVC) and *Aspergillus fumigatus* PCR combined with pyrosequencing (AfPCR-Pyro) for the detection of triazole resistance in aspergillosis patients.

**Methods:** We performed a retrospective audit over a 27-month period (between January 2017 and March 2019) at the Mycology Reference Centre Manchester (MRCM) and the UK National Aspergillosis Centre (NAC), UK. Respiratory samples collected from patients with chronic pulmonary aspergillosis (CPA) or allergic bronchopulmonary aspergillosis (ABPA) and analysed by both HVC and AfPCR-Pyro were included in the study. Hospital and laboratory databases were searched for patient history, laboratory findings and if the pyrosequencing result triggered a change in antifungal treatment.

**Results:** Three hundred and forty-nine samples from 162 patients were analysed for susceptibility using AfPCR-Pyro. of these 28% (96/349) were positive for *A. fumigatus* by HVC while 68% (236/349) were reported with no growth after 14 days. The remaining 3% (9/349) of samples were positive for other *Aspergillus* spp, but excluded from further analysis as pyrosequencing is limited to *A. fumigatus*. Resistance was found by pyrosequencing in 29% (99/340) of samples compared to 15% (52/340) by HVC. Resistance was found by both culture and pyrosequencing in 9% (30/340) of cases, with agreement in susceptibility profiles between the two methods in 66% (63/96) of cases. Resistance was identified by pyrosequencing in 23% (54/236) of culture negative samples. If pyrosequencing had not been used in these patients, 46 resistant cases would have been missed. Pyrosequencing identified the environmental TR34/L98H mutation in 20% (69/340) of samples. Importantly, resistance was recognised by pyrosequencing before HVC results in 17% (57/340) of cases. A change in antifungal therapy was initiated in 19% of cases (58/299) due to pyrosequencing results.

**Conclusion:** Compared to HVC, AfPCR-Pyro has a higher sensitivity for detecting triazole resistance. The technology allows for prompt recognition of resistant organisms and the selection of appropriate antifungal treatment. Increased availability of pyrosequencing would likely lead to improved patient outcomes and better antifungal stewardship.

### P162. Evaluation of two mass spectrometry platforms, the Bruker MALDI Biotyper and the MSI online application, for the identification of clinically important moulds

KristensenL.^1^GertsenJ.B.^1^PetersenT.M.^1^HandbergK.^1^NormandA.C.^2^HendrickxM.^3^Nørskov-LauritsenN.^1^1Department of Clinical Microbiology, Aarhus University Hospital, Aarhus N, Denmark2Ap-hp, Groupe Hospitalier Pitié-salpêtrière, Service de Parasitologie Mycologie, Paris, France3Service of Mycology and Aerobiology, Bccm/ihem Fungal Collection, Sciensano, Brussels, Belgium

**Objectives:** The matrix-assisted laser desorption ionisation time-of-flight mass spectrometry (MALDI-TOF MS) method has emerged as a rapid identification method of filamentous fungi. The aim of this study was to evaluate the performance of the Bruker MALDI Biotyper and the recently introduced MSI (mass spectrometry identification) platform to identify a collection of moulds, followed by an evaluation in the clinical setting.

**Methods:** Thirty-four well-characterised mould isolates (18 species from 10 genera) were selected from our collection of fungal strains. For the subsequent evaluation, 75 clinical isolates were prospectively collected. *Aspergillus fumigatus* and *Aspergillus flavus* with typical morphology were excluded. The isolates were cultured 2-3 days on Sabouraud glucose agar (Oxoid). Prior to MS analysis, a simple ethanol-formic acid-acetonitrile inactivation/extraction procedure was used. Acquisition of mass spectra was performed with the Bruker MALDI Biotyper, software v. 3.1. Data analysis was performed using the general Bruker library (MBT 7311 MSP) combined with the Bruker Fungi Library 1.0, and with the MSI online library after uploading of spectra. In the initial assessment, identification thresholds of 2.0 and 1.7 for species and genus, respectively, were used. The data were compared to a species identification threshold of 1.7. Based on these results the 1.7 threshold for species identification was applied in the subsequent evaluation.

For MSI online identification, a species identification threshold of 20 was used. Identification by sequencing of internal transcribed spacer (ITS2) was used as gold standard.

**Results:** Initial evaluation

ITS sequence identified 25 isolates to the species level and nine isolates to the species complex level (insufficient separation of closely related species).

Bruker (species threshold 2.0): 22 of 34 isolates (65%) were correctly identified to the species or species complex level, six isolates (18%) were correctly identified to the genus level, six isolates (18%) were not identified.

Bruker (species threshold 1.7): 28 of 34 isolates (82%) were correctly identified to the species or species complex level while six isolates (18%) were not identified.

MSI: 31 of 34 isolates (91%) were correctly identified to the species or species complex level. One *Fusarium oxysporum* was misidentified as *Fusarium acutatum*, two isolates (6%) were not identified. Clinical evaluation

The clinical isolates comprised 19 species from 11 genera.

ITS sequence identified 55 isolates to the species level and 20 isolates to the species complex level.

Bruker: 60 of 75 isolates (80%) were correctly identified to the species or species complex level. Fifteen isolates (20%) were not identified.

MSI: 70/75 isolates (93%) were correctly identified to the species or species complex level. Five isolates (7%) were not identified.

**Conclusion:** Both the Bruker library (species identification threshold 1.7) and the MSI platform were accurate and reliable in the initial evaluation. The results were confirmed in the clinical setting, however, compared to Bruker the MSI library obtained the highest degree of identification to the species or species complex level (93%) with a considerably lower number of unidentified isolates.

### P163. Rapid identification of a rare fungi: A skillful mycologist against the state of the art methods

SeethC. GronforsAxelovK. GiengUllbergM.ÖzenciV.Clinical Microbiology, Karolinska University Hospital, karolinska Institutet, Stockholm, Sweden

**Case Report:** Modern diagnostics methods in clinical mycology have become widely available. However, it is important to acknowledge that these methods cannot replace the conventional methods requiring theoretical knowledge and hands on experience. The patient was 52 y old, female with a history of cystic fibrosis. Previous sputum samples showed growth of Staphylococcus aureus and Pseudomonas aeruginosa. On 16 April 2019 the patient contacted cystic fibrosis clinic and presented decreased lung capacity. The Forced expiratory volume in one second (FEV1) was 53% expected value. Sputum sample was taken for both bacterial and fungal culture. Bacterial cultures showed growth of Pseudomonas aeruginosa on 17 April 2019. Growth of mold was observed after 6 days on Sabouraud agar at 30 C. Microscopy from agar plates showed hyphi without septa. In combination with the presence of abundant and relatively rapid growth the preliminary laboratory diagnosis was reported as Zygomycetes spp. on 23 April 2019. One day later new microscopy from the plates showed the presence of spherical-, multi-spored- sporangia without columellae. The laboratory technician who had never seen a similar morphology reported the isolate preliminary as *Mortierella* spp. The report was solely based on her theoretical knowledge in combination with clinical experience. The isolate could not be diagnosed by repetitive MALDI-TOF MS tests and was sent to internal transcribed spacer (ITS) sequencing. The results from the ITS sequencing showed that the isolate was Umbelopsis isabellina (99%) on 2 May 2019. The literature search showed that Umbelopsis isabellina is used as synonym for *Mortierella* spp. Mortierella spp. is a mold belonging to the order Mortierellales according to the most recent taxonomy and is considered a pathogen solely of animals. To our knowledge, there has been only one other case report describing an invasive disease in an immunocompromised patient with Mortierella wolfii, isolated from the liver biopsy and blood culture (Layios et al., 2014). In that case the isolate was identified by ITS sequencing. Therefore the present case is the first describing the preliminary identification of *Mortierella* spp. with a conventional method. It is important to note that the conventional method could identify *Mortierella* spp. seven days earlier than ITS sequencing. It is still not clear if the present case with *Mortierella* spp. is colonization or an infection. The patient is currently being followed up clinically and further lower respiratory samples will be taken up. In the laboratory it feels comfortable to have access to both conventional and modem diagnostic methods. The present study describes the importance of hands on experience in diagnosis of rare fungal patogens.

### P164. An inter-laboratory multi-centre evaluation of the performance of Mucorales PCR assays when testing serum specimens: A study by the ISHAM Fungal PCR Initiative and the ModiMucor study group - FPCRI Mucor Laboratory Working group (A. Alanio, M. Cogliati, S. Fuchs, F. Hagen, C. Halliday, R. Hare, C. Klaassen, M. Lackner M. Lengerova, W. Posch, B. Sendid, J. Springer, B. Willinger) - ModiMucor study group (F. Botterel, M. E. Bougnoux, S. Bretagne, C. Damiani, F. Dalle, J. Denis, M. Gitts-Muselli, X. Iriart, F. Morio, P. Poirier, E. Scherer)

RocchiS.^1^MengoliC.^2^WhiteP.^3^BarnesR.^4^DonnellyJ.P.^5^LoefflerJ.^6^MillonL.^1^1Parasitology - Mycology - Umr6249 Cnrs Chrono-environnement, University Hospital Besançon, Besançon, France2Molecular Medicine, University of Padova, Padova, Italy3Microbiology, Public Health Wales Microbiology, Cardiff, United Kingdom4Medical Microbiology and Infectious Diseases, Cardiff University School of Medicine, Cardiff, United Kingdom5Division of Infectious Diseases, San Antonio Center for Medical Mycology, San Antonio, United States of America6Department of Internal Medicine Ii, WÜ4i, University Hospital Wuerzburg, Wuerzburg, Germany

**Objectives:** Real-time qPCR detection of Mucorales DNA in serum, plasma and BAL fluid has been shown to be sensitive and early tool for diagnosing mucormycosis. Several qPCR assays have been developed, but little comparison and no standardization is available. In 2016, the Mucorales Laboratory Working Group of the ISHAM Fungal PCR Initiative (FPCRI) organised inter-laboratory evaluations of Mucorales PCR assays currently in use, with the objectives of: 1) determining the uniformity of qualitative detection (positive/negative) and 2) assessing qPCR performance. Participants were members of the FPCRI, laboratories involved in the ModiMucor* study and three other French laboratories which have already implemented this tool to diagnose mucormycosis.

**Methods:** For the 1^st^ panel (A), four sera (2ml/sample) were inoculated with genomic DNA (between 27 to 116 pg of DNA/mL of serum) from four different mucorales species (*Rhizomucor pusillus*, *Lichtheimia corymbifera, Cunninghamella bertholetiae*, *Rhizopus oryzae*). For the 2^nd^ panel (B), six sera (2 ml/sample) were inoculated with three concentrations of *R. pusillus* and *L. corymbifera* equivalent to one, 10 and 100 genomes/mL of serum. For each panel, a negative control serum was also sent. Twenty laboratories analysed the panel A (10 FPCRI, two Modimucor/FPCRI, seven Modimucor, three others French laboratories) and 22 laboratories analysed the panel B (two more FPCRI laboratories). All participants were requested to use their own DNA extraction methodology. The 12 French participants used the same qPCR technique (combination of three qPCR assays targeting the four most frequent genera: *Lichtheimia*, *Mucor, Rhizopus*, *Rhizomucor)* (1). For panel B, only quantitative PCR results were analysed (two techniques that used conventional PCR were excluded). Twenty-two laboratories returned multiple datasets, leading to 26 different datasets for analysis: - 16 datasets using a single technique (1) - 10 datasets using other techniques (Four Pathonostics MucorGenius®, Six "in-house" assays, (2-5), three of which were not previously described).

**Results:** For panel A, all datasets were negative when testing the negative control serum. Conversely, 85-90% of the sera inoculated with “common” Mucorales (*R. pusillus, R. or* yzae, *L. corymbifera*) were positive. Detection of sera containing *C. bertholetiae* DNA was lower (12%). For panel B, the probability of qPCR positivity increased predictably with DNA quantity, generating a mean PCR efficiency of 0.85. Detection of *Lichtheimia* DNA was optimal, irrespective of the technique. Regarding the qPCR systems, Millon et al. and MucorGenius® techniques generated greater positivity rates, with earlier Cq values.

**Conclusion:** Despite the diversity of techniques, Mucorales DNA detection in sera was very reproducible with little inter-laboratory variability providing support for including Mucorales PCR in the EORTC/MSG definitions of invasive fungal disease and for its use in the clinical diagnosis of mucormycosis ModiMucor*: French prospective multicenter study for evaluation of Mucorales PCR (PHRC (Projet Hospitalier de Recherche Clinique) national-Modimucor 2014-A00580-47.) **References** 1- Millon et al. CID 2013 2- Springer et al. J. Med. Microbiol. 2016 3- Lengerova et al. J Clin Microbiol 2014 4- Machouart et al. J Clin Microbiol 2006 5- Hrncirova K., J Clin Microbiol 2010

### P165. Comparison of a point-of-care (PreventID®) with the DermaGenius® Nail real-time PCR kit for the detection of dermatophytes species in nail samples

GaajetaanG.KampermannT.TegelenD. VanDingemansG.PathoNostics, Maastricht, Netherlands

**Objectives:** Compare the performance of a point-of-care test for the detection of dermatophyte fungi (PreventID®) with the DermaGenius® 2.0 Nail real-time multiplex PCR assay on a set of clinical nails. Both methods are compared to microscopy as the gold standard method.

**Methods:** Left over nail samples were split into different parts. The culture and microscopy of all nail samples was performed by an external clinical microbiology laboratory. One part of the nail sample was used for DNA-extraction with the PathoNostics Extraction kit and identification of the pathogen was enabled by using the DermaGenius® 2.0 Nail real-time multiplex PCR. Finally, an immunochromatographic poin-of-care test (PreventID® Dermatophyte) was applied to determine the presence of dermatophyte-derived antigens in the nails.

**Results:** Microscopy of the nails was considered as the gold standard. The DermaGenius® 2.0 Nail real-time multiplex PCR was able to detect all positive samples, resulting in a sensitivity of 100%. Specificity was however lower (83%) due to detection of dermatophyte DNA in microscopy-negative samples. Interestingly, these samples were culture-positive. Culture resulted both in a reduced specificity (67%) and sensitivity (57%). Both the sensitivity (79%) and specificity (83%) for the PreventID® Dermatophyte test were promising. Although the PCR assay is sensitive, the clinical relevance is still a matter of debate. High Ct-values indicate limited amounts of DNA and do not always represent a clear infection. Interestingly, the Ct-values for samples which were negative with the PreventID® test were between 26-33 with the DermaGenius® real-time PCR. In addition, readability of the test strips was limited in some samples while Ct-values of 22-28 with the DermaGenius® PCR was obtained, indicating a high fungal load. These samples were also positive with microscopy.

**Conclusion:** Microscopy and the DermaGenius® 2.0 Nail real-time PCR showed a good correlation and are useful for rapid identification of dermatophyte infection. For specific pathogen identification, the DermaGenius® PCR can be used. Culture can result in false negatives and in combination with a long turnaround time, it has limited value in routine diagnostics. The use of an immunochromatographic point-of-care test is uncommon in dermatophyte diagnostics but was included in this study to determine its suitability. Although the PreventID® Dermatophyte test results in a sensitivity of 79%, some dermatophyte infections with low Ct-values were missed. The difference in sensitivity could be explained by the fact that the PreventID® test is using a protein target while the DermaGenius® PCR detects DNA, which is normally not influenced by treatment. But more important is that the interpretation of the PreventID® test is based on visual inspection which makes the test less reliable. The PreventID® should be compared in a larger sample set to determine suitability for routine dermatophyte diagnostics. Currently, microscopy and the DermaGenius® 2.0 Nail real-time PCR seem to be the most reliable and suitable methods for diagnosis of dermatophyte infections in nails.

### P166. Polymerase chain reaction on respiratory tract specimens of immunocompromised patients to diagnose Pneumocystosis - A systematic review

BrownL.^1^Rautemaa-RichardsonR.^1^,^2^RowbothamE.^1^ChenS.^3^JonesB.^4^,^4^1Division of Infection, Immunity and Respiratory Medicine, University of Manchester, Manchester, United Kingdom2Mycology Reference Centre, Excellence Center For Medical Mycology (ecmm), Wythenshawe Hospital, Manchester University NHS Foundation Trust, Manchester, United Kingdom3Medicine, The University of Sydney, Camperdown, New South Wales, Australia4NHS Greater Glasgow and Clyde Health Board, Glasgow, United Kingdom5Institute of Infection Immunity & Inflammation, University of Glasgow, Glasgow, United Kingdom

**Objectives:** *Pneumocystis jirovecii* pneumonia (PCP) is a severe, life-threatening infection affecting immunocompromised patients. The highest rates are seen in those with HIV infection, but other groups are increasingly affected such as solid organ recipients and those receiving long-term corticosteroid therapy. Polymerase Chain Reaction (PCR) is highly sensitive for the detection of *P. jirovecii* when compared to microscopy, but may not differentiate between infection and colonisation. The Fungal PCR Initiative group (FPCRI) working group of the international Society for Human and Animal Mycology (ISHAM) aims to develop international standards for *P. jirovecii* PCR. A systematic review to determine the diagnostic accuracy of PCR testing in respiratory tract specimens for PCP was undertaken.

**Methods:** Following protocol registration with PROSPERO, a systematic review of the literature was performed using PubMed, Scopus, Embase and Cochrane without applying any language or date limits. Studies where PCR techniques were used on respiratory specimens for the diagnosis of PCP in humans were included if they (i) compared the PCR test results with the diagnosis made using standard laboratory methods and clinical presentation; (ii) sufficient information was provided to assess the strength of diagnosis; (iii) results were reported as false- positive, true-positive, false-negative and true-negative; and (iv) evaluation of the test(s) was performed in prospective cohorts of patient populations, defined as groups of individuals at high risk for PCP. Where relevant, the diagnostic performance of PCR techniques to the beta-d-glucan (BDG) assay, was compared. Breakpoint analyses for differentiating colonisation from infection will also be investigated.

**Results:** A total of 2843 unique records were identified. Abstracts of these records were sent out to six pairs of reviewers to be assessed against the inclusion and exclusion criteria. The reviewers were in agreement on 2371 abstracts; 472 abstracts with reviewer discrepancies were sent out for a third review. After screening, 2509 records were excluded and 334 full-text articles are now being assessed for eligibility by pairs of reviewers. Papers meeting the inclusion criteria after reading in full will be included in the meta-analysis to be carried out by the group. Risk of bias will be assessed using the QUADAS-2 tool.

**Conclusion:** This systematic review will provide essential information about the diagnostic accuracy of PCR tests in respiratory tract specimens for PCP required for the development of a standardised method for *Pneumocystis* PCR which will be later validated in clinical trials.

### P167. Evaluation of the LDBio Aspergillus ICT lateral flow assay as a point-of-care test for serological antibody detection in allergic bronchopulmonary aspergillosis

HunterE.^1^RichardsonM.D.^1^,^2^DenningD.^1^,^3^1Division of Infection, Immunity, and Respiratory Medicine, University of Manchester, Manchester, United Kingdom2Mycology Reference Centre, Excellence Center For Medical Mycology (ecmm), Wythenshawe Hospital, Manchester University NHS Foundation Trust, Manchester, United Kingdom3National Aspergillosis Centre, Manchester University NHS Foundation Trust, Manchester, United Kingdom

**Objectives:** Allergic bronchopulmonary aspergillosis (ABPA) is an immunological pulmonary disorder complicating asthma, as a result of hypersensitization to *Aspergillus* species (predominantly *A. fumigatus*). It is estimated to affect 2.5% of adults with asthma, which accounts for approximately 4.8 million patients worldwide. Early recognition and diagnosis of ABPA is critical to improve patient symptoms and prevent or delay progression of bronchiectasis and development of chronic pulmonary aspergillosis (CPA). It is especially important to make the distinction between asthma and *Aspergillus* sensitization. LDBio Diagnostics has recently commercialized a lateral flow assay (*Aspergillus* ICT) that detects *Aspergillus* antibodies less than 30 minutes, requiring minimal laboratory equipment. Herein, we evaluate this assay for specific diagnosis of ABPA as compared to asthma controls.

**Methods:** A retrospective, ongoing study was performed using 94 patient sera collected at the National Aspergillosis Centre (NAC, Manchester). Patient sera were selected based on a clinical diagnosis of ABPA and classified as ‘proven’ (*n* = 70) or ‘probable’ (*n* = 24). Control sera (*n* = 93) were obtained from the Manchester Allergy, Respiratory and Thoracic Surgery (ManARTS) biobank. LDBio *Aspergillus* ICT was performed as per manufacturer’s instruction. Results were read at intervals, and determined after 20 to 30 minutes as recommended (any line at the “T” marker considered positive), both manually and using the Qiagen ESEQuant LR3 lateral flow reader. Serological *Aspergillus*-specific IgG and IgE, and total IgE titres were measured by ImmunoCAP.

**Results:** We found the LDBIO *Aspergillus* ICT to have a sensitivity of 88.6% across for 70 proven ABPA cases, and 89.4% for all cases (‘proven’ and ‘probable’) combined. Specificity was 91.4% for 93 control sera (Table 1). A comparison of ICT result with *Aspergillus*-specific IgG and IgE titres showed no evident immunoglobulin isotype bias (Figure 1). Using digital measurements obtained using the Qiagen LR3, we found no correlation between ImmunoCAP *Aspergillus*-specific IgE level and *Aspergillus* ICT test line intensity. Finally, assessment of 3 assay lots using 30 samples showed perfect agreement between lots when tests were read visually. 

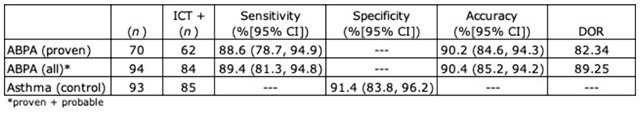

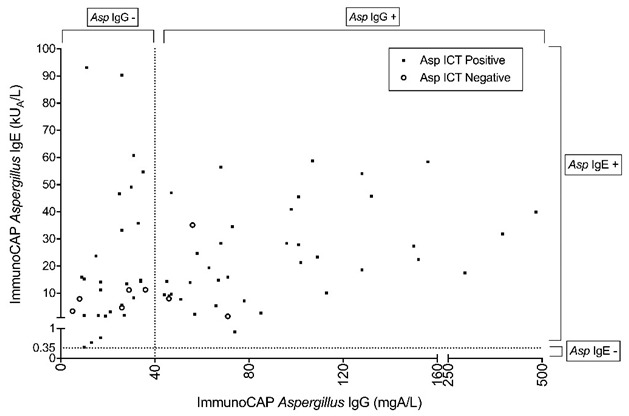





**Conclusion:** The LDBio *Aspergillus* ICT lateral flow assay exhibits good sensitivity for serological screening for ABPA. The assay is easy to perform and interpret, using minimal equipment and resources. As such, it provides a valuable point-of-care screening resource to rapidly distinguish between asthma and *Aspergillus* sensitization.

### P168. Effect of patient immunodeficiencies on the diagnostic performance of serological assays to detect Aspergillus-specific antibodies in chronic pulmonary aspergillosis

HunterE.^1^HarrisC.^2^RichardsonM.D.^1^,^3^DenningD.^1^,^2^1Division of Infection, Immunity, and Respiratory Medicine, University of Manchester, Manchester, United Kingdom2National Aspergillosis Centre, Manchester University NHS Foundation Trust, Manchester, United Kingdom3Mycology Reference Centre, Excellence Center For Medical Mycology (ecmm), Wythenshawe Hospital, Manchester University NHS Foundation Trust, Manchester, United Kingdom

**Objectives:** Prevalence of chronic pulmonary aspergillosis (CPA) is estimated at approximately 3 million cases worldwide, and serological detection of *Aspergillus*-specific antibody is a critical component in the CPA diagnostic pathway. Some patients with CPA, however, have subtle immune deficits, including low CD4 or CD19 (B cell) counts, mannose binding lectin deficiency and/or low gamma IFN or IL12 production. It is possible these deficits contribute to poor *Aspergillus* antibody production to one or more ordinarily immunodominant antigens, and may lead to false negative results by diagnostic assays used to detect *Aspergillus-*specific antibodies. Herein, we examine the potential effect of patient immunodeficiencies on the accuracy and performance of CPA serological diagnostics.

**Methods:** We analyzed patient data, as available, from 167 cases of clinically confirmed CPA that were previously evaluated by ImmunoCAP *Aspergillus*-specific IgG EIA and LDBio *Aspergillus* ICT lateral flow assay, to determine the presence of deficiencies in the following: mannose binding lectin (MBL), IgG, IgA, IgM, IL12 production, IL17 production, and/or cell markers (CD4, CD19, CD56). We compared patient data with test results (ImmunoCAP, ICT) as well as for ImmunoCAP ‘seronegative’ patients -- defined as cases for which ImmunoCAP *Aspergillus* IgG was consistently and repeatedly positive for up to 3 years of patient history. Seronegative cases accounted for 12.6% of all cases evaluated (*n* = 21). ‘Seropositive’ was defined as all other CPA cases (*n* = 146).

**Results:** We found the rate of false negatives by ImmunoCAP *Aspergillus* IgG EIA to be more prevalent in patients with low levels of IgG, IgA, or IgM. The performance LDBio *Aspergillus* ICT appears to relatively unaffected by these (Figure 1). Similar findings were observed for IFN gamma production. Additionally, though not significant, we observed a trend in lower levels of CD4, CD19, or CD56 cell markers amongst seronegative CPA patients as compared to seropositive CPA patients, according to ImmunoCAP results (Figure 2). 

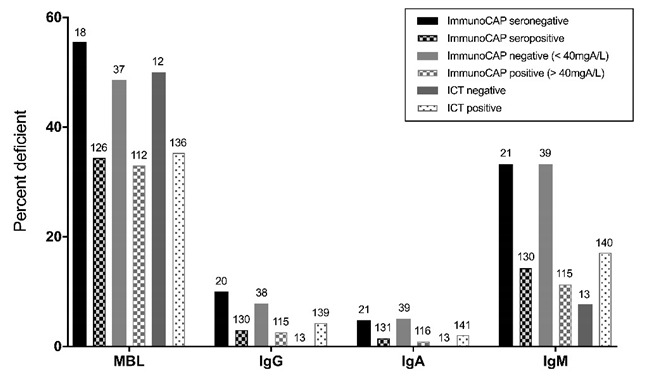

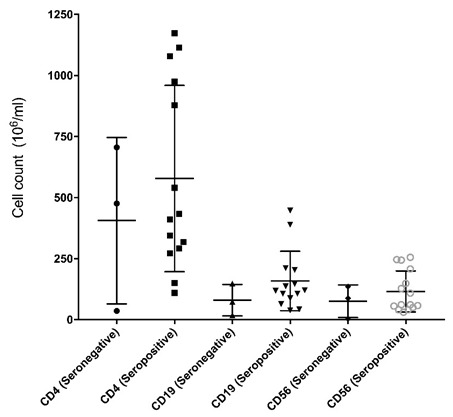





**Conclusion:** In select cases of CPA, ImmunoCAP EIA yields a false negative result, making serological diagnosis difficult and requiring increased diagnostic sensitivity. Our previous findings demonstrated that LDBio *Aspergillus* ICT show greatly improved sensitivity and ability to detect *Aspergillus*-specific antibody in patient sera, especially for ImmunoCAP IgG-negative (‘seronegative’) cases. Our analysis of patient immunodeficiencies reveals that ImmunoCAP false negatives are more prevalent in patients with certain immunodeficiencies, and that diagnostic performance of ImmunoCAP *Aspergillus-*specific IgG EIA may be more adversely affected by these patient conditions, as compared to LDBio *Aspergillus* ICT.

### P169. The diagnostic value of a molecular assay for Fusarium infections recently introduced in the Danish national reference laboratory

HareR.^1^Abou-ChakraN.^1^DatcuR.^1^ArendrupM.C.^1^,^2^,^3^1Unit of Mycology, Statens Serum Institut, Copenhagen, Denmark2University of Copenhagen, Dept. of Clinical Medicine, Copenhagen, Denmark3Department Og Clinical Microbiology, Rigshospitalet, Copenhagen, Denmark

**Objectives:** *Fusarium* keratitis and other infections remain difficult to diagnose even in the modern mycology laboratory. Molecular methods have enabled genus and species specific detection independent of culture and have improved diagnostic speed, sensitivity and accuracy of fungal infections. A specific diagnostic assay for *Fusarium* infections has been introduced in the Danish mycology reference laboratory and this study sought to evaluate the clinical performance.

**Methods:** Clinical samples from patients suspected for fusariosis were subjected to *Fusarium* PCR (detecting *Fusarium solani*, *Fusarium dimerum* and *Fusarium oxysporum*) during the period October-2017 to April-2019. The real-time multiplex *Fusarium* PCR assay using Taqman probes was slightly modified from that described by Salehi et al. 2016 (Pubmed ID 27605714). Patient samples were DNA extracted using the Qiacube (Qiagen, Denmark) standard protocol for blood and tissue samples, with up to 200 µL input volume and elution in 100 µL. Bronchoalveolar lavage samples were processed on the NucliSENS easyMAG (Biomérieux, Denmark) DNA extraction platform with up to 1 mL input volume and elution in 60 µL. Specimens were subjected to PCR in combination with culture and Blankophor microscopy, except for one patient (PCR only) due to insufficient amount of material. PCR was performed on the Applied Biosystems 7500 or the Quantstudio 5 thermocyclers (Thermo Fisher Scientific, Denmark). Positive and negative template controls were applied but not PCR inhibition or DNA extraction controls. PCR results were compared for each patient to our other laboratory results and to results from the Danish Microbiological Database (MiBa), which captures clinical microbiological results from every laboratory in the entire country.

**Results:** In total, 71 samples from 34 patients, among which 50 samples were from 29 patients with suspected keratitis, were included. Five patients (14.7%) accounted for 19/33 (57.6%) samples positive for *Fusarium* spp. DNA (Table). Four PCR positive patients provided 12/32 (37.5%) samples microscopy positive (septate hyphae) and 10/32 (31.3%) culture positive with *Fusarium* species (Table). The fifth PCR positive patient, was culture negative, but clinically diagnosed with a fungal keratitis and responded to targeted antifungal treatment. The remaining 29 patients provided 38 PCR negative samples. The majority of those patients (18/29) had culture positive samples for other potential culprits (clarification pending) and none were culture and microscopy positive for *Fusarium*. 

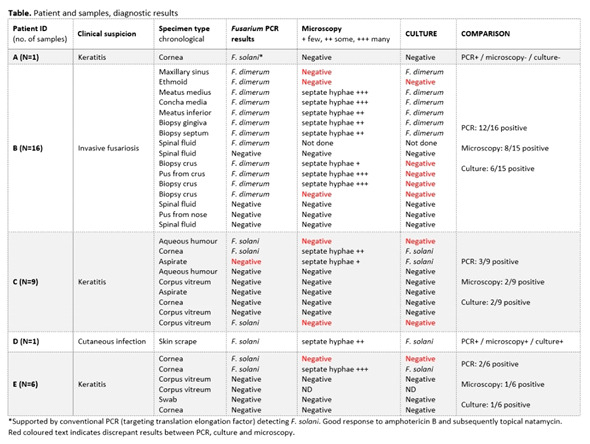



**Conclusion:** The diagnostic platform for *Fusarium* infections has proven accurate to the species level and was applied successfully in three incidents of *Fusarium* keratitis, one invasive fusariosis and one cutaneous infection. These data indicate a superior sensitivity of PCR compared to microscopy and culture. of note, no clinically false positive findings were identified. Still, the specificity needs to be confirmed including a larger sample set. PCR performance may be further improved upon ongoing optimisation of the extraction procedures and quality control as well as introduction of a PCR inhibition control. In conclusion, the *Fusarium* PCR assay appears to be a significant improvement of the overall diagnostic algorithm at this reference laboratory both in speed and accuracy and thus important for those suffering from this uncommon but sight or potentially life-threatening disease.

### P170. rMucorales PCR: a diagnostic improvement at the Danish national reference laboratory

HareR.^1^Abou-ChakraN.^1^DatcuR.^1^ArendrupM.C.^1^,^2^,^3^1Unit of Mycology, Statens Serum Institut, Copenhagen, Denmark2University of Copenhagen, Dept. of Clinical Medicine, Copenhagen, Denmark3Department Og Clinical Microbiology, Rigshospitalet, Copenhagen, Denmark

**Objectives:** Invasive *Mucorales* infections are associated with a poor prognosis for affected patients due to challenging therapeutic management strategies and often inadequate diagnostic tools. Molecular methods are increasingly complementing conventional culturing and microscopy to improve the overall diagnostic performance for invasive *Mucorales* infections. In the Danish mycology reference laboratory, a molecular assay detecting *Mucorales* DNA has been introduced which in a recent EQA panel managed by the *Mucorales* subgroup of the Fungal PCR Initiative scored 100% (7/7 correct). This study sought to evaluate the clinical performance.

**Methods:** Clinical samples from patients suspected for invasive *Mucorales* infections were subjected to two *Mucorales* PCR tests (1: *Rhizopus microsporus*, *Rhizopus arrhizus* and *Mucor* spp. and 2: *Lichtheimia* spp. and *Rhizomucor* spp.) during the study period October-2017 to April-2019. Both real-time multiplex *Mucorales* PCRs (Taqman probes) were slightly modified from those described by Salehi et al. 2016. Patient samples were DNA extracted using the Qiacube (Qiagen, Denmark) standard protocol for blood and tissue samples, with up to 200 µL input volume and elution in 100 µL. Formalin fixed paraffin-embedded tissue samples were processed using Neo-clear (Sigma-Aldrich, Denmark) followed by the Tissue protocol as described above. BALs were processed on the NucliSENS easyMAG (Biomérieux, Denmark) DNA extraction platform with up to 1 mL input volume and elution in 60 µL. PCR was performed on the Applied Biosystems 7500 and since mid-2018 the Quantstudio 5 thermocyclers (Thermo Fisher Scientific, Denmark). Positive and negative template controls were applied but not PCR inhibition or DNA extraction controls. PCR results were compared to culture and Blankophor microscopy whenever possible and to results from the Danish Microbiological Database (MiBa), which captures clinical microbiological results from every laboratory in the entire country.

**Results:** In total, 116 samples from 18 patients were included. Four patients diagnosed with invasive mucormycosis contributed with 91 samples of which 38 were *Mucorales* PCR-positive (41.8%, range 25-100% per patient) (Table). Among those four patients, microscopy revealed pauci-septate hyphae in 31/85 (36.5%) and *Mucorales* species was cultured from 10/81 (12.3%) samples (Table). All patients received targeted treatment and surgical interventions guided by PCR results and all survived. The remaining 14 patients had 25 PCR negative samples, of which 20/21 and 19/19 were also negative by microscopy and culture, respectively. One patient, was microscopy positive with pauci-septate hyphae and by pan-fungal ITS DNA sequencing, *Cunninghamella* spp. DNA was detected, which is not included in the current *Mucorales* tests. 

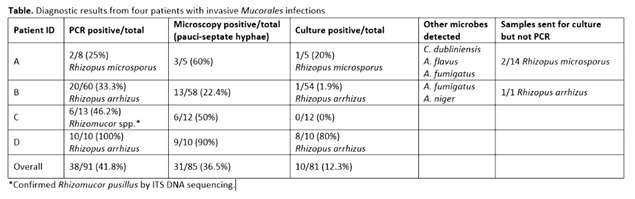



**Conclusion:** Both diagnostic tests for *Mucorales* proved accurate to the genus or species level and with a sensitivity comparable to that for microscopy but significantly superior to that for culture (p<0.001). No clinically false positive findings were reported suggesting a high specificity. Yet, further evaluation including a larger sample set is warranted. PCR performance may be further improved upon ongoing optimisation of extraction procedures and PCR parameters as well as introduction of a PCR inhibition control. In conclusion, the *Mucorales* PCR assay appears to improve the diagnostic armamentarium and may play a pivotal role in managing patients suffering from *Mucorales* infections.

### P171. An evaluation of the analytical specificity of PCR assays for the detection of Candida species – A study by the ISHAM working group, the Fungal PCR initiative

GortonR.^1^WhiteP.^2^LoefflerJ.^3^BarnesR.^4^DonnellyJ.P.^5^CrucianiM.^6^HareR.^7^LintonC.^7^,^8^JohnsonE.^8^Novak-FrazerL.^9^CogliatiM.^10^MengoliC.^11^WillingerB.^12^PoschW.^13^ClancyC.J.^14^MelchersW.^15^LacknerM.^13^HamalP.^16^PaholcsekM.^17^SendidB.^18^NguyenM.H.^14^ArastehfarA.^19^UgojiU.^20^VanderboschB.^15^SelitschB.^12^FuchsS.^13^VyzantidisT.^21^1Infection Sciences, Health Services Laboratories, London, United Kingdom2Microbiology, Public Health Wales Microbiology, Cardiff, United Kingdom3Department of Internal Medicine Ii, WÜ4i, University Hospital Wuerzburg, Wuerzburg, Germany4Medical Microbiology and Infectious Diseases, Cardiff University School of Medicine, Cardiff, United Kingdom5Division of Infectious Diseases, San Antonio Center for Medical Mycology, San Antonio, United States of America6Infectious Diseases, San Bonifacio Hospital, Verona, VERONA, Italy7Unit of Mycology, Statens Serum Institut, Copenhagen, Denmark8Public Health England UK National Mycology Reference Laboratory, Bristol, United Kingdom9Mycology Reference Centre Manchester, Manchester University NHS Foundation Trust, Manchester, United Kingdom10Laboratorio di Micologia Medica, Dipartimento di Scienze Biomediche per la Salute, Universita degli Studi di Milano, Milano, Italy11Molecular Medicine, University of Padova, Padova, Italy12Medical University of Vienna, Vienna, Austria13Division of Hygiene and Medical Microbiology, Medical University of Innsbruck, Innsbruck, Austria14University of Pittsburgh Medical Center, Pittsburgh, United States of America15Medical Microbiology, RadboudUMC, Nijmegen, Netherlands16Department of Microbiology, Palacký University, Faculty of Medicine and Dentistry and University Hospital Olomouc, Olomouc, Czech Republic17Faculty of Medicine, Department of Human Genetics, University of Debrecen, Debrecen, Hungary18Laboratory of Mycology and Parasitology, University Hospital of Lille, Lille Cedex, France19Westerdijk Fungal Biodiversity Institute, Utrecht, Netherlands20The Bourne Laboratory, Royal Holloway University of London, London, United Kingdom21Medical Biopathology-microbiology, Aristotle University of Thessaloniki, Thessaloniki, Greece

**Objectives:** The use of PCR to detect *Candida* in clinical samples has been described for two decades, yet standardization is limited, hampering its widespread application. Following the successful efforts of the Fungal PCR initiative (FPCRI) in standardizing *Aspergillus* PCR the FPCRI has expanded it efforts to standardising the molecular detection of other fungi, including *Candida*. This research describes the efforts of the FPCRI *Candida* Laboratory Working Party to determining the analytical specificity (detection range and cross-reactivity) of *Candida* PCR assays currently used in clinical mycology centres.

**Methods:** A panel of 12 samples containing genomic DNA extracted from nine yeast species (*Candida albicans* (2 concentrations), *Candida glabrata (Nakaseomyces glabrata*) (2 concentrations), *Candida krusei (Pichia krusei*), *Candida parapsilosis*, *Candida tropicalis*, *Candida auris, Clavispora lusitaniae, Meyerozyma guilliermondii* and *Dipodascus geotrichum)* and commercially bought human genomic DNA was distributed to 11 centres. The recipients were requested to perform *Candida* PCR following local protocols. Qualitative results (positive/negative), together with associated C*_T_* values (for real-time PCR) were reported for each sample, along with the assay’s technical details.. True positivity rates (detection of the correct species) and false positivity rates (detection of species not included in the panel, or misidentification as another species in the panel) were calculated for the overall panel and for individual samples. Regression analysis has been performed to identify any technical variables associated with improved analytical specificity.

**Results:** Across 11 datasets ten different PCR protocols were utilized. At the high DNA concentration detection of the five main causes of invasive candidiasis (*C. albicans*, *C. glabrata*, *C. krusei*, *C. parapsilosis*, *C. tropicalis*) was 92.7%, ranging from 100% for *C. parapsilosis* to 90.9% for the other species. Detection of less common yeast pathogens (*C. lusitaniae*, *M. guilliermondii*) was 36.3%. At the lower DNA concentration the detection of *C. albicans* and *C. glabrata* was 72.7% and 54.5%, respectively. For *C. auris*, 63.6% of datasets were negative, but 36.4% generated an incorrect identification. Similarly for *Dipodascus geotrichum*, 45.5% were correctly negative but 45.5% provided an incorrect identification; only one assay identified *Dipodascus geotrichum correctly*. One assay generated a false positive result when testing human DNA. Nine centers used real-time PCR. Analysis of C*_T_* values for the five main *Candida* species indicated variance in the sensitivity of the assays with a mean *Sd* of 5.7 (range 4.3 – 6.9) with higher variance in C*_T_* values observed for *C. parapsilosis, C. tropicalis* or lower concentrations of *C. albicans* and *C. glabrata,* shown in Figure 1 and Table 1.

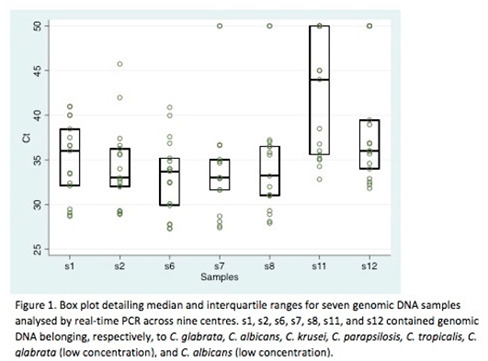

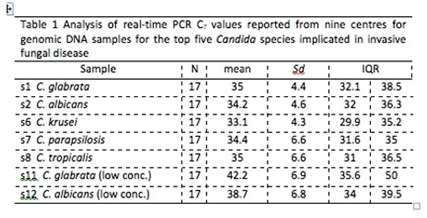





**Conclusion:** The detection of the main *Candida* pathogens that account for >95% of invasive disease is excellent, albeit at lower concentrations detection may be reduced. The detection of less common species is poor. In particular, only one of the 11 assays evaluated was designed to detect and identify *C. auris* but failed to do so which, given the susceptibility profile and infection control concerns associated with *C. auris*, highlights the design limitations of current Candida PCR assays. Preliminary analysis of C*_T_* values for the main *Candida* pathogens indicates differing sensitivity levels across real-time Candida assays.

### P172. An evaluation of human β-globin gene detection to assess the quality of broncho-alveolar lavage and sputum specimens submitted for the diagnosis of Pneumocystis Pneumonia (PcP).

GortonR.^1^WeyE.^2^HusseinM.^1^PatabendigeK.^1^JohnsonG.^3^1Infection Sciences, Health Services Laboratories, London, United Kingdom2Microbiology, Royal Free Hospital Foundation Trust, London, United Kingdom3Scientific Development, OLM diagnostics, Newcastle, United Kingdom

**Objectives:** The use of PCR to diagnose Pneumocystis Pneumonia (PcP) from clinical samples is recommended in recent guidelines for the diagnosis of PcP. One component of the diagnostic pathway, specifically sampling of broncho-alveolar lavage and sputum, is poorly controlled and often the laboratory receives specimen that is not standardised in volume or portion. This research aims to evaluate the use of a human β-globin gene (HgG) PCR from respiratory samples as a method for quality control of specimens, with the hypothesis that adequate levels of HgG present in a specimen is an indicator of a good quality sample,

**Methods:** DNA was extracted from 47 BAL and 18 sputua using the Diasorin Arrow with DNA extraction cartridges. 500μL of BAL concentrate was centrifuged at 8000g for 10 minutes. The supernatant discarded; 50μL of glass beads were added and ribolysed for 45 seconds. The lysate was suspended in 240 μL Diasorin buffer 2 and 10μl proteinase K and incubated at 56°C for 10 minutes. Extraction was performed as per manufacturer’s instructions on the Arrow platform. PCR was performed using the OLM PneumID assay with 6µL template in a total reaction volume of 20µL, following manufacturer’s instructions. PCR was performed on the RotorGeneQ platform. Outliers were determined using the 1.5 x IQR rule.

**Results:** HgG C*_T_* characteristics are presented in Table 1 and Figure 1a) and b).

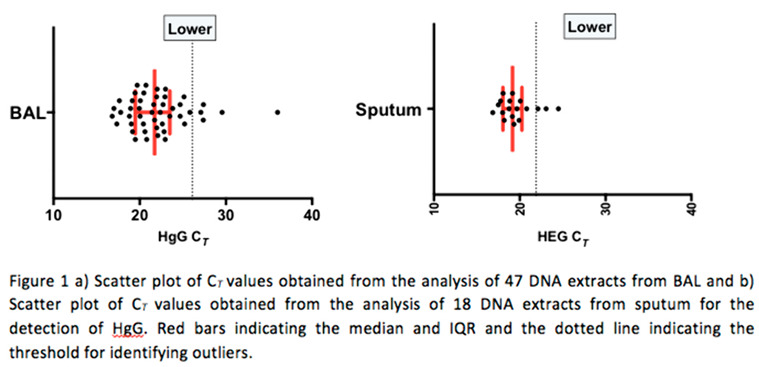

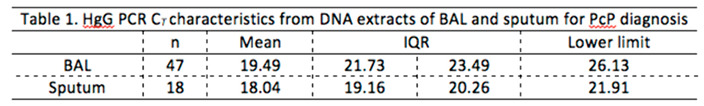





The mean HgG C*_T_* value from BAL was 19.49 (IQR 21.73, 23.49) and the lower threshold was determined to be 26.13. 10.6% (5/47) of the BAL samples had HgG amplification below the threshold. One sample was negative for *P. jirovecii*, two samples were sub-clinical (internal threshold 1E+5) positive for *P. jirovecii* measuring 2.3E+1 and 8.5 E+4 copies per mL. The remaining two samples were positive for *P. jirovecii* measuring 2.1 E+7 and 8.3 E+9 copies per mL. The mean HgG C*_T_* value from sputum was 18.04 (IQR 19.16, 20.26) and the lower threshold was determined to be 21.91. 16.7% (3/18) of the sputum samples had HgG amplification below the threshold. One sample was negative and two samples were positive for *P. jirovecii* at 9 E+5 and 1.9 E+6 copies per mL. From the total dataset of 65 specimens 47.7% (31/65) were positive for *P. jirovecii*. No significant difference (p = 0.38) was observed between the measured copies per mL using the OLM PneumID assay and the FTD Pneumocystis assay indicating that inclusion of the HgG PCR did not competitively inhibit the detection of *P. jirovecii*.

**Conclusion:** This study has demonstrated HgG detection enables the laboratory to quality control respiratory specimens by determining if the specimen contains sufficient host material, which can be used as an indicator of good quality specimen. In this study almost 90% of samples had measured HgG above the established threshold, therefore negative and positive P. jirovecii PCR results could be reported with confidence. Where the HgG is below the acceptable threshold, negative or low positive samples should be interpreted with caution, as results may be derived from poor quality samples, seen for >10% of samples in this study.

### P173. Optimization of Matrix-Assisted Laser Desorption/Ionization Time-Of-Flight Mass Spectrometry for identification of filamentous fungi

SalahH.^1^,^2^KoleckaA.^2^HoubrakenJ.^2^Taj-AldeenS.J.^1^BoekhoutT.^2^,^3^1Department of Laboratory Medicine and Pathology, Division of Microbiology, Hamad medical corporation, Doha, Qatar2Westerdijk fungal biodiversity Institute, Utrecht, Netherlands3Institute of Biodiversity and Ecosystem Dynamics, University of Amsterdam, Amsterdam, Netherlands

**Objectives:** Matrix-Assisted Laser Desorption/Ionization Time-Of-Flight Mass Spectrometry (MALDI-TOF MS) has been widely used in clinical laboratories for routine identification of bacteria and yeast. However, there remain difficulties when applied to filamentous fungi. The standard liquid cultivation method recommended for identification is labour intensive and time-consuming. The aim of this study was to develop a quicker and less laborious method for cultivating filamentous fungi which leads to a reliable identification using Bruker MALDI-TOF MS.

**Methods:** Reference strains of *Aspergillus* section *Fumigati* (*n* = 55) and section *Flavi* (*n* = 104), which were previously identified by molecular methods, were cultivated on top of and in between polycarbonate filters placed on Sabouraud’s dextrose agar (SDA) plate, and by the Bruker standard liquid cultivation method. After 24 hours of incubation at 35°C, mycelia were harvested for ethanol/formic acid protein extraction. Main Spectra Profiles (MSPs) were created and databases were constructed for each cultivation method. These databases were validated by identification of 50 reference strains and 100 clinical strains of *Aspergillus* and *Penicillium* species which were previously characterized by DNA sequencing.

**Results: Growth:** On top of a polycarbonate filter: The strains grew as a monolayer of mycelia with some spores in the middle. In between two polycarbonate filters: The strains grew as a mycelial monolayer without spores. Bruker method: Mycelial growth without sporulation **MSP creation:** 55/55 MSPs of *Aspergillus* section *Fumigati* strains were created by cultivation in between 2 polycarbonate filters, 52/55 by cultivation on top of a polycarbonate filter and 47/55 by Bruker standard method. Out of 104 *Aspergillus* section *Flavi* strains, 94, 92 and 76 MSPs were created by cultivation on top of and in between polycarbonate filters, and Bruker standard method, respectively. **Validation:** Identification of 50 reference strains of *Aspergillus* and *Penicillium* species against in-house databases resulted in 95%, 90% and 62% correct identifications for the strains cultivated on top of a polycarbonate filter, in between 2 polycarbonate filters and by Bruker method, respectively. Identification against Bruker database (BDAL) resulted in 79%, 64% and 61% correct identifications by 1 polycarbonate filter, 2 polycarbonate filters, and Bruker method, respectively. Identification against in-house databases and Bruker database together yielded 87%, 82% and 61% correct identifications by 1 polycarbonate filter, 2 polycarbonate filters, and Bruker method, respectively (Figure 1). 

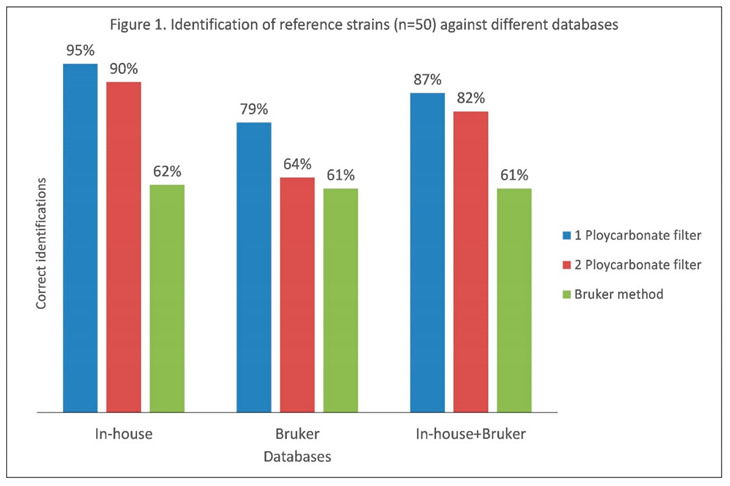



**Identification of clinical strains:** Out of 100 clinical *Aspergillus* and *Penicillium* strains cultivated on top of a polycarbonate filter, 81% were correctly identified against in-house databases of strains grown on top of a polycarbonate filter, 78% with 2 polycarbonate filters and 59% by Bruker commercial database (BDAL) (Figure 2). 

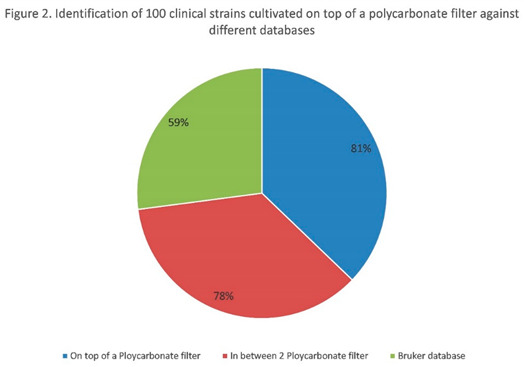



**Savings:** For 50 strains, the cultivation method using polycarbonate filter was 270 minutes quicker and 81 Euros cheaper than Bruker standard method.

**Conclusion:** Cultivation of filamentous fungi on top of a polycarbonate filter showed significantly improved results compared to the Bruker standard liquid cultivation method in terms of correct identification, MSP creation, time consumption, cost, and labour intensity. This can reliably be applied for identification of clinically significant filamentous fungi.

### P174. Interest of Mucorales PCRs in pulmonary samples and comparative evaluation of MucorGenius® assay and in-house Mucorales PCRs

GueganH.^1^IriartX.^2^Robert-GangneuxF.^1^BerryA.^2^BougnouxM.-E.^3^GangneuxJ.P.^1^1Laboratoire De Parasitologie-mycologie, CHU de Rennes, Rennes, France2Laboratoire De Parasitologie-mycologie, CHU de Toulouse, Toulouse, France3Laboratoire De Parasitologie Mycologie, APHP Hôpital Necker-Enfants-Malades, Paris, France

**Objectives:** Mucormycosis is a life-threatening fungal infection whose diagnosis remains challenging due to a lack of reliable diagnostic tests. Clinical and radiological signs are often unspecific. The contribution of histopathological and cultural procedures for early diagnosis is limited. In this context, Mucorales in-house real-time PCRs have been previously shown to be of high interest for a reliable and early diagnosis both in serum and respiratory specimens. A multiplex real-time PCR assay (MucorGenius^®^, Pathonostics, the Netherlands) has been newly marketed to detect the main genera involved in human mucormycosis. The aim of this study was i) to assess the diagnostic performances of MucorGenius® assay in pulmonary samples compared to in-house Mucorales PCRs, and ii) to evaluate the occurence of Mucorales detection among invasive aspergillosis (IA) patients.

**Methods:** We retrospectively included 320 pulmonary samples between January 2012 and May 2019: bronchoalveolar lavage fluids (*n* = 293), tracheal aspirations (*n* = 13), sputum samples (*n* = 5), pleural fluids (*n* = 3), and lung biopsies (*n* = 6) collected from 320 patients with respiratory symptoms at Rennes, Toulouse and Paris-Necker teaching Hospitals (France). Among the 320 patients, 30 patients were classified as a proven mucormycosis based on a positive direct examination and/or a positive culture in the respiratory sample and/or a positive serum mucorales PCR. Fifty-eight patients were proven/probable IA cases according to EORTC-MSG criteria. The 232 remaining patients were considered as having no IA and no mucormycosis (negative group). In-house real-time PCRs consisted in 3 assays targeting respectively *Mucor-Rhizopus spp*, *Lichteimia spp,* and *Rhizomucor spp*. MucorGenius^®^ targeted the same genera, including *Cunninghamella spp*. Amplification was performed according to manufacturer’s instructions using a LC480 (Roche) device.

**Results:** The global concordance of both tests was high, of 97.8% (kappa coefficient = 0.877). Discordant results were mainly related to a higher sensitivity of in-house PCRs compared to MucorGenius^®^ assay (100% (30/30*) versus* 90.0% (27/30). The 3 cases missed by MucorGenius^®^ PCR were related to low fungal burden (mean Cq of in-house PCRs of 39.5±1.4) and were positive for *Mucor-rhizopus* (*n* = 2) and *Rhizomucor* (*n* = 1) with in-house PCRs. The specificity of both tests was similar (98.6% (286/290) *versus* 99.3% (288/290). No Mucorales DNA was detected in IA patients using in-house PCRs whereas one was found positive with MucorGenius^®^ assay, without other biological evidence for mucormycosis. of the 4 positive-PCR samples in the negative group, one was probably a true positive result, from a real mucormycosis episod missed at the time of diagnosis (hematological malignancy, imaging compatible with IA during aplasia, early death under voriconazole treatment). The 3 other could be associated with fungal colonization.

**Conclusion:** The commercial MucorGenius^®^ assay provides the possibility to detect in one well the main mucorales responsible for human mucormycosis, and then could reduce the diagnostic delay and improve the outcome. MucorGenius^®^ assay is an easy to use marketed kit including internal quality control, that shows good diagnostic performances with 97.8% of concordance compared to in-house PCRs, missing 3 low fungal burden cases.

### P176. Development and Validation of an In-House Candida auris database in MALDI TOF MS to improve the yeast identification.

Ceballos-GarzonA.^1^,^2^GiraldoC.M. Parra^2^AmadoD.^2^VelezN.^2^RodríguezC.^3^1Department of Parasitology and Medical Mycology of The University of Nantes., Universite de Nantes, Nantes, France2Microbiology, Pontificia Universidad Javeriana, Bogotá, Colombia3Bruker Mexicana, Ciudad de México, Mexico

**Objectives:** to construct an In-house Colombian database to improve *Candida auris* identification by MALDI-TOF MS.

**Methods:** 30 clinical isolates of *C. auris* were used to construct an *in-house* data base. For each isolation, protein extraction was performed using the formic acid/ethanol method according to the Bruker Daltonics protocol. Minimum 20 mass spectra of each one was obtained. The library was named “Candida auris Colombia”. For its validation 300 Colombian isolates of *C. auris* were used, comparing the results between the BDAL (Bruker) and Candida auris Colombia (in house) libraries. Finally, Cohen’s kappa index was used to compare the results of the classification with both libraries.

**Results:** From 30 clinical isolates, 770 mass spectra were obtained for the construction of the database. The validation was carried out with 300 isolates to compare the identification of results in the BDAL and Candida auris Colombia libraries (Figure 1). Our library allowed us to obtain an identification result for 100% of the evaluated isolates and improve significantly the scores obtained, which shows a better performance compared to the library present in the Bruker spectrometer. 

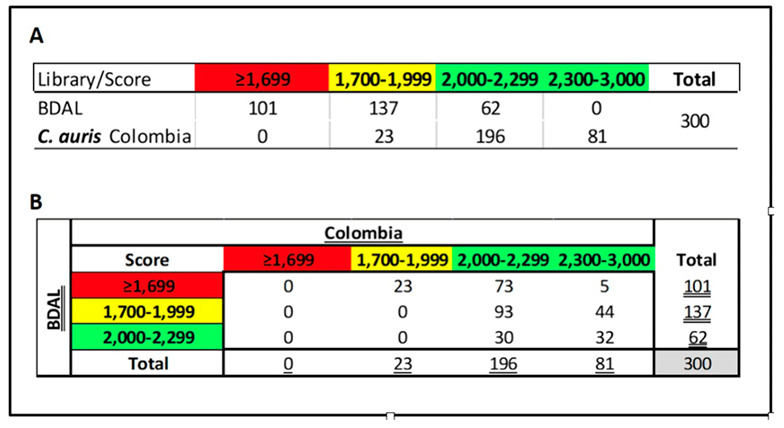



**Figure 1** BDAL and *Candida auris* Colombia libraries identification results. **A** Total results and **B** Matrix of concordance identification from BDAL and Colombia databases. The MALDI-TOF MS results according to the manufacturer’s technical specifications as follows: Correct genus and species identification (>2,300), correct genus and probably species identification (≥2.0), correct genus identification and (1.7–2.0) no reliable identification (<1.7).

**Conclusion:** The current bruker database has only 3 *C. auris* isolates from Korea and Japan, what it entails with identifications problems and low punctuation. Our database had a remarkable improvement in the identification of the 300 isolates, evidencing that the strengthening of the database is a great opportunity to improve the punctuation and *C. auris* identification.

### P177. Aspergillus-specific IgG assay optimum cut-off for the diagnosis of chronic pulmonary aspergillosis

WilopoB.^1^HunterE.^1^PageI.^1^MooreC.^1^,^2^RichardsonM.D.^1^,^2^DenningD.^1^,^2^1Division of Infection, Immunity and Respiratory Medicine, The University of Manchester, Manchester, United Kingdom2Mycology Reference Centre, Excellence Center For Medical Mycology (ecmm), Wythenshawe Hospital, Manchester University NHS Foundation Trust, Manchester, United Kingdom3National Aspergillosis Centre, Manchester University NHS Foundation Trust, Manchester, United Kingdom

**Objectives:** Chronic pulmonary aspergillosis (CPA) is a fungal disease characterised by cough, haemoptysis and weight loss present for at least for three months in non-immunocompromised people. It is caused by *Aspergillus* spp. especially *Aspergillus fumigatus* that can reach the respiratory tract by airborne transmission. The diagnosis of CPA can be made by a variety of clinical symptoms, radiological and laboratory evidence of *Aspergillus*. Most patients with CPA have positive *Aspergillus* precipitating antibodies or an abnormally high level of *Aspergillus*-specific IgG (Aspf IgG). The Bordier *Aspergillus fumigatus* ELISA kit is one assay that can detect Aspf IgG and the result is deemed positive when the optical density (OD) index is higher than 1.0. We have re-evaluated this cut-off in patients with CPA in the UK.

**Methods:** Aspf IgG levels in sera from 114 CPA patients and 150 British healthy controls were measured by the Bordier *Aspergillus fumigatus* ELISA kit. The results obtained were organised in MS Excel spreadsheets and analysed were performed using SPSS. Sensitivity and specificity at a number of points on the receiver operating characteristics (ROC) curve were described. The optimum diagnostic cut-off for the Bordier assay was calculated using Youden’s J statistic (sensitivity + specificity - 1).

**Results:** Based on statistical analyses, we determined that the ROC area under the curve (AUC) of the Bordier *Aspergillus fumigatus* ELISA kit was 0.928. This assay has 77.2% sensitivity and 99.3% specificity using the manufacturer’s cut-off (1.0). The sensitivity and specificity of this assay at various points on the ROC curve improves to 83.3% sensitivity and 97.3% specificity if the OD index cut-off is lowered to 0.9. At this cut-off, the diagnostic odds ratio and diagnostic accuracy are 145 and 90.9%.

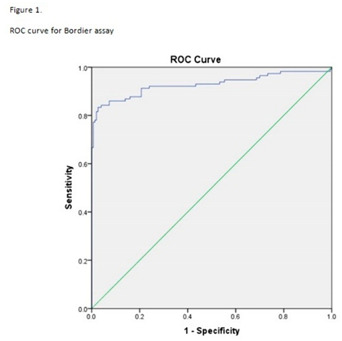

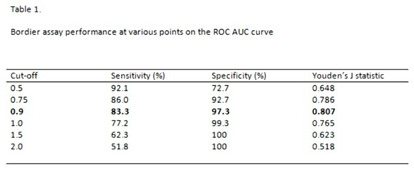





**Conclusion:** We have performed a ROC AUC analysis comparing Aspf IgG levels in a large group of patients with CPA compared to healthy British controls. Diagnostic performance improves if the OD index cut-off falls from 1.0 to 0.9.

### P178. Influence of Intravenous Human Immunoglobulins on the Accuracy of Aspergillus Galactomannan Antigen Assays

LiuW.-D.^1^ShihM.-C.^2^,^3^WangY.-W.^4^LinS.-C.^4^LeeY.-F.^5^LinS.-W.^4^,^6^ChouW.-C.^5^,^7^WuU.-I.^1^,^8^ChenY.-C.^1^,^9^1Department of Internal Medicine, Division of Infectious Disease, National Taiwan University Hospital, Taipei City, Taiwan2Institute of Epidemiology and Preventive Medicine, College of Public Health, National Taiwan University, Taipei City, Taiwan3Department of Medical Education, National Taiwan University Hospital, Taipei City, Taiwan4Department of Pharmacy, National Taiwan University Hospital, Taipei City, Taiwan5Department of Laboratory Medicine, National Taiwan University Hospital, Taipei city, Taiwan6Graduate Institute of Clinical Pharmacy, National Taiwan University, Taipei City, Taiwan7Department of Internal Medicine, Division of Hematology, National Taiwan University Hospital, Taipei City, Taiwan8Department of Internal Medicine, National Taiwan University Cancer Center, Taipei City, Taiwan9Center of Infection Control, National Taiwan University Hospital, Taipei City, Taiwan

**Objectives:** Galactomannan (GM) enzyme immunoassay (EIA), a diagnostic tool that detects the cell wall component secreted by *Aspergillus* species, provide a non-invasive method for early diagnosis of invasive aspergillosis (IA). However, its performance in diagnosing IA can be limited by potential false positivity caused by cross-reaction with other fungal species, food consumption and administration of certain antibiotics and blood products. False positive results were also demonstrated in a few patients having received intravenous immunoglobulins (IVIG). Some cases were attributed to cross-reactivity between the GM kits and saccharose contained in some IVIG products. Recently, we observed a growing number of patients prescribed with maltose-stabilized IVIG had false positive GM results despite absence of factors reported in the literature. We thus sought to determine if the false positivity was due to the processing procedures and presence of other unrecognized cross-reactive antigens contained in IVIG.

**Methods:** Two new, unopened bottles of human IVIG (TBSF, CSL Behring, Australia) was randomly obtained from our hospital pharmacy. Each vial contained 3g (6%) human IVIG and 14.6 mmol maltose in 50ml solution. From each bottle, solution were aspirated for dilution using 0.9% saline to the concentration of 1% and 0.1%, respectively in order to simulate the peak (15.8 g/L) and trough (7.4 g/L) of serum IVIG level in an adult having received IVIG infusion at a concentration of 0.2-0.6 g/Kg. A total of three different concentrations (6%, 1% and 0.1%) of IVIG together with negative controls (0.9% saline) were prepared in duplicates for GM detection using the Platelia *Aspergillus* EIA Kits (Bio-Rad) according to the manufacturer’s instructions. In order to explore if heat treatment and addition of EDTA required in standard sample processing procedures were associated with false positivity, samples were prepared with and without the investigated pretreatment, respectively for comparisons. A GM test result was considered positive if the optical density index (ODI) reached 0.5 or above. In addition, conventional bacteria and fungus cultures, pan-fungal polymerase chain reaction (PCR) were performed using undiluted samples from the bottles of IVIG to assess the presence of contaminants. All procedures were performed under strict sterile conditions.

**Results:** Using standard procedures with heat pretreatment and addition of EDTA, all IVIG samples tested positive for GM regardless of the concentrations used for the experiments (Figure 1), while all 0.9% saline controls yielded negative results. Pre-treatment of samples with boiling water-bath resulted in considerable elevation of GM ODI in both presence and absence of EDTA compared to samples without heat treatment. No fungus or other microorganisms were isolated from our IVIG samples using PCR or conventional culture methods.

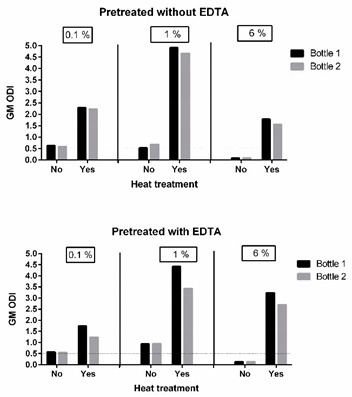



Figure 1. GM ODI of IVIG samples prepared with different concentrations and pretreatment methods.

**Conclusion:** IVIG treatment can cause false GM positivity owing to the reaction between the contents in the IVIG products and standard pretreatment procedures of the assays. Interpretation for a positive GM assay result should be cautious in patients having received human IVIG infusion.

### P179. Evaluation of the relevance of use of the BD BACTECTM mycosis IC/F, BD BACTECTM Plus Aerobic/F, BD BACTECTM Lytic/10 anaerobic/F et BD BACTECTM Peds Plus/F culture bottles system for fongemia detection: A 4-year retrospective study at the University Hospital of Dijon, France.

MagallonA.^1^BasmaciyanL.^1^ValotS.^1^CurraizeC. Goulard De^2^SautourM.^3^BadorJ.^2^DalleF.^4^1Mycology, University Hospital of Dijon, Dijon, France2Bacteriology, University Hospital of Dijon, Dijon, France3Laboratoire Parasitology Mycology, CHU Dijon, Dijon, France4Parasitology-mycology, University Hospital Dijon, Dijon, France

**Objectives:** Fongemia are severe invasive fungal diseases that combine rapid evolution with high mortality. The early diagnosis of these pathologies is therefore fundamental in the care of patients and is a vital emergency. Currently, only the BD BACTEC^TM^ Automated Blood Culture System (Becton Dickinson, Franklin Lakes, New Jersey, USA) has a bottle specifically dedicated to the detection of fungal agents, the BD BACTEC^TM^ Mycosis IC/F bottle. The objective of this study was to (i) evaluate the fongemia detection performance of the BD BACTEC^TM^ Mycosis IC/F culture bottles in comparison to the so-called "bacteriological" culture bottles (*i.e.* BD BACTEC^TM^ Plus Aerobic/F bottles, BD BACTEC^TM^ Lytic/10Anaerobic/F bottles, and BD BACTEC^TM^ Peds Plus/F) in different clinical situations and so (ii) the relevance of their use.

**Methods:** This retrospective study was conducted over a period of 4 years (*i.e.* from January 2013 to December 2016) at the University Hospital of Dijon. 331 pairs of blood cultures (*i.e.* each pair combining a BD BACTEC^TM^ Mycosis IC/F culture bottle with at least one of the bacteriological culture bottle). Univariate analysis by the MacNemar chi² test was carried out globally as well as in the subgroups corresponding to the various clinical situations encountered.

**Results:** of the 331 pairs of positive for fungi blood cultures included in the study, the BD BACTEC^TM^ Mycosis IC/F culture bottles showed a diagnostic advantage over bacteriological culture bottles in 57.4% of cases (p <0.01). In addition, for 96 pairs of blood cultures, only the BD BACTEC^TM^ Mycosis IC/F culture bottles was positive for fungi *versus* 66 pairs in which only the bacteriological culture bottles was positive for fungi. On the other hand, a fungus / bacterium co-infection was observed in 46 of the 331 pairs of blood cultures included in the study. In this "co-infection" subgroup, the overall "diagnostic advantage" variable was significantly in favor of the BD BACTEC^TM^ Mycosis IC/F culture bottles (*n* = 30, p<0.05). Multivariate analysis revealed an association between diagnostic performance and (i) the existence of yeast-bacteria co-infections, (ii) the mode of sampling, (iii) the type of fungus and (iv) diagnostic indication or follow-up of the patient, showing the relevance of the use of the BD BACTEC^TM^ Mycosis IC/F culture bottles in addition to those of bacteriology.

**Conclusion:** This study showed the necessity of the of the BD BACTEC^TM^ Mycosis IC/F culture bottles in the diagnosis of candidemias, in particular in case of bacterial co-infections.

### P180. rGliotoxin (GT) and bis-methyl-Gliotoxin (bmGT) characterization during Aspergilllus fumigatus infection.

LopezA. GomezHernándezC. RuedaPozoR. PandoReference Laboratory On Mycology, CNM-ISCIII, MAJADAHONDA-MADRID, Spain

**Objectives:** To develop a new method based on liquid-liquid extraction and chromatographic separation to specifically and simultaneously characterize both mycotoxins. We also describe the primary bmGT and GT’s role as defence molecule during *Aspergillus* progression in two cell lines infection model of IA that mimic human disease by measurement of GT and bmGT amount and their cytotoxic effect.

**Methods:** An UHPLC/PDA method was first developed and validated to specifically characterize both compounds. Chromatographic separation was achieved using an ACQUITY UPLC-H Class apparatus. The analytical conditions were selected after testing different parameters such as extraction procedures, column type, mobile phase composition, etc. We applied the developed method to define temporal kinetics of GT and bmGT production by using a fungal infection in two different cell line models, A549 (ATCC CCL-185, alveolar basal epithelial cell of human adenocarcinoma), and RAW 264.7 (ATCC TIB-71, murine macrophages) and an *Asperillus fumigatus* clinical isolate, GT producer. After the establishment of a specific infection model (ratio1:1, inoculum size 5x10t5 spores), we harvested each supernatant from cell line cultures at fixed time points, for assessment of GT/bmGT production. Galactomannan (GM) antigen was also quantified in order to establish the fungal burden at each time. In addition, a microscopic evaluation was performed to prove spores progression in each cell line. Specific stains (Sytox, calcofluor) were used to better define the characteristics of infection

**Results:** The best overall selectivity and peak shape were achieved by using a silica column (ACQUITY UPLC® HSS, C18, 2,1 x 75 mm 1,8μm particle size) combined with a mobile phase consisting of a gradient of water and acetonitrile and a flow rate of 0.1 mL/min. The developed method was applied to analyze the extracted supernatant (using chloroform) obtained from each condition. In the laboratory condition established, GT was firstly detected after 30 h of incubation. The kinetic profile showed a maximum production after 48 h and then a continuous decrease. Enrichment of GT in infected cell lines did not correlate with GM. Additionally, the level of bmGT, the inactive derivate of GT, was detected later. Contrary to GT, the levels of bmGT showed a continuous increase after 48 h of incubation. No great differences were found in GM, GT and bmGT amounts by cell lines studied. In both models, GT showed a concentration-dependent cytotoxic effect. of note, bmGT did not show any toxic effect and appear to be more stable than GT.

**Conclusion:** 1) GT and bmGT may be excreted to extracellular media during the course of *Aspergillus fumigatus* infection, although these components showed different kinetics during the *Aspergillus fumigatus* growth. 2) The chromatographic method described was successfully applied to characterize these secondary metabolites, having potential as a diagnostic technique for aspergillosis.

### P181. In vitro comparison between two commercial kits for the detection of Aspergillus galactomannan. Preliminary results.

GiannakopoulouD.-A.ZachrouE.ZarkadaE.VyzantiadisT.-A.First Department of Microbiology, Medical School, Aristotle University of Thessaloniki, Thessaloniki, Greece

**Objectives:** Galactomannan is an important component of fungal cell wall, mainly measured for the early diagnosis and monitoring of invasive Aspergillosis. Several commercial kits have been released and the aim of this study was to compare the recently available kit of Dynamiker Aspergillus Galactomannan Assay (Dynamiker Biotechnology Co, Ltd, China) to the internationally used and broadly evaluated Platelia^TM^ Aspergillus Ag (Bio-Rad, France).

**Methods:** Both kits are immunoenzymatic assays, based on the same principle with slight technical differences. An advantage of the Dynamiker method is the use of breakable strips, while both produce results after the calculation of an index value. The interpretation of positivity is based on the cut-off index of 0.5 for serum and 1.0 for BAL, defined after years of use and evaluation of results produced by the Bio-Rad kit. Forty four (39 serums, 4 BALs, 1 pleural fluid) specimens were tested by both methods (18 positive by the Bio-Rad kit). The positive and negative controls of the Bio-Rad kit were also tested by the Dynamiker one. Further on, in order to test the precision of the Dynamiker kit, twelve specimens were tested for their inter-day variation between two or three sequential measurements and their intra-day variation concerning double measurements. Alongside, two positive specimens (one serum and one BAL) were further diluted in three sequential two-fold sub dilutions (1:2, 1:4 and 1:8) in order to test the detection ability of the kit.

**Results:** The values (mean±SEM) were 1.12±0.24 by Bio-Rad and 0.68±0.12 by Dynamiker. Although, the strict numeric values were quite different, they didn’t differ statistically (p = 0.35) and they were positively correlated (r = 0.65, p = 0.000001). The precision of the Dynamiker kit was good with an intra-run CV (coefficient of variation) of 13.01% and an inter-run CV of 13.25%. However, while all negative (26 specimens) by Bio-Rad were found again negative by Dynamiker, this was not the case with the positive specimens, where there was a disagreement in six out of eighteen (33.3%, positive by Bio-Rad and negative by Dynamiker) and the strict numeric values differed statistically (p = 0.016). Both Bio-Rad positive and negative controls were found at the same level with the Dynamiker and all dilutions of the tested two positive samples provided positive results.

**Conclusion:** If the long used Bio-Rad kit is going to be considered the reference kit for the comparison of other recently available commercial kits, then it is probable that the here-tested Dynamiker kit is quite efficient and could probably offer a reliable alternative. However, these preliminary results show that there is a possible issue of sensitivity by not being able to detect a percentage of positive samples, while the kit is highly specific. Further in vitro studies with larger number of samples in combination to clinical information and outcome would provide a more documented and evaluated use of the kit.

### P182. External Validation of a bedside prediction rule for candidemic patients

AlobaidK.^1^ChadhaA.^2^AlansariA.^3^1Mycology Reference Lab, Mubarak Alkabeer hospital, Jabryia, Kuwait2Microbiology Unit, Mubarak Alkabeer hospital, Jabryia, Kuwait3Medical Department, Mubarak Alkabeer hospital, Jabryia, Kuwait

**Objectives:** The aim of this study is to examine Ostrosky-Zeichner’s rule (CPR) on predicting cases of candidemia among hospitalized patients in ICU and non ICU settings.

**Methods:** This is a prospective study on cases of candidemia from March 2017 till May 2018 in a secondary hospital. All candiemic cases were analyzed and tested by applying Ostrosky-Zeichner’s rule (CPR). Another group of patients who developed bacteremia were also tested as a control. The inclusion criteria for cases and controls are (1) positive blood culture in a patient with signs of infection, (2) hospital acquired infections. CPR was modified to include ICU and non ICU patients. The rule included presence of central line or broad spectrum antibiotic plus two of the following: steroids, immune suppressive agents, pancreatitis, major surgery, TPN, and major surgery.

**Results:** In the period from March 2017 till May 2018, 50 cases of candidemia were found. Two cases were excluded, as they represented contamination. Control group involved 60 patients. Majority of cases were hospitalized in medical wards (27), seven patients were surgical, 12 patients were hospitalized in ICU, and two patients were pediatric cases. **The sensitivity, specificity, PPV, and NPV of modified Ostrosky-Zeichner’s rule were 40.4%, 98.3%, 67%, and 95% respectively. Area under the curve (AUC ROC) is 0.69 and 95 % CI is (0.62-0.76).** Although 29 cases (59.6%) were not predicted by the rule, 6 patients (21%) had femoral catheters.

**Conclusion:** Clinical prediction rules are helpful adjunctive tool to rationalize the use of antifungals. Modified Ostrosky-Zeichner’s rule, in particular, appears to have good specificity for candidemic patients. The results also suggest that around 40% of patients can be treated empirically before culture results and this may improve survival. The possibility of femoral catheters as a risk factor for candidemia needs further evaluation. As shown in other studies, the high negative predictor value helps avoiding or stopping unnecessary use of antifungals.

### P183. Real Time PCR and galactomannan test for detection of Aspergillus spp. in bronchoalveolar lavage fluid of patients with aspergillosis

IgnatyevaS.^1^SpiridonovaV.^1^BogomolovaT.^2^ShadrivovaO.^3^BorzovaY.^1^DesyatikE.^2^VasilyevaN.^2^KlimkoN.^4^1Kaschkin Research Institute of Medical Mycology, North-Western State Medical University named after I.I. Mechnikov, Saint-Petersburg, Russian Federation2Kaschkin Research Institute of Medical Mycology. Department of Medical Microbiology, North-Western State Medical University named after I.I. Mechnikov, Saint-Petersburg, Russian Federation3Department of Clinical Mycology, Allergy and Immunology, North-Western State Medical University named after I.I. Mechnikov, Saint-Petersburg, Russian Federation4Department of Clinical Mycology, Allergology and Immunology, North-Western State Medical University named after I.I. Mechnikov, Saint-Petersburg, Russian Federation

**Objectives:** The success of treatment of fungal infection depends on early etiological diagnostics. The aim of the study was to test a real time PCR with High Resolution Melt analysis (mHRM-RT-PCR) and galactomannan (GM) for detection of *Aspergillus* spp. in bronchoalveolar lavage fluid (BAL) of patients with aspergillosis.

**Methods:** We tested 33 BAL from 33 patients with aspergillosis in Saint-Petersburg between 2015 and 2018 yy. As controls, 50 BAL were collected from patients without mycoses. BAL was examined by direct microscopy with calcofluor white and culture. Fungal DNA was extracted from clinical samples by a chloroform-isoamyl extraction method. DNA amplification was performed using aspergillus - specific primers and EvaGreen based mHRM-RT-PCR on Rotor-Gene 6000 cycler. Detection of GM was performed using “Platelia *Aspergillus* Ag” (Bio-Rad). The test was considered as positive with a cut-off of ≥0,5. We calculated the sensitivity and specificity for the PCR assay and GM with the level of statistical significance <0,05.

**Results:** Direct microscopic examination of BAL from patients with aspergillosis was positive in 42% cases, *Aspergillus* spp. were isolated in 91% cases. Cultures of 33 BAL samples revealed various *Aspergillus* spp.: *A*. *fumigatus* (63%), *A. niger* (19%), *A. flavus* (6%), *A.versicolor* (3%). From 3 patients (9%) two different species were isolated: *A.fumigatus* + *A.niger* (6%) and *A.fumigatus* + *A.flavus* (3%). GM test was positive in 23 BAL samples from patients with confirmed aspergillosis. Sensitivity of PCR assay in BAL was higher, than sensitivity of GM test (84% vs. 70%, p = 0,013). At the same time, specificity of GM test was higher than of PCR assay in patients without aspergillosis (87% vs. 71%, p = 0,018). The positive results of PCR assay in patients with aspergillosis correlated with positive results of microscopy and cultural investigations from BAL in 75% and 78%, positive GM test - in 55% and 95%, respectively. Complex investigation of biological materials by immunological and molecular methods increased the diagnostic effectiveness to 91 - 92%.

**Conclusion:** This study indicated that the mHRM-RT-PCR may be a very useful tool for detection of *Aspergillus* spp. in bronchoalveolar lavage fluid of patients with aspergillosis. Real Time PCR assay together with GM test increase the diagnostic effectiveness of biological materials investigations.

### P184. Detection and identification of mucormycosis etiologic agents in biological samples of oncohematological patients using multiplex real time PCR

IgnatyevaS.^1^SpiridonovaV.^1^BogomolovaT.^2^AvdeenkoY.^1^ShadrivovaO.^3^ZyuzginI.^4^ChudinovskikhY.^5^PopovaM.^6^UspenskayaO.^7^VasilyevaN.^2^KlimkoN.^8^1Kaschkin Research Institute of Medical Mycology, North-Western State Medical University named after I.I. Mechnikov, Saint-Petersburg, Russian Federation2Kaschkin Research Institute of Medical Mycology. Department of Medical Microbiology, North-Western State Medical University named after I.I. Mechnikov, Saint-Petersburg, Russian Federation3Department of Clinical Mycology, Allergy and Immunology, North-Western State Medical University named after I.I. Mechnikov, Saint-Petersburg, Russian Federation4N.N. Petrov National Medical Research Centre of Oncology, Saint Petersburg, Russian Federation5N.N. Petrov National Medical Research Centre of Oncology, Ministry of Health of Russian Federation, St. Petersburg, Russian Federation6I.Pavlov First Saint Petersburg State Medical University, St. Petersburg, Russian Federation7Leningrad Regional Clinical Hospital, St. Petersburg, Russian Federation8Department of Clinical Mycology, Allergology and Immunology, North-Western State Medical University n.a. I.I. Mechnikov, Saint-Petersburg, Russian Federation

**Objectives:** Mucormycosis is an opportunistic infection characterized by rapid progression, high morbidity and mortality. The success of treatment of fungal infection depends on early etiological diagnostics. The aim of the study was to test a multiplex real time PCR with High Resolution Melt analysis (mHRM-RT-PCR) on clinical samples for simultaneous detection and identification of Mucormycetes in biological samples.

**Methods:** We tested 12 clinical samples from 10 oncohematological patients with mucormycosis in Saint-Petersburg between 2014 and 2018 yy. As controls, 21 tissue samples and 20 BAL were collected from patients without mycoses. Fungal DNA was extracted from clinical samples by a chloroform-isoamyl extraction method. DNA amplification was performed using mucormycetes - specific primers and EvaGreen based mHRM-RT-PCR on Rotor-Gene 6000 cycler.

**Results:** The mHRM-RT-PCR allows to identify In clinical samples from patients with aspergillosis and mucormycosis the representatives of *Aspergillus* to the genus and Mucormycetes to the species level: *Rhizopus arrhizus, Mucor racemosus, Rhizomucor pusillus, Lichtheimia corymbifera*. We tested 3 native tissue, 7 formalin-fixed paraffin-embedded tissue samples and 2 BAL of patients with mucormycosis. In 1 of 3 native tissue and 2 BAL with positive direct microscopic examinations *Lichtheimia corymbifera* was isolated. In 7 formalin-fixed paraffin-embedded tissue non-septate mycelium of mucormycetes was detected. mHRM-RT-PCR was positive in native, formalin-fixed paraffin-embedded tissue samples and BAL of all patients with mucormycosis. PCR assay allowed to identify the representatives of Mucormycetes*: Lichtheimia corymbifera* in 10 and *Rhizomucor pusillus* in 2 from 12 samples. In biological specimens of 2 patients the PCR assay detected a mixed infection by *Aspergillus and* Mucormycetes *spp.* The results of mHRM-RT-PCR in patients with mucormycosis correlated with traditional methods. In 21 controls tissue samples and 20 BAL PCR test was negative.

**Conclusion:** The multiplex RT-PCR has high sensitivity and specificity in patients with mucormycosis. This study indicated that the mHRM-RT-PCR may be a very useful tool for detection of etiologic agents of mycoses, particularly in the case of a mixed infection caused by *Aspergillus* and Mucormycetes *spp.*

### P185. Clinical application of free circulating microRNAs in patients with hematologic malignancies as promising biomarkers in monitoring invasive aspergillosis

TolnaiE.NwozorK. OkechukwuFidlerG.SzaszR.RejtoL.BiroS.PaholcsekM.Faculty of Medicine, Department of Human Genetics, University of Debrecen, Debrecen, Hungary

**Objectives:** MicroRNAs (miRNAs) are small 19- to 23- nucleotide long, noncoding single stranded RNA molecules. They play an important role in regulating various biological processes through complementary base pairing with 3’- untranslated regions (3’ UTR) of the corresponding target mRNAs. MiRNAs are key regulators of various physiological and pathophysiological processes like cell proliferation, differentiation, apoptosis, inflammation and cancer. Altered expression levels of miRNAs have been associated with many diseases. Increasing number of evidences demonstrate that specific miRNAs can be a great promise as novel biomarkers for clinical diagnosis of many types of diseases due to their stability in various body fluids. However, little is known about the potential use of circulating miRNAs as biomarkers in fungal infections. Invasive aspergillosis (IA) is a life-threatening infection caused by *Aspergillus* especially in immunocompromised patients. With this pilot study our aim was to verify the different expression profiles of 21 pre-selected target miRNAs in hematologic malignant patients with (proven, probable IA) and without (possible IA and healthy controls) IA. We assessed the association of IA disease characteristics with the abundance of the free circulating microRNAs in the liquid biopsy samples of these patients to identify potential diagnostic markers for further studies.

**Methods:** We obtained whole blood (WB) samples from 40 patients at the onset of fevered neutropenia (FN) and from 5 healthy volunteers. Total RNA was isolated from the WB samples using the MagMax^TM^ mirVana^TM^ Total RNA Isolation Kit (Thermo Fisher Scientific). MiRNAs were reverse transcribed to cDNA with TaqMan^®^ Advanced miRNA cDNA Synthesis Kit (Thermo Fisher Scientific). TaqMan^®^ quantitative real-time PCR (miRNA assay) was used for detecting miRNA expression profiles. Relative miRNA expressions were calculated with the ΔΔ_Ct_ method.

**Results:** We found that 7 out of the 21 miRNAs were strongly associated with IA by being significantly (*p*<0.05; p<0,01) upregulated in our patient group compared to the control group. Increase in miRNA expressions were also observed in the case of further 7 miRNAs which expression differences did not prove to be statistically significant.

**Conclusion:** Based on the result of this pilot study, we detected significant expression differences in the case of seven miRNAs. We think that these may be promising and effective biomarker targets to support the diagnosis of IA. We also believe that the diagnostic strategy relying on the combination of multiple circulating miRNAs promises better positive predictive values in monitoring of IA.

### P190. rIndication of opportunistic micromycetes in mycobiota of municipal solid waste compost by metagenomic approach

KozlovG.^1^VasilyevaN.^2^RiabininI.^2^1Department of Technology For Microbiological Synthesis, Saint-Petersburg State Institute of Technology, Saint-Petersburg, Russian Federation2Kaschkin Research Institute of Medical Mycology. Department of Medical Microbiology, North-Western State Medical University named after I.I. Mechnikov, Saint-Petersburg, Russian Federation

**Objectives:** Objective of the study is to perform the genotaxonomic analysis of mycobiota of thermal-treated compost from municipal solid waste (CMSW) to identify the microfungi of medical interest.

**Methods:** The metagenomic study of samples using an internal transcribed spacer (ITS2) as a tacosomic marker was performed on the Illumina MiSeq instrument (Illumina, USA) according to the Manufacturer’s protocol. DNA-extraction, post-PCR purification of apmplicons and DNA concentration measurement were made by utilization PowerSoil DNA Isolation (Qiagen, Germany), AMpure XP magnetic beads (Beckman Coulter, USA) commercial kits and Qubit 4 Fluorometer (ThermoFisher Scientific, USA) respectively. Bioinformatic data processing was performed using QIIME 1.9.1 software, Silva and Unite databases. Classification algorithm of open-reference operational taxonomic units was utilized.

**Results:** Among a variety of microfungi from CMSW-mycobiota representatives of 20 taxa to varying degrees associated with pathologies and the indigenous human microbiota were revealed. They can be divided into three groups. Group A consist of micromycetes, which do not colonize the human body normaly (or occasional contaminants of the body’s open loci) but can cause opportunistic mycoses: *Aspergillus niger, Cryptococcus uniguttulatus, Rhodotorula glutinis*. Group B including micromycetes, sometimes occuring in biomaterials of human body, as well as colonizing environmental objects: *Candida zeylanoides*#, C. inconspicua*, *C. membranifaciens*#, Pichia norvegensis*, *Meyerozyma guilliermondii*#, *Kluyveromyces marxianus*, *Lodderomyces elongisporus*, Issathenkia orientalis*, *Trichosporon asahii* and *Cutaneotrichosporon cutaneum*. Group C: micromycetes that live mainly in the human body (sometimes in animals) and are not common inhabitants of abiotic objects: *C. albicans, C. parapsilosis*#, C. metapsilosis, C. glabrata*, *C. tropicalis*#, *Malassezia sp*.

**Conclusion:** CMSW is expected to be a habitat for microorganisms that are interesting in two aspects: (1) cause of their capability of carrying out uniqe metabolic pathways associated with the biodegradation of plant polymers, synthetic plastics and metallic materials and, on the other hand, (2) cause of representation by them the «microbiome imprint» of human population of a settlement area. Some of the detected yeast organisms are known to be resistant to antimycotics: the lists of groups B and C contain species with intrinsic resistance to fluconazole (*) as well as containing strains with secondary resistance mechanisms (#). The most unusual representative among the identified microbiota was *Malassezia sp*. Despite the deep adaptation of malasseziae to the humans and animals skin and numerous unsuccessful attempts to find their free-living related yeast, the application of molecular genetics technologies in the 21st century made it possible to identify the genetic material of these fungy in the most unexpected habitats (deep-sea sediments, invertebrates, some freshwater fishes, soil of arctic zones etc.). Ways to supply the high nutritional needs of malasseziae in the environment are not yet clear, theoretically, *Malassezia spp*. in CMSW can borrow the polyunsaturated fatty acids necessary for them directly from the remnants of dietary fats, or through symbiotic interaction with other members of the microbial community. Cunducted research made it possible to consider the CMSW-mycobiota as a potential niche associated with the circulation of opportunistic micromycetes in the anthropogenic ecosystem of the city.

### P191. Hunt for Cryptococcus: two years survey of the wild avian fauna of Southern France

DrakulovskiP.^1^GouveiaT.^1^PierruJ.^2^KrastevaD.^1^PottierC.^1^ArianielloE.^2^BelletV.^1^RogerF.^1^BertoutS.^1^1Laboratoire De Parasitologie Et Mycologie Médicale, University of Montpellier, Montpellier, France2Centre De Sauvegarde De La Faune Sauvage, LPO, Villeveyrac, France

**Objectives:** *Cryptococcus sp.* are environmental yeast whose natural biotope is still poorly understood. Indeed, *C. neoformans* has been isolated mostly from pigeon faeces but also from vegetable surfaces and several trees. *C. gattii* has been mostly isolated from decaying wood of various tree species, like *Eucalyptus, Pinus, Olea,* and *Ceratonia* trees but also from animals. *C. laurentii, C. albidus* have been isolated from soil, water but also from animal faeces. A few other species like *C. terrestris* or *C. aerius* are considered environmental only. In order to clarify the biotope of these yeasts, we are conducting a two year survey of the wild avian fauna in Southern France.

**Methods:** Six hundred and four birds from 70 genera and 86 species, randomly selected from the animals arriving for care at the Center for Protection of the Wild Fauna of Herault were sampled. Sampling was performed during care protocols either by direct cloacal swab or, when impossible, by swabbing of faecal drops present in cages. The samples were treated as per the standardized protocol used by the ISHAM Cryptococcal Working Group i.e transferred onto Niger Agar seed medium and grown at 28°C and 37°C during 7 days minimum. Each suspected *Cryptococcus* colony was checked for the presence of a capsule by India ink staining, for its urease activity and identified by API ID32C and ITS sequencing.

**Results:** Identification allowed to find 39 C. albidus, 30 C. magnus, 13 C. laurentii, 2 Cutaneotrichosporon curvatus, 2 C. oeirensis, 2 C. diffluens, 2 C. carnescens, 2 Filobasidiella elegans, 1 C. wiriengae, 1 C. terrestris and 1 C. aerius coming from 33 different bird species. No C. neoformans nor C. gatti were found so far. Global cryptococcal prevalence was of 15.7%.

**Conclusion:** Several *Cryptococcus* were found from bird wherein they were not described until now. As such, this study brings new data on the avian species potentially carrying various *Cryptococcus* sp yeast.

### P192. Microbial monitoring and environmental influence before and after hospital architectural design

BonadioF.^1^CaetanoM.^2^SiqueiraJ.P.^3^AlmeidaM.^1^1Infectious Disease, Medical School, sao jose do rio preto, Brazil2UNESP, São José do Rio Preto, Brazil3Infeciosas Disease, FAMERP, São José do Rio Preto, Brazil

**Objectives:** To assess the microbial contamination and the environmental impact of reform in a hemodynamic service hospital ward, in a Hospital of the Interior of the State of São Paulo, Brazil.

**Methods:** Environmental samples were collected from 10 surfaces (floor, walls, sinks, and counters), in two different moment, in rooms of medical procedures. RODAC® plates were imprinted in the different surfaces. Considering fungi and bacteria, plates were incubated at 35°C for up to hours to allow all organisms to grow. Colonies were isolates in agar BHI (DIFCO®) with identification of bacterial species conducted by automated system VITEK®, and fungi by slide culture in Potato Agar (DIFCO®) and biochemical characteristic. After first sampling, the rooms of the Hemodynamics Service in the hospital ward was reformed, using all principles and materials commercially available to control microorganisms. Innovative material and procedures of civil construction were adopted, including paint, mortar, and coating following normative criteria according to Brazilian legislation. Then, 10 new surface samples were obtained from the same points of the previous collection, at the same incubation, isolation, and identification criteria.

**Results:** In the first sampling, it was detected contaminated surfaces with high microbial load: 100% for bacteria, and 80% for fungi, ranging from 1 to 400 CFU, according to the collection point. Considering the microbial diversity, there was prevalence for Gram positive bacteria from Staphylococcus spp, and filamentous fungi, especially *Aspergillus* spp., *Acremonium* spp., and *Scopulariopsis* spp. After the reform, microbiological analysis showed a significant reduction of microbial counts, as well as reduction in the diversity of bacterial and fungal species. It was noted that 80% of the cultures were negative. No fungi were observed and the bacterial isolates are still being identified.

**Conclusion:** The hospital environment should be constantly monitored for its competence in non-proliferation of infectious agents. The presence of bacteria and fungi on surfaces may constitute a risk factor for dissemination and occurrence of infectious diseases, with great impact on Public Health. Old constructions may favor the dissemination of these organisms. This study confirmed the high microbial contamination in the hospital environment before reform and showed that a well-designed architectural design, with appropriate material and adoption of normative procedures, can minimize the risks of contamination and dissemination of potentially pathogenic microorganisms.

### P193. Study of fungal environmental contamination in nests of the penguin enclosure of a large French animal zoo park.

CartierN.^1^SénéchalR. Le^1^BecerraM.^1^BaillyE.^1^CateauE.^1^ChandenierJ.^2^,^3^MulotB.^4^LeclercA.^4^DesoubeauxG.^2^,^3^1Parasitology - Mycology - Tropical Medicine, Hôpital Bretonneau, Tours, France2Parasitology - Mycology - Tropical Medicine, Hôpital Bretonneau, TOURS, France3Cepr Inserm U1100, Université de Tours, Tours, France4Clinique Vétérinaire, ZooParc de Beauval & Beauval Nature, Beauval, France

**Objectives:** *Aspergillus fumigatus* is an environmental mold with opportunistic pathogenicity. It is responsible for aspergillosis, a disease particularly common in penguins that are kept in a confined or insufficiently ventilated environment. At ZooParc de Beauval (Saint-Aignan, France), the incidence of aspergillosis was estimated by autopsy at 4% in Humboldt penguins (*Spheniscus humboldti*) during the period [April 2018 - April 2019]. Antifungal treatment is based on the administration of azole drugs. This study assessed the environmental contamination in the penguin enclosure and searched for azole resistances in *A. fumigatus* isolates.

**Methods:** Two sets of environmental samples were carried out in the spring and fall 2018, by swabbing the artificial nests of the penguin enclosure in ZooParc de Beauval. The collected specimens were inoculated onto malt extract agar at 35°C. In case of fungal growth, a count of the colony number, as well as a phenotypic and molecular identification were achieved. Each DNA of *all the A. fumigatus* isolates was systematically sequenced to look for azole resistance mutations in *CYP51* gene.

**Results:** Overall in spring and fall sampling, 0.8 and 2.6 mean fungal CFU *per* sample of penguin nest were counted, *i.e.* 83% and 100% nests were contaminated with at least one fungal species, respectively. Moreover, *Aspergillus fumigati* section was isolated in 67% and 75% of them. Thus, the concordance rate was 67% between the two sets of samling. Through to DNA sequencing, all but one isolate (*Aspergillus nishimurae*) were eventually identified as *A. fumigatus stricto sensu*. To date, only one resistance mutation for azole drugs was detected once in the *CYP51* gene (G138A).

**Conclusion:** Despite the geographical location of ZooParc de Beauval in a rural environment, intensive adjacent agricultural practices do not exhibit any impact on the selection of resistant strains. However, the very high contamination rates herein encourage great caution and regular checks of the fungal populations.

### P200. rSpecific Identification and Antifungal Susceptibility Pattern of Fungal Species Isolated from Patients with Onychomycosis

HaghaniI.^1^Shams-GhahfarokhiM.^2^ShokohiT.^3^HedayatiM.T.^3^AslA. Dalimi^4^FathiM.^4^1Medical Mycology, Mazandaran university of Medical sciences, Sari, Iran2Department of Mycology, Faculty of Medical Sciences, Tarbiat Modares University, Tehran, Iran3Department of Medical Mycology, Invasive Fungi Research Center, School of Medicine, Mazandaran University of Medical Sciences, Sari, Iran, Sari, Iran4Department of Parasitology, Faculty of Medical Sciences, Tarbiat Modares University, Tehran, Iran

**Objectives:** Onychomycosis is a common nail problem, accounting for up to 50% of all nail diseases. The aim of the present study was to determine the species distribution based on the restriction fragment length polymorphism and susceptibility patterns of the causative agents of onychomycosis.

**Methods:** This cross-sectional study was conducted on nail samples collected from 257 patients suspected of onychomycosis within a year. Fungal isolates was identified by polymerase chain reaction-restriction fragment length polymorphisms (PCR-RFLP) with the enzymes *Msp I, Mva I, Alw I* and sequencing.

**Results:** According to the results, Out of the 257 patients participating in the study, onychomycosis was diagnosed in 180 (70.03 %.) cases, among which 51.1% were caused by non-dermatophyte molds (NDMs), 34.4% by yeasts, and 10.6% by dermatophytes. Numerous cryptic species recovered from onychomycosis for the first time. In the majority of cases, novel triazoles and imidazoles (i.e., efinaconazole, luliconazole, and lanoconazole) showed potent activity in comparison to other antifungal agents. The minimum inhibitory concentration (MIC) of luliconazole and lanoconazole ranged within 0.001 to >1μg/ml and their geometric mean MICs were 0.0154 and 0.0309 μg/ml against all isolates, respectively.

**Conclusion:** It seems that obtained data will be useful to improve the knowledge of researchers, clinicians, and dermatologists about onychomycosis distribution, species diversity and adoption of appropriate treatment.

### P201. Ibrexafungerp (formerly SCY-078) Demonstrates Activity Against Candida auris: In Vitro, In Vivo and Clinical Case Studies of Candidemia

BaratS.AnguloD.SCYNEXIS, Inc., Jersey City, United States of America

**Objectives:** *Candida auris* is a growing global threat; a pathogen associated with high mortality (up to 60%), multi-drug resistance, the ability to spread from person-to-person and surface-to-person, presenting high risk for outbreaks in healthcare facilities. Ibrexafungerp is a novel IV/oral glucan synthase inhibitor (triterpenoid) antifungal with activity against *Candida*, *Aspergillus* and *Pneumocystis* spp, in Phase 3 development. Given the potent activity of ibrexafungerp against *Candida* spp., Scynexis has embarked on a development program to understand the activity and effectiveness against *Candida auris*. We will present the current preclinical and clinical data sets of ibrexafungerp against *Candida auris*.

**Methods:** *In vitro* studies tested ibrexafungerp against >100 clinical isolates of *C. auris*. Other *in vitro* studies evaluated the effects of ibrexafungerp against *C. auris* biofilms. *In vivo* activity against *C.auris* was evaluated using a disseminated murine model and a cutaneous infection guinea pig model. In humans, an ongoing open-label trial of ibrexafungerp for treatment of patients with infections caused by *C. auris* (the CARES study) has been initiated in the USA and India.

**Results:** *In vitro and in vivo* studies demonstrated that ibrexafungerp is active against *C. auris*, including MDR strains. The MIC mode for ibrexafungerp was 1ug/ml and the MIC_50_ and MIC_90_ were 0.5 and 1 ug/ml, respectively. Many echinocandin resistant *C. auris* isolates have shown susceptibility to ibrexafungerp. Further, ibrexafungerp has been shown to reduce biofilm thickness. In animal models of *C. auris* infection, treatment with ibrexafungerp resulted in improved survival and reduced fungal burden in both the murine model of disseminated infection and the guinea pig model of cutaneous infection as compared to untreated controls. In humans, two patients with difficult to treat *C. auris* candidemias were enrolled in the CARES study and responded positively to oral ibrexafungerp with eradication of the infection.

**Conclusion:** This data demonstrate that ibrexafungerp possess potent *in vitro* and *in vivo* activity as well as promising clinical activity. Therefore, continued clinical evaluation of ibrexafungerp as an option to treat *C. auris* infections is warranted.

### P202. Microsporum ferrugineum in Germany – an anthropophilic dermatophyte on the rise

UhrlassS.^1^SitaruC.^2^ScholzC.^2^GebhardtM.^3^BaunackeA.^4^FriedrichsC.^5^Ranke-GreveI.^6^CleffmannU.^6^KochD.^1^MuetzeH.^1^KruegerC.^1^RahmigN.^7^HiplerU.-C.^7^NenoffP.^1^1Partnership Dr. C. Krueger & Prof. P. Nenoff, Laboratory of medical microbiology, Roetha OT Moelbis, Germany2Klinik Für Dermatologie Und Venerologie- Mykologisches Labor, Universitätsklinikum, Freiburg, Germany3Hautarztpraxis, Zwickau, Germany4Hautarztpraxis, Radeberg, Germany5Medizinisches Labor Ostsachsen, Görlitz, Germany6Dr. Stephan Bartels, Hautarztpraxis, Göttingen, Germany7Klinik Für Hautkrankheiten, Universitätsklinikum, Jena, Germany

**Objectives:** In Germany, the anthropophilic dermatophyte *Microsporum* (*M*.) *ferrugineum* was nearly never isolated during the last 50 years. Currently, starting with the year 2016, single strains were detected, and small outbreaks of infections due to this difficult to differentiate fungus have been observed in Germany. From July 2016 until April 2019, altogether 19 *M. ferrugineum* strains were isolated. The dermatophytes originated from patients all over Germany.

**Methods:** Cultural investigation revealed slowly growing colonies with white thallus and peripheral yellow brownish submerged hyphae bundles. The reverse side of the furrowed colonies showed cream coloured to yellow staining. Microscopically, large spherical and oval double-walled intercalary localized chlamydospores, typical ‘bamboo‘ hyphae, and acute-angled branched hyphae developed. Fungal culture material from all isolates was identified by PCR and genomic Sanger sequencing of the internal transcribed spacer (ITS) region and/or the translation elongation factor (TEF)-1α gene. Reference strain for comparative identification was *M*. *ferrugineum* CBS 497.48 (Centraalbureau voor Schimmelcultures CBS, Utrecht, The Netherlands, www.westerdijkinstitute.nl). Patients were children and adolescents under 18 years, mainly males. Suggested source of infection was martial sports, e.g. wrestling, judo, and boxing. Surprisingly, a significant part of affected patients counted to Russian Germans. A migrant 3 years old boy from Afghanistan suffering from tinea capitis was also among the patients. Another strain was isolated from a 10 years old wrestler with suspicion of tinea corporis. There was no background of migration or contact to foreigners, the boy did not stay abroad. The mycological challenge is the cultural identification of *M*. *ferrugineum*. Confusion with the morphological similar species *M*. *canis*, but also *M*. *audouinii*, and *Trichophyton verrucosum* is possible.

**Results:** The comparison of the found ITS-2 sequence with known sequences deposited at the database of the National Center for Biotechnology Information (NCBI), Bethesda, Maryland, U.S., confirmed the species identification *M. ferrugineum.* The phylogenetic analysis of the dermatophytes – the dendrogram of fungal strains – demonstrated the genetic differences between *M*. *ferrugineum* strains and *M*. *audouinii* or *M. canis*. The three species could clear distinguished from each other. In particular, sequencing of the TEF-1α gene allowed discrimination between *M*. *ferrugineum* and *M*. *audouinii* or *M. canis* better than sequencing the ITS-2-region. Meanwhile, the *M. ferrugineum* strain 208361/2016 is deposited at the „Deutsche Sammlung von Mikroorganismen und Zellkulturen” (DSMZ, German Collection of Micro organisms and Cell cultures) in Braunschweig, Germany (DSM no. 103785). The DNA sequences of the *M. ferrugineum* strains are currently deposited as MF173061- ITS - and as MF173060 - TEF-1α gene - at the NCBI database.

**Conclusion:** In Germany, in the last decades, *M*. *ferrugineum* was not isolated and described so far. Currently, with this forgotten dermatophyte must be expected in particular in migrants and asylum seekers. The species identification of *M*. *ferrugineum* represents a challenge for the dermatologist. If there are morphological and microscopic features suspicious for such a rare *Microsporum*-species, molecular identification of the causative pathogen should be performed. For oral therapy of tinea capitis due to *M*. *ferrugineum* griseofulvin should be given, alternatively itraconazole or fluconazole.

### P203. Arthroderma chiloniense - a new geophilic dermatophyte – isolated from human and hedgehog

UhrlassS.^1^RimekD.^2^HubkaV.^3^SchroedlW.^4^ReuschelM.^5^BraschJ.^6^MuetzeH.^1^KochD.^1^KruegerC.^1^GraeserY.^7^NenoffP.^1^1Partnership Dr. C. Krueger & Prof. P. Nenoff, Laboratory of medical microbiology, Roetha OT Moelbis, Germany2Gesundheitsschutz, Thueringer Landesamt für Verbraucherschutz, Bad Langensalza, Germany3Laboratory of Fungal Genetics and Metabolism, Institute of Microbiology ASCR, Prague, Czech Republic4Ag Mykologie, Institut für Bakteriologie und Mykologie, Leipzig, Germany5Klinik Für Heimtiere, Reptilien, Zier- Und Wildvögel, Stiftung Tierärztliche Hochschule, Hannover, Germany6Klinik Für Dermatologie, Venerologie Und Allergologie, Universitätsklinikum Schleswig-Holstein, Kiel, Germany7Institut Für Mikrobiologie Und Hygiene, Universitätsmedizin – Charité, Berlin, Germany

**Objectives:** Due tot he new taxonomy of the dermatophytes, geophilic fungal species are summarized within the genus *Arthroderma* (*A*.). Until now known *Arthroderma* species are e.g. *A*. *insingulare*, *A*. *gertleri*, *A*. *uncinatum*, *A*. *thuringiensis*, besides, there are the three relatively new species *A*. *amazonicum*, *A. crocatum,* and *A. vespertilii*. Altogether four strains of an until now not known geophilic fungus - suggested as mould or dermatophyte - were isolated between 2011 and 2017 from different patient´s materials. Underlying dermatoses were a hand eczema or suspected tinea manus, nail changes (nail plate pigmentation) to exclude an onychomycosis, and skin scrapings vom the lower leg and body trunk to exclude a tinea corporis. An additional strain of *A. chiloniense* was found in clinical material originationg from the skin and stings of a hedgehog with alopecia diffusa and scaly skin.

**Methods:** Conventional and molecular mycological diagnostic was performed. Kind of growth of the fungal colonies on common fungal media was assessed. Fungal culture material from all five isolates was identified by PCR and genomic Sanger sequencing of the internal transcribed spacer (ITS: 18S rRNA, ITS1, 5.8S rRNA, ITS2, and 28S rRNA) region, the translation elongation factor (TEF)-1α gene, and β-tubulin. Human strains 216228/16- DSM106573, 211495/17- DSM106575, 211496/17- DSM106574 and 215488/17- DSM108904 are deposited at the „Deutsche Sammlung von Mikroorganismen und Zellkulturen” (DSMZ, German Collection of Micro organisms and Cell cultures) in Braunschweig, Germany. The from hedgehog isolated strain with laboratory number 250173/2018 has already been submitted to the DSMZ, too.

**Results:** The white, flat, fast growing colonies presented with yellow reverse sides. Microconidia are small, elongated, sessile lateral directly at the hyphae or the conidiophores, also in dense aggregates. Later arthroconidia and multiple chlamydospores have developed, however, no macroconidia. Urea splitting of all isolates was positive. By sequencing of the gene regions ITS-1 and ITS-2, TEF-1α and β-tubulin showed, that the new species showed the highest genetic similarity to the species *A. crocatum* and *A. amazonicum*. Ultimately, the sequences of the here isolated unknown fungi could be assigned to the new described geophilic dermatophyte *Arthroderma chiloniense sp*. *nov*.

**Conclusion:** *A*. *chiloniense* presents a new geophilic dermatophyte, first described in Kiel, Germany, in 2018, and published 2019. This species is obviously widely prevalent in Germany. Isolated from human beings under the suspicion of a dermatophytosis of the skin or nails, seems to indicate that *A*. *chiloniense* possesses pathogeic potential. Here, A. chiloniense, for the first time was isolated from an animal, an European hedgehog.

### P204. The First Case of Candida auris Fungemia in Thailand

ChayakulkeereeM.^1^UngulkraiwitP.^2^ChongtrakoolP.^3^NgamskulrungrojP.^3^GrootT. De^4^MeisJ.^5^1Medicine, Division of Infectious Diseases and Tropical Medicine, Faculty of Medicine Siriraj Hospital, Mahidol University, Bangkok, Thailand2Department of Medicine, Vejthani Hospital, Bangkok, Thailand3Microbiology, Faculty of Medicine Siriraj Hospital, Mahidol University, Bangkok, Thailand4Medical Microbiology and Infectious Diseases, Canisius-Wilhelmina Hospital, Nijmegen, Netherlands5Department of Medical Microbiology and Infectious Diseases, Excellence Center For Medical Mycology (ecmm), Canisius Wilhelmina Hospital, Nijmegen, Netherlands

**Case Report:**

**Objectives:** *Candida auris* is an emerging fungal species resistant to several antifungal agents. There is an increasing worldwide awareness of *C. auris*. We therefore aim to report the first case of *C. auris* infection in Thailand. 

**Methods:** We present the first case of *C. auris* fungemia in an elderly woman from Thailand. 

**Results:** A 81-year-old woman was admitted in a private hospital in January 2018 due to status epilepticus which required ICU support with prolonged mechanical ventilation. In addition, she had chronic kidney disease requiring hemodialysis through her right subclavian central venous catheter. During 4 months in the hospital, she experienced catheter-related bacterial blood stream infection, nosocomial pneumonia, urinary tract infection and tracheobronchitis which was treated with several regimens of broad-spectrum antibiotics. On day 110 of hospitalization, she had hypotension with low-grade fever during hemodialysis. Blood cultures from her central venous catheter grew *Candida* spp. While peripheral line cultures remained negative. The central catheter was removed and she was treated with micafungin. The *Candida* isolate was subsequently identified as *Candida auris* by MALDI-TOF and sequencing. Swab cultures from the central catheter exit site also grew *C. auris*. She denied any travel outside of Thailand prior to admission. Despite intravascular catheter removal, she had again candidemia from *C. auris* three weeks later and micafungin was re-introduced. However, the patient died 6 days after restart of treatment. After diagnosis, strict contact precautions were installed with no further intra-hospital transmission. CLSI microbroth dilution antifungal susceptibility testing of all isolates showed resistance to fluconazole (32 mg/L) and voriconazole (0.25 mg/L), higher MICs of amphotericin B (1 mg/L) and low MICs to echinocandins (0.063 mg/L). Isavuconazole had low MICs of < 0.016 mg/L. By using AFLP and MLVA genotyping the isolates had the same genotype and were shown to belong to the south-Asian *C. auris* clade. 

**Conclusion:** We report the first case of *C. auris* (south-Asian clade I) fungemia from Thailand. The index case for this *C. auris* introduction into Thailand remained unclear and no secondary cases were discovered. Active surveillance for this emerging antifungal-resistant *Candida* species should be brought into attention to prevent healthcare-associated outbreaks.

### P205. Disseminated phaeohyphomycotic lymphadenitis with Cladophialophora carrionii

FarooqiJ.^1^MoinS.^1^MahmoodS.F.^2^IdressR.^1^NaqviF.^1^ZafarA.^1^1Pathology and Laboratory Medicine, Aga Khan University, Karachi-KHI, Pakistan2Medicine, Aga Khan University, Karachi, Pakistan

**Case Report:**

**Objective**: This study describes a case of disseminated phaeohyphomycotic lymphadenitis in a young woman with delayed diagnosis and good clinical response after appropriate treatment. 

**Methods**: A 32 year old lady presented with erythematous to violaceous papules, with oozing discharge bilaterally in her inguinal region, for a few months. The history revealed that she had a past history of tuberculous meningitis, treated with first line antituberculous therapy for eighteen months, and two years back she developed pigmented discharging lymph nodes bilaterally in her axillae. The histopathology of the biopsy showed chronic granulomatous inflammation with multiple branching septate fungal hyphae. She received amphotericin B for 21 days but without improvement. Now, sample from these inguinal lesions was sent for histopathology and culture. 

**Results**: Histopathology of the biopsy material showed dense inflammation and well-formed granuloma comprising of epithelioid cells and multinucleate giant cells. Multiple branching septate fungal hyphae noted in the inflammatory debri and in and around granuloma. PAS stain highlighted septate fungal hyphae. Gram stain revealed moderate septate hyphae with numerous pus cells. Culture on Sabouraud dextrose agar yielded velvety olive-black colony in the fourth week. Microscopic slide examination of culture material was suggestive of *Cladophialophora carrionii*. The patient was started on voriconazole and was continued this for 6 months and the patient improved. On discontinuing voriconazole after six months, there was a relapse in the disease. Voriconazole was restarted and the patient improved. 

**Conclusion**: Incomplete investigation of infectious lesions may delay diagnosis. Both histopathological and microbiological assessments are equally important for making a conclusive diagnosis. Antifungal therapy may delay growth of fungi that normally grow within a week, hence a longer incubation time may be warranted for fungal smear positive samples. In phaeohyphomycosis, the infection is usually introduced by traumatic inoculation or inhalation of the etiologic agent; and clinical presentations are greatly influenced by the immune status of the host. In our case there was no known immune deficiency and no trauma that the patient could recall.

### P206. Genomic sequencing of Candida auris cases in Canada, 2012 - Feb 2019

BharatA.^1^AlexanderD.^2^DingleT.C.^3^DufresneP.J.^4^HoangL.M.^5^KusJ.V.^6^SchwartzI.S.^7^GadeL.^8^LitvintsevaA.P.^8^McgeerA.^9^MulveyM.R.^1^1Antimicrobial Resistance and Nosocomial Infections, National Microbiology Laboratory Canada, Winnipeg, Canada2Cadham Provincial Laboratory, Winnipeg, Canada3Alberta Public Laboratories, Edmonton, Canada4Laboratoire de santé publique du Québec, Sainte-Anne-de-Bellevue, Canada5BC Centres for Disease Control, Vancouver, Canada6Public Health Ontario, Toronto, Canada7University of Alberta, Edmonton, Canada8Mycotic Diseases Branch, US Centers for Disease Control and Prevention, Atlanta, United States of America9Mount Sinai Hospital, Toronto, Canada

**Objectives:** *Candida auris* is an emerging yeast that is associated with high rates of antifungal resistance and healthcare-associated outbreaks that can be difficult to control. Our objective was to carry out genomic characterization of all known *C. auris* cases in Canada to monitor the emergence of this species.

**Methods:** Nineteen isolates of *C. auris* identified from 2012 - Feb 2019 were submitted to the Canadian Mycology Reference Centre which is in development at Canada’s National Microbiology Laboratory (NML). Isolates from all 19 cases were subjected to whole genome sequencing (WGS) on the Illumina Nextseq platform. Phylogenetic analysis based on nucleotide differences in the core genome was carried out with the SNVPhyl v1.1 pipeline and assemblies were performed with SPAdes v1.6.

**Results:** The isolates were obtained from axilla/groin (*n* = 7), blood (*n* = 4), ear (*n* = 3), and other sites (*n* = 5). All Canadian isolates of *C. auris* fell within the four known genomic lineages (clades), which were initially named after the continents where they were identified and then renamed Clades I - IV. Canadian cases were linked to the Clade I (South Asian clade, *n* = 10), Clade IV (South American clade, *n* = 4), Clade III (African clade, *n* = 3), and Clade II (East Asian clade, *n* = 1). The four geographical clades were highly divergent with approximately 15,000 - 60,000 single nucleotide variants (SNV) between clades. Canadian isolates within a clade were often clonal, for example, isolates from Clade IV (South American clade) differed by only 14-26 SNVs. Canadian isolates from Clade I (South Asian clade) differed by an average of 79 SNVs, however, epidemiologically-linked isolates within this clade differed by only 1-19 SNVs. We identified mutations associated with fluconazole resistance (ERG11 Y143R) and voriconazole resistance (ERG11 Y132F) but not echinocandin resistance (FKS1 hotspot mutations).

**Conclusion:** The number of known cases of *C. auris* in Canada remains low and all cases were susceptible to echinocandins. Whole genome sequencing confirmed the global origins of Canadian cases. Isolates tended to be clonal within each clade but high resolution WGS may be helpful in discriminating between patient transmission and separate introductions into a healthcare facility.

### P207. Misidentification of Candida duobushaemulonii associated to superficial infection and reduced susceptibility to azoles

JuradoI.^1^Varela-EchegarayE.^1^Marcos-AriasC.^1^GrootP. De^2^QuindósG.^1^ErasoE.^1^1Inmunology, Microbiology, Parasitology, University of the Basque Country (UVP/EHU), Bilbao, Spain2Regional Center For Biomedical Research, Castilla-la Mancha Science & Technology Park, University of Castilla-La Mancha, Albacete, Spain

**Objectives:** Candidiasis by *Candida haemulonii* complex (*Candida haemulonii* and *Candida duobushaemulonii*) and closely related species, such as *Candida auris* and *Candida pseudohaemulonii* that show reduced susceptibility to antifungal drugs are increasing. Conventional phenotypic diagnostic methods are unable to identify these species. Therefore, their prevalence may have been underestimated. The aim was to use novel molecular techniques to identify isolates from a stock culture collection with dubious identities, which could belong to emerging and multi-resistant species related to *C. haemulonii*.

**Methods:** The study included 150 clinical isolates from the yeast stock collection of the Medical Mycology Laboratory at the University of the Basque Country (UPV/EHU) collected between 1993 and 2017 that could be inaccurately identified by API^®^ID 32C (bioMérieux, France). Three clinical confirmed isolates (two *C. auris* and one *C. duobushaemulonii*) and one *C. pseudohaemulonii* reference strain (CBS 10004) were included as positive controls. All isolates were analysed by two different PCR techniques: a *C. auris*-specific PCR^1^ and a *C. haemulonii* complex-tetraplex PCR^2^. Species identities were confirmed by sequencing the ITS1-5.8S-ITS2 region. Finally, *in vitro* susceptibility to nine antifungal agents (anidulafungin, micafungin, caspofungin, posaconazole, voriconazole, itraconazole, fluconazole, amphotericin B and 5-fluorocytosine) was tested using Sensititre^TM^ YeastOneYO10 (Thermo Scientific, USA).

**Results:** *C. auris*-specific PCR accurately identified both confirmed isolates of *C. auris* and no bands were detected for the other isolates. Eight isolates previously identified as *C. haemulonii* were confirmed by tetraplex PCR. Neither *C. auris* nor *C. pseudohaemulonii* were detected in the collection. One isolate from a toenail previously identified in 1996 by API^®^ID 32C panel as *Candida intermedia* produced a 115 bp sized band in the tetraplex PCR, corresponding to *C. duobushaemulonii*. All identifications were confirmed by ITS sequencing. The *C. duobushaemulonii* isolate was re-analysed by the current API^®^ID 32C panel, which identified it as *Candida sake*. The *C. duobushaemulonii* isolate was susceptible dose-dependent to itraconazole (MIC 0.5 µg/mL) and fluconazole (MIC 32 µg/mL). All *C. haemulonii* were susceptible dose-dependent to itraconazole (MIC 0.5 µg/mL) and fluconazole (MIC 16 µg/mL) except two, who were resistant (MIC 1 and 64 µg/mL, respectively), and their susceptibility to amphotericin B was reduced (MIC 2->8 µg/mL). The *C. pseudohaemulonii* reference strain CBS 10004 was susceptible to all antifungal drugs. Both *C. auris* isolates showed resistance to fluconazole (MIC >256 µg/mL), dose-dependent susceptibility to itraconazole (MIC 0.25 µg/mL), and one of them was also susceptible dose-dependent to voriconazole (MIC 2 µg/mL).

**Conclusion:** *Candida haemulonii* complex and closely related species may have been associated to superficial infections prior to its emergence related to invasive infections, as indicated by the identification of a *C. duobushaemulonii* isolate from before 2012, when current classification of the complex was established. This complex shows reduced susceptibility to azoles and amphotericin B. **Funding:** GIC15/78 IT-990-16 (Gobierno Vasco) and SAF2017-86188-P (MINECO, Spain). 1. Ruiz-Gaitán et al. *Molecular identification of* Candida auris *by PCR amplification of species-specific GPI protein-encoding genes*. Int J Med Microbiol. 2018;308:812-818. 2. Arastehfar et al. *Low-cost tetraplex PCR for the global spreading multi-drug resistant fungus,* Candida auris *and its phylogenetic relatives*. Front Microbiol. 2018;29;9:1119.

### P208. Acute fungal obstructive pyelonephritis, echinocandin is a possible treatment? A case report

TestaJ.^1^,^2^CaloriA.^3^NicoliniL.A.^4^PizziM.G.^2^ZeroliC.G.^2^MarteganiR.^2^MenzaghiB.^2^1Biotechnologies and Life Sciences Department, Center for Clinical Ethics, University of Insubria, Varese, Italy2Busto Arsizio Hospital, Asst-valleolona, Infectious Diseases Unit, Busto Arsizio (VA), Italy3Busto Arsizio Hospital, Asst-valleolona, Urology Unit, Busto Arsizio, Italy4Department of Health Science (dissal), Infectious Diseases Unit, Genova, Italy

**Case Report:**

**Objectives/Introduction:** Urinary tract candidiasis is the most frequent nosocomial fungal infection worldwide; ascending acute pyelonephritis (AP) due to Candida species are uncommon complications. Nowadays we observe a change in the distribution of *Candida* species, with an increasing number of non-albicans Candidaisolations. In this setting Fluconazole is the treatment of choice; the role of echinocandin is unknow, because of the limited repository of antifungals that achieve adequate urinary concentrations. 

**Methods:** We report a *Candida tropicalis* complicated urinary tract infection (cUTI) in a patient with diabetes mellitus who successfully treated by high dosage of Caspofungin and oral fluconazole (FLC). The infection was triggered by the placement of a ureteral double J stent and bladder catheter, arranged for kidney stones. 

**Results:** A 67-year-old Italian woman affected by diabetes mellitus, metabolic syndrome and recent breast cancer, was transferred in the autumn of 2018 from an outside institution after double J stent positioning for left ureteral stone complicated by grade III hydronephrosis. At the admission fever and increase inflammatory values were present, so blood and urine cultures were performed. Empiric antibiotic therapy with carbapenems was started, without clinic improvement in 5-6 days, so we interrupted this therapy. We isolated only pan-sensitive *Candida tropicalis* in urine sample but with few colonies, so we considered as colonization. She therefore underwent to CT-scan with urological and thoracic studies: we found left AP complicated by micro abscesses and pulmonary thrombo-embolism. We decided to introduce high dosage of FLC and to remove ureteral catheter double J and bladder catheter; we isolated from them another time the same type of *Candida tropicalis*. Considering a diffuse disease, trans-thoracic echocardiography and fundus oculi exam were performed, excluding fungal manifestations. After few days she remained febrile and blood inflammation biomarkers not decreased very well, so we decided to switch therapy with an alternative one, suspending FLC, we started Caspofungin at the dosage of 70 mg/die. Immediately after initiation of treatment we observed a rapid improvement with disappearance of the fever. After two weeks, abdomen CT-scan showed an inflammation reduction and she was discharged home with maintenance high dosage FLC (800 mg/die), concluding antifungal therapy after 3 overall months. Finally, after therapy, CT-scan and urine culture were prescribed with no evidence of recurrence. The patient is currently alive, without urinary problems. 

**Conclusion:** *Candida tropicalis* is a rare cause of AP. Echinocandins have been traditionally avoided in the treatment of cUTI because of their poor urinary concentrations; their use with high dosage, owes an achieved high renal tissue concentration. This case shows that in fungal cUTI we can use high dosage echinocandin if fluconazole is no possible for any reasons. We want also to stress that is necessary a prolonged therapy in fungal AP.

### P209. The first two cases of Candida auris in The Netherlands

VogelzangE.^1^WeersinkA.^2^MeisJ.^3^DijkK. Van^1^1Medical Microbiology and Infection Control, AUmc, location VUmc, Amsterdam, Netherlands2Medical Microbiology and Immunology, Meander Medical Center, Amersfoort, Netherlands3Department of Medical Microbiology and Infectious Diseases, Excellence Center For Medical Mycology (ecmm), Canisius Wilhelmina Hospital, Nijmegen, Netherlands

**Objectives:** *Candida auris* is a rapidly emerging, multidrug resistant pathogenic yeast. In recent years an increasing number of *Candida auris* invasive infections and colonized patients have been reported from several continents, including the USA, Africa, Europe and Asia. Here, we describe the first two cases of *Candida auris* in The Netherlands.

**Methods:** Case 1 was a middle aged patient repatriated from an Indian hospital to a Dutch intensive care unit (ICU). He had been hospitalized for five weeks in an Indian hospital for treatment of sepsis with multiple organ failure due to pneumonia. He was mechanically ventilated and repatriated with a tracheostomy tube and continuous renal replacement therapy for which he had a central venous catheter. Routine admission screening cultures for MRSA and multidrug-resistant gram-negative bacteria showed yeast growth from Continuous renal replacement therapya removed central venous catheter which the Bruker MALDI-TOF MS identified as *Candida auris*. Case 2 was a middle aged patient, referred by the Amsterdam airport medical services to the hospital due to shortness of breath and inability to stand without assistance. He was in Amsterdam airport for a layover flying from India to the USA. Due to renal insufficiency, he had received hemodialysis in an Indian healthcare facility up to two days before presentation in our hospital. Due to subfebrile temperature a urine sample was cultured which grew 10^4-5 CFU yeasts. The Bruker MALDI-TOF MS identified the yeast as *Candida auris.*

**Results:** In both cases contact precautions were instituted since patients had been previously admitted to an Indian healthcare facility. Routine screening cultures were taken from nose, throat and perineum to identify potential multidrug-resistant gram-negative bacteria and MRSA. At present no routine screening for *Candida auris* is performed in the Netherlands. Screening failed to detect *Candida auris*, but groins and axilla screens were not included. Molecular typing with AFLP and MLVA showed that both isolates had closely similar genotypes and both belonged to the South Asian *Candida auris* Clade I. Broth microdilution showed that both *Candida auris* isolates had high fluconazole (>64 mg/L) and voriconazole (4 mg/L) MICs. Amphotericin B (0.5-1 mg/L), anidulafungin (<0.016-0.063 mg/L) and micafungin (0.063 mg/L) had low MICs. In both cases antifungal therapy was not indicated since there were no signs of an infection. Due to installment of contact precautions no transmission of *Candida auris* was observed.

**Conclusion:** We report the first two cases of *Candida auris* in The Netherlands in patients who were recently admitted to an Indian healthcare facility. The patients were immediately placed in contact isolation after admission and no transmission of *Candida auris* occurred. Concern is raised regarding surveillance for multidrug-resistant organisms, since routine screening of patients from foreign hospitals is still focused on multidrug-resistant gram-negative bacteria and MRSA and not yet on (multi)drug resistant yeast, such as *Candida auris*.

### P210. rEvaluation of the disinfectant activity of a portable ultraviolet C equipment

López-SaizA.SevillanoE.GuridiA.De-La-FuenteI.MateoE.ErasoE.QuindósG.Immunology, Microbiology and Parasitology, University of the Basque Country (UPV/EHU), Bilbao, Spain

**Objectives:** Healthcare-associated infections are caused by different microorganisms, including bacteria, viruses and fungi. In the recent years, *Candida auris* has emerged as a multidrug-resistant fungal pathogen difficult to eradicate from hospital environment. It has been isolated from medical equipment, and from different objects that hardly ever are disinfected and can act as fomites (bed hand-controllers or mobile phones). In some cases, routine chemical and physical decontamination approaches are used but not all the above-mentioned objects can be treated with them due to possible deterioration. Ultraviolet C radiation could represent an alternative, which does not cause aesthetic or functional alterations in inanimate objects. Therefore, the objective of this study was to evaluate the disinfection capacity of a portable ultraviolet C (UV Sanitizer Corvent®) equipment.

**Methods:** The disinfectant effect of the UV sanitizer equipment was evaluated using different exposure times (30 s, 1 min, 2 min, 3 min and 5 min), against five *Candida* isolates: three clinical strains of *C. auris*, and two reference strains, *Candida albicans* SC5314 and *Candida parapsilosis* ATCC 22019. These microorganisms were inoculated onto slides, starting from a suspension of 1.5-5.0 x 10^9^ ufc/mL, that was mixed with an interference solution to analyze the disinfecting effect in both clean and dirty conditions (presence of organic matter). Slides were exposed to ultraviolet C radiation inside the equipment. The results were compared with those obtained after the disinfection of the slides by immersion in 70% ethanol following the protocol described in EN 14562:2006 standard.

**Results:** Disinfection, considered as a logarithmic reduction equal to or greater than 5 from the initial inoculum, was achieved at a minimum exposition time of 3 min, in both clean and dirty conditions. The presence of organic matter interfered in the disinfection process to some extent, since the final values were slightly higher after the treatment of the samples in dirty conditions. The effectivity of 3 min immersion in 70% ethanol was very high against all microorganisms, obtaining a total reduction in all cases.

**Conclusion:** The ultraviolet C radiation applied for 3 minutes using the portable ultraviolet C UV Sanitizer Corvent® equipment was effective in the disinfection of slides inoculated with *C. auris*, *C. albicans* and *C. parapsilosis*. This equipment is a promising alternative for implementing disinfection protocols in hospitals and laboratories with inanimate objects of common use (mobile phones, TV or bed controllers) reducing risk of infection transmission.

### P211. Emergence of ERG11 Y132F azole resistant Candida parapsilosis blood isolates in a Turkish hospital: A 10 year experience (2009-2019)

ArastehfarA.^1^DaneshniaF.^1^Hilmioğlu-PolatS.^2^FangW.^3^YaşarM.^2^PolatF.^2^MetinD. Yeşim^2^ZomorodianK.^4^CoenyeT.^5^IlkitM.^6^PanW.^3^LiaoW.^3^HagenF.^1^KostrzewaM.^7^Lass-FlörlC.^8^BoekhoutT.^1^1Westerdijk Fungal Biodiversity Institute, Utrecht, Netherlands2Department of Microbiology, Faculty of Medicine, University of Ege, Izmir, Turkey3Medical Mycology, Shanghai Changzheng Hospital, Second Military Medical University, Shanghai, China4Basic Sciences In Infectious Diseases Research Center, Shiraz University of Medical Sciences, Shiraz, Iran5UGent, Ggent, Belgium6Division of Mycology, Department of Microbiology, Faculty of Medicine, University of Çukurova, Adana, Turkey7Research and Development, Microbiology and Diagnostics, Bruker Daltonik GmbH, Bremen, Germany8Div Hygiene & Med. Microbiology, Med. Univ. Innsbruck, Innsbruck, Austria

**Objectives:** The recent emergence of azole resistant *C. parapsilosis* isolates carrying Y132F mutation in *ERG11* is a matter of concern in India, Kuwait, South Korea, and USA. Given the horizontal transfer of *C. parapsilosis* and the propensity of Y132F mutants to colonize the hospital environments, their prompt identification followed by application of appropriate infection control strategies are of importance.

**Methods:** During 10 years (2009-2019), 228 non-duplicate isolates of *C. parapsilosis* were recovered from the blood samples of patients admitted to Ege University Hospital, Izmir, Turkey. All isolates were primarily identified by API 20C AUX and re-identified via MALDI-TOF MS. Regardless of fluconazole MIC values, *ERG11* sequencing was performed for all 228 isolates. In the next step, *MRR1, TAC1,* and *UPC2* sequencing was performed for 90 isolates containing all resistant isolates with or without *ERG11* Y132F mutation and randomly selected susceptible dose-dependent and susceptible isolates.

**Results:** Surprisingly, Y132F was the most predominant mutation in *ERG11* (*n* = 46, 20.2%), followed by K143R (*n* = 21, 9.2%), and G458S (*n* = 6, 2.6%), all implicated in fluconazole resistance. Moreover, Q250K and G307A were two newly suggestive mutations exclusively occurred in fluconazole resistant isolates. Among isolates harboring *ERG11* Y132F, 43.5%, 17.3%, and 10.8% of isolates carried nonsynonymous mutations in *UPC2, TAC1*, and *MRR1*, which is inconsistent to what was previously reported from a South Korean study. The first isolate carrying Y132F mutation emerged in 2012 and isolates carrying this mutation persistently recovered from the blood samples onwards (2012-2019). Strikingly, the number of isolates harboring *ERG11* Y132F, doubled and increased 5 fold between 2009-2015 and 2009-2019, respectively (Figure 1). Future studies are ongoing to determine the corresponding genotype of each isolate using microsatellite typing and the antifungal susceptibility data for voriconazole, micafungin, and anidulafungin remain to be completed.

**Conclusion:** We observed a significant and persistent isolation of emerging *ERG11* Y132F fluconazole-resistant *C. parapsilosis* isolates, which is a worrisome complication in our hospital requiring implementation of typing and sequencing of target genes followed by establishing appropriate infection control strategies to confine further spread of such isolates. The widespread use of azoles in clinical settings, especially developing countries, highlights the importance of worldwide surveillance studies to evaluate the prevalence and spread of *ERG11* Y132F isolates. Figure 1. Accumulation of *ERG11* Y132F mutation in *C. parapsilosis* isolates over 10 years of study from the blood samples of patients admitted to Ege University Hospital, Izmir, Turkey.

### P212. Candida auris: Epidemiology, Risk Factor Analysis and Management of Invasive Infections in Tertiary Care Hepatobiliary Centre

KaleP.^1^KhillanV.^1^SarinS.K.^2^1Clinical Microbiology, Institute of Liver and Biliary Sciences, New Delhi, India2Hepatology, Institute of Liver and Biliary Sciences, DELHI, India

**Objectives:** *Candida auris* involvement in candidemia and other deep-seated invasive infections is associated with very high mortality. The inability of several available commercial identification systems to quickly diagnose *C. auris* remains a challenge to early therapy. The data is limited especially in patients with hepatobiliary diseases, in whom the risk factors and mangement differs due to the risk of antifungal toxicity. We aimed to study the epidemiology, risk factors and therapeutic management of invasive infections caused by *C. auris in patients with hepatobiliary diseases.*

**Methods:** Single-center, prospective study of patients with suspected invasive fungal infections between January2017-December 2018. the patients with hepatobiliary diseases were included inthe study based on vlinical diagnosis and radiological features. Demographics, comorbidities, and laboratory variables were recorded. The samples recieved in the microbiology laboratory as a part of clinical diagnostic protocols were processed accordingly. The positive yeast cultures were subjected to identification by Vitek 2(Biomerieux, India) with updated software using YST cards, and antifungal sensitivity confirmed by Broth microdilution in accordance with CLSI guidelines. The final outcome considered were mortality within one month after diagnosis or discharge of the patient with stable parameters.

**Results:** Total of 109 isolates of C*. auris* from 55 patients, blood 14(12.8%), abdominal fluids 14(12.8%), urine 58(53.21%), respiratory 4(3.6%), liver abscess 2 (1.83%), pancreatic abscess 1(0.9%) and wound infections 3 (2.75%). Underlying disease was chronic liver disease 37 (68%), 10 (20%) post liver transplant patients, acute on chronic liver failure 4 (7%), acute liver failure 2 (3%), acute pancreatitis 1 (1.8%) and pancreatic neuroendocrine tumor 1 (1.8%). 14 (25%) patients were discharged, mortality was 41 (74.04%). Risk factors were MELD 40 (p0.04), Child C (p 0.05) and CTP score above 12. Prior use of steroids (p 0.02), neutropenia (p 0.03), prolonged hospital stay (0.029), use of broad spectrum antibiotics more than 7 days (p 0.05) were the risk factors significantly associated with development of *Candida auris* infections and higher mortality. Co-morbidities, acute renal failure, diabetes, hepatitis infection were not significantly associated with mortality. The antifungal resistance: fluconazole 68.42%, voriconazole 14.03%, flucytosine 59.64%, amphotericin B 28 % with no resistance to caspofungin and micafungin.

**Conclusion:** *C.auris* infections are associated with misidentification, intrahospital transmission, poor treatment outcomes and higher mortalities. Prompt detection, earlier initiation of therapy and effective surveillance can curtail *C. auris* in hospitals. Our study depicts the spectrum of invasive infections caused by *C. auris*, its prevalence, risk factors and therapeutic options. The presence of risk factors like steroid use, neutropenia, broad spectrum antibiotic use and hospital stay of more than 7 days should prompt towards escalating diagnostic measures for rapid identification of *C.auris* for early initiation of therapy. Active screening of patients with risk factors can also reduce mortality. The study results also help to guide empiric therapy with echinocandins as azoles and amphotericin B show high resistance in these isolates. This study will guide prophylactic and therapeutic options in hepatobiliary disease patients with underlying risk factors to reduce mortality.

### P213. Lignin degradation pathway by Scedosporium species

PoirierW.GiraudS.BoucharaJ.-P.Host-Pathogen Interaction Study Group, SFR ICAT 4208, Univ Angers, Univ Brest, Institute of Biology in Health, IRIS, University Hospital Center, Angers, France, Angers Cedex, France

**Objectives:** *Scedosporium* are usually soil saprophytes. Among the ten species recognized within the genus, three have been regularly reported as causing human infections, particularly in patients with cystic fibrosis (CF): *Scedosporium apiospermum*, *Scedosporium aurantiacum* and *Scedosporium boydii*. Because of their low sensitivity to almost all available antifungal drugs, a better understanding of the pathogenic mechanisms of these fungi is required in order to identify new therapeutic targets. Likewise, identification of the origin of the contamination of patients may be helpful to propose prophylactic measures. In this aim, environmental studies were conducted on the ecology of these opportunistic molds. *Scedosporium* species are abundant in human-made environments, in relation with specific traits of the fungi: ability to survive at low oxygen pressure and to degrade hydrocarbons. Recent studies have demonstrated the correlation between the lignocellulolytic properties of filamentous fungi and their ability to degrade environmental organic pollutants such as aromatic hydrocarbons. Among the lignocellulosic compounds, the particular properties of lignin make it an interesting study molecule. In this context, we attempted to elucidate the mechanisms developed by *Scedosporium* species to degrade this polyphenolic molecule, with a special emphasis on the opening of aromatic rings.

**Methods:** Culture in specific media and biochemical analyses were performed to characterize *Scedosporium* properties. Orthologue enzymes from the literature were used to screen *Scedosporium* genomic data by tBLASTn (Basic Local Alignment Search Tool) searches and real-time PCR experiments were then conducted to analyze the relative expression of these candidate genes.

**Results:** The lignolytic properties of *Scedosporium* species were first confirmed by cultivation of the fungi on agar media containing the different components of lignocellulose as carbon source. According to literature, the early steps in the lignin degradation pathway involve peroxidases and oxidases. Biochemical analyses allowed us to detect these enzyme activities in the culture supernatant of these fungi grown in lignin-containing media. Fifteen ortologue genes were identified by tBLASTn searches, four encoding members of the peroxidase family and the others encoding oxidase. However real-time PCR experiments revealed that only four of these genes encoding peroxidases (2) and oxidases (2) were over-expressed in the presence of lignin. Based on an exhaustive bibliography, at least three different pathways, *i.e* the β-ketoadipate, the oxoadipate and the gentisate pathways, were identified as potential routes for the degradation of molecules resulting from the early oxidation steps and the opening of the aromatic rings. In all *Scedosporium* species, enzymes involved in the gentisate pathway are encoded by genes organized into a six membered gene cluster as revealed by bioinformatic analyses, and real-time PCR experiments demonstrated the over-expression of all these genes in culture media containing dihydroxybenzoate.

**Conclusion:** Results obtained in this study confirm that *Scedosporium* species exhibit the enzymatic arsenal to degrade natural complex molecules and are capable to open aromatic rings. This may explain their presence in polluted environments, but one may also consider these enzymes as virulence factors since it has been suggested a link between the ability to use aromatic compounds and the propensity of these fungi to cause cerebral infections in receptive hosts.

### P214. Fusarium keratitis with poor prognosis: a report of four cases.

ChouaiebH.^1^YaacoubA.^1^MiliW.^2^IsmailS.^1^GhorbelM.^2^FathallahA.^1^1Parasitology Mycologie Laboratory, Farhat Hached university hospital, Sousse, Tunisia2Ophthalmology Department, Farhat Hached University Hospital, Sousse, Tunisia

**Case Report:**

**Introduction and objective** Keratitis caused by *Fusarium* species, a sight-threatening disease, is difficult to treat because *Fusarium spp* are highly resistant to most antifungals. Our objective was to describe four cases of Fusarium keratitis with poor prognosis. 

**Methods** Direct microscopic examination of corneal scrapings and culture on Sabouraud-chloramphenicol medium were performed. The identification of fungi was based on macroscopic and microscopic characteristics of culture. 

**Results** All patients were male and the median age was 53, 5 years (range: 38-70). The predisposing factors were corneal trauma by a vegetative matter in three cases (75%), past history of herpetic keratitis in one case (25%) and steroid use in one case (25%). Direct microscopic examination was positive in 75% of cases and culture was positive in all cases. Culture grew *Fusarium solani* (50%) and *Fusarium solani/Fusarium chlamydosporium* (50%). It is important to mention that the two patients with *Fusarium solani/Fusarium chlamydosporium* keratitis were hospitalized in the same room; the first one was referred to ophthalmology department for *Fusarium* keratitis, when the second one developed the infection during his hospitalization. On the other hand, the antifungal susceptibility of the two strains were identical, so, it is quiet likely that the second patient was contaminated by the first one. Three patients underwent surgery and received antifungal therapy. One patient was treated by topical Amphotericin B associated to oral Itraconazole, the second received topical Amphotericin B, topical Betadine and oral Voriconazole and the third patient was treated by diluted Betadine, topical and oral Voriconazole. The fourth patient was lost to follow-up; he was discharged against medical advice. In most cases, the outcome was adverse. 

**Conclusion** Keratomycosis is a severe disease that can lead to severe visual loss and even loss of the eye. Prompt diagnosis and early treatment are needed to avoid complications.

### P215. Candida blankii as an emerging pathogen of cystic fibrosis patients

BorzovaY.^1^VoroninaO.^2^RyzhovaN.^2^KundaM.^2^AksenovaE.^2^SimonovaO.^3^DavletgareevaD.^4^SuslovaI.^4^BogomolovaT.^1^SkorbunovaO.^4^KlimkoN.^5^VasilyevaN.^4^1Department of Medical Microbiology, North-Western State Medical University named after. I.I. Mechnikov, Sant-Petersburg, Russian Federation2N.F. Gamaleya National Research Center of Epidemiology and Microbiology, Ministry of Health of Russia, Moscow, Russian Federation3National Medical Research Center for Children’s Health, Ministry of Health, Moscow, Russian Federation4Kashkin Research Institute of Medical Mycology, North-Western State Medical University named after. I.I. Mechnikov, Sant-Petersburg, Russian Federation5Department of Clinical Mycology, Allergy and Immunology, North-Western State Medical University named after. I.I. Mechnikov, Sant-Petersburg, Russian Federation

**Objectives:** *Candida blankii* is emerging pathogen for children, and can be important for patients with cystic fibrosis (CF). Publications about *C. blankii* in CF patients are limited.

**Methods:** Tracheal aspirate and sputum of 255 CF children 0-18 years old were analyzed by DNA extraction, amplification of ITS1-5.8SrDNA-ITS2 fragments of Fungi genomes, Sanger sequencing and identification of sequences using BLAST NCBI. Massively parallel sequencing of amplicons based on the MiSeq Illumina platform was used for some samples. Bioproject PRJNA545010 was published in GenBank.

**Results:** *C. blankii* was revealed in the samples of two patients (P). P1 was 12 years old when *C. blankii* was detected in microbiome containing *Pseudomonas aeruginosa*, *Mycobacteroides abscessus*, but dominating bacteria were *Streptococcus* spp. The month later *C. blankii* was replaced by *C. sake*. In response to the therapy microbiome cleared of *M. abscessus*, but saved *P. aeruginosa.* The year later fungi and *P. aeruginosa* were not revealed in the low airway sample of P1. P2 has *C. albicans* and *P. aeruginosa* in the low airway sample at 7 years. Dominating bacteria were *Streptococcus* spp. At 8 years, when P2’s conditions worsened, *C. blankii*, *P. aeruginosa* and *Capnocytophaga* spp. as main bacteria were revealed. Active therapy eliminated *P. aeruginosa,* but did not influence on *C. blankii*. The year later C. blankii accompanied *Staphylococcus aureus* and *P. aeruginosa* in P2 microbiome.

**Conclusion:** Repeated isolation of *C. blankii* from low airway samples of CF patient suggests the possibility of respiratory tract colonization by *C. blankii.*

### P216. The mortality attributable in Candidemia to C. auris is higher than other Candida species: Myth or or reality?

MorenoC. Alvarez^1^Morales-LópezS.E.^2^RodriguezG.J.^3^RodriguezJ.Y.^3^RobertE.^4^PicotC.^5^Parra-GiraldoC.M.^6^PapeP. Le^7^1Universidad Nacional De Colombia, Bogotá, Clínica Universitaria Colombia, Colsanitas, Colombia, bogota, Colombia2Universidad Popular del Cesar, Valledupar, Colombia, valledupar, Colombia3Cesar, Cimce, Cl. c #d -, Valledupar, Cesar, Colombia4Ea1155 Iicimed, Nantes University Hospital, NANTES, France5Pontificia Département de Parasitologie-Mycologie, Université de Nantes, Nantes Atlantique Universités, EA1155-IICiMed, Faculté de Pharmacie, Nantes, France, Dantes, France6Unidad de Proteómica y Micosis Humanas, Grupo de Enfermedades Infecciosas Departamento de Microbiología, Facultad de Ciencias, Pontificia Universidad Javeriana, Bogotá D.C, Colombia, Bogota, Colombia7Ea1155 Parasitology and Mycology Department, Nantes University, Nantes, France

**Objectives:** *Candida auris* has emerged as a major threat in the healthcare setting, having caused invasive infections outbreaks around the world. Besides, it has been proved difficult to treat due to its multidrug-resistant nature and mortality rates have varied significantly among *30-60*% in candidemia (1-2). However, the overall attributable mortality rate related to candidemia is unclear, nor is there any difference with other candida species (*Candida non-auris*). We investigated epidemiological, clinical and laboratory features of patients with candidemia to evaluate the mortality by C. *auris* or C. non-auris.

**Methods:** A case-control study was conducted between 2013 and 2017 in 12 hospitals of Valledupar, Colombia comparing 22 cases of *C. auris* candidemia with 52 of *C. non-auris*. All isolates of candidemia were recovered to confirm identification by automated matrix-assisted laser desorption/ionization time of flight (MALDI-TOF MS) (Bruker Daltonik, Bremen, Germany) and susceptibility to widely used antifungals was determined with Sensititre Yeast One® AST plates (Thermo Fisher Scientific). Appropriate antifungal therapy was defined according to the antimicrobial susceptibility tests, for the case of *C. non-auris* the CLSI M27-A3 breakpoints were used, in the case of AmB, resistant isolates were defined as isolates with MIC> 1 μg / mL. For *C. auris*, breakpoints recommended by the CDC were used. The risk factors, clinical and microbiological characteristics and outcomes of patients with C. *auris* and C. non-auris candidemia (CAC) were compared. Odds ratio and corresponding 95% confidence intervals were calculated using EPI Info 7™.

**Results:** Seventy four patients of candidemia were enrolled. Total number of 22 cases (29.7%) and 52 controls (*C. albicans*, 21.6%; *C. parasilopsis*, 21.6%; *C. tropicalis*, 21.6%; *C. glabrata*, 1.4% ) were included and analyzed in this study. The comparison of the main clinical and epidemiological variables is summarized in Table 1. Previous fluconazole exposure was significantly higher in C. *auris* candidemia patients (OR 3.3: 1.15-9,5). *In vitro* antifungal susceptibility of *Candida* species isolates is presented in Table 2. Most C. auris isolates were resistant to fluconazole (86.3%) and amphotericin B (59%) while C. non-auris isolates were in general susceptible (fluconazole resistant, 1.9%; Amphotericine B resistant, 3.8%). No isolates resistant to echinocandins were detected. The average time to start antifungal therapy was 3.6 days. Sixty three (85.1%) patients received adequate antifungal therapy, without significant differences between the two groups (*C. auris* Vs *C. non-auris*; 86.4%vs 84.6%, respectively). The crude mortality at 30 and 90 days of candidemia was up to 37.8% and 40.5%, respectively. However, there was no difference in mortality both at 30 and 90 days, between the group with candidemia by C. *auris* and by C. non-auris, 31.8% vs 42.3% (OR 0.6; 95% IC 0.24-1.97) and 36.4% and 42.3% 0,77 (0,27-2,1).

**Conclusion:** Mortality due to candidemia is similar between C. *auris* and C. non-auris *in* this study. Appropriate antifungal therapy in both groups could contribute to finding no differences in outcomes. A high resistance to Anfo B was evidenced as previously described in Colombia(3).

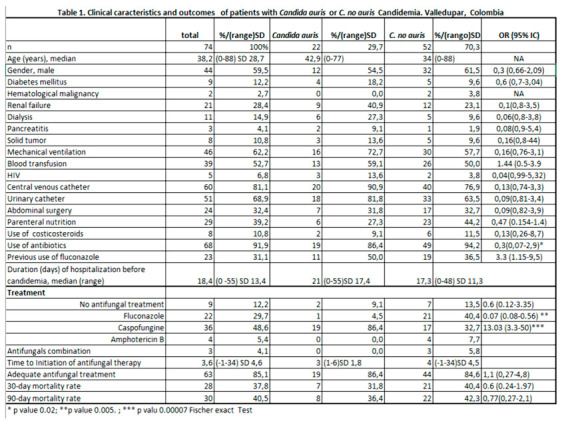

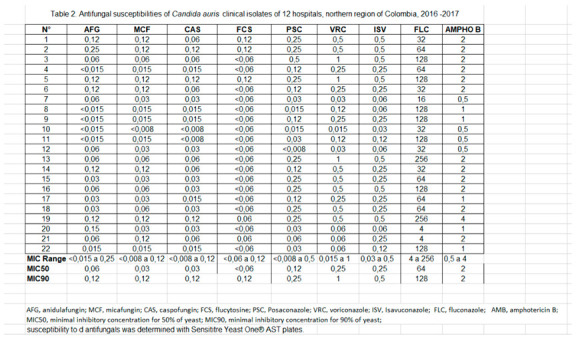





### P217. Genetic characteristic and susceptibility profiles of Candida auris clinical isolates from Russia

VasilyevaN.^1^PineginaO.^2^VybornovaI.^1^ChilinaG.^3^ApalkoS.^4^ScherbakS.^4^PchelinI.^5^TaraskinaA.^5^1Kashkin Research Institute of Medical Mycology, North-Western State Medical University named after I.I.Mechnikov, St. Petersburg, Russian Federation2Pavlov First Saint Petersburg State Medical University, Saint Petersburg, Russian Federation3Kaschkin Research Institute of Medical Mycology, North-Western State Medical University named after I.I. Mechnikov, Saint-Petersburg, Russian Federation4City Hospital No. 40, Saint Petersburg, Russian Federation5Kashkin Research Institute of Medical Mycology, North-Western State Medical University named after I.I. Mechnikov, Saint Petersburg, Russian Federation

**Objectives:** The yeast *Candida auris* is an emergent nosocomial pathogen. In October, 2017, the first case of candidaemia due to *C. auris* in Russia has been diagnosed. A large series of isolates was published from Moscow. However, the source of the pathogen and its spreading to other Russian cities remained obscure. Our objective was to provide genetic characteristic and describe antifungal drug susceptibility profiles of *C. auris* clinical isolates from Russia.

**Methods:** For the study, a total of six *C. auris* strains were available. Four originated from Moscow and two originated from St. Petersburg. We identified isolates by ribosomal ITS region sequencing. Whole genome sequence of one isolate was obtained by Illumina MiSeq technology. Antifungal susceptibility testing was performed using colorimetric Sensititre YeastOne YO10 broth dilution panels.

**Results:** For the first Russian *C. auris* isolate from Moscow, we obtained whole genome sequence. It clustered within Indian/Pakistani clade with isolates from Indian subcontinent. The earliest *C. auris* isolate from outside Moscow was obtained in St. Petersburg in March, 2019. All six studied isolates, including two strains from St. Petersburg, harbored exactly the same ITS region genotype (MK829041). Also, antifungal drug susceptibility profiles were highly similar. All isolates were resistant to fluconazole, but susceptible to echinocandins and amphotericin B.

**Conclusion:** Currenty, *C. auris* is being detected in two Russian cities. The spreading clone has Indian/Pakistani origin, probably independent from known outbreaks outside Indian subcontinent.

### P218. Candida auris in China

LiangG.Department of Medical Mycology, Institute of Dermatology, Chinese Academy of Medical Science, Nanjing, China

**Objectives:** Since 2009, *Candida auris* has been spreading globally, which is a serious threat to human health and has attracted the international attention. We analyzed the epidemiological trend and pathogenic characteristics of Candida auris in China and try to discover the problem behind and put forward the reasonable strategies to provide useful information for prevention and treatment of the diseases.

**Methods:** We reviewed all Candida auris cases reported from China by searching Pubmed, EMbase, Wanfang and CNKI; also deeply speculated the hidden clinical problems in China.

**Results:** First, there are more than 19 cases has been recorded in China. Second, immunosuppressor and Long-term use of broad-spectrum antibiotics may be the high risk. Thirdly, fifteen cases was reidentified as Candida auris by PCR and sequencing the ITS and D1/D2 regions which was misidentified as C. haemulonii by Vitek. Fourth, both Azole-resistant and non-Azole-resistant Candida auris was found. Fiveth, the infection by Candida auris can not be effective diagnosed and identified timely 3 years ago in the most third-grade class-A hospital in China, but now it is being improved because of popularized MALDI-TOF equipment.

**Conclusion:** The situation of Candida auris infection may be much severer than expected because of the misidentification especially in high-risk groups and more attention has been paid and better medical conditions has been strengthened for the Challenges ahead.

### P219. Candida auris Candidemia in a Tertiary Care Centre in North India: Rapid Identification from Direct Blood Culture Positive Samples by MALDI-TOF MS and Analysis of Risk Factors

MarakR.S.K.^1^YadavS.^2^DixitA.K.^2^1Microbiology, Sanjay Gandhi Postgraduate Institute of Medical Sciences, Lucknow, India2Microbiology, sanjay Gandhi Postgraduate Institute of Medical Sceinces, Lucknow, India

**Objectives:** To identify the *Candida auris* from direct positive blood cultures by MALDI-TOF MS and analyse the risk factors associated with this multidrug resistant yeast- *Candida auris*

**Methods:** A prospective study was conducted from February 2017 to January 2018 by taking 8 ml of positive blood cultures. After multiple washings and treating with triton X, it was directly subjected to the MALDI-TOF MS assay. The clinical data of candidemia cases due to *C. auris* were determined for significant associated risk factors with *C. auris* infection.

**Results:** of the 122 candidemia cases reported during the study, 23 (18.85%) were due to *C. auris*; being the 3^rd^ most common species after *C. tropicalis* and *C. parapsilosis*. The age ranged from 4-day old neonate to 80 years old and there was female preponderance (*n* = 13). MALDI-TOF MS was able to rapidly identify all *Candida auris* species on the same day when the blood culture was flagged positive thereby shortening the time for species identification by MALDI-TOF MS from culture. The underlying risk factors significantly associated with *C. auris* candidemia were diabetes mellitus, underlying respiratory illness, gastrointestinal surgery, prior antifungal exposure. Mortality was seen in 60.8%. All isolates were resistant to fluconazole (MIC ≥ 64µ/ml).

**Conclusion:** *Candida auris* is a recently described agent of fungemia that poses a serious global health threat. *C. auris* infection has been observed across India and notable for its antifungal resistance; often multidrug-resistant. *C. auris* is difficult to identify; polymerase chain reaction, DNA sequencing and MALDI-TOF MS can correctly identify it. *C. auris* known to cause outbreaks in healthcare settings and therefore, it is important to quickly identify *C. auris* in a hospitalized patient and the healthcare facilities can take special precautions to prevent its spread.

### P220. rEpidemiology of Cryptococcal antigenemia among HIV infected patients in southwestern, Nigeria 2018

OdegbemiO.B.^1^Dada-AdegbolaH.O.^2^AdeoyeI.^1^FayemiwoS.A.^3^1Epidemiology and Medical Statistics, University of Ibadan, Ibadan, Nigeria2Medical Microbiology and Parasitology, University of Ibadan, Ibadan, Nigeria3Medical Mycology Unit, Division of Infection, Immunity and Respiratory Medicine, School of Biological Sciences, University of Manchester, Manchester, United Kingdom

**Objectives:** Cryptococcal antigenemia occurs in Nigeria, but the magnitude of this disease remains unclear. This study was carried out i. To determine the prevalence of Cryptococcal antigen (CrAg) among HIV-seropositive subjects ii. To determine the prevalence of CrAg among HIV-seronegative subjects and iii. To assess the relationship between CD4 count and CrAg in HIV-seropositive subjects attending Adeoyo Maternity Teaching Hospital, Yemetu, Ibadan

**Methods:** It was hospital based case-control study using simple random sampling. Semi-structured interviewer administered questionnaire was used to collect data from subjects and retrospective review of CD4 count records in HIV infected subjects. Five millilitres venous blood was collected from each participant. Serum CrAg testing was done using CrAg Lateral Flow Assay. Data was analyzed using descriptive statistics and bivariate analysis at 5% confidence level.

**Results:** One hundred and fourteen HIV-seropositive individuals (cases) and 228 HIV-seropositive individuals (controls) were recruited. Mean age of cases was 41.2 ± 10.0 years and 85 (74.6%) were females while mean age of controls was 38.9 ± 13.7 years 156 (68.4%) were females. The prevalence of CrAg among cases was 11.4% and 7.0% among controls. Cases were about two times more likely to test positive for CrAg. However, the association was not statistically significant (OR: 1.71, 95%CI: 0.79 - 3.68). Individuals with CD4 counts of ≤100 cells/µl were 20 times more likely to have positive serum cryptococcal antigen than individuals with CD4 counts >100 cells/µl (OR: 20.3, 95%CI: 5.23-78.9).

**Conclusion:** This study demonstrated significant prevalence of Cryptococcal antigenemia among the study population; however, prevalence was higher among cases. Screening for CrAg should therefore be part of routine tests amongst all confirmed HIV-seropositive subjects, since asymptomatic cryptococcal antigenemia predicts impending cryptococcal infection with probable mortality.

### P221. High prevalence of Fusarium proliferatum isolates from onychomycosis and antifungal susceptibility pattern

HaghaniI.^1^Shams-GhahfarokhiM.^2^AslA. Dalimi^3^AbastabarM.^4^ShokohiT.^4^HedayatiM.T.^4^FathiM.^3^1Medical Mycology, Mazandaran university of Medical sciences, Sari, Iran2Department of Mycology, Faculty of Medical Sciences, Tarbiat Modares University, Tehran, Iran3Department of Parasitology, Faculty of Medical Sciences, Tarbiat Modares University, Tehran, Iran4Department of Medical Mycology, Invasive Fungi Research Center, School of Medicine, Mazandaran University of Medical Sciences, Sari, Iran, Sari, Iran

**Objectives:** Onychomycosis refers to any fungal infection of the nail and is usually caused by dermatophytes; however, non-dermatophyte molds (NDM) and yeasts are increasingly recognized as the pathogens accounting for nail disease. Regarding this, the present study was conducted to describe the molecular epidemiology of *Fusarium* onychomycosis in the North of Iran.

**Methods:** To this end, 257 nail samples collected from the Iranian patients clinically suspected of onychomycosis were subjected to direct microscopy, calcofluor white staining, and fungal culture. The characteristics of *Fusarium* isolates were further identified at a species level by determining multi-locus sequences for internal transcribed spacer and translation elongation factor 1 alpha

**Results:** According to the results, *Fusarium* species were isolated from 27 patients with onychomycosis. Based on previous partial genes analysis, the recognized species in our study were among the members of *F. fujikuroi* species complex (*n* = 14), *F. solani* species complex (*n* = 12), and *Fusarium incarnatum-equiseti* species complex (*n* = 1). In the present study, *F. proliferatum* was the dominant *Fusarium* species isolated from the samples. With regard to the increased prevalence of *Fusarium* onychomycosis and the intrinsic resistance of these agents to a broad range of antifungals, it is necessary to correctly identify *Fusarium* species. Our results were indicative of a change in the epidemiology of *Fusarium* species isolated from onychomycosis. The minimum inhibitory concentration (MIC) of luliconazole and lanoconazole ranged within 0.001 to - 1 μg/mL and their geometric mean MICs were 0.0103 and 0.0343 μg/mL against *Fusarium* species, respectively.

**Conclusion:** The increased prevalence of *Fusarium* onychomycosis and the intrinsic resistance of these infectious agents to a broad range of antifungals have highlighted the importance of the accurate identification of *Fusarium* species. According to the results of the present study, it seems that the epidemiology of *Fusarium* species isolated from onychomycosis has been changed. In addition we conclude that luliconazole and lanoconazole demonstrate potent activity against *Fusarium* species isolated from onychomycosis. These compounds are accordingly promising for the treatment of superficial fusariosis such as onychomycosis.

### P222. Fungal disease burden: an underestimated health challenge in Cote d’Ivoire

KoffiD.^1^IraB.^1^ToureA.O.^1^JambouR.^1^DenningD.^2^1Parasitology and Mycology, ABIDJAN, Côte d’Ivoire2University of Manchester, Manchester, United Kingdom

**Objectives:** Due to limited access to more powerful diagnostic tools, there are few data on the burden of fungal infections in Cote d’Ivoire, despite a high HIV and TB burden and many cutaneous diseases. Here we estimate the burden of serious fungal infections in this sub-Saharan country.

**Methods:** National demographics were used to perform a PubMed search and retrieve all published articles on fungal infections in Cote d’ivoire and countries bordering West Africa. When no data existed, risk populations were used to estimate frequencies of fungal infections, using previously described methodology by LIFE (www.LIFE-Worldwide.org).

**Results:** The population of Cote d’ivoire is around 24 millions; 37% are children, and 9% are >65 years. Tinea capitis in children is common, measured at 13,9% in the last epidemiological study (2013). Considering the prevalence of HIV infection (2.7% of the population, a total of ~500,000) and a hospital incidence of 6% of cryptococcosis, it is estimated that 3726 patients per year develop cryptococcosis. For pneumocystosis, it is suggested that 6023 new cases occur each year with the pevalence of 14,1% in paediatric HIV infection. An estimated 1567 new cases of chronic pulmonary aspergillosis occur after pulmonary tuberculosis (a 5 year prevalence of 4938 cases (20.3/100,000). Allergic bronchopulmonary aspergillosis (ABPA) and severe asthma with fungal sensitisation (SAFS) were estimated in 104/100,000 and 151/100,000 respectively, in 1,100,000 adult asthmatics. Vulvovaginal candidiasis (VVC) is common and recurrent VVC affects ~6% of women in their fertile years - 407,000 women. An unknown number develop candidaemia and invasive aspergillosis. There are no incidence data on fungal keratitis, histoplasmosis and chromoblastomycosis, although some cases of mycetoma and histoplasmosis have been reported.

**Conclusion:** The present study indicates that around to 6.8% (1.6 million) of the population is affected by a serious fungal infection, predominently tinea capitis in children and rVVC in women. These data should be used to inform epidemiological studies, diagnostic needs and therapeutic strategies in Cote d’Ivoire.

### P223. Frequency and molecular epidemiology of Aspergillus isolated from patients with suspicion of respiratory fungal infection

OliveiraM.^1^,^2^SimõesH.^3^VerissimoC.^1^SabinoR.^1^1Infectious Diseases, National Health Institute Dr. Ricardo Jorge, Lisbon, Portugal2Animal Biology Department, Faculty of Sciences of the University of Lisbon, Lisbon, Portugal3Infectious Diseases, National Health Institute, Lisbon, Portugal

**Objectives:** The aim of this study was to determine the frequency of *Aspergillus* detected in respiratory samples from a cohort of patients with suspicion of fungal infection of the respiratory tract as well as to determine the susceptibility to azoles of the isolates from the *Fumigati* section.

**Methods:** A retrospective study was performed involving samples obtained from 16 hospitals covering different districts of continental Portugal and Azores islands. One hundred and eighty-seven respiratory samples (101 bronchoalveolar lavage fluids, 52 bronchial lavages, 27 bronchial secretions, 6 expectorations and 1 bronchial aspirate) were collected between November 2011 and December 2017 from a cohort of 146 patients with suspicion of respiratory fungal infection (ages ranging from 20 to 87 years old). Demographic and clinical data were recorded. Detection of *Aspergillus* was done by culture, immunoenzimatic assay and/or molecular techniques. *Aspergillus* molecular identification to species level was performed by sequencing of the calmodulin and β-tubulin genes. To detect possible resistance to azoles, isolates belonging to section *Fumigati* were inoculated into Sabouraud dextrose agar media supplemented with 1 µg/ml or 4 µg/ml of voriconazole, 4 µg/ml of itraconazole and 0.5 µg/ml of posaconazole and their growth was observed and recorded after 7 days of incubation at 27ºC. Doubtful results were confirmed when possible by E-test and by real-time multiplex PCR for the detection of mutations in the Cyp51A gene.

**Results:** Fifty-seven (39.0%) of the studied patients were positive for *Aspergillus*. From the cases with a positive culture (*n* = 58) the species were identified by sequencing and belonged to six different sections. The most frequently isolated was the section *Nigri* (42.1%) followed by the *Fumigati* (33.3%) and *Flavi* sections (8.6%). Regarding the species, the most frequent was *A. niger sensu stricto* (33.9%) followed by *A. fumigatus sensu stricto* (32.1%). Nine cryptic species were also identified which frequency was 21.4%. In order to study the frequency of azole resistance in *Fumigati* isolates collected from the samples of this cohort as well from other biological products, 52 isolates - *Aspergillus fumigatus* sensu stricto (*n* = 45), *A. lentulus* (*n* = 4), *A. udagawae* (*n* = 2) and *A. pseudofelis* (*n* = 1) – were tested. The tested *A. fumigatus sensu stricto* isolates did not show resistance to azoles. An *A. udagawae* strain revealed low susceptibility to voriconazole (MIC was not determined due to loss of strain viability). An *A. pseudofelis* strain also showed decreased susceptibility to voriconazole (MIC = 1 μg/ml) as well as to and itraconazole (MIC = 2 μg/ml).

**Conclusion:** In this study, the genus *Aspergillus* was frequently isolated in the respiratory samples tested and a high number of cryptic species was detected. Although resistance to azoles was not a problem identified in the tested isolates, determination of the *in vitro* susceptibility profile and molecular identification of the *Aspergillus* species is essential to improve the diagnosis and management of aspergillosis since several cryptic species have intrinsic resistance to antifungal drugs.

### P224. Epidemiological analysis of a large Tinea epidemic in schools of a city in the northern suburbs of Paris, France

BrunS.VingataraminY.AkhoundiM.LintanfM.BruelC.IzriA.Parasitology-mycology, Avicenne Hospital, Bobigny, France

**Objectives:** The main goals of this investigation were i) to identify the causative agents of a large *Tinea* epidemic in several schools of a city in the northern suburbs of Paris, France, and ii) to determine the epidemiological factors which played an important role in dissemination of this dermatophyte infection.

**Methods:** Following a report to the Parasitology-Mycology Department of Avicenne hospital (Bobigny, France) regarding a possible *Tinea* epidemic in two schools of Clichy-sous-Bois city (northern-suburbs of Paris), all the children of these schools were screened for dermatophytosis by clinical and mycological examinations. Some children from other schools of this city and members of their families came to the same laboratory for dermatophytosis screening. Suspected lesions of hair or skin were sampled by scraping and swabbing for mycological diagnosis and demographic data were recorded in parallel. A dermatophytosis case was determined by positive direct microscopic examination (presence of fungal filaments or spores in hair or scales) and/or positive dermatophyte culture. A map of the cases repartition was realised. Next, in order to evaluate the intra- and interspecific genotypic relationships among *T. tonsurans* isolates, they were subjected to sequencing of two fragments of the alkalin protease 1 (*ALP1*) gene. The obtained sequences were aligned using the Basic Local Alignment Search Tool (BLAST) and compared to the sequences deposited in GenBank.

**Results:** During 15 months, 122 individuals (coming from 12 schools of the city and members of their families) were sampled and 139 samples were analysed for mycological direct microscopic examinations and mycological cultures. Ninety-three (70%) samples (79 scalp and 14 skin samples) were positive for dermatophyte culture or direct examination in 82 (67%) individuals. *Trichophyton tonsurans* was isolated in 60 samples (87%), followed by *Microsporum audouinii* (4 isolates), *Trichophyton rubrum* (4), *Trichophyton soudanense* (1) and *Microsporum canis* (1). The infected individuals had an age ranging from 3 months to 53 years with an average of 8 years old. All of these infected individuals were living in houses that, together with their schools, are located in an area of less than 1 km^2^. Most of them were originated or had parents coming from sub-Saharan Africa, followed by Haiti and North Africa. According to the sequence analysis of *ALP1* gene, the *T. tonsurans* isolates all shared the same nucleotide sequences, but were different from the only sequence available in GenBank coming from USA.

**Conclusion:** This study presents likely the largest *Tinea* epidemic recorded not only in France but also in Europe, with a large majority of infections caused by *T. tonsurans*. The genotypic analysis of the *T. tonsurans* isolates revealed the circulation of a unique strain in this city of the northern suburbs of Paris, distinct from the strain described in USA. The high population density together with the high poverty rate of this city are of the major factors, beside the high transmission rate of *T. tonsurans*, that have facilitated the transmission of the dermatophytes between the individuals in a same family and a same district, as well as in schools.

### P226. Presence of Malassezia furfur in the cutaneous microbiome of preterm infants in a Neonatal Intensive Care Unit

MoreiraR.T.F.^1^SilvaD.P.^1^MaranhãoF.C.A.^2^MoreiraI.F.^3^FariasF.I.R.^3^AlvarezJ.^4^CoutinhoS.D.A.^4^1Nursery School, Federal University of Alagoas, Maceió, Brazil2Biological Sciences Institute, Federal University of Alagoas, Maceió, Brazil3Medicine Faculty, Federal University of Alagoas, Maceió, Brazil4Post-graduation Program In Environmental and Experimental Pathology, Paulista University, São Paulo, Brazil

**Objectives:** In humans, the colonization of the skin by yeasts belonging to the genus *Malassezia* starts from the birth. They usually live in commensalism but may become pathogenic depending on predisposing factors and may cause superficial mycoses. Although in a lower percentage, *M. pachydermatis* and *M. furfur* are responsible for serious invasive infections in man. In outbreaks of fungemia reported in neonatal intensive care units (NICU), the neonates showed prematurity, low birth weight, multiple comorbidities and parenteral nutrition with lipid supplementation. The aim of this study was to investigate the occurrence of *Malassezia* spp. in the cutaneous microbiome of low and extremely low birth weight neonates hospitalized in a neonatal intensive care unit.

**Methods:** Forty newborns, gestational age between 21 and 34 weeks, weight between 405 and 1,430 grams, hospitalized in a NICU of a public hospital in the city of Maceió, Brazil, were evaluated. Using sterile swabs, samples from the oral and nasal cavities, axilla, inguinal and anal areas of the neonates were collected at the time of admission to the unit, which did not exceed 12 hours postpartum. The samples from the same areas were collected after 48 h, 96 h, and then twice a week until the discharge resulting from clinical improvement or death, making a total of 192 clinical samples. The samples were seeded on Dixon’s medium and microbial colonies were phenotypically characterized using standard biochemical tests. After extraction of DNA, the strains were submitted for 26S rDNA gene PCR amplification. Subsequently, the RFLP analysis using the restriction enzymes *BtsCI* and *HhaI* was performed on PCR products. The standard strain *Malassezia furfur* CBS-1878 was used as a positive control.

**Results:** Eight preterm infants (20% - 8/40), weighing between 585 and 1,344 g, presented the colonization by *Malassezia* sp from 48 h of life. Some newborns showed colonization from the 1^st^ to the 6^th^ week, before they left the NICU. There was a higher frequency of *Malassezia* in the axillae and anal region, followed by the nasal cavity and inguinal area. The difficulty in culture maintenance was observed and some strains failed to survive after the first isolation, making a total of 12 characterized strains. The biochemical phenotypic behavior of the 12 strains was similar and consistent with *M. furfur*. These strains submitted to PCR showed bands between 500 and 600 bp and presented the same pattern of bands in the RFLP analysis indicating *M. furfur*.

**Conclusion:** This study demonstrated the colonization by *M. furfur* in different sites of the cutaneous microbiome of preterm infants in NICU. Since the infections caused by these yeasts are less frequent, these agents are mostly neglected. However, our findings reveal the need for monitoring low and extremely low birth weight neonates, as this yeast may be pathogenic, as described in malasseziosis outbreaks in NICUs. It is worth to note that adequate isolation and cultivation techniques should be employed since most species of the genus *Malassezia* are lipid-dependent and do not grow in the media usually employed for fungal isolation.

### P227. Cryptococcosis in HIV-infected patients in Madagascar: high prevalence and mortality

RakotoariveloR.A.^1^RaberahonaM.^2^RasamoelinaT.^3^RabezanaharyA.^4^RakotomalalaF.A.^3^RazafinambinintsoaT.^2^BénetT.^5^VanhemsP.^6^RandriaM.J.D.D.^2^RomanòL.^7^CogliatiM.^8^CornetM.^9^AndrianariveloM.R.^3^1Service des Maladies Infectieuses, Hôpital Universitaire Tambohobe, Faculté de Médecine, Université de Fianarantsoa, Fianarantsoa, Madagascar2Service des Maladies Infectieuses, Hôpital Universitaire Joseph Raseta Befelatanana, Antananarivo, Madagascar3Centre d’Infectiologie Charles Mérieux, Université Ankatso, Antananarivo, Madagascar4Service de Pneumologie, Hôpital Régional de Reference, Toamasina, Madagascar5Service d’Hygiène, Epidémiologie et Prévention, Hospices Civils de Lyon, Lyon, France6Laboratoire des Pathogènes Emergents, Fondation Mérieux, Centre International de Recherche en Infectiologie, INSERM U1111, CNRS UMR5308, ENS de Lyon, UCBL1, Lyon, France7Laboratorio di Micologia Medica, Dipartimento di Scienze Biomediche per la Salute, Universita degli Studi di Milano, Milano, Italy, Milano, Italy8Laboratorio di Micologia Medica, Dipartimento di Scienze Biomediche per la Salute, Universita degli Studi di Milano, Milano, Italy9Université Grenoble Alpes, CNRS, CHU Grenoble Alpes, Grenoble INP, TIMC-IMAG, Grenoble, France, Grenoble, France

**Objectives:** In Madagascar, the epidemiology of cryptococcosis is poorly documented. The main objective was to estimate its prevalence in HIV-infected patients and to describe the fungal isolates and the patient outcome

**Methods:** An observational study was conducted in the hospitals of Antananarivo (central highlands) and Toamasina (eastern coast). Consecutive HIV-infected adults with CD4≤ 200/µl were enrolled between November 2014 and December 2016. The serum crytococcocal antigen (CrAg) was screened using a lateral flow immunoassay (IMMYCrAg ®). In case of positive result and clinically suspected cryptococcal meningoencephalitis (CM), a lumbar puncture was performed to evaluate the presence of CrAg in the cerebrospinal fluid (CSF), as well as India ink staining and culture. The isolates were identified using MALDI-ToF and genotyped. Antifungal susceptibility was evaluated using the E-test. A high-dose oral fluconazole (at least 1200/day) was given for 10-12 weeks followed by 200 mg/day. Adjusted prevalence ratios (aPR) based on multivariate robust variance Poisson regression, and 90-days survival based on Kaplan-Meier method were used

**Results:** Overall, 129 patients were included, median age was 37 years. At baseline, the median CD4 count was 83 cells/µl, 62.8% had a CD4 count <100 cells/µl and 38.1% were on ART. Overall, 17 patients had a serum CrAg-positive (CrAg+) and 112 CrAg-negative (CrAg-). CrAg+ patients were more likely than CrAg- patients to present low CD4 cells count 38 *Vs* 86; p = 0.018). Among the 17 CrAg+ in serum, 14 had a CSF CrAg+ of which 13 yielded *Cryptococcus* spp. in culture. After multivariate regression, adjusted on CD4 count, previous ART initiation (aPR = 0.4, 95% CI: 0.2-0.9) and presence of neck pain (aPR = 15.7, 95% CI: 3.4-72.1) were associated with CM. The prevalence of cryptococcal infection was 13.2% (95%CI 7.9–20.3) and that of CM was 10.9% (95%CI 6.1-17.5). The prevalence of cryptococcal infection in Toamasina (20%) was not different from the one in Antananarivo (11.1%) (p = 0.21). The mortality rates were 70.6% and 71.4% for cryptococcal infection and CM, respectively. Survival was significantly higher for CrAg- patients as compared with CrAg+ (p = 0.049, Log-rank test). All 17 clinical isolates obtained from CSF (13 at baseline and 4 during follow-up) were identified as *Cn* var. *grubii*. The 13 initial isolates were genotyped and belonged to the VNI type (3 isolates), the rarer but also globally distributed VNII type (4 isolates) and a hybrid intra-varietal type VNI/VNII (6 isolates). All three molecular types were found in Antananarivo whereas only VNI/VNII was found in Toamasina. Among the 13 *Cn* var. *grubii* strains, 2 acquired fluconazole resistance after 4 and 5 months of exposure, respectively.

**Conclusion:** Prevalence of cryptococcal infections and CM in HIV-infected patients in Madagascar were 13.2% and 10.9% respectively. The point-of-care LFA CrAg test confirmed to be reliable and cost effective for the diagnosis. Challenges to facilitate access to more effective molecules to treat patients with CM include reducing administrative formalities linked to drug importation and increasing priorities to implement national control programs.

### P228. The Burden of Serious Fungal Infections in Tajikistan

BobokhojaevO.^1^OsmanovA.^2^DenningD.^2^1Department of Phthisiopulmonology, Tajik State Medical University, Dushanbe, Tajikistan2Global Action Fund for Fungal Infections, Geneva, Switzerland

**Objectives:** The aim of this work is to estimate the burden of serious fungal infections in Tajikistan.

**Methods:** Initially, we identified all published data on serious fungal infections in the country. We have used the model proposed by LIFE (Leading International Fungal Education) to estimate the burden of infections where no local data exist.

**Results:** Tajikistan is the low-income (GDP Per Capita is 800 USD) country in Middle Asia with a population of 8.9 million people. 5% of the population lives on less than 1.9 USD a day and 54% live on less than 5.5 USD a day. There are 2,813,903 adult (15-50 yrs.) women in Tajikistan who comprise 32% of the population. It was estimated that 168,834 Tajik women develop recurrent vulvovaginal candidiasis ( = > 4 episodes/year). The number of HIV positive patients is 15,000; 10,067 (67%) of them do not receive antiretroviral therapy. We estimate 1,253 patients with oesophageal candidiasis and 4,530 patients with oral candidiasis annually, 27 cases of cryptococcal meningitis and PCP is approximated at 216 cases annually. According to our estimations there are 1,040 cases of chronic pulmonary aspergillosis (CPA) as a sequel of TB. Due to the fact, that CPA may occur as a consequence of multiple other pulmonary conditions, the total prevalence of 4,161 cases was estimated. We have estimated 6,008 cased of ABPA and 7,930 cases of SAFS; 196 annual deaths from asthma provides us with an estimation of 137 fungal asthma deaths annually. We have estimated 445 cases of candidemia a year applying a low European rate. There are approximately 205 cases of invasive aspergillosis in immunocompromised patients annually and 1,388 cases in patients with COPD which gives us a total of 1,593 cases of invasive aspergillosis annually. 

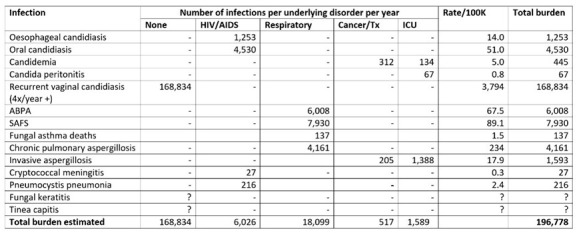



**Conclusion:** According to our estimations, there are 196,778 (2.21% of the population) people suffering from serious fungal infections in Tajikistan. Hence, improving diagnostics is the first step of understanding a scale of the fungal burden and the foundation of addressing the problem of serious fungal infections.

### P229. Cases of Mycetoma reported from a Histopathology laboratory of Pakistan: Two years’ Experience

ZeeshanM.^1^FatimaS.^1^ArifM.O.^2^FarooqiJ.^1^,^3^ArsalanA.^1^JabeenK.^1^,^2^,^4^ZafarA.^3^1Pathology and Laboratory Medicine, Aga Khan University, Karachi, Pakistan2Dow University of Health Science, Karachi, Pakistan3Pathology and Laboratory Medicine, Aga Khan University, Karachi-KHI, Pakistan4Pathology and Laboratory Medicine, Aga Khan University, Karachi, Pakistan, Pakistan

**Objectives:** Mycetoma is a chronic granulomatous infection that is found worldwide and is endemic in tropical and subtropical regions. Occurrence of mycetoma varies in different regions as per environmental temperature and rainfall in that particular area. The exact prevalence of this disease is largely unknown, though estimates are available for various countries. (van de Sande. 2013). Pakistan is a tropical country with diverse climatic conditions. Our green and lush north-eastern border consists of agricultural land is shared with Indian Punjab. South eastern part is close to Indian desert of Rajasthan, and the western border with Iran and Afghanistan is arid. There are multiple reports published from various regions of India as well as Iran. There is dearth of information regarding the frequency of mycetoma, both eumycetoma and actinomycetoma, from Pakistan. The objective of this study was to find out the number of cases reported during year 2017 and 2018 from histopathology department of Aga Khan University Hospital laboratory, a tertiary care hospital laboratory also providing diagnostic services to outside patients

**Methods:** Retrospective data of subcutaneous surgical tissue biopsies with positive findings of mycetoma were retrieved from hospital laboratory database from the years 2017-2018. Site of lesion, age, gender, type of mycetoma and geographical location of patients were analyzed The data was entered and descriptive epidemiologic assessment was carried out using MS excel 2013

**Results:** Total 30 cases of mycetoma reported in these two years. Eumycetoma was reported in the majority of the cases 25/30 (83%) while actinomycetoma in 4/30 (13%). In one case, both entities of mycetoma were detected in a single lesion (3%). Our cohort was all adults with age range of 25-60 years. Eumycetoma cases were mostly observed in male 18/25 (72%) whereas Actinomycetoma cases were mostly seen in females 3/4 (75%). In eumycetoma anatomical site involvement were predominantly lower extremities 22/25 (88%) followed by hand in rest of the other cases. All the reported cases of actinomycetoma had foot involvement only. In majority of cases tissue specimen culture was not performed 23/30 (77%). Out of 7 specimens only 2 were culture positive for fungal etiological agents (29%)

**Conclusion:** In view of our data, we can definitely describe the presence of mycetoma in Pakistan. However, there is a desperate need of national registry to report cases and creating strong liaison between dermatologists, radiologist, surgeons and clinical microbiology laboratory and Histopathologist, so that burden of the disease may be estimated. Only then can we stress upon our national health authorities to prioritize this neglected disease by improving patients’ access to specialized facilities for better management.

### P231. Radiological and microbiological profile of patients with allergic bronchopulmonary aspergillosis presented in outpatient pulmonary clinics of Karachi-Pakistan

IrfanM.^1^IqbalN.^1^,^2^JabeenK.^3^AwanS.^4^ZubairiA.B.S.^4^1Medicine, Aga Khan University, Karachi, Karachi, Pakistan2Medicine, Jinnah Medical and Dental College, Karachi, Pakistan, Pakistan3Pathology and Laboratory Medicine, Aga Khan University, Karachi, Pakistan, Pakistan4Medicine, Aga Khan University, Karachi, Karachi, Pakistan, Pakistan

**Objectives:** There is limited data available on microbiology and radiographic features in patients with allergic bronchopulmonary aspergillosis (ABPA) from Pakistan. The aim of the study is to determine the radiological and microbiological profile of patients visited outpatient pulmonary clinic of a tertiary care hospital of Karachi Pakistan

**Methods:** A retrospective study was conducted at pulmonary outpatient clinics, Aga Khan University Hospital, Karachi, Pakistan from January 2017 to December 2018. Patient with ABPA was enrolled using ISHAM criteria, data was collected on microbiology and radiology features.

**Results:** A total 7759 asthmatic patients presented at outpatient pulmonology clinics, 245 were labelled as ABPA, 167 patients fulfilled the inclusion criteria and 91(54.5%) were female (mean age 41.9 ± 13.0 years). High resolution CT scan (HRCT) chest was done in 126, out of these 104(82.5%) patients had bronchiectasis. Central bronchiectasis was noted in 98(94.2%), Mucus plugging in 71(56.3%) and hyperinflation seen in 30(23.4%) patients. Microbiological testing was available in 103(61.7%). The commonest bacterial pathogen isolated was *Pseudomonas aeruginosa* 32(31.1%) followed by *Hemophilus influenzae* 16(15.5%) and *Morexella catarrahlis* 7(9.7%). While among fungal cultures commonest isolated fungas was *Aspergillus fumigatus* 17(23.6%) followed by *Aspergillus flavus* 16(22.2%) and *Aspergillus Niger* 11(15.3%). Co infection (bacterial and fungi) isolated in 18(17.45%) patients.

**Conclusion:** Extensive bronchiectasis was noted in our cohort of patients with ABPA that leads to frequent bacterial exacerbation. *Pseudomonas aeruginosa* was found to be common among bacterial pathogens. Early diagnosis can prevent lung damage.

### P232. Epidemiology and outcomes of patients with invasive mould infections: a retrospective observational study at Karolinska University Hospital, 2010 - 2014

Böhlin-WienerA.^1^,^2^SzakosA.^3^KlingsporL.^2^1Clinical Microbiology, Karolinska University Hospital, Stockholm, Sweden2Laboratory Medicine, Karolinska Insitutet, Stockholm, Sweden3Pathology and Cytology, Karolinska University Hospital, Stockholm, Sweden

**Objectives:** To investigate the epidemiology and outcomes of invasive mould infections (IMI) during 2010-2014, compared to 2005-2009 (1).

**Methods:** Patients with proven or probable IMI were included. Data regarding patient baseline characteristics, underlying diseases, types of transplant, infecting mould species, sites of infection, antifungal therapies, etc were extracted from patient journals. Outcome was 90-days and 1-year survival post diagnosis.

**Results:** A total of 127 (vs 100) patients were included, an increase by 27% compared to the previous period. The rise in incidence occurred mainly during 2010-2011 (Fig 1). The majority, 54% (vs 66%) were in the age group 18-65 and 30% (vs 24%) were over 65 years old. Males predominated with 57% (vs 60%). Most isolates were *Aspergillus* spp, *n* = 59 (vs 75) followed by *Mucorales* spp, *n* = 14 (vs 9) and *Fusarium* spp, *n* = 8 (vs 3). In *n* = 7 cases (vs 10), more than one mould was isolated. An additional *n* = 45 had a positive galactomannan antigen test. Lungs were the most often affected organ, 92% (vs 88%) and haematological malignancies (HM) were the most common underlying diseases, 71% (vs 70%). In total, 431 (vs 354) allogeneic Haematopoietic Stem Cell Transplants (HSCT) were performed during the study period, of which 6.5% (vs 7.6%) developed IMIs. Following diagnosis, 75% (vs 87%) received antifungal therapy, usually voriconazole, 53% and liposomal amphotericin B, 38%. Breakthrough infections occurred in 33% of the cases in spite of prophylaxis, mainly posaconazole, 59% and caspofungin, 24%. The 90-days overall survival remained basically unchanged, 48% (vs 49%), but with significant yearly variations (Fig 2). The allogeneic HSCT and HM cohorts had highest overall survival rates, 68% respectively 55% (vs 37% respectively 43%). The 1-year overall survival declined to 35% (vs 46%) with lowest rate 13% in 2012.

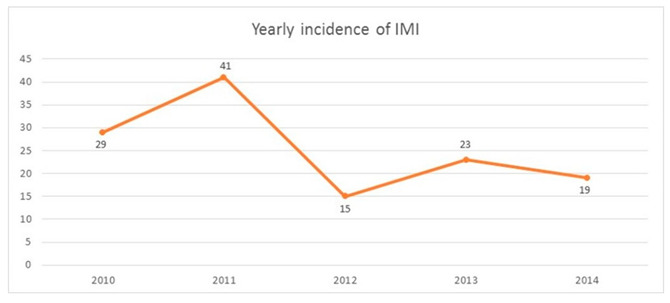



**Conclusion:** There were no significant changes in patient characteristics, mould epidemiology or underlying diseases. Survival rates improved for the HM and allogeneic HSCT cohorts. The yearly variations in mortality rates coincide with construction work around the hospital, notably with periods of intense blasting. During these periods there was also an increase in the incidence of IMIs.

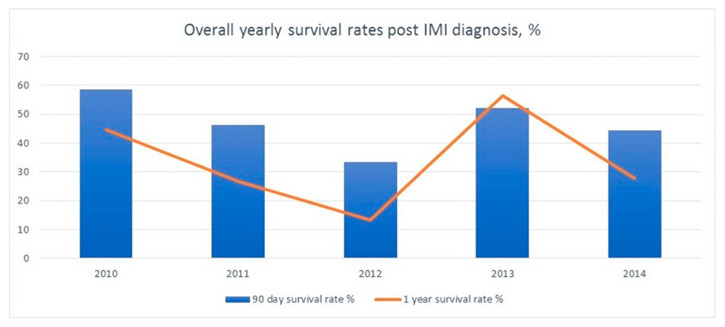



(1). Klingspor L, et al: Epidemiology and outcomes of patients with invasive mould infections: a retrospective observational study from a single center (2005-2009). Mycoses. 2015 Aug; 58(8):470-7

### P233. Epidemiology of candidemia and their antifungal susceptibility in our University Hospital from 2014 to 2018

VanniniM.^1^LieutierF.^2^EmeryS.^2^ReturN.^2^HasseineL.^1^1Laboratoire De Parasitologie Mycologie, CHU DE NICE, NICE, France2Service Pharmacie, CHU DE NICE, NICE, France

**Objectives:** Given the differences between local epidemiologies, we have to know our fungal epidemiology, in order to adapt our antifungal strategies. The purpose of this study is: **1**. to identify the epidemiology and antifungal susceptibilities of candidemia over 5 years; **2**. to evaluate the anti-fungal treatment and to see the survival in the third month (M3) of the patients after the diagnosis of fungemia.

**Methods:** This study was retrospective, conducted from January 2014 to December 2018. All patients who had at least one positive fungal blood culture were included. A single blood culture per patient was taken into account with a study of the MICs (Molecules tested: amphotericin B, caspofungin, voriconazole and fluconazole).

**Results:** In total, 306 *Candida* blood culture isolates were obtained from 280 patients (39 in 2014, 62 in 2015, 72 in 2016, 60 in 2017 and 47 in 2018). The three most common *Candida* spp isolated from blood cultures were *Candida albicans* (44%), *Candida glabrata* (22%) and *Candida; parapsilosis* (13%). Any resistance to the four tested antifungals is observed for *C. albicans* and *C.parapsilosis.* However, resistance was observed for *C. glabrata* in 10 % for amphotericin B, 28% for voriconazole, and 15% for fluconazole. In 2 % and 75%, there is respectively a resistance and an intermediate susceptibility to caspofungin. For the time being, the antifungal treatment was recovered for 170 patients. Among them, 67%, 26% and 5% received respectively caspofungin, fluconazole and ambisome. 2% have not received antifungal treatment, because they were at the end of their life. Evolution at M3 was reported and the mortality rate was 32%. Candidemia has probably a role in 51% of them.

**Conclusion:** We report an overall increase in candidemia but a constant distribution of *C albicans*, *C paraspsilosis* and *C glabrata*. The lack of antifungal resistance for *C. albicans* and *C. parapsilosis* for candidemia suggests a possibility of treatment with fluconazole as a first-line in our hospital, without waiting for the results of the antifungal susceptibilities. We have also to analyse the possible nosocomial etiology, and the link between antifungal resistance, and the cause of death of those patients.

### P234. Presence of Malassezia pachydermatis in the cutaneous microbiome of humans cohabiting with dogs

AlvarezJ.^1^CamargoL.^2^CorsiniC.^2^NevesJ.^1^CoutinhoS.D.A.^1^1Post-graduation Program In Environmental and Experimental Pathology, Paulista University, São Paulo, Brazil2Veterinary Hospital, Paulista University, São José dos Campos, Brazil

**Objectives:** *Malassezia* species are yeasts that are inhabitants of the cutaneous microbiome of animals and men; however, they can eventually cause infections. In dogs, *M. pachydermatis*, a zoophilic and non-lipid-dependent species, particularly causes otitis and dermatitis. Although malasseziosis is not considered zoonotic, it was suggested the disease may be transmitted to neonates by the professionals of intensive care units (ICU) who have dogs. No data on the exchange of *Malassezia* species between animals and men who have close contact are available. The aim of this study was to isolate and identify *M. pachydermatis* from the cutaneous microbiome of dog owners and compare with that of people who do not have contact with animals.

**Methods:** A total of 15 dogs with otitis and/or dermatitis caused by *M. pachydermatis* and their owners were selected. We collected three clinical samples of each owner (total 45 samples) from the upper trunk, scalp, and behind the ears. At the same time, three clinical samples were collected from 24 people (total 72 samples) of the same regions, who did not have any contact with animals. The samples were seeded on Dixon’s medium and the isolates obtained were inoculated on Sabouraud dextrose agar. Cultures that grew only on the Dixon’s medium were considered lipid-dependent *Malassezia* species and those on Sabouraud dextrose agar were considered as *M. pachydermatis*. For genus confirmation, DNA was extracted and 26S rDNA was amplified using the polymerase chain reaction. The standard strain *M. pachydermatis* CBS-1879 was used as a positive control.

**Results:** The presence of *Malassezia* spp. in the people living with dogs (80.0% - 12/15) and those who not living with animals (83.3% - 20/24) was similar (Table 1). *Malassezia* spp. were isolated from 37.8% (17/45) and 58.3% (42/72) of clinical samples from those cohabiting with dogs and those who were not, respectively (Table 1). *Malassezia pachydermatis* was the most common species in the cutaneous microbiome of the owners found in 52.9% (9/17) of the isolates, whereas lipid-dependent *Malassezia* spp. were isolated from 47.1% (8/17) (Table 1). *Malassezia pachydermatis* was isolated from the three sites sampled, scalp, upper trunk, and behind the ears. Only lipid-dependent *Malassezia* (100.0% of the isolates) was found in people who did not cohabit with dogs, *M. pachydermatis* was not isolated from these people (Table 1). "Dog cohabitation" has a statistically significant effect on the presence of *M. pachydermatis* in the cutaneous microbiome of the owners (*p* < 0.05). 

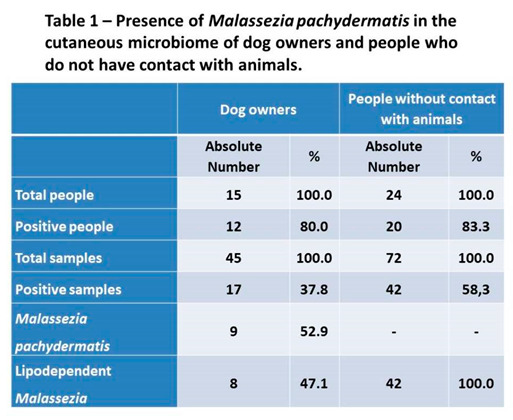



**Conclusion:** *Malassezia pachydermatis* was associated with the nosocomial outbreaks of fungemia in neonates hospitalized in ICUs, with the possibility of transmission occurring by health professionals who were having dogs and carried the yeast to the ICU. However, limited literature exists on the association of close cohabitation with dogs or other pets with the human microbiome. Our results show that there is a change in the *Malassezia* spp. in the cutaneous microbiome of people cohabiting with dogs, thus significantly increasing the presence of a zoophilic strain of *M. pachydermatis*.

### P235. “zygomyco.net”: 15 years of a global mucormycosis registry of the joined ECMM/ISHAM Working Group on zygomycosis.

SkiadaA.^1^Drogari-ApiranthitouM.^2^PetrikkosG.^3^11st Dpt. of Medicine, Laiko Hospital, Athens University, Athens, Greece2Infectious Diseases Research Laboratory, 4th Department of Internal Medicine, General University Hospital “Attikon”, School of Medicine, University of Athens, Athens, Greece3Infectious Diseases, School of Medicine, European University of Cyprus, Nikosia, Cyprus

**Objectives:** Taking into consideration the challenges related to epidemiology, treatment and outcome of mucormycosis, a Working Group (WG) on Zygomycosis was initially formed in ECMM, in 2004. It aimed to create a registry by collecting mucormycosis cases from European countries. From 2008 onwards the WG expanded under also the auspices of the ISHAM and it now includes participants from all over the world. The aims of this project are: 1) to conduct a prospective international registration of patients with mucormycosis using a well-established global network, 2) to collect and molecularly identify mucormycetes and tissue specimens from the patients enrolled in order to establish an international archive that will be used in future studies and 3) to provide information about this rare but devastating disease and enhance the collaboration of physicians and researchers all over the world.

**Methods:** Definitive and probable mucormycosis cases from each participating country were initially prospectively recorded in standardized case report forms (CRFs) and sent to the study coordinator. Since the creation of the registry platform (www.zygomyco.net) participants can submit cases directly. Isolated mucormycetes are identified at the participating institutions and molecular identification, if not available, is performed at an appointed laboratory (National Centre for Mycology, Madrid, Spain). In absence of culture data, paraffin-embedded tissues are sent to an appointed laboratory for PCR. Appropriate statistical methods are used for univariate and multivariate analysis.

**Results:** The WG on zygomycosis has organized 3 international fora on zygomycosis, co-organized international workshops, and meetings are regularly held. As an ongoing study, results are being presented at conferences and related papers are published. During 2005-2007, 230 cases were submitted and analyzed. The first analysis (A. Skiada *et al*., Clin Microbiol Infect 2011;17:1859–1867) has shown a wide variation regarding underlying diseases, clinical presentations, fungal species and treatment modalities in different countries. These observations have increased our understanding of the risk for transmission and diversity of this disease, which may lead to increased suspicion, earlier diagnosis and appropriate management. The analysis has also contributed to issuing guidelines on the diagnosis and treatment of mucormycosis. More than 300 further cases have been added during 2008-2018, from more than 15 countries, Brazil, India, Iran, Lebanon and the USA included.

**Conclusion:** The existence of the ECMM/ISHAM Working Group on Zygomycosis and its official website and registration platform, provide the means for a network of investigators, including many experts, to interact, exchange information and hopefully improve the outcome of mucormycosis. Focus on more detailed information on the development of the disease according to various underlying conditions and mucoralean species involved is a future perspective, as a tool for the construction of predictive risk models for patients at risk. This project can also be a powerful tool for the assessment of novel diagnostic procedures, therapeutic modalities and efficacy of new antifungals.

### P236. First report on the isolation of five new geophilic Arthroderma species in hedgehogs

UhrlassS.^1^RimekD.^2^HubkaV.^3^SchroedlW.^4^ReuschelM.^5^MuetzeH.^1^KochD.^1^KruegerC.^1^GraeserY.^6^NenoffP.^1^1Partnership Dr. C. Krueger & Prof. P. Nenoff, Laboratory of medical microbiology, Roetha OT Moelbis, Germany2Gesundheitsschutz, Thueringer Landesamt für Verbraucherschutz, Bad Langensalza, Germany3Laboratory of Fungal Genetics and Metabolism, Institute of Microbiology ASCR, Prague, Czech Republic4Ag Mykologie, Institut für Bakteriologie und Mykologie, Leipzig, Germany5Klinik Für Heimtiere, Reptilien, Zier- Und Wildvögel, Stiftung Tierärztliche Hochschule, Hannover, Germany6Institut Für Mikrobiologie Und Hygiene, Universitätsmedizin – Charité, Berlin, Germany

**Objectives:** During the last years an increase of rare and new dermatophytes in human beings, but also in animals has been observed in routine diagnostics of mycological laboratories, not at least due to the significantly improved molecular identification methods. The conventional diagnostics includes native preparation plus fungal culture. Today, molecular methods, e. g. polymerase chain reaction (PCR), in particular an in-house- PCR-ELISA, are completing mycological diagnostics.

**Methods:** Recently, between April and September 2018, a survey of the health of hedgehogs was performed in the city of Hanover, Germany. Altogether 50 European hedgehogs were included in this study. Skin and sting samples from the animals were collected, and mycological investigation was done as described before. A minor part of hedgehogs showed clinical signs of a dermatomycosis, most animals were, however, asymptomatic. For confirmation of cultural dermatophyte isolates, sequencing of the Internal Transcribed Spacer (ITS) region of the rDNA, and the Translation Elongation Factor (TEF)-1α gene was performed.

**Results:** The following dermatophytes were isolated from the hedgehogs: *Trichophyton erinacei* (8), *Arthroderma* (*A*.) *crocatum* (4), *A*. *quadrifidum* (2), *A*. *insingulare* (1), *A*. *chiloniense* (1) and *A*. *tuberculatum* (1). Based on their macro architecture, their microscopic features, and their genetic characteristics, all from hedgehogs isolated strains were compared with each one or two clinical isolates of the respective *Arthroderma* species. These reference strains were isolated from human skin samples, e.g. scrapings, nail samples, and hair roots, achieved between 2016 to 2018. Clinical diagnoses were tinea manuum, tinea corporis, tinea capitis, and tinea unguium.

**Conclusion:** Meanwhile, both the here described new animal isolates from hedgehogs, but also the human strains of different *Arthroderma* species are deposited at the deposited at the „Deutsche Sammlung von Mikroorganismen und Zellkulturen” (DSMZ, German Collection of Microorganisms and Cell cultures), Brunswick, Germany. The DNA sequences (ITS and TEF-1α) of all strains are available now at the gene bank of the National Center for Biotechnology Information (NCBI) in Bethesda, Maryland, USA.

### P237. Epidemiology, Clinical characteristics, and Outcome of adult Candidemia in a Chinese tertiary hospital: from 2008 to 2015

ZhangL.^1^LiuZ.^1^LiT.^1^XiaoM.^2^WangY.^2^XuY.^2^1Department of Infectious Disease, Peking Union Medical College Hospital, Beijing, China2Department of Clinical Laboratory, Peking Union Medical College Hospital, Beijing, China

**Objectives:** The aim of this study was to assess the epidemiology, clinical characteristics, risk factors and outcome of candidemia patients in a tertiary hospital.

**Methods:** A retrospective observational study of all cases of candidemia which occurred during hospitalization was carried out in a tertiary hospital including 6 intensive care units (ICUs), 22 medical and 24 surgical wards in Beijing, China(2008-2015). Demographic and clinical data were collected from the database of the patient’s medical records. Chi Square test (or Fisher’s Exact Test when expected frequencies were less than 5) and multivariate logistic regression models were used to identify the risk factors associated with the higher mortality during hospitalization.

**Results:** A total of 192 episodes of candidemia were identified in 181 patients(male 102,female 79, mean age 58.96±17.86). All cause mortality during hospitalization was 64/181 (35.4%), and attributable mortality was 50/181 (27.6%). Multivariate analysis showed that age≥65 years, ICU hospitalization and polymicrobial bloodstream infections were independently associated with a higher risk of crude mortality. *Candida albicans* was the predominant specie 100/192(52.1%) followed by *C.glabrata 31/192*(16.1%), *C.parapsilosis 28/192*(14.6%) and *C.tropicalis 26/192*(13.5%). Species distribution differed significantly according to different wards and sources. *Candida albicans* was predominant in obstetrics gynecological ward 9/12(75%), emergency ward 16/23(69.5%) and ICU 38/70(54.3%). Non-*albcan candida spp. were* predominant in surgical wards 23/37(62.2%) and internal medical wards 27/50(54%). Intra-abdominal infection34/192 (17.7%) was the most frequent infective source of candidemia, followed by pulmonary infection 34/192(16.1%) and CVC-related candidemia 27/192(14.1%), however, the source of infection was not identified in 71/192(40.0%) episodes. Meanwhile, we also found higher percentages of *C.albicans* in candidemia secondary to abdominal infection 22/34(64.7%) and multifocal infections 7/11(63.6%), but higher percentages of non-*albicans* in skin and soft tissue infection 4/6(66.7%) and CVC-related candidemia 17/27(63.0%) (Figure 2).Fluconazole resistance accounted for 1.0%, 3.6%,3.8% and 6.5% of isolates of *C.albicans* (1/100), *C.parapsilosis* (1/28), *C.tropcalis* (1/26) and *C.glabrata* (2/31), respectively.

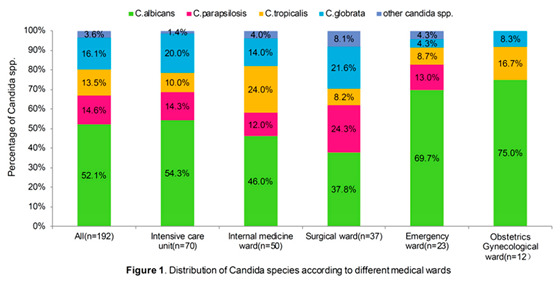

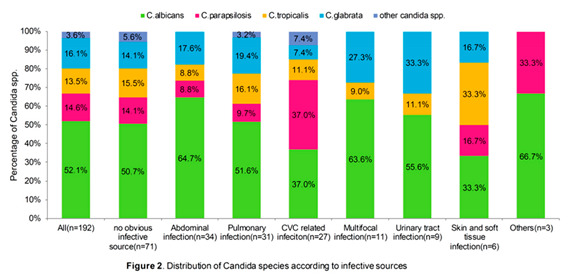





Previous antibiotic exposure (<30 days)154/181(85.1%), reservation of central venous catheter 150/181(82.3%), previous hospitalization history 140/181(77.3%) and retention of Foley catheter 122/181(67.4%) were the most frequent risk factors.

**Conclusion:** Our study showed that candiemia was associated with poor outcome and higher mortality. Identification of risk factors associated with candidemia diagnostic and prognositic may lead to better management in terms of preventive and therapeutic measures. Given the difference of candida species distribution in different wards and different infecive sources, improving the awareness of epidemiology are in urgent need in candidemia treament.

### P238. Epidemiology and Outcome of Culture-Proven Coccidioides immitis Infections in San Diego, California, United States

JenksJ.ReedS.HoeniglM.Medicine, University of California San Diego, San Diego, United States of America

**Objectives:** *Coccidioides* are dimorphic fungi endemic to the Southwestern United States *(Coccidioides immitis*) and parts of Central and South America (*Coccidioides posadasii*). Infection from these fungi, coccidioidomycosis, can cause a range of disease from self-limited acute pneumonia to severe, disseminated disease. The objective of this study was to describe clinical manifestations, treatment and outcome of patients with culture-proven *Coccidioides immitis* infections in San Diego, California, United States, an area endemic to *Coccidioides immitis.*

**Methods:** We performed a retrospective chart review of medical records of all patients with culture-proven *Coccidioides immitis* infection at UCSD between 5/01/2017 - 12/31/2018 (1 1/2 year period). The study was approved by the UCSD Human Research Protection Program.

**Results:** A total of 11 cases with detection of *Coccidioides immitis* in culture were identified (Table). of 11 cases, 10 were evaluable, and of those 1 had chronic coccidioidomycosis and 9 had proven acute infection. of those with acute infection, 56% (5/9) had traditional risk factors for coccidioidomycosis infection including African American race, immunosuppressive state, lung transplant status, HIV/AIDS, and type II diabetes mellitus, while the remaining 4/9 lacked traditional risk factors. Five of 9 (56%) had primary coccidiodomycosis of the lungs and 4/9 (44%) disseminated disease. of those who received antifungal therapy, 3/7 (43%) received combination therapy and 4/7 (57%) monotherapy with fluconazole. At 180 days, 5/9 (56%) were alive, 3/9 (33%) deceased, and 1/9 (11%) lost to follow-up. of those who were not alive at 180 days, 1/3 (33%) had disseminated disease and 2/3 (67%) primary coccidioidomycosis, both who were on immunosuppressive therapy.

**Conclusion:** Culture-proved *Coccidioides immitis* infection occurred in a variety of hosts, with 44% lacking typical risk factors for infection. Disseminated coccidioidomycosis infection occurred in 44%; of these, 50% lacked traditional risk factors and 75% were alive at 180 days.

### P239. Positive fungal culture in tissue specimens from burns patients: Data from a tertiary care hospital laboratory in Pakistan

JabeenK.^1^UmarS.^2^ShaheenN.^2^KhanM.^3^FarooqiJ.^2^1Pathology and Laboratory Medicine, Aga Khan University, Karachi, Pakistan, Pakistan2Pathology and Laboratory Medicine, Aga Khan University, Karachi, Pakistan3Medical College, Aga Khan University, Karachi, Pakistan

**Objectives:** Due to defective skin barrier burns patients are at great risk of development of fungal wound infections. Fungi are frequently cultured from tissue specimens from these patients either singly or in combination with bacteria. Spectrum of fungal wound infection in burns patients from Pakistan has not been reported previously. We evaluated positive cultures from burn wounds samples received at a tertiary care hospital laboratory in Karachi, Pakistan. Positivity rate of fungal isolation within these cases and spectrum of yeast and filamentous mold was reported.

**Methods:** We reviewed laboratory data of tissue and wound samples received for bacteriological assessment from burn patients at the Aga Khan University Hospital laboratory, Karachi, Pakistan from September 2017-March 2018. Specimens were inoculated on chocolate agar and sheep blood agar with colistin and nalidixic acid incubated in 5% CO2, a triplate with MacConkey agar, Mannitol salt agar and Sabouraud’s dextrose agar incubated in ambient environment, and sheep blood agar incubated in anaerobic environment. All cases were incubated at 37 °C and pus and tissue samples were also enriched by inoculating in cooked meat broth. Clinical and demographic information recorded on the worksheet at the time of clinical reporting was collected for the study. Frequencies were computed to describe the data.

**Results:** A total of seventy positive tissue cultures from burn patients were identified over the study period. These specimens were received for 36 male patients and 34 female patients with age ranging from eight months to eighty five years (average: 15.5 year). of these, 26 (37%) had growth of either filamentous mold (17 cases) or *Candida species* (11 cases). Two cases had growth of both mold and yeast. *Aspergillus flavus* was the most common mold (9 cases) followed by *Fusarium* species (3 cases) and *Lichtheimia corymbifera* (2 cases). *Candida tropicalis* was the most common yeast (5 cases) followed by *Candida parapsilosis* (3 cases). There was a concomitant bacterial growth in 19 cases predominantly of *Staphylococcus aureus* (13 cases), *Pseudomonas aeruginosa* (8 cases) and other Gram negative rods (6 cases).

**Conclusion:** High rate of fungal isolation from burn wound infection was seen in this study despite less optimal culture techniques. The findings in our study are consistent with the global increase in incidence of fungal infections in burn wounds. High index of suspicion of fungal infections by clinicians and revision of culture protocols in burn patients may be warranted for optimal management of these patients.

### P240. rFungal isolation from wound samples submitted for culture at a tertiary care hospital laboratory in Karachi, Pakistan

FarooqiJ.^1^LadakA.^2^ShaheenN.^3^JabeenK.^4^1Pathology and Laboratory Medicine, Aga Khan University, Karachi-KHI, Pakistan2Medical College, Aga Khan University, Karachi, Pakistan3Pathology and Laboratory Medicine, Aga Khan University, Karachi, Pakistan4Pathology and Laboratory Medicine, Aga Khan University, Karachi, Pakistan, Pakistan

**Objectives:** Skin and Soft tissue infections rank the fourth most common infections worldwide. These often emerge from chronic wounds, traumatic implantation and surgical wounds. Bacteria are the most common isolated organisms from such wounds. However, recently, many centers across the world have started to report an increase in fungal isolates, possibly due to excessive use of antibiotics and expansion in host risk factors. This study aims to present the frequency of reported fungal isolates in patients with skin and soft tissue infections and relate the incidence of these infections with concomitant risk factors.

**Methods:** We reviewed laboratory slips of all tissue and pus samples received for bacteriological assessment at the Aga Khan University, Hospital clinical laboratory from mid-September till to mid-October 2018. All samples were inoculated on Chocolate Agar and Sheep Blood Agar with colistin and nalidixic acid incubated in 5% CO_2_, a triplate with MacConkey Agar, Mannitol Salt Agar and Sabouraud’s Dextrose Agar incubated in ambient environment, and Sheep Blood Agar incubated in anaerobic environment. All cases were incubated at 37 °C and also enriched by inoculating in Cooked Meat Broth. Clinical information recorded on the worksheet at the time of clinical reporting was collected for the study. Any missing information was filled in from the in-house hospital information management software if available. Variables such as patient age, location, gender, relevant medical history, type and site of wound, microbial isolates and drug resistance were collected. Frequencies were computed to describe the data.

**Results:** We reviewed a total of 140 patients’ information over the study period. Our study population consisted of 48.6% (*n* = 68) females and 51.4% (*n* = 72) males with the median age of 30.5 years. of the 140 samples submitted, 76.4% (*n* = 107) were culture positive; pathogens were isolated from 81.3% of these (*n* = 87), and 9.3% yielded fungi. The categories of lesions included accidental wounds [8.6% (*n* = 12)], burn and blast injuries [2.9% (*n* = 4)], diabetic wounds [7.9% (*n* = 11)], nosocomial infections [15% (*n* = 21)], skin and soft tissue infections [57% (*n* = 80)] and unknown [8.6% (*n* = 12)]. The fungal isolates were spread across all categories of lesions. Skin and soft tissue infections had the highest number of fungal isolates (*n* = 4) while diabetic wounds had none. Yeasts (*Candida* species) were most commonly isolated (*n* = 4), followed by *Fusarium* species (*n* = 3), *Aspergillus* species (*n* = 2), *Bipolaris* species (*n* = 2) and *Curvularia* species (*n* = 1).

**Conclusion:** Our center has reported a high number of fungal isolates over the short study period. Our study raises the question of identifying underlying causes that has led to this high number of fungal isolates reported in just one month from samples sent only for bacterial culture. Moreover, centers across the region should be educated about the rising fungal load in the population especially wound samples. Setting an additional fungal isolation medium may be life saving for patients with severe necrotizing fungal wound infections.

### P241. Subcutaneous Mucormycosis – Beyond Rhizopus and Mucor species

SebastianC.^1^VR.^1^MathewJ.^2^SavioJ.^2^1Microbiology, St.John’s Medical College, Bangalore, India2Microbiology, St. John’s Medical College, Bangalore, India

**Case Report:** Introduction The *Mucorales, Apohysomyces* and *Saksenaea* species are unusual human pathogens that are reported with increasing frequency as a cause of subcutaneous infection in previously healthy patients following trauma or invasive procedures.Identification of these species in a routine laboratory is challenging due to their difficulty in sporulation. Special techniques for inducing sporulation must be employed for their identification. Objective To report 4 cases of subcutaneous mucormycosis due to the *mucorales* - *Apohysomyces* and *Saksenaea* species **Case 1** 25 years old farmer presented with necrotising fasciitis of the entire back following religious self-flagellation. Microscopic examination of the tissue revealed aseptate hyphae. Culture yielded a mucorale that was identified as *Apophysomyces variabilis.* Patient condition improved with wound debridement and liposomal Amphotericin B. He was discharged on Posaconazole. **Case 2** A 30 year old male patient, diabetic since 6 months presented with submandibular swelling of 10 days duration following an injury to the neck. Pus sample revealed aseptate hyphae. Culture isolate was identified as *Apophysomyces* species on culture. **Case 3** 55 years old patient presented with persistent fever and necrotising fasciitis of the right leg. It all started following a bite by a pig 15 days back. He was not a known case of diabetes mellitus but was diagnosed on admission. The tissue sample yielded *Apophysomyces elegans* on culture. **Case 4** 25 years old retro positive patient presented with a gluteal abscess following an intramuscular injection. The fungal culture of the tissue sample yielded *Saksenaea vasiformis.* **Conclusion** Mucormycosis is an angio-invasive infection which is rapidly progressive and associated with a high morbidity and mortality. *Rhizopus* species is the most common mucoralean worldwide causing mainly rhino-orbito-cerebral mucormycosis. Genera other than *Rhizopus* like *Apophysomyces* and *Saksenaeae* are associated with cutaneous and subcutaneous manifestations. More and more cases are presently being diagnosed in developing countries like India where the at-risk population of diabetic patients and other immunocompromised states are continually on the rise. 39 cases of mucormycosis were reported from our laboratory in the last 2 and a half years, from January 2017 to May 2019. 34 were rhino-orbito-cerebral mucormycosis caused by *Rhizopus* species. of the 5 cases of subcutaneous mucormycosis, 3 were caused by *Apophysomyces* species and one each by *Saksenaea vasiformis and Rhizopus arrhizus* respectively. All three Apophysomyces species were identified as *Apophysomyces elegans* in our laboratory. However sequencing at PGIMER Chandigarh has confirmed one of them as *A.variabilis.* Two of the four cases were diabetic patients and one was retro positive. All the four patients gave a history of trauma. In a country like India, mucormycosis should be considered in patients presenting with non healing wound especially following a trauma, even in young adults as they may be silent diabetics.

### P242. Changing trends in genus and species distributions of clinical fungal strains: An evaluation of ten-year data from a tertiary care center in Turkey

GulmezD.SığA.K.AkarN.DuyanS.Arikan-AkdagliS.Medical Microbiology, Mycology Laboratory, Hacettepe University Medical School, ANKARA, Turkey

**Objectives:** Opportunistic fungal infections may develop due to a wide variety of fungal species. Changes in species distribution profiles are reported from many centers and occasionally reflect an increase in rates of antifungal drug-resistant or -less susceptible species. The aim of this retrospective evaluation is to determine the fungal species and their relative isolation rates in the last ten years in our center with a specific focus on bloodstream yeast isolates and mould growth from lower respiratory tract specimens.

**Methods:** Fungal strains isolated from clinical samples from 2009 to 2018 were included. Isolation and identification were done by standard mycological methods. Fungal growth in all sample types was evaluated and repetetive growths from the same type of specimens were excluded. Isolated fungal species were analysed in course of time and with respect to the specimen types. Distribution of species in five-year periods (2009-2013 vs. 2014-2018) was compared by z test.

**Results:** A total of 23046 specimens sent to the laboratory during the study period revealed fungal growth. Excluding the episodes of repetetive growth, 17239 fungal strains were isolated from 15465 clinical samples. Total number of specimens and isolates markedly increased in time, from 757 and 819 in 2009 to 2511 and 2875 in 2018, respectively. Urinary tract (*n* = 7164, 46%) and lower respiratory tract (*n* = 4523, 29%) were the most common fungal isolation sites followed by blood (specimens from peripheral vein and catheter, *n* = 1076, 7%). For all specimen types, most common species were *Candida albicans* (58%) and *Candida glabrata* species complex (SC) (12%). Among moulds, *Aspergillus fumigatus* SC (51%) was the most common. Per the yeast species isolated from blood cultures (Figure 1), isolation rate of *C.albicans* decreased from 48% to 44% (p = 0.186) and that of *C.glabrata* SC increased from 10% to 15% (p = 0.014) in 2014-2018 as compared to 2009-2013. No significant change was observed in species distribution of isolates from sterile specimens other than blood. Distribution of mould genera from lower respiratory tract specimens is shown in Figure 2. Although *A.fumigatus* SC was always the most common, its rate of isolation decreased from 63% in 2009-2013 to 46% in 2014-2018 (p<0.001). In the same time intervals, isolation of *Aspergillus* species other than *A.fumigatus* SC increased from 26% to 34% (p = 0.145) and moulds other than *Aspergillus* (*Penicillium* spp., Order Mucorales, *Scedosporium* spp., *Alternaria* spp., *Paecilomyces* spp., dematiaceous fungi and unidentified moulds) increased from 11% to 20% (p = 0.185).

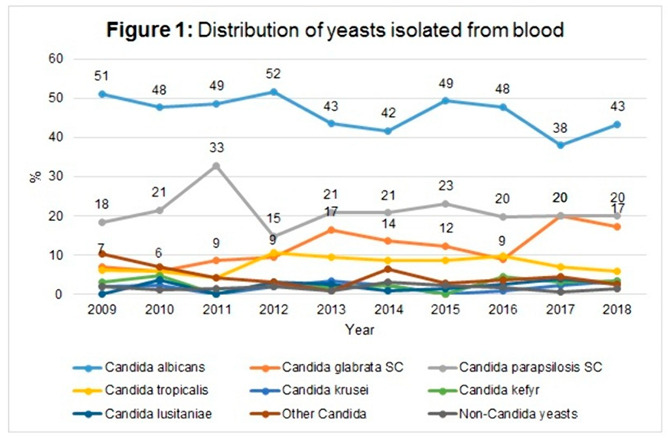

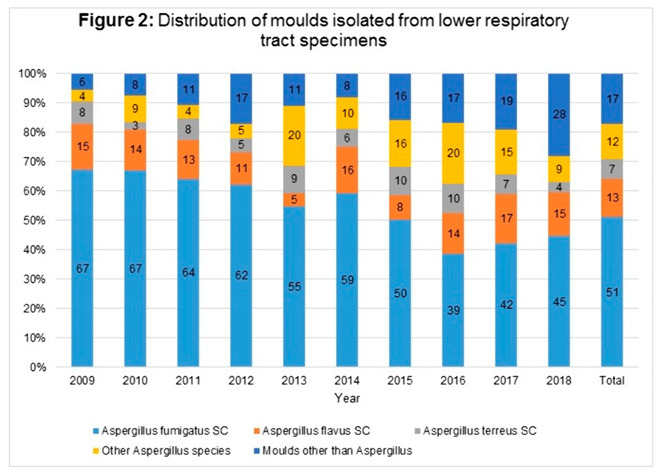





**Conclusion:** In our center, while the most commonly isolated yeast and mould species display a usual profile, species distribution is diverse. Evaluation of bloodstream yeast isolates revealed a decrease of *C.albicans* and an increase in *C.glabrata* SC, which appears as a recent change. Significance of increase in rate of *C.glabrata* SC remains noteworthy also with respect to potential resistance or decreased susceptibility to antifungal agents. Isolation of less common mould genera from lower respiratory tract samples remains remarkable.

### P243. A mucormycosis case during the catastrophic flood in Mandra, Attica, Greece, November 2017

SympardiS.^1^Drogari-ApiranthitouM.^2^ZervakakisI.^3^KalofonouM.^3^MinogiannisN.^3^PapakonstantinouH.^4^LiapiG.^4^ArvanitiA.^1^KaraiskouA.^1^GiannopoulouP.^5^Trikka-GrafakosE.^5^1Infection Control Team, Thriasio General Hospital of Elefsis, Elefsis, Athens, Greece2Infectious Diseases Research Laboratory, 4th Department of Internal Medicine, General University Hospital “Attikon”, School of Medicine, University of Athens, Athens, Greece3Department of Plastic Surgery, Thriasio General Hospital of Elefsis, Elefsis, Athens, Greece4Department of Pathology, Thriasio General Hospital of Elefsis, Elefsis, Athens, Greece5Laboratory of Microbiology, Thriasio General Hospital of Elefsis, Elefsis, Athens, Greece

**Case Report:**

**Objectives:** Cutaneous mucormycosis after major trauma may cause devastating morbidity and death if not treated promptly and appropriately. The presentation of this case report aims to sensitize health care professionals for the potential occurrence of cutaneous mucormycosis after natural disasters. 

**Methods:** Case report: A previous healthy 75-year-old woman was trapped in her flooded home during the flood in Mandra, Attica, Greece, which occurred on 15/11/2017. She remained four hours under water until rescued. She was transferred to the Emergency department (ED) of a General Hospital with dyspnoea and type-I acute respiratory failure (ARF) due to near-drowning. The patient had also multiple blunt injuries with skin rupture in both lower extremities. At the ED she received treatment for ARF consisting of oxygenation, tracheal aspiration, bronchodilators and antibiotics. Her wounds were surgically cleaned and sutured and tetanus immune globulin was administered. The Glasgow Coma Scale score (GCS) was evaluated at 13/15 and the patient was admitted to the Pneumology department for further treatment. At day 2 post-admission clinical improvement was documented, but on day 3 her consciousness level deteriorated (qSOFA score >2) with onset of fever and low blood pressure. The antibiotic scheme was changed. Upon re-evaluation of the leg wounds, development of necrotic eschars at the wound margins was observed. On day 4 the patient was put on mechanical ventilation (GCS<8/15), underwent extensive tissue debridement and liposomal amphotericin B 10mg /kg /24h was added to the antibiotic scheme on suspicion of mucormycosis. Excised tissue was sent to the Pathology and Clinical Microbiology departments for histological examination and culture. 

**Results:** The patient was transferred to a high dependency unit (HDU), however, on day 5 she suffered severe cardiovascular collapse while the infection on both legs had progressed over a large area. The patient’s family denied consent for bilateral hip disarticulation. The patient died on day 6 after hospital admission. Histology revealed fungal hyphae typical for Mucorales. 

**Conclusions:** Mucormycosis should be always suspected in victims of natural disasters. Infection can progress rapidly, requiring early therapy with antifungals, frequent inspection of the wounds and repeated surgical debridement. Early primary wound closure should be avoided.

### P244. The burden of serious fungal diseases in Sudan

AlbirairM.T.^1^FahalA.H.^2^DenningD.^3^,^4^1Blue Nile National Institute for Communicable Diseases (BNNICD), Wad Madani, Sudan2Mycetoma Research Center, University of Khartoum, Khartoum, Sudan3University of Manchester, Manchester, United Kingdom4National Aspergillosis Centre, Manchester University NHS Foundation Trust, Manchester, United Kingdom

**Objectives:** Sudan is a large north-African country with a population of about 40 million. Apart from mycetoma which is a substantial problem, the burden of fungal infections in Sudan is not well documented. Serious fungal infections are increasing with more immunocompromised patients, longer lives and a high frequency of respiratory diseases. The aim of this study was to estimate the burden of serious fungal infections in Sudan.

**Methods:** Epidemiological data reporting fungal infection rates in Sudan and nearby countries together with population estimates for general and specific at-risk patient groups were acquired. We used previously described deterministic modelling to estimate national incidence or prevalence.

**Results:** HIV rates are low in Sudan and we estimated 231, 1,378 and 3,568 cases with cryptococcal meningites, *Pneumocystis* pneumonia and oesophageal candidiasis, respectively. Asthma affects an estimated 480,000 adults, and 11,400 and 15,048 people were estimated to have allergic bronchopulmonary aspergillosis (ABPA) and severe asthma with fungal sensitisation (SAFS) respectively, probably with some overlap between them. We estimated 1,085 cases of all forms chronic pulmonary aspergillosis (CPA) with 50% assumed to occur post tuberculosis or are mis-diagnosed as TB. We estimated 354 cases of invasive aspergillosis complicating leukaemia, HIV and lung cancer. There are no COPD prevalence data. In the absence of local data, we have estimated candidaemia at 5/100,000 or 2,000 cases. We estimated ~530,000 women to be affected by recurrent vulvovaginal candidiasis (RVVC). As many as 3.6 million school-age children suffer from tinea capitis, based on a 21% rate. Mycetoma is a particular problem in Sudan and a minimum of 1.81/100,000 inhabitants or about 700 estimated cases are found there, predominantly eumycetoma. No reliable data exist on fungal keratitis, histoplasmosis or mucormycosis.

**Conclusion:** Our study revealed about 4.1 million (10.5%) of the Sudanese population suffer from serious fungal infections yearly with about 15,633 affected by life-threatening invasive fungal infections. Improvements in fungal disease diagnosis and management is required in Sudan.

### P245. Global epidemiology of mucormycosis: Preliminary analysis of the cases submitted to the zygomyco.net registry during the last 10 years

SkiadaA.^1^ChanderJ.^2^KlimkoN.^3^HamalP.^4^DolatabadiS.^5^Drogari-ApiranthitouM.^6^Lass-FlörlC.^7^KanjS.^8^LagrouK.^9^Arsic-ArsenijevicV.^10^ZimmerliS.^11^PetrikkosG.^12^11st Dpt. of Medicine, Laiko Hospital, Athens University, Athens, Greece2Department of Microbiology, Government Medical College Hospital, Chandigarh, India3Department of Clinical Mycology, Allergology and Immunology, North-Western State Medical University n.a. I.I. Mechnikov, Saint-Petersburg, Russian Federation4Department of Microbiology, Palacký University, Faculty of Medicine and Dentistry and University Hospital Olomouc, Olomouc, Czech Republic5Faculty of Engineering, Sabzevar University of New Technology, Sabzevar, Iran6Infectious Diseases Research Laboratory, 4th Department of Internal Medicine, General University Hospital “Attikon”, School of Medicine, University of Athens, Athens, Greece7Div Hygiene & Med. Microbiology, Med. Univ. Innsbruck, Innsbruck, Austria8Division of Infectious Diseases, American University of Beirut Medical Center, Beirut, Lebanon9Laboratory of Clinical Bacteriology and Mycology, Department of Microbiology and Immunology, Excellence Center For Medical Mycology (ecmm), KU Leuven, Leuven, Belgium10Medical Mycology Reference Laboratory, Faculty of Medicine University of Belgrade, Belgrade, Serbia11Department of Infectious Diseases, Inselspital, Bern University Hospital, University of Bern, University Hospital of Bern, Bern, Switzerland12Infectious Diseases, School of Medicine, European University of Cyprus, Nikosia, Cyprus

**Objectives:** Mucormycosis is an invasive fungal infection with high morbidity and mortality. Due to its rarity, registries are an important tool, used to collect information about risk factors, diagnosis and treatment of this potentially lethal infection. The aim of this study was to perform a preliminary analysis of the cases submitted to zygomyco.net, the ECMM/ISHAM Working Group on Zygomycosis global registry, during a 10-year period (2008-2018).

**Methods:** In order to submit a case, a participant must register to the site (www.zygomyco.net), giving his name, country of origin and e-mail address. Participants in the study can submit cases on-line in electronical CRFs. Alternatively, they can download a paper form and send it to the coordinators. The required data include demographics, underlying conditions, mode of transmission, clinical presentation, diagnosis, treatment and outcome. In order to be included in the study the case must pertain to a probable or proven case of mucormycosis. A data review committee reviewed all submitted cases.

**Results:** During the study period 365 cases were submitted from 16 countries (Austria, Belgium, Brasil, Czech Republic, Greece, India, Iran, Italy, Lebanon, Netherlands, Romania, Russia, Serbia, Switzerland, United Kingdom and USA). The largest proportion of cases (34%) were submitted from India. The analysis was done in the 330 cases which were complete. Sixty-two % of the patients were male. The mean age was 45y (range 1 month to 81 years). Underlying conditions were diabetes mellitus (37%), hematological malignancies (30%), other malignancies (7%), bone marrow transplantation (9%), solid organ transplantation (6%) and trauma (9%). Neutropenia was present in 25%. The main sites of infection were lungs (35%), sinus/orbital/cerebral region (34%), soft tissue (16%), disseminated (12%) and gastrointestinal (3%). Diagnosis was made only by histopathology in 11%, only by direct examination in 3%, only by culture in 7%, and with combined methods in the rest. Cultures were positive in 74.5% of cases (*Rhizopus* spp. 56%, *Lichtheimia* spp. 15%, *Mucor* spp. 8%, *Apophysomyces* spp. 6%, *Rhizomucor* spp. 4%, *Saksenaea* spp. 2%, *Syncephalastrum* spp. 2%, *Cunninghamella* spp. 1% and unspecified Mucorales 6%. Treatment was performed only by surgery in 3%, only by antifungals in 37% and by combined approach in 44%. In the remaining 16% of patients, either the diagnosis was made post-mortem or the patients declined treatment and left the hospital against medical advice. Crude mortality was 41%.

**Conclusion:** This is a preliminary analysis of the cases submitted to zygomyco.net in the period 2008 to 2018. A wide range of clinical presentations, underlying diseases and isolated species was observed. Diagnosis is still difficult in many parts of the world and mortality remains high. Further analysis of the data is going to highlight the differences occurring in various countries and the risk factors associated with mortality.

### P246. Isolation and phenotypic characterization of Sporothrix brasiliensis from cats with suspected sporotrichosis

Maschio-LimaT.^1^MarquesM.^2^AlmeidaB. De^1^LemesT.^1^BiancoL.^1^CaetanoM.^1^BrizzottiN.^2^SilvaC.^2^FernandesJ.^2^CastilhoE.^2^SiqueiraJ.P.^2^AlmeidaM.^2^1UNESP, São José do Rio Preto, Brazil2FAMERP, São José do Rio Preto, Brazil

**Objectives:** To confirm cases of suspected sporotrichosis in felines in the municipality of São José do Rio Preto, Brazil, by isolating and identifying the *Sporothrix* spp. in clinical samples of symptomatic animals; and to evaluate the clinical and epidemiological factors associated with the disease.

**Methods:** In a 15 months period (August 2017 to November 2018), 114 samples of secretion and/or biopsy of lesions from symptomatic cats were collected at the Center for Zoonoses Control and analyzed using traditional mycological techniques. Phenotypic features of the isolates were used to differentiate the species of the *Sporothrix schenckii* complex (*S. schenckii* var. *schenckii*, *S. brasiliensis*, *S. globosa*, *S. mexicana*, *S. luriei, S. albicans*). These features include presence or not of sessile pigmented conidia, colony growth rates at 30ºC and 37 ºC, and assimilation of sucrose and/or raffinose. Epidemiological aspects of the cats, which all had clinical suspicion of the disease, were analyzed based on the information presented in research sheets filled in at the time of collection by veterinarians.

**Results:** Regarding the origin of the animals, 65.79% (75) of the cats were erring, 23.68% (27) semi-domiciled, and 10.53% (12) fully domiciled. Most of the animals (99, 86.84%) were adults; 91 (79.82%) were males, and 23 (20.17%) were castrated. Multiple skin lesions were present in 82 (71.92%) animals, 67% (55) of them exclusively ulcerated. Indication of euthanasia was given to 66 (57.89%) cats. In relation to the mycological analyses, 83 (72.80%) samples were positive for fungus, exhibiting characteristics of the genus *Sporothrix*, i.e. creamy wrinkled colonies that become blackened due to the production of melanin, and septate hyphae with conidiophores giving rise to conidia in the shape of a daisy flower. The analyses revealed that the 83 isolates showed morphophysiological profiles characteristic of *S. brasiliensis*.

**Conclusion:** Considering the short period of analysis, and that the samples were provided from a single center, it is evident the high occurrence of feline sporotrichosis in the municipality of São José do Rio Preto, Brazil, caused by *S. brasiliensis*. The high percentage of positivity in adult, uncastrated and erring male felines highlights the need to create control measures in this city.

### P247. CauSTR – A rapid multiplex microsatellite typing tool for the multi-drug resistant yeast Candida auris

ThenE.^1^JongA. De^1^Ruiz-GaitanA.^2^,^3^Al-HatmiA.^1^,^4^Ben-AmiR.^5^,^6^KurzaiO.^7^,^8^EndeB. Gerrits Van Den^1^HagenF.^1^,^9^,^10^1Medical Mycology, Westerdijk Fungal Biodiversity Institute, Utrecht, Netherlands2Severe Infection Research Group, Medical Research Institute La Fe, Valencia, Spain3Department of Clinical Microbiology, La Fe University and Polytechnic Hospital, Valencia, Spain4Ministry of Health, Directory General of Health Services, Ibri, Oman5Tel Aviv Sourasky Medical Centre, Tel Aviv, Israel6Sackler Faculty of Medicine, Tel Aviv University, Tel Aviv, Israel7National Reference Center For Invasive Mycoses, Leibniz Institute for Natural Product Research and Infection Biology-Hans Knoell Institute, Jena, Germany8Institute For Hygiene and Microbiology, Julius Maximilian University of Wuerzburg, Wuerzburg, Germany9Medical Microbiology, University Medical Center Utrecht, Utrecht, Netherlands10Department of Dermatology, Jining No. 1 People’s Hospital, Jining, China

**Objectives:** Since the first description of *Candida auris* as a novel yeast pathogen it has quickly conquered hospital environments causing colonization of patients that ultimately can lead to bloodstream infections. During the past decade *C. auris* has been reported from 25 countries and a development of multi-drug resistance has been observed. Molecular epidemiological typing studies using whole genome sequencing, amplified fragment length polymorphism fingerprinting and multi-locus sequencing typing all identified four lineages linked to geographic regions. The aim was to develop a rapid and cost-effective molecular typing tool that relies on genetic diverse microsatellite loci.

**Methods:** The genome of *C. auris* strain B8441 (GenBank GCA_002759435.2) was used to search for repetitive loci using Tandem Repeat Finder (https://tandem.bu.edu/trf/trf.html). This resulted in 34 candidate loci that were tested by conventional PCRs using the CDC *C. auris* reference set. Nine microsatellite loci remained and were subsequently tested on a larger set of 92 *C. auris* isolates (India *n* = 28, Oman *n* = 24, Spain *n* = 13, Israel *n* = 11, Germany *n* = 6, Korea and Saudi Arabia *n* = 2 each, and *n* = 1 for Austria, Belgium, Japan, Kuwait, Switzerland and the Netherlands). Three multiplex PCRs were developed using fluorescently labeled primers to detect and differentiate the loci when analyzed on an ABI3730xL Genetic Analyzer.

**Results:** The genome size of *C. auris* strain B8441 is 12.366 Mbp and contains 2,374 microsatellites ranging from 1 bp repeat units up to a 461 bp repeat unit. Based on other fungal genomes it was expected that the smaller microsatellite units of 1–10 bp represent the majority of the microsatellites. But surprisingly, as can be observed in Figure 1, those with a repeat unit of 12, 15 and 18bp are more common than those containing 2–10bp repeat units. The final panel consisted of six tri-nucleotide, one with tetra-nucleotide, one nona-nucleotide and one undeca-nucleotide loci. The Simpson’s diversity index *D* ranged from 0.5387–0.8120 for individual loci, when all nine loci were considered. Fifty-two genotypes were observed with a Simpson’s *D* of 0.9656. The isolates from the endemic regions (India, Oman) were found to be clonal, while those from nosocomial outbreaks (Israel, Spain) could be linked to two different endemic regions. Single reported cases from Europe could be mostly linked to the South East *C. auris* population. 

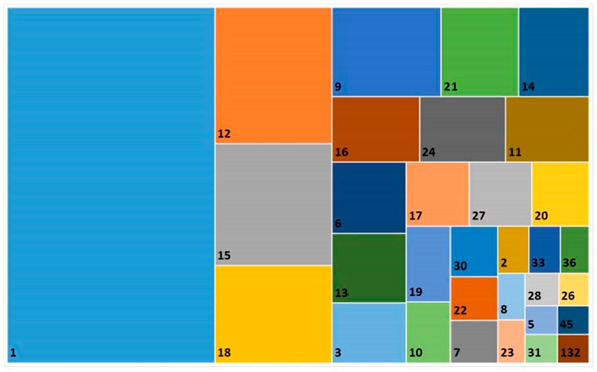



**Figure 1.** A selection of microsatellite types that occur at least ten times in the *C. auris* genome are shown. The number in each box stands for the size of the repeat unit and the size of the box represents the relative abundance of these specific repeat units.

**Conclusion:** Microsatellite loci are abundantly present in the *C. auris* genome but surprisingly the most variable repeat units (2–10bp) are sparsely distributed. The developed nine-loci microsatellite typing panel ‘CauSTR’ in this study is able to rapidly differentiate the four recognized lineages previously identified by other molecular methods and to observe subtle differences within *C. auris*.

### P248. Molecular characterization and epidemiology of Histoplasma isolates from Uruguay

CarbiaM.^1^EndeB. Gerrits Van Den^2^FernándezN.^3^SetropawiroM.^2^HagenF.^2^,^4^,^5^1Parasitology and Mycology, Instituto de Higiene, Montevideo, Uruguay2Medical Mycology, Westerdijk Fungal Biodiversity Institute, Utrecht, Netherlands3Sección Micología De La División Infectología, Hospital de Clínicas “José de San Martín”, Buenos Aires, Argentina4Medical Microbiology, University Medical Center Utrecht, Utrecht, Netherlands5Department of Dermatology, Jining No. 1 People’s Hospital, Jining, China

**Objectives:** Histoplasmosis is an invasive fungal infection, often encountered in HIV-positive subjects, caused by members of the genus *Histoplasma*. Historically three varieties were recognized within the single known pathogenic *Histoplasma* species, namely *H. capsulatum* variety *capsulatum*, variety *duboisii* (African histoplasmosis) and variety *farciminosum* (Equine histoplasmosis). However, a recent taxonomic revision based on whole genome sequencing, phenotypic and clinical data differentiated *H. capsulatum* var. *capsulatum* into four species: *H. capsulatum sensu stricto*, *H. mississippiense*, *H. ohiense* and *H. suramericana*. This study was initiated to retrospectively investigate the epidemiology of *Histoplasma* in Uruguay.

**Methods:** Forty-two clinical *Histoplasma* isolates obtained from 42 cases, collected during 1963-2019 and preserved at the Instituto de Higiene, Montevideo, Uruguay, were included. A set of 25 isolates that includes the four newly recognized *Histoplasma* species was used as reference. Fourteen patients (33.3%) were found to be HIV-positive, 12 (28.6%) had other underlying diseases/predisposing factors, 1 (2.4%) was immunocompetent and for the remaining 15 cases (35.7%) no clinical data was available. *Histoplasma* DNA was extracted using the CTAB-protocol and subsequently subjected to multi-locus sequence typing (partly sequencing of ITS, ARF, OLE, H-anti, M-anti and 60S) and amplified fragment length polymorphism fingerprinting. The mating-type *MAT1-1* and *MAT1-2* was determined by conventional PCR.

**Results:** There was a nearly equal distribution of mating-type *MAT1-1* and *MAT1-2* isolates, randomly distributed over time and among Uruguayan patients. By AFLP fingerprinting and multi-locus sequence typing it was observed that all Uruguayan isolates clustered together. However, based on the former molecular typing tool it appears that all these isolates were clonal while with the sequence-based approach more subtle clustering was observed. With both molecular tools the Uruguyuan *Histoplasma* isolates clustered closely together with the reference strain of *H. suramericana*.

**Conclusion:** Using AFLP fingerprinting and multi-locus sequencing typing a set of 42 *Histoplasma* isolates from Uruguay was found to belong to the newly described species *H. suramericana*. There was no link observed between the clinical background versus the molecular characteristics of the studied isolates.

### P249. The Burden of Serious Fungal Infections in Azerbaijan

OsmanovA.^1^DenningD.^1^,^2^HuseynovR.^3^1Global Action Fund for Fungal Infections, Geneva, Switzerland2Manchester Academic Health Science Centre, Manchester, United Kingdom3Azerbaijan Medical University, Baku, Azerbaijan

**Objectives:** We aim to calculate the burden of serious fungal infections in Azerbaijan.

**Methods:** We have performed search on published papers of serious fungal infections. Where no published data existed, we used population at-risk to identify burden of serious fungal infections. Our estimations were based on the model developed by LIFE (Leading International Fungal Education).

**Results:** Azerbaijan is in the South Caucasus region with the population of 9.8 M people and GDP per capita of 5,805 USD. There are 4,051,310 adult women (15-55 yrs) in Azerbaijan, so it was estimated that 176,961 women suffer from recurrent vulvovaginal candidiasis (rVVC) which is defined as = > 4 episodes/year. There are 8,000 PLHIV which is most likely an underestimate; 54% of them do not receive antiretroviral therapy. Hence, we estimate 2,673 and 1,291 patients with oral and oesophageal candidiasis respectively. There are probably 16 cases of cryptococcal meningitis and 64 cases of *Pneumocystis* pneumonia annually which is likely to be an underestimate. We have estimated 902 cases of chronic pulmonary aspergillosis (CPA) as a sequel of TB and 2,707 cases of CPA complicating other conditions such as pneumothorax, emphysema, and sarcoidosis; which results in the total of 3,609 cases of CPA. Based on asthma prevalence there are approximately 4,188 patients with allergic bronchopulmonary aspergillosis (ABPA) and 5,528 with severe asthma with fungal sensitisation (SAFS). We used a low European rate to estimate invasive candidiasis cases and there are approximately 493 and 74 with candidemia and invasive candidiasis respectively. We have estimated approximately 111 patients with invasive aspergillosis and 20 patients with mucormycosis. 

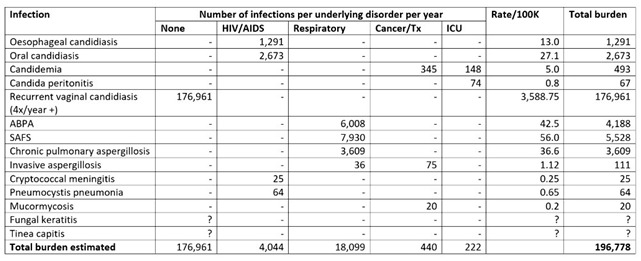



**Conclusion:** We have estimated 195,026 (2% of the population) people in Azerbaijan with serious fungal infections. Providing diagnostics tools for fungal infection will lead to better understanding of this problem and will allow to highlight this problem at the national level.

### P250. rDisability and disease after indoor macrocyclic Trichothecenes mycotoxin and microfungal exposure: exposure variable analysis indicates unexpected significant relationship between immunosuppression and MTHFR detoxification defects.

GrantI.H.^1^GellerJ.^2^SabathH.^3^1Community Medicine, New York Medical College, Tarrytown, United States of America2Information Revelations, Montclair NJ, United States of America3International Environmental Diagnostics Inc, Irvington NYw, United States of America

**Objectives:** Macrocyclic trichothecene mycotoxins are extremely toxic nanoparticulate exotoxins dangerous for all mammals, binding to surface dust where mold grow, aerosolized and inhaled if disturbed. They damage intracellular protein/DNA/RNA production and mitochondrial function causing premature cell death (apoptosis) on contact. A exotoxins, they remain toxic despite disinfection. They cause damage to mammalian skin, mucosa, phagocytes, neurons, and organs via systemic circulation after inhalation. Produced by *Stachybotrys* and *Trichoderma* in water-damaged indoor environments, they are more potently toxic than T2-toxin and doxynivalenol (DON, vomitoxin) mainly produced by *Fusarium* on agricultural grains. Objectives: Determine: - Severe illness frequency due to indoor mold/mycotoxin exposure. - Specific exposure risks - The impact of genetically-impaired intracellular MTHFR detoxification capacity. - Conditions that predispose to opportunistic fungal infection with such exposures (e.g. mucosal injury, immunosuppression, malnutrition)

**Methods:** Prospectively monitored 51(21M,30F) adults and children exposed to 30 Trichothecenes-contaminated indoor-environments with comprehensive environmental-mycological and medical investigations, fungal IGGs, urine mycotoxins(MCTs: Aflatoxin, Gliotoxin, Ochratoxin A, Trichothecenes), immune parameters(neutrophils, lymphocyte subsets, monocytes, IGG,IGA,IGM,IGE,subclasses), immunosuppressants, nutritional deficiencies (protein, Vitamin D,zinc), genetic Methylenetetrahydrofolate reductase (MTHFR) single-nucleotide-polymorphisms C677T&A1298C. Disease Severity Outcomes(DS), Environmental Mold-Contamination Severity(EMCS), Hazardous Exposure Activities(HEA), Exposure Duration (ED), MCT/Trichothecenes exposure(MTC/TE), and clinical findings were stratified and analyzed. Biostatistical analysis(Pearson Correlation Coefficients) correlated DS with EMCS, HEA, ED, MCT/TE, mucosal Injury(MI), Neurological disease, Cell-mediated Immunodeficiencies(CMIs), combined cellular-immunoglobulin immunodeficiencies, mucosal injury (MI), mold-specific IGG response(M-IGG), MCT excretion and MTHFR defects.

**Results:**

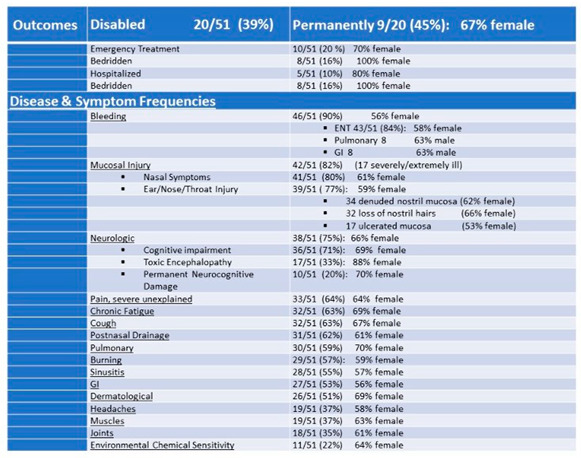

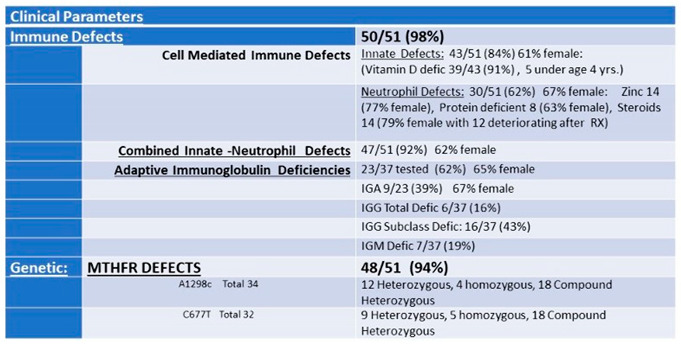





**CORRELATIONS:** Disease Severity based on formal disability classification/physical injury strongly correlated with Hazardous Activities (p = 0.000043), Neurological disease (p = 0.000263), Immunoglobulin deficiencies (p = 0.000923), Combined Cellular-Immoglobulin Deficiencies (p = 0.00502), Environmental Mold Contamination (p = 0.007531) and Mucosal Injury-the main expected injury from Trichothecenes inhalation (p = 0.018034). Mucosal injury strongly correlated with Neurological damage (p = 0.026842) and combined cellular-immune defects (p = 0.026842). Combined Cellular-Immunoglobulin Deficiencies strongly correlated Hazardous Activities (p = 0.009464). Development of mold-specific IGGs correlated with environmental mold-contamination (p = 0.26842) and Urine Trichothecenes(p<.00001). Females were more often adversely affected. Environmental Exposure Hazardous Activities (especially unprotected mold-disturbance, uncontained construction/repairs, basement-living, HVAC-contamination and sewage-overflow) correlated with enviromental Trichotehcenes level (p = 0.046588), Combined Cellular Deficiencies (p = 0.038993) and immunoglobulin deficiencies (p = 0.005952). Environmental Mold Contamination correlated with Disease Severity (p = 007531), Hazardous Activities (p = 0.002153), Environmental Trichothecenes level (p = 0.00502), multiple environmental mycotoxins (p = 0.02207), Combined Cellular-Immunoglobulin Deficiencies (p = 0.04658),immunoglobulin deficiencies (p = 0.032448) and elevated mold IGGs (p = 0.026842). Environmental mycotoxin contamination correlated with urine mycotoxin excretion (p = 0.046588). Environmental Trichothecenes contamination significantly correlated with Environmental Mold Contamination (p = 0.00502), Hazardous Activity (p = 0.046588) and Exposure Duration (p = 0.046588) Urine Macrocyclic Mycotoxin Score strongly correlated with the Environmental Mycotoxin Score (p = 0.046588).

**Conclusion:** Indoor exposure to macrocyclic Trichothecenes appears extremely hazardous. The high frequency of bleeding (90%), mucosal injury (82%), neurological damage (75%), pain (64%), chronic fatigue (63%), burning (57%), with expected respiratory/Gi /Dermatologic symptomatology is consistent with known Trichothecenes effects in animal models and military data. The high (94%)MTHFR methylation-defect incidence is striking compared to estimated USA 50-60% prevalence. MTHFR is a key intracellular detoxifying-enzyme protecting ribosomes/mitochondria RNA/DNA damage from Trichothecene injury via glutathione metabolism. Further investigation is needed to determine if Trichothecenes trigger genetic damage or autoimmunity. Urine macrocyclic Trichothecenes detection appears a useful marker for environmental macrocyclic Trichothecenes exposure. Public health education and epidemiological research are urgently needed to investigate correlation between environmental mold/mycotoxin exposure, multisystem illness, immunodeficiencies and female susceptibility.

### P251. Survivors of candidemia without antifungal treatment

HesstvedtL.^1^GaustadP.^2^AndersenC. Torp^2^MullerF.^2^NordoyI.^1^1Dept. of Infectious Diseases, Oslo University Hospital, Oslo, Norway2Dept. of Microbiology, Oslo University Hospital, Oslo, Norway

**Objectives:** Candidemia is associated with high mortality, which increases without antifungal treatment. The aim of this nationwide, retrospective study was to examine factors associated with 30-day survival in a subpopulation of candidemia patients who did not receive antifungal treatment.

**Methods:** We collected data by reviewing medical records from patients who were diagnosed with candidemia between January 1^st^ 2008 and December 31^st^ 2012. Age (at or below and above 65 years), sex, *Candida* species (*C. albians* vs. *C. glabrata*), predisposing illnesses (malignancy; gastrointestinal cancer, hematological malignancy or all other malignancy, organ transplantation, chronic obstructive pulmonary disease, diabetes mellitus, inflammatory bowel disease, intravenous drug users – IVDU) and health-care related risk factors (central venous catheter - CVC, urine catheter, mechanical ventilation, dialysis, abdominal surgery, neutropenia – neutrophils <0.5 x10^9^/L > 5 days, use of antibiotics, corticosteroids, chemotherapy, proton pump inhibitor –PPI or total parenteral nutrition-TPN) were recorded.

**Results:** In 144 candidemia patients (60.4% men, age 63.9 ± 16.2 years) not receiving any antifungal treatment, the overall 30-day survival rate was 36.8%. No significant difference was found between genders of untreated survivors (*n* = 53, 54.7% men, age 63.6±20.4) and untreated non-survivors (*n* = 91, 63.7% men, age 74.2±11.7) Overall significantly more of the patients < 65 years than ≥ 65 years survived without treatment (55.8% vs. 26.5%, p = 0.015). of predisposing illnesses malignancies excluding hematological and gastro-intestinal cancers was significantly less common in untreated survivors compared to non-survivors (23.5% vs. 76.5%, p = 0.02). of healthcare related risk factors the untreated survivors significantly less often were mechanically ventilated (34.5% vs. 65.5%, p = 0.049), less often had urine catheters (27.0% vs. 73.0%, p<0.001), received TPN (23.9% vs. 76.1%, p = 0.04) or PPI (28.4% vs. 71.6%, p = 0.03) compared to untreated non-survivors. Central venous catheters were more often removed in untreated survivors than in non-survivors (29.2% vs. 10.9%, p = 0.04). Untreated intravenous drug users (IVDU) (*n* = 9) all survived candidemia. No significant difference was seen between untreated survivors and non-survivors related to *C. albicans* or *C. glabrata* candidemia.

**Conclusion:** Patients surviving untreated candidemia were significantly younger, had less underlying malignancy and fewer risk factors for candidemia, in addition more often having their CVC removed than the patients who died without treatment. The prognosis in untreated candidemia is influenced by comorbidity and its subsequent treatment.

### P252. Mucormycosis – A clinicoepidemiological review of cases over 10 year

OSullivanM.Clinical Microbiology, Chrisitan Medical College, Vellore, Tamil Nadu, India

**Objectives:** Objectives: To study the epidemiology and outcomes of various agents causing mucormycosis in different clinical settings in a tertiary care hospital from South India.

**Methods:** Patients and methods: We reviewed details of 184 consecutive patients with culture-proven mucormycosis with consistent clinical syndrome and supporting features from September 2005 to September 2015.

**Results:** Results: The mean age of patients was 50.42 years; 70.97% were male. Unlike developed countries, *Rhizopus microsporus* (29/184; 15.7%) and *Apophysomyces elegans* (20/184; 10.8%) also evolved as important pathogens in addition to *Rhizopus arrhizus* in our setting. Paranasal sinuses (136/184; 73.9%) followed by musculoskeletal system (28/184; 15.2%) were the common areas of involvement. *A. elegans* typically produced skin and musculoskeletal disease in immune-competent individuals with trauma (12/20; 60%) and caused significantly lower mortality (*P* = 0.03). *R. microsporus* was more common in patients with haematological conditions (25% vs 15.7%) and was less frequently a cause for sinusitis than *R. arrhizus* (27.58% vs 10.9%). The overall mortality was 30.97%. Combination therapy with surgery and antifungals offered the best chance for cure.

**Conclusion:** Conclusions: Agents causing mucormycosis may have unique clinical and epidemiological characteristics.

### P253. Biliary Tract Candidiasis: an overview of the epidemiology in a general hospital

CostalesI.^1^MilianL.^2^VallinaE.^1^MartínezS.^1^TempladoA.^1^BogaJ.A.^1^PeláezT.^1^1Servicio De Microbiología Y Parasitología, HUCA, Oviedo, Spain2Servicio De Microbiología Y Parasitología, CAUSA, Salamanca, Spain

**Objectives:** Infections of the biliary tract are a common gastrointestinal disorder. Infections caused by *Candida* spp. and other fungal species have increased their incidence in the last few years. Knowing the autochthonous epidemiology is advisable in order to ensure an effective empirical therapy and an optimal management of the patients.

**Methods:** Biliary tract samples from patients diagnosed with acute cholangitis from January 2017 to June 2019 were included in the study. We considered only one one sample per patient and clinical episode. All isolates were identified using MALDI-TOF Biotyper® (Bruker Daltonics) methodology and yeast susceptibility testing were performed by Sensititre^TM^ YeastOne® (Thermo Scientific, TREK Diagnostic systems, UK). In case of polyfungical infections, all yeast isolates were identified and susceptibility test weas performed, but were considered as a single sample.

**Results:** From a total of 299 biliary samples analyzed, 80 of them (26.8%), corresponding to 65 patients, showed positive fungal growth. During the study period, the incidence of yeasts isolated from biliary tract increased from 2017 (0.49/1.000 admissions) to 2019 (0.95/1.000 admissions). The species most frequently recovered from biliary tract candidiasis episodes were *C. albicans* (56.25% of cases), *C. glabrata* (16.25%), *C. Krusei* (6.25%), *C. tropicalis* (6.25%), *C. parapsilosis* (6.25%) and other yeasts (8.75%). The percentages of isolation of different yeasts isolated in the first and last year of the study were respectively as follows: *C. albicans* (70.6% vs 53%), *C. glabrata* (11.7% vs 23.6%), *C. krusei* (5.9% vs 11.8%), *C. tropicalis* (0% vs 16.7%), *C. parapsilosis* (0% vs 13.3%) and other yeasts (11.8% vs 23.5%). A total of 51 cases (78.46%) of biliary tract candidiasis were monofungal infections and 14 (21.54%) were polyfungal infections.

**Conclusion:** In our hospital, the incidence of biliary tract candidiasis increased during the study period probably due to an improvement of culture methods, as well as greater clinical suspicion requesting a micologycal analysis of bile samples. Globally, *C. albicans* was the most frequent yeast species isolated, followed by *C. glabrata.* However, a significant increase in potentially fluconazole resistant species (*C. glabrata* and C. *krusei*) occurs in the last year of the study.

### P254. Prevalence of severe asthma with fungal sensitization (SAFS) in European population.

IunovidovaA.^1^KlimkoN.^2^SobolevA.^1^1Department of Clinical Mycology, Allergology and Immunology, North-Western State Medical University named after I.I, Mechnikov, Saint-Petersburg, Russian Federation2Department of Clinical Mycology, Allergology and Immunology, North-Western State Medical University n.a. I.I. Mechnikov, Saint-Petersburg, Russian Federation

**Objectives:** SAFS can be defined as a continuum of fungal sensitization with asthma at one end andallergis bronchopulmonary aspergillosis at the other. The main goal was to determine the prevalence of SAFS in the European population due to insufficient data.

**Methods:** Incident case and population data were obtained for SAFS fron the Surveillance, Epidemiology, and End Results between 2011 and 2018.

**Results:** Recent global estimates have found 10,000,000 cases of fungal asthma occur annually. The major assumptions made were based on previous studies of SAFS; severe asthma is thout to affect about 10% of adults and 33-70% are sensitised to fungi and that 2.77-2.90% of population have asthma. Although the prevalence of SAFS is uncertain as this disease has been recently described it was conservatively estimated that 30% of patients with severe asthma have or develop SAFS. Patients with SAFS probably constitute a significant proportion (~50% estimated) of the nearly 0.5 million asthma related deaths worldwide. The burden of SAFS has been estimated to 4,125,428 (+-174,033) cases in 43 countries.

**Conclusion:** The diagnostic gap between estimated burden and recorded cases numbers provides a clear cut target to close, to improve patient outcomes. Therecognitionoffungalinfectionsasamajorcontributortomortalityofseveralconditionsemphasizes the need for public health efforts in reducing the incidence and mortality of these infectious diseases

### P255. Epidemiological aspects of superficial mycosis among arabic school children in two localities in Sénégal (Thies and Touba)

BadianeA.DiongueK.SeckM.C.NdiayeM.NdiayeD.Parasitology and Mycology, Universite Cheikh Anta Diop de Dakar, Dakar, Senegal

**Objectives:** The objective of this study was to determine the prevalence of superficial mycosis in koranic schools of Thies and Touba, two cities located in western Senegal.

**Methods:** This study were conducted in children from seven (07) koranic schools in two cities of Senegal (Thies and Touba). A total of 210 children were recruited. For each child, depending of the lesions squama, hairs or nail pieces were collected using sterile material. Fungal pathogens were identified using direct examination with 30% Potassium Hydroxide (KOH) and culture on Sabouraud addionned with chloramphenicol and actidone (cycloheximide).

**Results:** The most prevalent lesion was tinea capitis (90%). The culture was positive for 25.1% of the pupils. The species identified were Trichophyton soudanense (85.18%), Microsporum audouinii langeronii (9.25%), Trichophyton rubrum (3.70%), and Chrysosporium keratinophilum (1.85%). Among infected participants, children aged 3 to 10 years were more contaminated (53.70%), followed by the age group of 11 to 15 years (44.44%) and participants over the age of 15 were less infected (1.85%). The prevalence of infection was higher among boys (85.18%).

**Conclusion:** This study showed a fairly prevalence of superficial mycoses among koranic school children.

### P256. Infections by Mucoraceous moulds recorded during a period of twelve years.

SamarasK.MarkantonatouA.-M.ZarkadaE.ZachrouE.VyzantiadisT.-A.First Department of Microbiology, Medical School, Aristotle University of Thessaloniki, Thessaloniki, Greece

**Objectives:** Mucoraceous moulds are very common in natural environment. They may rarely be the cause of severe or even fatal infections in immunocompromised patients, patients with uncontrolled diabetes mellitus or patients bearing deep and dirty wounds. The aim of this study was to record the cases of infections by mucoraceous moulds, microbiologically diagnosed in our laboratory and to detect the existence of any possible specific feature of these infections.

**Methods:** A retrospective recording was performed of all similar cases tested by our laboratory during the last twelve years

**Results:** Eleven cases of mucoraceous mould infections were recorded (7 male, 4 female). The fungal pathogens isolated were: 9 *Rhizopus arrhizus*, 1 *Mucor circinelloides* and 1 *Saksenaea vasiformis*. Seven patients were immunocompromised, two were suffering from uncontrolled diabetes mellitus and two of them had deep, dirty wounds. Five of the aforementioned cases concerned culture plates sent to the laboratory for identification of the fungal isolate. As for the other six specimens, direct microscopy of the clinical sample was positive for all of them. In 8 cases, antifungal susceptibility testing was performed and the range of MICs was 0.003-16 μg/ml for amphotericin B and 0.75-12 μg/ml for posaconazole. The outcome was favourable for five patients. In nine out of the eleven cases the infection was presented in spring or autumn, seasons that in our country are characterized by mild to moderate temperatures and relatively high humidity.

**Conclusion:** All patients had at least one risk factor for mucormycosis. A seasonal distribution was noticed, something that has been noted by other authors too. Positive direct microscopy in all clinical samples demonstrates the great importance of the procedure which provides promptly very characteristic images and contributes to the initiation of targeted treatment which may be life saving.

### P257. Molecular Epidemiology of Histoplasmosis in Germany

WilmesD.LosertH.L.LeibhanH.GerkrathJ.RickertsV.Fg16-mycology, Robert Koch Institute, Berlin, Germany

**Objectives:** Histoplasmosis is a systemic fungal infection caused by obligate pathogenic fungi of the genus *Histoplasma*. Autochthonous infections in wild and domesticated animals document the presence of Histoplasma in the environment in Germany. The aim of this study is to characterize the molecular epidemiology of Histoplasmosis in Germany by multilocus sequence typing (MLST) of *Histoplasma* cultivated from patients diagnosed in Germany and to compare them to strains characterized by a modified MLST from infected animals in Germany.

**Methods:** Since the year 2000, we participated to the diagnosis of 76 human cases (mean = 4/year) and 10 animal cases (7 cats, 3 badgers) of proven and probable infection. Twenty-two *Histoplasma* strains cultivated at our Laboratory, including two CBS reference strains were typed using a 4 gene scheme (arf, H-anti, ole and tub) previously described by Kasuga (1). Concatenated sequences were aligned with reported sequences of worldwide isolates. Then a modified MLST for formalin fixed paraffin (2) embedded samples was performed on pathology blocks from veterinary samples. Neighbour joining trees were constructed to demonstrate relationships using the Geneious software suite.

**Results:** All four genes could be sequenced for 16 of 22 isolates while the amplification of the ole gene failed for 6 isolate. The phylogenetic analysis demonstrated clustering of the German patient isolates with previously defined clades (African: *n* = 7; Latin American: *n* = 7; Bat associated clade1: *n* = 1; Netherlands: *n* = 1) corresponding to the patients travel history. of note, 3 patient isolates clustered with the Eurasian clade, in which also 7 animal samples (5 cats and 1 badger) clustered, suggesting related organisms. Two of the patients had a travel history to Southeast Asia, while for one no travel history was reported.

**Conclusion:** Our study demonstrates that histoplasmosis in Germany is mostly imported from endemic regions including Latin America, Africa and Southeast Asia. However, clustering of human isolates and veterinary cases from Germany within the Eurasian clade suggest the presence of *Histoplasma* in the German environment. Further molecular studies on human and veterinary cases and environmental sampling are needed to define environmental niches.

### P258. Burden of fungal infections in Morocco

LmimouniB.E.^1^AzelmatS.^2^HennequinC.^3^DenningD.^4^1School of Medicine and Pharmacy, University Mohamed The Fifth, Parasitology and Medical Mycology Lab, Military Teaching Hospital Mohamed the Fifth, Hay Riad, Rabat, Morocco2Parasitology and Medical Mycology, Military Teaching hospital Mohammed the Fifth, Rabat, Morocco3Parasitology-mycology, APHP, Hôpital St Antoine, Paris, France4University of Manchester, Manchester, United Kingdom

**Objectives:** We report for the first time in Morocco burden estimates of fungal infections. The aim of this study was to estimate the incidence and prevalence of fungal infection in Morocco.

**Methods:** We used the national demographic data and performed a PubMed and Scopus search to retrieve all published papers on fungal infections from Morocco. We have also modelled the likely burden of serious fungal infections using methodology previously published by LIFE.

**Results:** We report for the first time the main causes of these diseases in our country. Superficial mycoses and dermatophytic infection (onychomycosis, tinea capitis, others …) are very commonly diagnosed, We have found 13,294 cases in the published papers. Deep fungal infection are less found in the literature. Modlelling shows higher rates for all infections where modelling is possible, especially for pulmonary fungal infections. Literature Modelling Infection Rate/100,000 Total burden Rate/100,000 Total burden Cryptococcal meningitis 0.32 115 0.7 252 Pneumocystis pneumonia 0.22 78 1.2 420 Invasive aspergillosis 0.88 315 1.9 674 Mucormycosis 0.01 4 0.2 72 Chronic pulmonary aspergillosis post TB 0.74 266 8.6 3085 Allergic bronchopulmonary aspergillosis (ABPA) 0.01 4 51 18,400 Severe asthma with fungal sensitization (SAFS) 0.28 100 67 24,292 Candidaemia 1.47 528 5 1800 Oral Candidiasis 0.25 90 22 7800 Mycetoma 0.11 40 Recurrent Candida vaginitis (>4x/year) 0.17 30 2755 496,000 Histoplasmosis 0.01 4 - - Skin dermatophytic infection 13.97 5031 - - Onychomycosis 14.08 5069 - - Tinea capitis 8.87 3194 - - Deep dermatophytosis 0.02 10 - - Total burden estimated 14522 551,400

**Conclusion:** A focus on improving the diagnosis and epidemiological data related to fungal infection is necessary in Morocco.

### P259. Fusarium keratitis in the Netherlands (2005 – 2016).

SantosC. Oliveira Dos^1^KolwijckE.^1^EgginkC.^2^VerweijP.E.^3^1Medical Microbiology (777), Radboud University Medical Center, Nijmegen, Netherlands2Ophthalmology, Radboud University Medical Center, Nijmegen, Netherlands3Medical Microbiology, RadboudUMC, Nijmegen, Netherlands

**Objectives:** Recognizing the clinical picture of fungal keratitis is difficult. As a consequence, the fungal diagnosis might be delayed due to primary therapy with antibacterial agents and topical corticosteroids, resulting in spreading of the fungus in the cornea which ultimately may lead to more severe infection and monocular blindness. There seems to be a trend of increasing incidence of fungal keratitis in the Netherlands, especially caused by *Fusarium* species. Here, we describe the epidemiology and clinical management of fungal keratitis in the Netherlands.

**Methods:** The Dutch national reference center for medical mycology has received *Fusarium* strains isolated from 89 Dutch fungal keratitis patients, in the period 2005 up to 2017. The clinical data was added in retrospect by the ophthalmologists. All *Fusaria* were identified up to genus level using conventional techniques and/or molecular techniques. Antifungal susceptibility testing was performed according to the method described by the EUCAST. The antifungal agents tested were amphotericin B, voriconazole and natamycin. Susceptibility to the antisepticum chlorhexidine was also tested.

**Results:** In this period there was a mean incidence of *Fusarium* keratitis of 0.5 (range 0 – 1.5) per million per year. The male to female ratio was 1:3 (p = 0.014). The use of contact lenses in this cohort was high; 92.9% of patients with reported use or non-use of contact lenses. The time between start of symptoms to the diagnosis of fungal keratitis was significantly longer in the group with therapy failure opposed to the group with restored vision; respectively 22 days versus 15 days (p = 0.024). The need of a corneal transplantation or enucleation of the affected eye occurred in 27 cases (*n* = 20 [22.5%] resp. *n* = 7 [7,9%]). Enucleation occurred in patients with delayed diagnosis of the fungal culprit (>14 days after start symptoms). The mean visual acuity was moderately impaired with a logMAR of 0.8 (95% CI 0.6 – 1) at the time of isolation of the *Fusarium* strain. The final visual outcome had an average logMAR of 0.2 (95% CI 0.1 – 0.3), the equivalent of mild visual impairment. The treatment strategies differed greatly between the cases. of note, five patients were treated with chlorhexidine 0.02% monotherapy after *Fusarium* was isolated with good results; 4 patients had a successful outcome. The most frequently isolated species was *F. oxysporum* (*n* = 22, 24.7%) followed by *F. solani sensu stricto* (*n* = 16, 18%) and *F. petroliphilum* (*n* = 8, 9%). The lowest MICs were obtained with amphotericin B followed by natamycin, voriconazole, and chlorhexidine. 

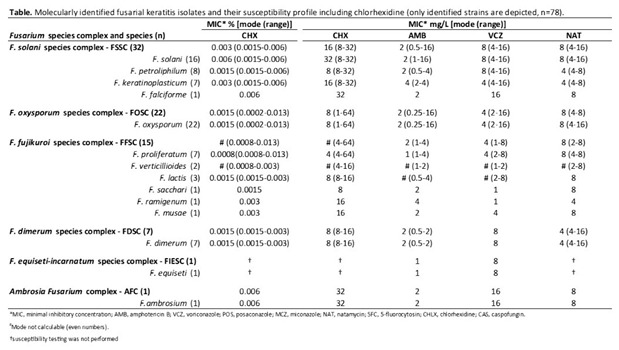



**Conclusion:** In our population trauma is not the main route of infection. An important risk factor in the Netherlands seems to be the use of contact lenses. When correct diagnosis and antifungal treatment is delayed the risk of losing the affected eye is great. The lack of a specific national guideline is reflected in the greatly varying treatment strategies. Chlorhexidine seems to be a good addition to the therapeutic options.

### P260. rFungal invasive infections after kidney tranplant: a monocenter study

ZaidanM.^1^MechrefZ.^1^Frank-SoltysiakM.^2^NguyenD.-P.^2^DurrbachA.^1^AngoulvantA.^3^,^4^1Nephrology, Hôpital Bicêtre, APHP, Le Kremlin Bicêtre, France2Dim Hups / Aphp Wind, Hôpital Bicêtre, APHP, Le Kremlin Bicêtre, France3Génétique Quantitative et Évolution – Le Moulon, INRA – Université Paris-Sud – CNRS – AgroParisTech, Gif-sur-Yvette, France4Mycology-parasitology, Hôpital Bicêtre, APHP, Le Kremlin Bicêtre, France

**Objectives:** Invasive fungal infections constitute an important cause of morbidity and mortality in solid organ transplantation recipients. Epidemiology of invasive fungal infections (IFI) after kidney transplantation in the modern era of immunosuppression is poorly described. We aim to describe the IFI epidemiology in our centre.

**Methods:** We retrospectively analyzed the epidemiology of IFI in a single-center cohort of kidney transplant adult recipients between January 2011 and December 2018.

**Results:** There were 1646 kidney transplanted patients hospitalized during the study period; 68 (4%) cases of IFI were identified. Sex ratio (M/F) was 1.1 and mean age 63 years (44 – 77). IFI occurred 12 (1-144) months after transplantation, in patients with immunosuppressive therapy containing tacrolimus, mofetil mycophenolate and corticosteroids. 100% of them had renal insufficiency, 68% had diabetes and 50% were neutropenic at the time of the IFI diagnosis. *A. fumigatus* proven or probable invasive aspergillosis (IA) was the leading cause (*n* = 28, 1.7 %) followed by pneumocystosis (*n* = 20, 1.22%), candidemia (*n* = 15. 0.92%) and other IFI (*n* = 6 (0.3%). Urinary source was identified in 77% of patients with candidemia. *C. albicans* (60%) was the leading species followed by *C. glabrata* and *C. parapsilosis* (20% each). Loss of graft occurred in 20% of them. Mortality was 50%, 40% and 10% for IA, candidemia and pneumocystosis respectively

**Conclusion:** In our centre IFIs, excepted pneumocystosis, were associated with high mortality rates in renal transplant patients. IA was the most common and was associated with the higher mortality. Candidemia occurred rarely but was associated with high mortality and loss of graft rates.

### P261. Prevalence and biotypes of vulvovaginal Candidiasis among reproductive-age women in Sarajevo.

KeserS. Kotoric^1^PilavA.^2^BektašS.^1^HodžićD.^1^BalihodžićA. Obradović^1^1Microbiology, P.I. INSTITUTE FOR PUBLIC. HEALTH OF CANTON. SARAJEVO, Dr. Mustafe Pintola 1/III 71 000 Sarajevo, Sarajevo, Bosnia and Herzegovina2Social Medicine, P.I. INSTITUTE FOR PUBLIC. HEALTH OF CANTON. SARAJEVO, Dr. Mustafe Pintola 1/III 71 000 Sarajevo, Sarajevo, Bosnia and Herzegovina

**Objectives:** The study aimed to determine the prevalence of VVC among reproductive-age women attending the Institute for Public Health of Canton Sarajevo. and to know the fungal profile/biotypes of *vulvovaginal candidiasis* using *Candida CHROMagar.*

**Methods:** This is a 4-month cross-sectional study performed at the Department of Medical Microbiology of the Institute for Public Health of Canton Sarajevo. A pair of high vaginal swab and endocervical swab samples was collected from each of 213 individual participating subjects. They were separately inoculated on Sabouraud’s dextrose agar and incubated aerobically at 33°C for 48 hours and *Candida CHROMagar*. Infection with *Candida species* was diagnosed by microscopy of a saline wet mount, Gram-stained smear and colony growth on SDA and *Candida CHROMagar*.

**Results:** of the 213 sampled, 109 were positive for candidiasis giving a prevalence rate of 51.1%. Candidiasis was observed mostly among the 20- to 30-year age-group. The ages of the women ranged from 16 to 45 years with a mean of 26.8 (standard deviation ± 4.93).Women aged 26–30 years recorded the highest prevalence of 37.6% (41/109). *Candida albicans* was the most prevalent species isolated in 85.3% of the women followed by *Candida crusei* 12.8% *and Candida parapsilosis* 1.83%.

**Conclusion:** High prevalence rate of vulvovaginal candidiasis among females in Sarajevo and the predominant agent *is Candida albicans*. In addition, correct identification of *Candida species* could play an important role in management and treatment of VVC.

### P262. Cryptococcal antigenemia in HIV-uninfected patients with stroke in Nigeria

FayemiwoS.A.^1^MakanjuolaO.^1^BongominF.^2^NwaokenyeJ.^3^AkinyemiR.^4^DialaS.^4^FaniyanM.^5^OwolabiM.^4^1Department of Medical Microbiology & Parasitology, College of Medicine, University of Ibadan, Ibadan, Nigeria2Department of Medical Microbiology and Immunology, Gulu University, Kampala, Uganda3Department of Medical Microbiology & Parasitology, University College Hospital, Ibadan, Nigeria4Centre For Genomic and Precision Medicine, College of Medicine, University of Ibadan, Ibadan, Nigeria5Department of Medicine, University of Ibadan, Ibadan, Nigeria

**Objectives:** Stroke continues to be a leading cause of morbidity and mortality globally. Infections have been associated with stroke occasionally, but are not considered to be a direct cause, and so they are not often included in the traditional stroke workup and management. Sub-Saharan Africa also has the highest incidence, prevalence, and fatality from stroke in the world. Cryptococcal infections occur globally and in a wide variety of individuals, ranging from those who are severely immunocompromised to those who are assumed to be immunocompetent. Recurrent strokes secondary to cerebral infarction may occur and has been reported in immunocompetent persons following reactivation of a latent infection. The study aimed to determine the prevalence of Cryptococcal antigenaemia in stroke patients, to identify risk factors for Cryptococcal antigenaemia in stroke patients and to compare Cryptococcal infection among stroke and non-stroke patients.

**Methods:** The Stroke Investigative Research and Education Network (SIREN) project, which this study was a part of, is a multicentre, multidisciplinary, case-control study carried out in Ghana and Nigeria. One of the objectives of this extensive study is to evaluate the risk factors of stroke in blacks in Sub-Saharan Africa. Cases were adults (aged ≥18 years) with stroke confirmed by CT or MRI. Controls were age-matched and gender-matched stroke-free adults (aged ≥18 years) recruited from the communities in catchment areas of cases. A simple random sampling was used to select blood samples of 50 stroke patients and 50 controls from the study population. Inclusion criteria were stroke patients and controls who were HIV negative confirmed by HIV testing. Serum from study participants was tested for the presence of Cryptococcal antigen with the Cryptococcal Antigen Lateral Flow Assay (LFA) (Dynamiker Cryptococcal Antigen Lateral Flow Assay (LFA)), a point-of-care test for the diagnosis of Cryptococcosis.

**Results:** Ninety-nine HIV-uninfected patients (50 cases and 49 controls) constituted the study population. Majority of the participants were males (*n* = 69, 70%), with a median age of 57 (range: 33-86) years. 36 (*n* = 50, 72%) for cases and 22 (*n* = 49, 44.9%) were hypertensive while six (*n* = 50, 12%) for cases and 14 (*n* = 49, 28.6%) were diabetic. Overall, CrAg was positive in 16% (*n* = 16) of the participants. Similar proportions of the cases and controls were CrAg positive (18% vs. 14%, p = 0.786). 8(*n* = 9, 89%) for cases and 3 (*n* = 7, 43%) hypertensive particiapnts were CrAg postive. Death rates was the same between CrAg positive and CrAg negative patients (13% Vs 13%, p = 1.000 by Fisher’s exact test). Multinomial Logistic Regression DM (odds ratios, 0.77 (95%CI: 0.55-1.08); p = 0.130).

**Conclusion:** Cryptococcal Infectious causes should be considered in the differential and work up of stroke in certain patient populations, especially in Sub-Saharan Africa, and appropriate treatments should be initiated to minimize adverse stroke-related outcomes.

### P263. Epidemiological, diagnosis and management of mycetoma cases in three hospital centers from 2008 to 2018 in Senegal

SowD.^1^NdiayeM.^2^SarrL.^3^DiousseP.^2^LyF.^2^FayeB.T.^4^SyllaK.^4^RanqueS.^5^FayeB.^4^1Service De Parasitologie-mycologie/section De Biologie, UFR Sciences de la Santé, Université Gaston Berger, Saint-Louis, Senegal2Service De Dermatologie, Faculté de médecine, UCAD, Dakar, Senegal3Service D’orthopédie, Faculté de médecine, UCAD, Dakar, Senegal4Service De Parasitologie, Faculté de médecine, UCAD, Dakar, Senegal5IHU-Méditerranée Infection, Marseille, France

**Objectives:** Mycetoma is a neglected tropical disease, characterized by the formation of tumor like-swellings and grains. This disease transmitted through subcutaneous tissue is caused by a range of actinomycetes (actinomycetoma) and fungi (eumycetoma). Treatments for eumycetoma are unsatisfactory leading to high amputation rates. Senegal represents one of the country where the disease is highly endemic. However, many mycetoma cases are underdiagnosed/misdiagnosed due to the lack of informations in health workers and at the community level. The purpose of this study is to generate accurate data of the geographical distribution of Mycetoma spp. cases and their management in hospital settings in Senegal.

**Methods:** In this retrospective study we report epidemiologic, clinical, diagnostic and therapeutic data of mycetoma observed in three hospital centers in Senegal in a 10 year-period (2008 to 2018).

**Results:** A total of 193 cases were included in the study. One hundred and sixty two patients (83.5%) were recruited in two hospitals of the capital Dakar and the other patients (16.5%) were diagnosed in Thies region located at 70km from Dakar. Most of the cases (47.2%) were eumycetoma followed by 36.8% for actinomycetoma cases and 16.1% undetermined form. The mean age was 38.3 years and the patients were predominately aged between 15 and 45 years (68.4%). There was a male predominance with a sex ratio of 2.94 and most patients were agricultural workers. One hundred fifty six patients (80.8%) have used local and oral phytotherapy before attending hospital with sometimes an evolution beyond 10 years (19.1%). The main affected localization was lower limbs (90.6%) and most of the patients had lesions over 10cm. Grains were observed in 85% cases including white (25.6%) and yellow (4.3%) grains. The diagnosis was difficult in many cases without positive results in direct microscopy, culture and histopathology explaining that 16.1% remained undetermined. In most of cases, actinomycetoma form were treated with cotrimoxazole plus amoxicillin/clavulanic acid plus streptomycin while eumycetoma cases were treated with terbinafin. The surgery was done in 100 cases (51.8%) including amputation performed in 45 patients.

**Conclusion:** This study has shown the difficulty of the diagnostic of mycetoma in Senegal, the delay in management cases and the lack of adequate therapeutic tools. The baseline data provided will contribute to the establishment of a surveillance system, in the context of advocating for much more attention.

### P264. Clinical and Microbiological Characterization of Candida parapsilosis complex Infection in a Tertiare Care Hospital from Cali, Colombia

Ceballos-GarzonA.^1^MartinezL.^3^Parra-GiraldoC.M.^2^MontealegreA.M. Sanz^3^MartinezD.M.^3^SuarezF. Rosso^3^1Department of Parasitology and Medical Mycology of The University of Nantes., Universite de Nantes, Nantes, France2Unidad de Proteómica y Micosis Humanas, Grupo de Enfermedades Infecciosas Departamento de Microbiología, Facultad de Ciencias, Pontificia Universidad Javeriana, Bogotá D.C, Colombia, Bogota, Colombia3Centro de investigaciones clínicas Fundación Valle del Lili, Cali, Colombia

**Objectives:** To determine the association between the resistance of *C. parapsilosis* complex to Fluconazole and adverse clinical outcomes in patients hospitalized in the Valle del Lili Foundation. **Methods:** An observational prospective study was conducted. The study included patients with *C. parapsilosis* complex (CPC) isolations attended at Valle del Lili Foundation (FVL) from 2016 to 2017. The 55 strains were identified by MALDI-TOF MS. Minimal inhibitory concentrations (MIC) were determined by broth microdilution (M27 A3 CLSI). Statistical univariate analysis was performed; differences between resistant cases and non-resistant cases were assessed through U Mann-Whitney test, Pearson chi-2 test or Fisher exact test. **Results:** 55 patients had CPC isolations during the study period: 18 newborns, 13 children, and 24 adults. Most isolations were from blood cultures (*n* = 31) (14 of them newborns), bronchoalveolar lavage (*n* = 9), peritoneal fluid (*n* = 8), and catheter tips (*n* = 3). The resistance was 36%. 52 strains were *C. parapsilosis* s.s., of them, 20 were FCZ resistant; 3 strains were *C. orthopsilosis*, all of them FCZ sensitive. The MIC50 = 1 µg/ml and MIC90 = 16 µg/ml. Patients with previous antifungal treatment had a higher risk of FCZ resistance (RR: 2. 14, 95% IC 1. 07-4. 26). The mortality brute rate was 30%, patients with renal failure death risk (RR: 2.8, 95% CI: 1. 4-6. 9), heart disease (RR: 3.14, 95% CI: 1. 4–6. 9), and hemodialysis (RR: 2.97, 95% CI: 1.38-6.4). Candidemia was present in 50% of deaths among children with parenteral nutrition.

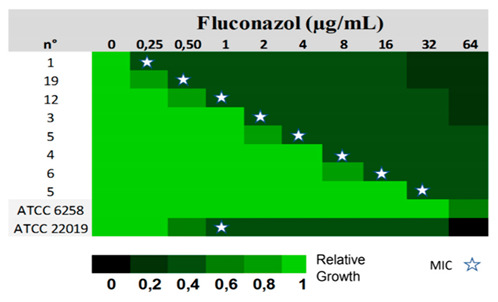



**Figure 1**. Minimal inhibitory concentrations (MIC) to *C. parapsilosis* complex (*n* = 55). Strains ATCC 6258 and ATCC 22019 was used as a control.

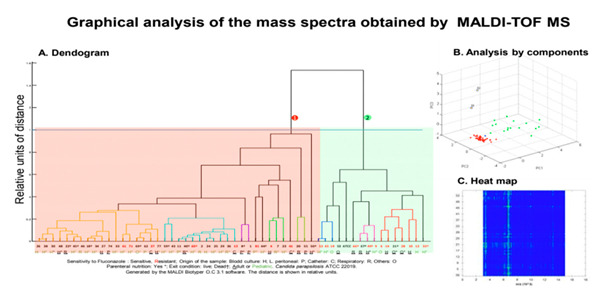



**Figure 2.** Graphical analysis of mass spectra obteined by MALDI-TOF MS of cytoplasmic protein extracs from clinical isolates of *C. parapsilosis* complex.

**Conclusion:** Fluconazole resistance in CPC has increased in the last decade. Newborns receiving parenteral nutrition had a higher proportion of CPC fungemia, we also found higher mortality rates among this population.

### P265. Characterisation and Antifungal Susceptibility of Cryptococcus species Isolated from Rodents

TirkeyN.N.SinghK.Animal Mycology Laboratory, Zoology, Mmv, Banaras Hindu University, VARANASI, India

**Objectives:** Rodents are recognized as intermediate hosts of zoonotic diseases that represent a serious threat to human health. *Cryptococcus* is an opportunistic fungal pathogen found worldwide and causes life-threatening meningoencephalitis especially in immunocompromised host. In this view, the present study was conducted to investigate the role of rodents in the epidemiology of cryptococcosis caused by *Cryptococcus neoformans*.

**Methods:** Rodents (*Rattus rattus, Mus musculus castaneus, Mus musculus domesticus*) were trapped from the city of Varanasi, India (eastern Uttar Pradesh). After behavioural and clinical examinations, they were euthanized. The organs were removed aseptically for histopathology and isolation of fungi. For isolation, homogenates were cultured on Sabouraud’s dextrose agar (SDA). The isolates were then grown in Staib’s and tobacco agar medium. In addition, all isolates were identified on the basis of different biochemical tests such as germ tube test, urease test and canavanine-glycine-bromothymol blue (CGB) test. Further, molecular characterization was carried out by using PCR and DNA sequence analysis. *In vitro* antifungal susceptibility profiles of *Cryptococcus* strains were performed by using broth microdilution method recommended by CLSI (2009) M27-A3.

**Results:** Out of 20 screened, 2 rodents were positive for *Cryptococcus* spp. At the necroscopy, there were no pathological lesions. *Cryptococcus* species were isolated from the lungs, kidney, brain and blood of the animals. The isolates were subjected to germ tube test and pseudohyphae were not observed after 24 hrs of incubation at 37°C in mammalian serum, therefore the test was considered as negative. The presence of brown colonies on Staib’s and tobacco agar media confirmed that the isolated pathogen is *Cryptococcus.* Further the isolates of *Cryptococcus* were tested for urease production using Christensen urea citrate agar medium in which all isolates were found to hydrolyse urea rapidly and reddish pink colour was obtained confirming it to be *Cryptococcus neoformans.* All isolates were negative for Canavanine glycine bromothymol blue (CGB) as they failed to produce a colour change in the medium. Molecular analyses showed that these isolates were *C. neoformans*. Histological sections of lungs stained with haematoxylin and eosin stain revealed the presence of *C. neoformans* like encapsulated cells. In *in vitro* antifungal testing, isolates were found susceptible to amphotericin B.

**Conclusion:** All the rodents screened are synanthropic. As they were found positive for *C. neoformans*, they could be a potential threat for human health. Therefore wide epidemiological survey should be conducted.

### P266. Prevalence and characterizaton of otomycosis among patients attending Ear, Nose and Throat clinics at Abia State University Teaching Hospital, Aba, South Eastern Nigeria

NgwoguA.^1^NgwoguK.^2^1Medical Microbiology, Abia State University Uturu, Aba, Nigeria2Histopathology, Abia State University Uturu, Aba, Nigeria

**Objectives:** Otomycosis or Fungal Otitis External is a superficial infection of the outer ear canal. This could lead to complications involving the middle ear. The prevalence of otomycosis has been studied in several parts of the world. It is seen more frequently as an opportunistic infection in Immunocompromised people and those with ear injury. This study was carried out to determine for the first time, the prevalence of otomycosis in patients attending the Ear, Nose and Throat (ENT) Clinics at Abia State University Teaching Hospital (ABSUTH), Aba, South Eastern Nigeria and to isolate and characterize fungal agents responsible for otomycosis.

**Methods:** A total of 380 patients presented at the ENT Clinics during the period of study. Out of this number, 100 samples were collected from those patients who presented with symptoms of ear infection (pruritis, otalgia, otorrhea and hypacusis) by the ENT Consultant Physician. Patients with other types of ear disorders other than otitis media and otitis externa and patients under antibiotic therapy within the last 2 weeks were excluded from the study. Demographic characteristics of the patients were noted. The samples which consisted of swabs and scrapings were examined in 10% potassium hydroxide solution and cultured on Sabouraud Dextrose Agar containing Chloramphenicol. Fungal growth were identified using slide culture techniques and physiological tests which include growth on some cspecialized media, germ tube test, urease test, growth in Corn Meal Agar Containing Tween 80, nitrate assimilation, sugar assimilation and sugar fermentation tests.

**Results:** Out of the 100 samples examined, (68% males and 32% females) were positive for fungal structures both in microscopy and culture. Males were significantly more affected than females in the ratio of 19: 4 (P<001). Although ear infection was highest within the age group 1-10 years, fungal infection was highest within the age group 31- 40 years. Otomycosis was highest amongst outdoor, unskilled labourers (8%), followed closely by students (6%) and lowest among civil servants (2%). The aetiologic agents isolated were Candida albicans (13%), Candida tropicalis (5%), Aspergillus fumigatus (3%) and Aspergillus niger (2%).

**Conclusion:** This study has shown for the first time that fungi are implicated in ear infections in ABSUTH. The prevalence of otomycosis in the present study is high. Clinical follow up with mycological diagnosis are important since symptoms (pruritis, otalgia, otorrhea and hypacusis) are not specific. In fact many cases of otomycosis are clinically misdiagnosed as otitis media leading to chronicity. Laboratory diagnosis and management of otomycosis though challenging is very important. This is to detect the causative organism and avoid wrong prescription of drugs. Recurrences are common in immunocompromised patients than in immunocompetent patients. General practitioners, otologists and laboratory personnel should remain alert for otomycosis. Diagnosis of ear infections should not be made on clinical diagnosis only, it should be confirmed by laboratory diagnosis. Specimens must be collected by an experienced Medical Personnel by aid of otoscope. The frequent use of earphones, pricking the ear with hard objects, and traumatic attacks on the ear are factors that could predispose to otomycosis.

### P267. Six Year Surveillance Study of Scedosporium and Lomentospora Isolates Collected from Humans in the United States (2012 - 2018)

BaumanS.GibasC.SandersC.MccarthyD.PattersonH.WiederholdN.Fungus Testing Laboratory, The University of Texas Health Science Center at San Antonio, San Antonio, United States of America

**Objectives:** *Scedosporium* species and *Lomentospora prolificans* are increasing causes of invasive infections in immunocompromised hosts, and many isolates have reduced susceptibility or are resistant to available antifungals. This is especially true for isolates of *L. prolificans*, which are often resistant to all clinically available antifungal agents. Although these fungi can be common colonizers in patients with cystic fibrosis, invasive infections can occur, as can breakthrough infections in persistently neutropenic and or lymphopenic patients, as well as lung transplant recipients. Our objective was to evaluate the species distribution and antifungal susceptibility patterns of *Scedosporium* and *Lomentospora* clinical isolates in the United States over a 6-year period.

**Methods:** The electronic database of our reference mycology laboratory (Fungus Testing Laboratory, UT Health San Antonio) was queried for *Scedosporium*/*Pseudallescheria*/ *Lomentospora* isolates received between September 2012 and October 2018. Fungal species identification was performed when requested by observation of phenotypic characteristics combined with DNA sequence analysis of ITS rDNA and the calmodulin gene. Antifungal susceptibility testing was performed by broth microdilution according to the CLSI M38 standard.

**Results:** 1376 isolates were received over this 6-year period. The overall species distribution as well as the distribution of species identified by our laboratory are shown in Table 1. Overall, *S. apiospermum* was the most prevalent species, followed by *S. boydii* and *L. prolificans*, and these 3 species accounted for ~89% of the isolates received. Voriconazole had the most potent *in vitro* activity of the azoles and amphotericin B tested against *Scedosporium* species (Voriconazole geometric mean [GM] MIC range 0.79 – 0.83 mg/L Table 2). In contrast, the activity of isavuconazole was markedly reduced (GM MIC range 6.25 – 6.68 mg/L). Caspofungin and micafungin also demonstrated *in vitro* activity against *Scedosporium* species, although the clinical relevance of the minimum effective concentration (MEC) is unknown. The *in vitro* potency of all agents was reduced against *L. prolificans.* However, greater variability was observed when all isolates were included in the analysis versus those that our laboratory identified, especially for *L. prolificans*, which is considered to be pan-resistant to clinically available agents. This suggests possible misidentification of species, which may be due to reliance on phenotypic characteristics.

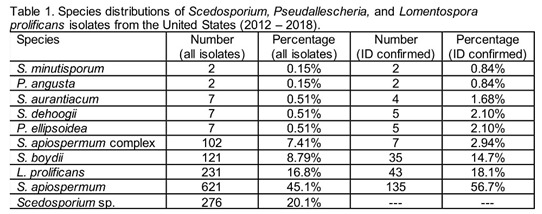

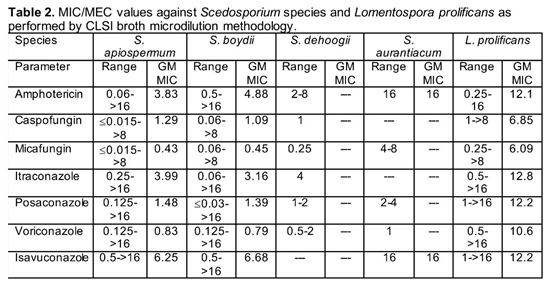





**Conclusion:** *Scedosporium* speices and *Lomentospora prolificans* are not uncommon fungi cultured from patients in the United States. The species distribution we observed is similar to those reported in previous surveillance studies from other countries. Although voriconazole had the most potent *in vitro* activity, variability was observed among the different species, with reduced activity observed for all antifungals against *L. prolificans*.

### P268. Molecular typing of isolates of Microsporum canis collected in Brasil

NevesJ.^1^AlvarezJ.^2^MolinarA.R.^1^PasquettiM.^1^CoutinhoS.D.A.^2^PeanoA.^1^1Departament of Veterinary Sciences, Sector of Parasitology, University of Turin, Grugliasco, Italy2Post-graduation Program In Environmental and Experimental Pathology, Paulista University, São Paulo, Brazil

**Objectives:** *Microsporum canis* is the most common dermatophyte in cats and dogs. *M. canis* is diffused worldwide and plays an important zoonotic role. Methods able to characterize a pathogen at a strain level are expected to provide useful information to gain insight into the dynamics of disease transmission. Among methods proposed for strain typing of dermatophytes, the microsatellite (MS) multilocus typing method is considered one of the most powerful tool. MS are tandem-repeating DNA sequences comprised of 1-6 bp per repeating unit, that are polymorphic in populations due to their propensity for insertion/deletion mutation of multiples of the repeating unit during replication. Multiple loci are generally used, so that a multilocus genotype is obtained (multi-locus microsatellite typing – MLMT). This study was aimed to investigate the intra-specific variability of strains of *M. canis* isolated from different animal hosts and environment sites in Brasil, using a panel of 8 MS.

**Methods:** Fungal strains were collected in different locations in Brasil (mainly in the city of Sao Paulo), from cats, dogs and environment sites. Overall, 73 strains of *Microsporum canis* from 12 outbreak episodes were included in the genetic analyses. DNA was extracted using a commercially available kit (NucleoSpin^®^ Tissue, Macherey-Nagel, Düren, Germany). PCR primers were designed against sequences flanking the MS detected by a BLAST (Basic Local Alignment Search Tool) search using the nucleotide sequence information assembled by the *Microsporum canis* CBS 113480 genome project (http://www.broadinstitute.org/annotation/genome/dermatophyte_comparative/MultiHome.html). Primer sequences for the 8 MS markers recognized as the most polymorphic were custom synthesized (Applied Biosystems UK) with a fluorescent label attached to the 5’ end of each forward primer. Microsatellite fragment analysis was performed using an ABI Prism 310 Genetic Analyzer (Applied Biosystems, FosterCity, CA) for capillary electrophoresis. The ML-MS genotypes for the Brasilian isolates were compared with the genotypes available in the database of the Departament of Veterinary Medicine of Turin, which includes more than 300 strains mainly sampled in Europe.

**Results:** Out of the analyzed strains, 5 different multilocus genotypes were detected. One genotype was more prevalent as it was shared by 11 strains coming from 9 episodes. Thus the genetic diversity was quite low, perhaps due to the fact that a restricted geographic area was sampled. A higher degree of diversity is instead present in the existing database.

**Conclusion:** These data allow enriching the database of MS profiles already available, which regards mainly strains isolated in Europe. Such an enrichment represents a key step to make the database a more and more powerful reference in the study of the dynamics of transmission of *M. canis*. Indeed, it must be pointed out that studies that report the same strain among all isolates from a suspected outbreak, occurring in a geographic region for which no baseline data on the degree of variation in the population exists, remain uninterpretable.

### P269. Genetic diversity of Pneumocystis jirovecii in a tertiary hospital in Spain

BonetL. Goterris^1^MurilloM. Guerrero^1^PasicL.^2^PagarolasA. Anton^1^CompanyJ. Aguilar^3^Ruiz-CampsI.^3^Martin-GomezM.T.^1^1Microbiology, Vall d’Hebron Universitary Hospital, Barcelona, Spain2Molecular Mycology Research Laboratory, Centre for Infectious Diseases and Microbiology, The University of Sydney, Sydney, Australia3Infectious Diseases, Vall d’Hebron Universitary Hospital, Barcelona, Spain

**Objectives:** *Pneumocystis jirovecii* (PJ) is an opportunistic fungus that can be found in the respiratory tract of humans as an asymptomatic colonizer or as a cause of severe pneumonia (PJP) in immunocompromised patients. Establishing associations between genetic types and clusters of cases, clinical manifestation or underlying pathology is not easy as there is no consensus on the better typing scheme. The aim of this study is to apply two multi-locus sequence typing (MLST) protocols to ascertain their ability to discriminate the genetic diversity of colonized and infected immunocompromised patients visited at a tertiary referral hospital in Spain.

**Methods:** Patients and samples: From April 2014 to April 2018, consecutive respiratory tract samples with a positive result for *P. jirovecii* by PCR were included. Only 1 sample per patient and respiratory episode was studied. Laboratory procedures: Two different MLST protocols were carried out. Protocol A (http://mlst.mycologylab.org/pjirovecii) targeted the B-tubulin, dihydropteroate synthase (DHPS) and mitochondrial 26S rRNA (mt26S) loci. Protocol B is a newly developed scheme targeting the cytochrome B (cyt), mt26s and superoxide dismutase (SOD) loci (Pasic et al. Establishment and optimization of a combined MLST scheme for *Pneumocystis jirovecii*. 21th ISHAM Congress 2018, P-223). Phylogenetic analyses: The obtained sequences were depurated and collapsed before achieving phylogenetic analyses. The resulting trees were constructed computing genetic distances among patients for each protocol. Patients were categorized into PJP (green) or PJ colonized (red) groups.

**Results:** Samples from 35 pneumonia cases and 51 colonizations were included. Successful amplification rate was non-significantly different for protocol A (total 58.1%; 26/35[LG1] PJP and 24/51 colonizations) and B (total 72.1%; 26/35 PJP and 34/51 colonizations). As shown in Figure 1, Protocol A allowed the identification of 11sequence-types (ST) whereas protocol B was able to find 21 different ST. Both protocols agreed in finding no apparent clustering of cases in time or space. However, whereas Protocol A grouped 73.1 %[LG2] of pneumonia cases into a group of 7 closely related ST and 61% of colonization cases into the other 4 ST, no apparent correlation between illness and ST could be seen with protocol B. 

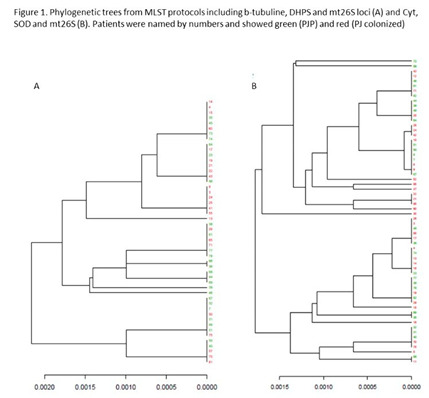



**Conclusion:** In the present study, two MLST protocols were carried out to describe PJ genetic diversity in our cohort. The two protocols provided complementary information: greater genetic diversity was documented with protocol B, whereas protocol A helped us to find sequence-types mostly associated with PJP or colonization. This study allowed us to demonstrate the absence of PJ outbreaks in our hospital during the study period.

### P270. rDisability and disease after hazardous indoor exposure to airborne Stachybotrys spores: exposure variable analysis shows significant correlation with injury to mucosa, phagocytes and neurons.

GrantI.H.^1^GellerJ.^2^SabathH.^3^1Community Medicine, New York Medical College, Tarrytown, United States of America2Information Revelations, Montclair NJ, United States of America3International Environmental Diagnostics Inc, Irvington NYw, United States of America

**Objectives:** While *Stachybotrys* is a mold that flourishes indoors with chronic water intrusion, its spores usually require physical disturbance of its colony growth or prolonged wetness to become airborne. The macrocyclic Trichothecenes mycotoxins in its spores are extremely toxic nanoparticulates dangerous for all mammals, damaging intracellular protein/DNA/RNA production and mitochondrial function causing premature cell death on contact: apoptosis. As exotoxins, they remain toxic despite disinfection, bind to dust causing damage to mammalian skin, mucosa, phagocytes, neurons, and organs via systemic circulation, especially with inhalation. Objectives: To determine: - The frequency of severe illness due to indoor airborne Stachybotrys spore exposure. - Specific exposure risks (fungi, hazardous exposure activities) - The impact of genetically-impaired intracellular MTHFR detoxification capacity. - Conditions that predispose to opportunistic fungal infection with such exposures (e.g. mucosal injury, immunosuppression, malnutrition)

**Methods:** Prospectively monitored 14 (7M, 7F) adults and children exposed to 5 documented Macrocyclic Trichothecenes-contaminated indoor-environments with airborne *Stachybotrys* spores with comprehensive environmental-mycological and medical investigations, fungal IGGs, urine mycotoxins (MCTs: Aflatoxin, Gliotoxin, Ochratoxin A, MT), immune parameters (neutrophils, lymphocyte subsets, monocytes, IGG, IGA, IGM, IGE, subclasses), immunosuppressants, nutritional deficiencies (protein, Vitamin D, zinc), genetic Methylenetetrahydrofolate reductase (MTHFR) single-nucleotide-polymorphisms C677T&A1298C. Disease Severity Outcomes (DS), Environmental Mold-Contamination Severity (EMCS), Hazardous Exposure Activities (HEA), Exposure Duration (ED), MCT/Trichothecenes exposure (MTC/TE), and clinical findings were stratified and analyzed. Biostatistical analysis (Pearson Correlation Coefficients) correlated DS with EMCS, HEA, ED, MCT/TE, mucosal Injury (MI), Neurological disease, Cell-mediated Immunodeficiencies (CMIs), combined cellular-immunoglobulin immunodeficiencies (CCIs), mucosal injury (MI), mold-specific IGG response(M-IGG) and genetic MTHFR deficiencies.

**Results:**

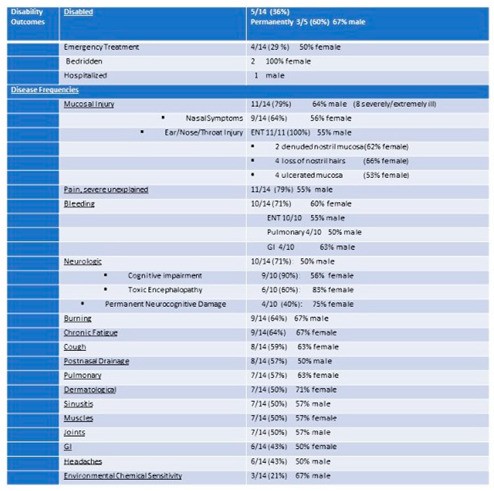

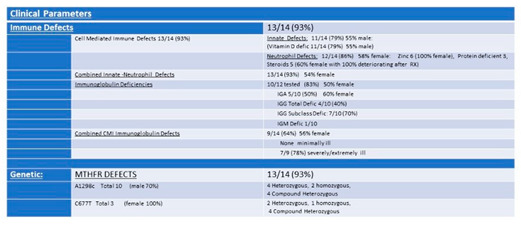





**CORRELATIONS:** Disease Severity based on formal disability classification or physical injury strongly correlated with Hazardous Activities (p = 0.01819), Immune deficiencies (p = 0.046229). Environmental Exposure Exposure Hazards (especially unprotected mold disturbance, uncontained construction/repairs, basement living, HVAC contamination and sewage overflow) correlated with immunodeficiencies (p = 0.046229). Environmental contamination with multiple mycotoxins correlated with multiple mycotoxin urinary excretion (p = 0.026354). Duration of exposure correlated with urinary excretion of multiple mycotoxins (p = 0.006314) as well as Trichothecenes p = 0.00875). Urine Trichothecenes correlated with immunoglobulin deficiencies (p = 0.037285).

**Conclusion:** The high frequency of immune deficiencies (93%), mucosal injury and pain (79%), bleeding, and neurological damage (71%), burning and chronic fatigue (64%) with expected respiratory/Gi /Dermatologic symptomatology in 50% is consistent with known Trichothecenes effects in animal models and military data. The high frequency of cell-mediated immune deficiency is consistent with invitro and in vivo research on Stachybotrys spores injuring phagocytes, supporting injury from inhalation of Stachybotrys spores. The high 94% MTHFR methylation-defect incidence is peculiarly striking compared to estimated USA 50-60% prevalence. MTHFR is a key intracellular detoxifying enzyme protecting ribosomes/mitochondria RNA/DNA damage from Trichothecene injury via glutathione metabolism. Further investigation is needed to determine if Trichothecenes trigger genetic damage. Urine macrocyclic Trichothecenes detection appears a useful marker for environmental macrocyclic Trichothecenes exposure. Public health education and epidemiological research are urgently needed to investigate correlation between environmental mold/mycotoxin exposure and immunodeficiencies.

### P271. Two different involvement of Aspergillus fumigatus in the same case: endophthalmitis and lumbar spondylodiscitis

DoganG.^1^TungerO.^1^KocogluF.^1^TemizC.^2^TemizP.^3^SenolS.^1^CetinC.B.^1^1Infectious Diseases and Clinical Microbiology, Manisa Celal Bayar University Faculty of Medicine, Manisa, Turkey2Neurosurgery, Manisa Celal Bayar University Faculty of Medicine, Manisa, Turkey3Pathology, Manisa Celal Bayar University Faculty of Medicine, Manisa, Turkey

**Case Report:**

*Aspergillus* species are mold type fungi that commonly found in nature and especially for immunocompromised people can cause opportunistic infections. *Aspergillus*-induced endogenous endophthalmitis is a very rare ophthalmic emergency that can result in visual loss. Bone infections caused by *Aspergillus* species are also rare clinical manifestation especially occurs in immunocompromised patients. In this case report, two rare clinical conditions such as endogenous endophthalmitis and lumbar spondylodiscitis due to Aspergillus are reviewed.

A 39-year-old woman with no known underlying disease was admitted to the Ophthalmology Departmant with loss of vision in her left eye. Approximately two months ago, there was a history of antibiotic use (moxifloxacin) for pneumonia. No additional physical examination findings were detected in the patient. Laboratory tests revealed erythrocyte sedimentation rate 59 mm / h, leukocyte count 9.81 10^3^ / /L, hematocrit 34.2%, platelet count 552 10^3^ / /L, neutrophil count 7.53 10^3^ / /L, C-reactive protein 1 mg / dl. Vasculitis was detected in ocular angiography and corticosteroid therapy (prednisolone 32 mg) was started by consultation of Rheumatology Department. Lumbar puncture was performed after normal result of cranial MRI that performed for differential diagnosis of vasculitis, and CSF examination was normal. The patient had undergone vitrectomy because the complaint did not regress despite steroid treatment. Direct examination of the vitreous specimen revealed gram positive bacteria and fungi, and intravitreal Liposomal AMB treatment with intravenous liposomal amphotericin-B (LAMB) and moxifloxacin was initiated. *Aspergillus fumigatus* was grown in vitreous samples. Bronchoscopy was performed and there was no cultural growth in BAL samples. Parenteral antimicrobial treatment was completed and was discharged after 14 days. The patient who had low back pain and morning stiffness was hospitalized in Rheumatology with the preliminary diagnosis of ankylosing spondylitis and received 250 mg intravenous methylprednisolone treatment for 3 consecutive days. Due to the absence of sacroileitis in MR imaging and monitoring of L4-L5 spondylodiscitis, piperacillin tazobactam and daptomycin were started empirically. The patient was than hospitalized in Infectious Diseases. In the pathological examination of the lumbar spine, fungal hyphae were seen and LAMB (3 mg / kg) was added to the treatment because of the positive galactomannan antigen (GMA). The patient whose complaints had regressed and whose GMA was negative was discharged with oral voriconazole after 45 days use of LAMB and 7 days use of parenteral voriconazole. The patient underwent oral voriconazole therapy for 160 days during outpatient follow-up, and regression was detected in control magnetic resonance imaging. CONCLUSION: In immunosuppressive and immunocompetent patients, fungal agents should be considered in case of treatment failure, and hidden foci such as endocardium, eye and vertebrae must be investigated because of hematogenous transmission.

### P272. Epidemiological trend, spectrum, clinical profile and outcome of fungal keratitis from Delhi, India

CapoorM.^1^KoccharS.^2^GuptaV.^2^1Microbiology, Vardhman Mahavir Medical College and Safdarjung Hospital, Delhi, India2Opthalmology, Vardhman Mahavir Medical College and Safdarjung Hospital, Delhi, India

**Objectives:** Fungal aetiology of keratitis ulcer is considered to be one of the leading causes of ocular morbidity, particularly in developing countries including India. More importantly, Fusarium and Aspergillus spp. are reported commonly implicating corneal ulcer and against this background the present work was undertaken so as to understand the current epidemiological trend, clinical profile and outcome of fungal keratitis.

**Methods:** During the study period (2013-2016), the clinical and mycological characteristics of fungal keratitis was investigated. Our aim was to discuss the predisposing factors, clinical manifestations, and epidemiological characteristics of the causative agents and describe the management strategies that have a high probability of successfully treating this disease. The corneal scrapings were processed by direct microscopic methods and standard culture techniques. All the samples were inoculated onto Sabouraud dextrose agar (SDA), potato dextrose agar (PDA), brain- heart infusion based blood agar in the form of a “C” streak. The SDA and PDA plates were incubated at 30°C for 7 days, and the other plates were incubated at 37°C for 7 days.

**Results:** A Total of 143 corneal scrapings from keratitis were processed, 72 (50.3%) revealed growth, of which 60 were culture positive for fungi and 12 were culture positive for bacteria. Direct microscopy revealed hyphae or budding yeasts or fungal elements in 60 cases. Among fungal aetiologies, *Aspergillus spp.* (51.7%) and *Fusarium spp.* (16.7%) were predominantly determined. The spectrum of mycotic keratitis cases was: *Aspergillus flavus (15), A.fumigatus (12), Fusarium solani (5), Fusarium oxysporum (3), Curvularia lunata (3), C. tropicalis (3), C. parapsilosis (2), Fusarium dimerum (2), Paecilomyces lilanicus (2), Alternaria alternata (2), A.terreus (2), A. tetrazonus (1), A.niger (1), Bipolaris (1), Candida krusei (1), Schizophilum commune (1), Scedosporium apiospermum (1), Chryseosporium keratinophillum (1), C. tropicalis (1)* and *Scytalidium dimidiatum (1).* of the 60 mycotic keratitis male preponderance was seen in 66.66%. Predisposing factors included trauma with vegetative material (98.66%), contact lens use (1.66%), and bird feather hit (1.66%). Pain, redness of eye and dimunition of vision was most common clinical complaints. Steroids and inflammatory drugs were most common pretreatment medication taken. The corneal examination revealed central corneal ulcer with feathery margins, a paracentral superficial ulcer; a central ulcer with an irregular edge, satellite infiltrations, and hypopyon etc. Majority of the patients were given natamycin eye drop alone. Concomitant systemic antifungals and supportive surgical intervention was done in 4 and three cases respectively. Complete loss of vision was seen in one eye was seen in single case.

**Conclusion:** Our study suggests that mycotic keratitis caused by Aspergillus spp. especially *A. flavus* may occur more often in a tropical climate, such as in Delhi, India. Aetiological agents other than Aspergillus species and Fusarium species have shown an upward trend. A combination of antifungal therapy and supportive surgical intervention may successfully resolve the mycotic infection Proper understanding of microbiological and clinical characteristics of keratomycosis will enable ophthalmologist to avoid unnecessary and indiscriminate use of steroids or antimicrobials.Early stage of diagnosis and formulation of an uncompromising management protocol can prevent profound visual morbidity.

### P273. Molecular epidemiological investigation of zoonotic outbreaks caused by Sporothrix species

CarvalhoJ.TerraP.PinheiroB.CamargoZ.P.D.RodriguesA.M.Department of Microbiology, Immunology and Parasitology, Federal University of São Paulo, São Paulo, Brazil

**Objectives:** Sporotrichosis is a mycosis mostly caused by a group of thermodimorphic fungi embedded in a clinical clade (i.e., *Sporothrix brasiliensis*, *S. schenckii*, *S. globosa*, and *S. luriei*). The infection is caused after a traumatic inoculation of *Sporothrix* propagules into the (sub)cutaneous tissue of mammals. *Sporothrix* species have been reported around the world, mostly in regions with humid (sub)tropical and temperate climates. During the last decades, vast zoonoses have been ongoing in Brazil with Rio de Janeiro as the epicenter of this cat-transmitted epidemic. In this scenario, the development of new molecular markers is mandatory to explore the genetic diversity and track *Sporothrix* expansion during outbreaks. The purpose of this study was to analyze the genetic diversity of *Sporothrix* originating from different geographic regions of Brazil, using AFLP (Amplified fragment length polymorphism) markers.

**Methods:** *Sporothrix* spp. genome sequences were retrieved from Genbank (https://www.ncbi.nlm.nih.gov/genbank/) and were *in silico*-digested with *Eco*RI and *Mse*I using the software AFLPinSilico. Only fragments of 50-500 bp were considered. A total of 256 combinations of selective pairs of primers (based on 2 selective nucleotides) were employed to generate 2.304 fingerprints profiles with different fragment numbers and sizes (0 to 56 fragments/per combination).

**Results:** *In silico* analysis highlighted 6 combinations to be tested in a subset of 15 medically relevant *Sporothrix* isolates. *In vitro*, AFLP presented strict concordance with *in silico* results considering the number and size of the amplicons resolved during capillary electrophoresis. A total of 58 AFLP markers were unambiguously scored in the electropherograms using the software BioNumerics v.7.6. Dendrograms were constructed (UPGMA, Jaccard Distance) based on a banding match table and isolates were correctly clustered according to the taxon name. Intraspecific variation was observed for all agents, including *S. brasiliensis*, a species previously known to be clonal. Based on the data above we recommend the use of 3 different AFLP combinations to explore genetic diversity and taxonomy in *Sporothrix*.

**Conclusion:** Independent experiments with similar results showed that the AFLP is highly reproducible, making this technique interesting to evaluate the genetic diversity and the spreading of *Sporothrix*. The present content in this study will enable the development of strategies to control the disease and will contribute to further studies about diversity in *Sporothrix*.

### P274. Epidemiological and molecular investigation of emerging Histoplasma species in Brazil

RodriguesA.M.^1^BealeM.^2^HagenF.^3^FisherM.^4^TerraP.^1^HoogG.S. De^5^BrilhanteS.^6^CordeiroR.^6^Castelo-BrancoD.^6^RochaM.^7^SidrimJ.^7^CamargoZ.P.D.^1^1Department of Microbiology, Immunology and Parasitology, Federal University of São Paulo, São Paulo, Brazil2Parasites and Microbes Programme, Wellcome Sanger Institute, Cambridge, United Kingdom3Westerdijk Fungal Biodiversity Institute, Utrecht, Netherlands4Department of Infectious Disease Epidemiology, Imperial College London, London, United Kingdom5Westerdijk Institute, Utrecht, Netherlands6Postgraduate Program In Medical Microbiology, Federal University of Ceará, Fortaleza, Brazil7Postgraduate Program In Veterinary Science, Federal University of Ceará, Fortaleza, Brazil

**Objectives:** Histoplasmosis is a serious infectious disease in humans caused by *Histoplasma* spp. (Onygenales), whose natural reservoirs are thought to be soil enriched with bird and bat guano. The true global burden of histoplasmosis is underestimated and frequently the pulmonary manifestations are misdiagnosed as tuberculosis. Molecular data on the epidemiology of *Histoplasma* are still scarce, even though there is increasing recognition of histoplasmosis in recent years in areas distant from the traditional endemic regions in the Americas.

**Methods:** We used multi-locus sequence data from protein-coding loci (ADP-ribosylation factor, H antigen precursor, and delta-9 fatty acid desaturase), DNA barcoding (ITS1/2+5.8s), AFLP markers and mating type analysis to determine the genetic diversity, population structure and recognize the existence of different phylogenetic species among 436 isolates of *Histoplasma* obtained globally.

**Results:** Our study describes new phylogenetic species and the molecular characteristics of *Histoplasma* lineages causing outbreaks with a high number of severe outcomes in Northeast Brazil between 2011 and 2015. Genetic diversity levels provide evidence for recombination, common ancestry and clustering of Brazilian isolates at different geographic scales with the emergence of LAm C, a new genotype assigned to a separate population cluster in Northeast Brazil that exhibited low diversity indicative of isolation. The global survey revealed that the high genetic variability among Brazilian isolates along with the presence of divergent cryptic species and/or genotypes may support the hypothesis of Brazil being the center of dispersion of *Histoplasma* in South America, possibly with the contribution of migratory hosts such as birds and bats. Outside Brazil, the predominant species depends on the region. We confirm that histoplasmosis has significantly broadened its area of occurrence, an important feature of emerging pathogens.

**Conclusion:** From a practical point of view, our data point to the emergence of histoplasmosis caused by a plethora of genotypes and will allow the proposal of public policies to contain the spread of histoplasmosis. Furthermore, with the description of such diversity, we open avenues for future comparative genomic studies, which will allow progress toward a consensus taxonomy, improve understanding of the presence of hybrids in natural populations of medically relevant fungi, test reproductive barriers and explore the significance of these variations.

### P275. Tuberculosis and Fungal Co-infection of Fungal Infections, University Hospital Center Zagreb, Croatia, During Past Two Years

JandrlićM.^1^SamarzijaM.^2^VranješV. Rezo^1^PleškoS.^3^MarekovićI.^3^JandrlicA.^4^1Department of Clinical and Molecular Microbiology, University Hospital Centre Zagreb, Zagreb, Croatia2Clinic For Lung Diseases, University Hospital Centre Zagreb, Zagreb, Croatia3Department of Clinical and Molecular Microbiology, University Hopital Centre Zagreb, Zagreb, Croatia4Laboratory Biomedicine, Faculty of Pharmacy, Ljubljana, Slovenia, Zagreb, Slovenia

**Objectives:** Pulmonary tuberculosis is one of the most important health concerns. Pulmonary fungal infections have clinical and radiological characteristics similar to tuberculosis which may be easily misdiagnosed as tuberculosis. This study aimed to evaluate *Mycobacterium tuberculosis* (MTB) and other non-tuberculous mycobacteria (NTM) with coinfection of fungal infections in patients, during last 2 years.

**Methods:** Microbiological and clinical reports of 149 patients with confirmed mycobacteria have been analyzed. Detection and identification of mycobacteria were performed by the standard culture method using the BACTEC MGIT 960 system (BD) and Lowenstein–Jensen medium. Identification of positive *Mycobacterium tuberculosis* complex (MTBC) was based on positive acid-fast bacilli microscopic smear (Ziehl–Neelsen and fluorochrome), positive niacin accumulation, MALDI-TOF and *Mycobacteria* PCR. TB isolates were also tested for their sensitivity toward first-line anti-MTB drugs.

**Results:** Between April 2017 and April 2019, at 149 patients were detected Mycobacterial infections (MTB 64%, NMT 36%). Mycological tests are not required in 36 24% patients. Mycological tests are required in 113 76% patients. The majority of patients had isolated several types of fungi, 160 episodes. The patients 113 were isolated yeasts, opportunistic and saprophytic molds (54%, 16%, 28%). Frequent Candida were C. albicans, *C. dubliniensis*, *C. parapsilosis* and *C. glabrata* respectively. Frequent *Aspergillus* were *A. niger*, *A. fumigatus* and *A. flavus* respectively. In addition to *Aspergillus*, small number of patient had *Fusarium*, *Rhizopus* and *Pseudallescheria boydii*. Saprophytic molds were confirmed in 28% of patients.

**Conclusion:** Our findings showed the co-infection of fungi agents in patients with tuberculosis that should be considered. Clinical data must be analyzed to be able to distinguish infections, colonization, or part of normal flora.

### P276. Prevalence of Aspergillus infection in delay resolution of community-acquired pneumonia in high-risk population in Taiwan

HuangS.F.WangF.-D.Internal Medicine, Taipei Veterans General Hospital, Taipei, Taiwan

**Objectives:** Pulmonary Aspergillosis is a world-widely distributed disease. The prevalence of *Aspergillus* infection in patients with delay resolution community acquired pneumonia (DR-CAP) or lower respiratory tract infection (LRTI) had not been investigated in Taiwan. The aims of the study was prospectively evaluate the prevalence of *Aspergillus* infection by serological testing and microbiological evidence in high risk patients presented with DR-CAP or LRTI.

**Methods:** Patients presented with LRTI or DR-CAP were prospectively enrolled. Patients with underling chronic pulmonary disease (CLD), active tuberculosis (TB), collagen fiber disorders were study groups, and others who were healthy without underling disorders except DR-CAP or LRTI. Delay resolution of community acquired pneumonia (DR-CAP) was defined as clinical diagnosis with community acquired pneumonia not significantly getting resolution after 3-4 weeks of appropriate anti-bacterial antibiotics. Serum gamma-immunoglobulin (IgG) concentration for *Aspergillus niger (A. niger)* and *Aspergillus fumigatus (A. fumigatus)* were measured by ELISA method. Positive results were recorded by the cut-off-value provided by the company (ImmunoCAP^@^, ThermoFisher Scientic Inc.). Microbiological evidence for mold infection was record according to the results of fungus culture from sputum, bronchioalveloar lavage fluid (BALF), or nasal/throat swab. Patients were excluded for pulmonary malignancy by diagnostic approach such as bronchoscopic and transthoracic lung biopsy.

**Results:** From 2018 April to 2019 Jan, a total of 99 patients were enrolled. Overall, 38.3%, 25.1%, and 11.1% of patients were with CLD, collagen fiber disorders, and active TB or old TB with complete anti-TB therapy (ATT), respectively. There were 19% of patients were healthy but presented with LRTI not response to anti-bacterial antibiotics. Overall, seroprevalence for *A. niger* and *A. fumigatus* were 32.3% and 22.9%, and the patients with CLD had a highest seroprevalence with 35.4% and 36.6%, respectively. In combination of microbiological and serological results, the prevalence for mold infection was 59%, and CLD remained a highest-risk disorder (48.7%). Overall, concordance between microbiological results for fungus culture and positive serological results for *A. niger* and *A. fumigatus* were 69.6% and 60%, respectively.

**Conclusion:** Both the serological and microbiological evidence for mold or *Aspergillus* infection in high-risk patients with DR-CAP or LRTI were high in Taiwan. We also firstly reporeted the seroprevalence for *Aspergillus spp.* around 10% in patients without underling high-risk disorders, that indicate the populational seroprevalence in north Taiwan.

### P280. rCo-infection Candida glabrata with other Candida species in nosocomial candiduria

AghiliS.R.^1^AbastabarM.^2^SoleimaniA.^3^VostaA. Akbari^3^AziziS.^4^RayatniaM.^5^SalmanianB.^6^1Invasive Fungi Research Center/department of Medical Mycology, Mazandaran University of Medical Sciences, Sari, Iran2Department of Medical Mycology, Invasive Fungi Research Center, School of Medicine, Mazandaran University of Medical Sciences, Sari, Iran, Sari, Iran3Student Research Committee, Mazandaran University of Medical Sciences, Sari, Iran4Department of Laboratory Medicine, Faculty of Allied Medical Sciences, Mazandaran University of medical science, Sari, Iran5Kidney Center of Imam Reza Hospital, Mazandaran University of Medical Sciences, Sari, Iran6Department of Scienes, Farhangian University, Seddighe tahereh branch, Sari, Iran

**Objectives:** *Candida glabrata* was formerly classified in the *Torulopsis* genera, but it is currently considered to belong to the *Candida* genus. Evidence showed that it is the second causative agent of nosocomial candidiasis, alone or coexisting with other yeasts, particularly other *Candida* species. The reason for this association is often unknown and some have suggested a synergistic pathogenesis. Due to the increasing resistance of *C. glabrata* to many common antifungal drugs, treatment in patients is also accompanied by some problems. Therefore, identification of co-infection in patients and the modification of therapeutic protocols is essential.

**Methods:** This descriptive laboratory-based surveillance study was investigated the incidence of nosocomial candiduria and it determined their etiologic agents in 555 hospitalized patients in two hospitals (253 hemodialysis patients in Hospital hemodialysis center of Amol city and 305 hospitalized patients with heart failure in Heart center of Sari city), Mazandaran province, Iran. The patients’ urine samples were cultured in mycological culture media and colony counts >10^4^ CFU/ml were considered significant for candiduria. Species identification was done based on color of colony on CHROMagar Candida and PCR-RFLP method, by ITS region and the MspI restriction enzyme and PCR amplification of *HWP1* gene.

**Results:** We found 40(15.8%) positive candiduria in hemodialysis patients and 58(18.8%) in heart failure patients. *C. glabrata*(47.1%), *C. albicans*(17.6%), *C. tropicalis*(15.7%), *C. parapsilosis*(15.7%) and *C. kefyr*(0.4%) were identified as candiduria agents in hemodialysis patients and *C. glabrata*(40.3%), C. albicans(40.3%), *C. tropicalis*(7.5%), *C. parapsilosis*(7.5%) and *C. krusei*(4.4%) were candiduria agents in heart failure patients. *C. glabrata*, alone as a single-species candiduria agent was detected in many patients. However, in these two patient groups, it has associated with other *Candida* species as mix-infection. Nine hemodialysis patients had mix-infection by *C. glabrata* associated with other *Candida* species (7 cases *C. glabrata* + *C. albicans*, 2 cases *C. glabrata* + *C. albicans* + *C. kefyr*) and 9 heart failure patients had mix-infection by *C. glabrata* associated with one other species too (4 cases *C. glabrata* + *C. albicans*, 2 cases *C. glabrata* + *C. tropicalis*, 2 cases *C. glabrata* + *C. parapsilosis* and 1 case *C. glabrata* + *C. krusei*).

**Conclusion:** Studies indicate increasing azole antifungal prophylaxis treatment and invasive application lead to an increase in low susceptibility to azoles *non albicans* species, and *C. glabrata* became second causative nosocomial infection agent now. The data suggest that the growth of other *Candida* species was stimulated by the presence of *C. glabrata* and this species in the biofilms more active than other species that produce biofilms with more biomass. Although, *C. glabrata* is generally considered a species of low virulence, mortality rate of patients with candidiasis caused by this species is higher than *C. albicans* because of complicates the treatment. Given the prevalence of *C. glabrata* and other *candida* species co-infection, it is necessary to understand the mechanisms used by *C. glabrata* in collaboration with them and the use of rapid identification methods for candidiasis agents can be a great help to treat these patients, more effectively.

### P281. An Open-Label Multicenter Observational Study of Anidulafungin For The Treatment of Invasive Candidiasis In Russia

KozlovaO.^1^RubinchikV.^2^SobolM.^3^LarionovaV.^4^TyrenkoV.^5^ZhuravelS.^6^PetrovaE.^7^TruhinaT.^8^KlimkoN.^1^1Department of Clinical Mycology, Allergology and Immunology, North-Western State Medical University n.a. I.I. Mechnikov, Saint-Petersburg, Russian Federation2V.A. Almazova NMI, St. Petersburg, Saint-Petersburg, Russian Federation3Irkutsk Regional Cancer Center, Irkutsk, Irkutsk, Russian Federation4n «N.N. Blokhin National Medical Research Center of Oncology» of the Ministry of Health of the Russian Federation, MoscowFederal State Budgetary Institutio, Moscow, Russian Federation5S.M. Kirov Military Medical Academy, St. Petersburg, Saint-Petersburg, Russian Federation6N.V. Sklifosovsky Research Institute for Emergency Medicine, Moscow, Moscow, Russian Federation7State Budgetary Healthcare Institution Infectious Diseases Hospital № 2 Moscow Health Department, Moscow, Moscow, Russian Federation8Tula Regional Oncology Center, Tula, Tula, Russian Federation

**Objectives:** Publications about “real life” using of anidulafungin are limited. Study of anidulafungin for the treatment of invasive candidiasis (IC) in actual clinical practice in Russia.

**Methods:** The primary endpoints were global response (clinical + microbiological response) at end of treatment (EOT), and the 30 days overall survival rate. For diagnosis of invasive candidiasis (IC) we used criteria EORTS/MSG, 2008.

**Results:** In 2015-2017 yy. in a prospective multicenter (*n* = 23) study included 300 adult patients who were treated with anidulafungin. Men were 57%, median age – 49 y (from 18 to 98). All examined patients had risk factors for IC. In 85% patients, a fever resistant to the use of broad-spectrum antibacterial drugs for ≥ 4 days was noted. Median APACHE II at the start of anidulafungin therapy was 18.8. Anidulafungin was used for the empirical therapy of IC in 82% patients. Global response at EOT was 79%, the 30 days overall survival rate was 78%. Anidulafungin was generally well tolerated with few treatment-related adverse events (hypokalemia – 1%, angioedema – 0.5%), which were not the cause of drug withdrawal. IC was laboratory confirmed in 25% patients. *Candida* non-*albicans* (57%) prevailed among IC pathogens. In patients with proven IC the 30 days overall survival rate was 66%, which is significantly higher than in the earlier multicenter study in Russia (43%).

**Conclusion:** The overall efficacy of anidulafungin was high (79%), the 30 days overall survival rate was 78%. Adverse events occurred in 1,5% cases and were not the cause of drug withdrawal. In 82% patients anidulafungin was used for empiric therapy. In patients with proven invasive candidiasis the 30 days overall survival rate was 66%.

### P282. Two cases of Pseudomembranous Tracheobronchitis due to Amphotericin B-resistant Aspergillus flavus

HareD.^1^GriffinA.^1^AkashehN.^2^BaconC.L.^3^Martin-LoechesI.^4^RogersT. R^1^,^5^ConnellB. O^1^1Department of Clinical Microbiology, St James’s Hospital, Dublin, Ireland2Department of Medicine, St. James’s Hospital, Dublin, Ireland3Department of Haematology, St. James’s Hospital, Dublin, Ireland4Department of Intensive Care Medicine, St. James’s Hospital, Dublin, Ireland5Trinity College Dublin, Dublin, Ireland

**Case Report:** Introduction: Pseudomembranous Aspergillus Tracheobronchitis (PMAT) is an uncommon manifestation of invasive pulmonary aspergillosis (IPA). Patients are typically immunocompromised although sporadic cases have been reported in previously immunocompetent individuals.^1^*Aspergillus flavus* is the second most frequent *Aspergillus* species causing bronchopulmonary disease and isolates typically display MICs to amphotericin B (amB) which are two-fold dilution steps higher than *A.fumigatus.*^2^ Although the clinical significance of *in vitro* antifungal susceptibility is debated, there is evidence to suggest that *A. flavus* isolates demonstrating resistance to amB are associated with increased mortality.^3^ We present two cases of *A. flavus* PMAT with raised MICs to amB and contrasting host risk-factors. Methods: Susceptibility testing was performed using E-test on RPMI agar and confirmed at a reference laboratory using broth-microdilution in accordance with CLSI methodology. As no species-specific breakpoints have been established for *A. flavus*, resistance was defined as ≥2mg/L based on expected MIC ranges.^3^ Results: Patient 1: A 39-year-old male was admitted for an Allogeneic Haematopoetic Stem Cell Transplant for Acute Lymphoblastic Leukaemia. Post-transplant, his medications included prophylactic liposomal amB. On day 4, he became febrile with an undetectable neutrophil count. Therapeutic doses of liposomal amB were subsequently added due to persistent fevers. Chest CT showed bronchial narrowing suggestive of mucous plugging and bronchoscopy revealed pseudomembranous tracheobronchitis. Bronchoalveolar-lavage (BAL) cultured *A. flavus* with an MIC to amB of 4.0mg/L. His antifungal regimen was changed to IV voriconazole and caspofungin. He required admission to ICU due to respiratory failure but died at 31 days post-transplant. Patient 2: A 51-year-old previously immunocompetent male presented to hospital with fevers and progressive shortness of breath. Chest X-ray showed patchy infiltrates and he was commenced IV co-amoxiclav, clarithromycin and oseltamivir. By day 2, he required admission to ICU due to respiratory failure. A nasopharyngeal aspirate PCR returned positive for Influenza A RNA and IV hydrocortisone was added as an adjunct to his antiviral therapy. Bronchoscopy showed a pseudomembranous tracheobronchitis and brushings cultured *A.flavus* with an MIC of 2.0mg/L to amB. He received dual antifungal therapy with IV voriconazole and amphotericin B but died at 31 days post admission. Conclusion: These cases contrast a neutropenic patient with classical risk-factors for IPA with a patient who was not immunocompromised but was diagnosed with PMAT following Influenza infection. Diagnosis may be delayed by the absence of clearly distinguishable symptoms, laboratory parameters or imaging findings, and cases may be refractory to antifungal therapy. Our experience is consistent with evidence demonstrating poor outcomes in patients with *A.flavus* infections with *in-vitro* resistance to amB, and underline the importance of performing susceptibility testing to guide antifungal therapy. References: 1. Khalid S *et al*. Pseudomembranous aspergillar tracheobronchitis in a non-neutropenic critically ill patient in the intensive care unit. J Community Hosp Intern Med Perspect. March 2017p.43-45 2. Van Der Linden, J. W.M *et al*. Aspergillus species intrinsically resistant to antifungal agents. Medical Mycology, Vol.49, Issue Supplement_1, April 2011,p.S82–S89 3. Inès Hadrich *et al.* Amphotericin B in vitro resistance is associated with fatal Aspergillus flavus infection, Medical Mycology, Vol.50, Issue 8, November 2012, p829–834

### P283. Influenza-associated invasive pulmonary aspergillosis in a 25-bed Greek ICU during 2018-2019 influenza season.

DoukaE.^1^PerlikosF.^1^MalachiasS.^1^GavrielatouE.^1^SarriK.^1^KougiasM.^1^AdamosG.^1^KostourouS.^2^ZakinthinosS.^1^1Icu, Evangelismos General Hospital, Athens, Greece2Infection Control, Evangelismos General Hospital, Athens, Greece

**Objectives:** Invasive pulmonary aspergillosis (IPA) is an opportunistic fungal infection that often affects severely immunocompromised hosts. It has been recently documented as a co-infection in critically ill patients with severe viral influenza infection, although the exact risk rate is less well determined. To study the prevalence of IPA in critically ill patients with severe influenza in Evangelismos General Hospital, Greece.

**Methods:** We retrospectively reviewed all the ICU records for the 2018-2019 influenza season. Influenza was diagnosed using the polymerase chain reaction. Co-infection had to be confirmed using standard bacteriological tests. 28-day ICU and hospital mortality was calculated.

**Results:** of the 69 patients diagnosed with influenza (34 H1NH, 31 Influenza A, 1 H1N2, 1 H1N3, 1 H1N4) in our 1000-bed tertiary hospital, 9 patients where admitted to the ICU (5 H1N1, 1 Influenza A, 1 H1N3, 1H1N4). Two patients had a co-infection (2,9% of the hospitalized patients with influenza and 22% of influenza patients admitted to the ICU). 3 ICU patients suffering influenza were severely immunocompromised but none of them developed IPA. Underlying medical conditions included hematology malignancy (3), diabetes mellitus (2), chronic renal failure (2). IPA was confirmed by histopathological findings in one patient and in combination of clinical, microbiological and radiological criteria in both. The galactomannan test in BAL was positive only in the patient with endobronchial bulky disease. Microbiology BAL cultures were *Aspergillus spp* positive both (*A. Fumigatus* and *A. Niger* respectively). Both patients were treated with isavuconazole (6 and 12 weeks respectively) and were completely cured. The 28-days ICU mortality was 22,2% in influenza ICU patients group. 90-day hospital mortality was 44,4%. None of the 2 patients with IPA co-infection has died.

**Conclusion:** IPA co-infection in critically ill patients with severe influenza has been increasingly reported worldwide in recent years. Infection with influenza may increase the risk of invasive aspergillosis by breaking down the bronchial mucosa facilitating Aspergillus invasion, inducing immunomodulatory effects. Aspergillus infection should be considered as a possible cause of persistent or progressing respiratory failure in immunocompetent critically ill patients suffering from influenza. The diagnosis of IPA requires awareness in order to conduct timely and appropriate testing. Microbiology laboratories also need to be aware of the suspicion of IPA. IPA is a rapidly progressive disease with high mortality rates up to 90%. Mortality can be reduced with prompt initiation of effective antifungal therapy. Isavuconazole has shown promising results in our clinical setting, for the primary treatment of IPA, especially in patients with endobronchial bulky disease and chronic renal failure.

### P284. Candida auris bloodstream infection in Egypt

El-KholyM.^1^ShawkyS.^2^FayedA.^3^MeisJ.^4^1Department of Microbiology and Biotechnology, College of Pharmacy, Arab Academy for Science, Technology and Maritime Transport (AASTMT), Alexandria, Egypt2Department of Microbiology, Medical Research Institute, University of Alexandria (MRI), Alexandria, Egypt3Department of Critical Care Medicine., Faculty of Medicine, University of Alexandria., Alexandria, Egypt4Department of Medical Microbiology and Infectious Diseases, Excellence Center For Medical Mycology (ecmm), Canisius Wilhelmina Hospital, Nijmegen, Netherlands

**Case Report:**

**Objectives:** *Candida auris* was first identified and described in 2009 in Japan. Genomic analysis has shown that *C. auris* emerged in at least 3 different continents, including the Middle-East, almost simultaneously. Here we report the first case of *C. auris* infection in Egypt. 

**Methods:** We present the first case of *C. auris* fungemia from a patient in Egypt. 

**Results:** A 53-year-old male was admitted in December 2017 to a hospital in Cairo just after returning travelling from Saudi Arabia. He suffered from vomiting, abdominal pain, bone aches and muscle weakness. Further investigations showed a positive C-ANCA and in a renal biopsy, small vessel vasculitis consistent with Wegeners granulomatosis. He received steroids and endoxane after which he improved temporarily. However, he became more ill and was transferred to ICU where he was intubated and received plasmapheresis. Due to kidney failure he required hemodialysis during his ICU admission. On day 40 of hospitalization he was transferred to a tertiary care facility in Alexandria with cardiovascular, respiratory and renal failure. He appeared septic with leukocytosis and elevated C-reactive protein of 223. Peripheral blood cultures were obtained and broad-spectrum empirical therapy with iv meropenem, colistin and caspofungin was started. Bronchoalveolar cultures showed growth of multidrug resistant *Pseudomonas aeruginosa* (only colistin and amikacin susceptible) and blood cultures showed the growth of yeasts which were identified as *C. auris* with the VITEK2 with updated database (version 8.01) and confirmed with MALDITOF. Despite the early proper empiric treatment with caspofungin the patient passed away on day 4 after admission. Urine cultures were negative for yeasts. After diagnosis, strict contact precautions were installed and no further *C. auris* cultures were identified in our healthcare facility in the past year. CLSI antifungal susceptibility testing showed resistance to fluconazole (64 mg/L), a higher MIC of amphotericin B (1 mg/L) and low MICs of echinocandins (0.063 mg/L). AFLP and MLVA genotyping showed that the isolate clustered with the south-Asian *C. auris* Clade. 

**Conclusion:** We report the first case of *C. auris* (south-Asian clade) bloodstream infection in Egypt. Since the patient resided shortly before in Saudi Arabia it is highly likely that he was colonized while staying abroad and that his fast progressing illness predisposed for this fatal outcome of *C. auris* fungemia. In the year after discovery no secondary cases were detected in our healthcare facility.

### P285. Invasive pulmonary aspergillosis in patients admitted to the intensive care unit with severe influenza pneumonia: An observational study from a developing country

IrfanM.^1^HussainM.^1^JabeenK.^2^AwanS.^3^FarooqiJ.^2^NasirN.^4^HasanZ.^5^1Medicine, Aga Khan University, Karachi, Karachi, Pakistan2Pathology and Laboratory Medicine, Aga Khan University, Karachi, Pakistan3Medicine, Aga Khan University, Karachi, Karachi, Pakistan, Pakistan4Adult Infectious Diseases, Aga Khan University, Karachi-KHI, Pakistan5Department of Pathology and Laboratory Medicine, Aga Khan University, Karachi, Pakistan

**Objectives:** Invasive pulmonary aspergillosis (IPA) in patients with influenza is increasingly being identified in recent years. Cases of influenza-associated IPA with high mortality have been reported from several developed countries with a reported incidence between 19-28 % (1, 2). So far no significant data is available from developing countries. The aim was to determine the incidence and outcome of IPA in patients with severe influenza pneumonia admitted in an intensive care unit (ICU) of a developing country.

**Methods:** This was an observational study on confirmed influenza patients with respiratory failure admitted to the Aga Khan University hospital (AKUH) ICU from November 2018- March 2019. Patients older than 18 years, acute respiratory failure, pulmonary infiltrates on imaging, and a confirmed influenza infection based on a positive influenza PCR were included. The diagnostic criteria used for IPA was based on suggestive clinical signs and symptoms, radiological findings and mycological data (2). Like other studies, we have also not included the host factors as this criterion was largely created for immunosuppressed hosts, and influenza-related aspergillosis may occur in previously normal hosts (2). The clinical characteristics, radiology, laboratory data and outcome were recorded on a predesigned performa.

**Results:** A total of 92 patients with confirmed influenza were admitted at AKUH during study period. of these 16/92 (17.02%) were admitted in ICU due to respiratory failure. Among these 16 patients, IPA was diagnosed in 5 patients, giving an incidence of 31.25%. The mean age of IPA patients was 61.2 (±5.02) years (Range 53-65 years) and 60% were female. Three (60%) patients had non H1N1 Influenza A and 2 (40%) had H1N1 Influenza A infection. Three (60%) patients had underlying diseases (diabetes, hypertension and ischemic heart disease) and one patient was immunosuppressed. Systemic steroids were used after ICU admission in all patients. On admission 3 (60%) patients had acute kidney injury and 2 (40%) had deranged liver enzymes. Invasive positive pressure ventilation (IPPV) was required in 04 (80%) patients and one patient was managed on noninvasive ventilation (NIV). The average duration of IPPV use was 10.8 (range: 5-24) days. All 5 patients had received voriconazole after diagnosis of IPA. The overall mortality rate of influenza patients admitted in ICU was 50% and in patients with IPA was 60%. The causes of death were ARDS and multisystem organ failure.

**Conclusion:** High incidence (31.25%) of IPA was found in influenza patients requiring ICU admission and was associated with high (60%) mortality. High index of suspicion, early diagnosis and appropriate treatment can improve outcome in these patient. Future large multicenter studies are required to assess the risk factors and role of antifungal prophylaxis to improve the outcome of influenza-associated aspergillosis. **References:** Schauwvlieghe A, Rijnders BJA, Philips N, Dutch-Belgian Mycosis study group et al. Invasive aspergillosis in patients admitted to the intensive care unit with severe influenza: a retrospective cohort study. *Lancet Respir Med* 2018. Huang L, Zhang N, Huang X, et al. Invasive pulmonary aspergillosis in patients with influenza infection: A retrospective study and review of the literature. Clin Respir J. 2019 Apr;13(4):202-211.

### P286. Hellenic Invasive Candidiasis Study (HELICAS): Identification and Antifungal Susceptibility of Candida species in Greek ICUs.

VrioniG.^1^TsilakisD.^2^GrammelisV.^2^CheliotisG.^3^BakaV.^4^ChristofidouM.^5^KarahaliosS.^6^KazilaP.^7^MamaliV.^8^MarakiS.^9^MaropoulosG.^10^PanopoulouM.^11^PerivoliotiE.^12^PetinakiE.^13^PlatsoukaE.^14^Themeli-DigalakiK.^8^TsiplakouS.^15^TsorliniE.^16^VagdatliE.^17^VogiatzakisE.^18^VourliS.^19^ZervaL.^19^KarahaliouE.^1^TheodoridouK.^1^TsianosC.^1^SkiadasI.^2^ArmaganidisA.^20^TsakrisA.^1^1Microbiology, Medical School, National and Kapodistrian University of Athens, Athens, Greece2Medical Affairs, Pfizer Hellas, Athens, Greece3Biometrics Program Manager, Health Data Specialists, Athens, Greece4Microbiology, Red Cross General Hospital, Athens, Greece5Microbiology, Rio University Hospital, Patras, Greece6Microbiology, Agioi Anargyroi Oncology Hospital, Athens, Greece7Microbiology, Theageneio Oncology Hospital, Thessaloniki, Greece8Microbiology, Tzaneio General Hospital, Athens, Greece9Microbiology, University Hospital Crete, Herakleio, Greece10Microbiology, Laiko General Hospital, Athens, Greece11Microbiology, University Hospital of Alexandroupolis, Alexandroupolis, Greece12Microbiology, Evangelismos General Hospital, Athens, Greece13Microbiology, Larissa University Hospital, Larissa, Greece14Microbiology, “Konstantopouleio-Patission” General Hospital of Neas Ionias, Athens, Greece15Microbiology, KAT General Hospital, Athens, Greece16Microbiology, “G. Papanikolaou” General Hospital, Thessaloniki, Greece17Microbiology, “Ippokrateio” General Hospital, Thessaloniki, Greece18Microbiology, Sotiria General Hospital, Athens, Greece19Microbiology, Attiko University Hospital, Athens, Greece20Intensive Cate Unit, Attiko University Hospital, Athens, Greece

**Objectives:** HELICAS (HELlenic Invasive CAndidiasis Study) was a prospective non-interventional study aiming to compare the cost of treatment of documented invasive candidiasis (IC) by *C. albicans* and *C.* non-*albicans* species, in 18 Greek intensive care units (ICUs). As part of this study, isolated *Candida* species were collected and analyzed. Additionally, the epidemiology of IC by *C. albicans* and *C.* non-*albicans,* was evaluated by collecting retrospective data from participating ICUs for an at least 3-month period.

**Methods:** A total of 100 adult subjects (49% *C. albicans*, 51% *C.* non*-albicans*) were enrolled according to a pre-specified sample size, for both *C. albicans* and *C.* non-*albicans* cases. Demographics, days of ICU stay, antifungal and concomitant medications, hospital supplies, laboratory investigations and treatment patterns, as well as patient outcomes and survival were documented. Eighty-two of the collected *Candida* isolates (39 *C. albicans*, 43 *C.* non-*albicans*) were sent to the Department of Microbiology, Athens Medical School, for further investigation. *Candida* isolates were identified by conventional, biochemical tests (CHROMagar *Candida* Medium, Becton Dickinson; API 32C, BioMérieux), supplemented with mass spectrometry (Vitek MS, BioMérieux), if the identification score was < 95%. Antifungal susceptibility testing for amphotericin B, 5-flucytocine, itraconazole, fluconazole, voriconazole, posaconazole, micafungin, caspofungin, and anidulafungin was performed with the broth microdilution method (MICRONAUT-AM MHK 2, MERLIN Diagnostika GmbH), according to CLSI 2012 M27-A3 document. Revised CLSI M27-S4 species–specific clinical breakpoints were applied.

**Results:** Eighty-two strains were studied, the majority isolated from candidemia cases; 39 (47.6%) *C. albicans*, 26 (31.7%) *C. parapsilosis*, 9 (11.0%) *C. tropicalis*, 6 (7.3%) *C. glabrata*, 1 (1.2%) *C. kefyr*, and 1 (1.2%) *C. lusitaniae*. All strains showed low MICs to amphotericin B, with MIC values ranging from 0.0625 to1 μg/ml. Sixteen isolates were resistant to fluconazole: 15 *C. parapsilosis* (MIC range 8-32 μg/ml) and 1 *C. albicans* (MIC ≥ 128 μg/ml), while 13 isolates exhibited dose-dependent susceptibility: 6 *C. glabrata*, 4 *C. parapsilosis,* 2 *C. tropicalis* and 1 *C. albicans*. Only 2 isolates, i.e.1 *C. albicans* (MIC ≥ 128 μg/ml) and 1 *C. parapsilosis*, were resistant to voriconazole, while 3 *C. parapsilosis* and 1 *C. tropicalis* exhibited dose-dependent susceptibility. Resistance to echinocandins was not detected in any strain. Intermediate susceptibility was demonstrated in 2 *C. tropicalis* to caspofungin and anidulafungin, and in 2 *C. glabrata* to caspofungin. Based on the epidemiology data, extrapolated to one year, the majority (64.3%) of IC cases in the participating ICUs were caused by *C.* non-*albicans* spp. (*C. parapsilosis*: 45.7%), with the remaining 35.7% being due to *C. albicans*.

**Conclusion:** Epidemiological patterns of IC reveal a predominance of *C.* non-*albicans* spp.in Greek ICUs, especially *C. parapsilosis*. Since 15/26 (57.7 %) of *C. parapsilosis* isolates were resistant to fluconazole, this observation raises concerns, as fluconazole has traditionally been considered the first-line choice for *C. parapsilosis*. In vitro antifungal susceptibility testing should be performed to assist in the selection of the most appropriate antifungal therapy, and in monitoring the development of resistance.

### P287. Species distribution and antifungal susceptibility patterns of Candida isolated from a Tunisian burn intensive care unit

CheikhrouhouS.^1^,^2^AlouiD.^1^,^2^BouchekouaM.^1^,^2^MessadiA.^2^,^3^ThabetL.^2^,^3^TrabelsiS.^1^,^2^KhaledS.^1^,^2^1Laboratory of Parasitology and Mycology, Charles Nicolle Teaching Hospital, Tunis, Tunisia2Faculty of Medicine of Tunis-Tunis El Manar University, Tunis, Tunisia3Ben Arous Trauma and burn center, Tunis, Tunisia, Ben Arous, Tunisia

**Objectives:** The objective of this study was to analyze the species distribution of *Candida* isolates from a Tunisian burn intensive care unit and their antifungal susceptibility patterns.

**Methods:** We performed a retrospective review of 35 severely burned patients admitted to the burn intensive care unit of Ben Arous Trauma and Burn Center with one or more positive culture sites for *Candida*, during the 28-month period from January 2017 to April 2019. The susceptibility for 6 antifungal drugs (5-fluorocytosine, fluconazole, ketoconazole, micronazole, itraconazole, amphotericin B) was determined using the Fungitest® broth dilution method for patients with infected normally sterile body sites or a *Candida* colonization index superior or equal to 0.4. Voriconazole and caspofungine were tested when needed.

**Results:** A total of 74 *Candida* isolates were obtained. Positive urine cultures yielded 30% of *Candida* isolates. The rectal site represented 15% of colonized body sites, followed by nasal and oral sites (13,5% each), axillary (9,4%), ocular (8%) and cutaneaous and auricular sites (5,4% each). *Candida albicans* was the predominant species (43.2%), followed by *C. glabrata* (31%), *C. tropicalis* (17,5%), *C. parapsilosis* (3%), *C. colliculosa* (3%) and *C. sake* (1%). Among the strains whose antifungal susceptibility were determined, the majority of *Candida* isolates were susceptible to fluconazole (64%), 20,5% of the isolates were intermediate and 15,4% were resistant to this antifungal drug, mainly *C. tropicalis* and *C. glabrata*. There was a high rate of strains with intermediate susceptibility to itraconazole (46%), miconazole (50%) and ketoconazole (23%). Only one strain was resistant to amphotericin B which was *C. tropicalis*.

**Conclusion:** Although the prevalence of non-*albicans* species has increased over recent years, *C. albicans* remains the most frequent species in burn unit-acquired candidiasis. Our antifungal susceptibility test results did not exhibit significant levels of resistance, but non-*albicans* species must be taken into consideration when choosing antifungal agents for calculated therapy.

### P288. Performance of the (1,3)-β-DG-glucan assay for detection of candidemia in adult ICU patients: a preliminary evaluation

NegroG.M.B. Del^1^NavesG.^2^FreitasV.L.^3^MagriM.^4^JuniorJ. De Almeida^5^AbdalaE.^6^SejasO.N.^6^1Laboratório De Investigação Médica Lim53, Insituto de Medicina Tropical, São Paulo, Brazil2Laboratório De Investigações Médicas 53, Insituto de Medicina Tropical, São Paulo, Brazil3Laboratório De Investigação Médica Em Imunologia Lim48, Insituto de Medicina Tropical, São Paulo, Brazil4Clinica De Molestias Infecciosas, Hospital das Clinicas da FMUSP, São Paulo, Brazil5Divisão De Laboratorio Central, Hospital das Clinicas da FMUSP, São Paulo, Brazil6Instituto do Cancer do Estado de São Paulo, São Paulo, Brazil

**Objectives:** Invasive candidiasis (IC), including candidemia episodes, is a considerable cause of morbidity and mortality in immunocompromised critically ill patients. Studies have been reported that early diagnosis of IC is crucial for an appropriate treatment and successful outcome of patients. Analyses using serum levels of the β-D-glucan (BDG) biomarker have shown promising results for the early diagnosis of IC/candidemia. This study aimed to evaluate the performance of the BDG assay as a diagnostic tool for candidemia detection in ICU adult patients with risk factors for IC.

**Methods:** Thirty-nine patients admitted to five ICU from two tertiary hospitals were enrolled, being 11 with proven candidemia (at least one blood culture positive for *Candida*) and 28 patients with negative bloodstream cultures but clinical findings consistent with candidemia (probable candidemia). Blood samples for cultures and BDG testing were collected before administration of antifungal therapy. *Candida* spp. were isolated from blood by using the Bactec™ system and identified by VITEK® MS which uses MALDI-TOF technology. BDG serum concentrations were measured using the Fungitell® assay following the standard operating procedures. All samples were tested in duplicate. The analyses were performed using SPSS Statistics version 24.0.

**Results:** All patients with proven candidemia presented positive results with significantly higher BDG values than those with probable candidemia (median 437.7 vs. 70.6 pg/ml, p <0.001). Among the probable candidemia patients, four (14.3%) showed positive BDG results (ranging from 85 to 276.5 pg/ml), two of them had proven bacteremia, and three patients (10.7%) presented indeterminate values (≥ 60 and ≤ 80 pg/ml). Seven patients of the probable candidemia group (negative bloodstream cultures) presented positive cultures for *Candida* spp. in blood samples harvested from central venous catheter. These seven patients showed significantly higher BDG values when compared to patients with no positive cultures at all (median 151.4 vs. 51.7 pg/ml, p< 0.007).

**Conclusion:** We presented preliminary results from a prospective study for early laboratory diagnosis of candidemia in ICU adult patients at high risk of this systemic infection. Patients with proven candidemia had significantly higher BDG levels than those patients in whom the bloodstream *Candida* infection could not be demonstrated. The BDG positive values observed in patients with positive blood catheter cultures signal that these patients might have true candidemia in spite of negativity of blood cultures. By the other hand, the possibility of false-positive results in this group should be considered and in this scenario another non-culture based methodology, such as real time PCR technique would be required to firmly establish the diagnosis of candidemia.

### P289. Influenza associated invasive pulmonary aspergillosis in Indian intensive care units

RudramurthyS. M^1^ChakrabartiA.^2^ShastriP.^3^PatelA.^4^SavioJ.^5^KarthikR.^6^KaurH.^2^1Medical Microbiolgoy, Postgraduate Institute of Medical Education and Research, Chandigarh, India2Medical Microbiology, Postgraduate Institute of Medical Education and Research, Chandigarh, India3Critical Care, Sri Gangaram Hospital, Delhi, India4Infectious Disease, Sterling Hospitals, Ahmedabad, India5Microbiology, St. John’s Medical College, Bangalore, India6Infectious Disease, Christian Medical College, Vellore, India

**Objectives:** Beyond the classical neutropenic hosts, invasive pulmonary aspergillosis (IPA) is increasingly reported in non-neutropenic critically ill patients. Influenza A H1NI infection is one among those emerging non-classical risk factors. In a multicentre prospective observational study on invasive mould infections (IMIs)in Indian ICUs, we identified 12 cases of IPA associated with influenza H1N1 [1]. Here, we analysed those influenza associated aspergillosis (IAA) cases to evaluate its epidemiology in Indian ICUs.

**Methods:** The study captured all cases of IMIs at 11 ICUs across India during April 2016 through September 2017. IMI was diagnosed as proven, probable and possible based on the EORTC-MSG criteria in the classical immunosuppressed subjects (2), the criteria proposed by Bulpa et al. in subjects with COPD (3), and Vandewoude et al. in non-COPD subjects admitted to the ICU (4). Demographic, clinical presentation, laboratory investigations, treatment and management of proven and probable IMIs were collected using predesigned proforma (1). The present study is the subgroup analysis of clinical and laboratory variables and management of 12 patients diagnosed as IAA.

**Results:** During the study period 135 IPA cases (proven and probable) were reported, 37 (27.5%) had classical risk factor and 98 (72.5%) non-classical risk factors. Among non-classical risk factors 12 patients had IAA; one patient was proven and 11 cases were probable IPA. Those patients were from four centres of India (north-1, south -2, and west-1) and majority (58.3%) cases from a single centre at Delhi. 10/12 cases occurred in summer. The mean age of those patients was 56.8 ± 12.34 years with equal distribution of sex. The mean APACHE score was 18.41 ± 4.56. The diagnosis of IPA was made at a mean of 3.83 ± 2.94 days after ICU admissions. The solid organ malignancies were co-morbidities in two patients, eight patients were on ventilator. *Aspergillus* species was isolated from ten cases (*Aspergillus flavus-5, A. fumigatus* -2, *Aspergillus* spp. – 2 and *A. flavus+A fumigatus* -1). Serum and/or BAL galactomannan was positive in eleven patients. Radiological evidence in the form of opacity (10 cases), cavity (1 case), nodules (5 cases), infiltrates (2 cases), consolidation (7 cases) or pleural effusion (3 cases) was noted in those patients. None of them had classical air crescent or halo sign. The patients were treated with voriconazole (8 cases) or voriconazole + amphotericin B deoxycholate (1 case), voriconazole + caspofungin (1 case) and liposomal amphotericin B (1 case). But, the 42-day or 84-day mortality remained at 58.3% despite targeted antifungal therapy.

**Conclusion:** The study emphasizes that influenza H1N1 is an emerging risk factor for IPA *in* Indian ICUs. Unlike other reports of *A. fumigatus, A. flavus* is the predominant causative agent in Indian scenario. During influenza season, critically ill patients should be thoroughly investigated for IPA. Early diagnosis and prompt therapy may reduce high mortality rate of IAA cases. **References** Chakrabarti et al. (2019). J Crit Care *51*, 64–70. De Pauw B et al (2008). Clin Infect Dis 46:1813–21. Bulpa P(2007) Eur. Respir. J. 30:782–800. Blot SI et al (2012) Am J Respir Crit Care Med: 186:56–64

### P290. rEarly detection of circulating candida antigen and antibodies in ICU patients with candidemia

AnithaS.^1^YaminiA.^1^KindoA.J.^2^1Microbiology, Sri Ramachandra Institute of Higher Education and Research Institute, Chennai, India2Microbiology, Sri Ramachandra Institute of Higher Education and Research, Chennai, India

**Objectives:** Early detection of candida in blood using lateral flow assays and candida mannan assay in ICU patients at risk of invasive candidiasis selected by candida scoring and to compare the results with the Gold standard blood culture method.

**Methods:** Patients having an ICU stay of > 7 days were taken up. *Candida* scoring was done and 33 patients who had scored ≥3 were considered for the study. Patients Age, gender, clinical presentations at admission, severity scores including Acute Physiological and Chronic Health Evaluation II (APACHE II), co- morbid medical conditions, surgical and invasive procedures, exposure to antibiotics, corticosteroids, antifungals, secondary bacterial infections, antifungal treatment instituted in the ICU and final outcome were recorded. Patients were divided into 2 groups based on the presence or absence of *candida*. The blood samples were collected at the onset of sepsis and assays were performed. The samples were tested for the presence of IgM and IgG antibodies specific for candida using lateral flow assay kits and mannan antigen using candida mannan assay kit (Dynamiker Biotechnology (Tianjin) Co., Ltd) which would provide early detection of *candida* in blood and thus facilitating in early start of suitable antifungal therapy and a favorable outcome.

**Results:** A total of 33 patients admitted to the ICU from Jan 2019-April 2019 based on *candida* scoring were considered for the study. of the 33 patients, six were proven cases of candidemia and the remaining 27 were did not grow *candida* in blood culture. The mean age of the patients who had candidemia ranged from 45-82yrs. Among the candidemia patients the male female ratio was equally distributed. The duration of stay in ICU was more for candidemia patients (median days 15). The APACHEII scoring was high among candidemia patient (median 54.5) compared to non candidemia patients (median 36.5). Also the *candida* scoring among candidemia patients was found to be high (median 4.5). The analysis of the results for the presence of IgG, IgM antibodies specific for *candida* and mannan antigen will be done and compared with gold standard blood culture method.

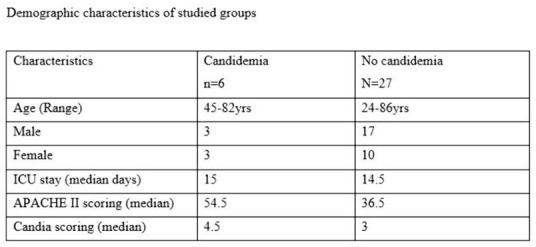



**Conclusion:** The three methods will be compared so that we don’t have to rely always on culture to start the treatment. Development of diagnostic and predictive tests will probably lead to increased use of preemptive therapy. The presence of biomarkers is an indicator to assist in decision to start or stop antifungal treatment. In case of positivity of fungal biomarker, it recommends to initiate preemptive therapy. In case of negativity of fungal biomarker, it suggests to stop empiric therapy.

### P291. An ICU Antifungal Stewardship Programme based on T2Candida PCR and Candida Mannan antigen.

Helweg-LarsenJ.^1^SteensenM.^2^PedersenF. Møller^3^JensenP. Bredahl^3^BondeJ.^2^PerchM.^4^MøllerK.^5^OlesenB. Riis^6^ArendrupM.C.^7^1Department of Infectious Medicine, Rigshospitalet, Copenhagen, Denmark2Department of Intensive Care Medicine, Copenhagen University Hospital Rigshospitalet, Copenhagen, Denmark3Department of Thoracic Anaesthesiology, Copenhagen University Hospital Rigshospitalet, Copenhagen, Denmark4Department of Cardiology, Copenhagen University Hospital Rigshospitalet, Copenhagen, Denmark5Department of Neuroanaestesiology, Copenhagen University Hospital Rigshospitalet, Copenhagen, Denmark6Department of Economy, Copenhagen University Hospital Rigshospitalet, Copenhagen, Denmark7Rigshospitalet, Dept. of Clinical microbiology, Copenhagen, Denmark

**Objectives:** Management of invasive candidiasis (IC) in ICU patients is challenging since traditional diagnostic methods and prediction scores have limited sensitivity and specificity. Non-culture-based biomarkers could potentially improve diagnosis and antifungal treatment (AFT), enabling safer discontinuation of unneeded therapy. We evaluated a new antifungal stewardship programme (AFSP) including T2Candida and *Candida* mannan antigen (MAg) screening of high-risk ICU patients.

**Methods:**

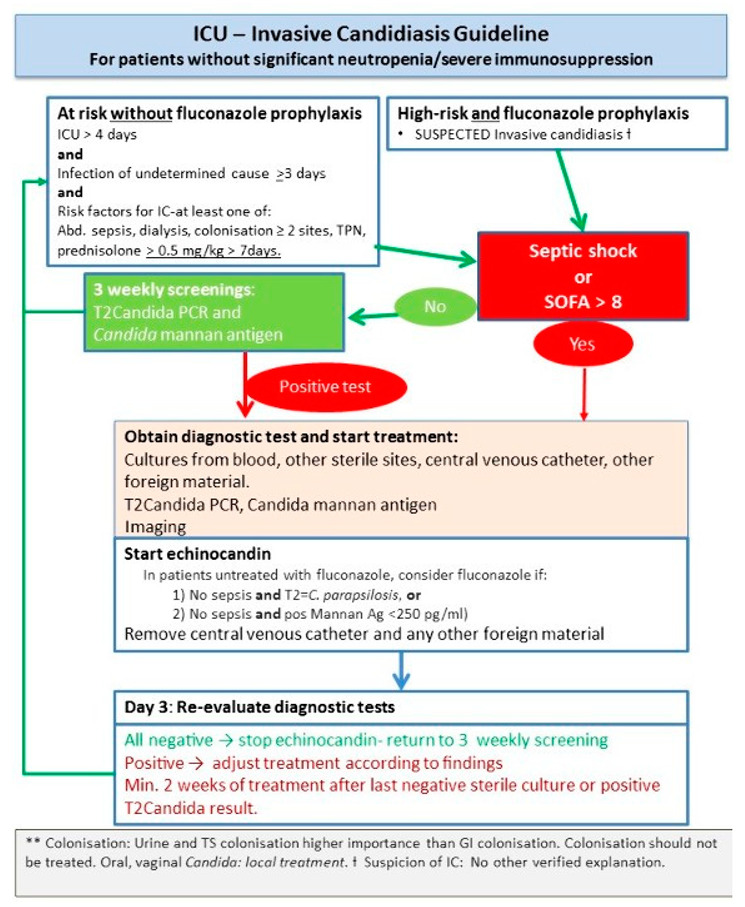

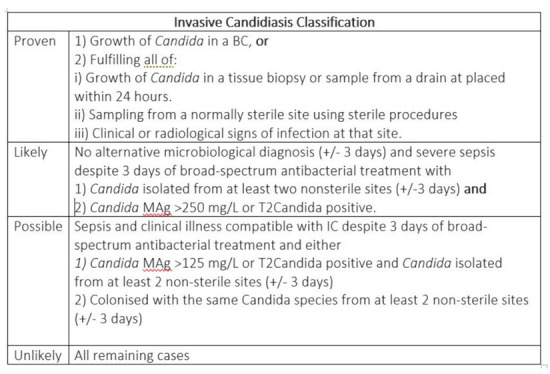





Patients in two ICU’s (abdominal/general and thoracic, respectively) at a tertiary university hospital were eligible. The Figure and Table summarise inclusion criteria, AFSP algorithm and IC classification criteria. The diagnostic value of T2Candida and MAg in relation to traditional methods were evaluated. Consumption of antifungals during the AFSP and the preceding 2 years was compared.

**Results:** 219 patients with 504 T2Candida/MAg samples were included. Median age was 63 years, median ICU stay was 19 days, 45% were receiving renal-replacement-therapy; 29% TPN, 22% underwent acute surgery and the overall in-hospital mortality was 42%. At inclusion, 32% of patients had received AFT for >3 days. 30 patients had positive T2Candida; 24 positive MAg and 18 had positive blood cultures (BC). IC was classified as proven in 29, likely in seven and possible in 10 patients. Defining IC as proven/likely versus unlikely, sensitivity/specificity/NPV values were 47%/100%/91% for BC; 56%/97%/90% for T2Candida; and 39%/96%/88% for MAg. MAg identified five patients with IC, in whom T2Candida or BC were negative. of these, one had proven *C. lusitaniae* IC and four had possible IC (colonizing isolates without species-identification). The best diagnostic performance was the combination of T2Candida&MAg taken ≤3 days after AFT: sensitivity/specificity/PPV/NPV of 70%/90%/ 63%/93%, versus 61%/93%/63%/ 92% for all patients, respectively. Overall, similar performance was observed if IC was defined as proven/likely/possible. The use of T2Candida/MAg contributed to 15% of patients receiving early (<3 days) AFT after a positive test, 25% of patients having early AFT discontinuation and 24% of patients being managed without AFT. No reduction or overall change in use of echinocandin or azole treatment was observed at the two ICU’s in the study period compared to the two previous years.

**Conclusion:** Introduction of an AFSP based on T2Candida and MAg screening of high-risk patients contributed to reducing unnecessary treatment but not overall AFT use. The diagnostic performance of T2Candida for IC was higher than for BC and MAg, but lower than initially reported. T2Candida detects cell-associated *Candida* DNA and thus depend on circulating *Candida* cells. A significant proportion of our patients were already on antifungal therapy at the time of sampling or suffered from BC-negative IC thus likely representing patients with a low number of circulating *Candida* cells. Moreover, a significant proportion of patients were only tested once or twice. Taken together, our study suggests that the combination of T2Candida and MAg improved diagnostic performance compared to T2Candida alone with a sensitivity of 70% and NPV of 93% in patients with ≤3 days AFT. This combination may contribute to improved management of IC in the setting of an AFSP particularly if initiated before AF prescription and with strict adherence to the AFSP.

### P292. Incidence, treatment and outcome of ICU-acquired Candidemia in 5 ICU from Cluj-Napoca, Romania during a 6 years period

LupseM.^1^RusM.A.^1^HerbelL.^2^FlontaM.^3^1Infectious Diseases, University of Medicine and Pharmacy Cluj-Napoca, Cluj-Napoca, Romania2Icu, University Hospital of Infectious Diseases, Cluj-Napoca, Romania3Microbiology, University Hospital of Infectious Diseases, Cluj-Napoca, Romania

**Objectives:** To evaluate the incidence, etiology and outcome of candidemia in ICU from our city

**Methods:** We retrospectively analyzed all cases of candidemia diagnosed based on patients files, considering only the first episode/patient. We included non-neutropenic patients from 5 ICU from our city (1pediatric ICU, 1 medical ICU and 3 surgical ICU) during 6 years (Jan 2007- Dec 2012). We evaluated: demographic data, etiology, antifungal treatment and outcome.

**Results:** There were 107 patients, average age 44.5 (median age 55, min 1 day, max 85 years, median age for PICU patients was 1.4 months), 50 female (46.73%). The incidence was 25.8/1000 admission in PICU and 2.8/1000 admission in SICU. The etiology was represented by *Candida albicans* in 53 (48%) patients, *Candida parapsilosis* in 31 (29%), *Candida glabrata* in 7 (6%) and other species in 15 patients. *Candida parapsilosis* was the most frequent etiology of candidemia for children (45%). Crude mortality rate was 59.8%, higher in adults and in patients infected with *Candida glabrata* (5/6) and *Candida tropicalis* (5/5). 55 patients were treated with fluconazole, 14 with caspofungin, 3 with voriconazole, 10 with ketoconazole and 25 patients did not receive any antifungals. Resistance to fluconazole was 23.36% (24.48% for *Candida albicans*).

**Conclusion:** Candidemia was a frequent situation in ICU non-neutropenic patients. The involvement of *Candida albicans* and *Candida* non-*albicans* was almost equal but significantly different in adults and children. Fluconazole was the most used antifungals but the resistance to Fluconazole was high. The mortality rate was very high, significantly higher in adults than in children and in *Candida* sp. other than *Candida parapsilosis*.

### P293. A Native Human Burn Infection Model of Candidiasis

BulmanF.^1^,^2^RosenbergerD.^2^,^3^MuellerC. Von^2^,^3^VylkovaS.^2^,^3^1Jena University Hospital, Jena, Germany2Septomics Research Center, Friedrich Schiller University, Jena, Germany3Leibniz Institute for Natural Product Research and Infection Biology, Hans Knöll Institute, Jena, Germany

**Objectives:** A tenfold rise in fungal burn wound infections (BWI) has been observed globally since the 1960s due to the introduction of broad spectrum antimicrobial agents (Sarabahi et al, 2012). The fungal opportunistic pathogen *Candida albicans* is a common isolate from BWI (Branski, Al-Mousawi et al. 2009). Systemic candidiasis originating from burn wounds can result in up to 70% mortality rates, yet nothing is known about the biology of infection. The objective of this study was to develop a reliable model of infection. Current animal models such as murine, rat and porcine have major differences in skin biology and inflammatory responses compared to human and are ethically questionable. Therefore, we have developed a novel *ex vivo* human skin model of burn wound *C. albicans* infection and examined the role of key fungal virulence determinants and host responses throughout the progression of infection.

**Methods:** NativeSkin® human tissue (Genoskin, France) was burned with a 350°C soldering iron for 15 sec, debrided and infection was initiated with 1x10^5^ cells of *C. albicans* wild-type SC5314 strain expressing luciferase. The progress of infection was measured by bioluminescence at 1 and 6 days after burn induction, followed by sample fixation for further histological analysis and immunofluorescence staining. Immune cell populations were analysed by flow cytometry and media was measured for the presence of proinflammatory cytokines and lactate dehydrogenase (LDH), a cytotoxicity marker. In some experiments, we supplemented the media with 2x10^6^ polymorphonuclear neutrophils (PMNs) to observe tissue infiltration and effects on healing of the burn.

**Results:** The protocol proved to reliably cause third degree burn wounds in an operator independent manner. Staining for high mobility group box 1 protein (HMGB1) showed that while the unburned skin retained viability after 6 days, the burned skin samples had marked necrosis. Histological analysis showed reduced depth of burn and area of burn injury at day 6 compared to day 1, indicative of tissue regeneration. Flow cytometry analysis of immune cell populations present in control (unburned) skin detected CD1a+ and CD207+ cells, indicating populations of viable langerhans and dendritic cells. Further, staining for neutrophil elastase (NE) showed the presence of PMNs at the edge of the burn. Levels of cytokines IL-1β, IL-6 and Il-8 were variable between skin donors and experimental conditions, with the highest concentration found in burned and infected samples. BWI with *C. albicans* resulted in prominent tissue penetration, especially following debridement of the burn.

**Conclusion:** A reliable and ethical human skin *ex vivo* model for fungal BWI has been established and various analyses methods were tested. Genoskin NativeSkin® samples exhibit good healing potential, have viable immune cell populations and allow supplementation with PMNs and other cells/factors, thus representing an excellent model for BWI candidiasis. The outlook includes testing key virulence determinants for candidiasis, including dual transcriptional profiling; mixed bacterial and fungal infections, and alterations of approved treatment regimes. Such a model may shed light on early signs and symptoms of candidiasis in the burn population, which could help to develop guidelines in patient care.

### P295. Laboratory tools applied in the diagnosis of invasive fungal infections at laboratory of medical parasitology and mycology of the CHU of Annaba. Algeria.

MansouriR.^1^ToutaS.^1^FetniA.^1^SaadniF.^1^AkilS.^2^GrifiF.^2^AtikA.^2^1Pharmacy, faculté de medecine, annaba, Algeria2Medecine, faculté de medecine, annaba, Algeria

Objectives: Invasive fungal infections play an important role as severe infection, constantly rising in the services in charge of patients at risk. Diagnosis and treatment are difficult because of the absence of pathognomonic signs and symptoms. The delay in diagnosis leads to delayed treatment and thus worsened prognosis The objective of this study is to identify the place of laboratory tools applied in the diagnosis of deep candidiasis, Cryptococcosis, Pneumocystosis and invasive aspergillosis.

Methods: This is a descriptive prospective study spanning 07 years (2010-2016) and having respective 998 samples from patients from several medical services: intensive care, Internal Medicine, major burned, Onco-Hematology, infectious diseases, Cardiology, Gastroenterology, Nephrology and Pediatrics. The seach of the mannan antigen for the diagnosis of invasive candidiasis and galactomannan antigen for the diagnosis of invasive aspergillosis occurs by Enzyme Linked ImmunoSorbent Assay. The search for the capsular antigen of Cryptococcus held by slide agglutination and Pneumocystis spp. by DFA. The search for antibody anti-Candida and anti-Aspergillus occurs by Enzyme Linked ImmunoSorbent Assay. Blood cultures and device samples are also studied for these patients.

Results: Among the 371 sera studied for suspected invasive candidiasis, 53 were positive (14.28%) and among 286 sera investigated for suspected invasive aspergillosis, 22 were positive (7.69%). Four cases of Cryptococcosis were confirmed and three cases of Pneumocystosis. Specie isolated from Blood cultures, device and peripheral samples were 93 % C. albicans, 1,5 % were other species: C. parapsilosis, C.zeylanoides, C. glabrata, C.krusei.

Conclusion: Invasive fungal infections remain under-diagnosed clinically and therefore mycologically. The mycological diagnosis associated with sero-immunological diagnosis is a decisive contribution in these conditions. A delay in diagnosis worsens the prognosis of these violations where the interests of a multidisciplinary proper care.

### P296. Non-HIV female cryptococcal meningitis with and without autoimmune diseases

LiuJ.YingJ.HuaP. FuThe Third Affiliated Hospital of Sun Yat-Sen University, Guangzhou, China

**Objectives:** Cryptococcal meningitis (CM) is a rare infection in autoimmune diseases (AIDs) patients. CM complicated with AIDs is more common in women. The aims of this study were to describe the differences of female CM patients with and without AIDs.

**Methods:** We retrospectively compared the differences between 21 non-HIV female CM with AIDs and 23 non-HIV female CM without AIDs from 2011 to 2018. Their demographic data, clinical manifestations, cerebrospinal fluid (CSF) and MR features were analysed.

**Results:** We found that female CM with AIDs had lower CSF pressure, higher CPR and ESR than female CM without AIDs when admitted to the hospital. There was no statistical difference in clinical and MRI features between two groups. After antifungal therapy, headache, fever, mRs scores and some CSF index were significantly improved in both groups. In female CM with AIDs, mRs was positively correlated with India ink Cryptococcus count and had no correlation with CSF pressure. But in female CM without AIDs, we found that mRs was positively correlated with CSF pressure and had no correlation with India ink Cryptococcus count.

**Conclusion:** Our data suggested that SLE was the most common in female CM with AIDs, and revealed for the first time a difference between female CM with and without AIDs. Although presenting symptoms of female CM patients with AIDs are usually unspecific. But it is curable if timely make the diagnosis and treatment.

### P297. Invasive aspergillosis in HIV-positive patients

ShadrivovaO.^1^LeonovaO.^2^BubnovaD.^3^VolkovaA.^4^PopovaM.^4^BogomolovaT.^5^IgnatyevaS.^5^ZubarovskayaL.^4^AfanasyevB.^4^VasilyevaN.^5^KlimkoN.^1^1Department of Clinical Mycology, Allergy and Immunology, North-Western State Medical University named after I.I.Mechnikov, St. Petersburg, Russian Federation2St. Petersburg Center for the Prevention and Control of AIDS and Infectious Diseases, St. petersburg, Russian Federation3North-Western State Medical University named after I.I.Mechnikov, St. Petersburg, Russian Federation4I.Pavlov First Saint Petersburg State Medical University, St. Petersburg, Russian Federation5Kashkin Research Institute of Medical Mycology, North-Western State Medical University named after I.I.Mechnikov, St. Petersburg, Russian Federation

**Objectives:** Analysis of underlying diseases, risk factors, etiology, clinical features, treatment and survival rates in HIV-positive patients with IA.

**Methods:** Retrospective analysis of the register of patients with IAin 1998-2018 yy. For diagnosis IA we used criteria EORTS/MSG, 2008.

**Results:** In group I we included 12 HIV-positive adult patients with IA from 25 to 52 years old, median – 34, males – 58%. The control group consisted of 545 adult patients withhematological malignancies, from 18 to 78 years old, median - 47, males – 58%. The study of risk factors showed significant differences between these groups. Lymphocytopenia was detected predominantly in HIV-positive patients 75% vs 56%, duration 35 vs 14,5 days, (p = 0.006), while agranulocytosis in this group was observed less frequently – 58% vs 81%, p = 0.008, duration 8 vs 13 days (p = 0.01). Among the HIV+ patients there were no recipients of allogeneic stem cell transplants (0% vs 19%, p = 0.001) and patients receiving immunosuppressive therapy (0% vs 29%, p = 0.02). The main sites of infection were lungs - 100% vs 98%, however hemoptysis and dissemination of infection more often registered in HIV+ patients – 17% vs 6% (p = 0.03)and 17% vs 8% (p = 0.04), respectively.Galactomannan test in BAL was positive in 42% vs 75% cases. *Aspergillus* spp. were isolated in 42% vs 44%, in all HIV+ patients the main etiological agent of IA was *A.fumigatus* - 100% vs 45% (p = 0.001). Mixed fungal infection was detected in 33% vs 11% (p = 0,001). «Proven» IA was diagnosed in 17% vs 7% (p = 0.02). Antifungal therapy was used in 100% vs 99% of patients; the most commonly used drug was voriconazole (58% vs 77%). Twelve weeks overall survival rate was 80% vs 81%.

**Conclusion:** The features of invasive aspergillosisin HIV-positive patientswere:prolonged lymphocytopenia (75%),agranulocytosis was a rare risk factor (58%), more frequent mixed infection - 33%, and high frequency of dissemination of aspergillosis – 17%.The overall 12-week survival did not differ in the studied groups (82% vs 79%).

### P298. Invasive aspergillosis in patients with multiple myeloma.

ShadrivovaO.^1^PivovarovaV.^1^ChudinovskikhY.^2^ShneyderT.^3^UspenskayaO.^3^PopovaM.^4^VolkovaA.^4^DesyatikE.^5^BorzovaY.^5^IgnatyevaS.^5^BogomolovaT.^5^ZubarovskayaL.^4^AfanasyevB.^4^VasilyevaN.^5^KlimkoN.^1^1Department of Clinical Mycology, Allergy and Immunology, North-Western State Medical University named after I.I.Mechnikov, St. Petersburg, Russian Federation2N.N. Petrov National Medical Research Centre of Oncology, Ministry of Health of Russian Federation, St. Petersburg, Russian Federation3Leningrad Regional Clinical Hospital, St. Petersburg, Russian Federation4I.Pavlov First Saint Petersburg State Medical University, St. Petersburg, Russian Federation5Kashkin Research Institute of Medical Mycology, North-Western State Medical University named after I.I.Mechnikov, St. Petersburg, Russian Federation

**Objectives:** Identification offeatures of invasiveaspergillosis (IA)in patients with multiple myeloma (MM).

**Methods:** Retrospective analysis of 337 adult hematological patients with IA. In the main group were included 39 patients with MM, median age – 56 years (41 - 79), females - 59%. The control groupincluded 298 hematological patients (acute leukemia – 45%, lymphoma – 36%; chronic leukemia – 13%, myelodysplastic syndrome – 5%; other – 1%), median age – 53 years (40 - 78), females – 56%. The EORTS/MSG 2008 criteria were used for IA diagnosis and assessment of response of therapy.

**Results:** The main risk factorsfor IA were steroid use (87.5% vs 59.5%, p = 0.03), severe neutropenia (51% vs 76%, p = 0.03; median 14 vs 18 days), lymphocytopenia (33% vs 53%, median 10 vs 12.5 days), auto-HSCT (28% vs 4%, p = 0.01), and allo-HSCT (8% vs 13%). The main sites of infection were lungs (97,4% vs 97,3%), usually bilateral (69% vs 77%). The main clinical symptoms were fever (80% vs 78%), cough (69% vs 61%), chest pain (16% vs 5%, p = 0.03), and hemoptysis (0% vs 6.4%, p = 0.001). *Aspergillus* spp. positive culture was received in 69% vs 46% patients. The etiology of IA in patients with MM: *A. niger*– 45%, *A. fumigatus*– 35%, *A.flavus*– 10%, *A. candidus*– 5%, *A. ochraceus*– 5%.Antifungal therapy (voriconazole – 68,2% vs 64%) was used in 100% MM and 98% control group patients. The overall 12-weeks survival rate was 96% vs 80%, p = 0.01.

**Conclusion:** The typical risk factors for invasive aspergillosis in multiple myeloma patients were steroids use (87,5%), and auto-HSCT (28%). The main sites of infection were lungs (97,4%). The main etiological agents were *A. niger*(45%) and *A. fumigatus*(35%). In multiple myelomapatients the overall 12-weeks survival rate was significantly higher compared to the control group (96%vs 80%, p = 0.01).

### P299. Analysis of invasive mycoses in hiv-infected patients by autopsy results in Saint Petersburg, Russia

ShadrivovaO.^1^BubnovaD.^2^PogromskaiaM.^3^KlimkoN.^1^1Department of Clinical Mycology, Allergy and Immunology, North-Western State Medical University named after I.I.Mechnikov, St. Petersburg, Russian Federation2North-Western State Medical University named after I.I.Mechnikov, St. Petersburg, Russian Federation3Department of Infectious Diseases, North-Western State Medical University named after I.I.Mechnikov, St. Petersburg, Russian Federation

**Objectives:** Retrospective analysis of the autopsy data of HIV-infected patients with invasive mycoses (IM).

**Methods:** In retrospective analysis were included 18 HIV-infected patients at the AIDS stage, who were hospitalized in 2018 in the Saint-Petersburg Botkin’s hospital. The median age of patients was 38 years (28 - 67), men – 83%.

**Results:** The duration of HIV infection before the IM development was > 10 years in 44% patients, 5-10 years - 17%, < 5 years – 11%. IM developed before the antiretroviral therapy in 28% patients. Viral hepatitis was concomitant infection in 56% patients, other viral infections (cytomegalovirus, Epstein-Barr and herpes zoster) – 61%, bacterial diseases – 44% (pneumonia – 39%, sepsis – 5%), pulmonary tuberculosis – 11%, toxoplasmosis – 11%. Chronic renal failure was comorbid pathology in 33% patients. The main risk factors of the IM development were CD3+CD4+ <200 cells/mcl – 100%, intensive care unit stay – 67%, and glucocorticosteroid therapy > 21 days (median - 29 days) – 17%. *Pneumocystis jirovecii* pneumonia (PjP) was diagnosed in 67% patients, cryptococcal meningitis – 28%, combination of PjP and cryptococcosis – 5%. PjP was diagnosed ante mortem in 77% patients. Therapeutic doses of co-trimoxazole were used in 60% PjP patients, low doses – 40%. Cryptococcosis was diagnosed ante mortem in 83% patients. Amphotericin B 50 mg/d was used in 67% of these patients(the median duration – 3,5 days (1 - 9), fluconazole 600–800 mg/d – 23%. The overall 14 days survival rate of patients with IM was 39%, 30 days – 28%, 12 weeks – 5,5%.

**Conclusion:** The main risk factor of invasive mycoses (*P. jirovecii* pneumonia – 67%, cryptococcosis – 28%, combination – 5%) in HIV-infected patients was a decrease of CD3 + CD4 + < 200 cells/mcl (100%). The overall 12-weeks survival rate on patients with invasive mycoses was 5,5%.

### P300. rFatal case of Cryptococcus albidus fungaemia in an elderly immunocompromised woman presented with pleural effusion

Rodríguez-LeguizamónG.^1^Coque-BurgosE.^2^Espitia-CastroO.^1^FiracativeC.^3^1Hospital Universitario Mayor - Méderi, Bogota, Colombia2Compensar, Bogota, Colombia3School of Medicine and Health Sciences, Universidad del Rosario, Bogota, Colombia

**Case Report:** Cryptococcosis is an important opportunistic fungal infection that occurs globally and in the vast majority of cases is caused by *Cryptococcus neoformans,* a basidiomycetous encapsulated yeast. Although this mycosis presents most frequently as meningitis or meningoencephalitis, pulmonary disease is an underdiagnosed clinical presentation, mainly due to nonspecific symptoms and broadly categorized findings. Similarly to *C. neoformans*, *Cryptococcus albidus* is a free-living, encapsulated yeast that resides in the environment, but that has been rarely recognized as a human pathogen, as is the case for other non-*neoformans* cryptococci, and whose clinical relevance remains unknown. Here we describe a fatal case of *C. albidus* fungaemia in an 85-year old woman from Colombia, who presented with pleural effusion. The patient had an underlying condition of type 2 diabetes mellitus (T2DM) and arterial hypertension and was admitted to the hospital for hyperglycemia, presenting fatigue, polyuria, polydipsia, and nocturia. On day 4 after admission, she had an episode of atrial fibrillation. A computed tomography (CT) scan of the head showed an old lacunar infarction on the left superior cerebellar artery and chronic ischemic microangiopathy. On day 7, hypoxia (85%) and another episode of atrial fibrillation occurred. Chest radiography showed opacification of the left hemithorax, cardiac silhouette was not visible, and prominent pulmonary hilar vessels with atelectasis on the apical right lobe were observed. A chest and an abdominal CT scans confirmed left-sided pleural effusion and showed an abdominal mass, which suggested a metastatic pleural neoplasm. The condition of the patient deteriorated and she died after 21 days of admission. *C. albidus* was recovered from blood culture *post-mortem* and identified with VITEK®2 YST ID card and confirmed by MALDI-TOF MS. This is the first report to describe a fatal case of cryptococcemia caused by a non-*neoformans* species, *C. albidus,* in a non-HIV, yet immunocompromised female patient, who presented with pleural effusion. Not only are cryptococcal infections of the pleura unusual amongst the pulmonary manifestations but, interestingly, such infections caused by a non-*neoformans* species are even rarer. With an incremental rise in the number of at-risk patients having predisposing factors such as T2DM, the incidence of cryptococcal infection due to non-*neoformans* species has also increased, with *C. albidus* and *C. laurentii* accounting for 80% of reported cases. Although bloodstream and central nervous system are the most common sites of infection of these infrequent pathogens, cases of pulmonary infection are also present and are mostly manifested as chronic, indolent illnesses and pulmonary involvement that includes pneumonia, lung abscess and empyema, which may appear as pulmonary nodules, masses or infiltrates, mediastinal lymphadenopathy or, less frequently, pleural effusions. Among the few cases of cryptococcal pleural effusion that have been reported, only one was caused by a non-*neoformans* species, specifically *C. laurentii*. This case of lethal fungaemia in an elderly woman highlights the importance of considering not only the advanced age and the underlying diseases of the patient, but also the emergence of new pathogens, including fungal species, in order to give an appropriate and prompt diagnostic.

### P301. Isavuconazole (ISAV) as Primary Anti-Fungal Prophylaxis (PAP) in Patients (pts) with Acute Myeloid Leukemia (AML) or Myelodysplastic Syndrome (MDS): An Open-Label, Prospective Study

BoseP.^1^MccueD.^1^WursterS.^1^WiederholdN.^2^KadiaT.^1^BorthakurG.^1^Ravandi-KashaniF.MasarovaL.^1^KonoplevaM.^1^EstrovZ.^1^TakahashiK.^1^YilmazM.^1^RauschC.^1^MarxK.^1^QiaoW.^1^HuangX.^1^BivinsC.^1^PierceS.^1^KantarjianH.^1^KontoyiannisD.^1^1Ut Md Anderson Cancer Center, 1515 Holcombe, Houston, United States of America2Pharmacy, The University of Texas Health Science Center at San Antonio, San Antonio, United States of America

**Objectives:** Mold-active antifungal prophylaxis (ppx) is recommended in neutropenic pts with newly diagnosed AML or MDS. ISAV is an extended spectrum triazole with superior tolerability, reliability of absorption, fewer drug-drug interactions, lack of QT_c_ prolongation or need for therapeutic drug monitoring, approved for the treatment of invasive aspergillosis (IA) and mucormycosis. NCT03019939 is an investigator-initiated, phase 2 trial of PAP with ISAV in pts with AML/MDS.

**Methods:** Treatment-naïve adult pts with AML or MDS initiating remission-induction chemotherapy (RIC) received ISAV per the dosing recommendations in the US label until recovery from neutropenia (neutrophils (ANC) ≥ 0. 5 x 10^9^/L) *and* attainment of complete remission (CR), occurrence of proven or probable invasive fungal infection (IFI, EORTC/MSG criteria), or for a maximum of 12 weeks. The primary endpoint was incidence of proven/probable IFI during the study period (up to 30 days from the last dose of ISAV).

**Results:** 67 pts were enrolled (April 28, 2017 to February 14, 2019) and 60 pts were eligible for assessment (median age 67 years, 57 pts with AML, median ANC on enrollment was 660). Reasons for study completion were achievement of CR with ANC recovery (*n* = 35), completion of 12 weeks of PAP (*n* = 9), possible IFI (*n* = 7), investigator decision (*n* = 3), death (*n* = 2, 1 disease progression, 1 cardiac arrest), proven/probable IFI (*n* = 3), and mild transaminitis, possibly ISAV-related (*n* = 2). The median durations of neutropenia and ISAV ppx were 33 (7-86) and 31 (7-86) days, respectively. One microbiologically-proven (gluteal abscess due to *Candida glabrata*) and 2 cases of probable breakthrough IFIs (probable IA with positive galactomannan) occurred (IFI incidence 5%). ISAV trough serum concentrations were available in 31 pts on both day 8 (median 3. 74 µg/mL, 2. 03-7. 65) and day 15 (median 4. 10 µg/mL, 2. 17-9. 25), and were not significantly different.

**Conclusion:** ISAV is a safe and effective alternative for PAP in pts with newly diagnosed AML/MDS undergoing RIC, with a breakthrough (proven/probable) IFI rate of 5%. ISAV serum levels were adequate in pts with AML/MDS undergoing RIC. Pharmacological features make ISAV attractive for PAP in the era of recently approved or emerging small-molecule AML therapies.

### P302. Retrospective analysis of invasive aspergillosis in pediatric patients with acute myeloid leukemia

DinikinaY.^1^ShadrivovaO.^2^BelogurovaM.^1^IgnatyevaS.^2^BogomolovaT.^2^KhostelidiS.^2^BoichenkoE.^3^,^4^KlimkoN.^2^1Department of Chemotherapy and Bone Marrow Transplantation For Children, Almazov National Research Centre, Saint-Petersburg, Russian Federation2Department of Clinical Mycology, Allergy and Immunology, North-Western State Medical University named after I.I.Mechnikov, St. Petersburg, Russian Federation3Pediatric Hemathology, City Clinical Hospital №1, Saint-Petersburg, Russian Federation4Department of Pediatric Oncohematology, City Clinical hospital №1, Saint-Petersburg, Russian Federation

**Objectives:** To examine the epidemiological, clinical, and therapeutic features of invasive aspergillosis cases in children with acute myeloid leukemia. To underline the necessity of early recognition and initiation of antifungal treatment for the infection control and possibility to continue anticancer treatment.

**Methods:** Retrospective analysis of IA cases in pediatric patients with AML without stem cell transplantation (SCT) registered from 1997 to January 2019. For the probable and proven IA diagnosis EORTC / MSG, 2008 criteria were used.

**Results:** 46 pediatric patients with malignancies were included, median age - 8 years (1 -18), males – 69,5%. Hematological malignancies were underlying diseases in 39 patients (84,7%), solid tumors – 7 (15,2%). Most frequent hematological diagnoses were myeloid leukemia - 16 (34,7%) and acute lymphoblastic leukemia (34,7%). Median age of pts with AML – 11 years, males – 62,5%. IA was diagnosed after cytostatic chemotherapy in 13 patients (81,3%), prior to chemotherapy in 3 (18,7%) pts. Median number of chemotherapy courses before IA manifestation was 3. Two pts were without neutropenia at AML and IA presentation. Risk factors were: prolonged neutropenia – 81,3%, median - 43,9 days, and lymphocytopenia – 81,3%, median – 27,4 days. Severe bacterial infections were diagnosed in 37,5% and cytomegalovirus infection in 18,7%. Main signs of IA were fever (93,7%), cough (75%), respiratory failure (33,4%). Main site of IA were lungs (93,7%). Disseminated IA was diagnosed in 2 pts (12,5%), endophthalmitis - 1 case (6,25%). Typical findings on chest CT scan included the halo sign – 37,5%. Bronchoscopy with bronchoalveolar lavage was performed in 7 (37,5%) pts. *Aspergillus* spp. were isolated in culture in 12,5%: *Aspergillus fumigatus* – 6,25%, *A.niger* – 6,25%. Galactomannan test was positive in 92,8%. According received results “probable IA” was diagnosed in 87,5% of pts and “proven” in 12,5%. Antifungal treatment received 100% of patients, with voriconazole only – 50%. Surgical treatment (splenectomy) was performed in 1 patient with disseminated IA. Combined antifungal therapy received 33,3% of pts. Overall 12-week survival was 87,5%. In all cases of IA before chemotherapy after infection control anticancer therapy was continued.

**Conclusion:** Among children with IA and malignancies, the AML patients without SCT account for 34%. The main risk factors of IA were prolonged neutropenia (81,25%) and lymphocytopenia (81,25%). The main sites of IA were lungs. 12-weeks overall survival was 87,5%. There were no contraindications for anticancer therapy resumption after infection control.

### P303. Species distribution and antifungal susceptibility profile of Candida spp. recovered from liver transplant patients with oral candidiasis

SabadinC.^1^LopesS.^1^GompertzO.^1^RigoL.^2^MeloA.^1^BarbosaD.^1^1Federal University of São Paulo, São Paulo, Brazil2Meridional Faculty – IMED, Passo Fundo, Brazil

**Objectives:** To identify by molecular method *Candida* isolates recovered from liver transplant patients with oral candidiasis and to determine the antifungal susceptibility profile.

**Methods:** In a prospective cohort study, 97 liver transplant patients from a hospital in southern Brazil were followed up from June 2016 to September 2018. On clinical examination of oral cavity 15 of them presented oral candidiasis and were included in this study. Two oral swabs were collected from these 15 patients with interval of 6 months apart (collections 1 and 2) to detect the presence of infection/colonization by *Candida* spp. Patients who had candidiasis were treated with topical nystatin for 30 days. The swabs were cultured on CHROMagar® *Candida* and the yeast colonies were genotypically identified by sequencing the intergenic transcribed sequence (ITS region) of ribossomal DNA. The susceptibility test was performed with the following antifungal agents: Fluconazole, Amphotericin B and Micafungin using broth microdilution method for yeasts recommended by Clinical and Laboratory Standards Institute (CLSI), document M27-A3.

**Results:** Among fifteen patients included in this study, eight(53.4%) presented oral candidiasis episodes in Collection 1 and seven (46.7%) in Collection 2. In fact, all these patients had isolates recovered in both collections, isolates from patients without clinical manifestation were classified as colonization. of those, eight patients who presented oral candidiasis in the Collection 1, five were atrophic candidiasis (AC) caused by *Candida glabrata*(*n* = 3), *C. albicans*(*n* = 1) and C. *tropicalis*(*n =* 1), and threepseudomembranous candidiasis (PC) caused by *C. albicans*(*n* = 2) and *C. tropicalis*(*n* = 1). In the Collection 2, seven patients presented oral candidiasis, five were AC, caused by *C. albicans*(*n* = 4) and *C. dubliniensis*(*n* = 1); two patients had PC caused by *C. albicans*. The yeasts classified as colonizers were *C. albicans*(*n* = 9), *C. glabrata*(*n* = 3), *C. dubliniensis*(*n* = 2) and *C. tropicalis*(*n* = 1). None of the patient had infection or colonization by more than one *Candida* species. Regarding fluconazole susceptibility, 10 of 15 *Candida* isolates recovered from patients with infection were susceptible (S), 4 isolates were susceptible-dose dependent (SDD) and 1 resistant (R). of those recovered from colonized patients 12 were S, 2 were SDD and 1 was R (Table 1). All isolates studied were susceptible to amphotericin B and micafungin. 

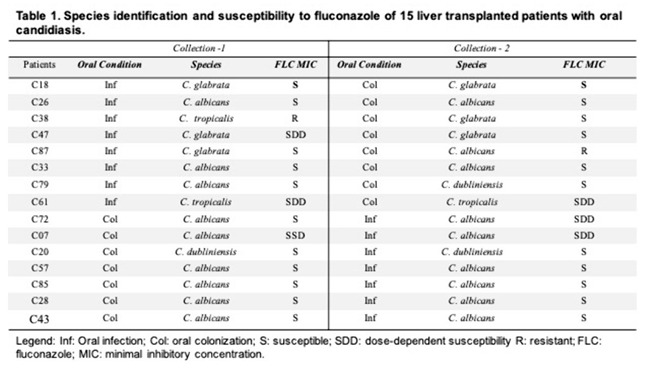



**Conclusion:** The main type of oral candidiasis identified in hepatic transplant patients was AC. *Candida albicans* was the most prevalent species identified in this study. The presence of *Candida* spp. was constant in the oral cavity regardless clinical manifestation. Most isolates were susceptible to antifungal agents, however, one *C. tropicalis* isolate and one *C. albicans* were resistant to fluconazole.

### P304. Cerebral Aspergillosis Lesion Study (CEREALS): a multicenter retrospective French study

SerrisA.^1^DanionF.^1^BenzakounJ.^2^SonnevilleR.^3^WolffM.^3^BretagneS.^4^SharsharT.^2^JouvionG.^5^HerbrechtR.^6^NaggaraO.^2^LortholaryO.^1^LanternierF.^1^1Infectious Diseases, Necker hospital, Paris, France2Neuroradiologie, Sainte Anne Hospital, Paris, France3Icu, Bichat hospital, Paris, France4Mycology, Saint Louis hospital, Paris, France5Pasteur Institute, Paris, France6Hematology, CHU de Strasbourg, Strasbourg, France

**Objectives:** Central nervous system (CNS) aspergillosis is a rare form of invasive aspergillosis. The objective of this study is to describe the clinical, mycological, radiological features and outcomes of the patients with of CNS aspergillosis.

**Methods:** A multicenter, retrospective study was performed involving adults and children with a diagnosis of proven or probable cerebral aspergillosis according to the European Organization for Research and Treatment of Cancer/Mycoses Study Group (EORTC/MSG) definitions of invasive fungal infections (IFI) published in 2008, modified by adding diabetes to the host criteria, between January 2006 and September 2018.

**Results:** 119 patients with CNS aspergillosis (54 proven, 65 probable) were included in 20 French hospitals. Sixty-six percent were men and the median age was 58 years [48-66]. Hematologic malignancies (39.5% of the patients), solid organ transplantation (28.6%), diabetes (8.4%), autoimmune diseases requiring steroid or immunosuppressive treatment (7.6%), cirrhosis (2.5%) and a history of neuro or sinus surgery (2.5%) were the predominant comorbid conditions. Eleven patients (9.2%) had no identified risk factors of IFI. Fever was present in 40.3% of the patients and 88% of them had an abnormal neurological examination. Only 23.5% of the patients had a history of neutropenia in the month preceding the diagnostic. Infection of the CNS occurred by hematogenous dissemination from an extracerebral site in 77 patients (64.7%) and direct extension from the paranasal sinuses in 27 patients (22.7%). Fifteen patients had no obvious primary organ involvement. Hematogenous dissemination was more frequent in patients with hematologic malignancies (68% of the patients) and solid organ transplantation (79%) whereas contiguous extension was more common in patients with diabetes (60%, p = 0.01). Sensitivity of β-D-glucan, galactomannan and aspergillus PCR in sera were 83.3%, 63.5% and 55.1% respectively. Galactomannan was positive in 68.9% of the cerebrospinal fluids tested. A fungal co-infection was diagnosed in 10.9% of the patients. Seventy-two percent of the patients were treated with voriconazole alone or in dual therapy as a first line treatment. Twenty-one patients underwent neurosurgical procedures, including abscess resection or drainage of abscesses and ventricular shunt. Forty-three percent of the patients presented a complication of the surgery such as edema, bleeding or nosocomial infection. Overall survival was 48.3% at 3 months and 37.7% at 12 months. Contiguous infection of the CNS was associated with a lower sensitivity of galactomannan antigen, a higher frequency of vascular complication and a better outcome. Patients with hematologic malignancy had a poorer survival (p = 0.04). Although a selection bias should be considered, the sub-group of patients who had had neurosurgery had a better survival (p = 0,003).

**Conclusion:** CNS aspergillosis should be considered in patients with hematologic malignancies and solid organ transplantation with or without neutropenia but also in patients with less (such as diabetes or cirrhosis) or no known immunosuppression. Mortality among CNS aspergillosis patients remains high. When feasible, neurosurgical procedures may improve prognosis although with significant morbidity.

### P305. Impact of Invasive Aspergillosis occuring during remission-induction therapy on outcome of Acute Myeloid Leukemia (SEIFEM 2012B Study).

CandoniA.^1^FarinaF.^2^PerruccioK.^3^BlasiR. Di^4^CriscuoloM.^4^CattaneoC.^5^DeliaM.^6^LazzarottoD.^1^DubbiniM.V.^1^DragonettiG.^4^FanciR.^7^MartinoB.^8^PrincipeM.I. Del^9^PotenzaL.^10^VianelliN.^11^ChierichiniA.^12^GarziaM.^13^NadaliG.^14^VergaL.^15^BuscaA.^16^PaganoL.^4^1University Hospital, Asuiud, Division of Hematology and Stem Cells Transplantation, Udine, Italy2IRCCS San Raffaele, Milan, Italy3Division of Pediatric Hematology, University Hospital of Perugia, Perugia, Italy4Division of Hematology, IRCCS A Gemelli-University CSR, Rome, Italy5Division of Hematology, Spedali Civili, Brescia, Brescia, Italy6Division of Hematology, University of Bari, Bari, Italy7Division of Hematology, University of Firenze, Firenze, Italy8Division of Hematology, Hospital B. Melacrino Morelli, Reggio Calabria, Reggio Calabria, Italy9Division of Hematology, University “Tor Vergata”, Rome, Rome, Italy10Division of Hematology, University of Modena and Reggio Emilia, Modena, Italy11Division of Hematology, University of Bologna, Bologna, Italy12Division of Hematology, San Giovanni Addolorata Hospital, Rome, Rome, Italy13Division of Hematology, San Camillo Hospital, Rome, Rome, Italy14Division of Hematology, University of Verona, Verona, Italy15Division of Hematology, San Gerardo Hospital, Monza, Monza, Italy16Division of Hematology, AOU Citta della Salute e della Scienza, Torino, Turin, Italy

**Objectives:** Acute myeloid leukemia (AML) patients are at high risk of invasive aspergillosis (IA) and required a mould active antifungal prophylaxis during induction chemotherapy (CHT). Although IA risk factors have been identified, few data are available on impact of IA, occurring during first remission induction phase, on overall AML outcome.

**Methods:** The primary endpoint of this prospective, multicentric, case-control study, was to evaluate if IA, occurring during first remission-induction CHT, can affect treatment schedule and, consequently, patient overall survival (OS). We identified 40 AML patients (cases) who developed IA during induction phase, 31 probable (67%) and 9 proven (33%) IA. These cases were matched with a control group (80 AML) without IA, balanced according to age, type of induction CHT, AML characteristics and cytogenetic-molecular risk factors. The overall response rate to induction CHT was the same in the 2 groups.

**Results:** In the 40 cases with AI, the overall response rate (ORR) to antifungal treatment was favorable (ORR 80%) but it was significantly affected by the achievement of leukemia complete remission (CR) with induction CHT. In fact, in cases with AML responsive to induction CHT, complete responses of IA to antifungal therapy were 96% compared to 21% in cases of AML not responsive to induction CHT (p <0.0001). The adherence to schedule and full doses of CHT was reported in 35% of cases (14/40) and in 76% of controls (61/80) (p = 0.0001). After induction CHT a significant higher number of cases (15/40; 37.5%) compared to controls (21/80; 26%) could not receive additional cycles of CHT (p = 0.01). The IFI related mortality was 22.5%. Comparing OS of 40 cases with the OS of the 80 controls, the median OS of cases was significantly worse with a difference of 12.3 months (12.1 vs 24.4 months, p = 0.04-**Figure 1A**). However, the occurrence of IA during induction phase did not have a significant impact on the OS of cases who achieved a CR with induction CHT which are able to proceed, despite the IA, with their intensive therapeutic program, achieving the same OS as the control group with an AML in CR (p = ns-**Figure 1B**). 

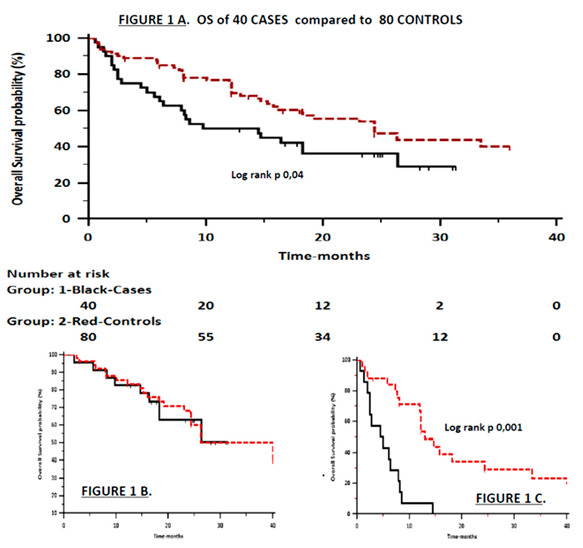



**Conclusion:** These data showed that IAs during induction CHT can delay the subsequent therapeutic program and has a significant impact on OS of AML, specifically in those patients with IA occurring during induction phase who did not achieved a CR of AML with the first course of CHT (**Figure 1C**).

### P306. Invasive pulmonary infection by Syncephalastrum: two case reports

IrshadM.^1^NasirN.^1^HashmiU. Haider^2^FarooqiJ.^3^MahmoodS.F.^1^1Adult Infectious Diseases, Aga Khan University, Karachi-KHI, Pakistan2Haemonc, Aga Khan University, Karachi-KHI, Pakistan3Microbiology, Aga Khan University, Karachi-KHI, Pakistan

**Case Report: Invasive pulmonary infection by Syncephalastrum: two case reports** Memoona Irshad; Nosheen Nasir; Urooj Haider Hashmi; Joveria Farooqi; Syed Faisal Mahmood **ABSTRACT** *Mucormycosis* also known as *Zygomycosis* is a class of fungi including three orders. The largest order is Mucorlaes. Most common species of this order are *Rhizopus* and *Mucor* but there are certain rare species such as Syncephalastrum spp. These are often responsible for opportunistic fungal infections in immunocompromised patients. *Syncephalastrum racemosum* is the species of the genus *Syncephalastrum* and is potentially fatal to human beings. Although usually results in cutaneous infections and onychomycosis, there have been few cases reports of respiratory and CNS infections in immunocompromised hosts. Since this is a rare entity, so hardly any data is available on treatment response to different antifungal agents. However recent improvements in diagnostic strategies result in early identification and treatment. Amphotericin B is the drug of choice due to its low MIC. **Objective:** To describe the clinical manifestations of the rare fungal species Syncephalastrum **Methods:** Two cases of this rare fungus presented with respiratory complaints during 2019 **Case presentation 1:** A 55 years old gentleman recently diagnosed with Acute Myeloid Leukemia and not on treatment, presented with severe shortness of breath in emergency. Patient was provisionally diagnosed with hospital acquired pneumonia in view of recent hospitalization and was started on empirical treatment with intravenous piperacillin tazobactam and vancomycin. However his symptoms progressively worsened. Sputum cultures of this patient showed Methicillin resistant *Staphylococcus aureus* and Syncephalastrum spp in a good quality specimen. Patient was continued on intravenous vancomycin and started on intravenous conventional amphotericin B. **Case presentation 2:** 73 years old gentleman with known comorbid of chronic obstructive pulmonary disease for which he was on long term systemic and inhaled steroids, presented with shortness of breath in emergency room. Chest radiograph showed bilateral infiltrates with left sided cavity formation. Patient was started on empirical treatment for hospital acquired pneumonia in view of his recent hospitalization. Sputum cultures of this patient showed methicillin resistant *Staphylococcus aureus, Aspergillus flavus* and *Syncephalastrum spp* in a good quality specimen. Patient was started on intravenous conventional amphotericin B along with vancomycin and voriconazole. However he developed amphotericin induced nephrotoxicity and doses were renally adjusted **Result:** Both cases were started on amphotericin B, case 1 continued to deteriorate rapidly and expired after 12 days of therapy. However case 2 is under treatment, patient developed amphotericin induced nephrotoxicity so he is currently on renal adjusted dose of amphotericin B and is currently doing well so we had different outcomes in two different patients on same treatment. **Conclusion:** Invasive pulmonary infection by *Syncephalastrum* species is a rare entity. It is usually identified and reported as an environmental contaminant but when identified as a pathogen it can have fatal outcome in immunocompromised patients. Due to rarity of this infection its course and treatment response are not completely understood.

### P307. A case report of Talaromyces marneffei pharyngo-laryngitis: A rare manifestation of talaromycosis

JitmuangA.^1^WongkamhlaT.^1^ChongtrakoolP.^2^1Medicine, Siriraj Hospital Faculty of Medicine, Mahidol University, Bangkok, Thailand2Microbiology, Mahidol University, Bangkok, Thailand

**Case Report:** *Talaromyces marneffei* is a dimorphic fungus predominantly endemic in Southeast Asia. After effective antiretroviral treatment, the incidence of *T. marneffei* infection in HIV-infected individuals has been decreasing. However, the rate of *T. marneffei* infection is rising among non-HIV immunodeficient persons, particulary patients with anti-interferon-gamma autoantibodies. of these, *T. marneffei* usually causes invasive and disseminated infection including fungemia. *T. marneffei* pharyngo-laryngitis is a very rare manifestation of talaromycosis. Herein, we demonstrate a case of *T. marneffei* pharyngo-laryngitis in a patient who had underlying anti-interferon-gamma autoantibodies. **Case report** A 52-year-old Thai woman has been diagnosed anti-interferon-gamma (IFN-ɣ) autoantibodies for 4 years. Last 4 years, she presented with prolonged fever, weight loss 10 kilograms, and multiple cervical lymphadenopathies. Tissue biopsy from left cervical lymph node grew *Mycobacterium abscessus*. Accordingly, disseminated *M. abscessus* infection was diagnosed. She denied using illicit drugs, herbal medicines, or corticosteroids. Immunological studies including anti-HIV testing were all negative, but anti-IFN-ɣ autoantibodies was highly positive. She received intravenous imipenem 2 g/day and amikacin 750 mg/day for a primary anti-mycobacterial therapy, later they were switched to oral clarithromycin 1,000 mg/day and linezolid 600 mg/day for a maintenance therapy. Following the treatment, she was in remission, and still has been receiving the oral maintenance therapy. Before this admission, she had sore throat, particularly more painful at right side of pharynx, and hoarseness for 3 weeks. She also had febrile and lost her weight 5 kilograms. She received oral amoxicillin 1.5 g/day from a primary physician, but her symptoms did not resolve. She denied foreign body sensation, and had no dysphagia, stridor, or difficult breathing. Physical examination revealed marked swelling and hyperemia of both sides of tonsils including uvula and palatal arches, and a single left submandibular lymph node, sized 1 cm. was identified. Indirect laryngoscopy demonstrated moderate swelling of epiglottis, arytenoid, and vocal cord with normal airway opening. There were no skin nodules or hepatosplenomegaly found. Blood chemistries including plain chest radiography were unremarkable. The patient was performed right tonsillar biopsy at 1 day following the admission. The tissue biopsy Gram’s stain including the pathological sections exhibited few oval and elongated yeast-like organisms with some central transverse septum seen, and it subsequently grew few mold colonies with diffusible red-colored pigment on fungal cultures suggestive of *T. marneffei*. An amphotericin B deoxycholate 45 mg/day was commenced as a primary therapy for 1 weeks, followed by an oral itraconazole 400 mg/day. Four weeks after the treatment, the swelling of pharynx and larynx were markedly reduced. She had continued itraconazole for 4 months, later the dose of 200 mg/day has been using for secondary prophylaxis. Her symptoms completely resolved without complication. **Conclusion:** In patients with anti-IFN-ɣ autoantibodies, *T. marneffei* can rarely cause a local infection involving pharynx and larynx. Fungal culture and pathological examination are warranted for diagnosis *T. marneffei* pharyngo-laryngitis. This condition requires a long term antifungal therapy.

### P308. Retrospective analysis of single nucleotide polymorphisms (SNPs) on TLR-4, PTX-3, Dectin-1 genes and the association with the development of invasive fungal infections among hematopoietic stem cell transplant recipients with oncohematological conditions

KalkanciA.^1^TugE.^2^FidanI.^1^TunccanO. Guzel^3^OzkurtZ.N.^4^YeginZ.A.^4^SahinE.A.^1^KuralayZ.^1^1Department of Medical Microbiology, Gazi University School of Medicine, Ankara, Turkey2Department of Medical Genetics, Gazi University School of Medicine, Ankara, Turkey3Department of Infectious Disease and Clinical Microbiology, Gazi University School of Medicine, Ankara, Turkey4Department of Internal Medicine, Division of Hematology, Gazi University School of Medicine, Ankara, Turkey

**Objectives:** *Candida* and *Aspergillus* are the two most common causes of invasive fungal infections after stem cell transplantation. The development of fungal infections can be associated with several risk factors however, it is still difficult to predict why certain individuals develop severe infections, while others, under similar conditions, do not. Genetic polymorphisms are recognized as potential factors that could affect in the progress of the infectious disease. Pattern recognition receptors (PRR) are germline encoded receptors that recognise a variety of pathogen associated molecular patterns (PAMPs) expressed by an invading microorganism. Single nucleotide polymorphisms (SNPs) on coding genes of PRRs; Toll like receptor-4 (TLR-4), dendritic cell-associated C-type lectin-1 (Dectin-1) as a C-type lectin receptor (CLR) and among signaling molecules; Pentraxin-3 (PTX-3) in the complement activation cascade were analysed in order to show associations with the development of invasive fungal infections (IFIs) among hematopoietic stem cell transplant recipients with hematological conditions.

**Methods:** In this case control study, a cohort of 62 patients with hematological malignancies were assigned by the EORTC-MSG criteria. There were 9 proven, 7 probable,13 possible, total 29 IFI patients and 33 patients with no evidence of fungal disease included in this study from 2008 to 2018 in Hospital of Gazi University. DNA and RNA were isolated from deposited bone marrow samples of the patients, using DNA and RNA Isolation kit (SNP Biotechnology, Turkey), respectively. Expression levels of *TLR4*, *Dectin-1* and *PTX-3* genes were determined by normalized to the *Actin* gene using the One-run RT-PCR kit (SNP Biotechnology, Turkey). The polymorphisms of *TLR4* (rs4986790, rs4986791), *Dectin-1* (rs16910526, rs7309123) and *PTX-3* (rs2305619, rs3816527) were determined by RT-PCR. Continuous variables are reported as means ± standard error mean (SEM), while categori cal variables are reported as frequencies and percentages (%). The odds ratios (ORs) and cor responding 95% confidence intervals (CIs) were calculated by unconditional logistic regres sion analysis. The homozygote for the most frequent allele was regarded as the reference group. SPSS statistical package, version 11.0 (SPSS Inc., Chicago, IL, USA) for Windows was used for statistical analyses. All *p* values was considered to be statistically significant when *p*< 0.05.

**Results:** *TLR-4*, *PTX-3*, *Dectin-1* genes were down-regulated in IFIs group when compared to patients with no evidence of fungal disease in the group of hematological conditions. A higher expression of *TLR-4* was revealed in controls (0.0626±0.032) compared to IFI patients (0.0077±0.014), significantly different (*p* = 0.026), as well as *PTX-3* in controls (0.5265±0.0043) compared to patients (0.0043±0.004), (*p* = 0.035). *Dectin-1* expression was down-regulated in IFI group (0.1887±0.072 & 0.0655±0.010), however, not statistically significant (*p*>0.05). Conditional logistic regression analyses indicated that the GT genotype of rs16910526 polymorphism in *Dectin-1* gene was associated with lower risk of IFIs. (Odds ratio = 3.635, 95% confidence interval = 0.690-3.138, *p* = 0.04

**Conclusion:** Our study continues to highlight the important role of the host’s underlying genetic profile in determining susceptibility to IFIs. Selective genetic immunodeficiencies stratify “at-risk” patients to specific pathogens. Dissecting the contribution that host genetic variation has on the susceptibility to fungal infection will be principal for personalizing medicine in infectious diseases.

### P309. Cell wall composition of Mucorales during spore germination: towards better understanding of host-pathogen interactions.

LecointeK.^1^CoulonP.^1^CornuM.^1^SendidB.^2^1Laboratory of Mycology and Parasitology, University Hospital of Lille, LILLE, France2Laboratory of Mycology and Parasitology, University Hospital of Lille, Lille, France

**Objectives:** The molecular composition and structural organization of the cell wall of filamentous fungi underlie the ability of the host to sense them as pathogen (Gow *et al*. 2017). Although the organization of the fungal cell wall, composed of 90% of polysaccharides (PS), is similar from one fungi to another, their expression on the cell wall may condition their ability to trigger pattern recognition receptors. Deciphering cell wall polysaccharide composition of filamentous fungi of medical concern can lead to a better understanding of mechanisms governing their pathogenicity. Those patterns of PS could also serve as biomarkers for the diagnosis of invasive fungal infections. Because the incidence of Mucormycosis, an emerging life-threatening infection due to Mucorales, is rising worldwide (Prakash and Chakrabarti 2019), we intend in this study to characterize the structure of cell wall of *Lichtheimia corymbifera* (one of the most clinically important mucorales) and how this fungus is able to interact with the host’s immune system.

**Methods:** Two strains of *L. corymbifera* were used, one reference strain - IHEM 21658 - and one strain isolated from skin lesions in burn patient. Spores and germ tubes were obtained from cultures on SDA-amikacin and liquid medium with yeast extracts, mannitol and glucose, respectively. Monosaccharides composition (MC) analysis of the cell wall was conducted on spores and germ tubes, on alkali-insoluble and alkali-soluble fractions through gas-chromatography using flame - ionisation detector. The same analysis was also performed on secreted extracellular polysaccharides obtained after an 8-day culture in YNB with glucose. Determination of the glycosidic linkages was performed on soluble oligosaccharides isolated sequentially after zymolyase and chitinase digestion. Confocal microscopy after immunofluorescence staining with two lectins (Con-A and WGA, binding mannose and N-acetyl-glucosamine -GlcNAc-, respectively), and one anti-β-1,3-glucan monoclonal antibody (1A5), was performed to determine the parietal remodeling during germination. THP-1- derived macrophages stimulated by various fungal fractions were used to assess fungi-host interaction in terms of Toll-like receptor and cytokine expression.

**Results:** MC of both strains showed a large amount of glucose (69.9%) with smaller amounts of mannose (16.5%) and GlcNAc (12.8%) and traces of glucuronic acid (0.9%) in spores. Enrichment using alkalino-treatment showed the presence of glucuronic acid and galactose in the alkali-soluble fraction. Fucose appeared in germ tubes. Analysis of extracellular polysaccharides showed the presence of fucose, glucose, mannose, galactose and GlcNAc. Tagging the spores with Con-A and WGA revealed the presence of mannose around spores, whereas mannose was spread more evenly in germ tubes. Chitin emerged along the germ tube and β-1,3-glucan was present in swollen conidia.

**Conclusion:** During the first hours of *L. corymbifera* germination, fungal cells synthetize polysaccharides composed chiefly by glucose, GlcNAc and mannose, and to a lesser extent by galactose, glucuronic acid and fucose. The role of these latter carbohydrates remains uncertain (Bartnicki-Garcia 1968). Confocal microscopy suggests that the emergence of these carbohydrates leads to a parietal reorganization of the cell wall. In the near future, data from the *in-vitro* model could help us to understand the impact of these changes on the primary immune response.

### P310. rSaprochaete clavata outbreak in a Hematology Unit in Marseille, France

MenuE.^1^CriscuoloA.^2^Desnos-OllivierM.^3^CassagneC.^4^,^5^RanqueS.^4^,^6^BergerP.^7^DromerF.^3^1AP-HM La Timone - IHU Méditerranée Infection, Marseille, France2Hub De Bioinformatique Et Biostatistique – Département Biologie Computationnelle, Usr 3756 Cnrs, Institut Pasteur, Paris, France3National Reference Center For Invasive Mycoses & Antifungals, Molecular Mycology Unit, Cnrs Umr2000, Institut Pasteur, Paris, France4IHU-Méditerranée Infection, Marseille, France5Vitrome, Aix Marseille UnivIRD, AP-HM, SSA, Marseille, France6Vitrome, Aix Marseille Univ, IRD, AP-HM, SSA, Marseille, France7Institut Paoli-Calmettes, Marseille, France

**Objectives: Objectives:** *Saprochaete clavata* is a rare pathogenic yeast that has been involved in invasive fungal infection outbreaks in immunocompromised patients especially in patient with haematological malignancies. From January 2016 to January 2018, infections due to *S. clavata* were diagnosed in nine patients at a cancer center in Marseille, France raising the question of a common source and of the mode of transmission.

**Methods: Methods:** We retrospectively reviewed patients’ files to assess risk factors, hospitalization units and treatments, and carried out an environmental survey (59 samples collected). Whole genome sequencing (WGS) was performed for 17 clinical isolates collected during (*n* = 15) or before (*n* = 2) the outbreak and 10 environmental isolates. The type strain (CBS425.71) and representative clinical isolates related (clade A) or unrelated (clade B) to the French national outbreak in 2012 (Vaux et al., 2014) were used as reference.

**Results: Results:** All nine patients were hospitalized at the stem-cell transplant or hematological intensive care units. The median age was 58 years (range: 38 to 68) and six (67%) were male. The most common underlying disease was acute myeloid leukemia (56%) and 67% of patients were treated with cytarabine. A total of 40 samples were found positive for *S. clavata*: blood (*n* = 35, 1 to 9 /8 patients), bronchoalveolar fluid or tracheal aspirates (*n* = 5), and fecal swab (*n* = 1). The case fatality rate at day 60 was 44%; death occurred at a median of 9.5 days after diagnosis. *Saprochaete clavata* was also cultured from 10 environmental samples including one from a surface in a patient’s room, 5 from the dishwasher (water outlet, inside and door seal) made available to patients and families, and 4 others from pitchers (lids and inside of 2 milk and coffee thermos). In order to control the outbreak, all the contaminated materials were replaced and the dishwasher was discarded. No new case was reported after the implementation of corrective actions. Phylogenetic analysis based on WGS data showed that all clinical and environmental isolates from Marseille belong to the same clade which was unrelated to clades A and B previously identified in France. of note, attempt to grow *S. clavata* failed at temperatures above 48°C.

**Conclusion: Conclusion:** Our results place the dishwasher as the source of the transmission of *S. clavata* and suggest that its heating system may have been deficient. Whether the source of *S. clavata* was a diary product as in previously reported outbreaks remains unclear. Vaux, S., Criscuolo, A., Desnos-Ollivier, M., et al. (2014). Multicenter outbreak of infections by Saprochaete clavata, an unrecognized opportunistic fungal pathogen. *MBio*, *5*(6), e02309-14.

### P311. 18F-FDG PET-computed tomography to assess chronic disseminated candidiasis: results of a French pilot prospective multicentric study (CANHPARI)

RammaertB.^1^,^2^MaunouryC.^3^RabeonyT.^4^ElieC.^4^BakouboulaP.^4^AlfandariS.^5^BergerP.^6^RubioM.-T.^7^BraunT.^8^CorreasJ.-M.^9^MontraversF.^10^LortholaryO.^2^1Current Address: Infectious Diseases, CHU Poitiers, Poitiers, France2Infectious Diseases, APHP, Hopital Necker-Enfants malades, Paris, France3Nuclear Medicine, Hopital europeen Georges Pompidou, Paris, France4Urc Necker-cochin, APHP, Paris, France5Infectious Diseases, Centre hospitalier Tourcoing, Tourcoing, France6Institut Paoli-Calmettes, Marseille, France7Hematology, CHU Nancy, Nancy, France8Hematology, APHP, hopital Avicenne, Bobigny, France9Adult Radiology, APHP, hopital necker-enfants malades, Paris, France10Nuclear Medicine, APHP, hopital tenon, Paris, France

**Objectives:** Chronic disseminated candidiasis (CDC) occurs after profound and prolonged neutropenia, and consists in persistent fever and multiple abscesses in liver and/or spleen. International guidelines recommend protracted course of antifungal therapy, although there is some evidence that CDC belongs to spectrum of immune reconstitution inflammatory syndromes. The aim of this study was to assess 18F-fluorodeoxyglucose positron emission tomography/computed tomography (PET/CT) in the diagnosis and follow-up of CDC.

**Methods:** A pilot prospective study was conducted in 38 French centers from November 2013 to March 2017 (NCT01916057). Hematological adult patients having experienced febrile profound (neutrophils <100/mm3) and prolonged (≥10 days) neutropenia, and suspect for CDC on imaging (<10 mm multiple nodules on liver and/or spleen) were included. PET/CT and abdominal conventional imaging (CT or MRI) were performed initially and at 3 months (M3). MSG/EORTC criteria were adapted to assess response to treatment. At M3, clinical success was defined by resolution of fever attributable to CDC, complete biological response was defined by normal liver enzymes and CRP ≤20 mg/L, and complete radiological response by disappearance of lesions compared to initial imaging. The primary endpoint was the global response to antifungal treatment at M3 defined by clinical success and extinction of hepatosplenic metabolic uptake on PET/CT.

**Results:** A total of 52 cases were included; 6 were secondarily excluded for alternative diagnosis and 2 for absence of PET/CT at diagnosis. Among the 44 remaining cases, CDC was proven in 7 (6 candidemia, 1 positive culture of liver biopsy), probable in 13, possible in 24. Patients were mostly male (55%), with a median age of 46 y.o. (IQR 32-62). Among 43 underlying hematological disease, there were 46% acute myeloid leukemia, 30% acute lymphoid leukemia, 17% allogenic and 2% autologous stem cell transplantation, and 5% lymphoma. One patient had a solid cancer. Corticosteroids were prescribed in 47% of patients ≤ 1 month before CDC diagnosis, growth factors in 68%, antifungal prophylaxis in 64%. Patients were colonized with ≥1 *Candida* species in 75% of cases before CDC diagnosis, mainly *C. albicans*. At diagnosis, fever and liver enzyme abnormalities were present in 81% and 82% of cases, respectively. C-reactive protein was elevated in 85%. All patients were treated with antifungal drugs, combined with corticosteroids in 41%. At M3, 25% (8/32) of evaluable patients met the primary endpoint criteria. However, among 35 patients who had clinical success, 77% (27/35) had complete biological response, but 83% (29/35) failed to respond on conventional imaging. Among these patients, extinction of hepatosplenic metabolic uptake on PET/CT was observed in 17% (5/29).

**Conclusion:** Clinical improvement of CDC occurs before radiological normalization. In patients with clinical success at M3, PET/CT could be more helpful than conventional imaging to guide antifungal duration.

### P312. Invasive fungal disease with isavuconazole treatment failure diagnosed using a combination of fungal biomarkers.

BellangerA.-P.^1^BerceanuA.^2^SchererE.^3^DesbrossesY.^2^DaguindeauE.^2^RocchiS.^1^MillonL.^1^1Mycology, University Hospital Jean Minjoz, Besancon, France2Hematology, University Hospital Jean Minjoz, BESANCON, France3Mycology - Umr6249 Cnrs Chrono-environnement, University Hospital Besançon, Besançon, France

**Case Report:** Isavuconazole is the most recent antifungal azole available to treat invasive fungal disease (IFD) and has been approved as first line treatment for invasive aspergillosis (IA) and as alternative treatment for mucormycosis. We present two cases of IFD with isavuconazole treatment failure in acute myeloid leukemia (AML) patients with prolonged neutropenia 100 days after hematopoietic stem cell transplantation (SCT) and severe digestive graft-versus-host disease (GVHD). The first patient was diagnosed with a probable invasive aspergillosis and a TR34/L98H azole resistant *Aspergillus fumigatus* and the second patient was diagnosed with a mixed *Aspergillus fumigatus-Rhizomucor* sp co-infection.The IFD were diagnosed using a combination of fungal biomarkers, applied systematically and including the galactomannan antigen (GM) detection and fungal qPCRs, targeting both *A. fumigatus* and Mucorales; this strategy proved to be optimal. These cases highlight the interest of applying a close surveillance using fungal biomarkers in very immunocompromised patients under antifungal treatment. Interestingly, the fungal biomarkers were alternatively positive. Moreover, these cases also raise several questions such as for example the influence of severe digestive GVHD on isavuconazole levels.

### P313. Six consecutive patients surviving invasive Mucormycosis: a result of improved interdisciplinary management?

DatcuR.^1^RisumM.^1^Helweg-LarsenJ.^2^KampmannP.^3^OvergaardU.M.^3^NielsenO.J.^3^MunksgaardL.^4^RubekN.^5^HansenL.V.^6^HareR.^1^ArendrupM.C.^1^,^7^,^8^1Mycology Unit, Statens Serum Institute, Copenhagen, Denmark2Department of Infectious Medicine, Rigshospitalet, Copenhagen, Denmark3Department of Hematology, Rigshospitalet, Copenhagen, Denmark4Department of Hematology, Roskilde Sygehus, Roskilde, Denmark5Department of Otorhinolaryngology, Rigshospitalet, Copenhagen, Denmark6Department of Orthopaedic Surgery, Rigshospitalet, Copenhagen, Denmark7Rigshospitalet, Dept. of Clinical microbiology, Copenhagen, Denmark8University of Copenhagen, Dept. of Clinical Medicine, Copenhagen, Denmark

**Objectives:** *Mucorales* infections are life-threatening, with mortality rates approaching 100% in disseminated cases. ESCMID/ECMM guidelines strongly recommend surgical debridement, liposomal amphotericin B, L-AmB (≥5 mg/kg/day), and reversal of predisposing conditions. We introduced a multidisciplinary and aggressive management approach for invasive mucormycosis. Here we present data on six patients managed by this approach, whom all had a successful outcome.

**Methods:** Management principle: repeated surgical debridement until biopsies taken from the resection margins were clean, defined by negative Blankophor microscopy, *Mucorales* specific PCR (both reported within 24h) and cultures. Cultured isolates underwent ITS sequencing for species identification and EUCAST E.Def 9.3.1 susceptibility testing. Targeted antifungal therapy (mono/combination) combined with topical therapy (when possible) was given according to the MIC (mg/L) and severity of infection. High trough azole levels above MIC were ensured by therapeutic drug monitoring (TDM) (mg/L). Fungal free survival was evaluated by case record review.

**Results:**

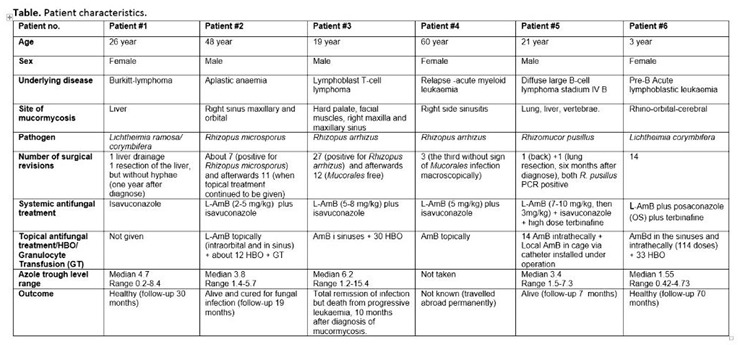



Demographic, microbiological, management and outcome data for the six patients are summarised in the Table. All were neutropenic after chemotherapy except patient #5 at time of diagnosis. Patient #1 developed *Lichtheimia ramosa/corymbifera* infection in the liver (PCR). Management included isavuconazole and surgery. Patient #2 developed sino-orbital *Rhizopus microsporus* infection. The patient had smoked cannabis. Therapy included surgery, L-AmB and isavuconazole. Isavuconazole MIC: 1 mg/L. Patient #3 developed *Rhizopus arrhizus* infection extending extensively from the hard palate (picture). The patient had smoked marihuana. Treatment consisted of unilateral hemimaxillectomy followed by frequent supplementary debridements guided by PCR of resection margins. The exposed bone was covered during palatal reconstruction with a free flap after several mycosis negative debridements. Antifungal therapy included L-AmB and isavuconazole (target TDM initially: >2 mg/L, then >6-8 mg/L until negative diagnostics followed by maintenance >2 mg/L (isavuconazole MIC: 1 mg/L). Patient #4 developed *Rhizopus arrhizus* sinuses infection during posaconazole prophylaxis. Management included L-AmB and surgery, followed by isavuconazole (isavuconazole MIC: 2 mg/L). Patient #5 developed disseminated *Rhizomucor pusillus* (lumbar spondylitis, a subdiaphragmatic and a lung abscess). Surgical debridement including removal of vertebrae L4 in toto and surrounding muscle and soft tissue. The spine was then stabilized with cage and pedicle screws. PET-CT afterwards showed regression of the spondylitis. Target TDM for isavuconazole >2 mg/L. Patient #6 developed *Lichtheimia corymbifera* sinus infection. Despite targeted posaconazole oral solution aiming a target for TDM >2 mg/L (MIC: 0.25 mg/L), the infection progressed to nasopharynx, orbit and brain. The patient recovered after surgical and triple-compound systemic and amphotericin B intrathecal antifungal therapy^1^. Outcome: At follow-up 6-70 months after diagnosis, patient #1-4 and #6 were cured of infection, and patient #5 has had no sign of relapse (7 months follow-up).

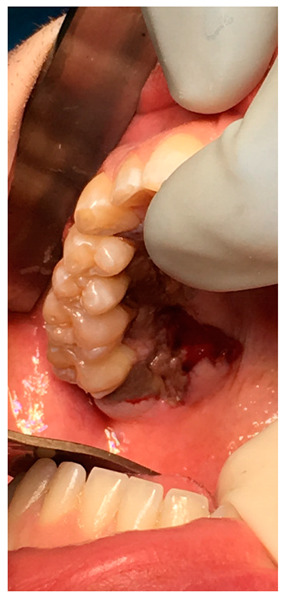



**Conclusion:** Our data suggest improved outcome of *Mucorales* infections by a multidisciplinary approach including: 1) Repeated (except patient #5) biopsy-controlled surgery until resection boarders are negative, 2) Systemic and in most cases combination, TDM-adjusted high dose systemic antifungal therapy, and 3) Topical therapy when possible to improve antifungal exposure in tissue not sufficiently perfused due to the angioinvasive pathology of the infection. ^1^Jensen, TSR et al. *J Pediatr Hematol Oncol*. 2017;39(4):211-215.

### P314. Majocchi’s Granuloma by Trichophytum rubrum in a kidney transplant patient - A Case Report

CruzS. Matos^1^SilvaL.^2^CatorzeG.^2^SabinoR.^3^VerissimoC.^3^ToscanoC.^1^1Microbiology Laboratory, Hospital Egas Moniz - CHLO, Lisbon, Portugal2Dermatology Department, Hospital Egas Moniz - CHLO, Lisbon, Portugal3Reference Unit For Parasitic and Fungal Infections, Infectious Diseases, National Health Institute Dr. Ricardo Jorge, Lisbon, Portugal

**Case Report:**

**Introduction**: *Trichophytum rubrum* is a filamentous fungus, with worldwide distribution, that usually causes superficial infections of skin and nails, namely *tinea pedis*, *tinea corporis*, *tinea cruris* and onychomycosis. Rarely, severe dermatophytosis can occur, presenting as deep dermatophytosis, Majocchi’s Granuloma or extensive dermatophytosis. 

**Objectives and Methods**: Case report of Majocchi’s Granuloma in a kidney transplant patient. 

**Results**: A case of a 55-year-old woman who underwent a kidney transplant 7 months before, under immunosuppressive therapy with tacrolimus and mycophenolate mofetil. She attended a Dermatology consultation to clarify skin lesions that appeared 6 months earlier. The skin exam revealed hard and painful plaque lesions on both legs, with an ulcer on the left leg lesion, violaceous papular lesions on the dorsum of the left foot and toes and a hard consistency nodule on the left leg. Some of the toe nails presented dystrophy or onycholysis. The patient denied any previous trauma or contact with plants or soil. Biopsies of lesions of the left leg and foot dorsum where sent for histology and mycological culture and toe nails for mycological culture. The histological examinations showed, in the reticular dermis and reaching the hypodermis, suppurative granulomas with multinucleated giant cells and areas of necrosis. PAS (Periodic Acid-Schiff) and GMS (Grocott’s Methenamine Silver) staining revealed multiple spores and septate hypha within the granulomas but not in the *stratum corneum*. No remnants of hair follicles where found. Culture of skin biopsies were positive for *Tricophytum rubrum* but nails´ culture was negative. Identification was further confirmed by sequencing of ITS region of ribosomal DNA (GenBank accession number MK967277). Oral Itraconazole 100mg bid and topic Sertoconazole where initiated. The patient was observed one month after and reported general malaise, tiredness, exertional dyspnea, whitish stools and increased abdominal volume. The physician chose to discontinue itraconazole and initiate oral terbinafine 250mg id. After two months on oral terbinafine, there was regression of the legs´ and left foot lesions with ulcer healing and disappearance of the left leg nodule. **Conclusion:** Diagnosis of deeper dermatophytosis is difficult, in part because there is no specific clinical presentation and, in many cases, it is even polymorphic. However, especially in patients with immunodeficiency, this hypothesis should be weighed. Confirmation is achieved by finding hyphae compatible with dermatophytes in the dermis and a positive culture for a dermatophyte. Treatment should include systemic antifungal agents, to which topical medication may be associated. Multiple therapeutic regimens have been proposed, but randomized trials or large case series are lacking. Antifungal therapy should be continued until the lesions are completely resolved. Surgical treatment has been reported as an option for highly localized lesions.

### P315. Clinical Characteristics and Outcomes of Lomentospora prolificans Infections in Fungiscope™ Registry

JenksJ.^1^SeidelD.^2^CornelyO.A.^3^KauffmanC.^4^MiceliM.^5^SlavinM.^6^ChenS.^7^MehtaS.^1^ReedS.^1^HoeniglM.^1^1Medicine, University of California San Diego, San Diego, United States of America2Department I of Internal Medicine, Ecmm Excellence Centre of Medical Mycology, Cologne Excellence Cluster On Cellular Stress Responses In Aging-associated Diseases (cecad), University Hospital Cologne, Cologne, Germany3Department I of Internal Medicine, Ecmm Excellence Centre of Medical Mycology, Cologne Excellence Cluster On Cellular Stress Responses In Aging-associated Diseases (cecad), German Centre For Infection Research, Partner Site Bonn-cologne, Clinical Trials C, University Hospital Cologne, Cologne, Germany4University of Michigan, Ann Arbor, United States of America5Medicine, University of Michigan, Ann Arbor, United States of America6Medicine, University of Melbourne, Parkville, Victoria, Australia7Medicine, The University of Sydney, Camperdown, New South Wales, Australia

**Objectives:** *Lomentospora prolificans* are filamentous fungi commonly found in soil and polluted waters and are emerging opportunistic pathogens in immunocompromised individuals, accounting for 2.4% and 11.1% of non-*Aspergillus* invasive fungal infections (IFIs) in haematopoietic stem cell transplant (HSCT) and solid organ transplant (SOT) infections, respectively. The objective of this study was to describe clinical manifestations, treatment and outcomes of patients with *L. prolificans* infections causing IFI in cases documented in Fungiscope™ - A Global Emerging Fungal Infection Registry, created in 2003.

**Methods:** We performed a retrospective review of medical records of all patients with IFIs caused by *L. prolificans* in Fungiscope™ between 01/01/2008 - 12/31/2017 (10 year period). Infections were determined to be disseminated if *L. prolificans* was isolated from the blood or two non-contiguous anatomic sites. The study was approved by the UCSD Human Research Protection Program (IRB #181119).

**Results:** A total of 37 cases with IFIs caused by *L. prolificans*, including 7 cases from the University of California San Diego, were identified (**Table 1**). In all but one case (36/37, 97.3%), a risk factor for IFI was found, including hematologic malignancy (20/37, 54.1%), solid organ transplant (SOT) (3/37, 8.1%), ICU stay or trauma (6/37, 16.2%). The majority of infections were diagnosed based on positive culture (35/37, 94.6%) or histopathologic findings plus culture (5/37, 13.5) and most patients had signs of IFI on CT scan (15/20, 75%), MRI (1/2, 50%), or CXR (1/2, 50%). At final assessment, 19/37 (51.3%) were deceased, 8/37 (21.3%) had complete cure, and 8/37 (21.6%) and partial resolution or stable disease. of those who received antifungal therapy following diagnosis and had an evaluable outcome (31/37, 84%), the majority were treated with combination antifungal therapy (24/31, 77%; 54% survived). Most antifungal regimens included voriconazole (VRC; 28/31, 90%; 54% survived) and/or terbinafine (TRB; 19/31, 61%; 63% survived). Among those receiving a combination of both VRC and TRB (+/- other antifungal agents) 11/17 (65%) survived, while 5/14 (36%) survived among those receiving other treatments (p = 0.11). Overall, 7/37 patients (18.9%) received surgery, including 5/7 enucleation or vitrectomy and two extensive debridement (1 died, 5 survived, 1 with unknown outcome). Table 1. 

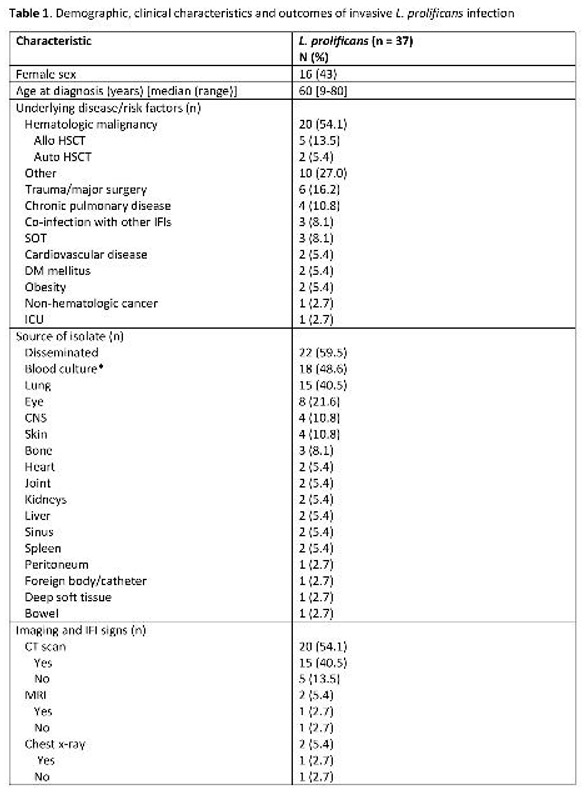

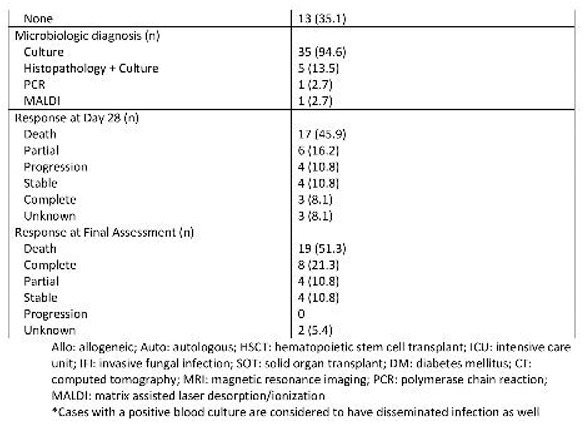





**Conclusion:** In this study, the majority of patients had a significant underlying risk factor for infection including hematologic malignancy, SOT, ICU admission, or preceding trauma. Despite the use of combination antifungal therapy, the overall mortality rate from invasive *L. prolificans* infection was 51%, with a trend towards lower infection-related mortality (35%) in those receiving a combination treatment including both VRC and TRB.

### P316. Making continuous prospective surveillance of invasive mold diseases real-time in hospitals using artificial intelligence

Ananda-RajahM.^1^,^2^SeahJ.^2^LiuM.^3^HaffariG.^3^BergemeirC.^3^PeelT.^1^1Infectious Diseases, Alfred Health, Melbourne, Australia2Alfred Health, Melbourne, Australia3It, Monash University, Clayton, Australia

**Objectives:** Surveillance, audit and feedback of invasive mold diseases (IMD) is fundamental to antifungal stewardship (AFS) but current methods are inefficient and restrictive. Continuous prospective surveillance of IMD in all haematology patients has not been possible because the majority of these infections present as a culture negative pneumonia. Artificial intelligence (AI) targeting medical imaging provides an opportunity to identify fungal pneumonia in real-time in order to present actionable data at the point of care. Here we report preliminary results of real-world prospective validation of deep learning based natural language processing (NLP) of chest imaging reports for real-time detection of IMD in haematology patients that is part of a multicentre clinical trial (Clin Trials.gov NCT03793231). **Objective:** To validate NLP of chest computed tomography reports for prospective surveillance of IMD in all hospitalised haematology patients. As a validation study, NLP alerts are not being fedback to clinicians but rather the machine is being validated against human surveillance. Presented here are preliminary results from one activated site, Alfred Health, a major quaternary Australian transplant centre.

**Methods:** Prospective active manual and electronic surveillance of invasive fungal diseases (IFD) among hospitalised patients under the haematology service commenced on 1 December 2018. Hospitalised patients with IFD were identified by research coordinators through active case finding every week. All suspected IFD cases were reviewed again at 30 days and data collected to determine if they met internationally accepted criteria for IFD diagnosis (EORTC/MSG 2008 definitions). Chest CT reports were prospectively processed by NLP hosted on the hospital server and predictions were compared to IFD cases detected by active surveillance.

**Results:** From 1 December 2018 to 31 April 2018, there were 8 patients with suspected IFD identified. of these, one case was excluded because it did not meet internationally accepted criteria for IFD, resulting in 7 patients with confirmed IFD associated with the following characteristics: all had IMD; all had pulmonary site of infection; 2 cases were probable/proven; 2 possible cases had a positive Aspergillus PCR on bronchoalveolar lavage. There were 6 cases of breakthrough IMD on antifungal prophylaxis involving posaconazole in 4, liposomal amphotericin in 1 and fluconazole in 1 case. of 90 chest CT reports screened by fungalAi-NLP over the study period, 6 of the 7 IFD patients were correctly flagged. The case missed by NLP was an intubated patient with probable pulmonary invasive Aspergillosis associated with isolation of *A. terreus*. The NLP output in this case had probabilities of 37.8% and 41.0% on serial reports which was below the 50% threshold for triggering a positive alert. The radiologist’s conclusion was not definitive in either report. There were 10 (11%) false positive reports. NLP is tuned for fungal pneumonia so may miss extra-pulmonary sites. False positives could be minimised with multimodal analysis combining other sources of data.

**Conclusion:** Real-time, embedded, NLP of chest imaging reports facilitates surveillance of diagnostically challenging IMD for an entire haematology population. NLP utilises diagnostic imaging readily available in hospitals rather than the electronic medical record. Generalisability of NLP will be enhanced by our multicentre clinical trial.

### P317. The case of successful treatment of disseminated cryptococcosis in a patient with Non-Hodgkin’s lymphoma

MelekhinaJ.^1^BorzovaY.^2^IgnatyevaS.^2^DesyatikE.^3^BogomolovaT.^4^ZiuzginI.^5^VasilyevaN.^2^KlimkoN.^1^1Department of Clinical Mycology, Allergology and Immunology, North-Western State Medical University named after I.I. Mechnikov, Saint-Petersburg, Russian Federation2Kaschkin Research Institute of Medical Mycology, Department of Medical Microbiology, North-Western State Medical University named after I.I. Mechnikov, Saint-Petersburg, Russian Federation3Kashkin Research Institute of Medical Mycology, North-Western State Medical University named after I.I.Mechnikov, St. Petersburg, Russian Federation4North-Western State Medical University named after I.I. Mechnikov, St. Petersburg, Russian Federation5FSBI «Petrov Research Institute of Oncology» of the Ministry of Healthcare of the Russian Federation, Saint-Petersburg, Russian Federation

**Case Report: Objectives.** Clinical case of successful treatment of disseminated cryptococcosis in patient with Non-Hodgkin’s lymphoma. **Methods.** The EORTC/MSG 2008 diagnostic criteria were used. **Results.** Patient B., 32-year-old, was hospitalized in a city hospital in June 2016. Non-Hodgkin’s lymphoma was diagnosed. He received 8 cycles of R-CHOP therapy, two cycles of R- DHAP with peripheral blood stem cell transplantation, one cycle of GDP protocol and after achieving partial remission, the patient was consolidated with peripheral blood stem cell transplantation using the BEAM conditioning regime. In September 2017, hematological remission was achieved. In October 2018 an infiltrate 17x12 mm in the right lung S4 was revealed on computed tomography (CT) scan. Surgical removal the lung lesion was performed. Histological examination revealed granulomatous inflammation, and culture was positive with *Cryptococcus neoformans*. Positive “Crypto +” test in blood serum and cerebrospinal fluid was detected and diagnosis disseminated cryptococcosis was established. The patient was treated with amphotericin B 50 mg/day for 14 days, followed by fluconazole 800 mg/day with positive effect. In February 2109 the decrease of infiltration and the reduction of the cavity were detected on chest CT scan. Cryptococcal antigen test was negative in the blood serum and in the cerebrospinal fluid. The total duration of antimycotic therapy was six months. **Conclusion**. Disseminated cryptococcosis is possible in patients with Non-Hodgkin’s lymphoma.

### P318. Comparison of fluconazole and posaconazole based antifungal prophylaxis in patients with newly diagnosed acute myeloid leukemia

KandemirO.^1^TombakA.^2^KoyuncuM. Bakır^3^ŞahinoğluS.^3^TürkerA.^3^HorasanE. Sahin^4^1Infection Dieseases, Mersin University, mersin, Turkey2Hematology, mersin university, MERSIN, Turkey3Mersin University, MERSIN, Turkey4Infection Diseases, mersin university, mersin, Turkey

**Objectives:** Antifungal prophylaxis with posaconazole during remission induction chemotherapy of patients with newly diagnosed acute myeloid leukemia has been shown to decrease invasive fungal infections (IFI) and increase overall survival. However, real-life studies about this issue is not much.

**Methods:** This was a retsopective cohort study incluiding a total of 58 patients with acute myeloid leukemia who were received intensive remission induction therapy. 27 patients received fluconazole prophylaxis between January 2013 and January 2015, 31 patients received posaconazole prohylaxis between February 2015 and February 2017.

**Results:**

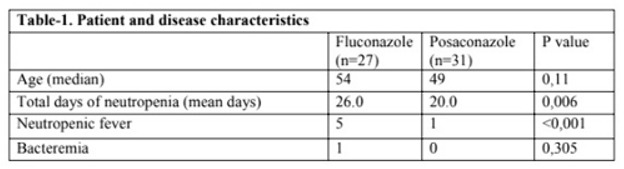

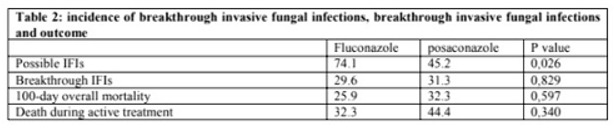





Demographic characteristics of the patients were similar in both groups (Table 1). At day 100, patients receiving posaconazole had a significantly lower incidence of possible IFIs (45.2% vs 74.1%, p = 0,026). There was no significant difference in 100-day overall mortality (32.3% in the posaconazole group vs. 25.9 % in the fluconazole group, p = 0,597), breakthrough IFIs (31.3% in the posaconazole group vs. 29.6 % in the fluconazole group, p = 0,829) and death during active treatment (44.4% in the posaconazole group vs. 32.3% in the fluconazole group, p = 0,340) (Table 2).

**Conclusion:** Posaconazole prophylaxis decreased the incidence of IFIs but did not improve short-term overall survival.

### P319. B-1 cell deficiency decreases the population and function of dendritic cells in murine encephalitozoonosis

PereiraA.^1^SantosD. Langanke Dos^2^Alvares-SaraivaA.^2^HurtadoE.^2^AlvarezJ.^2^LalloM.^2^1Biomedicina, Centro Universitário São Camilo, São Paulo, Brazil2Post-graduation Program In Environmental and Experimental Pathology, Paulista University, São Paulo, Brazil

**Objectives:** Microsporidia are obligate intracellular pathogens belonging to the *kingdom Fungi,* relatively common enteric microorganisms that usually result in self-limited or asymptomatic infections in humans. Although protection against microsporidia is predominantly dependent on the onset of a strong T cell response, dendritic cells (DCs) play a critical role in stimulating T cell response through pattern recognition receptors. Previous studies have suggested that DCs can regulate B cell activity and *Encephalitozoon intestinalis* infection blocks the differentiation of DCs, thus compromising the immune response against the pathogen, characterizing an evasion mechanism. The present study aimed to evaluate the relation between B-1 cells and DCs of *Peyer’s* patches (PPs) in mice infected with *Encephalitozoon cuniculi*, a species of microsporidia.

**Methods:** BALB/c mice, BALB/c Xid mice (deficient in B-1 cells) and Xid+B-1 (B-1 cells adoptive transfer to mice) were infected or not with *E. cuniculi* orally and the same immunosuppressed with cyclophosphamide (Cy) because it is an opportunistic pathogen. After 21 days of the infection, the DCs of PPs were quantified by flow cytometry.

**Results:** Infected mice had a lower population of DCs on PPs than uninfected animals (Figure 1). This result suggests the ability to block DC differentiation for the pathogen *E. cuniculi*. The BALB/c mice, with higher resistance to *E. cuniculi* infection, had the highest DCs populations when compared to the other groups (Figure 1). Xid animals showed the smallest populations of DCs, with the exception of the Xid animals that received B-1 cell transfer. MHC II expression decreased in Xid animals. Cy induced an increase in the DCs population in PPs from uninfected BALB/c mice in relation to the other uninfected groups, indicating an immunomodulatory effect of the drug. 

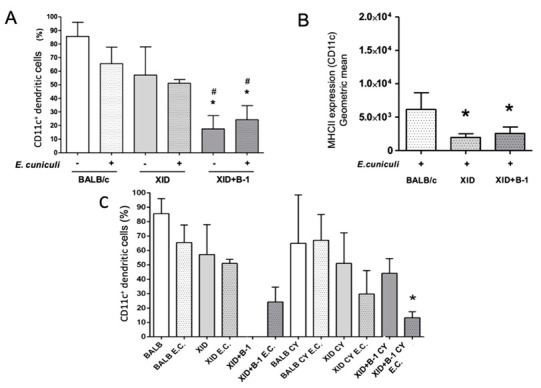



**Figure 1.** Evaluation of dendritic cells present in the Peyer’s Patches from BALB/c, BALB/c XID and XID+B-1 mice inoculated (+) or not (-) with *E. cuniculi*. A) Percentage of CD11c^+^ dendritic cells in non-treated mice. B) Geometric mean of the fluorescence intensity of the MHC II molecule in dendritic cells CD11c^+^. C) Percentage of CD11c^+^ dendritic cells. Two-way ANOVA analysis with Tukey post-test revealed # statistically significant differences and p <0.05*, versus BALB XID and versus XID+B-1, respectively (A). Data were transformed for statistical analysis, analysis of two-way ANOVA with Tukey test post revealed p <0.05* versus BALB c.

**Conclusion:** These data suggest that B-1 cell deficiency compromises the population and the DCs response of PPs in *E. cuniculi* infection.

### P320. rEvaluation of the Wako™ β-D-Glucan assay in Patient Serum for the Diagnosis of Invasive Aspergillosis.

HegartyF.Microbiology, Mater Misericordiae University Hopsital, Dublin, Ireland

**Objectives:** Invasive Aspergillosis is a fungal disease that can carry a high morbidity and mortality, especially in those patients who are immunosuppressed. Early diagnosis of Invasive Aspergillosis (IA) is key to implementing early treatment and improve patient prognosis. Currently in MMUH, serum β-D-Glucan (BDG) is available as an external referral test only (average TAT 109.5 days). Expanding our current repertoire of tests to include BDG, would greatly aid in the early diagnosis of IA. Delay or misdiagnosis of IA can result in drug toxicity due to inappropriate treatment, high medical expenses and mortality. Given the complexity of invasive fungal diseases a number of tests are required for correct diagnosis. Therefore implementation of another test that can quickly and accurately aid in the diagnosis of IA would have great benefits for overall patient care, whilst limiting unnecessary use of antifungal therapy within the hospital. **Aim:** To evaluate the Wako™ β-D-Glucan assay in the laboratory and its’ implications for clinical practice. To assess the contribution this test can make to patient care

**Methods:** Fifty-one random patient samples (serum) were used to complete this pilot study. Samples included ICU, lung transplant and haematology patients. Positive and negative controls were included weekly. The Wako™ β-D-Glucan was evaluated as per the manufacturer’s guidelines

**Results:** The results obtained from the BDG were compared to external referral results. They were also analysed in conjunction with previous microbiological cultures, galactomannan, OLM™ lateral flow device calcofluor/KOH results, histological specimens, radiological imaging and clinical details where applicable. EORTC criteria were used to categorise patients into proven, probable or possible IA. BDG results demonstrated 83% sensitivity and 100% specificity as well as a positive predictive value of 100% and a negative predictive value of 88%.

**Conclusion:** Based on results of this study, Wako™ β-D-Glucan will be introduced in MMUH, for patients with suspected IA, or those severely immune-suppressed.

### P320. rEvaluation of the Wako™ β-D-Glucan assay in Patient Serum for the Diagnosis of Invasive Aspergillosis.

HegartyF.Microbiology, Mater Misericordiae University Hopsital, Dublin, Ireland

**Objectives:** Invasive Aspergillosis is a fungal disease that can carry a high morbidity and mortality, especially in those patients who are immunosuppressed. Early diagnosis of Invasive Aspergillosis (IA) is key to implementing early treatment and improve patient prognosis. Currently in MMUH, serum β-D-Glucan (BDG) is available as an external referral test only (average TAT 109.5 days). Expanding our current repertoire of tests to include BDG, would greatly aid in the early diagnosis of IA. Delay or misdiagnosis of IA can result in drug toxicity due to inappropriate treatment, high medical expenses and mortality. Given the complexity of invasive fungal diseases a number of tests are required for correct diagnosis. Therefore implementation of another test that can quickly and accurately aid in the diagnosis of IA would have great benefits for overall patient care, whilst limiting unnecessary use of antifungal therapy within the hospital. **Aim:** To evaluate the Wako™ β-D-Glucan assay in the laboratory and its’ implications for clinical practice. To assess the contribution this test can make to patient care

**Methods:** Fifty-one random patient samples (serum) were used to complete this pilot study. Samples included ICU, lung transplant and haematology patients. Positive and negative controls were included weekly. The Wako™ β-D-Glucan was evaluated as per the manufacturer’s guidelines

**Results:** The results obtained from the BDG were compared to external referral results. They were also analysed in conjunction with previous microbiological cultures, galactomannan, OLM™ lateral flow device calcofluor/KOH results, histological specimens, radiological imaging and clinical details where applicable. EORTC criteria were used to categorise patients into proven, probable or possible IA. BDG results demonstrated 83% sensitivity and 100% specificity as well as a positive predictive value of 100% and a negative predictive value of 88%.

**Conclusion:** Based on results of this study, Wako™ β-D-Glucan will be introduced in MMUH, for patients with suspected IA, or those severely immune-suppressed.

### P321. Evaluation of OLM™ Lateral Flow Device (LFD) in Bronchoalveolar Lavage for the Diagnosis of Invasive Aspergillosis

HegartyF.Microbiology, Mater Misericordiae University Hopsital, Dublin, Ireland

**Objectives:** Invasive Aspergillosis is a fungal disease that can carry a high morbidity and mortality, especially in those patients who are immunosuppressed. Early diagnosis of Invasive Aspergillosis (IA) is key to implementing early treatment and improve patient prognosis. While respiratory samples may culture *Aspergillus*, it is essential to differentiate between active infection verses colonisation of the lung. This differentiation can often be difficult as well as time consuming. This potential for delay in treatment commencement could contribute to a poor outcome, or if treated incorrectly may expose the patient to unnecessary antifungal therapy. Therefore utilisation of a test that can reliably differentiate between colonisation and active infection would have benefits for patient care. **Aim:** To evaluate the OLM™ LFD in the laboratory and implications for clinical practice. To assess the contribution this test can make to patient care

**Methods:** Twenty random patient samples (bronchoalveolar lavage) were used to complete this pilot study. Nine samples were *Aspergillus* culture positive, fifteen samples were *Aspergillus* culture negative and two samples NEQAS controls; *Aspergillus* sp. and *Trichophyton* sp. culture positive. The OLM™ LFD was evaluated as per the manufacturer’s guidelines, which included the pre-treatment steps as required

**Results:** The results obtained from the LFD analysed in conjunction with previous microbiological cultures, calcofluor/KOH results, histological specimens, radiological imaging and clinical details where applicable. LFD results demonstrated 100% sensitivity and 85% specificity as well as a positive predictive value of 71% and a negative predictive value of 100%. The LFD detected the presence of *Aspergillus* in three samples which was missed in the previously carried out culture and calcofluor/KOH. In this instance, *Aspergillus* infection was suspected from patient scans, which nicely correlated with the LFD result.

**Conclusion:** Based on results of this study, OLM™ LFD will be introduced in MMUH, for patients with suspected IA, or those severely immune-suppressed.

### P322. The first case of invasive candidiasis caused by two pathogens (Candida albicans and Candida glabrata) in a patient with Hodgkin’s lymphoma

ShagdileevaE.^1^ShadrivovaO.^1^ChudinovskyJ.^2^MotalkinaM.^2^ZyuzginI.^2^BogomolovaT.^3^VybornovaI.^3^RyabininI.^3^HohlovaA.^2^ArtemevaA.^2^KlimkoN.^1^1North-West State Medical University named after I.I. Mechnikov, Saint-Petersburg, Russian Federation2N.N. Petrov National Medical Research Centre of Oncology, Saint Petersburg, Russian Federation3Kashkin Research Institute of Medical Mycology, North-Western State Medical University named after I.I.Mechnikov, St. Petersburg, Russian Federation

**Case Report:** Woman with HL, 32 years old, in severe condition was admitted to the Petrov Oncology National Medical Medical Research Centre for cytotoxic chemotherapy on 23.01.2019. Risk factors for IC were central venous catheter (CVC), broad-spectrum antibacterial drugs therapy, prolonged neutropenia, and glucocorticosteroids treatment. The patient didn’t receive antifungal prophylaxis. Clinical manifestation of IC was body temperature > 38 °C, refractory to antibiotic use. Diagnosis of invasive candidiasis was confirmed with blood culture – *C.albicans* with susceptibility to fluconazole and voriconazole *in vitro*. Pathogen identification was determined by MALDI-TOF mass spectrometry. In the first 24 hours after diagnosis antifungal therapy (caspofungin) was used, and CVC was removed. Anaphylactic reaction with angioedema developed after caspofungin administration, and liposomal amphotericin B (7 days), then fluconazole (7 days) and amphotericin B deoxyholate (6 days) were used. Death was caused by septic shock after 20 days of antifungal therapy. On the autopsy IC with lungs, liver, spleen, and skin involvement was found. Culture from the autopsy materials was identified as *C.glabrata* with dose-dependent susceptibility to fluconazole *in vitro.* **Conclusion.** In the treatment of invasive candidiasis in immunocompromised patients, the possibility of several pathogens should be considered.

### P323. Pulmonary aspergillosis in patient with autoimmune hepatitis: the report of unusual case

AntonetsA.^1^KitO.^2^KutsevalovaO.^3^PanovaN.^3^MiroshnichenkoD.^4^PopovyanE.^5^HomenkoO.^6^1Laboratory of Molecular Oncology, Rostov Research Institute of Oncology, Rostov-on-Don, Russian Federation2Rostov Research Institute of Oncology, Rostov-on-Don, Russian Federation3Laboratory of Clinical Microbiology, Rostov Research Institute of Oncology, Rostov-on-Don, Russian Federation4Clinical Pharmacology Department, Rostov Rostov Regional Clinical Hospital №2, Rostov-on-Don, Russian Federation5Pulmonology Department, Rostov Rostov Regional Clinical Hospital №2, Rostov-on-Don, Russian Federation6Hepathology Department, Central City Hospital №1 named N.A. Semashko, Rostov-on-Don, Russian Federation

**Case Report: Objectives:** To present the case of immunocompromised patient who was diagnosed with pulmonary aspergillosis in a short period of time after established diagnosis of autoimmune hepatitis. **Methods:** We examined 69-year-old man who had spitting blood, cough, signs of abscess pneumonia in x-ray examination after 5 weeks of immunosuppressive therapy with decreasing dose of glucocorticoids that was prescribed after the second episode of bilirubinemia when diagnosis of autoimmune hepatitis was established after scrutinizing diagnostic search. Sputum was analyzed with standard and fluorescent microscopy with white calcofluor and cultural method. Standard methods were applied to exclude tuberculosis and aspergillosis. Galactomannan (GM) detection was performed with immunoenzymatic assays XEMA GaIMAg EIA kit (Russia). Positive level of GM accounted for ≥0.59 OD (optical density). **Results:** Cultural methods revealed the presence in sputum *E.coli* (10^7^) and *A.flavus* (10^5^) (pic.1). 

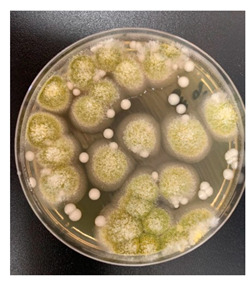



GM level initially was positive in blood (0.73 OD). Antifungal voriconazol therapy was started immediately. One week after starting treatment the second cultural testing of nose crust showed *A.flavus* (10^4^) (pic.2).

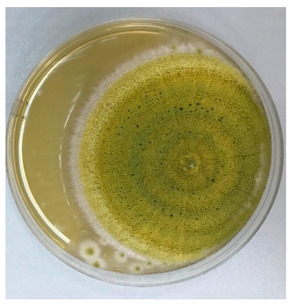



GM was negative in blood and urine (0.41 OD and 0.14 OD respectively), and positive in bronchoalveolar lavage fluide (BALF) accounting for 2.34 OD. Futher cultural testing did not detect *A.flavus.* GM level was positive only in BALF. Spiral computed tomography revealed several aspergillomas, the size of the largest one was 70x50 mm. The patient did not have leukopenia at any time from the first episode of hepatitis, however slight lymphopenia was observed. One month after diagnosis of pulmonary aspergillosis pneumothorax and secondary bacterial complications occurred. Despite this the patient has demonstrated positive clinical effect continuing antifungal and antibiotic therapy (in accordance with the sensitivity of microorganisms) with accompanying therapy by the third month since the diagnosis of aspergilllosis. The remission of autoimmune hepatitis has been observed. **Conclusion.** It should be taken into consideration that immunocompromised patients even without leukopenia/agranulocytosis and short period of immunosuppressive therapy are at risk of development fungal infection. GM testing demonstrates its usefulness for early diagnosis *Aspergillus spp.* A comprehensive approach to diagnosis and alertness of doctors for fungal infection can improve the detection of cases of aspergillosis.

### P324. Challenges in the management of fusariosis in oncology patients: a single-centre series of 27 cases

ItoR.^1^IbrahimK.^1^BonazziP.^1^JuniorJ. De Almeida^2^SantiagoM.^1^SilvaA.C. Da^1^VieiraM.^1^FreireM.^1^SilvaD.^3^MartinsM.^3^SzeszsM.^3^BonfiettiL.^3^PereiraJ.^1^RochaV.^2^DizM.D.P.^1^HoffP.^1^MelhemM.^3^AbdalaE.^1^1Instituto do Cancer do Estado de Sao Paulo, Sao Paulo, Brazil2Hospital da Clinicas da Universidade de Sao Paulo, Sao Paulo, Brazil3Instituto Adolfo Lutz, Sao Paulo, Brazil

**Objectives:** This study aims to describe clinical, epidemiological and laboratorial aspects of fusariosis in cancer patients.

**Methods:** We retrospectively identified patients from an oncology hospital with proven fusariosis, according to the EORTC/MSG modified criteria, since January 2011 to January 2018. Demographic, clinical and laboratorial data from patients were obtained. Mortality rates at 30 and 90 days were evaluated.

**Results:** Twenty-seven cases were identified. Median age was 55 years and 15 were female. Most patients had haematological malignancies (81.5%) and neutropenia (88.9%). All patients received chemotherapy and/or corticosteroids in the last 3 months. Eighteen had disseminated disease, 7 had fungaemia alone and 2 had localized skin infection. Out of 18 patients with disseminated fusariosis, 13 had skin involvement, 8 had pulmonary disease and 5 had sinusitis. Fourteen patients received a lipid formulation of amphotericin B and voriconazole, 10 amphotericin B alone and 3 died before treatment. Species identification was performed in 10 isolates, and all of them were from *F. solani* species complex. Six isolates had susceptibility profile to antifungals and all had MIC ≥8 mg/L to azoles; two isolates had MIC ≥2 mg/L to amphotericin. Mortality rates at 30 and 90 days were 51.9% and 63.0%, respectively. At 90 days, no patients with solid tumor or fungaemia had survived

**Conclusion:** Haematological malignancies, neutropenia, and the use of immunosuppressive therapies were the conditions most associated to fusariosis in cancer patients. Disseminated disease was the most common clinical presentation. Most patients received combined therapy, once the best therapeutic option has not yet been defined, and due to identification of isolates with high MICs to azoles and amphotericin. Mortality rates were higher in solid tumor patients and those with fungaemia alone.

### P325. Epidemiology of Pneumocystis jirovecii pneumonia and (non-)use of prophylaxis in three tertiary care hospitals.

DunbarA.^1^SchauwvliegheA.^2^AlgoeS.^1^HellemondJ. Van^3^ReyndersM.^4^VandecasteeleS.^5^BoelensJ.^6^DepuydtP.^7^RijndersB.^1^1Department of Infectious Diseases, Erasmus University Medical Center Rotterdam, Rotterdam, Netherlands2Department of Hematology, Erasmus Medical Center, Rotterdam, Netherlands3Medical Microbiology, Erasmus Medical Center, Rotterdam, Netherlands4Clinical Microbiology, AZ St-Jan Brugge-Oostende Hospital, Bruges, Belgium5Nephrology and Infectious Diseases, AZ St-Jan Brugge-Oostende Hospital, Bruges, Belgium6Laboratory Medicine, Ghent University Hospital, Ghent, Belgium7Intensive Care, Ghent University Hospital, Ghent, Belgium

**Objectives:** Pneumocystis jirovecii pneumonia (PCP) is caused by the fungus *Pneumocystis jirovecii (*PJ) and is almost exclusively diagnosed in severely immunocompromised patients. While in the recent past the vast majority of PCP-cases were diagnosed in patients with HIV, this is no longer the case in resource-rich countries with unrestricted access to HIV care. The benefit of prophylaxis against PJ has been convincingly demonstrated for patients with HIV and a CD4 T-cell count <200 cells/mm but the benefit of prophylaxis is less well established for other patient groups. This study aimed to describe the epidemiology of PJP in recent years and assess how many patients with PCP did or did not receive appropriate prophylaxis in the months preceding the infection.

**Methods:** A multicenter retrospective study was performed in 3 Belgian and Dutch tertiary care centers: Erasmus MC, University Medical Center in Rotterdam, AZ Sint-Jan in Bruges and UZ Gent in Ghent. A list of patients that underwent broncho-alveolar lavage sampling for PCP testing with an in-house PJ PCR was retrieved from the 3 microbiology labs. An in-house PJ PCR was used in each of the centers with a cycle threshold (Ct) value of <29 (Rotterdam and Bruges) or <31 (Ghent) considered a ***probable*** PCP. For patients with a positive PJ PCR but above this threshold a predefined case definition was used to classify patients as follows: a ***possible*** case of PCP had to fulfill all 3 of the following criteria; 1. PCR Ct value <35 and 2. Clinico-radiological diagnosis compatible with PCP and 3. Patient died or patient received PCP therapy and survived. Patient files from all PCR+ patients were reviewed to determine whether the patient fulfilled the PCP case definition and if antimicrobial prophylaxis had been provided prior to PCP. Disease specific guidelines (NIH AIDS, ECIL, ASOT, local hospital guidelines) were used to evaluate if prophylaxis was indicated. Fisher’s exact test, t-test or Mann-Whitney U tests were used as appropriate.

**Results:** From 2012 to 2018, 482 patients with positive PCR PJ of respiratory samples were reviewed. 278 were excluded mostly because CT value was>35 (*n* = 247). Other reasons were age<18, insufficient clinical data available, other respiratory sample than BAL. of the remaining 204, 153 had a probable (*n* = 90) or possible (*n* = 63) PCP. The remaining 51 were considered colonized (Table 1). PCP patients were HIV negative in 74%. Only 11 (7%) of PCP cases had received prophylaxis, despite that 133 (87%) had an indication for prophylaxis according to guidelines.

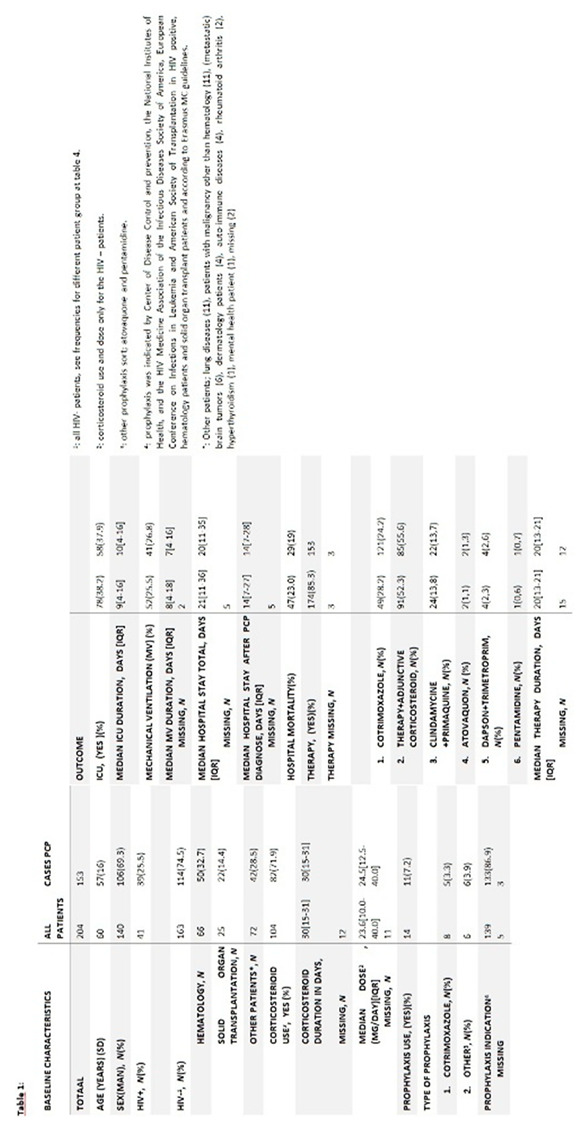



**Conclusion:** The vast majority of patients diagnosed with PCP had not received prophylaxis and therefore the infection could be considered at least partially preventable. In regions where HIV testing and treatment is available without restrictions, PCP is mainly diagnosed in non-HIV immunocompromised patients. Strategies to improve awareness of and compliance with antimicrobial prophylaxis guidelines in immunocompromised patients are urgently needed.

### P326. Prospective evaluation of Mucorales DNA qPCR detection in serum for early diagnosis of mucormycosis: First results from the ModiMucor study

MillonL.^1^CaillotD.^2^BerceanuA.^3^Gbaguidi-HaoreH.^4^Letscher-BruV.^5^DalleF.^6^DenisB.^7^BretagneS.^8^AlanioA.^9^BoutoilleD.^10^MorioF.^11^LanternierF.^12^BougnouxM.-E.^13^BotterelF.^14^ChouakiT.^15^CharbonnierA.^16^AderF.^17^DupontD.^18^RocchiS.^19^SchererE.^19^HerbrechtR.^20^1Parasitology - Mycology - Umr6249 Cnrs Chrono-environnement, University Hospital Besançon, Besançon, France2Clinical Haematology, University Hospital of Dijon, Dijon, France3Hematology, University Hospital Jean Minjoz, BESANCON, France4Infectious Risk Control, University Hospital Jean Minjoz, BESANCON, France5Parasitology-mycology, University Hospital of Strasbourg, Strasbourg, France6Parasitology-mycology, University Hospital Dijon, Dijon, France7Tropical and Infectious Diseases Department, Lariboisière Saint-Louis Fernand Widal Hospital, Assistance Publique-Hôpitaux de Paris (AP-HP), PARIS, France8Mycology, Saint Louis hospital, Paris, France9Paris-diderot, Sorbonne Paris Cité University, Institut Pasteur, Molecular Mycology Unit, Cnrs Cmr2000, Parasitology-Mycology Laboratory, Lariboisière Saint-Louis Fernand Widal Hospitals, Assistance Publique-Hôpitaux de Paris, Paris, France10Tropical Infectious Diseases, University Hospital of Nantes, Nantes, France11Ea1155 Iicimed, Nantes University Hospital, NANTES, France12Infectious Diseases, Necker hospital, Paris, France13Parasitology Mycology, APHP Hôpital Necker-Enfants-Malades, Paris, France14Parasitologie-mycologie, CHU Henri MONDOR, Créteil, France15Parasitology-mycology, University Hospital of Amiens, Amiens, France16Haematology, University Hospital of Amiens, Amiens, France17Infectious Diseases, University hopital of Lyon, Lyon, France18Parasitology and Medical Mycology / Umr 5292, Université Lyon 1 / Hospices Civils de Lyon, Lyon, France19Mycology - Umr6249 Cnrs Chrono-environnement, University Hospital Besançon, Besançon, France20Hematology, CHU de Strasbourg, Strasbourg, France

**Objectives:** Early initiation of specific treatment is essential to improve prognosis of mucormycosis. Several retrospective studies have reported that qPCR detection of Mucorales DNA in serum could anticipate the diagnosis of mucormycosis by an average of 8 days in hematological patients and in critically ill burn patients^1-4^. Modimucor is a French prospective multicentre study (9 university hospitals) that aims to assess the performance (sensitivity, specificity, positive and negative predictive values and likelihood ratios) of the circulating DNA detection test for the diagnosis of mucormycosis in comparison to CT scan imaging, histopathology, microscopy and culture.

**Methods:** Patients with risk factors for invasive mold infection (IMI) such as patients with hematological diseases, burns diabetes, solid organ transplantation, trauma, or diabetes, and patients diagnosed with mucormycosis were enrolled prospectively. Serum samples were collected twice-a-week, and Mucorales PCR were performed at the time of serum sampling, in each center, as previously described^1^. Clinical data and biological data were collected up to 6 months after enrollment. An interlaboratory quality control was done to ensure uniformity of the assays. Data analysis was performed using STATA 14 software.

**Results:** Were included 250 patients with suspicion of invasive fungal infection from January 2015 to June 2017 (Strasbourg *n* = 156; Dijon *n* = 39; Besançon *n* = 21; APHP Saint Louis *n* = 10; APHP Necker *n* = 9; Nantes *n* = 9; APHP Mondor *n* = 3; Amiens *n* = 2; Lyon *n* = 1). Classification of patients according to EORTC/MSG criteria was as follows: 47 probable (*n* = 13) or proven (*n* = 34) mucormycoses, 64 probable or proven aspergilloses, 5 other probable or proven IMI (fusariosis *n* = 2; scedosporiosis *n* = 2; histoplasmosis *n* = 1). A very low interlaboratory variation of the Mucorales PCR results was observed (SD = 1,8 cycles [range 1.3; 2.9]). At least one serum with positive Mucorales PCR was found for 35/47 patients with proven or probable mucormycosis. Among the 12 PCR-negative patients, 4 were given liposomal amphotericin B for more than 10 days at the date of serum sampling, and 3 patients had only a positive direct examination, without positive culture. Twenty-two patients with possible IMI had at least one positive Mucorales PCR. Sensitivity, specificity, positive and negative predictive values were 74.5% (35/47), 89.2% (181/203), 61.4% (35/57) and 93.8% (181/193), respectively. In addition, positive and negative likelihood ratios were 6.8 and 0.28, respectively. The first PCR-positive sample was observed in average 6.5 days [range 0-22; median = 4] before the sampling date of the first mycological positive specimen, and 7 days [range 0-44; median = 4] before the first imaging criteria.

**Conclusion:** This prospective study confirms the good performance of serum Mucorales PCR for early diagnosis of mucormycosis. This assay with an acceptable time frame and a reasonable cost helps to anticipate the diagnosis of mucormycosis and is becoming essential in the clinical diagnosis of mucormycosis. 1.Millon L. Clin Infect Dis, 2013; 2.Millon L. Clin Microbiol Infect 2016; 3.Caillot D. Open Forum Infect Dis, 2016; 4.Legrand M. Clin Infect Dis 2016.

### P327. Cryptococcal Infections among Human Immunodeficiency Virus /Acqiiref Immune Deficiency Syndrome (HIV/AIDS) Patients Accessing HAART Treatment in Ibadan

AdebiyiI.^1^,^2^OgunleyeV.^2^1Department of Medical Microbiology, University College Hospital, Ibadan, Ibadan, Nigeria2Department of Medical Microbiology, University College Hospital, Ibadan, Nigeria

**Objectives:** Cryptococcosis occurs in man and animals in almost all the regions of the world. People with AIDS are particularly vulnerable to this infection. In particular, a higher mortality due to the infection is said to be associated with a CD4+ count less than 200cells/μL. Although Nigeria has the second highest number of people living with HIV globally, there is paucity of data on the prevalence of cryptococcal infections among this people. This study aimed to determine the burden of cryptococcal infections among HIV/AIDS patients accessing highly active antiretroviral therapy (HAART) treatment in Ibadan as well as determine the CD4+ count associated with cryptococcal infections in this locality.

**Methods:** Adult male and female HIV/AIDS patients accessing HAART treatment at the President’s Emergency Plan for AIDS Relief (PEPFAR) clinic in Ibadan with CD4+ count less than 200cells/μL were enrolled into the study. Other HIV/AIDS patients with CD4+ count higher than 200cells/μL but who had a high index of cryptococcal meningitis were screened at the attending physician’s request. A cryptococcal antigen test was performed on blood samples from patients with CD4+ count less than 200cells/μL and CSF samples from patients referred by the attending physician. The CSF samples were also cultured on Sabouraud Dextrose Agar culture media to isolate the organism.

**Results:** 200 HIV/AIDS patients were enrolled in the study. The mean CD4+ count was 145.2cells/μL and 49% of participants were newly diagnosed and had not commenced HAART. The overall prevalence of cryptococcosis was 2%. All positive samples were from patients referred by the attending physician. All positive samples were from patients with CD4+ higher than 200cells/μL. 100% of patients with cryptococcosis were already receiving HAART.

**Conclusion:** Cryptococcosis was found in HIV/AIDS patients with CD4+ count higher than 200cells/μL. Hence timely and appropriate cryptococcal screening in HIV/AIDS patients could help reduce cases of and mortality due to cryptococcal infection and also guide treatment needs. Cryptococcal screening should always be implemented for HIV/AIDS patients with low CD4+ counts regardless of symptoms or receipt of HAART to identify asymptomatic infected patients. It is also important to promptly screen HIV/AIDS patients with CD4+ count higher than 200cells/μL with a high index of cryptococcal meningitis.

### P328. Disseminated phaeohyphomycosis associated to Tuberculosis in a pregnant woman

MhamdiG.^1^KilaniM.^1^AbdelmalekR.^1^BerricheA.^1^KilaniB.^1^AmmariL.^1^BenaissaH. Tiouiri^1^KallelA.^2^KallelK.^2^ChellyI.^3^HaouetS.^3^BoukribaS.^4^MizouniH.^4^1Department of Infectious Diseases, The Rabta hospital, Tunis, Tunisia2Department of Parasitology-mycology, The Rabta hospital, Tunis, Tunisia3Department of Anatomopathology, The Rabta hospital, Tunis, Tunisia4Department of Radiology, The Rabta hospital, Tunis, Tunisia

**Case Report:** Introduction: The phaeohyphomycosis are superficial or deep mycosis that occur due to pigmented frequently opportunistic fungus. We are reporting a case of co-infection, deep pheaohyphomycosis and tuberculosis in a 7 months pregnant patient unknowingly immunosupressed. Observation: The patient is Mrs Y.S. 24 years old, admitted for convulsions. The diagnosis of cerebral and lymph node tuberculosis was held because of right hemi-body convulsions, a positive quantiferon, an evocative lymph node aspiration cytology and the presence of tuberculomas on the cerebral MRI. The patient received a quadruple antitubercular treatment. In two months, there was recurrence of the seizures and persistance of a left abscessed cerebral lesion with an important edema contrasting with the disappearance of a big number of tuberculomas in MRI control. The examination found a discreet right mowing, no meningeal syndrome and a firm cervical lymph node of 1,5 cm. The HIV serology was negative. We have maintained the antitubercular treatment and associated a corticosteroid therapy. A complement of surgical exeresis was realised for treatment and diagnosis purposes. She developed two cervical renitent lymph nodes and new seizures. The CT Scan identified a numerous parenchymal pulmonary nodes some of which are excavated, many necrotic cervical lymph nodes and the reappearance of the cerebral lesion at surgical site. The lymph node puncture aspirated greyish pus containing mycelian filaments. The molecular biology identified *Arthrocladium*. The anatomopathology study of cerebral abscess confirmed both tubercular and mycosic co-infection. We have associated Amphotericin B and Itraconazole. The evolution was slowly favorable. Conclusion: phaeohyphomycosis is a rare pathology with a difficult diagnosis. The prognosis is dark for the systematic forms. The treatment is medico-surgical.

### P329. Mechanisms of Ibrutinib-dependent susceptibility to invasive aspergillosis

Armstrong-JamesD.^1^BercussonA.^1^GonzalesL. Herta^1^WarrisA.^2^1Nhli, Imperial College London, London, United Kingdom2Mrc Centre For Medical Mycology, University of Aberdeen, Aberdeen, United Kingdom

**Objectives:** Patients receiving Ibrutinib, a small molecule inhibitor of Bruton’s tyrosine kinase (Btk), are at increased risk of invasive and disseminated aspergillosis. Given the increasing use of this drug as front-line treatment for haematological malignancies, better understanding of the mechanistic basis for this risk is urgently required. Whilst Btk is recognised as an important tyrosine kinase in B cell receptor signalling pathways, we and others have shown an important role for Btk in macrophage-based phagocytic responses to *Aspergillus fumigatus* and *Candida albicans*. Furthermore, Ibrutinib is also known to inhibit other TEC kinases such as TEC, which is important for macrophage responses to *Candida albicans*. Our objective was to characterise the impact of Ibrutinib on macrophage behaviour during *A. fumigatus* exposure.

**Methods:** Human monocyte-derived and alveolar macrophages, or murine J774A.1 macrophages, [AB1] were infected with *A. fumigatus* after treatment with Ibrutinib and the impact on phagocytosis, cell signalling, fungal killing and cell death responses assayed using a combination of imaging, western blotting and cell death kinetic assays. Selected findings were confirmed to be Btk-specific by siRNA.

**Results:** We show that Ibrutinib is a potent inhibitor of both NFAT and NFκB responses in human macrophages during infection with *A. fumigatus*. We show that *A. fumigatus* induces human macrophage Btk phosphorylation and that Btk depletion impairs NFAT and NFκB responses in human macrophages. Furthermore, Ibrutinib strongly inhibited macrophage necrosis in response to infection. Our findings in a human in vitro model suggest Btk involvement in a TLR9-dependent endosomally driven pathway in accordance with previous findings in our murine model. How this mediates fungal dissemination is currently unknown.

**Conclusion:** These observations suggest that defects in macrophage Btk signalling contribute to susceptibility to pulmonary aspergillosis. We are currently developing in vivo models to further study the effects of ibrutinib on outcomes from pulmonary aspergillosis.

### P331. Ten year experience with fungal Infections in Acute Myeloid Leukemia in the Posaconazole Era

MariniC.ChorãoP.BergantimR.AguiarE. ValePiresJ. CancelaTrigoF.Centro Hospitalar Sao Joao, Porto, Portugal

**Objectives:** Posaconazole prophylaxis in patients with acute myeloid leukaemia (AML) changed the paradigm of fungal infections (FI). AIMS: Description of FI and analysis of factors that might predispose to this type of infection in non-allotransplant AML patients in the posaconazole era.

**Methods:** Retrospective analysis of 217 consecutive non-M3 AML patients diagnosed between June 2008 and June 2018 in a tertiary center and treated with intensive chemotherapy regimens, including autologous transplantation when performed in these patients. Posaconazole prophylaxis was introduced since January 2009. Multivariate regression analysis was performed to identify characteristics related to FI.

**Results:** Median age at diagnosis was 51 years [19;71], 52% were females and all patients had an age-adjusted Charlson score ≤3. Genetic risk group according to the European Leukemia Net 2017 classification was favourable in 24%, intermediate in 43% and adverse in 22%. Twelve percent of patients (*n* = 26) had secondary AML. Patients were included from completed remission-induction intensive therapy up to transplant referral, the latter which occurred in 48% of patients (*n* = 104), with a global median follow-up of 7 months [1;132]. Autologous transplantation was performed in 15% of patients (*n* = 34). Fluconazole prophylaxis was administered in 7 patients, all others received posaconazole. There were 20 patients that had episodes of prophylaxis interruption, 14 during auto-transplantation, 3 due to toxicity and 3 out of our protocol. During treatment, patients with no relapse presented a global median time of neutropenia of 69 days [26;421] whereas relapsed patients had a median of 104 days [30;356]. Ten percent of patients had >2 episodes of prolonged neutropenia (defined as ≥28 consecutive days of <500 neutrophils/microliter). Fungal infections were identified in 41 patients (19%), 3 of which were in the non-posaconazole group, and classified according to EORTC criteria, with 13 proven, 17 probable and 10 possible infections. In 32% of patients FI occurred during remission-induction (*n* = 13) of which 3 were diagnosed in the first week of neutropenia, 19% during consolidation treatment in first remission and 49% during relapse treatment (*n* = 20). of the confirmed infections, 5 were aspergillosis, 3 candidemia, 2 by *Fusarium* and 1 mucormycosis. Probable infections had pulmonary origin with no microbiology isolate or serologic positivity but identified by typical radiologic evidence; of these, broncho-alveolar lavage and/or pulmonary biopsy was performed in 6 patients (35%) with negative results. For possible infections, antifungal therapy was initiated in a pre-emptive manner for high risk patients with persistent fever despite large spectrum antibiotics. In a multivariate analysis for the posaconazole group, patients with FI had a higher odds of having relapsed AML, refractory AML at first approach and >2 episodes of prolonged neutropenia during treatment (p<0.01, p = 0.01 and p = 0.012, respectively). There was no mortality related to FI in this cohort of patients.

**Conclusion:** Despite posaconazole prophylaxis, FI is still prevalent in AML patients. Infection during salvage treatment for refractory or relapsed disease and >2 previous periods of neutropenia exceeding 4 weeks should raise awareness for an increased risk of FI.

### P332. Neuromeningeal cryptococcosis: experience of a department of infectious diseases

MhamdiG.^1^BachrouchS.^1^BerricheA.^1^AmmariL.^1^AbdelmalekR.^1^KilaniB.^1^BenaissaH. Tiouiri^1^KallelA.^2^KallelK.^2^1Department of Infectious Diseases, The Rabta hospital, Tunis, Tunisia2Department of Parasitology-mycology, The Rabta hospital, Tunis, Tunisia

**Objectives: Introduction:** Neuromeningeal cryptococcosis (NMC) is a serious infection. Frequently described during HIV infection, it can nevertheless occur on any field of cellular immunosuppression and even in the immunocompetent. **Aim:** describe the clinical, para-clinical, therapeutic and evolutionary aspects of NMC cases.

**Methods:** A descriptive retrospective study carried out at the infectious diseases’ department of La Rabta Hospital over 25 years, between January 1993 and December 2018, including all patients hospitalized for NMC.

**Results:** We collected 30 cases of (NMC). The median age is 34 years old [18-73] with a sex ratio of 1.7. 76 % of patients were HIV-positive. The average CD4 count was 58.8 cells / μl. Among HIV-negative patients, 4 patients were followed for lymphoma, one patient was diabetic, one had Histiocytosis X and only 1 was immunocompetent.

The median time of treatment was 6 days [1-75]. The main symptoms were fever (73%) and headache (93%). Seizures and signs of localization were present in 1/3 of the cases. A Physical meningeal syndrome was noted in 63% of the cases. Cerebrospinal fluid (CSF) analysis revealed a highly variable cytology [0-3000], hypoglycorrhachia (69%) and hyperproteinorachia (83%). The dosage of cryptococcal antigen in the CSF ranged between 1/64 and 1/25000. The treatment of attack was based primarily on Amphotericin B. The mortality was 56%.

**Conclusion:** NMC is a life-threatening fungal infection. Only a high level of clinical suspicion, early diagnosis and appropriate medical care can improve the prognosis.

### P333. Deep cutaneous mycoses in renal transplant patients: diagnostic and therapeutic challenges

BertinC.^1^SitterléE.^1^ScemlaA.^2^FraitagS.^3^DellièreS.^1^GueganS.^4^Leclerc-MercierS.^5^RouzaudC.^4^LanternierF.^6^BougnouxM.-E.^1^,^7^1Parasitology Mycology, APHP Hôpital Necker-Enfants-Malades, Paris, France2Transplantation, Hopital Necker, Paris, France3Anatomopathology, Hopital Necker, Paris, France4Infectious Diseases, Hopital Necker, Paris, France5Hopital Necker, Paris, France6Infectious Diseases, Necker hospital, Paris, France7Laboratoire De Parasitologie Mycologie, APHP Hôpital Necker-Enfants-Malades, Paris, France

**Objectives:** Deep cutaneous mycoses are infections extending throughout the dermis and hypodermis, often occurring after inoculation of pathogen fungi. In kidney transplant, deep cutaneous mycoses count for up to 5% of all documented infections. The aim of this study was to describe the clinical features, histopathological findings and mycological characteristics of deep cutaneous fungal infections in renal transplant patients, as well their management and outcome.

**Methods:** We performed a retrospective review of cases of proven deep cutaneous mycosis (histologically extending to dermis and/or hypodermis with mycological documentation) occurring among kidney transplant recipients in our institution since 2011. We studied the type of transplant, immunosuppression, histopathology, treatment with prospective follow-up of cutaneous lesions

**Results:** Nineteen kidney transplant patients were included; the average age was 41 (range, 28 to 91). The immunosuppressive regimens at the time of diagnosis included corticosteroids (*n* = 19) and anti calcineurin (tacrolimus (*n* = 18), cyclosporin (*n* = 1)). In 10 and 6 patients a treatment by mycophenolate mofetil and azathioprine was associated, respectively. Co-morbidities were frequent: diabetes mellitus (*n* = 12), cancer (*n* = 2), HIV (*n* = 1). In 70% of cases a skin cancer was initially suspected (epidermoid carcinoma *n* = 5, kaposi sarcoma *n* = 6). The skin lesions were solitary in ten patients, mainly nodular (84%) and affected the extremities (95%), without fever or fatigue in the majority of cases (63%). Histopathologic examination of skin biopsies showed pseudohyphae and/or irregularly branched hyphae (*n* = 16/16, 100%), a granulomatous dermatitis (*n* = 6/16, 37%), and an inflammatory infiltrate (*n* = 8/16, 50%). Mycological examination of the biopsy showed the presence of hyphae on direct examination in 84% of the cases (*n* = 16/19). Identification was obtained by culture and / or molecular biology. Eleven different fungal species were observed, including dematiaceous molds (*n* = 13), hyphomycetes (*n* = 4), dermatophyte (*n* = 1), and Mucorale (*n* = 1). No dissemination to other organs was observed. The detection of the fungal (1-3) beta-D-glucan antigen in serum was positive in 100% of the cases (*n* = 17/17). Fifteen patients received antifungal treatment with azoles (posaconazole *n* = 10, voriconazole *n* = 3), or liposomal amphotercin B (*n* = 2) for a mean duration of 3 months, and surgery was associated in 11 patients. Side effects were common (64%) with mainly tacrolimus overdosage (21%) or acute renal failure (50%).

**Conclusion:** This work highlights the great diversity of fungal species responsible for deep cutaneous mycoses in renal transplant patients. The mycological documentation is necessary to adapt the antifungal treatment according to the sensitivity to antifungal agents of the isolated species. Drug interactions between antifungals and immunosuppressive treatment require appropriate monitoring.

### P336. Neutrophil profiling in response to the human fungal pathogen, Candida albicans, in transplant recipients

MansourM.Dept of Medicine, Division of Infectious Diseases, Massachusetts General Hospital, Boston, United States of America

**Objectives:** Neutrophils are innate immune cells and the first line of defense against a variety of invading pathogens including yeast and mold species. While low circulating neutrophils numbers (neutropenia) is a clear risk factor for developing invasive fungal infections (IFI), there is lacking evidence if neutrophil dysfunction contributes to infectious risk. In this study, we profiled circulating neutrophil anti-*Candida* fungicidal activity from high-risk patients at our tertiary care hospital, specifically transplant recipients compared to healthy controls.

**Methods:** Solid-organ (SOT) and stem cell transplant (SCT) patients were identified and consented from September 2018 until April 2019. Healthy control patients (HC) were identified at primary care clinics. EDTA-anticoagulated peripheral blood was obtained from healthy and transplant patients 2-4 months post-transplant. Neutrophils were isolated by negative selection. *C. albicans* was co-incubated for 2 hours with and without human neutrophils at a multiplicity of infection (MOI) of 1, 5 and 10. Following neutrophil cell lysis, percent remaining live *Candida* was measured using a viability dye. In addition, growth inhibition of yeast by neutrophil swarming to *C. albicans* spotted onto glass slide arrays was also assessed by live-cell imaging.

**Results:** 22 SOT (15 kidneys, 7 livers), 20 SCT (allograft) and 22 HC were enrolled. Neutrophils from SCT and SOT had lower *C. albicans* killing percentages compared to HC at MOI 10 (HC: 47%, SOT: 29%, SCT 24% *p* = 0.0041); MOI 5 (HC: 72%, SOT: 35%, SCT 38% *p* < 0.0001) and MOI 1 (HC: 91%, SOT: 48%, SCT: 45% *p* < 0.0001). Neutrophil swarming and fungal control of *C. albicans* spots were significantly inhibited by neutrophils from SCT when compared to SOT and controls (*p* <0.0001). Analysis of medications, including tyrosine kinase inhibitor (TKI) use, did not demonstrate significant differences in a specific drug class when patient groups are compared (SCT versus SOT).

**Conclusion:** This study indicates that neutrophils from patients with SOT and SCT have a decreased ability to kill *C. albicans,* despite normal circulating numbers. There were no medications or laboratory values that predicted a functional neutrophil outcome. These data strongly support the use of functional neutrophil profiling to risk stratify those individuals at higher risk for invasive fungal infections, although additional studies are required to determine if PMN dysfunction results in increased clinical infection. These findings will inform us of potential therapeutic interventions for restoring immune function and preventing invasive fungal infections in high-risk transplant patients.

### P340. rInvasive candidiasis in neonatal patients in Saint-Petersburg, Russia

ShagdileevaE.^1^FaizullinaR.^2^BelovaO.^3^Voronovi_HS.^3^KuznetsovaT.^4^RubinG.^3^VorobevaS.^4^BogomolovaT.^2^VybornovaI.^2^KlimkoN.^1^1North-West State Medical University named after I.I. Mechnikov, Saint-Petersburg, Russian Federation2North-Western State Medical University named after I.I. Mechnikov, St. Petersburg, Russian Federation3City Children’s Hospital № 17, St. Petersburg, Russian Federation4City Children’s Hospital № 1, St. Petersburg, Russian Federation

**Objectives:** To investigate the etiology, risk factors, clinical manifestations, clinical features, and results of treatment of invasive candidiasis (IC) in neonatal patients in St. Petersburg, Russia.

**Methods:** A prospective study was conducted from January 2015 to February 2019. We evaluated neonates with clinical manifestations of infection and a confirmed diagnosis of IC. The EORTC/MSG 2008 criteria were used for IC diagnosis and evaluation of the effectiveness of therapy.

**Results:** “Proven” IC was detected in 29 neonates patients, girls – 62%. Premature neonates were 86%, twins – 21%. Median birth weight was 1280 g (560 – 4000 g), median gestational age at birth – 30 weeks (23 – 40). IC developed on the 5 – 88 days of hospitalization (median – 20). The main underlying conditions: premature neonates – 86%, intraamniotic infections – 72%, HIV – 7%. Broad-spectrum antibacterial drugs were prescribed for 100% patients, mechanical ventilation – 97%, central venous catheter (CVC) use – 93% (median time – 9,5 days, 4 -25 days), parenteral nutrition – 69 %. Antifungal prophylaxis with fluconazole received 93% patients. The main clinical variants of IC were candidemia – 97%, chorioretinitis – 6%, meningitis – 3%, and hepatitis – 3%. *Candida* spp. were isolated from CVC in 86% of patients, blood – 41%, cerebrospinal fluid – 3%. The pathogens of IC were *C.albicans –* 38%, *C.parapsilosis* – 32%, *C.famata* – 14%, *C.pelliculosa* – 7%, *C.tropicalis* – 3%, *C.guiliermondii* – 3%, *Candida* sp. – 3%. In vitro to voriconazole were resistant 3% *Candida* spp., fluconazole – 3%, dose-dependent susceptibility to fluconazole – 7%. In the first 24 hours after diagnosis antifungal treatment was used in 100% patients: fluconazole (100%), micafungin (38%), amphotericin B deoxycholate (14%), and voriconazole (14%). The median duration of treatment was 20 days (1 - 54). The overall 30-day survival rate was 86%.

**Conclusion:** IC developed in premature neonates (86%), the median gestational age at birth was 30 weeks, the median birth body weight - 1280 g. Risk factors for IC were broad-spectrum antibacterial drugs therapy (100%), mechanical ventilation (97%), and central venous catheter (93%). Main etiology agents were *C. albicans –* 38% and *C. parapsilosis* (32%). The main clinical variant of IC was candidemia (97%). Antifungal treatment was used in 100% patients, fluconazole – 100%, micafungin – 38%. The overall 30-day survival rate was 86%.

### P341. AIRE and STAT1 gene mutations in Children with Chronic Mucocutaneous Candidiasis in Saint-Petersburg, Russia

KozlovaO.^1^IsaevaN.^1^FrolovaE.^1^BogomolovaT.^1^SuspitsinE.^2^GabrusskaiaT.^3^KlimkoN.^1^1Department of Clinical Mycology, Allergology and Immunology, North-Western State Medical University n.a. I.I. Mechnikov, Saint-Petersburg, Russian Federation2St. Petersburg State Pediatric Medical University, Saint-Petersburg, Russian Federation3Saint-Petersburg State Pediatric Medical University, Saint-Petersburg, Russian Federation

**Objectives:** Publications on AIRE and STAT1 gene mutations in children with chronic mucocutaneous candidiasis (CMC) are limited.

**Methods:** Molecular genetic studies were performed with multigenic targeted sequencing (MiSeq, Illumina, USA).

**Results:** In prospective single center study included 11 patients with CMC. Median age was – 7.5 y, range - 6 to 13 y, males - 92%. Molecular genetic study has shown heterozygous mutation in the STAT1 gene in 4 patients and mutations in autoimmune regulator (AIRE) in 7 patients. CMC started between 2 months - 4 years (median – 2 y). Sites of CMC were oral mucosa – 100 %, skin – 64%, esophagus - 36%, and nails – 36%. *Candida albicans* was isolated in all patients. Recurring CMC was diagnosed more than 4 times per year in all patients. Endocrine disorders were present in patients with AIRE syndrome: primary adrenal insufficiency – 71%, hypoparathyroidism - 71%, autoimmune thyroiditis with hypothyroidism - 29%; autoimmune disorders: autoimmune gastritis – 71%, alopecia – 29%, and hemolytic anemia – 14%. Patients with impaired STAT1 did not have endocrine disorders and autoimmune gastritis was in one patient (29%).

**Conclusion:** In 100% patients CMC with mutations in autoimmune regulator AIRE endocrine system disorders and autoimmune diseases. STAT1 gene autoimmune diseases diagnosed in 29% patients. This work was supported by Russian Scientific Fund grant 18-15-00256

### P342. Pneumocystis jirovecii pneumonia associated with severe viral infection in children

KozlovaO.^1^MironukE.^2^KapilovV.^2^UstinovaA.^2^MuratovP.^2^SerikovaE.^2^PinevskaiaM.^2^IgnatyevaS.^1^KlimkoN.^1^1Department of Clinical Mycology, Allergology and Immunology, North-Western State Medical University n.a. I.I. Mechnikov, Saint-Petersburg, Russian Federation2Children’s City Multidisciplinary Clinical Center for High Medical Technologies. K.A. Rauffusa, Saint-Petersburg, Russian Federation

**Objectives:** As an opportunistic infection, *Pneumocystis jirovecii* pneumonia (PjP) is a severe and life-threatening complication in immunocompromised patients. Publications on PjP associated with severe viral infection in children are limited.

**Methods:** We report the 2 cases of PJP associated with severe viral infection in children.

**Results:** A 13-year-old girl admitted in children’s hospital with interstitial pneumonia, sepsis, and respiratory and cardiovascular failure. 7 days before, she presented with the history of fever and dry cough. The patient is diagnosed with influenza. She was in acute respiratory distress and required immediate intubation and transfer to the intensive care unit. On examination, she was hypoxic with SaO2 70-82 % despite 100% flow oxygen. Despite empiric intravenous therapy with ceftriaxone, amikacin, meropenem and vancomycin, she remained hypoxic. Lymphopenia (1,0 × 10^9^/l) was in blood analysis. HIV testing was negative. The chest CT scan showed bilateral interstitial infiltrates. MONOFLUO™ KIT *P. jirovecii* test and PCR test for *P. jirovecii* were positive in BAL. Intravenous trimethoprim–sulfamethoxazole (TMP–SMX) (12 mg/kg TMP) and prednisone 0.5 mg/kg twice daily were initiated, which resulted in improvement in respiratory status and weaning of respiratory support over 15 days (SatO2 - 88-92%, PO2 - 46-52%). A 16-year-old boy was diagnosed with chronic bronchiolitis, secondary arterial hypertension, and secondary asymmetric hypertrophic cardiomyopathy. 3 month before, he presented with the history of high fever and maculopapular rashes, spots Koplika and cough. Vaccination details were not available. The patient received several courses of antibiotic therapy and prednisone for a long time at a dose of 2.5-7.5 mg/kg without positive dynamics. Due to the development of severe respiratory failure, the patient was hospitalized. The patient had dyspnea of mixed character (SpO2 - 70-80%). The patient was immunocompetent (CD4 = 2,130*10^9^/l) HIV testing was negative. The serology for measles was positive. The CT showed bilateral interstitial infiltrates. MONOFLUO™ KIT *P. jirovecii* test was positive in BAL. Intravenous TMP–SMX (12 mg/kg TMP), which resulted in improvement in respiratory status and weaning of respiratory support over 7 days.

**Conclusion:** Severe viral infection associated with several weeks of immune suppression with the consequence may be the cause of secondary infections.

### P343. The first case of successful treatment of invasive mycosis caused by Candida guiliermondii and Exophiala dermatitidis in a neonatal patient

ShagdileevaE.^1^VorobevaS.^2^KotinaN.^2^BogomolovaT.^3^VybornovaI.^3^KlimkoN.^1^1North-West State Medical University named after I.I. Mechnikov, Saint-Petersburg, Russian Federation2City Children’s Hospital № 1, St. Petersburg, Russian Federation3Kashkin Research Institute of Medical Mycology, North-Western State Medical University named after I.I.Mechnikov, St. Petersburg, Russian Federation

**Case Report:** Newborn girl on the 2nd day of life was admitted to the City Children’s Hospital № 1 on 25.01.2019. Birth weight was 3050 g, gestational age at birth – 40 weeks. Mother 43 years old: pregnancy - 8, childbirth - 6, HIV since 2014. The underlying condition was early hemorrhagic disease of the newborn, gastric bleeding. Result of HIV-1 DNA PCR test was negative, and antiretroviral prophylaxis (zidovudine) was used. Risk factors for IM were prolonged central venous catheter (CVC) use and broad-spectrum antibacterial therapy. Clinical manifestation of IM was body temperature > 38 °C, refractory to broad-spectrum antibacterial therapy. A patient was treated empiricaly with fluconazole. Diagnosis of IM was confirmed with positive blood culture: resistant to fluconazole in vitro *C. guiliermondii* and *E.dermatitidis*. Antifungal treatment was changed to micafungin. Clinical condition was improved and results of multiple blood cultures were negative. The total duration of micafungin treatment was 30 days. **Conclusion.** In the treatment of invasive mycosis in neonatal patients, the possibility of several pathogens should be considered.

### P344. Invasive Fungal Infections in a Paediatric Haematological-Oncological centre: a retrospective study of 15 years.

TragiannidisA.^1^GiantsidiA.^2^KotsosD.^2^TsotridouE.^2^SarantiA.^2^AntariV.^2^HatzipantelisE.^2^VyzantiadisT.-A.^3^1Hematology Oncology Unit, 2nd Pediatric Deparment, Aristotle University of Thessaloniki, AHEPA Hospital, Thessaloniki, Greece2Hematology Oncology Unit, 2nd Pediatric Deparment, Aristotle University of Thessaloniki, AHEPA Hospital, Thessaloniki, Greece3First Department of Microbiology, Medical School, Aristotle University of Thessaloniki, Thessaloniki, Greece

**Objectives:** Despite the major progress made in the last few years regarding the prevention, diagnosis and treatment of invasive fungal infections (IFIs), they still represent an important cause of morbidity and mortality in paediatric patients with malignancies and those undergoing HSCT. The aim of this retrospective study was to collect data of the incidence and outcome of IFIs in children with haematological malignancies and solid tumors in a Paediatric Hematology Oncology Unit. Additionally, specific data on diagnostics, predisposing factors and treatment was also collected

**Methods:** Data was collected retrospectively from patients with an IFI (proven, probable, possible), who were diagnosed between January 1, 2003 and December 31, 2017 with haematological malignancies and solid tumors in the Haematological-Oncological Unit of the 2^nd^ Department of Paediatrics, AHEPA University Hospital. By reviewing the patients’ records, information regarding gender, age at the time of the diagnosis of the IFI, underlying disease, treatment phase, the presence of neutropenia and its duration (> or < 10 days) as well as severity (< or > 500/mm^3^) and the site of the fungal infection was obtained. Last follow-up was performed after 12 months after the end of chemotherapy and included the status of disease, cause of death and in case of the occurrence of an IFI, the outcome of the infection.

**Results:** Eleven out of the twenty-six children enrolled in the study were males. The underlying disease was ALL for 19 patients (73%) and AML for 6 (23%). Twenty of them were under parenteral nutrition during the manifestation of the IFIs and 20 of the patients were presented with neutropenia (ANC<500/mm3). The most common location of the IFIs were the lungs and the most common pathogen was Aspergillus spp. According to the revised criteria of the European Organization for Research and Treatment of Cancer/Invasive Fungal Infections Cooperative Group and the National Institute of Allergy and Infectious Diseases Mycoses Study Group (EORTC/MSG) for the diagnosis of IFSs, 5 of the diagnoses were classified as proven, of which two patients developed zygomycosis, one chronic disseminated candidiasis, one cryptococcosis and one systematic aspergillosis. Out of the rest 21 patients, 12 were classified as being affected by probable and 9 as being affected by possible IFIs. Eleven patients received a combination of antifungal drugs, whereas the rest of them were treated with monotherapy. Twenty received liposomal amphotericin B (LAMB), eleven voricanazole, two caspofungin, two micafungin and one fluconazole. IFI was lethal in 9 out of the 26 patients (34%). Patients with zygomycosis were successfully treated with surgical excision of the lesion combined with high dose of LAMB.

**Conclusion:** The results of this study suggest that, despite the progress regarding the diagnosis and treatment of IFIs with use of new antifungal drugs, mortality remains high. Therefore, it becomes obvious that early suspicion as well as prophylactic and empirical therapy are significant factors contributing in the improvement of the outcome of IFIs.

### P345. Candida sepsis successfully treated with micafungin add-on therapy in extremely preterm infants: report of two cases.

KimC.S.Department of Pediatrics, Keimyung University School of Medicine, Daegu, Republic of Korea

**Case Report: Objectives:** Candida sepsis is a common hospital-acquired infection in the neonatal intensive care unit and occurs usually in extremely preterm infants. Echinocandine is a new drug with a different mechanism of action from existing antifungal agents which induces fungal cell wall damage, and is mainly used in adults, and neonatal applications are extremely rare. **Case 1:** A female infant was born by an emergent Cesarean section with fetal distress at the gestational age 27 weeks. The birth weight was 720 g. After birth, the baby received intensive care for respiratory distress and feeding difficulties, including mechanical ventilation, central venous catheterization, and parenteral nutrition. On the 16th day of admission, the patient was suspected of sepsis and *Candida parapsilosis* was detected in the culture of both blood and central venous catheter specimens. Initially, amphotericin-B (initial dose: 0.5 mg/kg/day, maintenance dose: 1-1.5 mg/kg/day) was administered but fungemia persisted after 10 days of treatment. Thereafter, micafungin (therapeutic dose: 15 mg/kg/day) was added and administered concurrently. After 4 days of the combination therapy, CRP was decreased, and candida was no longer detected in blood samples. The patient was treated with amphotericin-B for 19 days and micafungin for 8 days, and there was no recurrence of fungal infection. There were no adverse effects of antifungal agents in laboratory findings at the end of treatment. **Case 2:** A female infant was born with vaginal delivery at the gestational age 26 weeks and 2 days. The birth weight was 750 g. After birth, the baby received neonatal intensive care, including mechanical ventilation and parenteral nutrition. On the 13th 15th day of hospitalization, *C. parapsilosis* was consecutively detected in blood samples of the patient. Initially, amphotericin-B was administered, but fungemia and elevated titers of CRP were persisted after 10 days of treatment. Thereafter, micafungin was added. Candida was no longer detected in the blood samples after 7 days of the combined therapy. The patient was treated with amphotericin-B for 25 days and micafungin for 14 days, and there was no recurrence of fungal infection. In addition, there were no adverse effects of antifungal agents in laboratory findings at the end of treatment. **Conclusion:** I report two cases of extremely preterm infants with Candida sepsis successfully treated with micafungin add-on therapy that did not resolve with amphotericin-B alone.

### P346. Invasive Aspergillosis in a Paediatric Haematological-Oncological center: a retrospective study of 15 years

TragiannidisA.^1^SarantiA.^2^GiantsidiA.^2^KotsosD.^2^TsotridouE.^2^PapambougioukiM.^2^HatzipantelisE.^2^VyzantiadisT.-A.^3^1Hematology Oncology Unit, 2nd Pediatric Deparment, Aristotle University of Thessaloniki, AHEPA Hospital, Thessaloniki, Greece2Hematology Oncology Unit, 2nd Pediatric Deparment, Aristotle University of Thessaloniki, AHEPA Hospital, Thessaloniki, Greece3First Department of Microbiology, Medical School, Aristotle University of Thessaloniki, Thessaloniki, Greece

**Objectives:** Invasive aspergillosis (IA) represents an important cause of morbidity and mortality in patients with primary and secondary immunodeficiencies and them undergoing Hematopoietic Stem Cell Transplantation (HSCT). In particular, Aspergillus spp. constitute the second most frequent cause of fungal infections in immunosuppresed patients, while IA seems to be responsible for higher mortality rates in comparison with candidiasis.

**Methods:** Retrospective case study of IA managed and treated in the Hematological-Oncological Unit between 2001 and 2016. The study included in total 16 children with possible, probable and proven IA according to the European Organization for Research and Treatment of Cancer/Invasive Fungal Infections Cooperative Group and the National Institute of Allergy and Infectious Diseases Mycoses Study Group (EORTC/MSG) Consensus Group criteria.

**Results:** Among the 16 children included in the study, there were 6 boys. The underlying condition was acute lymphoblastic leukaemia (ALL) in 10 patients (62.5%) and acute myeloid leukaemia (AML) in 6 (37.5%). Out of the 16 patients, 13 were neutropenic with an absolute neutrophilic count lower than 500/mm3 at the time of diagnosis. According to the revised criteria of the EORTC/MSG for the diagnosis of IFIs came up that in 1 patient the diagnosis was proven, the diagnosis of 9 patients was classified as probable and finally in 6 as possible. Combined antifungal treatment was administered in 4 patients, while 12 patients received a single antifungal agent. Liposomal amphotericin B (LAMB) was administered in 10/16 patients, voriconazole in 7/16, caspofungin in 1/16 (as combined treatment) and micafungin in 1/16 patients (as combined treatment). Six out of the 16 patients (37.5%) died due to the IFI.

**Conclusion:** According to the above findings there was concluded that despite the progress that was made regarding the diagnosis and treatment of IA with the use of newer formulations of antifungal agents, morbidity is still high. High suspicion and prompt initiation of empiric and prophylactic antifungal treatment in selected cases constitute significant factors for the improvement of the outcome of IA.

### P347. Thoracic actinomycosis mimicking tumor in a children

KozlovaO.^1^SuslovaI.^2^AvdeenkoY.^2^BorzovaY.^2^KlimkoN.^3^1Department of Clinical Mycology, Allergology and Immunology, North-Western State Medical University n.a. I.I. Mechnikov, Saint-Petersburg, Russian Federation2North-Western State Medical University n.a. I.I. Mechnikov, Saint-Petersburg, Russian Federation3Department of Clinical Mycology, Allergy and Immunology, North-Western State Medical University named after I.I.Mechnikov, St. Petersburg, Russian Federation

**Objectives:** Thoracic actinomycosis is rare and publications are limited.

**Methods:** Prospective, single-center study of 186 patients with different clinical forms of actinomycosis (2005 – 2017 yy.). Pulmonary actinomycosis was in 9% (*n* = 17) cases of them there were two children. We present two cases successful treatment of thoracic actinomycosis in children without risk factors whose initial diagnosis was malignancy.

**Results:** 14-year-old boy, previously healthy, was admitted with a history of continuous chest pain during the past three weekes, fever, dry cough and hemoptysis. He was referred to the oncology center with the hypothesis of malignant disease. Weight and height of the patient was appropriate the age and sex. Respiratory sounds were decreased in the right lower field. Laboratory findings demonstrated slight anemia (hemoglobin of 110 g/l) and elevated inflammatory markers, such as white blood cell count (leukocyte count of 16 * 10^9^/l) and C-reactive protein (25.5mg/dL). Tuberculin test was negative. A chest CT scan revealed a consolidation (70*40 mm) with spiculated margins and areas of ground-glass attenuation in the right lower lung. A percutaneous thoracoscopic biopsy of right lower lobe was performed. Histological examination revealed the presence filamentous organisms and sulfur granules, without evidence of malignancy. The thoracic actinomycosis was diagnosed. The patient received penicillin 15000000 units/day) during three weeks, followed by 5 months of amoxicillin (40mg/kg/day). During the first month of treatment, he showed clinical and radiograph improvement. He was followed up every 2 months until completing oral antibiotic therapy and remained asymptomatic year after the end of the treatment. Previously healthy 14-year-old girl was admitted with a history of hemoptysis during the past month, continuous chest pain, and fever. Respiratory sounds were decreased in the right lower lobe, the patient’s oxygen saturation was 96% while breathing ambient air. CT showed dense consolidation in the right lower lobe. The disease was initially interpreted as the malignant disease. A lobectomy was performed. Histological evaluation of surgical specimens with hematoxylin and eosin staining shows classic sulfur granules consistent with actinomycosis. She was started on intravenous sodium penicillin 8 000 000 units/day during two weeks, followed by 3 months of amoxicillin (40mg/kg/day), resulting in a rapid clinical response with the disappearance of all symptoms. General state of health was satisfactory, indicators of clinical and diagnostic studies within the normal range. CT imaging was normal.

**Conclusion:** Actinomycosis is an uncommon disease in children. Thoracic actinomycosis should be considered in the differential diagnosis of neoplasia.

### P348. Coccidioidomycosis disseminated disease in pediatric patient

Lora-TéllezV.Lara-HernándezM.F.D.C.Bonifaz-TrujilloA.Rodríguez-CoelloG.Mycology Laboratory, Hospital para el Niño Poblano, Puebla, Mexico

Case Report: Objective. To describe the clinical manifestations of the disease and laboratory diagnosis. Methods. Clinical characteristics, symptoms, direct examination of the pathological material, culture and biology molecular methods. Results. 11 years old female patient with malnutrition, who lives in semiarid region in the south-east of Mexico. 3 months of clinical evolution Fever 38°C Multiple lesions such as subcutaneous nodes tissue with localization in back, ilium region, knee, and feet. Without pleuritic, aching chest pain and cough. Subcutaneous abscesses at computer assisted tomography suggests with sarcoma diagnosis. Pathological material biopsy for laboratory diagnosis by Hematoxylin Eosin, Periodic Acid-Schiff stains, KOH 10% solution, culture on fungal media. Direct microscopic observation of the histopathological sections demonstrated the inflammatory response contained granulomatous elements, collections of neutrophils, areas of caseous necrosis and mature, ruptured spherules. The mature spherules measure 30 to 60 µm, with double walls up to 2 µm in thickness. Inside of the mature spherules multiple endospores measure 2 to 5 µm containing. The growth of colonies white and floccose at first 3 days was evident by culture methods. Coccidioidomycosis for diagnosis of the disease by the direct examination of the pathological material was enough. The fungal therapy with amphotericin B was successful. The molecular biology method by polymerase chain reaction in real time technique (PCR-rt) match with Coccidioides posadasii by the examination of the in vitro fungal growth. Conclusion. Coccidioidomycosis is usually acquired through inhalation of arthroconidia and the disease is known to be endemic to the United States, Mexico restricted to the semiarid and desert regions. It is also found in a lesser degree in Central and South America. At present, the etiological agents of coccidioidomycosis in man are Coccidioides immitis and Coccidioides posadasii. Although the both etiological agents have equal phenotypic characteristics and produced the same disease, there are differences between them, such as the geographic distribution and genetic characteristics. Coccidioides immitis is located in the north region of Mexico, whereas Coccidioides posadasii is located in the south region of the country. It is important to known the specific etiological agent for epidemiological reasons. Lesions of skin and subcutaneous tissue are among the most common manifestations of hematogenous dissemination. The usual dissemination as a complication of primary infection it was because malnutrition and the endemic region habitat.

### P349. Zygomycosis in pediatric patients and laboratory diagnosis.

Lora-TéllezV.Pérez-RicardezM.L.Lara-HernándezM.F.D.C.López-HernándezG.Mycology Laboratory, Hospital para el Niño Poblano, Puebla, Mexico

Case Report: Objective. To describe the distribution of the clinical manifestations and the etiological agents of zygomycosis identification in pediatric patients. Methods. Various clinical forms description in four pediatric patients with underlying condition. Etiological agent identification by direct examination and culture on Sabouraud Dextrose Agar of the pathological material. Results. Female 7 years old, acute lymphocytic leukemia as underlying condition. Sites of the infection were paranasal sinuses, eye´s orbital section, palate and face. Paranasal sinuses biopsy was selected as pathological material for direct examination and fungal culture. Aseptate hyphae branching at angle of 90 degrees approximately was observed by hematoxylin eosin and periodic acid-Schiff stains by direct examination. The etiological agent was Lichthiemia spp by fungal culture. Female 7 years old, acute lymphocytic leukemia as underlying condition. Site of the infection was paranasal sinuses. In the paranasal sinuses biopsy was observed aseptate hyphae 10 µm measure. Fungal culture on Sabouraud Dextrose Agar at 25°C, after 4 days a woolly colony growth was observed. Microscopic morphology of the colony was identified Mucor spp as etiological agent. Male 8 years old, burn trauma as underlying condition. Sites of the infection were legs, arms and hands. Tissue debridement was observed by direct examination with potassium hydroxide KOH 40% solution, and cultured on fugal media. A white-gray woolly colony growth was observed after 2 days on Sabouraud Dextrose Agar at 25°C, after 4 days incubation, the surface of the colony was covered with dark spots like a “salt – and- pepper” appearance. At microscope examination, the differential characteristics of the Rhizopus were observed, such as a single umbrached sporangiophore, a globose sporangia, sporangiospores and rhizoids. Microscopic morphology of the colony was identified Rhizopus spp. Female 1 month old, gastric perforation as underlying condition. At computer assisted tomography examination a mass like a tumor was observed in the gastric cavity. Gastric mucosa biopsy by direct examination multiple aseptate and very broad hyphae were observed. Histopathological examination of the gastric mucosa biopsy demonstrated the vascular invasion by hyphae, leading to thrombosis and infarction of tissue. Rhizopus spp as etiological agent by fungal culture examination. Conclusion. Zygomycosis is characterized by vascular invasion with hyphae, infarction, and necrosis of tissue and by an acute or subacute course. Zygomycosis caused by Rhizopus, Lichtheimia, Rhizomucor and Mucor has a wide geographic distribution. The distribution of the clinical manifestations was usual sites of infection because underlying condition such as acute lymphocytic leukemia with neutrocytopenia, and burn trauma. But gastric cavity was uncommon as site of infection due was introduced by oral-gastric catheter. Because the hallmark of Zygomycosis is vascular invasion by hyphae, leading to thrombosis and infarction of tissue, the antifungal therapy with Amphotericin B was successful while is delimited the original site of the infection, such as paranasal sinuses, before it presents in contiguous structures of the orbit, palate, face, nose, or brain. The early diagnosis as much as the site of infection contributed for a good clinical evolution.

### P350. rSpectrum of invasive yeast infections in children in Pakistan over the last five years: 2015-2019

FarooqiJ.^1^MemonS.^2^LaiqS.^1^NaqviF.^1^IqbalK.^1^TariqS.^1^ZafarA.^3^JabeenK.^4^1Pathology and Laboratory Medicine, Aga Khan University, Karachi-KHI, Pakistan2Microbiology, Karachi University, Karachi, Pakistan3Pathology and Laboratory Medicine, Aga Khan University, Karachi, Pakistan4Pathology and Laboratory Medicine, Aga Khan University, Karachi, Pakistan, Pakistan

**Objectives:** We aim to describe here the changing spectrum of invasive yeast infections in children in the last 5 years in Pakistan, using retrospective laboratory data from 2015-May 2019

**Methods:** Records of archived yeast isolates from Jan 2015-May 2019 kept at the Aga Khan University Clinical Laboratories, section of Microbiology in Karachi, were retrieved for analysis. A total of 1119 non-duplicate isolates were identified, of which 992 were invasive specimens from blood (833), CSF (51), wounds (47), intraabdominal collections (36), Central venous lines (17) and other sterile fluids (8). The patients were divided into neonates (259), paediatric age group (253) and adults (477). Frequencies of different *Candida* and non-*Candida* species isolated over the last 5 years were computed and compared for age groups using chi-square test. Hypothesis of a change in the spectrum over the years among each age group was also tested.

**Results:** *Candida* species made up 87.4% and 86.6% of all invasive yeasts in neonates and children, respectively. The most common group in neonates was a diverse group of rare *Candida* species (including *C. lusitaniae*, *C. guilliermondii* and *C. pelliculosa* to name a few) at 45.4%, followed by *C. tropicalis* (24%) and *C. parapsilosis* (15.7%); *Ustilago* species were the most common non-*Candida* yeasts at 9.2%. In children, the most common species was *C. tropicalis* (32%), followed by rare *Candida* species as described above (22.8%) and *C. albicans* (15.1%). *Ustilago* was again the most common non-*Candida* yeast at 11.1%. There was a statistically significant difference in the distribution of invasive yeasts between neonatal and paediatric age groups (p<0.001). The change in spectrum of invasive yeast over the years was significant for infants and children (p = 0.035) and adults (p = 0.029); however, we could not demonstrate a significant difference in neonatal (p = 0.096) and elderly (p = 0.270) spectrum of invasive yeast infections over the years. Fluconazole resistance in yeasts isolated from neonates was found to be 8.2% while it was 22.8% in infants and children, possibly due to an emergence of *C. auris* infections in children (12.8%) versus neonates (1.3%).

**Conclusion:** We conclude that it is essential to monitor the spectrum of yeast infections in neonates and children as they are the population where there is greatest diversity of invasive species, not limited to *Candida* species. Emerging pathogens are most likely to arise from this population, including resistant strains, hence, it is also important to monitor resistance trends.

### P351. Changing epidemiology of candida infection in NICU and PICU – multicenter study

PleškoS.^1^NovakM.^2^BenjakV.^2^HadžićD.^3^KardumD.^4^TomulićK. Lah^5^PolićB.^6^BucatM.^7^MišanovićV.^8^KondžaK.^9^MikšićM.^10^BakošM.^2^ĆaletaT.^2^1Depatment For Clinical and Molecular Microbiology, University Hospital Centre Zagreb, Zagreb, Croatia2Department of Pediatrics, University Hospital Center Zagreb, Zagreb, Croatia3Department of Intensive Therapy and Care Pediatrics Clinic, University Clinical Center Tuzla, Tuzla, Bosnia and Herzegovina4Department of Pediatrics, Neonatal Intensive Care Unit, University Hospital Osijek, Osijek, Croatia5Depatrtment of Pediatric, University Hospital centre Rijeka, Rijeka, Croatia6Department of Pediatrics, University Hospital Centre Split, Split, Croatia7Department of Pediatrics, Nicu, University Hospital Centre Split, Split, Croatia8Department of Pediatrics, University clinical Center Sarajevo, Sarajevo, Bosnia and Herzegovina9Department of Pediatrics, Childrens Hospital Zagreb, Zagreb, Croatia10Department of Pediatrics, university Hospital Centre Maribor, Maribor, Slovenia

**Objectives:** Invasive *Candida* infections are considered as very important cause of morbidity and mortality in neonatal intensive care unit (NICU) and pediatric intensive care unit (PICU). Although *C.albicans* is the most commonly isolated species from invasive candida infections the frequency of non-albicans candida has increased. Geographical differences in epidemiology, and even more important changes in susceptibility patterns have been described. It is very important to know the epidemiology in order to recognize patients in risk and to start the appropriate empirical therapy and improve patient outcome. The aim of this study was to determine changes in *Candida* infections epidemiology in NICU and PICU from three countries.

**Methods:** We included the data from three neighboring countries: Croatia, Bosnia and Herzegovina and Slovenia (10 centers altogether), during the two year period. We collected data about species of *Candida* causing infection, site of infection and antifungal susceptibility of strains. In first year, overall 65 patients with invasive fungal infection (IFI) (25 from NICU and 40 from PICU), and in second year 107 patients with IFI (59 from NICU and 48 from PICU) were enrolled in the study.

**Results:** During the first year of surveillance in NICU, *Candida albicans* was the most frequent isolate in IFI patients in 4 centers and *Candida parapsilosis* in one center. According to collected data, *Candida tropicalis, Candida krusei* and *Saccharomyces cerevisiae* were observed as rare and sporadic causes of IFI. During the second year of surveillance we observed the shift of species as leading cause of IFI between centers: *C. parapsilosis* was most frequent isolate and *C albicans* was less frequent. In some centers *C. famata, C. glabrata, C krusei, C. guillermondii* was reported, sporadically. In PICUs, during first year *C. albicans* was most common in four centers followed by *C. parapsilosis* except in one center where *C. lusitaniae* and *C. krusei* were recorded. *C. glabrata* and *C. guillermondii* were recorded, sporadically. In second year, we also noted the shift of species as leading cause of IFI: the number of *C. albicans* decreased and number of *C. parapsilosis* increased in 4 centers. It is important to emphasize that 60% of *C. parapsilosis* in second year were susceptible only to amphotericin B (resistant to fluconazole ad intermediate to echinocandins according to the EUCAST interpretation breakpoints). *C. glabrata*, *C. tropicalis*, *C. famata, Crytococcus laurentii* and *C. lusitaniae* were reported, sporadically.

**Conclusion:** The results of this observational study indicate the changing epidemiology of IFI pointing to differences in fungal isolates as leading causes of IFI between centers and in the same centers, during observational period of two years. In addition, this study noted the increase of MICs for some antifungals and shift in susceptibility of fungi, addressing the importance of monitoring and optimization of therapeutic guidelines of IFI in NICU and PICU.

### P355. Liposomal Vaccine Containing Aspergillus Proteins Protects Immunosuppressed Male and Female Mice Against Pulmonary Aspergillosis Caused by Azole Resistant or Sensitive Strains of Aspergillus fumigatus

SlarveM.^1^HolznechtN.^2^Adler-MooreJ.^2^HoS.^3^FujiG.^3^1Biology, Cal Poly Pomona, Riverside, United States of America2Biology, Cal Poly Pomona, Pomona, United States of America3Molecular Express, Inc, San Dominguez, United States of America

**Objectives:** This study tested the efficacy of an immunotherapeutic liposomal *Aspergillus* vaccine (VesiVax® LAsV), with or without additional antifungal drug treatment. The vaccine contained *Aspergillus* proteins Aspf3 and Aspf9, and the adjuvant lipidated tucaresol (LT1). LAsV was tested in female mice challenged with two azole resistant strains of *Aspergillus fumigatus* (V29 and V80), and in male mice with an azole sensitive strain of *A. fumigatus* (ATCC#13073).

**Methods:** Six week old Swiss Webster mice were vaccinated with LAsV (Molecular Express, Inc.) at 7.5 µg Aspf3/dose,7.5 µg Aspf9/dose and 5 µg LT1/dose, subcutaneously on d0, with intranasal boosts on d21 and d42. Control mice were given phosphate buffered saline (PBS). Prior to challenge, spleens and blood were collected (*n* = 5/group) for ELISpot cytokine analysis and anti-spore antibody agglutination assays. On d53, d55, and d57, vaccinated mice were immunosuppressed intraperitoneally with 28 mg/kg triamcinolone acetonide. On d56, mice were intranasally challenged with *A.fumigatus* conidia (2-2.8 X 10ex7/mouse depending on fungal strain). Some vaccinated groups were also given a short course of 7.5 mg/kg liposomal amphotericin B (AmBisome®, AmBi, Gilead Sciences Inc.) intravenously at 12h, 36h, and 60h post-challenge. Lungs were collected (*n* = 7/group) 3 days post challenge for fungal burden, and remaining mice (*n* = 9/group) were monitored for survival to d77.

**Results:** LAsV vaccinated male and female mice had reduced lung fungal burdens compared to PBS control mice regardless of the strain of *A. fumigatus* challenge. Female mice vaccinated with LAsV, challenged with strain V29 or V80, and then given subsequent AmBi treatment, had higher survival than PBS control mice (V29 = 79% vs 21%; V80 = 57% vs 25%) and the vaccine alone also yielded some protection when mice were challenged with V29 (55% survival). Non-vaccinated female mice given AmBi alone had only 37% survival following V29 challenge, and 22% survival with V80 challenge. Results in male mice vaccinated with LAsV and given AmBi after challenge, were similar to the female mice, yielding 55% survival following *A fumigatus* challenge (#13073) versus 22% survival for mice given AmBi alone or PBS. ELISpot cytokine analysis showed that LAsV vaccinated female and male mice had a significantly higher number of IL-4 than IFN-γ secreting splenocytes following incubation with Aspf3 and Aspf9 (p = 0.0178 for males, p = 0.0290 for females), which is characteristic of a Th2 response. Anti-spore antibody agglutination titers demonstrated that LAsV vaccinated mice also had increased anti-*A.fumigatus* spore antibodies in the serum than PBS control mice (p = 0.0009 for males, p<0.0001 for females).

**Conclusion:** Prophylactic LAsV vaccination of immunocompetent female or male mice, which were then immunosuppressed prior to Aspergillus challenge, followed by a short course of AmBi treatment, provided more protection against pulmonary aspergillosis than AmBi treatment alone whether the infection was caused by azole resistant or sensitive strains of *A.fumigatus.* Elevated levels of anti-*A.fumigatus* spore antibody titers correlated with protection, serving as a possible biomarker to indicate protective immune responsiveness to the vaccine. The LAsV has the potential to be used as an immunomodulatory treatment to further enhance the efficacy of antifungal drugs.

### P356. Anti–interferon-γ autoantibodies underlie disseminated Talaromyces marneffei infection

CaoC.Dermatology, The first affiliated hospital of Guangx medical university, Nanning, China

**Objectives:** Anti–interferon-γ autoantibodies underlie disseminated *Talaromyces marneffei* infection Obsjects: *T. marneffei* causes life-threatening opportunistic infections, mostly in Southeast Asia and South China. Most *T. marneffei* infections occur in patients infected with human immunodeficiency virus (HIV), but this infection can, nevertheless, occur in HIV-negative individuals in the absence of immunosuppression. We investigated the role of anti-interferon (IFN)-γ autoantibodies in severe *T. marneffei* infection in HIV-negative patients.

**Methods:** Anti–interferon-γ autoantibodies underlie disseminated *Talaromyces marneffei* infection Obsjects: *T. marneffei* causes life-threatening opportunistic infections, mostly in Southeast Asia and South China. Most *T. marneffei* infections occur in patients infected with human immunodeficiency virus (HIV), but this infection can, nevertheless, occur in HIV-negative individuals in the absence of immunosuppression. We investigated the role of anti-interferon (IFN)-γ autoantibodies in severe *T. marneffei* infection in HIV-negative patients. Method: We enrolled 58 HIV-negative adults who developed *T. marneffei* severe infections but were otherwise healthy in this study. We evaluated the presence of anti-IFN-γ autoantibodies and their neutralizing activity.

**Results:** The prevalence of neutralizing anti–IFN-γ autoantibodies was high (94.8%) in these previously healthy adults suffering from severe *T. marneffei* infections. The presence of anti–IFN-γ autoantibodies was strongly associated with HLA-DRB1*16:02 and DQB1*05:02 in these patients.

**Conclusion:** Adult-onset acquired immunodeficiency due to autoantibodies against IFN-γ is the major cause of severe *T. marneffei* infection in HIV-negative patients in the regions in which this fungus is endemic. The high prevalence of pathogenic HLA class II DRB1*16:02 and DQB1*05:02 alleles, which are strongly associated with anti-IFN-γ autoantibodies, may account for the restriction of severe *T.marneffei* infection to Southeast Asia. Our findings clarify the pathogenesis of *T. marneffei* infection and pave the way for the development of novel treatments.

### P358. Phagocytic activity and cytokines production by murine RAW-264.7 macrophages in response to Microsporum canis

NevesJ.^1^KonnoF.^1^LalloM.^1^Alvares-SaraivaA.^2^HurtadoE.^1^AlvarezJ.^1^CoutinhoS.D.A.^1^1Post-graduation Program In Environmental and Experimental Pathology, Paulista University, São Paulo, Brazil2Institute of Physical Activity and Sports Sciences, Cruzeiro do Sul University, São Paulo, Brazil

**Objectives:** Dermatophytes are important fungi for public health, as they are transmitted between animals and humans causing zoonoses. The innate immune system plays an important role in the establishment of dermatophytic infection; however, there are few studies on the relationship between innate immune response and *Microsporum canis*. Thus, the aim of this work was to evaluate the response in vitro of macrophages challenged with *Microsporum canis* microconidia. We evaluated the ability of macrophages stimulated or not with LPS, to phagocytize, to produce nitric oxide and cytokines, and the cell viability.

**Methods:** Murine Raw-264.7 macrophages were cultivated overnight on glass coverslips in a 24 well plates. Macrophages were infected with *M. canis* (ATCC-36299) in the ratio of 0.25:1 (microconidia/macrophages) and incubated at 37˚C with 5% CO_2_ for 30 min, 1 h, 3 h and 6 h. After incubation intervals, glass coverslips were collected and submitted to Giemsa stain. Two hundred cells were counted and calculated the percentage of macrophages that internalized at least one spore. LPS (1mcg/mL) was added to the macrophages 24 h prior *M. canis* infection in one group. Supernatants were collected for detection of nitric oxide and cytokine levels, and to check the cell viability.

**Results:** The phagocytic index showed an increase in phagocytosis over time (Figures 1 and 2), reaching the maximum at 6 h. The percentage of non-LPS stimulated macrophages that phagocytosed *M. canis* microconidia was 28.0%, 33.5%, 62.5% and 71.5%, respectively in 30 min, 1 h, 3 h and 6 h (Figure 1). In macrophages stimulated with LPS the phagocytic index was 34.5%, 47.5%, 67.0%, and 74.0%, respectively in 30 min, 1 h, 3 h and 6 h (Figure 1). It was observed similar nitric oxide production in both, macrophages challenged and not with *M. canis*. Levels of TNF-α have increased over time in macrophages challenged with *M. canis* stimulated or not with LPS when compared with macrophages non-infected (statistically significant). Higher levels of IL-6 were produced by macrophages stimulated with LPS and infected with *M. canis* at 1 h (statistical difference). Cell viability decreased over time, with the peak of macrophages killing at 6 h. Microbicity test showed that, even after phagocytosis, spores of *M. canis* remained viable inside macrophages.

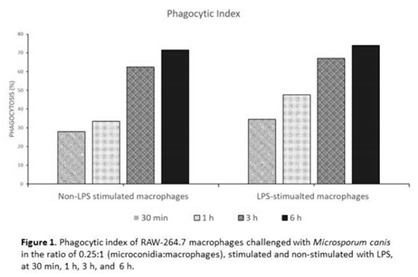

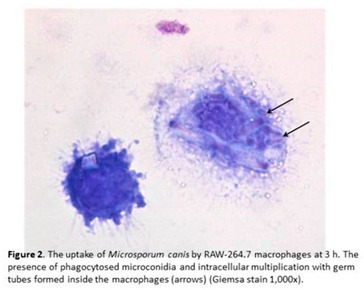





**Conclusion:** Raw-264.7 macrophages showed high phagocytic activity against *M. canis*, which increased when macrophages were previously stimulated with LPS. LPS stimulation also influenced the production of cytokines, which suggests greater modulation in the response to infection. Increased production of the pro-inflammatory cytokines TNF-α and IL-6, which are important in the recruitment of new phagocytic cells and in the resolution of the disease, have been found in macrophages challenged with *M. canis*. The results suggest that, although macrophages present phagocytic activity and cytokine production when challenged with *M. canis*, their viability and microbicidal activity are impaired.

### P359. Importance of using media with physiologic glucose concentration for studying host defence against C. albicans in vitro

KennoS.HarpfV.RambachG.SpethC.WürznerR.Div Hygiene & Med. Microbiology, Med. Univ. Innsbruck, Innsbruck, Austria

**Objectives:** *Candida albicans* is a clinically important opportunistic yeast and has evolved different immune evasion molecules on its cell surface. In the study on the role of one of these, the transmembrane protein “High affinity glucose transporter 1” (Hgt1p), involved in glucose metabolism, but also high-jacking the complement regulator factor H (FH) from host serum, we have used different media, one of them the commonly used YPD medium (yeast extract 1%, peptone 2% and dextrose 2%) to study the role of this evasion molecule in immune defence.

**Methods:** *C. albicans* strains (SC5314 und others) were opsonized with human serum and incubated with anti-FH and anti-complement C3b/iC3b antibodies; their deposition on the cell wall surface was assessed by fluorescence-activated cell sorter (FACS). In parallel, *C. albicans* strains were co-cultured with fresh human polymorphonuclear neutrophils (PMNs). All these experiments were done at different glucose concentrations, mainly because a glucose transporter was involved.

**Results:** *Candida* wild type strain SC5314 showed a clear FH deposition on its cell wall at the physiological glucose concentration of 0.1%, but this was completely higher and thus overestimated when using the two high glucose (0.3%, 2%) containing media. Consequently, the resulting low C3b/iC3b deposition at 0.1% was even lower at the unphysiologically high glucose levels. Also the thus impaired phagocytosis, observable at 0.1%, appeared much more dramatic at glucose levels which are not achievable in vivo.

**Conclusion:** The main events in immune defence, such as opsonisation by C3b/iC3b and phagocytosis by PMNs and the counteracting evasion strategy by *Candida* to high-jack FH are all highly dependent on the glucose concentration and all experimental data are thus completely misleading, if media with unphysiological glucose concentration are employed, such as the commonly used YPD.

### P360. rImmune response in C57BL mice in Encephalitozoon cuniculi infection

HidifiraA. Miyuki^1^,^2^PereiraA.^1^,^2^Alvares-SaraivaA.^1^HurtadoE.^1^KonnoF.^1^AlvarezJ.^1^LalloM.^1^,^2^1Post-graduation Program In Environmental and Experimental Pathology, Paulista University, São Paulo, Brazil2Biomedicina, Centro Universitário São Camilo, São Paulo, Brazil

**Objectives:** *Encephalitozoon cuniculi* is a microorganism belonging to the phylum Microsporidia and to the kingdom Fungi, considered an opportunistic and obligatory intracellular pathogen. The first line of defense against *E. cuniculi* is the innate immune response through the action of phagocytic cells and production of cytokines that are responsible for the activation of T lymphocytes. The immune response against *E. cuniculi* has not been fully elucidated and further studies are needed to understand the involvement of cell types such as macrophages in the role of microsporidiosis. This study evaluated the populations of macrophages and lymphocytic cells in *E. cuniculi* infection in C57BL/6 mice.

**Methods:** SPF C57BL/6 mice were used and divided in 4 groups: uninfected, infected, uninfected-immunosuppressed and infected-immunosuppressed. Immunosuppression was made by intraperitoneal injection of cyclophosphamide and euthanasia was performed by anesthetic deepening to collect the peritoneal lavage and remove the spleen. Lymphocytes and macrophages (F4/80) were identified by cell surface markers using flow cytometry.

**Results:** The results of the evaluation of spleen and peritoneal cells indicate that in the peritoneum there was an increase in CD8^+^ T cells in infected group in relation to the uninfected and infected-immunosuppressed groups and a decreased in CD4^+^ T population in infected-immunosuppressed group. The B cells populations were decreased in both groups infected (immunosuppressed and non-immunosuppressed). In the spleen, there was an increase of CD8^+^ T cells and B cells in infected-immunosuppressed mice and a decrease in CD4^+^ T in the same groups evaluated. Macrophage markers analysis revealed expression of F4/80^+^ CD80/86^high^ MCHII^high^ in both infected animals (immunosuppressed and non-immunosuppressed) in the peritoneal lavage and in the spleen these cells were increased only in infected-immunosuppressed group.

**Conclusion:** The results of this study help to understand the involvement of cells as macrophages in the *E. cuniculi* infection in C57BL/6 mice.

### P361. Level of Mannose binding Lectin protein in dermatophytic patients and its correlation with MIC values

HemanthV.^1^KindoA.J.^2^SubramanianA.^3^KrishnanA.^3^1Microbiology, Sri Ramachandra Medical college and Research Institute, chennai, India2Microbiology, Sri Ramachandra Medical College and Research Institute, SRIHER, Chennai, India3Dermatology, Sri Ramachandra Medical College and Research Institute, SRIHER, Chennai, India

**Objectives:** 1.Speciation of dermatophytes from the clinical isolates 2.To study the antimicrobial pattern of frequently used antifungal agents 3.To determine the serum MBL levels in patients with dermatophytosis 4.To compare MIC levels with serum MBL levels

**Methods: METHODS:** Clinically diagnosed dermatophytic patients were sampled for microscopy and culture, grown dermatophytes was subjected to phenotypic identification by conventional method. Antifungal susceptibility testing was done by broth microdilution method according to CLSI M38-A2 document guidelines. *Trichophyton mentagrophytes* ATCC 4439 was included in each run of susceptibility testing as a quality control. Final drug concentrations in the microdilution plates ranged from 64 to 0.25 µg/ml for fluconazole and AmphotericinB and from 16 to 0.25 µg/ml for Itraconazole, Voriconazole, Terbinafine and Griesofulvin. This is an ELISA based assay performed in microwells coated with a monoclonal antibody against the MBL carbohydrate- binding domain. Bound MBL is detected with the same antibody that has been labeled with biotin, followed by development with horseradish peroxidase (HRP)-conjugatedstrepavidin and incubation with a chromogenic substrate. Reading was taken at 620 nm.

**Results:** Out of the 100 clinical isolates tested, 58 of them were identified as *Trichophyton mentagrophytes*, 40 were *Trichophyton rubrum.* and the remaining two isolates were identified as *Epidermophyton Floccosum* and *Microsoprum nanum* Antifungal susceptibility testing was carried out for 100 isolates of *Trichophyton* species against 6 antifungal agents. All isolates showed detectable growth after 4-5days of incubation. MICS for *Trichophyton mentagrophytes* ATCC 4439 were within the established range. Mannose Binding Lectin ELISA: Results awaited for ELISA Assay.

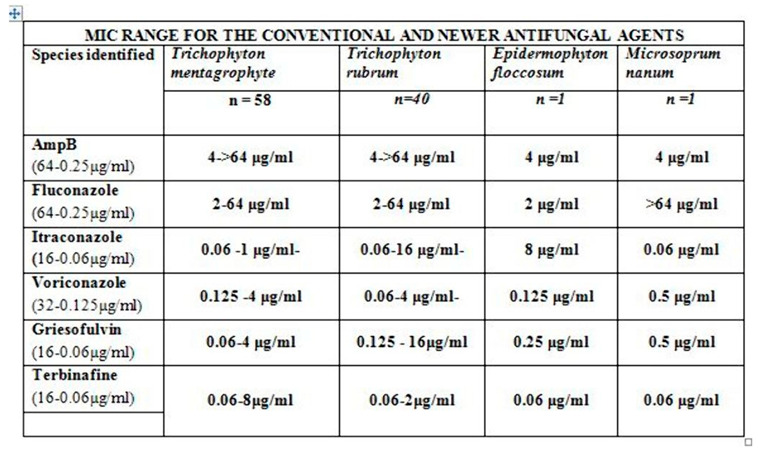



**Conclusion:** In this study *Trichophyton mentagrophytes* was the most common cause of dermatophytosis. The findings of this study showed that the patients with dermatophytosis have higher MBL serum levels, the acute phase protein plays a role in dermatophyte infections. It is well known that mutations in MBL genes are associated with reduced serum levels, which is a risk factor for infection. In this study, the high level of MBL levels could be used as prognostic marker for dermatophytic infections. Further studies with larger samples have to be under taken to come to definite conclusion.

### P362. Aspergillus infection in STAT3-deficient patients is associated with defective interferon-gamma and Th17 responses

DanionF.^1^AimaniandaV.^2^BayryJ.^3^DuréaultA.^1^BougnouxM.-E.^4^TcherakianC.^5^AlyanakianM.-A.^6^GueganH.^7^PuelA.^8^PicardC.^9^LortholaryO.^1^LanternierF.^1^LatgéJ.-P.^2^1Infectious Diseases, Necker hospital, Paris, France2Institut Pasteur, Paris, France3Equipe-immunopathologie Et Immunointervention Thérapeutique, INSERM, Paris, France4Parasitology Mycology, APHP Hôpital Necker-Enfants-Malades, Paris, France5Pneumology, Hopital Foch, Paris, France6Immunology, AP-HP Necker Hospital, Paris, France7Laboratoire De Parasitologie-mycologie, CHU de Rennes, Rennes, France8Inserm U1163, Institut Imagine, Paris, France9Centre D’étude Des Déficits Immunitaires (cedi), AP-HP Necker Hospital, Paris, France

**Objectives:** Signal transducer and activator of transcription 3 (STAT3)-deficient patients are highly susceptible to many bacterial and fungal infections including aspergillosis, although its pathogenesis remains largely unknown. **Objective**: To investigate the immune responses of STAT3-deficient patients against *Aspergillus fumigatus* infection

**Methods:** Phagocytic and killing efficiencies as well as cytokine responses of mononuclear cells and neutrophils of STAT3-deficient patients were investigated against *A. fumigatus*.

**Results:** We compared results among three groups, STAT3 deficient patients with or without aspergillosis and healthy controls. STAT3-deficient patients with aspergillosis showed elevated titers of *A. fumigatus* specific IgE and IgG compared to STAT3-deficient patients without aspergillosis as well as healthy controls. *A. fumigatus* conidial phagocytic and killing efficiencies of monocytes and neutrophils from STAT3-deficient patients with or without aspergillosis were similar to those from healthy controls. When stimulated with *A. fumigatus* conidia, peripheral blood mononuclear cells from patients with *STAT3*-mutation showed reduced levels of secreted (pg/mL) and intracellular (%) cytokines compared to healthy controls (IFN-γ: 1836 vs 3248 pg/mL and 3.7% vs 11.4%; IL-17: 16 vs 80 pg/mL and 2.6 vs 7.0%; IL-22: 64 vs 170 pg/mL). In addition, there were significantly lower levels of IFN-γ and TNF-α in STAT3-deficient patients with aspergillosis compared to those without aspergillosis (IFN-γ: 683 vs 3420 pg/mL; TNF-α: 779 vs 4743 pg/mL). In the STAT3-deficient cohort, three patients with aspergillosis were treated with IFN-γ in addition to antifungals; two of them showed favorable outcome.

**Conclusion:** STAT3-deficiency leads to defects in adaptive immune responses against *A. fumigatus*, particularly with a low IFN-γ response in those with aspergillosis, suggesting potential therapeutic benefit of recombinant IFN-γ in these patients.

### P363. Functionality of neutrophils against Aspergillus fumigatus in patients with alcoholic cirrhosis

BlaizeM.^1^BlezD.^1^LebrayP.^2^Meghraoui-KeddarA.^1^CombadièreC.^1^BoissonnasA.^1^RudlerM.^2^FekkarA.^1^1Sorbonne Université, Inserm, CNRS, Centre d’Immunologie et des Maladies Infectieuses, Cimi-Paris, F-75013, PARIS, France2AP-HP, Groupe Hospitalier Pitié-Salpêtrière, Service d’Hépato-Gastro-Entérologie, F-75013, Paris, France

**Objectives:** Patients with liver cirrhosis are at risk of developing bacterial or fungal infections. The incidence and severity of these infections depend on the stage of the disease. Severe alcoholic hepatitis, the most serious stage of liver failure in patients with alcohol abuse and chronic liver disease, has been associated with the development of many cases of invasive aspergillosis and is now considered a risk factor for it. A dysfunction of the immune system, namely systemic inflammation and immunodeficiency, is described in cirrhosis with, in particular, an alteration of the phagocytic and oxidative capacities of neutrophils. The latter is known to be the main immune effector against *Aspergillus*. Therefore, we aim to study neutrophil’s responses from patients with alcoholic cirrhosis at different stages of their disease against *Aspergillus fumigatus*.

**Methods:** To achieve this objective, we conducted a prospective monocentric study involving 23 patients and 12 healthy controls. Patients were divided into 4 groups: stable cirrhosis (*n* = 5), decompensated cirrhosis without severe alcoholic hepatitis (*n* = 3), decompensated cirrhosis with severe alcoholic hepatitis (*n* = 11) and decompensated cirrhosis with severe alcoholic hepatitis after 7 days of steroid treatment (*n* = 4). Interactions between neutrophils and *Aspergillus* hyphae were analyzed by video microscopy, while the oxidative burst of neutrophils before and after fungal stimulation and cytokine production was determined by flow cytometry and enzyme immunoassay (EIA).

**Results:** Compared to healthy controls, patients with severe alcoholic hepatitis had an increase in the production of reactive oxygen species by their neutrophils (1727 *versus* 2545, expressed as mean fluorescence intensity, p = 0.041). An increase in plasma IL-8 levels (95 *versus* 378 ng/mL respectively, p = 0.006) was also observed between these groups. Also among patients with severe alcoholic hepatitis, the production of reactive oxygen species decreased after 7 days of corticosteroids compared to other patients, both under basal conditions (937 *versus* 2545; expressed as mean fluorescence intensity, p = 0.011) or after stimulation with germinating conidia (2031 *versus* 9272; expressed as mean fluorescence intensity, p = 0.014). In addition, video microscopy experiments (Figure 1) showed that an alteration in the ability of neutrophils to kill *A. fumigatus* hyphae appears in the severe alcoholic hepatitis group after 7 days of corticosteroid therapy compared to patients sampled before corticosteroid therapy began (12% of mortality *versus* 46% respectively, p = 0.03) (Figure 2).

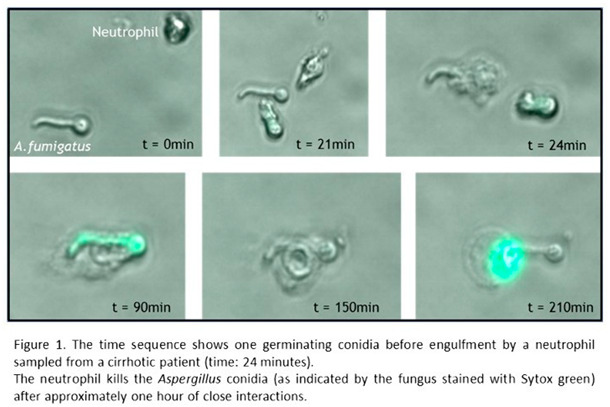

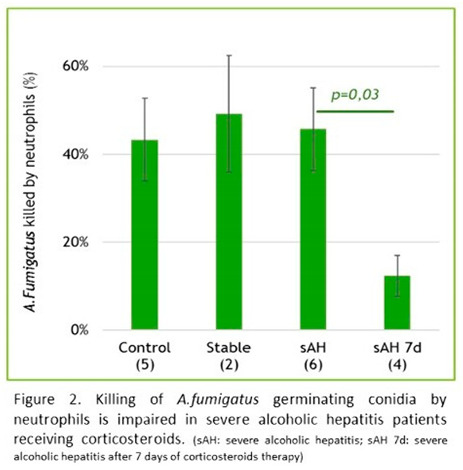





**Conclusion:** In conclusion, a pro-inflammatory state is present in patients with severe alcoholic cirrhosis as well as in patients with stable cirrhosis. The introduction of corticosteroids as a treatment for severe alcoholic hepatitis quickly leads to a dramatic decrease in the inflammatory environment and the neutrophil’s ability to kill *Aspergillus fumigatus*.

### P364. Impact of mould exposure on the immune response in the course of chronic obstructive pulmonary disease (COPD)

FrealleE.^1^,^2^PichavantM.^1^RouzicO. Le^3^RebouxG.^4^BautinN.^3^WilleminM.-C.^3^DelourmeJ.^3^SendidB.^2^NseirS.^5^FryS.^3^GossetP.^1^1Univ. Lille, CNRS, Inserm, CHU Lille, Institut Pasteur de Lille, U1019 – UMR8204 - CIIL - Center for Infection and Immunity of Lille, Lille, France2Parasitology-mycology, CHU Lille, Lille, France3Clinique Des Maladies Respiratoires, CHU Lille, Lille, France4Mycology - Umr6249 Cnrs Chrono-environnement, University Hospital Besançon, Besançon, France5Pôle De Réanimation, CHU Lille, Lille, France

**Objectives:** Chronic obstructive pulmonary disease (COPD) is a complex chronic respiratory disease which affects 8% of subjects over 45 years-old in France. It is mainly caused by cigarette smoke, which induces chronic lung inflammation and is considered a key etiological factor in the development and pathogenesis of COPD. Other environmental factors, such atmospheric pollution, can also be involved in the evolution of the disease, modulating inflammation and promoting exacerbations. Here, we assessed the contribution of domestic mould exposure to lung and systemic inflammation in COPD patients, during exacerbation or at stable state.

**Methods:** Sixty-two COPD patients were recruited during severe acute exacerbation requiring hospitalization between August 2011 and November 2015 in Lille University Hospital. Mycological analysis of sputa and anti-*Aspergillus* antibodies detection was performed in order to assess *Aspergillus* colonization and sensitization status. CXCL8, TNFα (recruiting/activation of monocytes and neutrophils), IL-1β, IL-6, IL-17, IL-22 (Th17 response) and CXCL4, CD62P, CD40L (platelet activation) levels were measured in the sputum and in the serum. The inflammatory response was compared at inclusion (exacerbation) and after an 18-months follow-up (at stable state) between (i) *Aspergillus fumigatus* colonized or non-colonized patients, (ii) *Aspergillus* sensitized or non-sensitized patients, and (iii) patient with or without mould exposure, which was assessed using electrostatic dust collectors (EDCs) that were exposed during 10 weeks in the patient’s home.

**Results:** *A. fumigatus* colonization was detected in 16.9% of patients at inclusion (during exacerbation) and 4% of patients at stable state. Anti-*Aspergillus* antibodies detection was positive in 32.2% of patients at inclusion and 36.4% after the 18-months follow-up. No significant differences were found between the cytokine levels during exacerbation or at stable state when *A. fumigatus* colonized or non-colonized patients, and *Aspergillus* sensitized or non-sensitized patients were compared. However, during exacerbation, patients with mould exposure showed significantly higher levels of serum CXCL8 (19.6±41.2 pg/mL vs <7 pg/mL for non-exposed patients, P = 0.001). Furthermore, at stable state, mould exposure was associated with significantly higher levels of serum IL-6 (28.9±87.9 pg/mL vs <2 pg/mL, P = 0.016), and significantly lower levels of serum IL-22 (86.8±31.6 pg/mL vs 285.8±363.3 pg/mL, P = 0.0008).

**Conclusion:** These data suggest that domestic mould exposure could contribute to the alteration of the inflammatory response in the course of COPD. *Funding: Conseil Régional Hauts-de-France, Gilead, MSD, Pfizer*

### P365. Effects of ibrutinib treatment on neutrophil response against A. fumigatus

BlaizeM.^1^BlezD.^1^SoussainC.^2^BoissonnasA.^1^Meghraoui-KeddarA.^1^MenezesN.^3^PortalierA.^4^CombadièreC.^1^LeblondV.^4^GhezD.^5^FekkarA.^1^1Sorbonne Université, Inserm, CNRS, Centre d’Immunologie et des Maladies Infectieuses, Cimi-Paris, F-75013, PARIS, France2Hématologie, Institut Curie - Site de Saint-Cloud, 35 rue Dailly, F-92210, Saint-Cloud, France3AP-HP, Groupe Hospitalier Pitié-Salpêtrière, Service de Parasitologie-Mycologie, F-75013, Paris, France4AP-HP, Groupe Hospitalier Pitié-Salpêtrière, Service d’Hématologie, F-75013, Paris, France5Département d’Hématologie, Gustave Roussy, 114 rue Edouard Vaillant, F-94805, Villejuif, France

**Objectives:** Ibrutinib, a small irreversible Bruton’s tyrosine kinase (Btk) inhibitor has become a leading therapy against chronic lymphoid leukemia and has significantly improved patient care. Recently, ibrutinib has been associated with the occurrence of invasive fungal infections, in particular invasive aspergillosis. This observation was surprising because patients with lymphoid malignancies are at low-risk of invasive aspergillosis. The mechanisms underlying the increased susceptibility to fungal infections associated with ibrutinib exposure are currently unknown. Polymorphonuclear neutrophil is the cornerstone of anti-*Aspergillus* immunity. However, since the potential effect of ibrutinib on the antifungal capacities of neutrophils has been studied to a very limited extent, we decided to do so in more detail.

**Methods:** To answer this question, we studied the consequences of *in vivo* and *in vitro* ibrutinib exposure on the neutrophil’s anti-*Aspergillus* responses. Thirty-three patients receiving ibrutinib for lymphoid malignancies were included. A total of 63 blood samples were collected from patients at different time points: just before ibrutinib treatment initiation (*n* = 23) then approximately 1 month (*n* = 22) and 3 months later (*n* = 18). Both flow cytometry and video-microscopy approaches were used to analyze neutrophils’ cell surface molecule expression, cytokine production, oxidative burst and killing activity against *Aspergillus*.

**Results:** We observed different alterations between the patient group at 1 month of treatment and the untreated group, as a decrease in the CD11b (major receptor for beta-glucan) expression or IL8 production. Above all, we observed a decrease in the production of reactive oxygen species (ROS) by neutrophils, both in the basal state (2333 *versus* 1131, expressed as mean fluorescence intensity, p<0,05) or after stimulation by *Aspergillus* germinating conidia (9330 *versus* 4569; p<0,05). Among the interactions observed between germinating conidia and neutrophils in 16-hours video microscopy co-cultures experiments, we focus on the engulfment and destruction of fungus by cells. A decrease in the ability of neutrophils to spread over the fungus was observed between treated and untreated patient groups (Figure 1). Regarding the destruction of *Aspergillus* by neutrophils, we observed a dramatic decrease with 50% of germinating conidia killed by neutrophils in the untreated group compared to only 1% in the group after one month of treatment and 0.8% in the group after 3 months of treatment (p<0.01) (Figure 2). The results were confirmed by the analysis of healthy neutrophils (from blood donors) exposed (or not) *in vitro* to ibrutinib. 

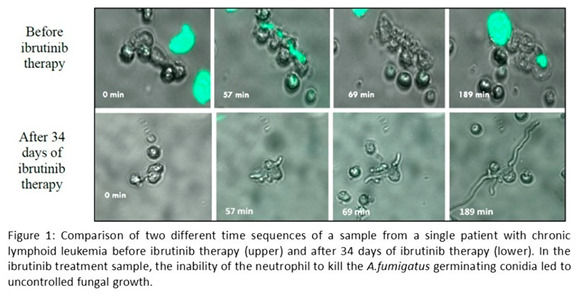

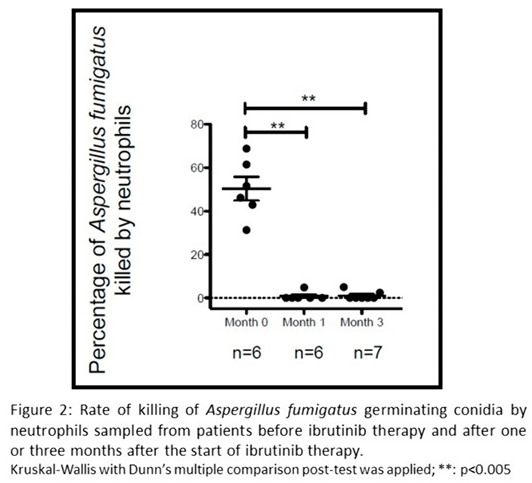





**Conclusion:** Ibrutinib is associated, both *in vitro* and in patients receiving this treatment, with several functional defects in neutrophils, including a decrease in the production of reactive oxygen species, a decrease in their ability to engulf the fungus and an inability to effectively kill *Aspergillus* hyphae. Our results provide a plausible explanation for the emergence of invasive aspergillosis in patients receiving ibrutinib. Further work is needed to understand the exact mechanisms underlying this effect, particularly if it is caused by inhibition of the Btk itself in neutrophils or by an off-target effect. On May 2019, part of the data has been provisionally accepted for publication in Haematologica journal.

### P366. Host innate immune response to echinocandins-resistant Candida glabrata clinical isolates: impact on the management of candidemia

BasmaciyanL.^1^GarnaudC.^2^BonF.^1^MaubonD.^2^AldebertD.^2^PapeP. Le^3^CornetM.^2^DalleF.^1^1Parasitology-mycology, University Hospital Dijon, Dijon, France2Univ. Grenoble Alpes, CNRS, CHU Grenoble Alpes, Grenoble INP*, TIMC-IMAG, 38000 Grenoble, France * Institute of Engineering Univ. Grenoble Alpes, GRENOBLE Cedex, France3Ea1155 Iicimed, Nantes University Hospital, NANTES, France

**Objectives:** *Candida glabrata* (*C. glabrata*) is a commensal yeast of the mucosal surfaces in humans. Incidence of invasive candidiasis due to this species has recently increased. Thus the high level of resistance that this species can present to different classes of antifungals, including echinocandins is of major concern. Nowadays, the impact of resistance acquisition in this opportunistic pathogen to the immune response of the host remains poorly understood. Knowing the mechanisms by which *Candida*’s intrinsic and adaptive resistance to antifungals can modulate (*i.e.* ’escape to’ or ’recognition by’) the immune system of the host is crucial. Indeed, most of the antifungal resistance mechanisms causes a direct or indirect modification of the fungal cell-wall. In addition, by establishing the first contact with the host tissues, the fungal cell-wall is involved in the recognition of *C. glabrata* by the host cells through interactions with Pattern Recognition Receptors (PRRs). We hypothesize that changes in the fungal cell-wall, as a result of resistance, could alter the recognition of *C. glabrata* by PRRs and thus trigger a different immune response, from a qualitative (*i.e.* cytokines production and immune cell recruitment) or quantitative (*i.e.* amplitude and duration of the immune response) point of view. The purpose of this study was (i) to characterize the cell wall modifications associated with resistance to echinocandins in *C. glabrata* and then (ii) to investigate the impact of such modifications on the virulence of the fungus and the host immune response.

**Methods:** Clinical isolates of *C. glabrata* included in the study displayed echinocandins’ resistance for which the mechanisms of resistance were molecularly characterized. The virulence of these clinical isolates was performed using two *in vitro* models of interaction of the fungus with the host compared to the ATCC reference strain: (i) the Yeast-to-Enterocyte interaction model and (ii) the Yeast-to-Macrophages interaction model. Cell wall characterization of the clinical isolates of *C. glabrata* was performed using microscopy and flow cytometry.

**Results:** Data support a change in the virulence and recognition by the host of echinocandins-resistant *C. glabrata* clinical isolates compared to the ATCC reference strain.

**Conclusion:** This study will allow (i) to better understand the prevalence of echinocandins-resistant *C. glabrata* isolates in high-risk patients, (ii) to specify whether high levels of *in vitro* resistance correlate or not to *in vivo* therapeutic failure and (iii) to establish effective immunotherapeutic strategies to combat *Candida* resistance to antifungals.

### P367. Gene variants of key TH-1 adaptive immune response proteins as predictors of invasive aspergillosis in oncohematological patients

TaraskinaA.^1^ShadrivovaO.^1^,^2^BoykoI.^1^FrolovaE.^1^UchevatkinaA.^1^FilippovaL.^1^VasilyevaN.^1^1Kashkin Research Institute of Medical Mycology, North-Western State Medical University named after I.I. Mechnikov, Saint-Petersburg, Russian Federation2Department of Clinical Mycology, Allergy and Immunology, North-Western State Medical University named after I.I.Mechnikov, St. Petersburg, Russian Federation

**Objectives:** Fungi of the genus *Aspergillus* are widely distributed in nature and are relevant opportunistic pathogens for immunocompromised patients, causing a life-threatening infectious fungal complication — invasive pulmonary aspergillosis (IPA). Disruption of immune barriers associated with suppressive therapy, such as cytostatic polychemotherapy, can affect both the mechanisms of the innate immune response and acquired, in particular, the differentiation of T-helper cells (Th). The regulation of T-cell effector functions occurs due to cytokine-induced intracellular signaling and transactivation of transcription factors. Single nucleotide polymorphisms (SNPs) of cytokine genes that direct T cell differentiation can lead to their different expression and / or affinity for complementary receptors on immune cells, increasing the risk of infectious processes ***The aim of this study*** was to investigate gene variants of key protein-inductor of Th1 adaptive immune response as biomarkers of IPA and effect on expression of IP-10 in serum in oncohematological patients.

**Methods:** 178 oncohematological patients on the background of cytostatic polychemotherapy with symptoms of lung injury were recruited. 86 oncohematological patients (44,5%) either developed proven or probable IPA as defined by criteria of EORTC/MSG 2008 (median age 43y ±14, range [22-77y], 57% males) whereas controls (92 oncohematological patients (55,5%) without IPA comparable in age and sex) did not fulfill these criteria. Polymerase chain reaction-restriction fragment length polymorphism (PCR-RFLP) was used to determine the SNPs of rs458917, rs56061981 *CXCL10* gene, rs1800629 *TNFα* gene and rs2430561 *INFγ* gene. Chemokine IP-10 amount was determined by the enzyme-linked immunosorbent assay with the use of commercial ELISA kit sets (Cloud-Clone Corp, USA) and is presented in ng/ml serum. Statistical analysis was performed using SPSS21 (IBM, USA).

**Results:** The genotype and allele distribution of the study SNPs were no significant differences between oncohematological patients with probable IPA and without IPA. In the study we found that allele variants of G-135A (rs56061981) do not appear to affect serum IP-10 levels although the presence of allele A is associated with a slight increase in serum chemokine, while the presence of minor allele-1447G (rs4508917) is associated with significantly higher concentrations of IP-10 in serum.

**Conclusion:** The presence of the *CXCL10* -1447G allele in serum of oncohematological patients was associated with increased IP-10 protein expression. The expansion of the studied groups of patients will allow making conclusions about the prospect of typing SNPs as a predictive marker of the risk of pulmonary mycosis development.

### P368. In vitro phagocytosis of Aspergillus fumigatus conidia by chicken thrombocytes and heterophils

BigeardP.FilleulC.VahsenT.ZapataL.BotterelF.GuillotJ.ArnéP.RiscoV.Ea Dynamyc Upec, Université de Créteil, Créteil, France

**Objectives:** Avian respiratory diseases can be responsible for substantial economic losses to the poultry industry. This is particularly true for aspergillosis, which is pervasive in both domestic and wild avian communities, and is a common cause of respiratory distress and morbidity. Mammal immune response against pulmonary aspergillosis is characterized by an early interaction of *Aspergillus* conidia with phagocytic cells such as macrophages and neutrophils, but knowledge remains limited regarding the avian immune response after inhalation of *Aspergillus* conidia. Avian thrombocytes and heterophils (homologues to mammal neutrophils) are known for their phagocytic activity, but nothing has been described so far regarding their potential role during *Aspergillus* infection in birds. This study aimed to investigate the phagocytic activity of chicken thrombocytes and heterophils against *Aspergillus* conidia.

**Methods:** We used filtered GFP- or DsRed-expressing *A. fumigatus* conidia to inoculate primary cultures of heterophils or thrombocytes previously purified by Percoll’s discontinuous gradient density from whole blood of chicken. Cultures and conidia inoculation were performed on poly-L lysine treated Labtek® slides or in suspension. When slides were used, cultures were fixed at different time points to measure internalization rates by immunolabeling with goat anti-chicken CD45 (leucocytes) and anti-chicken CD41 (thrombocytes) antibodies. When cells were in suspension, they were inoculated with *Aspergillus* conidia for 30 min, fixed and analyzed by flow cytometry (BD Accuri® C6) using the antibodies above described. Finally, activation of phagolysosomal acidification was evaluated by lysosome staining using the Lysotracker® method.

**Results:** We obtained cultures with up to 95.3% pure thrombocytes while heterophils purification was less efficient (<50%) because of heterophil layer contamination with erythrocytes and thrombocytes during the purification and heterophil aggregation during culture. Nevertheless, identification of avian heterophils by morphology and CD45+ labeling demonstrated conidial internalization as soon as 15 min after culture inoculation. Conidial internalization rate reached its peak 30 min after inoculation, with 22-28% of thrombocytes containing at least one conidia, while heterophils showed up to 93% of internalization. Eight hours after inoculation, conidial swelling and germination was observed in almost all thrombocytes while internalized conidia in heterophils remained quiescent. Lysotracker® staining confirmed phagolysosome fusion in heterophils but not in thrombocytes containing conidia.

**Conclusion:** The results of the present study confirm for the first time the phagocytic capacity of both chicken heterophils and thrombocytes to *A. fumigatus* conidia, and suggest differences in internalization efficacy between these phagocytic cells. In thrombocytes, phagocytic activity was lower than that detected in heterophils, which allowed conidia swelling and germination inside the cells. Further studies shall determine whether survival inside thrombocytes might be used by *Aspergillus* as a strategy to ensure dissemination in birds.

### P369. Evaluation of the liposome properties in the development of immune response during an immunization protocol with C. albicans proteins loaded on DODAB:MO liposomes

PachecoI.^1^,^2^Costa-BarbosaA.^1^,^2^BarbosaA.M.^3^,^4^DiasM.^1^,^2^Carvalho-PereiraJ.I.^1^,^2^OliveiraM.E.C.D. Real^5^PaisC.^1^,^2^CorreiaA.^6^,^7^,^8^TorradoE.^3^,^9^SampaioP.^1^,^2^1Institute of Science and Innovation For Bio-sustainability (ib-s), University of Minho, Braga, Portugal2Centre of Molecular and Environmental Biology (cbma), Department of Biology, University of Minho, Braga, Portugal3Life and Health Sciences Research Institute (icvs), School of Health Sciences, University of Minho, Braga, Portugal4I3s, Instituto De Investigação E Inovação Em Saúde, University of Porto, Porto, Portugal5Centre of Physics (cfum), University of Minho, Braga, Portugal6Ibmc, Instituto De Biologia Molecular E Celular, University of Porto, Porto, Portugal7I3s, Instituto De Investigação E Inovação Em Saúde, University of Porto, Braga, Portugal8G Dgaot, Faculdade De Ciências, University of Porto, Porto, Portugal9Icvs/3b’s Pt Associate Laboratory, University of Minho, Guimarães, Portugal

**Objectives:** *Candida albicans* is a normal colonizer of the human body, persisting in a harmless state most of the time. However, given the opportunity, this species becomes one of the most important opportunistic pathogens, which increases the risk of developing systemic candidiasis. A promising antifungal strategy is the use of liposomal vaccine delivery systems. Previous studies reported that cationic liposomes prepared with DODAB:MO (1:2) and loaded with *C. albicans* cell wall surface proteins (CWSPs) were stable, non-cytotoxic and avidly phagocytized by macrophages. However, standardizing these liposomal formulations is challenging and complicated, since their wide range of characteristics must be taken into consideration according to the application we desire and the performance we expect. The liposome preparation method greatly influences these characteristics. Our main objective was to analyze the implications of the liposome preparation method on the biophysical properties of the DODAB:MO liposomes loaded with *C. albicans* proteins and evaluate the consequences at the immune response level, in order to develop a formulation that could be used in vaccination strategies against systemic *C. albicans* infections.

**Methods:** CWSPs from *Candida albicans* SC5314 were encapsulated in DODAB:MO (1:2) liposomes. The DODAB:MO liposomes were prepared using the thin lipid-film hydration method. The lipid film was hydrated with ultrapure water (1º experiment) or with HEPES-buffer and sonicated to reduce the vesicles’ size (2º experiment). For both, the final concentration of CWSPs was 50 µg/mL in 25mM of HEPES-buffer and the final total lipid concentration was 888 µg/mL. The mean size (nm), polydispersity (PDI) and surface charge (ζ-potential) were evaluated with the Malvern ZetaSizer Nano ZS (Malvern, UK) particle analyzer and the immunization procedure was carried as previously reported (Carneiro *et al*., 2015). The production of IFN-γ, IL-17, IL-4 and IL-10 in splenic CD4^+^ T cells was evaluated through flow cytometry. “*ex vivo”* cytokine production was evaluated through re-stimulation of the splenocytes with CWSPs and CWSP-specific antibodies were quantified by ELISA.

**Results:** Liposomes from the first experiment had a size around 666.23 ± 91,31 nm, with a PDI of 0.36 ± 0.08, and a ζ-potential of -2.30 ± 0.31 mV. Changing the liposome preparation method, we obtained smaller liposomes (249.69 ± 29.34 nm), with a PDI of 0.20 ± 0.06 and a ζ-potential of -8.53 ± 0.66. While IL-4 was the prevailing cytokine when taking into consideration the liposomes from the first experiment, the liposomes from the second experiment led to much higher production of IFN-γ and IL-17, which clearly shifted the immune response from a Th2 to a Th1/Th17 response. The liposomes from the second experiment also demonstrated a significant increase (10x) in CWSP-specific antibodies.

**Conclusion:** Our results indicate that the biophysical properties of DODAB:MO liposomes are important in the type of adaptative cellular response, and that in reducing significantly these liposomes sizes a shift from a Th2 to a Th1/Th17 response was observed, which is known to be more protective against systemic candidiasis. Therefore, with this liposome system, the liposome size has a huge impact in the immune response.

### P370. rThe Impact of NFAT Inhibition on Neutrophil Effector Functions, Antifungal Defense, and Myelopoiesis in Cyclosporine A-Treated and NFATc1LysM Mice and in Patients after Allogeneic Hematopoietic Stem Cell Transplantation

TeschnerD.^1^PleinK.^1^MichelC.^1^PrueferS.^2^BrosM.^3^BeckertH.^4^Wagner-DrouetE.M.^1^TheobaldM.^1^SchildH.^2^RadsakM.P.^1^1Department of Hematology, Medical Oncology, & Pneumology, University Medical Center of the Johannes Gutenberg-University, Mainz, Germany2Institute of Immunology, University Medical Center of the Johannes Gutenberg-University, Mainz, Germany3Department of Dermatology, University Medical Center of the Johannes Gutenberg-University, Mainz, Germany4Clinic For Pneumology, Ruhrland Clinic, Faculty of Medicine of the University of Duisburg-Essen, Essen, Germany

**Objectives:** Calcineurin inhibitors substantially contribute to the risk for opportunistic fungal infections in patients after allogeneic transplantation (HSCT). It is well known that the nuclear factor of activated T cells (NFAT) is an important transcription factor downstream of calcineurin especially in T cells. However, NFAT also seems to play a role in innate antifungal immune responses by polymorphonuclear neutrophils (PMN), as well as in regulation of myelopoiesis and myeloid differentiation.

**Methods:** Firstly, isolated PMN from CsA-treated patients after allogeneic HSCT (*n* = 17) and healthy donors (*n* = 8) were analyzed were analyzed *in vitro* and *ex vivo* regarding their different effector functions. Secondly, we used a murine IPA model (C57BL/6) and treated CsA-treated or LysM-specific NFATc1 knockout (NFATc1^LysM^) mice with *Aspergillus fumigatus* intratracheally. PMN recruitment to the lungs and pulmonary fungal clearance were examined by analyzing bronchoalveolar lavages (BAL) and peripheral blood (PB) using flow cytometry and cytometric bead array and murine lungs by fungal culture assays and histopathologic examination. Furthermore, survival was studied with neutropenic animals serving as positive controls. In addition, we investigated myelopoiesis and myeloid differentiation by quantifying bone marrow derived myeloid progenitor cells from CsA treated or NFATc1^LysM^ mice using flow cytometry and simultaneously counting PMN in PB under steady state conditions.

**Results:** CsA significantly enhanced phagocytosis of PMN *in vitro* and *ex vivo* in patients’ blood samples. Moreover, PMNs migratory capabilities were reduced *in vitro,* whereas other effector functions or release of IL-8 were rather unaffected. PMNs of CsA-treated patients showed increased activation, degranulation and production of inflammatory mediators, but production of ROS was slightly decreased. In our *in vivo* model, IPA was lethal in neutropenic mice whereas solely CsA or vehicle treated mice survived the infection. CsA treatment resulted in enhanced PMN recruitment in BAL by trend, while pulmonary inflammation and PMN counts in PB remained stable. Indeed, fungal clearance was clearly constrained in CsA treated animals. In our murine knockout model, NFATc1^LysM^ mice infected with *Aspergillus fumigatus* showed unimpaired survival without displaying detectable differences in PMN recruitment or fungal clearance. However, pulmonary inflammation and PMN counts in PB seemed to be more pronounced in knockout mice. Interestingly, BALs of CsA treated mice showed increased levels of IL-6 by trend but decreased levels of MCP-1 and TNF-α. In contrast, MCP-1, RANTES and TNF-α were enhanced by trend in BALs of NFATc1^LysM^ mice, while IL-6 was reduced compared to wild type controls. PMN counts in PB were unaffected in NFATc1^LysM^ mice but distribution of bone marrow derived murine myeloid progenitor cells was significantly impaired especially in megakaryocyte-erythroid progenitor cells, whereas solely CsA treatment had no influence.

**Conclusion:** Results from our *in vitro* and *ex vivo* studies on patients’ blood samples as well as from our murine *in vivo* IPA model indicate that NFAT regulates not only myelopoiesis, but also PMN functionalities in mice and humans. Nevertheless, these interactions are obviously multidimensional and potentially derive from involvement of different pathways. The underlying molecular mechanisms and clinical relevance of our findings in HSCT remain to be determined.

### P375. Novel assay for sterol 24-C-methyltransferase enzymatic activity, a primary antifungal target of Abafungin

NaruseH.ItoT.NoshiE.TanakaY.MakiH.SHIONOGI & CO., LTD., Osaka, Japan

**Objectives:** Abafungin is a broad-spectrum antifungal agent. It has been presumed that abafungin inhibits sterol 24-C-methyltransferase (SMT) from the report that abafungin decreased L-[Methyl-14C]methionine incorporated intracellular sterol. SMT is one of the enzymes involved in ergosterol biosynthesis pathway which is well-known antifungal target (e.g. azoles). As *erg6* gene encoding SMT is conserved in many fungal species, SMT could be a promising drug target. However, it is not known whether abafungin actually inhibits SMT enzymatic activity, and there is no high throughput SMT enzyme assay for drug discovery. Therefore, we developed a novel SMT enzymatic assay by homogeneous time-resolved FRET (HTRF). We also evaluated the effects of SMT inhibition on antifungal activity.

**Methods:** HTRF is based on a competitive immunoassay between SAH, byproduct of SMT reaction, and d2 labelled SAH (SAH-d2) towards Lumi4-Tb cryptate labelled anti-SAH antibody. *A. fumigatus* SMT and its substrates, lanosterol and SAM, were mixed and incubated at room temperature, then SAH-d2 and anti-SAH antibody were added. After incubation at room temperature, HTRF signal was measured. The HTRF ratio was calculated as a two-wavelength signal ratio (665 nm/620 nm). SMT inhibitory activity of abafungin and its derivatives were calculated as IC_50_ values. MICs of these compounds against *A. fumigatus* and *C. albicans* were measured according to the method of the CLSI.

**Results:** SAH was detected by 0.001 μM to 0.1 μM in the optimized condition and SMT produced SAH in a dose dependent-manner. SMT of microsomal fraction indicated 3-fold higher enzymatic activity than that of cytoplasmic fraction. In this HTRF assay abafungin inhibited SAH production and exhibited IC_50_ of 0.21 μM. The MICs of abafungin against *A. fumigatus* and *C. albicans* were 2 μg/ml and 0.0625 μg/ml, respectively. The derivatives without SMT inhibitory activity didn’t exhibit antifungal activity against *A. fumigatus* and *C. albicans*.

**Conclusion:** We established a novel assay to evaluate *A. fumigatus* SMT activity. Abafungin was revealed to inhibit SMT enzymatic activity, which led to antifungal activity. This result would help us develop new antifungals targeting SMT.

### P376. Metagenomic analysis and culture-dependent diagnosis of cystic fibrosis patient airways

KressM.R. Von Zeska^1^TonaniL.^1^FonsecaL.M.^1^AlmeidaO.G.G.^1^CapizzaniC.P.D.C.^1^MartinisE.C.P. De^1^TorresL.A.G.M.M.^2^1Faculdade De Ciencias Farmacéuticas De Ribeirao Preto, Universidade de Sao Paulo, Ribeirao Preto-SP, Brazil2Faculdade De Medicina De Ribeirão Preto, Universidade de São Paulo, Ribeirao Preto-SP, Brazil

**Case Report:** Cystic fibrosis (CF) is a debilitating autosomal recessive disease in which the patient carries a mutation in the Cystic Fibrosis Transmembrane Conductance Regulator (CFTR) gene, which encodes a chloride channel involved in electronic exchanges through the plasma membrane of epithelial cell types. Consequently, a defective lung mucociliary clearance occurs, producing thick and sticky bronchial mucus, suitable for entrap airborne bacteria and fungi. The culture-dependent diagnostics of CF patients airways show the frequent colonization by *Haemophilus influenzae*, *Staphylococcus aureus*, *Pseudomonas aeruginosa, Burkholderia cepacia* complex and atypical mycobacteria. Additionally, prolonged antibiotic treatments contribute to the colonization by multidrug-resistant pathogens such as *Achromobacter xylosoxidans* and *Stenotrophomonas maltophilia*. On the other hand, culture-independent technologies, as the metagenomic approach, revealed that the CF patients airways are not inhabited by these few pathogens, but by complex polymicrobial communities. Fungal species are usually found colonizing the CF patients airways (*e.g.Aspergillus fumigatus* and *Candida albicans*), however, few metagenomics studies are reported in the literature. Here, we show the culture-dependent identification and metagenomic approach (16S ribosomal DNA and the internal transcribed spacers (ITS) of the nuclear ribosomal DNA) of CF patient sputum. The detailed description of the CF lung microbiome is presented and compared with culture-dependent identification. The isolation of microorganisms on media culture from the sputum of the CF patient revealed the bacteria *Stenotrophomonas maltophilia, Pseudomonas aeruginosa, Achromobacter xylosoxidans* and *Staphylococcus aureus,* and the fungi *Aspergillus fumigatus* and *Candida albicans*. The identification was performed by classical and molecular methods, respectively. As the culture-based identification, the 16S and ITS metagenome revealed the bacteria *Stenotrophomonas* sp., *Pseudomonas putida*, *Achromobacter* sp. and *Staphylococcus aureus*, and the fungi *Aspergillus fumigatus* and *Candida albicans*. But metagenome additionally identified *Streptococcus* sp., *Neisseria* sp. and *Saccharomyces cerevisiae* in high relative abundance, and *Tectona grandis*, *Actinobacillus* sp., *Schizophyllum commune, Agaricales* order and *Halosphaeriaceae*family in intermediate and low relative abundance. Thus, the culture-based analyses correlated qualitatively with the relative abundance of data on obtained bacterial and fungal taxa. However, additional identifications were obtained by metagenome data. Here we observed that the taxonomic identification by Regarding 16S and ITS metagenome to the species level were not achieved for the fungal kingdom and the estimation of the relative abundance of species in the community is imprecise. This is justified by the primer-based amplicons sequencing method which has a limited ability to resolve this taxonomic identification. Thus, although we have identified several species colonizing the CF patients airways, the metagenome shotgun approach is indicated to provide information about the composition of the community up to the level of clonal complexes.

### P377. Mechanisms of resistance to elevated copper in the pathogenic mold Aspergillus fumigatus

MeirZ.^1^SandovskyH.^1^ShadkchanY.^1^MasratiG.^2^WiemannP.^3^Ben-TalN.^2^KellerN. P.^3^OsherovN.^1^1Clinical Microbiology and Immunology, Tel Aviv University, Tel Aviv, Israel2Biochemistry & Molecular Biology, Tel Aviv University, Tel Aviv, Israel3Medical Microbiology and Immunology, University of Wisconsin, Madison, United States of America

**Objectives:** *Aspergillus fumigatus* is the most common opportunistic mold pathogen in the growing immunocompromised population. Patients with weak immune systems are at risk to develop Invasive Aspergillosis (IA), a deadly type of infection with high mortality rates caused by *A. fumigatus*. Fungi use unique pathways to control the levels of essential metals inside the cell. Copper is a transition metal essential for all living systems and its homeostasis in *A. fumigatus* under copper excess is dependent on the copper sensing transcription factor AceA and its downstream target, the copper transporter CrpA. Studies suggest that genes participating in copper homeostasis can be promising drug targets. Our goals were to reveal the detailed function of the copper transporter CrpA and to identify and characterize additional participants acting in the Cu-resistance pathway of *A. fumigatus* and assess their relevance to virulence.

**Methods:** To achieve these goals we constructed a model structure of *A. fumigatus* CrpA, in collaboration with Prof. Nir Ben-Tal, and identified evolutionarily conserved fungal-specific domains in the protein. We generated GFP-tagged CrpA and studied its subcellular localization. We performed RNAseq analysis of *A. fumigatus* exposed to high copper levels and deleted selected upregulated genes. Finally, we performed a screen to identify genes that provide Cu-resistance when overexpressed.

**Results:** Significant progress was made in the localization of CrpA protein. We located the protein mostly in the ER but also in the membrane. We also created an inducible overexpression strain of CrpA using the xylose-inducible promoter *xylP* and showed that CrpA overexpression confers increased copper resistance when exposed to copper *in vitro* but not *in vivo*. We generated deletion strains for six genes that were up-regulated in WT vs *AceA*-null strains following exposure to copper as identified by RNA-seq. These genes are chaperones and metal-binding proteins that may participate in *A. fumigatus* copper resistance. The phenotypes of these deletion strains will be described. We performed a genetic screen using an overexpression library in which *A. nidulans* was transformed with the AMA-1-based overexpression genomic library and plated on excess Cu. We found four new genes that confer resistance to *A. nidulans*

**Conclusion:** Our results provide a more detailed picture of copper resistance in *A. fumigatus*, indicating that is dependent on the combined activities of the CrpA copper exporter, Cu-chaperones and accessory stabilizing proteins.

### P378. Genotyping of Cryptococcus spp. isolated from HIV-negative patients with Neurocryptococcosis

NascimentoE.^1^BariãoP.H.G.^2^KressM. Von Zeska^2^MartinezR.^1^1Department of Internal Medicine, Medical School /University of Sao Paulo/Ribeirao Preto, Ribeirão Preto, Brazil2school of Pharmaceutical Sciences of Ribeirao Preto, University of Sao Paulo, Ribeirão Preto, Brazil

**Objectives:** The aim of this study was to determine the species and molecular types of *Cryptococcus* spp. isolates from patients not co-infected with HIV from the Medical Hospital of Ribeirao Preto - Medical School of Ribeirão Preto - University of Sao Paulo – Brazil, from 2000 to 2017, and correlate with the comorbidity of the patients.

**Methods:** A total of 43 strains of *Cryptococcus* spp. isolated from 43 patients not co-infected with HIV were genotyped. The species were determined by PCR with the primers CN70 and CN49. The molecular types *C. neoformans* VNI, VNII, VNIII and VNIV, and *C. gattii* VGI, VGII, VGIII and VGIV were determined by PCR-RFLP with the amplification of the *URA5* gene and subsequent enzymatic restriction with *HhaI* and *Cfr13I*. The identification of *C. gattii* and *C. laurentii* was confirmed by the sequencing of the rDNA ITS regions.

**Results:** From a total of 43 clinical isolates, 28 were identified as *C. neoformans*, 13 as *C. gattii* and 2 as *C. laurentii*. Among *C. neoformans* clinical isolates, 27 were identified with the molecular type VNI and 1 VNII. Additionally, 2 *C. gattii* VGI and 11 *C. gattii* VGII were identified. The correlation between species and patient comorbidity revealed 28 clinical cases (Group 1) of patients not coinfected with HIV but with other underlying diseases (*e. g.* diabetes, adenocarcinoma, chronic lung disease, transplants, systemic lupus erythematosus and others) and 15 clinical cases (Group 2) of immunocompetent patients. Most of the isolates identified in Groups 1 were *C. neoformans* (75%) and in Group 2 were *C. gattii* (47%). *C. laurentii* were isolated from the Group 2.

**Conclusion:** Most of the clinical isolates were identified as *C. neoformans* and were more present in individuals not co-infected with HIV but who had another underlying disease. On the other hand, *C. gattii* is more frequent in the group of immunocompetent patients. The molecular types VNI and VGII were the most identified in our study, which corroborate with other studies carried out in Southeast Brazil.

### P379. Classification of dermatophytes by a multilocus phylogenetic approach based on Tef-1α, beta tubulin and ITS genes.

SacheliR.^1^ToméC.^1^AdjeteyC.^1^DarfoufR.^1^PalmeiraL.^2^HayetteM.-P.^1^1Clinical Microbiology- Nrc For Mycoses, University Hospital of Liège, Liège, Belgium2Human Genetics, University Hospital of Liège, Liège, Belgium

**Objectives:** Identification of dermatophytes to the species level is epidemiologically, ecologically and therapeutically significant. The use of phylogenetic species concepts based on rDNA internal transcribed spacer *(ITS*) regions have improved the taxonomy of dermatophytes. However, it has been shown that for some species, confirmation and refinement using other genes are needed. Indeed, there are problems with accurate definition and characterization of dermatophytes especially among the *Trichophyton mentagrophytes* series. Intra- and interspecies variations of the translation elongation factor 1-α (*Tef-1α*) gene were evaluated as a new identification marker in a wide range of dermatophytes. The aim of this study was to evaluate the discriminatory power of a phylogenetic tool based on concatenated sequences of three genomic regions including *Tef-1α, ITS (ITS1* to *ITS2)* and *beta-tubulin* to differentiate: 1/ the anthropophilic *Trichophyton interdigitale* species from the zoophilic *Trichophyton mentagrophytes* 2/ the two closely related African anthropophilic species *Trichophyton violaceum* from *Trichophyton soudanense.*

**Methods:** 26 well characterized strains of *T. interdigitale/mentagrophytes* have been selected including 3 IHEM reference strains; 30 well characterized strains of *T. soudanense/T.violaceum* including 2 IHEM reference strains were also included. All strains were submitted to *ITS, beta-tubulin* and *Tef-1α* PCR/sequencing. Moreover, identification was completed by real time PCR DermaGenius^®^ (PathoNostics). Alignments were performed using MUSCLE in Seaview. The alignments for each of the three genes were then concatenated together based on the name of the sample and gaps were added between genes. Phylogenies were then inferred on the concatenated alignment using PhyML.

**Results:** After generation of the phylogenetic tree by concatenation of the three sequence genes, the differentiation between *T. interdigitale* and *T. mentagrophytes* was clear. The 16 strains of *T. interdigitale* were well classified into one distinct group on the dendrogram. The 10 strains of zoophilic *T. mentagrophytes* were well distinct of the anthropophilic group and defined by another clade on the dendrogram. Reference strains were correctly classified into the two previously defined groups. Regarding the differentiation between *T. soudanense* and *T. violaceum*, the concatenation permitted to define two well distinct clades on the dendrogram. One clade containing the 10 *T. violaceum* tested and the other containing the 20 *T. soudanense* included in the study. Reference strains were correctly classified into each corresponding clade.

**Conclusion:** The concatenation of *ITS, beta-tubulin* and *Tef-1α* genes sequences allows the generation of a discriminating dendrogram between the zoophilic *T. mentagrophytes* and the anthropophilic *T. interdigitale* and between the two closely related African anthropophilic species *T. violaceum* and *T. soudanense.* These species are not clearly well defined by *ITS* analysis only. This multilocus phylogenetic approach allows to better define the species boundaries between these dermatophytes species and facilitates the molecular characterization of these species in routine diagnostic.

### P380. rPulmonary mycobiome of patients with suspicion of respiratory fungal infection – an exploratory study

OliveiraM.^1^,^2^PintoM.^3^VerissimoC.^4^SabinoR.^2^1Animal Biology Department, Faculty of Sciences of the University of Lisbon, Lisbon, Portugal2Reference Unit For Parasitic and Fungal Infections, Infectious Diseases, National Health Institute Dr. Ricardo Jorge, Lisbon, Portugal3Bioinformatics Unit, Infectious Diseases, National Health Institute Dr. Ricardo Jorge, Lisbon, Portugal4Infectious Diseases, Reference Unit for Parasitic and Fungal Infections, National Health Institute Dr. Ricardo Jorge, Lisbon, Portugal

**Objectives:** This pilot study aimed to characterize the pulmonary mycobiome of patients with suspicion of fungal infection of the respiratory tract as well as to identify potentially pathogenic fungi colonizing/infecting their lungs.

**Methods:** A cohort of 10 patients was analyzed, including HIV+ patients and patients with active infection caused by *Mycobacterium* species. Their respiratory samples (bronchoalveolar lavage fluid/ bronchial secretions) were pre-treated with lyticase and proteinase K; DNA was extracted using the High Pure PCR Template Preparation kit following the manufacturer’s instructions. The internal transcribed spacer region 1 (ITS1) and calmodulin gene were amplified by PCR and the resulting amplicons were sequenced using the Illumina MiSeq platform with pair-end reads of 150 bp. The obtained results were analyzed using the PIPITS pipeline as described by Gweon *et al.* [1]. Operational taxonomic units (OTU) to which less than 0.1% of the total reads attributed were disregarded.

**Results:** Thirty-seven different OTU were identified from which two belonged to the *Plantae* kingdom, 11 had less than the 0.1% threshold of the total reads and were therefore disregarded. The remaining 24 different OTU (grouped in 17 phylotypes), were considered as part of the pulmonary mycobiome of patients. Two phyla were identified: *Basidiomycota* (33.3%) and *Ascomycota* (54.2%). Regarding the Basidiomycota phylum, reads were classified in three classes (Agaricomycetes, Tremellomycetes and Walleomycetes), while for the Ascomycota phylum four different taxonomical classes were identified: Pneumocystidomycetes, Dothideomycetes, Eurotiomycetes and Saccharomycetes, with the latter being the most frequent class. Twelve fungal genera were identified, being *Candida* the most frequently detected. The median number of fungal genera detected in patients’ pulmonary mycobiome was six (ranging from two up to nine). The genus *Papilotrema* and the potentially pathogenic genera *Cryptococcus* and *Pneumocystis* were exclusively found in the pulmonary mycobiome of HIV+ patients. Other potentially pathogenic fungi such as *Aspergillus* spp., *Trichosporon* spp., *Saccharomyces* spp. and *Schizophyllum* spp. were also detected.

**Conclusion:** This pilot study illustrates how the pulmonary mycobiome is rich and highly variable in patients with fungal infections. The obtained results suggest that the described metagenomic analysis may possess a great ability to quickly and effectively detect potentially pathogenic fungi in the mycobiome of patients, making it a promising future diagnostic tool. Thus, further optimization, standardization and clinical validation of these NGS methodologies should be warranted in the future. Reference: Gweon HS, Oliver A, Taylor J, Booth T, Gibbs M, Read DS, Griffiths RI, Schonrogge K. PIPITS: an automated pipeline for analyses of fungal internal transcribed spacer sequences from the Illumina sequencing platform. Methods Ecol Evol. 2015 Aug;6(8):973-980.

### P382. Comparative genomic analysis of Scedosporium species and identification of pathogenicity determinants.

DuvauxL.^1^,^2^ShillerJ.^3^VandeputteP.^2^BahutM.^4^LemaireC.^1^MeyerW.^5^OyantL. Hibrand Saint^4^RenouJ.P.^3^CamB. Le^1^BoucharaJ.-P.^2^GasteboisA.^2^1Irhs-ecofun, INRA, Beaucouzé, France2Geihp, Université d’Angers, Angers cedex, France3Irhs-bio-info, INRA, Beaucouzé, France4Sfr-quasav-anan, Université Angers, Beaucouzé, France5Molecular Mycology Research Laboratory, University of sydney, Sydney, Australia

**Objectives:** *Scedosporium* species are soil saprophytes that may cause a large variety of infections in human. Especially, some of them are capable to chronically colonize the lungs of patients suffering from cystic fibrosis (CF) which constitutes a major risk of disseminated infection in case of lung transplantation. Molecular and biochemical analysis revealed that the genus *Scedosporium* comprises at least ten distinct species with differences in their susceptibility to antifungals and in their pathogenicity. Among them, three species, *S. boydii, S. apiospermum* and *S. aurantiacum* are predominant in CF patients, while other species like *S. minutisporum* are occasionally encountered in this clinical context. Conversely, *S. dehoogii* has never been reported from respiratory secretions of CF patients whereas some environmental studies revealed its common occurrence in soils where it accounts for up to 40% of all *Scedosporium* isolates. The aim of our project was to identify by a comparative genomic approach, regions of the genome or genes that might be responsible for the ability of *Scedosporium* species to establish within the respiratory tract of CF patients and to cause chronic colonization of the airways.

**Methods:** Five clinical or environmental isolates from each of the five species *S. boydii, S. apiospermum, S. aurantiacum, S. minutisporum,* and *S. dehoogii* were sequenced. To identify candidate proteins that may enable *Scedosporium* species to establish within the respiratory tract of CF patients, we first searched clusters of orthologous proteins conserved in all isolates of the pathogenic species, but absent in *S. dehoogi* isolates. Genomic data were also analyzed searching for genes present in all pathogenic species, as well as in *S. dehoogii,* but exhibiting in this last species point mutations potentially leading to a loss of function.

**Results:** At the beginning of the project, the whole genome of a first strain of *S. apiospermum sensu stricto* (Vandeputte *et al.*, 2014) and of *S. aurantiacum* (Pérez-Bercoff *et al.*, 2015) were already available. The whole-genome sequencing project allowed us to obtain a total of 2.4-8.9 mega-reads per isolate which permitted good quality draft genome assemblies. For 3 species, we did *de novo* sequencing, genome assembly and performed comparative genomic analysis with the 25 strains. Finally, comparative genomics allowed us to identify a series of 134 genes lost or with a potential loss of function in *S. dehoogii* but still functional in all other species.

**Conclusion:** This comparative genomic analysis of the main *Scedosporium* species allowed us to identify a set of candidate genes potentially involved in the adaptative mechanisms developed by pathogenic *Scedosporium* species to establish within the respiratory tract of patients suffering from CF. Functional genomic approaches focused on the genes here identified will now be developed to elucidate their role in pathogenesis. Acknowledgment This project was financed by Angers University with the AAP-CS program.

### P383. Molecular phylogenetic study of strains morphologically identified as Exophiala dermatidis from clinical and environmental specimens in Japan

AlimuY.TanakaR.BanS.YaguchiT.Division of Bio-resource, Medical Mycology Research Center, Chiba University, Chiba, Japan

**Objectives:** With support from the National Bio-Resource Project, at the Medical Mycology Research Center (MMRC), Chiba University, the collection, storage and distribution of pathogenic fungi are carried out mainly on clinical isolates. The molecular phylogenetic position of the isolates, the mycological characterization, the genomic and the clinical information are managed and added to the database to increase the value of the stock and lead to an increase in the number of aliquots. Since old isolates are morphologically identified and conserved, their identifications are reviewed as needed based on the base sequence of the ITS region of rDNA. We report here on *Exophiala dermatitidis*, which is an important pathogen of deep-skin dermatomycosis, it has been reconfirmed with identification and fitted with the latest classification. *E. dermatitidis* has been reported to have a molecular phylogenetically polymorphism in strains from South America. However, the investigation of the distribution in Japan has not been reported. The aims of this study, is investigate the phylogenetic distribution of *E. dermatitidis* in Japan.

**Methods:** In this investigation, we examined 66 clinical and environmental isolates morphologically identified and conserved as *E. dermatidis* previously, which were stocked in Bioresource Management Office in MMRC. The identification based on the ITS region. The sequences were aligned and compared through neighbor-joining phylogenetic tree analyses on the ITS1 region, using the Molecular Evolutionary Genetics Analysis (MEGA) version 7.0.26 software package. Furthermore, the strains of different gene types will test for mycological characterization such as the growth temperature, the growth rate, and the drug sensitivity.

**Results:** The examined 66 clinical and environmental isolates were composed of 47 ones from Japan, 4 ones from USA, 5 ones from Brazil, 6 ones from Venezuela and 4 ones from China. According to establish a phylogenetic tree for the ITS1 region, we knew that 45 of 47 isolates from Japan, 2 of 4 ones from China, 2 of 6 ones from Venezuela and all of 4 ones from the USA were classified to the gene type A1. Only 2 of Japan and China‘s isolates belonged to the gene type A2, respectively. Interestingly, all of the isolates from Brazil were part of the gene type B and most of the Venezuela samples were close to the gene type C. The relationship between the types and their mycological characterizations was examined.

**Conclusion:** In our study, it is confirmed that, in South American strains, polymorphisms were found molecularly within species, and molecular phylogenetic diversity of the isolates in Japan was relatively uniform.

### P384. Investigation of the impact of amino acid changes in the substrate binding site of fungal sterol 14α-demethylase on protein structure and function using Saccharomyces cerevisiae as an expression model.

LacknerM.^1^KeniyaM.V.^2^MonkB.^2^1Division of Hygiene and Medical Microbiology, Medical University of Innsbruck, Innsbruck, Austria2Sir John Walsh Research Institute, University of Otago, Dunedin, New Zealand

**Objectives:** Mucormycetes are intrinsically short-tailed azoles resistant. We used the high resolution X-ray crystal structure of *S. cerevisiae* lanosterol 14α-demethylase (LDM) in complex with voriconazole (VCZ) to postulate that the amino acid substitution Y129F in *Rhizopus arrhizus* LDM (RaLDM) F5 is associated with their intrinsic resistance. Our aim was to experimentally test this hypothesis.

**Methods:** Full-length recombinant C-terminal hexahistidine-tagged LDM (F1 and F5) of *R. arrhizus* were overexpressed from the *PDR5* locus in a *S. cerevisiae* host lacking 7 drug efflux pumps. Their cognate NADPH-cytochromeP450 reductase (RaNCP) was overexpressed from the *PDR15*. Phenotypic and biochemical analysis were used to determine structural and functional features of RaLDMF1, RaLDMF5, without and with co-expression of RaNCP.

**Results:** RaLDMF1 and F5 were functionally overexpressed in *S. cerevisiae*. RaLDMF5 appeared to be the major contributor to azole resistance, with its expression resulting in a 32x higher VCZ minimal inhibitory concentration (MIC) than the recipient strain. Co-expression of RaLDMF5 with NCP resulted in additional 4-fold increase of VCZ MIC when compared with RaLDMF5 single expression. MICs for long-tailed azoles were unaffected by the LDM supplementation. *S cerevisiae* co-expressing RaLDMF1 with NCP had similar MIC values as the recipient strain. Azole-susceptibility patterns of *S. cerevisiae* co-expressing RaLDMF5 and RaNCP closely matched those of *R. arrhizus*. Phenotypic analysis of recombinant LDMs expressed in *S. cerevisiae* provides insights on the relevance of mutations located in the substrate binding pocket. This system allows assessment of effects on the drug target without interference from drug efflux pathways.

**Conclusion:** LDMF5 was found to be the dominant isoform in *R. arrhizus* that confers short-tailed azole resistance. Biochemical and structural analysis of the purified protein are expected to provide deeper understanding of the observed phenotypes.

### P385. A metagenomic and whole genome sequencing comparative approach to study the impact of azole therapy on chronic colonisation with Aspergillus fumigatus during cystic fibrosis.

GangneuxJ.P.^1^VandenborghtL.-E.^2^BidonB.^3^BelleguicC.^4^NeuvilleE. De^4^GueganH.^1^LeroyS.^5^LaviolleB.^4^HorsleyA.^6^DenningD.^7^CuomoC.^8^BoucharaJ.-P.^3^DelhaesL.^9^PaponN.^10^1Laboratoire De Parasitologie-mycologie, CHU de Rennes, Rennes, France2Genoscreen, Lille, France3CHU d’Angers, Angers, France4CHU de Rennes, Rennes, France5CHU de Nice, Nice, France6University Hospital of South Manchester, Manchester, United Kingdom7University of Manchester, Manchester, United Kingdom8Broad Institute of MIT and Harvard, Cambridge, United States of America9Parasitology Mycology, Univeristy of Bordeaux, Centre de Recherche Cardio-Thoracique de Bordeaux, U1045, F-33000, Bordeaux, France, Bordeaux, France10Ea Geihp, UFR Pharmacie d’Angers, Angers, France

**Objectives:** The impact of early azole therapy during cystic fibrosis (CF) remains debated. The ATCF (Antifungal Therapy during Cystic Fibrosis) is a longitudinal phase III study with the following objectives: - to assess the microbiological and clinical effects of azole therapy: negativation of sputum cultures for *Aspergillus*, course of FEV1, clinical signs and quality of life, and the impact on co-prescription of antibiotic courses and/or steroid therapies. - to study the genomic impacts of azole treatment on the evolution of micro- and mycobiota and on genomic evolution of *Aspergillus* isolates.

**Methods:** The ATCF study followed CF patients with a chronic colonisation with *A. fumigatus* and randomized to receive an early oral azole therapy with itraconazole (ITRA) or voriconazole (VORI) for 4 to 6 months in a phase III study. A long-term follow-up was conducted until 2 months after end of treatment (EOT). All sputa were submitted to DNA extraction for further metagenomic approach to determine the fungal (ITS2) and bacterial (16S) microbiota. Besides, twenty isolates of *Aspergillus fumigatus* recovered before and after treatment were sequenced with a shotgun strategy.

**Results:** Eleven patients were included and followed until 2 months after EOT. All assessable patients had a successful outcome, with a negativation of sputa, an increase in FEV1 and a decrease in the number of antibiotic/steroids co-prescriptions. However, 2 months after EOT, most of clinical assessment criteria were similar to the baseline values. Fungal diversity increased under treatment with a large decrease of the more abundant fungal taxa, particularly *Aspergillus*. However, 2 months after EOT the fungal diversity returned to baseline values and *Aspergillus* reappeared, mostly in a larger proportion. Besides, *Streptococcus, Pseudomonas* and *Staphylococcus* were the main genera detected and their proportion increased under azole therapy. The bacterial communities were similar after EOT compared to before. The analysis of micro – and mycobiota must be further documented. All SNPs and InDels variants were determined for 20 isolates. Isolates from a single sputum and more generally from a single patient during treatment were closely related. By contrast, isolates obtained 2 months after EOT were far different suggesting reinfections with new genotypes. In particular, one azole resistant *A. fumigatus* was recovered with a TR34/L98H mutation of the *CYP51A* gene, suggesting an environmental origin rather than an acquired resistance under azole drug pressure.

**Conclusion:** When a chronic colonisation with *A. fumigatus* is associated with clinical deterioration, EOT can temporarily reduce airway infection and inflammation, and extend periods of stable disease status. While only a limited number of patients were included, the longitudinal phase III design of ATCF study combined to a genomic approach underlines the need for azole therapy rationalization during CF. Indeed, the reversible effects 2 months after the EOT and the limited tolerance of both ITRA and VORI encourages short courses of therapy driven by the clinical improvement. The longitudinal changes of the mycobiota were restored 2 months after EOT and genetic characterization of isolates showed that reinfection with new genotypes is more frequent than *Aspergillus* in host evolutions under azole treatment.

### P386. Role of Candida glabrata candidate genes in echinocandin tolerance and resistance development

Garcia-RubioR.^1^ClearA.^1^HealeyK.R.^2^ShorE.^1^PerlinD.S.^1^1Center For Discovery and Innovation, Hackensack Meridian Health, Nutley, United States of America2Department of Biology, William Paterson University, Wayne, United States of America

**Objectives:** *Candida glabrata* is the second most prevalent bloodstream fungal pathogen in North America and Europe, and its prevalence is rising. Echinocandin class compounds are first-line agents for the treatment of these infections, but therapeutic failures are increasingly encountered. However, the reasons why *C. glabrata* shows elevated potential to develop echinocandin resistance, relative to other *Candida* sp., are currently not well understood. Although echinocandins are fungicidal drugs, multiple cellular mechanisms can stabilize *C. glabrata* during drug exposure and promote drug tolerance, allowing cells to survive long enough to acquire mutations leading to stable clinical drug resistance. Interestingly, genomic analyses of different *C. glabrata* clinical isolates have revealed a high level of genetic diversity both within the same MLST sequence type (ST) clade and between them. Depending on the population subset, genomic plasticity may be responsible for differences in tolerance phenotype. The objective of our research is to elucidate the factors involved in promoting echinocandin drug tolerance in *C. glabrata.*

**Methods:** Because echinocandins target the fungal cell wall, we have focused on interrogating the role of cell wall integrity pathway components in promoting echinocandin tolerance. We have used an *in vitro* killing assay developed in our group to measure tolerance over a range of micafungin concentrations (0.016-32 µg/ml). Multiple knock-out mutants (*∆slt2*, *∆snf1*, *∆rlm1*, *∆sbe2*, *∆mid2*, *∆cka1*, *∆cbk1*, *∆ypk2*, *∆slg1*, *∆yps1*, *∆ada2*, *∆mot3*, *∆gas1*, *∆rta1*, *∆chs5*, *∆crz1*, *∆pdr1*, *∆ssd1*, *∆sbe22*, *∆chs3*) were tested in this assay looking for significant changes in tolerance phenotypes. Additionally, to identify genetic polymorphisms that may be influencing echinocandin tolerance, we have sequenced some of the most relevant cell wall integrity pathway genes, particularly focusing on those whose deletion was found to affect drug tolerance in the killing assay.

**Results:** Most of the knock-out mutants showed no or minor changes in echinocandin tolerance. However, three mutants – *∆yps1*, *∆ypk2* and *∆slg1* – resulted in significantly reduced tolerance to micafungin. These genes were sequenced in a number of *C. glabrata* echinocandin sensitive clinical isolates. Different polymorphism combinations were found in these strains, with specific polymorphisms being characteristic of particular STs. Several of these genetic polymorphisms resulted in amino acid changes.

**Conclusion:** Changes in micafungin tolerance phenotype were found in mutants lacking certain cell wall integrity pathway genes, implicating them in promoting drug tolerance in *C. glabrata* and representing potential targets to limit development of antifungal drug resistance. Further studies will evaluate whether polymorphisms in these genes are responsible for variation in drug tolerance among clinical strains.

### P387. Genetic modification of opportunistic pathogenic Mucoromycotina species using a plasmid free CRISPR/Cas9 system

NagyG.^1^,^2^SzebenyiC.^1^,^2^VazA.^1^,^2^JágerO.^1^,^2^IbragimovaS.^1^GuY.^3^IbrahimA.S.^3^VágvölgyiC.^1^PappT.^1^,^2^1Deapartment of Microbiology, University of Szeged, Szeged, Hungary2MTA-SZTE “Lendület” Fungal Pathogenicity Mechanisms Research Group, Szeged, Hungary3Los Angeles Biomedical Research Institute at Harbor-UCLA Med Center, Torrance, United States of America

**Objectives:** Most fungal species, which can cause human infections belong to the genera *Candida* and *Aspergillus*, but in the recent years, the number of infections caused by Mucoromycotina species (e.g., *Rhizopus oryzae*, *Lichtheimia corymbifera* and *Mucor circinelloides*) has significantly increased. The properties and potential virulence factors that make the Mucoromycotina species enable to cause disease (mucormycosis) in patients, are not known. Potential virulence factor genes may play role in fungal dimorphism, proteolytic enzyme production, iron uptake, and production of cell surface proteins.

**Methods:** Genetic manipulation of Mucoromycotina species, based on homologous recombination still can be a great challenge. In this study, a plasmid free CRISPR/Cas9 system was used for the genetic manipulation of *Mucor*, *Lichtheimia* and *Rhizopus* species. PEG-mediated protoplast transformation was used to introduce the Cas9 enzyme and the synthesized gene-specific gRNA with or without a deletion cassette into the fungus.

**Results:** In case of *Lichtheimia* and *Rhizopus*, the plasmid free CRISPR/Cas9 system was successfully used to disrupt the *pyrG* gene by NHEJ. Our results suggested that the disruption of the *pyrG* gene has no effect on the growth of fungal strains. These strains and the developed methods will be suitable for further transformation procedure in the future.

**Conclusion:** Based on the potential virulence factor genes (*cotH*, *svf*, *cyp51*, *hsbA*), a mutant library has been constructed from *M. circinelloides.* We have started to analyse the function of these genes in the pathogenesis and azole resistance of *Mucor*. This study was supported by the “Lendület” Grant of the Hungarian Academy of Sciences (LP2016-8/2016) and the Hungary grant 20391-3/2018/FEKUSTRAT of the Ministry of Human Capacities.

### P388. Mycobiome and microbiome in chronic fungal rhinosinusitis

DellièreS.^1^AngebaultC.^1^,^2^FieuxM.^3^BonfilsP.^3^PodglagenI.^4^WoertherP.-L.^2^,^5^DannaouiE.^4^BotterelF.^1^,^2^1Mycology and Parasitology, Henri-Mondor, Créteil, France2Ea Dynamyc Upec, Université de Créteil, Créteil, France3Ent, HEGP, Paris, France4Microbiology, HEGP, Paris, France5Microbiology, Hôpital Henri-Mondor, Créteil, France

**Objectives:** Fungal ball rhinosinusitis is the main type of non-invasive fungal rhinosinusitis, presenting as a dense mass of mycelium obstructing the sinus. Despite fungal hyphae being observed on direct examination of biopsy samples, culture remains negative in more than 60% of cases.^1^ The aim of this study was to evaluate the performances/efficacy of targeted metagenomics to describe and analyze bacterial and fungal microbiota in fungal balls compared to other types of sinusitis and find possible microbial co-occurrence/associations intra or extra kingdom.

**Methods:** A total of 46 sinus biopsies were studied from 46 patients who underwent surgery for chronic rhinosinusitis from 2015 to 2017 in Paris, France. Direct examination of biopsies was performed by calcofluor and culture was performed on Sabouraud media. DNA was extracted and fungal ITS1 and ITS2 loci and bacterial V3-V4 16S ribosomal DNA locus were amplified and sequenced (MiSeq^TM^ Illumina, V2 kit, 500 cycles). To analyze the microbial diversity, operational taxonomic units were defined via QIIME and assigned to SILVA and UNITE databases for 16S and ITS, respectively. Statistical analyses were performed using SHAMAN. These data were compared to microbiological and clinical data.

**Results:** Among these 46 patients, 38 had fungal balls and 8 had non-fungal chronic rhinosinusitis and were considered as controls. Among the 38 fungal balls, direct examination and culture were positive for fungi in 95% and 30% of biopsies, respectively. By contrast, targeted metagenomic analysis of the 38 fungal balls yielded one or more fungal genera in 100% of cases, with *Aspergillus* spp. alone or in combination with other fungi in 92.1% (35/38) of patients. Control samples (*n* = 8) generated only a limited number of fungal reads, mainly from the genus *Malassezia* (4/8). Overall, the main bacterial genera found by targeted metagenomics were *Haemophilus* (20.1%), *Staphylococcus* (14.6%), *Pseudomonas* (7.66%) and *Enterobacter* (5.84%). Using principal coordinate analysis (Bray-Curtis distance; permanova test), we found significantly different bacterial and fungal metagenomic profiles in patients with fungal balls compare to controls (p = 0.047 and p<0.001, respectively) (Figure 1). One bacterial taxon, *Haemophilus*, was significantly over-represented in patients with fungal balls compared to controls (p<0.001). 

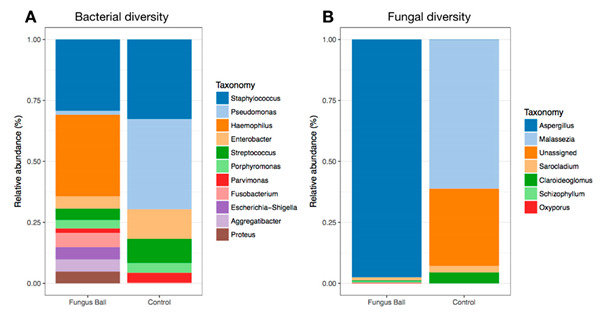



**Conclusion:** Targeted metagenomics allowed identification of fungal genera responsible for chronic rhinosinusitis when the culture remains negative. *Aspergillus* was the most commonly fungus identified. The genus *Haemophilus* appeared statistically associated with fungal balls. Fungal balls pathogenesis could result from microbial interactions between fungi and bacteria in mixed biofilm-like structure. 1. Jiang, R.-S., Huang, W.-C. & Liang, K.-L. Characteristics of Sinus Fungus Ball: A Unique Form of Rhinosinusitis. Clin Med Insights Ear Nose Throat (2018).

### P389. From swollen to germination; a transcriptomic approach in Aspergillus fumigatus

Perez-CuestaU.GuruceagaX.Ramirez-GarciaA.Abad-Diaz-De-CerioA.HernandoF.L.RementeriaA.Immunology, Microbiology and Parasitology, University of the Basque Country (UPV/EHU), Leioa, Spain

**Objectives:** The germination is the first adaptive process that *Aspergillus fumigatus* must overcome to colonize the environment or infect animals. The *A*. *fumigatus* germination process involves a first step known as swollen and the subsequent hyphae elongation. Morphologically, both are well-characterized processes but molecularly there are only a few studies describing the transition from swollen to germination in the literature. This study, therefore, focuses on the transcriptomic analysis of the genes differently expressed between swelling conidia and early hyphae output to understand the mechanisms involved in these steps.

**Methods:** The *A*. *fumigatus* reference strain Af293 was incubated at 37°C without agitation in RPMI supplemented with 5% fetal bovine serum for 4.5 and 6 hours to obtain swelling conidia or early hyphae respectively. Total RNA was extracted and maintained in RNA later until use. Finally, RNA samples were studied using the AWAFUGE microarray (Agilent Whole *A*. *fumigatus* Genome Expression 44K v.1) designed by our research group.

**Results:** The transcriptomic analysis detected 158 and 256 genes overexpressed in the swelling and early hyphal stages, respectively. GO enrichment analysis showed that transport (13.2%), organelle organization (11.9%) and regulation (10.7%) were the most common categories in the swelling stage, and transport (10.2%), regulation (10.2%) and filamentous growth (7.4%) in early hyphal stage. Unfortunately, only 36% and 53.5% of the overexpressed genes had known molecular function in swelling or hyphal stages, respectively. Among them, the oxidoreductase (17.6%), hydrolase (8.8%) and transferase (8.8%) were the most usual activities detected in swelling stage, while hydrolase (14.1%), oxidoreductase (10.2%) and transferase (6.3%) activities were the most abundant in early hyphal stage. According to component ontologies, the most overexpressed genes in the swelling conidia codified proteins located in mitochondrion (24.5%), membrane (23.3%) and nucleus (13.8%), while the ones in the early hyphae stage codified proteins of the membrane (13.7%), extracellular region (5.9%) and plasma membrane (4.7%). On the one hand, in the swelling step highlights the primary metabolism being the production of energy the principal priority. In addition, the regulation of glutamate homeostasis as well as the iron metabolism may also play a crucial role. On the other hand, in hyphal output, an overexpression of genes implicated in vitamins and cofactors production, calcium regulation and cell wall were detected.

**Conclusion:** This work might enable a better understanding of the germination process of *Aspergillus fumigatus* from a transcriptomic point of view. This project could involve important advances in the knowledge of the ecology and the mechanisms of adaptation of the fungus that could be exploited by both the biotechnological and the biosanitary sector.

### P390. rGenotyping of azole resistance in Candida auris

LiJ.^1^CosteA.T.^1^BachmannD.^1^SanglardD.^1^LamothF.^2^1Microbiology Institute, University Hospital Lausanne, University of Lausanne, Lausanne, Switzerland2Infectious Diseases Service, CHUV, Lausanne, Switzerland

**Objectives:** Since 2009, *Candida auris* has emerged as a multidrug-resistant yeast pathogen in all continents. Its ability to cause nosocomial outbreaks with high mortality rate has attracted worldwide attention. Four distinct geographic and genetic clades have been described: I: South Asia (India, Pakistan), II: East Asia (Japan, South Korea); III: South Africa; IV: South America (Venezuela, Colombia). Remarkably, rapid development of fluconazole resistance is a hallmark of most *C. auris* isolates. However, mechanisms of azole resistance and type of genetic mutations still remain unclear. Hotspot mutations in the target gene (*ERG11*) are classical cause of azole resistance in *Candida spp*, but other mechanisms exist, such as overexpression of drug transporters induced by gain-of-function mutations in transcription factors like *TAC1* and *MRR1*, both of which are important regulators of efflux systems in *Candida albicans*. Nevertheless, *TAC1* and *MRR1* have not been studied in *C. auris* yet. The objective of the study was to analyze the genotype of these genes in *C. auris* isolates from different clades and with different levels of azole resistance.

**Methods:** Twenty-two *C. auris* strains from India, Japan, Israel, Colombia and Switzerland were collected. Susceptibility to fluconazole (FLC) and voriconazole (VOR) was tested according to CLSI protocol. Minimal inhibitory concentration (MIC) thresholds for resistance were defined according to arbitrary cut-offs previously reported by Lockhart *et al*.: MIC ≥32 µg/mL for FLC and ≥2 µg/mL for VOR. Entire sequencing of *ERG11*, *TAC1* and *MRR1* was performed for all 22 isolates.

**Results:** By antifungal susceptibility testing, 2 isolates were classified as azole susceptible (MIC FLC 4 µg/mL and VOR ≤1 µg/mL), 13 as resistant to FLC only and 7 as resistant to both FLC and VOR. *ERG11* sequencing revealed three hotspot mutations that have already been described in *C. auris* (F126T, Y132F and K143R). These isolates (*n* = 8) exhibited high level resistance to FLC and variable VOR susceptibility. In addition, we identified two new hotspot mutations: V125A from Israeli and Swiss strains (n = 3) with high resistance to both FLC and VOR, and F444L from Colombian strains (n = 4) with high resistance to FLC and variable VOR susceptibility. Putative *TAC1(QG37_07465)* sequencing uncovered twenty mutations possibly associated with azole resistance (i.e. present in azole-resistant isolates, but not in susceptible ones): S89Y, E200K, F214L/S, R215K, K225N, K247E, I268V, Q298K, T346I, Q503R, F580L, P595H, P607S, S611P, A640V, Y647C, I772-, N773- and L774M. Compared to *ERG11* mutants exhibiting high FLC MIC (≥64 µg/mL) and relatively high VOR MIC (≥1 µg/mL), *ERG11* wild-type isolates harboring *TAC1* mutations exhibited intermediate level of FLC resistance (MIC: <64 µg/mL) and low VOR MIC (<1 µg/mL). Putative *MRR1(QG37_07783)* sequencing unveiled three mutations: N167K, G280R and N647T. *MRR1* mutations were always associated with *ERG11* mutations and their role in azole resistance thus remains unclear.

**Conclusion:** Overall, we identified two new mutations in *ERG11* (V125A and F444L) within conserved hotspot regions. Moreover, we identified twenty mutations in *TAC1-like* that were associated with intermediate level of fluconazole resistance in the absence of *ERG11* mutations. Their putative role in azole resistance deserves further investigations.

### P391. Comparison of phenotypic and genotypic methods of speciation and gene sequencing of Malassezia from the skin samples in a tertiary care hospital

RomaldN.^1^KindoA.J.^2^VeeraragavanM.^3^1Department of Microbiology, Sri Ramachandra Institute of Higher Education and Research, Chennai, India2Microbiology, Sri Ramachandra Medical College and Research Institute, SRIHER, Chennai, India3Dermatology, Sri Ramachandra Medical College and Research Institute, Chennai, India

**Objectives:** Speciate *Malassezia* phenotypically. Confirm the phenotypic identification through molecular method of speciating *Malassezia* by PCR and Gene Sequencing. Comparison between phenotypic and genotypic result.

**Methods:** *Malassezia* species was isolated from the skin scrappings of patients with hypo/hyper pigmented lesions. Microscopic Examination was done with 10%KOH. Samples showing meat ball and spaghetti appearance were cultured onto Sabourauds Dextrose Agar (SDA), SDA with olive oil overlay (SDA-O) and Modified Dixon’s agar (MDA) containing chloramphenicol and incubated at 32^o^ C upto 3 weeks. *Phenotypic Identification* Was done based on the colony growth characteristics, gram staining, urease test, catalase test, bile esculin with tween 60 hydrolysis and tween assimilation. *Molecular Identification* The isolates which were cultured and identified by phenotypic methods were confirmed by Molecular methods DNA was extracted by Phenol: Chloroform method. Extracted DNA was amplified by Polymerase chain reaction (PCR) using thermocycler *Primers used Pan-fungal PCR primers*:- PCR amplification of ITS1 and ITS4 regions ITS1 5΄ TCC GTA GGT GAA CCT GCG G 3΄ and ITS4 5΄ TCC TCC GCT TAT TGA TAT GC 3΄ *Malassezia* specific nested primers Pan fungal PCR positive samples were subjected to species specific nested PCR using *Malassezia* specific primer ITSIF-N 5΄GGATCATTAGTGATTGCCTTTATA 3΄ and ITS4-R 5΄ TCCTCCGCTTATTGATATG 3΄ **Agarose Gel Electrophoresis** was done and band obtained PCR products were sequenced by **Sanger’s sequencing** and **NCBI blast** was done and species identified.

**Results:** *Phenotypic method of speciation of Malassezia* Out of 31 samples 7 isolates were *Malassezia furfur* 12 isolates were *Malassezia sympodialis* 9 samples grew Malassezia globosa 3 samples grew *Malassezia obtusa* MOLECULAR IDENTIFICATION *Pan-fungal PCR* PCR by Pan fungal primers ITS 1 and ITS 4 shows band between 600-1000bp (fig). All samples showing positive band was subjected to *Malassezia* specific nested PCR. *Malassezia* specific nested PCR *Malassezia* specific nested PCR using *Malassezia* specific primers showed band at 200-300bp.(fig) **Sequence result(table)**

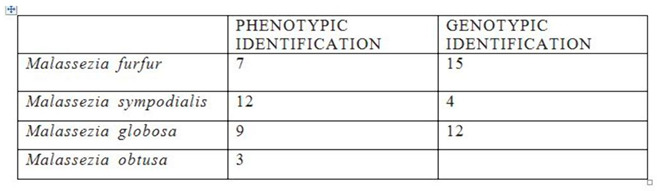

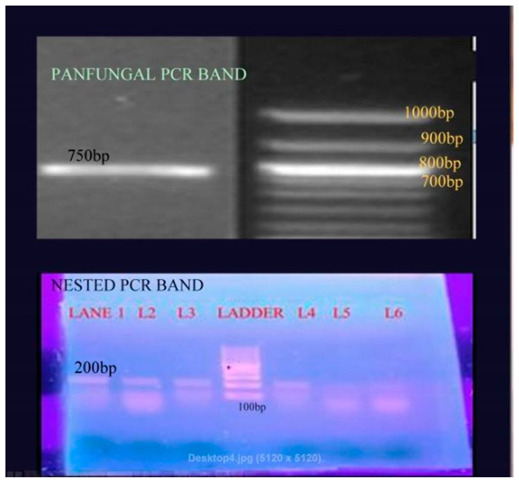





**Conclusion:** Phenotypic speciation showed *Malassezia sympodialis* as the predominant species Genotypic speciation showed *Malassezia furfur* as the predominant species and the least was *Malassezia sympodialis*. Genotypic speciation is more confirmatory than phenotypic method Speciation of *Malassezia* is important because of variation in resistant pattern to anti-fungals among different species.

### P392. First molecular characterization of Uruguayan Cryptococcus spp clinical isolates.

CabezaE.ArtetaZ.BetancorL.Montevideo, Hygiene institute, Faculty of medicine, University of the Republic, Montevideo, Uruguay

**Objectives:** To characterize Uruguayan *Cryptococcus spp* isolates from meningoencephalitis by PCR fingerprinting and MLST.

**Methods:** Clinical isolates Our laboratory receives clinical samples from fungal infections. From the analyzed period, between 1th of January of 2000 and 31th of december of 2015, 55 strains of *Cryptococcus spp* were selected to be studied. Phenotypic identification was performed in all cases. 52 *C. neoformans complex* and 3 *C.gattii complex*, were recovered from cerebrospinal fluid (CSF) samples (n = 51), bornquioloalveolar lavage (n = 2), blood culture (n = 1) and pleural effusion (n = 1). Characterization by PCR fingerprinting Molecular type was determined by PCR fingerprinting as previously described (Meyer 1993). The specific sequence used was the one from wild-type M13 minisatelite phage (5-GAGGGTGGCGGTTCT-3). PCR fingerprint patterns were assigned according to the main bands of the band pattern of the reference strains. MLST (Mutilocus sequence type) and phylogenetic analysis. MLST analysis was performed by the individual application of the six housekeeping genes CAP59, GPD1, LAC1, PLB1, SOD1 and URA5 along with the IGS1 region according as previously described (Meyer 2009). The allele types and the sequence type (ST) were identified via sequence comparisons with the *Cryptococcus* MLST database. The allelic profile of the strains and the global dataset were applied to the goeBURST algorithm in PHILOVIZ software. Clona complex (CC) was defined when the strain had no more than one difference in housekeeping gene allele. Statistical analysis X2 and Fischer’s exact test were performed for categorical variables and student’s T test for continuous variable.

**Results:** Molecular types of a total of 55 clinical isolates, molecular types were identified in 50, in the 5 remaining the pattern of bands is reproducible but not concordant with the patterns of the references strains. Three of them corresponded to *C.gattii complex.* 28 (56,0%) isolates were *C.neoformans var grubii*, VNI (now *C. neoformans*), 16 (32,0%) were *C.neoformans var neoformans*, VNIV (now *C. deneoformans*), 6 (12 %) were VNIII AD hybrids. The strains that were not identified by PCR fingerprinting were studied by MLST. MLST (Mutilocus sequence type) and phylogenetic analysis. 20 strains selected by their genetic variability and phenotipic characterization were analyzed by MLST. 7 different allele were identified for CAP59, 4 for GPD1,11 for LAC1, 7 for PLB1, 5 for SOD1, 7 for URA5 and 8 for IGS1 region. A total of 16 ST were observed, 13 *C. neoformans complex* isolates and 3 STs for 3 *C. gattii complex* isolates (Table 1). of the 13 STs corresponding to *C. neoformans complex*, 8 were not reported until now and were included in the database (ST605, ST606, ST607, ST608, ST609, ST610, ST611 and ST612). The more frequent ST were ST32 (n = 3), ST23 (n = 2), ST63 (n = 2).

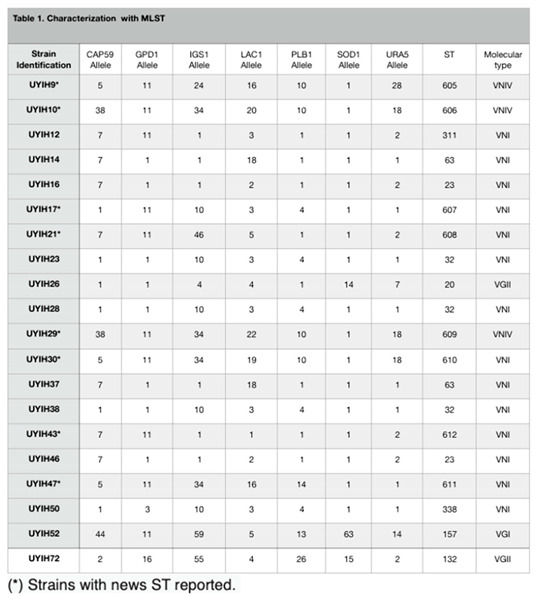



**Conclusion:** This is the first molecular characterization of Uruguayan isolates of *Cryptococcus spp* from cases of meningoencephalitis. We demonstrated the great genetic variability of circulating *Cryptococcus spp* strains in our environment that cause meningoencephalitis. The new STs reported are closely related with each other and with the rest of the STs, although they did not belong to the same CC.

### P393. Identification of genetic mediator of fluconazole tolerance in Candida albicans

DelarzeE.^1^TrachselE.^1^BrandtL.^1^DEnfertC.^2^SanglardD.^1^1Institute of Microbiology, Lausanne University Hospital and University of Lausanne, Lausanne, Switzerland2Pasteur Institute, Paris, France

**Objectives:** Fluconazole (FLC) resistance in *Candida albicans* has long been reported and is involving several well described molecular resistance mechanisms. FLC resistance is based on measurement of MIC values and threshold values have been provided to distinguish between wild-type susceptible isolates and resistant isolates. Another phenomenon, called FLC tolerance (known also as “trailing effect” is susceptibility assays) is characterized by the ability of *C. albicans* to grow persistently at supra-MIC drug concentrations without acquisition of drug resistance. Little is known about molecular mechanisms behind FLC tolerance and we aimed here to undertake identification of genetic mediators of FLC tolerance.

**Methods:** We used in this study a collection of 579 *C. albicans* strains, each of which could overexpress a specific *C. albicans* gene in a tetracycline-dependent manner and was tagged by a distinct sequence barcode. The whole collection was pooled in RPMI and, after repeated subcultures under FLC pressure at 32 µg/ml for 5 consecutive days in RPMI with 2% glucose, the relative population of each barcode was determined by barcode sequencing. In addition, each barcoded strain was subjected to single tolerance assays yielding tolerance indexes reflecting relative growth of strains at a single drug concentration (8 µg/ml FLC) after 24 h growth in RPMI in 2% glucose (growth conditions similar to EUCAST susceptibility assays).

**Results:** Combining this genetic selection with single isolates testing, two transcription factors (*CRZ1* and *GZF3*) and a protein kinase (*YCK2*) were confirmed to be positive mediators of FLC tolerance when overexpressed. *CRZ1* s part of the calcineurin pathway that is known critical for *C. albicans* in response to antifungal stress, while *GZF3* and *YCK2* are less well characterized. Using overexpression of these genes in deletion mutants, we showed that *GZF3* acts probably upstream of *CRZ1*. *CRZ1* and *GZF3* were deleted in azole-susceptible clinical strains but exhibiting different levels FLC tolerance. Our results demonstrate that *CRZ1* plays a critical role in FLC tolerance in these strains while *GZF3* deletion has minor effects. Consistent with these data, transcriptional profiles of clinical strains in the presence of FLC at supra-MIC drug concentrations was reminiscent of a calcineurin-like transcriptional response.

**Conclusion:** A genetic screen permitted the identification of mediators of FLC tolerance, one of which (*CRZ1*) is known to be part of the calcineurin pathway. FLC tolerance of clinical strains is dependent on the presence of at least *CRZ1*. The precise mechanisms of FLC tolerance mediated by *CRZ1* and the other identified genes still need further investigations.

### P394. Genotypic characterization of Trichosporon sp. from a Tertiary care centre in South India

PremamaliniT.^1^RajyoganandhS.V.^2^VeenaH.^2^VijayakumarR.^2^KindoA.J.^2^RungmeiS.K.M.^3^SridharanK.S.^2^VijayalakshmiB.^4^1Microbiology, Sri Ramachandra Medical College and Research Institute, SRIHER, chennai, India2Microbiology, Sri Ramachandra Medical College and Research Institute, SRIHER, Chennai, India3Microbiology, SG-PGI Lucknow, Lucknow, India4Infecious Disease Consultant, SIMS, Chennai, India

**Objectives:** To determine the species distribution of the *Trichosporon* sp. based on genotypic characterization. To assess the genetic relatedness of the most commonly isolated *Trichosporon asahii* strains using RAPD primers GAC1 and M13.

**Methods:** Seventy two *Trichosporon* clinical isolates recovered from patients of SRMC & RI, a few hospitals in Chennai, and a hospital in Lucknow, were considered for the study. PCR was performed initially with *Trichosporon* genus specific primers to double check for accurate identification of this genus. Genotypic characterization was done by PCR amplification of rRNA gene sequences by *T. asahii* specific primers which identified only *T. asahii* isolates. Species confirmation was done by IGS1 sequencing and phylogenetic analysis. Maximum parsimony clustering was used for construction of phylogenetic tree. Its stability was assessed by parsimony bootstrapping with 1,000 pseudo replications. Phylogenetic analysis was done by comparing the obtained nucleotide sequences with the nucleotide sequences of *Trichosporon* reference strains obtained from the GenBank database. DNA fingerprinting of the *T. asahii* isolates was carried out by RAPD analysis, using primers GAC-1 and M13.

**Results:** Distribution of sample source of the 72 isolates were as follows: 43 (59.7%) urine, 12 (16.7%) blood, 7 (9.7%) Percutaneous nephrostomy (PCN) tube, 5 (6.9%) respiratory isolates, 4 (5.6%) pus, and 1 (1.4%) peritoneal dialysate fluid. *Trichosporon* specific PCR confirmed all the 72 isolates as belonging to this genus*. Trichosporon asahii* specific PCR identified 65 isolates as *T. asahii* (Fig. 1). The remaining 7 isolates were identified further by phylogenetic analysis of the IGS1 sequences. In phylogenetic analysis, six representative *T. asahii* clinical isolates clustered together with *T. asahii* KR265116.1 and *T. asahii* KR872659.1 (bootstrap ≥95), confirming the molecular identification results. One isolate identified as *Trichosporon* species clustered in the same clade (bootstrap ≥79) was found to be closely related to *T. ovoides*. All these isolates belonged to the Ovoides clade. Two isolates clustered along with *T. loubieri* AB066428 & *T. loubieri* KT936593.1 (bootstrap ≥99). They belonged to the Gracile clade. The remaining 4 isolates clustered together in Cutaneum clade along with *T. cutaneum* AB 066415.1 (bootstrap ≥47) (Fig. 2). Finally, the 72 *Trichosporon* clinical isolates were identified as 65 *Trichosporon asahii*, 4 *Trichosporon cutaneum*, 2 *Trichosporon louberi* and one *Trichosporon* sp. closely related to *T. ovoides*. DNA fingerprinting of the mostly commonly isolated *T. asahii* (64/72) isolates carried out by RAPD analysis, identified more heterogeneity among the *T. asahii* isolates by GAC1 than M13 primer.

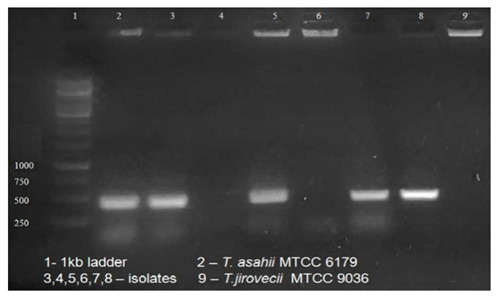

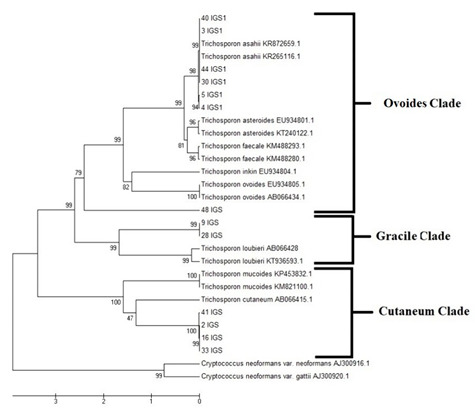





**Conclusion:** *Trichosporon asahii* is the most common species causing human trichosporonosis, though other members of the genus may also be involved. The members of the genus *Trichosporon* are most frequently under-reported due to inconsistency of the phenotypic methods used routinely in most laboratories. *Trichosporon* infections when treated with fluconazole or caspofungin, which is commonly used to treat yeast infections, results in poor clinical outcome. Hence, correct identification of the *Trichosporon* species using molecular methods, helps the clinician in providing appropriate treatment.

### P395. The lung mycobiome and the domestic environment

Rubio-PortilloE.^1^OrtsD.^2^LlorcaE.^2^FernándezC.^3^FerrerC.^4^GálvezB.^5^EstebanV.^6^RevellesE.^4^Pérez-MartínC.^4^Gómez-ImbernónE.^4^AdsuarJ.^4^PiquerasP.^4^AmatB.^5^AntónJ.^1^ColomM.F.^4^1Physiology, Genetics and Microbiology, University of Alicante, San Vicente del Raspeig, Spain2Pneumology, Hospital General Universitario de Elda, Elda, Spain3Pneumology, Hospital General Universitario de Alicante, Alicante, Spain4Medical Mycology Lab, University Miguel Hernandez, Sant Joan d’Alacant, Spain5Pneumology, Hospital Universitario del Vinalopó, Elche, Spain6Pneumology, Hospital Clínico Universitario de Valencia, Valencia, Spain

**Objectives:** The **goal** of the study was to assess the relationship between the fungal mycobiome detected in samples from the Lower Respiratory Tract (LRT) of non -infectious patients, and the mycobiota detected in the air and dust of their dwellings.

**Methods:** The mycobioma of the LRT was analysed by the study of Bronchoalveolar Lavage (BAL) samples taken from patients with indication of bronchoscopy. After informing and getting their consent for the study, they were asked about the possibility of a domestic sampling that was scheduled. Negative controls were included for every laboratory procedure and samples from the bronchoscopes were also taken as controls. All samples were processed for DNA extraction and for cultures. The fungal Internal Transcribed Spacer (ITS2) of the extracted DNA was amplified and sequenced by the illumina system. Results of the massive sequencing were analyzed by QIIME 1.8.0 and compared with the ones in the UNITE Database. NCBI nucleotide database was also used for identification. Cultures of samples were incubated at room temperature and 37ºC and the fungal isolates were studied for identification.

**Results:** Forty-six BAL, 4 samples from bronchoscope and 19 domestic environmental (MA) samples, together with a negative control from the PCR were submitted to massive sequencing analysis (Solexa/Illumina system). The first results yielded over 7000000 sequences (5060431 from BAL, 108178 from bronchoscopes and 10861275 from MA samples). In order to filter contaminants, we used a negative control from PCR and three negative controls from each sequencing run. Samples were clustered in different fungal Operational Taxonomic Units (OTUs) at 99% identity and sequences clustered in OTUs present in control samples were remove from the analysis. After contaminant filtering, we obtained 2840808 clean sequences belonging to 122 different fungal Operational Taxonomic Units (OTUs) at 97% identity. The similarity among different microbial communities was assessed using phylogenetic information. Among the fungal species detected by massive sequencing, 6 OTUs were detectable in more than 75% of the BAL and 75% of the MA samples. These sequences corresponded to *Cladosporium angustisporum*; *Candida orthopsilopsis*; *Rhodotorula mucilaginosa*; *Cryptococcus neoformans sensu stricto*; *Naganishia liquefaciens* and *Malassezia restricta*. Most of these species could be detected also by culture in the domestic environment, but almost none in BAL samples cultures. When comparing the mycobiome of the LRT of patients with the one of their domestic environment, important coincidences can be seen for specific species of *Candida, Cryptococcus* and *Malassezia*.

**Conclusion:** The results show an important similarity between the mycobiota of the environment and the mycobiome of the LRT samples of the patients. Most of the fungal species detected in both environments correspond to fungi which are important opportunistic pathogens like *Cryptococcus neoformans* and *Candida orthopsilosis*.

### P396. Identification of Mycotoxigenic Fungi from Grains in a Nigerian Region Using the Modern Polyphasic Methodology.

OkekeO.F.I.^1^FapohundaS.O.^2^SoaresC.^3^LimaN.^4^AyanbimpeG.M.^1^1Medical Microbiology, University of Jos, Plateau State, Nigeria2Bioscience and Biotechnology, Babcock University, Ogun State, Nigeria3Centre of Biological Engineering, Micoteca da Universidade do Minho, University of Minho, Campus of Gualtar, Braga, Portugal4Centre of Biological Engineering, Micoteca da Universidade do Minho, University of Minho, Campus of Gualtar, Braga, Portugal

**Objectives:** Mycotoxins are poisonous substances produced by mycotoxigenic fungi which contaminate agricultural commodities. Many foods and feeds can become contaminated with mycotoxins since they can form in commodities before harvest, during the time between harvesting and drying, and in storage. Mycotoxins may also be carried over to animal products due to consumption of contaminated feed. Maize (*Zea mays*) and guinea corn (*Sorghum bicolor*) form a major staple of the study area and are high risk commodities for contamination with mycotoxigenic fungi. This study was carried out to identify mycotoxigenic fungi from maize and guinea corn in Plateau state, north-central of Nigeria.

**Methods:** In a multistage sampling technique, markets for the study were selected. Using simple random sampling method, 270 samples were collected from various sampling points within selected markets where these grains are sold. Isolation and identification of the mycotoxigenic fungi employed the modern polyphasic methodology for filamentous fungi identification.

**Results:** The mycotoxigenic fungi *Aspergillus*, *Fusarium*, and *Penicillium* were isolated. Further work on these mycotoxigenic fungal genera identified them as follows: *A. niger*, *A. aculeatus*, *A. tamari, A. flavus*, *F. verticillioides* and *F. equiseti*. The *Penicillium* isolate was identified to belong to the section Sclerotiora and closely related to *P. malochii*. *Aspergillus* accounted for 75% of all mycotoxigenic fungi isolated while *Fusarium* and *Penicillium* accounted for 23% and 2% respectively.

**Conclusion:** Further work is also ongoing to determine if our strain of *Penicillium* is a *P. malochii* or an entirely novel species. Our findings indicate contamination of maize and guinea corn in the study area with mycotoxigenic fungi which may pose serious health risks for the human and animal population and also have implications for food safety and public health in Nigeria. In view of this, further investigation is necessary to assess the health risks linked with the consumption of these grains. Key words: Mycotoxigenic fungi, grains, Nigeria

### P397. Regulation of DNA repair machinery and Ras pathway in Paracoccidioides brasiliensis during oxidative stress

BatistaW.^1^NavarroM.^2^CastilhoD.^2^CaladoJ.^2^ConceiçãoP.^1^CastroB.^1^SilvaR.^2^ChavesA.^2^1Pharmaceutical Sciences, Universidade Federal de São Paulo, São Paulo, Brazil2Microbiologia E Imunologia, UNIFESP, São Paulo, Brazil

**Objectives:** Paracoccidioidomycosis is a granulomatous disease caused by fungi of the genus *Paracoccidioides* and widely distributed in Latin America. The disease has high disabling potential and yet is still absolutely neglected. These fungi undergo cell differentiation induced by changes in temperature and this event is critical to the ability to cause disease. The knowledge of which molecular patterns of the fungus may be true virulence factors is still limited by the difficulties inherent to genetic manipulation of these fungi. Studies that have tried to understand these molecular aspects rely on the method of transformation mediated by *Agrobacterium tumefaciens.* To overcome these barriers, we need to understand the mechanisms of DNA repair used by the fungus. This is because the establishment of a knockout strain depends on the occurrence of homologous recombination events. The aim of the present communication was to evaluate the role of the DNA repair machinery of *P. brasiliensis* in response to oxidative stress.

**Methods:** *P. brasiliensis* yeast cells were treated with different concentrations of H_2_O_2_, in the presence or absence of pharmacological inhibitors (Ras, Hog-1 or DNA ligase 4 pathways). We used the RBD-GST (Ras Binding Domain fused with GST) probe to detect the Ras activation and western blot to evaluate the Hog-1 phosphorilation levels. Gene expression was analyzed by qRT-PCR. Cell viability were assessed by MTT assay or cell counting.

**Results:** The double-strand breaks repair pathways communicate with the Ras GTPase and Hog1 Map kinase pathways. The genes *PbRAD51* and *PbRAD52* are active elements during DNA repair and participate in the response to oxidative stress in *P. brasiliensis.* In addition, inhibition of the non-homologous end joining (NHEJ) mediated repair pathway has been observed to provide a protective response to *P. brasiliensis* yeasts during a normally lethal oxidative stress condition for the fungus. Perhaps, this event is related to the greater efficiency of the repair by homologous recombination induced by the inhibition of NHEJ. Finally, it was observed that the combination of the damping of the NHEJ pathway, through the inhibition of DNL4 or GSK3, together with the induced oxidative stress do increase the efficiency of transformation processes in yeasts of *P. brasiliensis*. Indeed, inhibitors of NHEJ pathway caused a protective effect on yeast cells challenged with high concentration of H_2_O_2_. This effect correlates with an increase in expression of scavenger genes like catalase and superoxide dismutase.

**Conclusion:** Taken together, our results indicate that there is a significant crosstalk between two major cellular damage response pathways, ROS signaling and DNA repair, for *P. brasiliensis* survival.

### P400. rMatched-paired analysis of patients treated for invasive mucormycosis - standard treatment vs. posaconazole new formulations (MoveOn)

Salmanton-GarciaJ.^1^SeidelD.^1^KöhlerP.^1^MellinghoffS.^1^WisplinghoffH.^2^VehreschildJ.-J.^1^CornelyO.A.^1^VehreschildM.J.G.T.^1^1Department I of Internal Medicine, University Hospital of Cologne, Cologne, Germany2Antibiotic Resistance, Molecular Epidemiology, Labor Wisplinghoff, Cologne, Germany

**Objectives:** Current first-line (1st) antifungal treatment for invasive mucormycosis (IM) consists of liposomal amphotericin B (AMB). Salvage (SAL) treatment options are limited and often based on posaconazole oral suspension (POS*susp*). However, with the approval of posaconazole new formulations (POS*new*), patients could benefit from improved pharmacokinetics, safety and tolerability. Therefore, our aim is to assess the effectiveness of POS*new* as first-line and SAL treatments for IM.

**Methods:** We performed a case-matched analysis with proven or probable IM patients from the FungiScope® Registry. 1st-POS*new* and 1st-AMB+POS*new* cases were matched with 1st-AMB-based treatment controls, and SAL-POS*new* cases were matched with SAL-POS*susp* controls. Each case was matched with up to three controls based on severity, haematological/oncological malignancy, surgery and/or renal dysfunction.

**Results:** Five patients receiving first-line POS*new* alone, 18 receiving first-line POS*new* combined with AMB, and 22 receiving salvage POS*new* were identified. By day 42, favourable response was reported for 80.0% (n = 4/5) of patients receiving first-line POS*new*, for 27.8% (n = 5/18) receiving first-line POS*new* plus AMB, and for 50.0% (n = 11/22) receiving salvage POS*new*. Day-42 all-cause mortality of patients receiving POS*new* was lower compared to mortality in their respective controls (20.0% (n = 1/5) in 1st-POS*new vs.* 53.3% (n = 8/15) in 1st-AMB; 33.3% (n = 6/18) in 1st-AMB+POS*new vs.* 52.0% (n = 26/50) in 1st-AMB; 0.0% (n = 0/22) in SAL-POS*new vs.* 4.4% (n = 2/45) in SAL-POS*susp*).

**Conclusion:** In the observed patients, POS*new* was effective in terms of treatment response and associated mortality of IM. POS*new* may be an alternative for treatment of IM.

### P401. Efficacy of Celecoxib solely or in combination with Itraconazole in the treatment of murine paracoccidioidomycosis

BurgerE.^1^SantosL.A.^1^GrisoliaJ.C.^1^DiasN.A.^1^PaulaF.B.D.A.^2^OliveiraA.M. De^3^RodriguesA.M.^4^CamargoZ.P.D.^4^1Department of Microbiology and Immunology, Federal University of Alfenas, Alfenas, Brazil2Department of Clinical and Toxicological Analysis - Pharmaceutical Sciences Faculty, Federal University of Alfenas, Alfenas, Brazil3Laboratory of Pathology - José Do Rosário Vellano University, UNIFENAS, Alfenas, Brazil4Department of Microbiology, Immunology and Parasitology, Federal University of Sao Paulo, Sao Paulo, Brazil

**Objectives:** Our objective is to evaluate the effectiveness of Celecoxib, of Itraconazole and both, as a combined treatment for paracoccidioidomycosis, a highly prevalent granulomatous mycosis in Latin America using a murine model.

**Methods:** Mice were intraperitoneally infected with a virulent *P. brasiliensis* isolate and three days later, daily administration by gavage of either 3mg/mL Itraconazole (I), or 6mg/mL Celecoxib (C) or both 3mg/mL Itraconazole and 6mg/mL Celecoxib (I+C) was initiated and kept for 15 days. Controls included infected, non-treated mice (Pb) and non-infected mice. Body weight and survival rate were monitored daily. At 7 and 15 days of infection, delayed type hypersensitivity (DTH) was evaluated and serum was obtained for specific anti-*P. brasiliensis* IgG antibody determination. At the last day of treatment, the mice were sacrificed and spleen, lungs, liver and epiploo/pancreas were collected and their macroscopic characteristics were analyzed. Following, the organs were either processed and stained with H/E for general histology, or macerated, for quantification of viable fungi.

**Results:** There was no difference in the body weight of infected, treated or uninfected controls and there were no deaths registered during this early phase of the infection in mice from any group. In all experimental groups, DTH decreased from day 7 to day 15 and, in contrast, specific IgG titers increased from day 7 to day 15. But At 15 days, both, DTH reactivity and IgG titers were the highest in the Pb group, lower in the I group and in the C group, and the lowest in the I+C group. This decrease in both cellular and humoral specific immunity in the groups most treatment-covered may be explained by the elimination of viable *P. brasiliensis* by the drugs, rendering a full immune response less necessary. The histological aspects demonstrated that treatment with I+C was the one that reduced the most the number of lesions and the severity of the granulomatous response in all organs. When analyzing the control of fungal growth in the tissues, both mice from the I and from the C groups showed less fungi scattered in the tissue than mice from the Pb group; however the lowest numbers of fungi were found in mice from the I+C group. In the epiploo, which is the organ of shock for this model of *P. brasiliensis* infection, the animals of the I+C group had the fewest fungi with preserved morphology. The same was found in terms of viable fungi, as the number of CFUs was lower in all organs of treated mice, and more markedly in those receiving the combined I+C therapy.

**Conclusion:** We showed the effect of the antifungal drug Itraconazole and also of the anti-inflammatory drug Celecoxib at the tissue level. This is the first report of the direct effect of Celecoxib on *P. brasiliensis* as well as in combination with Itraconazole. Our results suggest that this combined treatment employing an antifungal and an anti-inflammatory drug may constitute a new efficient therapeutic strategy. Grants: CNPq 305216/2016-3 and FAPEMIG APQ 012941-16. L.A. Santos and J.C. Grisolia are recipient of CAPES scholarships.

### P402. Successful Treatment of a Patient with Retroperitoneal Abscess caused by Candida krusei with the Investigational Agent, Ibrexafungerp (formerly SCY-078): A Case Report from the FURI study

ThompsonG.^1^AnguloD.^2^1Infectious Disease, University of California at Davis, Davis, United States of America2SCYNEXIS, Inc., Jersey City, United States of America

**Case Report: Objectives:** Intraabdominal candidiasis is the second most common *Candida* infection after candidemia. *Candida albicans* is the predominant cause of these infections, with non*-albicans Candida* becoming more frequently observed in this clinical setting. Echinocandins are the recommended treatment for intraabdominal *Candida* infections but have varied clinical response. This is postulated to be based on poor drug levels in tissue and abscesses. Ibrexafungerp (formerly SCY-078) is a novel IV/oral glucan synthase inhibitor (triterpenoid) antifungal with activity against *Candida*, *Aspergillus* and *Pneumocystis.* A Phase 3 open-label, single-arm study of oral ibrexafungerp (FURI; NCT03059992) is ongoing for the treatment of patients intolerant of or with fungal disease refractory to standard antifungal therapy. We present a patient case of retroperitoneal abscess caused by *Candida krusei* from the FURI study. **Methods:** A 71 year old male patient with ischemic stroke, pulmonary edema, and tracheostomy was being treated for a retroperitoneal abscess, a complication of a perforated duodenal ulcer. *Candida krusei* was isolated in peri-duodenal drain cultures and the patient was initiated on micafungin therapy for 21 days. During micafungin therapy, cultures from the peri-duodenal drain on three occasions remained positive for *Candida krusei*. The patient underwent abscess drainage and was enrolled into the FURI study due to micafungin treatment failure and was treated with oral ibrexafungerp 750mg BID for two days followed by 750mg daily. **Results:** The patient was treated with oral ibrexafungerp therapy for 21 days. Clinical improvement was observed during therapy. At End of Treatment visit, the clinical signs and symptoms of fungal disease were considered by the investigator to be resolved and the physical exam noted minimal discharge from the patient’s retroperitoneal drain. As of the 6-week follow-up visit, clinical signs and symptoms of infection were still resolved. No drug-related adverse events were reported. **Conclusion:** This case report shows the potential of oral ibrexafungerp to treat intraabdominal infections caused by *Candida krusei* in patients who fail echinocandin therapy. Continued enrollment in the FURI study is warranted.

### P403. In vitro study of Antifungal Biopharmaceutical Dectin1-Fc

LimL.Bioprocessing Technology Institute/National University of Singapore, Singapore, Singapore

**Objectives:** Invasive fungal infections are a neglected category of disease that has a high mortality and implicated in immunocompromised patients. Dectin-1 (CLEC7A) is a type II transmembrane C-type lectin pattern recognition receptor expressed on cells of the innate immune system like macrophages, monocytes and neutrophils that recognizes β-glucans through its single C-type lectin-like domain at its extracellular region. It functions by binding to cell walls of different fungal species, such as *Saccharomyces cerevisiae*, *Candida albicans*, *Pneumocystis carinii*, *Coccidioides posadasii*, and *Aspergillus fumigatus*; triggering immune cell responses like phagocytosis and cytokine production. The absence of β-glucans in human cells presents the opportunity to exploit Dectin-1 as therapeutic vehicle to enhance immune response during a fungal infection. This project aims to develop recombinant Dectin1-Immunoglobulin G1 Fc fusion protein as a potential biopharmaceutical for passive immunization to aid the innate immune system against Candida albicans fungal infection. The in vitro properties of the recombinantly expressed fusion protein are examined in this study.

**Methods:** The fusion protein was expressed in DG44 Chinese Hamster Ovarian (CHO) cells and amplified with methotrexate. The fusion protein was extracted from culture supernatant via GE Histrap histag affinity and purified with GE Superdex 200 size exclusion chromatography on a GE Akta Purifier FPLC. Immunofluorescence imaging with anti-Fc Alexa Fluor 568 conjugated antibody from ThermoFisher Scientific was used to assay binding of the fusion protein to unicellular or hyphae Candida albicans. Binding affinity of the Fc fusion protein versus a human IgG1 to Fcγ receptors from R&D Systems were screen by surface plasmon resonance on a GE Biacore T200.

**Results:** Immunofluorescence imaging shows the fusion protein capable of binding to Candida albicans both in the unicellular and hyphae morphology. Surface plasmon resonance study of the Fc-fusion protein versus a IgG1 control reveal that the fusion protein binds to FcγRI, FcγRIIIa and FcRn. However, the binding constants for the Fc-fusion is generally weaker than a full fledge IgG1 antibody. Activity testing of the fusion protein using THP-1 monocytes against unicellular Candida albicans in a 1 hr phagocytosis assay shows a dose response. For the highest dose of 100μg/ml of fusion protein, a reduction of greater than 50% of surviving Candida albicans cells compared to the untreated control was observed.

**Conclusion:** The in vitro results reveal that the respective domains of the Fc-fusion protein function as intended though binding to the Fc receptors are not as strong as an antibody. This in turn may affect the efficacy of the drug. Further testing with primary immune cells would be appropriate to study the full therapeutic potential of this fusion protein. In addition, in vivo studies with an appropriate mouse model will give a better picture of the performance of the Fc-fusion protein drug.

### P404. The in vitro effect of farnesol on the activity of voriconazol and amphotericin B against Aspergillus isolates

OzY.^1^OnderS.^2^DurmazG.^2^1Department of Microbiology, Division of Mycology, Eskisehir Osmangazi University Medical Faculty, Eskisehir, Turkey2Department of Microbiology, Eskisehir Osmangazi University Medical Faculty, Eskisehir, Turkey

**Objectives:** Although new antifungal agents are being developed, there is an increasing resistance to standard antifungal therapy, and no new classes of antifungal agents have been approved for a long time. Currently, three antifungal drug classes including triazoles, polyenes and echinocandins are available to use in treatment of IFIs. However, treatment is often complicated due to their high toxicity, low tolerability, drug interactions and limited spectrums of activities. Therefore, the requirement of new drug or treatment alternatives especially those with a wider spectrum, lower toxicity and cheaper are increasing day by day. Farnesol is an extracellular quorum-sensing molecule producing by *C.albicans* and inhibits the yeast-to-hypha transition in *C.albicans* and blocks biofilm formation. FAR is also a sesquiterpene alcohol existing in many herbal products and exogenously FAR inhibits the conidiation in *Aspergillus niger* and the germination of macroconidia in *Fusarium graminearum*. However, the number of studies assessing the antifungal efficacy of FAR with standardised methods is limited. In this study, we investigated the contribution of farnesol on the activity of antifungals such as voriconazole and amphotericin B against clinical *Aspergillus* isolates.

**Methods:** Clinical *Aspergillus* isolates, *A.fumigatus* (n = 30) and *A.flavus* (n = 15), were included in this study. The MIC values of antifungal drugs and farnesol were determined against all *Aspergillus* isolates using reference broth microdilution method (CLSI M38-A2). The interactions of farnesol with voriconazole and amphotericin B were investigated by the checkerboard method and evaluated based on the fractional inhibitor concentration index (FICI). The FICI was obtained by summing the FIC values of each drug; the FIC was calculated for each agent by dividing the inhibitory concentration of each antifungal or compound when used in combination by its MIC. Synergy was defined as a FICI of ≤ 0.5; no interaction was defined as a FICI > 0.5 but < 4; and antagonism was defined as a FICI ≥ 4.

**Results:** The MIC ranges of farnesol, voriconazole and amphotericin B were 1500-6000 µM, 0.125-1 µg/mL and 0.125-0.5 µg/mL against *A.fumigatus* isolates, 3000->6000 µM, 0.125-0.5 µg/mL and 0.25-2 µg/mL against *A.flavus* isolates, respectively. The most common interaction in combination tests was “no interaction” and synergistic interaction was not detected in any combination. The combination of farnesol with voriconazole had antagonistic activity against 13 of 31 *A.fumigatus* isolates with FICI values of 4.01 to 8.5, and 4 of 14 *A.flavus* isolates with FICI values of 4.03 to 5. Similarly, the combination of farnesol with amphotericin B had antagonistic activity against 7 of 31 *A.fumigatus* isolates with FICI values of 4.01 to 8.03, and 5 of 14 *A.flavus* isolates with FICI values of 4.5 to 10.

**Conclusion:** Antifungal activity of farnesol and synergistic interaction when it was combined with antifungal drugs have been reported against *Candida* spp and its biofilm. However, farnesol did not produce any significant inhibitor activity alone or any synergistic interaction in combination with voriconazole or amphotericin B against *Aspergillus* isolates in this study. Conversely, considerably antagonistic interaction was observed; 38% for farnesol with voriconazole combination, 27% for farnesol with amphotericin B combination.

### P405. Olorofim is potent against Madurella mycetomatis – the most common causative agent of Eumycetoma

LimW.^1^KoningsM.^1^RijndersB.^1^FahalA.H.^2^BirchM.^3^SandeW. Van De^1^1Department of Microbiology and Infectious Diseases, Erasmus Medical Center, Rotterdam, Netherlands2Mycetoma Research Center, University of Khartoum, Khartoum, Sudan3F2G Ltd, Manchester, United Kingdom

**Objectives:** *Madurella mycetomatis* is the main causative agent of eumycetoma, a chronic granulomatous infection of the subcutaneous tissue. Currently, the only antifungal agents with activity against *M. mycetomatis* are agents acting on ergosterol in the fungal cell membrane. Itraconazole is currently the drug of choice, but the duration of treatment is long and therapeutic failure is common. Therefore, there is an urgent need to identify more potent antifungal agents with activity against *M. mycetomatis.* One of the novel classes of antifungal agents are the orotomides and olorofim is the leading representative of this class. It inhibits fungal pyrimidine biosynthesis and it is currently being evaluated for invasive fungal infections in patients lacking treatment options. To determine if olorofim has *in vitro* activity against *M. mycetomatis,* we carried out MIC determinations against 21 *M. mycetomatis* clinical isolates with different genetic and geographical background and compared the results to those obtained for itraconazole.

**Methods:** Minimal inhibitory concentrations (MIC) were determined for Olorofim and Itraconazole against 21 *M. mycetomatis* clinical isolates from Sudan (14), Algeria (1), Mali (1), India (1), Chad (1), the Netherlands (1) and also from an unknown origin (2). The filamentous nature of *M. mycetomatis* necessitates the need for homogenization of inoculum by sonication to obtain a standardized inoculum for testing. This creates turbidity in the wells in an MIC test which complicates visual reading of the antifungal activity, tests were therefore read using XTT as a colorometric agent to indicate growth. MICs was performed using our CLSI-based in vitro susceptibility testing method with XTT reading at 450nm at the end after a 7-day incubation period at 37˚C. As a number of *M. mycetomatis* isolates produces pigments that influences colour intensity and the endpoint reading, an 80% reduction in viable fungal mass was determined instead of a 100%.

**Results:** We showed that olorofim was highly active against all tested *M. mycetomatis* isolates. MICs obtained for olorofim ranged from <0.004 µg/ml to 0.125 µg/ml and 0.06 μg/ml olorofim was needed to inhibit 90% of the isolates (see results in Table). Olorofim MICs were consistently one-dilution more potent than the MIC values for itraconazole. For itraconazole, MICs ranged from 0.008 µg/ml to 0.25 µg/ml and 0.125 μg/ml was needed to inhibit 90% of the isolates. 

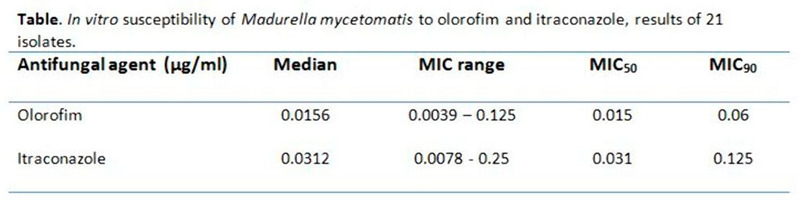



**Conclusion:** Our study showed that *M. mycetomatis* can also be inhibited with antifungal agents with a different mode of action to the azoles. Olorofim from the novel antifungal class of orotomides showed potent *in vitro* activity against all tested *M. mycetomatis* isolates and had MICs similar to or slightly lower than those for itraconazole - the current drug of choice for therapy. Further studies, including *in vivo* models are warranted to determine if olorofim would be a suitable alternative to itraconazole therapy.

### P407. Inhibiting fungal BET protein: a new hope to treat systemic fungal infections

KennaniS. El^1^PicaudS.^2^ChamplebouxM.^1^MaraisJ.^1^CourçonM.^3^ArlottoM.^1^RabutG.^4^FilippakopoulosP.^2^GovinJ.^1^1Signaling Through Chromatin, INSTITUTE FOR ADVANCED BIOSCIENCES, GRENOBLE, France2Ludwig Institute for Cancer Research, Nuffield Department of Clinical Medicine, University of Oxford, Oxford, United Kingdom3Biologie à Grande échelle, CEA, Grenoble, France4Institute of Genetics and Development, Rennes, France

**Objectives:** Invasive fungal infections kill an estimated 1.6 million people each year – as many deaths as are caused by tuberculosis or malaria. Currently, only four drug classes are available to treat these infections (polyenes, azoles, flucytosine and echinocandins). This limited repertoire of antifungal drugs, combined with an alarming rise in drug-resistant fungal strains, has created an urgent need for novel therapeutic agents. In the developed world, the most common fungal disease among hospitalized patients is invasive candidiasis. Among *Candida* species, *C. albicans* and *C. glabrata* rank first and second in isolation frequency, respectively, accounting for ~70% of all systemic candidiasis. This project investigates new antifungal strategies that target the BET family of transcriptional regulators in these *Candida* species. The fungal BET protein Bdf1 contains two bromodomains (BDs) and an extra-terminal domain (ET). We recently established the proof of concept that small-molecule inhibition of Bdf1 BDs compromises the viability and virulence of *C. albicans* (Mietton et al., Nature Communications 2017).

**Methods:** This project focuses on the ET domain and is based on a combination of genetics, medicinal chemistry, structural biology and biochemistry studies.

**Results:** It shows that the ET domain is essential for the survival of *C. albicans* and *C. glabrata in vitro*. Furthermore, we identified a specific groove on the ET domain and demonstrate that it is also essential for growth, paving the way for the potential discovery of selective inhibitors.

**Conclusion:** To conclude, epigenetic targets remain largely unexplored in the fungal infection field. Our investigation of Bdf1 as a therapeutic target represents a novel and highly promising area of exploration in the antifungal field.

### P408. Efficacy of intravenous posaconazole for the treatment of azole-resistant invasive aspergillosis

SeyedmousaviA.^1^Kwon-ChungJ.^2^BrüggemannR.J.M.^3^VerweijP.E.^4^1Department of Laboratory Medicine, Microbiology Service, National Institutes of Health Clinical Center, Bethesda, United States of America2Laboratory of Clinical Immunology and Microbiology, National Institute of Allergy and Infectious Diseases, Bethesda, Maryland, United States of America3Pharmacy, RadboudUMC, Nijmegen, Netherlands4Medical Microbiology, RadboudUMC, Nijmegen, Netherlands

**Objectives:** Azole antifungals play a prominent role in the management of patients with invasive aspergillosis (IA). However, azole resistance is a growing problem in patients with aspergillus infection, which translates into treatment failure. Alternative treatments with new formulations of azolesmay improve therapeutic outcome in IA even for the clinical cases caused by strains with low susceptibility to currently available azoles.

**Methods:** The *in vivo* efficacy of 0.25, 1, 4, 16 and 64 mg/kg/day recently introduced intravenous formulation of posaconazole (POS)was assessed in mice (CD-1 strain) infected with a wild type (POS MIC_EUCAST_, 0.031mg/L) and azole-resistant *Aspergillus fumigatus* (POS MIC_EUCAST_, 0.5 mg/L) harboring TR_34_/L98H mutation in Cyp-51A gene. Efficacy of POS treatment was assessed by tissue histopathology 3 days post infection and monitoring of survival for 14 days following intravenous inoculation.

**Results:** The survival curves for all control groups receiving 0.9% saline intravenously showed 100% mortality. The efficacy of POS treatment at the dosing regimens ≤4 mg/kg/day depended on the MIC of each isolate. However, the maximum effect (100% survival at day 14 post infection) was achieved with a POSdose of ≥ 16 mg/kg for both wild-type and TR_34_/L98H mutant isolates, and histopathological slides revealed limited number of inflammatory foci with or without detectable fungal elements in the Kidneys. The Hill-type model with a variable slope fitted the relationship between the dose and 14-day survival well, with R^2^ values of 0.99 for the wild type, and 0.95 for the TR_34_/L98Hisolate. The 50% effective dose (ED_50_) based on survival was 0.89 mg/kg (95% confidence interval [CI], 0.59 to 1.4 mg/kg) for the wild type, and 4.5 (95% CI, 2.1 to 7.6 mg/kg) for the TR_34_/L98Hisolate.

**Conclusion:** Overall, treatment with intravenous formulation of POS improved the survival of the mice in a dose-dependent manner. A dose-response relationship was observed regardless of the underlying azole-resistance mechanism. These results show intravenous formulation of POS to be a promising therapeutic agent for treatment of azole-resistant IA.

### P409. Anti-Candida activities of carvacrol, cinnamaldehyde, citral and thymol liposomes

Miranda-CadenaK.^1^DiasM.^2^,^3^Costa-BarbosaA.^2^,^3^CollinsT.^2^,^3^Marcos-AriasC.^1^ErasoE.^1^QuindósG.^1^SampaioP.^2^,^3^1Inmunología, Microbiología Y Parasitología, Universidad del País Vasco/Euskal Herriko Unibertsitatea UPV/EHU, Bilbao, Spain2Centre of Molecular and Environmental Biology (cbma), Department of Biology, University of Minho, Braga, Portugal3Institute of Science and Innovation For Bio-sustainability (ib-s), University of Minho, Braga, Portugal

**Objectives:** To develop and characterize carvacrol, cinnamaldehyde, citral and thymol-loaded DODAB:MO nanoparticles, to test their cytotoxicity on murine macrophages and to evaluate their anti-*Candida* activities in vitro.

**Methods:** Encapsulation was made using monoolein-based liposomes of DODAB:MO in a 1:2 molar ratio. DODAB:MO liposomes were prepared using thin lipid-film hydration method Concentrations tested were 32, 64 and 128 mg/L of carvacrol and thymol; 16, 32 and 64 mg/L of cinnamaldehyde and 64, 128 and 256 mg/L of citral. Mean size (nm), polydispersity (PDI) and surface charge (ζ-potential) were evaluated with Malvern ZetaSizer Nano ZS particle analyser (Malvern, UK). Encapsulation efficiency (%EE) was determined by HPLC-DAD, after separation by ultacentrigugation. Antifungal activities of encapsulated and non-encapsulated phytocompounds (Sigma-Aldrich, USA) was evaluated by microdilution against eight *Candida albicans*, one *Candida auris*, *Candida dubliniensis*, *Candida tropicalis* and *C. albicans* reference strain SC5314. Cytotoxicity was assayed on murine macrophage-like cell line RAW 264.7 by detection of cell metabolic activity by the reduction of MTT to formazan and lactate dehydrogenase activity (LDH) assay.

**Results:** Nanoparticles of 128 mg/L of carvacrol and thymol presented high %EE (70.8 and 69.1%, respectively) without loss of anti-*Candida* activity. Geometric means of MICs of carvacrol and thymol nanoparticles were 101.6 mg/L and 71.8 mg/L, respectively. Nanoparticles of 256 mg/L of citral presented the best %EE (83.3%), but they did not present antifungal activity. Cinnamaldehyde nanoparticles presented the lowest %EE (20.6 to 44.1%) and maintained their antifungal activity. All nanoparticles were stable during four weeks stocked at 4 ºC, with PDI values < 0.6 and ζ-potential among + 46.9 to + 55.6 mV. Thymol nanoparticles were the biggest with a particle size of 580.3 ±17.4 nm, followed by cinnamaldehyde, carvacrol and citral nanoparticles (567.9 ± 13.9, 510 ± 9.6 and 497.6 ± 13.8 nm, respectively). Non-encapsulated 32 µg/mL of thymol and carvacrol showed less cytotoxicity levels (76.5% and 74.8% cell viability by MTT assay, respectively; and ≥ 80% by LDH assay), followed by citral 64 µg/mL with 68% by MTT assay and 92.9% by LDH. Thymol and carvacrol nanoparticles were the best tolerated by macrophages with a higher cell viability by MTT assay (83.5-112.8% for thymol nanoparticles and 101-137% for carvacrol nanoparticles) when incubated with 18, 36 and 72 µg/mL of thymol nanoparticles and 18 and 36 µg/mL of carvacrol nanoparticles.

**Conclusion:** Monoolein-based liposomes of DODAB:MO (1:2) loaded with 128 µg/mL of carvacrol or 128 µg/mL of thymol can be a promising alternative of treatment of candidiasis, due to their striking physicochemical properties, appropriate encapsulation efficiencies, stability, reduced cytotoxicity and preserved anti-*Candida* activities. Work supported by Gobierno Vasco-Eusko Jaurlaritza (GIC15/78 IT-990-16) and the Federation of European Microbiological Societies by Research and Training grant (FEMS-GO-2017-020).

### P410. rIn vitro antifungal effect of plant-based compound CIN-102 on filamentous fungi

D’AgostinoM.Laboratoire SIMPA, Vandoeuvre-lès-Nancy, France

**Objectives:** Today the increase of invasive fungal infections due to the rise of immunosuppressive therapies is a real problem especially in hospitals. Moreover, the emergence of resistant strains induces a therapeutic failure. Face to this issues, new classes of antifungals are expected. The plant kingdom thus represents an immense potential of natural resources to exploit for these purposes. The SEPTEOS company has recently developed the CIN-102 compound, by using cinnamaldehyde and potentiating compounds from two synergic essential oils of cinnamon. Cinnamaldehyde has already been describe to kill bacteria, yeast and molds growth in both growing and non-growing state (not published). The objective of this study is to determine the spectrum of activity of CIN-102 on the main genus or species of filamentous fungi involved in human pathology.

**Methods:** The activity of CIN-102 product and its main constituent (cinnamaldehyde / CIN) are tested against a representative panel of strains of filamentous fungi (*Aspergillus (n = 39)*, *Fusarium (n = 19)* and *Scedosporium n = 21)*) of clinical importance. Clinical and reference strains have been studied, comprising isolates characterized by innate or acquired antifungal resistance patterns. A determination of MICs (minimum inhibitory concentrations) was performed using the M38-A2 CLSI reference method. The observed antifungal effect was then characterized by determining an inoculum effect.

**Results:** The MIC of CIN-102 determined for the different strains studied is mainly between 62 (n = 28) and 125 (n = 30) ug / ml, similar to the MIC determined for main active. Strains of the genus *Aspergillus* have on average a slightly higher MIC compared to the genus *Fusarium* and *Scedosporium:* MIC mainly to 125 μg / ml for *Aspergillus* (n = 23), and 62 μg / ml for *Fusarium* (n = 12) and *Scedosporium* (n = 12). Acquired resistance against azole antifungals (*Aspergillus fumigatus* TR46, TR34 and G54E resistant strains) or innate (naturally antifungals resistant *Scedosporium* strains) do not seem to impact the effect of the compound CIN-102 and cinnamaldehyde. The inoculum as well as the time and dose dependent effect of CIN-102 and cinnamaldehyde will also be characterized.

**Conclusion:** Although the MICs alone do not provide an opinion on the efficacy of CIN-102, these results already show the antifungal effect of the compound. The current results for CIN-102 are very close to those determined for cinnamaldehyde in our study but also in the literature (Homa et al., 2015) (Essid et al., 2017). Further studies will then be conducted, notably on the structural aspect and the mechanism of action, to complete the knowledge on this new molecule. Moreover, an in vivo model of invasive fungal infection is studied to develop our knowledge about this new molecule.

### P411. New strategies based on antifungal antibodies for the treatement of aspergillosis

ChauvinD.^1^HustM.^2^SchütteM.^2^ChesnayA.^1^,^3^ParentC.^1^MoreiraG.M.S.G.^2^ArroyoJ.F.^4^SanzA.B.^4^MartineauP.^5^PugnièreM.^5^BaillyE.^1^ChandenierJ.^1^,^3^Heuzé-VourcHN.^1^DesoubeauxG.^1^,^3^1Cepr Inserm U1100, Université de Tours, Tours, France2Institut Für Biochemie, Biotechnologie und Bioinformatik, Technische Universität Braunschweig, Braunschweig, Germany3Parasitology - Mycology - Tropical Medicine, Hôpital Bretonneau, TOURS, France4Microbiología Y Parasitología, Universidad Complutense, Madrid, Spain5Institut De Recherche En Cancérologie De Montpellier, Université de Montpellier, Montpellier, France

**Objectives:** *Aspergillus fumigatus* is an airborne opportunistic fungal pathogen responsible for severe infections. For instance, invasive pulmonary aspergillosis is a serious threat for individuals suffering from severe immunosuppression, with mortality rates > 50%. In parallel, allergic bronchopulmonary aspergillosis frequently encountered in cystic fibrosis patients, is also a comorbidity factor. In addition to diagnostic means lacking specificity, current treatments present a high toxicity which prevents their use in weakened subjects, resulting in impaired prognostic. Because of their low toxicity and high specificity, anti-infectious therapeutic antibodies could be a new alternative to conventional therapeutics. In this study, we investigated the potential of *Chitin Ring Formation* cell wall transglycosylases of *A. fumigatus* to be therapeutic targets for therapeutic antibodies.

**Methods:** *In vitro* neutralization test, *in vitro* cultures, fluorescence and confocal microscopy, DNA sequencing, as well as flow cytometry and animal models were used.

**Results:** We demonstrated that the Crf target was highly conserved, regardless of the pathophysiological context; whereas the *CRF1* gene was found to be 100% conserved in 92% of the isolates studied, Crf proteins were expressed in 98% of the strains. In addition, we highlighted the role of Crf proteins in fungal growth, using a deletion mutant for *CRF1* gene, for which a growth decrease of 23.6% was observed after 48 h. It was demonstrated that anti-Crf antibodies neutralized the enzymatic activity of recombinant Crf protein, and delayed fungal growth by 12.3% *in vitro* when added to spores. In a neutropenic rat model of invasive pulmonary aspergillosis, anti-Crf antibodies elicited a significant recruitment of neutrophils, macrophages and T CD4 lymphocytes but it was not correlated with a decrease of fungal burden in lungs and improvement in survival.

**Conclusion:** Overall, our study highlighted the potential relevance of targeting Crf cell wall protein with therapeutic antibodies.

### P412. Olorofim EUCAST susceptibility testing of contemporary moulds: in vitro activity and improved reproducibility

ArendrupM.C.^1^,^2^,^3^DatcuR.^1^HareR.^1^JørgensenK.M.^1^1Unit of Mycology, Statens Serum Institut, Copenhagen, Denmark2Dept. of Clinical Microbiology, Rigshospitalet, Copenhagen, Denmark3Dept. of Clinical Medicine, University of Copenhagen, Copenhagen, Denmark

**Objectives:** Olorofim is a novel antifungal orotomide compound for which we previously demonstrated good *in vitro* activity against various moulds. This study sought to (a) investigate whether MIC variation observed for *A. fumigatus* (unimodal range <0.004–0.25 mg/L) was due to inherent MIC variation or true differences in susceptibility and (b) present EUCAST MICs for clinical mould isolates from 2018.

**Methods:** MICs (mg/L) of olorofim were determined by EUCAST E.Def 9.3 using *A. fumigatus* ATCC 204305 for QC. MIC variability study: Fifteen *A. fumigatus* strains were selected from the prior study, as follows: five low MIC isolates: MIC = ≤0.002 (n = 2) and MIC = 0.004 (n = 3); five middle MIC isolates: MIC = 0.03 (n = 2) and MIC = 0.06 (n = 3); and five high MIC isolates: MIC = 0.125 (n = 1) and MIC = 0.25 (n = 4). MICs were determined visually by two-three observers blinded to the original MIC. MICs against 365 clinical mould isolates were determined during 2018. For *A. fumigatus* olorofim activity was evaluated individually for itraconazole susceptible (MIC≤1) and non-susceptible organisms (MIC>1). The ECOFFinder programme was used for determining statistical wildtype upper limits (highest MIC for organisms without phenotypically detectable acquired resistance mechanisms, WT-ULs) using 95%, 97.5% and 99% subset endpoints. For *Fusarium* isolates the 50%-MIC was also determined spectrophotometrically using 50% growth inhibition.

**Results:** Repetitive testing of low, medium and high MIC *A. fumigatus* isolates resulted in MICs, which for 14/15 isolates fell within two dilutions, whereas 1/10 MICs for isolate #12 and 1/80 MICs for the control strain fell one dilution below the two-dilution range when read by observer 1. The modal MIC for low/middle/high MIC isolates were as follows for repeated testing: Observer 1 and 2: 0.03/0.03/0.016 and for observer 3: 0.03/0.03/0.03. Routine testing during 2018 for species represented by ≥15 isolates generated uniform Gaussian MIC distributions spanning ≤5 dilutions with modal MICs = 0.03-0.06 for all isolates except non-*proliferatum Fusarium* spp. (n = 10) (Table). Modal MIC/WT-UL for *A. fumigatus* was 0.06/0.125 and was unaffected by itraconazole susceptibility and choice of ECOFFinder endpoint. Olorofim displayed MICs ≤0.25 against less common *Aspergillus* spp. except *A. montevidensis* (n = 1) as well as against dermatophytes and other moulds including: *Microsporum gypseum* (n = 1), *T. interdigitale* (n = 1), *T. rubrum* (n = 12), *Rasamsonia aegroticola* (n = 1)*, Rasamsonia argillacea* (n = 1)*, Scedosporium apiospermum* (n = 3)*, Scedosporium boydii* (n = 2). 50%-MICs for *Fusarium* were as follows: *F. dimerum* >8 (n = 2), *F. oxysporum* 0.06 (n = 1), *F. solani* complex MIC range 0.25-1, modal MIC 1 (n = 7), and *F. proliferatum* 0.03 (n = 1). 

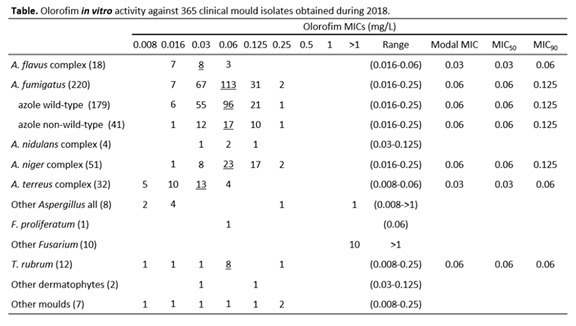



**Conclusion:** Upon repeated testing of *A. fumigatus* isolates with initially low/medium/high MICs, no differences were observed. This suggests that the initial distribution observed can be explained by technical variation and that olorofim has similar and uniform activity against all isolates within the wild-type population. This was confirmed when evaluating results for routine MIC testing during 2018. In conclusion, olorofim displayed promising *in vitro* activity against most moulds included in this study independent of azole susceptibility.

### P413. EUCAST susceptibility testing of manogepix and six comparative agents against contemporary Danish mould isolates

ArendrupM.C.^1^,^2^,^3^AstvadK.M.T.^1^JørgensenK.M.^1^1Unit of Mycology, Statens Serum Institut, Copenhagen, Denmark2Dept. of Clinical Microbiology, Rigshospitalet, Copenhagen, Denmark3Dept. of Clinical Medicine, University of Copenhagen, Copenhagen, Denmark

**Objectives:** The new antifungal fosmanogepix (APX001) is a first-in-class drug candidate and the methyl phosphate prodrug of the active moiety manogepix (APX001A). Manogepix targets the conserved fungal inositol acyltransferase enzyme Gwt1, thereby preventing GPI-anchored protein maturation and compromising fungal growth. Fosmanogepix is currently in Phase 2 clinical trials. Here we compared the *in vitro* activity of manogepix and six comparators against contemporary clinical mould isolates received for identification and EUCAST susceptibility testing.

**Methods:** A total of 163 clinical mould isolates obtained Aug 2016 to Sep 2017 were included. *Aspergillus* isolates were identified to the species complex level except *A. fumigatus* that was identified sensu stricto using thermotolerance. EUCAST E.Def 9.3.1 reference method using cell-culture treated microtitre plates (Nunc, ThermoFisher Scientific, cat. no. 167008) were prepared using serial two-fold dilutions of manogepix, amphotericin B, isavuconazole, voriconazole, itraconazole and posaconazole. The plates were stored at -80°C for ≥24 h prior to inoculation. Susceptibility endpoints were determined visually as MECs for manogepix (the lowest concentration resulting in aberrant growth) as complete visual or 50% spectrophotometric growth inhibition were not observed (data not shown). For the comparators MICs were read visually according to the EUCAST definition at the lowest concentration giving complete growth inhibition. The ECOFFinder programme was used for determining a statistical single-centre manogepix wild-type upper-limit for *A. fumigatus*.

**Results:** The manogepix MEC and comparator MICs are shown as modes (not done (ND) for isolates represented by five or fewer isolates) and ranges in the Table. Manogepix MECs were ≤0.125 mg/L against all *Aspergillus* and the two *Fusarium* isolates but no activity could be detected against the dermatophyte and three mucorales isolates included at the concentrations tested (MICs >0.5 mg/L). Manogepix *in vitro* activity remained the same against *A. fumigatus* isolates resistant to itraconazole (Table). Using the ECOFFinder programme, a single-centre wild-type upper-limit of 0.125 mg/L was determined for *A. fumigatus* (applying a 97.5% cut-off). 

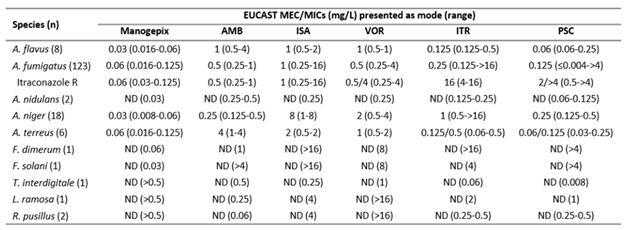



**Conclusion:** Manogepix displayed uniform and potent *in vitro* activity against the different *Aspergillus* species and *Fusarium* isolates included in the study as determined by MECs. The *in vitro* activity was unaffected by acquired resistance in *A. fumigatus*. Thus, fosmanogepix appears to be a promising therapeutic against these pathogens, many of which are difficult to treat.

### P414. Candida auris is Highly In Vitro Susceptible to Ibrexafungerp (formerly SCY-078) in EUCAST Antifungal Susceptibility Testing

JørgensenK.M.^1^HareR.^1^ChowdharyA.^2^ArendrupM.C.^1^,^3^,^4^1Unit of Mycology, Statens Serum Institut, Copenhagen, Denmark2Department of Medical Mycology, Vallabhbhai Patel Chest Institute, University of Delhi, Delhi, India3Dept. of Clinical Microbiology, Rigshospitalet, Copenhagen, Denmark4Dept. of Clinical Medicine, University of Copenhagen, Copenhagen, Denmark

**Objectives:** *Candida auris* is a multidrug-resistant yeast rapidly emerging as a significant cause of nosocomial infections. Ibrexafungerp is a novel oral synthetic enfumafungin derivative inhibiting glucan synthase. It is active *in vitro* and in animal models against *Candida*, *Aspergillus* and *Pneumocystis* and is currently in clinical development for mucocutaneous and invasive fungal infections. Here, we investigate the susceptibility of *C. auris* to ibrexafungerp (formerly SCY-078) compared to that of eight comparators and compared to the activity against *C. albicans* and *C. glabrata*.

**Methods:** EUCAST AFST according to E.Def 7.3.1 was performed for ibrexafungerp (Scynexis, Jersey City, NJ, USA) against 122 clinical *C. auris* isolates (from India (n = 120) and Oman (n = 2)) and three *C. auris* control strains JCM15448, KCTC17809 and KCTC17810. Sixteen Danish clinical *C. albicans* and 16 *C. glabrata* isolates and the control strains *C. albicans* ATCC64548, *C. krusei* ATCC6258 and *C. parapsilosis* ATCC22019 were included as comparators and controls. Cell-culture treated microtitre plates (Nunc, Thermo Fisher Scientific, cat. no. 167008) were prepared using the ISO method. The ibrexafungerp MICs were compared to previously published MICs for anidulafungin, micafungin, amphotericin B, fluconazole, isavuconazole, itraconazole, posaconazole and voriconazole.

**Results:** The *in vitro* activity of ibrexafungerp (IBX) against *C. auris* was uniform with MICs displaying a Gaussian distribution spanning 0.06-2 mg/L suggesting an equal efficacy across the 122 isolates (Table). The modal MIC and MIC_50_ were 0.5 mg/L. For the *C. auris* reference strains, the MICs were 0.06 mg/L, 0.125 mg/L and 0.5 mg/L, respectively. The MIC ranges for nine repetitive MIC determinations of the control strains MICs were as follows: *C. albicans* ATCC 64548: 0.06 mg/L, *C. krusei* ATCC 6258: 0.5-1 mg/L and *C. parapsilosis* ATCC 22019: 0.125-0.5 mg/L respectively. In contrast, MIC distributions for anidulafungin (ANF), micafungin (MCF), isavuconazole (ISA), voriconazole (VOR), itraconazole (ITR) and posaconazole (PRC) against *C. auris* were wide (spanning 10-13 two-fold dilutions) suggesting differential activity against the isolates. of note, ibrexafungerp MICs remained low (MICs of 0.25 mg/L and 0.5 mg/L) for the isolates resistant to anidulafungin and micafungin (MICs >32 mg/L). Finally, fluconazole (FLU) MICs for all but one isolate were 16- >256 mg/L suggesting almost universal fluconazole resistance whereas the amphotericin B (AMB) MICs clustered close to the non-species specific breakpoint of 1 mg/L. Next, ibrexafungerp activity was compared for *C. albicans, C. glabrata* and *C. auris*. MIC_50_, (range) were 0.06 mg/L (0.03-0.125 mg/L), 0.25 mg/L (0.25-0.5 mg/L) and 0.5 mg/L (0.06-2 mg/L), respectively. 

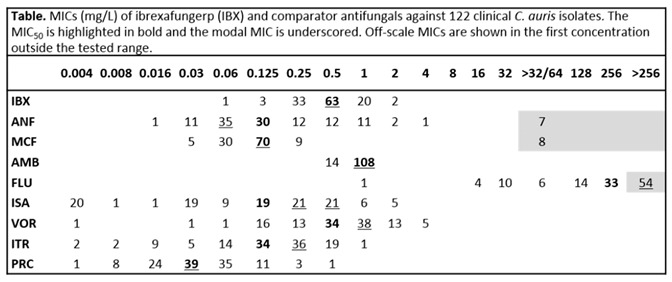



**Conclusion:** Ibrexafungerp shows promising *in vitro* activity against *C. auris* suggesting it may be a welcomed therapeutic against this emerging threat with few treatment options. Ibrexafungerp’s MICs were in general one step higher against *C. auris* than against *C. glabrata* and, notably, remained unchanged against *C. auris* isolates with antifungal resistance to the comparator drugs, including those highly echinocandin-resistant.

### P415. In vivo efficacy of olorofim against systemic infection caused by Scedosporium apiospermum, Pseudallescheria boydii, and Lomentospora prolificans in neutropenic CD-1 mice

SeyedmousaviA.^1^ChangY.C.^2^LawD.^3^BirchM.^3^RexJ.^3^Kwon-ChungJ.^4^1Department of Laboratory Medicine, Microbiology Service, National Institutes of Health Clinical Center, Bethesda, United States of America2Laboratory of Clinical Immunology and Microbiology, National Institute of Allergy and Infectious Diseases, National Institutes of Health, Bethesda, United States of America3F2G Ltd, Manchester, United Kingdom4Laboratory of Clinical Immunology and Microbiology, National Institute of Allergy and Infectious Diseases, Bethesda, Maryland, United States of America

**Objectives:** Clinically relevant memebrs of the *Scedosporium*/*Pseudallescheria* species complex and *Lomentospora prolificans* are generally resistant to currently available systemic antifungal agents and the infection due to these species is difficult to treat. Alternative treatments with new classes of antifungals may improve therapeutic outcomes of the disease caused by these difficult to treat moulds.

**Methods:** We studied the *in vivo* efficacy of olorofim (formerly F901318), a new fungicidal agent that prevents growth of manyascomycetous mold speciesvia inhibition of de novo pyrimidine biosynthesis, against scedosporiosis caused by three species in neutropenic CD-1 mice. Cyclophosphamide immunosuppressed mice infected by *Scedosporium apiospermum*, *Pseudallescheria boydii* and *Lomentospora prolificans* (tail vein infection) were treated by intraperitoneal administration of olorofim (15 mg/kgevery 8 h for 9 days). The efficacy of olorofimtreatment was assessed by survival rate 10 days post infection, and tissue histopathology 3 days post infection.

**Results:** In the neutropenic CD-1 mice, olorofim therapy significantly improved survival compared to the untreated controls; 80 %, 100 % and 100 % of treated mice survived infection by *Scedosporium apiospermum*, *Pseudallescheria boydii*, *Lomentospora prolificans,* respectively while less than 20% of the control mice (PBS-treated) survived at 10 days post infection. Furthermore, histopathological slides of kidneys revealed no detectable fungal elements in the olorofim-treated mice, whilst numerous fungal hyphae were present in control mice.

**Conclusion:** Overall, treatment with olorofimimproved the survival of the mice infected with all three species causing scedosporiosis and showed rapid clearance of fungi from the kidney by histological staining. These results show olorofim to be a promising therapeutic agent for systemic scedosporiosis, a difficult disease to treat with currently available antifngals.

### P416. APX001 (Fosmanogepix) is Effective in an Immunosuppressed Mouse Model of Fusariosis

GebremariamT.^1^AlkhazrajiS.^1^GuY.^1^AlqarihiA.^1^MamoueiZ.^1^ShawK.J.^2^IbrahimA.S.^1^1Los Angeles Biomedical Research Institute, Harbor-UCLA Medical Center, Torrance, United States of America2Amplyx Pharmaceuticals, San Diego, United States of America

**Objectives:** Fusariosis has high mortality rates. Owing to its rarity, comparative clinical trials are hard to perform. Animal models are an appropriate complementary avenue for evaluating antifungal therapy. Thus APX001 (fosmanogepix) was evaluated in an immunosuppressed murine model of hematogenously disseminated fusariosis.

**Methods:** The minimum effective concentration (MEC) of APX001A (manogepix, the active moiety of APX001) was determined against a *Fusarium solani* clinical isolate using CLSI M38 method. ICR mice were immunosuppressed with cyclophosphamide and cortisone acetate on days -2, and +3, relative to intravenous infection with 7.5×10^4^ cells of *F. solani*. For survival studies, treatment with placebo (diluent control), APX001 (78 or 104 mg/kg, PO), liposomal amphotericin B (LAmB, 15 mg/kg, IV), or voriconazole (Vori, 40 mg/kg, PO) began 16 h post-infection and continued for 8 days for APX001 or Vori and 4 days for LAmB. To extend the half-life of APX001, mice were administered 50 mg/kg of the cytochrome P450 inhibitor 1-aminobenzotriazole (ABT) 2 h prior to APX001 administration. Grapefruit juice in drinking water (50%) was given to Vori-treated mice to enhance the drug’s half-life. To assess tissue fungal burden, mice were sacrificed on Day +4 and organs processed for conidial equivalent (CE) by qPCR.

**Results:** The APX001A MEC value for the tested *F. solani* strain was 0.03 μg/mL. Treatment with APX001 or LAmB enhanced median survival time vs. placebo (12, and 10 days for 78, and 104 mg/kg of APX001, respectively vs. 10 days for LAmB treatment, vs. 8 or 7 days for Vori or placebo, respectively, *P* <0.01). Further, APX001 and LAmB treatments equally enhanced overall survival by day 21 when the experiment was terminated (40% for LAmB or APX001 at 78 mg/kg, and 20% for APX001 at 104 mg/kg, vs. 0% for placebo or Vori treatment). APX001 or LAmB treatments, but not Vori, resulted in ~2-3 log reduction in kidney, and brain CE vs. placebo.

**Conclusion:** APX001A showed significant antifungal activity against *F. solani in vitro* which translated to *in vivo* efficacy. APX001 was as effective as LAmB in this mouse model of fusariosis. Continued investigation of APX001 as a novel antifungal agent against this difficult to treat infection is warranted.

### P417. Evaluation of the In vitro Activity of Olorofim Against Fusarium Species

WiederholdN.^1^PattersonH.^1^BirchM.^2^LawD.^2^RexJ.^2^1Fungus Testing Laboratory, The University of Texas Health Science Center at San Antonio, San Antonio, United States of America2F2G Ltd, Manchester, United Kingdom

**Objectives:** Invasive infections caused by *Fusarium* species are often associated with significant morbidity and mortality in highly immunocompromised patients, and treatment options are limited. Several species are often resistant to treatment with clinically available antifungals. Olorofim (formerly F901318; F2G Ltd.) is a member of the orotomide class of antifungal agents that inhibits fungal pyrimidine biosynthesis and has potent activity against a variety of filamentous fungi. We evaluated the *in vitro* activity of olorofim against a selection of various *Fusarium* isolates. The *in vitro* activity was also measured for the clinically available antifungals amphotericin B, posaconazole, and voriconazole.

**Methods:** Clinical isolates (n = 111) of *Fusarium* species in the collection of the University of Texas Health Science Center Fungus Testing Laboratory were used. Susceptibility testing was performed by broth microdilution according the CLSI M38 reference standard. MICs for olorofim were determined after 48 hours of incubation using the 50% and 100% inhibition endpoint, while those of the control agents (amphotericin B, posaconazole, and voriconazole) were determined using the 100% inhibition endpoint.

**Results:** Olorofim demonstrated *in vitro* activity against *F. oxysporum* (MIC range 0.125-0.25 mg/L and 0.5 – 1 mg/L at the 50% and 100% endpoints, respectively)*, F. fujikuroi* (≤0.015 mg/L and ≤0.015-0.125 mg/L), *F. verticillioides* (0.06-0.125 mg/L and 0.06-0.25 mg/L), and *F. proliferatum* (≤0.015->8 mg/L and 0.06->8 mg/L) (Table). At the 50% inhibition endpoint, olorofim demonstrated *in vitro* activity against some isolates of the *F. solani* species complex (FSSC, now members of the genus *Neocosmospora* 0.5->8 mg/L) and the *F. incarnatum-equiseti* species complex (FIESC, 0.25->8mg/L). In contrast, when the 100% inhibition endpoint was used, limited to no *in vitro* activity was observed against these species complexes (4->8 mg/L), nor was activity observed against *F. dimerum* using either endpoint (>8 mg/L). Olorofim activity against some of the rarer species of *Fusarium* was mixed, with the growth of some species being inhibited at low MICs (i.e., *F. brachygibbosum, F. decemcellulare, F. redolens,* and *F. thapsinum*; 0.06-0.25 mg/L at 50% inhibition and 0.25-0.5 mg/L at 100% inhibition). In contrast, limited to no activity was also observed against other rarer species (i.e., *F. delphinoides, F. nygamai, F. pallidoroseum, F. petroliphilum,* and *P. pseudensiforme*; 0.25-8 mg/L at 50% inhibition and ≥8 mg/L at 100% inhibition). The MIC ranges for the control agents were also wide and differed across *Fusarium* species. The most consistent activity was observed with amphotericin B. 

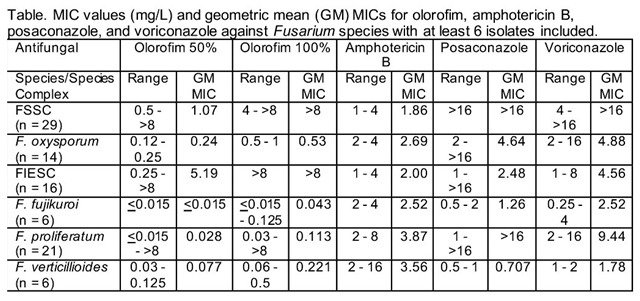



**Conclusion:** Olorofim demonstrated *in vitro* activity against some clinical isolates of *Fusarium* species. This activity appeared to be species-dependent, and was also dependent on the endpoint used (50% vs. 100% inhibition of growth). Further worked is needed to determine how the *in vitro* activity observed against *Fusarium* species in this study may translate into *in vivo* efficacy, especially for more common species for which there were differences between the activity of olorofim at the different of growth inhibition endpoints.

### P419. Nanoparticles as antimicrobial agent against Scytalidium hyalinum and Neoscytalidium dimidiatum

AlmeidaB. De^1^LemesT.^1^AlmeidaM.^2^VolantiD.^1^1Unesp, São José do Rio Preto, Brazil2FAMERP, São José do Rio Preto, Brazil

**Objectives:** The present study aimed to evaluate the antifungal activity of silver-based nanocomposites (NCs) deposited in hydroxyapatite (HAP), after synthesis by microwave, against clinical strains of *Scytalidium hyalinum* and *Neoscytalidium dimidiatum*.

**Methods:** The analysis was performed with Ag/HAP NCs (4 and 8% Ag) and pure Ag to evaluate the minimum inhibitory concentration to kill *Scytalidium hyalinum* and *Neoscytalidium dimidiatum*, according to CLSI M27-S4 with modifications considering, in triplicate. The fungi strains were maintained in RPMI broth culture media after overnight incubation at 37°C, and each inoculum adjusted according to 0.5 MacFarland standard tube. In 96-well plates, 100 µL of RPMI with fungal inoculum was added and NCs were dispersed into the 10% dimethyl sulfoxide (DMSO) - ranging from 1000 to 7.8 µg/mL. The plates were incubated for 24 hours. After growth, the cell viability was conducted by enzymatic reduction using triphenyl tetrazolium chloride (TTC).

**Results:** The highest antifungal activity was seen for 8% Ag/HAP NCs against *Scytalidium hyalinum* and *Neoscytalidium dimidiatum* with MIC 250 µg/ml. In addition, the pure Ag showed the worst antifungal activity with a MIC 1000 µg/mL for *Scytalidium hyalinum and* MIC 250 µg/mL for *Neoscytalidium dimidiatum.*

**Conclusion:** This study contributes to the knowledge of interactions between nanocomposites and fungal cell, as potential target of control infection caused by *Scytalidium hyalinum* and *Neoscytalidium dimidiatum.* It is clear the occurrence of the synergistic effect of Ag/HAP NCs in relation to pure Ag, event that must be considered, once HAP is biocompatible and could reduce the toxicity of Ag.

### P420. rAPX001 (Fosmanogepix) is Effective in an Immunosuppressed Mouse Model of Rhizopus oryzae Infection

GebremariamT.^1^AlkhazrajiS.^1^GuY.^1^AlqarihiA.^1^MamoueiZ.^1^ShawK.J.^2^IbrahimA.S.^1^1Los Angeles Biomedical Research Institute, Harbor-UCLA Medical Center, Torrance, United States of America2Amplyx Pharmaceuticals, San Diego, United States of America

**Objectives:** Mucormycosis is a life-threatening infection that predominantly occurs in immunocompromised hosts. The antifungal APX001A (manogepix) inhibits Gwt1, an enzyme required for the conserved glycosylphosphatidyl inositol (GPI) post-translational modification in eukaryotes. We previously reported the activity of APX001 (fosmanogepix, the prodrug of APX001A) against *Rhizopus delemar* (minimum effective concentration [MEC] = 0.25 µg/mL). Here we assessed the activity against *R. oryzae,* which has an elevated MEC value.

**Methods:** *R. oryzae* 99-892 MIC and MEC values were 0.125 µg/mL and 4.0 µg/mL for isavuconazole (ISAV) and APX001A, respectively. ICR mice were immunosuppressed with cyclophosphamide (200 mg/kg) and cortisone acetate (500 mg/kg) on Days -2, +3, and +8 relative to intratracheal infection with 2.5 x 10^5^ cells of *R. oryzae* 99-892. For survival studies, treatment with 104 mg/kg APX001 was compared to ISAV (110 mg/kg TID). Oral treatment started on Day +1 through Day +7, relative to infection for survival studies, and through Day +4 for tissue fungal burden studies (assessed by conidial equivalent [CE] using qPCR). Placebo mice received vehicle control. To extend the half-life of APX001, mice were administered 50 mg/kg of the cytochrome P450 inhibitor 1-aminobenzotriazole (ABT) 2 h prior to APX001 administration.

**Results:** APX001 and ISAV equally prolonged median survival time of mice (n = 20) versus placebo (12 and 14 days for APX001 and ISAV, respectively, vs. 8 days for placebo). Further, APX001 and ISAV treatment both resulted in 30% 21-day survival versus 0% survival of placebo mice (*P* <0.05 by Log Rank test). Both drug treatments resulted in ~1.5 log_10_ reduction in lung and brain CE vs. placebo-treated mice (n = 10, *P* <0.005 by Wilcoxon Rank Sum).

**Conclusion:** Despite a higher MEC value, APX001 showed significant efficacy against *R. oryzae* that was as protective as ISAV in immunosuppressed mice. Given the previously reported activity of APX001 against a strain of *R. delemar* with a lower MEC value, APX001 has now been shown to be efficacious against both species of *Rhizopus*, which together are responsible for ~60-70% of isolates causing lethal mucormycosis. Thus, continued investigation of APX001 against mucormycosis is warranted.

### P421. APX001 Protects Immunosuppressed Mice from Scedosporiosis

AlkhazrajiS.^1^GebremariamT.^1^AlqarihiA.^1^GuY.^1^ShawK.J.^2^IbrahimA.S.^1^1Los Angeles Biomedical Research Institute, Harbor-UCLA Medical Center, Torrance, United States of America2Amplyx Pharmaceuticals, San Diego, United States of America

**Objectives:** Scedosporiosis has high mortality rates. Owing to its rarity, comparative clinical trials are hard to perform. Animal models are an appropriate complementary avenue for evaluating antifungal therapy. Thus, APX001 was evaluated in an immunosuppressed murine model of invasive scedosporiosis.

**Methods:** The minimum effective concentration (MEC) of APX001A (the active moiety of APX001) was determined against 9 clinical isolates of *Scedosporium apiospermum, S. boydii* and *Lomentospora prolificans* using CLSI M38 methodology. ICR mice were immunosuppressed with cyclophosphamide and cortisone acetate on days -2, and +3, relative to intratracheal infection with 3.0×10^7^ cells of *S. apiospermum*. For survival studies, treatment with placebo (diluent control), APX001 (26, 52, 104, or 156 mg/kg, po), or liposomal amphotericin B (LAmB, 10 mg/kg, iv) began 16 h postinfection and continued for 7 or 11 days for APX and 4 days for LAmB. To extend the half-life of APX001, mice were administered 50 mg/kg of the cytochrome P450 inhibitor 1-aminobenzotriazole (ABT) 2 h prior to APX001 administration. To assess tissue fungal burden, mice were sacrificed on Day +4 and organs processed for conidial equivalent (CE) by qPCR.

**Results:** The APX001A MEC value for all tested isolates was 0.03 μg/mL. Treatment with ABT alone or LAmB, did not enhance survival of mice infected with *S. apiospermum* vs. placebo mice (n = 20 or 30). Treatment with APX001 at 26, 52, 104 or 156 mg/kg for 7 days enhanced median survival time vs. placebo (10, 12, 11, and 15 days for 26, 52, 104, and 156 mg/kg arms, respectively vs. 7 days for placebo, *P*<0.001). Further, APX001 treatment enhanced overall survival by day 21 when the experiment was terminated (30%, 40%, and 40%, respectively for 26, 52 or 156 mg/kg vs 10% for placebo). Survival after treatment with APX001 for 7 days or 11 days was not different. All APX001 treatments resulted in ~2 log reduction in lung, brain and kidney CE.

**Conclusion:** APX001A is cidal against *S. apiospermum, S. boydii* and *L. prolificans in vitro.* APX001 protected immunosuppressed mice from scedosporiosis due to *S. apiospermum.* Continued investigation of APX001 as a novel antifungal agent against scedosporiosis is warranted.

### P422. Evaluation of new therapeutic candidates for the treatment of Malassezia pachydermatis

SastoqueA.^1^,^2^,^3^EhemannK.^2^TrianaS.^2^,^3^Fernandez-NiñoM.^3^GonzálezA.^3^RestrepoS.^2^RamírezA.M. Celis^2^1Biotechnology Institute, Universidad Nacional de Colombia, Bogotá D.C., Colombia2Biological Sciences, Universidad de los Andes, Bogotá D.C., Colombia3Chemical Engineering, Universidad de los Andes, Bogotá D.C., Colombia

**Objectives:** *Malassezia pachydermatis* is a lipophilic and lipid-dependent yeast, that is part of the microbiota in domestic and wild animals’ skin. However, it could be associated with otitis in canines and bloodstream infections in humans. Diseases caused by *M. pachydermatis* frequently exhibit chronic and recurrent clinical course, and the current antifungal therapy based on azoles is associated with increased resistance as well as significant side effects. Genome sequencing, metabolic network reconstruction, and gene essentiality analyses have been performed, allowing to identify fifteen candidates as therapeutic targets such as homoserine dehydrogenase (HSD), homocitrate synthase (HCS) and saccharopine dehydrogenase (SDH). These enzymes participate in the pathways of L-lysine or L-threonine biosynthesis, which have been reported as antibacterials and with antifungal effect. To assess the impact of these amino acids over the growth of *Malassezia pachydermatis* we evaluated the inhibition of HCS and SDH by L-lysine and HSD by L-threonine based on the feedback control, we additionally conducted *in vitro* susceptibility tests.

**Methods:** Recombinant proteins were expressed in *Escherichia coli* and purified to determine enzymatic activity through spectrophotometry detection of corresponding cofactors. Moreover, *in vitro* susceptibility tests using a modified CLSI M27-A3 method and agar diffusion assays were performed. The concentrations tested were (μg/ml) from 1500 to 4000 using Sabouraud dextrose broth (SDB) with tween 40 and tween 60.

**Results:** Therefore, the results of our study showed L-lysine 1 mM and 75 mM were able to inhibit the enzymatic activity of HCS and SDH, respectively while L-threonine 1 mM inhibited the activity of HSD. Lysine and threonine showed to be competitive inhibitors affecting the substrate-enzyme complex (SE) formation. For L-lysine reduction in turbidity was observed at 3100 µg/mL by microdilution method. In addition, agar diffusion showed a diameter of inhibition of 13 mm using 50 mg/mL. On the contrary, L-threonine did not display inhibitory activity in *M. pachydermatis* at 50 mg/mL as the maximum allowed concentration to be evaluated.

**Conclusion:** This study provides evidence that these three enzymes are efficiently regulated by the feedback of the final product of their corresponding metabolic pathway. L-lysine is a potent inhibitor for *M. pachydermatis,* possible for saturation of metabolic pathways and accumulation of high levels of L-lysine that generate toxicity and amino acids starvation in the cell. L- lysine will be considered as a potential antifungal/fungal static candidate. For L-threonine it is necessary to evaluate higher concentrations.

### P423. The effect of the orotomide antifungal olorofim on the growth and viability of Scedosporium and Lomentospora species.

PréS. Du^1^LawD.^1^SibleyG.^1^ReadN.^2^BromleyM.^2^OliverJ.^1^BirchM.^1^1F2G Limited, Manchester, United Kingdom2Manchester Fungal Infection Group, University of Manchester, Manchester, United Kingdom

**Objectives:** Olorofim is the first member of the new orotomide class of systemic antifungals acting through inhibition of fungal DHODH. Olorofim is active against all species of Aspergillus tested to date including cryptic species. In addition, there is activity against a wide range of filamentous fungi including *Scedosporium, Paecilomyces* and endemic moulds. *Scedosporium* and *Lomentospora* species represent some of the most clinically challenging fungal infections often showing resistance to currently available antifungal agents. Previously olorofim has been shown to be highly active *in vitro* against a range of *Scedosporium* species and the closely related multidrug-resistant species *Lomentospora prolificans* (Wiederhold *et al* 2017, Biwas *et al* 2018). In this study we examined the effects of olorofim on isolates from the *Scedosporium* complex at a cellular level using microscopy and viability dyes.

**Methods:** *In vitro* MICs were undertaken using standard CLSI methodology. Images of the bottom of each well were obtained using a Leica SP8X confocal microscope. Studies on viability were carried out by allowing conidia to germinate for 8h and then exposing to olorofim at the MIC concentration for up to 120h. The viability dye DiBAC was used to determine cell death following exposure to olorofim over time.

**Results:** Olorofim MICs for *S. apiospermum* 451 and *L. prolificans* 206 were both determined to be 0.25 µg/mL. Imaging of microtitre wells above and below this MIC value showed that olorofim dramatically reduced growth of both *S. apiospermum* 451 and *L. prolificans* 206 at concentrations several dilutions below the MIC value. DiBAC staining was conducted with one of the study strains (*L. prolificans* 206) and showed that hyphae began to take up the dye after 24h and that the uptake increased steadily over time. When observations were stopped at 120h, >90% of hyphae had taken up DiBAC and were non-viable.

**Conclusion:** Olorofim demonstrated time-dependent killing of *L. prolificans* hyphae, consistent with previous observations with *Aspergillus spp*. Microscopic analysis of olorofim treated *S. apiospermum* and *L. prolificans* cultures, demonstrated profound effects on growth even at concentrations below the MIC level.

### P425. In Vitro Activity of APX001A (Manogepix) and Comparator Agents against 1,706 Fungal Isolates Collected During an International Surveillance Program (2017)

HubandM.D.^1^PfallerM.A.^1^FlammR.K.^1^BienP.^2^HodgesM.^2^CastanheiraM.^1^1JMI Laboratories, North Liberty, United States of America2Amplyx Pharmaceuticals, San Diego, United States of America

**Objectives:** Current antifungal agents cover a majority of opportunistic fungal pathogens; however, breakthrough invasive fungal infections continue to occur and increasingly involve relatively uncommon yeasts and/or moulds that tend to exhibit decreased susceptibility to current agents. APX001A (manogepix) is a first-in-class small molecule inhibitor of the fungal Gwt1 enzyme that is required for acylation of inositol during glycosylphosphatidylinositol (GPI) anchor biosynthesis. It is active against the major fungal pathogens: *Candida* (except *C. krusei*), *Aspergillus*, and hard-to-treat moulds, including *Fusarium* and *Scedosporium*. In this study, we tested APX001A, anidulafungin (ANF), micafungin (MCF), fluconazole (FLU), and others against 1,706 contemporary clinical fungal isolates collected worldwide during 2017.

**Methods:** A total of 1,706 non-duplicate fungal isolates were collected from 68 medical centers in North America (37.3%), Europe (43.4%), Asia-Pacific (12.7%), and Latin America (6.6%). Among the isolates tested, 78.5% were *Candida* spp, 3.9% were non-*Candida* yeasts, including 30 *Cryptococcus neoformans* var. *grubii* (1.8%), 14.7% were *Aspergillus*, and 2.9% were other moulds. All isolates were tested by CLSI reference broth microdilution methods.

**Results:** APX001A (MIC_50/90_, 0.008/0.06 µg/mL) was the most active agent tested against *Candida* isolates (Figure); corresponding ANF, MCF, and FLU MIC_90_ values were 16- to 64-fold higher. Similarly, APX001A (MIC_50/90_, 0.25/0.5 µg/mL) was ≥8-fold more active than ANF, MCF, and FLU against *C. neoformans* var. *grubii*. Against *Aspergillus*, AXP001A (MIC_50/90_, 0.015/0.03 µg/mL) was comparable in activity to ANF and MCF. APX001A (MIC_90_, 0.06 µg/mL) was ≥128-fold more active than ANF and MCF against *Scedosporium* isolates.

**Conclusion:** APX001A demonstrated potent *in vitro* activity against 1,706 fungal isolates, including echinocandin- and fluconazole-resistant strains. The extended spectrum of APX001A was also notable for its potency against many less common, yet antifungal-resistant, strains such as *Candida auris*, *A. lentulus*, *A. ustus, Fusarium solani* species complex, and *Scedosporium*. Further studies are needed to demonstrate the utility of APX001A in difficult-to-treat resistant fungal infections. 

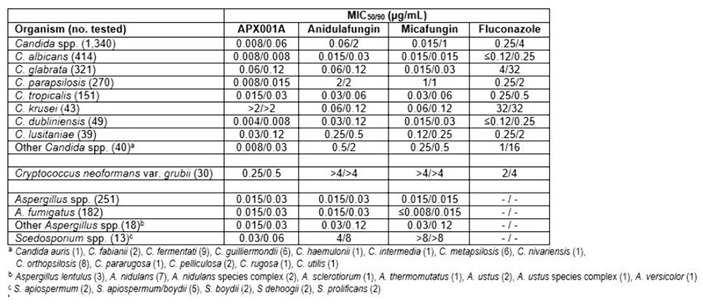



### P426. Rezafungin In Vitro Activity against Phase 2 STRIVE Part A and Contemporary Nordic Clinical Candida Isolates Determined by the EUCAST Reference Method

HellebergM.^1^JørgensenK.M.^2^DatcuR.^2^HareR.^2^ArendrupM.C.^2^,^3^,^4^1Dept. of Infectious Diseases, Rigshospitalet, Copenhagen, Denmark2Unit of Mycology, Statens Serum Institut, Copenhagen, Denmark3Rigshospitalet, Dept. of Clinical microbiology, Copenhagen, Denmark4University of Copenhagen, Dept. of Clinical Medicine, Copenhagen, Denmark

**Objectives:** Rezafungin is a novel echinocandin with favourable safety and pharmacokinetic characteristics, which allows for high drug exposures and once-weekly dosing. Epidemiological cut-off values (ECOFFs) of rezafungin have not yet been established. We determined the *in vitro* activity of rezafungin and four comparators against contemporary Nordic clinical isolates of *Candida* and other yeasts and established single centre wildtype upper limits (WT-UL). Subsequently, these WT-UL were used to evaluate rezafungin susceptibility for clinical isolates from the completed part A of STRIVE, the phase 2 trial of rezafungin for treatment of candidaemia and invasive candidiasis (NCT02734862).

**Methods:** 1,249 clinical isolates (19 *Candida,* 13 other yeast species) from Denmark, Norway and Sweden received at the Danish mycology reference laboratory in 2017-18 were identified using CHROMagar, matrix-assisted laser desorption ionization–time of flight mass spectrometry and, when needed, internal transcribed spacer sequencing. EUCAST E.Def 7.3.1 susceptibility testing included rezafungin, anidulafungin, micafungin, amphotericin B and fluconazole. WT-UL were established following EUCAST principles for visual and statistical ECOFF value setting for rezafungin and for comparators against species without EUCAST ECOFFs, allowing classification as wildtype or non-wildtype. The *FKS* target genes were sequenced for echinocandin non-wildtype isolates.

**Results:** Rezafungin MIC distributions for the most common *Candida* species and modal MICs for rezafungin and comparators are shown in Table 1. Rezafungin had species-specific *in vitro* activity similar to that of anidulafungin and micafungin. At a mg/L basis rezafungin was overall less active (modal MICs ≥ 2 two-fold dilutions higher) than anidulafungin and micafungin, but equally or more active than fluconazole and amphotericin B, except against *C. parapsilosis*, other *Candida* (amphotericin B only) and other yeasts. Rates of rezafungin non-wildtype isolates among the most common *Candida* species were <3%, only marginally higher than for amphotericin B and lower than for fluconazole, anidulafungin and micafungin (Table 2). We identified 27 (2.0%) echinocandin non-wildtype isolates: *C. albicans* (n = 11, 1.9%), *C. glabrata* (n = 11, 3.3%), *C. tropicalis* (n = 2, 2.7%), *C. dubliniensis* (n = 2, 2.9%) and *C. krusei* (n = 1, 1.2%). Alterations in Fks1 and/or Fks2 hot-spots (or within ±3 amino acids) were found in 20 of 25 sequenced non-wildtype isolates: Fks1: *C. albicans*: D648Y, P1354P/S (2), P1354S (3), R1361R/S, R1361G; *C. tropicalis*: F650S and S654P; *C. dubliniensis*: S645P (2); *C. krusei*: S659F. Fks2: *C. glabrata*: S663P (2), S663F (3). Fks1+Fks2: *C. glabrata*: S663F/L630Q and L664Q+Y658N+premature stop-codon. The rezafungin MICs for non-wildtype isolates were 0.25–2 mg/L. Rezafungin susceptibility testing of 95 *Candida* blood isolates from the STRIVE trial yielded MIC distributions comparable to those for the Nordic clinical isolates (Table 1). One *C. rugosa* isolate had an MIC of >8 mg/L, suggesting intrinsic resistance, and one *C. glabrata* isolate had an MIC of 0.5 mg/L, but no mutations in *FKS* target genes.

**Conclusion:** Rezafungin displayed broad *in vitro* activity with a species-specific susceptibility pattern similar to that of other echinocandins. WT-UL were suggested for the most common species. Few non-wildtype strains with alterations in *FKS* target genes or reduced susceptibility to rezafungin were identified among clinical *Candida* isolates from Nordic surveillance and the STRIVE trial.

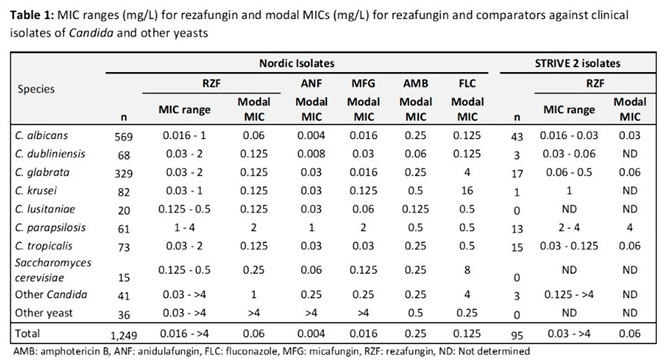

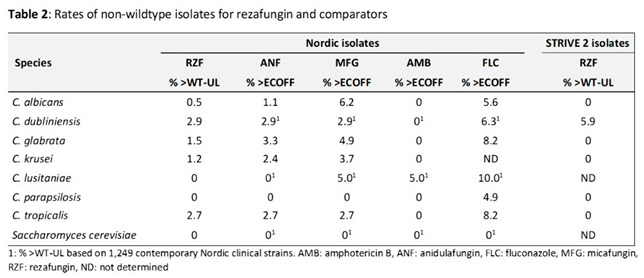





### P427. An evaluation of the safety and efficacy of nebulised amphotericin B deoxycholate (Fungizone) for treatment of pulmonary aspergillosis

LangridgeP.OtuA.DenningD.National Aspergillosis Centre, Manchester University NHS Foundation Trust, Manchester, United Kingdom

**Objectives:** We evaluated the safety and efficacy of nebulised Fungizone in the long-term treatment of various forms of pulmonary aspergillosis

**Methods:** This was a retrospective service evaluation of 177 patients with various forms of pulmonary aspergillosis attending the National Aspergillosis Centre in Manchester. Due to risk of intolerance/bronchospasm, patients first received a challenge test with nebulised Fungizone in the hospital setting with spirometry pre/ post Fungizone. To improve tolerability, nebulised short acting beta agonist was given prior to administration of Fungizone (10mg in 4ml water for injection).

**Results:** The baseline FEV1 and FVC of the patients ranged from 0.94-2.97L and 13.3-5.15L respectively. 66% (117) were able to tolerate their first test dose, of whom 26 (21%) stopped using the nebulised therapy before their next clinic visit. Reasons cited for Fungizone intolerance included increased breathlessness (8), wheeze (7), bronchospasm (6), sore throat/voice (4), coughing (4), chest tightness (3), light-headedness/dizziness (2), diarrhoea (2), fatigue (2), mouth blisters/ulcers (2), headaches (2), nausea/vomiting (2) in addition to other symptoms. Eighteen continued nebulisation of Fungizone 10mg twice a day for >3 months. Eleven had allergic bronchopulmonary aspergillosis (ABPA), 4 had severe asthma with fugal sensitization (SAFS), 2 had Aspergillus bronchitis and 1 had Aspergillus sensitisation with cavitating nodules. Treatment courses ranged from 4months to 4years. At their first clinic follow-up, there were reported benefits with sputum production (7), sinus symptoms (2), dyspnoea (2), exercise tolerance (2), reduction in prednisolone maintenance dose (1) and one demonstrated radiologic improvement. Three had interruptions to their Fungizone treatment which had to be restarted following clinical deterioration off therapy (2 of whom had dose change to once daily 12.5mg). Five patients have remained on treatment with sustained benefit (e.g. clinical stability, marked reduction in frequency of chest infections, reduction in Aspergillus specific IgE and increased exercise tolerance). Four patients discontinued therapy as there was no perceived clinical benefit. Aspergillus PCR on sputum at first repeated measure following initiation of Fungizone treatment changed from positive to negative in 7 and stayed negative in 7. No pre-treatment PCR tests were done in 5 patients and no follow-up PCR tests were performed in 6 patients. Fungal cultures of sputum went from positive to negative after >3 months treatment in 6 patients, remained positive in 3 patients, remained negative in 4 patients and became positive in 2 patients. 

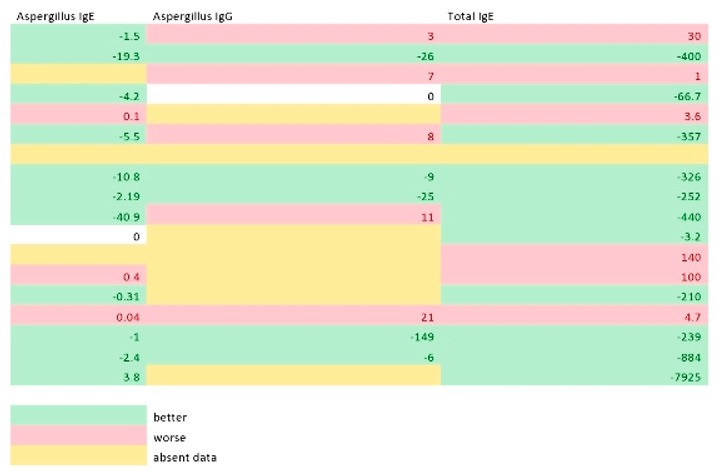



**Conclusion:** Nebulised Fungizone is a poorly tolerated treatment for pulmonary Aspergillosis with high dropout rates. There appears to be both clinical and serologic benefits following sustained treatment with nebulised Fungizone in some patients

### P428. In-Silico and In-Vitro Evaluation of Anticandidal Activity of Cinnamaldehyde

UttamG.^1^SinghK.^1^KatiyarD.^2^1Animal Mycology Laboratory, zoology, Mmv, Banaras Hindu University, Varanasi, India2Chemistry, Mmv, Banaras Hindu University, Varanasi, India

**Objectives:** *Candida albicans* is a common commensal which resides in human body and causes invasive fungal infections especially in immunocompromised hosts. This study aims to evaluate the anticandidal activity of cinnamaldehyde through *in-silico* approach and *in-vitro* methods.

**Methods:** Interactions between cinnamaldehyde and potential drug targets of *C. albicans* was elucidated using *in-silico* approach which was further validated by determination of minimum inhibitory concentration (MIC) and the minimum fungicidal concentration (MFC) of cinnamaldehyde using broth microdilution method recommended by CLSI 2009 for yeast (M27-A2). The effect of cinnamaldehyde on micromorphology of *C. albicans* was studied. Sorbitol Assay (effect on cell wall) and Ergosterol Binding Assay of *C. albicans* were also performed. Time-kill curve was conducted using cell viability testing.

**Results:** *In-silico* studies revealed that cinnamaldehyde showed good binding affinity with potential targets of *C. albicans* such as ergosterol, N-myristoyl transferase and Secreted aspartic proteinase 5. MIC and MFC of cinnamaldehyde were found to be 8.2 μg/ml and 16.4 μg/ml respectively. In the morphological interference assays, it was observed that the cinnamaldehyde inhibited pseudohyphae, blastospores and chlamydospores formation. The time-kill curve of cinnamaldehyde showed that it required only few hours of exposure to effectively kill >90% of the inoculum.

**Conclusion:** Cinnamaldehyde showed good *in-vitro* antifungal potential against *C. albicans*. However, evaluation of pharmacodynamics and pharmacokinetics studies is desirous for complete elucidation of its action as a therapeutic which will be helpful for mankind.

### P429. Antifungal Activity of Cinnamaldehyde against Some Human Pathogenic Fungi

SinghK.KumariA.UttamG.SrivastavaN.Animal Mycology Laboratory, Department of Zoology, Mmv, Banaras Hindu University, Varanasi, India

**Objectives:** Despite the availability of effective antifungals, the morbidity and mortality of fungal infections has remained high especially in individuals with impaired immunity. The limited targets of existing antifungals may be one of the reasons for the emergence of drug-resistant strains. In this regard, the anti-fungal potential of cinnamaldehyde, a plant product and active constituent of cinnamon was tested through *in-silico* approach which was further validated by *in-vitro* methods.

**Methods:** Antifungal activity of cinnamaldehyde was tested against Cryptococcus neoformans (ATCC 6352), Candida albicans (MTCC 3017), Aureobasidium pullulans var. pullulans (CBS 577.93), Aspergillus terreus, Alternaria alternata and Fusarium oxysporum (MTCC 2773) using broth micro-dilution methods recommended by CLSI (2009) for yeasts and moulds. Further, PDB structures of drug targets of said fungus species were downloaded or molleded (if not available) and their interactions with cinnamaldehyde were elucidated in-silico using RCSB Protein databank, PHYRE2 and PatchDock servers.

**Results:** Cinnamaldehyde showed potent anti-fungal activity against all the fungal pathogens screened. However, *Cryptococcus neoformans* (ATCC 6352) was found highly susceptible against it with the MIC value 1.367±0.260 mg/ml. *In-silico* interaction between targets of *Cryptococcus neoformans* (lanosterol 14-alpha demethylase), *Candida albicans* (ergosterol, N-myristoyl transferase and Secreted aspartic proteinase 5), *Aureobasidium pullulans* (chitinase), *Aspergillus terreus* (chitinase), *Alternaria alternata* (ornithine descarboxylase) and *Fusarium oxysporum* (Thiamine thiazole synthase) with cinnamaldehyde exhibited high binding energies and hydrogen bonds.

**Conclusion:** Cinnamaldehyde possesses broad spectrum and potential anti-fungal activity. Being a plant product, it also has advantage for structural diversity and therefore, could be a potential drug candidate for the treatment of fungal diseases. However, its drug likeness properties should be evaluated by *ex-vivo* and *in-vivo* studies.

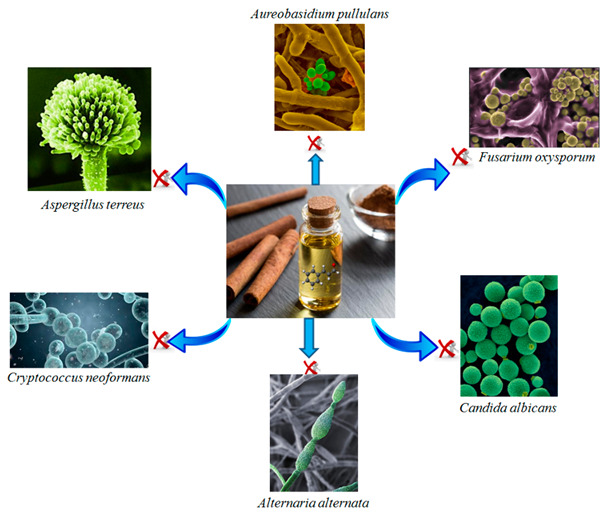



### P430. rAssessment of anti-Candida activity of Benzo(a)phenoxazines

Carvalho-PereiraJ.I.^1^,^2^GonçalvesM.S.^3^SousaM.J.^1^,^2^SampaioP.^1^,^2^1Centre of Molecular and Environmental Biology (cbma), Department of Biology, University of Minho, Braga, Portugal2Institute of Science and Innovation For Bio-sustainability (ib-s), University of Minho, Braga, Portugal3Centre of Chemistry, University of Minho, Braga, Portugal

**Objectives:** Fungal infections represent a major health problem due to their prevalence, increasing number of cases and high complexity of the etiological agents involved. *Candida* species are the most common and clinically important being able to cause from less-severe superficial lesions to life-threatening disseminated mycosis. *C. albicans* remains the most common species isolated, however, infections caused by non-*albicans* species, such as *C. auris,* are increasing. Current treatments for fungal infections are not 100% effective due to the drug toxicity, pathogen resistance, low drug bioavailability and late diagnosis. At the moment, few groups of antimycotics are available to deal with these infections. The loss of clinical efficacy of these drugs creates a potential risk of spreading of resistant strains through the emergence of clones with high pathogenic potential. This illustrates that the development of new antimicrobials is crucial. Phenoxazine derivatives, namely benzo(*a*)phenoxazines have been shown to present good antifungal properties. Thus, in this work we evaluated the antifungal activity of five benzo(α)phenoxazin derivatives, against distinct *Candida* species and selected the most promising to develop an effective antifungal nanoformulation.

**Methods:** The revised *European Committee on Antimicrobial Susceptibility Testing* (EUCAST) protocol was used to determine the minimum inhibitory concentrations (MICs) of five benzo(α)phenoxazines against seven *Candida* species, namely *C.albicans, C.glabrata, C.parapsilosis, C.tropicalis, C.krusei, C.bracarensis* and *C.auris.* The most effective compounds were selected for the development of formulations based on DODAB:MO liposomes. All formulations were evaluated regarding the mean size, polydispersity index, surface charge, liposome stability and encapsulation efficiency. The most stable formulations were selected for antifungal activity re-evaluation against all seven *Candida* species and for cytotoxicity assessment with different cell lines and using both the MTT and the lactate dehydrogenase (LDH) release assays.

**Results:** demonstrated that all five benzo(α)phenoxazin derivatives tested presented antifungal activity against all seven *Candida* species, with MICs between 3.75 to 60 μM. Two of these benzo(*a*)phenoxazin derivatives presented MICs of 3.75-15 μM against all species tested, being selected to develop DODAB:MO formulations. The liposomal formulations were positively charged, presenting mean sizes between 300-500nm and polydispersity indexes of 0.4-0.5. The encapsulation efficiency was between 80-95% for both benzo(α)phenoxazin derivatives tested. The activity of formulation against all seven *Candida* species were evaluate with the liposomal formulations. Results demonstrated that empty liposomes did not affect *Candida* growth and that the encapsulation of benzo(α)phenoxazins increased their activity against *Candida* species. The cytotoxicity of the promising formulations was tested using different cell lines and the non-toxic formulations were selected.

**Conclusion:** The results obtained demonstrated that benzo(α)phenoxazins derivatives could be potential antifungal drugs since present activity against all *Candida* species tested, including the multidrug resistant *C. auris*. Moreover, the encapsulation of the benzo(α)phenoxazin derivatives increase the antifungal activity and reduce the cytotoxicity of compounds.

### P431. Inhibitory effect of a curcumin-based compound against Epidermophyton floccosum clinical isolates

PattiniV. Camilo^1^PolaquiniC.R.^1^LemesT.^1^BrizzottiN.^2^SiqueiraJ.P.^2^AlmeidaM.^2^RegasiniL.O.^3^1São Paulo State University - Institute of Biosciences, Humanities and Exact Sciences (UNESP - IBILCE), São José do Rio Preto, Brazil2FAMERP, São José do Rio Preto, Brazil3Chemistry and Environmental Sciences, UNESP (Sao Paulo State University), São José do Rio Preto, Brazil

**Objectives:** The present study aimed to evaluate the antifungal activity of a curcumin-based compound against clinical strains of *Epidermophyton floccosum* isolated from dermatomycoses.

**Methods:** For this study, the curcumin-based compound was obtained from the Laboratory of Antibiotics and Chemotherapeutics – LAQ, of the Institute of Biosciences, Letters and Exact Sciences (IBILCE, Unesp São José do Rio Preto). This new compound is a hybrid curcumin compound with substitutions to make the substance more stable and active. The clinical strains were from the culture collection of the Laboratory of Microbiology of the Medical School in São José do Rio Preto (FAMERP), Brazil. Susceptibility tests were performed for two *E.floccosum* strains (6069 and 6113), using the Clinical and Laboratory Standards Institute M38-A2 guidelines as reference. Percentage of inhibition was calculated by spectrophotometry using the: I = 1 - (AbsT-AbsCT / AbsCC) × 100; where: I = percentage of inhibition; AbsT = absorbance of the inoculum plus the compound solution; AbsCT absorbance of sterility control; AbsCC = absorbance of growth control. The curcumin (Merck®) was used as a control.

**Results:** The minimum inhibitory concentration (MIC) obtained for the curcumin-based compound against *E.floccosum* 6069 and 6113 strains was 15.62μg/mL, inhibiting 80 and 87% the growth of the strains, respectively. On the other hand, pure curcumin (Merck®) exhibited no activity at the concentrations tested.

**Conclusion:** The curcuminoid showed potent antifungal activity against *E. floccosum* strains, higher than pure curcumin (Merck®), showing a potential alternative against *E. floccosum.* In the future, toxicity tests will be performed allowing new therapeutic approaches in the treatment of dermatomycoses.

### P432. Antifungal activity of Kombucha Hibiscus sabdariffa infusion extract against Trichophyton mentagrophytes and Trichophyton rubrum.

PattiniV. Camilo^1^BrizzottiN.^2^LemesT.^1^SiqueiraJ.P.^2^MarquesM.^2^CaetanoM.^3^Maschio-LimaT.^3^AlmeidaB. De^3^BiancoL.^1^SilvaC.^2^PolaquiniC.R.^1^RegasiniL.O.^4^CastilhoE.^2^AlmeidaM.^2^1São Paulo State University - Institute of Biosciences, Humanities and Exact Sciences (UNESP - IBILCE), São José do Rio Preto, Brazil2FAMERP, São José do Rio Preto, Brazil3São Paulo State University - Institute of Biosciences, Humanities and Exact Sciences (UNESP - IBILCE), São José do Rio Preto, Brazil4Chemistry and Environmental Sciences, UNESP (Sao Paulo State University), São José do Rio Preto, Brazil

**Objectives:** The present study aimed to explore the antifungal potential of Kombucha infusion extracts on planktonic cells of potentially pathogenic fungal isolates, as an alternative to synthetic drugs.

**Methods:** Firstly, a SCOBY (*Symbiotic Culture of Bacteria and Yeast*), obtained from a local natural products store, was placed in 1 liter of distilled water with 20g of sucrose weekly for 30 days to remove any previous residues. The *Hibiscus sabdariffa* infusion (HI) was prepared with four grams of leaves/flowers boiled in 450 mL of sterile mineral water for 5 minutes and filtered on filter paper. The initial fermentation was obtained by mixing 20 g of SCOBY and 80 g of sucrose to the infusion, and the final volume was adjusted to 800 mL with sterile mineral water. The culture was then incubated at 25 °C for 20 days. The experiment was carried out in triplicate. After incubation, the solution was filtered in the MILLIPORE^®^ System with 0.2 μm membrane. The chemical compounds of the filtrate were extracted using ethyl acetate. To obtain the dry and purified extract of the chemical compounds, the acetate phase was placed on a rotary evaporator. Minimal inhibitory concentrations (MIC) and minimal fungicide concentrations (MFC) of Kombucha HI extract against *Trichophyton rubrum* and *Trichophyton mentagrophytes* clinical isolates were investigated by the microdilution method, using as reference the document M38-A2 of the *Clinical and Laboratory Standards Institute*.

**Results:** The results obtained for the MIC of Kombucha HI extract against the *T. mentagrophytes* and *T. rubrum* strain were 125 μg/mL and 62.5 μg/mL, respectively. For the MFC, values observed were 250 μg/mL for *T. mentagrophytes* and 125 Oμg/mL for *T. rubrum.*

**Conclusion:** Kombucha HI extract showed antifungal activity against *T. mentagrophytes* and *T. rubrum*. In the future, isolation and identification of the extract compounds may allow new therapeutic approaches in the control of fungal infections.

### P433. Characterization of the specificity of the Ca37 monoclonal antibody and its protective role against Candida albicans infection

AntoranA.^1^Aparicio-FernandezL.^1^BuldainI.^1^Martin-SoutoL.^1^AreitioM.^1^RementeriaA.^1^GhannoumM.A.^2^FuchsB.B.^3^MylonakisE.^3^HernandoF.L.^1^Ramirez-GarciaA.^1^1Immunology, Microbiology and Parasitology, University of the Basque Country (UPV/EHU), Leioa, Spain2Dermatology, Case Western Reserve University, Cleveland, United States of America3Division of Infectious Diseases, Rhode Island Hospital and Alpert Medical School of Brown University, Providence, United States of America

**Objectives:** *Candida albicans* is a commensal yeast of mucosal surfaces of human body. However, when the host is immunocompromised, this yeast can cause invasive infections, that present an unacceptably high mortality rate (up to 50%). Therefore, looking for alternative treatments, we have focused on monoclonal antibodies. Alcohol dehydrogenase (Adh) protein was selected for antibody development, due to its role in carcinogenic acetaldehyde production, its surface location and its immunogenicity potential. The objective of this work was to characterize the specificity of this Ca37 monoclonal antibody against *Candida albicans* and its ability to interfere with yeast infection.

**Methods:** Adh1 protein was cloned in *Escherichia coli*. This recombinant protein was used for mice immunization and Ca37 monoclonal antibody development. Proteomic analysis by two dimensional (2-DE) western blotting and LC-MS/MS identification, in yeast and hyphal fractions, were performed in order to identify the proteins detected by Ca37 and to determine its specificity against Adh1 protein. Indirect immunofluorescence was carried out to study surface location of the protein. In this case, sodium metaperiodate treatment was used for surface carbohydrate removal. Effect of Ca37 on yeast germination, growth and interaction with antifungals was studied. *Galleria mellonella in vivo* model infection, using an *ΔADH1* mutant and parental strains was used to study the effect of *ADH1* gene loss on virulence, and for testing Ca37 antibody role as therapeutic agent against *C. albicans* infection.

**Results:** The 2-DE western blot performed on cell wall-associated protein fraction showed that Ca37 detected Adh1 protein in yeast and Adh2 in hyphal cells. Immunofluorescence results indicated that the epitope recognized by the Ca37 was partially covered by carbohydrates, as antibody only bound to fungal cells after sodium metaperiodate treatment, or after long incubation times. Yeast germination was slightly inhibited, but growth inhibition reached up to 90%. The antibody also showed an additive effect with amphotericin B and fluconazole antifungals. Finally, in *Galleria mellonella* infection, we detected a higher survival when using the *ΔADH1* strain, and the same level of survival using fluconazole or Ca37 treatment.

**Conclusion:** The Ca37 monoclonal antibody is specific for Adh proteins, which are located at fungal cell surface, partially hidden by carbohydrates, and seem relevant for virulence *in vivo*. Moreover, Ca37 is effective against yeast growth, provides an additive effect with antifungal drugs, and confers protection *in vivo*. Altogether, Adh proteins seem interesting targets against *Candida albicans* infections. *Financial support: This study was founded by a grant of UPV/EHU (PPG17/41). LA-F is recipient of a PhD grant of UPV/EHU and LM-S of the Basque Government.*

### P436. Rezafungin Clinical Safety and Efficacy in the Treatment of Candidemia and/or Invasive Candidiasis: Combined Results from the STRIVE Phase 2 Trial Parts A and B

ThompsonG.R.^1^HonoreP.M.^2^HorcajadaJ.P.^3^FortunJ.^4^HitesM.^5^BassettiM.^6^MenaK.^7^NavaltaL.^7^VianiR.^7^SandisonT.^7^PappasP.^8^1University of Davis Medical Center, Davis, United States of America2Brugmann University Hospital, Brussels, Belgium3Hospital del Mar, Barcelona, Spain4University Hospital Ramon y Cajal, Madrid, Spain5Erasme Hospital, Brussels, Belgium6Dept of Medicine, University of Udine, Udine, Italy7Cidara Therapeutics, San Diego, United States of America8University of Alabama at Birmingham, Birmingham, United States of America

**Objectives:** Rezafungin (RZF) is a novel echinocandin designed to have next-generation properties, such as increased stability and safety and novel pharmacokinetics/pharmacodynamics (PK/PD), distinct from other systemic antifungals. The PK profile of RZF includes a prolonged half-life and front-loaded plasma exposure that allow for once-weekly therapy, as well as maximized pharmacometrics of antifungal efficacy. STRIVE is a global, double-blind, randomized, clinical trial that compared the safety and efficacy of RZF once-weekly to caspofungin once-daily in patients with candidemia and/or invasive candidiasis (IC). After successful completion of STRIVE Part A (Thompson et al, 2018), a second randomization (Part B) was conducted. Combined results from Parts A and B are presented herein.

**Methods:** Adults (≥18 y) with systemic signs and mycological confirmation of candidemia and/or IC were randomized to RZF once-weekly or standard of care (SOC) with caspofungin for ≥14 days (up to 4 weeks) (Figure 1). Efficacy was assessed by overall response (resolution of clinical signs of infection + mycological eradication) at day 14 (primary endpoint) and PI assessment of clinical response at day 14 and all-cause mortality at day 30 (secondary endpoints). Safety was evaluated by adverse events (AEs) and mortality. 

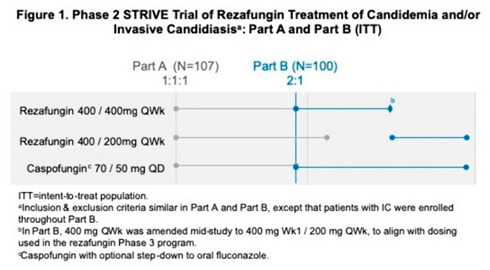



**Results:** of 207 patients enrolled, 183 patients were included in the microbiological intent-to-treat population. Treatment groups were well balanced and matched in demographics and baseline characteristics. Patients with IC comprised ~21% of the combined population. RZF 400 mg/200 mg demonstrated greater efficacy than SOC, with the highest rates of overall success and clinical cure and the lowest rate of 30-day all-cause mortality of all 3 groups (Table 1). The rate of all-cause mortality through follow-up in the pooled RZF group was 15.6% (19/122) and 19.7% (12/61) for SOC. No concerning safety trends were observed. The incidence of ≥1 treatment-emergent adverse event (TEAE) related to study drug was 13/134 (9.7%) among RZF-treated patients (pooled) and 9/68 (13.2%) with SOC. Between the pooled RZF and SOC groups, rates of study drug–related TEAEs leading to study discontinuation (2.2% and 1.5%, respectively) and serious AEs (1.5% and 2.9%, respectively) were comparable. 

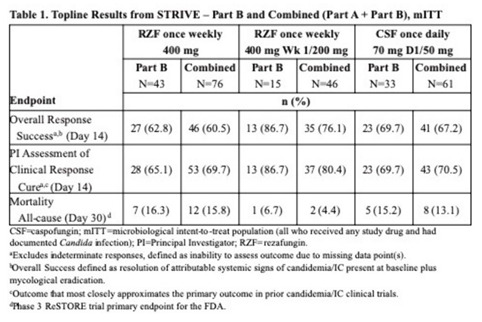



**Conclusion:** RZF was safe and efficacious in the Phase 2 trial of RZF treatment in patients with candidemia and/or IC. RZF 400 mg/200 mg, the dosing regimen used in the ongoing Ph 3 study, demonstrated greater efficacy than SOC and the most favorable efficacy of all groups. No concerning trends in TEAEs were observed and, again, the RZF 400 mg/200 mg once weekly group demonstrated the most favorable safety outcomes in terms of TEAEs related to study drug. The recently completed Part B of STRIVE, combined with the previous success of STRIVE Part A, demonstrates the safety and efficacy of RZF treatment and supports its Phase 3 development.

### P440. rInvasive aspergillosis in adult non-hematologic patients

ShadrivovaO.^1^TonkoshkurM.^1^DesyatikE.^2^VolkovaA.^3^PopovaM.^3^ErmolovaS.^4^BogomolovaT.^2^IgnatyevaS.^2^ZubarovskayaL.^3^AfanasyevB.^3^KlimkoN.^1^1Department of Clinical Mycology, Allergy and Immunology, North-Western State Medical University named after I.I.Mechnikov, St. Petersburg, Russian Federation2Kashkin Research Institute of Medical Mycology, North-Western State Medical University named after I.I.Mechnikov, St. Petersburg, Russian Federation3I.Pavlov First Saint Petersburg State Medical University, St. Petersburg, Russian Federation4Department of Pulmonology, 3Leningrad Regional Clinical Hospital, St. Petersburg, Russian Federation

**Objectives:** To investigate the features of invasive aspergillosis (IA) in non-hematologic patients.

**Methods:** Retrospective analysis of clinical data of adult non-hematologicpatients with IA. The EORTS/MSG 2008 criteria were used for IA diagnosis and assessment of response of therapy.

**Results:** In the study were included 46 non-hematologic patients with IA, median age – 48 years (19 - 99), females – 57%. The background conditions were autoimmune diseases – 24%, kidney diseases with acute or chronic renal failure – 20%, oncology – 15%, heart and vascular diseases – 11%, severe viral-bacterial pneumonia – 8%, HIV infection – 7%, lung pathology – 7%, severe influenza H3N2 – 4%, other – 4%. The risk factors of IA were glucocorticosteroid therapy – 50%, prolonged lymphocytopenia (<1,0 ×10^9^/l, median – 18 days) – 48%, immunosuppressive therapy – 35%, ICU stay – 30%, precede surgical treatment – 28%, organs transplantation – 20%, severe neutropenia (median – 10 days) – 13%, diabetes – 11%, allogeneic hematopoetic stem cells transplantation (HSCT) – 2%, autologous HSCT – 2%. The main sites of infection were lungs – 85%, central nervous system (CNS) – 11%, and paranasal sinuses – 9%. Disseminated (≥2 organs) IA was in 17% patients. Clinical signs of IA were fever (≥ 38,5 °C) – 79%, cough – 76%, respiratory failure – 59%, chest pain – 26%, and hemoptysis – 22%. CT scan signs were bilateral lesions – 56%, air crescent sign – 12%. The main etiological agents were *A.fumigatus* (62%), *A.niger* (14%), *A.flavus*(14%), *A.ustus* (5%) and *A.ochraceus* (5%). «Proven» IA was diagnosed in 28% patients, «probable» – 72%. Antifungal therapy was used in 93% patients, surgical treatment – 7%. Overall 12-weeks survival of patients was 76%.

**Conclusion:** Invasive aspergillosisdeveloped in non-hematologic patientsusually with autoimmune (24%), renal (20%) or oncology diseases (15%). The main risk factors were glucocorticosteroid therapy (50%), lymphocytopenia (48%), immunosuppressive therapy (35%), organs and hematopoetic stem cells transplantation (24%). The main etiological agents were *A.fumigatus* (62%), *A.niger* (14%), and *A.flavus*(14%). The main sites of infection were lungs (85%), disseminated (≥2 organs) infection was in 17% patients. Overall 12-weeks survival of patients was 76%.

### P441. A plea to preserve fungal pathogens in public microbial resource centres

StubbeD.^1^HendrickxM.^2^BeckerP.^2^1Mycology & Aerobiology, Bccm/ihem Collection, Sciensano, Brussels, Belgium2Mycology, Sciensano, BCCM/IHEM, Brussels, Belgium

**Objectives:** Open science aims at sharing scientific outputs in order to maximize the impact of research. This allows follow-on studies, facilitates new discoveries, improves reproducibility of experiments and favours transparency of results. Although open data is becoming a well-known concept, less attention is given to the availability of research materials. In medical sciences, public culture collections represent an historical example of open science, thanks to their longstanding experience in the preservation of living microorganisms and their distribution for further scientific investigations or development. These microbial resource centres provide well-characterized, quality-controlled and authenticated strains and associated data. In medical mycology, the diversity of fungal pathogens is important and needs to be secured following (inter)national legislations for future basic and applied health questions. We wish to promote the deposit of isolates in public collections. However, the responsibility to make fungal strains available is shared by researchers, funding agencies and publishers.

**Methods:** We made a random selection of articles in the field of medical mycology to check whether or not studied isolates were deposited and made available in public collections.

**Results:** A random selection was made of 90 articles issued between 2017 and 2019 in seven journals specialized in medical mycology. Out of them, only 13 (less than 15%) mentioned the deposit of the strains.

**Conclusion:** Medical mycologists need to be more aware towards strain conservation. Governmental funding policies should request the deposit of strains isolated during financed projects. Regarding publishers, most journals encourage authors to make biological material available but few specifically require deposit and most isolates investigated in publications are not preserved. Editors should therefore implement mechanisms for active agreement by authors to deposit strains when submitting an article. Such mechanisms could follow Transparency and Openness Promotion guidelines^1^ for journals that include a standard for research materials. Two level of stringency are proposed for this standard: (1) the article states whether strains are available and, if so, where to access them and (2) the mandatory deposit of the strains in a trusted repository.

### P442. Fungicidal and Yeasticidal activity of chemical disinfectant and antiseptic products: European standards & Real hospital life - How to be successful?

Loeffert-FrémiotS.Microbiology Laboratory, Member of Wg 1 of The Cen, Laboratoires ANIOS, LILLE, France

**Objectives:** Chemical disinfectants and antiseptic products are a crucial part of the infection control strategy in hospital and are upregulated by European standards. If infection control fails, the consequences could be severe especially for immunocompromised. This is why it is important that testing procedures of chemical chemical disinfectants are sufficient and well understand to ensure that disinfectants for the medical area are reliable. The aim of this work is to help users to understand where, when and how to use these products and to make and update of the conception and evolution of these standards following what is happening in real work.

**Methods:** Explain the European Standards to which products have to conform in order to support the claims for Fungicidal and yeasticidal activity. This include the description of EN 14885 “Chemical disinfectants and antiseptics – Application of European Standards for chemical disinfectants and antiseptics” (2018) requirements and the tests conditions of the EN 13624 “Chemical disinfectants and antiseptics – Quantitative suspension test for the evaluation of fungicidal or yeasticidal activity in the medical area -Test method and requirements (phase 2, step 1)” (2013). In order to adapt the European standards to what happen in medical area, standards are systematically revised by group of expert members of the Working Group 1 of the European Comission of standardization (Commission Europeenne de Normalisation: CEN).

**Results:** It is known that some strains could be challenging for healthcare workers by their emerging character or by the apparition/ evolution of their resistance in different part of European country. Therefore, European standards need to continuously follow and adapt to the field situation. The choice of test organisms and conditions of EN 13624 is under discussion to see their relevance. It is a critical point that need to be raised and share with expert people of the field.

**Conclusion:** European Standards requirements must be continuously revised to be suitable for effective infection strategy in medical area and particularly in healthcare settings. These standards are more often not really known by healthcare people working on the field, fighting everyday against resitant pathogens. They need to have all the keys to ensure a reliable startegy against these pathogens.

### P443. Comparing the efficacy of three adsorbents, bentonite, activated charcoal and fuller’s earth on the detoxification of aflatoxin-contaminated feeds.

MgbeahuruikeA.Department of Veterinary Pathology and Microbiology, Faculty of Veterinary Medicine, University of Nigeria, Nsukka, Nsukka, Enugu, Nigeria

**Objectives:** To test the ability of three low-cost and locally available adsorbents (activated charcoal, bentonite and fuller’s earth) to detoxify poultry feeds contaminated with aflatoxin.

**Methods:** Mould growth was induced on poultry feeds by sprinkling water on the feeds until the feed was fully wet, the feed was packed in dark nylon bags and covered tightly. The bags were stored in dark environments for 3 weeks. The feeds were divided into 4 groups, Contaminated but untreated, Contaminated but treated with bentonite, Contaminated but treated with Activated charcoal and contaminated but treated with Fuller’s earth. Aflatotoxin concentration in the feeds was measured using reverse phase HPLC. Fungal contaminants in the feeds were isolated and molecularly identified. Phylogentic analysis was reconstructed using MEGA 5.0. Measurement of performance indices of the birds like heamatological and serum biochemical parameters were carried out and analysed statistically using Minitab.

**Results:** Bentonite was the most effective adsorbent and lowered the total aflatoxin concentration from 120 ± 38 µg/kg to 15 ± 5.0 µg/kg, while the concentration was reduced to 20 ± 6.5 µg/kg in feed treated with fuller’s earth. The three adsorbent treatments resulted in 63-100% weight increase of the birds, compared with birds fed the untreated, aflatoxin-containing feed. Based on sequencing of the internal transcribed spacer and phylogenetic analysis, 86% of the identified fungal contaminants were *Aspergillus* species, with *Aspergillus tamarii* and *A. nominus* being the most prevalent with a frequency of occurrence of 21% each. The number of white blood cells in blood samples was increased by 13-17% in birds that consumed adsorbent-treated feed, compared with the untreated, aflatoxin-contaminated feed. Serum biochemical analysis further showed that aspartate amino transferase activity was 2.3 to 2.6 fold higher in birds that consumed the contaminated but untreated feed, compared with feed treated with adsorbents. Hepatic lesions were also prominent in the liver of the birds fed contaminated but untreated feed but were reduced in the group that were fed the adsorbent-treated feeds, especially in the bentonite-treated feed.

**Conclusion:** Conclusively, the adsorbents were able to reduce the concentration of aflatoxin in the feed and this reflected positively on the general performance of the birds.

### P444. Evaluation of the growth of Sporothrix schenckii and Sporothrix brasiliensis when exposed to the iron element

ScrofernekerM.L.^1^HellwigA.H. Da Silva^2^AbreuH. Klein De^2^RibeiroA. Carvalho^1^OliveiraH. Tedesco De^1^PezziE. Heidrich^1^ZanetteR. Adriel^3^1Department of Microbiology, Immunology and Parasitology, Universidade Federal do Rio Grande do Sul, Porto Alegre, Brazil2Postgraduate Program In Medicine: Medical Sciences, Universidade Federal do Rio Grande do Sul, Porto Alegre, Brazil3Postgraduate Program In Biological Sciences: Pharmacology and Therapeutics, Universidade Federal do Rio Grande do Sul, Porto Alegre, Brazil

**Objectives:** The aim is to evaluate the viability of *Sporothrix* spp. when exposed to iron, using 11 isolates of *S*. *schenckii* and 10 isolates from *S*. *brasiliensis*.

**Methods:** All the samples used were previously analyzed by molecular methods. Conidia were collected from cultures with seven days of growth in potato dextrose agar medium and suspended in 0.85% saline solution. Each suspension was standardized in a Neubauer chamber, adjusted to the concentration of 10^6^ conidia / ml, and inoculated at the center of petri plates containing potato dextrose agar plus the following iron concentrations (FeCl_3_): 0.0625%; 0.125%; 0.25%. The isolates were also inoculated into plates containing potato dextrose agar pure or added with 1 mM ascorbic acid and 1 mM ferrozine to eliminate traces of iron present in the medium. Plates were incubated in triplicates at 35 ° C for 28 days to measure the mean diameter of colonies (mm) and perform analysis of variance followed by the Tukey post-hoc test in the GraphPad Prism statistical program, with significance p < 0.05.

**Results:** It has been found that high iron concentrations are toxic to *Sporothrix* spp., as seen in the concentration of 0.25% FeCl_3_, where there was no growth of the isolates. In addition, medium with absence of iron also had to lower growth of *Sporothrix* spp. when compared to the potato dextrose agar medium and the medium plus 0.0625% and 0.125% FeCl_3_.

**Conclusion:** It is possible to conclude that the viability of *Sporothrix schenckii* and *Sporothrix brasiliensis* is affected by iron, which is an important element for its biological activity.

### P445. The interaction between Sporothrix schenckii sensu sctricto and Sporothrix brasiliensis with Acanthamoeba castellanii

ScrofernekerM.L.^1^TavaresP. Lemos^2^BerteF. Kercher^1^RibeiroA. Carvalho^1^RottM. Brittes^1^1Department of Microbiology, Immunology and Parasitology, Universidade Federal do Rio Grande do Sul, Porto Alegre, Brazil2Postgraduate Program In Medicine: Medical Sciences, Universidade Federal do Rio Grande do Sul, Porto Alegre, Brazil

**Objectives:** The aim of our study was to verify the profile of interaction between *S. schenckii sensu stricto* and *S. brasiliensis* and *A. castellanii* by an *in vitro* model of coculture as a modulator factor of both microorganisms intrinsic characteristics.

**Methods:** For this purpose the phagocytosis index of *S. schenckii sensu stricto* and *S. brasiliensis* by *A. castellanii* was evaluated; the *S. schenckii sensu stricto and S. brasiliensis* viability after contact with *A. castellanii*; the amoeba viability after contact with the fungus; and the influence of *S. schenckii sensu stricto and S. brasiliensis* in encystment process of *A. castellanii* were performed. The analyses indicated that *S. schenckii* and *S. brasiliensis* have suffered phagocytic events by *A. castellanii* and this index is significantly higher for *S. schenckii* in the first two hours compared to *S. brasiliensis.*

**Results:** Our results showed a significant increase in conidia and hyphae count after 72h of coculture of S. *brasiliensis* and lysis of amoebae occurred after fungal internalization.

**Conclusion:** *S. schenckii sensu stricto* and *S. brasiliensis* probably used amoebae as nutritional source. Our results were obtained *in vitro* and may not demonstrate the same behavior *in vivo*, being required *in vivo* studies with co-infection in order to have a thorough understanding of this relationship.

### P446. Estimation of fungal risk in cystic fibrosis: lessons from the MucoFong prospective project

DelhaesL.^1^FrancisF.^2^EnaudR.^3^SoretP.^4^ConsortiaM.^5^BuiS.^3^FayonM.^3^ThiebautR.^2^1Parasitology Mycology, Univeristy of Bordeaux, Centre de Recherche Cardio-Thoracique de Bordeaux, U1045, F-33000, Bordeaux, France, Bordeaux, France2CHU Bordeaux, Department of Public Health, Bordeaux, France3University of Bordeaux, Centre de Recherche Cardio-Thoracique de Bordeaux, U1045, CHU de Bordeaux, CRCM Pédiatrique, CIC 1401, Bordeaux, France4University of Bordeaux, Inserm, Bordeaux Population Health Research Center, UMR 1219, INRIA SISTM Team, Bordeaux, France5The Mucofong Investigation Group, Bordeaux, France

**Objectives:** Beside the main objectives of the multicentric project "MucoFong" (PHRC-19021906) that evaluated the respiratory fungal composition in CF and provided the first French guidelines for mycological analysis of CF sputum, data collected during this two-year follow-up of 299 CF patients (3 visits per patient including biological, radiological, and clinical data) were analyzed to assess associations between the microorganisms identified in the sputum and the clinical evolution.

**Methods:** Relations between microorganisms identified in the sputum and clinical course of patients were longitudinally analyzed taking as output variable FEV1 (Forced Expiratory Volume in 1s expressed as percent of predicted value). As data were collected several times throughout the follow-up, a mixed-model analysis was performed to evaluate the effect of a sporadic or chronic colonization with bacteria and/or fungi at the inclusion on the evolution of lung function over two years. Univariate plus multivariate analysis were achieved. A sensitivity analysis was performed to identify interactions between microbes.

**Results:** *P. aeruginosa*, *A. xylosoxidans*, *S. maltophilia*, and *C. albicans* were associated to a more significant decrease in FEV1 during the two-year follow-up, adjusted on age, gender, hospital centre, BMI, and gastrooesophageal reflux disease. Unexpectedly, FEV1 evolution was lower of 11.26% in patients presenting an intermittent colonisation with non-pneumoniae *Streptococcus* than others.

**Conclusion:** To conclude, these results were in agreement with data recently published, and provided new insights into the bacterial but also the fungal colonization as a key factor in assessing and predicting lung function evolution in CF patients. The Mucofong Investigation Group: Marc Pihet, Emilie Fréalle, Yolande Lemeille, Claudine Pinel, Hervé Pelloux, Gilles Gargala, Loic Favennec, Isabelle Accoceberry, Isabelle Durand-Joly, Frédéric Dalle, Frédéric Huet, Annlyse Fanton, Amale Boldron, Guy-André Loeuille, Philippe Domblides, Bérengère Coltey, Isabelle Pin, Catherine Llerena, Françoise Troussier, Christine Person, Christophe Marguet, Nathalie Wizla, Caroline Thumerelle, Dominique Turck, Anne Prévotat, Benoit Wallaert, Sylvie Leroy, Jean-Philippe Bouchara Grant: Project MucoFng 2.0 (RF20160501626/1/1/275) from Vaincre la Mucoviscidose (VLM)

### P447. Evaluation of metabolic adaptations of Fonsecaea pedrosoi using tricyclazole and FTIR

ScrofernekerM.L.^1^KoehlerA.^2^CorbelliniV.^3^1Department of Microbiology, Immunology and Parasitology, Universidade Federal do Rio Grande do Sul, Porto Alegre, Brazil2Postgraduate Program In Medicine: Medical Sciences, Universidade Federal do Rio Grande do Sul, Porto Alegre, Brazil3Department of Chemistry and Physics, Universidade de Santa Cruz do Sul, Santa Cruz do Sul, Brazil

**Objectives:** Evaluating the metabolic adaptations of *Fonsecaea pedrosoi* when exposed to tricyclazole using Fourier Transform Infrared Spectroscopy (FTIR).

**Methods:** Twelve isolates of *F. pedrosoi* from the collection of the Pathogenic Fungi Laboratory, Department of Microbiology, ICBS, UFRGS, were cultivated for 14 days at 30ºC in Petri dishes with Sabouraud agar. For each isolate were used two plates with 4 µg/mL or 16 µg/mL of tricyclazole. The spectra were acquired through attenuated total reflection (ATR) using fragments of the cultures on dehydrated agar. Five spectra were recorded from 4000 to 650 cm^-1^ for each isolate. The data set was preprocessed and normalized by amplitude. The Pirouette® software was used to perform the Principal Component Analysis (PCA) and the Partial Least Squares Discriminant Analysis (PLS-DA) using the first derivative and an orthogonal signal correction. These analyzes were perfomed to verify if it was possible to separate the classes (growth with 4 or 16 µg/mL) through the spectra, showing if there are metabolic adaptations of *F. pedrosoi* when exposed to tricyclazole.

**Results:** The PCA didn’t allowed class differentiation due to irrelevante background information. With PLS-DA, using only one component, the minimum error of class prediction was about 0.001, with Bayes limit between classes close to 0.5 and 100% of accuracy in the identification of the cultures in 4 or 16 µg/mL of tricyclazole. The results show that the metabolic adpatations are reproducible, but discrete, and the regions of the spectra that most contributed to the differentiation were the bands of 989 cm^-1^ and 1086 cm^-1^ (sugars); 1508 cm^-1^ (proteins) and 1617 cm^-1^ (unsaturated carbon bonds, evidence of the formation of the 1,8-dihydroxynaphtalene (DHN) melanin precursors).

**Conclusion:** There are metabolic adaptations of *F. pedrosoi* with the exposition to tricyclazole. These adaptations can be evaluated through FTIR, which is relevant for the development of techniques to make correlations with models of virulence and pathogenicity of these fungi.

### P449. Bridging the knowledge gap on mycoses in Africa; setting up a Pan-African Mycology Working Group

OladeleR.^1^AkaseI.^2^FahalA.^3^GovenderN.^4^HoeniglM.^5^GangneuxJ.-P.^6^ChillerT.^7^CornelyO.A.^8^DenningD.^9^ChakrabartiA.^10^1Medical Microbiology & Parasitology, College of Medicine of University of Lagos, Lagos, Nigeria2Internal Medicine, Lagos University Teaching Hospital, Lagos, Nigeria3Mycetoma Research Centre, Kartoum, Sudan4National Institute for Communicable Diseases, Johannesburg, South Africa5Division of Infectious Diseases, Department of Medicine, UCSD, San Diego, United States of America, Austria, United States of America6Universite de Rennes, Rennes, France7Mycotic Division, Centre for Disease Control, Atlanta, United States of America8Department I of Internal Medicine, University Hospital of Cologne, Cologne, Germany, Cologne, Germany9Global Action Fund for Fungal Infections, Geneva, Switzerland10Medical Microbiology, Postgraduate Institute of Medical Education and Research, Chandigarh, India

**Objectives:** The African continent has an estimated population of 1.3 billion people accounting for roughly about a quarter of the world’s population and has an estimated 700,000 fungal related deaths in the setting of HIV infections. From the data generated by GAFFI; an estimated 47,643,919 Africans suffer from fungal diseases, of which 1,728,657 suffer from a serious fungal infection annually (GAFFI Factsheet). This is a course for concern considering the fact that this is data from only 15 (out of 57) African countries (Table 1). Almost all African countries lack a surveillance system for fungal infections; the only exception is South Africa. Interestingly, only South Africa has a reference mycology laboratory. Also, there is a pervasive picture of inadequate/poor diagnostic capacity, low level of awareness/ index of suspicion among health care workers and policymakers, unavailability, and non-accessibility to antifungal medications. Most African countries have poorly funded and overburdened health systems. Additionally, a high prevalence of HIV in Sub-Saharan Africa contributes to a high burden of opportunistic fungal infections. Recently, the African sub-region developed a network of mycology experts whose goal is to provide a common platform for a compressive discussion and collaboration on fungal infections.

**Methods:** Recent outreach efforts of ISHAM and also the European Confederation of Medical Mycology (ECMM) aimed to increase the involvement of African countries and experts in e.g. the “One World One Guideline” initiative and also the ECMM Academy. The Medical Mycology Society of Nigeria with support from ISHAM and ECMM held its first international conference on March 2019, which cumulated with the establishment of the PanAfrican Mycology Working Group (PAMWG).

**Results:** PAMWG will organize and engage African leaders in the field of mycology in the African sub-region linked to ISHAM. The aim is is to provide better interaction&synergy among regional leaders in order to develop educational programs for capacity building to aid in the diagnosis and care of patients with fungal infections. It will also encourage countries’ initiatives to develop clinical guidelines for the clinical management of fungal infections and support the need for the establishment of reference mycology laboratories. Objectives The ISHAM-PAMWG aims to provide a platform for all the leaders in the field on mycology from different African countries in order to initiate epidemiological studies, regional guidelines, and educational programs to increase the capacity/ expertise of African specialists in the early detection and treatment of fungal infections. These activities will be achieved via the following: Education and training programs for health care workers involved in the care and evaluation of patients with fungal infections. South-south, and north-south collaborations and partnership. Organization of regional collaborative studies. Participation in the ECMM One World One Guideline program. Encourage all PAWG members to affiliate with ISHAM Organize local meetings in African countries in collaboration with ISHAM and ECMM Participation of leading centers in Africa in the ECMM Excellence Center program To advocate the recognition of neglected fungal diseases in Africa.

**Conclusion:** Meeting the objectives listed above will generate the needed data for advocacy needed to drive government commitment to allocate the resources required.

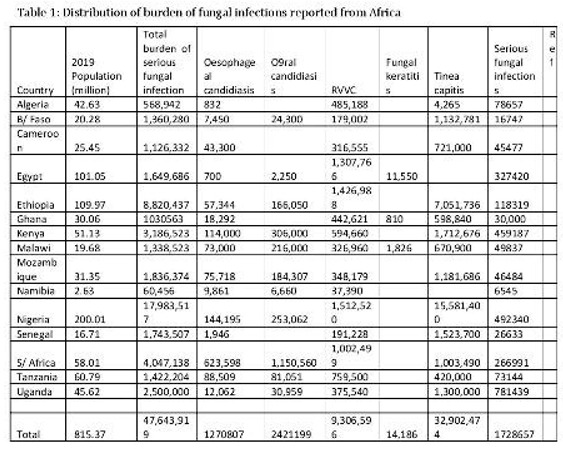

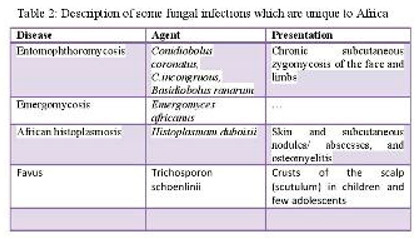

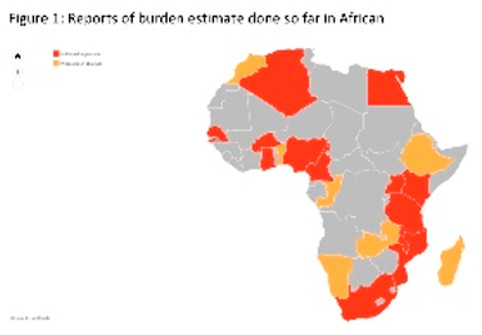







### P450. rImportance of Sphingolipid Biosynthetic Pathway for Growth, Biofilm Formation and Membrane Integrity of the Pathogenic Fungus Scedosporium boydii

PinheiroR. Rollin^1^RochettiV. Pereira^1^XistoM.I.D.S.^1^BastosB.^2^RellaA.^3^RozentalS.^2^PoetaM. Del^3^Barreto-BergterE.^1^1Instituto De Microbiologia Paulo De Góes, Universidade Federal do Rio de Janeiro, Rio de Janeiro, Brazil2Instituto De Biofísica Carlos Chagas, Universidade Federal do Rio de Janeiro, Rio de Janeiro, Brazil3Department of Molecular Genetics and Microbiology, Stony Brook University, Stony Brook/New York, United States of America

**Objectives:** *Scedosporium boydii* is a worldwide emerging pathogen able to cause a wide-spectrum infection ranging from mycetoma to invasive infections in immunocompromised patients. It is also considered the second more frequent pathogenic fungus associated to cystic fibrosis patients. Sphingolipids are abundant components of membranes in fungal cells, playing a variety of roles such as heat stress response, signal transduction, hyphal growth, endocytosis and apoptosis. Particularly, glucosylceramides (GlcCer) have been studying by our group during the last decades in *Scedosporium/Lomentospora* complex, being associated to fungal growth and pathogenesis. This study aims to evaluate the sphingolipid synthesis during the germination process, as well as the importance of different sphingolipids for fungal growth, membrane integrity using some inhibitors of the biosynthetic pathway.

**Methods:** *S. boydii* conidia were treated with Myriocin, a serine palmitoyltransferase inhibitor, or PPMP (1-phenyl-2-palmitoylamino-3-morpholino-1-propanol), a glucosylceramide synthase inhibitor, and the results were compared to the non-treated control. Sphingolipid synthesis was evaluated by mass spectrometry of lipids extracted by fungal cells treated or not with the inhibitors. The effect of the inhibitors on fungal growth was analyzed by germination assay, colony forming units (CFU) quantification and biofilm formation. Filipin staining, checked by fluorescence microscopy, was performed to evaluate the accumulation of membrane microdomains on hyphal tips. Membrane integrity was analyzed by transmission electron microscopy (TEM) and fungal susceptibility to membrane stressors, such as SDS and NaCl. Susceptibility to antifungal agents in the presence of Myriocin and PPMP was evaluated through the checkerboard method.

**Results:** *S. boydii* treated with Myriocin and PPMP displayed decreased sphingolipid production and showed a reduced growth, forming less CFU and biofilms compared to the control. Additionally, the germination process was impaired, since treated cells formed short germ tubes compared to mature hyphae in the control. It was confirmed with filipin staining, in which lipid raft accumulation in hyphal tips, essential for hyphae elongation, was not observed in treated cells. Analysis by TEM revealed the presence of regions with loss of membrane integrity, what was confirmed through the increase in membrane susceptibility to SDS and NaCl in treated cells. Myriocin-treated cells became more susceptible to different classes of antifungal drugs, suggesting that sphingolipids on the membrane could interfere in drug susceptibility.

**Conclusion:** Sphingolipids have been considering a potent target for the develop of new antifungal drugs. Thus, the study of these molecules in pathogenic fungal becomes a useful approach in medical mycology. The present data highlight the participation of sphingolipids in different fungal cell events that are crucial for fungal growth, virulence and drug resistance. Hyphae elongation and biofilm formation are important processes for mold proliferation and host tissue invasion. In addition, membrane integrity, especially regarding lipid raft areas, is essential for signal transduction, stress resistance and a variety of other physiological processes in fungal cells. For these reasons, the use of sphingolipid inhibitors helped to show how these molecules are promising targets for new drugs against *Scedosporium/Lomentospora* infections.

### P451. Studying the unknown: implementation of two recognized protocols to characterize lipid droplets in Malassezia pachydermatis

MantillaM.^1^ArizaG.^1^CabreraC.^2^RestrepoS.^1^RamírezA.M. Celis^1^1Biological Sciences, Universidad de los Andes, Bogotá D.C., Colombia2Chemistry, Universidad de los Andes, Bogotá D.C., Colombia

**Objectives:** *Malassezia pachydermatis* is a lipid-dependent yeast that is part of animal skin mycobiota and is associated with otitis externa and dermatitis in animals, as well as fungemia in humans. The lipid metabolism of this yeast is of high relevance because it may play an essential role during pathogenesis; however, these processes are yet to be well understood. Lipid droplets (LD) have become a subject of study in *Malassezia,* as they are known to store lipids and proteins in other biological models such as *Mycobacteria,* Hepatitis B virus, *Saccharomyces cerevisiae* and, mammal eukaryotic cells. Therefore, this study aims to standardize for the first time two protocols for the analysis of LD in *Malassezia pachydermatis*: The first one to characterize their structure by observation under a laser scanning confocal fluorescence microscopy; and the second one for the extraction and purification of LD for future lipidomic analysis.

**Methods:** Here we standardized two protocols to describe LD: the first one to characterize the structure is based on co-staining yeast cells and LDs with different fluorochromes: Nile Red (Sigma-Aldrich), LipidTox red neutral (Invitrogen) and BODIPY 493/503 (Invitrogen), in combination with calcofluor white (Sigma-Aldrich). The first three fluorochromes bind to neutral lipids present in the LD and calcofluor binds to the cellulose and chitin in the fungal cell wall. The second protocol to extract and purify LD induces yeast cell lysis by a combination of chemical and physical techniques, followed by the modification of the density of the solution with different buffers to allow the extraction and purification of LD.

**Results:** We standardized a protocol to optimize LD fluorescent staining to characterize their structure under confocal microscopy. We found that the optimal steps are as follows: 1) Preparation of the staining solution with a mixture of 4% formaldehyde and the three neutral lipid fluorophores. 2) Incubation at room temperature of LipidTox and Nile Red for 2 hours, and BODIPY for 30 minutes. 3) Double washing with PBS, resuspension in 4% formaldehyde and mounting on microscope slides. 4) Addition of calcofluor white, and 0.01% and 10%(v/v) KOH to the slide. We also optimized the protocol to get the of rupture *M. pachydermatis* cells for the extraction and purification of LDs. We found that the optimal conditions are: 1) Enzymatic digestion with *Trichoderma harzianum* enzymes (Sigma-Aldrich) for 2 hours at 47ºC. 2) Mechanical disruption by adding 0.5 mm zirconium pearls to the sample and ten cycles of 1 minute of a vortex and 30 seconds on ice. In addition, cell wall removal by stroking the sample 10 times with a Dounce homogenizer. 3) Finally, several ultracentrifugation cycles in buffers containing Ficoll 400 (Sigma-Aldrich) to separate the LDs in the supernatant from the organelles that stay in the pellet.

**Conclusion:** Improving and standardizing these protocols can contribute to the study of the morphology, structure and, in the future, the lipid composition of LD in *Malassezia pachydermatis.* This study also could disclose clues about the lipid metabolism field in this yeast.

### P452. Characterization of the local inflammatory reaction after inhalative infection of immunocompromised mice with the mucormycete Rhizopus arrhizus

SpethC.^1^,^2^MarcabruniJ.^1^ParthN.^1^KociF.^1^Lass-FlörlC.^2^MaierH.^3^RambachG.^4^1Div Hygiene & Med. Microbiology, Med. Univ. Innsbruck, Innsbruck, Austria2Christian Doppler Laboratory for Invasive Fungal Infections, Innsbruck, Austria3Institute of Pathology, INNPATH, Innsbruck, Austria4Div Hygiene & Med. Microbiology, Med. Univ. Innsbruck, Medical University of Innsbruck, Austria

**Objectives:** The mucormycete species *Rhizopus arrhizus* mainly enters the body of immunosuppressed patients via inhalation of the spores. The local pathogenesis of inflammatory processes in the lung is poorly understood. For that reason we investigated the inflammatory reaction and the antifungal immune reaction in the lung of *Rhizopus*-infected mice.

**Methods:** Mice treated with cortisone or cyclophosphamide were intravenously infected with a high dose of *Rhizopus arrhizus* spores. For comparison mice with selective deficiency in neutrophils, alveolar macrophages or complement were used. Lung parameters were investigated by histology and by isolation of bronchoalveolar lavage (BAL). Immune processes were quantified in blood samples taken from the animals.

**Results:** Lack of neutrophils as well as treatment with cyclophosphamide were the most prominent risk factors for a lethal outcome after *Rhizopus* infection; in contrast, depletion of alveolar macrophages or cortisone treatment represented only moderate risk factors. Interestingly, complement deficiency even ameliorated the outcome, presumably due to a reduced inflammatory process. Lack of macroscopic organ abnormalities beside the lung indicated that fungal infection was mainly limited to the lung. BAL analyses revealed clear signs of excessive inflammation such as presence of erythrocytes, massive infiltration of granulocytes and high levels of the marker haptoglobin. Furthermore, macrophages in the lung were significantly augmented and these immune cells were highly activated. Most spores found in BAL were bound to or phagocytosed by neutrophils and macrophages and only few fungal hyphae were visible.

**Conclusion:** Inflammatory processes in the lung after inhalation of *Rhizopus arrhizus* are the main reason for fatal outcome; fungal growth and/or dissemination plays a minor role.

### P453. Pathogenesis and amphotericin B treatment of Lichtheimia corymbifera infection in a murine model

RambachG.^1^,^2^MarcabruniJ.^1^ParthN.^1^Lass-FlörlC.^1^,^2^SpethC.^1^,^2^1Div Hygiene & Med. Microbiology, Med. Univ. Innsbruck, Innsbruck, Austria2Christian Doppler Laboratory for Invasive Fungal Infections, Innsbruck, Austria

**Objectives:** Immunocompromised individuals are the main risk group for invasive mucormycosis, but the relevance of the precise nature of immunodeficiency is insufficiently investigated. Furthermore, only limited information exists about differences in the efficiency of amphotericin B (AmB) therapy in various patient groups. For that purpose we aimed to study pathogenesis and AmB treatment of inhalative *Lichtheimia corymbifera* infection and to compare the susceptibility of pancytopenic mice versus mice with selective defects in innate immunity.

**Methods:** Mice treated with cyclophosphamide or cortisone or selectively deficient in neutrophils, alveolar macrophages or complement, were inhalatively infected with *Lichtheimia corymbifera.* Half of the animals received therapy with liposomal AmB. Survival, clinical status and immunological processes were monitored.

**Results:** Immunosuppression of the animals with cyclophosphamide represented the most relevant risk factor for fatal outcome of *Lichtheimia* infection, followed by treatment with cortisone. Although neutropenia is assumed to be the correlate for susceptibility against fungal infection, selective depletion of neutrophils predisposed only moderately for severe disease and mortality. Interestingly, neither complement deficiency nor depletion of alveolar macrophages represented major risk factors for the animals. Various clinical parameters such as body temperature and weight loss as well as immunological parameters confirmed these results. For those animals dying from invasive *Lichtheimia* infection, neurological symptoms were the second most reason for scarifying the mice. Treatment with liposomal AmB successfully improved the outcome in the animal groups with neutropenia, cortisone acetate and cyclophosphamide with the most prominent effect in the cortisone group.

**Conclusion:** Affectation of several immune cell types is necessary to make the animals susceptible for invasive mucormycosis by *Lichtheimia corymbifera*, whereas selective loss of neutrophils or alveolar macrophages have only minor effects.

### P454. The role of Aspergillus fumigatus fumagillin in fungal virulence.

GuruceagaX.^1^Perez-CuestaU.^1^PellonA.^2^UribeB.^3^GonzalezO.^3^KellerN. P.^4^HernandoF.L.^1^AnguitaJ.^2^AlonsoR.M.^3^Ramirez-GarciaA.^1^RementeriaA.^1^1Immunology, Microbiology and Parasitology, University of the Basque Country (UPV/EHU), Leioa, Spain2Inflammation and Macrophage Plasticity Laboratory, CIC bioGUNE, Derio, Spain3Department of Analytical Chemistry, University of the Basque Country (UPV/EHU), Leioa, Spain4Department of Medical Microbiology and Immunology, University of Wisconsin, Madison, United States of America

**Objectives:** *Aspergillus fumigatus* is a saprophytic fungus capable to cause pulmonary infections in immunosuppressed patients. We have previously demonstrated an overexpression of 67% of the genes involved in the fumagillin metabolic pathway during experimental infection of immunosuppressed mice. The aim of this work was to demonstrate the implication of this molecule in fungal pathogenesis.

**Methods:** We performed a set of *in vitro* experiments using commercial toxin preparations to first standardize the UHPLC-PDA analytical method and then identify its toxic effects on macrophages (RAW 264.7), melanome (B16-F10), and the lung epithelial (A549) cell lines. Moreover, we carried out fungus-host cell co-incubation assays using murine primary bone marrow derived macrophages (BMMs). Finally, *in vivo* assays were performed by intranasal infection of immunosuppressed Swiss mice treated with 100 mg/kg of cyclophosphamide intraperitoneally every 72 h, starting four days before the infection. The mice, in group on 10, were infected with, the non-fumagillin producer mutant strain, *ΔfumR,* as well as the wild type (WT), *ΔakuB^ku80^*, and the complemented strains. All the experiments were repeated at least thrice.

**Results:** The UHPLC-PDA method assay showed that *A. fumigatus* WT strain is able to produce the toxin at a 1.31 μg/ml growing in RPMI during 24 h at 37°C. Furthermore, when we incubated the cell lines in the presence of 1 μg/ml of commercial toxin for 24 h, eighty percent of the toxin disappeared from the media of A549 cultures, 58% in the case of B16-F10 melanome and only 4% in cultures with RAW 264.7 cells. However, the viability of the A549 cells was not significantly affected, while B16-F10 and RAW264.7 cells showed a significant reduction in their viability compared to. untreated cells. Assays *in vitro* showed that the null strain, *ΔfumR,* was significantly more susceptible to phagocytosis by the BMMs than the complemented or WT control strains. On the other hand, no differences in germination were observed during the co-incubation with BMMs, but *ΔfumR* showed a significant increase in the percentage of double branches conidia compared to the other two strains. Indeed around 25% of the germinated *ΔfumR* conidia showed two branches after 8 hours of co-incubation. The *in vivo* study did not show any significant differences in mice survival between the groups infected with the *ΔfumR* and the WT strains. In contrast, mice infected with the complemented strain showed a higher mortality rate, maybe due to the increase secretion of fumagillin by complemented strain, as we showed in the *in vitro* assays.

**Conclusion:** This study shows that *A. fumigatus* is able to secrete the mycotoxin fumagillin, a toxin that penetrates inside the cells causing cell death. Furthermore, this toxin could be a fungal defense factor against phagocytosis and may play an important role during *A. fumigatus* pulmonary infection. **Acknowledgement.** This study was funded by a grant of UPV/EHU (PPG17/41). Pre-doctoral grants of the Basque Government and UPV/EHU have supported XG, and BU and UPC, respectively.

### P456. Establishment of an experimental protocol of chronic tobacco exposure to study its effects on a mice model of pulmonary paracoccidioidomycosis

BuccheriR.^1^,^2^NetoA. Nunes Duarte^1^SilvaF.L. Batista Da^2^CarvalhoG.O. Marques Haddad^2^SilvaL. Buffoni Roque Da^3^NetoR. Soares De Azevedo^1^TabordaC.P.^3^BenardG.^4^1Department of Pathology, Medical School, University of São Paulo, São Paulo, Brazil2Laboratório De Investigação Médica Lim53, Insituto de Medicina Tropical, São Paulo, Brazil3Laboratório De Fungos Dimórficos Patogêncios, Biomedical Sciences Institute, University of São Paulo, São Paulo, Brazil4Laboratório De Investigação Médica Lim53, Instituto de Medicina Tropical/Faculdade de Medicina da Universidade de São Paulo, São Paulo, Brazil

**Objectives:** Paracoccidioidomycosis (PCM) is caused by *Paracoccidioides spp*, and the most important systemic mycosis in non-immunosuppressed patents in Latin America. It has two major clinical presentations, the acute/subacute, more rare (≤5% of the cases) form (AF), and the more prevalent, chronic form (CF). In the AF, the phagocytic-mononuclear system is predominantly affected, while the lung is the target organ in the CF. The AF in general develops some time (weeks to months) after exposure to *Paracoccidioides* spp. and the CF develops from reactivated quiescent foci many years or decades after the initial exposure. However, we still do not know the factors that trigger the reactivation of the fungus and the development of the CF. of note is the observation that most (>90%) CF patients refer tobacco smoking. It is well known that chronic tobacco exposure leads to imbalance of the pulmonary immune responses, and has a role in the development of other chronic pulmonary infections such as tuberculosis. We therefore aimed to develop a protocol of chronic tobacco exposure to study its effects on an experimental mice model of pulmonary PCM. We present here the preliminary results of this protocol.

**Methods:** C57BL/6 animals were exposed to cigarette smoke for 120 minutes (equivalent to ~16 cigarettes) twice/day, 5 days/week, for a total of 20 weeks, using a previously described device [Biselli PJC et al. Braz J Med Biol Res. 2011;44:460-468]. Three groups of animals were studied: an infected/exposed group, inoculated intra-tracheally with 10^2^ *P. brasiliensis* yeast cells 2 weeks prior to initiation of tobacco exposure, an infected only group, and an exposed only group. The tobacco exposure protocol was adjusted to result in carboxyhemoglobin (COHb) levels of 10-15% after the daily first exposure, and 15-20% after the second exposure, to simulate the levels observed in chronic moderate smokers [Stewart RD et al. JAMA. 1974;26:1187-95]. Animals were sacrificed at weeks 4, 12 and 20 and their lungs examined for colony forming units, area involved by the granulomatous inflammation and histological assessment of the effects of tobacco exposure.

**Results:** The tobacco exposure protocol was successful in mimicking the effect of chronic tobacco smoking in humans: a) the mean level of COHb after the first tobacco exposure (measured monthly) was 15,5% (range 10,9-16,1%), and of 20,5% (range 15,9-21,1%) after the second exposure, while in the no-exposed infected group it was 0,1% (range 0-0,3%); b) By week 20 there were moderate dysplasia of the columnar epithelium, increased mucous production, alveolar septum destruction and focal areas of bronchiolitis. Preliminary data showed that tobacco exposure increased both the CFU and the area involved by the granulomatous inflammation as shown below. 

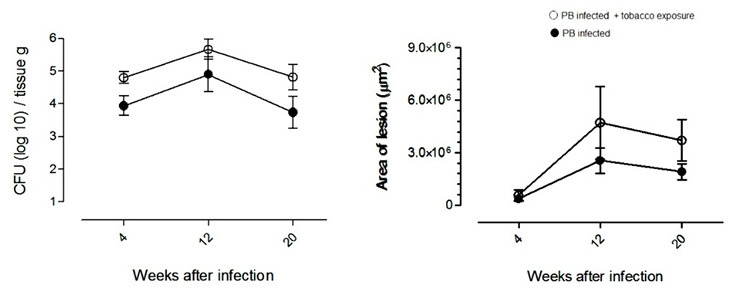



**Conclusion:** These preliminary results indicate that: 1) We were able to establish a protocol of tobacco exposure mimicking that observed in patients with chronic form paracoccidioidomycosis. 2) Tobacco exposure impacts the pulmonary disease in a mouse model of PCM. Financial support: Fapesp #2016/08730-6 and #2017/12374-3. CPT and GB receive a senior researcher fellowship from CNPq

### P457. Phenotypic characterization of Aspergillus fumigatus maiA deficient strain.

GuruceagaX.^1^Perez-CuestaU.^1^Martin-VicenteA.^2^SouzaA.C.O.^2^NyweningA.V.^2^AbdallahQ. Al^2^GeW.^2^HernandoF.L.^1^FortwendelJ.^2^Ramirez-GarciaA.^1^RementeriaA.^1^1Immunology, Microbiology and Parasitology, University of the Basque Country (UPV/EHU), Leioa, Spain2Department of Clinical Pharmacy and Translational Science, University of Tennessee Health Science Center (UTHSC), Memphis, United States of America

**Objectives:** Invasive aspergillosis is the most dangerous disease produced by *Aspergillus fumigatus*. To identify genes important for host-pathogen interactions, we analyzed fungal gene expression in contact with macrophages and lung epithelium cell lines, as well as during an *in vivo* infection in immunosuppressed mice and detected multiple genes overexpressed in all the situations. One of these genes, *maiA*, is involved in the Tyrosine metabolic pathway and could be an important gene for energy production during host-pathogen interactions. For that, the aim of this work was to characterize a mutant strain deficient in *maiA* gene.

**Methods:** The loss of function mutant strain was built by CRISPR-Cas9 technology using the *A. fumigatus* genetic background Af-293. The transformation method was carried out using a standard protocol. For the phenotypic spot characterization assays, we used Glucose minimal medium (GMM) agar plates supplemented with Congo red (80 µg/ml), Calcofluor white (80 µg/ml), Voriconazole (0,5 µg/ml), Caspofungin (4 µg/ml) or Anidulafungin (4 µg/ml) and minimal medium supplemented with L-tyrosine (10 mM) or Phenylalanine (10 mM) as the sole carbon source. Antifungal susceptibility tests using Voriconazole, Itraconazole, Isavuconazole, Posaconazole, Caspofungin and Anidulafungin were carried out following the EUCAST method. Finally, glucan quantification, following the Aniline blue assay, and pyomelanin quantification, using GMM broth and GMM broth supplemented with L-Tyrosine (10mM) or Phenylalanine (10mM), were also performed. All the assays were performed in triplicate.

**Results:** The CRISPR-Cas9 technique provided a gene-targeting efficiency of 60% (3 out of 5 colonies) using the *A. fumigatus* genetic background Af-293. After spot dilution assays analysis, differences in the fungal growth between the wild type and the mutant strain were not found in presence of Calcofluor white, Voriconazole, Caspofungin or Anidulafungin. In contrast, Δ*maiA* was hypersusceptible to Congo red despite a lack of significantly different amounts of glucan per milligram of mycelia. Furthermore, no significant differences were observed in the MIC or MEC for the triazoles or the echinocandins, respectively. However, Δ*maiA* was unable to use either L-tyrosine or Phenylalanine as the sole carbon source and pyomelanin production by the mutant strain was significantly higher than the wild type when GMM broth was supplemented with L-tyrosine or Phenylalanine. In GMM broth non-supplemented, both the wild type and Δ*maiA* showed the same pyomelanin production pattern.

**Conclusion:** CRISPR-Cas9 is an accurate technique that allowed us to generate the mutant strain Δ*maiA* using the *A. fumigatus* genetic background Af-293 with a great efficiency. This mutant was not able to grow in presence of Congo red or to use L-Tyrosine or Phenylalanine as a sole carbon source. Pyomelanin production was higher for Δ*maiA* in the presence of both amino acids, which is consistent with results published using other mutants of the Tyrosine degradation pathway. Future studies will explore in deep the role of *maiA* in animal models of IA. **Acknowledgement.** This study was funded by a grant of UPV/EHU (PPG17/41). EMBO Short-Term Fellowships and pre-doctoral grants of the UPV/EHU have supported XG and UPC respectively.

### P458. Scedosporium apiospermum sidD gene is responsible for the biosynthesis of the hydroxamate-type siderophore Nα-methylcoprogen B, and is essential for fungal growth

GovicY. Le^1^,^2^HavlicekV.^3^PaponN.^1^GalS. Le^1^BoucharaJ.-P.^1^,^4^VandeputteP.^1^,^4^1Host-Pathogen Interaction Study Group, SFR ICAT 4208, Univ Angers, Univ Brest, Institute of Biology in Health, IRIS, University Hospital Center, Angers, France, Angers Cedex, France2Laboratory of Parasitology-Mycology, University Hospital Center, Fort-de-France, Martinique, Fort-de-France, Martinique3Institute of Microbiology, Academy of Science, Prague, Czech Republic4Laboratory of Parasitology-Mycology, University Hospital Center, Angers, France, Angers, France

**Objectives:** *Scedosporium* species are filamentous fungi which commonly colonize the airways of patients with cystic fibrosis (CF). Nevertheless, there is only scarce information on their virulence mechanisms. Iron acquisition is crucial for the survival of nearly all microorganisms and is implicated in virulence of numerous pathogens that chronically inhabit the CF lung. Several genes relevant for siderophore-mediated iron uptake were previously identified in *Scedosporium apiospermum* genome, notably an orthologue of *sidD*, a non-ribosomal peptide synthetase (NRPS) gene that was demonstrated to drive the production of the extracellular hydroxamate-type siderophore fusarinine C (FsC) in *Aspergillus fumigatus*. Here, we aimed to determine the chemical structure of the siderophore produced by *S. apiospermum sidD* gene and to assess its role in fungal growth.

**Methods:** First, *sidD* gene was disrupted in a wild-type (WT) strain after previous disruption of the gene *ku70* encoding one of the components of the DNA repair system. Next, siderophore analysis was performed on lyophilized filtrates from WT and *ΔsidD* strains grown in minimal Yeast Nitrogen Base (YNB) medium for 48 h at 37°C. WT strain was cultured in YNB supplemented or not with 100 µM bathophenantroline disulfonate (BPS), while the mutants (*n* = 3) were grown in YNB without BPS only. The impact of *sidD* deletion was then studied by cultivating the fungus on yeast extract-peptone-dextrose-agar (YPDA) plates containing BPS and supplemented with culture filtrates from either WT or mutant strains. Finally, the ability of *S. apiospermum* to assimilate iron from xenosiderophores was assessed by using iron-saturated ferrichrome, ferrioxamine or pyoverdine (20 µM each) as sole source of iron.

**Results:** Analysis of the extracellular siderophores by high-resolution mass spectrometry performed on culture filtrate from WT strain yielded a molecular mass of *m/z* (M-2H + Fe)^+^ = 794.314 for the iron-saturated siderophore, perfectly matching N^α^-methylcoprogen B. This compound was absent in all *ΔsidD* mutant strains, indicating that *sidD* gene is essential for its synthesis. Other siderophores like FsC or TAFC were not found in any sample. Moreover, the disruption of *sidD* resulted in the lack of growth under iron-restricted conditions, and growth could be restored by supplementation of the culture medium (YPDA+BPS) with a culture filtrate from the WT strain. Conversely, incorporation of lyophilized filtrates from mutant strains in the culture medium did not rescue growth, corroborating the biochemical analyses. Interestingly, the use of iron-saturated ferrichrome or ferrioxamine as sole source of iron permitted a normal growth of all strains, indicating their direct assimilation by the fungus independently of siderophore production. Conversely, pyoverdine supported growth of the WT strain only, suggesting that N-methylcoprogen B is important for iron acquisition from pyoverdine.

**Conclusion:** Our results revealed that the *S. apiospermum sidD* gene drives the synthesis of a unique extracellular siderophore, *N*^α^-methylcoprogen B, that is essential for fungal growth. This compound seems important for iron acquisition from pyoverdine, which might explain why *S. apiospermum* and *Pseudomonas aeruginosa* are rarely found concomitantly in the CF lungs. Works are in progress to determine the contribution of *sidD* in the virulence of *S. apiospermum*.

### P459. Effect of Candida albicans on the malignant characteristic of tumor cells

Aparicio-FernandezL.AntoranA.Martin-SoutoL.BuldainI.AreitioM.RementeriaA.HernandoF.L.Ramirez-GarciaA.Immunology, Microbiology and Parasitology, University of the Basque Country (UPV/EHU), Leioa, Spain

**Objectives:** *Candida albicans* is an opportunistic pathogenic fungus usually present in different areas of the human body such as mouth and gut. Under certain circumstances, this yeast is able to reach the bloodstream causing disseminated candidiasis. Therefore, *C. albicans* is in close relationship with human cells, being in constant contact with them. This microorganism has been related to cancer and metastasis through different mechanisms such as carcinogenic product synthesis and induction of pro-metastatic microenvironment. Among the types of cancer studied in relation to *C. albicans* are oral malignancies and melanoma metastasis. Hence, this work aims to determinate whether the exposure to *C. albicans* is able to change tumor cells phenotype, increasing their aggressiveness.

**Methods:** Three tumor cell lines were used for this study: murine melanoma cells (B16M), human oral squamous cell carcinoma (H357) and murine colon carcinoma cells (C26). First, an adhesion assay was performed to test the effect of *Candida albicans* on tumor cells. Then, cancer cell lines were stimulated with antibiotic-inactivated fungus and conditioned media of the fungus, and wound healing assay was performed to measure migration activity of tumor cells. Afterwards, RNA was extracted from tumor cells to examine the expression of cancer-related genes by RT-qPCR and the supernatant was collected to measure the level of pro-inflamatory and cancer-related cytokines by ELISA.

**Results:** First, we observed that *C. albicans* was able to adhere to the three tumor cell lines. Then, wound healing assays with both antibiotic-inactivated *C. albicans* and conditioned media of *C. albicans* showed an increase in migration capacity of the different cell lines in comparison with the control, which indicates that the exposure to this fungus can induce a phenotypical change in tumor cells. The pro-tumor microenvironment was also altered, being the expression of different studied cytokines and cancer-related genes increased in presence of the fungus.

**Conclusion:** These results suggest that exposure to *C. albicans* induce alteration or changes in tumor cells and the related microenvironment that can lead to an increased malignancy. *Acknowledgement: This study was funded by a grant of UPV/EHU (PPG17/41). Pre-doctoral Grants of Basque Government and UPV/EHU have supported LMS and LAF respectively.*

### P465. Refining the therapeutic range of posaconazole and isavuconazole for efficient therapeutic drug monitoring using a bioassay approach

WillemanT.^1^ToniniJ.^2^CécileG.^3^BaillyS.^4^GandiaP.^5^Stanke-LabesqueF.^4^MaubonD.^6^Gautier-VeyretE.^4^1Laboratoire de Pharmacologie, Pharmacogénétique et Toxicologie, CHU Grenoble Alpes, France, GRENOBLE Cedex, France2Laboratoire de Pharmacologie, Pharmacogénétique et Toxicologie, CHU Grenoble Alpes, France, GRENOBLE Cedex, France3Univ. Grenoble Alpes, CNRS, CHU Grenoble Alpes, Grenoble INP*, TIMC-IMAG, 38000 Grenoble, France * Institute of Engineering Univ. Grenoble Alpes, GRENOBLE, France4Univ. Grenoble Alpes, Inserm, CHU Grenoble Alpes, HP2, 38000 Grenoble, France, GRENOBLE, France5UMR1436-INTHERES, 31076 Toulouse, France, Toulouse, France6Univ. Grenoble Alpes, CNRS, CHU Grenoble Alpes, Grenoble INP*, TIMC-IMAG, 38000 Grenoble, France * Institute of Engineering Univ. Grenoble Alpes, GRENOBLE Cedex, France

**Objectives:** Therapeutic drug monitoring for posaconazole (POS) tablet and isavuconazole (ISA) is still under debate and target trough concentrations (C_min_) need to be specified. We aimed to investigate the pharmacokinetic-pharmacodynamic relationship of POS and ISA, using a bioassay approach as surrogate marker of antifungal activity, in order to refine the therapeutic C_min_ of both antifungals.

**Methods:** A bioassay using a cellulose disk diffusion method was performed to determine the growth inhibition zone (GIZ) of POS and ISA on *A.fumigatus* (POS and ISA) and *C. parapsilosis* (ISA only). GIZs of plasma from patients undergoing routine TDM for POS (n = 136) or ISA (n = 40) were determined.

**Results:** GIZs of plasma patients and antifungal C_min_ were highly correlated both for ISA (*A fumigatus*: rho = 0.942, p<0.0001; *C. parapsilosis*: rho = 0.949, p<0.0001) and POS (rho = 0.922, p<0.0001), and these relationships were represented with a Michaelis-Menten model. Based on this modeling, the recommended thresholds of 0.7, 1, and 1.25 mg/L for the POS C_min_ corresponded to 50.1, 55.2, and 59.1% of the maximal GIZ, respectively. We propose an upper threshold of 4.8 mg/L for the POS C_min_ and a lower threshold of 2.0 mg/L for the C_min_ of ISA, as they respectively corresponded to concentrations leading to 90 % and 50 % of the maximal GIZ on *A.fumigatus*.

**Conclusion:** The determination of antifungal activity using this bioassay allowed refining target C_min_ for both POS and ISA, especially the upper threshold of POS (4.8 mg/L) and the lower threshold of ISA (2.0 mg/L).

### P466. Prospective evaluation of a combined genetic score for the individualization of voriconazole dose

Gautier-VeyretE.^1^Thiebaut-BertrandA.^2^RoustitM.^1^FonroseX.^3^BolcatoL.^4^DepeissesJ.^2^LefebvreC.^5^MassonD.^6^SchummerG.^7^CahnJ.-Y.^8^Stanke-LabesqueF.^1^1Univ. Grenoble Alpes, Inserm, CHU Grenoble Alpes, HP2, 38000 Grenoble, France, GRENOBLE, France2Hématologie Clinique CHU Grenoble Alpes, Grenoble, France3Chu Grenoble Alpes, Laboratoire de Pharmacologie, Pharmacogénétique et Toxicologie, GRENOBLE, France4Laboratoire de Pharmacologie, Pharmacogénétique et Toxicologie, GRENOBLE, France5Laboratoire d’Hématologie, CHU Grenoble Alpes, Grenoble, France6Etablissement Français du Sang, La Tronche, France7Centre de ressources biologiques, CHU Grenoble Alpes, GRENOBLE Cedex, France8Univ. Grenoble Alpes, CHU Grenoble Alpes, TIMC-Therex, Grenoble, France

**Objectives:** Voriconazole (VRC) exhibits a highly variable pharmacokinetics which is partly related to genetic polymorphisms affecting activities of cytochromes (CYP) 2C19 and 3A. This prospective observational study aimed to evaluate the impact of a previously described genetic score combining CYP2C19 and CYP3A genotypes (*Gautier-Veyret, AAC 2015*) to predict infratherapeutic plasma VRC trough concentration (Cmin) (defined as VRC Cmin < 1mg/L) in order to individualize VRC dose.

**Methods:** CYP2C19, CYP3A4 and CYP3A5 genotypes were determined in hematological patients treated by VRC and who benefited from longitudinal therapeutic drug monitoring in Grenoble University Hospital between January 2016 and December 2018. The genetic score was determined for each patient and patients were classified according to their genetic score ≤ or > 2. The higher the genetic score, the faster the metabolism of the patient. Statistical analyzes were performed on VRC Cmin adjusted on dose (VRC Cmin/D), considering either the initial VRC Cmin/D or all VRC Cmin.

**Results:** Forty-three patients (43 % of female, median age [Q1-Q3]: 57 [45-67] years) were included and 127 VRC Cmin analyzed. Ninety five percent of patients (41/43) received VRC for curative treatment of invasive aspergillosis (22 possible, 17 probable and 2 proven). Median initial VRC Cmin was 2.2 [1.1-4.3] mg/L and 21 % of patients (n = 9/43) had infratherapeutic initial VRC Cmin. Sixty-three percent of patients had at least one genetic variant for CYP2C19, 3A4 or 3A5, with 8 patients with a genetic score <2. Among 9 patients with infratherapeutic initial VRC Cmin, 8 (89%) had a genetic score ≥ 2. In univariate analysis, initial VRC Cmin/D, as well as all VRC Cmin/D was not influenced by genetic score, but significantly associated with advanced age, increased CRP and bilirubin levels. Multivariate analysis performed in all VRC Cmin/D identified CRP (p<0.0001), bilirubin (p = 0.01) and age (p = 0.007) as independent determinants of VRC Cmin/D, while genetic score had no impact. Genetic score was significantly associated with VRC Cmin/D only in the subgroup of VRC Cmin (n = 51) determined in absence of severe inflammation (defined by CRP level < median CRP of 65 mg/L).

**Conclusion:** Our genetic score that combined genetic variants of CYP2C19, 3A4 and 3A5 seems interesting to predict subtherapeutic VRC Cmin, but its impact is suppressed by severe inflammation. Strategies for individualization of VRC dose must integrate pharmacogenetics variants, but also the inflammatory status of patient.

### P467. Pharmacokinetics of posaconazole in critically ill patients undergoing extracorporeal membrane oxygenation (ECMO)

DaeleR. Van^1^,^2^BrüggemannR.J.M.^3^,^4^HoltappelsM.^5^DepuydtP.^6^RijndersB.^7^CottonF.^8^FageD.^8^DreesenE.^2^WautersJ.^9^,^10^SprietI.^1^,^2^1Pharmacy Department, University Hospitals Leuven, Leuven, Belgium2Department of Pharmaceutical and Pharmacological Sciences, KU Leuven, Leuven, Belgium3Radboud University Medical Center, Department of Pharmacy and Radboud Institute for Health Sciences, Nijmegen, Netherlands4Center of Expertise In Mycology Radboudumc /cwz, Radboud University Medical Center, Nijmegen, Netherlands5Laboratory For Clinical Infectious and Inflammatory Disorders, KU Leuven, Leuven, Belgium6Department of Intensive Care, Ghent University Hospital, Ghent, Belgium7Department of Infectious Diseases, Erasmus University Medical Center Rotterdam, Rotterdam, Netherlands8Department of Clinical Chemistry, LHUB-ULB, Erasme Hospital and Université Libre de Bruxelles, Bruxelles, Belgium9Department of Microbiology and Immunology, KU Leuven, Leuven, Belgium10Medical Intensive Care Unit, University Hospitals Leuven, Leuven, Belgium

**Objectives:** The use of posaconazole as prophylaxis for influenza-associated aspergillosis is currently investigated in critically ill patients. An important subset of patients admitted to the intensive care unit (ICU) for influenza is undergoing extracorporeal membrane oxygenation (ECMO) for severe acute hypoxemic respiratory failure. Little is known on the exposure to posaconazole in this population. This study presents the pharmacokinetics (PK) of seven ICU patients treated with intravenous posaconazole while on ICU undergoing ECMO.

**Methods:** All but one of the included patients were participating in the posaconazole-arm of the ongoing multicenter Posa-Flu study (NCT03378479), assessing the efficacy of a 7-day prophylactic intravenous treatment with posaconazole in ICU patients admitted for severe influenza. The additional patient was a lung transplant recipient treated with posaconazole for invasive aspergillosis. Blood samples were collected at an early (day 2-3) and late (day 5-7) time point, using a full (predose, 1.5,2,3,4,6,8,10,12,18,24h) or limited (predose, 1.5-3, 4-8, 8-12, 12-24h) sampling scheme. Trough concentrations were collected on all other days. All patients received an intravenous loading dose of 300 mg BID followed by a maintenance dose of 300 mg QD. Written informed consent was obtained from the patients or their relatives. PK parameters were estimated in Excel^®^.

**Results:** All patients received veno-venous ECMO and two patients were supported by continuous veno-venous hemodialysis (CVVH). The median area under the curve (AUC_0-24_), clearance (CL) and volume of distribution (Vd) were 35.69 mg.h/L, 8.42 L/h and 421.0 L, respectively. Exposure documented in the ECMO patients is shown in Table 1, along with the results previously documented in healthy volunteers, patients with hematological disease and critically ill patients. As shown in Figure 1, all trough concentrations (C_min_) were above 0.7 mg/L, which is the suggested threshold for prophylaxis in patients with hematological disease, and 71% of the C_min_ were also ≥ 1 mg/L, which is the suggested hematological threshold for treatment of invasive aspergillosis (ECIL-6 and ESCMID-ECMM-ERS 2017). The median average concentration (C_avg_) of both exposure days, i.e. 1.49 mg/L, was within the European Medicines Agency (EMA) target range (0.5-2.5 mg/L). The targeted posaconazole AUC_0-24_ of 20.87-22.5 mg.h/L for a MIC value of 0.125 mg/L (DRU 2014), was also achieved. 

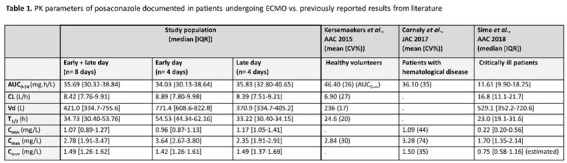

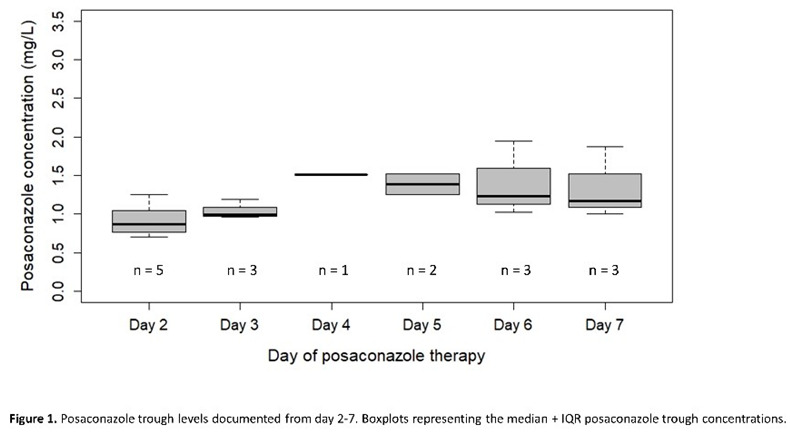





**Conclusion:** Posaconazole exposure was similar compared to that documented in hematology patients but higher than in ICU patients without ECMO. The lower limit for prophylaxis, as recommended in patients with hematological malignancies, is attained in all patients. This should be interpreted with caution as the optimal exposure to ensure prophylactic efficacy might be different in critically ill patients admitted for severe influenza. This optimal exposure is currently being investigated in the Posa-Flu trial. Based on this limited dataset, ECMO does not seem to influence posaconazole exposure. An *a priori* dose adjustment does not seem to be necessary but therapeutic drug monitoring is recommended to confirm the exposure. Despite the fact that this observation should be established in a larger number of patients, these results suggest that posaconazole exposure is maintained in critically ill patients undergoing ECMO. Hence we recommend the standard dosing regimen.

### P468. Pharmacokinetics of posaconazole in normal-weight and morbidly obese adults

WasmannR.E.^1^,^2^SmitC.^3^,^4^DonselaarM. Van^1^DongenE. Van^5^WiezerR.^6^BurgerD.^1^KnibbeC.^3^,^4^BrüggemannR.J.M.^1^,^2^1Pharmacy, Radboudumc, Nijmegen, Netherlands2Center of Expertise in Mycology Radboudumc / CWZ, Nijmegen, Netherlands3Clinical Pharmacy, St. Antonius Hospital, Nieuwegein, Netherlands4Division of Systems Biomedicine and Pharmacology, Leiden Academic Centre For Drug Research, Leiden University, Leiden, Netherlands5Department of Anesthesiology, Intensive Care and Pain Management, St. Antonius Hospital, Nieuwegein, Netherlands6Department of Surgery, St. Antonius Hospital, Nieuwegein, Netherlands

**Objectives:** One in five individuals will be obese in 2025. Obesity is associated with underdosing of antimicrobial drugs for prophylaxis and treatment of (life-threatening) infections. Posaconazole is a broad-spectrum triazole antifungal drug frequently used for prophylaxis and treatment of mold infections. There is anecdotal evidence that posaconazole exposure is impacted by weight resulting in suboptimal target attainment. We performed a prospective clinical trial to investigate the effect of body size in obese subjects and determined the probability of reaching the target trough concentration for prophylaxis (>0.7mg/L) and therapy (>1.0mg/L).

**Methods:** Obese subjects (BMI above 35 kg/m2) undergoing bariatric surgery and normal-weight subjects (BMI 18.5-25 kg/m2) were included in an open-label, single-dose, multicenter, pharmacokinetic study. Obese subjects were randomized to receive either 300 or 400 mg posaconazole IV while normal-weight subjects all received 300 mg posaconazole IV. Blood samples were collected at nine time points up to 24 hours after start of infusion. Statistical analysis and population pharmacokinetic modeling was performed using R and NONMEM 7.3.

**Results:** 8 obese subjects on 300 mg, 8 obese subjects on 400 mg and 8 normal-weight subjects on 300 mg were included. Weight [range] was 129 kg [109-190]), 144 kg [107-175]), 72.3 kg [61.4-85.4] respectively. The observed geometric mean [range] AUC_0-24h_ in normal-weight versus obese subjects receiving 300 mg posaconazole was 21.4 mg*h/L [15.6-29.1] versus 13.1 mg*h/L [9.1-18.5] (p<0.05). Obese subjects receiving 400 mg posaconazole had an AUC_0-24h_ of 16.8 mg*h/L [12.2-25.6].A two-compartment model with first-order elimination, a proportional residual error model and inter-individual variability on the central compartment best described the data. Total body weight was the best predictor of variability of clearance and the central and peripheral compartment. Simulations demonstrated that a 300 mg dose results in a >90% PTA in patients up to 190kg for prophylaxis. For treatment, a 300 mg dose is sufficient up to 120 kg after which a dose increase to 400 mg daily should result in a >90% PTA up to 170 kg.

**Conclusion:** We found that obese individuals have a significant lower exposure to posaconazole compared to normal-weight individuals even when using a 400 mg dose. Nevertheless, PTA (at 0.7 mg/L) was high using the IV formulation. In the treatment setting higher dosages are warranted.

### P469. The effects of Cytochrome P450 2C19 profile on voriconazole serum trough concentrations and hepatotoxicity in Haematology patients

ChongL.L.^1^LingL.M.^2^1Haematology, Tan Tock Seng Hospital, Singapore, Singapore2Infectious Diseases, Tan Tock Seng Hospital, Singapore, Singapore

**Objectives:** Cytochrome P450 (CYP) 2C19 genetic polymorphism contributes to variability of voriconazole pharmacokinetics. Supra-therapeutic voriconazole trough concentrations have been correlated with hepatotoxicity. This study aims to establish the effects of CYP2C19 profile on voriconazole troughs and hepatotoxicity.

**Methods:** A prospective, observational study was done to determine voriconazole trough concentrations at pre-steady state (day 2 to 4 of therapy) and steady state (day 6 onwards). Blood sampling was repeated weekly until week 12 of recruitment or death. The CYP2C19 genotype was also identified for patients on voriconazole.

**Results:** 11 patients were analyzed for CYP2C19 polymorphism: 1*/1* normal metabolizers (n = 5, 45 %), 1*/2* intermediate metabolizers (n = 3, 27 %) and 2*/2* poor metabolizers (n = 3, 27 %), respectively. 4 out of 6 patients were intermediate or poor metabolizers whom had supra-therapeutic voriconazole concentrations (trough > 5.5 mg/L). All poor metabolizers developed abnormal liver function tests (range of ALP: 230 to 630).

**Conclusion:** Knowledge of CYP2C19 profile would allow pre-emptive dose adjustments during the first week of voriconazole therapy and thus reduce the incidence of hepatotoxicity.

### P470. rObese patients require a fixed dose of liposomal amphotericin B

WasmannR.E.^1^,^2^SmitC.^3^DongenE. Van^5^WiezerR.^6^Adler-MooreJ.^7^BeerY. De^8^BurgerD.^1^KnibbeC.^3^,^4^BrüggemannR.J.M.^9^,^10^1Pharmacy, Radboudumc, Nijmegen, Netherlands2Center of Expertise in Mycology Radboudumc / CWZ, Nijmegen, Netherlands3Clinical Pharmacy, St. Antonius Hospital, Nieuwegein, Netherlands4Division of Systems Biomedicine and Pharmacology, Leiden Academic Centre For Drug Research, Leiden University, Leiden, Netherlands5Department of Anesthesiology, Intensive Care and Pain Management, St. Antonius Hospital, Nieuwegein, Netherlands6Department of Surgery, St. Antonius Hospital, Nieuwegein, Netherlands7Biology, Cal Poly Pomona, Pomona, United States of America8Department of Clinical Pharmacy & Toxicology, Maastricht University Medical Center+, Maastricht, Netherlands9Pharmacy, RadboudUMC, Nijmegen, Netherlands10Center of Expertise In Mycology Radboudumc /cwz, Radboud University Medical Center, Nijmegen, Netherlands

**Objectives:** In 2014, approximately 11% of adult men and 15% of adult women were obese worldwide, a number that is increasing. Like other patients, obese patients may be confronted with invasive fungal disease and require treatment with antifungal agents such as liposomal amphotericin B (L-AmB, Ambisome®). We performed a pharmacokinetic study in morbidly obese individuals to define the optimal dose of L-Amb by quantifying the impact of obesity on the pharmacokinetics of L-AmB.

**Methods:** In this open-label study, morbidly obese but otherwise healthy subjects (BMI > 40 kg/m^2^) received L-AmB received a single dose of 1 or 2 mg/kg (n = 8+8) the day before undergoing laparoscopic bariatric surgery. Each subject was intensively sampled (n = 9) up to 48 hours to determine amphotericin B plasma concentrations. The following information was collected: body size descriptors (e.g. total body weight [TBW], lean body weight, BMI, ideal body weight calculated according to Janmahasatian *et al.*, body surface area), age and sex. Population pharmacokinetic analyses was performed in NONMEM 7.3 with PsN 4.7. For the covariate analyses the relationships between empirical Bayes estimates and covariates were investigated in scatter plots. The final model was used to simulated AUC_0-24h_ and C_max_ in steady state conditions in 10.000 subjects with body weights uniformly distributed between 60 and 180 kg. Each virtual subject < 100 kg received a daily 3 mg/kg L-AmB infusion in one hour, subjects ≥100 kg received either 3 mg/kg or a fixed 300 mg dose as a one hour infusion.

**Results:** 16 morbidly obese subjects with median [range] BMI of 45.9 [40.2-52.1] kg/m^2^ and a total body weight of 137 [104-177] kg were included. A two-compartment model best explained the data. There was no relationship between clearance of L-AmB and any of the body size descriptors. A linear relationship was found between the central volume of distribution (V_c_) and TBW. Simulations show that the AUC_0-24_ increases with weight when patients are dosed on a mg per kg basis). In parallel, C_max_ also increases with body weight when L-AmB is dosed on a per kg basis. This phenomenon is primarily driven by the absolute increase in the dose with a clearance that does not change with weight. When using a dose cap of 300 mg for patients > 100 kg the AUC would remain similar across weight while a similar C_max_ (13% lower) in a 140 kg patient compared to a 70 kg patient will be obtained.

**Conclusion:** Based on our results we see limitations to using a mg per kg dose regime for adult patients as clearance and therefore exposure to AmB is not affected by body weight. In addition, body weight derived dosing might lead to an increased risk of toxicity in heavy patients since toxicity is associated with AUC. A fixed dose regimen would not only be more appropriate but also more practical for clinicians. In obese patients specifically, we recommend using the licensed 3 mg/kg dose and cap the dose at a maximum weight of 100 kg resulting in a 300 mg fixed dose.

### P471. Evaluation of the in vitro antifungal activities of combinations of amphotericin B with anidulafungin or caspofungin against Candida auris by time-kill curves

CaballeroU.^1^Nuñez-MarcosS.^1^,^2^ErasoE.^2^PemánJ.^3^QuindósG.^2^JauregizarN.^1^1Pharmacology, University of the Basque Country, Leioa, Spain2Inmunology, Microbiology, Parasitology, University of the Basque Country (UVP/EHU), Bilbao, Spain3Servicio De Microbiología, Hospital Universitario y Politécnico La Fe, Valencia, Spain

**Objectives:** *Candida auris* has recently emerged as a cause of nosocomial outbreaks. The health alert caused by this pathogen is related to the difficulty of a proper identification, its persistence in the hospital environment and multidrug resistance. Antifungal combination therapy may allow to overcome this multiresistance. Despite their relevance, research about the activity of antifungal drug combinations against *C. auris* is still scarce. The objective of this study was to evaluate the activity of the combinations of amphotericin B with anidulafungin or caspofungin against *C. auris* clinical isolates by time-kill curves.

**Methods:** *In vitro* static time-kill curves experiments were performed in duplicate in microtitre plates in RPMI-1640 medium with six *C. auris* clinical blood isolates (Hospital La Fe, Valencia, Spain). Inoculum size ranged from 1 to 5 x 10^5^ CFU/mL and concentrations assayed in combination experiments were 0.5 and 1 mg/L for amphotericin B and 0.25, 0.5, 1 and 2 mg/L for anidulafungin and caspofungin. Samples for viable counts were taken at 0, 2, 4, 6, 8, 24 and 48 h. Plots of averaged colony counts (log CFU/mL) versus time were constructed for data analysis. The first-order killing rate constant K (h^-1^) obtained after fitting an exponential equation to the data and time to achieve the fungicidal endpoint t_99.9%_ (h) were calculated to assess antifungal activity and to compare different combinations between them. Fungistatic or fungicidal activities, and synergistic, antagonist or indifferent interactions between these drugs were considered according to published definitions [1].

**Results:** Combinations of 0.5 mg/mL of amphotericin B with 0.5, 1 and 2 mg/mL of anidulafungin or caspofungin were synergistic (>2 log reduction compared to the drug with greater activity) and fungistatic. However, combinations of 1 mg/mL of amphotericin B with 0.25 and 0.5 mg/mL of anidulafungin or caspofungin were additive (<2 log reduction) and fungicidal. There were not significant differences between the use of anidulafungin or caspofungin in combinations with amphotericin B.

**Conclusion:** Combination of amphotericin B with anidulafungin and caspofungin showed promising results. Combinations of 0.5 mg/L of amphotericin B with low doses of echinocandins were synergistic, whereas the interaction with 1 mg/mL of amphotericin B was additive but exerted fungicidal activity. This is a remarkable finding, specially if we take into account that echinocandins do not show fungicidal behaviour against *C. auris*. The results could support the use of these antifungal combinations for treating *C. auris* deep infections. Funding: This study was partially financed by GIC 15/78 IT-990-16 (Gobierno Vasco-Eusko Jaurlaritza), PI17/01538 (FIS, Spain) and SAF2017-86188-P (MINECO, Spain). Unai Caballero has received a grant by the University of the Basque Country (UPV/EHU) [1] Serena C et al., *In vitro* interactions of micafungin with amphotericin B against clinical isolates of *Candida* spp. Antimicrob Agents Chemother. 2008; 52(4):1529-32

### P472. Time-kill Studies of Caspofungin in an Ex vivo model in Relation to Plasma Protein Levels: Is the Protein Binding Less Than Expected?

KurlandS.^1^FurebringM.^1^ShamsA.^1^ChryssanthouE.^2^SjölinJ.^1^LöwdinE.^1^1Section of Infectious Diseases, Department of Medical Sciences, Uppsala University, Uppsala, Sweden2Clinical Microbiology, Karolinska University Hospital, Stockholm, Sweden

**Objectives:** The echinocandin caspofungin is used as first-line therapy in candidemia, the most common invasive candida infection. The protein bound fraction of caspofungin is 96.5% (in healthy volunteers). In our recent study on critically ill patients treated with caspofungin, a median AUC in plasma of 58 (range 44-113) mg*h/L was reported, corresponding to an average plasma concentration of 2.4 (1.8-4.7) mg/L. This would result in a free unbound concentration of 0.08 (0.06-0.16) mg/L, concentrations equivalent to the MIC distribution for wild type *Candida glabrata*. For fungicidal effect higher free concentrations would be needed. In critically ill patients the plasma protein level is habitually reduced. The objectives of this study were to determine the killing effect of caspofungin on *C. glabrata* with clinically relevant concentrations of caspofungin in an *ex vivo* model using human plasma and furthermore to study the efficacy in relation to different plasma protein levels.

**Methods:** The efficacy of caspofungin on four blood isolates of *C. glabrata* was determined by time-kill studies performed in plasma from healthy blood donors. In order to investigate the significance of the protein level on the killing effect of caspofungin, *C. glabrata* with an inoculum of 5x10^4^ CFU/ml was added to plasma, plasma diluted with 50% Ringer´s Acetate (Ri-Ac) and plasma diluted with 75% Ri-Ac. The MIC values for the isolates were determined by Sensititre Yeast One method and were 0.06, 0.12 and 1 mg/L, respectively. The caspofungin concentrations investigated were 1, 2, 5 and 9 mg/L. CFU/ml were determined at 0, 2, 6 and 24 hours.

**Results:** The results showed a higher reduction in CFU/ml than expected based on MIC and the calculated unbound caspofungin concentration. There was a ≥ 2 log reduction in log CFU/ml with a concentration of 1 mg/L (corresponding to 0.035 mg/L free drug/unbound concentration) for the two strains with MIC 0.06 mg/L. For the strain with MIC 0.12 mg/Lthe corresponding reduction was approximately 1 log CFU/ml. Furthermore, there was a limited but significant decrease in CFU/ml in the time-kill studies performed in plasma with reduced levels of protein in comparison with plasma with normal protein level. For the strain with MIC 1 mg/L there was no reduction in CFU/ml in plasma. Although, there was a killing with the concentrations 5 and 9 mg/L in the experiments in plasma diluted with 75% Ri-Ac.

**Conclusion:** The killing of *C. glabrata* with caspofungin in plasma was higher than expected based on MIC and the reported amount of unbound caspofungin in normal plasma. At lower protein levels this killing was augmented which is consistent with an increased concentration of free caspofungin.

### P475. Subcutaneous nodule caused by Phaeoacremonium fuscum in a non-immunocompromised patient.

ColmanS.CattoirL.BoelA.Microbiology, OLV Hospital, Aalst, Belgium

**Case Report:** Introduction Phaeohyphomycosis (PHM) is a chronic infection caused by dematiaceous fungi which usually involves the skin and subcutaneous tissue. As a result of the use of molecular diagnostic techniques, previously unrecognized causative agents of phaeohyphomycosis have been discovered. Here we report the first case of phaeohyphomycosis caused by *Phaeoacremonium fuscum* in an immunocompetent patient. Case presentation A 60 year old Filipino male presented with rotator cuff pathology to the orthopedics consultation. On physical examination a painless, soft, subcutaneous mass above the right patella was noted, which didn’t cause the patient any complaints. The suprapatellar nodule had been growing slowly for 3 years. The patient denied any apparent antecedent trauma, arthropod bite, family history or contact with a person with similar lesions. The man still visits the Philippines on an infrequent basis. He had no fever, weight loss or other symptoms. Detailed physical examination further revealed a 4x3 cm, immobile, non-pulsatile light red mass. There were no other signs of inflammation of the skin. A serous effusion was aspirated from the mass. Fungal culture of the aspirated fluid showed growth of a filamentous fungus on chocolate blood agar and Sabouraud’s dextrose agar. Molecular identification was performed by sequence analysis of the internal transcribed spacer region 2 (ITS2). BLAST search revealed a similarity of 100% with *Phaeoacremonium fuscum*. The antifungal susceptibility profile was established by EUCAST broth microdilution: itraconazole >16 mg/L, posaconazole 0.25 mg/L, amphotericin B 0.50 mg/L, voriconazole 0.50 mg/L. This susceptibility profile is similar to those previously published. A wide local excision of the lesion was performed a couple of weeks later. The patient had an uneventful postoperative recovery. No antifungal therapy was started postoperatively since the patient was not immunocompromised and there was lack of reimbursement. Histopathological examination of the specimen showed fibrous tissue with a mixed cellular infiltrate including lymphocytes, plasma cells, macrophages and neutrophils. Periodic acid-schiff stain and Grocott stain showed evidence of branching septate hyphae. Discussion PHM is caused by brown pigmented fungi containing melanin in their cell wall. Phaeoacremonium species are known as vascular plant pathogens, responsible for wilting and dieback of woody plants. They are also opportunistic pathogens in humans, mainly causing cutaneous or subcutaneous PHM. However, sporadic cases of respiratory tract infection, septic arthritis and disseminated infection are described. The true prevalence of Phaeoacremonium species is probably underestimated due to the asymptomatic nature of most subcutaneous lesions and the difficult identification. There is no standard antifungal regimen or evidence regarding the necessity for antifungal treatment as concomitant with surgery for localized infections. Long courses of antifungals, such as voriconazole treatment, are often employed to reduce the risk of recurrence. Case reports suggest that surgery can be curative if the infection is localized and adequately removed, as was the case in our patient. Regardless of the immune status of the patient, clinicians should consider the possibility of a fungal infection when they encounter chronic subcutaneous nodules without major symptoms.

### P476. Belgian national survey on tinea capitis: epidemiology and molecular investigations.

SacheliR.^1^MenatongX.^1^LabarbeC.^1^CrutzenC.^1^HaragS.^2^AndréJ.^2^EvrardS.^3^LagrouK.^4^LaffineurK.^5^RousseauxD.^6^DetollenaereL.^1^AdjeteyC.^1^SeidelL.^7^HayetteM.-P.^1^1Clinical Microbiology- Nrc For Mycoses, University Hospital of Liège, Liège, Belgium2Dermatology, CHU St Pierre, Brussels, Belgium3Clinical Microbiology, CHR Citadelle, Liège, Belgium4Clinical Bacteriology and Mycology, Katholieke Universiteit Leuven, Leuven, Belgium5Clinical Biology, Clinique St Luc Bouge, Bouge, Belgium6Clinical Biology, Centre Hospitalier Chretien Heusy, Verviers, Belgium7Statistics, University Hospital of Liège, Liège, Belgium

**Objectives:** Tinea capitis (TC) is a superficial infection of the scalp caused by dermatophyte fungi which affects mainly prepubescent children. This last decade, a huge increase of African anthropophilic strains causing tinea capitis, has been observed in Europe, probably due to immigration waves from African countries. The Belgian National Reference Center for Mycosis (NRC) has conducted a surveillance study about TC in 2018. This work presents final results of the study for the epidemiological part and preliminary results for the molecular part.

**Methods:** Belgian laboratories were invited to send all dermatophytes strains isolated from the scalp from January to December 2018. Dermatologists were involved and were asked to fill a form containing several epidemiological information about the patient. Strains identification was confirmed by ITS sequencing. A multiplex pan-dermatophyte real time PCR assay (DermaGenius^®^, PathoNostics) was applied if necessary. Typing of the *M.audouinii* strains was done using rep-PCR method (Diversilab, BioMérieux).

**Results:** A total of 337 strains have been collected from 337 patients. The main population concerned by TC was children from 5-9 years (165/337, 49,01%). Males (214/337, 63,5%) were more affected than females (123/337, 36,5%), the sex ratio M/F was of 1,74. The majority of the strains was collected in Brussels area (181/337, 53,8%), followed by Liege area (73/337; 21,7%). Other Belgian cities were less concerned by TC. Among known ethnical origins (n = 119), African people (114/119, 96,2%) were more concerned by TC than European people (5/119, 3,8%), (p<0,0001). The majority of patients were from Guinea (26/119, 21,8%), followed by Cameroun (14/119, 11,8%) and RDC (14/119, 11,8%), many other African nationalities were represented (12 different countries, all over Africa). The main transmission mode of TC was the familial way (83,3% among known cases n = 126, 105/126). The major etiological agent was *Microsporum audouinii* (118/337, 35%) followed by *Trichophyton soudanense* (83/337, 24,6%), *T. tonsurans* (27/337, 17%), *M. canis* (36/337, 10,7%), *T. violaceum* (28/337, 8,3%), *T. benhamiae* (7/337, 2,1%), *T. mentagrophytes* (5/337, 1,48%) and *M. incurvatum* (1/337, 0,3%). This last rare dermatophyte has never been reported as responsible for TC in Belgium before. *M. audouinii* strains have been genotyped by rep-PCR and three genotypic variants have been characterized, one of them circulating mainly in Brussels area. No link with a particular ethnical origin could be found among genotypic groups.

**Conclusion:** African anthropophilic dermatophytes such as *M.audouinii* and *T. soudanense* are mainly responsible for tinea capitis in Belgium. Large cosmopolitan cities like Brussels and Liege are the most concerned. People from African origin are mostly affected by TC. Among the *M. audouinii* strains circulating in Belgium, a genotypic diversity has been characterized.

### P477. Identification of dermatophytes isolated from tinea capitis infections using a liquid media MALDI-TOF MS protocol

LecerfP.^1^JazaeriY.^1^HendrickxM.^2^RichertB.^1^PackeuA.^3^1Faculty of Medecine, Université Libre de Bruxelles, Brussels, Belgium2Mycology, Sciensano, BCCM/IHEM, Brussels, Belgium3Mycology & Aerobiology, Sciensano, Brussels, Belgium

**Objectives:** *Tinea capitis* is a common childhood superficial infection of the scalp caused by dermatophytes. Conventional methods based on morphological characteristics to identify theses fungi are time-consuming and complex, requiring expert mycological knowledge. Recently, MALDI-TOF MS has become a powerful tool in clinical microbiology for rapid identification of these microorganisms. In the present study, a liquid media MALDI-TOF MS protocol was developed and validated for the fast and accurate identification of dermatophytes species responsible for *tinea capitis* infection, using both dermatophytes from reference strains and clinical isolates. The main purpose was to work with primary isolates in order to avoid the culture step and lead to a faster diagnosis. Identification techniques (conventional identification and MALDI-TOF MS based on liquid and solid culture) were evaluated and compared for their rate of correct identification and turnaround time.for the fast and accurate identification of dermatophytes species responsible for *tinea capitis* infection, using both dermatophytes from reference strains and clinical isolates. The main purpose was to work with primary isolates in order to avoid the culture step and lead to a faster diagnosis. Identification techniques (conventional identification and MALDI-TOF MS based on liquid and solid culture) were evaluated and compared for their rate of correct identification and turnaround time.

**Methods:** The in-house BCCM/IHEM database made from references strains on solid culture was used. First, the liquid media MALDI-TOF MS protocol was validated using references strains. Secondly, the same protocol was applied on clinical isolates collected from Saint-Pierre University Hospital and paralleled with solid media MALDI-TOF MS protocol.

**Results:** The use of the liquid media MALDI-TOF MS protocol resulted in a rate of 100% of correct identification at the species level for reference strains and 78,8% for clinical isolates. The protocol was statistically significantly faster than the conventional method. The results of the different methods disagreed for 17 isolates. The complete extraction from the solid media MALDI-TOF MS gave the highest correct species identification with the highest mean of log-scores.

**Conclusion:** The liquid media MALDI-TOF MS technique is an accurate method for the correct identification of dermatophytes at the species level and is much faster than the conventional technique. The main advantage lies in not needing a culture step for primary isolates, thus leading to a faster diagnosis. For the identification of closely related species, the complete extraction from solid media MALDI-TOF MS protocol remains the most reliable technique.

### P478. Dermatophytes’ identification by Matrix-assisted laser desorption ionization-time of flight mass spectrometry (MALDI-TOF MS) - the experience of a clinical laboratory

VerissimoC.SimõesH.SabinoR.SimõesD.Infectious Disease, National Health Institute, Lisbon, Portugal

**Objectives:** Dermatophytes are a challenging group of fungi that infect the keratinized tissues. The taxonomy of these fungi has changed recently with the reclassification of some species and description of new ones. However, many clinical laboratories still base the identification of dermatophytes on their phenotype. Since dermatophytes are very pleomorphic, macro and micromorphology are often insufficient to reach a correct classification and may lead to misidentifications. The identification based on MALDI-TOF relies on the protein profile of the microorganism. Thus, this study aims to summarize our current laboratorial experience of dermatophyte identification using MALDI-TOF MS.

**Methods:** From january to april 2018, 95 dermatophytes isolates, collected from human keratinized samples and also from quality control programs were characterized by phenotypic analysis, and by VITEK MS V3.2 bioMerieux. Before identification procedure, isolates were inoculated on Sabouraud Dextrose agar plates and incubated at 27°C during 5 to 10 days. Species were identified taking into account clinical features, as well as cultural, microscopic and physiological characteristics. Prior to MALDI-TOF MS analysis, the samples were pre-treated according to the manufacturer’s protocol for filamentous fungi. Molecular identification by sequencing of the internal transcribed spacer 1 (ITS1) was performed in 34 of those isolates

**Results:** Through phenotypic analysis eight different species were identified (54 Trichophyton rubrum; 4 T.soudanense; 22 T.interdigitale; 1 T.mentagrophytes; 3 T.tonsurans; 7 Microsporum canis; 3 M.audouinii; 1 Microsporum spp.- (non canis or audouinii). MALDI-TOF analysis showed an identification agreement in 80 cases (84,2%) with a confidence level of 99,9%. Eight isolates showed divergent identification results: three T.rubrum were identified as T.violaceum, three T.soudanense were identified as T.rubrum, one T.mentagrophytes was identified as T.interdigitale and one T.tonsurans was identified as T.rubrum. In four cases MALDI-TOF analysis did not get a profile. The ITS sequencing analysis of discrepant results corroborated the MALDI-TOF identification in five of them. On the other hand, T.soudanense was only identified by phenotypic analysis since MALDI-TOF and ITS sequencing result was T.rubrum. MALDITOF identification of T.violaceum was not confirmed by ITS sequencing that identified T. rubrum instead, in accordance with the phenotypic identification.

**Conclusion:** Correct identification of dermatophytes to species level requires sequencing of the ITS, LSU, and/or beta-tubulin regions. The implementation of this methodology in a clinical laboratory is expensive and time consuming. MALDI-TOF identification is a good option for dermatophytes’ identification performed in laboratory routine, since costs of consumables as well as time of sample preparation are lower than for PCR analysis and doesn’t require long training period as phenotypic identification does. In this study, however, both methods failed to identify some species variants like Trichophyton soudanense or T. violaceum. The combined use of both MALDI-TOF and phenotypic methods seems to be the better approach for dermatophytes’ identification since some species show significant phenotypic and clinical differences.

### P479. A retrospective study of dermatophyte infections caused by Tricophyton rubrum and T. interdigitale in the County of Stockholm, Sweden.

VargasL.AlvaradoE.StenströmC.ForsblomS.GirestamB.LokoG.ChryssanthouE.Clinical Microbiology, Karolinska University Hospital, Stockholm, Sweden

**Objectives:** Dermatophytosis is a common fungal infection caused by keratinophilic fungi that are capable of invading nail, hair and superficial layers of the skin of humans and animals. The aim of this study was to determine the epidemiologic profile of the most frequent dermatophytosis such as *Tinea unguium* and *T. pedis* caused by *Tricophyton rubrum* or *T. interdigitale* in Stockholm, Sweden.

**Methods:** In 2015 we developed and validated a rapid and sensitive real-time PCR method for detection of the two most prevalent dermatophytes in Northern Europe. Fungal DNA was extracted directly from clinical samples (toe or finger nail and skin scrapings from the feet) by using proteinase K and heat as pre-lysis step, followed by automated DNA extraction on the PSH/MagNA Pure 96 Compact. Real-time PCR was performed using pan-dermatophyte primers for detection of all dermatophytes and specific primers for identification of *T. rubrum* and *T. interdigitale*. Specimens from finger nails were also cultured on Chrom agar plates for detection of onychomycosis caused by yeasts.

**Results:** Laboratory records comprising PCR results of 13,683 specimens (3,304 skin respective 10,379 nail fragments) collected from May 2015 through March 2019 were retrospectively analyzed. *Tricophyton rubrum* (42%) was the predominant pathogen identified from these cases, followed by *T. interdigitale* (4,9%). In contrast, 2% of the samples were positive for pan-Derm indicating the prevalence of infections caused by other pathogens distinct to *T. rubrum* or *T. interdigitale*. Sex distribution analysis of the patients showed that males (40%) were more susceptible to suffer dermatophyte infections compared to females (21,9%). (Furthermore, the prevalence of onychomycosis in finger nails caused by yeasts were also evaluated in this study.) Culture analysis from total 941 finger nails showed that 25% were positive for yeast infection. *Candida parapsilosis* (52%), followed by *C. albicans* (35%) and *C. guilliermondii* (6%) were the predominant species isolated in such cultures.

**Conclusion:** The findings presented in our study are in concordance with previous studies where *T. rubrum* is the most frequent dermatophyte species isolated in the general population of industrialized countries. Detection of dermatophytes using real-time PCR represents a faster and sensitive method to evaluate large volume of clinical samples.

### P480. rEvaluation of Diafactory tinea unguium®, a new rapid immunochromatographic test for the diagnostic of onychomycosis

RupinE.PellouxH.Fricker-HidalgoH.LecciaM.-T.CognetO.Parasitology-mycology, Grenoble Alps University Hospital, GRENOBLE, France

**Objectives:** Suspected nail onychomycosis remains a frequent and still increasing problem despite therapeutic advances. Based on clinical features the diagnosis of onychomycosis is confirmed by direct examination and culture. Direct examination can be done on the same day but cultures required several weeks what delays diagnosis and therapy. Furthermore, direct examination and culture have low sensitivity. Diafactory tinea unguium^®^, a new rapid immunochromatography test was recently developed. The aim of this study was to compare this new test to conventional methods (direct examination and culture) in suspected tinea unguium.

**Methods:** Diafactory tinea unguium^®^ (Biosynex, France) detects dermatophytes by using monoclonal antibodies that react specifically with polysaccharide present in the cell wall of dermatophytes. Direct microscopic examination was performed using KOH enhanced with chlorazol black E and dimethyl sulfoxide. Cultures were carried out on Sabouraud medium with or without cycloheximide (bioMérieux, France), incubated at 25°C. The three methods were evaluated on nails samples (n = 70) collected from patients (n = 50) with suspected nail mycosis infection in the Grenoble Alps University Hospital.

**Results:** Among the 70 samples, cultures were positive in 53 nails. Dermatophytes were isolated in 40 samples (*Trichophyton rubrum* n = 35, *Trichophyton interdigitale* n = 3, *Trichophyton tonsurans* n = 2), yeasts in 8 samples (*Candida parapsilosis* n = 6, *Candida albicans* n = 2), and moulds in 5 samples (*Scopulariopsis* spp. n = 4, *Fusarium* spp. n = 1). The results were positive with Diafactory tinea unguium^®^ in 48 samples, correlated with direct microscopy (i.e. presence of filamentous fungal elements) in 36 samples and with cultures in 39 samples. In one case, the result of Diafactory tinea unguium^®^ was negative and the culture was positive *(T. rubrum*). In three cases, the result of Diafactory tinea unguium^®^ was negative and the direct examination was positive. In the 13 cases of moulds or yeasts isolation, the immunochromatographic test was negative. The positive and negative predictive value of Diafactory tinea unguium^®^ were respectively 75% and 86%.

**Conclusion:** In order to reinforce the importance of confirmation in the diagnosis of mycological onychomycosis, Diafactory tinea unguium^®^ provides a reliable, convenient and quick method (5 to 30 minutes). Because of its good detection capacity, this device improved the accuracy of diagnosis of tinea inguium in complement of conventional fungus testing methods.

### P481. Molecular identification of Dermatophytes from clinical samples

GazitZ.^1^Gefen-HaleviS.^2^BelausovN.^1^KellerN.^1^SmollanG.^1^1Clinical Microbiology, Sheba medical center, Ramat Gan, Israel2Clinical Microbiology, Sheba medical center, ramat gan, Israel

**Objectives:** Dermatophytes are fungi that can invade and infect the keratinized layers of skin, hair, and nails. Three genera of fungi, Trichophyton (T), Microsporum (M), and Epidermophyton (E), account for most Dermatophytic infections and cause a pathology called tinea (ringworm fungi). The gold-standard method for dermatophyte identification is microscopy and culture. Microscopy has low specificity and culture has low

**Methods:** (1) The gold-standard methods routinely used in the laboratory were microscopy using 30% KOH and culture using DTM (dermatophyte test medium) agar incubated at 28 °C for up to 4 weeks. For 12 months, from 1.1.2016 until 31.12.2016 samples from skin, nail and hair from patients with suspected tinea were analyzed using the gold-standard methods. (2) A new Israeli commercial molecular kit called DERMADYN Dermatophyes detection test V.01 (Dyn R&D) kit that identifies 7 dermatophytes; *T. rubrum* complex*, T. tonsurans, T. violaceum, M. gypseum, E. floccosum, M. canis and T. mentagrophytes* complex was evaluated compared to the gold-standard on 103 samples. (3) From 1.8.18 until 31.12.18, 1179 samples were analyzed for the presence of Dermatophytes using the molecular kit only.

**Results:** Using the gold-standard only 480 (23.6%) samples were positive for dermatophytes with a distribution of 462 (23%), 12 (0.6%), 2 (0.1%), 2 (0.1%) 1 (0.05%), 1 (0.05%) positive for *T. rubrum* complex*, T. mentagrophytes* complex*, M. canis, Scopulariopsis brevicaulis, T. tonsurans, and M. gypseum*, respectively. Discrepancies between microscopy and culture were found in 27% of the samples. (2) There was an agreement between the gold-standard and the molecular kit in 60 out of the 103 samples tested (58%), a complete disagreement in 9 (9%) and a partial disagreement in 34 (33%) of them. (3) Using the molecular approach 578 (49%) samples were positive for dermatophytes with the distribution of 551 (46.7%), 2 (0.2%), 8 (0.7%), 6 (0.5%), 1 (0.08%) and 10 (0.85%) positive for *T. rubrum complex, T. mentagrophytes* complex*, M. canis, T. tonsurans, M. gypseum and E. floccosum*, respectively.

**Conclusion:** Twice the number of dermatophytes were identified using the molecular approach compared to the gold-standard with a similar distribution of species having *T. rubrum* complex the main pathogen. The molecular assay has a much shorter training period, a high capacity of performance and high sensitivity. Although the kit contains only 7 dermatophytes, these are the most prevalent species in Israel. As discrepancies between microscopy and culture is a common problem and as culture has low sensitivity Labs should consider evaluating a molecular based diagnostic method for dermatophyte identification to increase detection rates.

### P483. In the Aspergillus section Nigri, Aspergillus welwitschiae is more often responsible for otomycosis than Aspergillus tubingensis and Aspergillus niger

Gits-MuselliM.^1^,^2^HamaneS.^2^VerillaudB.^3^CherpinE.^2^GuigueN.^2^Garcia-HermosoD.^1^AlanioA.^4^BretagneS.^4^1Molecular Mycology, Institut Pasteur, PARIS, France2Parasitology-mycology Laboratory, Lariboisière Saint-Louis Fernand Widal Hospital, Assistance Publique-Hôpitaux de Paris (AP-HP), PARIS, France3Ent Department, Lariboisière Saint-Louis Fernand Widal Hospital, Assistance Publique-Hôpitaux de Paris (AP-HP), PARIS, France4Paris-diderot, Sorbonne Paris Cité University, Institut Pasteur, Molecular Mycology Unit, Cnrs Cmr2000, Parasitology-Mycology Laboratory, Lariboisière Saint-Louis Fernand Widal Hospitals, Assistance Publique-Hôpitaux de Paris, Paris, France

**Objectives:** Fifteen percent of external otitis are otomycosis (Ninkovic et al. 2008). *Aspergillus* section *Nigri* are the most frequent molds isolated from external ear conduct (EEC) samples followed by *Aspergillus* section *Flavi* (R. Sarvan et al. 2012). *Aspergillus* section *Nigri* include several cryptic species difficult to differentiate using phenotypic features. We wondered whether some species of the Nigri section could be more frequently observed in some specific anatomical sites.

**Methods:** We identified at the species level all the isolates of *Aspergillus* section *Nigri* consecutively collected between 2010 and 2017 in our laboratory and stored frozen at -20 C°. After thawing and subculture on Sabouraud medium, DNA was extracted using a MagnaPure DNA extractor and species identification was performed upon the partial sequence of the calmodulin gene. The two primers used for amplification of the targeted gene were CMD5 (CCGAGTACAAGGARGCCTTC) and CMD6 (CCGATRGAGGTCATRACGTGG) (Hong et al. 2005). The sequences were compared to the Mycobank database. The sequences obtained were aligned to build a phylogenic tree using Geneious software.

**Results:** A total of 186 strains were sequenced: 75 EEC (64 patients), 78 respiratory samples (78 patients), and 33 air environmental isolates. When considering the first strain per patient, we identified by decreasing frequency: *Aspergillus tubingensis* (78/175; 45 %), *Aspergillus welwitschiae* (59/175; 34 %), *Aspergillus niger* (34/175; 19 %), *Aspergillus luchuensis* (2/175; 1%), and *Aspergillus japonicus* (2/175; 1%). When several isolates were available for a given patients, the following isolates belonged to the same species. The species repartition was statistically different when comparing EEC samples to respiratory and air samples with *A. welwitschiae* overrepresented in otomycosis (53% 34/64) (p<0.001). In contrast, the distribution of the species was not different between respiratory and air samples, with 46% and 61% *A. tubingensis* respectively (Figure 1).

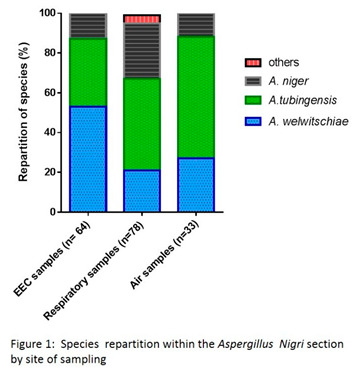



Inside each species, the sequence identity was high (96.5% for *A. tubingensis*, 99.3% *A. welwitschiae*, and 99.4% *A. niger* isolates) with no difference according to the isolation site. The phylogenetic tree based on the calmodulin gene partial sequences showed a stackable organization to the tree generated using whole genome sequencing (Vesth et al. (2018)), with *Aspergillus luchuensis* included within the *A. tubingensis* clade, and *A. welwitschiae* closed to the *A. niger* clade (Figure 2). 

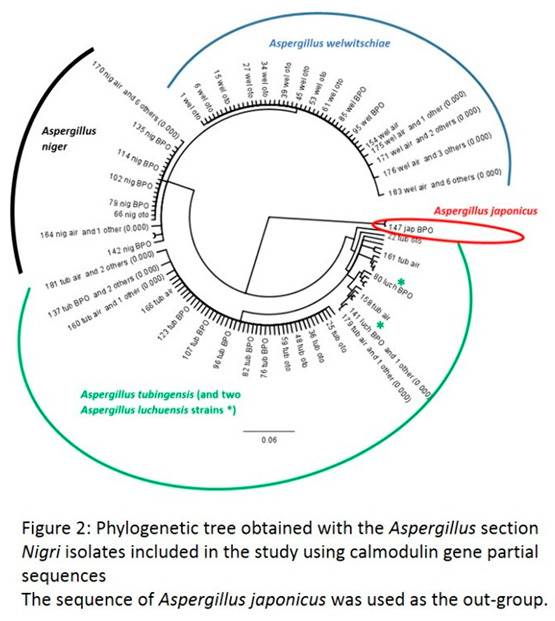



**Conclusion:** Among the 175 Aspergillus species belonging to the Nigri section, *A. welwitschiae* was found significantly associated with otomycosis whereas the species distribution was similar between respiratory and environmental isolates. This finding suggests specific physical properties of *A. welwitschiae* to thrive in the external ear. One hypothesis could be a better fitness to infect EEC containing different lipids linked to the ear-wax.

### P484. Fungal necrotizing external otitis

AlouiD.^1^TbiniM.^2^JaafouraH.^2^CheikhrouhouS.^1^BouchekouaM.^1^TrabelsiS.^1^SalahM. Ben^2^KhaledS.^1^1Laboratory of Parasitology and Mycology, Charles Nicolle Hospital, tunis, Tunisia2Oto-rhino-laryngology, Charles Nicolle Hospital, tunis, Tunisia

**Objectives:** Malignant otitis externa is an uncommon, but potentially fatal, complication of otitis externa affecting immunocompromised and elderly patients, particularly those with diabetes. Fungal infection is rare, severe and the clinical presentations pose significant problems in diagnosis and treatment. The purpose of our study is to report the diagnostic and therapeutic difficulties of fungal necrotizing external otitis.

**Methods:** It is a retrospective study spread over 5 years conducted between January 2012 and December 2017. It included patients with documented diagnosis of fungal necrotizing external otitis.

**Results:** There were eight men and three women, all diabetic with a mean age of 64 years. The reason for the consultation was treatment-resistant otalgia and otorrhea. The clinical examination and the TDM (tomodensitometry) were in favor of a necrotizing external ear infection. All patients were treated with antipyocyanic therapy antibiotic (ceftazidime and ciprofloxacin) for an average duration of one month. A swab of the infected ear was taken for mycological examination and a mycosic agent was revealed within an average of 45 days. *Candida albicans* was isolated in four cases and *Candida parapsilosis* in one case. Concerning molds, *Aspergillus flavus* was isolated in four cases, *Aspergillus fumigatus* and *Rhizopus spp* in one case respectively. Treatment was based on Amphotericin B in two cases and on Amphotericin B followed by Voriconazole in the other cases. The average duration of treatment was four months. Seven patients evolved well, two were lost to follow-up and two died. The average follow-up period was 11 months.

**Conclusion:** When a necrotizing external ear infection, occurs in an immunocompromised patient, with no response to antibiotic therapy, a fungal infection should be suspected and treated early and effectively to ensure a better prognosis.

### P485. Chromagars for differentiation between Trichophyton benhamiae, Microsporum canis and M. audouinii

RamirezE. AlvaradoVargasL.StenströmC.ForsblomS.GirestamB.LokoG.ChryssanthouE.Clinical Microbiology, Karolinska University Hospital, Stockholm, Sweden

**Objectives: Objective**: *Trichophyton benhamiae* has increasingly been isolated in Europe during the last years. In culture, this dermatophyte is difficult to differentiate macroscopically from immature colonies of *Microsporum canis* and sometimes from non-sporulating form of *M. audouinii*. Here we have used different culture conditions aiming to establish the macroscopic differences between these species, which could be useful for identification.

**Methods: Method**: *T benhamiae* (6 isolates), *M. canis* (8 isolates), and *M. audouinii* (5 isolates) were cultured at 28 °C on Trichophyton agars for nutritional requirements (5 d), urease (5 d), Chromagar CandiSelect™ (BioRad) (7-10 d) and CHROMAgar™Candida (CHROMAgar) (7-10 d). Identification of *T. benhamiae*, *M. canis* and non-sporulating form of *M. audouinii* were confirmed by ITS sequencing.

**Results: Results**: Five of 6 *T. benhamiae* isolates had requirement for vitamins (inositol and thiamine) and had positive urease test after 5 days incubation at 28 °C. *M. canis* and *M. audouinii* isolates showed no vitamin requirements. *M. audouinii* showed weak or negative urease test. The tested dermatophyte species could be differentiated by colony colour on Chromagar CandiSelect™: *T. benhamiae* presented turquoise diffuse pigment, meanwhile *M. canis* had light yellow and *M. audouinii* purple pigment around the colony. On CHROMAgar™Candida the colonies showed macroscopical differences: *T. benhamiae* had colonies with white centre and yellow margin, and the other two dermatophytes had brownish colonies.

**Conclusion: Conclusion**: Identification and differentiation between *T. benhamiae, M.canis* (immature colonies) and *M. audouinii* (non-sporulating form) by conventional methods is difficult. Using biochemical tests like vitamin requirements, urease and Chromagars, may be helpful for identification.

### P487. First results in the study of mycetoma in Turkana (Kenya)

ColomM.F.^1^FerrerC.^1^LetingS.^2^RamírezL.^1^EkaiJ.^2^HernándezC.^3^1Medical Mycology Lab, University Miguel Hernandez, Sant Joan d’Alacant, Spain2Medical Diagnosis Laboratory, Lodwar County Referral Hospital, Lodwar, Kenya3General Surgery, Hospital Clínico San Carlos, Madrid, Spain

**Objectives:** The aim of the study was to assess the prevalence of mycetoma in the county of Turkana (Northwest Kenya), as well as to describe the main causative agents involved in the disease and their possible environmental origin, using simple methods that can be applied locally.

**Methods:** Based on the data collected by the team of cooperative medicine *Cirugia en Turkana*, a specific study for mycetoma was started during the 16th campaign of that team in February 2019. Patients with suspected mycetoma were studied at the Lodwar County Referral Hospital (LCRH). After informing the patient and getting their consent, the lesions were examined and sampled (mainly by biopsy) and clinical data were recorded. Samples were washed in sterile saline solution and cut in fragments. Some of these were inoculated in Sabouraud Dextrose Agar and Malt Extract Agar plates. One fragment of each sample was used for DNA extraction. The DNA and the rest of the fragments of samples were kept at -20ºC. Environmental samples were also taken from soils, trees and shrubs in different locations of the county. These samples were also fragmented and inoculated in the same culture media. All cultures were incubated at room temperature at the LCRH laboratory. The DNA obtained from clinical samples was submitted to PCR amplification of the ITS-5.8S and the V4-V5 16S rRNA gene region, for the detection and identification of fungi and bacteria respectively.

**Results:** In this 2019 campaign 11 patients were studied. All were men between 13 and 70 Yo (mean age 34). Ten of them were shepherds and one worked as a builder. Lesions were mainly located at the feet (91%). Three of them referred black grains in the exudate and 6 yellow or cream coloured. Culture of clinical and environmental samples yield 5 and 24 fungal colonies respectively. For clinical samples, most of cultures were negative. From environmental samples, fungi morphologically compatible with *Madurella mycetomatis* and *Trematosphaeria grisea* were obtained as well as other mycetoma-related genera (*Alternaria and Aspergillus*). Molecular identification of showed *Aspergillus terreus*, *Aspergillus flavus*, and *Paecilomyces formosus* in the environment. *Cellulosimicrobium cellulans,* an actinobacteria that has not been previously reported as causative agent of mycetoma, was detected in two of the clinical samples, *Alternaria sp*. in one and *Penicillium spp* in 5 *(P. glabrum, P. spinulosum, P. corylophilus)*. After DNA amplification, three samples remained negative.

**Conclusion:** The detection of *Cellulosimicrobium cellulans* could be a significative result of this study. Nevertheless, it should be confirmed in more samples and patients. For *Alternaria sp*, and *Aspergillus sp*, they had been reported as mycetoma agents previously. The lack of results from some of the yellow grain mycetomas could be since some patients were under antibiotic treatment and, for the black grain ones, this is probably due to the low sensitivity of the method in the detection of these fungi. The isolation of mycetoma agents from the environmental samples confirms that not only Acacia thorns, but also other vegetal products, can be the origin and vehicle of inoculation of the pathogens

### P488. Examining the efficacy of Origanum heracleoticum essential oil against fungal pathogens of the nails: preliminary results.

SamarasK.MarkantonatouA.-M.VyzantiadisT.-A.First Department of Microbiology, Medical School, Aristotle University of Thessaloniki, Thessaloniki, Greece

**Objectives:** Fungal infections of the nails (onychomycoses) are very common and often need long term treatments, not always effective. In addition to the available chemical antifungal agents, natural products have been used as well. Scope of this study was to test the possible efficacy of commercial antifungal regimens based on *Origanum heracleoticum* essential oil as well as the efficacy of their main antifungal substance, carvacrol, against common fungal pathogens of the nails.

**Methods:** Three different regimens were tested. Regimen (A), contains *Origanum heracleoticum* essential oil (purity>99%, carvacrol >86%), whereas regimen (B) contains the essential oil (28% v/v) in combination with other natural essential oils. Both formulations are suitable for external use. The antifungal activity of pure carvacrol was also tested. Possible indication of the aforementioned regimens (A and B) is the treatment of microbial pathogens of the nails. The proposed dosage for regimen (A) is 4-6 drops (20 μL per drop) oregano essential oil diluted in a teaspoon (5 mL) of another oil (for example olive oil), which is equivalent to carvacrol concentration of approximately 13.76-20.64 mg/ml. Regimen (B) is proposed to be used undiluted (carvacrol concentration approximately 240 mg/ml). In order to demonstrate their possible antifungal activity, serial dilutions of regimens (A), (B) and pure carvacrol from 1,024 mg/L to 0.031 mg/L were tested against dermatophytic (*Trichophyton rubrum* and *Trichophyton interdigitale*) and non dermatophytic isolates (*Candida albicans, Candida parapsilosis, Candida glabrata* and *Scopulariopsis brevicaulis*) which are all common causes of onychomycoses. The fungal strains were examined also for their sensitivity in fluconazole and itraconazole. The susceptibility testing was performed according to the proposed EUCAST methodology (microdilution method).

**Results:** All tested regimens exhibited antifungal activity in high concentrations. Depending on the fungal strain the range of MICs was 64-256 mg/L for regimen (A), 64-512 mg/L for regimen (B) and 64-512 mg/L for pure carvacrol. The ranges of MICs were 0.063-4 mg/L for itraconazole and 0.5-64 mg/L for fluconazole.

**Conclusion:** The results of the study demonstrate that the regimens tested may have antifungal activity against both dermatophytic and non dermatophytic isolates, although in much higher concentrations than the commonly used antifungal drugs. However, being substances of only local use on the fungal lesions, there is the probability that the above mentioned concentrations could be achieved on the nail application. Further experimental data and studies of clinical use are required in order to specify potential benefit, duration of treatment and possible toxicity.

### P489. Cutaneous ulcer due to Candida parasilosis associated with skin trauma in an adult patient with psoriasis vulgaris

GeG.^1^ShiD.^2^1Jining Medical University, Jining, China2Jining No. 1 People’s Hospital, Jining, China

**Case Report:** The *Candida parapsilosis* (*C. parapsilosis*) family has emerged as a major opportunistic and nosocomial pathogen. It causes multifaceted pathology in immuno-compromised and normal hosts. Common symptoms of *Candida* skin infections include thickening of the skin, hyperkeratosis, and erythema. *C. parapsilosis* rarely induced the skin ulcer without assistance from other pathogens. Here, we present 27-year-old female with cutaneous ulcer on her left foot due to *C. parapsilosis* after trauma. She has a 6-month history of psoriasis vulgaris without treatment. *C. parapsilosis* was isolated from skin lesion for several times and was confirmed by morphological and cultural characteristics as well as by DNA molecular analysis. Patient was successfully treated with oral itraconazole in 200 mg daily for seven weeks. To our knowledge, it was the first report that *C. parapsilosis* induced cutaneous ulcer in a patient with psoriasis; Cutaneous candidiasis maybe associated with the skin barrier damage (the trauma) or psoriasis.

### P490. rTwo genetic lines of Trichophyton rubrum population in Russia

PchelinI.MochalovY.ChilinaG.VasilyevaN.TaraskinaA.Kashkin Research Institute of Medical Mycology, North-Western State Medical University named after I.I. Mechnikov, Saint Petersburg, Russian Federation

**Objectives:** *Trichophyton rubrum* is the most prevalent agent of onychomycosis and tinea pedis in most countries, including Russia. However, data on its population structure remain controversial, with some studies reporting the absence of the structure and others reporting bipartite structure. We aimed at studying *T. rubrum* population by microsatellite analysis and typing of TERG_02941 locus.

**Methods:** The study included 59 clinical isolates of *T. rubrum* from Saint Petersburg and Yekaterinburg, Russia. Species identification was done by ITS rDNA region sequencing. Typing was performed by real time PCR of the locus TERG_02941 and microsatellite analysis of 12 loci, including 4 original ones. The data were processed in the R software using the polysat and poppr packages. Genetic distances were calculated by Bruvo method.

**Results:** Ribosomal ITS region sequencing revealed nucleotide sequence, identical to that one, deposited under KT285224 accession in all but two isolates. Real-time PCR of the TERG_02941 locus revealed one SNP at position 793. At this position, adenine was found in 59% of isolates and guanine was found in 34% of isolates. The scatterplot, based on the results of the analysis of the lengths of microsatellite loci by the method of principal components, contained two separate clouds of points. One of the clouds was formed by isolates with TERG_02941 793A genotype, the other — by 793G isolates (Fig. 1). The value of the Simpson index, calculated for equal samples, was not significantly different for TERG_02941 genotypes. The association index, *rˉ_d_* = 0.02483, was calculated using the Multilocus Style Permutation method after deleting one non-informative locus and performing clonal correction. The value laid beyond the right boundary of the distribution of expected frequencies. The probability *rˉ_d_* was estimated as 0.002, which made it possible to reject the null hypothesis of the absence of connections between the markers. The *χ*^2^ test showed no connection between the TERG_02941 isolate genotype and the localization of the lesion on the patient’s body.

**Conclusion:** Microsatellite analysis with an extended set of loci confirms the presence of two genetic lines of the fungus *T. rubrum* in the territory of the Russian Federation, with the same diversity. In the population of the fungus there is a significant deviation from the Hardy-Weinberg equilibrium.

### P491. Comparison of three skin sampling methods and two media for culturing Malassezia yeast

AbdillahA.^1^KhelaifiaS.^2^RaoultD.^2^BittarF.^3^RanqueS.^1^1Vitrome, Aix Marseille Univ, IRD, AP-HM, SSA, Marseille, France2Culturomics, IHU Méditerranée Infection, Marseille, France3Mephi, Aix Marseille Univ, IRD, AP-HM, SSA, Marseille, France

**Objectives:** *Malassezia* species are lipid-dependent basidiomycetous yeast. They are resident commensals of the human skin but also cause common skin diseases, such as pityriasis versicolor, seborrheic dermatitis, *etc*. Whether they are associated with other human diseases remains an open question, partly because of the lack of efficient medium to culture these fastidious yeast. Besides, the absence of a standardized skin sampling technique introduces a major bias in culture-based descriptive studies. The objectives of this study were: 1) to compare three methods of skin sampling for the detection of *Malassezia* yeast and 2) to evaluate the performances of « FastFung » a new culture medium based on the Schædler agar, for the growth of *Malassezia* spp. by culture compared to the reference Dixon medium.

**Methods:** A total of 10 healthy volunteers were included in this study. Fifteen skin samples were collected at 5 different body sites for each participant using three sampling methods: a) dry cotton swabs, b) swabs in a liquid transport medium « Transwab®», and c) a sterile gauze. The three methods were applied successively in a random order, which was noted, at each sampling site. Each skin sample was then plated in parallel, in a random order on both Dixon and FastFung media. All plates were incubated aerobically at 30°C and cultures were examined each day for one week for *Malassezia* spp. growth.

**Results:** *Malassezia* yeast grew in 93/300 (31%) culture plates, corresponding to 150 samples. A total of 1 082 *Malassezia* spp. colonies were isolated and identified by MALDI-TOF Mass Spectrometry, of which 455 (42%) were *M. globosa*, 424 (40%) *M. sympodialis*, and 203 (18%) *M. restricta*. Multivariate statistical analysis found a significant independent effect (*P*<10^-3^) of the sampling methods. of the 93 positive culture, 62 (67%) were sampled using a sterile gauze, 17 (18%) with Transwab^®^, and 14 (15%) with the dry cotton swab. We also found a statistically significant independent effect (*P* = 0.003) of the culture medium, with the FastFung and Dixon media yielding 58 (62%) and 35 (38%) positive culture, respectively. Interestingly, the majority of the fastidious *M. globosa* and *M. restricta* species were isolated on the FastFung medium.

**Conclusion:** Our findings demonstrate that sterile gauze is the most efficient and reliable skin sampling technique to culture *Malassezia* yeast; and that the novel FastFung culture medium is suitable, and more efficient than the Dixon medium for *Malassezia* spp. culture. We propose implementing sterile gauze skin sampling and the FastFung culture medium in the routine *Malassezia* spp. cultivation procedures.

### P495. Morphological and molecular characterization of the T. benhamiae isolates.

PackeuA.^1^BaertF.^1^GoensK.^1^RoesemsS.^1^StubbeD.^2^HendrickxM.^3^1Mycology & Aerobiology, Sciensano, Brussels, Belgium2Mycology & Aerobiology, Bccm/ihem Collection, Sciensano, Brussels, Belgium3Mycology, Sciensano, BCCM/IHEM, Brussels, Belgium

**Objectives:** Dermatophytes are keratinophilic fungi responsible for causing dermatophytosis in both man and animals. Recently, several genera were (re)introduced in addition to *Epidermophyton*, *Microsporum*, and *Trichophyton* and depending on their primary habitat, divided into geophilic, zoophilic and anthropophilic species. Due to the fact that they are affecting more than 20-25% of the world’s population, able to affect healthy individuals and in some cases are communicable makes this specific group of fungi of great importance for public health. Recent taxonomical changes (de Hoog *et al.*, 2017) proposed an increase in the number of genera, simplifying identification of dermatophytes in routine diagnostics. The implementation of these changes necessitated a taxonomic re-evaluation of the dermatophytes within the Belgian BCCM/IHEM culture collection. and furthermore, an evaluation of such a large dataset leads to further insights in the taxonomy of this specific group of dermatophytes.

**Methods:** In the present study, the members of the *T. benhamiae* and *T. bullosum* clade were evaluated, using different molecular methods (sequencing and MALDI-TOF MS). The obtained results were coupled with conventional physiological, morphological, clinical, and geographical data of the strains in order to obtain a correct nomenclature for each analysed isolate. As proposed by de Hoog *et al.*, *T. benhamiae* strains can be categorized into two subclades: the *T. bullosum* clade and the *T. benhamiae* clade. The *T. bullosum* clade comprises the type strain of this zoophilic species isolated from cutaneous lesion of a horse described for the first time in 1933 by Lebasque. The *T. benhamiae* clade comprises isolates from the zoophilic species *T. benhamiae*, *T. erinacei*, *T. verrucosum* and *T. eriotrephon* and the anthropohilic *T. concentricum* species. However, in both clades delineation of the different species still remains unclear. Especially for *T. benhamiae* a lot of confusion still exists, where 2 different phenotypes (white and yellow) have been described.

**Results:** Preliminary molecular results (ITS and BT), obtained with the BCCM-IHEM isolates, point to the existence of only one *T. benhamiae* clade with well supported clades for the species *T. erinacei*, *T. verrucosum* and *T. eriotrephon*. The remaining species are, however, not well resolved. While most *T. benhamiae* strains clustered together with *T. concentricum* as a subclade in the ITS+BT phylogeny, some strains clustered with *T. bullosum* forming a highly supported clade.

**Conclusion:** In order to better delineate the different taxonomic entities and to define criteria for their identification, a combining of all results obtained via the proposed polyphasic approach will be primordial.

### P496. Phenotypic and phylogenetic characterization of malbranchea-like isolates from clinical specimens in United States

Cano-LiraJ.F.^1^Rodriguez-AndradeE.^1^WiederholdN.^2^StchigelA.^1^1Mycology, Universitat Rovira i Virgili, Reus, Spain2Pathology, The University of Texas Health Science Center at San Antonio, San Antonio, United States of America

**Objectives:** The main objective of this work was to identify phenotypically and molecularly twenty-two strains from clinical specimens in USA, preliminary classified as *Malbranchea* spp.

**Methods:** For cultural characterization, the strains were grown onto potatoes extract agar (PDA), phytone yeast extract agar and oatmeal agar, and the colonies measured and described after two weeks at 25°C. Vegetative and reproductive structures were observed and documented from slide cultures mounted on water and 60% lactic acid by a BH1 Olympus microscope. Photo micrographs were taken using a Zeiss Axio-Imager M1 light microscope with a DeltaPix Infinity X digital camera using Nomarski differential interference contrast. For phylogenetic analysis, total DNA was extracted from colonies after 7-10 days of growth on PDA at 25 °C. DNA was used to amplify and to sequence a fragment of the 28S nrRNA gene (LSU) using the primer pair LR0R and LR5, and the ribosomal internal transcribed spacers (ITS) by using the primer pair ITS5 and ITS4. A preliminary molecular identification was performed with ITS and LSU sequences using the Basic Local Alignment Search Tool (BLAST; https://blast.ncbi.nlm.nih.gov/Blast.cgi). A maximum level of identity of ≥ 98% respect to the sequences of type or reliable reference strains deposited in the GenBank/EMBL database, and the phenotypic characteristics, were used for identification at species-level. LSU sequences generated by us were aligned with other from known taxa belonging to the order Onygenales by ClustalW application of MEGA v. 6.06. The phylogenetic tree was built using maximum-likelihood (ML) and Bayesian-inference (BI) methods with RAxML v. 8.2.10 and MrBayes v3.2.6 computer programs, respectively. *Oidiodendron truncatum* and *Myxotrichum deflexum* (*Myxotrichaceae* family) were used as outgroup.

**Results:** Hitherto, taking into account the phylogenetic data and the phenotypic features, eleven strains were identified at species level. We report the finding of *Auxarthron alboluteum* (two strains), *A. conjugatum* (two strains), *A. umbrinum* (three strains) and *A. zuffianum* (one strain); and of *Malbranchea aurantiaca* (two strains) and *M. flocciformis* (one strain). One strain of *Arachnomyces*, two of *Auxarthron*, one of *Malbranchea* and two of *Spiromastigoides* could not be identified at species-level. In addition, two strains did not matched at genus-level.

**Conclusion:** The phenotypic characterization and the phylogenetic analysis carried out by us only allowed us to identify at species-level 11/19 strains previously identified as *Malbranchea* spp. Most of these strains finally resulted known species of the genus *Auxarthron*, and only three of them could be identified as *Malbranchea* species. This indicates that the presumptive morphological identification is unreliable, and molecular data are needed for the correct identification of this sort of fungi. Surprisingly, eight of nineteen strains were identified at genus-level or not identified, which suggests that these malbranchea-like fungi could be putatively new taxa

### P497. Fast mold identification with MALDI-TOF MS: The importance of the spectra acquisition method

MucekK.MaierT.TimkeM.KostrzewaM.Research and Development, Microbiology and Diagnostics, Bruker Daltonik GmbH, Bremen, Germany

**Objectives:** Invasive fungal infections are a major cause for morbidity and mortality in ill, immunocompromised and paediatric patients [1, 2]. Clinical diagnosis of mold infections and identification of fungal contamination is often based on morphological criteria which encounter issues like robustness of phenotypic procedures, often sporulation as a prerequisite for identification and very few skilled mycologists that are capable of differentiating molds in the medical environment. In order to solve this situation one of the most promising alternative to identify clinically and environmental relevant molds is MALDI-TOF MS. Through the generation of protein spectra and the comparison with a reference library, molds can be identified fast and reliable. However, in some cases the acquisition of mass spectra particular from filamentous fungi is challenging since the frond mycelium often results in reduced spectra quality.

**Methods:** A minimum about 56 filamentous fungi was measured with MALDI-TOF in duplicates with at least two different operators. Each strain was cultivated on Sabouraud agar for 24 – 48 h, front mycel was smeared on a MALDI target plate. One of two spots was pre-treated with formic acid before all spots were overlaid with HCCA matrix and the measurement started. Two different acquisition methods were applied. The Bruker ‘standard’ method and the ‘filamentous fungi’ method was compared. Subsequently all spectra were matched against the Filamentous Fungi Library 3.0.

**Results:** After cultivation of filamentous fungi biological material could be transferred successfully to a MALDI-TOF target plate in duplicates for each strain. The results showed that the rate of spectra acquisition by using the filamentous fungi method was significant higher (about 25%) compared to the standard acquisition method. It could be shown additionally that the acquired spectra resulted in an increased identification rate.

**Conclusion:** The MALDI Biotyper Filamentous Fungi Module with adapted acquisition settings for filamentous fungi improves the identification rate for molds. MALDI-TOF based differentiation is less error prone and faster compared to classical identification. Results can be easily obtained in one hour from cultivation plate. Moreover, the adapted filamentous fungi method compared to the standard spectra acquisition method is a vast advantage. Time consuming repetition of target preparation and re-measurement is reduced as well as previous protein extraction for MALDI-TOF measurement. Thus, mold infections like aspergillosis or mucormycosis or fungal contaminations are identified more reliable and faster. [1] Prattes, J., Heldt, S., Eigl, S. et al. Curr Fungal Infect Rep (2016) 10: 43. https://doi.org/10.1007/s12281-016-0254-5 [2] Gullo, A. Drugs (2009) 69(Suppl 1): 65. https://doi.org/10.2165/11315530-000000000-00000

### P498. Trichophyton mentagrophytes – a new genotype in Cambodia

UhrlassS.^1^SithachM.^2^KochD.^1^WittigF.^1^MuetzeH.^1^KruegerC.^1^NenoffP.^1^1Partnership Dr. C. Krueger & Prof. P. Nenoff, Laboratory of medical microbiology, Roetha OT Moelbis, Germany2Department of Dermatology, Preah Kossamak Hospital, Phnom Penh, Cambodia

**Objectives:** According to the currently suggested classification and new taxonomy of the dermatophytes, the former *Trichophyton mentagrophytes* (*TM*)-complex is now differentiated in *TM* (zoophilic strains) and *Trichophyton interdigitale* (*TI*, anthropophilic strains).

**Methods:** Mycological diagnostics was done during a survey of superficial dermatophytoses in Cambodia.

**Results:** 1) The new genotype “Cambodia” of *TM* (strain 201341/18) was found in a 8 years old male child with skin lesions starting already 3 months ago, prior the dermatology consultation. Inflammatory papules on the right part of the scalp were to be seen. 2) A 31 years old male suffered from a tinea cruris. The dermatophyte isolate was found to be the new *TM* genotype Cambodia (strain 218292/17). 3) A 26 years old male farmer showed lesions appearing since one year, started with scaly pruriginous plaques on both hips. Clinical diagnosis was tinea corporis. By mycological diagnostics, from skin scrapings, a dermatophyte was isolated. By sequencing of the DNA it was identified as *TM* genotype VIII India (strain 218294/17). 4) A one-year-old male was diagnosed as tinea corporis. The dermatophyte which was growing from skin scrapings of the baby´s cutaneous lesions was identified as *T*. *interdigitale* (*TI* strain 250037/18 genotype II* - mixed type between *TI* and *TM*).

**Conclusion:** Today, due to sequencing of the ITS region of the rDNA, at least 10 genotypes of TM and TI are known. These genotypes are mostly associated to the geographic origin or to the source of infection. Currently, two genotypes of *TI* (ITS Type I [Europe] and ITS Type II [Cosmopolite]), were described. *TM,* however, comprises the common zoophilic “German” ITS Type III (Europe) and III* (Cosmopolite), genotype IV (UK, USA, South Africa and Feancde), V (Asia, Egypt, Iran, Iraq and Japan), VI (Europe, Russia and Finland), VII (from Thailand originating isolates in Switzerland and Germany, USA, Australia, Georgia, Russia, and Vietnam), VIII (Asia, India, Iran, Oman, Australia), and last but not least the Australian genotype (ITS Type IX). Based on results of sequencing preferably of the ITS region of the rDNA, we are able to demonstrate that 2 out of 3 TM strains in Cambodia represent a new, until now not described, ITS genotype which is called now the “Cambodian” genotype of TM. Surprisingly, we found one additional TM strain of the Indian genotype VIII. An association between this dermatophytosis in Cambodia and the current Indian outbreak of superficial dermatophytosis can only be suspected. The fourth here isolated dermatophyte belongs to the until now very rarely described genotype *TI* II*. Phylogenetic, this genotype is the so called mixed type between anthropophilic *TI* and zoophilic *TM*. Each genotype of the TM/TI complex seems to be specific for e.g. a geographic area, for a country, or for a distinct animal which represents the source of infection, speaking for a special way of transmission of the infection.

### P499. Novel Cunninghamella species causing mucormycosis in an apparently immunocompetent individual

HallurV.^1^SableM.^2^CP.^3^RudramurthyS. M^4^SharmaP.^5^PrakashH.^4^ChakrabartiA.^4^1Microbiology, All India Institute of Medical Sciences, Bhubaneswar, Bhubaneswar, India2Pathology, All India Institute of Medical Sciences, Bhubaneswar, Bhubaneswar, India3Ent, All India Institute of Medical Sciences, Bhubaneswar, Bhubaneswar, India4Medical Microbiology, Postgraduate Institute of Medical Education and Research, Chandigarh, India5Ent, All India Institute of Medical Sciences Bhubaneswar, Bhubaneswar, India

**Case Report: Title:** Novel *Cunninghamella* species causing mucormycosis in an apparently immunocompetent individual Mucormycosis due to *Cunninghamella* spp. is extremely rare clinical entity. We report here a case of mucormycosis due to *Cunninghamella* sp. in an apparently immunocompetent individual. **Material & Methods: Patient & microbiological methods**: Tissue biopsy from the lesion was subjected to histopathology, and culture. Formalin fixed paraffin embedded tissue (FFPE) and culture were sent to Mycology Reference Laboratory at PGIMER Chandigarh for species identification & susceptibility testing. **Molecular identification:** DNA was extracted from FFPE tissue and cultures as described previously by Rickerts *et. al.(1)* & Prakash *et.al*. (2) DNA from FFPE tissue was PCR amplified using mucoromycetes specific primers. The ITS and 26s genes of ribosomal DNA of the isolate were amplified using pan fungal primers. Consensus sequences obtained from amplified products were analyzed using the National Center for Biotechnology Information (NCBI) Basic Local Alignment Search Tool (BLAST). **Phylogenetic characterization:** The 26S rDNA and ITS sequences of different *Cunninghamella* were retrieved from NCBI nucleotide database. Phylogenetic analysis was performed as per Prakash *et.al*.(2)

**Results: Patient & microbiological methods:** A 26-year-old healthy man presented with slowly progressive swelling on the left cheek for 2 years and ulceration over the swelling for two months. Laboratory investigations revealed a normal blood sugar, hemogram, CD4 counts, liver and renal function tests. He was non-reactive for HIV. Contrast enhanced computed tomography scan of the head and neck showed mild enhancing lesions on left side of the face along with thickening of floor of maxilla. Biopsy from the lesion showed focal areas of chronic inflammation with multinucleated giant cells containing broad aseptate fungal hyphae. A rapidly growing white mould with aseptate hyphae & erect sporangiophores ending in a swollen vesicle, with 1-spored sporangiola suggestive of *Cunninghamella* was isolated. The isolate had minimum inhibitory concentrations (MICs) of 4µg/ml for amphotericin B, itraconazole, and posaconazole; and 3µg/ml for Terbinafine as per CLSI M38A2. He was managed with repeated debridement over 7.5 months, amphotericin B and posaconazole for 1.5 months & 6 months respectively. As there was no improvement, he was finally treated with oral Itraconazole. At present the ulcer has healed, but the swelling is still persisting. **Molecular identification**: The fungus was identified as *Cunnighamella sp.* by analysis of DNA sequence of the FFPE tissue. ITS sequences of the isolate showed 97.54% base identity with uncultured *Cunninghamella* spp. (MG571234.1) isolated from Thailand. **Phylogenetic analysis:** Phylogenetic analysis of ITS and 26s regions of all known *Cunninghamella* species with the present isolate suggested that the isolate may be a new or undefined species under genus *Cunninghamella*. **Conclusion:** We report recalcitrant mucormycosis due to a *Cunninghamella* sp., which is phylogenetically different from other described species in this genus. Further molecular characterization is essential to identify the species. References: Eur J Clin Microbiol Infect Dis. 2006 Jan;25(1):8-13. Med Mycol. 2016 Aug 1;54(6):567-75. doi: 10.1093

### P500. rResolving Trichophyton benhamiae complex: zoonotic pathogens on the rise

CmokovaA.^1^,^2^KolarikM.^1^StubbeD.^3^DobiasR.^4^LyskovaP.^5^HoyerL.^6^JanouskovcovaH.^7^WiegandC.^8^MalatovaN.^9^KanoR.^10^NenoffP.^11^UhrlassS.^12^PeanoA.^13^MenclK.^14^MaierT.^15^KuklovaI.^16^HubkaV.^1^,^17^1Institute of Microbiology, Czech Academy of Science, Prague, Czech Republic2Department of Botany, Charles University, Prague, Czech Republic3Mycology & Aerobiology, Sciensano, Brussels, Belgium4Bacteriology and Mycology, Public Health Institute in Ostrava, Ostrava, Czech Republic5Institute of Health in Usti nad Labem, Usti nad Labem, Czech Republic6College of Veterinary Medicine, University of Illinois at Urbana-Champaign, Urbana, United States of America7University Hospital in Pilsen, Pilsen, Czech Republic8Department of Parasitology, Humboldt University, Berlin, Germany9Hospital Ceske Budejovice, Ceske Budejovice, Czech Republic10Nihon University School of Veterinary Medicine, Tokyo, Japan11Laboratory For Medical Microbiology, Mölbis, Mölbis, Germany12Laboratory For Medical Microbiology, Mölbis, Tallinn, Germany13Universita degli Studi di Torino, Turin, Italy14Pardubice Regional Hospital, Pardubice, Czech Republic15Research and Development, Microbiology and Diagnostics, Bruker Daltonik GmbH, Bremen, Germany16First Faculty of Medicine, Charles University in Prague, Prague, Czech Republic17Department of Botany, Charles University, Prague, Czech Republic

**Objectives:** Species of *Trichophyton benhamiae* complex have a worldwide distribution and their host range includes many pet and livestock animals. Nowadays, this complex has become very important due to epidemic spread of *T. benhamiae* complex species in children and their pet animals in Europe. No causal mechanism has been found that would explain this increase. Moreover, the incidence of several other species from this complex is also increasing in Europe due to higher interest of people in pet hedgehogs (host of *T. erinacei*), insufficient prophylactic vaccination (*T. verrucosum*) or increasing use of molecular methods in the dermatological diagnostics leading to more accurate identification (*T. bullosum*, *T. eriotrephon*). A considerable genetic and phenotypic variability has been revealed in these emerging pathogens, but the species limits are not always clearly defined. Therefore, the aim of this study was to elucidate species boundaries and phylogenetic relationships between species belonging to the *T. benhamiae* complex.

**Methods:** A total of 352 clinical isolates from *T*. *benhamiae* complex associated with human and animal dermatophytoses were analysed using molecular markers (DNA sequence data and microsatellites), and morphological and physiological methods.

**Results:** Members of the *T. benhamiae* complex were resolved into three major clades (designated *T. benhamiae*, *T. erinacei* and *T. africanum* clades). All analyses supported delimitation of four species in the *T. benhamiae* clade, i.e., *T. benhamiae*, *T. concentricum*, and two novel species, *T. europaeum* and *T. japonicum*. In contrast to DNA sequence and based phylogeny, the population genetic and phenotypic data distinguished North American strains of *T. benhamiae* (mostly isolated from dogs) and emerging European strains associated with quinea pigs. The status of new variety, *T. benhamiae var. luteum*, is proposed for these strains. The *T. erinacei* clade consisted of three species, namely *T. erinacei*, *T. verrucosum* and *T. eriotrephon*. *Trichophyton bullosum* and one new species, *T. africanum* (previously African race of *Arthroderma benhamiae*) together with *T. bullosum* formed the *T. bullosum* clade

**Conclusion:** Species boundaries in the *T. benhamiae* clade were resolved by using polyphasic approach. This approach supported recognition of nine species, including three new zoophilic species. *Trichophyton benhamiae* was split into two varieties; *T. benhamiae var. luteum* is currently responsible for the European outbreak of zoonotic infections.

## Video abstracts

### V01. Subcutaneous nodule caused by Phaeoacremonium fuscum in a non-immunocompromised patient

ColmanS.CattoirL.BoelA.Microbiology, OLV Hospital, Aalst, Belgium

**Objectives:** *Phaeohyphomycosis* (PHM) is a chronic infection caused by dematiaceous fungi which usually involves the skin and subcutaneous tissue. As a result of the use of molecular diagnostic techniques, previously unrecognized causative agents of *phaeohyphomycosis* have been discovered. Here we report the first case of phaeohyphomycosis caused by *Phaeoacremonium fuscum* in an immunocompetent patient.

**Methods:** Case presentation A 60 year old Filipino male presented with rotator cuff pathology to the orthopedics consultation. On physical examination a painless, soft, subcutaneous mass above the right patella was noted, which didn’t cause the patient any complaints. The suprapatellar nodule had been growing slowly for 3 years. The patient denied any apparent antecedent trauma, arthropod bite, family history or contact with a person with similar lesions. The man still visits the Philippines on an infrequent basis. He had no fever, weight loss or other symptoms. Detailed physical examination further revealed a 4x3 cm, immobile, non-pulsatile light red mass. There were no other signs of inflammation of the skin. A serous effusion was aspirated from the mass.

**Results:** Fungal culture of the aspirated fluid showed growth of a filamentous fungus on chocolate blood agar and Sabouraud’s dextrose agar. Molecular identification was performed by sequence analysis of the internal transcribed spacer region 2 (ITS2). BLAST search revealed a similarity of 100% with *Phaeoacremonium fuscum*. The antifungal susceptibility profile was established by EUCAST broth microdilution: itraconazole >16 mg/L, posaconazole 0.25 mg/L, amphotericin B 0.50 mg/L, voriconazole 0.50 mg/L. This susceptibility profile is similar to those previously published. A wide local excision of the lesion was performed a couple of weeks later. The patient had an uneventful postoperative recovery. No antifungal therapy was started postoperatively since the patient was not immunocompromised and there was lack of reimbursement. Histopathological examination of the specimen showed fibrous tissue with a mixed cellular infiltrate including lymphocytes, plasma cells, macrophages and neutrophils. Periodic acid-schiff stain and Grocott stain showed evidence of branching septate hyphae.

**Conclusion:** PHM is caused by brown pigmented fungi containing melanin in their cell wall. Phaeoacremonium species are known as vascular plant pathogens, responsible for wilting and dieback of woody plants. They are also opportunistic pathogens in humans, mainly causing cutaneous or subcutaneous PHM. However, sporadic cases of respiratory tract infection, septic arthritis and disseminated infection are described. The true prevalence of Phaeoacremonium species is probably underestimated due to the asymptomatic nature of most subcutaneous lesions and the difficult identification. There is no standard antifungal regimen or evidence regarding the necessity for antifungal treatment as concomitant with surgery for localized infections. Long courses of antifungals, such as voriconazole treatment, are often employed to reduce the risk of recurrence. Case reports suggest that surgery can be curative if the infection is localized and adequately removed, as was the case in our patient. Regardless of the immune status of the patient, clinicians should consider the possibility of a fungal infection when they encounter chronic subcutaneous nodules without major symptoms.

### V02. Trichosporon diagnosis- The Right path

PremamaliniT.^1^KumarP. Kennedy^1^KumaranN. Anish^1^KindoA.J.^2^1Microbiology, Sri Ramachandra Medical College and Research Institute, SRIHER, Chennai, India2Microbiology, Sri Ramachandra Medical college and Research Institute, chennai, India

**Objectives:** To discuss about the correct path in identifying *Trichosporon* sp. To emphasize the importance of performing conventional laboratory diagnostic methods like microscopy, along with molecular methods in the diagnosis of *Trichosporon* sp.

**Methods:** Yeast-like colonies were observed for microscopic characteristics by Gram staining. The isolates which showed the presence of budding yeast cells, pseudohyphae and arthroconidia were tested for production of the enzyme urease. Urease positive test isolates were provisionally identified as belonging to the genus *Trichosporon*. To confirm the identity of all phenotypically identified *Trichosporon* isolates, *Trichosporon* genus specific colony PCR was performed. This pair of primer is *Trichosporon* specific and it amplified part of the nucleotide sequences of the rDNA small subunit (18S).

**Results:** The study of colony morphology of the *Trichosporon* isolates on SDA at 25 °C after 10 days of incubation revealed varied colony morphology. Most commonly, they may appear as white to cream, farinose or cerebriform colonies, with furrows. The important characteristic feature of this yeast is the production of arthroconidia and enzyme urease. Performing Gram stain or Dalmau technique to search for arthroconidia is a very useful tool for screening of *Trichosporon* spp. Finally, the accurate identification of this yeast was done using DNA based methods like *Trichosporon* genus specific colony PCR. The members of the genus *Trichosporon* produced DNA bands of approximately 170 bp.

**Conclusion:** *Trichosporon* sp. has been so far reported as the second most common cause of disseminated yeast infections in humans, next to the genus *Candida*. They are emerging opportunistic pathogens causing disseminated infections in immunocompromised patients. Such infections are usually difficult to diagnose, do not respond to treatment with routinely used antifungal agents, and are associated with high mortality rates. Hence, correct identification of *Trichosporon* sp. helps the clinician in providing appropriate treatment. This also applies for other emerging yeast infections, where combination of conventional and molecular methods may help in timely and accurate diagnosis.

### V03. Disseminated rhinosporidiosis with different morphological lesions involving various anatomical sites.

ChanderJ.^1^PuniaR.P.S.^2^DassA.^3^AttriA.K.^4^1Microbiology, Government Medical College Hospital, Chandigarh, India2Pathology, Government Medical College Hospital, Chandigarh, India3Ent, Government Medical College Hospital, Chandigarh, India4General Surgery, Government Medical College Hospital, Chandigarh, India

**Objectives:** Rhinosporidiosis is a chronic granulomatous disease caused by *Rhinosporidium seeberi,* which is a taxonomically debated endosporulating aquatic eukaryotic organism since long. Rhinosporidiosis predominantly involves the mucous membranes of the nose and eyes mainly conjunctiva. In recent past, giant lesions and disseminated type of disease, has also been reported in some of the clinical settings. This disease is hyperendemic in the Indian subcontinent, particularly in Sri Lanka and southern India. However, sporadic cases have occasionally been reported from other continents.

**Methods:** A case report of rare disseminated rhinosporidiosis with varied morphological lesions involving simultaneously three anatomical sites i.e. nasal, cutaneous and bones in a hepatitis B virus surface antigen (HBsAg) positive patient. A 35-years old male, resident of district Madhubani, Bihar, fisherman by occupation presented with complaints of left sided nasal obstruction, insidious in onset, gradually progressive and associated with change in voice since the last 6 years. Also, there was slowly progressive swelling over medial aspect of left ankle for the last 1 year and a similar looking swelling, smaller in size also appeared in the left post-auricular region four months back and has gradually increased in size. On examination, a polypoidal fungating mass was present over left medial aspect of foot which was non-tender, highly vascular, large, hemi-spherical in shape and bled on touch. On anterior rhinoscopy, a warty, irregular, non-tender mass, not bleeding on touch, occupying whole of left nasal cavity was seen. Oral cavity showed bulging soft palate and a bilobed pale polypoidal mass with smooth surface hanging from nasopharynx to oropharynx. There was a solitary ovoid growth measuring 2 x 2 cm with normal appearing overlying skin in the left upper post-auricular region. Serum of the patient on testing for HBsAg was positive. The patient was known chronic alcoholic and smoker.

**Results:** A wedge biopsy of the lesion when examined in normal saline showed sporangia of *R.seeberi* in different stages of development. Routine haematoxylin and eosin (H & E) staining, periodic acid-Schiff (PAS) staining as well as mucicarmine staining of the tissue also revealed findings of rhinosporidiosis with many sporangia and scattered endospores seen in a background of inflammatory infiltrate composed of polymorphs, lymphocytes and plasma cells including the bony involvement. A final diagnosis of disseminated rhinosporidiosis presenting simultaneously with three different kinds of lesions; nasal, cutaneous and osteomyelitis of medial malleolus and distal end of tibia was made. The patient was treated surgically by performing below knee amputation; transnasal and transoral excision with electrocoagulation as well as excision of postauricular mass was also performed. Histopathology of the excised bone and other growths was consistent with the preoperative diagnosis. The patient was discharged and advised dapsone therapy 100 mg bd to prevent recurrence. Presently, the patient is on follow up and there is no recurrence of the disease.

**Conclusion:** This is prudent for clinicians, microbiologists and pathologists to keep rhinosporidiosis as a differential diagnosis when managing patients with polypoidal growths even in non-endemic areas. In such a background high index of suspecion is the key to reach the final diagnosis.

